# Effects of two kinds of imidazolium-based ionic liquids on the characteristics of steroid-transformation *Arthrobacter simplex*

**DOI:** 10.1186/s12934-016-0518-3

**Published:** 2016-07-01

**Authors:** Yanbing Shen, Lifang Wang, Jingting Liang, Rui Tang, Min Wang

**Affiliations:** Key Laboratory of Industrial Fermentation Microbiology, Ministry of Education, College of Biotechnology, Tianjin University of Science and Technology, Tianjin, 300457 People’s Republic of China

**Keywords:** Ionic liquids, *Arthrobacter simplex*, Biotransformation, Cell membrane permeability, Lipid, Protein

## Abstract

**Background:**

Ionic liquids (ILs) are a promising alternative for organic solvents because these liquids exhibit unique properties and enhanced steroid 1-dehydrogenation biotransformation caused by *Arthrobacter simplex* CPCC 140451 (*ASP*). However, the effect of ILs on the whole cell itself remains poorly understood and must be further investigated.

**Results:**

A comparative investigation was performed to determine the effect of imidazolium-based ILs, namely, hydrophobic [PrMIm]PF_6_, and hydrophilic [PrMIm]BF_4_, on the steroid conversion, activity, permeability, and material basis of *ASP* cells. Both ILs weakened permeability barriers, enhanced steroid transformation, whereas reduced the activity of cells. The influence of [PrMIm]PF_6_ on the steroid conversion, permeability and activity of cells is more serious than that of [PrMIm]BF_4_ Transmission electron microscopy micrographs directly showed wrinkles, gross creases, and several small pores in ILs-treated cells surface. The total lipid content of [PrMIm]BF_4_-treated cells reduced by 8.3 %, while that of [PrMIm]PF_6_-treated cells reduced twice more, among which the content of long-chain fatty acids was decreased, whereas the content of unsaturated fatty acids was increased. The protein profile of LC–MS/MS revealed that the reduced proteins of cells treated with the two ILs were mainly located in the cytoplasm and plasma membrane, 19.27 % of reduced proteins were located on the cell membrane for [PrMIm]PF_6_-pretreated cells, whereas only 12.8 % for [PrMIm]BF_4_-pretreated cells. It suggests that most reduced proteins functioned in energy production and conversion, material transport and metabolism, signal recognition and transmission, transcription, and translation and posttranslational modification. In particular, the identified differential proteins functioned in the pentose phosphate pathway, synthesis of purines and pyrimidines, and oxidative phosphorylation and fatty acid pathway.

**Conclusion:**

Treatment with ILs improved permeability at the molecular level and exerted significant positive effects on steroid conversion. This study provides a material basis and elucidates the mechanisms underlying cellular changes that enhanced conversion rate.

**Electronic supplementary material:**

The online version of this article (doi:10.1186/s12934-016-0518-3) contains supplementary material, which is available to authorized users.

## Background

Steroid hormones constitute a class of important drug intermediates, and dehydrogenated steroids are more effective in treating diseases than their precursors [1]. The use of microbes in dehydrogenation exhibits advantages such as few processing steps, simple procedure, and high product purity for steroids [2]. However, the poor solubility of steroids limits the interaction between the substrate and the intracellular dehydrogenase. In this regard, researchers added organic solvents, cyclodextrin, surfactant, and ionic liquids (ILs) as reaction media to the fermentation medium; these additives mainly function in the rate-limiting step by improving steroid solubility [3–7]. During the last decade, ILs have attracted considerable academic and industrial interest as a class of emerging liquid salts with relatively low melting points, practically negligible vapor pressure, high solvation ability, low viscosity, and biological compatibility [8]. Hence, ILs are promising candidates for conventional reaction media for whole-cell biocatalysis [9, 10].

Since the first report on ILs and biocatalysis involving whole-cell preparation of *Rhodococcus* R312 in the biphase [BMIM]PF_6_-water system, studies demonstrated that the high solubility of the hydrophobic compound and efficient conversion can be achieved using ILs [11]. These improvements may be due to the function of ILs as hydrophobic product reservoirs to deliver steroids into the aqueous phase while avoiding the rate-limiting step and securing effective phase separation because of the high density, low viscosity, and fine-tunable chemical properties of these liquids [12]. For instance, conversion rate in the treatment group increased to 70 %, whereas that of the control group (no ILs system) was 30 % when the biotransformation of 11α-hydroxy-16,17α-epoxyprogesterone was conducted in the [BMIM]NTf_2_–aqueous biphasic system [13]. ILs also affects the growth of microorganisms. [EMIM](L)-Lac or [BMIM](L)-Lac promoted the growth of *Arthrobacter simplex* at low concentrations (<2.5 mmol L^−1^). In another study, *Saccharomyces cerevisiae* presented holes, wrinkles, and irregular appearance under high ILs concentrations, indicating that ILs interact with cell membrane components [14–16]. Paul et al. suggested that [BMIM][BF_4_] can directly interact with the globular transport protein bovine serum albumin (BSA), which is the major component of cell membrane protein [17].

Correlative reports are available regarding the direct effect of ILs on the biocatalyst itself. Ming-Liang et al. analyzed the microscopic structure of *Armillaria luteo*-*virens Sacc* cells in the [EMIM][BF_4_] environment through scanning electron microscopy (SEM) and revealed that the microscopic structure of IL incubated cells was damaged slightly [18]. Nasir Mehmood et al. observed that yeast cells became holed, softened, and gelified after pretreatment with [EMIM][OAc] or [EMIM][MeO(H)] [19]; another research preliminary analysis suggested that ILs are efficient lysis reagents that can cause protein extraction in yeast cells [20]. However, the mechanism of ILs interaction with whole cells is poorly understood and remains to be further investigated.

Numerous studies reported that enzyme activity, stability, and stereo selectivity remain high in pure imidazolium-based ILs with short alkyl side-chains [3, 4, 20]. According to our previous experiments, both [PrMIm]PF_6_ and [PrMIm]BF_4_ can enhance conversion while slightly affecting the cell activity, and a difference solubilization on water-insoluble substrates was observed between hydrophobic [PrMIm]PF_6_ and hydrophilic [PrMIm]BF_4_.

In this study, we investigated the effect of [PrMIm]PF_6_ and [PrMIm]BF_4_ on the permeability, activity, material basis, and metabolic pathway of steroid-transforming *A. simplex* (*ASP)* cells. In this work, 1-dehydrogenation of the cortisone acetate, an important reaction in steroid medicine, was used as model reaction. A strategy in which *ASP* cells were pretreated with two ILs and washed for three times with KH_2_PO_4_–NaOH buffer (PBS) was employed to imitate the interaction process between ILs and *ASP* cells during bioconversion. The obtained data will improve our understanding of the mechanism underlying the effect of ILs on the bioconversion of hydrophobic compounds by altering cell envelope permeability.

## Methods

### Materials

Substrate cortisone acetate (CA, 99.4 % purity) was purchased from Tianjin Pharmaceutical Company. Standard C_12_–C_28_ fatty acids were purchased from Sigma-Aldrich Co. ILs [PrMIm]BF_4_ and [PrMIm]PF_6_ (99.9 % purity) were purchased from Lanzhou Greenchem ILs (LICP, CAS, China). All chemical solvents and salts used were of analytical grade or higher.

### Microorganism cultivation

The strain *A. simplex* CPCC 140451 (*ASP*) used throughout the investigation was stored in our laboratory and cultivated in two stages [21]. The resting cells were harvested, washed, and re-suspended as described previously [6].

### Treatment with two kinds of ILs, steroid transformation, and analysis

The pretreatment strategy was carried out by adding 5 mL *ASP* suspension into 15 mL PBS buffer containing two ILs [0.5 % (v/v)] respectively. After incubation for a certain period, the cells were washed three times with PBS to remove ILs and re-suspended in the same buffer. Pretreated cells were added to the 20 mL buffer system with cortisone acetate (CA, 3 g L^−1^) to start the biotransformation at 34 °C and 180 rpm. The untreated cells were used in the same system in a separate experiment as the control. HPLC samples were withdrawn and extracted by chloroform, then dried in vacuum. The obtained solid extracts were redissolved in eluent [dichloromethane: ether: methanol, 86:12:2 (v/v/v)] and filtered through a 0.45 μm filter for HPLC (Agilent 1100, USA, 240 nm). Analysis was performed on a Kromasil 100-5SIL, 250 mm × 4.6 mm (5 μm) column with a flow rate of 0.8 mL min^−1^ at 30 °C.

### Determination of cell activity

The consumption of glucose has been used as an essentially interchangeable means to estimate the activity of cells [22]. In this work, metabolic activity retention (MAR) value was used to evaluate the relative sugar metabolism activity of the ILs-treated cells compared with the control group (no ILs pre-treatment). *ASP* cells were pre-treated by 0.5 % (v/v) two ILs for 2 h, respectively. Cells (ILs-treatment and without treatment) were resuspended in 20 mL PBS containing 10 g/L glucose to start the sugar metabolism at 32 °C and 160 rpm for 10 h and then centrifuged (1000×*g*, 5 min), respectively. The residual sugar content in supernatant was monitored by SBA-40C, and MAR value was calculated according to following formula.

$${\text{R}}(\%) = \frac{{{\text{Glucose}}\,{\text{consumption}}\,{\text{of}}\,{\text{ILs}}\hbox{-}{\text{pretreated}}\,{\text{cells}}}}{{{\text{Glucose}}\,{\text{consumption}}\,{\text{of control}}\,{\text{cells}}}} \times 100\,\%$$

### Cell membrane permeability

Cell membrane permeability was characterized by the leakage of cytoplasmic inclusions. Nucleic acid and protein exhibited the maximum ultraviolet (UV) absorption peak at 260 and 280 nm, respectively. The cell suspension (with ILs pre-treatment) was centrifuged (5000×*g*, 5 min), and UV absorption was determined at 260 and 280 nm, respectively. The changes of UV absorption can exactly represent the leakage of cytoplasmic inclusions and reveal the changes of cell membrane permeability as described previously [23]. Besides, changes in cell membrane integrity can be directly observed using a transmission electron microscopy (TEM) diagram. The sample preparation was as described previously [6].

### Lipid analysis

Lipids were successively extracted from 2 g of dried bacteria with 35 mL of methanol, 35 mL of chloroform, and 35 mL of methanol/chloroform [2:1(v)], and the same step was repeated once. All extract edliquor was collected into a round-bottom flask and then placed into a rotary evaporator [24]. The lipids were dried under a stream of nitrogen. Prior to flame ionization detector (FID)–mass spectrometry (MS) analysis, the lipid extract operations aimed at sample preparation was injected into the gas chromatography (GC) bottle in the next step as described previously [6]. Changes in the fatty acid composition were analyzed using a triple quadrupole instrument model QUATTRO II (MS) equipped with an FID operating at a typical flow rate of 20 mL/min.

### Protein analysis and identification

Cells were treated by 2.5 % (v/v) [PrMIm]BF_4_ and [PrMIm]PF_6_ in PBS for 2 h, respectively, and then, untreated cells and ILs-treated cells were collected and washed three times with PBS. The pellets were resuspended in lysis buffer containing 1 mmol L^−1^ phenylmethylsulfonyl fluoride (PMSF), a protease inhibitor. The suspensions were immersed in an ice bath and sonicated for 90 cycles (10 s percycle at 10 s intervals). The suspensions were withdrawn and centrifuged (12,000×*g*, 10 min). Protein contents in supernatural fluids were estimated using a Bradford protein assay, with BSA as standard. The proteins were precipitated overnight with four volumes of acetone and carefully removed after centrifugation at 10,000×*g* for 10 min at 4 °C. The pellet was digested with trypsin, and HPLC–MS/MS was performed using an LTQ linear ion trap mass spectrometer (Thermo, San Jose, CA, USA). Subsequently, 20 μL of the prepared specimen was injected and washed out at a rate of 200 μL min^−1^. Gradient elution conditions were as described previously [6]. The international standard (IS)-optimized mass parameters used in this study were as follows: voltage of the capillary and the cone, 3.5 kV; source temperature, 260 °C; and desolvation temperature, 260 °C. System control and data collection were performed using Xcalibur software version 1.4 (Thermo). The acquired MS/MS spectra were compared against the *Arthrobacter* database from NCBI by using *TurboSEQUEST* program in the Proteomics Discovery 1.2 software suite (Thermo). For data analysis, protein data with relative expression (*R*) of >2 and <0.5 and *P* value <0.05 were selected to ensure upregulation and downregulation authenticity. The *G*_i_ numbers of the selected proteins were imported into the Kyoto Encyclopedia of Genes and Genomes (KEGG) Database (http://www.genome.jp/kegg/) for biological pathway analysis and the results are shown in Additional files 1, 2, 3, 4.

## Results

### Effect of two kinds of ILs on steroid conversion, activity, and permeability of *ASP* cells

**Fig. 1:**
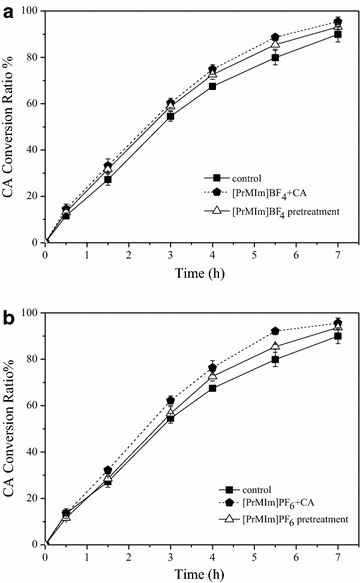
Effect of two kinds of ILs on CA conversion. CA conversion ratios when *ASP* cells are pre-incubated with 0.5 % (v/v) two kinds of ILs and without ILs. The ILs-treated *ASP* cells were washed with PBS for three times and resusupended in the same buffer, respectively. The conversion ratios of CA were determined by HPLC analysis. **a** CA conversion in presence of [PrMIm]BF_4_ or pretreatment. **b** CA conversion in presence of [PrMIm]PF_6_ or pretreatment. Standard deviations are indicated by error bars or are within *each symbol*

**Fig. 2:**
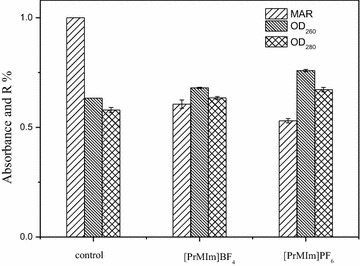
Effect of two kinds of ILs on the activity and permeability of cells. *ASP* cells were pretreated for 2 h by two kinds of ILs sat 0.5 % (v/v) and washed with PBS for three times, respectively. The relative activity compared with control was determined by MAR value. The permeability of cells can be characterized by nucleic acid and protein leakage, respectively. Standard deviations are indicated by *error bars* or are within *each symbol*

In general, apparent steroid-transforming activity is not only determined by the quantity of steroid-transforming enzyme and its activity but also by the substrate availability and membrane permeability of steroids [25]. In this case, *ASP* cells were pretreated with two kinds of ILs and washed for three times with PBS, which ensured no solubilization of ILs to the steroids. The effects among the ILs containing system, the ILs pretreated-cell system, and the control cells (no ILs and no ILs pretreatment) on steroid biotransformation were compared, and the results are shown in Fig. 1a, b. Firstly, conversion of cortisone acetate (CA) in ILs-containing system was higher than that in control group, which proved that ILs can enhance the conversion of steroid. Then, conversion of the substrate of the ILs pretreated-cell system was higher than that of the control group, suggesting that ILs can enhance steroid conversion after the cells were treated by ILs. To explore the mechanism, we further investigated the effect of ILs on cells activity. As shown in Fig. 2, after both kinds of ILs pretreatment, cells activity reduced approximately 45 % of the control cells. Interestingly, the conversion of CA by ILs pretreated cells was increased, whereas cells activity decreased. Thus, we speculated that membrane permeability was altered. Moreover, what should be noticed from Figs. 1a, b and 2 is that there was not obvious difference on steroid conversion and the conversion rate between hydrophobic [PrMIm]PF_6_ pretreated-cell system and hydrophilic [PrMIm]BF_4_ pretreated-cell system, however, [PrMIm]PF_6_ pretreated-cells exhibited lower activity than [PrMIm]BF_4_ pretreated-cells, we speculated it might due to [PrMIm]PF_6_ could increase membrane permeability in larger degree than [PrMIm]BF_4_. The leakage of cytoplasmic inclusions was determined under UV at 260 and 280 nm to study the effect of ILs on *ASP* membrane permeability, and the result is shown in Fig. 2. The contents of cytoplasmic inclusions in the supernatant increased in both types of ILs pretreated cells, indicating that the cell membrane permeability was enhanced.

### Alteration of the cell ultrastructure

**Fig. 3:**
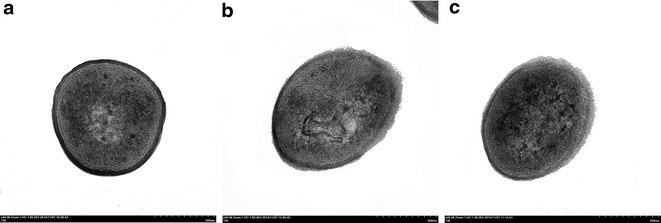
TEM images showing cell morphological changes. **a** Cells without treatment (control). **b** Cells pre-treated with 2.5 % (v/v) [PrMIm]PF_6_. **c** Cells pre-treated with 2.5 % (v/v) [PrMIm]BF_4_

The TEM micrograph directly showed the morphology alteration of pretreated cells. Figure 3a reveals that the control cells (without ILs) were rod-like, smooth, and intact, with the typical appearance of rod bacterium from different cross-sections. After cells exposure to [PrMIm]PF_6_ and [PrMIm]BF_4_ [2.5 %(v/v), respectively] for 2 h, wrinkles, gross creases, and several tiny pores were observed clearly (Fig. 3b, c). Further comparison indicated that the damage degree caused by hydrophobic [PrMIm]PF_6_ on the cell envelope is more serious than that by hydrophilic [PrMIm]BF_4_. This finding could be due to the fact that hydrophobic ILs easily binds to lipids and fatty proteins of the cell envelope through hydrogen bonds compared with hydrophilic ILs [14].

### ILs affects cell lipid composition

**Fig. 4:**
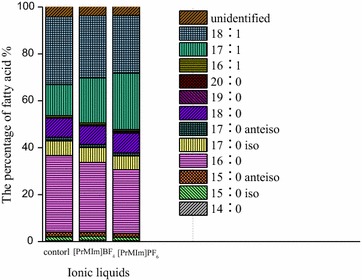
Effect of two kinds of ILs on fatty acid composition of cells. The percentage of loss total lipids (*triangle*) was extracted from samples of lyophilized cells (200 mg each) with 100 mL chloroform/methanol (2:1, v/v). The extracts were separated by filtration, evaporated, methanolyzed. The fatty acid contents of two kinds of ILs treated cell membrane and untreated cells were determined using GC–MS

Lipids and proteins are the material basis of cell membrane permeability. Thus, we further investigated the effect of [PrMIm]PF_6_ and [PrMIm]BF_4_ [2.5 %(v/v)] on the lipids of the *ASP* cell membrane (Fig. 4). The preparations differed to a considerable extent in terms of lipid concentrations, with cells being exposed to different ILs. The percentage of lost lipids in cells exposed to [PrMIm]PF_6_ was 19.9 %, which is approximately twice higher than that exposed to [PrMIm]BF_4_. This result may due to lipophilic [PrMIm]PF_6_ combining more easily to lipids than the hydrophilic [PrMIm]BF_4_.

GC analysis data showed that fatty acid play a vital role in cell membrane lipid, the loss of the cell membrane lipids caused a damage of cellular function, such as disorders of phospholipid bilayer, variation of cell membrane components and composition. As shown in Table 1, the quantity of fatty acid components of [PrMIm]PF_6_-pretreated cells and [PrMIm]BF_4_-pretreated cells was both lower than that of control cells (without ILs pretreatment). This finding is in agreement with the low overall content of non-covalently bound lipids in [PrMIm]PF_6_-pretreated cells. As shown in Fig. 4, C16 and C18 were the main fatty acid components of the *ASP* cell membrane, as evidenced by both fatty acids composing approximately 80 % of the total lipids in all preparations. The relative percentage of C17:1 in the [PrMIm]BF_4_-cells increased by 44 %, whereas that in [PrMIm]PF_6_-cells increased by 80 %. The increase of the relative percentage of C17:1 was caused by the binding of ILs with polar head group of cell membrane lipid [26]. Other variations in the fatty acid patterns were not as evident as those stated above.

**Table 1:** Effect of two ILs on contents of total lipids

Ionic liquids	The contents of total lipids (mg/g)
Control	46.62 ± 1.15
[PrMIm]BF_4_	42.75 ± 0.62
[PrMIm]PF_6_	37.33 ± 1.20

The content of unsaturated fatty acid cells and long-chain fatty acids of ILs treated cells were significantly altered. The unsaturated fatty acid content in [PrMIm]PF_6_-pretreated cells increased by 14.9 %, whereas that of [PrMIm]BF_4_-pretreated cells increased by 8.18 % compared with that in control cells. The content of long-chain fatty acids in [PrMIm]PF_6_-pretreated cells and [PrMIm]BF_4_-pretreated cells decreased by 10.88 and 6.11 %, respectively. All of these data reveal that unsaturated fatty acids increase and long-chain fatty acids decrease, thereby cell membrane improving fluidity and further enhancing the cell permeability to the substrate and the nutrient. These conclusions are consistent with Fig. 4.

### ILs affects cell protein composition

**Fig. 5:**
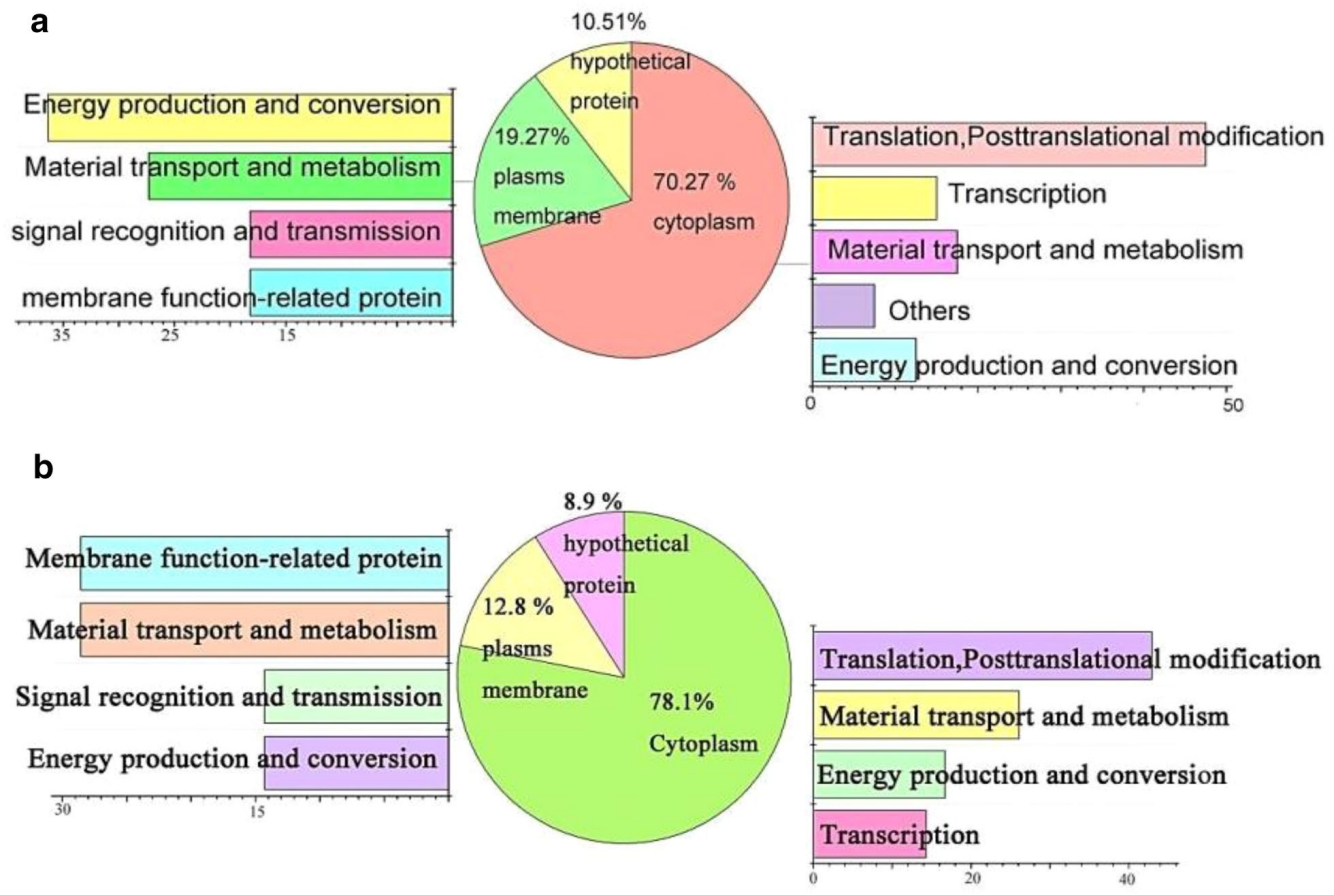
The location and function of reduced protein. Subcellular localization of the reduced proteins of two kinds of ILs treated cells and control cells (no ILs treatment). **a** Reduced proteins of [PrMIm]BF_4_-treated cell. **b**  Reduced proteins of [PrMIm]PF_6_-treated cell. The reduced proteins were determined by LC–MS/MS analysis, and peptide mass fingerprint was searched in NCBI

The protein contents of cells treated with or without 2.5 % (v/v) [PrMIm]PF_6_ or [PrMIm]BF_4_ were studied (Fig. 5). The percentage of lost protein was observed in all preparations.

Proteomic profiling of three group cells, *ASP* cells untreated and treated with 2.5 % (v/v) [PrMIm]PF_6_ and 2.5 % (v/v) [PrMIm]BF_4_, was performed. ILs affect protein composition; 57 proteins apparently reduced in [PrMIm]PF_6_-pretreated cells compared with those in the control, whereas 55 proteins reduced in [PrMIm]BF_4_-pretreated cells. The characteristics of the reduced proteins in both preparations are summarized in Fig. 5a, b.

Differential proteins were analyzed according to protein localization and functions. As shown in Fig. 5a, 70.27 % of 57 reduced proteins in [PrMIm]PF_6_-treated cells were located in the cytoplasm; 47.5 % of this amount functioned in translation, posttranslational, and modification. Quintiles were approximately located in the plasma membrane and mainly functioned in energy production and conversion; these quintiles including ATP synthase F0F1 subunit alpha, ATP F0F1 synthase subunit beta, and ATP-dependent Clp protease ATP-binding protein. For [PrMIm]BF_4_-treated cells 78.1 % of 55 reduced proteins were situated in the cytoplasm and functioned similarly to cells treated by [PrMIm]PF_6_. However, only 12.8 % were located in the plasma membrane, and these proteins mainly participated in membrane function-related protein and material transport and metabolism. Approximately 10 % of the proteins were hypothetical in both preparations.

### The functions of differential proteins in metabolic pathway

**Fig. 6:**
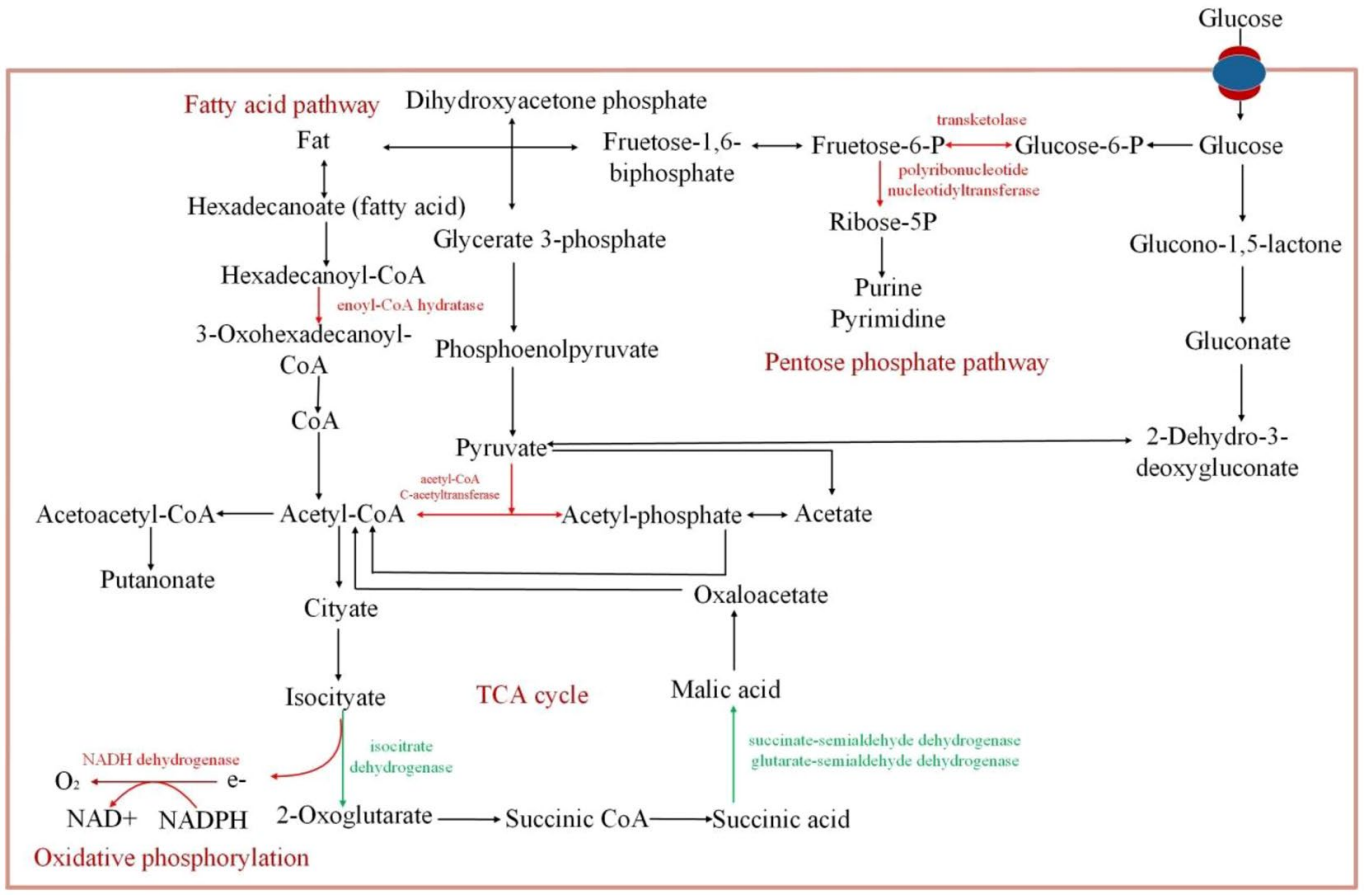
The functions of differential proteins in metabolic pathway. Identified differential proteins were searched according to KEGG, and the picture of metabolic pathways is drawn by Microsoft Visio. The function of increased proteins pathways and enzymes are marked in *red* in pathway, whereas the function of reduced proteins and enzymes are marked in *green* in pathway

Considering ILs may penetrate cells, and the side chain of ILs may bind with hydrophilic parts with lipophilic lipid of cell membrane; ILs might affect the intracellular activities, for instance, protein degeneration, DNA damage, monooxygenase/AMP deaminase etc. Thus, further analysis of the functions of differential proteins associated with KEGG metabolic pathway is shown in Fig. 6. The reduced proteins of [PrMIm]PF_6_-treated cells included isocitratelyase, succinate-semialdehyde dehydrogenase, glutarate-semialdehyde dehydrogenase, and acetyl-CoA C-acetyltransferase, which mostly functione in the citric acid cycle. By contrast, the reduced proteins of [PrMIm]BF_4_-treated cells were gamma-glutamylputrescine oxidase and monoamine oxidase, which are functional for arginine, proline, histidine, tryptophan, and tyrosine metabolism.

In addition to the reduced proteins, the increased proteins were further analyzed according to their functions in metabolic pathways (red line in Fig. 6). For [PrMIm]PF_6_-treated cells, transketolase increased. The increased proteins enoyl–CoA hydratase functioned in the transformation of fatty acids into palmitic acid, and increased NADH dehydrogenase functioned in the phosphorylation pathway. These findings are consistent with the results in Fig. 4. For [PrMIm]BF_4_-treated cells, increased transketolase functioned in transforming β-d-fructose into Ricose-5P, which is a vital step in the pentose phosphate pathway.

The functions of differential proteins of two kinds of ILs-treated cells in the metabolic pathways are different. The functions of proteins identified in [PrMIm]BF_4_-treated cells mainly focus on the synthesis of purines and pyrimidines and oxidative phosphorylation, whereas those in [PrMIm]PF_6_-treated cells functioned the fatty acid pathway and the citric acid cycle. These variations could be caused by different hydrophilic properties of the two kinds of ILs.

## Discussion

Studies revealed that the application of ILs in pharmaceuticals enhances the steroid conversion [1, 13, 27, 28]. This enhancement is mainly demonstrated by the high stability and activity of enzymes as well as the solubilization of water-insoluble substrate as a result of the unique properties of ILs [3, 4, 25]. This paper studied the effect of two imidazolium-based ILs on the characteristics of *ASP* cells, particularly the effect of ILs on cell envelope permeability to further investigate the mechanism underlying such changes. We mainly focused on lipids and proteins.

The steroidal conversion rates of the ILs containing system and ILs pretreated *ASP* cells were significantly improved. In the pretreated system, ILs did not solubilize CA by washing the pretreated cells for three times. This finding indicates that increase in steroid conversion was not only due to substrate solubilization. Hence, improvement in substrate solubility caused by ILs is not the influential factor for biotransformation in this experiment. This fact suggests that substrate availability was not distinct between the two experimental groups when CA contents are equal. Thus, we hypothesize that ILs improved biotransformation through other modes besides solubilization.

Activity, substrate utilization, and cell membrane permeability to steroids were three vital factors during steroid bioconversion [25]. In this paper, CA conversion was increased under both ILs containing environment and ILs pretreated conditions regardless of solubilization of CA substrate, whereas cells activity decreased. These results indicate a significant increase in cell permeability to CA. In this work, leakage of cytoplasmic inclusions was observed after treatment by two ILs, thus proving that the permeability of cell membrane improved with the increase in cell inclusion leakage. Moreover, hydrophobic ILs caused a more serious damage to cell membrane and cell activity in comparison with hydrophilic ILs because hydrophobic ILs more easily bind with lipid, further destroying the structure of cell envelope and cell integrity [15]. The TEM micrographs showed these alterations intuitively, ILs pretreated cells showed wrinkles, gross creases, and several tiny pores, whereas control cells maintained cell integrity. These observations illustrate that ILs pretreatment alters the sharpness and surface structure of cells.

The thickness of the cell membrane is negatively correlation with permeability [29, 30]. Membrane thickness is related with the length of the cell membrane fatty acyl chain. The changes in cell envelope components are closely related to the integrity and permeability of the membrane [31–33]. As shown in Fig. 4 and Table 1, the structure of the cell lipid bilayer remained. However, after pretreatment, the cell membrane became evidently thinner, and the content of long-chain fatty acid decreased, whereas the amount of unsaturated fatty acids increased. The high accumulation of extracellular unsaturated fatty acids was attributed to the enhanced release of lipids from the cell envelope after IL pretreatment. This increased release may contribute to altered permeability. Thin and unsaturated cell membranes are more permeable relative to thicker and more saturated membranes [30]. The result indicates that unsaturated fatty acids increased, whereas long-chain fatty acids decreased. Both changes are significantly related to membrane fluidity and permeability.

The leakage of proteins was observed in *ASP* after treatment by two ILs. This leakage may be due to the unique chemical structure of ILs that can interact with cell protein directly and increase cell envelope permeability [34]. Numerous proteins function in maintaining cell growth and vitality; therefore, we speculate that protein leakage is closely related to changes in activity and cell permeability. However, the specific mechanism of ILs interaction with cell membrane proteins has yet to be illustrated and needs further study.

In the comparison of the proteomic profiling of *ASP* control cells with that of cells exposed to ILs, we found that, the majority of reduced proteins were located in the cytoplasm and mainly functioned in the replication and transcription of DNA, transport and metabolism of substances, and energy conversion, among others. A smaller portion of the proteins was located on the cell membrane but performed important functions in maintaining cellular morphology and membrane functions, as exemplified by ATP F0F1 synthase subunit beta, monosaccharide ABC transporter, and ATP-dependent Clp protease ATP-binding protein. This function may be due to ILs connecting with proteins through hydrogen bonds, further causing the protein to split. Moreover, the binding between ILs and the lipids of the membrane skeleton is attributed to cytoskeleton loss.

Approximately quintile of reduced proteins were located on the cell membrane in [PrMIm]PF_6_-pretreated cells, whereas only 12.8 % were observed in [PrMIm]BF_4_-pretreated cells. These different findings may be due to the easier binding of the hydrophobic [PrMIm]PF_6_ with hydrophobic amino acids or proteins, as well as the distinct positions of different proteins in the cell.

The functions of differential proteins in metabolic pathway of two ILs-treated *ASP* cells were further analyzed. As shown in Fig. 6, differential proteins mainly functioned in the pentose phosphate pathway, fatty acid synthesis pathways, citric acid cycle, and oxidative phosphorylation. The increased proteins mainly functioned in pentose phosphate pathway and oxidative phosphorylation, these pathways can provid energy and material bases for the synthesis of purine and pyridine and fatty acids [35]. The different functions of the differential proteins in two kinds of ILs-treated cells maybe because that hydrophobic [PrMIm]PF_6_ more easily binds with the lipid and affects lipid composition. This is consistent with the results of fatty acid component analysis according to Fig. 4.

We speculated two main causes of the decrease in activity. On the one hand, the interaction between the ion core of ILs and proteins and lipids induced the components to fall from membrane skeleton, thereby enhancing cell envelope permeability until the structure was destroyed and further decreasing the content and activity of cells [36, 37]. On the other hand, ILs affects activity conformation of proteins that perform key functions in cells metabolism activity [38].

## Conclusion

We found that ILs altered lipid and protein profiles, leading to altered membrane permeability and subsequently promoting the steroid transforming activity. Further research provided theoretical data for the material basis of cell physiological functions changes. We used LC–MS analysis to identify the types and functions of distinct cell proteins of 2.5 % (v/v) [PrMIm]PF_6_-treated cells, [PrMIm]BF_4_-treated cells, and the control cells. The mechanisms of ILs with cells themselves are complex and exert an effect not only on solubilization but also on permeability. These reactions offer important scientific significance and research value.

## Additional files

10.1186/s12934-016-0518-3 The identification of the control cells proteins.
10.1186/s12934-016-0518-3 The identification of the [PrMIm]BF_4_-treated cells proteins.
10.1186/s12934-016-0518-3 The identification of the [PrMIm]PF_6_-treated cells proteins.
10.1186/s12934-016-0518-3 The bioinformatics analysis of *ASP* reduced proteins affected by ILs. NCBI searches were performed in Uniprot protein database for analysis of the location and function classify.

## Abbreviations

IL(s): ionic liquid(s)
*ASP*: *Arthrobacter simplex* CPCC 140451
[PrMIm]BF_4_: 1-propyl-3-methylimidazolium tetrafluoroborate
[PrMIm]PF_6_: 1-propyl-3-methylimidazolium hexafluorophosphate
TEM: transmission electron microscopy
PBS: KH_2_PO_4_–NaOH buffer
CA: cortisone acetate
MAR: metabolic activity retention
UV: ultraviolet
FID: flame ionization detector
PMSF: phenylmethylsulfonyl fluoride
KEGG: Kyoto Encyclopedia of Genes and Genomes
