# Supplementary material for: Effects of two kinds of imidazolium-based ionic liquids on the characteristics of steroid-transformation Arthrobacter simplex
Source: Microb Cell Fact. 2016 Jul 1;15:118. doi: 10.1186/s12934-016-0518-3 (PMC4930596; doi:10.1186/s12934-016-0518-3)
Supplement: Supplementary file 1 — 10.1186/s12934-016-0518-3 The identification of the control cells proteins. [file 12934_2016_518_MOESM1_ESM.pdf]

Additional file 1: The identification of the control *ASP* proteins

Accession	Coverage	PSMs	Peptides	AAs	MW [kDa]	calc. pI	Score	Description
gi672940878	70.35	234	26	543	56.5	4.86	685.38	chaperonin GroEL [Pimelobacter simplex]
gi672940874	51.93	129	20	622	65.8	4.68	379.91	molecular chaperone DnaK [Pimelobacter simplex]
gi219861633	25.37	105	2	67	7.2	4.88	129.52	cold-shock DNA-binding domain protein (plasmid) [Arthrobacter chlorophenolicus A6]
gi443482191	25.37	66	2	67	7.2	4.94	129.45	cold-shock DNA-binding protein family protein [Arthrobacter nitrophenolicus]
gi116610479	6.06	51	1	264	27.9	4.96	112.25	short-chain dehydrogenase/reductase SDR [Arthrobacter sp. FB24]
gi672940880	22.28	43	12	736	79.9	4.86	109.71	catalase/peroxidase KatG [Pimelobacter simplex]
gi651431914	2.35	37	1	597	64.4	4.67	108.74	ABC transporter substrate-binding protein [Arthrobacter sanguinis]
gi742859324	7.49	159	1	307	33.6	7.06	103.29	LysR family transcriptional regulator [Arthrobacter sp. W1]
gi928488106	3.97	27	1	277	30.0	5.72	102.30	flagellar biosynthesis protein FliA [Arthrobacter alpinus]
gi323471231	4.13	9	1	387	41.1	4.75	95.81	D-mannitol 1-phosphate 5-dehydrogenase [Arthrobacter phenanthrenivorans Sphe3]
gi517602201	16.54	60	1	127	13.7	4.59	86.47	MULTISPECIES: glyoxalase [Arthrobacter]
gi749402670	2.90	53	1	552	57.1	5.72	83.97	acetolactate synthase [Arthrobacter sp. AK-YN10]
gi1906824	11.26	34	4	462	50.0	11.56	82.31	hypothetical protein [Pimelobacter simplex]
gi674647015	11.01	17	6	545	57.1	4.84	81.12	60 kDa chaperonin 1 [Arthrobacter sp. 11W110_air]
gi737783399	3.41	35	1	410	44.0	5.21	80.56	N-isopropylammelide isopropylaminohydrolase [Arthrobacter sp. 35W]
gi939050621	30.40	22	4	125	13.0	4.48	80.41	50S ribosomal protein L7 [Arthrobacter sp. JCM 19049]
gi910737718	3.79	36	1	422	42.8	9.13	72.19	putative ankyrin-containing lipoprotein Lxx09580 [Arthrobacter sp. Hiyo4]
gi476402309	23.47	22	1	98	10.4	5.01	70.84	co-chaperonin GroES [Arthrobacter crystallopoietes BAB-32]
gi919218844	1.25	5	1	1116	125.2	5.47	68.53	hypothetical protein [Arthrobacter sp. YC-RL1]
gi939050482	4.45	17	1	292	32.3	4.64	56.56	hypothetical protein [Arthrobacter sp. JCM 19049]
gi119949505	4.39	33	1	319	35.2	7.21	54.65	putative transcriptional regulator, LysR family [Arthrobacter aurescens TC1]
gi737800623	5.67	18	1	300	32.6	5.43	53.98	SPFH/Band 7/PHB domain protein [Arthrobacter castelli]
gi651495418	4.21	38	1	309	34.2	10.10	53.49	pseudouridine synthase [Arthrobacter sp. H20]
gi674645160	6.39	14	1	360	37.6	5.14	52.91	putative enoyl-CoA hydratase [Arthrobacter sp. 11W110_air]
gi518311313	4.21	34	1	309	34.3	9.73	49.79	hypothetical protein [Arthrobacter sp. TB 23]
gi930828157	2.95	3	1	440	47.5	6.90	49.09	SAM-dependent methyltransferase [Arthrobacter arilaitensis]
gi651440580	2.47	4	1	486	54.6	5.44	48.01	hypothetical protein [Arthrobacter sp. H14]
gi916835164	3.47	23	1	404	44.3	5.88	46.89	ABC transporter [Arthrobacter sp. H14]
gi651495309	8.33	12	1	264	28.8	7.55	45.86	glycerophosphodiester phosphodiesterase [Arthrobacter sp. H20]
gi403228123	4.14	27	1	338	36.2	5.59	45.68	glycine betaine/carnitine/choline transport ATP-binding protein OpuCA [Arthrobacter sp. Rue
gi823667653	3.50	22	1	400	43.1	4.96	45.32	ABC transporter [Arthrobacter sp. YC-RL1]
gi518311951	6.39	3	1	219	23.2	6.64	40.96	MULTISPECIES: NUDIX domain-containing protein [Arthrobacter]
gi723607705	3.11	2	1	418	46.6	5.38	39.40	creatine amidinohydrolase [Arthrobacter sp. PAMC25486]
gi651429323	12.58	8	1	159	17.8	8.06	39.25	hypothetical protein [Arthrobacter sanguinis]
gi742072721	3.60	17	1	333	35.9	6.47	38.98	LacI family transcriptional regulator [Arthrobacter sp. MWB30]
gi937262120	8.94	14	1	235	24.7	9.38	38.65	50S ribosomal protein L1 [Arthrobacter sp. Edens01]
gi910743568	4.01	15	1	324	34.9	4.74	37.69	DNA repair protein RecN [Arthrobacter sp. Hiyo8]
gi651444347	3.59	1	1	390	42.5	5.11	36.01	exonuclease SbcD [Arthrobacter nicotinovorans]
gi652425290	8.97	11	1	145	16.2	6.00	34.21	MarR family transcriptional regulator [Arthrobacter castelli]
gi916813687	4.53	14	1	309	34.3	6.28	33.14	hypothetical protein [Arthrobacter nicotinovorans]
gi767258484	7.11	14	4	830	91.6	5.55	32.42	NDP-hexose 4-ketoreductase [Arthrobacter sp. IHBB 11108]
gi910747080	15.38	34	1	104	10.8	11.18	31.97	hypothetical protein AHiyo8_47820 [Arthrobacter sp. Hiyo8]
gi652425065	10.66	1	1	122	13.4	10.40	31.06	50S ribosomal protein L14 [Arthrobacter castelli]
gi116609762	8.24	13	5	740	79.9	5.05	30.18	isocitrate dehydrogenase, NADP-dependent [Arthrobacter sp. FB24]
gi403230217	12.22	10	4	483	52.3	4.98	29.44	ATP synthase subunit beta [Arthrobacter sp. Rue61a]
gi651503952	7.56	4	1	172	19.3	8.21	29.30	hypothetical protein [Arthrobacter sp. 35W]
gi737786847	12.16	10	4	485	52.6	4.91	29.26	ATP synthase subunit beta [Arthrobacter albus]
gi757623099	4.54	14	2	595	63.1	6.49	28.73	HNH endonuclease [Arthrobacter sp. SPG23]
gi651457048	25.37	12	2	134	15.0	9.06	27.53	30S ribosomal protein S16 [Arthrobacter sp. 35/47]
gi927032930	21.77	6	2	124	12.9	4.51	27.10	50S ribosomal protein L7 [Arthrobacter sp. LS16]
gi651465980	16.60	9	3	235	25.0	9.36	27.05	50S ribosomal protein L1 [Arthrobacter sp. 35/47]
gi476402832	23.03	4	2	152	17.1	11.62	26.97	50S ribosomal protein L20 [Arthrobacter crystallopoietes BAB-32]
gi737809001	23.81	3	2	147	16.6	10.90	26.97	50S ribosomal protein L20 [Arthrobacter sp. H5]
gi551253914	20.09	8	3	234	24.8	9.29	26.85	50S ribosomal protein L1 [Arthrobacter sp. PAO19]

gi928487058	9.45	5	1	127	14.2	6.54	26.54	MerR family transcriptional regulator [Arthrobacter alpinus]
gi651431165	16.43	7	4	487	53.0	4.84	26.54	ATP synthase subunit beta [Arthrobacter sanguinis]
gi927296035	15.21	2	1	217	22.7	5.60	25.45	LuxR family transcriptional regulator [Arthrobacter sp. ERGS1:01]
gi323469212	2.95	1	1	713	78.1	5.55	24.91	DNA/RNA endonuclease G, NUC1 [Arthrobacter phenanthrenivorans Sphe3]
gi651434056	10.80	5	3	537	56.5	4.92	24.70	molecular chaperone GroEL [Arthrobacter sp. H41]
gi654813326	13.76	2	2	218	23.7	10.05	24.38	50S ribosomal protein L20 [Arthrobacter sp. MA-N2]
gi910746946	5.07	2	1	296	31.7	5.06	24.01	UTP--glucose-1-phosphate uridylyltransferase [Arthrobacter sp. Hiyo8]
gi740685683	5.37	7	1	428	46.4	6.06	23.79	ATP-binding protein [Arthrobacter sp. PAMC25486]
gi918221850	2.42	8	1	619	69.0	7.36	23.62	glycosyltransferase [Arthrobacter sp. I3]
gi654823558	5.34	8	1	262	27.1	9.85	23.31	oxidoreductase [Arthrobacter sp. I3]
gi189041365	18.85	4	2	122	13.3	10.15	22.84	RecName: Full=50S ribosomal protein L14
gi674644816	2.58	4	2	737	78.9	4.93	22.58	Polyribonucleotide nucleotidyltransferase [Arthrobacter sp. 11W110_air]
gi651431596	8.53	7	1	129	13.3	4.54	22.46	50S ribosomal protein L7/L12 [Arthrobacter sanguinis]
gi723610089	7.71	12	4	739	78.9	5.03	22.46	isocitrate dehydrogenase [Arthrobacter sp. PAMC25486]
gi551254729	12.65	11	1	166	18.4	5.81	22.19	hypothetical protein [Arthrobacter sp. PAO19]
gi651439383	1.68	1	1	597	64.0	5.21	22.03	peptidoglycan glycosyltransferase [Arthrobacter sp. H14]
gi674645749	1.14	1	1	1317	144.9	6.98	21.92	ATP-dependent RNA helicase HrpB [Arthrobacter sp. 11W110_air]
gi928487494	2.36	6	1	594	63.2	5.47	21.66	hypothetical protein AOC05_11905 [Arthrobacter alpinus]
gi740683298	5.72	4	2	507	54.3	7.49	21.60	LuxR family transcriptional regulator [Arthrobacter sp. PAMC25486]
gi640194107	4.03	3	1	472	53.7	8.25	21.58	hypothetical protein [Arthrobacter sp. 31Y]
gi742758238	14.48	9	1	145	15.6	5.00	21.48	hypothetical protein RM50_04295 [Arthrobacter phenanthrenivorans]
gi443480201	9.86	7	1	142	15.7	4.97	21.39	DNA-binding ferritin-like protein [Arthrobacter nitrophenolicus]
gi757625353	6.76	7	4	740	79.8	5.05	21.14	isocitrate dehydrogenase [Arthrobacter sp. SPG23]
gi910248756	10.77	1	1	195	21.2	6.20	21.12	DNA-binding protein [Arthrobacter siccitolerans]
gi654816002	1.53	2	1	848	93.5	5.16	21.02	DEAD/DEAH box helicase [Arthrobacter sp. UNC362MFTsu5.1]
gi908698500	6.91	11	1	188	19.6	10.30	20.95	Holliday junction resolvase [Arthrobacter sp. RIT-PI-e]
gi470220234	14.34	15	3	279	30.6	11.33	20.89	50S ribosomal protein L2 [Arthrobacter gangotriensis Lz1y]
gi470219995	6.68	6	3	614	66.1	4.74	20.81	chaperone protein DnaK [Arthrobacter gangotriensis Lz1y]
gi307745781	11.11	2	1	162	18.3	9.07	20.47	putative MarR-family transcriptional regulator [Arthrobacter arilaitensis Re117]
gi937259012	1.38	6	1	1158	123.6	5.27	20.41	1-pyrroline-5-carboxylate dehydrogenase [Arthrobacter sp. Edens01]
gi33571522	1.51	2	1	793	84.1	5.49	20.36	probable dehydrogenase/oxidase [Bordetella pertussis Tohama I]
gi737789341	2.56	4	2	742	80.1	4.92	20.32	polynucleotide phosphorylase [Arthrobacter albus]
gi545107935	2.56	5	2	1327	147.8	6.99	20.14	ATP-dependent helicase [Arthrobacter sp. AK-YN10]
gi765009954	4.41	3	1	454	47.7	5.20	19.97	4-aminobutyrate aminotransferase [Arthrobacter sp. A3]
gi759726407	10.23	8	3	606	63.8	5.12	19.91	acetyl-CoA carboxylase [Arthrobacter sp. I3]
gi517591758	5.76	4	1	191	20.2	5.01	19.63	phosphoribosylglycinamide formyltransferase [Arthrobacter sp. 135MFCol5.1]
gi542106946	4.74	8	3	739	79.6	5.03	19.32	isocitrate dehydrogenase [Arthrobacter sp. AK-YN10]
gi742752690	4.01	5	1	424	45.3	6.54	19.27	dehydrogenase [Arthrobacter phenanthrenivorans]
gi476400198	2.62	7	3	1296	142.8	6.25	19.13	DNA-directed RNA polymerase subunit beta' [Arthrobacter crystallopoietes BAB-32]
gi910739216	5.88	5	1	221	25.4	5.77	18.74	conserved hypothetical protein [Arthrobacter sp. Hiyo4]
gi737815326	5.32	9	3	827	91.4	5.55	18.74	NDP-hexose 4-ketoreductase [Arthrobacter sp. H14]
gi769942322	4.12	3	1	388	41.4	7.06	18.72	hypothetical protein [Arthrobacter sp. IHBB 11108]
gi737813326	3.47	5	1	432	47.5	4.65	18.42	HCC family HlyC/CorC transporter [Arthrobacter sp. H14]
gi757626101	8.81	6	1	159	17.6	5.01	18.33	DNA-binding protein [Arthrobacter sp. SPG23]
gi639129639	5.44	12	1	331	33.7	5.25	18.21	thiamine-monophosphate kinase [Arthrobacter sp. CAL618]
gi648573957	7.08	5	1	226	25.1	6.70	18.07	fructose 2,6-bisphosphatase [Arthrobacter sp. 162MFSha1.1]
gi651488500	3.72	3	1	619	66.1	5.59	18.02	hypothetical protein, partial [Arthrobacter sp. H20]
gi307745569	9.31	8	2	333	36.1	4.75	17.97	DNA-directed RNA polymerase alpha chain [Arthrobacter arilaitensis Re117]
gi636845165	5.46	5	1	293	31.7	5.15	17.65	aminoglycoside resistance protein, partial [Arthrobacter sp. TB 26]
gi654814371	4.22	5	1	474	50.2	6.06	17.48	aspartate ammonia-lyase [Arthrobacter sp. MA-N2]
gi759736473	1.93	3	1	880	92.0	7.21	17.35	hypothetical protein [Arthrobacter sp. L77]
gi930826750	2.76	2	1	435	44.9	5.62	17.16	glutamate-1-semialdehyde aminotransferase [Arthrobacter arilaitensis]
gi759730994	3.34	5	1	539	59.5	5.57	17.11	choline oxidase [Arthrobacter sp. L77]
gi823666499	2.62	7	2	1296	143.2	6.61	17.06	DNA-directed RNA polymerase subunit beta' [Arthrobacter sp. YC-RL1]
gi927293872	3.36	3	2	745	79.7	4.98	16.80	polynucleotide phosphorylase [Arthrobacter sp. ERGS1:01]

gi910748533	4.72	4	1	233	25.7	5.12	16.68	30S ribosomal protein S1 [Arthrobacter sp. Hiyo8]
gi476399388	4.49	1	1	334	35.5	9.41	16.68	hypothetical protein D477_018711 [Arthrobacter crystallopoietes BAB-32]
gi742754810	6.56	3	2	488	53.1	5.02	16.60	S-adenosyl-L-homocysteine hydrolase [Arthrobacter phenanthrenivorans]
gi916782233	5.34	8	1	337	36.4	5.95	16.56	hypothetical protein [Arthrobacter sp. 35W]
gi654824189	2.84	2	1	915	94.8	7.50	16.56	phosphoenolpyruvate synthase [Arthrobacter sp. I3]
gi919218798	2.99	1	1	536	55.8	11.50	16.49	hypothetical protein [Arthrobacter sp. YC-RL1]
gi651502944	2.52	3	3	1309	144.7	6.87	16.49	ATP-dependent helicase [Arthrobacter sp. 35W]
gi443483172	6.57	2	2	426	46.2	5.26	16.44	ATP-dependent protease ATP-binding subunit ClpX [Arthrobacter nitrophenolicus]
gi652424247	2.79	3	1	537	55.4	9.50	16.19	hypothetical protein [Arthrobacter castelli]
gi640202524	3.22	8	2	962	102.1	5.68	16.18	cell division protein FtsK [Arthrobacter sp. 31Y]
gi654818882	4.78	5	1	335	36.5	5.72	16.00	aminoglycoside resistance protein [Arthrobacter sp. UNC362MFTsu5.1]
gi930826745	1.96	3	1	1070	117.1	5.11	15.92	ATP-dependent DNA helicase [Arthrobacter arilaitensis]
gi470220608	8.92	3	2	437	45.8	5.27	15.91	pyrimidine-nucleoside phosphorylase [Arthrobacter gangotriensis Lz1y]
gi521358281	9.97	6	2	311	33.2	5.55	15.91	prephenate dehydratase [Streptomyces rapamycinicus NRRL 5491]
gi511534741	2.55	1	1	470	51.1	6.47	15.81	transcriptional activator pmfR (plasmid) [Arthrobacter nicotinovorans]
gi927293758	4.66	5	1	451	48.6	11.02	15.80	hypothetical protein AL755_06480 [Arthrobacter sp. ERGS1:01]
gi919219015	7.80	1	1	282	31.3	9.61	15.74	hypothetical protein [Arthrobacter sp. YC-RL1]
gi323471431	5.62	2	1	409	41.4	6.46	15.62	3-oxoacyl-(acyl-carrier-protein) synthase II (plasmid) [Arthrobacter phenanthrenivorans Sphe
gi654811873	6.54	4	1	214	23.3	7.34	15.47	hypothetical protein [Arthrobacter sp. MA-N2]
gi517598984	4.63	3	1	475	50.3	6.21	15.41	glycoside hydrolase [Arthrobacter sp. 162MFSha1.1]
gi910738525	3.01	5	1	499	53.1	7.65	14.85	probable NADH dehydrogenase [Arthrobacter sp. Hiyo4]
gi651479522	3.59	1	1	529	57.5	5.19	14.73	GTP-binding protein [Arthrobacter sp. Br18]
gi651434734	3.80	2	1	553	62.0	5.17	14.71	catalase [Arthrobacter sp. H41]
gi742855609	5.96	3	2	520	55.5	9.04	14.67	signal recognition particle protein [Arthrobacter sp. W1]
gi119950562	4.98	6	2	422	46.6	8.68	14.65	L-lactate dehydrogenase [Arthrobacter aurescens TC1]
gi551256381	4.26	20	1	517	55.2	8.24	14.58	hypothetical protein [Arthrobacter sp. PAO19]
gi654826121	2.70	2	1	592	64.2	5.40	14.57	glyoxylate carboligase [Arthrobacter sp. H5]
gi119950023	2.25	1	1	488	54.6	6.86	14.46	cardiolipin synthetase [Arthrobacter aurescens TC1]
gi742759528	1.91	2	1	889	95.4	6.24	14.43	phosphoenolpyruvate synthase [Arthrobacter phenanthrenivorans]
gi917013195	12.10	2	1	157	17.0	10.30	14.24	hypothetical protein [Arthrobacter sanguinis]
gi651431168	1.28	2	1	545	59.1	5.05	14.22	ATP synthase subunit alpha [Arthrobacter sanguinis]
gi816850540	16.02	5	2	206	23.3	5.45	14.20	superoxide dismutase [Pimelobacter simplex]
gi640203244	4.50	2	1	489	51.7	5.21	14.14	hypothetical protein [Arthrobacter sp. 31Y]
gi517599113	2.02	1	1	892	95.9	5.34	14.12	alanine--tRNA ligase [Arthrobacter sp. 162MFSha1.1]
gi359306031	3.93	5	2	865	95.8	5.35	14.03	chaperone ClpB [Arthrobacter globiformis NBRC 12137]
gi640201864	4.97	4	1	322	34.6	4.86	14.02	aldose epimerase [Arthrobacter sp. 31Y]
gi654811523	0.74	1	1	1348	141.5	6.98	13.98	hypothetical protein [Arthrobacter sp. MA-N2]
gi515767041	7.41	3	2	526	56.2	9.47	13.91	signal recognition particle protein [Arthrobacter sp. M2012083]
gi910251060	8.18	1	1	220	23.5	8.07	13.87	DNA-3-methyladenine glycosidase [Arthrobacter siccitolerans]
gi518312892	25.37	5	1	67	7.2	4.88	13.86	MULTISPECIES: cold-shock protein [Arthrobacter]
gi765009289	4.48	9	1	201	20.8	9.70	13.82	hypothetical protein [Arthrobacter sp. A3]
gi937258697	2.56	2	1	897	96.0	5.36	13.82	alanine--tRNA ligase [Arthrobacter sp. Edens01]
gi323468360	1.20	2	1	830	90.4	6.43	13.73	ATP-dependent DNA helicase PcrA [Arthrobacter phenanthrenivorans Sphe3]
gi765009240	21.70	1	1	106	11.6	10.52	13.57	DNA-binding protein [Arthrobacter sp. A3]
gi651504265	10.21	5	2	235	24.9	9.26	13.55	50S ribosomal protein L1 [Arthrobacter sp. 35W]
gi918469314	2.81	2	1	676	74.6	6.44	13.54	glycogen debranching protein [Arthrobacter crystallopoietes]
gi470220534	6.41	4	1	312	34.5	8.79	13.44	glycosyltransferase [Arthrobacter gangotriensis Lz1y]
gi511534683	2.37	2	1	337	35.8	5.82	13.43	putative LacI-family transcriptional regulator (plasmid) [Arthrobacter nicotinovorans]
gi517604588	3.82	1	1	262	26.7	6.80	13.37	3-oxoacyl-ACP reductase [Arthrobacter sp. 131MFCol6.1]
gi651484943	5.05	2	1	436	45.4	6.34	13.30	acetyl-CoA acetyltransferase [Arthrobacter sp. Br18]
gi823667929	3.43	1	1	613	66.3	7.09	13.26	long-chain fatty acid--CoA ligase [Arthrobacter sp. YC-RL1]
gi476400133	5.86	1	1	222	25.0	7.28	13.20	Transcriptional regulator [Arthrobacter crystallopoietes BAB-32]
gi742858244	13.08	2	1	107	12.0	10.68	13.17	hypothetical protein [Arthrobacter sp. W1]
gi651431081	0.92	2	1	1199	130.5	5.07	13.11	chromosome segregation protein SMC [Arthrobacter sanguinis]
gi910695627	6.54	4	1	260	27.3	8.07	13.08	UPF0001 protein Cgl2153/cg2364 [Arthrobacter sp. Hiyo6]

gi654826881	4.26	4	1	399	42.5	7.05	12.95	pilus assembly protein CpaE [Arthrobacter sp. H5]
gi910250709	3.14	2	1	446	45.9	8.22	12.87	3-ketoacyl-ACP reductase [Arthrobacter siccitolerans]
gi651504005	5.00	4	1	300	32.5	5.12	12.81	UTP--glucose-1-phosphate uridylyltransferase [Arthrobacter sp. 35W]
gi517601711	1.38	5	1	1157	126.8	5.66	12.80	ATP-binding protein [Arthrobacter sp. 162MFSha1.1]
gi915933404	4.56	3	1	285	31.1	5.38	12.80	chromosome partitioning protein [Arthrobacter globiformis]
gi917013410	6.13	8	1	163	17.1	10.29	12.76	50S ribosomal protein L15 [Arthrobacter sanguinis]
gi639129536	8.86	8	2	237	24.9	5.58	12.76	copper oxidase [Arthrobacter sp. CAL618]
gi910738261	16.38	5	1	116	12.6	5.03	12.71	conserved hypothetical protein [Arthrobacter sp. Hiyo4]
gi443479714	12.42	2	2	330	35.0	5.33	12.70	oxidoreductase, aryl-alcohol dehydrogenase like protein [Arthrobacter nitrophenolicus]
gi908740221	4.64	5	1	323	33.6	4.44	12.67	hypothetical protein [Arthrobacter arilaitensis]
gi910746129	7.50	7	2	440	47.1	10.52	12.62	hypothetical protein AHiyo8_38310 [Arthrobacter sp. Hiyo8]
gi767259001	1.31	3	1	612	67.0	5.12	12.60	aspartyl-tRNA synthetase [Arthrobacter sp. IHBB 11108]
gi517593335	6.59	3	2	410	44.3	9.35	12.59	DNA polymerase IV [Arthrobacter sp. 135MFCol5.1]
gi742755147	2.64	1	1	455	47.1	5.77	12.55	transglycosylase [Arthrobacter phenanthrenivorans]
gi742758858	1.23	7	1	1134	120.7	5.35	12.54	histidine kinase [Arthrobacter phenanthrenivorans]
gi651443967	9.79	4	2	235	24.9	9.25	12.53	50S ribosomal protein L1 [Arthrobacter nicotinovorans]
gi517608893	9.09	4	1	209	21.2	4.86	12.49	hypothetical protein [Arthrobacter sp. 161MFSha2.1]
gi786034325	3.59	2	2	1003	109.2	6.47	12.49	glutamine-synthetase [Arthrobacter chlorophenolicus]
gi551254024	3.93	2	1	280	28.6	4.89	12.42	pyrroline-5-carboxylate reductase [Arthrobacter sp. PAO19]
gi919218795	4.58	4	1	262	29.4	10.61	12.42	hypothetical protein [Arthrobacter sp. YC-RL1]
gi927294171	6.75	4	1	237	25.4	5.19	12.22	haloacid dehalogenase [Arthrobacter sp. ERGS1:01]
gi928486771	1.18	3	1	760	81.1	5.36	12.21	hypothetical protein AOC05_06795 [Arthrobacter alpinus]
gi737787162	4.05	2	2	740	80.7	4.87	12.20	isocitrate dehydrogenase [Arthrobacter albus]
gi765005689	1.61	3	1	746	81.4	5.68	12.18	ATPase AAA [Arthrobacter sp. A3]
gi651431090	12.23	2	1	188	20.0	5.22	12.18	methyltransferase [Arthrobacter sanguinis]
gi823665440	7.86	4	2	509	53.4	6.89	12.14	oxidoreductase [Arthrobacter sp. YC-RL1]
gi737793085	22.86	5	1	70	7.3	6.00	12.09	hypothetical protein [Arthrobacter nicotinovorans]
gi476402260	3.05	5	1	492	52.0	5.53	12.04	ADP-ribosylation/crystallin J1 [Arthrobacter crystallopoietes BAB-32]
gi636843728	3.62	5	1	470	51.9	8.76	12.03	hypothetical protein [Arthrobacter sp. TB 26]
gi760112652	6.30	8	1	254	26.3	10.45	11.95	ABC transporter [Arthrobacter chlorophenolicus]
gi916869562	7.35	2	2	408	44.2	9.60	11.84	transposase [Arthrobacter sp. Br18]
gi916324758	2.01	1	1	745	82.2	5.85	11.84	choline transporter [Arthrobacter gangotriensis]
gi651437930	8.94	3	1	235	24.9	9.26	11.80	50S ribosomal protein L1 [Arthrobacter sp. H14]
gi927031409	7.33	5	1	232	25.8	5.19	11.76	dihydrofolate reductase [Arthrobacter sp. LS16]
gi737788603	2.40	1	1	625	68.5	5.36	11.74	glycerophosphodiester phosphodiesterase [Arthrobacter albus]
gi542110244	5.25	8	1	324	35.7	6.40	11.74	hypothetical protein M707_02675 [Arthrobacter sp. AK-YN10]
gi937261964	7.73	1	1	181	18.9	9.76	11.74	ArsR family transcriptional regulator [Arthrobacter sp. Edens01]
gi323469187	4.78	1	1	230	24.5	5.27	11.72	conserved hypothetical protein TIGR00370 [Arthrobacter phenanthrenivorans Sphe3]
gi651483475	0.82	1	1	1222	131.3	5.31	11.70	chromosome segregation protein SMC [Arthrobacter sp. Br18]
gi937262500	5.20	5	2	654	70.8	6.73	11.69	acyl-CoA dehydrogenase [Arthrobacter sp. Edens01]
gi476399710	1.79	1	1	446	50.3	5.95	11.66	hypothetical protein D477_017277 [Arthrobacter crystallopoietes BAB-32]
gi908699658	3.54	1	1	396	40.6	5.76	11.65	acetyl-CoA acetyltransferase [Arthrobacter sp. RIT-PI-e]
gi470217162	1.94	2	1	412	43.2	5.55	11.64	beta-ketoadipyl CoA thiolase [Arthrobacter gangotriensis Lz1y]
gi359306171	5.06	2	1	257	28.1	6.58	11.60	hypothetical protein ARGLB_047_01190 [Arthrobacter globiformis NBRC 12137]
gi757624850	6.15	7	1	325	34.9	9.33	11.57	hypothetical protein TV39_09305 [Arthrobacter sp. SPG23]
gi116612391	3.15	5	2	889	95.3	5.19	11.57	ATPase AAA-2 domain protein [Arthrobacter sp. FB24]
gi937259576	4.88	2	1	328	35.1	6.04	11.56	hydroxyacid dehydrogenase [Arthrobacter sp. Edens01]
gi651455857	2.47	7	2	930	100.3	4.81	11.53	aconitate hydratase [Arthrobacter sp. 35/47]
gi651430386	3.04	2	1	428	45.9	8.25	11.51	two-component system sensor histidine kinase [Arthrobacter sanguinis]
gi906448044	22.55	4	2	102	11.6	9.57	11.42	30S ribosomal protein S10 [Arthrobacter sp. RIT-PI-e]
gi765006673	0.75	1	1	1606	170.5	6.06	11.41	DEAD/DEAH box helicase [Arthrobacter sp. A3]
gi914717426	1.51	2	1	729	76.0	5.59	11.37	acetyl-CoA carboxylase [Arthrobacter sp. ZBG10]
gi737788007	6.51	3	1	261	28.9	6.43	11.36	iron ABC transporter ATP-binding protein [Arthrobacter albus]
gi937259580	3.18	1	1	691	76.7	7.39	11.31	hypothetical protein AO716_06275 [Arthrobacter sp. Edens01]
gi723607785	2.09	6	1	812	88.5	6.16	11.28	hypothetical protein ART_1562 [Arthrobacter sp. PAMC25486]

gi917022183	2.17	2	1	552	56.9	6.55	11.27	FAD-binding protein [Arthrobacter sp. UNC362MFTsu5.1]
gi786031836	4.54	2	2	749	80.4	5.81	11.26	peptide ABC transporter ATP-binding protein [Arthrobacter chlorophenolicus]
gi515764459	2.21	2	1	588	63.4	5.29	11.16	flavin oxidoreductase [Arthrobacter sp. M2012083]
gi916835008	7.81	2	2	320	34.7	6.54	11.16	LysR family transcriptional regulator [Arthrobacter sp. H14]
gi927294035	1.58	4	1	1013	109.7	6.34	11.13	glutamine-synthetase [Arthrobacter sp. ERGS1:01]
gi928485861	3.20	6	1	656	69.5	7.94	11.09	ABC transporter [Arthrobacter alpinus]
gi759709557	4.32	3	1	486	52.7	5.63	11.07	FAD-dependent oxidoreductase [Arthrobacter sp. 9MFCol3.1]
gi823666269	4.55	2	1	572	58.4	4.64	11.04	dihydrolipoamide acetyltransferase [Arthrobacter sp. YC-RL1]
gi742071065	3.85	1	1	546	57.1	9.33	11.04	hypothetical protein ANMWB30_22640 [Arthrobacter sp. MWB30]
gi551253978	2.07	1	1	482	51.1	5.08	10.99	branched-chain alpha-keto acid dehydrogenase subunit E2 [Arthrobacter sp. PAO19]
gi162954244	1.47	3	1	954	105.1	6.81	10.99	ATP-dependent DNA helicase [Renibacterium salmoninarum ATCC 33209]
gi917760524	1.91	2	1	629	66.1	5.45	10.91	hypothetical protein [Arthrobacter sp. L77]
gi359305886	2.34	1	1	896	95.5	5.55	10.90	putative acetate--CoA ligase [Arthrobacter globiformis NBRC 12137]
gi651500601	0.98	2	1	1021	111.9	7.08	10.88	transposase [Arthrobacter sp. 35W]
gi742071095	1.27	1	1	866	89.1	4.40	10.79	hypothetical protein ANMWB30_22940 [Arthrobacter sp. MWB30]
gi914717881	1.51	2	1	1189	127.0	5.36	10.77	chromosome segregation protein SMC [Arthrobacter sp. ZBG10]
gi916870072	9.42	3	1	191	20.5	6.34	10.74	hypothetical protein [Arthrobacter sp. Br18]
gi674644458	6.00	4	1	400	41.6	5.34	10.74	N-acetylglucosamine repressor [Arthrobacter sp. 11W110_air]
gi917732919	0.97	1	1	1857	204.4	5.29	10.74	hypothetical protein [Arthrobacter sp. MWB30]
gi737805202	9.32	3	1	236	25.2	5.69	10.73	DtxR family transcriptional regulator [Arthrobacter sp. Br18]
gi757623679	2.05	2	1	1022	108.4	5.15	10.73	chromosome segregation protein SMC [Arthrobacter sp. SPG23]
gi914714323	1.59	1	1	816	84.5	7.02	10.70	hypothetical protein [Arthrobacter sp. ZBG10]
gi917013199	4.40	5	2	727	78.7	9.36	10.65	hypothetical protein [Arthrobacter sanguinis]
gi636845208	6.13	1	1	326	34.3	5.36	10.64	prephenate dehydratase [Arthrobacter sp. TB 26]
gi917442227	3.29	1	1	729	78.5	5.22	10.62	ATP-dependent DNA helicase RecQ [Arthrobacter albus]
gi910249965	4.71	2	1	403	45.1	9.66	10.60	hypothetical protein [Arthrobacter siccitolerans]
gi219859433	23.29	2	1	73	7.6	8.97	10.56	conserved hypothetical protein [Arthrobacter chlorophenolicus A6]
gi119949759	5.15	7	1	388	40.0	6.54	10.56	putative cysteine desulfurase [Arthrobacter aurescens TC1]
gi648575107	1.51	5	1	1058	116.3	5.60	10.56	cytochrome P450 [Arthrobacter sp. 131MFCol6.1]
gi476399111	4.81	6	2	457	48.9	4.96	10.54	succinate-semialdehyde dehydrogenase [Arthrobacter crystallopoietes BAB-32]
gi939050616	8.02	3	2	399	42.2	5.36	10.47	aspartate aminotransferase [Arthrobacter sp. JCM 19049]
gi742861262	7.50	3	1	280	30.7	5.14	10.46	oxidoreductase [Arthrobacter sp. W1]
gi640204645	1.21	4	1	746	84.2	8.60	10.46	glycosyltransferase [Arthrobacter sp. 31Y]
gi786025726	7.23	3	2	553	58.8	6.19	10.45	two-component system sensor histidine kinase [Arthrobacter chlorophenolicus]
gi517590747	2.06	1	1	921	97.3	5.92	10.37	histidine kinase [Arthrobacter sp. 135MFCol5.1]
gi470217420	5.15	7	1	408	44.4	6.16	10.35	tellurite resistance protein [Arthrobacter gangotriensis Lz1y]
gi910250094	1.16	1	1	1033	110.0	5.19	10.32	chromosome segregation protein SMC [Arthrobacter siccitolerans]
gi928542246	4.37	3	1	481	52.8	5.16	10.32	portal protein [Arthrobacter phage Brent]
gi517602196	7.26	1	1	358	38.7	6.15	10.31	GNAT family N-acetyltransferase [Arthrobacter sp. 131MFCol6.1]
gi737787955	4.02	3	2	696	78.7	4.97	10.28	excinuclease ABC subunit B [Arthrobacter albus]
gi476398940	4.35	4	2	966	100.8	5.78	10.27	molybdopterin binding aldehyde oxidase and xanthine dehydrogenase [Arthrobacter crystallo
gi916259816	5.08	4	1	236	24.6	6.20	10.24	2-deoxy-D-gluconate 3-dehydrogenase [Arthrobacter sp. TB 23]
gi551255539	2.55	1	1	471	50.2	5.45	10.23	flavoprotein [Arthrobacter sp. PAO19]
gi759712277	4.10	2	1	415	43.4	8.09	10.23	two-component system sensor histidine kinase [Arthrobacter sp. 162MFSha1.1]
gi651465772	1.76	1	1	909	94.7	5.05	10.21	hypothetical protein [Arthrobacter sp. 35/47]
gi651430369	13.42	2	1	149	15.5	6.58	10.21	50S ribosomal protein L9 [Arthrobacter sanguinis]
gi517605103	3.11	3	1	482	50.5	8.50	10.20	phytoene dehydrogenase [Arthrobacter sp. 131MFCol6.1]
gi654814020	9.03	2	2	321	34.3	5.33	10.16	ABC transporter [Arthrobacter sp. MA-N2]
gi654817067	10.24	2	1	205	23.7	6.55	10.15	nuclease PIN [Arthrobacter sp. UNC362MFTsu5.1]
gi737787456	3.41	1	1	469	50.7	6.02	10.14	hypothetical protein [Arthrobacter albus]
gi636846478	4.69	1	1	341	36.6	6.28	10.14	peroxidase, partial [Arthrobacter sp. TB 26]
gi359304196	2.27	6	2	1275	139.9	6.38	10.10	2-oxoglutarate dehydrogenase E1 component [Arthrobacter globiformis NBRC 12137]
gi652423038	1.49	3	2	937	104.8	6.86	10.09	RNA helicase [Arthrobacter castelli]
gi939037486	8.71	2	1	241	26.1	7.75	10.09	hypothetical protein [Arthrobacter nitroguajacolicus]
gi517593626	3.76	7	1	213	23.3	5.58	10.08	hypothetical protein [Arthrobacter sp. 135MFCol5.1]

gi518313374	1.65	1	1	726	79.3	5.27	10.05	NAD-dependent DNA ligase LigA [Arthrobacter sp. TB 23]
gi651504417	2.87	4	1	523	55.7	6.73	10.02	sodium:proton antiporter [Arthrobacter sp. 35W]
gi639130880	1.65	5	1	1032	116.5	5.77	10.02	restriction endonuclease subunit R [Arthrobacter sp. CAL618]
gi723609806	6.31	5	1	206	22.2	6.43	10.01	two-component system response regulator [Arthrobacter sp. PAMC25486]
gi359305890	1.94	3	2	1186	120.9	10.56	9.99	putative ABC transporter permease/ATP-binding protein CydDC [Arthrobacter globiformis NB
gi910749230	2.97	6	1	674	76.4	6.47	9.97	conserved hypothetical protein (plasmid) [Arthrobacter sp. Hiyo8]
gi162955635	4.65	2	1	409	43.0	5.34	9.96	3-ketoacyl-CoA thiolase [Renibacterium salmoninarum ATCC 33209]
gi359304264	2.31	1	1	910	101.6	5.15	9.95	protein translocase subunit SecA [Arthrobacter globiformis NBRC 12137]
gi765010063	1.91	2	1	1150	122.1	5.16	9.93	1-pyrroline-5-carboxylate dehydrogenase [Arthrobacter sp. A3]
gi517601118	4.51	4	1	377	40.1	5.31	9.93	oxidoreductase [Arthrobacter sp. 162MFSha1.1]
gi927294133	2.96	2	1	405	43.9	6.87	9.89	lipase [Arthrobacter sp. ERGS1:01]
gi927296423	0.86	1	1	1276	137.2	7.15	9.87	ABC transporter [Arthrobacter sp. ERGS1:01]
gi723608175	5.85	1	1	171	18.6	6.20	9.85	Transcriptional regulator, AsnC family [Arthrobacter sp. PAMC25486]
gi930826823	3.51	3	2	655	74.6	5.59	9.85	hypothetical protein AOZ07_11405 [Arthrobacter arilaitensis]
gi742856325	13.64	4	1	154	16.5	5.19	9.84	hypothetical protein [Arthrobacter sp. W1]
gi742072944	3.55	1	1	394	40.8	7.25	9.84	tRNA(Ile)-lysidine synthetase [Arthrobacter sp. MWB30]
gi759702913	6.44	3	1	326	35.0	7.18	9.81	spermidine synthase [Arthrobacter globiformis]
gi927293366	1.05	1	1	1045	116.2	7.74	9.78	transposase (plasmid) [Arthrobacter sp. ERGS1:01]
gi742757181	3.76	5	1	479	50.3	5.47	9.75	succinate-semialdehyde dehydrogenase [Arthrobacter phenanthrenivorans]
gi910283724	5.44	1	1	349	36.7	5.15	9.74	hypothetical protein [Arthrobacter sp. A3]
gi928489026	20.65	1	1	92	10.2	5.06	9.72	hypothetical protein AOC05_17360 [Arthrobacter alpinus]
gi928486718	6.06	3	2	693	78.4	5.26	9.71	excinuclease ABC subunit B [Arthrobacter alpinus]
gi919134895	1.75	1	1	1260	137.0	5.55	9.71	hypothetical protein [Arthrobacter chlorophenolicus]
gi919218771	3.00	1	1	500	55.3	9.66	9.71	hypothetical protein [Arthrobacter sp. YC-RL1]
gi640199553	3.27	1	1	520	55.3	6.55	9.69	HNH endonuclease [Arthrobacter sp. 31Y]
gi636844938	6.04	6	1	182	19.5	4.94	9.69	ATP synthase F0F1 subunit B [Arthrobacter sp. TB 26]
gi359305020	1.37	4	1	952	97.6	4.82	9.67	hypothetical protein ARGLB_075_00010 [Arthrobacter globiformis NBRC 12137]
gi651494048	4.80	2	1	250	27.4	11.65	9.66	membrane protein [Arthrobacter sp. H20]
gi636845306	4.87	6	1	472	48.9	5.99	9.65	acetyl-CoA acetyltransferase [Arthrobacter sp. TB 26]
gi640198468	3.26	1	1	307	33.1	6.90	9.64	LysR family transcriptional regulator [Arthrobacter sp. 31Y]
gi916691824	3.51	1	1	541	56.1	10.30	9.64	hypothetical protein [Arthrobacter castelli]
gi359307391	1.22	2	1	1150	126.2	5.96	9.64	hypothetical protein ARGLB_015_00190 [Arthrobacter globiformis NBRC 12137]
gi916820388	1.53	1	1	1108	119.8	10.13	9.63	hypothetical protein [Arthrobacter sp. H20]
gi651485308	3.68	6	1	707	75.7	5.33	9.61	3-hydroxyacyl-CoA dehydrogenase [Arthrobacter sp. Br18]
gi759715616	6.43	7	2	420	45.1	5.01	9.60	GTP-binding protein, partial [Arthrobacter sp. AK-YN10]
gi640203686	1.61	1	1	1055	114.8	6.46	9.60	beta-phosphoglucomutase [Arthrobacter sp. 31Y]
gi651430802	2.17	2	1	507	55.4	4.87	9.58	aldehyde dehydrogenase [Arthrobacter sanguinis]
gi759761949	2.75	5	2	692	77.5	5.85	9.57	ATP-dependent Lon protease [Arthrobacter sp. Rue61a]
gi652424037	1.22	2	1	572	62.3	5.47	9.56	hypothetical protein [Arthrobacter castelli]
gi470220215	3.83	2	1	235	24.7	9.26	9.56	50S ribosomal protein L1 [Arthrobacter gangotriensis Lz1y]
gi476400413	8.33	8	1	264	27.7	11.25	9.51	cobalt transport protein [Arthrobacter crystallopoietes BAB-32]
gi916691433	4.20	2	2	691	76.1	6.13	9.51	acyl-CoA dehydrogenase [Arthrobacter castelli]
gi476402199	16.44	3	1	73	8.4	9.19	9.51	translation initiation factor IF-1 [Arthrobacter crystallopoietes BAB-32]
gi443479672	16.99	5	2	153	15.5	10.87	9.50	hypothetical protein G205_23057 [Arthrobacter nitrophenolicus]
gi651468817	2.47	3	1	526	58.4	9.39	9.49	hypothetical protein [Arthrobacter nicotinovorans]
gi518313582	7.06	1	1	326	34.8	9.77	9.49	sporulation protein [Arthrobacter sp. TB 23]
gi759718178	2.39	1	1	587	62.3	5.17	9.44	arsenical pump-driving ATPase [Arthrobacter sp. FB24]
gi651449859	4.88	3	1	328	34.5	4.79	9.42	malate dehydrogenase [Arthrobacter nicotinovorans]
gi916872065	3.05	4	1	426	46.7	9.26	9.38	exodeoxyribonuclease VII large subunit [Arthrobacter sp. H5]
gi635351885	0.97	6	1	821	89.1	5.69	9.38	hypothetical protein ARTSIC4J27_2201 [Arthrobacter siccitolerans]
gi916870042	3.34	1	1	658	70.1	6.51	9.35	hypothetical protein [Arthrobacter sp. Br18]
gi359305975	6.90	1	1	290	30.6	9.00	9.28	putative DNA glycosylase [Arthrobacter globiformis NBRC 12137]
gi332743185	2.70	5	1	519	58.5	9.36	9.28	hypothetical protein STTU_0837 [Streptomyces sp. Tu6071]
gi723607993	1.63	10	1	675	69.8	5.36	9.24	methylcrotonoyl-CoA carboxylase subunit alpha [Arthrobacter sp. PAMC25486]
gi917760320	1.18	1	1	1102	122.9	5.01	9.22	isoleucine--tRNA ligase [Arthrobacter sp. L77]

gi823668203	6.23	5	2	401	42.0	5.45	9.21	beta-ketoadipyl CoA thiolase [Arthrobacter sp. YC-RL1]
gi910694411	9.94	7	1	171	18.9	4.87	9.19	ribosome maturation factor RimP [Arthrobacter sp. Hiyo6]
gi648575582	7.88	5	1	203	21.9	7.65	9.18	TetR family transcriptional regulator [Arthrobacter sp. 161MFSha2.1]
gi651474245	4.10	1	1	536	57.6	5.82	9.18	PucR family transcriptional regulator [Arthrobacter nicotinovorans]
gi359304436	3.15	1	1	413	43.3	5.87	9.15	hypothetical protein ARGLB_085_03020 [Arthrobacter globiformis NBRC 12137]
gi323470940	0.97	1	1	1237	136.7	6.55	9.14	respiratory nitrate reductase alpha subunit apoprotein [Arthrobacter phenanthrenivorans Sph
gi359307533	0.90	1	1	1003	111.7	5.19	9.12	putative McrBC restriction endonuclease system protein McrB [Arthrobacter globiformis NBRC
gi742071929	2.49	1	1	522	54.2	5.19	9.11	aspartyl/glutamyl-tRNA amidotransferase subunit A [Arthrobacter sp. MWB30]
gi928488768	6.11	1	1	360	39.8	10.70	9.11	hypothetical protein AOC05_08450 [Arthrobacter alpinus]
gi489899647	2.27	1	1	837	89.9	5.85	9.08	nitrite reductase large subunit [Arthrobacter globiformis]
gi742853148	0.90	7	1	1326	146.2	7.05	9.07	ATP-dependent helicase [Arthrobacter sp. W1]
gi119947513	4.74	3	1	211	23.3	10.89	9.03	putative S4 domain protein [Arthrobacter aurescens TC1]
gi517601145	3.76	1	1	479	50.4	5.47	9.03	NAD-dependent succinate-semialdehyde dehydrogenase [Arthrobacter sp. 162MFSha1.1]
gi359305572	2.99	6	1	735	74.8	5.86	9.03	subtilisin family peptidase [Arthrobacter globiformis NBRC 12137]
gi759719843	0.90	2	1	1217	130.8	5.24	9.02	chromosome segregation protein SMC [Arthrobacter sp. FB24]
gi908691070	5.71	2	2	280	30.6	8.21	9.02	transcriptional regulator, IclR family protein [Arthrobacter sp. H41]
gi910249918	4.49	3	1	245	26.6	7.23	9.02	peptide ABC transporter ATP-binding protein [Arthrobacter siccitolerans]
gi443480591	4.43	3	1	384	40.7	6.15	9.01	sarcosine oxidase [Arthrobacter nitrophenolicus]
gi910739864	7.27	2	2	509	53.7	8.51	9.00	putative Rieske 2Fe-2S iron-sulfur protein YhfW [Arthrobacter sp. Hiyo4]
gi651429303	1.83	1	1	763	74.1	4.96	8.99	hypothetical protein [Arthrobacter sanguinis]
gi737788815	0.54	1	1	1681	180.0	6.13	8.99	DEAD/DEAH box helicase [Arthrobacter albus]
gi359305906	4.15	6	1	410	41.7	5.92	8.98	dihydrolipoamide acyltransferase [Arthrobacter globiformis NBRC 12137]
gi551254154	2.29	1	1	349	38.4	6.74	8.98	membrane protein [Arthrobacter sp. PAO19]
gi470216707	1.18	2	1	764	83.8	5.34	8.96	UvrD/REP helicase [Arthrobacter gangotriensis Lz1y]
gi636844661	4.61	1	1	412	42.7	5.30	8.94	acetyl-CoA acetyltransferase [Arthrobacter sp. TB 26]
gi651462200	1.96	3	1	561	57.4	4.64	8.93	phosphoenolpyruvate-protein phosphotransferase [Arthrobacter sp. 35/47]
gi927295127	5.45	3	2	550	59.3	5.12	8.92	arginyl-tRNA synthetase [Arthrobacter sp. ERGS1:01]
gi651431530	10.89	3	1	101	11.1	10.11	8.92	50S ribosomal protein L23 [Arthrobacter sanguinis]
gi767259114	4.01	1	1	424	44.8	7.27	8.90	hypothetical protein UM93_08705 [Arthrobacter sp. IHBB 11108]
gi930827435	3.61	2	1	471	50.1	5.81	8.88	branched-chain alpha-keto acid dehydrogenase subunit E2 [Arthrobacter arilaitensis]
gi753932119	2.83	7	2	882	97.5	5.74	8.87	hypothetical protein [Arthrobacter arilaitensis]
gi652424727	4.44	4	1	293	32.8	5.60	8.86	protein iolH [Arthrobacter castelli]
gi651503087	6.18	4	1	372	39.5	5.95	8.85	GTPase [Arthrobacter sp. 35W]
gi651439668	11.33	6	1	203	21.6	10.08	8.84	hypothetical protein [Arthrobacter sp. H14]
gi651430260	1.04	1	1	578	65.9	5.47	8.84	ATPase AAA [Arthrobacter sanguinis]
gi651439573	1.50	2	1	866	94.6	5.50	8.84	glycogen phosphorylase [Arthrobacter sp. H14]
gi476402334	4.18	2	1	287	32.2	5.72	8.82	hypothetical protein D477_004132 [Arthrobacter crystallopoietes BAB-32]
gi908698192	5.43	1	1	313	31.6	5.77	8.81	NADH-ubiquinone oxidoreductase [Arthrobacter sp. RIT-PI-e]
gi765008507	9.84	2	2	315	32.2	5.12	8.80	glycine/betaine ABC transporter substrate-binding protein [Arthrobacter sp. A3]
gi916926310	8.06	4	1	397	39.4	6.32	8.76	acetyl-CoA acetyltransferase [Arthrobacter sp. 9MFCol3.1]
gi119951743	23.40	5	1	94	10.2	8.54	8.76	conserved hypothetical protein (plasmid) [Arthrobacter aurescens TC1]
gi742859076	6.10	1	1	164	18.2	9.86	8.75	hypothetical protein [Arthrobacter sp. W1]
gi765010970	2.93	1	1	512	55.8	6.90	8.74	ABC transporter ATP-binding protein [Arthrobacter sp. A3]
gi759730350	5.40	2	1	315	32.4	5.06	8.74	electron transfer flavoprotein subunit alpha [Arthrobacter sp. L77]
gi910251686	5.20	2	2	827	88.5	6.67	8.74	hypothetical protein [Arthrobacter siccitolerans]
gi654818331	3.15	2	1	539	56.7	9.74	8.72	ABC transporter [Arthrobacter sp. UNC362MFTsu5.1]
gi651460932	2.70	1	1	814	89.5	5.94	8.70	ATP-dependent DNA helicase PcrA [Arthrobacter sp. 35/47]
gi674646020	11.05	9	1	190	21.0	4.83	8.67	Adenylate kinase [Arthrobacter sp. 11W110_air]
gi476399035	4.30	2	1	395	40.8	7.87	8.66	transcriptional regulator/sugar kinase [Arthrobacter crystallopoietes BAB-32]
gi517603993	2.20	7	1	681	75.8	6.06	8.64	alpha-1,4-glucan--maltose-1-phosphate maltosyltransferase [Arthrobacter sp. 131MFCol6.1]
gi648224420	6.30	2	1	254	27.3	7.14	8.63	IclR family transcriptional regulator [Arthrobacter sp. M2012083]
gi910741425	6.97	1	1	201	21.8	5.14	8.63	acetyl-coenzyme A synthetase [Arthrobacter sp. Hiyo4]
gi937257996	2.16	1	1	924	97.8	5.49	8.63	hypothetical protein AO716_07640 [Arthrobacter sp. Edens01]
gi767257639	1.81	1	1	1107	119.0	5.01	8.62	DNA helicase UvrD [Arthrobacter sp. IHBB 11108]
gi640196635	4.99	1	1	401	40.2	5.12	8.61	glycerate kinase [Arthrobacter sp. 31Y]

gi937258804	2.01	1	1	698	76.7	6.51	8.59	acyl-CoA dehydrogenase [Arthrobacter sp. Edens01]
gi914716984	12.32	4	2	203	21.9	8.88	8.59	TetR family transcriptional regulator [Arthrobacter sp. ZBG10]
gi651502581	4.90	4	1	531	55.4	5.08	8.58	3-phosphoglycerate dehydrogenase [Arthrobacter sp. 35W]
gi359305033	5.10	2	1	431	48.5	5.38	8.58	putative oxidoreductase [Arthrobacter globiformis NBRC 12137]
gi910741225	6.90	1	1	348	38.0	10.18	8.58	uncharacterized protein Rv2248/MT2308 [Arthrobacter sp. Hiyo4]
gi119947680	2.05	1	1	536	56.3	5.20	8.58	UDP-N-acetylmuramyl-tripeptide synthetase [Arthrobacter aurescens TC1]
gi765009334	6.69	4	2	493	53.6	5.25	8.57	ATPase [Arthrobacter sp. A3]
gi651488072	2.72	2	1	367	40.7	10.11	8.56	integrase [Arthrobacter sp. H20]
gi937257906	7.22	1	1	180	20.8	5.35	8.55	hypothetical protein AO716_07050 [Arthrobacter sp. Edens01]
gi910249923	1.02	5	1	1177	126.2	5.38	8.54	DNA helicase UvrD [Arthrobacter siccitolerans]
gi640194528	3.71	3	1	539	57.7	9.74	8.54	ABC transporter [Arthrobacter sp. 31Y]
gi307744541	1.37	3	1	1023	111.4	5.35	8.54	putative nuclease SbcCD subunit C [Arthrobacter arilaitensis Re117]
gi937259376	1.60	4	1	877	96.6	5.14	8.53	DNA polymerase I [Arthrobacter sp. Edens01]
gi918266973	3.45	1	1	377	40.9	6.16	8.52	putative ribose/galactose/methyl galactoside import ATP-binding protein 2 [Arthrobacter sp.
gi823667377	3.64	2	1	412	45.7	7.66	8.51	hypothetical protein AA310_17330 [Arthrobacter sp. YC-RL1]
gi654812960	2.77	1	1	794	84.5	6.90	8.51	transcription accessory protein [Arthrobacter sp. MA-N2]
gi517593656	6.30	4	1	365	39.7	5.71	8.47	hypothetical protein [Arthrobacter sp. 135MFCol5.1]
gi937261830	4.93	2	1	345	37.2	6.81	8.47	epimerase [Arthrobacter sp. Edens01]
gi476398905	22.55	1	1	102	11.4	5.05	8.46	hypothetical protein D477_021043 [Arthrobacter crystallopoietes BAB-32]
gi545108181	1.58	2	1	444	50.0	5.60	8.45	alcaligin biosynthesis enzyme [Arthrobacter sp. AK-YN10]
gi737773318	2.79	2	1	502	53.5	8.94	8.45	FAD-dependent oxidoreductase [Arthrobacter sp. MA-N2]
gi910744863	3.54	2	1	424	45.3	7.27	8.45	glutathione-binding protein GsiB [Arthrobacter sp. Hiyo8]
gi759732469	1.17	1	1	941	102.7	5.58	8.42	phosphoenolpyruvate carboxylase [Arthrobacter sp. L77]
gi189044289	11.20	2	1	125	14.1	10.56	8.42	RecName: Full=30S ribosomal protein S13
gi765012058	11.30	1	1	230	24.0	5.53	8.41	histidine phosphatase [Arthrobacter sp. A3]
gi489896321	4.50	1	1	333	34.7	4.92	8.41	dihydroxyacetone kinase subunit DhaK [Arthrobacter globiformis]
gi517603091	1.77	1	1	621	64.8	9.31	8.40	hypothetical protein [Arthrobacter sp. 131MFCol6.1]
gi737803430	3.13	2	1	415	45.7	7.02	8.40	transposase [Arthrobacter sp. Br18]
gi476400289	8.54	1	1	199	22.1	9.86	8.40	metal-dependent hydrolase [Arthrobacter crystallopoietes BAB-32]
gi917013474	5.35	1	1	187	21.1	6.57	8.39	hypothetical protein [Arthrobacter sanguinis]
gi910745975	6.33	1	1	332	35.2	4.94	8.38	probable xylitol oxidase [Arthrobacter sp. Hiyo8]
gi910744355	2.94	2	1	510	56.1	6.54	8.38	L-arabinose transport ATP-binding protein AraG [Arthrobacter sp. Hiyo8]
gi917013276	2.00	2	1	1049	107.7	5.17	8.37	hydrogenase expression protein [Arthrobacter sanguinis]
gi742072644	3.25	4	1	431	46.0	8.13	8.37	HNH endonuclease domain protein [Arthrobacter sp. MWB30]
gi116611561	2.61	3	1	345	38.4	5.29	8.36	aldo/keto reductase [Arthrobacter sp. FB24]
gi639128918	3.18	2	1	534	57.8	8.31	8.36	ATP/GTP-binding protein [Arthrobacter sp. CAL618]
gi517593548	2.81	1	1	392	44.0	7.59	8.35	hypothetical protein, partial [Arthrobacter sp. 135MFCol5.1]
gi652423642	5.88	1	1	357	37.8	5.17	8.33	alkene reductase [Arthrobacter castelli]
gi403231734	4.68	2	1	299	32.3	5.50	8.32	diacylglycerol kinase catalytic domain-containing protein [Arthrobacter sp. Rue61a]
gi654818151	3.52	1	1	341	36.0	9.83	8.28	FAD-binding monooxygenase [Arthrobacter sp. UNC362MFTsu5.1]
gi517602410	1.47	1	1	1088	117.4	5.77	8.26	hypothetical protein [Arthrobacter sp. 131MFCol6.1]
gi742758401	10.24	2	1	205	23.4	6.55	8.25	nuclease PIN [Arthrobacter phenanthrenivorans]
gi652424519	3.97	4	1	252	27.2	5.45	8.25	GntR family transcriptional regulator [Arthrobacter castelli]
gi917739807	2.51	3	1	995	111.0	5.62	8.24	restriction endonuclease subunit R [Arthrobacter sp. W1]
gi917013443	2.38	1	1	715	75.0	5.27	8.24	potassium transporter KtrB [Arthrobacter sanguinis]
gi635353177	1.38	2	1	653	68.3	5.38	8.23	copper-translocating P-type ATPase [Arthrobacter siccitolerans]
gi737799682	1.21	1	1	994	112.3	6.09	8.21	restriction endonuclease [Arthrobacter sp. TB 23]
gi515768115	1.84	2	1	761	85.4	6.15	8.21	hypothetical protein [Arthrobacter sp. M2012083]
gi723606870	0.96	2	1	934	100.3	4.89	8.19	aconitate hydratase [Arthrobacter sp. PAMC25486]
gi737781380	1.46	2	1	1161	126.2	6.79	8.18	DNA polymerase III subunit alpha [Arthrobacter sp. 35W]
gi740684044	4.73	2	1	402	41.8	5.35	8.17	acetyl-CoA acetyltransferase [Arthrobacter sp. PAMC25486]
gi443482553	4.97	6	1	382	39.8	6.13	8.16	ROK family transcriptional regulator [Arthrobacter nitrophenolicus]
gi910693128	13.08	5	1	107	11.9	10.05	8.16	DNA gyrase subunit B, partial [Arthrobacter sp. Hiyo6]
gi765007624	4.91	1	1	468	50.0	6.34	8.16	aldehyde dehydrogenase [Arthrobacter sp. A3]
gi927033875	5.32	5	2	489	54.5	8.56	8.15	aspartate aminotransferase [Arthrobacter sp. LS16]

gi651466753	3.56	1	1	309	32.4	5.33	8.14	nicotinate-nucleotide pyrophosphorylase [Arthrobacter sp. 35/47]
gi767259331	3.30	1	1	455	49.6	5.14	8.13	beta-glucosidase [Arthrobacter sp. IHBB 11108]
gi651507327	4.17	1	1	360	37.8	5.95	8.13	alkene reductase [Arthrobacter sp. 35W]
gi651493143	4.43	2	1	361	39.2	5.26	8.12	inositol-3-phosphate synthase [Arthrobacter sp. H20]
gi759730901	4.49	2	1	245	26.5	7.15	8.11	peptide ABC transporter ATP-binding protein [Arthrobacter sp. L77]
gi654822017	4.30	1	1	395	42.2	9.11	8.09	FAD-binding monooxygenase [Arthrobacter sp. I3]
gi767257607	2.46	3	1	406	44.2	4.94	8.09	oxidoreductase [Arthrobacter sp. IHBB 11108]
gi759731904	4.93	4	1	365	41.0	9.66	8.08	hypothetical protein [Arthrobacter sp. L77]
gi476399853	3.11	2	1	514	55.8	10.20	8.08	signal recognition particle protein [Arthrobacter crystallopoietes BAB-32]
gi651470002	3.30	1	1	637	72.7	8.78	8.06	glycosyltransferase [Arthrobacter nicotinovorans]
gi515764892	1.66	1	1	422	43.5	5.43	8.04	ROK family transcriptional regulator [Arthrobacter sp. M2012083]
gi723607639	5.90	1	1	322	34.9	6.90	8.02	ABC-type multidrug transport system, ATPase component [Arthrobacter sp. PAMC25486]
gi759747354	2.63	1	1	608	67.1	9.57	8.02	MarR family transcriptional regulator [Arthrobacter sp. 31Y]
gi767258216	4.19	1	1	382	40.9	4.91	8.01	hypothetical protein UM93_12350 [Arthrobacter sp. IHBB 11108]
gi654811920	7.35	2	1	272	29.9	10.15	7.99	hypothetical protein [Arthrobacter sp. MA-N2]
gi654818780	13.21	1	1	159	17.1	5.21	7.99	ArsR family transcriptional regulator [Arthrobacter sp. UNC362MFTsu5.1]
gi515767825	1.10	5	1	1003	107.7	5.11	7.98	chromosome segregation protein SMC [Arthrobacter sp. M2012083]
gi910742004	2.78	1	1	539	55.7	6.23	7.98	putative HMP/thiamine import ATP-binding protein YkoD [Arthrobacter sp. Hiyo4]
gi786027108	3.12	2	1	353	37.4	6.76	7.98	glycosyl transferase family 1 [Arthrobacter chlorophenolicus]
gi910743982	1.78	1	1	673	72.9	5.66	7.97	hypothetical protein AHiyo8_16840 [Arthrobacter sp. Hiyo8]
gi517610087	1.71	1	1	586	64.8	5.66	7.97	hypothetical protein [Arthrobacter sp. 161MFSha2.1]
gi910742815	11.16	3	2	224	24.6	7.36	7.96	FAD dependent oxidoreductase [Arthrobacter sp. Hiyo8]
gi674644307	7.52	2	1	306	32.1	4.97	7.96	2-dehydro-3-deoxygluconokinase [Arthrobacter sp. 11W110_air]
gi919219083	6.05	1	1	281	31.5	9.55	7.94	hypothetical protein [Arthrobacter sp. YC-RL1]
gi823666791	2.43	1	1	741	78.8	7.55	7.94	hypothetical protein AA310_13715, partial [Arthrobacter sp. YC-RL1]
gi930828168	5.46	1	1	458	47.8	5.27	7.94	hypothetical protein AOZ07_11020 [Arthrobacter arilaitensis]
gi916781934	2.94	3	1	613	67.3	5.31	7.94	aspartyl-tRNA synthetase [Arthrobacter sp. 35W]
gi116608862	12.31	3	2	268	28.6	9.35	7.93	ABC transporter related protein [Arthrobacter sp. FB24]
gi916691329	10.60	1	1	217	21.8	10.30	7.93	Holliday junction resolvase [Arthrobacter castelli]
gi470217945	1.38	2	1	580	63.1	5.66	7.92	oligopeptide ABC transporter substrate-binding protein [Arthrobacter gangotriensis Lz1y]
gi639129646	3.23	1	1	558	62.1	10.30	7.92	transposase [Arthrobacter sp. CAL618]
gi742756026	2.23	2	1	539	61.1	7.65	7.91	deoxyribodipyrimidine photolyase [Arthrobacter phenanthrenivorans]
gi651448486	5.05	2	1	297	32.3	7.01	7.91	LysR family transcriptional regulator [Arthrobacter nicotinovorans]
gi162955752	14.10	2	1	78	9.0	12.48	7.91	LSU ribosomal protein L34P [Renibacterium salmoninarum ATCC 33209]
gi918449310	0.89	2	1	1795	198.5	6.83	7.91	hypothetical protein [Arthrobacter sp. SPG23]
gi542110443	1.94	1	1	877	92.3	7.93	7.89	helicase [Arthrobacter sp. AK-YN10]
gi651441012	1.70	1	1	587	62.7	8.31	7.88	23S rRNA methyltransferase [Arthrobacter sp. 9MFCol3.1]
gi648574351	1.43	1	1	700	75.8	4.87	7.88	hypothetical protein [Arthrobacter sp. 162MFSha1.1]
gi551255613	3.46	1	1	665	71.0	5.38	7.88	ATP-dependent helicase [Arthrobacter sp. PAO19]
gi916820129	3.74	1	1	348	38.7	9.77	7.88	hypothetical protein [Arthrobacter sp. H20]
gi517593206	3.80	1	1	368	37.6	7.01	7.88	hypothetical protein [Arthrobacter sp. 135MFCol5.1]
gi651502230	1.72	1	1	930	96.0	5.91	7.88	phosphoenolpyruvate synthase [Arthrobacter sp. 35W]
gi759710542	4.22	2	1	450	46.4	6.15	7.87	flavoprotein [Arthrobacter sp. 135MFCol5.1]
gi651491530	4.21	1	1	309	32.4	8.60	7.87	formamidopyrimidine-DNA glycosylase [Arthrobacter sp. H20]
gi759962312	5.92	3	1	304	31.8	5.03	7.86	prephenate dehydratase [Nocardia thailandica]
gi162953597	8.94	9	1	235	26.0	5.26	7.86	iron dependent repressor [Renibacterium salmoninarum ATCC 33209]
gi759747186	4.93	1	1	406	43.8	5.60	7.84	two-component system sensor histidine kinase [Arthrobacter sp. 31Y]
gi910739458	6.58	1	1	228	24.1	9.26	7.84	hypothetical protein AHiyo4_22800 [Arthrobacter sp. Hiyo4]
gi651435178	1.16	1	1	1384	149.2	6.80	7.84	hypothetical protein [Arthrobacter sp. H41]
gi517591434	4.69	1	1	213	23.1	7.43	7.84	HxlR family transcriptional regulator [Arthrobacter sp. 135MFCol5.1]
gi823667882	2.39	1	1	710	78.2	6.34	7.83	choline transporter [Arthrobacter sp. YC-RL1]
gi162953843	2.87	2	1	487	53.8	6.20	7.83	sensory transduction protein kinase [Renibacterium salmoninarum ATCC 33209]
gi928489002	4.86	1	1	453	46.4	5.80	7.82	hypothetical protein AOC05_16645 [Arthrobacter alpinus]
gi917442168	0.67	1	1	1195	128.8	5.16	7.81	chromosome segregation protein SMC [Arthrobacter albus]
gi927294593	5.27	4	1	474	49.7	4.84	7.81	hypothetical protein AL755_12135 [Arthrobacter sp. ERGS1:01]

gi927296447	2.13	1	1	470	48.7	4.97	7.79	oxidoreductase [Arthrobacter sp. ERGS1:01]
gi757622369	4.66	1	1	343	36.4	9.96	7.79	FAD-binding monooxygenase [Arthrobacter sp. SPG23]
gi119948361	4.71	1	1	297	32.0	5.64	7.78	putative transketolase subunit A [Arthrobacter aurescens TC1]
gi910283858	1.72	1	1	816	86.4	7.65	7.78	transcription accessory protein [Arthrobacter sp. A3]
gi476399103	5.34	5	2	468	49.9	4.78	7.77	succinate-semialdehyde dehydrogenase [Arthrobacter crystallopoietes BAB-32]
gi918268738	17.65	2	1	136	14.4	10.14	7.77	50S ribosomal protein L1 [Arthrobacter sp. Hiyo1]
gi917760327	1.97	2	1	507	55.1	4.36	7.77	trigger factor [Arthrobacter sp. L77]
gi443483195	9.83	1	1	234	23.6	10.08	7.77	hypothetical protein G205_01374 [Arthrobacter nitrophenolicus]
gi470220787	4.38	5	1	320	33.6	6.16	7.75	2,3,4,5-tetrahydropyridine-2,6-dicarboxylate N-succinyltransferase [Arthrobacter gangotriens
gi359305903	4.95	2	1	505	51.9	6.14	7.75	putative aminopeptidase [Arthrobacter globiformis NBRC 12137]
gi916816404	3.88	1	1	438	48.6	8.53	7.75	multidrug transporter [Arthrobacter sp. MA-N2]
gi928488075	1.00	2	1	1100	120.9	6.07	7.73	chromosome segregation protein SMC [Arthrobacter alpinus]
gi757623578	2.74	2	1	475	48.8	8.05	7.73	histidine kinase [Arthrobacter sp. SPG23]
gi116611579	2.76	1	1	399	42.4	6.54	7.72	Flp pilus assembly protein ATPase CpaE-like protein [Arthrobacter sp. FB24]
gi937259003	4.44	2	1	225	23.2	6.80	7.72	hypothetical protein AO716_13740 [Arthrobacter sp. Edens01]
gi908698631	3.78	1	1	291	29.8	4.87	7.70	inositol monophosphatase [Arthrobacter sp. RIT-PI-e]
gi928488730	10.06	2	1	179	19.6	9.86	7.70	hypothetical protein AOC05_07365 [Arthrobacter alpinus]
gi759736215	1.20	1	1	1002	111.1	6.47	7.68	glutamine-synthetase [Arthrobacter sp. L77]
gi914715519	6.20	6	2	742	81.0	5.59	7.67	AAA family ATPase [Arthrobacter sp. ZBG10]
gi517606688	2.11	5	1	711	73.7	5.92	7.67	potassium-transporting ATPase subunit B [Arthrobacter sp. 161MFSha2.1]
gi511534814	7.88	2	1	241	26.8	10.24	7.67	putative transposase, partial (plasmid) [Arthrobacter nicotinovorans]
gi654819440	4.76	1	1	252	28.1	5.66	7.66	MerR family transcriptional regulator [Arthrobacter sp. UNC362MFTsu5.1]
gi939050386	3.66	1	1	273	32.1	10.11	7.66	lipase [Arthrobacter sp. JCM 19049]
gi211588222	3.33	1	1	480	52.4	5.64	7.65	Pc20g09930 [Penicillium rubens Wisconsin 54-1255]
gi323469163	1.35	1	1	518	53.7	5.45	7.65	ABC-type dipeptide transport system, periplasmic component [Arthrobacter phenanthrenivora
gi927296556	3.44	1	1	320	35.3	6.65	7.65	hypothetical protein AL755_19490 [Arthrobacter sp. ERGS1:01]
gi476402266	6.94	1	1	173	19.4	6.70	7.65	hypothetical protein D477_004501 [Arthrobacter crystallopoietes BAB-32]
gi323470958	7.01	1	1	214	24.0	5.39	7.65	polyketide cyclase / dehydrase family protein [Arthrobacter phenanthrenivorans Sphe3]
gi651482518	2.34	4	1	342	38.6	4.96	7.64	AP endonuclease [Arthrobacter sp. Br18]
gi910743180	7.25	3	1	193	20.8	6.13	7.64	nickel-binding periplasmic protein [Arthrobacter sp. Hiyo8]
gi359306548	10.34	2	1	145	15.8	5.30	7.64	hypothetical protein ARGLB_037_01050 [Arthrobacter globiformis NBRC 12137]
gi916710559	1.64	2	1	548	60.6	8.87	7.64	hypothetical protein [Arthrobacter sp. CAL618]
gi928486310	1.48	3	1	675	72.5	6.70	7.63	2,4-dienoyl-CoA reductase [Arthrobacter alpinus]
gi476402312	1.60	2	1	501	53.1	5.80	7.63	inosine-5'-monophosphate dehydrogenase [Arthrobacter crystallopoietes BAB-32]
gi917022122	1.69	1	1	887	92.9	6.34	7.63	haloacid dehalogenase [Arthrobacter sp. UNC362MFTsu5.1]
gi765006101	1.39	1	1	574	62.8	6.77	7.61	long-chain fatty acid--CoA ligase [Arthrobacter sp. A3]
gi757625673	2.19	1	1	640	71.6	5.68	7.60	hypothetical protein TV39_04745 [Arthrobacter sp. SPG23]
gi651502464	1.40	1	1	1216	127.9	5.53	7.59	chromosome segregation protein SMC [Arthrobacter sp. 35W]
gi917022133	3.79	7	1	343	37.4	6.01	7.59	hypothetical protein [Arthrobacter sp. UNC362MFTsu5.1]
gi910742478	2.11	1	1	616	66.8	5.80	7.58	ATP-dependent Clp protease ATP-binding subunit ClpE [Arthrobacter sp. Hiyo8]
gi651502286	1.87	1	1	535	58.7	5.27	7.57	peptide chain release factor 3 [Arthrobacter sp. 35W]
gi928487111	3.54	2	1	311	33.9	9.82	7.57	recombinase XerC [Arthrobacter alpinus]
gi654818153	2.10	3	2	1622	178.5	6.07	7.57	glutamate dehydrogenase [Arthrobacter sp. UNC362MFTsu5.1]
gi760166555	8.44	4	1	225	23.8	4.78	7.57	methyltransferase [Arthrobacter crystallopoietes]
gi927032118	4.14	1	1	362	39.9	9.98	7.57	transposase [Arthrobacter sp. LS16]
gi823666801	2.22	3	1	810	86.5	6.28	7.57	transcription accessory protein [Arthrobacter sp. YC-RL1]
gi937262373	7.12	1	1	267	28.8	9.47	7.56	cell division ATP-binding protein FtsE [Arthrobacter sp. Edens01]
gi116612243	4.08	1	1	515	52.6	5.96	7.55	aldehyde dehydrogenase [Arthrobacter sp. FB24]
gi765004940	6.60	2	2	409	43.9	9.72	7.54	transposase [Arthrobacter sp. A3]
gi916358036	1.78	1	1	561	60.6	5.77	7.53	acetolactate synthase [Arthrobacter sp. 131MFCol6.1]
gi742070313	6.49	3	1	308	33.3	9.67	7.53	tyrosine recombinase XerC [Arthrobacter sp. MWB30]
gi652424573	2.00	2	1	998	111.4	5.31	7.51	alpha-mannosidase [Arthrobacter castelli]
gi937258845	3.29	1	1	334	35.7	5.17	7.50	glyceraldehyde-3-phosphate dehydrogenase [Arthrobacter sp. Edens01]
gi443480396	7.52	2	1	226	25.7	5.73	7.47	RNA polymerase sigma factor SigA [Arthrobacter nitrophenolicus]
gi759702539	3.23	1	1	310	32.8	4.86	7.47	phosphoesterase [Arthrobacter globiformis]

gi651437717	2.55	1	1	432	47.0	4.97	7.46	hypothetical protein [Arthrobacter sp. H14]
gi517592070	11.11	3	1	153	16.6	10.14	7.45	MarR family transcriptional regulator [Arthrobacter sp. 135MFCol5.1]
gi476400347	1.57	1	1	572	61.8	6.13	7.44	succinate dehydrogenase flavoprotein subunit [Arthrobacter crystallopoietes BAB-32]
gi723610519	4.95	4	1	444	46.7	4.97	7.44	hypothetical protein ART_4296 [Arthrobacter sp. PAMC25486]
gi403230588	4.07	2	1	491	51.8	9.45	7.43	MFS-type transporter [Arthrobacter sp. Rue61a]
gi737808666	2.25	1	1	622	66.6	5.07	7.43	amylo-alpha-1,6-glucosidase [Arthrobacter sp. H5]
gi737802478	6.49	1	1	416	44.1	6.35	7.41	histidine kinase [Arthrobacter castelli]
gi737787378	10.78	1	1	102	11.2	9.82	7.41	50S ribosomal protein L21 [Arthrobacter albus]
gi749401933	7.14	1	1	308	33.3	5.57	7.41	PucR family transcriptional regulator, partial [Arthrobacter sp. AK-YN10]
gi515766930	1.37	1	1	878	95.0	5.41	7.40	trehalose phosphatase [Arthrobacter sp. M2012083]
gi767257660	1.80	1	1	555	60.4	7.18	7.40	DNA helicase [Arthrobacter sp. IHBB 11108]
gi359307188	5.63	2	1	284	30.8	7.64	7.40	putative oxidoreductase [Arthrobacter globiformis NBRC 12137]
gi517598084	3.80	8	1	527	56.8	9.50	7.38	signal recognition particle protein [Arthrobacter sp. 162MFSha1.1]
gi640203952	6.87	3	1	335	35.7	9.19	7.37	peptide ABC transporter ATP-binding protein [Arthrobacter sp. 31Y]
gi170783480	4.28	8	1	397	43.1	5.77	7.36	putative oxidoreductase domain protein (plasmid) [Arthrobacter sp. AK-1]
gi930827072	3.93	2	1	280	28.5	4.86	7.36	pyrroline-5-carboxylate reductase [Arthrobacter arilaitensis]
gi914713760	1.81	5	1	775	83.7	6.07	7.35	hypothetical protein [Arthrobacter sp. ZBG10]
gi918268509	5.88	4	1	340	38.5	7.80	7.35	trehalose-phosphate synthase [Arthrobacter sp. Hiyo1]
gi651485724	2.79	3	1	574	62.6	6.83	7.33	glycerol-3-phosphate dehydrogenase [Arthrobacter sp. Br18]
gi918267322	5.49	3	1	237	25.1	7.49	7.33	alpha/Beta hydrolase fold [Arthrobacter sp. Hiyo1]
gi470217673	5.43	4	2	552	55.9	4.77	7.32	phosphoenolpyruvate-protein phosphotransferase [Arthrobacter gangotriensis Lz1y]
gi443479811	1.80	1	1	555	59.4	5.14	7.32	monooxygenase FAD-binding protein [Arthrobacter nitrophenolicus]
gi651495855	5.22	1	1	268	27.7	5.03	7.28	SDR family oxidoreductase [Arthrobacter sp. H20]
gi927031643	5.09	3	1	334	35.9	7.56	7.27	methionine ABC transporter ATP-binding protein [Arthrobacter sp. LS16]
gi651446800	0.90	1	1	1217	129.6	4.97	7.27	urea carboxylase [Arthrobacter nicotinovorans]
gi674645832	12.47	3	2	361	37.4	5.77	7.25	hypothetical protein BN1051_02030 [Arthrobacter sp. 11W110_air]
gi651506959	0.90	1	1	1004	106.9	6.98	7.25	hypothetical protein [Arthrobacter sp. 35W]
gi470217250	2.30	3	1	610	66.0	8.79	7.24	stage V sporulation protein D [Arthrobacter gangotriensis Lz1y]
gi742856703	2.97	1	1	472	51.6	6.14	7.24	oxidoreductase [Arthrobacter sp. W1]
gi823666859	4.78	1	1	335	35.4	4.94	7.23	aldo/keto reductase [Arthrobacter sp. YC-RL1]
gi542106556	1.07	1	1	1220	131.9	4.97	7.23	methionine synthase [Arthrobacter sp. AK-YN10]
gi651438297	3.52	2	1	426	45.7	4.67	7.22	enolase [Arthrobacter sp. H14]
gi652423530	1.42	3	1	1622	183.2	4.94	7.21	glutamate dehydrogenase [Arthrobacter castelli]
gi910697394	1.83	3	1	710	77.2	5.06	7.21	mannosylglycerate hydrolase [Arthrobacter sp. Hiyo6]
gi916691453	9.35	2	1	246	25.9	6.04	7.21	hypothetical protein [Arthrobacter castelli]
gi162953844	1.48	3	1	1626	180.7	5.14	7.19	NAD-specific glutamate dehydrogenase [Renibacterium salmoninarum ATCC 33209]
gi910748137	6.82	4	2	308	32.2	9.54	7.19	chromosome partition protein Smc [Arthrobacter sp. Hiyo8]
gi908697758	5.16	1	1	155	15.2	6.95	7.19	hypothetical protein [Arthrobacter sp. RIT-PI-e]
gi654812862	2.57	1	1	778	84.5	6.06	7.18	hypothetical protein [Arthrobacter sp. MA-N2]
gi654815214	3.95	2	1	354	38.1	7.65	7.17	dihydroorotate dehydrogenase 2 [Arthrobacter sp. PAO19]
gi651457997	3.87	1	1	336	35.6	7.03	7.17	DNA polymerase III subunit delta [Arthrobacter sp. 35/47]
gi654817209	2.31	9	1	563	59.8	5.60	7.16	PucR family transcriptional regulator [Arthrobacter sp. UNC362MFTsu5.1]
gi919108184	3.42	2	2	1198	122.3	6.19	7.16	hypothetical protein [Arthrobacter sp. IHBB 11108]
gi927293998	13.77	3	2	167	18.2	4.51	7.15	hypothetical protein AL755_08120 [Arthrobacter sp. ERGS1:01]
gi651430481	2.19	2	1	456	49.7	10.18	7.14	hypothetical protein [Arthrobacter sanguinis]
gi737787152	3.86	4	1	363	40.6	7.44	7.14	ACP synthase [Arthrobacter albus]
gi443480776	1.85	2	1	702	76.5	7.08	7.14	acyl-CoA oxidase domain-containing protein [Arthrobacter nitrophenolicus]
gi323469345	3.65	3	1	384	40.0	5.54	7.14	ADP-heptose:LPS heptosyltransferase [Arthrobacter phenanthrenivorans Sphe3]
gi651438494	5.32	3	1	263	28.7	6.16	7.12	alpha/beta hydrolase [Arthrobacter sp. H14]
gi786032010	31.78	4	2	107	11.6	4.96	7.11	GTP cyclohydrolase [Arthrobacter chlorophenolicus]
gi930825570	2.98	3	1	671	72.3	5.11	7.09	hypothetical protein AOZ07_04190 [Arthrobacter arilaitensis]
gi759735038	14.41	4	1	118	12.9	6.79	7.09	hypothetical protein [Arthrobacter sp. L77]
gi651503346	5.32	2	2	545	58.0	5.10	7.09	heme ABC transporter ATP-binding protein [Arthrobacter sp. 35W]
gi636844516	3.28	1	1	457	48.5	4.93	7.09	succinate-semialdehyde dehydrogenase [Arthrobacter sp. TB 26]
gi767257555	8.78	5	2	410	46.2	9.98	7.08	hypothetical protein UM93_07750 [Arthrobacter sp. IHBB 11108]

gi119947828	3.80	2	1	368	38.6	6.19	7.07	putative myo-inositol 2-dehydrogenase [Arthrobacter aurescens TC1]
gi928487574	1.01	1	1	1382	151.2	5.55	7.06	AAA family ATPase [Arthrobacter alpinus]
gi916871367	14.66	1	1	116	13.3	9.38	7.05	AsnC family transcriptional regulator [Arthrobacter sp. H5]
gi651468249	3.11	5	1	515	54.2	7.50	7.04	hypothetical protein [Arthrobacter nicotinovorans]
gi551254359	2.98	2	1	504	53.5	5.85	7.04	methylmalonate-semialdehyde dehydrogenase [Arthrobacter sp. PAO19]
gi910251229	2.58	2	1	854	90.0	9.23	7.04	transglutaminase [Arthrobacter siccitolerans]
gi917442113	4.79	2	2	480	51.4	4.96	7.03	aspartate ammonia-lyase [Arthrobacter albus]
gi928486287	20.54	2	2	185	20.8	5.20	7.02	ribosome-recycling factor [Arthrobacter alpinus]
gi723609786	12.50	7	1	88	9.3	9.60	7.02	hypothetical protein ART_3563 [Arthrobacter sp. PAMC25486]
gi517023959	6.96	1	1	316	33.0	6.80	7.02	prephenate dehydratase [Salinispora pacifica]
gi219858748	3.35	2	1	686	73.4	5.67	7.02	conserved hypothetical protein [Arthrobacter chlorophenolicus A6]
gi651491342	3.01	2	1	565	61.4	6.84	7.01	3'-5' exonuclease [Arthrobacter sp. H20]
gi908690701	1.26	1	1	792	82.4	5.77	7.01	hypothetical protein [Arthrobacter sp. H41]
gi742855770	7.97	4	1	276	28.5	4.89	7.01	nicotinate-nucleotide pyrophosphorylase [Arthrobacter sp. W1]
gi651431275	4.15	2	1	434	47.5	6.65	7.01	exodeoxyribonuclease VII large subunit [Arthrobacter sanguinis]
gi517605286	4.19	1	1	310	33.2	8.54	7.00	LysR family transcriptional regulator [Arthrobacter sp. 131MFCol6.1]
gi764161672	13.19	9	1	144	16.2	5.38	7.00	hypothetical protein ArV1_071 [Arthrobacter phage vB_ArtM-ArV1]
gi359305402	8.16	7	2	331	34.2	7.85	6.99	putative ABC transporter substrate-binding protein [Arthrobacter globiformis NBRC 12137]
gi927033011	6.80	2	1	353	38.9	9.29	6.99	cytochrome C biogenesis protein [Arthrobacter sp. LS16]
gi786028847	1.18	1	1	1190	128.5	5.26	6.96	chromosome segregation protein SMC [Arthrobacter chlorophenolicus]
gi517601220	4.21	2	1	309	34.5	5.39	6.96	hypothetical protein [Arthrobacter sp. 162MFSha1.1]
gi916834713	4.52	8	1	310	32.1	9.57	6.96	hypothetical protein [Arthrobacter sp. H14]
gi742072402	23.08	4	2	52	5.4	12.02	6.96	hypothetical protein ANMWB30_07730 [Arthrobacter sp. MWB30]
gi542106319	0.87	1	1	1147	125.5	5.54	6.94	ATP-binding protein [Arthrobacter sp. AK-YN10]
gi443482459	2.17	2	1	692	74.5	6.95	6.93	acyltransferase [Arthrobacter nitrophenolicus]
gi759734851	4.39	2	1	319	32.6	5.85	6.92	methionyl-tRNA formyltransferase [Arthrobacter sp. L77]
gi476400051	10.81	1	1	148	15.6	4.79	6.92	OsmC-like protein [Arthrobacter crystallopoietes BAB-32]
gi652424923	3.39	4	1	354	38.2	4.88	6.92	hypothetical protein [Arthrobacter castelli]
gi910746224	7.69	2	1	273	28.5	5.34	6.92	putative monooxygenase YxeK [Arthrobacter sp. Hiyo8]
gi648224374	12.02	6	2	233	24.7	9.91	6.92	ABC transporter ATP-binding protein [Arthrobacter sp. M2012083]
gi910697555	3.56	1	1	562	61.6	5.19	6.91	DNA-directed RNA polymerase subunit beta [Arthrobacter sp. Hiyo6]
gi737813310	8.48	3	2	342	36.0	8.38	6.91	DNA polymerase III subunit delta [Arthrobacter sp. H14]
gi636843843	9.20	2	1	250	25.4	7.42	6.90	short-chain dehydrogenase [Arthrobacter sp. TB 26]
gi551256426	1.91	2	1	471	52.7	5.78	6.90	glycosyltransferase family 2 [Arthrobacter sp. PAO19]
gi648574590	4.60	3	1	261	28.0	5.10	6.90	MULTISPECIES: iron ABC transporter ATP-binding protein [Arthrobacter]
gi443480105	10.04	7	1	239	24.6	5.33	6.90	xylanase/chitin deacetylase [Arthrobacter nitrophenolicus]
gi937257965	5.93	1	1	270	29.4	10.70	6.90	hypothetical protein AO716_07450 [Arthrobacter sp. Edens01]
gi723608233	7.51	1	1	173	19.0	4.98	6.89	hypothetical protein ART_2010 [Arthrobacter sp. PAMC25486]
gi323468508	4.82	2	1	436	45.6	6.25	6.88	hypothetical protein Asphe3_10060 [Arthrobacter phenanthrenivorans Sphe3]
gi723607973	6.53	4	1	352	36.6	5.31	6.87	3-hydroxyisobutyryl-CoA hydrolase [Arthrobacter sp. PAMC25486]
gi651438300	2.55	2	1	509	55.6	5.74	6.87	ABC transporter ATP-binding protein [Arthrobacter sp. H14]
gi910251429	1.40	1	1	1002	108.6	6.46	6.87	hypothetical protein [Arthrobacter siccitolerans]
gi517605536	1.93	2	1	1038	108.6	5.07	6.87	hypothetical protein [Arthrobacter sp. 131MFCol6.1]
gi162955208	2.38	2	1	589	66.4	9.77	6.86	decaprenyl-phosphate-mannose--protein mannosyltransferase [Renibacterium salmoninarum
gi651499186	6.21	1	1	145	15.4	8.79	6.86	hypothetical protein [Arthrobacter sp. 35W]
gi162952963	4.18	2	1	526	56.8	5.45	6.86	GTP-binding protein [Renibacterium salmoninarum ATCC 33209]
gi757625391	4.44	2	1	315	32.6	4.93	6.85	exopolyphosphatase [Arthrobacter sp. SPG23]
gi928985695	2.75	2	1	473	48.7	9.54	6.85	hypothetical protein [Arthrobacter sp. ERGS1:01]
gi914713789	5.73	2	1	157	16.9	6.19	6.85	ArsR family transcriptional regulator [Arthrobacter sp. ZBG10]
gi219860985	9.61	1	1	229	23.7	7.46	6.85	short-chain dehydrogenase/reductase SDR [Arthrobacter chlorophenolicus A6]
gi823666980	3.25	2	1	707	74.7	6.99	6.85	nitrite reductase [Arthrobacter sp. YC-RL1]
gi307743838	2.19	2	1	547	58.2	5.07	6.84	thiamine pyrophosphate binding domain-containing protein [Arthrobacter arilaitensis Re117]
gi654812752	10.00	3	2	320	34.4	6.33	6.82	acyl dehydratase [Arthrobacter sp. MA-N2]
gi640196826	2.78	1	1	756	77.8	6.38	6.81	carbonic anhydrase [Arthrobacter sp. 31Y]
gi119947949	3.93	2	1	433	45.4	4.94	6.79	putative serpin (serine proteinase inhibitor) family protein [Arthrobacter aurescens TC1]

gi908690795	4.29	4	1	513	54.4	7.58	6.79	FAD-dependent oxidoreductase [Arthrobacter sp. H41]
gi359306186	2.57	2	2	1051	110.0	4.97	6.79	putative glycosidase [Arthrobacter globiformis NBRC 12137]
gi651436602	3.05	3	1	459	50.0	7.81	6.78	MFS transporter [Arthrobacter sp. H41]
gi742857001	6.10	1	1	213	23.0	6.70	6.78	methyltransferase [Arthrobacter sp. W1]
gi916871536	8.26	2	2	351	37.4	5.88	6.77	hypothetical protein [Arthrobacter sp. H5]
gi742754774	1.91	1	1	732	76.8	6.93	6.77	nitrite reductase [Arthrobacter phenanthrenivorans]
gi654811984	5.10	2	1	294	32.1	10.20	6.77	secretion system protein [Arthrobacter sp. MA-N2]
gi403231679	5.76	1	1	243	26.6	4.61	6.77	hypothetical protein ARUE_c42290 [Arthrobacter sp. Rue61a]
gi323471395	6.79	5	1	265	27.6	4.82	6.76	Zn-dependent alcohol dehydrogenase, class III (plasmid) [Arthrobacter phenanthrenivorans
gi162953256	5.88	1	1	255	27.4	4.69	6.76	putative lipoprotein [Renibacterium salmoninarum ATCC 33209]
gi759734368	4.74	2	1	359	36.7	7.11	6.76	bifunctional diaminohydroxyphosphoribosylaminopyrimidine deaminase/5-amino-6-(5-phosph
gi910743997	4.78	2	1	502	52.3	5.39	6.75	bifunctional protein GlmU [Arthrobacter sp. Hiyo8]
gi916692460	1.37	6	1	873	98.4	6.24	6.74	hypothetical protein [Arthrobacter castelli]
gi937259383	14.81	3	2	216	23.5	5.36	6.74	transcriptional regulator [Arthrobacter sp. Edens01]
gi654824105	6.30	1	1	397	40.3	5.03	6.74	flagellin [Arthrobacter sp. I3]
gi470221287	4.53	2	1	331	35.4	6.18	6.74	Zinc-binding dehydrogenase family protein [Arthrobacter gangotriensis Lz1y]
gi674644286	3.72	2	1	323	35.6	9.66	6.74	Eukaryotic DNA topoisomerase I, catalytic core [Arthrobacter sp. 11W110_air]
gi307743667	1.61	7	1	1181	128.1	6.34	6.74	putative conjugal transfer protein [Arthrobacter arilaitensis Re117]
gi517592431	0.88	1	1	1248	131.7	4.97	6.73	urea amidolyase [Arthrobacter sp. 135MFCol5.1]
gi403231782	3.87	3	1	336	36.2	5.16	6.72	sporulation initiation inhibitor protein Soj [Arthrobacter sp. Rue61a]
gi651437007	3.95	2	1	430	46.7	10.83	6.72	hypothetical protein [Arthrobacter sp. H41]
gi674644781	4.90	3	1	204	21.8	6.95	6.71	HTH-type transcriptional repressor KstR2 [Arthrobacter sp. 11W110_air]
gi162953548	1.63	2	1	676	68.9	5.21	6.71	sialidase [Renibacterium salmoninarum ATCC 33209]
gi443482594	0.88	2	1	1358	142.4	6.98	6.71	DNA segregation ATPase FtsK [Arthrobacter nitrophenolicus]
gi765012041	6.10	2	1	295	31.3	6.43	6.70	ABC transporter [Arthrobacter sp. A3]
gi918269017	20.31	9	1	64	7.0	5.02	6.69	dipeptide transport system permease protein DppB [Arthrobacter sp. Hiyo1]
gi323469047	1.60	2	1	1185	130.5	5.25	6.69	DNA-directed DNA polymerase III PolC [Arthrobacter phenanthrenivorans Sphe3]
gi759704592	6.48	6	1	355	37.0	9.25	6.68	hypothetical protein [Arthrobacter globiformis]
gi749401543	5.63	1	1	302	32.2	9.38	6.68	FAD-binding dehydrogenase, partial [Arthrobacter sp. AK-YN10]
gi723610320	3.85	3	2	986	105.8	6.35	6.67	hypothetical protein ART_4097 [Arthrobacter sp. PAMC25486]
gi476401847	3.86	2	1	337	36.9	7.20	6.67	ATP dependent DNA ligase [Arthrobacter crystallopoietes BAB-32]
gi737796581	1.65	1	1	1151	123.5	5.95	6.67	1-pyrroline-5-carboxylate dehydrogenase [Arthrobacter sp. H20]
gi765007133	4.43	1	1	361	38.8	7.69	6.67	methionine ABC transporter ATP-binding protein [Arthrobacter sp. A3]
gi937262564	8.29	3	1	217	23.1	6.11	6.67	ABC transporter ATP-binding protein [Arthrobacter sp. Edens01]
gi652423784	3.20	2	1	563	59.7	5.88	6.66	formate--tetrahydrofolate ligase [Arthrobacter castelli]
gi908696808	3.61	1	1	527	56.0	5.49	6.66	GTPase CgtA [Arthrobacter sp. RIT-PI-e]
gi930825401	9.27	1	1	259	28.0	4.92	6.66	hypothetical protein AOZ07_03210 [Arthrobacter arilaitensis]
gi219859596	3.38	2	2	650	71.5	5.01	6.66	Endothelin-converting enzyme 1 [Arthrobacter chlorophenolicus A6]
gi916863381	5.91	1	1	254	27.8	5.91	6.65	GlcNAc-PI de-N-acetylase [Arthrobacter sp. 35/47]
gi908690403	4.00	1	1	425	46.3	6.68	6.65	exodeoxyribonuclease VII large subunit [Arthrobacter sp. H41]
gi517599801	4.69	1	1	469	48.9	4.89	6.65	aldehyde dehydrogenase [Arthrobacter sp. 162MFSha1.1]
gi737811255	6.02	2	1	299	32.6	6.70	6.64	peptidase M24, partial [Arthrobacter sp. 35/47]
gi654827277	4.39	2	1	433	46.2	6.15	6.64	SAM-dependent methyltransferase [Arthrobacter sp. H5]
gi737787422	4.71	7	1	297	31.5	5.22	6.64	pyridoxal biosynthesis lyase PdxS [Arthrobacter albus]
gi654819157	3.39	3	1	383	40.9	8.95	6.64	diguanylate cyclase [Arthrobacter sp. UNC362MFTsu5.1]
gi674645444	4.12	1	1	437	45.8	8.48	6.64	Oxygen regulatory protein NreC [Arthrobacter sp. 11W110_air]
gi651431158	3.44	1	1	262	28.0	4.92	6.64	alpha/beta hydrolase [Arthrobacter sanguinis]
gi551256928	12.86	2	1	70	7.4	4.88	6.63	MT0933-like antitoxin protein [Arthrobacter sp. PAO19]
gi116609026	5.81	2	1	344	37.2	10.29	6.63	NAD-dependent epimerase/dehydratase [Arthrobacter sp. FB24]
gi759747114	5.79	1	1	242	26.8	6.16	6.63	DNA alkylation repair protein [Arthrobacter sp. 31Y]
gi651431618	3.92	2	1	332	35.3	6.67	6.63	hypothetical protein [Arthrobacter sanguinis]
gi551254395	3.07	3	1	618	66.5	7.37	6.63	ABC transporter [Arthrobacter sp. PAO19]
gi470215932	2.55	1	1	353	37.4	9.38	6.62	binding-protein-dependent transport system inner membrane protein [Arthrobacter gangotrie
gi470216410	0.68	1	1	1611	170.0	6.06	6.62	ATP-dependent helicase [Arthrobacter gangotriensis Lz1y]
gi654822191	2.76	1	1	688	70.4	5.19	6.61	5-oxoprolinase [Arthrobacter sp. I3]

gi651443101	0.62	1	1	1441	156.3	7.85	6.61	peptide synthetase [Arthrobacter sp. 9MFCol3.1]
gi910743300	3.96	3	1	328	35.8	6.02	6.59	probable aldo-keto reductase 2 [Arthrobacter sp. Hiyo8]
gi910693554	15.09	2	1	106	11.7	4.88	6.59	triostin synthetase I [Arthrobacter sp. Hiyo6]
gi470217279	2.75	1	1	545	58.7	4.65	6.59	family 5 extracellular solute-binding protein [Arthrobacter gangotriensis Lz1y]
gi162954191	2.37	2	1	337	35.9	6.02	6.59	3-dehydroquinate synthase [Renibacterium salmoninarum ATCC 33209]
gi786028637	2.73	5	2	1025	107.1	5.03	6.58	chromosome segregation protein SMC [Arthrobacter chlorophenolicus]
gi652423331	23.47	4	1	98	10.4	4.75	6.58	molecular chaperone GroES [Arthrobacter castelli]
gi652424912	2.46	1	1	284	30.5	10.71	6.58	type II secretion system protein F [Arthrobacter castelli]
gi551253699	1.99	1	1	1104	121.5	5.00	6.57	ATP-dependent DNA helicase [Arthrobacter sp. PAO19]
gi651440408	11.66	2	1	163	17.7	4.75	6.57	hypothetical protein [Arthrobacter sp. H14]
gi823666890	3.07	1	1	456	47.9	9.61	6.57	ATPase [Arthrobacter sp. YC-RL1]
gi908690231	5.00	5	1	360	39.3	5.08	6.57	phenylalanine--tRNA ligase subunit alpha [Arthrobacter sp. H41]
gi323467661	8.86	3	2	237	26.2	6.34	6.57	transcriptional regulator [Arthrobacter phenanthrenivorans Sphe3]
gi359305985	3.29	4	1	578	61.0	5.25	6.56	putative two-component histidine kinase [Arthrobacter globiformis NBRC 12137]
gi910283552	5.54	2	1	343	36.8	5.74	6.56	hypothetical protein [Arthrobacter sp. A3]
gi640200020	5.95	1	1	252	28.2	5.54	6.56	MerR family transcriptional regulator [Arthrobacter sp. 31Y]
gi648572636	5.04	1	1	238	25.5	6.35	6.56	histidine kinase [Arthrobacter sp. 135MFCol5.1]
gi910249980	0.44	1	1	1361	142.1	7.78	6.56	hypothetical protein [Arthrobacter siccitolerans]
gi760166910	1.67	1	1	836	85.1	4.32	6.55	hypothetical protein [Arthrobacter crystallopoietes]
gi403229371	4.16	1	1	409	45.7	5.12	6.54	putative cytochrome P450 [Arthrobacter sp. Rue61a]
gi759736450	1.13	2	1	887	94.2	5.36	6.53	hypothetical protein [Arthrobacter sp. L77]
gi910745609	3.86	1	1	337	36.2	4.98	6.53	DNA integrity scanning protein DisA [Arthrobacter sp. Hiyo8]
gi737809772	4.32	2	1	301	32.7	10.10	6.52	hypothetical protein [Arthrobacter sp. 35/47]
gi517602032	2.58	1	1	581	62.4	5.11	6.52	hypothetical protein [Arthrobacter sp. 162MFSha1.1]
gi723608409	4.83	2	1	290	31.3	6.06	6.52	citrate lyase subunit beta [Arthrobacter sp. PAMC25486]
gi742859436	4.88	5	1	430	45.7	9.60	6.51	two-component system sensor histidine kinase [Arthrobacter sp. W1]
gi916259981	4.42	4	1	430	46.2	6.60	6.51	histidine kinase [Arthrobacter sp. TB 23]
gi307745088	9.35	5	1	246	26.5	5.53	6.51	putative CbiX family protein [Arthrobacter arilaitensis Re117]
gi651434522	3.17	2	1	379	40.6	5.01	6.50	homoserine acetyltransferase [Arthrobacter sp. H41]
gi654827570	12.14	1	1	140	15.8	11.24	6.49	heat-shock protein [Arthrobacter sp. H5]
gi737775726	17.65	2	2	170	18.8	9.70	6.49	hypothetical protein [Arthrobacter sp. MA-N2]
gi916782430	5.54	2	1	325	34.3	5.19	6.48	mandelate racemase [Arthrobacter sp. 35W]
gi917013309	8.77	1	1	171	19.3	5.80	6.47	hypothetical protein [Arthrobacter sanguinis]
gi917022132	5.41	1	1	314	33.1	5.02	6.47	aldo/keto reductase [Arthrobacter sp. UNC362MFTsu5.1]
gi654826089	0.99	3	1	1618	178.0	6.60	6.46	hypothetical protein [Arthrobacter sp. H5]
gi723609355	4.84	4	1	310	33.5	10.08	6.46	sensor histidine kinase [Arthrobacter sp. PAMC25486]
gi651455211	2.99	4	1	468	52.2	6.09	6.46	hypothetical protein [Arthrobacter nicotinovorans]
gi654826511	1.69	1	1	949	100.9	5.66	6.45	glycine dehydrogenase [Arthrobacter sp. H5]
gi639130259	2.38	2	1	589	66.7	5.19	6.45	hypothetical protein [Arthrobacter sp. CAL618]
gi910697367	5.47	1	1	201	22.9	6.65	6.45	biphenyl dioxygenase subunit beta [Arthrobacter sp. Hiyo6]
gi651500577	2.60	2	1	385	38.8	5.33	6.45	hypothetical protein [Arthrobacter sp. 35W]
gi403228078	4.93	5	1	345	37.0	5.16	6.45	putative oxidoreductase [Arthrobacter sp. Rue61a]
gi517604094	0.87	2	1	1723	181.0	5.91	6.44	DEAD/DEAH box helicase [Arthrobacter sp. 131MFCol6.1]
gi916691436	2.51	4	1	399	41.9	9.39	6.44	hypothetical protein [Arthrobacter castelli]
gi930824883	3.82	1	1	471	50.4	9.28	6.44	MFS transporter [Arthrobacter arilaitensis]
gi551256727	3.40	2	1	530	56.6	6.34	6.44	phytoene dehydrogenase [Arthrobacter sp. PAO19]
gi651453770	3.62	1	1	387	40.4	6.23	6.43	membrane protein [Arthrobacter nicotinovorans]
gi518312740	5.51	2	1	345	36.0	5.71	6.43	hypothetical protein [Arthrobacter sp. TB 23]
gi916290016	2.96	2	1	371	40.3	6.21	6.42	MULTISPECIES: cyclic pyranopterin phosphate synthase MoaA [Arthrobacter]
gi786027369	4.00	8	1	350	34.2	5.54	6.42	glycoside hydrolase, partial [Arthrobacter chlorophenolicus]
gi640199153	1.29	2	1	855	95.6	6.57	6.42	hypothetical protein [Arthrobacter sp. 31Y]
gi757622607	14.53	7	1	172	18.4	6.70	6.41	MarR family transcriptional regulator [Arthrobacter sp. SPG23]
gi908690437	8.81	1	1	159	17.9	8.41	6.41	hypothetical protein [Arthrobacter sp. H41]
gi219858261	5.17	1	1	348	36.2	5.35	6.40	UspA domain protein [Arthrobacter chlorophenolicus A6]
gi515766293	1.78	1	1	841	90.8	5.91	6.40	DNA topoisomerase IV subunit A [Arthrobacter sp. M2012083]

gi476401370	8.02	1	1	237	24.9	6.95	6.40	redox-sensing transcriptional repressor Rex [Arthrobacter crystallopoietes BAB-32]
gi652423808	12.82	3	1	156	17.4	7.28	6.39	succinate dehydrogenase [Arthrobacter castelli]
gi518313564	2.75	1	1	437	46.5	6.39	6.39	hypothetical protein [Arthrobacter sp. TB 23]
gi119951470	1.80	1	1	1165	128.7	5.05	6.38	helicase, SNF2 family (plasmid) [Arthrobacter aurescens TC1]
gi937258918	6.17	1	1	373	40.4	8.07	6.38	DNA alkylation repair protein [Arthrobacter sp. Edens01]
gi504874821	5.43	2	1	221	23.6	4.64	6.38	alkylmercury lyase [Arthrobacter sp. J3-40]
gi914715090	0.43	1	1	3508	366.0	5.10	6.38	non-ribosomal peptide synthetase [Arthrobacter sp. ZBG10]
gi654817508	2.47	2	1	527	54.1	6.07	6.38	ABC transporter [Arthrobacter sp. UNC362MFTsu5.1]
gi654815894	2.60	3	2	962	105.9	6.79	6.38	RNA helicase [Arthrobacter sp. UNC362MFTsu5.1]
gi518311459	3.41	13	1	440	45.7	4.87	6.38	MULTISPECIES: hypothetical protein [Arthrobacter]
gi908698822	15.15	6	1	132	14.6	9.45	6.38	30S ribosomal protein S16 [Arthrobacter sp. RIT-PI-e]
gi652424277	5.42	1	1	369	40.9	6.16	6.37	hypothetical protein [Arthrobacter castelli]
gi914715602	1.94	3	1	773	83.0	5.00	6.37	maltooligosyl trehalose synthase [Arthrobacter sp. ZBG10]
gi908690617	2.01	3	1	448	47.8	5.26	6.36	acetyl-CoA carboxylase [Arthrobacter sp. H41]
gi170783524	11.11	1	1	198	21.6	9.96	6.36	putative site-specific recombinase (plasmid) [Arthrobacter sp. AK-1]
gi908698559	1.24	1	1	1612	172.9	6.55	6.35	hypothetical protein [Arthrobacter sp. RIT-PI-e]
gi757623662	3.99	2	1	276	28.8	5.58	6.35	fumarylacetoacetate hydrolase [Arthrobacter sp. SPG23]
gi651458056	1.23	3	1	649	71.8	5.08	6.34	peptidase M13 [Arthrobacter sp. 35/47]
gi517609075	2.60	1	1	384	41.7	9.45	6.34	DNA polymerase IV [Arthrobacter sp. 161MFSha2.1]
gi652425848	7.76	4	1	245	26.2	6.55	6.34	uridylate kinase [Arthrobacter castelli]
gi760111938	3.03	1	1	462	50.6	5.27	6.34	magnesium chelatase [Arthrobacter chlorophenolicus]
gi759731418	3.40	1	1	589	63.8	4.92	6.34	glycoside hydrolase [Arthrobacter sp. L77]
gi759729737	6.17	2	1	324	34.9	9.98	6.34	3-methyladenine DNA glycosylase [Arthrobacter sp. L77]
gi654823193	7.61	2	1	184	19.5	7.68	6.34	haloacid dehalogenase [Arthrobacter sp. I3]
gi476402610	2.50	1	1	360	38.7	5.12	6.33	Holliday junction DNA helicase RuvB [Arthrobacter crystallopoietes BAB-32]
gi759763875	4.12	4	1	340	36.0	5.22	6.33	fructose 1,6-bisphosphatase [Arthrobacter gangotriensis]
gi654828174	3.93	1	1	356	39.2	5.96	6.32	cyclic pyranopterin phosphate synthase MoaA [Arthrobacter sp. H5]
gi927294015	6.93	1	1	332	35.9	5.96	6.32	16S rRNA methyltransferase [Arthrobacter sp. ERGS1:01]
gi742758541	4.78	1	1	356	36.9	6.70	6.32	tRNA(Ile)-lysidine synthetase [Arthrobacter phenanthrenivorans]
gi742859140	2.51	2	1	478	50.3	5.31	6.32	flavoprotein [Arthrobacter sp. W1]
gi403228039	3.30	1	1	273	29.5	5.94	6.32	Fe3+-siderophore ABC transporter, ATP-binding protein [Arthrobacter sp. Rue61a]
gi636843797	3.35	3	1	478	51.9	9.92	6.31	oxidoreductase [Arthrobacter sp. TB 26]
gi918265741	3.08	3	1	422	45.1	9.33	6.31	uncharacterized protein MJ1024 [Arthrobacter sp. Hiyo1]
gi927292803	5.99	1	1	284	30.7	6.60	6.30	glutamine amidotransferase (plasmid) [Arthrobacter sp. ERGS1:01]
gi737789562	3.73	1	1	402	42.4	5.31	6.30	signal recognition particle-docking protein FtsY [Arthrobacter albus]
gi786026898	4.38	3	2	958	97.1	8.07	6.30	hypothetical protein [Arthrobacter chlorophenolicus]
gi917021963	7.49	1	1	227	22.8	5.29	6.30	keto-deoxy-phosphogluconate aldolase [Arthrobacter sp. UNC362MFTsu5.1]
gi908642132	5.91	1	1	186	20.2	5.87	6.30	RNA polymerase subunit sigma-70 [Arthrobacter phenanthrenivorans]
gi651497863	3.05	2	1	492	52.5	4.94	6.29	S-adenosyl-L-homocysteine hydrolase [Arthrobacter sp. 35W]
gi930827483	8.37	2	1	263	28.4	5.12	6.29	hypothetical protein AOZ07_15140 [Arthrobacter arilaitensis]
gi910283916	4.33	2	1	254	26.0	9.61	6.29	hypothetical protein [Arthrobacter sp. A3]
gi916692014	3.44	1	1	407	44.9	6.10	6.29	carbohydrate-binding protein [Arthrobacter castelli]
gi518313676	25.37	7	1	67	7.2	4.72	6.28	MULTISPECIES: cold-shock protein [Arthrobacter]
gi928486765	5.95	6	1	353	38.4	5.44	6.28	lipoate--protein ligase [Arthrobacter alpinus]
gi759704530	2.40	1	1	499	54.3	5.74	6.27	hypothetical protein [Arthrobacter globiformis]
gi517604021	3.78	1	1	344	35.0	8.81	6.27	hypothetical protein [Arthrobacter sp. 131MFCol6.1]
gi910283799	9.36	3	1	171	18.6	9.94	6.27	30S ribosomal protein S9 [Arthrobacter sp. A3]
gi916692274	3.94	1	1	507	54.6	4.87	6.27	histidine kinase [Arthrobacter castelli]
gi937256401	4.36	2	1	505	54.2	4.81	6.27	oxidoreductase [Arthrobacter sp. Edens01]
gi443481304	7.92	2	1	101	11.7	5.73	6.27	hypothetical protein G205_13072 [Arthrobacter nitrophenolicus]
gi636847109	3.44	2	1	465	51.6	7.43	6.27	hypothetical protein [Arthrobacter sp. TB 26]
gi910738356	3.99	2	1	426	46.8	9.60	6.27	uncharacterized oxidoreductase y4hM [Arthrobacter sp. Hiyo4]
gi737808298	0.76	1	1	1181	127.6	6.57	6.27	conjugal transfer protein [Arthrobacter sp. H5]
gi939036471	4.14	2	1	266	28.6	10.49	6.27	hypothetical protein [Arthrobacter nitroguajacolicus]
gi517599926	2.47	1	1	607	66.6	5.41	6.26	ABC transporter ATPase [Arthrobacter sp. 162MFSha1.1]

gi517603069	4.58	1	1	306	31.6	9.66	6.26	membrane protein [Arthrobacter sp. 131MFCol6.1]
gi470221631	3.14	2	1	350	38.3	5.38	6.26	hypothetical protein ADIAG_00484 [Arthrobacter gangotriensis Lz1y]
gi307743321	5.68	1	1	264	28.7	5.16	6.26	haloacid dehalogenase-like hydrolase [Arthrobacter arilaitensis Re117]
gi162954034	5.47	1	1	439	46.8	8.29	6.26	opine oxidase [Renibacterium salmoninarum ATCC 33209]
gi551256327	5.15	1	1	330	36.4	5.62	6.26	16S rRNA (cytosine(1402)-N(4))-methyltransferase [Arthrobacter sp. PAO19]
gi640198590	2.83	1	1	459	49.5	5.69	6.26	UDP-glucose 6-dehydrogenase [Arthrobacter sp. 31Y]
gi910250538	3.16	2	1	253	26.3	5.96	6.25	3-oxoacyl-ACP reductase [Arthrobacter siccitolerans]
gi654827857	3.87	3	1	388	40.2	6.80	6.25	ROK family transcriptional regulator [Arthrobacter sp. H5]
gi759734146	1.81	1	1	607	62.4	4.55	6.25	dihydrolipoamide acetyltransferase [Arthrobacter sp. L77]
gi359307048	2.43	6	1	452	46.6	5.30	6.25	D-beta-D-heptose 7-phosphate kinase/D-beta-D-heptose 1-phosphate adenosyltransferase [
gi119949312	2.53	1	1	554	60.2	5.66	6.24	ATP-dependent DNA helicase, RecQ family [Arthrobacter aurescens TC1]
gi119951337	2.07	1	1	628	68.2	5.00	6.24	putative tetratricopeptide repeat family protein [Arthrobacter aurescens TC1]
gi910249149	13.83	2	1	94	9.2	9.98	6.24	hypothetical protein [Arthrobacter siccitolerans]
gi651498748	6.30	1	1	349	36.6	9.60	6.24	translation initiation factor IF-3 [Arthrobacter sp. 35W]
gi910251605	2.20	2	1	318	33.2	7.01	6.24	diacylglycerol kinase [Arthrobacter siccitolerans]
gi765008511	2.51	1	1	1035	114.1	5.31	6.24	alpha-mannosidase [Arthrobacter sp. A3]
gi219857742	5.63	3	1	231	25.8	7.15	6.24	transcriptional regulator, GntR family [Arthrobacter chlorophenolicus A6]
gi737800851	1.72	1	1	523	58.2	6.18	6.24	copper oxidase [Arthrobacter castelli]
gi910739934	6.82	2	1	352	36.8	4.98	6.24	adenylate cyclase [Arthrobacter sp. Hiyo4]
gi654811434	6.18	2	1	275	28.7	7.27	6.24	ATP synthase subunit delta [Arthrobacter sp. MA-N2]
gi928488301	1.97	1	1	557	60.6	7.17	6.24	ribonuclease [Arthrobacter alpinus]
gi651429678	2.55	1	1	510	56.5	5.52	6.24	ribosome-associated GTPase EngA [Arthrobacter sanguinis]
gi759733764	5.67	2	1	300	30.6	5.36	6.23	succinyl-CoA synthetase subunit alpha [Arthrobacter sp. L77]
gi476402051	2.87	1	1	558	63.6	5.83	6.23	malate synthase [Arthrobacter crystallopoietes BAB-32]
gi116609117	4.33	1	1	323	34.8	5.92	6.23	transcriptional regulator, LuxR family [Arthrobacter sp. FB24]
gi737785672	1.40	2	1	428	45.2	5.07	6.23	ABC transporter substrate-binding protein [Arthrobacter nitrophenolicus]
gi517598950	3.78	2	1	397	42.7	5.71	6.23	pilus biosynthesis protein CpaE [Arthrobacter sp. 162MFSha1.1]
gi723608659	7.69	4	1	247	24.9	5.08	6.23	hypothetical protein ART_2436 [Arthrobacter sp. PAMC25486]
gi910694808	10.26	2	1	156	16.4	8.05	6.22	conserved hypothetical protein [Arthrobacter sp. Hiyo6]
gi656049967	3.02	2	1	331	34.7	5.06	6.22	prephenate dehydratase [Brevibacterium album]
gi515767277	11.21	1	1	107	11.6	4.60	6.21	hypothetical protein [Arthrobacter sp. M2012083]
gi823665823	2.02	1	1	891	96.4	5.38	6.21	hypothetical protein AA310_07830 [Arthrobacter sp. YC-RL1]
gi737803727	3.94	2	1	330	37.0	9.10	6.21	transposase, partial [Arthrobacter sp. Br18]
gi674644640	4.53	2	1	265	28.6	11.40	6.21	RDD family protein [Arthrobacter sp. 11W110_air]
gi723608035	3.25	1	1	400	42.7	5.05	6.21	UBA/THIF-type NAD/FAD binding protein [Arthrobacter sp. PAMC25486]
gi737783886	13.54	1	1	96	10.9	11.21	6.21	hypothetical protein [Arthrobacter nitrophenolicus]
gi652424589	7.48	4	1	254	26.5	5.16	6.20	SDR family oxidoreductase [Arthrobacter castelli]
gi170783527	15.24	2	1	105	11.7	5.47	6.20	unknown (plasmid) [Arthrobacter sp. AK-1]
gi910252045	4.83	2	1	269	28.5	9.52	6.20	short-chain dehydrogenase [Arthrobacter siccitolerans]
gi542107238	2.98	2	1	637	72.6	8.94	6.20	glycosyltransferase [Arthrobacter sp. AK-YN10]
gi162953197	3.88	1	1	361	39.0	5.19	6.20	glycine cleavage system T protein (aminomethyltransferase) [Renibacterium salmoninarum A
gi910740003	3.96	1	1	278	29.8	5.16	6.19	valine--tRNA ligase [Arthrobacter sp. Hiyo4]
gi757626189	1.89	1	1	1005	108.0	4.82	6.19	alpha-amylase [Arthrobacter sp. SPG23]
gi654826993	1.37	2	1	1172	129.0	5.27	6.19	ATP-binding protein [Arthrobacter sp. H5]
gi759729120	4.37	1	1	229	25.3	4.98	6.19	GntR family transcriptional regulator [Arthrobacter sp. UNC362MFTsu5.1]
gi918268088	2.32	1	1	475	52.7	9.92	6.19	conserved hypothetical protein [Arthrobacter sp. Hiyo1]
gi636846712	45.98	4	2	87	9.4	8.47	6.19	hypothetical protein [Arthrobacter sp. TB 26]
gi742758498	6.69	1	1	299	31.8	5.01	6.19	oxidoreductase [Arthrobacter phenanthrenivorans]
gi640194849	3.55	3	1	338	36.1	5.44	6.19	peptidase M4 [Arthrobacter sp. 31Y]
gi759734148	3.91	1	1	460	48.4	5.62	6.18	dihydrolipoamide dehydrogenase [Arthrobacter sp. L77]
gi910742732	5.45	1	1	257	27.1	10.15	6.18	ribosomal RNA small subunit methyltransferase E [Arthrobacter sp. Hiyo8]
gi723607408	1.75	1	1	458	49.8	8.62	6.17	hypothetical protein ART_1185 [Arthrobacter sp. PAMC25486]
gi918265928	2.57	1	1	506	55.0	8.87	6.17	xylose import ATP-binding protein XylG [Arthrobacter sp. Hiyo1]
gi759725836	3.46	1	1	433	46.5	9.52	6.17	glycosyl transferase [Arthrobacter sp. I3]
gi674644141	26.19	2	1	84	9.0	4.88	6.16	phosphoribosylformylglycinamidine synthase subunit PurS [Arthrobacter sp. 11W110_air]

gi654825967	1.90	1	1	843	92.9	5.17	6.16	DEAD/DEAH box helicase [Arthrobacter sp. H5]
gi359305774	0.60	1	1	1490	160.1	5.74	6.16	hypothetical protein ARGLB_054_00530 [Arthrobacter globiformis NBRC 12137]
gi737810496	1.22	1	1	1151	130.7	5.55	6.15	hypothetical protein [Arthrobacter sp. 35/47]
gi937258433	1.76	1	1	796	84.5	5.97	6.15	daunorubicin resistance protein DrrC [Arthrobacter sp. Edens01]
gi910747026	3.83	1	1	235	25.4	6.93	6.15	uncharacterized protein YbjT [Arthrobacter sp. Hiyo8]
gi307744487	3.42	1	1	263	29.0	9.54	6.15	putative sugar ABC transporter, ATP-binding subunit [Arthrobacter arilaitensis Re117]
gi765009391	3.54	2	1	367	39.5	6.00	6.15	3-dehydroquinate synthase [Arthrobacter sp. A3]
gi928485980	4.55	3	1	330	36.0	5.36	6.14	hypothetical protein AOC05_01410 [Arthrobacter alpinus]
gi323471037	3.64	1	1	330	35.4	4.96	6.14	putative oxidoreductase, aryl-alcohol dehydrogenase like protein [Arthrobacter phenanthreni
gi918268357	5.45	1	1	330	35.4	9.23	6.14	probable sensor histidine kinase TcrY [Arthrobacter sp. Hiyo1]
gi740685259	2.53	1	1	871	94.4	5.49	6.14	glycogen phosphorylase [Arthrobacter sp. PAMC25486]
gi767257367	9.21	1	1	239	25.3	10.67	6.14	IclR family transcriptional regulator [Arthrobacter sp. IHBB 11108]
gi651487073	3.74	1	1	374	40.2	4.83	6.14	DNA polymerase III subunit beta [Arthrobacter sp. Br18]
gi551254278	8.50	2	2	341	38.0	7.85	6.13	hypothetical protein [Arthrobacter sp. PAO19]
gi323470952	3.53	1	1	368	36.5	4.96	6.13	ABC-type antimicrobial peptide transport system, permease component [Arthrobacter phena
gi916691670	1.97	7	1	660	72.8	10.08	6.13	hypothetical protein [Arthrobacter castelli]
gi219859496	6.88	1	1	247	27.2	5.01	6.13	transcriptional regulator, MerR family [Arthrobacter chlorophenolicus A6]
gi918267670	4.53	1	1	375	39.3	10.23	6.12	O-acetyl transferase [Arthrobacter sp. Hiyo1]
gi767257349	1.09	2	1	1198	129.5	5.07	6.12	chromosome segregation protein SMC [Arthrobacter sp. IHBB 11108]
gi767258125	2.02	1	1	446	47.1	9.52	6.12	MFS transporter [Arthrobacter sp. IHBB 11108]
gi823666983	2.44	1	1	860	90.6	5.64	6.12	nitrite reductase [Arthrobacter sp. YC-RL1]
gi908698275	1.91	1	1	892	95.6	5.40	6.12	alanine--tRNA ligase [Arthrobacter sp. RIT-PI-e]
gi937261706	2.76	2	1	544	61.2	5.59	6.11	malate synthase [Arthrobacter sp. Edens01]
gi760112697	1.60	1	1	1190	128.3	5.30	6.10	chromosome segregation protein SMC [Arthrobacter chlorophenolicus]
gi918268734	23.86	1	1	88	9.5	10.21	6.10	protein translocase subunit SecE [Arthrobacter sp. Hiyo1]
gi759710090	4.61	2	1	412	45.3	9.76	6.10	sugar translocase [Arthrobacter sp. 135MFCol5.1]
gi654828673	2.60	2	1	462	49.9	4.88	6.09	glycosyl hydrolase family 32 [Arthrobacter sp. H5]
gi654823567	8.95	1	1	257	27.5	5.10	6.09	1,6-dihydroxycyclohexa-2,4-diene-1-carboxylate dehydrogenase [Arthrobacter sp. I3]
gi939036831	8.67	1	1	196	20.3	9.86	6.09	hypothetical protein [Arthrobacter nitroguajacolicus]
gi910743482	4.60	1	1	457	47.0	5.40	6.09	UDP-N-acetylmuramate--L-alanine ligase [Arthrobacter sp. Hiyo8]
gi307743859	0.90	1	1	1108	121.1	5.87	6.09	conserved hypothetical protein [Arthrobacter arilaitensis Re117]
gi652423494	1.65	2	1	726	78.5	6.83	6.09	ATP-dependent DNA helicase [Arthrobacter castelli]
gi908697697	6.27	3	1	335	35.2	5.50	6.09	hypothetical protein [Arthrobacter sp. RIT-PI-e]
gi651500178	6.37	1	1	251	27.0	5.41	6.09	carboxymuconolactone decarboxylase [Arthrobacter sp. 35W]
gi651460049	2.59	1	1	694	73.6	5.40	6.08	enoyl-CoA hydratase [Arthrobacter sp. 35/47]
gi908697627	4.24	2	1	542	56.5	4.97	6.08	bifunctional phosphoribosylaminoimidazolecarboxamide formyltransferase/inosine monophos
gi930827727	3.39	1	1	295	31.0	6.23	6.08	5,10-methylene-tetrahydrofolate cyclohydrolase [Arthrobacter arilaitensis]
gi927295589	10.31	1	1	194	19.7	4.73	6.08	50S ribosomal protein L10 [Arthrobacter sp. ERGS1:01]
gi759732757	19.30	1	1	57	5.8	6.05	6.08	hypothetical protein [Arthrobacter sp. L77]
gi908699402	5.88	3	1	289	29.8	4.41	6.08	flagellar biosynthesis protein flip [Arthrobacter sp. RIT-PI-e]
gi219859349	2.59	2	2	849	90.9	6.00	6.07	osmosensitive K+ channel signal transduction histidine kinase [Arthrobacter chlorophenolicus
gi930827859	3.40	3	1	588	64.9	6.10	6.06	peptide synthetase [Arthrobacter arilaitensis]
gi652423186	4.56	2	2	592	64.1	5.27	6.06	ABC transporter substrate-binding protein [Arthrobacter castelli]
gi908698018	6.46	1	1	325	34.9	6.74	6.06	acyl-CoA thioesterase [Arthrobacter sp. RIT-PI-e]
gi742072575	7.91	1	1	215	22.5	6.95	6.06	hypothetical protein ANMWB30_04160 [Arthrobacter sp. MWB30]
gi786033428	2.51	3	1	439	48.6	5.53	6.06	alpha/beta hydrolase [Arthrobacter chlorophenolicus]
gi916820283	5.65	1	1	301	31.5	4.77	6.05	flagellar biosynthesis protein flip [Arthrobacter sp. H20]
gi515767309	6.90	1	1	348	36.0	5.02	6.05	hypothetical protein [Arthrobacter sp. M2012083]
gi651494736	1.75	1	1	800	87.5	5.41	6.04	phosphoenolpyruvate synthase [Arthrobacter sp. H20]
gi542109329	1.97	2	1	913	101.8	5.20	6.04	preprotein translocase subunit SecA [Arthrobacter sp. AK-YN10]
gi654814807	8.73	1	1	126	13.7	9.89	6.03	hypothetical protein [Arthrobacter sp. MA-N2]
gi116609390	4.47	1	1	380	41.1	7.27	6.03	DNA polymerase III, delta prime subunit [Arthrobacter sp. FB24]
gi219861025	2.59	5	1	887	97.2	5.20	6.03	conserved hypothetical protein [Arthrobacter chlorophenolicus A6]
gi759747025	1.82	2	1	878	94.7	7.33	6.02	LuxR family transcriptional regulator [Arthrobacter sp. 31Y]
gi542109239	15.45	8	1	110	12.1	4.88	6.01	hypothetical protein M707_07265 [Arthrobacter sp. AK-YN10]

gi518312200	6.00	1	1	200	22.3	5.07	6.01	MULTISPECIES: transcriptional regulator [Arthrobacter]
gi927293966	2.79	1	1	430	45.0	8.13	6.00	ABC transporter substrate-binding protein [Arthrobacter sp. ERGS1:01]
gi767258496	2.66	1	1	376	40.4	8.73	6.00	fusaric acid resistance protein [Arthrobacter sp. IHBB 11108]
gi910283668	6.19	1	1	291	30.4	6.40	6.00	hypothetical protein [Arthrobacter sp. A3]
gi476402859	2.19	3	1	684	73.0	5.17	5.99	5-oxoprolinase [Arthrobacter crystallopoietes BAB-32]
gi915933253	6.67	3	1	345	35.0	10.74	5.99	iron ABC transporter permease [Arthrobacter globiformis]
gi930826142	4.68	2	1	299	32.3	5.54	5.99	UTP--glucose-1-phosphate uridylyltransferase [Arthrobacter arilaitensis]
gi917572261	4.82	2	1	394	43.2	6.73	5.99	phosphodiesterase [Arthrobacter sp. PAO19]
gi737810320	2.93	1	1	443	46.2	5.02	5.99	glutamyl-tRNA reductase [Arthrobacter sp. 35/47]
gi910744511	4.21	1	1	214	23.2	5.96	5.98	inositol 2-dehydrogenase 1 [Arthrobacter sp. Hiyo8]
gi651497907	1.58	1	1	888	94.8	5.47	5.98	hypothetical protein [Arthrobacter sp. 35W]
gi403231949	2.36	1	1	593	64.0	5.17	5.97	protein CalT6 (plasmid) [Arthrobacter sp. Rue61a]
gi517601005	1.36	1	1	954	98.3	8.76	5.97	hypothetical protein [Arthrobacter sp. 162MFSha1.1]
gi651429224	2.87	2	1	348	37.0	4.96	5.96	hypothetical protein [Arthrobacter sanguinis]
gi674646043	1.23	1	1	1947	195.7	10.08	5.96	hypothetical protein BN1051_02242 [Arthrobacter sp. 11W110_air]
gi930826704	5.67	2	1	388	42.7	8.84	5.95	DNA polymerase IV [Arthrobacter arilaitensis]
gi742757466	5.83	2	1	412	44.9	7.62	5.95	cyclopropane-fatty-acyl-phospholipid synthase [Arthrobacter phenanthrenivorans]
gi323468304	2.35	4	1	723	75.4	5.12	5.95	translation elongation factor 2 (EF-2/EF-G) [Arthrobacter phenanthrenivorans Sphe3]
gi116610275	4.50	2	1	222	24.0	5.63	5.95	lipoate-protein ligase B [Arthrobacter sp. FB24]
gi542106407	12.50	2	1	80	9.3	6.04	5.94	hypothetical protein M707_21935 [Arthrobacter sp. AK-YN10]
gi759734840	3.91	2	1	486	51.9	6.00	5.93	ATPase AAA [Arthrobacter sp. L77]
gi937259419	3.08	1	1	292	30.9	4.53	5.93	hypothetical protein AO716_05280 [Arthrobacter sp. Edens01]
gi651480202	6.54	1	1	382	41.7	5.25	5.93	pyruvate dehydrogenase [Arthrobacter sp. Br18]
gi917021979	9.52	2	1	189	20.1	5.12	5.93	hypothetical protein [Arthrobacter sp. UNC362MFTsu5.1]
gi751834814	2.20	1	1	635	68.2	6.90	5.93	hypothetical protein RSOLAG1IB_07959 [Rhizoctonia solani AG-1 IB]
gi116610273	4.72	1	1	254	27.8	11.08	5.92	integral membrane protein [Arthrobacter sp. FB24]
gi545107386	4.32	2	1	301	34.0	9.72	5.92	hypothetical protein [Arthrobacter sp. AK-YN10]
gi674645126	1.82	1	1	988	103.9	8.19	5.91	tRNA3(Ser)-specific nuclease WapA precursor [Arthrobacter sp. 11W110_air]
gi737793660	4.48	1	1	402	43.9	7.21	5.91	restriction endonuclease [Arthrobacter nicotinovorans]
gi654815370	2.42	1	1	455	48.3	7.55	5.91	serine--pyruvate aminotransferase [Arthrobacter sp. PAO19]
gi219860471	2.48	1	1	404	44.1	10.35	5.91	monooxygenase FAD-binding [Arthrobacter chlorophenolicus A6]
gi918267558	12.20	1	1	164	17.8	7.05	5.90	organic hydroperoxide resistance transcriptional regulator [Arthrobacter sp. Hiyo1]
gi323469589	10.98	1	1	173	19.4	9.83	5.89	uncharacterized conserved protein [Arthrobacter phenanthrenivorans Sphe3]
gi359304378	2.32	2	1	561	60.7	5.47	5.89	putative peptide ABC transporter peptide-binding protein [Arthrobacter globiformis NBRC 12[illegible]
gi307746454	3.59	1	1	306	32.4	5.21	5.89	conserved hypothetical protein [Arthrobacter arilaitensis Re117]
gi742754729	9.16	3	1	251	25.9	5.91	5.89	short-chain dehydrogenase [Arthrobacter phenanthrenivorans]
gi916869743	10.14	1	1	148	15.5	10.32	5.89	hypothetical protein [Arthrobacter sp. Br18]
gi403311678	10.70	2	2	215	22.8	7.47	5.88	putative MobA-like protein (plasmid) [Arthrobacter sp. Rue61a]
gi749401408	1.08	1	1	1201	124.2	5.29	5.88	fibronectin, partial [Arthrobacter sp. AK-YN10]
gi759734215	3.64	1	1	385	39.4	6.76	5.87	gamma-glutamyl kinase [Arthrobacter sp. L77]
gi542106827	1.81	1	1	553	58.2	5.19	5.87	phosphoglucomutase [Arthrobacter sp. AK-YN10]
gi517593533	4.31	5	1	418	44.8	5.72	5.87	hypothetical protein [Arthrobacter sp. 135MFCol5.1]
gi651429337	3.16	1	1	632	66.1	5.48	5.87	2-succinyl-5-enolpyruvyl-6-hydroxy-3-cyclohexene-1-carboxylate synthase [Arthrobacter san[illegible]
gi648572686	3.96	5	1	404	42.7	6.57	5.87	dephospho-CoA kinase [Arthrobacter sp. 135MFCol5.1]
gi737778385	2.31	4	1	607	65.8	5.25	5.85	peptide ABC transporter ATPase [Arthrobacter sanguinis]
gi545109763	4.34	2	1	392	39.8	11.62	5.85	MFS transporter [Arthrobacter sp. AK-YN10]
gi307743478	6.71	1	1	149	15.7	8.22	5.85	AsnC/Lrp-family transcriptional regulator [Arthrobacter arilaitensis Re117]
gi723607702	1.71	1	1	1111	120.2	5.31	5.85	hypothetical protein ART_1479 [Arthrobacter sp. PAMC25486]
gi517607940	4.38	5	1	502	53.1	4.84	5.84	hypothetical protein [Arthrobacter sp. 161MFSha2.1]
gi930826447	3.48	2	2	574	62.3	5.80	5.84	pyruvate dehydrogenase [Arthrobacter arilaitensis]
gi916259928	20.00	2	1	120	12.6	6.14	5.84	hypothetical protein [Arthrobacter sp. TB 23]
gi737813422	3.62	3	1	359	39.2	5.47	5.84	carboxylate--amine ligase [Arthrobacter sp. H14]
gi443480347	3.38	1	1	414	42.2	6.02	5.83	peptidase S8/S53 subtilisin kexin sedolisin [Arthrobacter nitrophenolicus]
gi723609944	3.31	1	1	363	36.4	5.19	5.83	hypothetical protein ART_3721 [Arthrobacter sp. PAMC25486]
gi551254183	2.82	2	1	425	47.1	5.15	5.83	serine--tRNA ligase [Arthrobacter sp. PAO19]

gi651441353	2.83	2	1	848	91.1	7.47	5.82	AMP-dependent synthetase [Arthrobacter sp. 9MFCol3.1]
gi654823329	16.11	2	1	149	16.8	10.29	5.82	AraC family transcriptional regulator [Arthrobacter sp. I3]
gi916869608	5.36	1	1	317	34.2	9.80	5.81	pseudouridine synthase [Arthrobacter sp. Br18]
gi116612111	2.70	1	1	556	60.1	5.31	5.81	putative signal transduction histidine kinase [Arthrobacter sp. FB24]
gi757624374	4.19	9	1	334	36.3	5.26	5.81	flavodoxin [Arthrobacter sp. SPG23]
gi927293150	3.40	2	1	412	43.7	5.55	5.81	hypothetical protein AL755_02905 (plasmid) [Arthrobacter sp. ERGS1:01]
gi927293634	3.05	1	1	426	45.4	4.68	5.80	enolase [Arthrobacter sp. ERGS1:01]
gi917013476	4.13	2	1	460	49.3	6.27	5.80	AAA family ATPase [Arthrobacter sanguinis]
gi908699570	3.65	5	1	658	68.2	5.33	5.80	PTS mannose transporter subunit IIA [Arthrobacter sp. RIT-PI-e]
gi910250417	2.65	1	1	453	51.4	6.44	5.80	hypothetical protein [Arthrobacter siccitolerans]
gi476399190	9.22	1	1	217	22.5	10.32	5.79	MFS superfamily transporter, partial [Arthrobacter crystallopoietes BAB-32]
gi767258004	4.65	1	1	473	49.5	5.12	5.79	branched-chain alpha-keto acid dehydrogenase subunit E2 [Arthrobacter sp. IHBB 11108]
gi652422699	3.64	1	1	632	67.0	5.47	5.79	acetolactate synthase 1 catalytic subunit [Arthrobacter castelli]
gi823666664	2.45	2	1	611	65.7	6.57	5.79	ABC transporter [Arthrobacter sp. YC-RL1]
gi927295073	1.64	1	1	1034	106.5	4.97	5.79	hypothetical protein AL755_15435 [Arthrobacter sp. ERGS1:01]
gi737765091	3.34	1	1	628	68.2	5.01	5.78	hypothetical protein [Arthrobacter sp. 161MFSha2.1]
gi651438938	2.00	2	1	551	59.2	5.05	5.78	urocanate hydratase [Arthrobacter sp. H14]
gi767256771	5.84	2	1	291	31.2	8.72	5.78	hypothetical protein UM93_02335 [Arthrobacter sp. IHBB 11108]
gi917760241	1.83	1	1	1310	135.8	11.63	5.77	hypothetical protein [Arthrobacter sp. L77]
gi640199857	6.85	4	1	321	35.0	9.42	5.77	ABC transporter permease [Arthrobacter sp. 31Y]
gi635352876	4.76	1	1	273	29.7	10.05	5.77	membrane insertase, YidC/Oxa1 family domain protein [Arthrobacter siccitolerans]
gi517603095	5.84	1	1	257	30.2	6.81	5.76	hypothetical protein [Arthrobacter sp. 131MFCol6.1]
gi443482046	4.81	2	1	374	39.6	6.19	5.75	glutamate dehydrogenase [Arthrobacter nitrophenolicus]
gi930825623	5.28	2	1	246	26.0	8.43	5.75	hypothetical protein AOZ07_04480 [Arthrobacter arilaitensis]
gi651500415	0.79	2	1	881	90.4	7.88	5.75	helicase [Arthrobacter sp. 35W]
gi518312131	2.30	1	1	868	93.9	6.32	5.75	hypothetical protein [Arthrobacter sp. TB 23]
gi917759806	3.12	3	1	609	68.2	9.33	5.74	ABC transporter [Arthrobacter sp. L77]
gi489894054	2.33	2	1	472	51.7	5.76	5.74	FMNH2-dependent monooxygenase [Arthrobacter globiformis]
gi928488787	1.08	1	1	1200	127.5	5.16	5.74	chromosome segregation protein SMC [Arthrobacter alpinus]
gi927294653	3.16	1	1	601	63.2	8.95	5.73	preprotein translocase subunit SecD [Arthrobacter sp. ERGS1:01]
gi517591891	3.76	2	1	346	36.7	8.38	5.73	hypothetical protein [Arthrobacter sp. 135MFCol5.1]
gi910248959	7.17	1	1	307	34.3	6.73	5.73	AraC family transcriptional regulator [Arthrobacter siccitolerans]
gi116610539	1.36	1	1	883	94.2	5.77	5.72	osmosensitive K+ channel signal transduction histidine kinase [Arthrobacter sp. FB24]
gi928485938	24.00	1	1	75	8.0	9.85	5.72	hypothetical protein AOC05_01070 [Arthrobacter alpinus]
gi162953675	2.76	2	1	362	39.0	9.13	5.72	conserved hypothetical protein [Renibacterium salmoninarum ATCC 33209]
gi939051213	3.78	3	1	185	19.7	11.33	5.72	hypothetical protein [Arthrobacter sp. JCM 19049]
gi737777559	1.81	1	1	830	88.1	5.69	5.71	DNA topoisomerase IV subunit A [Arthrobacter sanguinis]
gi742759349	3.54	2	1	536	57.0	9.41	5.71	FAD-dependent oxidoreductase [Arthrobacter phenanthrenivorans]
gi674646613	1.44	4	1	486	49.9	5.01	5.70	Allantoinase [Arthrobacter sp. 11W110_air]
gi908699494	9.05	2	1	232	25.6	6.67	5.70	GntR family transcriptional regulator [Arthrobacter sp. RIT-PI-e]
gi517590015	12.12	1	1	165	17.8	11.53	5.70	hypothetical protein [Arthrobacter sp. 135MFCol5.1]
gi517599714	2.03	2	1	639	67.4	7.62	5.69	C4-dicarboxylate ABC transporter permease [Arthrobacter sp. 162MFSha1.1]
gi651500938	1.01	1	1	691	75.0	6.77	5.69	acyl-CoA dehydrogenase [Arthrobacter sp. 35W]
gi654824450	8.03	3	1	249	26.1	9.54	5.69	ABC transporter ATPase [Arthrobacter sp. I3]
gi1906823	10.85	3	1	212	22.7	10.96	5.69	hypothetical protein [Pimelobacter simplex]
gi162953186	16.92	2	1	130	13.7	8.68	5.69	transcriptional regulator, GntR family [Renibacterium salmoninarum ATCC 33209]
gi651437429	10.24	1	1	127	14.4	11.31	5.69	tRNA synthetase RNA-binding protein [Arthrobacter sp. H14]
gi651435294	2.92	1	1	411	42.7	6.20	5.68	threonine dehydratase [Arthrobacter sp. H41]
gi910744498	7.38	1	1	298	32.3	9.63	5.68	ribose import ATP-binding protein RbsA [Arthrobacter sp. Hiyo8]
gi470216243	1.84	1	1	653	72.2	7.58	5.68	excinuclease ABC subunit C [Arthrobacter gangotriensis Lz1y]
gi517598656	9.13	1	1	241	26.5	6.42	5.66	hypothetical protein [Arthrobacter sp. 162MFSha1.1]
gi542107235	4.12	3	1	582	63.4	9.07	5.65	hypothetical protein M707_18145 [Arthrobacter sp. AK-YN10]
gi307744186	3.85	2	1	519	53.8	9.29	5.65	conserved membrane protein [Arthrobacter arilaitensis Re117]
gi654823265	8.24	6	1	279	28.4	5.15	5.65	hypothetical protein [Arthrobacter sp. I3]
gi910740677	23.17	1	1	82	9.3	10.35	5.64	probable transposase for insertion sequence element ISRM3-like [Arthrobacter sp. Hiyo4]

gi910252478	6.67	1	1	240	25.6	6.16	5.64	short-chain dehydrogenase [Arthrobacter siccitolerans]
gi654817405	7.19	1	1	278	29.6	5.16	5.62	alpha/beta hydrolase [Arthrobacter sp. UNC362MFTsu5.1]
gi323468471	21.62	5	1	111	11.8	6.80	5.62	cupin domain-containing protein [Arthrobacter phenanthrenivorans Sphe3]
gi307745403	2.42	2	1	538	58.7	8.47	5.62	putative ATP-dependent RNA helicase [Arthrobacter arilaitensis Re117]
gi674644368	6.09	1	1	312	31.2	9.95	5.62	hypothetical protein BN1051_00520 [Arthrobacter sp. 11W110_air]
gi916816420	2.39	1	1	376	41.5	11.15	5.62	hypothetical protein [Arthrobacter sp. MA-N2]
gi737789872	1.83	1	1	766	83.6	5.33	5.61	ATPase AAA [Arthrobacter albus]
gi652424263	7.14	1	1	224	25.4	8.60	5.61	hypothetical protein [Arthrobacter castelli]
gi116612595	3.33	1	1	661	71.6	6.16	5.61	acyl-CoA dehydrogenase domain protein [Arthrobacter sp. FB24]
gi765008612	2.86	1	1	769	79.2	6.71	5.60	carbonate dehydratase [Arthrobacter sp. A3]
gi219858740	1.24	2	1	1051	118.0	6.01	5.59	type III restriction protein res subunit [Arthrobacter chlorophenolicus A6]
gi759731480	4.56	1	1	241	26.3	6.55	5.59	hypothetical protein [Arthrobacter sp. L77]
gi470216926	2.81	1	1	392	41.4	5.99	5.59	penicillin binding protein [Arthrobacter gangotriensis Lz1y]
gi937258046	6.95	1	1	187	20.8	7.52	5.59	hypothetical protein AO716_07970 [Arthrobacter sp. Edens01]
gi651472483	2.68	1	1	411	45.9	4.92	5.58	hypothetical protein [Arthrobacter nicotinovorans]
gi517605320	4.67	1	1	300	32.2	5.29	5.58	aminoglycoside phosphotransferase [Arthrobacter sp. 131MFCol6.1]
gi219859718	2.52	1	1	516	54.6	5.33	5.58	band 7 protein [Arthrobacter chlorophenolicus A6]
gi908697791	2.55	1	1	666	72.2	8.70	5.58	hypothetical protein [Arthrobacter sp. RIT-PI-e]
gi654827225	3.80	1	1	500	52.6	5.41	5.58	dehydrogenase [Arthrobacter sp. H5]
gi928486236	1.15	1	1	1478	158.1	6.28	5.57	cell division protein FtsK [Arthrobacter alpinus]
gi359303444	2.36	1	1	635	67.5	9.42	5.56	putative ABC transporter permease/ATP-binding protein [Arthrobacter globiformis NBRC 121
gi723608487	5.58	1	1	430	44.5	6.27	5.56	uroporphyrin-III C-methyltransferase [Arthrobacter sp. PAMC25486]
gi914713901	5.63	1	1	444	48.1	5.10	5.56	ribonuclease D [Arthrobacter sp. ZBG10]
gi636843666	3.91	1	1	486	53.6	5.30	5.56	ATPase AAA, partial [Arthrobacter sp. TB 26]
gi651506215	4.61	1	1	347	37.9	6.58	5.55	hypothetical protein [Arthrobacter sp. 35W]
gi651439404	1.39	1	1	1006	111.2	6.54	5.55	glutamine-synthetase [Arthrobacter sp. H14]
gi219860458	1.74	1	1	689	73.3	5.92	5.55	protein of unknown function DUF839 [Arthrobacter chlorophenolicus A6]
gi470216075	4.18	1	1	335	37.2	5.05	5.54	magnesium and cobalt transport protein CorA [Arthrobacter gangotriensis Lz1y]
gi930828077	4.69	2	1	426	44.0	9.01	5.54	C4-dicarboxylate transporter [Arthrobacter arilaitensis]
gi652422609	11.41	1	1	184	19.8	4.67	5.53	ATP synthase F0F1 subunit B [Arthrobacter castelli]
gi542106680	1.29	1	1	1161	129.5	5.30	5.53	ATP-binding protein [Arthrobacter sp. AK-YN10]
gi910250818	2.53	1	1	671	74.3	5.63	5.52	hypothetical protein [Arthrobacter siccitolerans]
gi648575162	1.91	2	1	577	59.6	5.59	5.51	L-aspartate oxidase [Arthrobacter sp. 161MFSha2.1]
gi916834607	1.50	1	1	1133	125.7	5.62	5.51	hypothetical protein [Arthrobacter sp. H14]
gi916870122	17.61	2	2	318	33.9	5.21	5.50	hypothetical protein [Arthrobacter sp. Br18]
gi786028535	6.60	2	1	288	29.8	5.20	5.49	protein-(glutamine-N5) methyltransferase, release factor-specific [Arthrobacter chlorophenol
gi443480002	2.94	3	1	476	52.9	10.08	5.49	hypothetical protein G205_21149 [Arthrobacter nitrophenolicus]
gi517600184	2.78	1	1	396	42.8	5.88	5.49	dehydrogenase [Arthrobacter sp. 162MFSha1.1]
gi916869712	5.30	2	1	264	27.4	10.13	5.48	hypothetical protein [Arthrobacter sp. Br18]
gi654824711	5.45	5	1	257	27.4	5.99	5.48	DeoR family transcriptional regulator [Arthrobacter sp. I3]
gi759718925	5.82	1	1	275	28.0	7.24	5.47	hypothetical protein [Arthrobacter sp. FB24]
gi742072345	5.58	1	1	484	51.4	8.47	5.47	HTH-type transcriptional regulator YdfD [Arthrobacter sp. MWB30]
gi916872069	1.62	4	1	1297	142.0	6.34	5.47	AAA family ATPase [Arthrobacter sp. H5]
gi651454653	13.30	5	1	188	20.5	6.81	5.46	MarR family transcriptional regulator [Arthrobacter nicotinovorans]
gi742851216	5.69	2	1	246	25.0	4.31	5.46	hypothetical protein [Arthrobacter sp. W1]
gi753931349	9.09	6	1	121	13.1	8.51	5.46	single-stranded DNA-binding protein [Arthrobacter arilaitensis]
gi652422482	4.80	1	1	563	61.4	5.05	5.45	AMP-binding protein [Arthrobacter castelli]
gi737813075	4.94	1	1	263	27.8	8.03	5.45	hypothetical protein [Arthrobacter sp. H14]
gi119948794	3.73	1	1	482	51.8	5.02	5.44	mitomycin radical oxidase [Arthrobacter aurescens TC1]
gi651430627	1.75	1	1	570	59.8	5.05	5.44	dihydroxy-acid dehydratase [Arthrobacter sanguinis]
gi551256741	6.82	6	1	132	15.0	9.20	5.44	AraC family transcriptional regulator [Arthrobacter sp. PAO19]
gi323470115	6.96	4	1	316	33.9	8.48	5.44	ABC-type dipeptide/oligopeptide/nickel transport system, permease component [Arthrobacte
gi636845070	4.48	4	1	469	47.0	5.20	5.43	flagellar hook protein FlgK [Arthrobacter sp. TB 26]
gi823666014	4.62	1	1	455	49.6	6.83	5.40	glycerol-3-phosphate ABC transporter substrate-binding protein [Arthrobacter sp. YC-RL1]
gi910249775	4.21	1	1	309	33.4	8.60	5.40	type II secretion system protein F [Arthrobacter siccitolerans]

gi742755707	3.18	1	1	472	49.4	6.29	5.39	cysteine desulfurase [Arthrobacter phenanthrenivorans]
gi737808296	6.80	2	1	206	23.4	5.25	5.38	hypothetical protein [Arthrobacter sp. H5]
gi651472389	2.12	2	1	425	45.0	9.98	5.37	MFS transporter [Arthrobacter nicotinovorans]
gi910745641	2.74	3	1	329	35.9	9.28	5.37	UPF0699 transmembrane protein YdbT [Arthrobacter sp. Hiyo8]
gi930826101	3.00	1	1	567	59.0	5.59	5.37	dihydroxy-acid dehydratase [Arthrobacter arilaitensis]
gi914717103	2.04	2	1	588	61.9	5.34	5.36	ABC transporter [Arthrobacter sp. ZBG10]
gi937258270	2.95	2	1	611	68.2	5.48	5.36	hypothetical protein AO716_09285 [Arthrobacter sp. Edens01]
gi737787338	2.14	1	1	560	62.5	5.07	5.35	ABC transporter ATP-binding protein [Arthrobacter albus]
gi759714812	3.31	2	1	302	34.2	7.21	5.35	transposition protein TniB [Arthrobacter sp. AK-YN10]
gi927032029	4.20	8	1	381	39.0	6.76	5.34	cysteine desulfurase [Arthrobacter sp. LS16]
gi651450981	2.72	1	1	478	54.2	5.16	5.34	glycoside hydrolase [Arthrobacter nicotinovorans]
gi742853338	1.35	2	1	1187	131.4	5.55	5.32	DNA polymerase III subunit alpha [Arthrobacter sp. W1]
gi517605990	5.84	1	1	308	33.3	9.67	5.32	tyrosine recombinase XerC [Arthrobacter sp. 161MFSha2.1]
gi910694102	6.81	1	1	235	25.3	5.24	5.32	hypothetical protein AHiyo6_28660, partial [Arthrobacter sp. Hiyo6]
gi651486945	2.27	3	1	484	51.1	5.81	5.32	inosine-5-monophosphate dehydrogenase [Arthrobacter sp. Br18]
gi769944147	1.61	4	1	745	77.4	6.68	5.31	carbonate dehydratase [Arthrobacter sp. IHBB 11108]
gi521040827	5.26	2	1	228	25.7	5.31	5.31	hypothetical protein [Arthrobacter sp. M2012083]
gi723607119	2.78	1	1	431	47.1	4.88	5.31	hypothetical protein ART_0896 [Arthrobacter sp. PAMC25486]
gi910696539	8.96	1	1	134	14.7	9.52	5.31	oxidoreductase [Arthrobacter sp. Hiyo6]
gi737809913	5.29	6	1	170	19.2	11.25	5.31	hypothetical protein [Arthrobacter sp. 35/47]
gi648259714	3.02	1	1	298	31.9	8.87	5.31	DNA lyase [Arthrobacter sp. TB 23]
gi917441938	13.58	2	1	162	17.2	9.85	5.31	hypothetical protein [Arthrobacter albus]
gi323469500	13.45	1	1	119	12.6	7.15	5.30	hypothetical protein Asphe3_20300 [Arthrobacter phenanthrenivorans Sphe3]
gi116609729	5.65	2	1	283	30.7	5.41	5.30	Methyltransferase type 11 [Arthrobacter sp. FB24]
gi654812984	1.15	5	1	1047	117.0	5.88	5.29	helicase [Arthrobacter sp. MA-N2]
gi518314110	2.40	2	1	458	48.7	5.05	5.29	cysteine synthase [Arthrobacter sp. TB 23]
gi742854811	1.83	1	1	1092	117.3	4.93	5.29	carbamoyl phosphate synthase large subunit [Arthrobacter sp. W1]
gi518311829	7.94	2	1	214	22.5	5.16	5.28	MULTISPECIES: para-aminobenzoate synthase [Arthrobacter]
gi651506456	5.25	2	1	305	33.2	7.05	5.27	hypothetical protein [Arthrobacter sp. 35W]
gi910739986	5.34	3	1	262	30.0	11.63	5.26	ribonuclease E/G-like protein [Arthrobacter sp. Hiyo4]
gi651462770	2.23	1	1	314	35.6	9.91	5.26	hypothetical protein [Arthrobacter sp. 35/47]
gi551256284	3.13	1	1	702	77.3	7.49	5.25	DNA topoisomerase IV subunit B [Arthrobacter sp. PAO19]
gi639129021	2.17	2	1	877	95.6	5.02	5.25	DNA polymerase I [Arthrobacter sp. CAL618]
gi651447759	5.18	1	1	309	35.5	10.05	5.25	hypothetical protein [Arthrobacter nicotinovorans]
gi928488104	4.87	6	1	472	48.8	5.27	5.24	hypothetical protein AOC05_15810 [Arthrobacter alpinus]
gi917442177	4.51	3	1	377	39.3	4.75	5.24	hypothetical protein [Arthrobacter albus]
gi742854494	4.56	3	1	307	33.5	6.55	5.24	hypothetical protein [Arthrobacter sp. W1]
gi662774342	4.28	4	1	304	31.5	5.00	5.24	MULTISPECIES: prephenate dehydratase [Mycobacterium]
gi916820398	2.27	10	1	750	80.3	7.36	5.24	hypothetical protein [Arthrobacter sp. H20]
gi476401876	1.42	1	1	564	58.1	6.10	5.24	metal-dependent hydrolase, TIM barrel fold protein [Arthrobacter crystallopoietes BAB-32]
gi823668656	6.62	1	1	151	16.6	8.84	5.23	hypothetical protein AA310_00650 [Arthrobacter sp. YC-RL1]
gi651441621	2.57	7	1	428	44.3	7.06	5.23	siroheme synthase CysG [Arthrobacter sp. 9MFCol3.1]
gi636843787	5.01	2	1	359	38.7	6.54	5.23	sialic acid transporter [Arthrobacter sp. TB 26]
gi651431156	1.18	2	1	676	70.6	4.81	5.22	hypothetical protein [Arthrobacter sanguinis]
gi651464991	3.01	1	1	599	65.0	5.35	5.22	proline--tRNA ligase [Arthrobacter sp. 35/47]
gi470217234	1.80	5	1	1169	129.1	5.43	5.22	DNA polymerase III subunit alpha [Arthrobacter gangotriensis Lz1y]
gi823666886	3.35	5	1	477	52.4	4.79	5.22	Putrescine oxidase [Arthrobacter sp. YC-RL1]
gi651494129	2.54	4	1	315	32.3	5.50	5.21	nicotinate-nucleotide pyrophosphorylase [Arthrobacter sp. H20]
gi757622445	5.20	2	1	346	37.7	5.35	5.21	sialic acid transporter [Arthrobacter sp. SPG23]
gi767259109	1.94	1	1	310	33.3	7.01	5.21	ATPase [Arthrobacter sp. IHBB 11108]
gi910251865	1.52	2	1	660	71.0	5.03	5.21	levanase [Arthrobacter siccitolerans]
gi674645861	5.83	1	1	326	34.6	6.65	5.21	Methionine import ATP-binding protein MetN [Arthrobacter sp. 11W110_air]
gi517591231	3.48	1	1	488	51.4	6.13	5.21	ATPase AAA [Arthrobacter sp. 135MFCol5.1]
gi908698536	3.92	1	1	434	46.2	5.05	5.21	tryptophan synthase subunit beta [Arthrobacter sp. RIT-PI-e]
gi916781593	3.41	6	1	323	35.5	9.96	5.20	hypothetical protein [Arthrobacter sp. 35W]

gi930826116	5.63	1	1	391	43.8	7.27	5.20	hypothetical protein AOZ07_07335 [Arthrobacter arilaitensis]
gi219858569	9.52	2	1	231	23.1	4.73	5.20	molybdopterin-guanine dinucleotide biosynthesis protein [Arthrobacter chlorophenolicus A6]
gi147829023	3.37	1	1	475	48.8	9.06	5.20	putative membrane protein (plasmid) [Clavibacter michiganensis subsp. michiganensis NCPP
gi674644252	3.88	3	1	387	42.2	9.61	5.20	hypothetical protein BN1051_00403 [Arthrobacter sp. 11W110_air]
gi116609564	1.81	2	1	1215	131.7	5.59	5.19	conserved hypothetical protein [Arthrobacter sp. FB24]
gi651431806	1.84	3	1	871	97.9	6.42	5.19	hypothetical protein [Arthrobacter sanguinis]
gi737776999	3.16	4	1	697	76.0	5.00	5.19	cell division protein FtsH [Arthrobacter sanguinis]
gi517598211	1.63	1	1	490	51.8	4.93	5.19	diaminopimelate decarboxylase [Arthrobacter sp. 162MFSha1.1]
gi323467642	2.18	4	1	458	49.4	5.05	5.18	NAD-dependent aldehyde dehydrogenase [Arthrobacter phenanthrenivorans Sphe3]
gi723607488	1.90	1	1	633	70.3	6.30	5.17	hypothetical protein ART_1265 [Arthrobacter sp. PAMC25486]
gi910738995	2.18	1	1	412	43.2	4.92	5.17	DNA repair protein RecN [Arthrobacter sp. Hiyo4]
gi517608980	23.00	2	1	100	10.5	10.01	5.17	hypothetical protein [Arthrobacter sp. 161MFSha2.1]
gi651443075	1.80	1	1	555	58.1	10.10	5.17	ABC transporter [Arthrobacter sp. 9MFCol3.1]
gi918266944	13.11	3	1	122	12.9	9.58	5.16	hypothetical protein AHiyo1_34090 [Arthrobacter sp. Hiyo1]
gi542110736	4.67	1	1	321	34.8	6.96	5.16	LysR family transcriptional regulator [Arthrobacter sp. AK-YN10]
gi651463030	2.32	8	1	561	61.3	7.14	5.16	ATP-dependent DNA helicase PcrA [Arthrobacter sp. 35/47]
gi476401154	3.07	1	1	358	37.6	4.73	5.15	hypothetical protein D477_010104 [Arthrobacter crystallopoietes BAB-32]
gi651486201	11.17	2	1	197	21.7	9.80	5.15	hypothetical protein [Arthrobacter sp. Br18]
gi916834558	9.09	1	1	121	13.3	8.46	5.14	hypothetical protein [Arthrobacter sp. H14]
gi654819248	12.84	2	2	296	30.7	5.34	5.14	short-chain dehydrogenase [Arthrobacter sp. UNC362MFTsu5.1]
gi742757520	2.55	2	1	432	47.3	6.58	5.13	cyclopropane-fatty-acyl-phospholipid synthase [Arthrobacter phenanthrenivorans]
gi928488753	1.01	1	1	1090	121.0	5.10	5.13	isoleucine--tRNA ligase [Arthrobacter alpinus]
gi742755018	5.97	3	1	201	21.4	4.68	5.13	alanine racemase [Arthrobacter phenanthrenivorans]
gi914715157	5.46	2	1	238	24.8	5.54	5.13	GCN5 family acetyltransferase [Arthrobacter sp. ZBG10]
gi757625222	7.94	3	1	214	23.7	7.34	5.12	GCN5 family acetyltransferase [Arthrobacter sp. SPG23]
gi654827976	5.63	4	1	231	25.6	5.86	5.12	hypothetical protein [Arthrobacter sp. H5]
gi476400938	2.30	1	1	479	49.5	5.24	5.11	L-aspartate oxidase, partial [Arthrobacter crystallopoietes BAB-32]
gi937258776	2.53	3	2	948	104.4	7.09	5.11	RNA helicase [Arthrobacter sp. Edens01]
gi674644635	3.05	6	1	524	50.8	5.00	5.11	hypothetical protein BN1051_00791 [Arthrobacter sp. 11W110_air]
gi910743105	18.87	2	1	106	11.1	4.93	5.11	hypothetical protein AHiyo8_08070 [Arthrobacter sp. Hiyo8]
gi576470298	6.02	4	1	216	22.7	5.36	5.11	prephenate dehydratase family protein [Mycobacterium abscessus 1948]
gi767257336	12.00	10	1	175	19.6	4.42	5.11	ribosome maturation factor RimM [Arthrobacter sp. IHBB 11108]
gi910742221	3.85	1	1	286	30.4	8.27	5.10	shikimate dehydrogenase [Arthrobacter sp. Hiyo4]
gi908698302	5.14	3	1	311	31.3	5.25	5.10	NADPH:quinone reductase [Arthrobacter sp. RIT-PI-e]
gi651466083	8.22	2	1	146	15.4	4.88	5.10	acyl dehydratase [Arthrobacter sp. 35/47]
gi910746378	7.73	1	1	181	19.3	5.16	5.10	fumarylacetoacetate hydrolase domain-containing protein 2 [Arthrobacter sp. Hiyo8]
gi651437648	3.72	2	1	430	46.8	8.97	5.10	DNA polymerase [Arthrobacter sp. H14]
gi651461001	2.52	1	1	795	83.9	5.94	5.09	hypothetical protein [Arthrobacter sp. 35/47]
gi470221086	10.26	1	1	78	8.9	9.32	5.09	hypothetical protein ADIAG_01038 [Arthrobacter gangotriensis Lz1y]
gi917530436	7.95	2	1	151	16.0	4.78	5.09	FHA domain-containing protein [Arthrobacter sp. PAMC25486]
gi359305538	6.92	2	1	159	17.3	9.35	5.09	hypothetical protein ARGLB_064_00880 [Arthrobacter globiformis NBRC 12137]
gi640200244	5.45	4	1	312	32.7	5.02	5.09	phosphoglycerate dehydrogenase [Arthrobacter sp. 31Y]
gi753931683	3.14	3	1	477	50.7	4.73	5.08	phosphomannomutase [Arthrobacter arilaitensis]
gi823668058	1.31	1	1	994	110.1	5.29	5.07	alpha-mannosidase, partial [Arthrobacter sp. YC-RL1]
gi823668025	6.41	1	1	312	31.7	6.43	5.07	hypothetical protein AA310_05315 [Arthrobacter sp. YC-RL1]
gi652425503	9.35	6	1	214	22.9	5.02	5.07	hypothetical protein [Arthrobacter castelli]
gi651506641	3.40	3	1	235	25.6	6.20	5.07	GntR family transcriptional regulator [Arthrobacter sp. 35W]
gi737796447	3.90	1	1	462	48.3	8.65	5.07	Mg chelatase-like protein, partial [Arthrobacter sp. H20]
gi640202480	10.46	1	1	153	16.8	7.02	5.06	hypothetical protein [Arthrobacter sp. 31Y]
gi737814712	17.86	2	1	84	9.4	4.84	5.05	hypothetical protein, partial [Arthrobacter sp. H14]
gi737814146	22.81	3	1	57	5.8	5.19	5.05	hypothetical protein [Arthrobacter sp. H14]
gi910749080	4.30	5	1	302	33.1	8.34	5.05	probable oxidoreductase OrdL (plasmid) [Arthrobacter sp. Hiyo8]
gi651466358	2.55	3	1	825	88.8	6.07	5.05	DNA topoisomerase IV subunit A [Arthrobacter sp. 35/47]
gi651429854	1.92	2	1	312	31.9	4.59	5.04	zinc-binding dehydrogenase [Arthrobacter sanguinis]
gi760112710	5.40	3	1	278	30.7	5.11	5.03	DNA-directed RNA polymerase sigma-70 factor [Arthrobacter chlorophenolicus]

gi786025818	4.66	4	1	279	30.8	9.91	5.03	hypothetical protein [Arthrobacter chlorophenolicus]
gi219859887	5.04	15	1	258	28.3	4.73	5.03	5-carboxymethyl-2-hydroxymuconate Delta-isomerase [Arthrobacter chlorophenolicus A6]
gi737779809	4.49	6	1	245	26.0	6.80	5.03	aldolase [Arthrobacter sp. CAL618]
gi914715201	8.98	1	1	167	17.5	8.21	5.02	HxlR family transcriptional regulator [Arthrobacter sp. ZBG10]
gi651434146	11.90	2	1	126	13.0	4.54	5.01	50S ribosomal protein L7/L12 [Arthrobacter sp. H41]
gi919218792	22.00	3	1	100	11.3	4.87	5.01	hypothetical protein [Arthrobacter sp. YC-RL1]
gi517602891	4.31	3	1	348	37.0	6.60	5.01	hypothetical protein [Arthrobacter sp. 131MFCol6.1]
gi742757173	5.20	2	1	404	44.1	8.57	5.01	mechanosensitive ion channel protein MscS [Arthrobacter phenanthrenivorans]
gi767257207	2.42	2	1	413	45.2	5.58	5.01	RNA polymerase subunit sigma-24 [Arthrobacter sp. IHBB 11108]
gi765006852	4.23	2	1	355	36.7	5.19	5.01	dipeptide epimerase [Arthrobacter sp. A3]
gi651445222	3.76	3	1	266	28.4	6.70	5.00	IclR family transcriptional regulator [Arthrobacter nicotinovorans]
gi654827483	6.10	3	1	295	33.0	9.52	5.00	XRE family transcriptional regulator [Arthrobacter sp. H5]
gi359307060	5.53	4	1	235	25.2	9.67	4.98	hypothetical protein ARGLB_027_00860 [Arthrobacter globiformis NBRC 12137]
gi759765729	3.29	2	1	426	44.8	7.36	4.97	hypothetical protein [Arthrobacter gangotriensis]
gi937262282	11.64	1	1	146	15.9	5.22	4.97	preprotein translocase subunit TatA [Arthrobacter sp. Edens01]
gi359304985	2.99	2	1	334	35.6	5.16	4.97	dihydroxyacetone kinase substrate-binding subunit DhaK [Arthrobacter globiformis NBRC 12[illegible]
gi765011169	0.46	1	1	3477	371.9	5.38	4.97	non-ribosomal peptide synthetase [Arthrobacter sp. A3]
gi914713332	2.85	1	1	491	51.3	5.21	4.97	branched-chain alpha-keto acid dehydrogenase subunit E2 [Arthrobacter sp. ZBG10]
gi786030005	11.40	5	1	114	12.6	6.40	4.97	hypothetical protein [Arthrobacter chlorophenolicus]
gi551254578	4.62	2	1	433	46.0	5.78	4.96	CAIB/BAIF family acyl-CoA thioesterase [Arthrobacter sp. PAO19]
gi723608396	7.43	4	1	377	40.2	4.91	4.96	aspartate-semialdehyde dehydrogenase [Arthrobacter sp. PAMC25486]
gi910696228	10.17	3	1	118	12.3	5.48	4.96	hypothetical protein AHiyo6_13490 [Arthrobacter sp. Hiyo6]
gi517591853	5.17	4	1	290	30.8	5.33	4.95	hypothetical protein [Arthrobacter sp. 135MFCol5.1]
gi908698087	8.31	6	1	313	31.4	4.64	4.95	ribokinase [Arthrobacter sp. RIT-PI-e]
gi654814276	6.30	4	1	254	27.3	5.33	4.95	hypothetical protein [Arthrobacter sp. MA-N2]
gi116610225	4.26	2	1	399	40.8	9.64	4.95	Peptidoglycan-binding LysM [Arthrobacter sp. FB24]
gi542107553	5.35	4	1	318	32.7	4.97	4.95	hypothetical protein M707_16600 [Arthrobacter sp. AK-YN10]
gi937259001	3.72	2	1	403	43.2	9.67	4.95	transposase [Arthrobacter sp. Edens01]
gi640199853	16.54	1	1	133	14.8	9.25	4.94	DNA-binding protein [Arthrobacter sp. 31Y]
gi651453122	13.59	3	1	103	11.1	9.74	4.94	hypothetical protein [Arthrobacter nicotinovorans]
gi910744127	6.03	3	1	199	22.0	10.67	4.93	carboxylate-amine ligase RHA1_ro04240 [Arthrobacter sp. Hiyo8]
gi116611335	5.47	1	1	256	28.6	9.03	4.93	glycosyl transferase, family 2 [Arthrobacter sp. FB24]
gi765012357	4.24	1	1	472	50.0	10.26	4.92	MFS transporter [Arthrobacter sp. A3]
gi918423049	3.82	2	1	314	34.6	9.95	4.92	hypothetical protein [Arthrobacter sp. AK-YN10]
gi403230605	7.69	3	1	364	38.8	4.91	4.92	aspartate-semialdehyde dehydrogenase Asd [Arthrobacter sp. Rue61a]
gi654817103	5.83	1	1	326	35.1	6.90	4.92	ribose-phosphate pyrophosphokinase [Arthrobacter sp. UNC362MFTsu5.1]
gi323468729	2.02	4	1	840	87.5	8.37	4.92	transglutaminase-like enzyme, predicted cysteine protease [Arthrobacter phenanthrenivorans
gi542107378	4.26	1	1	305	31.2	4.83	4.92	carbohydrate kinase [Arthrobacter sp. AK-YN10]
gi651457044	23.75	3	1	80	8.9	7.44	4.92	KH domain-containing protein [Arthrobacter sp. 35/47]
gi916781771	0.95	4	1	838	93.2	5.31	4.91	beta-mannosidase [Arthrobacter sp. 35W]
gi765006934	6.40	1	1	297	32.1	6.90	4.91	hypothetical protein [Arthrobacter sp. A3]
gi765005658	3.74	3	1	321	35.3	5.05	4.90	proline iminopeptidase [Arthrobacter sp. A3]
gi928985866	4.51	2	1	466	49.7	5.53	4.90	hypothetical protein [Arthrobacter sp. ERGS1:01]
gi640194451	3.86	2	1	466	50.4	5.22	4.90	glucose-6-phosphate dehydrogenase [Arthrobacter sp. 31Y]
gi219862195	5.60	1	1	250	26.2	4.74	4.90	NmrA family protein (plasmid) [Arthrobacter chlorophenolicus A6]
gi116610433	2.26	1	1	354	38.8	9.98	4.90	transposase IS116/IS110/IS902 family protein [Arthrobacter sp. FB24]
gi917022244	6.53	3	1	291	31.1	9.70	4.89	short-chain dehydrogenase [Arthrobacter sp. UNC362MFTsu5.1]
gi910744775	2.84	1	1	423	46.2	9.60	4.89	glutathione-binding protein GsiB [Arthrobacter sp. Hiyo8]
gi908699567	3.76	5	1	346	36.3	6.99	4.89	LacI family transcriptional regulator [Arthrobacter sp. RIT-PI-e]
gi786035308	11.33	4	1	203	22.2	4.17	4.88	ribosome maturation factor RimM [Arthrobacter chlorophenolicus]
gi517592038	2.14	2	1	560	60.0	6.62	4.88	ATP-dependent DNA helicase RecQ [Arthrobacter sp. 135MFCol5.1]
gi470221462	3.06	1	1	588	61.5	6.04	4.88	hypothetical protein ADIAG_00315 [Arthrobacter gangotriensis Lz1y]
gi651480651	7.55	7	1	159	17.4	7.61	4.88	MarR family transcriptional regulator [Arthrobacter sp. Br18]
gi651439092	7.51	6	1	293	31.1	5.39	4.88	universal stress protein UspA [Arthrobacter sp. H14]
gi545108041	7.95	4	1	239	26.1	6.23	4.88	hypothetical protein [Arthrobacter sp. AK-YN10]

gi470219967	7.27	3	1	344	35.7	5.66	4.87	Malate dehydrogenase [Arthrobacter gangotriensis Lz1y]
gi359303411	2.49	1	1	361	37.9	4.97	4.87	zinc-containing alcohol dehydrogenase [Arthrobacter globiformis NBRC 12137]
gi639130981	2.85	1	1	562	60.0	9.03	4.86	hypothetical protein [Arthrobacter sp. CAL618]
gi723608027	2.85	3	1	281	29.0	4.89	4.86	Uroporphyrinogen-III synthase [Arthrobacter sp. PAMC25486]
gi323468141	7.59	1	1	303	32.8	11.37	4.86	conserved hypothetical protein TIGR01777 [Arthrobacter phenanthrenivorans Sphe3]
gi517591329	2.41	2	1	457	49.9	7.21	4.85	cyclopropane-fatty-acyl-phospholipid synthase [Arthrobacter sp. 135MFCol5.1]
gi742069127	4.98	4	1	321	31.9	4.64	4.85	ribokinase RbsK [Arthrobacter sp. MWB30]
gi930826546	6.71	2	1	313	33.7	6.18	4.85	OpcA protein [Arthrobacter arilaitensis]
gi786030155	3.88	1	1	438	46.1	9.82	4.85	membrane protein [Arthrobacter chlorophenolicus]
gi403231189	5.17	1	1	348	36.7	5.63	4.85	D-3-phosphoglycerate dehydrogenase SerA [Arthrobacter sp. Rue61a]
gi323469641	2.78	1	1	432	46.6	6.80	4.85	dinucleotide-utilizing enzyme possibly involved in molybdopterin or thiamin biosynthesis [Art
gi635353709	12.36	4	1	89	10.4	7.27	4.85	putative uncharacterized protein [Arthrobacter siccitolerans]
gi654817553	0.95	1	1	1154	123.3	5.74	4.83	peptidase S41 [Arthrobacter sp. UNC362MFTsu5.1]
gi910738409	4.55	4	1	308	33.7	5.60	4.83	transcriptional regulatory protein EmbR [Arthrobacter sp. Hiyo4]
gi742755467	1.68	2	1	831	89.2	8.60	4.83	acyltransferase [Arthrobacter phenanthrenivorans]
gi651434395	5.92	2	1	355	37.7	5.87	4.83	uroporphyrinogen decarboxylase [Arthrobacter sp. H41]
gi517603031	2.69	4	1	335	34.7	9.42	4.83	two-component system sensor histidine kinase [Arthrobacter sp. 131MFCol6.1]
gi737786463	3.75	2	1	293	32.9	6.58	4.83	DNA methyltransferase [Arthrobacter albus]
gi323467579	8.77	8	1	285	29.2	4.78	4.82	hypothetical protein Asphe3_00450 [Arthrobacter phenanthrenivorans Sphe3]
gi640199686	9.64	6	1	249	26.2	4.79	4.82	hypothetical protein [Arthrobacter sp. 31Y]
gi652424810	3.42	3	1	527	55.2	5.24	4.82	histidine ammonia-lyase [Arthrobacter castelli]
gi470217616	1.92	1	1	884	95.7	5.22	4.82	Aminopeptidase N [Arthrobacter gangotriensis Lz1y]
gi915330549	1.90	1	1	686	75.4	6.34	4.82	hypothetical protein [Arthrobacter chlorophenolicus]
gi654819266	2.40	2	1	375	40.4	7.62	4.81	hypothetical protein [Arthrobacter sp. UNC362MFTsu5.1]
gi737787196	4.98	1	1	462	50.3	7.66	4.81	NADH dehydrogenase [Arthrobacter albus]
gi786028298	5.43	1	1	221	22.2	5.14	4.81	ArsR family transcriptional regulator [Arthrobacter chlorophenolicus]
gi476400548	3.03	4	1	462	50.2	9.29	4.81	transmembrane protein [Arthrobacter crystallopoietes BAB-32]
gi359306088	32.81	1	1	64	7.0	5.00	4.81	hypothetical protein ARGLB_047_00350 [Arthrobacter globiformis NBRC 12137]
gi918449462	2.07	2	1	581	63.5	5.22	4.80	arginine--tRNA ligase [Arthrobacter sp. SPG23]
gi654817463	8.63	1	1	313	34.1	5.59	4.80	oxidoreductase [Arthrobacter sp. UNC362MFTsu5.1]
gi742070534	4.82	2	1	249	26.4	5.45	4.80	hypothetical protein ANMWB30_25750 [Arthrobacter sp. MWB30]
gi916820453	27.27	2	1	77	9.2	5.10	4.80	transposase [Arthrobacter sp. H20]
gi651429966	4.56	2	1	373	40.6	5.66	4.80	two-component system sensor histidine kinase [Arthrobacter sanguinis]
gi742756397	4.36	2	1	528	55.7	6.30	4.79	oxidoreductase [Arthrobacter phenanthrenivorans]
gi937259294	2.66	3	1	639	68.8	5.53	4.79	choline transporter [Arthrobacter sp. Edens01]
gi910748105	7.39	2	1	203	21.6	4.92	4.79	3-isopropylmalate dehydratase large subunit [Arthrobacter sp. Hiyo8]
gi119951273	5.58	2	1	251	28.0	5.67	4.79	conserved hypothetical protein [Arthrobacter aurescens TC1]
gi930827521	3.25	1	1	584	62.5	6.87	4.78	glutathione ABC transporter ATP-binding protein [Arthrobacter arilaitensis]
gi910737949	7.14	4	1	140	15.0	9.36	4.78	hypothetical protein AHiyo4_07710 [Arthrobacter sp. Hiyo4]
gi651440836	3.63	1	1	413	43.1	5.57	4.78	carnitine dehydratase [Arthrobacter sp. 9MFCol3.1]
gi916815814	6.63	2	2	407	44.3	9.91	4.78	transposase [Arthrobacter sp. MA-N2]
gi910251393	2.75	2	1	363	39.2	9.11	4.78	peptide ABC transporter ATPase [Arthrobacter siccitolerans]
gi910744114	4.39	8	1	433	45.3	10.83	4.78	uncharacterized ABC transporter permease protein YufP [Arthrobacter sp. Hiyo8]
gi910249352	7.12	2	1	295	30.5	4.60	4.78	hypothetical protein [Arthrobacter siccitolerans]
gi515767642	4.97	5	1	322	35.3	8.51	4.78	NUDIX hydrolase [Arthrobacter sp. M2012083]
gi910694679	20.49	1	1	122	13.5	10.15	4.77	uncharacterized symporter YjmB [Arthrobacter sp. Hiyo6]
gi914713560	3.90	3	1	513	52.8	6.34	4.77	acetyl-CoA carboxyl transferase [Arthrobacter sp. ZBG10]
gi651474370	2.89	1	1	311	34.7	5.49	4.77	hypothetical protein [Arthrobacter nicotinovorans]
gi654823191	3.91	3	1	256	28.5	7.27	4.77	RNA polymerase subunit sigma-28 [Arthrobacter sp. I3]
gi640197989	1.12	2	1	1337	137.9	4.60	4.77	hypothetical protein [Arthrobacter sp. 31Y]
gi652452337	6.90	1	1	319	32.7	5.40	4.77	prephenate dehydratase [Geodermatophilaceae bacterium URHA0031]
gi651496120	12.02	1	1	183	19.5	10.26	4.77	hypothetical protein [Arthrobacter sp. H20]
gi930828122	8.04	1	1	286	30.3	4.84	4.76	NGG1p interacting factor NIF3 [Arthrobacter arilaitensis]
gi928486438	4.17	1	1	503	52.5	5.69	4.76	UDP-N-acetylmuramoyl-tripeptide--D-alanyl-D-alanine ligase [Arthrobacter alpinus]
gi910248900	4.73	1	1	402	40.7	5.24	4.76	N-acetylglucosamine 6-phosphate deacetylase [Arthrobacter siccitolerans]

gi910696370	19.13	3	1	115	12.4	5.25	4.76	uncharacterized HTH-type transcriptional regulator YagI [Arthrobacter sp. Hiyo6]
gi674646243	3.85	2	1	597	63.2	6.98	4.76	DNA polymerase I, thermostable [Arthrobacter sp. 11W110_air]
gi307744942	1.46	2	1	616	66.7	5.74	4.76	HNH endonuclease domain-containing protein [Arthrobacter arilaitensis Re117]
gi654818961	9.15	2	1	164	17.9	6.39	4.75	CoA-binding protein [Arthrobacter sp. UNC362MFTsu5.1]
gi551256888	4.17	2	1	408	41.1	5.85	4.75	glycerate kinase [Arthrobacter sp. PAO19]
gi517590304	3.23	1	1	402	41.9	5.30	4.75	hypothetical protein [Arthrobacter sp. 135MFCol5.1]
gi639129656	3.81	1	1	499	54.8	7.44	4.75	hypothetical protein [Arthrobacter sp. CAL618]
gi723608088	8.97	1	1	234	25.8	5.67	4.75	hypothetical protein ART_1865 [Arthrobacter sp. PAMC25486]
gi219860303	10.14	3	1	138	15.4	11.12	4.75	ribosomal protein L16 [Arthrobacter chlorophenolicus A6]
gi823665507	9.85	8	1	203	22.1	9.07	4.75	hypothetical protein AA310_05795 [Arthrobacter sp. YC-RL1]
gi307746563	4.34	1	1	461	48.3	5.20	4.75	malate dehydrogenase (oxaloacetate-decarboxylating) [Arthrobacter arilaitensis Re117]
gi927032810	3.76	1	1	426	47.1	5.81	4.74	hypothetical protein AFL94_11430 [Arthrobacter sp. LS16]
gi823666112	9.41	2	1	202	22.5	5.03	4.74	DSBA oxidoreductase [Arthrobacter sp. YC-RL1]
gi674646607	5.82	2	1	189	20.3	5.26	4.74	Bacterial regulatory proteins, tetR family [Arthrobacter sp. 11W110_air]
gi910283613	1.66	2	1	721	74.0	5.55	4.74	hypothetical protein [Arthrobacter sp. A3]
gi928486224	2.25	1	1	711	73.7	9.83	4.74	hypothetical protein AOC05_03085 [Arthrobacter alpinus]
gi823665685	3.85	2	1	364	38.9	5.11	4.74	oxidoreductase [Arthrobacter sp. YC-RL1]
gi765006105	1.60	1	1	875	95.1	5.08	4.73	DNA polymerase I [Arthrobacter sp. A3]
gi359307692	4.29	1	1	396	41.6	7.11	4.73	putative transcriptional regulator [Arthrobacter globiformis NBRC 12137]
gi654825760	1.95	1	1	665	71.5	5.94	4.73	hypothetical protein [Arthrobacter sp. H5]
gi654825678	9.57	2	1	94	10.3	10.37	4.73	hypothetical protein [Arthrobacter sp. H5]
gi916813788	2.36	1	1	890	94.3	7.09	4.73	FAD-dependent oxidoreductase [Arthrobacter nicotinovorans]
gi786031636	5.36	1	1	336	35.5	5.81	4.73	VWA domain-containing protein [Arthrobacter chlorophenolicus]
gi476398739	6.99	6	1	186	21.3	7.43	4.73	integrase/recombinase, partial [Arthrobacter crystallopoietes BAB-32]
gi476401362	8.39	1	1	155	16.2	7.42	4.72	ArsR family transcriptional regulator [Arthrobacter crystallopoietes BAB-32]
gi737771591	1.26	1	1	949	104.3	5.49	4.72	hypothetical protein [Arthrobacter sp. TB 26]
gi323471188	3.11	1	1	482	53.8	5.10	4.72	hypothetical protein Asphe3_37840 [Arthrobacter phenanthrenivorans Sphe3]
gi654824595	10.71	3	1	112	12.9	8.59	4.72	ArsR family transcriptional regulator [Arthrobacter sp. I3]
gi651482837	3.55	4	1	563	59.8	4.83	4.71	acetolactate synthase [Arthrobacter sp. Br18]
gi635350625	2.56	2	1	508	53.8	4.74	4.71	TAP-like family protein [Arthrobacter siccitolerans]
gi910738655	2.94	1	1	442	47.7	9.92	4.71	inner membrane metabolite transport protein YhjE [Arthrobacter sp. Hiyo4]
gi674644772	4.10	6	1	244	26.6	7.21	4.70	Transcriptional regulatory protein YpdB [Arthrobacter sp. 11W110_air]
gi654822099	10.00	2	1	250	27.6	6.54	4.70	hypothetical protein [Arthrobacter sp. I3]
gi742853362	9.38	5	2	373	39.1	6.74	4.70	UDP-N-acetylglucosamine--N-acetylmuramyl-(pentapeptide) pyrophosphoryl-undecaprenol N
gi914715291	2.45	3	1	327	32.9	11.36	4.70	hypothetical protein [Arthrobacter sp. ZBG10]
gi765004279	7.42	2	1	337	36.1	6.47	4.70	hypothetical protein [Arthrobacter sp. A3]
gi928486422	14.11	2	2	163	17.9	4.55	4.70	hypothetical protein AOC05_04555 [Arthrobacter alpinus]
gi551254973	3.54	2	1	452	50.4	5.94	4.69	ATPase [Arthrobacter sp. PAO19]
gi517608989	11.11	3	2	351	37.0	5.74	4.69	hypothetical protein [Arthrobacter sp. 161MFSha2.1]
gi916834853	2.55	1	1	550	57.9	5.25	4.69	histidine kinase [Arthrobacter sp. H14]
gi359307198	5.20	1	1	327	34.2	5.90	4.69	putative LacI family transcriptional regulator [Arthrobacter globiformis NBRC 12137]
gi723608363	10.26	1	1	195	19.9	4.67	4.68	LSU ribosomal protein L10p (P0) [Arthrobacter sp. PAMC25486]
gi651502616	4.16	1	1	505	54.0	4.84	4.68	microcystinase C [Arthrobacter sp. 35W]
gi928487378	6.20	1	1	258	28.0	4.86	4.68	hypothetical protein AOC05_11070 [Arthrobacter alpinus]
gi917732924	3.31	3	1	302	32.7	10.24	4.68	hypothetical protein [Arthrobacter sp. MWB30]
gi723609851	3.22	2	1	373	39.6	6.25	4.68	hypothetical protein ART_3628 [Arthrobacter sp. PAMC25486]
gi917739673	4.97	1	1	382	40.0	10.48	4.68	hypothetical protein [Arthrobacter sp. W1]
gi910250664	5.77	2	1	156	16.9	4.45	4.68	glyoxalase family protein [Arthrobacter siccitolerans]
gi470220807	23.26	2	1	43	5.1	9.96	4.67	hypothetical protein ADIAG_00753 [Arthrobacter gangotriensis Lz1y]
gi551256694	3.99	2	1	501	55.8	5.06	4.67	glutamate--tRNA ligase [Arthrobacter sp. PAO19]
gi443481594	7.97	1	1	251	26.9	10.52	4.67	hypothetical protein G205_11704 [Arthrobacter nitrophenolicus]
gi918267295	21.62	1	1	74	8.4	8.60	4.67	hypothetical protein AHiyo1_29370 [Arthrobacter sp. Hiyo1]
gi307744088	19.40	2	1	67	7.9	6.67	4.67	hypothetical protein AARI_08340 [Arthrobacter arilaitensis Re117]
gi759702733	16.95	1	1	59	6.1	9.99	4.67	hypothetical protein [Arthrobacter globiformis]
gi470221416	4.45	2	1	382	37.3	5.01	4.67	glycerate kinase [Arthrobacter gangotriensis Lz1y]

gi116612852	19.54	1	1	87	9.8	10.13	4.67	hypothetical protein Arth_4403 (plasmid) [Arthrobacter sp. FB24]
gi674645318	4.53	3	1	265	30.0	8.15	4.67	HTH-type transcriptional repressor DasR [Arthrobacter sp. 11W110_air]
gi211582727	3.89	1	1	489	53.1	5.88	4.66	Pc12g13000 [Penicillium rubens Wisconsin 54-1255]
gi908697922	4.29	1	1	373	38.5	9.33	4.66	hypothetical protein [Arthrobacter sp. RIT-PI-e]
gi927293457	3.03	3	1	264	29.2	5.30	4.66	hypothetical protein AL755_04355 [Arthrobacter sp. ERGS1:01]
gi162953037	2.45	1	1	531	59.2	9.31	4.66	conserved hypothetical protein [Renibacterium salmoninarum ATCC 33209]
gi651431353	4.05	2	1	518	56.7	5.74	4.66	ABC transporter ATP-binding protein [Arthrobacter sanguinis]
gi651439118	5.44	2	1	331	36.6	5.01	4.66	xylose isomerase [Arthrobacter sp. H14]
gi759763288	5.24	2	1	248	26.9	7.49	4.65	glutamate ABC transporter ATP-binding protein [Arthrobacter gangotriensis]
gi786025451	3.22	6	1	590	62.2	5.11	4.65	hypothetical protein [Arthrobacter chlorophenolicus]
gi517598813	3.77	2	1	478	51.0	6.61	4.65	PucR family transcriptional regulator [Arthrobacter sp. 162MFSha1.1]
gi470217479	8.00	2	1	250	27.5	4.64	4.65	MerR family transcriptional regulator [Arthrobacter gangotriensis Lz1y]
gi939050231	6.43	4	1	249	26.9	9.72	4.65	hypothetical protein [Arthrobacter sp. JCM 19049]
gi654814499	5.46	3	1	403	42.7	5.58	4.65	hypothetical protein [Arthrobacter sp. MA-N2]
gi928487990	1.65	2	1	788	86.3	5.20	4.65	kojibiose phosphorylase [Arthrobacter alpinus]
gi937262367	8.10	2	2	284	30.5	9.96	4.65	type II secretion system protein F [Arthrobacter sp. Edens01]
gi760164629	4.35	1	1	368	39.7	4.81	4.65	transaldolase [Arthrobacter crystallopoietes]
gi654811382	2.51	2	1	557	58.2	5.31	4.64	L-aspartate oxidase [Arthrobacter sp. MA-N2]
gi654812248	3.30	2	1	364	38.4	10.96	4.64	fusaric acid resistance protein [Arthrobacter sp. MA-N2]
gi939051011	5.70	4	1	316	35.6	5.71	4.64	cytochrome, partial [Arthrobacter sp. JCM 19049]
gi403228167	1.97	1	1	711	76.9	5.60	4.64	putative glycogen debranching enzyme family protein [Arthrobacter sp. Rue61a]
gi759725696	6.25	1	1	208	22.0	9.98	4.64	hypothetical protein [Arthrobacter sp. I3]
gi518312608	19.60	2	2	199	21.5	6.55	4.64	MULTISPECIES: uracil phosphoribosyltransferase [Arthrobacter]
gi470221751	5.63	2	1	284	31.1	4.69	4.64	Chlorocatechol 1,2-dioxygenase [Arthrobacter gangotriensis Lz1y]
gi759704613	4.20	1	1	286	31.4	7.28	4.63	pseudouridine synthase [Arthrobacter globiformis]
gi636846874	4.37	1	1	526	54.2	5.38	4.63	transcriptional regulator [Arthrobacter sp. TB 26]
gi742855455	1.73	1	1	635	70.7	7.71	4.63	glycosyltransferase [Arthrobacter sp. W1]
gi518313695	8.00	3	1	275	30.2	4.89	4.63	hypothetical protein [Arthrobacter sp. TB 23]
gi551254533	7.33	2	1	232	25.7	4.98	4.63	DtxR family transcriptional regulator [Arthrobacter sp. PAO19]
gi651435100	5.33	3	1	394	40.5	4.59	4.63	cell division protein FtsZ [Arthrobacter sp. H41]
gi651439827	16.15	1	1	130	14.9	11.22	4.63	50S ribosomal protein L20 [Arthrobacter sp. H14]
gi916357121	21.37	1	1	117	12.2	5.48	4.62	phosphotransferase [Arthrobacter sp. 162MFSha1.1]
gi910694916	3.37	1	1	416	46.2	5.25	4.62	proteasome-associated ATPase [Arthrobacter sp. Hiyo6]
gi930828291	2.74	2	1	401	43.6	5.17	4.62	acyl-CoA dehydrogenase [Arthrobacter arilaitensis]
gi654817597	3.13	1	1	511	56.6	5.44	4.62	alpha-L-arabinofuranosidase [Arthrobacter sp. UNC362MFTsu5.1]
gi654827966	4.71	3	2	594	64.8	6.40	4.62	hypothetical protein [Arthrobacter sp. H5]
gi639130721	3.31	2	1	513	55.3	9.36	4.62	transposase [Arthrobacter sp. CAL618]
gi674645913	5.73	2	1	192	20.4	6.93	4.61	DNA-3-methyladenine glycosylase 1 [Arthrobacter sp. 11W110_air]
gi651442867	13.29	4	1	158	17.2	7.52	4.61	hypothetical protein [Arthrobacter sp. 9MFCol3.1]
gi674645845	1.49	1	1	939	100.6	5.45	4.61	hypothetical protein BN1051_02043 [Arthrobacter sp. 11W110_air]
gi654823627	3.34	1	1	718	74.7	6.11	4.61	ATPase [Arthrobacter sp. I3]
gi658509193	7.23	2	1	318	32.6	5.14	4.61	ABC transporter substrate-binding protein [Arthrobacter sp. TB 26]
gi918469367	7.01	2	1	157	17.2	8.98	4.61	ribonucleoside-diphosphate reductase [Arthrobacter crystallopoietes]
gi651438782	4.01	3	1	548	58.4	5.19	4.61	acetolactate synthase [Arthrobacter sp. H14]
gi651480070	3.81	1	1	473	51.6	5.20	4.61	pyridine nucleotide-disulfide oxidoreductase [Arthrobacter sp. Br18]
gi323469213	4.66	1	1	408	40.9	5.00	4.60	subtilisin-like serine protease [Arthrobacter phenanthrenivorans Sphe3]
gi443481783	11.11	2	1	144	15.6	4.50	4.60	glyoxalase/bleomycin resistance protein/dioxygenase [Arthrobacter nitrophenolicus]
gi908699212	6.84	1	1	190	20.1	8.75	4.60	molybdenum cofactor biosysynthesis protein [Arthrobacter sp. RIT-PI-e]
gi910695704	6.73	1	1	208	22.0	6.39	4.60	HTH-type transcriptional repressor PurR [Arthrobacter sp. Hiyo6]
gi695200271	4.04	2	1	421	45.8	6.13	4.60	unknown (plasmid) [Arthrobacter keyseri]
gi759726122	4.38	1	1	365	38.4	5.55	4.60	histidine kinase, partial [Arthrobacter sp. I3]
gi917760292	2.57	2	1	662	73.4	5.27	4.59	WYL domain-containing protein [Arthrobacter sp. L77]
gi674645612	1.35	2	1	740	80.2	5.82	4.59	ATP-dependent DNA helicase RecG [Arthrobacter sp. 11W110_air]
gi640201901	14.75	1	1	183	19.1	9.72	4.59	peptidase A24 [Arthrobacter sp. 31Y]
gi737783903	4.00	1	1	400	41.1	4.96	4.59	transcriptional regulator [Arthrobacter nitrophenolicus]

gi654823147	3.23	3	1	372	39.7	6.95	4.59	aminotransferase [Arthrobacter sp. I3]
gi654816944	6.52	2	1	138	15.2	5.27	4.59	hypothetical protein [Arthrobacter sp. UNC362MFTsu5.1]
gi742072549	2.93	1	1	682	70.4	5.67	4.59	hypothetical protein ANMWB30_03900 [Arthrobacter sp. MWB30]
gi359306617	2.93	1	1	376	40.6	6.33	4.59	ABC transporter ATP-binding protein [Arthrobacter globiformis NBRC 12137]
gi737781474	5.10	1	1	255	27.9	6.65	4.59	ABC transporter ATP-binding protein [Arthrobacter sp. 35W]
gi654818982	2.63	4	1	419	46.1	10.01	4.59	hypothetical protein [Arthrobacter sp. UNC362MFTsu5.1]
gi542109712	4.33	2	1	300	32.9	5.24	4.59	GlcNAc-PI de-N-acetylase [Arthrobacter sp. AK-YN10]
gi759733978	5.42	2	1	443	47.2	6.28	4.59	phosphoribosyl transferase [Arthrobacter sp. L77]
gi910743620	14.29	1	1	140	15.4	4.63	4.58	putative 8-oxo-dGTP diphosphatase 2 [Arthrobacter sp. Hiyo8]
gi648572501	1.47	2	1	817	85.4	6.64	4.58	glycoside hydrolase [Arthrobacter sp. 135MFCol5.1]
gi307745978	5.06	1	1	257	28.0	8.91	4.58	IclR-family transcriptional regulator [Arthrobacter arilaitensis Re117]
gi917442270	3.34	3	1	509	54.5	4.73	4.58	hypothetical protein [Arthrobacter albus]
gi219858979	0.44	1	1	2040	211.4	4.92	4.58	Fibronectin type III domain protein [Arthrobacter chlorophenolicus A6]
gi910692721	4.06	4	1	320	34.6	5.31	4.58	uncharacterized oxidoreductase YuiH, partial [Arthrobacter sp. Hiyo6]
gi916820212	5.11	4	1	235	25.0	5.07	4.58	hypothetical protein [Arthrobacter sp. H20]
gi542107939	15.38	3	1	78	9.1	4.53	4.58	hypothetical protein M707_14715 [Arthrobacter sp. AK-YN10]
gi823665387	4.95	1	1	202	22.2	9.31	4.58	CDP-diacylglycerol--glycerol-3-phosphate 3-phosphatidyltransferase [Arthrobacter sp. YC-RL
gi651481532	9.04	1	1	177	18.8	7.66	4.58	hypothetical protein [Arthrobacter sp. Br18]
gi917739657	4.20	3	1	262	28.1	4.79	4.58	hypothetical protein [Arthrobacter sp. W1]
gi517609022	6.43	3	1	389	44.8	6.23	4.58	hypothetical protein [Arthrobacter sp. 161MFSha2.1]
gi939051103	7.14	2	2	364	39.4	5.44	4.58	hypothetical protein [Arthrobacter sp. JCM 19049]
gi749402470	4.55	1	1	286	30.7	5.91	4.58	16S rRNA methyltransferase [Arthrobacter sp. AK-YN10]
gi403229886	4.46	2	1	314	34.3	6.71	4.58	alpha/beta hydrolase fold superfamily protein [Arthrobacter sp. Rue61a]
gi651486518	1.98	2	1	353	37.6	9.91	4.58	hypothetical protein, partial [Arthrobacter sp. Br18]
gi757624831	2.87	2	1	557	60.4	5.05	4.58	Triostin synthetase I [Arthrobacter sp. SPG23]
gi116611372	2.91	5	1	412	45.2	10.74	4.57	protein of unknown function DUF58 [Arthrobacter sp. FB24]
gi307743705	2.98	2	1	403	42.1	5.77	4.57	acetyl-CoA C-acyltransferase [Arthrobacter arilaitensis Re117]
gi767256629	12.50	4	1	112	12.9	6.79	4.57	ArsR family transcriptional regulator [Arthrobacter sp. IHBB 11108]
gi651491330	1.68	1	1	714	81.2	5.99	4.57	ribonucleotide-diphosphate reductase subunit alpha [Arthrobacter sp. H20]
gi515765127	7.64	1	1	144	16.1	5.39	4.57	CBS domain-containing protein [Arthrobacter sp. M2012083]
gi760164105	7.89	2	1	304	32.1	5.67	4.57	2-dehydropantoate 2-reductase [Arthrobacter crystallopoietes]
gi906448504	1.92	3	1	937	98.6	5.53	4.57	hypothetical protein AC792_01700 [Arthrobacter sp. RIT-PI-e]
gi518312772	4.01	2	1	424	46.0	5.48	4.57	hypothetical protein [Arthrobacter sp. TB 23]
gi651467723	4.75	1	1	337	36.7	5.50	4.57	UDP-glucose 4-epimerase [Arthrobacter sp. 35/47]
gi651503327	7.17	1	1	251	27.3	6.06	4.57	MerR family transcriptional regulator [Arthrobacter sp. 35W]
gi737789394	7.08	2	1	212	23.8	9.95	4.57	translation initiation factor IF-3 [Arthrobacter albus]
gi910697868	3.86	1	1	311	34.4	5.20	4.57	conserved hypothetical protein [Arthrobacter sp. Hiyo6]
gi162955574	7.43	3	1	175	18.6	9.63	4.57	A3(2) glycogen metabolism cluster I [Renibacterium salmoninarum ATCC 33209]
gi786030531	6.43	1	1	342	35.8	7.12	4.56	hydroxyacid dehydrogenase [Arthrobacter chlorophenolicus]
gi927293838	3.18	1	1	409	43.8	6.92	4.56	histidine kinase [Arthrobacter sp. ERGS1:01]
gi476399977	3.55	1	1	422	45.5	5.50	4.56	diaminobutyrate--2-oxoglutarate aminotransferase [Arthrobacter crystallopoietes BAB-32]
gi470221479	1.59	2	1	502	52.8	8.06	4.56	sulfate transporter [Arthrobacter gangotriensis Lz1y]
gi651473745	2.54	2	1	355	37.4	11.22	4.56	fusaric acid resistance protein [Arthrobacter nicotinovorans]
gi737811861	9.85	1	1	264	27.9	7.97	4.56	ABC transporter [Arthrobacter sp. 35/47]
gi765010538	4.67	1	1	300	32.5	7.09	4.56	hypothetical protein [Arthrobacter sp. A3]
gi654818047	7.78	2	1	180	19.3	9.44	4.55	hypothetical protein [Arthrobacter sp. UNC362MFTsu5.1]
gi910741474	10.17	1	1	177	19.3	5.36	4.55	uncharacterized protein C4H3.03c [Arthrobacter sp. Hiyo4]
gi470220405	6.52	3	1	414	46.3	8.68	4.55	transposase IS204//IS1096/IS1165 family protein [Arthrobacter gangotriensis Lz1y]
gi914717172	2.87	1	1	557	60.2	5.64	4.55	Pup deamidase/depupylase [Arthrobacter sp. ZBG10]
gi307743957	5.69	3	1	299	32.9	6.39	4.55	LysR-family transcriptional regulator [Arthrobacter arilaitensis Re117]
gi476400560	5.59	1	1	322	34.2	5.63	4.55	hypothetical protein D477_013005 [Arthrobacter crystallopoietes BAB-32]
gi116611798	4.12	3	1	364	38.8	5.33	4.55	transcriptional regulator, LacI family [Arthrobacter sp. FB24]
gi476401398	5.81	4	1	327	36.8	6.46	4.55	phenylacetate-CoA oxygenase subunit PaaA [Arthrobacter crystallopoietes BAB-32]
gi910743763	29.63	3	1	81	9.1	4.93	4.55	ribosome-recycling factor [Arthrobacter sp. Hiyo8]
gi786029889	4.27	1	1	468	49.9	4.83	4.55	glycosyl hydrolase family 32 [Arthrobacter chlorophenolicus]

gi307744745	2.64	1	1	568	63.6	5.26	4.55	putative fatty-acid--Co-A ligase [Arthrobacter arilaitensis Re117]
gi517608657	11.61	1	1	155	16.9	6.13	4.55	GCN5 family N-acetyltransferase [Arthrobacter sp. 161MFSha2.1]
gi786026143	1.86	3	1	484	52.8	5.81	4.54	pilus assembly protein CpaF [Arthrobacter chlorophenolicus]
gi759710143	7.37	1	1	339	35.0	5.40	4.54	hypothetical protein [Arthrobacter sp. 135MFCol5.1]
gi723609883	6.73	4	1	208	21.2	4.72	4.54	hypothetical protein ART_3660 [Arthrobacter sp. PAMC25486]
gi916820473	9.80	2	1	204	22.1	5.14	4.54	hypothetical protein [Arthrobacter sp. H20]
gi918469412	2.33	2	1	472	50.7	5.15	4.54	succinate-semialdehyde dehydrogenase [Arthrobacter crystallopoietes]
gi910694737	7.06	2	1	269	29.5	10.24	4.54	hypothetical protein AHiyo6_24300, partial [Arthrobacter sp. Hiyo6]
gi307745410	3.00	3	1	367	38.8	5.19	4.54	conserved hypothetical protein [Arthrobacter arilaitensis Re117]
gi674645958	3.90	1	1	385	40.8	9.28	4.54	Carnitine transport ATP-binding protein OpuCA [Arthrobacter sp. 11W110_air]
gi916814537	4.30	2	1	279	28.7	5.05	4.54	hypothetical protein [Arthrobacter nicotinovorans]
gi916813773	17.44	1	1	86	9.2	9.52	4.54	hypothetical protein [Arthrobacter nicotinovorans]
gi916710608	3.21	1	1	405	43.6	10.87	4.54	hypothetical protein [Arthrobacter sp. CAL618]
gi635352555	4.32	2	1	301	32.4	10.29	4.54	bacterial type II secretion system F domain protein [Arthrobacter siccitolerans]
gi930827203	2.12	3	1	614	65.7	9.28	4.53	multidrug ABC transporter ATP-binding protein [Arthrobacter arilaitensis]
gi916692220	3.27	1	1	306	32.5	6.16	4.53	hypothetical protein [Arthrobacter castelli]
gi323471565	7.63	2	1	262	29.0	4.92	4.53	hypothetical protein Asphe3_41840 (plasmid) [Arthrobacter phenanthrenivorans Sphe3]
gi635354038	5.60	2	1	268	29.2	5.00	4.53	aldo/keto reductase family protein [Arthrobacter siccitolerans]
gi219857798	11.41	3	2	377	40.6	5.64	4.53	oxidoreductase domain protein [Arthrobacter chlorophenolicus A6]
gi636843428	4.06	1	1	394	40.1	5.57	4.53	FAD/NAD(P)-binding oxidoreductase, partial [Arthrobacter sp. TB 26]
gi765013549	3.07	1	1	391	42.0	8.21	4.53	hypothetical protein [Arthrobacter sp. A3]
gi517604971	3.30	2	1	575	56.7	5.54	4.53	hypothetical protein [Arthrobacter sp. 131MFCol6.1]
gi219860655	6.20	2	1	258	28.3	4.91	4.53	Phosphoglycerate mutase [Arthrobacter chlorophenolicus A6]
gi914715152	4.63	4	1	389	41.0	9.36	4.53	hypothetical protein [Arthrobacter sp. ZBG10]
gi757623381	4.48	1	1	491	50.1	8.51	4.52	protoporphyrinogen oxidase [Arthrobacter sp. SPG23]
gi928486226	5.88	5	1	204	23.2	10.24	4.52	hypothetical protein AOC05_03105 [Arthrobacter alpinus]
gi219858401	11.43	3	1	210	22.8	7.72	4.52	transcriptional regulator, ArsR family [Arthrobacter chlorophenolicus A6]
gi674644294	5.34	2	1	262	28.8	9.23	4.52	Bicarbonate transport ATP-binding protein CmpD [Arthrobacter sp. 11W110_air]
gi470219943	3.92	1	1	408	43.9	5.60	4.52	dyp-type peroxidase family protein [Arthrobacter gangotriensis Lz1y]
gi930825471	5.95	1	1	269	28.2	4.84	4.52	alpha-dehydro-beta-deoxy-D-glucarate aldolase [Arthrobacter arilaitensis]
gi916876263	0.43	1	1	3503	372.3	5.07	4.52	non-ribosomal peptide synthetase [Arthrobacter sp. 31Y]
gi517607314	2.35	1	1	554	59.1	5.36	4.51	fumarate reductase [Arthrobacter sp. 161MFSha2.1]
gi648574344	4.20	1	1	286	29.9	6.18	4.51	NAD(P)-dependent oxidoreductase [Arthrobacter sp. 162MFSha1.1]
gi786035048	3.27	1	1	703	77.1	5.62	4.51	glycogen debranching protein [Arthrobacter chlorophenolicus]
gi162952758	4.93	1	1	406	44.6	6.29	4.51	glycosyltransferase [Renibacterium salmoninarum ATCC 33209]
gi918269151	3.84	3	1	391	42.0	7.97	4.51	UPF0118 membrane protein YrrI [Arthrobacter sp. Hiyo1]
gi908699028	5.93	1	1	253	27.7	5.25	4.51	diguanylate phosphodiesterase [Arthrobacter sp. RIT-PI-e]
gi928487912	2.09	1	1	335	37.4	8.72	4.51	hypothetical protein AOC05_14460 [Arthrobacter alpinus]
gi757624889	1.77	1	1	451	48.9	6.49	4.51	monooxygenase [Arthrobacter sp. SPG23]
gi636847103	4.74	1	1	401	43.3	7.14	4.51	hypothetical protein [Arthrobacter sp. TB 26]
gi651434036	2.47	1	1	446	47.4	3.71	4.50	sugar ABC transporter substrate-binding protein [Arthrobacter sp. H41]
gi651464454	1.45	2	1	692	76.0	5.24	4.50	glycogen debranching protein [Arthrobacter sp. 35/47]
gi134074836	2.75	1	1	327	36.2	7.42	4.50	unnamed protein product [Aspergillus niger]
gi654815660	6.35	1	1	252	26.4	6.24	4.50	esterase [Arthrobacter sp. UNC362MFTsu5.1]
gi307743576	43.14	2	1	51	6.1	6.57	4.50	hypothetical protein AARI_03140 [Arthrobacter arilaitensis Re117]
gi910696854	8.07	1	1	161	18.7	5.14	4.50	alpha-crystallin [Arthrobacter sp. Hiyo6]
gi908699075	2.00	1	1	400	41.8	5.95	4.50	hypothetical protein [Arthrobacter sp. RIT-PI-e]
gi651437905	5.96	1	1	218	23.2	10.21	4.50	50S ribosomal protein L3 [Arthrobacter sp. H14]
gi654825445	7.07	2	1	184	18.4	8.94	4.50	hypothetical protein [Arthrobacter sp. I3]
gi652423585	4.37	2	1	252	27.0	4.92	4.50	hypothetical protein [Arthrobacter castelli]
gi927294400	9.09	1	1	253	28.3	8.19	4.50	hypothetical protein AL755_10835 [Arthrobacter sp. ERGS1:01]
gi476399575	2.14	2	1	701	76.4	6.55	4.50	acyl-CoA oxidase domain-containing protein [Arthrobacter crystallopoietes BAB-32]
gi652424301	4.89	1	1	327	34.7	9.06	4.50	tyrosine recombinase XerD [Arthrobacter castelli]
gi323467813	2.13	1	1	798	88.6	6.09	4.50	phosphoketolase [Arthrobacter phenanthrenivorans Sphe3]
gi916816463	5.05	2	1	277	31.7	7.36	4.50	hypothetical protein [Arthrobacter sp. MA-N2]

gi654824516	8.92	1	1	269	28.6	6.43	4.50	alpha/beta hydrolase [Arthrobacter sp. I3]
gi759730259	1.98	1	1	504	54.9	6.09	4.49	hypothetical protein [Arthrobacter sp. L77]
gi403229246	2.21	1	1	589	65.7	6.62	4.49	hypothetical protein ARUE_c17620 [Arthrobacter sp. Rue61a]
gi760164809	14.29	1	1	126	14.3	4.65	4.49	hypothetical protein [Arthrobacter crystallopoietes]
gi742757901	3.93	1	1	433	44.6	5.67	4.49	glycine oxidase [Arthrobacter phenanthrenivorans]
gi652424097	1.55	2	1	711	76.6	5.55	4.49	anthranilate synthase [Arthrobacter castelli]
gi517604405	6.91	2	1	188	18.6	9.79	4.49	hypothetical protein [Arthrobacter sp. 131MFCol6.1]
gi910738592	11.61	2	2	155	17.1	11.75	4.49	transglutaminase domain protein [Arthrobacter sp. Hiyo4]
gi651448921	2.14	1	1	608	63.8	6.23	4.49	ABC transporter [Arthrobacter nicotinovorans]
gi219860589	15.58	1	1	77	8.0	4.84	4.49	carbon storage regulator, CsrA [Arthrobacter chlorophenolicus A6]
gi652425820	4.49	3	1	535	57.4	5.45	4.48	methylcrotonoyl-CoA carboxylase [Arthrobacter castelli]
gi517608717	10.07	2	1	149	16.7	9.47	4.48	AraC family transcriptional regulator [Arthrobacter sp. 161MFSha2.1]
gi930827784	4.43	4	1	271	28.7	5.35	4.48	citrate lyase [Arthrobacter arilaitensis]
gi476401625	7.33	1	1	191	20.8	5.83	4.48	XRE family transcriptional regulator [Arthrobacter crystallopoietes BAB-32]
gi652422968	5.67	1	1	194	21.3	7.08	4.48	bifunctional pyrimidine regulatory protein PyrR uracil phosphoribosyltransferase [Arthrobacte
gi765012202	3.33	1	1	570	62.5	6.89	4.48	ABC transporter [Arthrobacter sp. A3]
gi737800903	6.93	1	1	231	25.0	5.02	4.48	histidine kinase [Arthrobacter castelli]
gi651503228	1.35	1	1	1628	179.0	5.07	4.48	glutamate dehydrogenase [Arthrobacter sp. 35W]
gi651463914	9.36	1	1	171	18.3	9.55	4.48	hypothetical protein [Arthrobacter sp. 35/47]
gi930826892	9.38	1	1	224	25.0	5.82	4.48	GntR family transcriptional regulator [Arthrobacter arilaitensis]
gi910739655	23.33	2	1	90	9.7	12.18	4.48	hypothetical protein AHiyo4_24770 [Arthrobacter sp. Hiyo4]
gi917407385	3.52	1	1	653	68.8	6.21	4.48	hypothetical protein [Arthrobacter nitrophenolicus]
gi517590801	3.56	1	1	253	27.0	5.21	4.48	hypothetical protein [Arthrobacter sp. 135MFCol5.1]
gi767257086	4.81	2	1	270	29.7	6.81	4.47	hypothetical protein UM93_04595 [Arthrobacter sp. IHBB 11108]
gi939050297	2.93	1	1	376	40.4	8.28	4.47	histidine kinase [Arthrobacter sp. JCM 19049]
gi757624401	6.19	1	1	194	21.0	6.73	4.47	hypothetical protein TV39_10340 [Arthrobacter sp. SPG23]
gi759733375	5.47	1	1	384	41.0	4.91	4.47	mannose-1-phosphate guanylyltransferase [Arthrobacter sp. L77]
gi910283735	3.51	1	1	285	29.6	7.44	4.47	hypothetical protein [Arthrobacter sp. A3]
gi919218901	1.11	1	1	1439	148.9	6.09	4.47	hypothetical protein [Arthrobacter sp. YC-RL1]
gi443479958	11.70	1	1	171	19.1	8.40	4.47	hypothetical protein G205_21464 [Arthrobacter nitrophenolicus]
gi737800883	1.55	1	1	582	64.2	5.47	4.47	1,4-alpha-glucan-branching protein [Arthrobacter castelli]
gi542109842	1.37	4	1	949	101.0	6.00	4.47	phosphoenolpyruvate synthase [Arthrobacter sp. AK-YN10]
gi759725635	2.84	2	1	844	90.3	7.87	4.47	AMP-dependent synthetase [Arthrobacter sp. I3]
gi765013016	6.29	1	1	175	19.9	6.24	4.47	RecX family transcriptional regulator [Arthrobacter sp. A3]
gi652425698	5.35	1	1	355	36.3	4.64	4.47	hypothetical protein [Arthrobacter castelli]
gi918268222	2.85	1	1	421	44.6	10.04	4.47	xylose transport system permease protein XylH [Arthrobacter sp. Hiyo1]
gi651494474	2.34	1	1	513	55.5	5.40	4.47	GTP-binding protein [Arthrobacter sp. H20]
gi916820629	4.10	1	1	415	44.0	5.21	4.47	aspartate aminotransferase [Arthrobacter sp. H20]
gi470220706	3.41	1	1	381	42.7	6.38	4.46	UDP-N-acetylglucosamine 2-epimerase [Arthrobacter gangotriensis Lz1y]
gi910746757	8.90	1	1	191	21.2	7.65	4.46	hypothetical protein AHiyo8_44590 [Arthrobacter sp. Hiyo8]
gi742855736	11.27	1	1	142	15.6	9.83	4.46	hypothetical protein [Arthrobacter sp. W1]
gi737788100	3.68	1	1	353	38.8	10.07	4.46	hypothetical protein [Arthrobacter albus]
gi119951036	3.35	1	1	657	70.5	6.19	4.46	1-deoxy-D-xylulose-5-phosphate synthase [Arthrobacter aurescens TC1]
gi759728182	0.83	1	1	1206	129.6	5.17	4.46	chromosome segregation protein SMC [Arthrobacter sp. UNC362MFTsu5.1]
gi823667110	4.62	1	1	390	42.0	6.06	4.46	molecular chaperone GroES [Arthrobacter sp. YC-RL1]
gi219860683	4.46	3	1	224	24.1	5.52	4.46	pentapeptide repeat protein [Arthrobacter chlorophenolicus A6]
gi742851455	3.32	2	1	452	48.0	4.41	4.46	sugar ABC transporter substrate-binding protein [Arthrobacter sp. W1]
gi910739882	16.46	1	1	79	8.0	11.78	4.46	hypothetical protein AHiyo4_27040 [Arthrobacter sp. Hiyo4]
gi760164491	5.93	1	1	253	27.0	10.98	4.46	hypothetical protein [Arthrobacter crystallopoietes]
gi928486239	3.45	2	1	464	48.3	6.29	4.46	hypothetical protein AOC05_03205 [Arthrobacter alpinus]
gi786028522	3.47	1	1	490	51.7	4.96	4.46	diaminopimelate decarboxylase [Arthrobacter chlorophenolicus]
gi542109132	14.40	2	1	125	13.2	5.06	4.45	ArsR family transcriptional regulator [Arthrobacter sp. AK-YN10]
gi517599164	2.87	2	1	487	52.0	5.22	4.45	hypothetical protein [Arthrobacter sp. 162MFSha1.1]
gi470221254	6.06	1	1	297	32.6	5.41	4.45	pseudaminic acid synthase [Arthrobacter gangotriensis Lz1y]
gi930825394	9.85	3	1	132	13.8	5.80	4.45	hypothetical protein AOZ07_03145 [Arthrobacter arilaitensis]

gi737807785	3.70	1	1	486	51.8	4.56	4.45	hydrolase [Arthrobacter sp. H5]
gi119948356	4.20	1	1	405	44.2	5.66	4.45	putative glutaryl-CoA dehydrogenase [Arthrobacter aurescens TC1]
gi323468182	1.08	1	1	1664	176.3	5.86	4.45	ATP dependent helicase, Lhr family [Arthrobacter phenanthrenivorans Sphe3]
gi517606017	3.38	1	1	325	35.8	8.16	4.45	5-hydroxymethyluracil DNA glycosylase [Arthrobacter sp. 161MFSha2.1]
gi443482763	7.47	2	2	348	37.6	9.25	4.45	HNH nuclease [Arthrobacter nitrophenolicus]
gi323471213	3.46	1	1	491	51.9	5.31	4.45	carboxylesterase type B [Arthrobacter phenanthrenivorans Sphe3]
gi742072155	7.66	4	1	261	28.5	6.76	4.45	hypothetical protein ANMWB30_08590 [Arthrobacter sp. MWB30]
gi742072919	10.33	2	1	184	18.8	4.44	4.45	haloacid dehalogenase-like hydrolase [Arthrobacter sp. MWB30]
gi916820585	5.20	1	1	269	28.8	9.60	4.45	CAAX protease [Arthrobacter sp. H20]
gi937259184	6.87	3	1	233	24.8	5.03	4.44	cytidylate kinase [Arthrobacter sp. Edens01]
gi939036231	7.38	1	1	366	38.3	4.97	4.44	hypothetical protein [Arthrobacter nitroguajacolicus]
gi674646229	5.86	1	1	222	24.0	6.65	4.44	Ktr system potassium uptake protein A [Arthrobacter sp. 11W110_air]
gi786025523	7.48	1	1	214	23.2	6.93	4.44	protein-tyrosine phosphatase [Arthrobacter chlorophenolicus]
gi908699323	5.30	1	1	396	42.5	6.04	4.44	Zn-dependent alcohol dehydrogenase [Arthrobacter sp. RIT-PI-e]
gi742072618	6.19	1	1	226	25.2	5.67	4.44	GntR family transcriptional regulator [Arthrobacter sp. MWB30]
gi476401380	5.12	2	1	293	31.0	8.57	4.44	1,4-dihydroxy-2-naphthoate octaprenyltransferase [Arthrobacter crystallopoietes BAB-32]
gi767257080	4.57	1	1	372	40.1	5.26	4.44	ATP-dependent DNA helicase RuvB [Arthrobacter sp. IHBB 11108]
gi757624730	3.14	2	1	414	44.4	6.30	4.44	lipase/esterase [Arthrobacter sp. SPG23]
gi651458305	7.53	1	1	239	25.1	5.05	4.44	histidine kinase [Arthrobacter sp. 35/47]
gi928488141	4.70	1	1	404	43.8	9.89	4.44	hypothetical protein AOC05_16065 [Arthrobacter alpinus]
gi908698542	2.98	1	1	436	48.4	8.95	4.44	glycosyl transferase [Arthrobacter sp. RIT-PI-e]
gi517591906	6.20	1	1	242	25.7	5.86	4.44	L-ribulose-5-phosphate 4-epimerase [Arthrobacter sp. 135MFCol5.1]
gi651484709	4.48	1	1	268	29.7	10.14	4.43	methylase [Arthrobacter sp. Br18]
gi757624363	3.50	5	1	400	43.7	5.85	4.43	acyl-CoA dehydrogenase [Arthrobacter sp. SPG23]
gi908740250	4.64	1	1	237	25.2	5.85	4.43	hypothetical protein [Arthrobacter arilaitensis]
gi403228327	6.82	1	1	264	27.8	5.60	4.43	putative oxidoreductase [Arthrobacter sp. Rue61a]
gi651442741	4.33	1	1	624	66.1	6.62	4.43	sodium:proton antiporter [Arthrobacter sp. 9MFCol3.1]
gi654816140	7.01	3	1	314	31.4	9.64	4.43	lipase [Arthrobacter sp. UNC362MFTsu5.1]
gi928486178	2.76	2	1	907	98.1	6.02	4.43	hypothetical protein AOC05_02740 [Arthrobacter alpinus]
gi930825585	4.66	1	1	386	41.3	5.39	4.43	phosphoribosylaminoimidazole carboxylase [Arthrobacter arilaitensis]
gi517602246	7.52	1	1	306	31.4	7.08	4.43	methionyl-tRNA formyltransferase [Arthrobacter sp. 131MFCol6.1]
gi910697681	3.26	2	1	429	46.4	8.16	4.43	putative binding protein BRA0748/BS1330_II0741 [Arthrobacter sp. Hiyo6]
gi162953858	9.13	2	1	263	29.7	10.43	4.43	conserved hypothetical protein [Renibacterium salmoninarum ATCC 33209]
gi823666654	7.58	4	1	264	29.1	8.43	4.43	endonuclease III [Arthrobacter sp. YC-RL1]
gi918269053	6.27	2	1	255	26.4	4.94	4.43	glycosyl transferase, family 2 [Arthrobacter sp. Hiyo1]
gi640193382	3.67	1	1	354	38.0	5.86	4.43	SAM-dependent methyltransferase [Arthrobacter sp. 31Y]
gi723608246	9.73	1	1	257	26.1	8.72	4.43	hypothetical protein ART_2023 [Arthrobacter sp. PAMC25486]
gi359303679	2.99	1	1	536	56.7	5.81	4.43	putative IclR family transcriptional regulator [Arthrobacter globiformis NBRC 12137]
gi116609979	3.64	2	1	302	32.1	6.46	4.43	transcriptional regulator, LysR family [Arthrobacter sp. FB24]
gi651487005	4.95	2	1	222	23.2	5.22	4.43	hypothetical protein [Arthrobacter sp. Br18]
gi651435651	7.78	1	1	180	19.5	5.02	4.43	peptidylprolyl isomerase [Arthrobacter sp. H41]
gi674644661	1.78	2	1	897	95.0	6.64	4.42	Bacterial regulatory proteins, luxR family [Arthrobacter sp. 11W110_air]
gi651480838	7.62	1	1	210	23.1	6.11	4.42	NUDIX domain-containing protein [Arthrobacter sp. Br18]
gi323467676	2.58	1	1	621	65.3	5.26	4.42	dihydroxyacid dehydratase [Arthrobacter phenanthrenivorans Sphe3]
gi917441875	11.43	1	1	140	15.5	4.93	4.42	hypothetical protein [Arthrobacter albus]
gi476400726	4.51	1	1	288	30.7	4.98	4.42	nucleoside-diphosphate sugar epimerase [Arthrobacter crystallopoietes BAB-32]
gi359303710	4.83	2	1	269	28.8	5.15	4.42	oxidoreductase [Arthrobacter globiformis NBRC 12137]
gi651503276	2.49	3	1	563	60.6	5.74	4.42	peptide ABC transporter ATPase [Arthrobacter sp. 35W]
gi654824986	4.30	1	1	442	48.2	5.59	4.42	glucarate dehydratase [Arthrobacter sp. I3]
gi635353290	8.08	1	1	198	20.3	4.70	4.42	hypothetical protein ARTSIC4J27_633 [Arthrobacter siccitolerans]
gi930827282	3.59	1	1	502	55.6	6.60	4.42	hypothetical protein AOZ07_14010 [Arthrobacter arilaitensis]
gi517600313	12.90	1	1	155	16.7	7.69	4.42	hypothetical protein [Arthrobacter sp. 162MFSha1.1]
gi757625262	2.38	1	1	882	94.9	5.54	4.41	hypothetical protein TV39_05730 [Arthrobacter sp. SPG23]
gi651431178	15.70	2	1	121	13.0	10.43	4.41	50S ribosomal protein L24 [Arthrobacter sanguinis]
gi651445506	1.63	1	1	676	74.0	5.14	4.41	beta-galactosidase [Arthrobacter nicotinovorans]

gi916691333	2.11	2	1	711	76.2	8.02	4.41	primosomal protein N' [Arthrobacter castelli]
gi937261723	1.53	1	1	1045	109.3	4.54	4.41	hypothetical protein AO716_04925 [Arthrobacter sp. Edens01]
gi517603846	4.41	1	1	431	47.4	9.70	4.41	hypothetical protein [Arthrobacter sp. 131MFCol6.1]
gi916872012	2.98	2	1	570	61.1	4.55	4.41	beta-lactamase [Arthrobacter sp. H5]
gi737788785	5.69	1	1	281	29.9	5.12	4.41	aldolase [Arthrobacter albus]
gi910251499	5.02	1	1	259	27.9	6.24	4.41	amidohydrolase [Arthrobacter siccitolerans]
gi219859140	2.65	1	1	452	48.9	5.55	4.41	Peptidase M1 membrane alanine aminopeptidase [Arthrobacter chlorophenolicus A6]
gi908698688	2.56	1	1	429	47.8	6.67	4.41	transposase [Arthrobacter sp. RIT-PI-e]
gi651460740	6.43	1	1	373	39.2	9.35	4.41	FAD-binding monooxygenase [Arthrobacter sp. 35/47]
gi443481943	2.36	1	1	466	49.9	6.38	4.41	CdaR family transcriptional regulator [Arthrobacter nitrophenolicus]
gi916781622	2.81	1	1	462	47.3	5.25	4.41	hypothetical protein [Arthrobacter sp. 35W]
gi476401541	11.63	1	1	86	9.0	7.15	4.40	hypothetical protein D477_008083 [Arthrobacter crystallopoietes BAB-32]
gi162954532	4.92	3	1	305	32.9	7.59	4.40	dipeptide transport ATP-binding protein [Renibacterium salmoninarum ATCC 33209]
gi651447172	3.31	6	1	605	67.5	5.14	4.40	siderophore biosynthesis protein IucA [Arthrobacter nicotinovorans]
gi651501312	4.94	1	1	324	33.4	8.13	4.40	NADPH:quinone reductase [Arthrobacter sp. 35W]
gi917013346	8.89	1	1	225	24.2	9.69	4.40	hypothetical protein [Arthrobacter sanguinis]
gi134057804	3.53	1	1	368	39.7	6.13	4.40	unnamed protein product [Aspergillus niger]
gi742070640	5.28	1	1	265	28.9	8.63	4.40	glutamate transport ATP-binding protein GluA [Arthrobacter sp. MWB30]
gi654826513	3.50	2	1	457	48.4	5.08	4.40	serine ammonia-lyase [Arthrobacter sp. H5]
gi916834996	5.88	2	1	204	21.9	7.14	4.40	Holliday junction resolvase [Arthrobacter sp. H14]
gi767258512	15.34	1	1	163	18.0	5.30	4.40	hypothetical protein UM93_14445 [Arthrobacter sp. IHBB 11108]
gi823665760	1.76	2	1	569	63.4	5.29	4.40	AMP-dependent synthetase [Arthrobacter sp. YC-RL1]
gi119950375	11.41	1	1	149	16.1	6.10	4.40	putative histidine triad protein (HIT domain) [Arthrobacter aurescens TC1]
gi737773928	3.71	1	1	404	42.4	5.60	4.40	pyridine nucleotide-disulfide oxidoreductase [Arthrobacter sp. MA-N2]
gi651506398	8.12	1	1	197	20.6	5.91	4.40	hypothetical protein [Arthrobacter sp. 35W]
gi916692000	3.67	2	1	327	36.5	5.57	4.39	aminoglycoside resistance protein [Arthrobacter castelli]
gi651429136	2.06	1	1	534	58.3	5.78	4.39	hypothetical protein [Arthrobacter sanguinis]
gi930825730	1.66	1	1	782	87.3	5.11	4.39	transcriptional regulator [Arthrobacter arilaitensis]
gi737788102	3.30	1	1	303	33.3	5.44	4.39	N-acetylneuraminate synthase [Arthrobacter albus]
gi651434716	3.08	1	1	357	40.8	7.44	4.39	methicillin resistance protein [Arthrobacter sp. H41]
gi648572614	12.12	2	1	165	17.8	5.02	4.39	OHCU decarboxylase [Arthrobacter sp. 135MFCol5.1]
gi515766339	5.57	2	1	305	34.1	4.78	4.39	myo-inosose-2 dehydratase [Arthrobacter sp. M2012083]
gi918267481	2.20	2	1	635	68.9	6.20	4.39	alpha-L-Rhamnosidase [Arthrobacter sp. Hiyo1]
gi162953574	25.29	1	1	87	9.3	9.99	4.39	conserved hypothetical protein [Renibacterium salmoninarum ATCC 33209]
gi651441390	6.07	1	1	214	22.6	9.95	4.38	hypothetical protein [Arthrobacter sp. 9MFCol3.1]
gi918269321	10.74	1	1	121	13.5	7.28	4.38	nucleoid-associated protein Lsr2 [Arthrobacter sp. Hiyo1]
gi551255993	1.84	1	1	488	50.5	5.27	4.38	ferredoxin [Arthrobacter sp. PAO19]
gi928487887	2.96	10	1	575	60.1	10.65	4.38	DEAD/DEAH box helicase [Arthrobacter alpinus]
gi651444419	4.18	1	1	239	23.1	5.24	4.38	hypothetical protein [Arthrobacter nicotinovorans]
gi651434286	4.96	2	1	282	30.0	5.11	4.38	pyridoxal biosynthesis lyase PdxS [Arthrobacter sp. H41]
gi517608157	5.03	1	1	358	38.8	6.18	4.38	glycosyl transferase [Arthrobacter sp. 161MFSha2.1]
gi170783590	2.36	1	1	593	64.4	9.31	4.38	conjugative transfer gene complex protein (plasmid) [Arthrobacter sp. Chr15]
gi937258457	2.86	1	1	384	40.6	5.07	4.38	cystathionine gamma-synthase [Arthrobacter sp. Edens01]
gi737796401	3.83	1	1	287	30.8	6.23	4.38	cystathionine gamma-synthase, partial [Arthrobacter sp. H20]
gi910740029	6.85	1	1	292	30.9	5.34	4.38	hypothetical protein AHiyo4_28510 [Arthrobacter sp. Hiyo4]
gi910694427	9.74	2	1	154	15.9	7.81	4.38	hypothetical protein AHiyo6_26480 [Arthrobacter sp. Hiyo6]
gi359306602	3.45	1	1	348	38.8	5.26	4.38	5-methyltetrahydropteroyltriglutamate--homocysteine methyltransferase [Arthrobacter globif
gi928487129	3.81	3	1	341	37.6	9.51	4.38	hypothetical protein AOC05_09320 [Arthrobacter alpinus]
gi737813246	4.20	1	1	357	36.9	5.54	4.38	hypothetical protein [Arthrobacter sp. H14]
gi443480594	4.39	2	1	296	31.5	11.97	4.38	RNA-binding protein, partial [Arthrobacter nitrophenolicus]
gi910696390	6.21	1	1	161	17.4	4.56	4.37	acetolactate synthase [Arthrobacter sp. Hiyo6]
gi765007653	2.19	2	1	776	84.3	5.85	4.37	hypothetical protein [Arthrobacter sp. A3]
gi765007054	9.13	1	1	230	25.1	6.11	4.37	DNA-binding response regulator [Arthrobacter sp. A3]
gi742856826	8.02	1	1	212	23.1	9.26	4.37	hypothetical protein [Arthrobacter sp. W1]
gi651440115	6.82	1	1	264	29.5	7.08	4.37	ABC transporter ATP-binding protein [Arthrobacter sp. H14]

gi928488726	2.60	1	1	654	71.3	4.94	4.37	peptidase M13 [Arthrobacter alpinus]
gi652425501	6.37	1	1	251	27.6	6.81	4.37	GntR family transcriptional regulator [Arthrobacter castelli]
gi307744936	15.12	1	1	86	9.4	10.93	4.37	30S ribosomal protein S20 [Arthrobacter arilaitensis Re117]
gi914714321	3.52	1	1	398	43.0	6.39	4.36	pyridine nucleotide-disulfide oxidoreductase [Arthrobacter sp. ZBG10]
gi470220946	1.22	1	1	1065	111.6	5.17	4.36	SMC domain-containing protein [Arthrobacter gangotriensis Lz1y]
gi742851338	3.09	1	1	453	47.7	6.48	4.36	adenosylmethionine--8-amino-7-oxononanoate aminotransferase BioA [Arthrobacter sp. W1]
gi470216257	2.56	3	1	703	74.8	5.02	4.36	transketolase [Arthrobacter gangotriensis Lz1y]
gi765013344	1.21	1	1	829	83.9	6.06	4.36	hypothetical protein [Arthrobacter sp. A3]
gi640203182	5.25	1	1	343	37.9	5.50	4.36	hypothetical protein [Arthrobacter sp. 31Y]
gi916816421	4.89	3	1	368	39.0	6.20	4.36	hypothetical protein [Arthrobacter sp. MA-N2]
gi910743902	8.61	1	1	151	16.0	9.42	4.36	acetyl-/propionyl-coenzyme A carboxylase alpha chain [Arthrobacter sp. Hiyo8]
gi742858238	4.67	1	1	300	32.8	5.58	4.36	tRNA dimethylallyltransferase [Arthrobacter sp. W1]
gi927295829	2.77	1	1	614	65.7	8.69	4.36	ABC transporter [Arthrobacter sp. ERGS1:01]
gi919219009	3.12	1	1	353	40.3	8.53	4.36	MULTISPECIES: hypothetical protein [Arthrobacter]
gi651439384	3.31	1	1	544	57.4	5.34	4.36	UDP-N-acetylmuramoylalanyl-D-glutamate--2, 6-diaminopimelate ligase [Arthrobacter sp. H1
gi742755210	2.23	1	1	763	86.5	5.82	4.36	hypothetical protein RM50_14990 [Arthrobacter phenanthrenivorans]
gi723608499	8.89	1	1	90	10.4	9.79	4.36	hypothetical protein ART_2276 [Arthrobacter sp. PAMC25486]
gi162953087	9.71	1	1	103	11.3	11.34	4.36	hypothetical protein RSal33209_0858 [Renibacterium salmoninarum ATCC 33209]
gi767257750	2.62	1	1	535	57.0	5.08	4.36	GMP synthase [Arthrobacter sp. IHBB 11108]
gi648574473	6.27	2	1	335	34.3	7.64	4.35	LacI family transcriptional regulator [Arthrobacter sp. 162MFSha1.1]
gi749401654	1.87	1	1	427	45.7	5.68	4.35	histidine kinase [Arthrobacter sp. AK-YN10]
gi937262279	8.88	5	1	214	22.4	6.58	4.35	methyltransferase [Arthrobacter sp. Edens01]
gi636846236	12.81	1	1	203	21.1	7.34	4.35	TetR family transcriptional regulator [Arthrobacter sp. TB 26]
gi939050628	15.04	3	1	113	11.7	10.37	4.35	hypothetical protein [Arthrobacter sp. JCM 19049]
gi737808227	4.14	1	1	411	43.5	5.21	4.35	aspartate aminotransferase [Arthrobacter sp. H5]
gi323471533	4.10	1	1	390	42.9	5.39	4.35	MoxR-like ATPase (plasmid) [Arthrobacter phenanthrenivorans Sphe3]
gi760164811	9.02	10	1	122	13.4	6.51	4.35	hypothetical protein [Arthrobacter crystallopoietes]
gi910250633	9.93	1	1	282	30.4	6.71	4.35	hypothetical protein [Arthrobacter siccitolerans]
gi307743239	2.99	1	1	569	61.9	9.22	4.35	mobilisation protein (plasmid) [Arthrobacter arilaitensis Re117]
gi323468942	4.30	1	1	372	40.0	5.20	4.35	histidinol phosphate aminotransferase apoenzyme [Arthrobacter phenanthrenivorans Sphe3]
gi908697281	1.87	1	1	482	51.5	4.93	4.35	hypothetical protein [Arthrobacter sp. RIT-PI-e]
gi930824979	1.82	1	1	494	51.8	9.52	4.35	amino acid permease [Arthrobacter arilaitensis]
gi551256780	1.83	1	1	493	51.7	9.42	4.35	amino acid permease [Arthrobacter sp. PAO19]
gi742756624	10.22	1	1	137	15.8	8.72	4.35	hypothetical protein RM50_09335 [Arthrobacter phenanthrenivorans]
gi767259053	2.46	2	1	529	57.6	6.44	4.35	ABC transporter substrate-binding protein [Arthrobacter sp. IHBB 11108]
gi359307508	15.93	2	1	113	11.8	10.65	4.35	hypothetical protein ARGLB_013_00040 [Arthrobacter globiformis NBRC 12137]
gi765006278	10.36	2	1	222	23.2	6.80	4.35	hypothetical protein [Arthrobacter sp. A3]
gi518313565	1.82	1	1	658	70.7	9.88	4.35	MULTISPECIES: hypothetical protein [Arthrobacter]
gi518312673	5.71	1	1	385	42.0	5.26	4.34	hypothetical protein [Arthrobacter sp. TB 23]
gi162954821	15.05	1	1	93	10.6	6.95	4.34	sulfate transport ATP-binding protein [Renibacterium salmoninarum ATCC 33209]
gi939036741	6.75	1	1	237	24.5	5.21	4.34	short-chain dehydrogenase [Arthrobacter nitroguajacolicus]
gi917760016	4.19	1	1	477	46.6	4.86	4.34	hypothetical protein [Arthrobacter sp. L77]
gi162952304	13.55	1	1	155	16.0	5.76	4.34	transcriptional regulator, AraC family [Renibacterium salmoninarum ATCC 33209]
gi651493690	2.15	1	1	511	54.4	5.57	4.34	sugar ABC transporter ATPase [Arthrobacter sp. H20]
gi674645034	1.05	1	1	1147	118.6	6.61	4.34	ATP-binding/permease protein CydD [Arthrobacter sp. 11W110_air]
gi690772417	8.59	3	1	198	22.3	5.71	4.34	hypothetical protein HMPREF2128_08265 [Arthrobacter albus DNF00011]
gi757624992	8.50	3	1	247	27.3	6.02	4.34	chemotaxis protein CheY [Arthrobacter sp. SPG23]
gi930825243	13.51	2	1	148	16.5	6.15	4.34	thioesterase [Arthrobacter arilaitensis]
gi916815807	9.92	2	1	131	14.4	9.32	4.34	hypothetical protein [Arthrobacter sp. MA-N2]
gi116611942	2.78	1	1	576	61.8	6.57	4.33	ResB family protein [Arthrobacter sp. FB24]
gi916782278	6.28	1	1	366	40.5	5.78	4.33	hypothetical protein [Arthrobacter sp. 35W]
gi927295608	2.80	1	1	322	35.2	8.31	4.33	NUDIX hydrolase [Arthrobacter sp. ERGS1:01]
gi470220289	9.60	1	1	250	25.9	6.64	4.33	3-oxoacyl-(acyl-carrier-protein) reductase [Arthrobacter gangotriensis Lz1y]
gi753934206	5.90	1	1	356	38.6	9.94	4.33	transposase [Arthrobacter arilaitensis]
gi443482393	1.86	1	1	485	51.0	8.69	4.33	phytoene dehydrogenase-like oxidoreductase [Arthrobacter nitrophenolicus]

gi640203143	8.24	1	1	255	26.4	6.01	4.33	short-chain dehydrogenase [Arthrobacter sp. 31Y]
gi651480018	3.23	1	1	279	30.0	5.47	4.33	hypothetical protein [Arthrobacter sp. Br18]
gi786030719	5.21	2	1	365	39.2	5.38	4.33	lipoate--protein ligase [Arthrobacter chlorophenolicus]
gi939036376	19.70	1	1	66	7.2	9.14	4.33	hypothetical protein [Arthrobacter nitroguajacolicus]
gi639131099	10.75	1	1	186	21.1	10.33	4.33	integrase, partial [Arthrobacter sp. CAL618]
gi910250276	1.93	1	1	776	84.0	4.94	4.33	5-methyltetrahydropteroyltriglutamate--homocysteine methyltransferase [Arthrobacter siccito
gi759747291	2.11	1	1	617	66.5	5.94	4.33	NADH oxidase [Arthrobacter sp. 31Y]
gi737780214	1.75	2	1	514	56.4	6.13	4.32	MULTISPECIES: ABC transporter ATP-binding protein [Arthrobacter]
gi116611873	4.54	1	1	529	55.1	5.19	4.32	lipopolysaccharide biosynthesis [Arthrobacter sp. FB24]
gi116611833	2.66	1	1	638	65.0	10.15	4.32	O-antigen polymerase [Arthrobacter sp. FB24]
gi651430677	3.35	1	1	657	70.7	9.74	4.32	DNA-binding protein [Arthrobacter sanguinis]
gi648575211	4.56	1	1	329	35.1	5.52	4.32	oxidoreductase [Arthrobacter sp. 161MFSha2.1]
gi470217495	7.87	2	1	178	18.4	6.52	4.32	thioredoxin reductase [Arthrobacter gangotriensis Lz1y]
gi651450573	4.58	1	1	262	28.3	6.74	4.32	esterase [Arthrobacter nicotinovorans]
gi651498996	3.03	2	1	363	38.7	9.98	4.32	pseudouridine synthase [Arthrobacter sp. 35W]
gi517590499	9.54	5	1	304	32.9	5.43	4.32	LysR family transcriptional regulator [Arthrobacter sp. 135MFCol5.1]
gi476401491	10.09	2	1	109	12.8	10.95	4.31	ATP/GTP-binding protein [Arthrobacter crystallopoietes BAB-32]
gi916290029	4.62	1	1	390	43.3	5.68	4.31	cytochrome [Arthrobacter sp. Rue61a]
gi636844557	7.31	2	1	219	22.9	6.79	4.31	hypothetical protein, partial [Arthrobacter sp. TB 26]
gi651429194	2.81	1	1	498	53.0	9.07	4.31	multidrug MFS transporter [Arthrobacter sanguinis]
gi651444469	1.83	2	1	1145	116.4	7.52	4.31	ABC transporter [Arthrobacter nicotinovorans]
gi916820459	1.34	1	1	820	90.0	6.98	4.31	hypothetical protein [Arthrobacter sp. H20]
gi639130031	2.87	3	1	663	71.1	5.12	4.31	propionyl-CoA synthetase [Arthrobacter sp. CAL618]
gi654811626	6.79	1	1	280	29.5	7.50	4.31	hypothetical protein [Arthrobacter sp. MA-N2]
gi930826827	3.16	1	1	570	64.2	6.62	4.31	hypothetical protein AOZ07_11425 [Arthrobacter arilaitensis]
gi674645649	8.38	1	1	167	18.5	5.59	4.31	hypothetical protein BN1051_01842 [Arthrobacter sp. 11W110_air]
gi654823421	3.35	1	1	448	46.8	4.83	4.31	beta-Ala-His dipeptidase [Arthrobacter sp. I3]
gi674644475	3.40	2	1	353	36.4	5.87	4.31	Lipopolysaccharide core heptosyltransferase RfaQ [Arthrobacter sp. 11W110_air]
gi767257683	3.04	1	1	427	47.4	6.16	4.31	hypothetical protein UM93_08680 [Arthrobacter sp. IHBB 11108]
gi517598384	4.31	1	1	418	45.7	9.80	4.30	membrane protein [Arthrobacter sp. 162MFSha1.1]
gi910251202	5.39	1	1	371	39.9	5.16	4.30	homoserine acetyltransferase [Arthrobacter siccitolerans]
gi162953027	2.13	1	1	423	45.5	10.07	4.30	DNA methylation and regulatory protein ADA [Renibacterium salmoninarum ATCC 33209]
gi651503806	2.17	2	1	414	44.7	8.40	4.30	exodeoxyribonuclease VII large subunit [Arthrobacter sp. 35W]
gi757623582	5.28	1	1	341	36.1	6.24	4.30	6-phosphofructokinase [Arthrobacter sp. SPG23]
gi403231642	1.57	1	1	893	93.3	4.98	4.30	FG-GAP repeat domain protein [Arthrobacter sp. Rue61a]
gi443481707	4.88	1	1	246	26.0	8.28	4.30	ANTAR domain-containing protein [Arthrobacter nitrophenolicus]
gi517609560	4.22	1	1	308	32.7	5.81	4.30	hypothetical protein [Arthrobacter sp. 161MFSha2.1]
gi323467686	2.99	1	1	368	42.5	6.87	4.30	uncharacterized conserved protein [Arthrobacter phenanthrenivorans Sphe3]
gi551254329	6.69	1	1	254	26.6	6.96	4.29	3-oxoacyl-ACP reductase [Arthrobacter sp. PAO19]
gi359304096	2.30	1	1	870	93.1	6.16	4.29	putative non-ribosomal peptide synthetase [Arthrobacter globiformis NBRC 12137]
gi517601608	5.84	1	1	257	27.1	5.85	4.29	hypothetical protein [Arthrobacter sp. 162MFSha1.1]
gi753931795	11.71	1	1	111	12.4	9.44	4.29	hypothetical protein [Arthrobacter arilaitensis]
gi651439464	3.22	2	1	311	34.4	9.74	4.29	hypothetical protein [Arthrobacter sp. H14]
gi916290060	3.88	1	1	309	33.1	5.71	4.29	MULTISPECIES: AAA family ATPase [Arthrobacter]
gi518311603	3.92	1	1	434	47.5	5.07	4.29	MULTISPECIES: fuconate dehydratase [Arthrobacter]
gi767259187	5.77	2	1	312	34.4	9.79	4.29	ABC transporter permease [Arthrobacter sp. IHBB 11108]
gi654818827	1.75	1	1	857	90.8	6.13	4.29	ABC transporter [Arthrobacter sp. UNC362MFTsu5.1]
gi927295811	1.82	1	1	658	71.6	5.29	4.29	acetyl-CoA synthetase [Arthrobacter sp. ERGS1:01]
gi323470123	3.36	1	1	446	48.2	8.18	4.29	Exodeoxyribonuclease VII large subunit [Arthrobacter phenanthrenivorans Sphe3]
gi517608510	4.73	3	1	528	55.2	6.43	4.29	hypothetical protein [Arthrobacter sp. 161MFSha2.1]
gi654812113	5.38	1	1	279	28.4	9.33	4.29	lipase [Arthrobacter sp. MA-N2]
gi760112268	5.23	3	1	421	46.2	5.30	4.29	carbohydrate-binding protein [Arthrobacter chlorophenolicus]
gi759711176	13.29	1	1	173	18.5	6.80	4.28	GNAT family acetyltransferase [Arthrobacter sp. 135MFCol5.1]
gi518311814	2.71	1	1	369	39.4	6.64	4.28	hypothetical protein [Arthrobacter sp. TB 23]
gi651439776	4.70	1	1	234	25.8	5.87	4.28	hypothetical protein [Arthrobacter sp. H14]

gi651435946	3.46	1	1	549	59.5	5.38	4.28	arginine--tRNA ligase [Arthrobacter sp. H41]
gi916863518	2.58	1	1	426	45.5	11.28	4.28	MFS transporter [Arthrobacter sp. 35/47]
gi515766294	2.68	1	1	895	94.6	5.76	4.28	GCN5 family acetyltransferase [Arthrobacter sp. M2012083]
gi928487775	4.16	1	1	385	41.3	4.91	4.28	pyruvate dehydrogenase [Arthrobacter alpinus]
gi917759797	2.61	1	1	575	61.5	6.40	4.28	hypothetical protein [Arthrobacter sp. L77]
gi742754086	2.33	1	1	686	71.9	5.49	4.28	ATP-dependent helicase [Arthrobacter phenanthrenivorans]
gi917739313	9.43	1	1	106	11.8	8.34	4.28	hypothetical protein [Arthrobacter sp. W1]
gi635353604	1.93	1	1	571	63.1	9.01	4.28	hypothetical protein ARTSIC4J27_349 [Arthrobacter siccitolerans]
gi927293773	4.09	2	1	513	53.3	6.46	4.28	carboxylesterase [Arthrobacter sp. ERGS1:01]
gi651454586	1.60	1	1	689	73.4	5.25	4.27	anthranilate synthase [Arthrobacter nicotinovorans]
gi323467918	9.84	1	1	183	19.5	6.54	4.27	transcriptional regulator, TetR family [Arthrobacter phenanthrenivorans Sphe3]
gi767256868	1.28	2	1	1174	126.7	5.34	4.27	peptidase S41 [Arthrobacter sp. IHBB 11108]
gi518310948	2.64	1	1	417	45.7	7.12	4.27	transposase [Arthrobacter sp. TB 23]
gi927295219	1.41	1	1	1626	180.5	5.14	4.27	glutamate dehydrogenase [Arthrobacter sp. ERGS1:01]
gi470221523	8.79	1	1	239	25.8	7.15	4.27	IclR family transcriptional regulator [Arthrobacter gangotriensis Lz1y]
gi517589918	2.25	1	1	622	67.2	6.76	4.27	hypothetical protein [Arthrobacter sp. 135MFCol5.1]
gi518311982	3.04	1	1	560	59.1	10.76	4.27	DEAD/DEAH box helicase [Arthrobacter sp. TB 23]
gi908698780	5.86	1	1	273	28.3	4.96	4.27	hypothetical protein, partial [Arthrobacter sp. RIT-PI-e]
gi636845038	3.39	1	1	443	48.0	10.13	4.27	hypothetical protein [Arthrobacter sp. TB 26]
gi916926338	4.13	1	1	508	53.2	9.48	4.26	HNH endonuclease [Arthrobacter sp. 9MFCol3.1]
gi307745674	4.44	1	1	360	38.7	5.35	4.26	2-oxoacid dehydrogenase E1 component beta chain [Arthrobacter arilaitensis Re117]
gi476402537	5.11	1	1	372	40.8	7.84	4.26	glycosyl transferase group 1 [Arthrobacter crystallopoietes BAB-32]
gi551255228	4.02	1	1	224	24.1	4.86	4.26	DNA-binding response regulator [Arthrobacter sp. PAO19]
gi939050744	13.16	2	1	152	17.2	6.57	4.26	hypothetical protein [Arthrobacter sp. JCM 19049]
gi918265159	28.00	1	1	75	8.4	9.64	4.26	hypothetical protein AHiyo1_50850 [Arthrobacter sp. Hiyo1]
gi470217010	12.87	1	1	101	11.0	9.98	4.26	regulatory protein ArsR [Arthrobacter gangotriensis Lz1y]
gi651429342	1.39	2	1	717	80.0	4.68	4.26	protease 2 [Arthrobacter sanguinis]
gi737782040	2.95	1	1	407	41.7	6.00	4.26	phosphopantothenoylcysteine decarboxylase [Arthrobacter sp. 35W]
gi767257611	2.12	1	1	520	53.9	5.10	4.26	glutamyl-tRNA amidotransferase [Arthrobacter sp. IHBB 11108]
gi737800382	3.77	1	1	398	41.7	9.64	4.26	membrane protein [Arthrobacter castelli]
gi917442286	5.06	2	1	356	38.3	5.67	4.26	NAD kinase [Arthrobacter albus]
gi908690513	6.19	1	1	323	33.8	7.65	4.26	dehydrogenase, partial [Arthrobacter sp. H41]
gi823667357	12.10	2	1	157	16.2	11.60	4.26	hypothetical protein AA310_17190 [Arthrobacter sp. YC-RL1]
gi359306351	3.74	1	1	428	46.0	10.21	4.25	hypothetical protein ARGLB_039_00620 [Arthrobacter globiformis NBRC 12137]
gi476400874	2.29	1	1	436	46.6	6.57	4.25	geranylgeranyl reductase [Arthrobacter crystallopoietes BAB-32]
gi767258650	8.22	1	1	304	32.5	5.03	4.25	arginase [Arthrobacter sp. IHBB 11108]
gi518311944	5.62	2	1	338	36.7	5.15	4.25	hypothetical protein [Arthrobacter sp. TB 23]
gi443483077	1.66	1	1	663	67.4	5.16	4.25	chromosome segregation ATPase-like protein [Arthrobacter nitrophenolicus]
gi651431360	7.46	1	1	295	30.6	5.27	4.25	bifunctional 5,10-methylene-tetrahydrofolate dehydrogenase/5,10-methylene-tetrahydrofolat
gi927293693	6.57	2	1	274	30.3	8.79	4.25	hypothetical protein AL755_06035 [Arthrobacter sp. ERGS1:01]
gi723607056	7.48	2	1	254	27.7	9.45	4.25	hypothetical protein ART_0833 [Arthrobacter sp. PAMC25486]
gi786025592	6.10	1	1	328	33.6	6.81	4.25	two-component system sensor histidine kinase [Arthrobacter chlorophenolicus]
gi219858082	6.87	1	1	335	35.3	8.68	4.25	oligopeptide/dipeptide ABC transporter, ATPase subunit [Arthrobacter chlorophenolicus A6]
gi410689798	53.85	1	1	39	4.4	11.06	4.25	hypothetical protein (plasmid) [Arthrobacter sp. J3-40]
gi910692929	4.05	2	1	222	23.3	5.78	4.25	alcohol dehydrogenase, partial [Arthrobacter sp. Hiyo6]
gi652422610	5.88	2	1	272	28.9	6.33	4.25	ATP synthase subunit delta [Arthrobacter castelli]
gi737807755	4.86	1	1	288	30.3	5.97	4.25	hypothetical protein, partial [Arthrobacter sp. H5]
gi654822455	5.31	3	1	320	33.1	5.14	4.25	FAD-binding molybdopterin dehydrogenase [Arthrobacter sp. I3]
gi517599066	8.11	1	1	259	27.0	6.70	4.25	16S rRNA methyltransferase [Arthrobacter sp. 162MFSha1.1]
gi930828265	3.24	1	1	247	26.5	4.82	4.25	glutamine amidotransferase [Arthrobacter arilaitensis]
gi737777033	2.67	2	1	825	86.2	4.81	4.24	DNA polymerase III subunit gamma/tau [Arthrobacter sanguinis]
gi654818399	9.86	1	1	213	22.9	6.16	4.24	GntR family transcriptional regulator [Arthrobacter sp. UNC362MFTsu5.1]
gi914715064	5.64	1	1	319	33.5	5.29	4.24	D-glycerate dehydrogenase [Arthrobacter sp. ZBG10]
gi740685326	2.78	1	1	540	56.1	8.54	4.24	hypothetical protein [Arthrobacter sp. PAMC25486]
gi908698730	13.48	1	1	141	15.0	5.81	4.24	hypothetical protein [Arthrobacter sp. RIT-PI-e]

gi359303909	4.20	2	1	333	32.9	5.03	4.24	glycerate kinase [Arthrobacter globiformis NBRC 12137]
gi737789565	1.90	1	1	684	73.6	9.17	4.24	hypothetical protein [Arthrobacter albus]
gi219858589	7.46	1	1	134	14.6	5.08	4.24	4-oxalocrotonate tautomerase [Arthrobacter chlorophenolicus A6]
gi928486427	2.32	1	1	604	64.8	6.84	4.24	long-chain fatty acid--CoA ligase [Arthrobacter alpinus]
gi767257233	3.70	1	1	378	41.3	8.54	4.24	SAM-dependent methyltransferase [Arthrobacter sp. IHBB 11108]
gi937258530	18.49	1	1	119	13.6	10.32	4.24	50S ribosomal protein L19 [Arthrobacter sp. Edens01]
gi651464150	4.96	1	1	484	51.3	7.17	4.23	FAD-dependent oxidoreductase [Arthrobacter sp. 35/47]
gi916359192	7.74	1	1	297	30.7	6.11	4.23	glycosyl transferase family 1 [Arthrobacter sp. 135MFCol5.1]
gi928485944	3.63	1	1	413	43.9	6.35	4.23	acetyl-CoA acetyltransferase [Arthrobacter alpinus]
gi470217563	2.95	1	1	407	44.4	4.92	4.23	Recombinational DNA repair ATPase (RecF pathway) [Arthrobacter gangotriensis Lz1y]
gi916871567	5.65	2	1	230	24.8	7.50	4.23	ABC transporter ATP-binding protein [Arthrobacter sp. H5]
gi757625652	2.42	1	1	413	42.3	4.77	4.23	acetylornithine deacetylase [Arthrobacter sp. SPG23]
gi443481490	3.90	1	1	487	54.5	5.73	4.23	alkaline phosphatase [Arthrobacter nitrophenolicus]
gi767258852	5.82	1	1	275	30.6	5.55	4.23	exodeoxyribonuclease III [Arthrobacter sp. IHBB 11108]
gi651452222	5.32	1	1	451	46.0	4.97	4.23	amino acid ABC transporter substrate-binding protein [Arthrobacter nicotinovorans]
gi674646316	2.88	2	1	626	66.0	4.88	4.23	Thiol-disulfide oxidoreductase YkuV [Arthrobacter sp. 11W110_air]
gi786025635	8.08	1	1	260	28.8	7.99	4.23	hypothetical protein [Arthrobacter chlorophenolicus]
gi915933806	2.18	1	1	596	62.5	6.67	4.23	multidrug ABC transporter ATPase [Arthrobacter globiformis]
gi917441798	1.99	1	1	553	58.2	5.01	4.22	hypothetical protein [Arthrobacter albus]
gi910740462	3.72	1	1	296	32.2	6.05	4.22	periplasmic oligopeptide-binding protein [Arthrobacter sp. Hiyo4]
gi927293763	1.12	1	1	2052	210.9	5.30	4.22	hypothetical protein AL755_06530 [Arthrobacter sp. ERGS1:01]
gi910252063	3.02	2	1	298	33.4	7.30	4.22	sugar ABC transporter permease [Arthrobacter siccitolerans]
gi910748329	8.16	1	1	196	20.9	4.58	4.22	aminopeptidase N [Arthrobacter sp. Hiyo8]
gi767259252	7.14	3	1	182	19.2	5.54	4.22	hypothetical protein UM93_13230 [Arthrobacter sp. IHBB 11108]
gi219859141	0.76	1	1	1316	137.7	8.66	4.22	non-ribosomal peptide synthetase [Arthrobacter chlorophenolicus A6]
gi651495243	6.36	2	1	220	23.6	6.70	4.22	hypothetical protein [Arthrobacter sp. H20]
gi551253735	7.05	3	1	298	32.2	9.89	4.22	glutamate ABC transporter permease [Arthrobacter sp. PAO19]
gi916870254	6.13	2	1	326	33.6	6.54	4.22	homoserine kinase [Arthrobacter sp. Br18]
gi737780728	5.00	1	1	460	50.6	9.42	4.22	mRNA 3'-end processing factor [Arthrobacter sp. CAL618]
gi517602605	2.81	1	1	427	45.5	10.74	4.22	hypothetical protein [Arthrobacter sp. 131MFCol6.1]
gi162955046	4.39	2	1	342	36.6	5.48	4.21	NADH-dependent dehydrogenase [Renibacterium salmoninarum ATCC 33209]
gi651439277	2.22	2	1	451	48.1	4.84	4.21	MBL fold metallo-hydrolase [Arthrobacter sp. H14]
gi470220410	6.38	1	1	329	35.0	4.50	4.21	Iron(III) dicitrate-binding protein [Arthrobacter gangotriensis Lz1y]
gi640197052	1.06	1	1	663	72.4	6.20	4.21	hypothetical protein [Arthrobacter sp. 31Y]
gi910252452	2.58	1	1	621	65.1	5.21	4.21	dihydroxy-acid dehydratase [Arthrobacter siccitolerans]
gi757625571	3.01	1	1	432	46.5	6.81	4.21	histidine kinase [Arthrobacter sp. SPG23]
gi916834934	6.67	1	1	210	23.2	10.45	4.21	hypothetical protein [Arthrobacter sp. H14]
gi742755834	2.41	1	1	415	44.9	9.67	4.21	DNA polymerase IV [Arthrobacter phenanthrenivorans]
gi930826241	6.21	1	1	338	37.0	7.09	4.21	hypothetical protein AOZ07_08115 [Arthrobacter arilaitensis]
gi476402983	4.11	1	1	341	34.5	7.66	4.21	porphyrin biosynthesis protein HemD [Arthrobacter crystallopoietes BAB-32]
gi219858116	14.13	1	1	184	19.7	10.15	4.21	conserved hypothetical protein [Arthrobacter chlorophenolicus A6]
gi914716013	5.49	1	1	273	27.9	8.24	4.20	hypothetical protein [Arthrobacter sp. ZBG10]
gi515767124	4.48	1	1	268	28.2	4.65	4.20	hypothetical protein [Arthrobacter sp. M2012083]
gi910737996	8.39	1	1	298	33.4	9.33	4.20	glycogen phosphorylase [Arthrobacter sp. Hiyo4]
gi765012847	2.80	2	1	608	62.4	8.72	4.20	hypothetical protein [Arthrobacter sp. A3]
gi916863688	6.11	1	1	393	41.2	9.09	4.20	hypothetical protein [Arthrobacter sp. 35/47]
gi470217836	3.08	1	1	292	29.9	6.87	4.20	Universal stress protein family protein [Arthrobacter gangotriensis Lz1y]
gi765013389	7.95	1	1	239	25.8	7.55	4.20	hypothetical protein [Arthrobacter sp. A3]
gi928485804	2.43	1	1	493	55.4	6.23	4.19	trehalose-phosphate synthase [Arthrobacter alpinus]
gi908698923	1.53	1	1	524	58.2	5.03	4.19	diguanylate phosphodiesterase [Arthrobacter sp. RIT-PI-e]
gi323468336	1.57	2	1	827	88.0	7.08	4.19	hypothetical protein Asphe3_08230 [Arthrobacter phenanthrenivorans Sphe3]
gi635351184	5.33	1	1	244	29.9	13.16	4.19	hypothetical protein ARTSIC4J27_2892 [Arthrobacter siccitolerans]
gi517608723	5.70	4	1	263	27.6	5.14	4.19	D-beta-D-heptose 1-phosphate adenosyltransferase [Arthrobacter sp. 161MFSha2.1]
gi910250391	5.17	4	1	290	29.5	5.33	4.19	orotidine 5'-phosphate decarboxylase [Arthrobacter siccitolerans]
gi910737739	2.97	1	1	404	42.4	10.21	4.19	glutathione transport system permease protein GsiD [Arthrobacter sp. Hiyo4]

gi910740853	3.43	1	1	495	55.4	5.67	4.19	DNA-directed RNA polymerase subunit beta [Arthrobacter sp. Hiyo4]
gi545108917	5.41	1	1	370	39.4	6.47	4.19	hypothetical protein [Arthrobacter sp. AK-YN10]
gi906446250	1.25	1	1	881	95.3	5.96	4.19	hypothetical protein AC792_10360 [Arthrobacter sp. RIT-PI-e]
gi470217266	1.21	1	1	912	101.8	5.48	4.19	preprotein translocase subunit SecA [Arthrobacter gangotriensis Lz1y]
gi767258478	6.76	3	1	296	30.2	5.30	4.19	NADH-ubiquinone oxidoreductase [Arthrobacter sp. IHBB 11108]
gi914713872	5.94	1	1	421	45.7	5.60	4.18	XRE family transcriptional regulator [Arthrobacter sp. ZBG10]
gi908696786	7.19	1	1	292	32.9	9.80	4.18	transposase [Arthrobacter sp. RIT-PI-e]
gi648574562	2.93	2	1	375	40.0	5.40	4.18	XshC-Cox1 family protein [Arthrobacter sp. 131MFCol6.1]
gi651498146	2.04	1	1	1027	106.1	5.29	4.18	hypothetical protein [Arthrobacter sp. 35W]
gi651452652	0.75	1	1	2121	230.8	5.71	4.18	hypothetical protein [Arthrobacter nicotinovorans]
gi116610602	3.76	1	1	479	50.6	5.33	4.18	succinate semialdehyde dehydrogenase [Arthrobacter sp. FB24]
gi908698399	3.37	1	1	534	57.1	6.13	4.18	methylcrotonoyl-CoA carboxylase [Arthrobacter sp. RIT-PI-e]
gi651445282	7.30	1	1	178	19.1	4.89	4.18	GCN5 family acetyltransferase [Arthrobacter nicotinovorans]
gi917441963	4.74	1	1	359	38.6	5.43	4.18	uroporphyrinogen decarboxylase [Arthrobacter albus]
gi545111367	1.84	1	1	651	71.6	5.78	4.18	acetyl-coenzyme A synthetase [Arthrobacter sp. AK-YN10]
gi916820561	2.41	2	1	373	40.9	10.42	4.18	transposase [Arthrobacter sp. H20]
gi651430853	3.09	1	1	485	52.7	5.67	4.18	glutamate synthase [Arthrobacter sanguinis]
gi651464842	2.25	1	1	667	73.7	6.52	4.17	DNA primase [Arthrobacter sp. 35/47]
gi759733266	12.99	1	1	177	17.8	5.47	4.17	2-C-methyl-D-erythritol 2,4-cyclodiphosphate synthase [Arthrobacter sp. L77]
gi545108994	3.20	1	1	562	62.1	9.80	4.17	hypothetical protein [Arthrobacter sp. AK-YN10]
gi654819694	4.30	1	1	372	38.5	5.83	4.17	methylitaconate delta2-delta3-isomerase [Arthrobacter sp. UNC362MFTsu5.1]
gi639129678	6.14	1	1	114	12.9	7.21	4.17	DNA-binding protein [Arthrobacter sp. CAL618]
gi723606311	5.92	1	1	169	18.5	4.70	4.17	hypothetical protein ART_0088 [Arthrobacter sp. PAMC25486]
gi470220603	3.52	1	1	426	45.0	4.56	4.17	enolase [Arthrobacter gangotriensis Lz1y]
gi652423928	1.72	1	1	407	42.4	10.87	4.17	MFS transporter [Arthrobacter castelli]
gi759714682	2.82	3	1	497	50.6	5.73	4.16	carbohydrate kinase [Arthrobacter sp. AK-YN10]
gi908698116	1.16	1	1	1119	115.9	5.11	4.16	hypothetical protein [Arthrobacter sp. RIT-PI-e]
gi786031557	5.05	1	1	317	33.7	9.79	4.16	formamidopyrimidine-DNA glycosylase [Arthrobacter chlorophenolicus]
gi742755139	2.36	4	1	593	64.5	9.57	4.16	glycosyltransferase [Arthrobacter phenanthrenivorans]
gi910250341	9.58	1	1	167	18.2	7.03	4.16	metal-dependent phosphodiesterase [Arthrobacter siccitolerans]
gi908699543	9.49	3	1	137	15.3	9.85	4.16	hypothetical protein [Arthrobacter sp. RIT-PI-e]
gi476401113	7.39	1	1	176	18.2	5.80	4.16	hypothetical protein D477_010381 [Arthrobacter crystallopoietes BAB-32]
gi742853813	10.75	1	1	186	21.2	9.91	4.16	hypothetical protein [Arthrobacter sp. W1]
gi515765822	3.83	1	1	574	62.7	6.79	4.16	histidinol phosphatase [Arthrobacter sp. M2012083]
gi636843437	7.98	1	1	238	25.0	4.16	4.16	beta-lactamase, partial [Arthrobacter sp. TB 26]
gi765010324	3.12	2	1	449	48.1	5.01	4.16	hypothetical protein [Arthrobacter sp. A3]
gi648259882	7.24	1	1	152	16.2	7.14	4.16	MULTISPECIES: ribonucleoside-diphosphate reductase [Arthrobacter]
gi753932431	1.11	1	1	1079	115.8	6.90	4.16	hypothetical protein [Arthrobacter arilaitensis]
gi652424369	3.60	2	1	333	35.0	8.66	4.15	hypothetical protein [Arthrobacter castelli]
gi930825702	10.00	1	1	100	11.2	9.13	4.15	hypothetical protein AOZ07_04905 [Arthrobacter arilaitensis]
gi654822944	2.21	2	1	453	47.5	6.04	4.15	MBL fold metallo-hydrolase [Arthrobacter sp. I3]
gi737802934	4.18	2	1	263	28.5	9.14	4.15	hydrolase [Arthrobacter castelli]
gi219860235	10.11	1	1	178	19.2	11.21	4.15	membrane protein-like protein [Arthrobacter chlorophenolicus A6]
gi914715286	5.39	1	1	371	39.4	5.21	4.15	homoserine acetyltransferase [Arthrobacter sp. ZBG10]
gi823667925	0.99	1	1	1111	118.5	6.23	4.15	hypothetical protein AA310_04725 [Arthrobacter sp. YC-RL1]
gi651507319	7.54	1	1	199	21.8	5.49	4.15	recombinase RecR [Arthrobacter sp. 35W]
gi910283929	5.76	1	1	399	42.3	5.90	4.15	hypothetical protein [Arthrobacter sp. A3]
gi517600612	5.50	1	1	218	23.9	9.26	4.15	TetR family transcriptional regulator [Arthrobacter sp. 162MFSha1.1]
gi737812226	17.82	2	1	101	11.0	11.43	4.15	transposase, partial [Arthrobacter sp. H14]
gi927295707	3.22	1	1	311	32.3	5.30	4.15	ABC transporter substrate-binding protein [Arthrobacter sp. ERGS1:01]
gi636843860	14.40	1	1	125	13.4	6.51	4.15	ArsR family transcriptional regulator [Arthrobacter sp. TB 26]
gi651433680	4.56	1	1	263	28.3	5.12	4.14	DNA-binding protein [Arthrobacter sp. H41]
gi323471661	12.79	1	1	86	10.2	9.82	4.14	hypothetical protein Asphe3_42790 (plasmid) [Arthrobacter phenanthrenivorans Sphe3]
gi651440208	3.62	1	1	304	32.7	5.83	4.14	ABC transporter ATP-binding protein [Arthrobacter sp. H14]
gi403227960	6.34	1	1	268	28.7	6.81	4.14	putative HTH-type transcriptional regulator YagI [Arthrobacter sp. Rue61a]

gi927292839	4.12	1	1	485	50.2	5.15	4.14	hypothetical protein AL755_00865 (plasmid) [Arthrobacter sp. ERGS1:01]
gi927031770	2.62	4	1	535	56.3	5.78	4.14	dehydrogenase [Arthrobacter sp. LS16]
gi910252173	6.90	1	1	145	16.1	4.96	4.14	dihydroneopterin aldolase [Arthrobacter siccitolerans]
gi652425609	2.20	1	1	499	56.6	5.20	4.14	hypothetical protein [Arthrobacter castelli]
gi916692479	5.85	1	1	325	34.1	10.30	4.14	hypothetical protein [Arthrobacter castelli]
gi443482450	1.45	1	1	1380	150.1	5.94	4.14	hypothetical protein G205_05756 [Arthrobacter nitrophenolicus]
gi823667385	18.84	1	1	69	8.0	12.37	4.14	hypothetical protein AA310_17370, partial [Arthrobacter sp. YC-RL1]
gi517592170	1.71	1	1	643	68.7	6.15	4.14	hypothetical protein [Arthrobacter sp. 135MFCol5.1]
gi928488409	7.87	1	1	216	23.1	8.27	4.14	hypothetical protein AOC05_17770 [Arthrobacter alpinus]
gi639129689	4.75	2	1	421	43.3	9.91	4.14	arabinose ABC transporter permease [Arthrobacter sp. CAL618]
gi767258793	5.99	1	1	167	18.8	5.01	4.14	hypothetical protein UM93_16535 [Arthrobacter sp. IHBB 11108]
gi765007344	6.59	1	1	334	36.4	6.60	4.13	NAD-dependent dehydratase [Arthrobacter sp. A3]
gi928486910	1.48	2	1	1152	124.7	7.09	4.13	DNA polymerase III subunit alpha [Arthrobacter alpinus]
gi737775428	3.34	1	1	359	37.8	9.23	4.13	membrane protein [Arthrobacter sp. MA-N2]
gi759736232	5.95	1	1	454	46.7	5.06	4.12	histidinol dehydrogenase [Arthrobacter sp. L77]
gi636843911	10.42	1	1	192	20.8	8.50	4.12	heme-binding protein [Arthrobacter sp. TB 26]
gi910741511	3.46	1	1	347	39.1	8.60	4.12	stearoyl-CoA 9-desaturase [Arthrobacter sp. Hiyo4]
gi162954409	6.86	2	1	277	29.8	9.74	4.12	hypothetical protein RSal33209_2192 [Renibacterium salmoninarum ATCC 33209]
gi930826271	1.91	2	1	470	51.0	8.94	4.11	GntR family transcriptional regulator [Arthrobacter arilaitensis]
gi674645218	4.17	1	1	408	43.3	4.98	4.11	Phosphoglycerate kinase [Arthrobacter sp. 11W110_air]
gi636844286	5.42	2	1	277	30.8	6.14	4.11	exodeoxyribonuclease III [Arthrobacter sp. TB 26]
gi928487350	2.57	1	1	973	102.4	6.67	4.10	hypothetical protein AOC05_10835 [Arthrobacter alpinus]
gi654824073	5.00	1	1	280	30.4	5.02	4.10	ATP phosphoribosyltransferase [Arthrobacter sp. I3]
gi759734230	2.53	1	1	475	49.9	5.22	4.10	pyridoxal-dependent decarboxylase [Arthrobacter sp. L77]
gi323468725	3.94	1	1	609	64.5	5.91	4.10	AMP-forming long-chain acyl-CoA synthetase [Arthrobacter phenanthrenivorans Sphe3]
gi323469900	10.92	1	1	119	12.2	6.76	4.10	hypothetical protein Asphe3_24550 [Arthrobacter phenanthrenivorans Sphe3]
gi489899939	7.27	1	1	220	23.8	6.34	4.10	mechanosensitive ion channel protein MscS [Arthrobacter globiformis]
gi517599818	3.15	1	1	381	40.4	5.34	4.09	ROK family transcriptional regulator [Arthrobacter sp. 162MFSha1.1]
gi551256920	0.74	1	1	1486	163.9	5.48	4.09	hypothetical protein [Arthrobacter sp. PAO19]
gi917745741	4.62	2	1	411	41.3	5.52	4.09	N-acetylglucosamine 6-phosphate deacetylase [Arthrobacter phenanthrenivorans]
gi635353001	3.09	1	1	647	70.9	6.44	4.09	bilirubin oxidase [Arthrobacter siccitolerans]
gi359304459	2.66	2	1	602	64.9	5.36	4.09	glucosamine--fructose-6-phosphate aminotransferase [Arthrobacter globiformis NBRC 12137
gi906446000	4.13	2	1	412	43.4	8.54	4.09	hypothetical protein AC792_10825, partial [Arthrobacter sp. RIT-PI-e]
gi928487674	14.29	5	1	133	13.8	8.68	4.09	GntR family transcriptional regulator [Arthrobacter alpinus]
gi654819460	10.86	4	1	221	24.2	6.81	4.09	GntR family transcriptional regulator [Arthrobacter sp. UNC362MFTsu5.1]
gi403231188	4.23	1	1	378	38.9	6.18	4.09	putative xylose repressor XylR [Arthrobacter sp. Rue61a]
gi651429935	7.27	1	1	330	35.9	5.78	4.09	2-oxoisovalerate dehydrogenase [Arthrobacter sanguinis]
gi651508004	5.35	1	1	318	34.4	5.41	4.09	hypothetical protein [Arthrobacter sp. 35W]
gi403227706	4.60	1	1	457	47.9	6.55	4.08	DNA repair protein RadA [Arthrobacter sp. Rue61a]
gi742756585	4.24	3	1	236	23.9	8.56	4.08	hypothetical protein RM50_09080 [Arthrobacter phenanthrenivorans]
gi443481080	4.22	1	1	379	39.7	5.76	4.08	signal transduction histidine kinase [Arthrobacter nitrophenolicus]
gi652423345	1.57	1	1	1337	144.6	5.30	4.08	ATPase AAA [Arthrobacter castelli]
gi654812725	5.42	1	1	240	25.4	9.00	4.08	ABC transporter ATP-binding protein [Arthrobacter sp. MA-N2]
gi916834922	6.32	1	1	253	27.9	9.73	4.08	hypothetical protein, partial [Arthrobacter sp. H14]
gi517592089	4.92	1	1	244	23.6	8.21	4.08	hypothetical protein [Arthrobacter sp. 135MFCol5.1]
gi917739898	6.34	1	1	205	22.0	4.75	4.08	hypothetical protein [Arthrobacter sp. W1]
gi652422602	2.37	3	1	758	81.6	7.53	4.08	transcription termination factor Rho [Arthrobacter castelli]
gi917013451	2.97	1	1	303	33.5	10.17	4.08	hypothetical protein [Arthrobacter sanguinis]
gi517603229	4.01	1	1	299	32.5	10.02	4.07	tyrosine recombinase XerC [Arthrobacter sp. 131MFCol6.1]
gi742755017	6.07	1	1	412	43.1	5.81	4.07	alanine racemase [Arthrobacter phenanthrenivorans]
gi910747717	16.90	1	1	142	14.8	11.15	4.07	hypothetical protein AHiyo8_54190 [Arthrobacter sp. Hiyo8]
gi917739712	11.54	3	1	156	17.2	8.82	4.07	hypothetical protein [Arthrobacter sp. W1]
gi914714574	4.37	1	1	252	26.5	5.82	4.07	oxidoreductase [Arthrobacter sp. ZBG10]
gi910740000	12.09	1	1	91	9.1	4.88	4.07	uncharacterized oxidoreductase Rv0484c/MT0502 [Arthrobacter sp. Hiyo4]
gi723609447	2.56	1	1	939	103.1	5.39	4.07	phosphoenolpyruvate carboxylase [Arthrobacter sp. PAMC25486]

gi767256537	12.29	3	1	179	20.1	9.42	4.07	hypothetical protein UM93_00565 [Arthrobacter sp. IHBB 11108]
gi518312144	6.91	2	1	246	26.4	5.36	4.06	MULTISPECIES: hypothetical protein [Arthrobacter]
gi753937926	2.56	1	1	352	36.8	5.88	4.06	tRNA(Ile)-lysidine synthetase [Arthrobacter phenanthrenivorans]
gi914715097	4.21	4	1	451	48.3	8.12	4.06	transcriptional regulator [Arthrobacter sp. ZBG10]
gi517604666	3.55	1	1	451	48.5	5.33	4.06	acetyl-CoA carboxylase biotin carboxylase subunit [Arthrobacter sp. 131MFCol6.1]
gi359304216	3.03	1	1	462	48.4	9.35	4.06	putative M23 family peptidase [Arthrobacter globiformis NBRC 12137]
gi759715954	2.37	1	1	590	62.0	10.78	4.06	DEAD/DEAH box helicase [Arthrobacter sp. AK-YN10]
gi651434574	2.99	1	1	468	48.9	5.92	4.06	flavoprotein disulfide reductase [Arthrobacter sp. H41]
gi189043022	7.38	1	1	244	25.8	4.69	4.06	RecName: Full=1-(5-phosphoribosyl)-5-[(5-phosphoribosylamino)methylideneamino] imidaz
gi723607468	1.65	2	1	909	104.1	7.91	4.06	hypothetical protein ART_1245 [Arthrobacter sp. PAMC25486]
gi476399150	1.49	2	1	672	71.4	6.60	4.06	2,4-dienoyl-CoA reductase [Arthrobacter crystallopoietes BAB-32]
gi640203024	8.93	1	1	168	17.2	9.31	4.06	aminoacyl-tRNA deacylase [Arthrobacter sp. 31Y]
gi910249733	2.92	1	1	377	40.6	5.41	4.05	GNAT family acetyltransferase [Arthrobacter siccitolerans]
gi928985585	4.32	1	1	370	40.6	4.70	4.05	hypothetical protein [Arthrobacter sp. ERGS1:01]
gi542110117	1.95	2	1	461	50.2	5.06	4.05	hypothetical protein M707_02010 [Arthrobacter sp. AK-YN10]
gi323471361	5.22	3	1	498	52.8	10.15	4.05	sphingosine/diacylglycerol kinase-like enzyme [Arthrobacter phenanthrenivorans Sphe3]
gi651434454	5.11	1	1	470	51.2	7.47	4.05	two-component system sensor histidine kinase [Arthrobacter sp. H41]
gi640204570	11.28	1	1	195	21.3	9.61	4.05	invertase [Arthrobacter sp. 31Y]
gi916863256	2.69	1	1	446	46.6	6.09	4.05	acetyl-CoA acetyltransferase [Arthrobacter sp. 35/47]
gi918265976	5.45	1	1	220	23.6	5.96	4.05	lipoprotein-releasing system ATP-binding protein LolD [Arthrobacter sp. Hiyo1]
gi648573979	3.78	1	1	423	43.9	10.11	4.05	MFS transporter [Arthrobacter sp. 162MFSha1.1]
gi651441296	4.35	2	1	299	32.3	6.54	4.05	inorganic polyphosphate kinase [Arthrobacter sp. 9MFCol3.1]
gi517605239	1.76	2	1	910	96.4	7.34	4.05	magnesium-transporting ATPase [Arthrobacter sp. 131MFCol6.1]
gi636844937	3.28	1	1	274	28.7	8.37	4.05	ATP synthase subunit delta [Arthrobacter sp. TB 26]
gi443481509	14.85	1	1	101	11.7	12.26	4.05	hypothetical protein G205_11835 [Arthrobacter nitrophenolicus]
gi742071408	9.05	1	1	210	22.3	9.23	4.04	TetR family transcriptional regulator [Arthrobacter sp. MWB30]
gi928486767	1.14	1	1	879	95.5	4.88	4.04	aminopeptidase [Arthrobacter alpinus]
gi760113087	7.92	3	1	366	40.5	5.20	4.04	hypothetical protein [Arthrobacter chlorophenolicus]
gi219859767	4.79	1	1	459	47.8	5.74	4.04	gamma-glutamyl phosphate reductase [Arthrobacter chlorophenolicus A6]
gi927294332	3.83	1	1	287	30.6	5.16	4.04	hypothetical protein AL755_10385 [Arthrobacter sp. ERGS1:01]
gi917739415	6.93	4	1	231	24.2	4.73	4.04	cytidylate kinase [Arthrobacter sp. W1]
gi651473452	6.19	1	1	339	36.9	5.40	4.04	hypothetical protein [Arthrobacter nicotinovorans]
gi916834880	2.41	3	1	539	57.0	6.39	4.04	hypothetical protein [Arthrobacter sp. H14]
gi652424386	3.16	2	1	475	54.2	5.82	4.04	deoxyribodipyrimidine photolyase [Arthrobacter castelli]
gi723608283	4.62	1	1	325	33.4	9.58	4.03	histidine kinase [Arthrobacter sp. PAMC25486]
gi742069622	1.89	1	1	371	40.0	8.18	4.03	hypothetical protein ANMWB30_34190 [Arthrobacter sp. MWB30]
gi518311002	7.19	1	1	306	33.4	6.54	4.03	ABC transporter ATP-binding protein [Arthrobacter sp. TB 23]
gi910740437	5.23	5	1	421	46.9	9.17	4.03	uncharacterized protein YcaQ [Arthrobacter sp. Hiyo4]
gi737787081	2.85	1	1	561	60.9	7.30	4.03	FAD-binding dehydrogenase [Arthrobacter albus]
gi470215962	6.18	1	1	178	20.0	4.73	4.02	dihydrofolate reductase [Arthrobacter gangotriensis Lz1y]
gi219858781	2.00	1	1	600	64.0	5.53	4.02	transcriptional regulator, SARP family [Arthrobacter chlorophenolicus A6]
gi917022062	2.62	1	1	573	61.3	5.66	4.02	hypothetical protein [Arthrobacter sp. UNC362MFTsu5.1]
gi542106955	5.57	1	1	395	41.0	6.84	4.01	ArsR family transcriptional regulator [Arthrobacter sp. AK-YN10]
gi517592732	4.37	1	1	412	43.6	5.55	4.01	hypothetical protein [Arthrobacter sp. 135MFCol5.1]
gi517600681	5.47	1	1	274	29.1	5.47	4.01	xylose isomerase [Arthrobacter sp. 162MFSha1.1]
gi648573141	1.68	1	1	895	97.4	7.75	4.01	LuxR family transcriptional regulator [Arthrobacter sp. 135MFCol5.1]
gi517602378	5.17	2	1	232	24.3	6.68	4.01	hypothetical protein [Arthrobacter sp. 131MFCol6.1]
gi937259577	1.92	1	1	1040	109.3	5.72	4.01	FAD-linked oxidase [Arthrobacter sp. Edens01]
gi916816149	5.12	1	1	371	38.6	10.26	4.01	hypothetical protein [Arthrobacter sp. MA-N2]
gi515766351	3.29	1	1	516	54.4	5.95	4.01	aldehyde dehydrogenase [Arthrobacter sp. M2012083]
gi219858678	5.92	1	1	287	30.7	5.07	4.01	Xylose isomerase domain protein TIM barrel [Arthrobacter chlorophenolicus A6]
gi545107663	2.95	1	1	407	45.0	6.25	4.00	hypothetical protein [Arthrobacter sp. AK-YN10]
gi916834738	2.71	2	1	701	73.2	6.33	4.00	hypothetical protein [Arthrobacter sp. H14]
gi654811566	5.03	1	1	378	40.2	5.02	4.00	acyl-CoA dehydrogenase [Arthrobacter sp. MA-N2]
gi517598822	3.53	1	1	453	48.5	7.68	4.00	transcriptional regulator [Arthrobacter sp. 162MFSha1.1]

gi119947548	4.52	2	1	354	40.2	5.63	3.99	putative ATP dependent DNA ligase [Arthrobacter aurescens TC1]
gi651499114	7.43	1	1	175	19.3	5.00	3.99	cell division protein SepF [Arthrobacter sp. 35W]
gi910249704	2.81	2	1	320	36.0	10.18	3.99	hypothetical protein [Arthrobacter siccitolerans]
gi635353343	2.81	3	1	569	61.7	5.21	3.99	ABC transporter transmembrane region family protein [Arthrobacter siccitolerans]
gi551254432	9.62	3	1	104	11.8	8.57	3.99	hypothetical protein [Arthrobacter sp. PAO19]
gi723609889	1.70	1	1	705	72.2	8.05	3.99	hypothetical protein ART_3666 [Arthrobacter sp. PAMC25486]
gi928987362	3.52	1	1	512	55.1	9.99	3.98	hypothetical protein [Arthrobacter sp. ERGS1:01]
gi162954137	6.13	1	1	326	35.4	7.33	3.98	GTP-binding protein [Renibacterium salmoninarum ATCC 33209]
gi651502394	2.31	2	1	390	40.0	6.65	3.98	DNA processing protein DprA [Arthrobacter sp. 35W]
gi162954194	7.19	1	1	278	29.2	6.80	3.97	shikimate 5-dehydrogenase [Renibacterium salmoninarum ATCC 33209]
gi517604961	3.75	1	1	480	51.1	6.65	3.97	MULTISPECIES: PucR family transcriptional regulator [Arthrobacter]
gi928487773	3.45	1	1	464	47.6	5.73	3.97	branched-chain alpha-keto acid dehydrogenase subunit E2 [Arthrobacter alpinus]
gi652425361	9.63	1	1	270	28.7	7.05	3.97	hypothetical protein [Arthrobacter castelli]
gi654826846	10.90	1	1	156	17.2	10.33	3.97	30S ribosomal protein S7 [Arthrobacter sp. H5]
gi927032785	4.89	2	1	327	35.0	4.82	3.97	delta-aminolevulinic acid dehydratase [Arthrobacter sp. LS16]
gi470220502	1.35	1	1	1181	121.3	5.26	3.97	putative membrane protein mmpL3 [Arthrobacter gangotriensis Lz1y]
gi742751664	5.33	1	1	450	48.1	5.20	3.97	flagellar basal body P-ring biosynthesis protein FlgA [Arthrobacter phenanthrenivorans]
gi767257662	6.02	1	1	349	35.6	11.05	3.97	hypothetical protein UM93_08525 [Arthrobacter sp. IHBB 11108]
gi307744933	3.67	1	1	409	44.5	5.20	3.97	coproporphyrinogen oxidase [Arthrobacter arilaitensis Re117]
gi749401264	11.71	1	1	111	13.1	9.91	3.96	taurine dioxygenase, partial [Arthrobacter sp. AK-YN10]
gi542109934	2.49	1	1	241	25.3	4.67	3.96	haloacid dehalogenase [Arthrobacter sp. AK-YN10]
gi757625135	14.18	3	1	134	14.9	6.80	3.96	transcriptional regulator [Arthrobacter sp. SPG23]
gi636845163	3.66	5	1	437	47.4	5.76	3.95	tyrosine--tRNA ligase [Arthrobacter sp. TB 26]
gi910737277	9.13	1	1	219	24.2	5.22	3.95	transcriptional regulator protein [Arthrobacter sp. Hiyo4]
gi307746073	2.46	2	1	366	39.3	6.80	3.95	alanine racemase-like protein [Arthrobacter arilaitensis Re117]
gi476400395	2.27	2	1	484	51.5	5.48	3.94	inosine 5-monophosphate dehydrogenase [Arthrobacter crystallopoietes BAB-32]
gi307744673	2.01	1	1	547	59.8	6.49	3.94	putative GTPase [Arthrobacter arilaitensis Re117]
gi757626201	7.36	2	1	299	30.4	4.88	3.94	oxidoreductase [Arthrobacter sp. SPG23]
gi916781922	1.55	1	1	579	62.2	4.82	3.94	hypothetical protein [Arthrobacter sp. 35W]
gi759724578	3.97	1	1	403	44.1	6.05	3.94	multicopper oxidase [Arthrobacter sp. I3]
gi915933352	2.83	2	1	353	36.4	6.84	3.94	alanine racemase [Arthrobacter globiformis]
gi939036210	3.08	1	1	389	42.4	7.90	3.94	hypothetical protein [Arthrobacter nitroguajacolicus]
gi162955748	5.26	1	1	190	20.4	4.31	3.94	Jag protein [Renibacterium salmoninarum ATCC 33209]
gi443479901	3.36	1	1	387	41.9	5.68	3.93	aminotransferase [Arthrobacter nitrophenolicus]
gi823666530	8.33	1	1	252	29.2	5.90	3.93	GTP pyrophosphokinase [Arthrobacter sp. YC-RL1]
gi939050480	4.76	2	1	231	24.9	5.00	3.93	EAL domain-containing protein [Arthrobacter sp. JCM 19049]
gi517593488	2.17	1	1	460	47.9	5.36	3.93	hypothetical protein [Arthrobacter sp. 135MFCol5.1]
gi545111347	4.69	2	1	277	31.1	9.20	3.93	hypothetical protein [Arthrobacter sp. AK-YN10]
gi914716304	1.33	1	1	1275	140.1	5.63	3.92	hypothetical protein [Arthrobacter sp. ZBG10]
gi674646627	13.25	1	1	166	18.2	4.55	3.92	hypothetical protein BN1051_02837 [Arthrobacter sp. 11W110_air]
gi767257176	4.93	1	1	446	48.2	6.57	3.92	ABC transporter substrate-binding protein [Arthrobacter sp. IHBB 11108]
gi636847476	3.32	1	1	301	32.5	4.83	3.91	hypothetical protein [Arthrobacter sp. TB 26]
gi542108146	3.23	1	1	465	50.2	5.72	3.91	mycothione reductase [Arthrobacter sp. AK-YN10]
gi517604300	2.83	1	1	566	58.4	6.11	3.91	NADH dehydrogenase [Arthrobacter sp. 131MFCol6.1]
gi917013268	2.78	1	1	647	69.4	6.27	3.91	hypothetical protein [Arthrobacter sanguinis]
gi742851431	3.41	1	1	381	41.2	5.21	3.91	homoserine acetyltransferase [Arthrobacter sp. W1]
gi489896221	2.03	1	1	590	62.1	6.35	3.91	two-component system sensor histidine kinase [Arthrobacter globiformis]
gi470220703	7.96	1	1	201	22.3	9.54	3.91	sugar transferase [Arthrobacter gangotriensis Lz1y]
gi930825774	3.70	1	1	595	64.8	5.12	3.91	prolyl-tRNA synthetase [Arthrobacter arilaitensis]
gi654822767	3.64	1	1	439	46.4	5.41	3.91	FAD-binding domain-containing protein [Arthrobacter sp. I3]
gi403230495	2.61	1	1	383	42.5	4.91	3.90	carboxylate-amine ligase [Arthrobacter sp. Rue61a]
gi651444030	2.61	1	1	383	42.5	4.91	3.90	carboxylate--amine ligase [Arthrobacter nicotinovorans]
gi737811977	4.84	1	1	372	39.8	6.09	3.90	glycine/betaine ABC transporter ATPase [Arthrobacter sp. 35/47]
gi937259160	7.50	1	1	320	34.1	7.84	3.90	glucokinase [Arthrobacter sp. Edens01]
gi654819712	4.72	1	1	381	39.6	5.94	3.90	cysteine desulfurase [Arthrobacter sp. UNC362MFTsu5.1]

gi651494906	15.15	1	1	66	7.3	7.14	3.89	hypothetical protein [Arthrobacter sp. H20]
gi640196954	2.70	1	1	407	43.7	9.07	3.89	hypothetical protein [Arthrobacter sp. 31Y]
gi918268208	3.53	1	1	340	36.9	4.73	3.89	adenine deaminase [Arthrobacter sp. Hiyo1]
gi551255412	13.29	1	1	158	16.7	5.50	3.89	ribonuclease [Arthrobacter sp. PAO19]
gi753933722	3.62	2	1	552	61.0	8.24	3.89	hypothetical protein [Arthrobacter arilaitensis]
gi403231817	20.00	1	1	85	9.4	6.64	3.89	hypothetical protein ARUE_232p00300 (plasmid) [Arthrobacter sp. Rue61a]
gi916781944	4.33	1	1	393	41.3	5.85	3.89	SAM-dependent methyltransferase [Arthrobacter sp. 35W]
gi674644508	7.81	2	1	256	26.4	5.16	3.89	3-oxoacyl-[acyl-carrier-protein] reductase FabG1 [Arthrobacter sp. 11W110_air]
gi116610061	2.53	2	1	277	28.8	5.50	3.88	HpcH/HpaI aldolase [Arthrobacter sp. FB24]
gi651430906	2.53	1	1	434	45.3	6.25	3.88	acetyl-CoA acetyltransferase [Arthrobacter sanguinis]
gi1333652	16.67	5	1	84	9.0	10.33	3.88	ribosomal protein L27, partial [Arthrobacter sp. TE1826]
gi651444668	2.78	1	1	683	70.5	7.33	3.88	peptide ABC transporter ATPase [Arthrobacter nicotinovorans]
gi517592304	12.12	1	1	165	17.7	6.02	3.88	hypothetical protein [Arthrobacter sp. 135MFCol5.1]
gi476399039	6.29	1	1	175	19.7	6.44	3.88	hypothetical protein D477_020493 [Arthrobacter crystallopoietes BAB-32]
gi651492553	1.79	1	1	838	90.8	5.94	3.88	glycine cleavage system protein T [Arthrobacter sp. H20]
gi651457598	1.84	2	1	761	78.2	5.52	3.87	carbonate dehydratase [Arthrobacter sp. 35/47]
gi651497633	5.17	1	1	232	24.7	7.05	3.87	ABC transporter ATP-binding protein [Arthrobacter sp. 35W]
gi652422991	12.79	1	1	86	9.2	11.60	3.87	30S ribosomal protein S20 [Arthrobacter castelli]
gi639129450	4.89	1	1	307	32.2	8.35	3.87	formamidopyrimidine-DNA glycosylase [Arthrobacter sp. CAL618]
gi759725619	3.79	1	1	475	49.0	9.85	3.86	amino acid transporter [Arthrobacter sp. I3]
gi908697197	8.29	1	1	217	23.4	5.30	3.86	GntR family transcriptional regulator [Arthrobacter sp. RIT-PI-e]
gi674646834	1.58	1	1	1141	126.5	5.34	3.86	hypothetical protein BN1051_03046 [Arthrobacter sp. 11W110_air]
gi742758985	6.80	1	1	147	15.7	5.68	3.86	hypothetical protein RM50_01650 [Arthrobacter phenanthrenivorans]
gi928488874	2.05	1	1	341	36.3	8.46	3.86	LacI family transcriptional regulator [Arthrobacter alpinus]
gi930827339	3.98	1	1	377	41.3	5.59	3.86	mandelate racemase/muconate lactonizing protein [Arthrobacter arilaitensis]
gi443481338	6.74	2	1	282	28.8	4.88	3.86	hypothetical protein G205_13307, partial [Arthrobacter nitrophenolicus]
gi916863737	12.26	1	1	106	12.0	9.55	3.86	hypothetical protein [Arthrobacter sp. 35/47]
gi651440670	2.14	1	1	654	70.6	4.88	3.85	aconitate hydratase [Arthrobacter sp. H14]
gi307743798	4.51	1	1	288	30.5	4.98	3.85	DSBA-like thioredoxin domain-containing protein [Arthrobacter arilaitensis Re117]
gi767256571	1.99	1	1	602	62.7	4.88	3.85	phosphomannomutase [Arthrobacter sp. IHBB 11108]
gi116610015	3.52	1	1	398	44.3	6.77	3.84	glycosyl transferase, group 1 [Arthrobacter sp. FB24]
gi518313138	15.70	2	1	121	13.7	6.58	3.84	hypothetical protein [Arthrobacter sp. TB 23]
gi765004252	8.92	1	1	157	17.4	11.14	3.84	hypothetical protein [Arthrobacter sp. A3]
gi910692753	4.20	1	1	262	27.7	6.43	3.84	methylcrotonoyl-CoA carboxylase beta chain, mitochondrial [Arthrobacter sp. Hiyo6]
gi403229865	2.33	2	1	386	42.6	7.72	3.83	PhoH-like protein [Arthrobacter sp. Rue61a]
gi119950295	2.05	1	1	880	95.4	5.15	3.83	DNA polymerase I [Arthrobacter aurescens TC1]
gi443480790	3.73	1	1	295	33.3	6.20	3.83	aminoglycoside phosphotransferase [Arthrobacter nitrophenolicus]
gi759768302	2.25	1	1	533	56.9	8.88	3.83	ABC transporter [Arthrobacter sp. SPG23]
gi917739709	3.45	3	1	290	30.2	5.36	3.83	molybdenum cofactor guanylyltransferase [Arthrobacter sp. W1]
gi737812242	3.66	5	1	437	49.3	10.64	3.83	integrase [Arthrobacter sp. H14]
gi927031387	4.50	1	1	289	30.3	5.50	3.82	thioredoxin [Arthrobacter sp. LS16]
gi654816132	4.72	1	1	212	23.2	5.57	3.82	TetR family transcriptional regulator [Arthrobacter sp. UNC362MFTsu5.1]
gi910742967	8.64	2	1	162	17.2	10.04	3.82	diacylglycerol kinase catalytic region [Arthrobacter sp. Hiyo8]
gi489899701	1.43	1	1	491	51.8	4.98	3.82	NAD-dependent succinate-semialdehyde dehydrogenase [Arthrobacter globiformis]
gi823668473	14.63	2	1	41	4.9	6.67	3.81	transposase, partial [Arthrobacter sp. YC-RL1]
gi916863551	5.24	1	1	286	29.6	6.89	3.81	hypothetical protein [Arthrobacter sp. 35/47]
gi759732475	0.66	1	1	1669	177.4	5.55	3.81	DEAD/DEAH box helicase [Arthrobacter sp. L77]
gi651495996	4.63	2	1	324	34.4	6.54	3.81	metal ABC transporter ATPase [Arthrobacter sp. H20]
gi737773933	18.97	1	1	58	6.3	10.93	3.81	hypothetical protein [Arthrobacter sp. MA-N2]
gi542109953	1.07	1	1	839	91.8	6.43	3.81	ATP-dependent DNA ligase [Arthrobacter sp. AK-YN10]
gi928542292	27.27	1	1	77	8.5	10.11	3.81	hypothetical protein SEA_BRENT_49 [Arthrobacter phage Brent]
gi651429493	5.46	1	1	293	30.9	5.35	3.80	3-methyl-2-oxobutanoate hydroxymethyltransferase [Arthrobacter sanguinis]
gi517610021	1.78	1	1	507	55.4	5.00	3.80	alpha-N-arabinofuranosidase [Arthrobacter sp. 161MFSha2.1]
gi652425293	10.06	1	1	179	19.2	6.37	3.78	RecX family transcriptional regulator [Arthrobacter castelli]
gi639130340	3.09	1	1	421	44.9	4.70	3.78	cytochrome C biogenesis protein ResB, partial [Arthrobacter sp. CAL618]

gi443480022	3.87	1	1	362	38.8	6.32	3.78	amidohydrolase [Arthrobacter nitrophenolicus]
gi359304065	2.88	1	1	312	34.5	7.14	3.78	putative LysR family transcriptional regulator [Arthrobacter globiformis NBRC 12137]
gi937258821	4.58	1	1	131	14.0	6.68	3.77	hypothetical protein AO716_12690 [Arthrobacter sp. Edens01]
gi917012979	3.65	1	1	384	41.0	5.17	3.76	amidohydrolase [Arthrobacter sanguinis]
gi640204718	2.63	1	1	609	64.8	7.72	3.76	hypothetical protein [Arthrobacter sp. 31Y]
gi652424140	3.75	1	1	480	53.1	9.86	3.76	hypothetical protein [Arthrobacter castelli]
gi927294707	1.00	1	1	1098	121.7	5.06	3.76	isoleucine--tRNA ligase [Arthrobacter sp. ERGS1:01]
gi927295539	5.85	4	1	359	38.9	5.17	3.76	inositol-3-phosphate synthase [Arthrobacter sp. ERGS1:01]
gi515765192	7.17	1	1	223	23.5	4.79	3.75	haloacid dehalogenase [Arthrobacter sp. M2012083]
gi651467251	1.33	7	1	1277	140.7	6.10	3.75	alpha-ketoglutarate decarboxylase [Arthrobacter sp. 35/47]
gi403231869	6.74	1	1	193	22.1	10.04	3.75	hypothetical protein ARUE_232p00820 (plasmid) [Arthrobacter sp. Rue61a]
gi403228276	4.87	1	1	534	57.5	6.83	3.75	glutathione import ATP-binding protein GsiA [Arthrobacter sp. Rue61a]
gi767256513	5.47	1	1	439	44.7	7.27	3.74	peptidase S8 [Arthrobacter sp. IHBB 11108]
gi823668044	2.39	1	1	627	69.9	6.34	3.74	DNA primase [Arthrobacter sp. YC-RL1]
gi910740179	8.29	2	1	205	22.6	4.63	3.74	ketol-acid reductoisomerase [Arthrobacter sp. Hiyo4]
gi767258351	12.12	1	1	99	11.0	6.52	3.73	CopY family transcriptional regulator [Arthrobacter sp. IHBB 11108]
gi654816505	5.75	4	1	313	32.7	5.22	3.73	hydroxyacid dehydrogenase [Arthrobacter sp. UNC362MFTsu5.1]
gi551255262	2.92	1	1	274	30.3	8.37	3.73	DNA glycosylase [Arthrobacter sp. PAO19]
gi910697365	5.56	1	1	288	31.6	6.24	3.73	uncharacterized oxidoreductase MSMEG_2408/MSMEI_2347 [Arthrobacter sp. Hiyo6]
gi651499640	2.50	1	1	440	46.2	5.82	3.73	branched-chain alpha-keto acid dehydrogenase subunit E2 [Arthrobacter sp. 35W]
gi742068702	19.51	1	1	41	4.6	10.78	3.73	hypothetical protein ANMWB30_43230 [Arthrobacter sp. MWB30]
gi470217137	2.54	1	1	512	54.2	4.87	3.72	Microcystin LR degradation protein MlrC [Arthrobacter gangotriensis Lz1y]
gi651437319	4.90	1	1	204	23.7	7.84	3.72	hypothetical protein [Arthrobacter sp. H14]
gi323470024	2.51	1	1	398	41.6	6.13	3.72	hypothetical protein Asphe3_25840 [Arthrobacter phenanthrenivorans Sphe3]
gi476401793	3.59	1	1	362	39.4	4.83	3.71	putative N-acetylmuramoyl-L-alanine amidase [Arthrobacter crystallopoietes BAB-32]
gi651498679	1.79	1	1	952	100.9	5.88	3.70	cell division protein FtsK [Arthrobacter sp. 35W]
gi916871456	0.90	1	1	1111	123.8	5.11	3.70	isoleucine--tRNA ligase [Arthrobacter sp. H5]
gi654821963	3.15	1	1	413	44.7	5.25	3.70	hypothetical protein [Arthrobacter sp. I3]
gi654812269	10.00	1	1	170	17.4	10.80	3.70	hypothetical protein [Arthrobacter sp. MA-N2]
gi651434174	9.17	3	1	218	23.1	10.46	3.69	50S ribosomal protein L3 [Arthrobacter sp. H41]
gi742072539	7.25	1	1	331	35.4	7.61	3.69	hypothetical protein ANMWB30_05500 [Arthrobacter sp. MWB30]
gi937257847	3.51	1	1	456	49.1	5.66	3.69	hypothetical protein AO716_06715 [Arthrobacter sp. Edens01]
gi937256537	3.88	1	1	309	30.7	4.48	3.69	carbohydrate kinase [Arthrobacter sp. Edens01]
gi916926385	7.79	1	1	321	33.2	9.32	3.68	hypothetical protein [Arthrobacter sp. 9MFCol3.1]
gi937259094	2.68	1	1	708	78.0	7.03	3.68	DNA topoisomerase IV [Arthrobacter sp. Edens01]
gi307743580	1.57	3	1	1148	119.5	5.11	3.67	DNA polymerase III, subunits gamma and tau [Arthrobacter arilaitensis Re117]
gi515764962	2.15	1	1	698	73.5	7.28	3.67	primosomal protein N' [Arthrobacter sp. M2012083]
gi742756300	3.42	1	1	497	53.5	7.30	3.67	GTP-binding protein [Arthrobacter phenanthrenivorans]
gi928489012	5.17	1	1	232	23.4	7.53	3.66	hypothetical protein AOC05_16855 [Arthrobacter alpinus]
gi757624851	3.21	1	1	436	46.5	6.40	3.66	ABC transporter substrate-binding protein [Arthrobacter sp. SPG23]
gi255103417	2.60	1	1	385	40.1	6.92	3.66	HNH nuclease-like protein [Arthrobacter sp. 32c]
gi651430170	2.46	1	1	407	43.7	5.44	3.66	cytosine deaminase [Arthrobacter sanguinis]
gi651480606	8.21	4	1	195	21.1	9.76	3.66	serine acetyltransferase [Arthrobacter sp. Br18]
gi651505079	3.40	1	1	294	31.2	9.23	3.65	prenyltransferase [Arthrobacter sp. 35W]
gi518312915	2.75	1	1	364	39.7	6.52	3.65	cysteine synthase [Arthrobacter sp. TB 23]
gi737815141	2.62	1	1	343	36.7	6.92	3.65	sugar ABC transporter [Arthrobacter sp. H14]
gi654811898	1.76	1	1	454	48.8	6.77	3.65	transcriptional regulator [Arthrobacter sp. MA-N2]
gi651497619	7.59	1	1	237	25.6	9.33	3.64	ABC transporter ATP-binding protein [Arthrobacter sp. 35W]
gi635353007	7.89	1	1	190	20.1	8.46	3.64	hipA-like C-terminal domain protein [Arthrobacter siccitolerans]
gi916259772	6.01	1	1	383	41.4	9.19	3.64	DNA polymerase IV [Arthrobacter sp. TB 23]
gi930827578	1.56	1	1	577	62.6	5.07	3.63	multidrug ABC transporter ATP-binding protein [Arthrobacter arilaitensis]
gi937259024	6.79	1	1	162	18.1	8.62	3.62	hypothetical protein AO716_13860 [Arthrobacter sp. Edens01]
gi939050199	3.36	1	1	298	34.0	7.24	3.62	hypothetical protein [Arthrobacter sp. JCM 19049]
gi517599451	7.09	1	1	268	28.0	5.50	3.62	D-beta-D-heptose 1-phosphate adenosyltransferase [Arthrobacter sp. 162MFSha1.1]
gi939036845	3.45	1	1	406	43.8	7.08	3.61	hypothetical protein [Arthrobacter nitroguajacolicus]

gi219862240	2.02	1	1	545	59.2	9.38	3.61	Relaxase/mobilization nuclease family protein (plasmid) [Arthrobacter chlorophenolicus A6]
gi470216161	6.80	1	1	294	31.6	6.16	3.61	Alpha/beta hydrolase family protein [Arthrobacter gangotriensis Lz1y]
gi476402908	1.95	2	1	616	69.3	9.51	3.61	hypothetical protein D477_001269 [Arthrobacter crystallopoietes BAB-32]
gi759731441	1.45	1	1	1448	154.3	6.09	3.61	hypothetical protein [Arthrobacter sp. L77]
gi674645853	2.40	1	1	541	54.7	4.92	3.61	Phosphotransferase enzyme family protein [Arthrobacter sp. 11W110_air]
gi930828163	5.73	1	1	384	43.4	9.14	3.61	hypothetical protein AOZ07_10765 [Arthrobacter arilaitensis]
gi189043025	2.50	1	1	440	45.8	5.11	3.60	RecName: Full=Glutamyl-tRNA reductase; Short=GluTR
gi654816342	2.36	1	1	551	56.6	8.24	3.60	histidine kinase [Arthrobacter sp. UNC362MFTsu5.1]
gi119949705	3.73	1	1	402	42.7	5.02	3.60	Aspartate aminotransferase [Arthrobacter aurescens TC1]
gi651440390	13.01	1	1	146	16.6	5.60	3.60	hypothetical protein [Arthrobacter sp. H14]
gi928487000	2.25	1	1	622	66.3	6.43	3.60	AMP-dependent synthetase [Arthrobacter alpinus]
gi651483019	1.25	1	1	1515	164.4	5.64	3.60	glutamate synthase [Arthrobacter sp. Br18]
gi757624584	5.06	1	1	237	23.5	5.29	3.59	hypothetical protein TV39_09680 [Arthrobacter sp. SPG23]
gi723606682	4.80	1	1	271	27.6	9.41	3.59	putative ABC-type antimicrobial peptide transport system, ATPase component [Arthrobacter
gi651485852	3.45	1	1	348	38.5	5.58	3.58	aldo/keto reductase [Arthrobacter sp. Br18]
gi470216936	4.96	1	1	343	36.5	5.29	3.58	LacI family transcriptional regulator [Arthrobacter gangotriensis Lz1y]
gi515767214	5.28	1	1	360	40.3	4.91	3.58	hypothetical protein [Arthrobacter sp. M2012083]
gi674644160	3.13	1	1	224	22.8	10.40	3.58	Leucine efflux protein [Arthrobacter sp. 11W110_air]
gi910251716	2.55	1	1	667	69.5	9.14	3.57	MFS transporter [Arthrobacter siccitolerans]
gi651431454	1.47	1	1	1154	123.7	5.21	3.57	1-pyrroline-5-carboxylate dehydrogenase [Arthrobacter sanguinis]
gi786027730	2.88	1	1	451	48.1	5.59	3.56	ATP-binding protein [Arthrobacter chlorophenolicus]
gi359304241	8.61	1	1	209	22.9	8.78	3.56	putative glycosyltransferase [Arthrobacter globiformis NBRC 12137]
gi767256859	3.24	1	1	340	37.3	6.47	3.56	methionine ABC transporter ATP-binding protein [Arthrobacter sp. IHBB 11108]
gi517591778	3.19	1	1	470	48.6	6.47	3.55	hypothetical protein [Arthrobacter sp. 135MFCol5.1]
gi916782238	4.79	1	1	334	35.9	6.47	3.55	hypothetical protein [Arthrobacter sp. 35W]
gi652423751	6.55	1	1	290	30.6	5.16	3.55	shikimate dehydrogenase [Arthrobacter castelli]
gi723608297	5.85	1	1	359	38.7	5.17	3.54	inositol-3-phosphate synthase [Arthrobacter sp. PAMC25486]
gi908698227	4.26	1	1	516	56.2	6.20	3.53	(dimethylallyl)adenosine tRNA methylthiotransferase [Arthrobacter sp. RIT-PI-e]
gi119949802	5.00	1	1	200	21.7	10.05	3.53	site-specific recombinase, resolvase family [Arthrobacter aurescens TC1]
gi551254639	14.58	1	1	144	16.2	5.81	3.52	hypothetical protein [Arthrobacter sp. PAO19]
gi937259169	4.24	1	1	495	53.3	5.54	3.52	FAD-dependent oxidoreductase [Arthrobacter sp. Edens01]
gi759734715	3.06	1	1	360	38.2	9.55	3.52	dihydroorotate dehydrogenase 2 [Arthrobacter sp. L77]
gi651439964	14.18	1	1	134	14.5	10.51	3.51	hypothetical protein [Arthrobacter sp. H14]
gi910697359	13.95	1	1	86	9.2	9.52	3.50	ABC transporter, substrate binding protein [Arthrobacter sp. Hiyo6]
gi927032490	8.52	1	1	317	34.1	8.29	3.49	LysR family transcriptional regulator [Arthrobacter sp. LS16]
gi937259139	2.12	1	1	472	48.7	5.77	3.46	UDP-N-acetylmuramate--alanine ligase [Arthrobacter sp. Edens01]
gi759728158	2.02	2	1	593	62.9	5.58	3.42	ABC transporter [Arthrobacter sp. UNC362MFTsu5.1]
gi517604450	2.73	6	1	878	93.3	5.40	3.42	hypothetical protein [Arthrobacter sp. 131MFCol6.1]
gi910252315	1.64	1	1	917	99.3	4.97	3.42	alpha-L-rhamnosidase [Arthrobacter siccitolerans]
gi759708658	1.16	1	1	773	86.2	8.76	3.40	MULTISPECIES: GTP pyrophosphokinase [Arthrobacter]
gi651460825	7.41	2	1	135	14.6	4.50	3.39	glyoxalase [Arthrobacter sp. 35/47]
gi823665684	0.79	1	1	890	97.3	5.25	3.38	hypothetical protein AA310_06900 [Arthrobacter sp. YC-RL1]
gi927294007	2.16	2	1	462	50.5	5.85	3.36	phospho-2-dehydro-3-deoxyheptonate aldolase [Arthrobacter sp. ERGS1:01]
gi323471007	7.81	2	1	256	26.3	7.47	3.35	ABC-type dipeptide/oligopeptide/nickel transport system, ATPase component [Arthrobacter p
gi910252726	1.75	1	1	571	61.2	4.98	3.32	ABC transporter substrate-binding protein [Arthrobacter siccitolerans]
gi928488535	5.56	1	1	198	21.5	7.02	3.31	hypothetical protein AOC05_01015 [Arthrobacter alpinus]
gi910743433	8.99	2	1	189	21.3	5.34	3.30	tricorn protease homolog 1 [Arthrobacter sp. Hiyo8]
gi910696719	6.88	3	1	218	24.1	11.28	3.28	uncharacterized HTH-type transcriptional regulator YagI, partial [Arthrobacter sp. Hiyo6]
gi737788109	8.08	1	1	99	11.5	5.11	3.27	30S ribosomal protein S6 [Arthrobacter albus]
gi470215981	10.81	1	1	185	19.8	6.01	3.27	hypothetical protein ADIAG_03942 [Arthrobacter gangotriensis Lz1y]
gi651442632	0.51	1	1	1571	174.8	5.53	3.26	hypothetical protein [Arthrobacter sp. 9MFCol3.1]
gi918267307	12.42	1	1	161	16.8	9.04	3.25	ATP-dependent zinc metalloprotease FtsH [Arthrobacter sp. Hiyo1]
gi723608038	7.12	5	1	267	28.4	4.72	3.25	ABC-type metal ion transport system, periplasmic component/surface antigen [Arthrobacter
gi651430001	4.42	2	1	339	36.1	5.07	3.22	fructose-bisphosphate aldolase [Arthrobacter sanguinis]
gi403231944	3.77	4	1	478	52.2	5.73	3.22	Xaa-Pro aminopeptidase (plasmid) [Arthrobacter sp. Rue61a]

gi937262471	1.53	6	1	1048	108.2	4.54	3.17	hydrogenase expression protein [Arthrobacter sp. Edens01]
gi651490343	9.69	3	1	227	25.2	10.43	3.13	membrane protein [Arthrobacter sp. H20]
gi916926330	17.52	1	1	137	14.5	10.52	3.13	hypothetical protein [Arthrobacter sp. 9MFCol3.1]
gi119947489	7.28	1	1	206	23.5	10.93	3.07	ribosomal protein S4 [Arthrobacter aurescens TC1]
gi219859138	2.60	1	1	847	89.6	5.02	3.06	phenylalanyl-tRNA synthetase, beta subunit [Arthrobacter chlorophenolicus A6]
gi914715535	2.60	1	1	847	88.9	5.12	3.06	phenylalanine--tRNA ligase subunit beta [Arthrobacter sp. ZBG10]
gi916872160	4.13	1	1	387	42.1	6.07	3.05	hypothetical protein [Arthrobacter sp. H5]
gi757623028	4.15	6	1	458	49.7	5.31	3.05	oxidoreductase [Arthrobacter sp. SPG23]
gi470217785	2.52	1	1	555	58.2	5.17	3.04	amidohydrolase 3 [Arthrobacter gangotriensis Lz1y]
gi116612413	4.32	1	1	417	43.4	10.58	3.03	transcriptional regulator, LacI family [Arthrobacter sp. FB24]
gi916691870	2.11	1	1	997	106.8	5.59	3.02	FAD-linked oxidase [Arthrobacter castelli]
gi654811983	9.27	1	1	151	15.9	4.53	3.00	hypothetical protein [Arthrobacter sp. MA-N2]
gi470217502	9.28	1	1	194	20.9	9.69	3.00	serine O-acetyltransferase [Arthrobacter gangotriensis Lz1y]
gi910748827	7.33	1	1	191	20.4	5.11	2.98	S-adenosylmethionine synthase [Arthrobacter sp. Hiyo8]
gi916357998	2.54	1	1	670	70.7	6.18	2.98	MULTISPECIES: ATP-dependent helicase [Arthrobacter]
gi651447798	8.75	1	1	263	26.7	5.63	2.97	3-hydroxy-2-methylbutyryl-CoA dehydrogenase [Arthrobacter nicotinovorans]
gi908696957	4.12	3	1	340	36.4	9.82	2.97	cytochrome C oxidase assembly protein [Arthrobacter sp. RIT-PI-e]
gi359303579	4.45	1	1	292	32.4	4.77	2.96	hypothetical protein ARGLB_113_00560 [Arthrobacter globiformis NBRC 12137]
gi910748786	12.61	2	1	119	12.3	9.19	2.96	hypothetical protein AHiyo8_64880 [Arthrobacter sp. Hiyo8]
gi323470419	6.39	1	1	266	27.8	5.19	2.96	hypothetical protein Asphe3_29930 [Arthrobacter phenanthrenivorans Sphe3]
gi651488993	4.64	1	1	237	25.4	9.55	2.96	hypothetical protein, partial [Arthrobacter sp. H20]
gi359303439	11.40	1	1	193	21.2	10.89	2.96	thymidylate kinase [Arthrobacter globiformis NBRC 12137]
gi737800388	3.73	1	1	402	42.7	4.68	2.95	signal recognition particle-docking protein FtsY [Arthrobacter castelli]
gi307745899	8.60	1	1	221	24.0	6.06	2.95	two-component system response regulator [Arthrobacter arilaitensis Re117]
gi307744528	9.38	1	1	192	21.4	4.78	2.94	conserved hypothetical protein [Arthrobacter arilaitensis Re117]
gi403228954	6.51	1	1	292	31.0	5.59	2.93	putative acetyltransferase [Arthrobacter sp. Rue61a]
gi470217076	3.05	4	1	853	86.7	4.88	2.93	peptide ABC transporter permease [Arthrobacter gangotriensis Lz1y]
gi910696926	4.92	1	1	305	33.1	7.17	2.92	uncharacterized HTH-type transcriptional regulator YwbI [Arthrobacter sp. Hiyo6]
gi916872061	12.27	1	1	163	17.6	6.61	2.91	AsnC family transcriptional regulator [Arthrobacter sp. H5]
gi443480549	8.61	2	1	151	15.8	9.45	2.91	16S ribosomal RNA methyltransferase RsmE [Arthrobacter nitrophenolicus]
gi654813637	4.65	2	1	387	43.2	6.14	2.90	hypothetical protein [Arthrobacter sp. MA-N2]
gi518311759	8.99	1	1	189	20.2	4.92	2.90	phosphoribosylglycinamide formyltransferase [Arthrobacter sp. TB 23]
gi928487147	6.21	2	1	338	34.2	4.60	2.89	thiamine-monophosphate kinase [Arthrobacter alpinus]
gi823666748	4.26	2	1	470	49.3	4.87	2.87	aldehyde dehydrogenase [Arthrobacter sp. YC-RL1]
gi916782143	4.50	1	1	400	41.1	6.52	2.87	hypothetical protein [Arthrobacter sp. 35W]
gi740685469	2.03	2	1	543	57.3	5.49	2.87	acetolactate synthase [Arthrobacter sp. PAMC25486]
gi651462138	4.29	2	1	303	32.5	5.33	2.86	pantoate--beta-alanine ligase [Arthrobacter sp. 35/47]
gi759704880	6.77	3	1	251	28.5	5.73	2.85	ArsR family transcriptional regulator [Arthrobacter globiformis]
gi307744711	4.23	6	1	307	33.9	8.57	2.85	formamidopyrimidine-DNA glycosylase [Arthrobacter arilaitensis Re117]
gi543385185	8.14	1	1	307	31.6	6.21	2.84	prephenate dehydratase [Corynebacterium pseudodiphtheriticum 090104]
gi116609456	20.00	2	1	95	10.1	5.31	2.84	protein of unknown function DUF909 [Arthrobacter sp. FB24]
gi651499592	24.64	1	1	69	7.8	11.25	2.84	hypothetical protein [Arthrobacter sp. 35W]
gi654818729	6.49	1	1	231	22.8	5.33	2.83	calcium-binding protein [Arthrobacter sp. UNC362MFTsu5.1]
gi910696821	13.67	1	1	139	15.7	8.76	2.83	uncharacterized ABC transporter extracellular-binding protein PH1214, partial [Arthrobacter s
gi403228560	1.74	2	1	1205	130.7	5.44	2.83	hypothetical protein ARUE_c10610 [Arthrobacter sp. Rue61a]
gi910252222	5.04	2	1	417	46.6	5.30	2.83	creatininase [Arthrobacter siccitolerans]
gi651508035	4.15	4	1	434	46.2	5.40	2.82	ABC transporter substrate-binding protein [Arthrobacter sp. 35W]
gi674645614	2.65	1	1	377	40.6	5.36	2.82	UDP-N-acetylglucosamine 2-epimerase [Arthrobacter sp. 11W110_air]
gi765009036	2.45	4	1	572	61.4	7.37	2.82	peptide ABC transporter ATPase [Arthrobacter sp. A3]
gi916692276	3.48	2	1	575	59.8	9.55	2.81	hypothetical protein [Arthrobacter castelli]
gi654828002	3.33	1	1	240	26.2	5.31	2.81	hypothetical protein [Arthrobacter sp. H5]
gi517602231	4.70	1	1	362	39.1	5.76	2.81	threonine aldolase [Arthrobacter sp. 131MFCol6.1]
gi219860784	5.78	1	1	225	23.8	5.02	2.80	NUDIX hydrolase [Arthrobacter chlorophenolicus A6]
gi765008258	5.46	3	1	458	48.2	5.30	2.80	aspartate ammonia-lyase [Arthrobacter sp. A3]
gi916820249	2.65	1	1	490	51.2	4.75	2.80	hypothetical protein [Arthrobacter sp. H20]

gi928487038	2.82	1	1	497	51.5	5.58	2.79	aldehyde dehydrogenase [Arthrobacter alpinus]
gi937262349	2.75	3	1	436	45.0	5.21	2.79	chromosome partitioning protein [Arthrobacter sp. Edens01]
gi515766654	4.19	4	1	310	33.4	5.72	2.78	LysR family transcriptional regulator [Arthrobacter sp. M2012083]
gi757625802	6.15	1	1	195	21.4	5.33	2.78	adenylate kinase [Arthrobacter sp. SPG23]
gi470217443	7.18	2	1	181	20.0	4.35	2.78	16S rRNA processing protein [Arthrobacter gangotriensis Lz1y]
gi542110482	7.82	10	1	307	33.4	5.63	2.78	glmZ(sRNA)-inactivating NTPase [Arthrobacter sp. AK-YN10]
gi930826078	5.25	1	1	305	32.4	6.98	2.78	hypothetical protein AOZ07_07105 [Arthrobacter arilaitensis]
gi917013313	5.86	2	1	290	30.5	4.97	2.77	hypothetical protein [Arthrobacter sanguinis]
gi517603512	5.25	1	1	305	33.9	10.02	2.77	hypothetical protein [Arthrobacter sp. 131MFCol6.1]
gi908697902	10.20	2	1	147	16.1	5.20	2.77	heat-shock protein Hsp20 [Arthrobacter sp. RIT-PI-e]
gi737814164	3.64	2	1	385	40.1	5.02	2.77	hypothetical protein, partial [Arthrobacter sp. H14]
gi162953491	3.12	1	1	385	40.3	11.69	2.77	efflux ABC transporter, permease protein [Renibacterium salmoninarum ATCC 33209]
gi323468918	9.77	1	1	174	18.0	5.73	2.76	molybdopterin adenylyltransferase [Arthrobacter phenanthrenivorans Sphe3]
gi723606815	11.11	1	1	135	15.0	9.58	2.76	hypothetical protein ART_0592 [Arthrobacter sp. PAMC25486]
gi674645956	4.49	1	1	245	25.4	9.95	2.75	Glycine betaine/carnitine/choline transport system permease protein OpuCB [Arthrobacter sp
gi651501255	6.38	2	1	298	29.9	5.00	2.75	N-acetylmuramic acid 6-phosphate etherase [Arthrobacter sp. 35W]
gi640203272	1.23	3	1	1377	144.4	4.87	2.75	hypothetical protein [Arthrobacter sp. 31Y]
gi652423710	5.98	1	1	251	26.5	5.21	2.75	3-hydroxybutyrate dehydrogenase [Arthrobacter castelli]
gi749400970	6.01	1	1	183	19.1	5.31	2.74	phosphoribosylaminoimidazole synthetase, partial [Arthrobacter sp. AK-YN10]
gi910252630	4.80	1	1	479	48.8	5.36	2.74	3-carboxy-cis,cis-muconate cycloisomerase [Arthrobacter siccitolerans]
gi910695847	10.99	1	1	91	10.1	9.52	2.74	30S ribosomal protein S3 [Arthrobacter sp. Hiyo6]
gi470220102	8.85	1	1	192	20.5	5.21	2.74	N-acetyltransferase GCN5 [Arthrobacter gangotriensis Lz1y]
gi651444128	5.71	3	1	280	29.4	5.62	2.73	hypothetical protein [Arthrobacter nicotinovorans]
gi651483419	6.32	1	1	285	29.8	4.77	2.73	hypothetical protein [Arthrobacter sp. Br18]
gi517607385	2.95	1	1	509	54.9	6.23	2.73	hypothetical protein [Arthrobacter sp. 161MFSha2.1]
gi518313502	4.90	5	1	469	49.2	4.73	2.72	aldehyde dehydrogenase [Arthrobacter sp. TB 23]
gi823665611	6.50	1	1	246	27.5	8.21	2.72	ADP-ribose pyrophosphatase [Arthrobacter sp. YC-RL1]
gi517605914	4.52	1	1	221	23.7	9.98	2.72	MULTISPECIES: hypothetical protein [Arthrobacter]
gi937261966	2.16	3	1	417	44.7	8.76	2.72	lactate dehydrogenase [Arthrobacter sp. Edens01]
gi910748131	10.22	1	1	137	14.7	5.29	2.72	ribonuclease 3 [Arthrobacter sp. Hiyo8]
gi918267722	8.48	1	1	165	18.3	11.62	2.72	hypothetical protein AHiyo1_24560 [Arthrobacter sp. Hiyo1]
gi470217754	2.38	1	1	462	49.6	7.21	2.71	FAD dependent oxidoreductase [Arthrobacter gangotriensis Lz1y]
gi476401684	4.56	1	1	417	44.8	5.97	2.71	acyl-CoA dehydrogenase [Arthrobacter crystallopoietes BAB-32]
gi740685269	3.32	5	1	452	47.3	5.38	2.71	FAD-linked oxidase [Arthrobacter sp. PAMC25486]
gi916813681	3.14	3	1	382	40.5	8.07	2.71	hypothetical protein [Arthrobacter nicotinovorans]
gi823666224	3.69	2	1	407	43.8	7.28	2.71	lipase/esterase [Arthrobacter sp. YC-RL1]
gi651494084	3.36	1	1	476	51.5	9.14	2.71	transposase [Arthrobacter sp. H20]
gi640197967	3.48	1	1	489	51.4	8.91	2.71	MFS transporter [Arthrobacter sp. 31Y]
gi651481550	3.51	1	1	427	46.6	8.21	2.70	cyclopropane-fatty-acyl-phospholipid synthase [Arthrobacter sp. Br18]
gi928486808	3.20	2	1	469	51.1	7.25	2.70	transcriptional regulator [Arthrobacter alpinus]
gi116609642	2.90	2	1	552	56.8	5.00	2.70	thiamine pyrophosphate enzyme TPP binding domain protein [Arthrobacter sp. FB24]
gi759711222	7.95	2	1	176	18.2	7.12	2.69	macro domain-containing protein [Arthrobacter sp. 135MFCol5.1]
gi723607174	7.88	1	1	292	29.0	5.78	2.69	orotidine 5'-phosphate decarboxylase [Arthrobacter sp. PAMC25486]
gi937258415	7.59	1	1	316	33.3	4.96	2.69	hypothetical protein AO716_10170 [Arthrobacter sp. Edens01]
gi517590701	8.23	1	1	243	26.8	5.77	2.68	GntR family transcriptional regulator [Arthrobacter sp. 135MFCol5.1]
gi917530260	10.00	1	1	120	13.2	8.91	2.68	MerR family transcriptional regulator [Arthrobacter sp. PAMC25486]
gi674645705	3.12	2	1	321	33.2	4.73	2.68	Thioredoxin-1 [Arthrobacter sp. 11W110_air]
gi654822493	5.65	1	1	248	27.1	6.39	2.68	cobalt ABC transporter ATP-binding protein [Arthrobacter sp. I3]
gi116612624	19.39	1	1	98	10.5	9.69	2.68	transcriptional regulator, XRE family [Arthrobacter sp. FB24]
gi654812369	9.39	3	1	213	22.9	5.00	2.68	NADP oxidoreductase [Arthrobacter sp. MA-N2]
gi517600163	4.37	1	1	458	48.4	9.79	2.67	3-(2-hydroxyphenyl) propionic acid transporter [Arthrobacter sp. 162MFSha1.1]
gi908691081	1.44	1	1	834	90.5	5.34	2.67	hypothetical protein [Arthrobacter sp. H41]
gi674646875	4.99	2	1	421	44.3	5.25	2.67	N-carbamoyl-L-amino acid hydrolase [Arthrobacter sp. 11W110_air]
gi470221494	5.16	1	1	349	36.3	5.52	2.67	ABC transporter [Arthrobacter gangotriensis Lz1y]
gi359305549	10.53	1	1	209	23.0	8.84	2.67	hypothetical protein ARGLB_064_00990 [Arthrobacter globiformis NBRC 12137]

gi359304298	3.04	1	1	560	56.1	5.41	2.67	S1 family peptidase [Arthrobacter globiformis NBRC 12137]
gi918268572	15.09	1	1	106	10.8	7.59	2.67	2-succinyl-5-enolpyruvyl-6-hydroxy-3-cyclohexene-1-carboxylate synthase [Arthrobacter sp.
gi654816628	2.74	1	1	438	46.9	7.03	2.67	RNA polymerase subunit sigma-24 [Arthrobacter sp. UNC362MFTsu5.1]
gi767258175	6.00	1	1	300	33.4	8.76	2.66	hypothetical protein UM93_12055 [Arthrobacter sp. IHBB 11108]
gi823668639	5.01	1	1	519	57.7	5.20	2.66	lysyl-tRNA synthetase [Arthrobacter sp. YC-RL1]
gi918017349	4.73	1	1	296	33.3	10.02	2.66	hypothetical protein [Arthrobacter sp. FB24]
gi917022313	3.67	4	1	627	63.0	4.72	2.66	hypothetical protein [Arthrobacter sp. UNC362MFTsu5.1]
gi749401208	4.84	1	1	434	46.0	5.41	2.66	ABC transporter substrate-binding protein [Arthrobacter sp. AK-YN10]
gi737773732	3.36	3	1	387	42.1	8.43	2.66	DNA polymerase IV [Arthrobacter sp. MA-N2]
gi753938929	5.75	5	1	313	34.4	7.01	2.66	hypothetical protein [Arthrobacter phenanthrenivorans]
gi737789965	3.29	1	1	487	52.9	5.54	2.66	mycothione reductase [Arthrobacter albus]
gi219858140	2.69	1	1	633	66.1	9.01	2.65	hypothetical protein Achl_0483 [Arthrobacter chlorophenolicus A6]
gi765008661	2.04	1	1	636	66.9	4.83	2.65	NHL repeat-containing protein [Arthrobacter sp. A3]
gi767257798	4.65	2	1	301	33.5	9.54	2.65	pseudouridine synthase [Arthrobacter sp. IHBB 11108]
gi928486296	5.95	3	1	336	36.2	5.54	2.64	2-oxoisovalerate dehydrogenase [Arthrobacter alpinus]
gi765005702	2.43	1	1	575	60.5	5.14	2.64	DNA repair protein RecN [Arthrobacter sp. A3]
gi737782441	4.68	2	1	278	28.7	5.25	2.64	hypothetical protein [Arthrobacter sp. 35W]
gi737789934	1.67	1	1	718	78.8	5.11	2.64	ribosome-associated GTPase EngA [Arthrobacter albus]
gi767257276	7.24	3	1	304	33.5	6.35	2.64	acyl-CoA thioesterase [Arthrobacter sp. IHBB 11108]
gi470216505	3.48	1	1	517	56.3	7.09	2.64	hypothetical protein ADIAG_03494 [Arthrobacter gangotriensis Lz1y]
gi690772340	12.62	4	1	206	21.4	5.34	2.64	hypothetical protein HMPREF2128_07765 [Arthrobacter albus DNF00011]
gi551256519	2.34	1	1	856	92.3	6.61	2.64	DEAD/DEAH box helicase [Arthrobacter sp. PAO19]
gi470216531	12.50	1	1	128	13.4	8.76	2.64	hypothetical protein ADIAG_03146 [Arthrobacter gangotriensis Lz1y]
gi640194070	3.82	2	1	262	28.8	6.23	2.64	hypothetical protein [Arthrobacter sp. 31Y]
gi742072975	9.34	1	1	257	26.6	5.29	2.63	short-chain dehydrogenase/reductase SDR [Arthrobacter sp. MWB30]
gi765011384	1.31	2	1	996	110.3	5.07	2.63	alpha-mannosidase [Arthrobacter sp. A3]
gi640199795	8.13	1	1	246	26.3	5.15	2.63	GntR family transcriptional regulator [Arthrobacter sp. 31Y]
gi443482569	5.62	1	1	409	42.3	5.94	2.63	class V aminotransferase [Arthrobacter nitrophenolicus]
gi651505196	5.65	2	1	301	32.6	4.92	2.63	phosphoribosylaminoimidazole-succinocarboxamide synthase [Arthrobacter sp. 35W]
gi654814289	16.42	1	1	134	14.5	5.45	2.63	hypothetical protein [Arthrobacter sp. MA-N2]
gi640197725	2.38	1	1	757	80.2	9.60	2.63	copper resistance protein CopD [Arthrobacter sp. 31Y]
gi443480668	9.09	1	1	275	29.0	5.33	2.63	methionine aminopeptidase [Arthrobacter nitrophenolicus]
gi219860289	9.09	1	1	275	28.8	5.30	2.63	methionine aminopeptidase, type I [Arthrobacter chlorophenolicus A6]
gi918265738	2.78	1	1	503	57.3	9.77	2.63	putative uncharacterized protein YkfC [Arthrobacter sp. Hiyo1]
gi654823781	5.12	1	1	254	27.4	9.83	2.63	IclR family transcriptional regulator [Arthrobacter sp. I3]
gi359306637	5.68	2	1	317	32.8	5.15	2.62	putative fructokinase [Arthrobacter globiformis NBRC 12137]
gi651436423	3.86	2	1	233	24.9	6.57	2.62	cytidylate kinase [Arthrobacter sp. H41]
gi910697041	22.86	1	1	70	7.7	5.95	2.62	hypothetical protein AHiyo6_07230 [Arthrobacter sp. Hiyo6]
gi910741657	2.78	1	1	395	42.9	5.50	2.62	conserved hypothetical protein [Arthrobacter sp. Hiyo4]
gi476400856	8.45	1	1	213	24.0	9.60	2.62	hypothetical protein D477_011586 [Arthrobacter crystallopoietes BAB-32]
gi162954177	5.00	1	1	200	21.7	5.25	2.62	guanylate kinase [Renibacterium salmoninarum ATCC 33209]
gi651436124	9.01	2	1	222	23.8	6.65	2.62	potassium transporter [Arthrobacter sp. H41]
gi757625792	11.21	2	1	214	23.2	6.81	2.62	ArsR family transcriptional regulator [Arthrobacter sp. SPG23]
gi723609872	10.33	4	1	242	26.7	9.38	2.62	hypothetical protein ART_3649 [Arthrobacter sp. PAMC25486]
gi651481941	3.37	1	1	416	43.4	4.96	2.62	phosphoglycerate kinase [Arthrobacter sp. Br18]
gi916813760	9.41	1	1	255	27.2	10.98	2.61	phosphoesterase [Arthrobacter nicotinovorans]
gi652424373	2.29	1	1	785	85.8	5.30	2.61	hypothetical protein [Arthrobacter castelli]
gi742069927	4.65	1	1	344	35.6	5.78	2.61	oxidoreductase, 2-nitropropane dioxygenase family protein [Arthrobacter sp. MWB30]
gi651487046	9.05	1	1	243	25.9	6.29	2.61	hypothetical protein [Arthrobacter sp. Br18]
gi910693892	3.31	1	1	483	52.6	4.97	2.61	NADPH-ferredoxin reductase FprA [Arthrobacter sp. Hiyo6]
gi119950593	5.10	1	1	294	32.2	8.19	2.61	putative DNA-binding protein [Arthrobacter aurescens TC1]
gi542107826	12.04	1	1	191	20.9	5.99	2.61	hypothetical protein M707_15160 [Arthrobacter sp. AK-YN10]
gi640201828	4.04	1	1	322	33.6	4.93	2.61	exopolyphosphatase [Arthrobacter sp. 31Y]
gi470216728	6.96	1	1	316	32.9	4.84	2.61	anthranilate phosphoribosyltransferase [Arthrobacter gangotriensis Lz1y]
gi914713650	5.32	2	1	282	29.7	5.96	2.60	phenazine biosynthesis protein PhzF [Arthrobacter sp. ZBG10]

gi219862041	1.07	1	1	1401	151.8	5.27	2.60	DNA polymerase III, alpha subunit (plasmid) [Arthrobacter chlorophenolicus A6]
gi737793116	5.24	1	1	267	29.5	4.98	2.60	N-acyl homoserine lactonase [Arthrobacter nicotinovorans]
gi916869837	2.31	1	1	476	51.0	5.03	2.60	glucose-6-phosphate dehydrogenase [Arthrobacter sp. Br18]
gi742851573	9.39	3	1	213	22.4	4.63	2.60	septum formation inhibitor Maf [Arthrobacter sp. W1]
gi910745702	4.75	1	1	358	40.7	5.53	2.60	hypothetical protein AHiyo8_34040 [Arthrobacter sp. Hiyo8]
gi918265038	7.56	1	1	291	31.9	4.35	2.60	conserved hypothetical protein [Arthrobacter sp. Hiyo1]
gi651429705	5.26	1	1	171	18.7	6.23	2.60	hypothetical protein [Arthrobacter sanguinis]
gi759724242	4.34	4	1	461	48.3	5.19	2.59	reductase flavoprotein subunit [Arthrobacter sp. I3]
gi767257978	5.80	2	1	414	43.6	7.91	2.59	siroheme synthase CysG [Arthrobacter sp. IHBB 11108]
gi654815578	14.29	2	1	112	12.8	6.57	2.59	ArsR family transcriptional regulator [Arthrobacter sp. PAO19]
gi937259158	7.09	1	1	254	27.0	6.81	2.59	esterase [Arthrobacter sp. Edens01]
gi737774800	8.64	2	1	243	27.1	6.55	2.59	DNA-binding response regulator [Arthrobacter sp. MA-N2]
gi910738612	4.74	1	1	253	27.2	4.78	2.59	bifunctional protein GlmU [Arthrobacter sp. Hiyo4]
gi737812921	6.12	1	1	278	30.3	9.14	2.59	ABC transporter [Arthrobacter sp. H14]
gi470217919	2.61	1	1	345	35.3	9.88	2.59	benzoate transport protein [Arthrobacter gangotriensis Lz1y]
gi737809186	1.45	1	1	898	97.1	4.55	2.59	alpha-amylase [Arthrobacter sp. H5]
gi737785595	4.61	3	1	304	32.0	4.97	2.59	hypothetical protein [Arthrobacter nitrophenolicus]
gi307744793	11.74	1	1	213	22.3	6.68	2.59	putative transcriptional regulator [Arthrobacter arilaitensis Re117]
gi651501784	5.25	1	1	324	37.2	4.84	2.59	ribonucleotide-diphosphate reductase subunit beta [Arthrobacter sp. 35W]
gi219859086	20.22	1	1	89	10.3	9.99	2.58	ribosomal protein S15 [Arthrobacter chlorophenolicus A6]
gi162953552	4.30	3	1	302	32.4	5.11	2.58	glucosamine--fructose-6-phosphate aminotransferase (isomerizing) [Renibacterium salmonin
gi916876186	5.38	1	1	409	44.7	9.99	2.58	hypothetical protein [Arthrobacter sp. 31Y]
gi654818666	9.93	1	1	151	15.8	4.98	2.58	molybdenum cofactor biosynthesis protein MoaE [Arthrobacter sp. UNC362MFTsu5.1]
gi651501408	2.30	1	1	652	70.9	4.78	2.58	peptidase M13 [Arthrobacter sp. 35W]
gi470216245	6.07	1	1	346	36.6	5.41	2.58	gluconeogenesis factor [Arthrobacter gangotriensis Lz1y]
gi119951380	10.69	4	1	159	16.0	4.23	2.58	putative LysM domain protein [Arthrobacter aurescens TC1]
gi651497443	6.44	1	1	264	28.8	9.89	2.58	hypothetical protein [Arthrobacter sp. 35W]
gi823666919	8.59	1	1	256	28.2	5.36	2.58	IclR family transcriptional regulator [Arthrobacter sp. YC-RL1]
gi742851351	17.53	1	1	97	10.6	9.92	2.57	XRE family transcriptional regulator [Arthrobacter sp. W1]
gi742859399	11.84	1	1	152	16.6	5.47	2.57	glutamyl-tRNA amidotransferase [Arthrobacter sp. W1]
gi937256403	3.87	5	1	388	39.6	7.12	2.57	pyridine nucleotide-disulfide oxidoreductase [Arthrobacter sp. Edens01]
gi908698172	3.24	3	1	401	43.2	10.32	2.57	histidine kinase [Arthrobacter sp. RIT-PI-e]
gi742851929	7.60	1	1	250	27.1	6.93	2.57	GntR family transcriptional regulator [Arthrobacter sp. W1]
gi551255650	1.83	1	1	493	53.7	5.54	2.57	sugar phosphate isomerase [Arthrobacter sp. PAO19]
gi723608746	3.18	4	1	535	57.4	5.66	2.57	arabinose efflux permease family protein [Arthrobacter sp. PAMC25486]
gi916816399	2.72	1	1	514	56.0	6.68	2.57	acetyl-CoA hydrolase [Arthrobacter sp. MA-N2]
gi723607095	4.24	1	1	354	36.9	5.21	2.57	anthranilate phosphoribosyltransferase [Arthrobacter sp. PAMC25486]
gi651484496	4.98	1	1	281	31.0	5.10	2.57	UDP-glucose 4-epimerase [Arthrobacter sp. Br18]
gi742861338	4.82	1	1	249	27.5	11.37	2.57	membrane protein [Arthrobacter sp. W1]
gi742755913	2.56	1	1	468	48.3	5.67	2.57	branched-chain alpha-keto acid dehydrogenase subunit E2 [Arthrobacter phenanthrenivorans
gi651491320	4.32	2	1	417	46.3	5.85	2.56	cyclopropane-fatty-acyl-phospholipid synthase [Arthrobacter sp. H20]
gi640203379	11.22	1	1	196	21.6	4.84	2.56	adenylate kinase [Arthrobacter sp. 31Y]
gi918449396	3.29	1	1	395	43.2	6.16	2.56	hypothetical protein [Arthrobacter sp. SPG23]
gi916357913	4.66	1	1	322	35.0	6.29	2.56	GCN5 family acetyltransferase [Arthrobacter sp. 131MFCol6.1]
gi742755484	2.44	2	1	1025	107.8	8.63	2.56	monovalent cation/H+ antiporter subunit A [Arthrobacter phenanthrenivorans]
gi307744157	4.81	1	1	208	22.8	5.72	2.56	putative SAM-dependent methyltransferase [Arthrobacter arilaitensis Re117]
gi910697286	2.44	1	1	533	56.7	9.60	2.56	L-asparagine permease 2 [Arthrobacter sp. Hiyo6]
gi307743276	8.79	1	1	182	19.3	4.82	2.56	hypothetical secreted protein (plasmid) [Arthrobacter arilaitensis Re117]
gi723606519	5.54	1	1	271	28.7	5.19	2.56	indole-3-glycerol phosphate synthase [Arthrobacter sp. PAMC25486]
gi119948826	5.81	1	1	327	34.1	6.13	2.56	hypothetical protein AAur_2324 [Arthrobacter aurescens TC1]
gi916820226	0.92	2	1	1087	120.7	5.74	2.56	hypothetical protein [Arthrobacter sp. H20]
gi910744225	6.38	3	1	235	25.4	8.91	2.56	UDP-glucose 6-dehydrogenase TuaD [Arthrobacter sp. Hiyo8]
gi359307159	4.61	1	1	477	50.5	4.97	2.56	putative amidase [Arthrobacter globiformis NBRC 12137]
gi658509201	5.29	1	1	208	21.6	9.45	2.55	membrane protein [Arthrobacter sp. TB 26]
gi916782099	3.17	1	1	252	26.0	6.54	2.55	cation transporter [Arthrobacter sp. 35W]

gi908698566	4.45	1	1	427	44.6	5.16	2.55	Hrp-dependent type III effector protein [Arthrobacter sp. RIT-PI-e]
gi910744136	17.76	3	1	107	11.8	11.65	2.55	hypothetical protein AHiyo8_18380 [Arthrobacter sp. Hiyo8]
gi759734724	3.37	2	1	652	71.9	5.02	2.55	peptidase M13 [Arthrobacter sp. L77]
gi918267533	3.44	4	1	349	39.5	7.55	2.55	hypothetical protein AHiyo1_22200 [Arthrobacter sp. Hiyo1]
gi910737946	22.50	1	1	80	8.3	8.27	2.55	hypothetical protein AHiyo4_07680 [Arthrobacter sp. Hiyo4]
gi908697223	4.72	1	1	233	23.8	5.53	2.55	histidine phosphatase [Arthrobacter sp. RIT-PI-e]
gi916820619	4.35	2	1	322	35.0	5.54	2.55	response regulator receiver protein [Arthrobacter sp. H20]
gi937256367	5.67	1	1	194	21.3	4.89	2.55	hypothetical protein AO716_14910 [Arthrobacter sp. Edens01]
gi38200850	7.20	2	1	236	25.9	6.65	2.55	Conserved hypothetical protein [Corynebacterium diphtheriae]
gi737790167	2.69	4	1	595	63.6	5.86	2.55	iron ABC transporter ATP-binding protein [Arthrobacter albus]
gi654812985	6.15	1	1	195	22.0	11.59	2.55	HNH endonuclease [Arthrobacter sp. MA-N2]
gi403231903	22.22	1	1	45	5.5	9.99	2.55	hypothetical protein ARUE_232p01160 (plasmid) [Arthrobacter sp. Rue61a]
gi742071298	9.05	1	1	199	19.8	8.53	2.55	hypothetical protein ANMWB30_18240 [Arthrobacter sp. MWB30]
gi742070955	6.83	1	1	161	16.8	5.03	2.54	ybaK/ebsC protein [Arthrobacter sp. MWB30]
gi323470585	6.20	2	1	258	28.4	5.01	2.54	fructose-2,6-bisphosphatase [Arthrobacter phenanthrenivorans Sphe3]
gi723608234	1.12	1	1	1613	181.1	5.07	2.54	putative helicase [Arthrobacter sp. PAMC25486]
gi742852793	3.52	2	1	512	54.7	5.03	2.54	ATPase [Arthrobacter sp. W1]
gi765004213	3.87	1	1	491	51.6	10.14	2.54	MFS transporter [Arthrobacter sp. A3]
gi674646161	5.05	6	1	297	32.3	10.07	2.54	PAP2 superfamily protein [Arthrobacter sp. 11W110_air]
gi927033710	10.93	1	1	183	19.8	6.20	2.54	hypothetical protein AFL94_16960 [Arthrobacter sp. LS16]
gi307745705	12.75	3	1	149	16.5	5.43	2.54	hypothetical membrane protein [Arthrobacter arilaitensis Re117]
gi742752258	3.21	4	1	467	51.8	5.45	2.54	glucuronate isomerase [Arthrobacter phenanthrenivorans]
gi636846706	10.00	1	1	170	17.9	7.01	2.54	universal stress protein UspA [Arthrobacter sp. TB 26]
gi916781678	6.12	1	1	294	30.3	7.58	2.54	hypothetical protein [Arthrobacter sp. 35W]
gi910741089	3.89	1	1	437	46.7	5.38	2.54	putative binding protein BRA0748/BS1330_II0741 [Arthrobacter sp. Hiyo4]
gi823668020	3.51	1	1	427	44.6	4.91	2.54	allantoate amidohydrolase [Arthrobacter sp. YC-RL1]
gi916710599	3.42	1	1	438	47.0	5.95	2.54	hypothetical protein [Arthrobacter sp. CAL618]
gi823666275	4.82	1	1	249	27.6	11.18	2.54	membrane protein [Arthrobacter sp. YC-RL1]
gi918265814	16.51	1	1	109	12.5	6.29	2.54	hypothetical protein AHiyo1_43230 [Arthrobacter sp. Hiyo1]
gi518312377	3.82	1	1	498	53.8	5.19	2.54	hypothetical protein [Arthrobacter sp. TB 23]
gi823668321	6.98	1	1	258	27.4	5.86	2.54	D-beta-D-heptose 1-phosphate adenosyltransferase [Arthrobacter sp. YC-RL1]
gi765003943	9.09	4	1	132	14.4	9.98	2.54	hypothetical protein [Arthrobacter sp. A3]
gi767258458	2.76	2	1	544	57.8	5.17	2.54	phosphoglucomutase [Arthrobacter sp. IHBB 11108]
gi651464369	6.45	1	1	341	36.0	5.67	2.54	LacI family transcriptional regulator [Arthrobacter sp. 35/47]
gi651434482	3.03	1	1	396	43.6	5.24	2.53	acyl-CoA dehydrogenase [Arthrobacter sp. H41]
gi147829024	2.32	1	1	560	61.2	9.58	2.53	hypothetical protein pCM1_0013 (plasmid) [Clavibacter michiganensis subsp. michiganensis
gi654826824	5.36	1	1	280	30.0	9.85	2.53	hypothetical protein [Arthrobacter sp. H5]
gi517598073	5.80	1	1	276	30.1	6.62	2.53	S26 family signal peptidase [Arthrobacter sp. 162MFSha1.1]
gi910250411	16.67	3	1	84	9.3	4.59	2.53	excisionase [Arthrobacter siccitolerans]
gi737781056	6.34	1	1	410	42.0	9.83	2.53	ABC transporter permease [Arthrobacter sp. 35W]
gi359306658	4.17	2	1	408	43.2	5.02	2.53	hypothetical protein ARGLB_037_02150 [Arthrobacter globiformis NBRC 12137]
gi927032138	10.15	1	1	197	21.6	5.76	2.53	methyltransferase [Arthrobacter sp. LS16]
gi674644425	2.44	1	1	409	41.4	10.32	2.53	Inner membrane transport protein YnfM [Arthrobacter sp. 11W110_air]
gi916871812	6.89	4	1	334	34.8	7.65	2.53	DNA-binding protein [Arthrobacter sp. H5]
gi742072112	13.73	1	1	102	11.1	10.27	2.53	hypothetical protein ANMWB30_09470 [Arthrobacter sp. MWB30]
gi742070688	5.12	1	1	215	24.3	5.31	2.53	TetR family transcriptional regulator [Arthrobacter sp. MWB30]
gi910741949	7.20	2	1	236	24.7	6.35	2.53	N-acetylglucosamine-6-phosphate deacetylase [Arthrobacter sp. Hiyo4]
gi517604311	3.84	1	1	443	46.3	7.43	2.53	peptidase [Arthrobacter sp. 131MFCol6.1]
gi654811375	5.60	1	1	268	29.1	5.49	2.53	transglutaminase [Arthrobacter sp. MA-N2]
gi737812442	3.56	1	1	365	40.6	10.71	2.52	integrase [Arthrobacter sp. H14]
gi635351936	5.21	2	1	384	40.3	6.11	2.52	chorismate synthase [Arthrobacter siccitolerans]
gi937262072	17.39	2	1	92	10.1	4.51	2.52	antibiotic biosynthesis monooxygenase [Arthrobacter sp. Edens01]
gi939050412	4.59	1	1	370	41.3	6.46	2.52	hypothetical protein [Arthrobacter sp. JCM 19049]
gi767257327	6.17	8	1	308	33.8	9.63	2.52	recombinase XerC [Arthrobacter sp. IHBB 11108]
gi939050536	7.07	1	1	198	21.4	6.23	2.52	hypothetical protein [Arthrobacter sp. JCM 19049]

gi674644087	6.21	4	1	306	33.1	4.22	2.52	hypothetical protein BN1051_00229 [Arthrobacter sp. 11W110_air]
gi674646122	2.81	1	1	533	58.1	7.28	2.52	zeta-carotene-forming phytoene desaturase [Arthrobacter sp. 11W110_air]
gi25169031	10.49	2	1	162	18.1	5.15	2.52	putative chromosome partitioning protein (plasmid) [Arthrobacter nicotinovorans]
gi910283947	3.18	2	1	377	39.7	7.58	2.52	tRNA(Ile)-lysidine synthetase [Arthrobacter sp. A3]
gi919108052	4.23	1	1	331	36.5	8.57	2.52	hypothetical protein [Arthrobacter sp. IHBB 11108]
gi517605231	4.41	1	1	431	45.3	7.39	2.51	hypothetical protein [Arthrobacter sp. 131MFCol6.1]
gi542110621	4.58	4	1	262	27.0	6.39	2.51	cystathionine beta-lyase [Arthrobacter sp. AK-YN10]
gi651481493	10.58	2	1	189	19.4	4.75	2.51	FMN reductase [Arthrobacter sp. Br18]
gi927032264	5.17	5	1	445	46.0	9.47	2.51	3-ketoacyl-ACP reductase [Arthrobacter sp. LS16]
gi918265421	5.36	2	1	224	24.3	4.86	2.51	stilbene synthase 1 [Arthrobacter sp. Hiyo1]
gi648573355	4.46	1	1	314	32.5	5.45	2.51	diaminopimelate epimerase [Arthrobacter sp. 135MFCol5.1]
gi916863367	2.44	1	1	409	41.4	11.37	2.51	MFS transporter [Arthrobacter sp. 35/47]
gi757623814	4.26	2	1	305	32.0	9.58	2.51	diacylglycerol kinase [Arthrobacter sp. SPG23]
gi517601205	13.74	1	1	182	19.2	6.52	2.51	hypothetical protein [Arthrobacter sp. 162MFSha1.1]
gi542108295	5.91	1	1	220	23.9	5.55	2.51	hypothetical protein M707_12960 [Arthrobacter sp. AK-YN10]
gi517602216	6.11	2	1	458	48.4	7.06	2.51	SAM-dependent methyltransferase [Arthrobacter sp. 131MFCol6.1]
gi939036339	31.75	2	1	63	6.7	8.22	2.51	hypothetical protein [Arthrobacter nitroguajacolicus]
gi723608391	6.12	2	1	147	15.5	5.31	2.51	hypothetical protein ART_2168 [Arthrobacter sp. PAMC25486]
gi654811603	23.08	2	1	78	8.0	5.26	2.50	thiamine biosynthesis protein ThiS [Arthrobacter sp. MA-N2]
gi910742271	11.76	1	1	136	14.3	11.55	2.50	hypothetical protein AHiyo4_50930 [Arthrobacter sp. Hiyo4]
gi323470886	3.38	1	1	385	42.1	9.41	2.50	transposase [Arthrobacter phenanthrenivorans Sphe3]
gi116611493	2.46	1	1	406	45.1	5.68	2.50	aminotransferase [Arthrobacter sp. FB24]
gi759714979	4.47	1	1	403	42.8	7.15	2.50	phosphodiesterase [Arthrobacter sp. AK-YN10]
gi654826489	5.05	3	1	396	42.4	6.38	2.50	aminotransferase class V [Arthrobacter sp. H5]
gi786026644	5.98	1	1	368	39.8	5.15	2.50	carboxylate--amine ligase [Arthrobacter chlorophenolicus]
gi742855586	4.39	4	1	456	50.0	6.10	2.50	hypothetical protein [Arthrobacter sp. W1]
gi219859610	9.63	4	1	187	20.4	9.96	2.50	conserved hypothetical protein [Arthrobacter chlorophenolicus A6]
gi652423525	2.15	1	1	836	94.8	9.01	2.50	hypothetical protein [Arthrobacter castelli]
gi551255890	4.15	1	1	313	33.1	9.10	2.50	membrane protein [Arthrobacter sp. PAO19]
gi723606587	9.05	1	1	232	25.4	5.73	2.50	two component transcriptional regulator, LuxR family [Arthrobacter sp. PAMC25486]
gi908697219	4.26	1	1	376	38.8	9.50	2.50	hypothetical protein [Arthrobacter sp. RIT-PI-e]
gi162954562	6.76	1	1	207	21.8	5.05	2.50	riboflavin synthase, alpha chain [Renibacterium salmoninarum ATCC 33209]
gi759718209	21.95	1	1	82	8.8	4.22	2.50	hypothetical protein [Arthrobacter sp. FB24]
gi517606023	14.89	2	1	188	20.1	5.06	2.50	methyltransferase [Arthrobacter sp. 161MFSha2.1]
gi651445215	4.00	1	1	500	52.9	10.01	2.50	PA-phosphatase [Arthrobacter nicotinovorans]
gi517604583	11.39	1	1	202	22.0	6.54	2.49	XRE family transcriptional regulator [Arthrobacter sp. 131MFCol6.1]
gi307744722	4.22	1	1	569	59.5	6.93	2.49	putative EmrB/QacA subfamily drug resistance transporter [Arthrobacter arilaitensis Re117]
gi916869680	3.75	3	1	267	29.6	8.81	2.49	hypothetical protein [Arthrobacter sp. Br18]
gi927293178	5.30	1	1	132	14.6	5.30	2.49	hypothetical protein AL755_03120 (plasmid) [Arthrobacter sp. ERGS1:01]
gi908697718	3.80	1	1	526	56.1	4.94	2.49	transcriptional regulator [Arthrobacter sp. RIT-PI-e]
gi927032764	4.01	1	1	299	31.8	10.14	2.49	hypothetical protein AFL94_11130 [Arthrobacter sp. LS16]
gi917441776	4.97	4	1	302	33.8	4.88	2.49	hypothetical protein [Arthrobacter albus]
gi910695149	13.40	1	1	97	10.3	6.29	2.49	60 kDa chaperonin 2 [Arthrobacter sp. Hiyo6]
gi517589921	4.00	1	1	250	26.0	4.74	2.49	hypothetical protein [Arthrobacter sp. 135MFCol5.1]
gi937258978	8.03	1	1	274	29.7	7.05	2.49	GCN5 family acetyltransferase [Arthrobacter sp. Edens01]
gi651441369	3.28	1	1	670	73.7	4.97	2.49	hypothetical protein [Arthrobacter sp. 9MFCol3.1]
gi674644309	6.52	1	1	368	42.2	6.13	2.49	Region found in RelA / SpoT proteins [Arthrobacter sp. 11W110_air]
gi742852745	3.13	2	1	352	38.5	6.47	2.49	hypothetical protein [Arthrobacter sp. W1]
gi910693140	7.14	1	1	140	15.4	9.45	2.49	HTH-type transcriptional regulator AlsR, partial [Arthrobacter sp. Hiyo6]
gi323471534	2.25	1	1	668	72.8	6.34	2.49	hypothetical protein Asphe3_41530 (plasmid) [Arthrobacter phenanthrenivorans Sphe3]
gi928488494	3.89	1	1	180	19.5	9.95	2.49	hypothetical protein AOC05_18430 [Arthrobacter alpinus]
gi470220463	13.41	1	1	179	19.9	4.67	2.49	ferroxidase [Arthrobacter gangotriensis Lz1y]
gi927296159	2.56	2	1	546	58.1	6.32	2.49	hypothetical protein AL755_06235 [Arthrobacter sp. ERGS1:01]
gi737790423	12.82	1	1	156	17.2	10.30	2.49	30S ribosomal protein S7 [Arthrobacter albus]
gi651493542	2.11	1	1	475	49.5	7.01	2.48	hypothetical protein [Arthrobacter sp. H20]

gi914714102	8.42	1	1	273	27.8	5.22	2.48	hypothetical protein [Arthrobacter sp. ZBG10]
gi737782940	1.49	2	1	606	63.8	8.50	2.48	multidrug ABC transporter ATPase [Arthrobacter sp. 35W]
gi518312104	6.42	2	1	218	23.2	6.40	2.48	hypothetical protein [Arthrobacter sp. TB 23]
gi654816840	11.59	1	1	138	14.3	10.27	2.48	camphor resistance protein CrcB [Arthrobacter sp. UNC362MFTsu5.1]
gi759727559	4.90	1	1	286	29.6	8.88	2.48	ABC transporter permease [Arthrobacter sp. UNC362MFTsu5.1]
gi930826034	3.80	2	1	316	34.5	9.50	2.48	hypothetical protein AOZ07_06845 [Arthrobacter arilaitensis]
gi517590891	7.07	1	1	410	43.6	6.16	2.48	phosphodiesterase [Arthrobacter sp. 135MFCol5.1]
gi927295389	3.45	1	1	435	46.7	5.77	2.48	monooxygenase [Arthrobacter sp. ERGS1:01]
gi908697554	4.77	1	1	461	50.4	9.63	2.48	polyprenyl glycosylphosphotransferase [Arthrobacter sp. RIT-PI-e]
gi759772091	2.23	1	1	539	59.0	5.44	2.48	Pup deamidase/depupylase [Arthrobacter sp. SPG23]
gi674645417	1.12	2	1	1165	126.0	6.37	2.48	DNA polymerase III subunit alpha [Arthrobacter sp. 11W110_air]
gi908697824	5.32	6	1	376	39.6	5.57	2.48	GTPase [Arthrobacter sp. RIT-PI-e]
gi443482252	50.00	1	1	34	3.4	4.75	2.47	HAD-superfamily hydrolase [Arthrobacter nitrophenolicus]
gi654814744	9.26	1	1	108	11.5	7.42	2.47	hypothetical protein [Arthrobacter sp. MA-N2]
gi654825619	6.27	2	1	335	34.0	8.13	2.47	hypothetical protein [Arthrobacter sp. H5]
gi767257262	2.18	1	1	872	96.7	5.07	2.47	valine--tRNA ligase [Arthrobacter sp. IHBB 11108]
gi359306524	6.65	3	1	316	33.0	5.38	2.47	hydroxyacid oxidoreductase [Arthrobacter globiformis NBRC 12137]
gi470216008	4.90	1	1	204	21.8	11.94	2.47	hypothetical protein ADIAG_03969 [Arthrobacter gangotriensis Lz1y]
gi742759509	1.74	1	1	803	87.0	4.84	2.47	phosphoenolpyruvate synthase [Arthrobacter phenanthrenivorans]
gi742068729	2.85	1	1	667	68.7	4.91	2.47	beta-N-acetylglucosaminidase [Arthrobacter sp. MWB30]
gi651430831	9.76	1	1	205	22.3	5.00	2.47	dephospho-CoA kinase [Arthrobacter sanguinis]
gi651505239	4.27	1	1	328	34.3	5.85	2.47	ornithine cyclodeaminase [Arthrobacter sp. 35W]
gi742071319	6.47	2	1	309	33.8	5.44	2.47	inorganic polyphosphate/ATP-NAD kinase PpnK [Arthrobacter sp. MWB30]
gi937256450	9.35	2	1	139	15.4	9.29	2.47	cation transport regulator ChaB [Arthrobacter sp. Edens01]
gi757622367	4.86	1	1	370	39.9	6.20	2.47	ABC transporter [Arthrobacter sp. SPG23]
gi219858940	1.86	3	1	912	97.4	5.49	2.47	transcriptional regulator, LuxR family [Arthrobacter chlorophenolicus A6]
gi651432327	9.32	1	1	161	17.8	9.86	2.47	hypothetical protein [Arthrobacter sp. H41]
gi910251057	2.39	2	1	544	57.5	5.58	2.47	histidine kinase [Arthrobacter siccitolerans]
gi910744696	19.54	1	1	87	8.7	9.47	2.47	probable polyketide synthase 1 [Arthrobacter sp. Hiyo8]
gi651447030	8.47	3	1	295	31.9	7.36	2.47	XRE family transcriptional regulator [Arthrobacter nicotinovorans]
gi910283775	4.66	1	1	279	30.8	5.63	2.47	hypothetical protein [Arthrobacter sp. A3]
gi470220613	4.35	1	1	368	38.2	4.77	2.47	putative lipoprotein [Arthrobacter gangotriensis Lz1y]
gi916813737	4.20	3	1	357	39.2	9.96	2.46	hypothetical protein [Arthrobacter nicotinovorans]
gi723607977	6.27	1	1	255	26.0	5.69	2.46	putative electron transfer flavoprotein subunit beta [Arthrobacter sp. PAMC25486]
gi927032669	8.63	1	1	197	21.9	5.74	2.46	hypothetical protein AFL94_10550 [Arthrobacter sp. LS16]
gi916691583	3.30	3	1	394	42.0	5.40	2.46	hypothetical protein [Arthrobacter castelli]
gi737779487	6.23	1	1	257	27.1	4.93	2.46	SAM-dependent methyltransferase [Arthrobacter sp. CAL618]
gi517600623	2.57	1	1	428	45.4	9.26	2.46	hypothetical protein [Arthrobacter sp. 162MFSha1.1]
gi654816223	1.77	1	1	508	51.3	6.25	2.46	bifunctional ADP-dependent (S)-NAD(P)H-hydrate dehydratase/NAD(P)H-hydrate epimerase
gi652424059	4.53	1	1	265	27.7	4.55	2.46	hydroxypyruvate isomerase [Arthrobacter castelli]
gi927293387	6.27	2	1	303	33.0	8.24	2.46	LysR family transcriptional regulator (plasmid) [Arthrobacter sp. ERGS1:01]
gi737812148	8.25	1	1	194	22.1	10.99	2.46	hypothetical protein [Arthrobacter sp. H14]
gi517603741	2.24	1	1	402	44.6	6.13	2.46	hypothetical protein [Arthrobacter sp. 131MFCol6.1]
gi742853133	8.27	1	1	278	29.5	5.00	2.46	thiosulfate sulfurtransferase [Arthrobacter sp. W1]
gi910252271	2.86	1	1	420	44.7	6.46	2.46	transcriptional regulator [Arthrobacter siccitolerans]
gi551256227	4.33	2	1	416	44.4	5.02	2.46	3,4-dihydroxy-2-butanone-4-phosphate synthase [Arthrobacter sp. PAO19]
gi515765596	20.59	1	1	68	7.5	6.58	2.46	transcriptional regulator [Arthrobacter sp. M2012083]
gi551254174	4.90	1	1	306	33.7	8.34	2.46	hypothetical protein [Arthrobacter sp. PAO19]
gi759704796	6.56	1	1	183	19.7	11.05	2.46	hypothetical protein [Arthrobacter globiformis]
gi674643883	4.56	1	1	263	29.1	10.62	2.46	hypothetical protein BN1051_00019 [Arthrobacter sp. 11W110_air]
gi757622424	10.31	1	1	97	10.5	10.59	2.45	hypothetical protein TV39_20865 [Arthrobacter sp. SPG23]
gi643036657	14.72	1	1	163	17.8	8.57	2.45	CAZy families GT2 protein, partial [uncultured Arthrobacter sp.]
gi759746455	9.61	5	1	229	24.0	8.21	2.45	short-chain dehydrogenase [Arthrobacter sp. 31Y]
gi651438601	9.51	2	1	263	28.2	9.96	2.45	hypothetical protein [Arthrobacter sp. H14]
gi927033660	31.67	2	1	60	6.6	8.24	2.45	hypothetical protein AFL94_16665 [Arthrobacter sp. LS16]

gi910739083	2.00	1	1	650	66.7	7.50	2.45	UDP-N-acetylglucosamine--N-acetylmuramyl-(pentapeptide) pyrophosphoryl-undecaprenol N
gi759713924	2.61	1	1	536	58.3	5.55	2.45	ABC transporter substrate-binding protein [Arthrobacter sp. AK-YN10]
gi919107987	2.64	1	1	606	64.1	10.39	2.45	hypothetical protein [Arthrobacter sp. IHBB 11108]
gi640199628	13.07	3	1	199	20.6	7.15	2.45	3-hexulose-6-phosphate isomerase [Arthrobacter sp. 31Y]
gi765008390	7.80	1	1	218	23.1	4.89	2.45	pterin-4-alpha-carbinolamine dehydratase [Arthrobacter sp. A3]
gi403228029	4.92	3	1	305	32.3	5.95	2.45	hypothetical protein ARUE_c05160 [Arthrobacter sp. Rue61a]
gi651495858	10.24	2	1	166	18.2	4.86	2.45	polyketide cyclase [Arthrobacter sp. H20]
gi765005105	3.23	1	1	526	56.9	5.94	2.45	hypothetical protein [Arthrobacter sp. A3]
gi918265712	6.95	1	1	331	35.3	8.97	2.45	transcriptional regulator LsrR [Arthrobacter sp. Hiyo1]
gi916835203	7.62	1	1	302	31.9	5.47	2.45	prephenate dehydratase [Arthrobacter sp. H14]
gi786027696	10.24	1	1	166	18.4	5.01	2.45	polyketide cyclase [Arthrobacter chlorophenolicus]
gi759767544	2.55	2	1	666	74.3	6.42	2.45	cellulose synthase [Arthrobacter sp. SPG23]
gi219858416	4.78	1	1	293	33.0	5.80	2.45	aminoglycoside phosphotransferase [Arthrobacter chlorophenolicus A6]
gi917013335	6.01	2	1	283	29.9	5.15	2.45	indole-3-glycerol phosphate synthase [Arthrobacter sanguinis]
gi651482662	3.35	2	1	508	56.7	7.52	2.45	XRE family transcriptional regulator [Arthrobacter sp. Br18]
gi651446911	5.00	1	1	240	25.9	5.17	2.45	histidine kinase [Arthrobacter nicotinovorans]
gi765008538	2.29	1	1	437	46.9	4.81	2.44	aminotransferase [Arthrobacter sp. A3]
gi219858786	9.16	4	1	273	29.6	9.19	2.44	short-chain dehydrogenase/reductase SDR [Arthrobacter chlorophenolicus A6]
gi674644282	7.02	2	1	285	30.2	4.86	2.44	Putative phosphatase YwpJ [Arthrobacter sp. 11W110_air]
gi654828318	10.00	1	1	220	24.1	5.33	2.44	hypothetical protein [Arthrobacter sp. H5]
gi651507355	8.57	1	1	210	22.9	5.01	2.44	TetR family transcriptional regulator [Arthrobacter sp. 35W]
gi674644707	3.77	4	1	504	51.9	6.86	2.44	C4-dicarboxylate transport protein [Arthrobacter sp. 11W110_air]
gi914716120	1.30	1	1	847	86.1	7.56	2.44	PKD domain-containing protein [Arthrobacter sp. ZBG10]
gi759734196	6.92	1	1	260	27.3	8.81	2.44	potassium ABC transporter [Arthrobacter sp. L77]
gi517599284	4.17	1	1	408	41.0	5.31	2.44	hypothetical protein [Arthrobacter sp. 162MFSha1.1]
gi542109615	8.79	1	1	239	25.3	4.82	2.44	TetR family transcriptional regulator [Arthrobacter sp. AK-YN10]
gi786033441	3.22	2	1	404	42.9	6.05	2.44	dephospho-CoA kinase [Arthrobacter chlorophenolicus]
gi928486354	4.15	1	1	313	34.2	7.23	2.44	tRNA dimethylallyltransferase [Arthrobacter alpinus]
gi749400984	4.39	1	1	296	31.9	6.77	2.44	LuxR family transcriptional regulator, partial [Arthrobacter sp. AK-YN10]
gi359307792	2.21	3	1	678	71.4	5.49	2.44	putative oxidoreductase [Arthrobacter globiformis NBRC 12137]
gi651466771	5.83	1	1	223	23.7	6.34	2.44	TetR family transcriptional regulator [Arthrobacter sp. 35/47]
gi914716864	3.78	1	1	370	39.6	6.43	2.43	hypothetical protein [Arthrobacter sp. ZBG10]
gi916813714	8.13	1	1	283	29.2	4.82	2.43	dehydrogenase [Arthrobacter nicotinovorans]
gi219861224	4.17	1	1	503	54.3	5.39	2.43	5-carboxymethyl-2-hydroxymuconate semialdehyde dehydrogenase [Arthrobacter chloropher
gi737801159	2.65	1	1	528	57.8	5.07	2.43	peptidase M20 [Arthrobacter castelli]
gi654811296	2.11	3	1	616	66.4	9.57	2.43	hypothetical protein [Arthrobacter sp. MA-N2]
gi542106362	8.33	2	1	180	19.9	4.94	2.43	transcriptional regulator [Arthrobacter sp. AK-YN10]
gi759736809	13.79	2	1	145	15.2	5.29	2.43	molybdenum cofactor biosynthesis protein MoaE [Arthrobacter sp. L77]
gi639128933	17.86	1	1	84	9.2	9.63	2.43	hypothetical protein [Arthrobacter sp. CAL618]
gi742855149	8.40	3	1	262	28.5	6.04	2.43	hypothetical protein [Arthrobacter sp. W1]
gi914714546	4.43	3	1	316	33.5	5.10	2.43	hypothetical protein [Arthrobacter sp. ZBG10]
gi916359101	4.86	1	1	350	37.5	5.35	2.43	oxidoreductase [Arthrobacter sp. 135MFCol5.1]
gi916816292	7.07	2	1	283	31.2	5.25	2.43	ATPase [Arthrobacter sp. MA-N2]
gi307743562	4.26	1	1	376	42.1	6.15	2.43	23S rRNA (uracil-5-)-methyltransferase RumB [Arthrobacter arilaitensis Re117]
gi551255190	4.26	1	1	376	41.9	6.60	2.43	23S rRNA methyltransferase [Arthrobacter sp. PAO19]
gi760125100	10.00	2	1	270	28.4	5.14	2.43	Zn-dependent hydrolase [Arthrobacter aurescens]
gi476401548	19.61	1	1	102	11.3	4.53	2.43	hypothetical protein D477_008118 [Arthrobacter crystallopoietes BAB-32]
gi522097319	5.44	1	1	423	46.1	5.40	2.43	acyl-CoA dehydrogenase [Arthrobacter sp. 161MFSha2.1]
gi307746594	5.03	1	1	338	35.5	9.77	2.43	RHBT family transporter [Arthrobacter arilaitensis Re117]
gi928485884	2.29	1	1	393	43.1	6.04	2.43	hypothetical protein AOC05_00720 [Arthrobacter alpinus]
gi116610584	4.41	5	1	363	40.2	5.21	2.43	Nitrilase/cyanide hydratase and apolipoprotein N-acyltransferase [Arthrobacter sp. FB24]
gi910251770	4.28	3	1	327	33.9	5.40	2.43	NAD(P)H-quinone oxidoreductase [Arthrobacter siccitolerans]
gi307746295	7.53	2	1	372	39.9	10.43	2.43	putative aromatic-ring hydroxylase [Arthrobacter arilaitensis Re117]
gi910738120	12.41	1	1	145	15.0	6.93	2.43	uncharacterized oxidoreductase YisS [Arthrobacter sp. Hiyo4]
gi767257005	2.26	1	1	532	58.0	5.88	2.43	ABC transporter [Arthrobacter sp. IHBB 11108]

gi654812750	6.30	1	1	397	43.6	5.30	2.43	acyl-CoA dehydrogenase [Arthrobacter sp. MA-N2]
gi307745253	5.24	2	1	229	25.0	5.08	2.43	conserved hypothetical protein [Arthrobacter arilaitensis Re117]
gi517599326	5.49	1	1	437	43.1	5.92	2.43	hypothetical protein [Arthrobacter sp. 162MFSha1.1]
gi723607825	6.67	1	1	195	21.6	6.99	2.43	hypothetical protein ART_1602 [Arthrobacter sp. PAMC25486]
gi189038613	5.36	3	1	429	46.5	5.54	2.43	RecName: Full=Adenylosuccinate synthetase; Short=AMPSase; Short=AdSS; AltName: Full=
gi910696342	6.92	2	1	260	27.1	9.54	2.43	hypothetical protein AHiyo6_12660 [Arthrobacter sp. Hiyo6]
gi443482988	2.06	1	1	535	55.8	10.43	2.43	binding-protein-dependent transport system inner membrane protein, partial [Arthrobacter n
gi219858309	8.63	1	1	255	27.9	5.03	2.43	ANTAR domain protein with unknown sensor [Arthrobacter chlorophenolicus A6]
gi939051271	6.73	1	1	223	24.3	8.57	2.43	hypothetical protein [Arthrobacter sp. JCM 19049]
gi908690919	1.37	1	1	805	85.6	8.32	2.43	transcription accessory protein [Arthrobacter sp. H41]
gi654819665	7.79	1	1	244	26.7	4.96	2.43	DSBA oxidoreductase [Arthrobacter sp. UNC362MFTsu5.1]
gi823665505	10.17	2	1	236	25.0	5.01	2.42	hypothetical protein AA310_05785 [Arthrobacter sp. YC-RL1]
gi651477258	5.95	2	1	185	18.7	5.15	2.42	2-C-methyl-D-erythritol 2,4-cyclodiphosphate synthase [Arthrobacter nicotinovorans]
gi654811802	3.45	2	1	377	41.4	4.93	2.42	GCN5 family acetyltransferase [Arthrobacter sp. MA-N2]
gi403229067	9.70	2	1	237	25.4	6.07	2.42	putative C4-dicarboxylate response regulator DctR [Arthrobacter sp. Rue61a]
gi515768202	9.70	2	1	237	25.4	6.07	2.42	hypothetical protein [Arthrobacter sp. M2012083]
gi918268602	10.00	1	1	150	16.1	6.52	2.42	alcohol dehydrogenase [Arthrobacter sp. Hiyo1]
gi116610709	7.58	1	1	277	29.8	8.68	2.42	transcriptional regulator, IclR family [Arthrobacter sp. FB24]
gi323467818	5.84	1	1	377	40.7	6.42	2.42	uncharacterized conserved protein [Arthrobacter phenanthrenivorans Sphe3]
gi927294370	3.77	2	1	318	35.8	9.36	2.42	transposase [Arthrobacter sp. ERGS1:01]
gi119948710	2.76	1	1	507	55.0	5.44	2.42	aldehyde dehydrogenase (NAD) family protein [Arthrobacter aurescens TC1]
gi928487039	3.40	1	1	353	36.7	6.54	2.42	3-isopropylmalate dehydrogenase [Arthrobacter alpinus]
gi639129560	5.24	1	1	286	30.6	5.67	2.42	hypothetical protein, partial [Arthrobacter sp. CAL618]
gi639128882	10.81	1	1	111	12.1	9.77	2.42	hypothetical protein [Arthrobacter sp. CAL618]
gi937262362	5.00	2	1	300	33.8	6.61	2.42	glycosyl transferase [Arthrobacter sp. Edens01]
gi914715016	3.44	1	1	640	66.7	6.54	2.42	GTP cyclohydrolase [Arthrobacter sp. ZBG10]
gi651504586	4.35	3	1	368	37.9	6.86	2.42	hypothetical protein [Arthrobacter sp. 35W]
gi651429520	3.64	1	1	412	42.1	4.55	2.42	cell division protein FtsZ [Arthrobacter sanguinis]
gi359307222	7.48	1	1	214	23.0	4.84	2.42	hypothetical protein ARGLB_023_00300 [Arthrobacter globiformis NBRC 12137]
gi917022425	4.87	2	1	431	44.9	5.53	2.42	hypothetical protein [Arthrobacter sp. UNC362MFTsu5.1]
gi651454821	4.72	1	1	318	34.8	9.23	2.42	sugar ABC transporter permease [Arthrobacter nicotinovorans]
gi742759352	20.29	2	1	69	7.5	5.38	2.42	hypothetical protein RM50_01220 [Arthrobacter phenanthrenivorans]
gi119947592	2.97	2	1	471	50.5	6.14	2.42	putative signal transduction histidine kinase [Arthrobacter aurescens TC1]
gi636847449	5.00	2	1	400	42.1	6.01	2.42	chorismate synthase [Arthrobacter sp. TB 26]
gi640194298	7.97	1	1	138	15.0	5.48	2.41	hypothetical protein [Arthrobacter sp. 31Y]
gi917759903	8.26	2	1	218	22.3	6.54	2.41	sulfurtransferase [Arthrobacter sp. L77]
gi327165072	5.41	3	1	388	43.0	5.82	2.41	mimosinase [Arthrobacter sp. Ryudai-S1]
gi937262654	3.49	1	1	401	41.2	9.96	2.41	MFS transporter [Arthrobacter sp. Edens01]
gi742855359	5.35	1	1	449	48.2	6.84	2.41	two-component system sensor histidine kinase [Arthrobacter sp. W1]
gi654818977	3.13	1	1	607	65.7	5.66	2.41	acetyl-CoA synthetase [Arthrobacter sp. UNC362MFTsu5.1]
gi654825696	10.17	1	1	118	13.3	9.74	2.41	hypothetical protein [Arthrobacter sp. H5]
gi307745434	25.58	1	1	86	8.7	5.21	2.41	thiamineS [Arthrobacter arilaitensis Re117]
gi937261823	9.95	2	1	211	23.4	9.67	2.41	hypothetical protein AO716_00860 [Arthrobacter sp. Edens01]
gi517591984	9.32	1	1	311	32.7	5.31	2.41	deoxyribose-phosphate aldolase [Arthrobacter sp. 135MFCol5.1]
gi443480914	2.60	1	1	423	43.9	5.48	2.41	proteinase inhibitor I4 serpin [Arthrobacter nitrophenolicus]
gi639131020	5.49	1	1	328	36.4	5.19	2.41	GNAT family acetyltransferase [Arthrobacter sp. CAL618]
gi651501550	1.61	1	1	685	72.8	6.98	2.41	primosomal protein N' [Arthrobacter sp. 35W]
gi916834493	4.74	1	1	274	30.0	8.98	2.41	hypothetical protein [Arthrobacter sp. H14]
gi916820150	7.04	2	1	199	22.4	6.54	2.41	hypothetical protein [Arthrobacter sp. H20]
gi765013249	8.01	1	1	287	31.1	6.43	2.41	XRE family transcriptional regulator [Arthrobacter sp. A3]
gi654814508	3.25	1	1	461	48.3	5.06	2.41	succinate-semialdehyde dehydrogenase [Arthrobacter sp. MA-N2]
gi658509348	3.85	2	1	234	26.5	5.20	2.41	hypothetical protein [Arthrobacter sp. TB 26]
gi403311741	1.31	1	1	1141	126.0	6.13	2.41	putative type II restriction enzyme, methylase subunit (plasmid) [Arthrobacter sp. Rue61a]
gi476399762	5.78	1	1	294	31.5	9.36	2.41	ABC-2 type transporter [Arthrobacter crystallopoietes BAB-32]
gi910738580	4.27	1	1	492	53.6	5.01	2.41	adenosylhomocysteinase [Arthrobacter sp. Hiyo4]

gi939051736	9.26	1	1	162	17.7	11.66	2.41	hypothetical protein [Arthrobacter sp. JCM 19049]
gi654824465	12.98	1	1	131	14.2	5.11	2.41	hypothetical protein [Arthrobacter sp. I3]
gi759704261	4.68	2	1	299	30.3	5.33	2.41	2-hydroxy-3-oxopropionate reductase [Arthrobacter globiformis]
gi652422781	4.17	1	1	312	33.7	6.42	2.40	hypothetical protein [Arthrobacter castelli]
gi914715882	3.99	1	1	451	48.0	5.57	2.40	serine hydroxymethyltransferase [Arthrobacter sp. ZBG10]
gi767258586	4.18	1	1	287	30.1	11.41	2.40	hypothetical protein UM93_14955 [Arthrobacter sp. IHBB 11108]
gi654817827	3.45	2	1	464	50.9	9.29	2.40	MFS transporter [Arthrobacter sp. UNC362MFTsu5.1]
gi930827085	3.13	1	1	416	44.3	6.77	2.40	type II/IV secretion system protein E [Arthrobacter arilaitensis]
gi916871262	5.79	4	1	328	35.1	7.02	2.40	hypothetical protein [Arthrobacter sp. H5]
gi823667498	5.86	2	1	239	26.0	9.54	2.40	teichoic acid ABC transporter ATP-binding protein [Arthrobacter sp. YC-RL1]
gi323469839	7.76	1	1	245	26.8	4.86	2.40	haloacid dehalogenase superfamily enzyme, subfamily IA [Arthrobacter phenanthrenivorans
gi116613124	6.25	1	1	256	27.4	5.06	2.40	Cobyrinic acid a,c-diamide synthase (plasmid) [Arthrobacter sp. FB24]
gi648573264	19.83	3	1	116	12.7	8.59	2.40	hypothetical protein [Arthrobacter sp. 135MFCol5.1]
gi116611824	5.69	2	1	246	26.6	5.15	2.40	putative GAF sensor protein [Arthrobacter sp. FB24]
gi443483043	3.33	3	1	660	69.8	7.77	2.40	putative amino acid transporter [Arthrobacter nitrophenolicus]
gi759764370	2.79	1	1	680	72.2	5.40	2.40	anthranilate synthase [Arthrobacter gangotriensis]
gi910748047	5.08	2	1	197	20.7	5.96	2.40	cysteine desulfurase [Arthrobacter sp. Hiyo8]
gi927031639	6.05	1	1	380	39.8	5.22	2.40	hypothetical protein AFL94_04100 [Arthrobacter sp. LS16]
gi910743055	5.75	1	1	226	24.9	5.87	2.40	uncharacterized HTH-type transcriptional regulator YdhC [Arthrobacter sp. Hiyo8]
gi651431227	4.62	2	1	346	37.3	5.01	2.40	2-hydroxyacid dehydrogenase [Arthrobacter sanguinis]
gi651440584	12.62	2	1	103	12.0	10.48	2.40	hypothetical protein [Arthrobacter sp. H14]
gi914716213	2.13	1	1	1171	121.5	5.17	2.40	hypothetical protein [Arthrobacter sp. ZBG10]
gi119951674	11.67	1	1	120	13.3	8.63	2.40	putative transcriptional regulator, ArsR family (plasmid) [Arthrobacter aurescens TC1]
gi652422825	14.09	1	1	149	16.6	5.03	2.40	DNA starvation/stationary phase protection protein [Arthrobacter castelli]
gi910740226	7.98	1	1	213	21.2	9.28	2.40	hypothetical protein AHiyo4_30480 [Arthrobacter sp. Hiyo4]
gi823666286	3.08	1	1	454	47.1	5.24	2.40	histidinol dehydrogenase [Arthrobacter sp. YC-RL1]
gi910248884	8.26	1	1	121	13.7	11.53	2.40	heat-shock protein [Arthrobacter siccitolerans]
gi757625047	2.98	1	1	504	54.3	9.76	2.40	3-methyladenine DNA glycosylase [Arthrobacter sp. SPG23]
gi648573005	5.30	1	1	472	50.7	4.94	2.40	phosphomannomutase [Arthrobacter sp. 135MFCol5.1]
gi928487385	1.49	1	1	805	87.7	5.39	2.40	salicylyl-CoA 5-hydroxylase [Arthrobacter alpinus]
gi323470444	7.47	1	1	241	25.2	5.48	2.40	response regulator containing a CheY-like receiver domain and an HTH DNA-binding domain
gi723609648	7.58	2	1	211	23.1	6.34	2.39	hypothetical protein ART_3425 [Arthrobacter sp. PAMC25486]
gi651502518	4.61	3	1	347	36.2	5.16	2.39	glycerol-3-phosphate dehydrogenase [Arthrobacter sp. 35W]
gi640201840	4.81	1	1	312	32.7	7.14	2.39	diacylglycerol kinase [Arthrobacter sp. 31Y]
gi219858231	3.10	1	1	516	53.8	5.39	2.39	cytidyltransferase-related domain protein [Arthrobacter chlorophenolicus A6]
gi927031683	8.27	1	1	254	27.2	5.22	2.39	glycosyl transferase family 8 [Arthrobacter sp. LS16]
gi443480682	4.62	2	1	411	44.0	9.51	2.39	glycosyl hydrolase [Arthrobacter nitrophenolicus]
gi723606570	4.35	1	1	437	47.5	5.94	2.39	ferredoxin reductase [Arthrobacter sp. PAMC25486]
gi928488025	4.43	1	1	429	45.9	6.74	2.39	histidine kinase [Arthrobacter alpinus]
gi910695299	7.43	1	1	148	15.4	7.69	2.39	glutamyl-tRNA(Gln) amidotransferase subunit A, partial [Arthrobacter sp. Hiyo6]
gi674644943	4.21	2	1	214	23.1	5.36	2.39	Trans-aconitate 2-methyltransferase [Arthrobacter sp. 11W110_air]
gi927293137	3.04	1	1	428	47.4	8.84	2.39	hypothetical protein AL755_02820 (plasmid) [Arthrobacter sp. ERGS1:01]
gi636847316	7.76	1	1	232	25.4	6.25	2.39	hypothetical protein [Arthrobacter sp. TB 26]
gi765006233	6.20	2	1	355	38.5	6.87	2.39	cytochrome P450 [Arthrobacter sp. A3]
gi323468653	6.14	1	1	342	37.5	6.06	2.39	tryptophanyl-tRNA synthetase [Arthrobacter phenanthrenivorans Sphe3]
gi551256178	5.05	1	1	297	33.2	6.55	2.39	glmZ(sRNA)-inactivating NTPase [Arthrobacter sp. PAO19]
gi640197296	5.71	1	1	245	25.9	5.44	2.39	TetR family transcriptional regulator [Arthrobacter sp. 31Y]
gi759763592	4.99	2	1	361	40.4	5.12	2.39	peptide chain release factor 1 [Arthrobacter gangotriensis]
gi517606255	3.05	2	1	689	72.9	6.25	2.39	NADH:flavin oxidoreductase [Arthrobacter sp. 161MFSha2.1]
gi916872188	7.55	1	1	265	29.4	9.33	2.39	hypothetical protein [Arthrobacter sp. H5]
gi116612314	6.36	1	1	346	37.1	5.29	2.39	protein of unknown function DUF21 [Arthrobacter sp. FB24]
gi518312073	4.34	2	1	438	45.6	5.00	2.39	hypothetical protein [Arthrobacter sp. TB 23]
gi119948430	11.90	2	1	126	13.2	5.48	2.39	hypothetical protein AAur_0450 [Arthrobacter aurescens TC1]
gi759721703	26.92	3	1	78	8.4	10.36	2.39	hypothetical protein [Arthrobacter nicotinovorans]
gi162953477	5.53	2	1	217	22.9	9.42	2.39	signal peptidase I [Renibacterium salmoninarum ATCC 33209]

gi910737909	7.84	1	1	204	22.5	5.67	2.39	probable transcriptional regulatory protein TcrX [Arthrobacter sp. Hiyo4]
gi916813747	9.62	1	1	208	23.3	6.98	2.39	TetR family transcriptional regulator [Arthrobacter nicotinovorans]
gi927033872	9.13	6	1	230	25.6	6.81	2.39	hypothetical protein AFL94_01605 [Arthrobacter sp. LS16]
gi640200444	6.86	1	1	204	22.5	6.14	2.39	TetR family transcriptional regulator [Arthrobacter sp. 31Y]
gi652425335	2.88	1	1	625	67.0	5.97	2.39	hypothetical protein [Arthrobacter castelli]
gi636846066	5.67	1	1	406	44.6	5.33	2.39	methionine synthase [Arthrobacter sp. TB 26]
gi765013745	3.93	2	1	331	34.8	5.10	2.39	universal stress protein UspA [Arthrobacter sp. A3]
gi654812930	11.50	1	1	113	12.1	5.21	2.39	nitrogen regulatory protein P-II 1 [Arthrobacter sp. MA-N2]
gi723607742	7.76	1	1	335	34.3	6.01	2.39	hypothetical protein ART_1519 [Arthrobacter sp. PAMC25486]
gi737814707	5.58	1	1	233	25.4	9.69	2.39	cell division protein FtsE [Arthrobacter sp. H14]
gi760125709	7.48	1	1	214	23.8	4.65	2.39	hypothetical protein [Arthrobacter aurescens]
gi219860348	3.10	1	1	323	35.0	5.19	2.39	mycothiol biosynthesis acetyltransferase [Arthrobacter chlorophenolicus A6]
gi742857135	4.26	1	1	329	35.3	9.92	2.39	hypothetical protein [Arthrobacter sp. W1]
gi757622855	4.08	1	1	294	31.8	6.16	2.39	aminoglycoside phosphotransferase [Arthrobacter sp. SPG23]
gi823666178	15.33	1	1	150	15.8	5.96	2.39	hypothetical protein AA310_09940 [Arthrobacter sp. YC-RL1]
gi654813997	10.04	1	1	269	29.5	5.40	2.38	rRNA methyltransferase [Arthrobacter sp. MA-N2]
gi654815117	3.99	1	1	401	43.8	5.02	2.38	acyl-CoA dehydrogenase [Arthrobacter sp. PAO19]
gi742858673	3.85	1	1	390	41.7	9.64	2.38	hypothetical protein [Arthrobacter sp. W1]
gi307744998	2.24	2	1	670	74.4	5.00	2.38	peptidyl-dipeptidase [Arthrobacter arilaitensis Re117]
gi910248908	5.48	1	1	310	31.2	5.63	2.38	threonine dehydratase [Arthrobacter siccitolerans]
gi917442366	3.11	1	1	515	57.2	8.38	2.38	cytochrome C biogenesis protein ResB [Arthrobacter albus]
gi116611557	5.02	1	1	319	35.2	9.60	2.38	conserved hypothetical protein [Arthrobacter sp. FB24]
gi511534834	12.78	1	1	133	15.0	11.88	2.38	hypothetical protein (plasmid) [Arthrobacter nicotinovorans]
gi654812027	5.08	1	1	354	38.2	9.99	2.38	cytochrome C biogenesis protein [Arthrobacter sp. MA-N2]
gi918268170	14.19	1	1	155	17.4	7.72	2.38	hypothetical protein AHiyo1_15210 [Arthrobacter sp. Hiyo1]
gi443480186	3.40	1	1	471	53.5	5.07	2.38	hypothetical protein G205_20133 [Arthrobacter nitrophenolicus]
gi651435604	3.48	1	1	316	33.3	5.17	2.38	ABC transporter [Arthrobacter sp. H41]
gi518312615	6.27	1	1	303	31.3	6.10	2.38	hypothetical protein [Arthrobacter sp. TB 23]
gi767259171	1.35	1	1	592	64.4	5.03	2.38	hypothetical protein UM93_10745 [Arthrobacter sp. IHBB 11108]
gi640200226	2.75	1	1	472	51.8	5.73	2.38	diguanylate phosphodiesterase [Arthrobacter sp. 31Y]
gi651444234	3.35	1	1	328	35.3	5.43	2.38	dehydrogenase [Arthrobacter nicotinovorans]
gi764161608	7.09	1	1	127	14.2	11.31	2.38	hypothetical protein ArV1_006 [Arthrobacter phage vB_ArtM-ArV1]
gi910696533	4.44	1	1	270	29.7	11.30	2.38	hypothetical protein AHiyo6_11180 [Arthrobacter sp. Hiyo6]
gi939036117	7.11	1	1	197	22.4	9.29	2.38	hypothetical protein [Arthrobacter nitroguajacolicus]
gi116609621	1.96	1	1	562	61.2	5.73	2.38	ATP-dependent DNA helicase, RecQ family [Arthrobacter sp. FB24]
gi476400890	8.73	1	1	275	28.3	5.17	2.38	flagellin domain-containing protein [Arthrobacter crystallopoietes BAB-32]
gi652423898	3.61	1	1	332	36.8	8.90	2.38	hypothetical protein [Arthrobacter castelli]
gi162955494	2.32	2	1	560	62.0	5.12	2.38	ABC transporter ATP-binding protein [Renibacterium salmoninarum ATCC 33209]
gi937256733	4.95	2	1	424	42.8	10.74	2.38	MFS transporter [Arthrobacter sp. Edens01]
gi651429242	1.98	1	1	808	86.0	5.44	2.38	hypothetical protein [Arthrobacter sanguinis]
gi219859703	13.42	2	1	149	15.1	7.09	2.38	UspA domain protein [Arthrobacter chlorophenolicus A6]
gi635350591	6.57	1	1	274	29.9	4.94	2.38	aldo/keto reductase family protein [Arthrobacter siccitolerans]
gi119947778	3.96	1	1	454	49.0	9.60	2.38	putative glucosyltransferase [Arthrobacter aurescens TC1]
gi767258213	2.75	3	1	619	67.3	5.26	2.38	hypothetical protein UM93_12335 [Arthrobacter sp. IHBB 11108]
gi742851781	4.79	3	1	501	52.2	5.36	2.38	peptidase S8 [Arthrobacter sp. W1]
gi742758475	5.46	1	1	403	42.2	5.67	2.37	CoA-transferase [Arthrobacter phenanthrenivorans]
gi928486509	32.31	1	1	65	7.0	9.98	2.37	hypothetical protein AOC05_05090 [Arthrobacter alpinus]
gi910741537	5.46	1	1	238	25.4	8.70	2.37	uncharacterized HTH-type transcriptional regulator PH0140 [Arthrobacter sp. Hiyo4]
gi406689804	2.06	1	1	388	42.9	5.36	2.37	xylose isomerase [Streptomyces sp. SM8]
gi759773096	1.55	1	1	1358	141.6	7.39	2.37	hypothetical protein [Arthrobacter sp. SPG23]
gi651438986	2.55	1	1	548	60.8	9.10	2.37	membrane protein [Arthrobacter sp. H14]
gi786027543	6.21	1	1	322	33.0	6.06	2.37	hypothetical protein [Arthrobacter chlorophenolicus]
gi219859590	6.46	1	1	356	38.0	9.29	2.37	Dihydroorotate oxidase [Arthrobacter chlorophenolicus A6]
gi654828015	3.62	1	1	276	31.0	5.43	2.37	hypothetical protein [Arthrobacter sp. H5]
gi636843850	3.20	1	1	344	36.4	5.41	2.37	butanediol dehydrogenase [Arthrobacter sp. TB 26]

gi674645697	8.56	1	1	187	20.6	4.89	2.37	hypothetical protein BN1051_01892 [Arthrobacter sp. 11W110_air]
gi476403005	8.44	1	1	237	26.3	5.81	2.37	GntR family transcriptional regulator [Arthrobacter crystallopoietes BAB-32]
gi651437000	9.88	1	1	253	27.6	5.88	2.37	GlcNAc-PI de-N-acetylase [Arthrobacter sp. H41]
gi651475706	6.34	1	1	268	26.7	4.82	2.37	molybdate-binding protein [Arthrobacter nicotinovorans]
gi759769467	3.34	1	1	509	54.9	9.13	2.37	transposase [Arthrobacter sp. SPG23]
gi639130233	3.04	1	1	428	46.0	6.40	2.37	hypothetical protein [Arthrobacter sp. CAL618]
gi928488366	2.18	1	1	550	56.9	5.06	2.37	hypothetical protein AOC05_17500 [Arthrobacter alpinus]
gi914716585	2.43	1	1	371	39.0	6.86	2.37	phospho-2-dehydro-3-deoxyheptonate aldolase [Arthrobacter sp. ZBG10]
gi910250111	1.59	1	1	1072	111.0	5.97	2.37	hydrogenase expression protein [Arthrobacter siccitolerans]
gi930825831	3.90	1	1	385	39.3	4.84	2.37	hypothetical protein AOZ07_05685 [Arthrobacter arilaitensis]
gi908697156	7.31	1	1	301	33.3	6.11	2.37	restriction endonuclease [Arthrobacter sp. RIT-PI-e]
gi910738082	3.09	1	1	356	38.1	9.10	2.37	uncharacterized ABC transporter ATP-binding protein YdiF [Arthrobacter sp. Hiyo4]
gi518313156	4.08	2	1	417	44.1	4.21	2.37	hypothetical protein [Arthrobacter sp. TB 23]
gi651502299	2.52	2	1	476	51.6	7.05	2.37	glyceraldehyde-3-phosphate dehydrogenase [Arthrobacter sp. 35W]
gi674644564	5.41	1	1	333	35.1	5.27	2.37	Bifunctional protein FolD protein [Arthrobacter sp. 11W110_air]
gi908690794	6.38	2	1	188	20.0	5.03	2.37	TetR family transcriptional regulator [Arthrobacter sp. H41]
gi654812742	6.91	2	1	275	28.8	6.57	2.37	SDR family oxidoreductase [Arthrobacter sp. MA-N2]
gi119949371	5.13	2	1	409	43.7	6.96	2.37	Type I phosphodiesterase / nucleotide pyrophosphatase superfamily protein [Arthrobacter au
gi917442015	4.06	3	1	320	32.9	4.50	2.37	hypothetical protein [Arthrobacter albus]
gi914713436	8.38	1	1	167	16.7	11.34	2.37	hypothetical protein [Arthrobacter sp. ZBG10]
gi517590278	5.33	1	1	244	26.9	5.17	2.37	flavodoxin [Arthrobacter sp. 135MFCol5.1]
gi908697381	2.60	1	1	538	56.9	5.30	2.36	hypothetical protein [Arthrobacter sp. RIT-PI-e]
gi823667146	5.25	1	1	305	32.3	6.18	2.36	monooxygenase [Arthrobacter sp. YC-RL1]
gi651439295	6.54	1	1	260	28.4	6.24	2.36	IclR family transcriptional regulator [Arthrobacter sp. H14]
gi917530498	4.02	1	1	423	45.3	9.33	2.36	permease [Arthrobacter sp. PAMC25486]
gi937258523	2.84	1	1	528	56.4	9.39	2.36	signal recognition particle [Arthrobacter sp. Edens01]
gi917013378	5.61	1	1	196	21.6	5.64	2.36	hypothetical protein [Arthrobacter sanguinis]
gi162953526	8.67	1	1	173	17.6	5.22	2.36	carboxypeptidase G2 precursor [Renibacterium salmoninarum ATCC 33209]
gi116608892	5.42	1	1	461	47.5	5.55	2.36	FAD dependent oxidoreductase [Arthrobacter sp. FB24]
gi654817784	13.74	1	1	131	14.0	5.47	2.36	6-pyruvoyl tetrahydrobiopterin synthase [Arthrobacter sp. UNC362MFTsu5.1]
gi908698630	12.26	1	1	155	17.1	5.44	2.36	hypothetical protein [Arthrobacter sp. RIT-PI-e]
gi759730907	7.88	1	1	292	31.2	5.20	2.36	4-amino-4-deoxychorismate lyase [Arthrobacter sp. L77]
gi928488915	1.67	1	1	780	83.5	5.07	2.36	hypothetical protein AOC05_13790 [Arthrobacter alpinus]
gi307746241	4.52	1	1	376	40.3	5.08	2.36	putative M23 family peptidase [Arthrobacter arilaitensis Re117]
gi119947467	2.73	1	1	512	53.8	5.24	2.36	putative lipoprotein [Arthrobacter aurescens TC1]
gi916259925	5.07	1	1	276	29.4	10.59	2.36	MULTISPECIES: nitrate ABC transporter permease [Arthrobacter]
gi651431413	3.09	4	1	713	77.7	5.59	2.36	ATP-dependent DNA helicase RecQ [Arthrobacter sanguinis]
gi759711877	7.63	1	1	249	26.6	8.65	2.36	GCN5 family acetyltransferase [Arthrobacter sp. 162MFSha1.1]
gi765013764	3.96	1	1	429	45.4	5.17	2.36	ABC transporter substrate-binding protein [Arthrobacter sp. A3]
gi927293371	37.50	1	1	64	7.4	9.99	2.36	hypothetical protein AL755_03860 (plasmid) [Arthrobacter sp. ERGS1:01]
gi937258885	2.54	1	1	747	78.3	4.88	2.36	hypothetical protein AO716_13055 [Arthrobacter sp. Edens01]
gi162954695	6.49	2	1	308	33.0	6.01	2.36	ribosomal large subunit pseudouridine synthase D [Renibacterium salmoninarum ATCC 3320
gi910694207	7.23	1	1	166	18.5	6.20	2.36	mini-circle uncharacterized 19.1 kDa protein [Arthrobacter sp. Hiyo6]
gi939050778	15.18	1	1	112	12.4	6.18	2.36	hypothetical protein [Arthrobacter sp. JCM 19049]
gi359304089	5.57	1	1	341	38.1	5.64	2.36	putative TerC family integral membrane protein [Arthrobacter globiformis NBRC 12137]
gi910695541	2.35	1	1	425	46.4	7.44	2.36	glyceraldehyde-3-phosphate dehydrogenase, partial [Arthrobacter sp. Hiyo6]
gi765013831	5.65	2	1	372	40.4	5.35	2.36	galactonate dehydratase [Arthrobacter sp. A3]
gi737796102	26.67	1	1	75	7.4	11.69	2.36	hypothetical protein [Arthrobacter sp. H20]
gi906445992	7.31	1	1	219	23.7	5.38	2.36	hypothetical protein AC792_10980 [Arthrobacter sp. RIT-PI-e]
gi767257245	11.11	1	1	189	21.1	6.34	2.36	nicotinate-nucleotide adenylyltransferase [Arthrobacter sp. IHBB 11108]
gi323470573	7.66	3	1	222	24.2	8.18	2.36	response regulator with putative antiterminator output domain [Arthrobacter phenanthrenivo
gi470221442	7.88	4	1	241	25.3	9.77	2.36	hypothetical protein ADIAG_00295 [Arthrobacter gangotriensis Lz1y]
gi654824539	6.11	1	1	262	26.9	4.86	2.36	phosphonomutase [Arthrobacter sp. I3]
gi116611878	8.44	1	1	225	23.5	9.86	2.36	hypothetical protein Arth_3226 [Arthrobacter sp. FB24]
gi916863396	3.22	1	1	311	33.4	9.38	2.36	metallophosphoesterase [Arthrobacter sp. 35/47]

gi930826861	9.63	1	1	301	31.5	4.59	2.36	hypothetical protein AOZ07_11605 [Arthrobacter arilaitensis]
gi162954765	3.81	1	1	315	33.6	7.39	2.36	conserved hypothetical protein [Renibacterium salmoninarum ATCC 33209]
gi517593615	3.92	2	1	332	35.2	9.28	2.36	dehydrogenase [Arthrobacter sp. 135MFCol5.1]
gi910252324	4.00	1	1	400	41.9	5.21	2.36	acetyl-CoA acetyltransferase [Arthrobacter siccitolerans]
gi116612650	4.80	1	1	479	50.3	9.03	2.36	sugar transporter [Arthrobacter sp. FB24]
gi307745748	3.27	2	1	397	41.7	4.91	2.36	putative membrane-associated serine protease [Arthrobacter arilaitensis Re117]
gi742071112	3.09	1	1	291	31.6	4.59	2.36	hypothetical protein ANMWB30_23110 [Arthrobacter sp. MWB30]
gi767258594	6.05	1	1	248	26.0	5.41	2.35	3-hydroxybutyrate dehydrogenase [Arthrobacter sp. IHBB 11108]
gi518313385	10.10	1	1	208	21.4	4.77	2.35	hypothetical protein [Arthrobacter sp. TB 23]
gi765008861	9.90	2	1	202	22.6	7.52	2.35	amino acid acetyltransferase [Arthrobacter sp. A3]
gi654819452	11.43	1	1	140	15.6	11.77	2.35	peptide chain release factor 1 [Arthrobacter sp. UNC362MFTsu5.1]
gi307743855	5.19	1	1	308	32.8	6.77	2.35	cobalt-zinc-cadmium resistance protein CzcD [Arthrobacter arilaitensis Re117]
gi551255156	5.56	1	1	270	28.7	10.37	2.35	nitrate/sulfonate/bicarbonate ABC transporter permease [Arthrobacter sp. PAO19]
gi648574560	4.85	2	1	206	22.9	6.00	2.35	translation factor Sua5 [Arthrobacter sp. 131MFCol6.1]
gi651430713	5.79	1	1	311	34.7	9.29	2.35	hypothetical protein [Arthrobacter sanguinis]
gi652423905	7.39	1	1	203	22.0	5.08	2.35	TetR family transcriptional regulator [Arthrobacter castelli]
gi927296407	4.25	1	1	306	34.1	9.95	2.35	amino acid ABC transporter permease [Arthrobacter sp. ERGS1:01]
gi737812897	4.35	1	1	230	23.8	4.94	2.35	exopolyphosphatase, partial [Arthrobacter sp. H14]
gi767256573	3.40	1	1	471	48.9	5.43	2.35	flavoprotein disulfide reductase [Arthrobacter sp. IHBB 11108]
gi742859898	2.01	1	1	597	62.5	8.78	2.35	lactate permease [Arthrobacter sp. W1]
gi515764790	2.51	1	1	439	46.9	6.00	2.35	serine hydroxymethyltransferase [Arthrobacter sp. M2012083]
gi517605479	4.35	1	1	620	64.1	9.32	2.35	hypothetical protein [Arthrobacter sp. 131MFCol6.1]
gi470220474	2.86	1	1	665	72.6	5.24	2.35	hydantoinase B/oxoprolinase [Arthrobacter gangotriensis Lz1y]
gi910696440	4.80	1	1	229	24.4	6.01	2.35	uncharacterized protein YwhH [Arthrobacter sp. Hiyo6]
gi742756090	4.08	3	1	319	32.6	4.72	2.35	cyclase [Arthrobacter phenanthrenivorans]
gi443483219	2.11	3	1	427	44.6	8.87	2.35	inner-membrane translocator [Arthrobacter nitrophenolicus]
gi737776215	2.16	1	1	416	45.5	7.33	2.35	alpha-hydroxy-acid oxidizing enzyme [Arthrobacter sanguinis]
gi517605938	4.49	2	1	356	37.5	5.20	2.35	pyruvate dehydrogenase E1 subunit alpha [Arthrobacter sp. 131MFCol6.1]
gi654815230	21.15	1	1	104	12.3	10.32	2.35	hypothetical protein [Arthrobacter sp. PAO19]
gi759761578	5.60	3	1	464	50.6	8.98	2.35	MFS transporter permease [Arthrobacter sp. Rue61a]
gi674644399	5.03	1	1	358	40.5	9.11	2.35	FemAB family protein [Arthrobacter sp. 11W110_air]
gi651443831	7.63	1	1	262	28.4	6.02	2.35	ABC transporter [Arthrobacter nicotinovorans]
gi742757995	10.85	3	1	212	21.7	5.38	2.35	thiamine-phosphate pyrophosphorylase [Arthrobacter phenanthrenivorans]
gi651478935	4.37	3	1	343	37.1	5.45	2.35	dehydratase [Arthrobacter sp. Br18]
gi918267932	3.72	2	1	323	34.6	10.05	2.35	L-arabinose transport system permease protein AraQ [Arthrobacter sp. Hiyo1]
gi914716619	3.96	2	1	429	46.9	5.38	2.35	ABC transporter substrate-binding protein [Arthrobacter sp. ZBG10]
gi654814842	4.20	1	1	381	39.5	5.31	2.35	hypothetical protein [Arthrobacter sp. MA-N2]
gi767259106	3.28	1	1	305	31.1	5.29	2.35	porphobilinogen deaminase [Arthrobacter sp. IHBB 11108]
gi640202929	5.36	1	1	336	37.2	5.16	2.35	GCN5 family acetyltransferase [Arthrobacter sp. 31Y]
gi917441853	0.98	1	1	1126	124.6	5.24	2.35	hypothetical protein [Arthrobacter albus]
gi910740555	2.60	1	1	346	37.4	4.81	2.35	xaa-Pro aminopeptidase 1 [Arthrobacter sp. Hiyo4]
gi651502890	1.95	1	1	616	67.1	6.18	2.35	capsule biosynthesis protein CapD [Arthrobacter sp. 35W]
gi910252339	6.23	1	1	257	27.0	5.14	2.35	electron transfer flavoprotein subunit beta [Arthrobacter siccitolerans]
gi740684104	3.41	1	1	410	43.0	7.23	2.35	glycine oxidase [Arthrobacter sp. PAMC25486]
gi476401944	5.41	1	1	259	26.8	5.34	2.35	FAD-dependent pyridine nucleotide-disulfide oxidoreductase, partial [Arthrobacter crystallopo
gi937258264	1.93	2	1	727	72.4	5.19	2.35	PTS lactose transporter subunit IIC [Arthrobacter sp. Edens01]
gi916691644	2.75	2	1	364	39.3	5.35	2.35	methylthioribose-1-phosphate isomerase [Arthrobacter castelli]
gi652423074	1.25	1	1	879	96.4	4.93	2.35	aminopeptidase N [Arthrobacter castelli]
gi927031295	4.17	1	1	312	33.0	5.15	2.35	formimidoylglutamase [Arthrobacter sp. LS16]
gi476400085	4.72	1	1	318	34.4	9.23	2.34	transcriptional regulator, AraC family protein [Arthrobacter crystallopoietes BAB-32]
gi916816071	2.76	1	1	760	79.7	9.23	2.34	transglutaminase [Arthrobacter sp. MA-N2]
gi116611095	8.15	1	1	270	29.3	7.42	2.34	SPFH domain, Band 7 family protein [Arthrobacter sp. FB24]
gi759730411	4.03	1	1	298	33.7	6.34	2.34	glycosyl transferase family 2 [Arthrobacter sp. L77]
gi767256470	29.11	1	1	79	8.9	9.98	2.34	hypothetical protein UM93_00070 [Arthrobacter sp. IHBB 11108]
gi759735058	5.35	2	1	299	32.0	6.23	2.34	glyoxalase [Arthrobacter sp. L77]

gi476400689	4.85	1	1	392	43.2	4.94	2.34	cytochrome P450 [Arthrobacter crystallopoietes BAB-32]
gi917530545	3.61	1	1	332	35.4	5.41	2.34	proline racemase [Arthrobacter sp. PAMC25486]
gi918268052	8.57	2	1	105	11.6	5.08	2.34	uncharacterized protein YabN [Arthrobacter sp. Hiyo1]
gi742755813	5.52	1	1	308	32.7	6.35	2.34	pseudouridine synthase [Arthrobacter phenanthrenivorans]
gi651456335	4.58	2	1	415	43.6	4.83	2.34	phosphoglycerate kinase [Arthrobacter sp. 35/47]
gi654822731	4.67	1	1	214	22.4	4.68	2.34	tRNA threonylcarbamoyladenosine biosynthesis protein TsaE [Arthrobacter sp. I3]
gi916691171	4.55	1	1	440	49.2	5.49	2.34	hypothetical protein [Arthrobacter castelli]
gi786033932	4.29	3	1	233	25.1	10.10	2.34	hypothetical protein [Arthrobacter chlorophenolicus]
gi674645478	5.49	1	1	273	28.3	4.64	2.34	Virginiamycin B lyase [Arthrobacter sp. 11W110_air]
gi917746045	3.01	1	1	564	58.1	5.19	2.34	hypothetical protein [Arthrobacter phenanthrenivorans]
gi786026289	7.91	3	1	316	32.8	5.64	2.34	ATPase [Arthrobacter chlorophenolicus]
gi323467788	6.69	1	1	299	31.8	5.00	2.34	dehydrogenase of unknown specificity, short-chain alcohol dehydrogenase -like protein [Arth
gi651489085	3.49	1	1	344	38.6	10.08	2.34	integrase [Arthrobacter sp. H20]
gi116610859	15.31	1	1	98	11.2	5.02	2.34	Antibiotic biosynthesis monooxygenase [Arthrobacter sp. FB24]
gi635352948	4.84	2	1	248	27.0	4.64	2.34	hypothetical protein ARTSIC4J27_985 [Arthrobacter siccitolerans]
gi307744449	3.19	1	1	313	34.1	9.07	2.34	putative type II secretion system protein F [Arthrobacter arilaitensis Re117]
gi476400969	2.70	1	1	408	43.8	8.63	2.34	FAD dependent oxidoreductase [Arthrobacter crystallopoietes BAB-32]
gi759734893	12.32	1	1	138	15.1	5.53	2.34	nucleoside diphosphate kinase [Arthrobacter sp. L77]
gi651444893	4.68	2	1	235	26.0	7.28	2.34	hypothetical protein [Arthrobacter nicotinovorans]
gi359303991	7.11	1	1	239	26.1	5.06	2.34	hypothetical protein ARGLB_092_00560 [Arthrobacter globiformis NBRC 12137]
gi403231832	2.85	1	1	702	74.4	10.04	2.34	hypothetical protein ARUE_232p00450 (plasmid) [Arthrobacter sp. Rue61a]
gi674643987	4.92	1	1	244	25.6	5.41	2.34	ANTAR domain protein [Arthrobacter sp. 11W110_air]
gi651499850	3.59	1	1	446	44.5	10.04	2.34	MFS transporter [Arthrobacter sp. 35W]
gi551255283	6.04	1	1	265	27.6	4.74	2.34	alpha/beta hydrolase [Arthrobacter sp. PAO19]
gi916820305	6.08	1	1	263	27.7	5.06	2.34	hypothetical protein [Arthrobacter sp. H20]
gi910748568	5.26	2	1	228	25.3	6.55	2.34	uvrABC system protein C [Arthrobacter sp. Hiyo8]
gi651498550	5.45	1	1	330	35.0	6.74	2.34	XRE family transcriptional regulator [Arthrobacter sp. 35W]
gi765010988	7.33	2	1	300	30.2	4.81	2.34	oxidoreductase [Arthrobacter sp. A3]
gi651435833	7.66	1	1	209	22.6	5.27	2.34	hypothetical protein [Arthrobacter sp. H41]
gi307744368	4.78	1	1	209	22.2	4.84	2.34	hypothetical membrane protein [Arthrobacter arilaitensis Re117]
gi470221266	11.42	2	1	219	23.4	4.89	2.34	Putative pterin-4-alpha-carbinolamine dehydratase [Arthrobacter gangotriensis Lz1y]
gi651438145	4.55	1	1	374	40.1	8.44	2.34	cytochrome C biogenesis protein [Arthrobacter sp. H14]
gi723609951	5.86	2	1	307	34.4	9.07	2.34	hypothetical protein ART_3728 [Arthrobacter sp. PAMC25486]
gi939051185	6.29	1	1	159	17.8	6.89	2.34	hypothetical protein [Arthrobacter sp. JCM 19049]
gi517591548	4.53	1	1	397	40.9	4.67	2.34	AMP-dependent synthetase [Arthrobacter sp. 135MFCol5.1]
gi759724698	31.58	3	1	57	5.8	8.41	2.34	hypothetical protein [Arthrobacter sp. I3]
gi917739720	3.82	1	1	393	41.3	4.92	2.33	acetylornithine deacetylase [Arthrobacter sp. W1]
gi518314115	8.88	1	1	214	23.3	8.47	2.33	MOSC domain-containing protein [Arthrobacter sp. TB 23]
gi162955444	9.95	1	1	211	23.5	6.15	2.33	nicotinate-nucleotide adenylyltransferase [Renibacterium salmoninarum ATCC 33209]
gi767258610	8.82	1	1	136	14.3	9.69	2.33	hypothetical protein UM93_15130 [Arthrobacter sp. IHBB 11108]
gi654822250	3.82	3	1	419	46.9	5.58	2.33	hypothetical protein [Arthrobacter sp. I3]
gi916863622	4.77	4	1	398	42.9	8.66	2.33	HipA-like protein [Arthrobacter sp. 35/47]
gi927295672	7.89	1	1	152	16.3	5.91	2.33	Fur family transcriptional regulator [Arthrobacter sp. ERGS1:01]
gi917013232	2.37	1	1	379	41.8	4.81	2.33	hypothetical protein [Arthrobacter sanguinis]
gi695270320	3.45	2	1	261	28.4	7.09	2.33	hypothetical protein [Arthrobacter globiformis]
gi640199358	4.29	1	1	233	25.6	5.48	2.33	GntR family transcriptional regulator [Arthrobacter sp. 31Y]
gi695208025	3.05	1	1	393	43.3	6.48	2.33	hypothetical transcriptional regulator (plasmid) [Arthrobacter nicotinovorans]
gi916781722	2.44	1	1	410	44.3	6.86	2.33	lipase [Arthrobacter sp. 35W]
gi914717019	8.93	1	1	224	24.6	7.05	2.33	DNA alkylation repair protein [Arthrobacter sp. ZBG10]
gi476401826	6.29	1	1	334	37.0	9.61	2.33	dipeptide/oligopeptide/nickel ABC transporter permease [Arthrobacter crystallopoietes BAB-3
gi917739576	3.50	2	1	314	34.1	7.85	2.33	phytoene synthase [Arthrobacter sp. W1]
gi910249986	1.69	1	1	651	70.2	7.52	2.33	acyltransferase [Arthrobacter siccitolerans]
gi910697723	12.88	1	1	132	13.9	6.02	2.33	inulin fructotransferase [DFA-I-forming] [Arthrobacter sp. Hiyo6]
gi737802872	4.85	1	1	433	47.9	5.29	2.33	hypothetical protein, partial [Arthrobacter castelli]
gi551254710	3.66	1	1	492	51.7	9.72	2.33	membrane protein [Arthrobacter sp. PAO19]

gi545109534	3.81	1	1	394	40.9	5.15	2.33	1-deoxy-D-xylulose-5-phosphate reductoisomerase [Arthrobacter sp. AK-YN10]
gi443481210	20.45	1	1	88	9.7	5.76	2.33	flavin reductase domain-containing protein [Arthrobacter nitrophenolicus]
gi651466164	5.81	1	1	258	28.1	6.32	2.33	glucosamine-6-phosphate deaminase [Arthrobacter sp. 35/47]
gi515767314	7.49	1	1	307	33.2	6.70	2.33	ABC transporter [Arthrobacter sp. M2012083]
gi723608112	6.43	1	1	280	30.1	9.66	2.33	hypothetical protein ART_1889 [Arthrobacter sp. PAMC25486]
gi551256537	2.12	2	1	614	65.7	7.09	2.33	multidrug ABC transporter ATPase [Arthrobacter sp. PAO19]
gi939036615	7.14	1	1	112	12.1	7.43	2.33	hypothetical protein [Arthrobacter nitroguajacolicus]
gi918265925	4.53	1	1	265	29.1	6.54	2.33	sugar phosphatase YfbT [Arthrobacter sp. Hiyo1]
gi786026577	2.32	1	1	474	51.6	5.39	2.33	dTDP-4-dehydrorhamnose reductase [Arthrobacter chlorophenolicus]
gi759765018	4.23	1	1	284	30.9	5.39	2.33	hypothetical protein [Arthrobacter gangotriensis]
gi658509811	5.18	1	1	386	41.2	5.91	2.33	diguanylate cyclase [Arthrobacter sp. TB 26]
gi651469979	19.48	3	1	77	8.3	10.92	2.33	hypothetical protein [Arthrobacter nicotinovorans]
gi740680753	3.06	1	1	360	38.5	5.24	2.33	oxidoreductase [Arthrobacter sp. PAMC25486]
gi651507568	4.29	2	1	303	32.2	7.71	2.33	AraC family transcriptional regulator [Arthrobacter sp. 35W]
gi652424475	3.98	2	1	427	44.6	4.32	2.33	hypothetical protein [Arthrobacter castelli]
gi651434445	2.06	1	1	534	57.2	5.47	2.33	methylcrotonoyl-CoA carboxylase [Arthrobacter sp. H41]
gi916781576	4.04	2	1	470	49.5	9.69	2.33	hypothetical protein [Arthrobacter sp. 35W]
gi654827179	4.36	1	1	390	41.7	8.88	2.33	hypothetical protein [Arthrobacter sp. H5]
gi651444597	3.35	1	1	358	37.1	5.88	2.33	maleylacetate reductase [Arthrobacter nicotinovorans]
gi928487361	7.03	1	1	327	35.1	5.92	2.32	AAA family ATPase [Arthrobacter alpinus]
gi914713619	2.46	1	1	487	52.3	9.41	2.32	hypothetical protein [Arthrobacter sp. ZBG10]
gi674645159	4.96	1	1	363	38.0	10.70	2.32	Alpha/beta hydrolase family protein [Arthrobacter sp. 11W110_air]
gi162952689	50.00	1	1	42	4.9	10.24	2.32	hypothetical protein RSal33209_0454 [Renibacterium salmoninarum ATCC 33209]
gi170783571	22.00	1	1	100	9.9	4.41	2.32	hypothetical protein (plasmid) [Arthrobacter sp. Chr15]
gi674645264	5.16	2	1	446	45.7	9.04	2.32	3-oxoacyl-[acyl-carrier-protein] reductase FabG [Arthrobacter sp. 11W110_air]
gi323467957	4.27	2	1	281	30.6	5.24	2.32	methylase involved in ubiquinone/menaquinone biosynthesis [Arthrobacter phenanthrenivora
gi727802698	3.37	1	1	326	35.7	5.72	2.32	chorismate mutase [Bifidobacterium saguini]
gi767257694	2.87	2	1	592	63.6	5.31	2.32	ABC transporter substrate-binding protein [Arthrobacter sp. IHBB 11108]
gi654825885	4.33	1	1	277	29.9	8.53	2.32	hypothetical protein [Arthrobacter sp. H5]
gi914713722	5.03	1	1	318	33.0	7.05	2.32	diacylglycerol kinase [Arthrobacter sp. ZBG10]
gi916259890	4.34	1	1	392	42.6	6.35	2.32	UDP-N-acetyl glucosamine 2-epimerase [Arthrobacter sp. TB 23]
gi219858369	3.96	1	1	480	50.3	5.55	2.32	histidine kinase [Arthrobacter chlorophenolicus A6]
gi910747536	3.70	1	1	270	28.9	4.97	2.32	D-mannonate oxidoreductase [Arthrobacter sp. Hiyo8]
gi765005762	2.63	1	1	457	47.2	8.88	2.32	C4-dicarboxylate transporter [Arthrobacter sp. A3]
gi916876125	16.67	1	1	144	14.6	12.38	2.32	hypothetical protein [Arthrobacter sp. 31Y]
gi757624413	10.22	1	1	225	23.7	6.73	2.32	dienelactone hydrolase [Arthrobacter sp. SPG23]
gi674645391	2.41	1	1	456	46.4	6.20	2.32	Nitrate/nitrite sensor protein NarX [Arthrobacter sp. 11W110_air]
gi323468165	5.77	1	1	381	40.6	9.39	2.32	transposase [Arthrobacter phenanthrenivorans Sphe3]
gi443482062	5.26	3	1	494	52.6	5.02	2.32	hypothetical protein G205_08193 [Arthrobacter nitrophenolicus]
gi910742187	8.76	1	1	194	21.0	10.54	2.32	putative peptide transport permease protein Mb1314c [Arthrobacter sp. Hiyo4]
gi910745557	22.39	1	1	67	7.3	6.60	2.32	hypothetical protein AHiyo8_32590 [Arthrobacter sp. Hiyo8]
gi908699246	7.61	1	1	289	30.9	5.50	2.32	3-hydroxybutyryl-CoA dehydrogenase [Arthrobacter sp. RIT-PI-e]
gi219859630	3.79	1	1	290	29.7	5.15	2.32	orotidine 5'-phosphate decarboxylase [Arthrobacter chlorophenolicus A6]
gi737812750	3.48	1	1	316	34.0	5.86	2.32	exopolyphosphatase [Arthrobacter sp. H14]
gi917530148	6.78	6	1	236	26.7	5.85	2.32	hypothetical protein [Arthrobacter sp. PAMC25486]
gi674646279	3.71	1	1	539	56.8	10.67	2.32	ATP-dependent RNA helicase RhlE [Arthrobacter sp. 11W110_air]
gi695210640	4.14	1	1	290	32.0	10.30	2.32	hypothetical protein (plasmid) [Arthrobacter aurescens]
gi910737241	11.46	1	1	157	17.6	6.95	2.32	uncharacterized protein YdfG [Arthrobacter sp. Hiyo4]
gi651440966	7.39	1	1	230	23.9	4.78	2.32	hypothetical protein [Arthrobacter sp. 9MFCol3.1]
gi910741731	14.69	1	1	143	14.8	9.99	2.32	3-isopropylmalate dehydratase large subunit [Arthrobacter sp. Hiyo4]
gi652424058	3.49	1	1	315	35.9	9.42	2.32	hypothetical protein [Arthrobacter castelli]
gi786029192	3.80	1	1	631	65.7	10.86	2.32	DEAD/DEAH box helicase [Arthrobacter chlorophenolicus]
gi737805713	2.84	1	1	388	41.9	9.39	2.31	hypothetical protein [Arthrobacter sp. Br18]
gi928486450	10.98	1	1	264	28.0	5.48	2.31	polyphosphate glucokinase [Arthrobacter alpinus]
gi219859233	10.86	1	1	267	28.0	5.67	2.31	ROK family protein [Arthrobacter chlorophenolicus A6]

gi786027743	3.28	5	1	549	56.7	4.91	2.31	thiamine pyrophosphate-binding protein [Arthrobacter chlorophenolicus]
gi652422578	6.59	1	1	349	36.7	9.23	2.31	hypothetical protein [Arthrobacter castelli]
gi918469404	2.19	1	1	548	57.0	9.91	2.31	hypothetical protein [Arthrobacter crystallopoietes]
gi914717124	4.44	1	1	518	57.5	5.06	2.31	methionine--tRNA ligase [Arthrobacter sp. ZBG10]
gi737776751	7.42	1	1	256	27.6	9.91	2.31	CAAX amino protease [Arthrobacter sanguinis]
gi910746894	10.96	2	1	219	24.0	11.72	2.31	hypothetical protein AHiyo8_45960 [Arthrobacter sp. Hiyo8]
gi910741723	3.92	1	1	255	27.2	8.44	2.31	acyl-coenzyme A oxidase 4, peroxisomal [Arthrobacter sp. Hiyo4]
gi551254457	3.10	2	1	678	73.0	5.26	2.31	biotin carboxyl carrier protein [Arthrobacter sp. PAO19]
gi930825273	8.28	1	1	145	16.0	5.17	2.31	hypothetical protein AOZ07_02435 [Arthrobacter arilaitensis]
gi640200570	15.89	3	1	151	16.0	11.02	2.31	sulfate transporter [Arthrobacter sp. 31Y]
gi916781925	1.93	1	1	1087	118.2	5.12	2.31	hypothetical protein [Arthrobacter sp. 35W]
gi162953528	2.51	1	1	438	47.0	5.83	2.31	transcriptional regulator [Renibacterium salmoninarum ATCC 33209]
gi914714171	4.47	1	1	515	55.9	4.92	2.31	glycosyl hydrolase family 32 [Arthrobacter sp. ZBG10]
gi914717750	9.26	3	1	216	23.2	10.56	2.31	metal-dependent hydrolase [Arthrobacter sp. ZBG10]
gi737810166	9.19	1	1	185	19.9	7.55	2.31	shikimate kinase [Arthrobacter sp. 35/47]
gi470216541	3.41	1	1	352	38.3	6.80	2.31	molybdenum cofactor biosynthesis protein A [Arthrobacter gangotriensis Lz1y]
gi737801365	6.71	1	1	328	36.0	5.85	2.31	membrane protein [Arthrobacter castelli]
gi651434807	7.22	5	1	291	30.8	6.42	2.31	hypothetical protein [Arthrobacter sp. H41]
gi910745732	6.14	1	1	228	24.2	6.10	2.31	uncharacterized HTH-type transcriptional regulator YdhC [Arthrobacter sp. Hiyo8]
gi928487668	8.20	1	1	244	24.6	10.42	2.31	permease [Arthrobacter alpinus]
gi470221476	3.43	1	1	321	34.4	5.60	2.31	ribonuclease Z [Arthrobacter gangotriensis Lz1y]
gi651485639	4.66	2	1	365	39.6	5.14	2.31	4-hydroxy-3-methylbut-2-enyl diphosphate reductase [Arthrobacter sp. Br18]
gi823668427	4.50	1	1	378	40.6	6.37	2.31	acyl-CoA dehydrogenase [Arthrobacter sp. YC-RL1]
gi654818220	7.74	1	1	310	33.7	4.97	2.31	phenylacetate-CoA oxygenase [Arthrobacter sp. UNC362MFTsu5.1]
gi737801428	6.77	1	1	251	27.0	5.01	2.31	GntR family transcriptional regulator [Arthrobacter castelli]
gi737788414	11.52	2	1	165	17.7	11.00	2.31	FHA domain-containing protein [Arthrobacter albus]
gi476400733	1.79	1	1	559	59.1	6.23	2.31	thioredoxin reductase [Arthrobacter crystallopoietes BAB-32]
gi517609013	3.64	1	1	714	73.8	5.19	2.31	hypothetical protein [Arthrobacter sp. 161MFSha2.1]
gi823668021	6.11	2	1	409	42.8	5.58	2.31	N-formimino-L-glutamate deiminase [Arthrobacter sp. YC-RL1]
gi910696131	4.15	1	1	434	47.6	6.00	2.31	long-chain-fatty-acid--CoA ligase [Arthrobacter sp. Hiyo6]
gi654812769	3.35	1	1	328	32.8	5.06	2.31	electron transfer flavoprotein subunit alpha [Arthrobacter sp. MA-N2]
gi916815820	6.35	2	1	299	32.9	8.46	2.31	hypothetical protein [Arthrobacter sp. MA-N2]
gi765012622	11.11	2	1	126	13.4	5.25	2.31	transcriptional regulator [Arthrobacter sp. A3]
gi403231401	18.70	3	1	123	13.9	8.56	2.31	putative transcriptional regulator, ArsR family [Arthrobacter sp. Rue61a]
gi910249425	11.33	1	1	150	16.4	6.68	2.31	methionyl-tRNA formyltransferase [Arthrobacter siccitolerans]
gi910697338	3.77	1	1	265	28.0	7.23	2.31	hypothetical protein AHiyo6_04980 [Arthrobacter sp. Hiyo6]
gi517606871	2.74	1	1	657	71.5	9.35	2.31	amino acid transporter [Arthrobacter sp. 161MFSha2.1]
gi927295413	25.51	1	1	98	10.6	10.80	2.31	hypothetical protein AL755_17685 [Arthrobacter sp. ERGS1:01]
gi359304143	6.30	1	1	254	26.7	6.55	2.31	putative ABC transporter ATP-binding protein [Arthrobacter globiformis NBRC 12137]
gi654826270	3.48	1	1	374	38.7	5.12	2.31	sodium:proton antiporter [Arthrobacter sp. H5]
gi517602923	2.23	1	1	719	74.5	7.85	2.31	primosomal protein N' [Arthrobacter sp. 131MFCol6.1]
gi542107842	4.88	1	1	471	49.6	9.41	2.30	amino acid transporter [Arthrobacter sp. AK-YN10]
gi219858983	3.33	1	1	451	46.2	7.75	2.30	RDD domain containing protein [Arthrobacter chlorophenolicus A6]
gi636846358	4.89	4	1	266	28.2	6.30	2.30	3-methyl-2-oxobutanoate hydroxymethyltransferase, partial [Arthrobacter sp. TB 26]
gi737811675	5.37	1	1	298	32.7	5.58	2.30	ABC transporter [Arthrobacter sp. 35/47]
gi930825961	2.21	1	1	452	46.3	5.22	2.30	3-phosphoshikimate 1-carboxyvinyltransferase [Arthrobacter arilaitensis]
gi476403164	6.87	1	1	262	28.5	4.86	2.30	N-methyltryptophan oxidase [Arthrobacter crystallopoietes BAB-32]
gi651506468	3.01	1	1	299	32.4	10.59	2.30	secretion system protein [Arthrobacter sp. 35W]
gi651490840	9.13	4	1	219	24.4	5.22	2.30	PhoU family transcriptional regulator [Arthrobacter sp. H20]
gi910697680	12.50	2	1	136	14.8	9.72	2.30	trehalose/maltose transport system permease protein MalF [Arthrobacter sp. Hiyo6]
gi742855954	1.02	1	1	1172	126.3	5.47	2.30	1-pyrroline-5-carboxylate dehydrogenase [Arthrobacter sp. W1]
gi651444519	4.90	1	1	306	32.1	6.39	2.30	methionyl-tRNA formyltransferase [Arthrobacter nicotinovorans]
gi723607950	3.59	1	1	334	35.7	9.58	2.30	heat shock protein DnaJ domain-containing protein [Arthrobacter sp. PAMC25486]
gi742856633	4.59	1	1	305	33.0	5.43	2.30	glutamyl-Q tRNA(Asp) ligase [Arthrobacter sp. W1]
gi939051101	11.11	1	1	126	13.8	10.11	2.30	transposase [Arthrobacter sp. JCM 19049]

gi742758610	4.71	3	1	488	53.3	9.33	2.30	oxidoreductase [Arthrobacter phenanthrenivorans]
gi517590290	12.15	1	1	214	22.5	4.73	2.30	hypothetical protein [Arthrobacter sp. 135MFCol5.1]
gi927293534	2.66	1	1	564	59.6	5.05	2.30	acetolactate synthase [Arthrobacter sp. ERGS1:01]
gi765011295	19.88	1	1	161	16.3	5.00	2.30	LysR family transcriptional regulator [Arthrobacter sp. A3]
gi765013689	6.97	1	1	373	37.6	5.08	2.30	phosphate ABC transporter substrate-binding protein [Arthrobacter sp. A3]
gi674646926	4.24	5	1	566	58.4	5.72	2.30	Sulfoacetaldehyde acetyltransferase [Arthrobacter sp. 11W110_air]
gi910741973	2.40	1	1	500	53.1	9.72	2.30	putative membrane protein ActII-3 [Arthrobacter sp. Hiyo4]
gi742070188	6.02	1	1	299	31.8	4.89	2.30	thiosulfate sulfurtransferase SseB [Arthrobacter sp. MWB30]
gi307743752	2.19	1	1	638	68.2	4.46	2.30	putative extracellular nuclease [Arthrobacter arilaitensis Re117]
gi443481201	8.07	1	1	322	35.0	5.40	2.30	F420-dependent glucose-6-phosphate dehydrogenase [Arthrobacter nitrophenolicus]
gi937259036	7.47	1	1	174	18.5	6.61	2.30	hypothetical protein AO716_13925 [Arthrobacter sp. Edens01]
gi908697266	5.88	1	1	289	32.4	6.71	2.30	DNA methyltransferase [Arthrobacter sp. RIT-PI-e]
gi916863369	1.80	1	1	667	74.9	6.27	2.30	hypothetical protein [Arthrobacter sp. 35/47]
gi654817228	9.79	1	1	143	15.6	10.11	2.30	hypothetical protein [Arthrobacter sp. UNC362MFTsu5.1]
gi515767827	5.81	1	1	310	31.5	4.92	2.30	major intrinsic protein [Arthrobacter sp. M2012083]
gi116609072	5.01	3	1	339	36.5	5.12	2.30	PHP C-terminal domain protein [Arthrobacter sp. FB24]
gi765005798	4.47	1	1	313	32.3	6.71	2.30	fatty acid-binding protein DegV [Arthrobacter sp. A3]
gi917442021	6.69	1	1	314	33.0	9.77	2.30	hypothetical protein [Arthrobacter albus]
gi651430811	4.81	1	1	187	21.2	5.07	2.30	pyridoxamine 5'-phosphate oxidase [Arthrobacter sanguinis]
gi651441333	1.43	1	1	1046	112.3	6.09	2.30	acriflavin resistance protein [Arthrobacter sp. 9MFCol3.1]
gi742859737	7.47	1	1	281	30.4	6.96	2.30	GntR family transcriptional regulator [Arthrobacter sp. W1]
gi651443002	1.17	2	1	1365	151.8	5.45	2.30	hypothetical protein [Arthrobacter sp. 9MFCol3.1]
gi914715733	5.67	1	1	282	29.7	5.50	2.30	hypothetical protein [Arthrobacter sp. ZBG10]
gi476399560	4.20	1	1	333	36.2	7.49	2.30	AsnC family transcriptional regulator [Arthrobacter crystallopoietes BAB-32]
gi916691541	3.73	1	1	295	31.5	4.89	2.30	methyltransferase type 11 [Arthrobacter castelli]
gi652423979	14.12	1	1	177	19.3	4.78	2.30	hypothetical protein [Arthrobacter castelli]
gi307744521	3.97	1	1	252	26.9	5.99	2.30	putative drug resistance ATP-binding protein [Arthrobacter arilaitensis Re117]
gi651500258	3.74	1	1	294	32.4	7.81	2.30	XRE family transcriptional regulator [Arthrobacter sp. 35W]
gi916835127	6.38	2	1	141	15.9	4.88	2.30	hypothetical protein [Arthrobacter sp. H14]
gi916259828	3.04	1	1	395	40.6	5.12	2.30	hypothetical protein [Arthrobacter sp. TB 23]
gi908698609	2.93	1	1	443	45.7	4.92	2.30	homoserine dehydrogenase [Arthrobacter sp. RIT-PI-e]
gi916692215	7.07	1	1	283	31.5	9.17	2.30	hypothetical protein [Arthrobacter castelli]
gi307746154	1.77	1	1	621	68.7	5.45	2.30	type I restriction-modification system modification subunit [Arthrobacter arilaitensis Re117]
gi914717284	3.89	1	1	334	35.9	7.33	2.30	exopolyphosphatase [Arthrobacter sp. ZBG10]
gi651504457	2.65	1	1	869	91.5	5.74	2.30	histidine kinase [Arthrobacter sp. 35W]
gi162955583	6.42	1	1	265	28.9	6.55	2.30	auxin-induced protein PCNT115 [Renibacterium salmoninarum ATCC 33209]
gi728817754	2.12	1	1	756	83.9	7.33	2.29	Primary amine oxidase [Gossypium arboreum]
gi651429772	2.17	1	1	784	83.1	5.62	2.29	hypothetical protein [Arthrobacter sanguinis]
gi928486289	8.76	1	1	137	15.1	10.20	2.29	hypothetical protein AOC05_03560 [Arthrobacter alpinus]
gi162955721	21.05	5	1	95	10.8	5.27	2.29	SSU ribosomal protein S6P [Renibacterium salmoninarum ATCC 33209]
gi759731481	8.05	2	1	236	25.7	5.01	2.29	transcriptional regulator [Arthrobacter sp. L77]
gi937259233	5.08	1	1	374	39.1	5.36	2.29	phosphoribosylaminoimidazole carboxylase [Arthrobacter sp. Edens01]
gi757625630	11.11	1	1	153	16.4	10.48	2.29	MarR family transcriptional regulator [Arthrobacter sp. SPG23]
gi476401162	5.98	2	1	234	25.0	4.96	2.29	ATPase component of ABC-type transport system [Arthrobacter crystallopoietes BAB-32]
gi674646707	3.82	1	1	471	50.2	9.50	2.29	Gamma-glutamylputrescine oxidoreductase [Arthrobacter sp. 11W110_air]
gi651486872	7.94	4	1	277	28.4	4.94	2.29	hypothetical protein [Arthrobacter sp. Br18]
gi651429434	8.08	1	1	297	32.3	5.01	2.29	hypothetical protein [Arthrobacter sanguinis]
gi742858932	2.68	1	1	298	32.2	6.46	2.29	hypothetical protein [Arthrobacter sp. W1]
gi515768054	1.92	1	1	729	78.2	5.45	2.29	glycosyl transferase [Arthrobacter sp. M2012083]
gi908699531	3.18	1	1	346	36.5	5.41	2.29	ABC transporter [Arthrobacter sp. RIT-PI-e]
gi916869580	5.13	1	1	390	42.5	6.87	2.29	hypothetical protein [Arthrobacter sp. Br18]
gi674645172	4.37	1	1	435	46.2	8.94	2.29	Proline/betaine transporter [Arthrobacter sp. 11W110_air]
gi517601939	7.64	2	1	288	30.5	10.21	2.29	SDR family oxidoreductase [Arthrobacter sp. 162MFSha1.1]
gi654817049	2.41	1	1	540	57.8	8.40	2.29	peptide ABC transporter substrate-binding protein [Arthrobacter sp. UNC362MFTsu5.1]
gi908691057	7.63	4	1	262	27.5	4.09	2.29	hypothetical protein [Arthrobacter sp. H41]

gi651440852	4.15	1	1	289	30.1	5.05	2.29	alpha/beta hydrolase [Arthrobacter sp. 9MFCol3.1]
gi927295095	7.60	2	1	263	27.5	7.43	2.29	ABC transporter [Arthrobacter sp. ERGS1:01]
gi742858018	4.24	2	1	425	45.3	6.38	2.29	amino acid dehydrogenase [Arthrobacter sp. W1]
gi359307156	10.38	1	1	183	19.2	5.29	2.29	putative oxidoreductase [Arthrobacter globiformis NBRC 12137]
gi162954020	3.47	1	1	461	50.2	5.66	2.29	4-aminobutyrate aminotransferase [Renibacterium salmoninarum ATCC 33209]
gi908697387	48.65	1	1	37	4.3	10.43	2.29	50S ribosomal protein L36 [Arthrobacter sp. RIT-PI-e]
gi823667821	4.46	1	1	224	24.8	5.86	2.29	GntR family transcriptional regulator [Arthrobacter sp. YC-RL1]
gi654823198	5.49	1	1	237	24.4	5.27	2.29	short-chain dehydrogenase [Arthrobacter sp. I3]
gi598062302	3.51	1	1	456	48.9	5.57	2.29	PuhA [Arthrobacter sp. BS2(2014)]
gi918221963	3.39	1	1	383	39.6	7.09	2.29	acetylornithine deacetylase, partial [Arthrobacter sp. I3]
gi652423098	5.93	1	1	253	26.6	4.77	2.29	6-phosphogluconolactonase [Arthrobacter castelli]
gi910743671	9.09	1	1	275	28.6	8.12	2.29	4-hydroxy-tetrahydrodipicolinate reductase [Arthrobacter sp. Hiyo8]
gi695210562	11.43	2	1	105	10.9	11.55	2.29	hypothetical protein (plasmid) [Arthrobacter aurescens]
gi723606314	0.97	1	1	924	98.1	5.58	2.29	glycine dehydrogenase [Arthrobacter sp. PAMC25486]
gi737800993	3.20	1	1	562	57.9	4.79	2.29	leucyl aminopeptidase [Arthrobacter castelli]
gi476402118	5.46	1	1	238	24.6	5.59	2.29	cbiX family protein [Arthrobacter crystallopoietes BAB-32]
gi759731883	3.50	1	1	343	37.8	5.25	2.29	luciferase [Arthrobacter sp. L77]
gi674646232	1.98	2	1	907	99.7	5.95	2.29	DNA topoisomerase 1 [Arthrobacter sp. 11W110_air]
gi443481028	6.42	1	1	358	36.5	10.70	2.29	ferric enterobactin transport system permease [Arthrobacter nitrophenolicus]
gi403231796	4.82	1	1	332	37.1	9.33	2.29	hypothetical protein ARUE_232p00090 (plasmid) [Arthrobacter sp. Rue61a]
gi443480031	8.70	2	1	253	27.3	5.40	2.29	ribonuclease HII [Arthrobacter nitrophenolicus]
gi910737245	7.51	1	1	213	22.5	5.14	2.29	anthranilate synthase component 2 [Arthrobacter sp. Hiyo4]
gi910692723	5.31	1	1	245	25.8	6.29	2.29	L-Asparaginase [Arthrobacter sp. Hiyo6]
gi359307409	5.50	1	1	218	23.8	6.35	2.29	putative GntR family transcriptional regulator [Arthrobacter globiformis NBRC 12137]
gi918268179	9.66	2	1	176	18.9	7.36	2.29	hypothetical protein AHiyo1_15300 [Arthrobacter sp. Hiyo1]
gi918266640	6.91	1	1	304	34.0	5.96	2.29	formyltetrahydrofolate deformylase [Arthrobacter sp. Hiyo1]
gi443480129	7.64	1	1	144	15.2	9.36	2.28	hypothetical protein G205_20629 [Arthrobacter nitrophenolicus]
gi916782050	12.99	1	1	177	18.3	5.01	2.28	hypothetical protein [Arthrobacter sp. 35W]
gi651438728	2.43	1	1	453	49.2	4.88	2.28	putrescine oxidase [Arthrobacter sp. H14]
gi476399763	7.22	1	1	194	21.8	8.31	2.28	hypothetical protein D477_017052 [Arthrobacter crystallopoietes BAB-32]
gi640196718	5.49	1	1	255	26.5	5.64	2.28	short-chain dehydrogenase [Arthrobacter sp. 31Y]
gi636846192	7.26	1	1	179	18.4	5.07	2.28	GCN5 family acetyltransferase [Arthrobacter sp. TB 26]
gi742069585	3.47	1	1	317	34.6	9.86	2.28	hypothetical protein ANMWB30_33820 [Arthrobacter sp. MWB30]
gi654815177	4.42	4	1	430	47.0	5.26	2.28	cysteine desulfurase [Arthrobacter sp. PAO19]
gi323467746	1.83	1	1	981	104.4	5.33	2.28	sarcosine oxidase, alpha subunit family, heterotetrameric form [Arthrobacter phenanthrenivo
gi219857943	4.46	1	1	426	46.8	5.24	2.28	seryl-tRNA synthetase [Arthrobacter chlorophenolicus A6]
gi916782316	3.09	1	1	518	54.2	4.84	2.28	hypothetical protein [Arthrobacter sp. 35W]
gi757626070	17.05	1	1	88	9.6	9.66	2.28	Rho termination factor [Arthrobacter sp. SPG23]
gi219859088	4.92	1	1	447	48.1	4.82	2.28	peptidase M16 domain protein [Arthrobacter chlorophenolicus A6]
gi749401042	7.57	1	1	304	33.5	7.42	2.28	amino acid transporter, partial [Arthrobacter sp. AK-YN10]
gi635352590	4.59	1	1	283	30.0	5.07	2.28	rhodanese-like domain protein [Arthrobacter siccitolerans]
gi162953422	2.71	1	1	553	60.3	4.98	2.28	Xaa-Pro aminopeptidase [Renibacterium salmoninarum ATCC 33209]
gi359305472	3.52	1	1	454	48.0	8.09	2.28	hypothetical protein ARGLB_064_00210 [Arthrobacter globiformis NBRC 12137]
gi742756912	2.53	1	1	950	100.7	5.49	2.28	glycine dehydrogenase [Arthrobacter phenanthrenivorans]
gi927294469	7.50	2	1	240	24.6	4.72	2.28	hypothetical protein AL755_11345 [Arthrobacter sp. ERGS1:01]
gi939051203	16.67	1	1	90	9.7	9.50	2.28	hypothetical protein [Arthrobacter sp. JCM 19049]
gi651495900	2.48	1	1	323	34.8	6.54	2.28	hypothetical protein [Arthrobacter sp. H20]
gi476399053	3.86	1	1	544	57.4	5.14	2.28	phosphoglucomutase [Arthrobacter crystallopoietes BAB-32]
gi910697163	3.93	1	1	305	32.2	7.24	2.28	protein p49, partial [Arthrobacter sp. Hiyo6]
gi908698746	4.18	1	1	263	28.2	5.07	2.28	trehalose-phosphatase [Arthrobacter sp. RIT-PI-e]
gi476401437	4.53	1	1	353	39.6	5.83	2.28	ATP-dependent DNA ligase [Arthrobacter crystallopoietes BAB-32]
gi908697533	2.48	1	1	606	66.1	5.20	2.28	glycerophosphodiester phosphodiesterase [Arthrobacter sp. RIT-PI-e]
gi651438746	17.14	2	1	140	15.5	11.85	2.28	peptide chain release factor 1 [Arthrobacter sp. H14]
gi723609223	3.91	1	1	563	59.2	10.48	2.28	ATP-dependent RNA helicase [Arthrobacter sp. PAMC25486]
gi651431449	8.11	1	1	296	30.4	5.33	2.28	hypothetical protein [Arthrobacter sanguinis]

gi654823533	7.69	3	1	325	35.6	8.12	2.28	NUDIX hydrolase [Arthrobacter sp. I3]
gi551256777	5.28	1	1	265	28.7	4.91	2.28	nitrilase [Arthrobacter sp. PAO19]
gi518311692	3.05	1	1	524	56.7	4.86	2.28	hypothetical protein [Arthrobacter sp. TB 23]
gi910697804	8.33	1	1	180	20.0	7.31	2.28	hypothetical protein AHiyo6_00750 [Arthrobacter sp. Hiyo6]
gi517600373	3.33	1	1	600	58.5	6.07	2.28	serine protease [Arthrobacter sp. 162MFSha1.1]
gi767259345	1.87	1	1	587	63.4	5.52	2.28	hypothetical protein UM93_16710 [Arthrobacter sp. IHBB 11108]
gi651429248	6.16	1	1	146	16.1	6.43	2.28	hypothetical protein [Arthrobacter sanguinis]
gi116610250	7.14	1	1	308	32.8	6.33	2.28	ribosomal large subunit pseudouridine synthase D [Arthrobacter sp. FB24]
gi723608345	6.79	1	1	280	29.8	9.00	2.28	50S ribosomal protein L4/L1 family protein [Arthrobacter sp. PAMC25486]
gi219859419	13.68	1	1	117	11.3	12.31	2.28	conserved hypothetical protein [Arthrobacter chlorophenolicus A6]
gi937257939	6.82	1	1	308	32.7	5.66	2.28	aldose epimerase [Arthrobacter sp. Edens01]
gi219861373	3.65	1	1	438	48.1	5.22	2.28	AAA ATPase central domain protein [Arthrobacter chlorophenolicus A6]
gi652425639	22.37	1	1	76	8.9	10.39	2.28	hypothetical protein [Arthrobacter castelli]
gi651434916	1.98	1	1	404	43.5	9.47	2.28	pyridine nucleotide-disulfide oxidoreductase [Arthrobacter sp. H41]
gi910283573	11.16	2	1	224	24.1	6.00	2.28	GTP cyclohydrolase [Arthrobacter sp. A3]
gi307744889	2.43	1	1	535	57.1	4.93	2.28	putative amidohydrolase family protein [Arthrobacter arilaitensis Re117]
gi651441088	4.52	1	1	177	19.3	5.26	2.28	hypothetical protein [Arthrobacter sp. 9MFCol3.1]
gi518314042	4.42	1	1	385	40.5	10.39	2.28	hypothetical protein [Arthrobacter sp. TB 23]
gi737787543	3.24	1	1	586	65.1	4.98	2.28	2-isopropylmalate synthase [Arthrobacter albus]
gi917529968	1.87	3	1	482	53.7	5.15	2.28	lysine--tRNA ligase [Arthrobacter sp. PAMC25486]
gi651444109	4.19	1	1	310	34.0	5.31	2.28	hypothetical protein [Arthrobacter nicotinovorans]
gi928488591	6.63	1	1	347	36.9	5.31	2.27	phosphoesterase [Arthrobacter alpinus]
gi918265142	11.35	1	1	141	14.9	10.68	2.27	hypothetical protein AHiyo1_51550 [Arthrobacter sp. Hiyo1]
gi928486383	14.13	1	1	92	9.7	4.97	2.27	hypothetical protein AOC05_04180 [Arthrobacter alpinus]
gi914714772	8.00	1	1	200	18.8	8.88	2.27	hypothetical protein [Arthrobacter sp. ZBG10]
gi908697213	6.38	1	1	329	35.3	5.53	2.27	aldo/keto reductase [Arthrobacter sp. RIT-PI-e]
gi937258360	1.57	2	1	573	64.2	5.83	2.27	hypothetical protein AO716_09850 [Arthrobacter sp. Edens01]
gi908691012	2.35	1	1	468	50.2	6.73	2.27	hypothetical protein [Arthrobacter sp. H41]
gi651504853	4.76	1	1	294	30.8	9.85	2.27	1,4-dihydroxy-2-naphthoate octaprenyltransferase [Arthrobacter sp. 35W]
gi651438146	4.76	1	1	294	30.8	8.87	2.27	1,4-dihydroxy-2-naphthoate octaprenyltransferase [Arthrobacter sp. H14]
gi648575451	6.45	1	1	279	30.9	9.45	2.27	sugar ABC transporter permease [Arthrobacter sp. 161MFSha2.1]
gi654813906	1.85	2	1	1081	120.0	5.94	2.27	formate dehydrogenase [Arthrobacter sp. MA-N2]
gi651435972	7.63	1	1	118	13.0	6.79	2.27	hypothetical protein [Arthrobacter sp. H41]
gi910693797	5.69	1	1	211	22.7	5.49	2.27	probable lipid kinase YegS-like, partial [Arthrobacter sp. Hiyo6]
gi910746321	4.52	1	1	354	36.2	9.50	2.27	uncharacterized MFS-type transporter YcnB [Arthrobacter sp. Hiyo8]
gi651434620	4.89	2	1	348	37.2	7.39	2.27	epimerase [Arthrobacter sp. H41]
gi910738855	14.40	1	1	125	13.4	10.43	2.27	hypothetical protein AHiyo4_16770 [Arthrobacter sp. Hiyo4]
gi930825408	6.90	2	1	348	37.7	6.54	2.27	luciferase [Arthrobacter arilaitensis]
gi742851444	7.69	1	1	208	22.6	5.55	2.27	methyltransferase type 12 [Arthrobacter sp. W1]
gi651438624	4.06	1	1	493	52.0	8.59	2.27	hypothetical protein [Arthrobacter sp. H14]
gi443480585	8.53	1	1	129	13.9	7.61	2.27	Urease accessory protein UreF [Arthrobacter nitrophenolicus]
gi823668330	4.62	1	1	433	46.0	5.14	2.27	hypothetical protein AA310_03010 [Arthrobacter sp. YC-RL1]
gi648224145	3.82	1	1	497	53.4	5.47	2.27	pyruvate kinase [Arthrobacter sp. M2012083]
gi518311145	4.56	1	1	461	46.9	9.57	2.27	MULTISPECIES: hypothetical protein [Arthrobacter]
gi910695644	4.81	2	1	312	32.9	10.77	2.27	probable peptide ABC transporter permease protein y4tP [Arthrobacter sp. Hiyo6]
gi518311086	5.49	1	1	273	30.1	8.31	2.27	MULTISPECIES: hypothetical protein [Arthrobacter]
gi636844232	7.14	1	1	126	13.6	9.45	2.27	sulfurtransferase [Arthrobacter sp. TB 26]
gi307746355	13.64	1	1	88	9.8	9.74	2.27	conserved hypothetical protein [Arthrobacter arilaitensis Re117]
gi636846445	6.03	1	1	282	31.1	6.18	2.27	ABC transporter, partial [Arthrobacter sp. TB 26]
gi517604579	3.87	1	1	439	45.4	8.78	2.27	membrane protein [Arthrobacter sp. 131MFCol6.1]
gi916781870	4.34	1	1	438	45.9	5.60	2.27	hypothetical protein [Arthrobacter sp. 35W]
gi359304831	2.11	1	1	617	63.3	11.60	2.27	hypothetical protein ARGLB_080_01455 [Arthrobacter globiformis NBRC 12137]
gi737809103	3.95	1	1	430	46.0	5.94	2.27	nicotinate phosphoribosyltransferase [Arthrobacter sp. H5]
gi919108276	1.11	2	1	1356	148.5	5.40	2.27	hypothetical protein [Arthrobacter sp. IHBB 11108]
gi927033414	5.75	1	1	313	33.0	4.98	2.27	ornithine cyclodeaminase [Arthrobacter sp. LS16]

gi651457104	12.14	1	1	140	14.8	9.29	2.27	MerR family transcriptional regulator [Arthrobacter sp. 35/47]
gi651444708	3.10	1	1	516	56.0	4.84	2.27	glycosyl hydrolase family 32 [Arthrobacter nicotinovorans]
gi476401744	2.37	1	1	885	91.9	6.61	2.27	fatty acid Co-A ligase [Arthrobacter crystallopoietes BAB-32]
gi307745860	4.09	1	1	440	47.2	5.99	2.27	glycine hydroxymethyltransferase [Arthrobacter arilaitensis Re117]
gi908698193	2.78	1	1	503	56.2	4.92	2.27	lysine--tRNA ligase [Arthrobacter sp. RIT-PI-e]
gi765008421	8.11	1	1	148	16.3	6.57	2.27	acetyltransferase [Arthrobacter sp. A3]
gi927293823	4.97	1	1	302	32.3	5.81	2.27	LysR family transcriptional regulator [Arthrobacter sp. ERGS1:01]
gi917442142	6.69	3	1	284	30.9	8.69	2.27	hypothetical protein [Arthrobacter albus]
gi927033979	15.49	1	1	71	7.7	7.27	2.27	hypothetical protein AFL94_05745 [Arthrobacter sp. LS16]
gi651464965	5.45	1	1	202	21.7	5.00	2.27	hypothetical protein [Arthrobacter sp. 35/47]
gi639130677	2.44	1	1	657	72.3	5.55	2.27	WYL domain-containing protein [Arthrobacter sp. CAL618]
gi517592001	7.50	1	1	160	16.9	7.05	2.27	ArsR family transcriptional regulator [Arthrobacter sp. 135MFCol5.1]
gi542110546	4.10	1	1	390	41.1	5.54	2.27	glutamine--scyllo-inositol aminotransferase [Arthrobacter sp. AK-YN10]
gi908699232	1.29	1	1	1009	109.1	6.06	2.27	monooxygenase [Arthrobacter sp. RIT-PI-e]
gi823668412	6.88	1	1	218	22.5	8.18	2.27	hypothetical protein AA310_02705 [Arthrobacter sp. YC-RL1]
gi742757912	6.76	1	1	414	44.4	5.00	2.27	ferrochelatase [Arthrobacter phenanthrenivorans]
gi930826409	6.25	1	1	160	18.1	6.93	2.27	hypothetical protein AOZ07_09030 [Arthrobacter arilaitensis]
gi470220753	7.77	1	1	206	23.0	9.96	2.26	ribosomal-protein-alanine N-acetyltransferase [Arthrobacter gangotriensis Lz1y]
gi219861672	6.30	1	1	270	30.2	8.97	2.26	hypothetical protein Achl_4062 (plasmid) [Arthrobacter chlorophenolicus A6]
gi765013631	3.99	1	1	501	52.6	6.29	2.26	two-component system sensor histidine kinase [Arthrobacter sp. A3]
gi786034067	0.77	1	1	1163	124.0	5.88	2.26	1-pyrroline-5-carboxylate dehydrogenase [Arthrobacter chlorophenolicus]
gi658509391	4.98	1	1	221	23.2	5.21	2.26	phosphoglycerate mutase [Arthrobacter sp. TB 26]
gi786027320	10.94	1	1	192	20.6	5.24	2.26	GCN5 family acetyltransferase [Arthrobacter chlorophenolicus]
gi927296399	5.32	1	1	301	31.1	4.58	2.26	NGG1p interacting factor NIF3 [Arthrobacter sp. ERGS1:01]
gi910746678	29.73	1	1	74	7.6	9.44	2.26	hypothetical protein AHiyo8_43800 [Arthrobacter sp. Hiyo8]
gi517606928	6.12	1	1	343	37.6	4.64	2.26	adenosine deaminase [Arthrobacter sp. 161MFSha2.1]
gi759442233	4.14	1	1	338	37.0	5.00	2.26	chorismate mutase [Bifidobacterium callitrichos]
gi116610571	3.91	1	1	384	40.7	6.70	2.26	oxidoreductase domain protein [Arthrobacter sp. FB24]
gi652423220	1.22	1	1	984	103.8	5.36	2.26	oxidoreductase [Arthrobacter castelli]
gi910695492	12.18	1	1	156	16.2	5.49	2.26	hydrogenase nickel incorporation protein HypB, partial [Arthrobacter sp. Hiyo6]
gi635353905	2.58	1	1	620	69.1	6.23	2.26	3'-5' exonuclease family protein [Arthrobacter siccitolerans]
gi823667914	2.78	1	1	396	42.4	5.99	2.26	peptidase M19 [Arthrobacter sp. YC-RL1]
gi823665311	3.32	1	1	331	34.3	4.75	2.26	hypothetical protein AA310_18055 [Arthrobacter sp. YC-RL1]
gi910250887	2.64	1	1	455	50.5	4.84	2.26	hypothetical protein [Arthrobacter siccitolerans]
gi640193625	4.64	1	1	431	47.3	10.39	2.26	membrane protein [Arthrobacter sp. 31Y]
gi916357985	8.97	1	1	234	27.1	8.03	2.26	hypothetical protein [Arthrobacter sp. 131MFCol6.1]
gi651499816	9.00	1	1	200	21.9	5.03	2.26	TetR family transcriptional regulator [Arthrobacter sp. 35W]
gi759733607	2.17	1	1	508	52.2	5.03	2.26	hypothetical protein [Arthrobacter sp. L77]
gi908697195	12.21	1	1	131	14.5	4.48	2.26	glyoxalase [Arthrobacter sp. RIT-PI-e]
gi930825753	3.87	1	1	543	58.7	4.75	2.26	ABC transporter substrate-binding protein [Arthrobacter arilaitensis]
gi517592280	6.29	1	1	334	37.3	6.24	2.26	hypothetical protein [Arthrobacter sp. 135MFCol5.1]
gi116613004	3.04	4	1	560	62.5	9.74	2.26	hypothetical protein Arth_4276 (plasmid) [Arthrobacter sp. FB24]
gi928488710	9.61	1	1	229	25.1	6.32	2.26	GlcNAc-PI de-N-acetylase [Arthrobacter alpinus]
gi651442047	7.10	2	1	169	18.7	9.55	2.26	hypothetical protein [Arthrobacter sp. 9MFCol3.1]
gi654813702	16.67	1	1	150	16.2	5.15	2.26	universal stress protein UspA [Arthrobacter sp. MA-N2]
gi651442365	1.69	1	1	1184	125.7	5.67	2.26	1-pyrroline-5-carboxylate dehydrogenase [Arthrobacter sp. 9MFCol3.1]
gi517605538	7.56	1	1	238	25.0	6.80	2.26	MULTISPECIES: ArsR family transcriptional regulator [Arthrobacter]
gi927296191	3.85	1	1	364	39.1	5.05	2.26	pyruvate dehydrogenase [Arthrobacter sp. ERGS1:01]
gi916574023	6.63	1	1	166	16.7	7.30	2.26	hypothetical protein [Arthrobacter sp. TB 26]
gi635350630	6.28	1	1	223	24.5	6.07	2.26	putative uncharacterized protein [Arthrobacter siccitolerans]
gi651440897	9.19	2	1	272	29.9	7.50	2.26	PEP synthetase regulatory protein [Arthrobacter sp. 9MFCol3.1]
gi651482543	2.70	2	1	333	36.7	7.62	2.26	Tat pathway signal protein [Arthrobacter sp. Br18]
gi162953743	12.61	1	1	119	13.5	4.78	2.26	hypothetical protein RSal33209_1522 [Renibacterium salmoninarum ATCC 33209]
gi517590385	4.46	1	1	561	57.1	9.44	2.26	hypothetical protein [Arthrobacter sp. 135MFCol5.1]
gi910742367	2.74	1	1	438	45.4	8.27	2.26	hypothetical protein AHiyo8_00690 [Arthrobacter sp. Hiyo8]

gi119948094	5.62	1	1	409	42.9	5.15	2.26	putative xanthine dehydrogenase accessory factor [Arthrobacter aurescens TC1]
gi518311844	10.43	1	1	115	12.2	8.82	2.26	membrane protein insertion efficiency factor YidD [Arthrobacter sp. TB 23]
gi823665363	3.08	1	1	585	62.5	6.93	2.26	23S rRNA methyltransferase [Arthrobacter sp. YC-RL1]
gi323470098	5.67	1	1	247	26.7	7.14	2.26	amino acid ABC transporter ATP-binding protein, PAAT family [Arthrobacter phenanthrenivor
gi517605266	5.74	1	1	296	29.8	5.54	2.26	2-hydroxy-3-oxopropionate reductase [Arthrobacter sp. 131MFCol6.1]
gi910697215	4.51	1	1	377	39.6	6.06	2.26	uncharacterized protein YJL213W [Arthrobacter sp. Hiyo6]
gi674646953	4.39	1	1	319	32.0	5.36	2.25	putative L-asparaginase [Arthrobacter sp. 11W110_air]
gi767258075	2.48	2	1	322	35.2	8.51	2.25	membrane protein [Arthrobacter sp. IHBB 11108]
gi651432906	8.06	1	1	248	26.7	7.12	2.25	hypothetical protein [Arthrobacter sp. H41]
gi652425742	4.28	1	1	374	40.0	4.96	2.25	2-polyprenyl-6-methoxyphenol hydroxylase [Arthrobacter castelli]
gi916359281	5.70	1	1	386	39.3	5.76	2.25	molybdopterin molybdenumtransferase MoeA [Arthrobacter sp. 135MFCol5.1]
gi742072447	6.60	2	1	197	19.5	5.87	2.25	lipoprotein [Arthrobacter sp. MWB30]
gi654811269	34.33	1	1	67	7.4	10.59	2.25	hypothetical protein [Arthrobacter sp. MA-N2]
gi640194001	1.50	1	1	1604	171.8	6.55	2.25	hypothetical protein [Arthrobacter sp. 31Y]
gi323468273	4.74	2	1	380	41.1	6.43	2.25	DNA polymerase III, delta' subunit [Arthrobacter phenanthrenivorans Sphe3]
gi648260004	30.59	1	1	85	8.9	9.52	2.25	hypothetical protein [Arthrobacter sp. TB 23]
gi470216638	5.80	2	1	276	29.1	4.98	2.25	hypothetical protein ADIAG_03253 [Arthrobacter gangotriensis Lz1y]
gi651501618	7.21	1	1	208	23.7	10.95	2.25	30S ribosomal protein S4 [Arthrobacter sp. 35W]
gi757623579	2.84	2	1	529	53.9	5.06	2.25	phage-shock protein [Arthrobacter sp. SPG23]
gi359303570	19.39	1	1	98	10.8	5.01	2.25	putative GntR family transcriptional regulator [Arthrobacter globiformis NBRC 12137]
gi515767468	5.83	1	1	240	26.3	5.81	2.25	hypothetical protein [Arthrobacter sp. M2012083]
gi542107110	3.83	1	1	261	28.0	5.91	2.25	glycosyl transferase family 8 [Arthrobacter sp. AK-YN10]
gi307745178	4.13	1	1	436	47.6	7.88	2.25	hypothetical membrane protein [Arthrobacter arilaitensis Re117]
gi930827612	10.73	1	1	177	19.5	7.83	2.25	hypothetical protein AOZ07_15875 [Arthrobacter arilaitensis]
gi823668543	2.86	1	1	559	62.8	7.66	2.25	DNA helicase UvrD [Arthrobacter sp. YC-RL1]
gi654814666	3.77	1	1	345	36.4	5.99	2.25	tRNA(Ile)-lysidine synthetase [Arthrobacter sp. MA-N2]
gi910738303	14.05	1	1	121	12.7	10.35	2.25	HTH-type transcriptional regulator RegA [Arthrobacter sp. Hiyo4]
gi654811590	5.71	1	1	333	33.8	5.26	2.25	hydroxymethylbilane synthase [Arthrobacter sp. MA-N2]
gi654826408	4.55	1	1	264	27.2	5.27	2.25	triosephosphate isomerase [Arthrobacter sp. H5]
gi654822550	28.09	1	1	89	9.6	8.63	2.25	protein translocase TatA [Arthrobacter sp. I3]
gi116609662	18.97	1	1	58	6.3	4.51	2.25	hypothetical protein Arth_0989 [Arthrobacter sp. FB24]
gi916869944	9.94	1	1	171	17.3	5.10	2.25	hypothetical protein, partial [Arthrobacter sp. Br18]
gi651438994	8.47	2	1	177	19.1	6.20	2.25	hypothetical protein [Arthrobacter sp. H14]
gi916813599	3.55	1	1	338	36.0	5.69	2.25	peptidase M4 [Arthrobacter nicotinovorans]
gi916816438	2.98	1	1	537	56.3	4.78	2.25	glucose-methanol-choline oxidoreductase [Arthrobacter sp. MA-N2]
gi742757217	3.49	2	1	487	51.8	5.43	2.25	mannitol dehydrogenase [Arthrobacter phenanthrenivorans]
gi916869910	6.50	1	1	246	26.8	5.91	2.25	GntR family transcriptional regulator [Arthrobacter sp. Br18]
gi928985725	7.21	1	1	305	31.7	5.77	2.25	hypothetical protein [Arthrobacter sp. ERGS1:01]
gi910696780	5.73	1	1	314	34.1	6.13	2.25	protein involved in mismatch repair, partial [Arthrobacter sp. Hiyo6]
gi914715374	6.98	1	1	215	22.0	4.89	2.25	non-canonical purine NTP pyrophosphatase [Arthrobacter sp. ZBG10]
gi651444321	2.09	1	1	430	45.9	8.63	2.25	permease [Arthrobacter nicotinovorans]
gi723607469	1.99	1	1	752	83.3	4.91	2.25	hypothetical protein ART_1246 [Arthrobacter sp. PAMC25486]
gi162955741	2.96	1	1	574	60.7	10.08	2.25	virulence factor [Renibacterium salmoninarum ATCC 33209]
gi651441755	2.70	1	1	407	43.2	5.94	2.25	SAM-dependent methyltransferase [Arthrobacter sp. 9MFCol3.1]
gi476401294	6.76	1	1	355	36.4	5.60	2.25	Oxidoreductase, Zn-dependent and NAD(P)-binding protein [Arthrobacter crystallopoietes BA
gi759711228	2.68	1	1	336	35.6	5.92	2.25	LacI family transcriptional regulator [Arthrobacter sp. 135MFCol5.1]
gi765005422	8.23	1	1	231	25.4	5.53	2.25	hypothetical protein [Arthrobacter sp. A3]
gi652425008	4.08	1	1	466	50.4	6.05	2.25	hypothetical protein [Arthrobacter castelli]
gi916781590	3.33	2	1	360	40.4	9.88	2.25	hypothetical protein [Arthrobacter sp. 35W]
gi916782314	4.10	1	1	415	44.1	6.81	2.25	hypothetical protein [Arthrobacter sp. 35W]
gi914713586	5.48	1	1	292	32.3	9.07	2.25	XRE family transcriptional regulator [Arthrobacter sp. ZBG10]
gi910743843	16.67	1	1	114	12.2	8.15	2.25	6-phospho-alpha-glucosidase [Arthrobacter sp. Hiyo8]
gi930827273	4.32	1	1	278	30.6	9.70	2.25	alpha/beta hydrolase [Arthrobacter arilaitensis]
gi916926231	3.01	1	1	399	40.3	11.41	2.25	MFS transporter [Arthrobacter sp. 9MFCol3.1]
gi742851681	5.02	1	1	438	48.8	6.92	2.25	hypothetical protein [Arthrobacter sp. W1]

gi908698662	1.48	1	1	1215	131.3	5.58	2.25	transcription-repair coupling factor [Arthrobacter sp. RIT-PI-e]
gi927294487	3.26	1	1	491	54.8	5.06	2.25	Fe-S cluster assembly protein SufB [Arthrobacter sp. ERGS1:01]
gi654825563	4.30	1	1	395	42.4	6.67	2.24	phosphodiesterase [Arthrobacter sp. H5]
gi517604105	4.73	1	1	338	36.2	5.96	2.24	aldo/keto reductase [Arthrobacter sp. 131MFCol6.1]
gi651437821	6.35	1	1	299	32.7	7.18	2.24	dehydratase [Arthrobacter sp. H14]
gi545111035	2.39	1	1	419	46.7	4.84	2.24	hypothetical protein [Arthrobacter sp. AK-YN10]
gi937262210	5.95	1	1	252	27.7	5.86	2.24	iron ABC transporter ATP-binding protein [Arthrobacter sp. Edens01]
gi219862060	6.19	1	1	291	32.0	5.63	2.24	hypothetical protein Achl_4450 (plasmid) [Arthrobacter chlorophenolicus A6]
gi648573109	7.60	1	1	342	37.0	5.59	2.24	iditol 2-dehydrogenase [Arthrobacter sp. 135MFCol5.1]
gi517607727	1.40	1	1	645	69.7	6.19	2.24	hypothetical protein [Arthrobacter sp. 161MFSha2.1]
gi307743772	7.50	2	1	280	30.8	4.92	2.24	putative 2,5-didehydrogluconate reductase [Arthrobacter arilaitensis Re117]
gi323469157	2.37	1	1	422	47.6	5.66	2.24	hypothetical protein Asphe3_16850 [Arthrobacter phenanthrenivorans Sphe3]
gi786032123	5.73	1	1	279	29.6	4.78	2.24	hypothetical protein, partial [Arthrobacter chlorophenolicus]
gi918269039	5.14	1	1	214	22.2	9.26	2.24	hypothetical protein AHiyo1_06320 [Arthrobacter sp. Hiyo1]
gi927034091	8.59	1	1	163	18.8	10.92	2.24	hypothetical protein AFL94_10795 [Arthrobacter sp. LS16]
gi937259491	3.26	1	1	583	63.6	9.63	2.24	long-chain fatty acid--CoA ligase [Arthrobacter sp. Edens01]
gi651454897	6.88	2	1	320	34.5	4.91	2.24	peptidase M19 [Arthrobacter nicotinovorans]
gi937258535	3.31	1	1	513	53.2	6.64	2.24	Mg chelatase-like protein [Arthrobacter sp. Edens01]
gi759732332	3.54	1	1	339	35.2	5.16	2.24	universal stress protein UspA [Arthrobacter sp. L77]
gi476399340	4.20	1	1	500	54.9	7.03	2.24	nucleoside-diphosphate sugar epimerase [Arthrobacter crystallopoietes BAB-32]
gi504874622	2.21	1	1	589	62.8	8.79	2.24	traG-family protein [Arthrobacter sp. 31-32]
gi359304869	12.64	1	1	87	8.6	8.51	2.24	putative pilin component [Arthrobacter globiformis NBRC 12137]
gi910748477	6.74	1	1	282	30.3	10.71	2.24	uncharacterized transporter Mb2022c [Arthrobacter sp. Hiyo8]
gi639130344	5.73	1	1	279	29.4	6.80	2.24	GTPase, partial [Arthrobacter sp. CAL618]
gi517592157	8.16	1	1	196	21.7	6.24	2.24	hypothetical protein [Arthrobacter sp. 135MFCol5.1]
gi359303805	4.40	1	1	637	65.6	9.95	2.24	hypothetical protein ARGLB_097_00020 [Arthrobacter globiformis NBRC 12137]
gi767258906	3.52	1	1	426	45.1	8.32	2.24	hypothetical protein UM93_01770 [Arthrobacter sp. IHBB 11108]
gi918267964	6.85	1	1	336	35.9	6.42	2.24	catabolite control protein A [Arthrobacter sp. Hiyo1]
gi928488653	4.42	1	1	249	27.0	9.44	2.24	cytidylate kinase [Arthrobacter alpinus]
gi910738410	2.17	1	1	644	70.0	5.97	2.24	putative HTH-type transcriptional regulator Rv0890c/MT0914 [Arthrobacter sp. Hiyo4]
gi359304990	3.39	2	1	383	39.6	5.50	2.24	sugar ABC transporter sugar-binding protein [Arthrobacter globiformis NBRC 12137]
gi910697602	13.33	1	1	165	18.0	6.95	2.24	conserved hypothetical protein [Arthrobacter sp. Hiyo6]
gi119948553	6.49	1	1	262	27.0	4.86	2.24	oxidoreductase, short chain dehydrogenase/reductase family [Arthrobacter aurescens TC1]
gi823668198	4.18	1	1	407	43.9	4.53	2.24	ATP-binding protein [Arthrobacter sp. YC-RL1]
gi908699302	6.69	1	1	254	26.5	5.33	2.24	3-oxoacyl-ACP reductase [Arthrobacter sp. RIT-PI-e]
gi937257881	1.96	1	1	459	50.9	5.15	2.24	glutamine synthetase [Arthrobacter sp. Edens01]
gi928487215	5.80	1	1	362	37.8	5.35	2.24	hypothetical protein AOC05_09960 [Arthrobacter alpinus]
gi916814539	6.90	2	1	261	29.0	8.48	2.24	arginine ABC transporter ATP-binding protein [Arthrobacter nicotinovorans]
gi910745527	5.97	1	1	134	14.4	10.86	2.24	3-oxoacyl-[acyl-carrier-protein] reductase 1 [Arthrobacter sp. Hiyo8]
gi219858146	3.43	1	1	350	38.1	6.61	2.24	NAD-dependent epimerase/dehydratase [Arthrobacter chlorophenolicus A6]
gi930827628	1.94	1	1	463	51.7	5.30	2.24	glutamine synthetase [Arthrobacter arilaitensis]
gi323471294	5.03	1	1	358	40.3	5.48	2.24	ATP-dependent DNA ligase [Arthrobacter phenanthrenivorans Sphe3]
gi651504344	4.93	1	1	365	38.5	7.28	2.24	hypothetical protein [Arthrobacter sp. 35W]
gi518312816	8.52	1	1	176	18.8	5.34	2.24	hypothetical protein [Arthrobacter sp. TB 23]
gi759713891	4.82	1	1	353	37.1	6.65	2.24	DNA processing protein DprA, partial [Arthrobacter sp. AK-YN10]
gi517599078	2.50	1	1	841	92.5	5.16	2.24	leucine--tRNA ligase [Arthrobacter sp. 162MFSha1.1]
gi786028902	7.72	1	1	272	29.9	5.35	2.24	ubiquinone biosynthesis methyltransferase UbiE [Arthrobacter chlorophenolicus]
gi219861924	8.33	1	1	288	32.9	9.91	2.24	helicase-associated (plasmid) [Arthrobacter chlorophenolicus A6]
gi162954165	4.90	1	1	204	22.4	6.92	2.24	transcriptional regulator, TetR family [Renibacterium salmoninarum ATCC 33209]
gi307743324	1.60	1	1	1060	117.0	5.45	2.24	glycosyl hydrolase, family 65 [Arthrobacter arilaitensis Re117]
gi651439049	3.27	1	1	367	38.9	5.73	2.24	oxidoreductase [Arthrobacter sp. H14]
gi765010889	4.07	1	1	344	37.1	10.04	2.24	hypothetical protein [Arthrobacter sp. A3]
gi651503432	7.50	1	1	240	26.7	5.24	2.24	chlorite dismutase [Arthrobacter sp. 35W]
gi654817306	10.77	1	1	130	13.6	9.72	2.24	epimerase [Arthrobacter sp. UNC362MFTsu5.1]
gi654823101	4.91	1	1	346	36.9	6.09	2.24	capsular biosynthesis protein [Arthrobacter sp. I3]

gi674646748	6.47	1	1	232	23.4	6.68	2.24	Thiamine-phosphate synthase [Arthrobacter sp. 11W110_air]
gi219859112	4.68	1	1	299	32.7	6.90	2.24	tRNA delta(2)-isopentenylpyrophosphate transferase [Arthrobacter chlorophenolicus A6]
gi654819718	4.99	1	1	341	36.9	5.08	2.24	hypothetical protein [Arthrobacter sp. UNC362MFTsu5.1]
gi910844926	4.29	1	1	490	53.4	9.92	2.23	polyprenyl glycosylphosphotransferase [Arthrobacter sp. ZBG10]
gi470216165	7.85	1	1	191	20.8	5.17	2.23	peptide deformylase [Arthrobacter gangotriensis Lz1y]
gi654812205	15.48	1	1	155	16.8	8.91	2.23	transcriptional regulator [Arthrobacter sp. MA-N2]
gi476400517	10.32	1	1	155	16.4	5.05	2.23	carboxypeptidase YodJ [Arthrobacter crystallopoietes BAB-32]
gi742756236	4.64	1	1	345	37.3	5.10	2.23	pyruvate dehydrogenase [Arthrobacter phenanthrenivorans]
gi654819368	6.25	2	1	352	36.9	5.97	2.23	LacI family transcriptional regulator [Arthrobacter sp. UNC362MFTsu5.1]
gi914717668	7.64	1	1	157	16.4	7.36	2.23	thioesterase [Arthrobacter sp. ZBG10]
gi542109779	3.45	1	1	464	49.8	5.64	2.23	hypothetical protein M707_03665 [Arthrobacter sp. AK-YN10]
gi910283787	13.95	1	1	86	10.1	6.04	2.23	hypothetical protein [Arthrobacter sp. A3]
gi162952537	9.29	1	1	183	19.2	9.25	2.23	conserved hypothetical membrane protein [Renibacterium salmoninarum ATCC 33209]
gi636846863	11.52	1	1	165	18.4	7.27	2.23	hypothetical protein [Arthrobacter sp. TB 26]
gi651455092	7.09	1	1	268	26.9	4.93	2.23	molybdate-binding protein [Arthrobacter nicotinovorans]
gi635353132	4.08	1	1	515	53.3	5.97	2.23	GDSL-like Lipase/Acylhydrolase family protein [Arthrobacter siccitolerans]
gi927294865	3.67	1	1	327	33.4	5.49	2.23	sulfonate ABC transporter substrate-binding protein [Arthrobacter sp. ERGS1:01]
gi651430210	13.48	2	1	89	10.0	11.40	2.23	50S ribosomal protein L28 [Arthrobacter sanguinis]
gi927032278	6.57	1	1	274	29.7	8.88	2.23	UDP-diphosphatase [Arthrobacter sp. LS16]
gi551254175	3.08	1	1	454	46.9	5.48	2.23	D-alanyl-D-alanine carboxypeptidase [Arthrobacter sp. PAO19]
gi652424244	6.35	1	1	362	38.9	5.15	2.23	polyprenyl synthetase [Arthrobacter castelli]
gi937262020	3.01	1	1	299	31.5	10.61	2.23	hypothetical protein AO716_02015 [Arthrobacter sp. Edens01]
gi916692309	2.61	1	1	574	63.8	4.63	2.23	hypothetical protein [Arthrobacter castelli]
gi916813928	2.50	1	1	761	82.2	5.64	2.23	glycosyl hydrolase [Arthrobacter nicotinovorans]
gi652424899	5.21	1	1	365	40.1	5.07	2.23	hypothetical protein [Arthrobacter castelli]
gi928487931	2.75	1	1	291	31.8	10.08	2.23	LysR family transcriptional regulator [Arthrobacter alpinus]
gi651486920	11.27	1	1	142	15.6	6.52	2.23	diadenosine tetraphosphate hydrolase [Arthrobacter sp. Br18]
gi651484600	8.53	1	1	258	28.1	5.83	2.23	glutamine ABC transporter ATP-binding protein [Arthrobacter sp. Br18]
gi651461998	20.00	2	1	85	9.1	6.64	2.23	hypothetical protein [Arthrobacter sp. 35/47]
gi443479666	10.70	1	1	187	21.2	6.54	2.23	NUDIX hydrolase [Arthrobacter nitrophenolicus]
gi742755718	0.85	1	1	1061	115.2	6.49	2.23	beta-phosphoglucomutase [Arthrobacter phenanthrenivorans]
gi545108942	12.24	1	1	147	15.2	10.14	2.23	membrane protein [Arthrobacter sp. AK-YN10]
gi518313548	3.16	1	1	348	39.0	4.96	2.23	hypothetical protein [Arthrobacter sp. TB 23]
gi910744032	1.84	1	1	705	73.9	10.02	2.23	hypothetical protein AHiyo8_17340 [Arthrobacter sp. Hiyo8]
gi786027580	3.35	1	1	477	50.5	8.25	2.23	amino acid transporter [Arthrobacter chlorophenolicus]
gi119947933	2.13	1	1	469	47.5	5.71	2.23	rfaE bifunctional protein [Arthrobacter aurescens TC1]
gi759734578	6.73	1	1	208	21.9	5.27	2.23	DNA-binding response regulator [Arthrobacter sp. L77]
gi654813490	6.09	1	1	345	37.8	5.94	2.23	ATPase [Arthrobacter sp. MA-N2]
gi636844100	11.89	1	1	185	19.8	6.20	2.23	oxidoreductase, partial [Arthrobacter sp. TB 26]
gi517598256	4.11	1	1	341	35.8	6.54	2.23	hypothetical protein [Arthrobacter sp. 162MFSha1.1]
gi654826692	7.31	1	1	342	37.2	8.85	2.23	cytochrome C biogenesis protein [Arthrobacter sp. H5]
gi918266949	9.05	1	1	210	21.3	4.56	2.23	metalloreductase STEAP4 [Arthrobacter sp. Hiyo1]
gi162955371	3.83	1	1	548	56.3	5.40	2.23	metalloprotease [Renibacterium salmoninarum ATCC 33209]
gi757623918	8.29	1	1	181	19.1	7.96	2.23	pyridoxamine 5-phosphate oxidase [Arthrobacter sp. SPG23]
gi116610440	3.46	1	1	260	28.5	5.73	2.23	two component transcriptional regulator, winged helix family [Arthrobacter sp. FB24]
gi910742235	10.26	1	1	156	17.8	5.72	2.23	protocatechuate 3,4-dioxygenase beta chain [Arthrobacter sp. Hiyo4]
gi916876121	11.98	1	1	192	21.1	9.89	2.23	hypothetical protein [Arthrobacter sp. 31Y]
gi908697415	1.86	1	1	538	58.1	5.21	2.23	phytoene dehydrogenase [Arthrobacter sp. RIT-PI-e]
gi918266663	6.88	1	1	320	34.0	9.79	2.23	probable ABC transporter permease protein YurN [Arthrobacter sp. Hiyo1]
gi910834043	9.22	1	1	141	15.6	9.17	2.23	hypothetical protein ACU18_18830, partial [Arthrobacter sp. ZBG10]
gi737786658	3.36	1	1	298	32.4	5.38	2.23	hypothetical protein [Arthrobacter albus]
gi928488480	1.81	1	1	720	76.7	6.18	2.23	recombinase RecQ [Arthrobacter alpinus]
gi917013527	26.92	1	1	104	11.9	11.31	2.22	hypothetical protein [Arthrobacter sanguinis]
gi517602548	3.95	1	1	582	60.5	6.11	2.22	hypothetical protein [Arthrobacter sp. 131MFCol6.1]
gi119949047	5.68	1	1	387	41.8	6.02	2.22	putative glutaryl-CoA dehydrogenase [Arthrobacter aurescens TC1]

gi635350870	27.78	1	1	72	8.1	4.67	2.22	DNA binding, excisionase family domain protein [Arthrobacter siccitolerans]
gi654813044	4.32	1	1	347	38.1	6.81	2.22	tryptophan--tRNA ligase [Arthrobacter sp. MA-N2]
gi652423016	5.68	1	1	352	36.3	5.40	2.22	anthranilate phosphoribosyltransferase [Arthrobacter castelli]
gi908690616	6.21	1	1	354	38.1	7.12	2.22	LacI family transcriptional regulator [Arthrobacter sp. H41]
gi928487460	4.70	1	1	234	25.8	5.24	2.22	hypothetical protein AOC05_11670 [Arthrobacter alpinus]
gi910740657	8.18	1	1	220	22.5	5.88	2.22	sugar isomerase [Arthrobacter sp. Hiyo4]
gi910249447	13.67	1	1	139	15.2	4.91	2.22	arsenate reductase [Arthrobacter siccitolerans]
gi640199319	5.85	1	1	325	34.4	5.17	2.22	serine hydrolase [Arthrobacter sp. 31Y]
gi551255883	2.74	1	1	511	53.2	9.10	2.22	hypothetical protein [Arthrobacter sp. PAO19]
gi939051104	8.72	1	1	172	18.8	5.80	2.22	transferase [Arthrobacter sp. JCM 19049]
gi542109612	2.94	1	1	477	49.3	9.70	2.22	sodium:proton antiporter [Arthrobacter sp. AK-YN10]
gi652423720	5.32	1	1	432	46.9	5.14	2.22	hypothetical protein [Arthrobacter castelli]
gi652422495	1.05	1	1	859	90.1	4.92	2.22	RND transporter [Arthrobacter castelli]
gi759703855	4.97	1	1	342	37.1	5.71	2.22	GCN5 family acetyltransferase [Arthrobacter globiformis]
gi551255982	7.39	1	1	230	24.0	5.48	2.22	hypothetical protein [Arthrobacter sp. PAO19]
gi916870087	4.61	1	1	304	31.8	5.02	2.22	hypothetical protein [Arthrobacter sp. Br18]
gi515764693	9.09	1	1	187	20.3	4.98	2.22	hypothetical protein [Arthrobacter sp. M2012083]
gi652422867	3.53	1	1	283	30.6	5.07	2.22	tRNA (guanine-N1)-methyltransferase [Arthrobacter castelli]
gi823667894	4.46	1	1	426	45.1	4.58	2.22	enolase [Arthrobacter sp. YC-RL1]
gi359306165	10.34	1	1	261	27.3	6.67	2.22	hypothetical protein ARGLB_047_01120 [Arthrobacter globiformis NBRC 12137]
gi640196885	4.12	1	1	486	52.6	6.54	2.22	hypothetical protein [Arthrobacter sp. 31Y]
gi746184670	3.22	1	1	497	54.0	6.27	2.22	PucR family transcriptional regulator [Arthrobacter sp. MWB30]
gi654827332	5.65	1	1	478	50.6	6.18	2.22	hypothetical protein [Arthrobacter sp. H5]
gi674644947	2.84	1	1	387	39.9	4.68	2.22	Quinoprotein glucose dehydrogenase B precursor [Arthrobacter sp. 11W110_air]
gi410690433	7.36	1	1	258	29.2	11.37	2.22	hypothetical protein (plasmid) [Arthrobacter sp. J3-49]
gi910249145	2.90	1	1	689	69.6	5.19	2.22	tape measure domain-containing protein [Arthrobacter siccitolerans]
gi910697013	5.80	1	1	345	36.7	8.44	2.22	uncharacterized protein HI_1028 [Arthrobacter sp. Hiyo6]
gi654816227	2.55	1	1	353	37.7	9.31	2.22	hypothetical protein [Arthrobacter sp. UNC362MFTsu5.1]
gi551254494	2.17	1	1	461	49.9	4.86	2.22	allantoicase [Arthrobacter sp. PAO19]
gi765004876	2.81	1	1	392	40.1	8.40	2.22	potassium transporter [Arthrobacter sp. A3]
gi823665891	1.99	1	1	503	52.5	4.83	2.22	flavoprotein [Arthrobacter sp. YC-RL1]
gi640193266	3.40	1	1	500	54.5	5.83	2.22	ribose ABC transporter ATP-binding protein [Arthrobacter sp. 31Y]
gi517609079	8.07	1	1	223	22.6	10.24	2.22	hypothetical protein [Arthrobacter sp. 161MFSha2.1]
gi930827190	4.34	1	1	369	39.6	5.62	2.22	oxidoreductase [Arthrobacter arilaitensis]
gi443482390	6.61	1	1	257	27.2	5.92	2.22	sugar metabolism transcriptional regulator [Arthrobacter nitrophenolicus]
gi823666606	2.73	1	1	439	49.0	6.23	2.22	phenylacetate--CoA ligase [Arthrobacter sp. YC-RL1]
gi651433180	1.69	1	1	826	92.7	5.95	2.22	kojibiose phosphorylase [Arthrobacter sp. H41]
gi640203596	4.04	1	1	322	35.1	9.94	2.22	sugar ABC transporter permease [Arthrobacter sp. 31Y]
gi916863219	1.42	1	1	632	67.9	7.17	2.22	hypothetical protein [Arthrobacter sp. 35/47]
gi470217718	3.20	1	1	438	48.7	10.49	2.22	SH3 domain-containing protein [Arthrobacter gangotriensis Lz1y]
gi652425241	8.63	1	1	278	30.3	4.78	2.22	DNA-directed RNA polymerase sigma-70 factor [Arthrobacter castelli]
gi746182437	4.83	1	1	269	29.3	8.53	2.22	hypothetical protein [Arthrobacter sp. MWB30]
gi918267235	13.33	1	1	120	13.0	7.05	2.22	HTH-type transcriptional regulator KmtR [Arthrobacter sp. Hiyo1]
gi918266171	5.43	1	1	276	29.0	6.02	2.22	phospho-2-dehydro-3-deoxyheptonate aldolase [Arthrobacter sp. Hiyo1]
gi654818919	8.37	1	1	263	26.8	4.96	2.22	aldolase [Arthrobacter sp. UNC362MFTsu5.1]
gi917760514	7.62	1	1	223	24.0	6.21	2.22	hypothetical protein [Arthrobacter sp. L77]
gi651507295	5.52	1	1	308	34.4	6.58	2.22	3-methyladenine DNA glycosylase [Arthrobacter sp. 35W]
gi652424837	3.72	1	1	323	35.0	5.16	2.22	ATPase [Arthrobacter castelli]
gi651481030	4.67	1	1	257	28.8	8.43	2.22	GntR family transcriptional regulator [Arthrobacter sp. Br18]
gi651465402	3.72	1	1	457	50.3	5.43	2.22	serine/threonine protein kinase [Arthrobacter sp. 35/47]
gi930827428	3.09	1	1	324	35.8	5.11	2.22	hypothetical protein AOZ07_14850 [Arthrobacter arilaitensis]
gi518314114	4.80	1	1	250	26.3	5.90	2.22	SDR family oxidoreductase [Arthrobacter sp. TB 23]
gi648224306	6.11	1	1	229	25.0	4.88	2.22	hypothetical protein [Arthrobacter sp. M2012083]
gi542108271	4.41	1	1	499	54.6	5.22	2.22	glycerol kinase [Arthrobacter sp. AK-YN10]
gi403231380	5.83	1	1	223	24.5	5.20	2.22	transcriptional regulator, GntR family [Arthrobacter sp. Rue61a]

gi652424793	1.26	1	1	636	71.9	5.30	2.22	hypothetical protein [Arthrobacter castelli]
gi916834965	3.72	1	1	591	64.0	4.54	2.22	phosphoesterase [Arthrobacter sp. H14]
gi910744605	5.13	1	1	273	28.3	10.32	2.21	hypothetical protein AHiyo8_23070 [Arthrobacter sp. Hiyo8]
gi760112094	2.82	1	1	568	61.4	5.94	2.21	ABC transporter ATP-binding protein [Arthrobacter chlorophenolicus]
gi914716634	2.45	1	1	858	91.1	9.64	2.21	hypothetical protein [Arthrobacter sp. ZBG10]
gi517607426	2.54	2	1	472	51.1	6.21	2.21	bifunctional dTDP-4-keto-L-rhamnose reductase/dTDP-4-keto-6-deoxyglucose-3,5-epimerase
gi914717142	6.69	2	1	344	35.7	5.33	2.21	glycerol-3-phosphate dehydrogenase [Arthrobacter sp. ZBG10]
gi916876266	3.83	1	1	444	47.0	7.05	2.21	acyl-CoA thioesterase [Arthrobacter sp. 31Y]
gi823668649	5.88	1	1	204	23.1	9.00	2.21	hypothetical protein AA310_00610 [Arthrobacter sp. YC-RL1]
gi927296536	4.02	1	1	373	38.4	5.14	2.21	hypothetical protein AL755_18490 [Arthrobacter sp. ERGS1:01]
gi651433784	5.29	1	1	416	43.1	4.94	2.21	phosphoglycerate kinase [Arthrobacter sp. H41]
gi403229643	2.66	1	1	414	45.5	5.57	2.21	putative DNA ligase-like protein [Arthrobacter sp. Rue61a]
gi910738448	7.78	1	1	270	28.6	6.14	2.21	probable serine/threonine-protein kinase CPE1738 [Arthrobacter sp. Hiyo4]
gi518312785	4.01	1	1	274	29.3	5.62	2.21	hypothetical protein, partial [Arthrobacter sp. TB 23]
gi916863315	3.25	1	1	308	33.3	5.92	2.21	response regulator receiver protein [Arthrobacter sp. 35/47]
gi823666448	5.04	1	1	357	36.4	5.50	2.21	O-sialoglycoprotein endopeptidase [Arthrobacter sp. YC-RL1]
gi403227640	5.63	1	1	231	23.8	4.89	2.21	putative oxidoreductase [Arthrobacter sp. Rue61a]
gi737812444	2.61	1	1	498	53.3	5.24	2.21	betaine-aldehyde dehydrogenase [Arthrobacter sp. H14]
gi916871988	9.47	1	1	190	20.7	6.39	2.21	hypothetical protein [Arthrobacter sp. H5]
gi930826377	3.23	1	1	465	51.0	6.48	2.21	hypothetical protein AOZ07_08870 [Arthrobacter arilaitensis]
gi742857924	6.04	1	1	265	27.7	4.65	2.21	alpha/beta hydrolase [Arthrobacter sp. W1]
gi928488539	2.53	1	1	553	58.2	5.41	2.21	hypothetical protein AOC05_01155 [Arthrobacter alpinus]
gi517598601	8.70	1	1	115	12.1	4.64	2.21	hypothetical protein [Arthrobacter sp. 162MFSha1.1]
gi651440629	6.27	1	1	255	27.8	5.47	2.21	phosphatidylinositol kinase [Arthrobacter sp. H14]
gi443481933	10.78	1	1	204	22.3	5.03	2.21	flavoprotein [Arthrobacter nitrophenolicus]
gi916872070	7.25	2	1	276	30.5	6.18	2.21	hypothetical protein [Arthrobacter sp. H5]
gi742758795	4.69	1	1	469	49.4	4.89	2.21	aldehyde dehydrogenase [Arthrobacter phenanthrenivorans]
gi476402608	2.08	1	1	577	61.4	5.30	2.21	preprotein translocase subunit SecD [Arthrobacter crystallopoietes BAB-32]
gi403227758	2.99	1	1	334	35.0	5.86	2.21	HTH-type transcriptional repressor PurR [Arthrobacter sp. Rue61a]
gi927031300	11.63	2	1	129	14.0	8.50	2.21	hypothetical protein AFL94_01935 [Arthrobacter sp. LS16]
gi651466046	5.57	2	1	359	38.4	5.26	2.21	mycothiol acetyltransferase [Arthrobacter sp. 35/47]
gi916820537	3.23	1	1	588	64.5	5.53	2.21	hypothetical protein [Arthrobacter sp. H20]
gi119951723	12.99	1	1	154	16.9	9.50	2.21	hypothetical protein AAur_pTC20009 (plasmid) [Arthrobacter aurescens TC1]
gi910747361	2.66	1	1	640	68.0	10.40	2.21	conserved hypothetical protein [Arthrobacter sp. Hiyo8]
gi651456356	6.35	1	1	299	32.7	6.46	2.21	glmZ(sRNA)-inactivating NTPase [Arthrobacter sp. 35/47]
gi928488809	4.52	1	1	332	36.6	8.72	2.21	hypothetical protein AOC05_10185 [Arthrobacter alpinus]
gi742070422	2.80	1	1	465	51.0	4.88	2.21	hypothetical protein ANMWB30_24630 [Arthrobacter sp. MWB30]
gi757623261	2.51	1	1	836	82.8	4.83	2.21	hypothetical protein TV39_16500 [Arthrobacter sp. SPG23]
gi654813541	4.62	1	1	346	38.6	7.93	2.21	AraC family transcriptional regulator [Arthrobacter sp. MA-N2]
gi737790474	14.63	1	1	123	13.1	11.49	2.21	50S ribosomal protein L18 [Arthrobacter albus]
gi654811709	5.66	1	1	212	23.2	6.70	2.21	DNA-binding response regulator [Arthrobacter sp. MA-N2]
gi648224433	8.76	1	1	137	15.6	6.68	2.21	hypothetical protein [Arthrobacter sp. M2012083]
gi908697361	3.04	1	1	657	68.2	5.19	2.21	ATPase [Arthrobacter sp. RIT-PI-e]
gi640203913	6.38	1	1	141	15.6	7.15	2.21	cation transport regulator ChaB [Arthrobacter sp. 31Y]
gi914713769	9.74	1	1	195	21.4	9.39	2.21	hypothetical protein [Arthrobacter sp. ZBG10]
gi674645775	3.55	1	1	366	40.2	5.03	2.21	Carboxylate-amine ligase YbdK [Arthrobacter sp. 11W110_air]
gi910251643	3.31	1	1	362	37.7	5.43	2.21	dehydrogenase [Arthrobacter siccitolerans]
gi636845901	3.20	1	1	532	56.1	5.10	2.21	AMP-dependent synthetase [Arthrobacter sp. TB 26]
gi737800392	5.11	1	1	274	29.2	5.97	2.21	glycosyl transferase family 8 [Arthrobacter castelli]
gi910739897	14.37	2	1	160	17.8	9.20	2.21	transcriptional repressor SdpR [Arthrobacter sp. Hiyo4]
gi651499884	6.63	2	1	377	40.1	5.08	2.21	oxidoreductase [Arthrobacter sp. 35W]
gi517608605	4.45	1	1	292	31.1	6.28	2.21	silent information regulator protein Sir2 [Arthrobacter sp. 161MFSha2.1]
gi742758091	6.94	1	1	144	15.7	5.99	2.21	MarR family transcriptional regulator [Arthrobacter phenanthrenivorans]
gi937258129	4.91	1	1	387	41.1	5.02	2.21	acyl-CoA dehydrogenase [Arthrobacter sp. Edens01]
gi359307688	2.95	1	1	543	59.9	5.11	2.21	putative oxidoreductase [Arthrobacter globiformis NBRC 12137]

gi742069216	6.83	1	1	278	29.9	4.77	2.21	FG-GAP repeat-containing protein [Arthrobacter sp. MWB30]
gi323470491	2.13	1	1	422	46.5	6.35	2.21	3-deoxy-D-manno-octulosonate 8-phosphate phosphatase, YrbI family [Arthrobacter phenan
gi723609653	4.72	1	1	318	34.1	5.80	2.21	hypothetical protein ART_3430 [Arthrobacter sp. PAMC25486]
gi542108100	2.86	1	1	315	32.4	5.99	2.20	ribokinase [Arthrobacter sp. AK-YN10]
gi443483064	7.20	1	1	236	26.1	9.74	2.20	undecaprenylphosphate glycosylphosphotransferase [Arthrobacter nitrophenolicus]
gi737814265	11.01	1	1	227	23.9	5.59	2.20	hypothetical protein [Arthrobacter sp. H14]
gi307745463	3.51	1	1	399	43.3	6.09	2.20	putative acyl-CoA dehydrogenase [Arthrobacter arilaitensis Re117]
gi116610834	2.42	1	1	454	51.2	6.65	2.20	protein of unknown function DUF245 domain protein [Arthrobacter sp. FB24]
gi927034153	10.43	1	1	163	17.4	9.85	2.20	hypothetical protein AFL94_13590 [Arthrobacter sp. LS16]
gi823668224	6.59	1	1	258	27.0	6.40	2.20	3-hydroxy-2-methylbutyryl-CoA dehydrogenase [Arthrobacter sp. YC-RL1]
gi928986016	9.87	1	1	152	16.8	10.24	2.20	hypothetical protein [Arthrobacter sp. ERGS1:01]
gi640195238	20.00	1	1	125	13.8	5.06	2.20	protein translocase TatA [Arthrobacter sp. 31Y]
gi654814801	6.00	1	1	300	32.6	10.10	2.20	secretion system protein [Arthrobacter sp. MA-N2]
gi443481368	8.85	2	1	192	20.7	4.46	2.20	mannose-1-phosphate guanylyltransferase [Arthrobacter nitrophenolicus]
gi517591624	4.78	1	1	230	24.8	4.81	2.20	GCN5 family acetyltransferase [Arthrobacter sp. 135MFCol5.1]
gi116612952	5.58	1	1	251	25.6	5.63	2.20	hypothetical protein Arth_4505 (plasmid) [Arthrobacter sp. FB24]
gi918265205	7.77	1	1	103	11.5	6.89	2.20	protein SamA [Arthrobacter sp. Hiyo1]
gi723607361	3.13	1	1	256	26.9	5.25	2.20	dehydrogenase [Arthrobacter sp. PAMC25486]
gi651458035	5.58	1	1	251	26.3	6.55	2.20	16S rRNA methyltransferase [Arthrobacter sp. 35/47]
gi910283564	2.72	1	1	368	37.8	6.39	2.20	alcohol dehydrogenase [Arthrobacter sp. A3]
gi917013203	3.96	1	1	404	44.5	5.11	2.20	pyruvate dehydrogenase [Arthrobacter sanguinis]
gi654825945	7.03	1	1	313	33.0	11.46	2.20	cytochrome oxidase assembly protein [Arthrobacter sp. H5]
gi654826735	4.01	1	1	274	29.4	7.03	2.20	hypothetical protein [Arthrobacter sp. H5]
gi517591457	6.17	1	1	227	25.2	6.81	2.20	DNA-binding response regulator [Arthrobacter sp. 135MFCol5.1]
gi930825097	3.75	1	1	320	35.1	9.16	2.20	hypothetical protein AOZ07_01475 [Arthrobacter arilaitensis]
gi918267700	7.54	1	1	199	21.3	7.88	2.20	mercuric reductase [Arthrobacter sp. Hiyo1]
gi517604605	3.69	1	1	434	46.5	6.05	2.20	cation transporter [Arthrobacter sp. 131MFCol6.1]
gi742755146	8.40	1	1	119	12.8	4.88	2.20	molecular chaperone DnaK [Arthrobacter phenanthrenivorans]
gi219858171	9.18	1	1	196	21.0	5.33	2.20	aceytltranferase [Arthrobacter chlorophenolicus A6]
gi651490999	10.75	1	1	186	20.4	4.98	2.20	oligoribonuclease [Arthrobacter sp. H20]
gi219862150	5.03	1	1	298	33.5	8.35	2.20	hypothetical protein Achl_4539 (plasmid) [Arthrobacter chlorophenolicus A6]
gi753931631	3.91	1	1	537	56.0	5.76	2.20	metalloendopeptidase [Arthrobacter arilaitensis]
gi162953150	5.92	1	1	321	34.3	4.73	2.20	hydrolase, alpha/beta fold superfamily [Renibacterium salmoninarum ATCC 33209]
gi651506146	6.90	1	1	232	24.6	6.70	2.20	ABC transporter ATP-binding protein [Arthrobacter sp. 35W]
gi515765708	5.14	1	1	253	28.1	6.81	2.20	hypothetical protein [Arthrobacter sp. M2012083]
gi470217478	8.02	1	1	237	25.6	11.53	2.20	hypothetical protein ADIAG_02311 [Arthrobacter gangotriensis Lz1y]
gi119948580	4.86	1	1	185	21.5	7.68	2.18	conserved hypothetical protein [Arthrobacter aurescens TC1]
gi517598319	2.02	1	1	544	58.3	6.24	2.17	peptide ABC transporter substrate-binding protein [Arthrobacter sp. 162MFSha1.1]
gi759709270	1.85	2	1	596	66.3	6.49	2.16	hypothetical protein [Arthrobacter sp. 9MFCol3.1]
gi518312557	4.48	1	1	268	30.2	8.81	2.13	UDP pyrophosphate synthase [Arthrobacter sp. TB 23]
gi823667078	2.71	1	1	295	31.2	5.62	2.12	hypothetical protein AA310_15485 [Arthrobacter sp. YC-RL1]
gi939050942	6.19	1	1	210	23.0	5.44	2.11	hypothetical protein [Arthrobacter sp. JCM 19049]
gi723606672	2.55	1	1	314	33.4	6.62	2.10	putative transcriptional regulator, LysR family [Arthrobacter sp. PAMC25486]
gi823667933	1.11	1	1	812	87.0	5.95	2.10	hypothetical protein AA310_04775 [Arthrobacter sp. YC-RL1]
gi765005967	3.75	1	1	267	28.5	8.21	2.10	ArsR family transcriptional regulator [Arthrobacter sp. A3]
gi723608174	4.00	1	1	350	35.1	5.44	2.09	hypothetical protein ART_1951 [Arthrobacter sp. PAMC25486]
gi658509243	6.43	1	1	171	19.4	8.88	2.08	ATPase AAA [Arthrobacter sp. TB 26]
gi674646365	2.29	1	1	262	27.8	10.78	2.08	Putative aliphatic sulfonates transport permease protein SsuC [Arthrobacter sp. 11W110_air]
gi517600445	2.00	1	1	300	32.2	6.02	2.08	hypothetical protein [Arthrobacter sp. 162MFSha1.1]
gi918266255	4.91	1	1	163	16.9	10.33	2.07	hypothetical protein AHiyo1_41410 [Arthrobacter sp. Hiyo1]
gi737798106	4.00	1	1	150	16.5	11.94	2.05	hypothetical protein [Arthrobacter sp. H20]
gi910248822	6.87	1	1	131	13.5	4.53	2.05	hypothetical protein [Arthrobacter siccitolerans]
gi403228758	3.29	1	1	365	39.4	5.80	2.04	putative integral membrane protein [Arthrobacter sp. Rue61a]
gi937262330	5.56	1	1	126	13.8	5.50	2.04	chorismate mutase [Arthrobacter sp. Edens01]
gi654814119	2.01	1	1	348	38.2	4.93	2.02	agmatine deiminase [Arthrobacter sp. MA-N2]

gi759734770	4.86	1	1	247	25.6	5.80	2.02	glycerol transporter [Arthrobacter sp. L77]
gi759730944	3.96	1	1	328	35.5	4.96	2.02	hypothetical protein [Arthrobacter sp. L77]
gi116610519	2.18	1	1	505	53.8	5.26	2.01	Aldehyde dehydrogenase (NAD(+)) [Arthrobacter sp. FB24]
gi651440976	2.18	1	1	505	54.0	5.12	2.01	sorbosone dehydrogenase [Arthrobacter sp. 9MFCol3.1]
gi916692307	3.09	1	1	194	22.1	10.51	2.01	hypothetical protein [Arthrobacter castelli]
gi652422771	5.22	1	1	134	14.4	6.30	2.01	toxin [Arthrobacter castelli]
gi443482140	5.53	1	1	199	22.0	6.06	2.01	PadR family transcripitonal regulator [Arthrobacter nitrophenolicus]
gi651446876	1.04	1	1	672	71.1	5.10	2.00	hypothetical protein [Arthrobacter nicotinovorans]
gi928487976	4.02	1	1	199	21.4	5.19	2.00	hypothetical protein AOC05_14925 [Arthrobacter alpinus]
gi443482585	2.49	2	1	401	45.2	9.89	2.00	hypothetical protein G205_04491 [Arthrobacter nitrophenolicus]
gi517603035	5.44	1	1	239	26.1	5.86	1.99	hypothetical protein [Arthrobacter sp. 131MFCol6.1]
gi742757450	2.78	1	1	360	38.6	9.19	1.99	pyridine nucleotide-disulfide oxidoreductase [Arthrobacter phenanthrenivorans]
gi786031137	2.58	1	1	271	28.5	7.31	1.99	methyltransferase type 12 [Arthrobacter chlorophenolicus]
gi542110287	3.82	1	1	157	17.2	6.02	1.99	peroxiredoxin [Arthrobacter sp. AK-YN10]
gi740682089	1.84	1	1	326	35.2	5.88	1.98	ribose-phosphate pyrophosphokinase [Arthrobacter sp. PAMC25486]
gi759718364	6.96	1	1	158	17.1	6.54	1.98	GCN5 family acetyltransferase [Arthrobacter sp. FB24]
gi674646241	2.90	1	1	414	42.7	8.79	1.98	Putative conjugal transfer proteinc/MT3759 [Arthrobacter sp. 11W110_air]
gi910744877	1.80	1	1	557	62.1	5.60	1.98	1,4-alpha-glucan branching enzyme GlgB [Arthrobacter sp. Hiyo8]
gi908698446	6.94	1	1	173	18.9	5.25	1.98	NUDIX domain-containing protein [Arthrobacter sp. RIT-PI-e]
gi359307499	1.42	1	1	565	60.3	5.44	1.97	putative glycosidase [Arthrobacter globiformis NBRC 12137]
gi403228666	3.97	1	1	277	30.2	9.45	1.97	nodulation protein J [Arthrobacter sp. Rue61a]
gi765009820	3.70	1	1	243	27.0	5.24	1.96	hypothetical protein [Arthrobacter sp. A3]
gi910697222	14.47	1	1	76	8.7	8.72	1.96	hypothetical protein AHiyo6_05730 [Arthrobacter sp. Hiyo6]
gi759725917	2.16	1	1	416	44.2	10.29	1.96	MFS transporter [Arthrobacter sp. I3]
gi635349975	5.18	1	1	193	20.6	6.79	1.95	enoyl-CoA hydratase subunit II [Arthrobacter siccitolerans]
gi359306518	1.87	1	1	482	51.5	9.70	1.95	cell division protein RodA [Arthrobacter globiformis NBRC 12137]
gi916691169	1.41	1	1	498	53.3	6.21	1.95	PucR family transcriptional regulator [Arthrobacter castelli]
gi916691942	3.33	1	1	330	35.3	5.17	1.95	exopolyphosphatase [Arthrobacter castelli]
gi937256683	2.89	1	1	380	41.1	6.90	1.95	hypothetical protein AO716_16775 [Arthrobacter sp. Edens01]
gi927032021	12.35	2	1	81	9.4	6.13	1.95	hypothetical protein AFL94_06505 [Arthrobacter sp. LS16]
gi470221394	4.31	1	1	255	27.7	7.83	1.95	coenzyme A transferase [Arthrobacter gangotriensis Lz1y]
gi740683626	2.27	1	1	396	43.2	5.47	1.95	acyl-CoA dehydrogenase [Arthrobacter sp. PAMC25486]
gi918268389	5.28	1	1	265	28.7	6.57	1.94	uncharacterized 24.6 kDa protein in ccpA 3'region [Arthrobacter sp. Hiyo1]
gi654816436	1.62	1	1	555	61.0	5.17	1.94	choline oxidase [Arthrobacter sp. UNC362MFTsu5.1]
gi651443918	2.38	1	1	504	52.4	5.91	1.94	histidine kinase [Arthrobacter nicotinovorans]
gi786030108	1.74	2	1	576	60.7	4.94	1.94	exo-alpha-sialidase [Arthrobacter chlorophenolicus]
gi476402625	6.94	1	1	144	15.7	8.28	1.94	hypothetical protein D477_002643 [Arthrobacter crystallopoietes BAB-32]
gi767258068	4.07	1	1	221	23.9	4.58	1.93	NUDIX hydrolase [Arthrobacter sp. IHBB 11108]
gi517590532	2.59	1	1	309	32.3	5.72	1.93	4-amino-4-deoxychorismate lyase [Arthrobacter sp. 135MFCol5.1]
gi917760256	7.77	1	1	103	10.7	11.65	1.93	hypothetical protein [Arthrobacter sp. L77]
gi116609269	2.99	1	1	301	32.7	9.39	1.93	conserved hypothetical protein [Arthrobacter sp. FB24]
gi927294307	3.00	1	1	233	25.9	6.02	1.93	phosphatidylinositol kinase [Arthrobacter sp. ERGS1:01]
gi759724208	2.55	1	1	432	45.8	9.29	1.93	membrane protein [Arthrobacter sp. I3]
gi753941382	10.53	1	1	76	8.2	4.97	1.92	hypothetical protein [Arthrobacter phenanthrenivorans]
gi916324777	2.19	1	1	319	33.3	8.81	1.92	arsenic resistance protein [Arthrobacter gangotriensis]
gi648575025	4.37	1	1	252	26.7	5.36	1.92	GntR family transcriptional regulator [Arthrobacter sp. 131MFCol6.1]
gi654816539	4.05	1	1	148	16.2	5.31	1.92	hypothetical protein [Arthrobacter sp. UNC362MFTsu5.1]
gi937261731	3.17	1	1	315	33.3	9.17	1.92	ABC transporter permease [Arthrobacter sp. Edens01]
gi908699387	6.71	1	1	149	15.8	6.19	1.92	hypothetical protein [Arthrobacter sp. RIT-PI-e]
gi737813229	2.03	1	1	492	52.3	5.39	1.92	gamma-aminobutyraldehyde dehydrogenase [Arthrobacter sp. H14]
gi742855502	2.96	1	1	338	35.4	4.91	1.91	hypothetical protein [Arthrobacter sp. W1]
gi651461364	4.91	1	1	163	18.0	5.11	1.91	GNAT family N-acetyltransferase [Arthrobacter sp. 35/47]
gi470220611	1.64	1	1	428	44.2	8.34	1.91	putative permease [Arthrobacter gangotriensis Lz1y]
gi916870208	2.86	1	1	315	32.7	5.52	1.91	nicotinate-nucleotide pyrophosphorylase [Arthrobacter sp. Br18]
gi737809920	2.70	1	1	259	27.5	5.07	1.91	SAM-dependent methyltransferase [Arthrobacter sp. 35/47]

gi910742334	8.87	1	1	124	13.8	9.92	1.91	protein UmuC [Arthrobacter sp. Hiyo8]
gi651448532	3.07	1	1	358	35.5	9.72	1.91	hypothetical protein [Arthrobacter nicotinovorans]
gi674646997	2.91	1	1	275	29.7	6.57	1.91	Protein FdhD [Arthrobacter sp. 11W110_air]
gi476400946	3.37	1	1	267	29.5	9.96	1.90	RDD domain-containing protein [Arthrobacter crystallopoietes BAB-32]
gi517592204	3.26	1	1	215	23.8	5.30	1.90	hypothetical protein [Arthrobacter sp. 135MFCol5.1]
gi823668167	1.42	1	1	776	78.7	4.61	1.90	ABC transporter [Arthrobacter sp. YC-RL1]
gi917760458	3.41	1	1	323	32.9	5.36	1.90	4-diphosphocytidyl-2C-methyl-D-erythritol kinase [Arthrobacter sp. L77]
gi930825495	4.76	1	1	126	14.0	9.41	1.90	hypothetical protein AOZ07_03745 [Arthrobacter arilaitensis]
gi908698611	1.65	1	1	546	58.7	5.44	1.90	arginine--tRNA ligase [Arthrobacter sp. RIT-PI-e]
gi652423041	8.40	1	1	119	13.1	10.71	1.90	hypothetical protein [Arthrobacter castelli]
gi359303569	2.95	1	1	271	28.5	5.91	1.90	putative IclR family transcriptional regulator [Arthrobacter globiformis NBRC 12137]
gi910739579	2.82	1	1	355	38.7	6.04	1.90	aminoacylase-1 [Arthrobacter sp. Hiyo4]
