# Supplementary material for: Effects of two kinds of imidazolium-based ionic liquids on the characteristics of steroid-transformation Arthrobacter simplex
Source: Microb Cell Fact. 2016 Jul 1;15:118. doi: 10.1186/s12934-016-0518-3 (PMC4930596; doi:10.1186/s12934-016-0518-3)
Supplement: Supplementary file 2 — 10.1186/s12934-016-0518-3 The identification of the [PrMIm]BF4-treated cells proteins. [file 12934_2016_518_MOESM2_ESM.pdf]

Additional file 2: The identification of the [PrMIm]-BF_4 -treated cells proteins

Accession	Coverage	PSMs	Peptides	AAs	MW [kDa]	calc. pI	Score	Description
gi672940878	73.11	218	27	543	56.5	4.86	680.51	chaperonin GroEL [Pimelobacter simplex]
gi672940874	46.46	114	19	622	65.8	4.68	346.39	molecular chaperone DnaK [Pimelobacter simplex]
gi672940880	29.62	58	15	736	79.9	4.86	128.57	catalase/peroxidase KatG [Pimelobacter simplex]
gi116610479	6.06	52	1	264	27.9	4.96	120.38	short-chain dehydrogenase/reductase SDR [Arthrobacter sp. FB24]
gi749402670	2.90	60	1	552	57.1	5.72	94.39	acetolactate synthase [Arthrobacter sp. AK-YN10]
gi737783399	3.41	43	1	410	44.0	5.21	87.98	N-isopropylammelide isopropylaminohydrolase [Arthrobacter sp. 35W]
gi1906824	21.00	34	5	462	50.0	11.56	87.12	hypothetical protein [Pimelobacter simplex]
gi219861633	25.37	65	1	67	7.2	4.88	84.58	cold-shock DNA-binding domain protein (plasmid) [Arthrobacter chlorophenolicus A6]
gi443482191	25.37	40	1	67	7.2	4.94	83.69	cold-shock DNA-binding protein family protein [Arthrobacter nitrophenolicus]
gi742859324	7.49	123	1	307	33.6	7.06	81.13	LysR family transcriptional regulator [Arthrobacter sp. W1]
gi928488106	3.97	17	1	277	30.0	5.72	76.04	flagellar biosynthesis protein FliA [Arthrobacter alpinus]
gi651431914	2.35	19	1	597	64.4	4.67	74.51	ABC transporter substrate-binding protein [Arthrobacter sanguinis]
gi939050621	30.40	21	4	125	13.0	4.48	69.29	50S ribosomal protein L7 [Arthrobacter sp. JCM 19049]
gi910737718	3.79	29	1	422	42.8	9.13	66.88	putative ankyrin-containing lipoprotein Lxx09580 [Arthrobacter sp. Hiyo4]
gi476402309	23.47	22	1	98	10.4	5.01	63.79	co-chaperonin GroES [Arthrobacter crystallopoietes BAB-32]
gi517602201	16.54	49	1	127	13.7	4.59	62.75	MULTISPECIES: glyoxalase [Arthrobacter]
gi939050482	4.45	21	1	292	32.3	4.64	62.28	hypothetical protein [Arthrobacter sp. JCM 19049]
gi119949505	4.39	33	1	319	35.2	7.21	60.42	putative transcriptional regulator, LysR family [Arthrobacter aurescens TC1]
gi517593463	7.49	10	2	387	40.8	4.78	56.03	mannitol-1-phosphate 5-dehydrogenase [Arthrobacter sp. 135MFCol5.1]
gi518311313	11.33	30	2	309	34.3	9.73	55.76	hypothetical protein [Arthrobacter sp. TB 23]
gi937259457	12.66	12	6	545	56.9	4.86	52.76	molecular chaperone GroEL [Arthrobacter sp. Edens01]
gi914716454	13.84	12	6	542	56.7	4.87	52.66	molecular chaperone GroEL [Arthrobacter sp. ZBG10]
gi910747355	3.63	12	4	1185	131.5	6.99	50.43	DNA-directed RNA polymerase subunit beta' [Arthrobacter sp. Hiyo8]
gi651495418	4.21	33	1	309	34.2	10.10	49.61	pseudouridine synthase [Arthrobacter sp. H20]
gi910743568	4.01	21	1	324	34.9	4.74	48.20	DNA repair protein RecN [Arthrobacter sp. Hiyo8]
gi674646068	12.77	20	2	235	24.7	9.38	47.20	50S ribosomal protein L1 [Arthrobacter sp. 11W110_air]
gi737800623	5.67	16	1	300	32.6	5.43	46.23	SPFH/Band 7/PHB domain protein [Arthrobacter castelli]
gi651461023	11.03	10	5	544	56.8	4.86	42.80	molecular chaperone GroEL [Arthrobacter sp. 35/47]
gi517601515	3.99	10	1	526	55.1	6.57	41.82	histidine kinase [Arthrobacter sp. 162MFSha1.1]
gi307745606	5.17	11	5	1296	142.7	6.89	41.59	DNA-directed RNA polymerase beta prime chain [Arthrobacter arilaitensis Re117]
gi651495309	8.33	10	1	264	28.8	7.55	41.43	glycerophosphodiester phosphodiesterase [Arthrobacter sp. H20]
gi910748533	11.59	26	4	233	25.7	5.12	40.27	30S ribosomal protein S1 [Arthrobacter sp. Hiyo8]
gi910739216	5.88	18	1	221	25.4	5.77	38.00	conserved hypothetical protein [Arthrobacter sp. Hiyo4]
gi742757319	3.99	3	1	526	54.9	6.29	37.78	histidine kinase [Arthrobacter phenanthrenivorans]
gi910747080	15.38	26	1	104	10.8	11.18	37.24	hypothetical protein AHiyo8_47820 [Arthrobacter sp. Hiyo8]
gi916835164	3.47	23	1	404	44.3	5.88	37.19	ABC transporter [Arthrobacter sp. H14]
gi511534741	2.55	5	1	470	51.1	6.47	37.05	transcriptional activator pmfR (plasmid) [Arthrobacter nicotinovorans]
gi651440580	2.47	3	1	486	54.6	5.44	36.83	hypothetical protein [Arthrobacter sp. H14]
gi674645160	6.39	18	1	360	37.6	5.14	36.80	putative enoyl-CoA hydratase [Arthrobacter sp. 11W110_air]
gi307745781	11.11	8	1	162	18.3	9.07	35.94	putative MarR-family transcriptional regulator [Arthrobacter arilaitensis Re117]
gi918221850	2.42	12	1	619	69.0	7.36	35.84	glycosyltransferase [Arthrobacter sp. I3]
gi651429323	12.58	10	1	159	17.8	8.06	35.71	hypothetical protein [Arthrobacter sanguinis]
gi639129536	8.86	29	2	237	24.9	5.58	35.56	copper oxidase [Arthrobacter sp. CAL618]
gi757623099	2.86	15	1	595	63.1	6.49	35.01	HNH endonuclease [Arthrobacter sp. SPG23]
gi916813687	4.53	28	1	309	34.3	6.28	34.52	hypothetical protein [Arthrobacter nicotinovorans]
gi919218844	1.25	1	1	1116	125.2	5.47	33.96	hypothetical protein [Arthrobacter sp. YC-RL1]
gi403228123	4.14	20	1	338	36.2	5.59	33.74	glycine betaine/carnitine/choline transport ATP-binding protein OpuCA [Arthrobacter sp. Rue61a]
gi927031692	6.27	29	1	271	30.1	7.06	33.59	30S ribosomal protein S2 [Arthrobacter sp. LS16]
gi742752690	4.01	8	1	424	45.3	6.54	32.83	dehydrogenase [Arthrobacter phenanthrenivorans]
gi651457048	14.93	14	1	134	15.0	9.06	30.98	30S ribosomal protein S16 [Arthrobacter sp. 35/47]
gi759736849	27.13	3	2	129	14.6	11.37	29.93	50S ribosomal protein L20 [Arthrobacter sp. L77]
gi737809001	23.81	3	2	147	16.6	10.90	29.67	50S ribosomal protein L20 [Arthrobacter sp. H5]
gi674645749	1.14	3	1	1317	144.9	6.98	29.61	ATP-dependent RNA helicase HrpB [Arthrobacter sp. 11W110_air]
gi652425065	21.31	2	2	122	13.4	10.40	29.03	50S ribosomal protein L14 [Arthrobacter castelli]
gi740685683	5.37	19	1	428	46.4	6.06	28.85	ATP-binding protein [Arthrobacter sp. PAMC25486]
gi189041365	18.85	6	2	122	13.3	10.15	28.52	RecName: Full=50S ribosomal protein L14
gi651455857	6.67	19	5	930	100.3	4.81	28.28	aconitate hydratase [Arthrobacter sp. 35/47]
gi737786847	12.16	7	4	485	52.6	4.91	28.24	ATP synthase subunit beta [Arthrobacter albus]

gi476402832	23.03	4	2	152	17.1	11.62	27.91	50S ribosomal protein L20 [Arthrobacter crystallopoietes BAB-32]
gi636845165	9.56	7	2	293	31.7	5.15	27.28	aminoglycoside resistance protein, partial [Arthrobacter sp. TB 26]
gi116609762	7.03	13	4	740	79.9	5.05	27.15	isocitrate dehydrogenase, NADP-dependent [Arthrobacter sp. FB24]
gi651490707	12.73	7	4	534	56.5	4.92	27.05	molecular chaperone GroEL [Arthrobacter sp. H20]
gi403230217	12.22	7	4	483	52.3	4.98	26.88	ATP synthase subunit beta [Arthrobacter sp. Rue61a]
gi651465980	14.04	7	3	235	25.0	9.36	26.87	50S ribosomal protein L1 [Arthrobacter sp. 35/47]
gi917739896	3.50	14	1	400	42.9	4.83	26.56	ABC transporter [Arthrobacter sp. W1]
gi651440931	1.47	4	1	1088	117.4	5.57	26.06	hypothetical protein [Arthrobacter sp. 9MFCol3.1]
gi654823558	5.34	20	1	262	27.1	9.85	26.03	oxidoreductase [Arthrobacter sp. I3]
gi651437930	8.94	9	1	235	24.9	9.26	25.59	50S ribosomal protein L1 [Arthrobacter sp. H14]
gi937259012	1.38	7	1	1158	123.6	5.27	25.32	1-pyrroline-5-carboxylate dehydrogenase [Arthrobacter sp. Edens01]
gi654813160	5.81	6	2	602	63.3	5.03	25.23	acetyl-CoA carboxylase [Arthrobacter sp. MA-N2]
gi767256780	0.41	1	1	3915	423.6	4.87	24.75	hypothetical protein UM93_02395 [Arthrobacter sp. IHBB 11108]
gi927296035	15.21	2	1	217	22.7	5.60	24.66	LuxR family transcriptional regulator [Arthrobacter sp. ERGS1:01]
gi918449287	2.10	2	1	905	99.8	5.67	24.57	hypothetical protein [Arthrobacter sp. SPG23]
gi654826196	6.17	1	1	519	54.0	5.12	24.42	UDP-N-acetylmuramoylalanine--D-glutamate ligase [Arthrobacter sp. H5]
gi470220234	14.34	20	3	279	30.6	11.33	23.92	50S ribosomal protein L2 [Arthrobacter gangotriensis Lz1y]
gi737815326	3.99	9	2	827	91.4	5.55	23.75	NDP-hexose 4-ketoreductase [Arthrobacter sp. H14]
gi551254729	12.65	3	1	166	18.4	5.81	23.66	hypothetical protein [Arthrobacter sp. PAO19]
gi323469187	4.78	9	1	230	24.5	5.27	23.25	conserved hypothetical protein TIGR00370 [Arthrobacter phenanthrenivorans Sphe3]
gi652425290	8.97	8	1	145	16.2	6.00	23.23	MarR family transcriptional regulator [Arthrobacter castelli]
gi654828077	9.33	7	4	536	56.6	4.98	23.15	molecular chaperone GroEL [Arthrobacter sp. H5]
gi470220257	12.54	8	3	335	36.1	4.78	23.05	DNA-directed RNA polymerase subunit alpha [Arthrobacter gangotriensis Lz1y]
gi651499303	3.50	1	1	657	70.5	6.37	22.90	1-deoxy-D-xylulose-5-phosphate synthase [Arthrobacter sp. 35W]
gi639129639	5.44	6	1	331	33.7	5.25	22.90	thiamine-monophosphate kinase [Arthrobacter sp. CAL618]
gi910738525	3.01	7	1	499	53.1	7.65	22.88	probable NADH dehydrogenase [Arthrobacter sp. Hiyo4]
gi443479714	5.15	2	1	330	35.0	5.33	22.67	oxidoreductase, aryl-alcohol dehydrogenase like protein [Arthrobacter nitrophenolicus]
gi930824867	9.09	5	2	484	52.2	5.08	22.46	peptidoglycan glycosyltransferase [Arthrobacter arilaitensis]
gi910697512	11.82	7	4	406	44.4	6.38	22.34	ATP synthase subunit alpha [Arthrobacter sp. Hiyo6]
gi654814374	5.24	4	1	439	48.1	5.31	22.31	ATPase AAA [Arthrobacter sp. MA-N2]
gi517598428	5.79	3	1	328	34.8	5.21	21.73	O-succinylbenzoate synthase [Arthrobacter sp. 162MFSha1.1]
gi928489026	20.65	3	1	92	10.2	5.06	21.40	hypothetical protein AOC05_17360 [Arthrobacter alpinus]
gi737813326	3.47	7	1	432	47.5	4.65	20.99	HCC family HlyC/CorC transporter [Arthrobacter sp. H14]
gi759730994	3.34	4	1	539	59.5	5.57	20.92	choline oxidase [Arthrobacter sp. L77]
gi654827760	9.01	3	1	233	25.8	6.21	20.90	hypothetical protein [Arthrobacter sp. H5]
gi651431177	18.85	4	2	122	13.4	10.32	20.89	50S ribosomal protein L14 [Arthrobacter sanguinis]
gi551253914	20.09	7	3	234	24.8	9.29	20.69	50S ribosomal protein L1 [Arthrobacter sp. PAO19]
gi910283858	1.72	4	1	816	86.4	7.65	20.68	transcription accessory protein [Arthrobacter sp. A3]
gi476402260	3.05	11	1	492	52.0	5.53	20.22	ADP-ribosylation/crystallin J1 [Arthrobacter crystallopoietes BAB-32]
gi742758238	14.48	10	1	145	15.6	5.00	20.17	hypothetical protein RM50_04295 [Arthrobacter phenanthrenivorans]
gi470220608	8.92	3	2	437	45.8	5.27	20.14	pyrimidine-nucleoside phosphorylase [Arthrobacter gangotriensis Lz1y]
gi737790428	6.30	4	2	397	43.8	5.02	20.03	elongation factor Tu [Arthrobacter albus]
gi767258484	5.78	10	3	830	91.6	5.55	19.85	NDP-hexose 4-ketoreductase [Arthrobacter sp. IHBB 11108]
gi723607705	3.11	2	1	418	46.6	5.38	19.82	creatine amidinohydrolase [Arthrobacter sp. PAMC25486]
gi518312892	25.37	9	1	67	7.2	4.88	19.73	MULTISPECIES: cold-shock protein [Arthrobacter]
gi651493900	3.35	7	2	865	93.0	5.16	19.41	ATPase AAA [Arthrobacter sp. H20]
gi918469314	2.81	5	1	676	74.6	6.44	19.34	glycogen debranching protein [Arthrobacter crystallopoietes]
gi359303839	1.57	1	1	1020	108.1	5.11	19.23	nuclease SbcCD subunit C [Arthrobacter globiformis NBRC 12137]
gi542106946	3.52	6	2	739	79.6	5.03	19.07	isocitrate dehydrogenase [Arthrobacter sp. AK-YN10]
gi916782233	5.34	8	1	337	36.4	5.95	19.04	hypothetical protein [Arthrobacter sp. 35W]
gi651434056	12.66	5	4	537	56.5	4.92	19.03	molecular chaperone GroEL [Arthrobacter sp. H41]
gi517603447	4.26	5	1	399	40.6	5.97	18.89	acetyl-CoA acetyltransferase [Arthrobacter sp. 131MFCol6.1]
gi654814371	4.22	6	1	474	50.2	6.06	18.85	aspartate ammonia-lyase [Arthrobacter sp. MA-N2]
gi162955135	9.21	6	1	228	23.9	9.29	18.79	hypothetical protein RSal33209_2928 [Renibacterium salmoninarum ATCC 33209]
gi928487494	2.36	6	1	594	63.2	5.47	18.66	hypothetical protein AOC05_11905 [Arthrobacter alpinus]
gi816850540	22.82	11	3	206	23.3	5.45	18.45	superoxide dismutase [Pimelobacter simplex]
gi742855609	5.38	3	2	520	55.5	9.04	18.41	signal recognition particle protein [Arthrobacter sp. W1]
gi927294035	1.58	2	1	1013	109.7	6.34	18.38	glutamine-synthetase [Arthrobacter sp. ERGS1:01]
gi517605103	3.11	11	1	482	50.5	8.50	18.15	phytoene dehydrogenase [Arthrobacter sp. 131MFCol6.1]

gi651504005	5.00	5	1	300	32.5	5.12	18.13	UTP--glucose-1-phosphate uridylyltransferase [Arthrobacter sp. 35W]
gi759709557	4.32	2	1	486	52.7	5.63	17.87	FAD-dependent oxidoreductase [Arthrobacter sp. 9MFCol3.1]
gi674644816	4.75	4	3	737	78.9	4.93	17.81	Polyribonucleotide nucleotidyltransferase [Arthrobacter sp. 11W110_air]
gi928487058	9.45	3	1	127	14.2	6.54	17.81	MerR family transcriptional regulator [Arthrobacter alpinus]
gi651504241	4.44	6	3	1172	128.8	4.96	17.74	DNA-directed RNA polymerase subunit beta [Arthrobacter sp. 35W]
gi757624358	9.31	9	3	462	51.0	5.34	17.52	magnesium chelatase [Arthrobacter sp. SPG23]
gi651440061	1.05	1	1	1622	183.1	5.01	17.51	glutamate dehydrogenase [Arthrobacter sp. H14]
gi651431596	8.53	6	1	129	13.3	4.54	17.49	50S ribosomal protein L7/L12 [Arthrobacter sanguinis]
gi823666113	3.26	2	1	430	46.8	5.16	17.49	ATP-dependent protease [Arthrobacter sp. YC-RL1]
gi914715369	1.56	2	1	1024	108.0	5.19	17.43	chromosome segregation protein SMC [Arthrobacter sp. ZBG10]
gi651467196	1.45	2	1	1313	146.0	7.14	17.42	ATP-dependent helicase [Arthrobacter sp. 35/47]
gi759728512	5.09	2	1	373	41.5	5.64	17.42	hypothetical protein [Arthrobacter sp. UNC362MFTsu5.1]
gi648573277	3.73	4	1	456	48.1	9.88	17.41	3-phenylpropionic acid transporter [Arthrobacter sp. 135MFCol5.1]
gi651503952	7.56	1	1	172	19.3	8.21	17.41	hypothetical protein [Arthrobacter sp. 35W]
gi443482459	2.17	4	1	692	74.5	6.95	17.38	acyltransferase [Arthrobacter nitrophenolicus]
gi359307391	1.22	5	1	1150	126.2	5.96	17.37	hypothetical protein ARGLB_015_00190 [Arthrobacter globiformis NBRC 12137]
gi765009289	4.48	12	1	201	20.8	9.70	17.24	hypothetical protein [Arthrobacter sp. A3]
gi654826881	4.26	3	1	399	42.5	7.05	17.18	pilus assembly protein CpaE [Arthrobacter sp. H5]
gi919219015	7.80	3	1	282	31.3	9.61	17.13	hypothetical protein [Arthrobacter sp. YC-RL1]
gi910695627	6.54	4	1	260	27.3	8.07	17.02	UPF0001 protein Cgl2153/cg2364 [Arthrobacter sp. Hiyo6]
gi651444228	0.68	1	1	1618	180.0	5.25	17.01	glutamate dehydrogenase [Arthrobacter nicotinovorans]
gi908690403	4.00	8	1	425	46.3	6.68	16.75	exodeoxyribonuclease VII large subunit [Arthrobacter sp. H41]
gi930827823	10.08	3	1	258	26.8	4.60	16.69	electron transfer flavoprotein subunit beta [Arthrobacter arilaitensis]
gi914717426	1.51	1	1	729	76.0	5.59	16.52	acetyl-CoA carboxylase [Arthrobacter sp. ZBG10]
gi651457246	6.83	4	2	498	55.2	5.34	16.51	glutamate--tRNA ligase [Arthrobacter sp. 35/47]
gi908690795	4.29	2	1	513	54.4	7.58	16.47	FAD-dependent oxidoreductase [Arthrobacter sp. H41]
gi162953844	0.55	1	1	1626	180.7	5.14	16.35	NAD-specific glutamate dehydrogenase [Renibacterium salmoninarum ATCC 33209]
gi640202524	3.22	7	2	962	102.1	5.68	16.31	cell division protein FtsK [Arthrobacter sp. 31Y]
gi760112652	6.30	10	1	254	26.3	10.45	16.31	ABC transporter [Arthrobacter chlorophenolicus]
gi765013208	3.47	2	2	1209	129.8	5.30	16.30	chromosome segregation protein SMC [Arthrobacter sp. A3]
gi759736570	2.79	2	1	501	52.3	5.22	16.26	glutamyl-tRNA amidotransferase [Arthrobacter sp. L77]
gi928486094	3.52	4	2	738	79.3	5.11	16.01	isocitrate dehydrogenase [Arthrobacter alpinus]
gi651505257	3.78	9	2	874	93.9	5.07	16.00	ATPase AAA [Arthrobacter sp. 35W]
gi517591228	2.69	1	1	892	95.9	5.31	15.94	alanine--tRNA ligase [Arthrobacter sp. 135MFCol5.1]
gi737790493	9.42	4	2	329	35.8	4.60	15.86	DNA-directed RNA polymerase subunit alpha [Arthrobacter albus]
gi654818882	4.78	5	1	335	36.5	5.72	15.81	aminoglycoside resistance protein [Arthrobacter sp. UNC362MFTsu5.1]
gi651444609	3.67	2	1	491	55.4	6.18	15.78	hypothetical protein [Arthrobacter nicotinovorans]
gi759733764	12.00	6	2	300	30.6	5.36	15.76	succinyl-CoA synthetase subunit alpha [Arthrobacter sp. L77]
gi443479756	2.79	2	1	359	38.6	9.64	15.71	LysR family transcriptional regulator [Arthrobacter nitrophenolicus]
gi651430724	2.11	1	1	949	98.8	6.25	15.47	translation initiation factor IF-2 [Arthrobacter sanguinis]
gi651435178	1.16	1	1	1384	149.2	6.80	15.46	hypothetical protein [Arthrobacter sp. H41]
gi636846879	5.22	4	1	345	38.0	7.42	15.37	lysophospholipase [Arthrobacter sp. TB 26]
gi740683298	3.16	2	1	507	54.3	7.49	15.32	LuxR family transcriptional regulator [Arthrobacter sp. PAMC25486]
gi518311459	3.41	12	1	440	45.7	4.87	15.31	MULTISPECIES: hypothetical protein [Arthrobacter]
gi916863318	3.16	2	1	475	51.5	7.93	15.24	malate:quinone oxidoreductase [Arthrobacter sp. 35/47]
gi916876272	0.47	1	1	2548	276.5	5.43	15.20	hypothetical protein [Arthrobacter sp. 31Y]
gi162954797	12.12	2	1	165	17.9	11.19	15.17	CPRD14 protein [Renibacterium salmoninarum ATCC 33209]
gi757623679	3.42	10	2	1022	108.4	5.15	15.03	chromosome segregation protein SMC [Arthrobacter sp. SPG23]
gi759730827	2.18	4	1	689	73.9	6.86	14.91	hypothetical protein [Arthrobacter sp. L77]
gi219859433	23.29	6	1	73	7.6	8.97	14.91	conserved hypothetical protein [Arthrobacter chlorophenolicus A6]
gi910739807	2.42	2	1	496	54.9	5.50	14.91	peptidyl-dipeptidase dcp [Arthrobacter sp. Hiyo4]
gi332743185	2.70	6	1	519	58.5	9.36	14.88	hypothetical protein STTU_0837 [Streptomyces sp. Tu6071]
gi737786822	0.65	1	1	1381	153.0	7.91	14.86	ATP-dependent helicase [Arthrobacter albus]
gi489899738	8.56	2	1	222	24.5	6.30	14.84	two-component system response regulator [Arthrobacter globiformis]
gi517591758	5.76	3	1	191	20.2	5.01	14.83	phosphoribosylglycinamide formyltransferase [Arthrobacter sp. 135MFCol5.1]
gi737790199	2.78	7	2	862	93.9	4.94	14.79	ATPase AAA [Arthrobacter albus]
gi737813222	9.70	2	1	165	18.1	9.86	14.77	hypothetical protein [Arthrobacter sp. H14]
gi119951337	3.82	3	2	628	68.2	5.00	14.75	putative tetratricopeptide repeat family protein [Arthrobacter aurescens TC1]
gi636845208	6.13	3	1	326	34.3	5.36	14.73	prephenate dehydratase [Arthrobacter sp. TB 26]

gi910283724	10.60	4	2	349	36.7	5.15	14.72	hypothetical protein [Arthrobacter sp. A3]
gi545107909	7.71	4	3	545	58.9	5.11	14.72	ATP synthase subunit alpha [Arthrobacter sp. AK-YN10]
gi928485861	3.20	2	1	656	69.5	7.94	14.49	ABC transporter [Arthrobacter alpinus]
gi651464991	3.01	3	1	599	65.0	5.35	14.42	proline--tRNA ligase [Arthrobacter sp. 35/47]
gi652424793	1.26	5	1	636	71.9	5.30	14.35	hypothetical protein [Arthrobacter castelli]
gi648573957	7.08	2	1	226	25.1	6.70	14.31	fructose 2,6-bisphosphatase [Arthrobacter sp. 162MFSha1.1]
gi551255613	3.46	1	1	665	71.0	5.38	14.28	ATP-dependent helicase [Arthrobacter sp. PAO19]
gi759715228	1.41	3	2	1847	202.0	5.21	14.24	hypothetical protein, partial [Arthrobacter sp. AK-YN10]
gi737789341	3.91	9	3	742	80.1	4.92	14.23	polynucleotide phosphorylase [Arthrobacter albus]
gi654819266	2.40	4	1	375	40.4	7.62	14.23	hypothetical protein [Arthrobacter sp. UNC362MFTsu5.1]
gi545108041	7.95	4	1	239	26.1	6.23	14.18	hypothetical protein [Arthrobacter sp. AK-YN10]
gi476399998	7.79	4	3	462	52.5	5.54	14.15	glycyl-tRNA ligase [Arthrobacter crystallopoietes BAB-32]
gi916813860	5.32	6	1	263	28.6	5.95	14.13	methyltransferase type 11 [Arthrobacter nicotinovorans]
gi162953613	0.69	4	1	1303	143.7	6.61	14.12	2-oxoglutarate dehydrogenase E1 component [Renibacterium salmoninarum ATCC 33209]
gi521093715	0.57	2	1	2270	239.9	4.74	14.09	hypothetical protein [Arthrobacter sp. TB 23]
gi937262500	6.12	5	2	654	70.8	6.73	14.08	acyl-CoA dehydrogenase [Arthrobacter sp. Edens01]
gi916710577	1.47	4	1	1156	125.0	5.86	14.05	TrwC relaxase [Arthrobacter sp. CAL618]
gi674644000	3.40	1	1	294	31.8	7.05	14.04	putative HTH-type transcriptional regulatorc/MT2039 [Arthrobacter sp. 11W110_air]
gi759711759	2.47	1	1	811	86.8	6.16	14.03	nitrite reductase large subunit [Arthrobacter sp. 162MFSha1.1]
gi476402255	1.56	1	1	1151	123.4	5.27	14.00	hypothetical protein D477_004446 [Arthrobacter crystallopoietes BAB-32]
gi742072721	3.60	1	1	333	35.9	6.47	13.98	LacI family transcriptional regulator [Arthrobacter sp. MWB30]
gi654818577	2.02	1	1	496	55.1	6.00	13.92	RNA nucleotidyltransferase [Arthrobacter sp. UNC362MFTsu5.1]
gi742070144	2.59	1	1	580	60.1	5.33	13.90	hypothetical protein ANMWB30_28290 [Arthrobacter sp. MWB30]
gi917739479	2.25	4	2	1602	178.3	5.26	13.90	glutamate dehydrogenase [Arthrobacter sp. W1]
gi219860985	9.61	9	1	229	23.7	7.46	13.87	short-chain dehydrogenase/reductase SDR [Arthrobacter chlorophenolicus A6]
gi542108507	6.77	2	2	443	46.4	6.95	13.77	zinc metalloprotease [Arthrobacter sp. AK-YN10]
gi759715093	2.95	1	1	508	55.6	6.34	13.76	isoniazid-inducible protein iniA, partial [Arthrobacter sp. AK-YN10]
gi928487111	3.54	1	1	311	33.9	9.82	13.72	recombinase XerC [Arthrobacter alpinus]
gi119949243	3.29	1	1	516	56.3	4.97	13.71	putative glycosyl hydrolases family 32 protein [Arthrobacter aurescens TC1]
gi918265604	8.73	6	2	275	29.6	5.02	13.60	chaperone protein DnaK [Arthrobacter sp. Hiyo1]
gi910746946	5.07	3	1	296	31.7	5.06	13.59	UTP--glucose-1-phosphate uridylyltransferase [Arthrobacter sp. Hiyo8]
gi916816200	7.98	2	1	263	27.2	6.40	13.54	hypothetical protein [Arthrobacter sp. MA-N2]
gi403231944	3.77	1	1	478	52.2	5.73	13.48	Xaa-Pro aminopeptidase (plasmid) [Arthrobacter sp. Rue61a]
gi476400347	1.57	1	1	572	61.8	6.13	13.45	succinate dehydrogenase flavoprotein subunit [Arthrobacter crystallopoietes BAB-32]
gi927294653	3.49	3	2	601	63.2	8.95	13.43	preprotein translocase subunit SecD [Arthrobacter sp. ERGS1:01]
gi470220703	7.96	4	1	201	22.3	9.54	13.42	sugar transferase [Arthrobacter gangotriensis Lz1y]
gi652423332	4.91	3	2	530	56.0	4.78	13.34	molecular chaperone GroEL [Arthrobacter castelli]
gi651459247	3.52	7	2	739	79.8	4.98	13.31	isocitrate dehydrogenase [Arthrobacter sp. 35/47]
gi651435994	8.02	3	1	262	28.1	10.95	13.30	phosphatidic acid phosphatase [Arthrobacter sp. H41]
gi476399710	3.36	8	2	446	50.3	5.95	13.28	hypothetical protein D477_017277 [Arthrobacter crystallopoietes BAB-32]
gi740683971	1.19	2	1	670	74.6	6.37	13.27	glycosyl transferase [Arthrobacter sp. PAMC25486]
gi930825471	5.95	3	1	269	28.2	4.84	13.26	alpha-dehydro-beta-deoxy-D-glucarate aldolase [Arthrobacter arilaitensis]
gi636843386	2.02	1	1	941	99.2	6.00	13.25	oxidoreductase [Arthrobacter sp. TB 26]
gi919218749	2.04	3	2	442	48.5	9.01	13.25	hypothetical protein [Arthrobacter sp. YC-RL1]
gi749402661	5.35	1	1	486	51.9	8.85	13.22	hypothetical protein M707_12880 [Arthrobacter sp. AK-YN10]
gi651479423	16.81	5	2	119	13.4	10.32	13.20	50S ribosomal protein L19 [Arthrobacter sp. Br18]
gi489901122	5.72	3	2	577	63.2	6.25	13.16	multidrug ABC transporter ATP-binding protein [Arthrobacter globiformis]
gi765009954	4.41	1	1	454	47.7	5.20	13.14	4-aminobutyrate aminotransferase [Arthrobacter sp. A3]
gi908698210	8.17	5	2	514	53.0	8.66	13.08	oxidoreductase [Arthrobacter sp. RIT-PI-e]
gi651431168	4.95	4	3	545	59.1	5.05	13.03	ATP synthase subunit alpha [Arthrobacter sanguinis]
gi737790715	5.30	7	1	396	40.3	5.27	12.96	acetyl-CoA acetyltransferase [Arthrobacter albus]
gi651500415	2.04	1	1	881	90.4	7.88	12.95	helicase [Arthrobacter sp. 35W]
gi917739807	1.41	2	1	995	111.0	5.62	12.93	restriction endonuclease subunit R [Arthrobacter sp. W1]
gi723608975	8.37	2	1	203	21.6	7.58	12.86	transposase, IS111A/IS1328/IS1533 [Arthrobacter sp. PAMC25486]
gi917739783	5.38	3	1	353	38.3	6.90	12.83	hypothetical protein [Arthrobacter sp. W1]
gi723607993	1.63	11	1	675	69.8	5.36	12.83	methylcrotonoyl-CoA carboxylase subunit alpha [Arthrobacter sp. PAMC25486]
gi927293886	0.84	1	1	956	101.1	5.62	12.79	cell division protein FtsK [Arthrobacter sp. ERGS1:01]
gi476400146	8.94	5	2	235	24.9	9.38	12.70	50S ribosomal protein L1 [Arthrobacter crystallopoietes BAB-32]
gi737787955	5.75	4	2	696	78.7	4.97	12.59	excinuclease ABC subunit B [Arthrobacter albus]

gi928488672	2.15	1	1	559	56.8	4.78	12.58	dihydrolipoamide acetyltransferase [Arthrobacter alpinus]
gi651479522	3.59	2	1	529	57.5	5.19	12.55	GTP-binding protein [Arthrobacter sp. Br18]
gi651457875	2.69	1	1	892	96.5	5.43	12.53	alanine--tRNA ligase [Arthrobacter sp. 35/47]
gi742754810	6.56	3	2	488	53.1	5.02	12.47	S-adenosyl-L-homocysteine hydrolase [Arthrobacter phenanthrenivorans]
gi470216707	1.18	6	1	764	83.8	5.34	12.37	UvrD/REP helicase [Arthrobacter gangotriensis Lz1y]
gi651484943	5.05	2	1	436	45.4	6.34	12.35	acetyl-CoA acetyltransferase [Arthrobacter sp. Br18]
gi910738049	6.75	1	1	385	40.4	5.27	12.34	probable sensor histidine kinase TcrY [Arthrobacter sp. Hiyo4]
gi517593335	6.59	3	2	410	44.3	9.35	12.30	DNA polymerase IV [Arthrobacter sp. 135MFCol5.1]
gi916782265	2.40	2	1	459	48.4	9.64	12.29	hypothetical protein [Arthrobacter sp. 35W]
gi759735310	2.87	2	1	592	64.6	6.57	12.28	hypothetical protein [Arthrobacter sp. L77]
gi759719843	1.48	4	1	1217	130.8	5.24	12.20	chromosome segregation protein SMC [Arthrobacter sp. FB24]
gi674646334	3.15	2	1	349	39.1	5.59	12.18	Glutathionyl-hydroquinone reductase YqjG [Arthrobacter sp. 11W110_air]
gi654816002	1.53	1	1	848	93.5	5.16	12.18	DEAD/DEAH box helicase [Arthrobacter sp. UNC362MFTsu5.1]
gi517592070	17.65	7	2	153	16.6	10.14	12.17	MarR family transcriptional regulator [Arthrobacter sp. 135MFCol5.1]
gi323471533	4.10	4	1	390	42.9	5.39	12.14	MoxR-like ATPase (plasmid) [Arthrobacter phenanthrenivorans Sphe3]
gi219862159	6.41	4	1	359	39.0	6.90	12.13	hypothetical protein Achl_4548 (plasmid) [Arthrobacter chlorophenolicus A6]
gi443482553	4.97	7	1	382	39.8	6.13	12.11	ROK family transcriptional regulator [Arthrobacter nitrophenolicus]
gi470217266	1.21	1	1	912	101.8	5.48	12.09	preprotein translocase subunit SecA [Arthrobacter gangotriensis Lz1y]
gi759731923	4.31	3	2	510	54.4	5.29	12.09	hypothetical protein [Arthrobacter sp. L77]
gi517604861	3.08	11	1	552	59.6	6.30	12.03	elongation factor 3 [Arthrobacter sp. 131MFCol6.1]
gi443482594	0.88	2	1	1358	142.4	6.98	12.02	DNA segregation ATPase FtsK [Arthrobacter nitrophenolicus]
gi742853148	0.90	5	1	1326	146.2	7.05	11.98	ATP-dependent helicase [Arthrobacter sp. W1]
gi818631433	6.83	3	1	278	29.9	10.36	11.91	putative cell-division protein (plasmid) [Arthrobacter sp. 68b]
gi927296152	8.54	2	2	328	35.1	7.44	11.91	hypothetical protein AL755_05945 [Arthrobacter sp. ERGS1:01]
gi651502464	1.64	2	1	1216	127.9	5.53	11.90	chromosome segregation protein SMC [Arthrobacter sp. 35W]
gi551256381	4.26	11	1	517	55.2	8.24	11.85	hypothetical protein [Arthrobacter sp. PAO19]
gi403231979	0.80	1	1	1120	123.1	5.94	11.84	hypothetical protein ARUE_232p01920 (plasmid) [Arthrobacter sp. Rue61a]
gi116612156	3.60	1	1	445	47.9	5.54	11.84	glutamate dehydrogenase (NADP) [Arthrobacter sp. FB24]
gi636845532	2.92	4	2	1028	115.5	5.36	11.80	hypothetical protein [Arthrobacter sp. TB 26]
gi737781476	1.21	2	1	663	74.0	6.32	11.77	glycosyl transferase [Arthrobacter sp. 35W]
gi927031409	7.33	7	1	232	25.8	5.19	11.77	dihydrofolate reductase [Arthrobacter sp. LS16]
gi930826087	3.04	3	1	559	61.1	5.43	11.74	hypothetical protein AOZ07_07160 [Arthrobacter arilaitensis]
gi823666269	4.55	1	1	572	58.4	4.64	11.73	dihydrolipoamide acetyltransferase [Arthrobacter sp. YC-RL1]
gi648573984	0.73	2	1	1235	136.3	6.38	11.72	nitrate reductase [Arthrobacter sp. 162MFSha1.1]
gi910739393	4.78	1	1	460	47.2	5.71	11.70	xylulose kinase [Arthrobacter sp. Hiyo4]
gi323467686	2.99	1	1	368	42.5	6.87	11.68	uncharacterized conserved protein [Arthrobacter phenanthrenivorans Sphe3]
gi359307531	2.52	11	2	953	103.7	5.72	11.68	hypothetical protein ARGLB_012_00140 [Arthrobacter globiformis NBRC 12137]
gi652422968	5.67	7	1	194	21.3	7.08	11.67	bifunctional pyrimidine regulatory protein PyrR uracil phosphoribosyltransferase [Arthrobacter cast
gi916324758	2.01	1	1	745	82.2	5.85	11.67	choline transporter [Arthrobacter gangotriensis]
gi635351885	0.97	6	1	821	89.1	5.69	11.62	hypothetical protein ARTSIC4J27_2201 [Arthrobacter siccitolerans]
gi551254533	7.33	3	1	232	25.7	4.98	11.61	DtxR family transcriptional regulator [Arthrobacter sp. PAO19]
gi116609666	4.25	1	1	400	45.2	9.70	11.60	phage integrase family protein [Arthrobacter sp. FB24]
gi654813352	7.36	11	2	557	62.0	5.07	11.53	hypothetical protein [Arthrobacter sp. MA-N2]
gi917760505	8.27	3	2	508	53.5	9.42	11.51	3-methyladenine DNA glycosylase [Arthrobacter sp. L77]
gi651436738	1.44	1	1	1113	119.2	4.91	11.50	carbamoyl phosphate synthase large subunit [Arthrobacter sp. H41]
gi928487106	3.53	1	1	312	31.4	5.17	11.50	ACP S-malonyltransferase [Arthrobacter alpinus]
gi723609944	3.31	6	1	363	36.4	5.19	11.49	hypothetical protein ART_3721 [Arthrobacter sp. PAMC25486]
gi919218901	1.11	1	1	1439	148.9	6.09	11.44	hypothetical protein [Arthrobacter sp. YC-RL1]
gi489894845	0.58	2	1	1726	190.7	5.85	11.39	ATP-dependent helicase [Arthrobacter globiformis]
gi908696808	3.61	2	1	527	56.0	5.49	11.38	GTPase CgtA [Arthrobacter sp. RIT-PI-e]
gi654823461	5.51	3	2	363	40.1	8.78	11.38	mannosyltransferase [Arthrobacter sp. I3]
gi908697046	1.64	4	1	609	65.1	5.71	11.37	hypothetical protein [Arthrobacter sp. RIT-PI-e]
gi737787422	4.71	6	1	297	31.5	5.22	11.35	pyridoxal biosynthesis lyase PdxS [Arthrobacter albus]
gi917013195	12.10	1	1	157	17.0	10.30	11.31	hypothetical protein [Arthrobacter sanguinis]
gi470221631	3.14	1	1	350	38.3	5.38	11.29	hypothetical protein ADIAG_00484 [Arthrobacter gangotriensis Lz1y]
gi910251443	4.36	1	1	527	58.5	5.91	11.28	alkaline phosphatase [Arthrobacter siccitolerans]
gi910738356	3.99	2	1	426	46.8	9.60	11.28	uncharacterized oxidoreductase y4hM [Arthrobacter sp. Hiyo4]
gi654827857	3.87	5	1	388	40.2	6.80	11.25	ROK family transcriptional regulator [Arthrobacter sp. H5]
gi928487898	4.03	3	2	620	66.2	4.77	11.25	molecular chaperone DnaK [Arthrobacter alpinus]

gi652423951	1.03	1	1	1744	183.5	5.35	11.24	DEAD/DEAH box helicase [Arthrobacter castelli]
gi651431090	12.23	1	1	188	20.0	5.22	11.24	methyltransferase [Arthrobacter sanguinis]
gi189029025	2.15	1	1	465	47.9	5.22	11.23	RecName: Full=UDP-N-acetylmuramate--L-alanine ligase; AltName: Full=UDP-N-acetylmuramoyl-L
gi470220215	3.83	2	1	235	24.7	9.26	11.23	50S ribosomal protein L1 [Arthrobacter gangotriensis Lz1y]
gi765008001	2.02	1	1	889	95.7	5.86	11.21	hypothetical protein [Arthrobacter sp. A3]
gi542107926	1.20	1	1	585	61.3	5.14	11.20	acetyl-COA carboxylase [Arthrobacter sp. AK-YN10]
gi518312002	2.75	3	1	546	59.7	5.01	11.20	hypothetical protein [Arthrobacter sp. TB 23]
gi651485932	3.67	2	1	300	32.8	6.18	11.19	LysR family transcriptional regulator [Arthrobacter sp. Br18]
gi742852742	2.41	4	2	582	63.3	5.20	11.16	hypothetical protein [Arthrobacter sp. W1]
gi307746328	2.40	1	1	500	54.4	8.37	11.15	D-serine/D-alanine/glycine transporter [Arthrobacter arilaitensis Re117]
gi930827562	2.96	6	1	405	43.9	6.83	11.11	lactate dehydrogenase [Arthrobacter arilaitensis]
gi640194107	9.53	10	2	472	53.7	8.25	11.11	hypothetical protein [Arthrobacter sp. 31Y]
gi116611372	8.50	9	2	412	45.2	10.74	11.11	protein of unknown function DUF58 [Arthrobacter sp. FB24]
gi757626376	14.48	2	1	145	15.9	9.52	11.02	ATPase [Arthrobacter sp. SPG23]
gi654812977	4.32	3	1	324	35.6	5.39	11.01	aldo/keto reductase [Arthrobacter sp. MA-N2]
gi674644754	4.35	2	1	391	39.6	4.89	10.98	NAD-dependent malic enzyme [Arthrobacter sp. 11W110_air]
gi917442071	1.72	1	1	1107	115.8	6.70	10.97	hypothetical protein [Arthrobacter albus]
gi639130872	24.75	5	1	101	10.4	5.34	10.96	hypothetical protein, partial [Arthrobacter sp. CAL618]
gi759721321	22.86	4	1	70	7.3	6.00	10.94	hypothetical protein [Arthrobacter nicotinovorans]
gi917442168	0.84	2	1	1195	128.8	5.16	10.93	chromosome segregation protein SMC [Arthrobacter albus]
gi651440552	7.32	2	1	205	23.5	5.85	10.90	nuclease PIN [Arthrobacter sp. H14]
gi651443101	0.62	1	1	1441	156.3	7.85	10.85	peptide synthetase [Arthrobacter sp. 9MFCol3.1]
gi737786465	1.58	1	1	506	55.7	8.92	10.82	hypothetical protein [Arthrobacter albus]
gi640201541	2.74	1	1	583	63.1	8.12	10.81	HNH endonuclease [Arthrobacter sp. 31Y]
gi654814020	4.98	1	1	321	34.3	5.33	10.81	ABC transporter [Arthrobacter sp. MA-N2]
gi517605193	2.35	1	1	722	77.7	5.90	10.81	peptide ABC transporter ATP-binding protein [Arthrobacter sp. 131MFCol6.1]
gi307745306	3.81	1	1	446	50.0	5.00	10.75	glutamate--ammonia ligase, type I [Arthrobacter arilaitensis Re117]
gi651483475	2.21	5	2	1222	131.3	5.31	10.72	chromosome segregation protein SMC [Arthrobacter sp. Br18]
gi476399575	2.14	1	1	701	76.4	6.55	10.70	acyl-CoA oxidase domain-containing protein [Arthrobacter crystallopoietes BAB-32]
gi765009240	21.70	2	1	106	11.6	10.52	10.70	DNA-binding protein [Arthrobacter sp. A3]
gi737787338	2.14	1	1	560	62.5	5.07	10.68	ABC transporter ATP-binding protein [Arthrobacter albus]
gi444896989	4.86	11	1	473	46.8	6.05	10.68	Conserved protein [Mycobacterium tuberculosis H37Rv]
gi927033186	2.22	5	1	810	86.5	6.24	10.67	transcription accessory protein [Arthrobacter sp. LS16]
gi542110244	5.25	5	1	324	35.7	6.40	10.67	hypothetical protein M707_02675 [Arthrobacter sp. AK-YN10]
gi928488758	2.62	1	1	343	36.6	6.46	10.64	fructose-bisphosphate aldolase [Arthrobacter alpinus]
gi517592619	3.19	1	1	502	55.7	5.99	10.64	RNA nucleotidyltransferase [Arthrobacter sp. 135MFCol5.1]
gi517604925	4.90	2	1	245	26.4	5.25	10.63	MULTISPECIES: hypothetical protein [Arthrobacter]
gi823667929	3.43	4	1	613	66.3	7.09	10.62	long-chain fatty acid--CoA ligase [Arthrobacter sp. YC-RL1]
gi928486873	10.38	2	1	106	11.2	10.54	10.60	DNA-binding protein [Arthrobacter alpinus]
gi910741760	1.28	1	1	627	67.8	8.85	10.59	methionine synthase [Arthrobacter sp. Hiyo4]
gi359306031	3.93	7	2	865	95.8	5.35	10.58	chaperone ClpB [Arthrobacter globiformis NBRC 12137]
gi742758956	2.59	1	1	540	55.1	9.76	10.58	hypothetical protein RM50_01435 [Arthrobacter phenanthrenivorans]
gi651439573	1.50	1	1	866	94.6	5.50	10.57	glycogen phosphorylase [Arthrobacter sp. H14]
gi767257814	4.03	2	1	496	53.9	6.01	10.56	hypothetical protein UM93_09560 [Arthrobacter sp. IHBB 11108]
gi910747899	1.66	4	1	603	67.4	8.34	10.53	multifunctional 2-oxoglutarate metabolism enzyme [Arthrobacter sp. Hiyo8]
gi757623381	4.48	3	1	491	50.1	8.51	10.51	protoporphyrinogen oxidase [Arthrobacter sp. SPG23]
gi674645239	6.39	3	3	532	57.5	5.68	10.50	putative ABC transporter ATP-binding protein YheS [Arthrobacter sp. 11W110_air]
gi910249031	1.94	2	1	412	44.9	9.07	10.48	transposase [Arthrobacter siccitolerans]
gi654824595	10.71	1	1	112	12.9	8.59	10.46	ArsR family transcriptional regulator [Arthrobacter sp. I3]
gi742758858	1.23	4	1	1134	120.7	5.35	10.46	histidine kinase [Arthrobacter phenanthrenivorans]
gi517600184	2.78	3	1	396	42.8	5.88	10.42	dehydrogenase [Arthrobacter sp. 162MFSha1.1]
gi760112697	1.60	2	1	1190	128.3	5.30	10.41	chromosome segregation protein SMC [Arthrobacter chlorophenolicus]
gi443480776	1.85	2	1	702	76.5	7.08	10.34	acyl-CoA oxidase domain-containing protein [Arthrobacter nitrophenolicus]
gi517591906	6.20	1	1	242	25.7	5.86	10.30	L-ribulose-5-phosphate 4-epimerase [Arthrobacter sp. 135MFCol5.1]
gi476402199	16.44	4	1	73	8.4	9.19	10.30	translation initiation factor IF-1 [Arthrobacter crystallopoietes BAB-32]
gi823666980	3.25	2	1	707	74.7	6.99	10.29	nitrite reductase [Arthrobacter sp. YC-RL1]
gi654825837	4.17	2	2	696	74.1	8.21	10.28	primosomal protein N' [Arthrobacter sp. H5]
gi927032000	0.68	2	1	1034	111.4	5.30	10.28	hypothetical protein AFL94_06390 [Arthrobacter sp. LS16]
gi759755866	2.42	1	1	869	91.7	5.55	10.24	ABC transporter [Arthrobacter sp. 131MFCol6.1]

gi737788140	3.56	2	1	478	49.8	9.55	10.23	hypothetical protein [Arthrobacter albus]
gi307744582	4.47	1	1	380	39.1	6.32	10.23	putative cysteine desulfurase [Arthrobacter arilaitensis Re117]
gi759705414	2.00	4	1	501	54.2	8.66	10.23	malate:quinone oxidoreductase [Arthrobacter globiformis]
gi651481425	4.41	1	1	431	46.8	8.76	10.22	alpha-hydroxy-acid oxidizing enzyme [Arthrobacter sp. Br18]
gi927031387	4.50	2	1	289	30.3	5.50	10.20	thioredoxin [Arthrobacter sp. LS16]
gi162954191	2.37	5	1	337	35.9	6.02	10.20	3-dehydroquinate synthase [Renibacterium salmoninarum ATCC 33209]
gi170783527	15.24	1	1	105	11.7	5.47	10.19	unknown (plasmid) [Arthrobacter sp. AK-1]
gi742759528	2.02	2	2	889	95.4	6.24	10.19	phosphoenolpyruvate synthase [Arthrobacter phenanthrenivorans]
gi654814989	4.91	1	1	326	35.7	5.90	10.18	hypothetical protein [Arthrobacter sp. MA-N2]
gi651434955	1.07	2	1	654	71.6	5.34	10.18	acetyl-coenzyme A synthetase [Arthrobacter sp. H41]
gi759729754	2.03	1	1	639	68.9	6.52	10.17	cold-shock protein [Arthrobacter sp. L77]
gi654812221	3.83	3	2	574	60.3	10.52	10.16	DEAD/DEAH box helicase [Arthrobacter sp. MA-N2]
gi470217420	5.15	5	1	408	44.4	6.16	10.14	tellurite resistance protein [Arthrobacter gangotriensis Lz1y]
gi470215904	2.16	1	1	371	40.3	6.54	10.14	putative ATPase (AAA+ superfamily) [Arthrobacter gangotriensis Lz1y]
gi654818670	4.73	3	1	528	54.5	9.17	10.13	oxidoreductase [Arthrobacter sp. UNC362MFTsu5.1]
gi910251060	8.18	1	1	220	23.5	8.07	10.13	DNA-3-methyladenine glycosidase [Arthrobacter siccitolerans]
gi674646806	1.87	2	1	961	101.9	7.03	10.12	Putative HTH-type transcriptional regulatorc/MT0914 [Arthrobacter sp. 11W110_air]
gi742072368	4.10	2	1	463	48.8	5.00	10.11	amino acid decarboxylase, pyridoxal-dependent protein [Arthrobacter sp. MWB30]
gi910695657	33.71	2	2	89	10.3	9.98	10.10	30S ribosomal protein S15 [Arthrobacter sp. Hiyo6]
gi654817676	2.70	2	2	1183	126.0	5.57	10.10	1-pyrroline-5-carboxylate dehydrogenase [Arthrobacter sp. UNC362MFTsu5.1]
gi476401728	8.33	2	1	264	27.4	5.20	10.09	1,6-dihydroxycyclohexa-2,4-diene-1-carboxylate dehydrogenase [Arthrobacter crystallopoietes BA[illegible]
gi930826069	2.78	4	1	468	49.2	10.43	10.07	hypothetical protein AOZ07_07055 [Arthrobacter arilaitensis]
gi937259163	1.50	3	1	1133	121.9	5.26	10.06	pyruvate carboxylase [Arthrobacter sp. Edens01]
gi916813652	5.87	2	1	341	37.3	9.19	10.06	hypothetical protein [Arthrobacter nicotinovorans]
gi916691436	2.76	1	1	399	41.9	9.39	10.06	hypothetical protein [Arthrobacter castelli]
gi517604325	2.06	1	1	535	57.3	5.87	10.05	acetyl-CoA carboxylase subunit beta [Arthrobacter sp. 131MFCol6.1]
gi767257052	1.06	3	1	658	73.5	5.26	10.01	hypothetical protein UM93_04370 [Arthrobacter sp. IHBB 11108]
gi517599720	6.54	1	1	321	34.4	4.78	10.00	hypothetical protein [Arthrobacter sp. 162MFSha1.1]
gi759766426	2.39	4	1	1045	116.2	7.75	9.99	transposase [Arthrobacter gangotriensis]
gi906448305	1.66	2	1	1147	124.7	5.68	9.99	DNA helicase UvrD [Arthrobacter sp. RIT-PI-e]
gi545108181	1.58	4	1	444	50.0	5.60	9.97	alcaligin biosynthesis enzyme [Arthrobacter sp. AK-YN10]
gi651465402	3.72	4	1	457	50.3	5.43	9.97	serine/threonine protein kinase [Arthrobacter sp. 35/47]
gi651492702	12.41	1	1	137	15.5	6.79	9.95	acetyltransferase [Arthrobacter sp. H20]
gi443481084	4.99	1	1	341	36.2	6.24	9.94	6-phosphofructokinase [Arthrobacter nitrophenolicus]
gi636844661	4.61	3	1	412	42.7	5.30	9.93	acetyl-CoA acetyltransferase [Arthrobacter sp. TB 26]
gi651430353	2.58	4	1	465	51.0	5.31	9.92	adenylosuccinate lyase [Arthrobacter sanguinis]
gi517604571	16.35	8	1	159	15.5	9.44	9.89	hypothetical protein [Arthrobacter sp. 131MFCol6.1]
gi742857001	6.10	5	1	213	23.0	6.70	9.89	methyltransferase [Arthrobacter sp. W1]
gi651466358	2.55	2	1	825	88.8	6.07	9.88	DNA topoisomerase IV subunit A [Arthrobacter sp. 35/47]
gi517609727	1.12	3	1	1157	126.2	5.66	9.87	ATP-binding protein [Arthrobacter sp. 161MFSha2.1]
gi654827932	4.77	14	1	503	52.6	6.02	9.87	L-aspartate oxidase [Arthrobacter sp. H5]
gi652425063	4.19	1	1	191	21.5	9.44	9.85	50S ribosomal protein L5 [Arthrobacter castelli]
gi651442834	6.12	3	1	392	40.7	10.81	9.85	FAD-binding monooxygenase [Arthrobacter sp. 9MFCol3.1]
gi910742743	8.70	1	1	138	14.8	9.54	9.84	cell envelope-related transcriptional attenuator [Arthrobacter sp. Hiyo8]
gi162954743	3.64	1	1	220	21.9	9.36	9.83	transglycosylase family protein [Renibacterium salmoninarum ATCC 33209]
gi908740221	4.64	8	1	323	33.6	4.44	9.80	hypothetical protein [Arthrobacter arilaitensis]
gi916863743	1.12	2	1	1164	121.0	7.49	9.79	hypothetical protein [Arthrobacter sp. 35/47]
gi918265178	2.80	2	1	607	65.2	7.66	9.79	type IV secretion system-coupling protein virD4 [Arthrobacter sp. Hiyo1]
gi651494343	1.87	7	1	750	81.8	5.54	9.75	ATPase AAA [Arthrobacter sp. H20]
gi937262087	5.88	6	1	391	42.0	5.76	9.73	hypothetical protein AO716_02400 [Arthrobacter sp. Edens01]
gi759732475	0.66	1	1	1669	177.4	5.55	9.72	DEAD/DEAH box helicase [Arthrobacter sp. L77]
gi635350643	1.34	1	1	1563	159.8	5.03	9.68	calcineurin-like phosphoesterase family protein [Arthrobacter siccitolerans]
gi116609487	1.20	2	1	830	90.4	6.29	9.67	ATP-dependent DNA helicase PcrA [Arthrobacter sp. FB24]
gi652423476	2.70	5	1	445	46.1	4.97	9.66	glutamyl-tRNA reductase [Arthrobacter castelli]
gi757622664	2.70	2	2	1183	125.8	5.76	9.65	1-pyrroline-5-carboxylate dehydrogenase [Arthrobacter sp. SPG23]
gi636847477	0.96	2	1	1047	112.1	5.94	9.64	acriflavin resistance protein [Arthrobacter sp. TB 26]
gi307743957	5.69	5	1	299	32.9	6.39	9.64	LysR-family transcriptional regulator [Arthrobacter arilaitensis Re117]
gi545107753	3.54	1	1	395	44.5	9.85	9.62	hypothetical protein [Arthrobacter sp. AK-YN10]
gi908697138	3.06	5	1	818	89.7	5.94	9.62	ATP-dependent DNA helicase PcrA [Arthrobacter sp. RIT-PI-e]

gi910697570	3.79	2	1	501	54.5	6.96	9.61	phage terminase [Arthrobacter sp. Hiyo6]
gi489896221	4.92	8	2	590	62.1	6.35	9.60	two-component system sensor histidine kinase [Arthrobacter globiformis]
gi927032129	2.01	1	1	547	59.3	6.35	9.60	hypothetical protein AFL94_07260 [Arthrobacter sp. LS16]
gi737790717	2.05	3	1	1168	129.0	4.88	9.59	DNA-directed RNA polymerase subunit beta [Arthrobacter albus]
gi910738261	16.38	3	1	116	12.6	5.03	9.59	conserved hypothetical protein [Arthrobacter sp. Hiyo4]
gi928487147	6.21	2	1	338	34.2	4.60	9.57	thiamine-monophosphate kinase [Arthrobacter alpinus]
gi307744773	2.13	1	1	517	55.1	8.41	9.56	Mg chelatase-like protein [Arthrobacter arilaitensis Re117]
gi759715954	4.07	4	2	590	62.0	10.78	9.55	DEAD/DEAH box helicase [Arthrobacter sp. AK-YN10]
gi759725340	4.66	2	1	386	41.7	6.21	9.54	DNA-binding protein [Arthrobacter sp. I3]
gi742070646	7.89	1	1	228	24.4	8.00	9.53	putative GntR family transcriptional regulator [Arthrobacter sp. MWB30]
gi476399853	3.11	2	1	514	55.8	10.20	9.53	signal recognition particle protein [Arthrobacter crystallopoietes BAB-32]
gi930827440	2.66	5	1	451	50.9	5.82	9.51	hypothetical protein AOZ07_14910 [Arthrobacter arilaitensis]
gi916691453	9.35	1	1	246	25.9	6.04	9.49	hypothetical protein [Arthrobacter castelli]
gi648573884	3.37	1	1	624	65.9	6.07	9.48	sodium:proton antiporter [Arthrobacter sp. 162MFSha1.1]
gi517592142	5.57	3	1	323	35.3	7.90	9.47	hypothetical protein [Arthrobacter sp. 135MFCol5.1]
gi937258478	1.37	3	1	1167	130.2	5.63	9.46	hypothetical protein AO716_10570 [Arthrobacter sp. Edens01]
gi470220534	6.41	3	1	312	34.5	8.79	9.44	glycosyltransferase [Arthrobacter gangotriensis Lz1y]
gi652422987	1.54	1	1	1172	126.9	5.16	9.43	1-pyrroline-5-carboxylate dehydrogenase [Arthrobacter castelli]
gi651499610	2.75	1	1	545	58.3	5.81	9.39	PucR family transcriptional regulator [Arthrobacter sp. 35W]
gi767258788	2.33	2	1	687	75.2	4.86	9.38	multidrug ABC transporter ATP-binding protein [Arthrobacter sp. IHBB 11108]
gi654815372	8.60	4	1	221	24.3	5.68	9.37	GntR family transcriptional regulator [Arthrobacter sp. PAO19]
gi162953407	6.80	2	1	250	27.1	6.65	9.37	glutamate transport ATP-binding protein [Renibacterium salmoninarum ATCC 33209]
gi917760239	6.03	4	1	282	31.0	5.31	9.36	phytanoyl-CoA dioxygenase [Arthrobacter sp. L77]
gi723607408	1.75	2	1	458	49.8	8.62	9.35	hypothetical protein ART_1185 [Arthrobacter sp. PAMC25486]
gi652423525	3.71	3	2	836	94.8	9.01	9.35	hypothetical protein [Arthrobacter castelli]
gi910749230	2.97	2	1	674	76.4	6.47	9.33	conserved hypothetical protein (plasmid) [Arthrobacter sp. Hiyo8]
gi651439668	16.26	3	2	203	21.6	10.08	9.33	hypothetical protein [Arthrobacter sp. H14]
gi511534683	2.37	1	1	337	35.8	5.82	9.32	putative LacI-family transcriptional regulator (plasmid) [Arthrobacter nicotinovorans]
gi652424519	3.97	2	1	252	27.2	5.45	9.32	GntR family transcriptional regulator [Arthrobacter castelli]
gi917760327	1.97	4	1	507	55.1	4.36	9.31	trigger factor [Arthrobacter sp. L77]
gi652424855	3.64	4	1	275	28.2	6.15	9.29	purine nucleoside phosphorylase [Arthrobacter castelli]
gi470220755	2.69	1	1	484	51.1	6.19	9.29	Aminoglycoside phosphotransferase [Arthrobacter gangotriensis Lz1y]
gi918267951	6.61	1	1	257	28.4	5.54	9.23	hypothetical protein AHiyo1_12560 [Arthrobacter sp. Hiyo1]
gi323471156	7.50	2	1	280	30.5	4.98	9.23	putative oxidoreductase, aryl-alcohol dehydrogenase like protein [Arthrobacter phenanthrenivoran
gi927294171	11.39	4	2	237	25.4	5.19	9.23	haloacid dehalogenase [Arthrobacter sp. ERGS1:01]
gi742757987	3.69	2	1	407	44.3	5.94	9.22	pilus assembly protein CpaF [Arthrobacter phenanthrenivorans]
gi654822399	4.03	1	1	422	44.5	5.14	9.22	amidohydrolase [Arthrobacter sp. I3]
gi470217940	3.22	4	1	466	51.5	6.25	9.20	monooxygenase [Arthrobacter gangotriensis Lz1y]
gi742853362	9.38	2	2	373	39.1	6.74	9.19	UDP-N-acetylglucosamine--N-acetylmuramyl-(pentapeptide) pyrophosphoryl-undecaprenol N-acety
gi654822242	4.69	1	1	426	45.9	8.32	9.19	hypothetical protein, partial [Arthrobacter sp. I3]
gi476399111	4.81	3	2	457	48.9	4.96	9.19	succinate-semialdehyde dehydrogenase [Arthrobacter crystallopoietes BAB-32]
gi910746129	4.09	7	1	440	47.1	10.52	9.19	hypothetical protein AHiyo8_38310 [Arthrobacter sp. Hiyo8]
gi742071844	3.49	4	2	889	96.6	6.15	9.16	LuxR family transcriptional regulator [Arthrobacter sp. MWB30]
gi359307287	2.16	2	1	742	81.4	5.57	9.16	hypothetical protein ARGLB_016_00020 [Arthrobacter globiformis NBRC 12137]
gi542110119	1.60	4	1	1123	124.8	5.47	9.15	hypothetical protein M707_02020 [Arthrobacter sp. AK-YN10]
gi651496204	3.20	2	1	719	76.3	9.39	9.14	copper resistance protein CopD [Arthrobacter sp. H20]
gi928488768	6.39	1	1	360	39.8	10.70	9.13	hypothetical protein AOC05_08450 [Arthrobacter alpinus]
gi937262492	4.78	6	2	837	85.2	4.65	9.12	hypothetical protein AO716_04750 [Arthrobacter sp. Edens01]
gi443479856	1.44	1	1	762	82.4	5.11	9.12	NAD-dependent DNA ligase [Arthrobacter nitrophenolicus]
gi307743637	3.86	1	1	518	56.1	7.55	9.11	conserved hypothetical protein [Arthrobacter arilaitensis Re117]
gi723607964	3.10	1	1	258	27.8	4.97	9.09	hypothetical protein ART_1741 [Arthrobacter sp. PAMC25486]
gi917739663	4.60	3	1	261	27.8	8.62	9.09	multidrug ABC transporter ATPase [Arthrobacter sp. W1]
gi542109239	15.45	8	1	110	12.1	4.88	9.08	hypothetical protein M707_07265 [Arthrobacter sp. AK-YN10]
gi219860116	5.25	1	1	476	48.1	9.04	9.07	protoporphyrinogen oxidase [Arthrobacter chlorophenolicus A6]
gi919218798	2.99	8	1	536	55.8	11.50	9.06	hypothetical protein [Arthrobacter sp. YC-RL1]
gi656049967	3.02	2	1	331	34.7	5.06	9.06	prephenate dehydratase [Brevibacterium album]
gi757624693	4.97	1	1	443	46.7	11.58	9.05	MFS transporter [Arthrobacter sp. SPG23]
gi910696411	4.19	1	1	167	18.2	9.19	9.05	putative ATP-dependent RNA helicase QP509L [Arthrobacter sp. Hiyo6]
gi927294713	3.41	4	2	381	41.2	8.05	9.03	alpha-mannosyltransferase [Arthrobacter sp. ERGS1:01]

gi517598828	4.42	3	1	498	54.8	5.59	9.02	catalase [Arthrobacter sp. 162MFSha1.1]
gi910249923	1.02	1	1	1177	126.2	5.38	9.02	DNA helicase UvrD [Arthrobacter siccitolerans]
gi119949331	1.52	1	1	923	96.7	8.32	9.02	putative ATP-dependent helicase HrpB [Arthrobacter aurescens TC1]
gi737789400	8.73	4	1	126	14.6	11.46	9.01	50S ribosomal protein L20 [Arthrobacter albus]
gi470217590	4.32	2	2	533	56.9	5.64	9.00	ABC transporter [Arthrobacter gangotriensis Lz1y]
gi765011146	3.25	3	1	553	60.9	4.69	9.00	peptide ABC transporter substrate-binding protein [Arthrobacter sp. A3]
gi765003731	3.19	1	1	376	40.3	5.08	8.99	hypothetical protein [Arthrobacter sp. A3]
gi674644784	4.65	1	1	387	41.6	5.08	8.98	Acyl-CoA dehydrogenase [Arthrobacter sp. 11W110_air]
gi640203988	5.32	3	1	432	46.2	5.74	8.98	hypothetical protein [Arthrobacter sp. 31Y]
gi927295767	6.30	1	1	270	29.7	8.56	8.98	haloacid dehalogenase [Arthrobacter sp. ERGS1:01]
gi723608353	4.26	3	2	704	77.5	5.10	8.96	elongation factor G [Arthrobacter sp. PAMC25486]
gi635349793	1.95	1	1	922	98.3	8.34	8.94	glycosyl transferase, WecB/TagA/CpsF family protein [Arthrobacter siccitolerans]
gi757625673	2.19	2	1	640	71.6	5.68	8.93	hypothetical protein TV39_04745 [Arthrobacter sp. SPG23]
gi551253699	1.99	2	1	1104	121.5	5.00	8.93	ATP-dependent DNA helicase [Arthrobacter sp. PAO19]
gi116613053	3.56	2	1	421	46.8	6.13	8.91	hypothetical protein Arth_4325 (plasmid) [Arthrobacter sp. FB24]
gi640203840	3.29	2	1	487	52.2	8.56	8.90	polysaccharide biosynthesis protein [Arthrobacter sp. 31Y]
gi927032442	8.17	5	2	404	45.9	9.72	8.90	integrase [Arthrobacter sp. LS16]
gi359305179	3.91	3	1	358	37.7	8.32	8.89	putative glycosidase [Arthrobacter globiformis NBRC 12137]
gi742072155	7.66	6	1	261	28.5	6.76	8.89	hypothetical protein ANMWB30_08590 [Arthrobacter sp. MWB30]
gi937259160	4.69	3	1	320	34.1	7.84	8.89	glucokinase [Arthrobacter sp. Edens01]
gi476399508	1.04	3	1	1634	180.0	5.85	8.89	putative helicase [Arthrobacter crystallopoietes BAB-32]
gi162955428	3.14	1	1	350	37.2	8.87	8.86	diacylglycerol kinase family protein [Renibacterium salmoninarum ATCC 33209]
gi651430802	2.17	3	1	507	55.4	4.87	8.85	aldehyde dehydrogenase [Arthrobacter sanguinis]
gi937258105	3.38	1	1	385	39.4	5.03	8.85	malate dehydrogenase [Arthrobacter sp. Edens01]
gi928488104	4.87	4	1	472	48.8	5.27	8.84	hypothetical protein AOC05_15810 [Arthrobacter alpinus]
gi470217279	2.75	2	1	545	58.7	4.65	8.82	family 5 extracellular solute-binding protein [Arthrobacter gangotriensis Lz1y]
gi674647070	1.78	5	1	450	51.0	5.57	8.81	hypothetical protein BN1051_03290 [Arthrobacter sp. 11W110_air]
gi651456539	1.76	1	1	512	56.3	5.38	8.81	ATPase [Arthrobacter sp. 35/47]
gi219858652	1.40	2	1	1215	131.4	5.87	8.80	conserved hypothetical protein [Arthrobacter chlorophenolicus A6]
gi651491680	1.54	3	1	1104	118.1	4.96	8.79	carbamoyl phosphate synthase large subunit [Arthrobacter sp. H20]
gi648224528	4.44	2	1	405	44.1	5.25	8.79	glutaryl-CoA dehydrogenase [Arthrobacter sp. M2012083]
gi918265738	2.78	2	1	503	57.3	9.77	8.79	putative uncharacterized protein YkfC [Arthrobacter sp. Hiyo1]
gi759763875	6.47	5	2	340	36.0	5.22	8.79	fructose 1,6-bisphosphatase [Arthrobacter gangotriensis]
gi910740437	5.23	8	1	421	46.9	9.17	8.79	uncharacterized protein YcaQ [Arthrobacter sp. Hiyo4]
gi651489744	4.33	1	1	323	33.4	7.42	8.78	alcohol dehydrogenase [Arthrobacter sp. H20]
gi914715064	5.64	1	1	319	33.5	5.29	8.78	D-glycerate dehydrogenase [Arthrobacter sp. ZBG10]
gi916359192	4.38	5	1	297	30.7	6.11	8.78	glycosyl transferase family 1 [Arthrobacter sp. 135MFCol5.1]
gi672940882	12.57	5	2	350	37.2	5.12	8.78	recombination protein RecA [Pimelobacter simplex]
gi517607564	1.63	1	1	1166	127.5	6.84	8.77	DNA polymerase III subunit alpha [Arthrobacter sp. 161MFSha2.1]
gi517599398	5.72	3	2	577	63.2	8.50	8.77	multidrug ABC transporter ATP-binding protein [Arthrobacter sp. 162MFSha1.1]
gi636844977	2.21	2	1	589	62.7	8.79	8.77	conjugal transfer protein [Arthrobacter sp. TB 26]
gi651441866	7.33	1	1	232	24.7	9.95	8.77	hypothetical protein [Arthrobacter sp. 9MFCol3.1]
gi765011613	4.15	1	1	410	44.4	9.73	8.76	hypothetical protein [Arthrobacter sp. A3]
gi119947513	4.74	3	1	211	23.3	10.89	8.75	putative S4 domain protein [Arthrobacter aurescens TC1]
gi910250341	9.58	3	1	167	18.2	7.03	8.74	metal-dependent phosphodiesterase [Arthrobacter siccitolerans]
gi636845306	4.87	5	1	472	48.9	5.99	8.73	acetyl-CoA acetyltransferase [Arthrobacter sp. TB 26]
gi517606011	2.70	3	2	1187	127.5	5.19	8.72	chromosome partitioning protein Smc [Arthrobacter sp. 161MFSha2.1]
gi307745677	8.81	2	1	261	27.8	5.24	8.72	phenylacetic acid degradation protein PaaB [Arthrobacter arilaitensis Re117]
gi654817057	2.87	2	2	732	76.0	6.38	8.72	ATPase [Arthrobacter sp. UNC362MFTsu5.1]
gi823665443	2.32	3	1	862	89.9	8.54	8.71	hydrolase [Arthrobacter sp. YC-RL1]
gi654822099	10.00	2	1	250	27.6	6.54	8.70	hypothetical protein [Arthrobacter sp. I3]
gi470217035	2.69	2	1	558	59.6	7.31	8.70	fumarate reductase/succinate dehydrogenase flavoprotein domain-containing protein [Arthrobacte
gi767259001	1.31	2	1	612	67.0	5.12	8.69	aspartyl-tRNA synthetase [Arthrobacter sp. IHBB 11108]
gi654815847	7.01	4	1	157	17.3	4.41	8.68	16S rRNA maturation RNase YbeY [Arthrobacter sp. UNC362MFTsu5.1]
gi742857458	3.21	2	1	467	50.9	5.80	8.67	PucR family transcriptional regulator [Arthrobacter sp. W1]
gi910252267	2.87	2	2	522	56.0	4.98	8.66	choline dehydrogenase [Arthrobacter siccitolerans]
gi769942322	4.12	1	1	388	41.4	7.06	8.66	hypothetical protein [Arthrobacter sp. IHBB 11108]
gi742855455	1.73	4	1	635	70.7	7.71	8.66	glycosyltransferase [Arthrobacter sp. W1]
gi786032010	21.50	1	1	107	11.6	4.96	8.65	GTP cyclohydrolase [Arthrobacter chlorophenolicus]

gi917760229	2.21	2	1	544	60.7	4.93	8.63	alpha-amylase [Arthrobacter sp. L77]
gi651440579	4.35	2	1	322	36.1	5.16	8.61	hypothetical protein [Arthrobacter sp. H14]
gi917739446	5.54	3	1	415	44.6	5.21	8.61	hypothetical protein [Arthrobacter sp. W1]
gi917760320	1.18	1	1	1102	122.9	5.01	8.60	isoleucine--tRNA ligase [Arthrobacter sp. L77]
gi927294628	9.91	2	1	111	11.9	11.39	8.59	DNA-binding protein [Arthrobacter sp. ERGS1:01]
gi737796581	3.91	3	2	1151	123.5	5.95	8.59	1-pyrroline-5-carboxylate dehydrogenase [Arthrobacter sp. H20]
gi917530512	0.95	1	1	1158	124.6	5.22	8.58	DNA helicase UvrD [Arthrobacter sp. PAMC25486]
gi403231739	2.16	1	1	510	53.4	10.80	8.58	putative integral membrane protein [Arthrobacter sp. Rue61a]
gi470217312	1.24	1	1	1376	143.3	7.09	8.57	cell division protein FtsK [Arthrobacter gangotriensis Lz1y]
gi914715191	3.67	1	1	218	23.9	6.65	8.57	molybdenum cofactor sulfurase [Arthrobacter sp. ZBG10]
gi910744863	3.54	3	1	424	45.3	7.27	8.56	glutathione-binding protein GsiB [Arthrobacter sp. Hiyo8]
gi323468742	1.97	2	2	912	97.5	6.13	8.55	response regulator containing a CheY-like receiver domain and an HTH DNA-binding domain prote
gi723609197	1.31	1	1	837	89.3	6.24	8.55	hypothetical protein ART_2974 [Arthrobacter sp. PAMC25486]
gi651459896	3.51	1	1	370	40.4	6.20	8.55	hypothetical protein [Arthrobacter sp. 35/47]
gi910696462	5.03	4	1	199	21.7	6.95	8.54	hhh-Gpd family protein [Arthrobacter sp. Hiyo6]
gi723606315	1.24	1	1	1131	120.5	5.29	8.54	pyruvate carboxylase [Arthrobacter sp. PAMC25486]
gi737807785	3.70	3	1	486	51.8	4.56	8.51	hydrolase [Arthrobacter sp. H5]
gi651486518	1.98	1	1	353	37.6	9.91	8.51	hypothetical protein, partial [Arthrobacter sp. Br18]
gi742070409	1.47	1	1	1092	121.9	6.52	8.51	hypothetical protein ANMWB30_24500 [Arthrobacter sp. MWB30]
gi119951606	5.05	4	1	317	34.0	10.26	8.51	putative Membrane protein (plasmid) [Arthrobacter aurescens TC1]
gi757622607	14.53	8	1	172	18.4	6.70	8.50	MarR family transcriptional regulator [Arthrobacter sp. SPG23]
gi767258179	0.89	2	1	2461	257.0	4.96	8.50	polyketide synthase [Arthrobacter sp. IHBB 11108]
gi927293998	13.77	3	2	167	18.2	4.51	8.49	hypothetical protein AL755_08120 [Arthrobacter sp. ERGS1:01]
gi737789559	2.53	2	1	513	55.6	9.54	8.49	signal recognition particle protein [Arthrobacter albus]
gi654814666	3.77	1	1	345	36.4	5.99	8.49	tRNA(Ile)-lysidine synthetase [Arthrobacter sp. MA-N2]
gi759732307	5.25	1	1	362	38.8	5.33	8.48	oxidoreductase [Arthrobacter sp. L77]
gi759763288	5.24	3	2	248	26.9	7.49	8.47	glutamate ABC transporter ATP-binding protein [Arthrobacter gangotriensis]
gi648224640	2.47	2	1	850	93.2	4.77	8.46	aminopeptidase N [Arthrobacter sp. M2012083]
gi767257607	2.46	4	1	406	44.2	4.94	8.46	oxidoreductase [Arthrobacter sp. IHBB 11108]
gi476398940	2.48	1	1	966	100.8	5.78	8.46	molybdopterin binding aldehyde oxidase and xanthine dehydrogenase [Arthrobacter crystallopoiet
gi403230524	4.52	2	1	398	42.7	5.76	8.45	putative flp pilus assembly protein CpaE [Arthrobacter sp. Rue61a]
gi470216083	2.18	1	1	550	57.7	10.48	8.41	oligopeptide ABC transporter membrane protein [Arthrobacter gangotriensis Lz1y]
gi517601958	4.74	2	1	274	30.4	5.25	8.41	hypothetical protein [Arthrobacter sp. 162MFSha1.1]
gi517602930	1.82	1	1	1099	118.4	4.92	8.41	carbamoyl phosphate synthase large subunit [Arthrobacter sp. 131MFCol6.1]
gi648574351	1.43	1	1	700	75.8	4.87	8.41	hypothetical protein [Arthrobacter sp. 162MFSha1.1]
gi470221148	9.61	3	2	406	44.5	7.30	8.40	Transposase DDE domain protein [Arthrobacter gangotriensis Lz1y]
gi917732919	0.54	1	1	1857	204.4	5.29	8.40	hypothetical protein [Arthrobacter sp. MWB30]
gi767258838	5.18	3	1	425	45.3	5.78	8.39	serine hydroxymethyltransferase [Arthrobacter sp. IHBB 11108]
gi914715090	0.43	2	1	3508	366.0	5.10	8.39	non-ribosomal peptide synthetase [Arthrobacter sp. ZBG10]
gi915933709	1.41	2	1	854	89.9	8.98	8.39	transcription accessory protein [Arthrobacter globiformis]
gi937259069	1.52	1	1	657	70.4	6.38	8.38	1-deoxy-D-xylulose-5-phosphate synthase [Arthrobacter sp. Edens01]
gi910749243	4.13	2	1	412	46.3	9.23	8.38	transposase for transposon Tn1546 (plasmid) [Arthrobacter sp. Hiyo8]
gi323468320	3.43	3	1	437	48.9	6.89	8.38	uncharacterized methicillin resistance protein [Arthrobacter phenanthrenivorans Sphe3]
gi916290016	2.96	2	1	371	40.3	6.21	8.38	MULTISPECIES: cyclic pyranopterin phosphate synthase MoaA [Arthrobacter]
gi937258504	1.41	2	1	1277	143.4	6.64	8.38	hypothetical protein AO716_10715 [Arthrobacter sp. Edens01]
gi517607940	4.38	10	1	502	53.1	4.84	8.37	hypothetical protein [Arthrobacter sp. 161MFSha2.1]
gi910695374	5.29	2	1	378	40.0	5.26	8.37	non-hemolytic phospholipase C [Arthrobacter sp. Hiyo6]
gi170783480	4.28	1	1	397	43.1	5.77	8.36	putative oxidoreductase domain protein (plasmid) [Arthrobacter sp. AK-1]
gi742758980	2.82	1	1	497	52.1	6.33	8.36	hypothetical protein RM50_01610 [Arthrobacter phenanthrenivorans]
gi908698815	6.58	3	1	243	25.6	8.37	8.36	ribonuclease HII [Arthrobacter sp. RIT-PI-e]
gi918265016	2.58	1	1	582	64.0	6.74	8.36	oxygen-dependent choline dehydrogenase [Arthrobacter sp. Hiyo1]
gi916816046	2.56	1	1	586	64.3	6.87	8.36	choline dehydrogenase [Arthrobacter sp. MA-N2]
gi654824189	2.84	1	1	915	94.8	7.50	8.36	phosphoenolpyruvate synthase [Arthrobacter sp. I3]
gi930827792	7.14	1	1	308	32.9	9.09	8.36	sugar ABC transporter permease [Arthrobacter arilaitensis]
gi927294785	6.51	2	1	215	23.6	5.83	8.36	hypothetical protein AL755_13475 [Arthrobacter sp. ERGS1:01]
gi742756220	2.22	4	1	856	90.1	6.80	8.35	histidine kinase [Arthrobacter phenanthrenivorans]
gi737790086	1.56	1	1	703	75.3	5.05	8.35	3-hydroxyacyl-CoA dehydrogenase [Arthrobacter albus]
gi928486230	0.44	1	1	2031	209.3	5.03	8.34	hypothetical protein AOC05_03140 [Arthrobacter alpinus]
gi916692542	4.58	1	1	262	28.3	5.63	8.34	hypothetical protein [Arthrobacter castelli]

gi916691894	2.00	1	1	551	57.1	7.59	8.33	hypothetical protein [Arthrobacter castelli]
gi916692568	11.23	1	1	187	21.0	10.84	8.33	hypothetical protein [Arthrobacter castelli]
gi823666701	1.61	1	1	991	105.4	9.07	8.33	monovalent cation/H+ antiporter subunit A [Arthrobacter sp. YC-RL1]
gi651467251	1.33	1	1	1277	140.7	6.10	8.32	alpha-ketoglutarate decarboxylase [Arthrobacter sp. 35/47]
gi542106319	0.87	3	1	1147	125.5	5.54	8.32	ATP-binding protein [Arthrobacter sp. AK-YN10]
gi928486812	2.25	7	2	933	100.7	4.94	8.31	aconitate hydratase [Arthrobacter alpinus]
gi654824711	5.45	4	1	257	27.4	5.99	8.31	DeoR family transcriptional regulator [Arthrobacter sp. I3]
gi674646386	4.02	1	1	423	44.4	9.00	8.31	Signal transduction histidine-protein kinase/phosphatase DegS [Arthrobacter sp. 11W110_air]
gi162955663	4.10	2	1	390	41.9	9.23	8.30	sensor transduction protein kinase [Renibacterium salmoninarum ATCC 33209]
gi654828559	9.77	1	1	256	26.2	5.24	8.29	3-hydroxy-2-methylbutyryl-CoA dehydrogenase [Arthrobacter sp. H5]
gi674646805	2.10	2	1	477	48.1	9.70	8.29	4-hydroxybenzoate transporter PcaK [Arthrobacter sp. 11W110_air]
gi640199153	1.29	1	1	855	95.6	6.57	8.29	hypothetical protein [Arthrobacter sp. 31Y]
gi651429917	2.96	3	1	304	33.5	4.97	8.28	30S ribosomal protein S2 [Arthrobacter sanguinis]
gi652425501	6.37	3	1	251	27.6	6.81	8.27	GntR family transcriptional regulator [Arthrobacter castelli]
gi640197027	0.89	1	1	1350	149.9	6.07	8.27	hypothetical protein [Arthrobacter sp. 31Y]
gi910748137	2.27	2	1	308	32.2	9.54	8.27	chromosome partition protein Smc [Arthrobacter sp. Hiyo8]
gi636845070	4.48	7	1	469	47.0	5.20	8.27	flagellar hook protein FlgK [Arthrobacter sp. TB 26]
gi323468508	3.21	4	1	436	45.6	6.25	8.26	hypothetical protein Asphe3_10060 [Arthrobacter phenanthrenivorans Sphe3]
gi742072447	6.60	4	1	197	19.5	5.87	8.26	lipoprotein [Arthrobacter sp. MWB30]
gi119949382	2.60	1	1	384	41.7	9.58	8.26	putative DNA polymerase IV [Arthrobacter aurescens TC1]
gi695270312	5.79	2	1	311	32.5	6.73	8.26	ABC transporter membrane protein [Arthrobacter globiformis]
gi517599014	3.45	2	2	579	63.2	7.06	8.25	FAD-dependent oxidoreductase [Arthrobacter sp. 162MFSha1.1]
gi651429487	11.60	4	2	250	27.5	11.27	8.25	membrane protein [Arthrobacter sanguinis]
gi651496580	1.49	2	1	740	82.3	5.99	8.25	polyphosphate kinase [Arthrobacter sp. H20]
gi928488301	1.97	2	1	557	60.6	7.17	8.25	ribonuclease [Arthrobacter alpinus]
gi910250857	4.59	2	1	283	31.0	6.81	8.24	LLM class F420-dependent oxidoreductase [Arthrobacter siccitolerans]
gi651502944	0.92	1	1	1309	144.7	6.87	8.23	ATP-dependent helicase [Arthrobacter sp. 35W]
gi917013136	4.80	1	1	375	40.4	8.53	8.22	fusaric acid resistance protein [Arthrobacter sanguinis]
gi765012154	3.36	3	1	506	55.6	5.15	8.22	arabinose isomerase [Arthrobacter sp. A3]
gi323469212	2.95	1	1	713	78.1	5.55	8.22	DNA/RNA endonuclease G, NUC1 [Arthrobacter phenanthrenivorans Sphe3]
gi652423338	1.52	3	1	594	63.5	9.04	8.21	multidrug ABC transporter permease [Arthrobacter castelli]
gi639129088	8.03	4	2	361	39.2	5.26	8.20	MULTISPECIES: inositol-3-phosphate synthase [Arthrobacter]
gi908698279	8.04	2	1	224	22.6	10.37	8.19	hypothetical protein [Arthrobacter sp. RIT-PI-e]
gi116613121	0.95	1	1	1575	173.2	5.68	8.19	helicase domain protein (plasmid) [Arthrobacter sp. FB24]
gi910248995	3.52	2	1	256	27.0	5.52	8.19	ABC transporter ATP-binding protein [Arthrobacter siccitolerans]
gi651439153	10.26	3	2	341	37.2	4.92	8.18	ketol-acid reductoisomerase [Arthrobacter sp. H14]
gi937256729	7.06	1	1	326	34.3	6.44	8.18	ABC transporter [Arthrobacter sp. Edens01]
gi517602366	2.99	1	1	469	49.7	6.47	8.13	aspartate ammonia-lyase [Arthrobacter sp. 131MFCol6.1]
gi918449395	1.77	1	1	792	88.1	8.27	8.12	hypothetical protein [Arthrobacter sp. SPG23]
gi652425820	4.49	4	1	535	57.4	5.45	8.11	methylcrotonoyl-CoA carboxylase [Arthrobacter castelli]
gi517593077	6.22	1	1	402	42.5	5.20	8.11	aspartate aminotransferase [Arthrobacter sp. 135MFCol5.1]
gi517604021	3.78	2	1	344	35.0	8.81	8.09	hypothetical protein [Arthrobacter sp. 131MFCol6.1]
gi654823495	1.20	1	1	914	98.2	5.53	8.09	LuxR family transcriptional regulator [Arthrobacter sp. I3]
gi542106680	1.46	2	2	1161	129.5	5.30	8.08	ATP-binding protein [Arthrobacter sp. AK-YN10]
gi517591256	1.63	1	1	1163	126.5	6.89	8.08	DNA polymerase III subunit alpha [Arthrobacter sp. 135MFCol5.1]
gi470221675	3.11	2	1	386	41.6	8.90	8.08	hypothetical protein ADIAG_00528 [Arthrobacter gangotriensis Lz1y]
gi651496304	2.79	4	1	681	73.4	5.47	8.07	anthranilate synthase [Arthrobacter sp. H20]
gi116609659	2.50	3	2	1040	115.3	5.68	8.07	type I site-specific deoxyribonuclease, HsdR family [Arthrobacter sp. FB24]
gi908690919	1.37	1	1	805	85.6	8.32	8.05	transcription accessory protein [Arthrobacter sp. H41]
gi930828275	4.21	1	1	309	34.2	5.20	8.05	alpha/beta hydrolase [Arthrobacter arilaitensis]
gi651463667	2.12	1	1	566	60.4	5.03	8.05	amidase [Arthrobacter sp. 35/47]
gi651434620	7.18	2	2	348	37.2	7.39	8.03	epimerase [Arthrobacter sp. H41]
gi518312761	0.78	1	1	1153	128.5	6.51	8.01	hypothetical protein [Arthrobacter sp. TB 23]
gi542107238	2.98	1	1	637	72.6	8.94	8.00	glycosyltransferase [Arthrobacter sp. AK-YN10]
gi740685326	2.22	1	1	540	56.1	8.54	8.00	hypothetical protein [Arthrobacter sp. PAMC25486]
gi635353212	4.56	1	1	263	28.9	6.15	7.99	ABC transporter family protein [Arthrobacter siccitolerans]
gi695200266	12.50	3	1	64	6.8	4.28	7.99	phthalate dioxygenase ferredoxin subunit (plasmid) [Arthrobacter keyseri]
gi930827677	1.81	3	1	553	58.6	5.08	7.99	amidohydrolase [Arthrobacter arilaitensis]
gi737777007	1.27	1	1	632	69.1	4.97	7.99	hypothetical protein [Arthrobacter sanguinis]

gi939037114	9.93	10	2	151	15.9	4.45	7.99	hypothetical protein [Arthrobacter nitroguajacolicus]
gi654811984	5.10	4	1	294	32.1	10.20	7.98	secretion system protein [Arthrobacter sp. MA-N2]
gi937257990	2.24	1	1	715	78.3	7.25	7.96	hypothetical protein AO716_07600 [Arthrobacter sp. Edens01]
gi908740251	2.62	1	1	344	39.3	10.24	7.96	transposase [Arthrobacter arilaitensis]
gi517601711	1.38	1	1	1157	126.8	5.66	7.95	ATP-binding protein [Arthrobacter sp. 162MFSha1.1]
gi443480201	9.86	3	1	142	15.7	4.97	7.94	DNA-binding ferritin-like protein [Arthrobacter nitrophenolicus]
gi928486771	3.29	2	2	760	81.1	5.36	7.94	hypothetical protein AOC05_06795 [Arthrobacter alpinus]
gi551254154	11.46	3	2	349	38.4	6.74	7.94	membrane protein [Arthrobacter sp. PAO19]
gi759734436	0.93	2	1	970	106.8	6.52	7.94	ABC-ATPase UvrA [Arthrobacter sp. L77]
gi640194001	1.50	1	1	1604	171.8	6.55	7.94	hypothetical protein [Arthrobacter sp. 31Y]
gi470220759	3.91	2	1	409	45.3	5.39	7.94	aspartate aminotransferase [Arthrobacter gangotriensis Lz1y]
gi640195016	0.80	2	1	1618	180.3	5.17	7.93	glutamate dehydrogenase [Arthrobacter sp. 31Y]
gi916869520	4.19	2	1	334	35.4	7.83	7.93	dephospho-CoA kinase [Arthrobacter sp. Br18]
gi307743838	2.19	1	1	547	58.2	5.07	7.93	thiamine pyrophosphate binding domain-containing protein [Arthrobacter arilaitensis Re117]
gi742753833	5.10	3	2	412	45.2	5.48	7.92	DNA polymerase [Arthrobacter phenanthrenivorans]
gi470217222	5.06	2	2	474	52.7	5.00	7.92	glutamine synthetase, type I [Arthrobacter gangotriensis Lz1y]
gi786035378	3.22	2	1	528	59.2	7.06	7.92	hypothetical protein [Arthrobacter chlorophenolicus]
gi636843303	5.99	2	2	568	60.4	5.20	7.92	hypothetical protein [Arthrobacter sp. TB 26]
gi914715201	8.98	2	1	167	17.5	8.21	7.90	HxlR family transcriptional regulator [Arthrobacter sp. ZBG10]
gi652424044	10.25	1	1	244	26.1	9.69	7.90	ABC transporter [Arthrobacter castelli]
gi359305589	1.26	1	1	1430	154.2	6.25	7.90	hypothetical protein ARGLB_059_00060 [Arthrobacter globiformis NBRC 12137]
gi654826167	2.13	1	1	516	53.5	5.90	7.90	aminopeptidase A [Arthrobacter sp. H5]
gi919108184	1.42	1	1	1198	122.3	6.19	7.90	hypothetical protein [Arthrobacter sp. IHBB 11108]
gi908699232	1.09	1	1	1009	109.1	6.06	7.90	monooxygenase [Arthrobacter sp. RIT-PI-e]
gi517592650	5.15	1	1	330	35.8	5.69	7.89	hypothetical protein [Arthrobacter sp. 135MFCol5.1]
gi928985989	1.20	1	1	581	64.2	9.50	7.89	hypothetical protein [Arthrobacter sp. ERGS1:01]
gi759734887	2.26	3	1	708	75.7	5.27	7.89	3-hydroxyacyl-CoA dehydrogenase [Arthrobacter sp. L77]
gi651494129	2.54	3	1	315	32.3	5.50	7.88	nicotinate-nucleotide pyrophosphorylase [Arthrobacter sp. H20]
gi759729243	1.06	4	1	1703	175.7	4.94	7.88	hypothetical protein, partial [Arthrobacter sp. UNC362MFTsu5.1]
gi737793059	8.71	1	1	264	28.7	9.23	7.88	IclR family transcriptional regulator [Arthrobacter nicotinovorans]
gi786027108	3.12	2	1	353	37.4	6.76	7.88	glycosyl transferase family 1 [Arthrobacter chlorophenolicus]
gi759712486	4.32	2	1	532	58.8	7.80	7.87	phospholipase D [Arthrobacter sp. 162MFSha1.1]
gi651502022	4.97	1	1	382	41.0	5.64	7.87	adenosine deaminase [Arthrobacter sp. 35W]
gi116610294	0.84	1	1	1197	122.1	7.18	7.85	ABC transporter related protein [Arthrobacter sp. FB24]
gi635351545	3.30	1	1	637	68.6	6.51	7.85	NADH:flavin oxidoreductase / NADH oxidase family protein [Arthrobacter siccitolerans]
gi742070006	10.06	5	2	358	39.1	5.92	7.85	N5,N10-methylenetetrahydromethanopterin reductase [Arthrobacter sp. MWB30]
gi908697824	5.32	2	1	376	39.6	5.57	7.85	GTPase [Arthrobacter sp. RIT-PI-e]
gi695270320	3.45	5	1	261	28.4	7.09	7.84	hypothetical protein [Arthrobacter globiformis]
gi654815214	3.95	2	1	354	38.1	7.65	7.83	dihydroorotate dehydrogenase 2 [Arthrobacter sp. PAO19]
gi517602891	4.31	1	1	348	37.0	6.60	7.83	hypothetical protein [Arthrobacter sp. 131MFCol6.1]
gi651492611	3.44	1	1	581	61.5	4.94	7.83	type IV secretion protein Rhs [Arthrobacter sp. H20]
gi636845902	23.15	1	1	108	11.2	9.72	7.82	hypothetical protein [Arthrobacter sp. TB 26]
gi919219140	1.68	1	1	955	101.1	5.22	7.82	hypothetical protein [Arthrobacter sp. YC-RL1]
gi916781908	2.51	1	1	479	51.1	6.81	7.82	hypothetical protein [Arthrobacter sp. 35W]
gi651500258	3.74	2	1	294	32.4	7.81	7.81	XRE family transcriptional regulator [Arthrobacter sp. 35W]
gi918269288	13.91	1	1	115	12.3	5.21	7.81	hypothetical protein AHiyo1_09310 [Arthrobacter sp. Hiyo1]
gi636843728	3.62	4	1	470	51.9	8.76	7.81	hypothetical protein [Arthrobacter sp. TB 26]
gi517610087	1.71	1	1	586	64.8	5.66	7.80	hypothetical protein [Arthrobacter sp. 161MFSha2.1]
gi759962312	5.92	3	1	304	31.8	5.03	7.79	prephenate dehydratase [Nocardia thailandica]
gi515766654	4.19	2	1	310	33.4	5.72	7.78	LysR family transcriptional regulator [Arthrobacter sp. M2012083]
gi723607855	4.24	2	1	495	51.4	5.02	7.77	peptidase M23 [Arthrobacter sp. PAMC25486]
gi517592305	1.00	1	1	802	88.5	6.93	7.77	hypothetical protein [Arthrobacter sp. 135MFCol5.1]
gi517605563	2.87	12	1	488	52.0	5.91	7.77	two-component system sensor histidine kinase [Arthrobacter sp. 131MFCol6.1]
gi823666791	1.89	1	1	741	78.8	7.55	7.74	hypothetical protein AA310_13715, partial [Arthrobacter sp. YC-RL1]
gi219861174	3.25	2	2	893	95.9	6.40	7.74	transcriptional regulator, LuxR family [Arthrobacter chlorophenolicus A6]
gi651496130	1.49	1	1	1614	179.7	5.22	7.74	glutamate dehydrogenase [Arthrobacter sp. H20]
gi640196635	4.99	2	1	401	40.2	5.12	7.73	glycerate kinase [Arthrobacter sp. 31Y]
gi937262221	1.08	1	1	1291	140.8	6.14	7.73	AAA family ATPase [Arthrobacter sp. Edens01]
gi910740864	8.49	1	1	106	11.2	8.13	7.73	50S ribosomal protein L11 [Arthrobacter sp. Hiyo4]

gi916324918	2.52	1	1	755	83.8	8.09	7.72	GTP pyrophosphokinase [Arthrobacter gangotriensis]
gi937259089	0.98	1	1	1118	116.9	6.81	7.72	hypothetical protein AO716_14225 [Arthrobacter sp. Edens01]
gi916781965	7.14	6	2	434	43.9	5.08	7.71	hypothetical protein [Arthrobacter sp. 35W]
gi917441835	1.05	6	1	1626	179.2	4.88	7.70	hypothetical protein [Arthrobacter albus]
gi359306548	10.34	2	1	145	15.8	5.30	7.69	hypothetical protein ARGLB_037_01050 [Arthrobacter globiformis NBRC 12137]
gi545109684	8.06	1	1	248	27.3	5.73	7.69	DNA-binding response regulator [Arthrobacter sp. AK-YN10]
gi759708689	7.22	1	1	291	30.3	7.94	7.67	ArsR family transcriptional regulator [Arthrobacter sp. 9MFCol3.1]
gi651429854	1.92	5	1	312	31.9	4.59	7.67	zinc-binding dehydrogenase [Arthrobacter sanguinis]
gi517593626	3.76	10	1	213	23.3	5.58	7.66	hypothetical protein [Arthrobacter sp. 135MFCol5.1]
gi489899647	1.31	1	1	837	89.9	5.85	7.66	nitrite reductase large subunit [Arthrobacter globiformis]
gi307745289	19.57	2	1	46	5.2	5.24	7.66	hypothetical protein AARI_20430 [Arthrobacter arilaitensis Re117]
gi910251947	12.62	3	2	325	34.3	11.05	7.65	short-chain dehydrogenase [Arthrobacter siccitolerans]
gi737787378	10.78	1	1	102	11.2	9.82	7.64	50S ribosomal protein L21 [Arthrobacter albus]
gi518312131	2.30	1	1	868	93.9	6.32	7.63	hypothetical protein [Arthrobacter sp. TB 23]
gi737804797	2.61	1	1	460	49.7	6.58	7.63	hypothetical protein [Arthrobacter sp. Br18]
gi651467158	2.08	1	1	721	78.6	6.61	7.63	transcription termination factor Rho [Arthrobacter sp. 35/47]
gi517593654	4.03	1	1	372	39.2	6.87	7.63	hypothetical protein [Arthrobacter sp. 135MFCol5.1]
gi403228653	2.05	2	1	537	57.9	5.68	7.62	putative regulatory protein [Arthrobacter sp. Rue61a]
gi517593198	6.29	2	2	461	52.3	5.54	7.62	glycine--tRNA ligase [Arthrobacter sp. 135MFCol5.1]
gi916357998	2.54	2	1	670	70.7	6.18	7.61	MULTISPECIES: ATP-dependent helicase [Arthrobacter]
gi307743859	0.90	4	1	1108	121.1	5.87	7.60	conserved hypothetical protein [Arthrobacter arilaitensis Re117]
gi476399402	12.08	1	1	149	15.7	8.91	7.60	50S ribosomal protein L9 [Arthrobacter crystallopoietes BAB-32]
gi916816358	3.78	2	1	503	53.6	5.39	7.59	hypothetical protein [Arthrobacter sp. MA-N2]
gi742756015	6.28	2	1	366	38.6	6.44	7.58	glycine/betaine ABC transporter ATPase [Arthrobacter phenanthrenivorans]
gi219858786	9.16	6	2	273	29.6	9.19	7.57	short-chain dehydrogenase/reductase SDR [Arthrobacter chlorophenolicus A6]
gi635350638	2.52	2	2	953	98.5	6.64	7.57	permease family protein [Arthrobacter siccitolerans]
gi674646399	1.75	1	1	798	85.2	6.81	7.56	UvrABC system protein A [Arthrobacter sp. 11W110_air]
gi910740677	23.17	4	1	82	9.3	10.35	7.56	probable transposase for insertion sequence element ISRM3-like [Arthrobacter sp. Hiyo4]
gi928488541	3.33	1	1	661	71.7	7.78	7.56	ABC transporter [Arthrobacter alpinus]
gi476402702	4.26	1	1	235	25.2	5.20	7.55	signal peptidase I [Arthrobacter crystallopoietes BAB-32]
gi939051583	4.78	2	1	230	24.2	8.97	7.54	hypothetical protein [Arthrobacter sp. JCM 19049]
gi116609868	2.44	2	1	492	53.6	4.97	7.54	adenosylhomocysteinase [Arthrobacter sp. FB24]
gi307745253	5.24	1	1	229	25.0	5.08	7.54	conserved hypothetical protein [Arthrobacter arilaitensis Re117]
gi674646766	5.00	9	1	340	34.8	4.01	7.54	NMT1/THI5 like protein [Arthrobacter sp. 11W110_air]
gi476398905	22.55	3	1	102	11.4	5.05	7.53	hypothetical protein D477_021043 [Arthrobacter crystallopoietes BAB-32]
gi723606516	2.49	1	1	522	55.9	4.97	7.51	anthranilate synthase, component I [Arthrobacter sp. PAMC25486]
gi517599073	14.75	6	2	183	19.9	9.96	7.50	hypothetical protein [Arthrobacter sp. 162MFSha1.1]
gi162954744	15.09	3	2	285	30.0	9.16	7.49	short chain dehydrogenase [Renibacterium salmoninarum ATCC 33209]
gi759730539	3.45	1	1	522	53.2	5.58	7.49	ferredoxin [Arthrobacter sp. L77]
gi930827610	5.27	5	2	512	55.4	7.88	7.48	hypothetical protein AOZ07_15860 [Arthrobacter arilaitensis]
gi927033877	2.91	2	1	585	63.4	5.38	7.48	sulfoacetaldehyde acetyltransferase [Arthrobacter sp. LS16]
gi476400809	7.37	1	1	312	33.9	10.30	7.48	polar amino acid ABC transporter [Arthrobacter crystallopoietes BAB-32]
gi651429520	3.64	1	1	412	42.1	4.55	7.47	cell division protein FtsZ [Arthrobacter sanguinis]
gi116610537	1.84	1	1	708	73.2	6.34	7.47	K+-transporting ATPase, B subunit [Arthrobacter sp. FB24]
gi189044289	11.20	3	1	125	14.1	10.56	7.46	RecName: Full=30S ribosomal protein S13
gi470221120	5.03	1	1	298	32.3	5.08	7.46	UTP-glucose-1-phosphate uridylyltransferase [Arthrobacter gangotriensis Lz1y]
gi927032947	2.85	6	1	738	82.8	6.01	7.46	polyphosphate kinase [Arthrobacter sp. LS16]
gi323470024	8.29	3	2	398	41.6	6.13	7.46	hypothetical protein Asphe3_25840 [Arthrobacter phenanthrenivorans Sphe3]
gi937258264	1.93	2	1	727	72.4	5.19	7.46	PTS lactose transporter subunit IIC [Arthrobacter sp. Edens01]
gi823667654	3.33	1	1	330	36.3	9.29	7.45	hypothetical protein AA310_14655 [Arthrobacter sp. YC-RL1]
gi640204533	8.29	1	1	181	19.5	7.97	7.44	hypothetical protein [Arthrobacter sp. 31Y]
gi219857693	8.96	3	2	346	37.6	7.01	7.44	oligopeptide/dipeptide ABC transporter, ATPase subunit [Arthrobacter chlorophenolicus A6]
gi910250713	5.12	2	1	469	50.4	6.05	7.44	glucose-1-phosphate adenylyltransferase [Arthrobacter siccitolerans]
gi651458056	1.23	1	1	649	71.8	5.08	7.42	peptidase M13 [Arthrobacter sp. 35/47]
gi742856637	3.79	1	1	264	28.6	5.19	7.42	recombinase RecR [Arthrobacter sp. W1]
gi914717881	1.51	1	1	1189	127.0	5.36	7.41	chromosome segregation protein SMC [Arthrobacter sp. ZBG10]
gi927296442	4.01	2	1	499	53.6	7.58	7.41	hypothetical protein AL755_15840 [Arthrobacter sp. ERGS1:01]
gi515764882	1.73	1	1	579	61.0	6.15	7.41	cyclic nucleotide-binding protein [Arthrobacter sp. M2012083]
gi636846281	2.65	1	1	603	64.6	5.15	7.41	proline--tRNA ligase [Arthrobacter sp. TB 26]

gi910250111	1.59	1	1	1072	111.0	5.97	7.41	hydrogenase expression protein [Arthrobacter siccitolerans]
gi919218885	7.66	12	1	235	24.4	6.21	7.39	MULTISPECIES: hypothetical protein [Arthrobacter]
gi636844188	4.75	1	1	400	43.9	6.54	7.38	acyl-CoA dehydrogenase [Arthrobacter sp. TB 26]
gi323471431	5.62	2	1	409	41.4	6.46	7.38	3-oxoacyl-(acyl-carrier-protein) synthase II (plasmid) [Arthrobacter phenanthrenivorans Sphe3]
gi307745648	4.68	2	1	385	40.8	5.52	7.37	cystathionine gamma-synthase [Arthrobacter arilaitensis Re117]
gi639130865	0.61	1	1	1139	119.8	5.03	7.36	MULTISPECIES: hypothetical protein [Arthrobacter]
gi307746367	6.67	1	1	135	15.0	10.81	7.36	putative very short patch repair endonuclease [Arthrobacter arilaitensis Re117]
gi937257906	7.22	1	1	180	20.8	5.35	7.36	hypothetical protein AO716_07050 [Arthrobacter sp. Edens01]
gi918267322	5.49	2	1	237	25.1	7.49	7.35	alpha/Beta hydrolase fold [Arthrobacter sp. Hiyo1]
gi359304065	2.88	2	1	312	34.5	7.14	7.35	putative LysR family transcriptional regulator [Arthrobacter globiformis NBRC 12137]
gi757625471	2.80	1	1	500	56.1	6.95	7.35	trehalose-phosphate synthase [Arthrobacter sp. SPG23]
gi219858146	3.43	2	1	350	38.1	6.61	7.34	NAD-dependent epimerase/dehydratase [Arthrobacter chlorophenolicus A6]
gi910739719	2.50	2	1	561	61.0	7.77	7.33	glycerol-3-phosphate dehydrogenase [Arthrobacter sp. Hiyo4]
gi916691815	6.03	2	1	315	35.6	9.80	7.33	hypothetical protein [Arthrobacter castelli]
gi737800851	1.72	2	1	523	58.2	6.18	7.33	copper oxidase [Arthrobacter castelli]
gi916691498	7.01	1	1	214	22.8	4.44	7.33	ATPase [Arthrobacter castelli]
gi910251299	1.35	3	1	1110	115.5	9.55	7.33	glycosyl transferase [Arthrobacter siccitolerans]
gi737811202	5.41	1	1	222	24.5	6.55	7.33	transcriptional regulator [Arthrobacter sp. 35/47]
gi737810157	3.61	1	1	499	52.9	8.21	7.32	methyltransferase [Arthrobacter sp. 35/47]
gi636844937	3.28	2	1	274	28.7	8.37	7.31	ATP synthase subunit delta [Arthrobacter sp. TB 26]
gi119947909	3.31	2	1	272	28.3	5.99	7.30	putative glycosyl transferase, group 1 family protein [Arthrobacter aurescens TC1]
gi939051617	14.29	6	1	119	12.2	5.01	7.29	hypothetical protein [Arthrobacter sp. JCM 19049]
gi930828077	4.69	2	1	426	44.0	9.01	7.29	C4-dicarboxylate transporter [Arthrobacter arilaitensis]
gi651437799	3.95	2	2	532	57.6	5.58	7.29	ABC transporter [Arthrobacter sp. H14]
gi786035308	11.33	3	1	203	22.2	4.17	7.28	ribosome maturation factor RimM [Arthrobacter chlorophenolicus]
gi939050222	4.49	1	1	401	44.0	8.82	7.28	hypothetical protein [Arthrobacter sp. JCM 19049]
gi654823491	0.88	2	1	907	95.9	6.48	7.27	hypothetical protein [Arthrobacter sp. I3]
gi908697697	6.27	4	1	335	35.2	5.50	7.27	hypothetical protein [Arthrobacter sp. RIT-PI-e]
gi823665684	1.12	2	1	890	97.3	5.25	7.27	hypothetical protein AA310_06900 [Arthrobacter sp. YC-RL1]
gi749402511	5.19	1	1	385	41.9	6.87	7.26	galactose-1-phosphate uridylyltransferase [Arthrobacter sp. AK-YN10]
gi119947828	3.80	3	1	368	38.6	6.19	7.26	putative myo-inositol 2-dehydrogenase [Arthrobacter aurescens TC1]
gi119951571	2.64	1	1	341	36.3	4.83	7.25	hypothetical protein AAur_pTC10275 (plasmid) [Arthrobacter aurescens TC1]
gi651466753	3.56	1	1	309	32.4	5.33	7.24	nicotinate-nucleotide pyrophosphorylase [Arthrobacter sp. 35/47]
gi914713438	6.93	1	1	202	21.9	8.32	7.23	TetR family transcriptional regulator [Arthrobacter sp. ZBG10]
gi917760194	1.84	1	1	544	57.9	6.80	7.23	hypothetical protein [Arthrobacter sp. L77]
gi651447330	9.15	3	1	164	17.8	7.05	7.23	CoA-binding protein [Arthrobacter nicotinovorans]
gi651480651	19.50	9	2	159	17.4	7.61	7.22	MarR family transcriptional regulator [Arthrobacter sp. Br18]
gi651440408	11.66	3	1	163	17.7	4.75	7.21	hypothetical protein [Arthrobacter sp. H14]
gi648575107	1.51	1	1	1058	116.3	5.60	7.21	cytochrome P450 [Arthrobacter sp. 131MFCol6.1]
gi759733891	3.18	1	1	471	53.3	6.04	7.20	sugar phosphotransferase [Arthrobacter sp. L77]
gi119950006	3.77	7	1	478	53.5	5.44	7.20	hypothetical protein AAur_3505 [Arthrobacter aurescens TC1]
gi927271281	4.80	1	1	229	24.4	7.40	7.19	hypothetical protein [Arthrobacter sp. LS16]
gi652424727	4.44	2	1	293	32.8	5.60	7.19	protein iolH [Arthrobacter castelli]
gi916781934	4.73	3	2	613	67.3	5.31	7.17	aspartyl-tRNA synthetase [Arthrobacter sp. 35W]
gi927296197	3.03	1	1	330	36.3	5.44	7.16	aminoglycoside resistance protein [Arthrobacter sp. ERGS1:01]
gi636844618	5.41	5	1	296	32.3	8.94	7.16	pseudouridine synthase [Arthrobacter sp. TB 26]
gi517601118	4.51	2	1	377	40.1	5.31	7.16	oxidoreductase [Arthrobacter sp. 162MFSha1.1]
gi910742220	5.56	5	1	288	31.5	7.12	7.15	pca regulon regulatory protein [Arthrobacter sp. Hiyo4]
gi119950385	3.50	2	1	343	36.7	6.14	7.15	putative transcriptional regulator, lacI family [Arthrobacter aurescens TC1]
gi359306171	5.06	5	1	257	28.1	6.58	7.14	hypothetical protein ARGLB_047_01190 [Arthrobacter globiformis NBRC 12137]
gi908697114	6.19	5	2	291	28.8	10.64	7.14	glycosyl transferase family 9 [Arthrobacter sp. RIT-PI-e]
gi928487887	4.70	3	2	575	60.1	10.65	7.14	DEAD/DEAH box helicase [Arthrobacter alpinus]
gi654827570	12.14	4	1	140	15.8	11.24	7.14	heat-shock protein [Arthrobacter sp. H5]
gi918269359	14.94	6	1	87	9.2	11.21	7.13	hypothetical protein AHiyo1_10100 [Arthrobacter sp. Hiyo1]
gi359305890	0.84	2	1	1186	120.9	10.56	7.13	putative ABC transporter permease/ATP-binding protein CydDC [Arthrobacter globiformis NBRC 12
gi916691819	6.27	3	2	399	41.5	5.33	7.12	cystathionine gamma-synthase [Arthrobacter castelli]
gi119949883	1.55	2	2	1480	159.5	5.96	7.12	putative FtsK/SpoIIIE family protein [Arthrobacter aurescens TC1]
gi823666831	1.48	2	1	540	60.8	5.82	7.12	biotin carboxylase [Arthrobacter sp. YC-RL1]
gi749401809	1.16	1	1	1294	134.0	5.27	7.12	fibronectin, partial [Arthrobacter sp. AK-YN10]

gi515764599	0.80	1	1	870	93.9	8.60	7.11	hypothetical protein [Arthrobacter sp. M2012083]
gi651491554	2.60	1	1	461	49.1	4.68	7.11	folylpolyglutamate synthase [Arthrobacter sp. H20]
gi307743685	1.03	1	1	1554	166.6	6.00	7.10	putative ATP-dependent DNA helicase [Arthrobacter arilaitensis Re117]
gi674645111	2.80	4	1	393	39.6	7.25	7.09	Riboflavin biosynthesis protein RibD [Arthrobacter sp. 11W110_air]
gi517591457	6.17	1	1	227	25.2	6.81	7.09	DNA-binding response regulator [Arthrobacter sp. 135MFCol5.1]
gi723607889	3.26	2	1	675	70.8	8.44	7.09	hypothetical protein ART_1666 [Arthrobacter sp. PAMC25486]
gi307743418	5.99	6	1	217	23.1	5.74	7.09	GntR-family transcriptional regulator [Arthrobacter arilaitensis Re117]
gi470217673	4.53	3	2	552	55.9	4.77	7.09	phosphoenolpyruvate-protein phosphotransferase [Arthrobacter gangotriensis Lz1y]
gi307746453	2.20	5	1	454	49.3	10.11	7.08	putative family 2 glycosyl transferase [Arthrobacter arilaitensis Re117]
gi908698295	2.26	2	1	707	74.7	5.39	7.08	3-hydroxyacyl-CoA dehydrogenase [Arthrobacter sp. RIT-PI-e]
gi786026240	6.34	4	1	205	21.5	10.65	7.06	hypothetical protein [Arthrobacter chlorophenolicus]
gi928488136	5.15	2	1	272	28.9	9.28	7.06	hypothetical protein AOC05_16010 [Arthrobacter alpinus]
gi757625105	9.66	2	1	207	21.8	5.24	7.05	NADPH-dependent FMN reductase [Arthrobacter sp. SPG23]
gi116608862	12.31	6	2	268	28.6	9.35	7.05	ABC transporter related protein [Arthrobacter sp. FB24]
gi910251429	1.40	1	1	1002	108.6	6.46	7.05	hypothetical protein [Arthrobacter siccitolerans]
gi928486178	2.76	4	1	907	98.1	6.02	7.04	hypothetical protein AOC05_02740 [Arthrobacter alpinus]
gi651429105	1.60	1	1	1567	164.1	9.80	7.04	hypothetical protein [Arthrobacter sanguinis]
gi517603547	5.42	2	1	332	34.7	9.80	7.04	hypothetical protein [Arthrobacter sp. 131MFCol6.1]
gi654811802	3.45	1	1	377	41.4	4.93	7.04	GCN5 family acetyltransferase [Arthrobacter sp. MA-N2]
gi910250916	1.72	1	1	1163	119.3	9.26	7.03	ABC transporter [Arthrobacter siccitolerans]
gi908696949	1.36	2	1	589	63.0	5.39	7.03	hypothetical protein [Arthrobacter sp. RIT-PI-e]
gi742072112	18.63	3	2	102	11.1	10.27	7.03	hypothetical protein ANMWB30_09470 [Arthrobacter sp. MWB30]
gi760112933	1.65	2	1	967	105.3	5.19	7.02	glycoside hydrolase [Arthrobacter chlorophenolicus]
gi651502890	1.95	1	1	616	67.1	6.18	7.02	capsule biosynthesis protein CapD [Arthrobacter sp. 35W]
gi359305033	5.10	1	1	431	48.5	5.38	7.02	putative oxidoreductase [Arthrobacter globiformis NBRC 12137]
gi910740820	6.30	4	1	127	13.7	11.40	7.01	50S ribosomal protein L18 [Arthrobacter sp. Hiyo4]
gi760166942	6.89	1	1	305	32.6	5.81	7.01	ribokinase [Arthrobacter crystallopoietes]
gi636843716	4.12	3	1	437	47.9	5.58	7.01	hypothetical protein [Arthrobacter sp. TB 26]
gi823667385	18.84	1	1	69	8.0	12.37	7.01	hypothetical protein AA310_17370, partial [Arthrobacter sp. YC-RL1]
gi917739780	5.11	5	1	411	44.4	5.10	7.01	aspartate aminotransferase [Arthrobacter sp. W1]
gi759735038	14.41	3	1	118	12.9	6.79	7.00	hypothetical protein [Arthrobacter sp. L77]
gi443482747	4.88	2	2	451	48.2	7.49	7.00	arabinose efflux permease family protein [Arthrobacter nitrophenolicus]
gi908642046	4.63	4	1	518	56.6	9.64	7.00	hypothetical protein [Arthrobacter phenanthrenivorans]
gi651443831	8.78	2	2	262	28.4	6.02	6.99	ABC transporter [Arthrobacter nicotinovorans]
gi674644399	2.23	1	1	358	40.5	9.11	6.99	FemAB family protein [Arthrobacter sp. 11W110_air]
gi116609579	1.40	1	1	860	91.1	6.21	6.98	excinuclease ABC, A subunit [Arthrobacter sp. FB24]
gi759734302	5.56	3	1	306	33.7	5.91	6.98	multidrug ABC transporter ATPase [Arthrobacter sp. L77]
gi654817782	5.58	2	1	412	44.7	5.29	6.98	hypothetical protein [Arthrobacter sp. UNC362MFTsu5.1]
gi759710723	1.98	1	1	1062	118.0	5.36	6.98	DEAD/DEAH box helicase [Arthrobacter sp. 135MFCol5.1]
gi930827219	2.69	1	1	782	84.4	7.17	6.97	hypothetical protein AOZ07_13650 [Arthrobacter arilaitensis]
gi515767214	5.28	4	1	360	40.3	4.91	6.96	hypothetical protein [Arthrobacter sp. M2012083]
gi910251571	3.76	4	1	479	50.7	5.64	6.96	NAD-dependent succinate-semialdehyde dehydrogenase [Arthrobacter siccitolerans]
gi517604194	1.08	5	1	742	80.7	5.49	6.95	ATPase AAA [Arthrobacter sp. 131MFCol6.1]
gi636845624	1.33	1	1	676	72.3	5.86	6.95	multidrug ABC transporter ATP-binding protein [Arthrobacter sp. TB 26]
gi219858662	1.81	3	1	775	84.5	5.57	6.95	oxidoreductase alpha (molybdopterin) subunit [Arthrobacter chlorophenolicus A6]
gi742070366	3.28	2	1	579	61.5	5.63	6.95	ATP/GTP-binding protein [Arthrobacter sp. MWB30]
gi930827980	6.32	1	1	380	41.1	9.79	6.94	fusaric acid resistance protein [Arthrobacter arilaitensis]
gi654813438	2.57	3	1	505	51.8	6.16	6.94	aminopeptidase A [Arthrobacter sp. MA-N2]
gi636843428	4.06	2	1	394	40.1	5.57	6.94	FAD/NAD(P)-binding oxidoreductase, partial [Arthrobacter sp. TB 26]
gi651491530	4.21	1	1	309	32.4	8.60	6.93	formamidopyrimidine-DNA glycosylase [Arthrobacter sp. H20]
gi517599220	1.80	1	1	500	53.9	6.80	6.93	hypothetical protein [Arthrobacter sp. 162MFSha1.1]
gi753938505	9.02	1	1	133	15.1	11.37	6.92	hypothetical protein [Arthrobacter phenanthrenivorans]
gi939050729	9.26	1	1	162	18.0	9.47	6.92	hypothetical protein [Arthrobacter sp. JCM 19049]
gi651439799	3.75	2	2	587	64.3	5.14	6.92	aspartyl-tRNA synthetase [Arthrobacter sp. H14]
gi674643936	1.34	1	1	1492	153.2	4.46	6.91	Endonuclease YhcR precursor [Arthrobacter sp. 11W110_air]
gi910283708	2.99	3	1	368	40.5	7.61	6.90	hypothetical protein [Arthrobacter sp. A3]
gi651434286	4.96	1	1	282	30.0	5.11	6.90	pyridoxal biosynthesis lyase PdxS [Arthrobacter sp. H41]
gi917442028	0.87	1	1	915	96.2	5.08	6.90	hypothetical protein [Arthrobacter albus]
gi517591329	2.41	2	1	457	49.9	7.21	6.90	cyclopropane-fatty-acyl-phospholipid synthase [Arthrobacter sp. 135MFCol5.1]

gi403231041	1.91	2	1	577	62.7	5.83	6.90	ABC transporter ATP-binding protein [Arthrobacter sp. Rue61a]
gi119950264	5.52	1	1	344	38.3	5.71	6.89	putative multidrug ABC transporter, ATP-binding protein [Arthrobacter aurescens TC1]
gi651463030	2.32	4	1	561	61.3	7.14	6.89	ATP-dependent DNA helicase PcrA [Arthrobacter sp. 35/47]
gi651435604	3.48	1	1	316	33.3	5.17	6.89	ABC transporter [Arthrobacter sp. H41]
gi737786841	3.33	1	1	270	28.9	5.22	6.88	ATP synthase subunit delta [Arthrobacter albus]
gi917441993	6.64	3	2	286	32.1	5.24	6.88	chromosome partitioning protein ParB [Arthrobacter albus]
gi916863396	3.22	2	1	311	33.4	9.38	6.88	metallophosphoesterase [Arthrobacter sp. 35/47]
gi651506641	3.40	2	1	235	25.6	6.20	6.87	GntR family transcriptional regulator [Arthrobacter sp. 35W]
gi910692799	9.64	1	1	197	21.2	9.85	6.87	dihydrolipoyllysine-residue acetyltransferase component of pyruvate dehydrogenase complex, part
gi919108297	3.29	1	1	456	47.2	5.24	6.86	protoporphyrinogen oxidase [Arthrobacter sp. IHBB 11108]
gi742754121	2.30	1	1	479	51.9	6.33	6.86	(dimethylallyl)adenosine tRNA methylthiotransferase [Arthrobacter phenanthrenivorans]
gi359306186	1.24	2	1	1051	110.0	4.97	6.86	putative glycosidase [Arthrobacter globiformis NBRC 12137]
gi914717100	2.97	1	1	538	56.1	10.70	6.86	ABC transporter [Arthrobacter sp. ZBG10]
gi636843684	3.05	5	1	622	65.9	7.21	6.86	AMP-dependent synthetase [Arthrobacter sp. TB 26]
gi651459322	2.15	2	1	326	33.7	5.54	6.86	hypothetical protein [Arthrobacter sp. 35/47]
gi765008507	9.84	2	2	315	32.2	5.12	6.84	glycine/betaine ABC transporter substrate-binding protein [Arthrobacter sp. A3]
gi307743478	6.71	3	1	149	15.7	8.22	6.83	AsnC/Lrp-family transcriptional regulator [Arthrobacter arilaitensis Re117]
gi654823577	2.59	3	1	502	54.1	7.43	6.83	hypothetical protein [Arthrobacter sp. I3]
gi910740689	3.98	2	1	377	40.8	5.01	6.83	GMP synthase [glutamine-hydrolyzing] [Arthrobacter sp. Hiyo4]
gi917739523	3.33	5	1	390	42.7	5.47	6.82	hypothetical protein [Arthrobacter sp. W1]
gi910746224	7.69	2	1	273	28.5	5.34	6.82	putative monooxygenase YxeK [Arthrobacter sp. Hiyo8]
gi518313829	5.08	4	1	492	52.7	5.14	6.81	MULTISPECIES: hypothetical protein [Arthrobacter]
gi545109521	9.06	1	1	265	27.8	5.07	6.81	succinyl-CoA--3-ketoacid-CoA transferase [Arthrobacter sp. AK-YN10]
gi823668704	5.73	2	1	279	30.7	5.02	6.81	hypothetical protein AA310_00090 [Arthrobacter sp. YC-RL1]
gi119950615	2.09	1	1	478	54.2	5.05	6.80	putative glycosyl hydrolases family 32 protein [Arthrobacter aurescens TC1]
gi757624199	5.88	2	1	323	34.7	6.01	6.80	5,10-methylenetetrahydrofolate reductase [Arthrobacter sp. SPG23]
gi443483272	2.46	1	1	814	87.5	5.63	6.80	response regulator containing a CheY-like receiver domain and an HTH DNA-binding domain-conta
gi737802872	4.85	2	1	433	47.9	5.29	6.79	hypothetical protein, partial [Arthrobacter castelli]
gi470216563	1.61	2	1	496	51.9	5.01	6.79	succinate-semialdehyde dehydrogenase [Arthrobacter gangotriensis Lz1y]
gi917021972	3.16	1	1	506	54.8	5.22	6.77	ATPase [Arthrobacter sp. UNC362MFTsu5.1]
gi162952720	2.47	2	1	486	52.4	8.51	6.76	phosphate regulon sensor protein [Renibacterium salmoninarum ATCC 33209]
gi910740994	4.88	2	1	410	45.4	9.74	6.76	putative lambdoid prophage Rac integrase [Arthrobacter sp. Hiyo4]
gi517610163	2.26	1	1	310	33.9	9.52	6.76	hypothetical protein [Arthrobacter sp. 161MFSha2.1]
gi916870072	9.42	2	1	191	20.5	6.34	6.76	hypothetical protein [Arthrobacter sp. Br18]
gi162955217	2.46	2	1	488	53.0	4.88	6.76	adenosylhomocysteinase [Renibacterium salmoninarum ATCC 33209]
gi489900701	3.24	4	1	463	51.2	5.14	6.75	two-component system sensor histidine kinase [Arthrobacter globiformis]
gi162955208	2.38	2	1	589	66.4	9.77	6.75	decaprenyl-phosphate-mannose--protein mannosyltransferase [Renibacterium salmoninarum ATCC
gi927270102	9.86	1	1	71	7.8	8.76	6.75	hypothetical protein [Arthrobacter sp. LS16]
gi651439807	3.22	3	1	745	81.5	5.29	6.75	ATPase AAA [Arthrobacter sp. H14]
gi767256812	3.94	2	1	457	49.7	7.53	6.74	hypothetical protein UM93_02675 [Arthrobacter sp. IHBB 11108]
gi651447410	2.96	4	1	473	51.9	5.41	6.74	diguanylate phosphodiesterase [Arthrobacter nicotinovorans]
gi918268572	15.09	2	1	106	10.8	7.59	6.73	2-succinyl-5-enolpyruvyl-6-hydroxy-3-cyclohexene-1-carboxylate synthase [Arthrobacter sp. Hiyo1
gi723606833	3.73	1	1	241	26.9	5.22	6.73	hypothetical protein ART_0610 [Arthrobacter sp. PAMC25486]
gi651443289	5.08	3	1	256	28.6	8.35	6.73	endonuclease III [Arthrobacter sp. 9MFCol3.1]
gi928542246	4.37	1	1	481	52.8	5.16	6.73	portal protein [Arthrobacter phage Brent]
gi654819760	6.42	1	1	327	33.5	10.49	6.72	ABC transporter permease [Arthrobacter sp. UNC362MFTsu5.1]
gi542107235	3.61	1	1	582	63.4	9.07	6.72	hypothetical protein M707_18145 [Arthrobacter sp. AK-YN10]
gi918449462	2.07	2	1	581	63.5	5.22	6.72	arginine--tRNA ligase [Arthrobacter sp. SPG23]
gi769939789	9.87	3	2	314	33.7	7.28	6.72	hypothetical protein [Arthrobacter sp. IHBB 11108]
gi749401240	3.48	8	1	517	58.9	5.91	6.72	hypothetical protein M707_26470, partial [Arthrobacter sp. AK-YN10]
gi476400766	7.50	3	1	160	16.6	5.15	6.72	hypothetical protein D477_012051 [Arthrobacter crystallopoietes BAB-32]
gi910748773	3.13	2	1	415	45.9	9.61	6.72	GTP pyrophosphokinase [Arthrobacter sp. Hiyo8]
gi759704592	3.10	1	1	355	37.0	9.25	6.71	hypothetical protein [Arthrobacter globiformis]
gi918267149	3.27	1	1	581	59.9	6.49	6.71	probable copper-exporting P-type ATPase V [Arthrobacter sp. Hiyo1]
gi307745579	8.47	1	1	236	24.5	10.04	6.71	30S ribosomal protein S5 [Arthrobacter arilaitensis Re117]
gi937256401	4.36	2	1	505	54.2	4.81	6.70	oxidoreductase [Arthrobacter sp. Edens01]
gi640202141	0.98	1	1	2038	210.1	4.97	6.70	hypothetical protein [Arthrobacter sp. 31Y]
gi928487039	3.40	2	1	353	36.7	6.54	6.70	3-isopropylmalate dehydrogenase [Arthrobacter alpinus]
gi917407320	3.03	2	1	396	41.2	9.92	6.69	hypothetical protein [Arthrobacter nitrophenolicus]

gi737804784	3.05	3	1	328	35.5	9.80	6.69	methicillin resistance protein [Arthrobacter sp. Br18]
gi119951673	3.90	2	1	436	48.8	9.77	6.69	IS1380 family transposase (plasmid) [Arthrobacter aurescens TC1]
gi757622543	2.21	1	1	452	47.2	6.40	6.68	histidine kinase [Arthrobacter sp. SPG23]
gi786026497	3.85	2	1	338	35.2	5.33	6.68	hypothetical protein [Arthrobacter chlorophenolicus]
gi916259919	0.49	1	1	3080	321.7	4.56	6.68	hypothetical protein, partial [Arthrobacter sp. TB 23]
gi917022397	5.95	2	1	336	35.6	5.26	6.68	aryldialkylphosphatase [Arthrobacter sp. UNC362MFTsu5.1]
gi723610558	15.25	7	1	118	12.7	7.27	6.67	Transcriptional regulator, GntR family [Arthrobacter sp. PAMC25486]
gi403231902	8.79	2	2	364	39.0	5.97	6.67	hypothetical protein ARUE_232p01150 (plasmid) [Arthrobacter sp. Rue61a]
gi470216257	2.56	12	1	703	74.8	5.02	6.67	transketolase [Arthrobacter gangotriensis Lz1y]
gi759712277	4.10	2	1	415	43.4	8.09	6.66	two-component system sensor histidine kinase [Arthrobacter sp. 162MFSha1.1]
gi737786780	1.33	1	1	675	71.6	10.65	6.66	hypothetical protein [Arthrobacter albus]
gi916863381	5.91	2	1	254	27.8	5.91	6.66	GlcNAc-PI de-N-acetylase [Arthrobacter sp. 35/47]
gi757624374	4.19	5	1	334	36.3	5.26	6.66	flavodoxin [Arthrobacter sp. SPG23]
gi470216503	14.05	4	1	121	13.4	5.60	6.66	Hpt domain protein [Arthrobacter gangotriensis Lz1y]
gi928485980	4.55	3	1	330	36.0	5.36	6.66	hypothetical protein AOC05_01410 [Arthrobacter alpinus]
gi640193758	3.75	1	1	320	33.7	7.56	6.65	hypothetical protein [Arthrobacter sp. 31Y]
gi219857742	5.63	4	1	231	25.8	7.15	6.65	transcriptional regulator, GntR family [Arthrobacter chlorophenolicus A6]
gi517607330	2.43	3	1	412	44.2	6.77	6.65	dephospho-CoA kinase [Arthrobacter sp. 161MFSha2.1]
gi928542296	4.08	2	1	319	35.8	5.43	6.64	hypothetical protein SEA_BRENT_53 [Arthrobacter phage Brent]
gi916834713	4.52	4	1	310	32.1	9.57	6.64	hypothetical protein [Arthrobacter sp. H14]
gi542109329	1.97	2	1	913	101.8	5.20	6.64	preprotein translocase subunit SecA [Arthrobacter sp. AK-YN10]
gi359304601	7.88	1	1	165	18.0	6.20	6.64	putative MarR family transcriptional regulator [Arthrobacter globiformis NBRC 12137]
gi119947449	2.10	3	1	523	56.2	7.96	6.63	Na+/H+ antiporter [Arthrobacter aurescens TC1]
gi753932119	1.81	3	1	882	97.5	5.74	6.63	hypothetical protein [Arthrobacter arilaitensis]
gi759725502	4.23	2	1	449	47.9	4.75	6.62	kynureninase [Arthrobacter sp. I3]
gi517604278	4.30	2	1	442	47.0	4.77	6.62	kynureninase [Arthrobacter sp. 131MFCol6.1]
gi654826271	8.04	1	1	199	23.0	9.96	6.62	membrane protein [Arthrobacter sp. H5]
gi759733607	2.17	2	1	508	52.2	5.03	6.62	hypothetical protein [Arthrobacter sp. L77]
gi767259044	4.77	1	1	398	42.8	8.56	6.61	DNA processing protein DprA [Arthrobacter sp. IHBB 11108]
gi737789394	7.08	1	1	212	23.8	9.95	6.61	translation initiation factor IF-3 [Arthrobacter albus]
gi651444445	10.48	3	2	353	37.0	4.94	6.61	3-oxoacyl-ACP synthase [Arthrobacter nicotinovorans]
gi723607909	0.82	1	1	1468	149.6	7.39	6.60	hypothetical protein ART_1686 [Arthrobacter sp. PAMC25486]
gi162955635	4.65	1	1	409	43.0	5.34	6.60	3-ketoacyl-CoA thiolase [Renibacterium salmoninarum ATCC 33209]
gi651455215	7.14	3	2	322	35.6	4.97	6.60	hypothetical protein [Arthrobacter nicotinovorans]
gi654818982	2.63	5	1	419	46.1	10.01	6.60	hypothetical protein [Arthrobacter sp. UNC362MFTsu5.1]
gi635352150	7.34	1	1	259	29.8	9.29	6.59	ABC transporter family protein [Arthrobacter siccitolerans]
gi910283811	3.26	1	1	522	57.2	10.71	6.59	hypothetical protein [Arthrobacter sp. A3]
gi910250475	1.85	5	1	809	91.3	6.10	6.59	phosphoketolase [Arthrobacter siccitolerans]
gi930828168	5.46	2	1	458	47.8	5.27	6.58	hypothetical protein AOZ07_11020 [Arthrobacter arilaitensis]
gi651435294	2.92	2	1	411	42.7	6.20	6.58	threonine dehydratase [Arthrobacter sp. H41]
gi651442322	7.75	1	1	271	29.5	5.45	6.58	ubiquinone biosynthesis methyltransferase UbiE [Arthrobacter sp. 9MFCol3.1]
gi651494736	1.75	1	1	800	87.5	5.41	6.58	phosphoenolpyruvate synthase [Arthrobacter sp. H20]
gi359303909	4.20	2	1	333	32.9	5.03	6.58	glycerate kinase [Arthrobacter globiformis NBRC 12137]
gi927294101	0.79	1	1	1137	116.8	6.76	6.57	hypothetical protein AL755_08750 [Arthrobacter sp. ERGS1:01]
gi648573264	19.83	4	1	116	12.7	8.59	6.57	hypothetical protein [Arthrobacter sp. 135MFCol5.1]
gi119950295	3.86	2	2	880	95.4	5.15	6.57	DNA polymerase I [Arthrobacter aurescens TC1]
gi937262654	3.49	1	1	401	41.2	9.96	6.56	MFS transporter [Arthrobacter sp. Edens01]
gi654825836	3.22	2	1	404	43.0	5.00	6.56	S-adenosylmethionine synthetase [Arthrobacter sp. H5]
gi255103417	2.60	2	1	385	40.1	6.92	6.56	HNH nuclease-like protein [Arthrobacter sp. 32c]
gi403229491	3.48	2	1	632	67.3	4.79	6.55	chaperone protein Dank [Arthrobacter sp. Rue61a]
gi518311499	2.68	1	1	336	36.2	5.74	6.54	MULTISPECIES: hypothetical protein [Arthrobacter]
gi517603980	2.41	2	1	580	62.8	6.47	6.54	multidrug ABC transporter ATP-binding protein [Arthrobacter sp. 131MFCol6.1]
gi162954137	6.13	1	1	326	35.4	7.33	6.54	GTP-binding protein [Renibacterium salmoninarum ATCC 33209]
gi764161690	24.32	6	1	74	8.0	6.54	6.53	hypothetical protein ArV1_089 [Arthrobacter phage vB_ArtM-ArV1]
gi910740136	26.58	4	1	79	8.8	4.83	6.53	D-alanine--D-alanine ligase [Arthrobacter sp. Hiyo4]
gi742856755	8.53	1	1	258	27.5	5.26	6.53	D-beta-D-heptose 1-phosphate adenosyltransferase [Arthrobacter sp. W1]
gi323470123	3.36	2	1	446	48.2	8.18	6.53	Exodeoxyribonuclease VII large subunit [Arthrobacter phenanthrenivorans Sphe3]
gi759703955	2.73	3	1	807	85.4	9.31	6.52	hypothetical protein, partial [Arthrobacter globiformis]
gi674645399	1.70	3	1	589	62.3	5.35	6.52	Protein translocase subunit SecD [Arthrobacter sp. 11W110_air]

gi908698065	4.44	1	1	270	28.3	8.53	6.52	ABC transporter [Arthrobacter sp. RIT-PI-e]
gi916691310	5.07	5	2	730	78.7	5.60	6.52	hypothetical protein [Arthrobacter castelli]
gi723608339	15.29	1	1	85	9.6	10.71	6.51	50S ribosomal protein L16 [Arthrobacter sp. PAMC25486]
gi654827609	0.65	1	1	1222	134.7	5.47	6.50	glycogen branching protein [Arthrobacter sp. H5]
gi757624245	9.09	2	1	143	16.4	5.02	6.50	ATPase [Arthrobacter sp. SPG23]
gi307743604	4.91	2	1	224	24.2	5.20	6.50	two-component system response regulator [Arthrobacter arilaitensis Re117]
gi914716987	3.90	4	1	308	32.9	9.74	6.50	recombinase XerC [Arthrobacter sp. ZBG10]
gi551255566	9.68	2	1	186	20.8	6.30	6.50	MarR family transcriptional regulator [Arthrobacter sp. PAO19]
gi742856826	8.02	1	1	212	23.1	9.26	6.50	hypothetical protein [Arthrobacter sp. W1]
gi917759878	1.83	4	1	982	105.8	9.28	6.50	hypothetical protein [Arthrobacter sp. L77]
gi652425848	7.76	1	1	245	26.2	6.55	6.50	uridylate kinase [Arthrobacter castelli]
gi759772657	2.78	1	1	431	45.2	5.73	6.49	acetyl-CoA acetyltransferase [Arthrobacter sp. SPG23]
gi823667377	3.64	2	1	412	45.7	7.66	6.49	hypothetical protein AA310_17330 [Arthrobacter sp. YC-RL1]
gi476398982	3.17	1	1	663	72.3	4.89	6.48	ABC transporter, partial [Arthrobacter crystallopoietes BAB-32]
gi737788233	1.37	1	1	657	73.1	5.34	6.48	DNA gyrase subunit B [Arthrobacter albus]
gi651490999	10.75	1	1	186	20.4	4.98	6.48	oligoribonuclease [Arthrobacter sp. H20]
gi219858589	7.46	2	1	134	14.6	5.08	6.48	4-oxalocrotonate tautomerase [Arthrobacter chlorophenolicus A6]
gi937257995	1.37	2	1	879	94.1	5.68	6.47	hypothetical protein AO716_07635 [Arthrobacter sp. Edens01]
gi742757520	2.55	2	1	432	47.3	6.58	6.46	cyclopropane-fatty-acyl-phospholipid synthase [Arthrobacter phenanthrenivorans]
gi651500864	4.49	2	1	312	33.1	5.69	6.46	aldo/keto reductase [Arthrobacter sp. 35W]
gi757624419	4.35	1	1	460	50.7	6.84	6.46	mRNA 3'-end processing factor [Arthrobacter sp. SPG23]
gi323469031	2.00	1	1	550	57.4	5.19	6.45	UDP-N-acetylmuramoylalanyl-D-glutamate--2,6-diaminopimelate ligase [Arthrobacter phenanthren
gi162954511	9.49	2	1	137	14.5	7.23	6.45	hypothetical protein RSal33209_2295 [Renibacterium salmoninarum ATCC 33209]
gi470216679	6.49	4	1	231	25.0	12.16	6.45	hypothetical protein ADIAG_03294 [Arthrobacter gangotriensis Lz1y]
gi937258921	8.56	1	1	222	23.0	4.60	6.45	hypothetical protein AO716_13255 [Arthrobacter sp. Edens01]
gi742071944	1.81	1	1	1435	152.0	4.98	6.45	hypothetical protein ANMWB30_14570 [Arthrobacter sp. MWB30]
gi767257978	2.66	1	1	414	43.6	7.91	6.44	siroheme synthase CysG [Arthrobacter sp. IHBB 11108]
gi914713872	4.28	2	1	421	45.7	5.60	6.44	XRE family transcriptional regulator [Arthrobacter sp. ZBG10]
gi737789513	1.66	1	1	724	80.6	5.26	6.44	ribonucleotide-diphosphate reductase subunit alpha [Arthrobacter albus]
gi765006852	4.23	1	1	355	36.7	5.19	6.43	dipeptide epimerase [Arthrobacter sp. A3]
gi651485724	2.79	1	1	574	62.6	6.83	6.43	glycerol-3-phosphate dehydrogenase [Arthrobacter sp. Br18]
gi654825408	5.86	2	2	461	52.3	5.47	6.43	glycine--tRNA ligase [Arthrobacter sp. I3]
gi908697779	2.10	1	1	903	100.8	5.29	6.42	preprotein translocase subunit SecA [Arthrobacter sp. RIT-PI-e]
gi910252037	4.21	4	2	689	69.9	6.09	6.42	PTS lactose transporter subunit IIC [Arthrobacter siccitolerans]
gi916870246	1.94	1	1	464	50.4	7.64	6.42	hypothetical protein [Arthrobacter sp. Br18]
gi359304698	2.71	2	2	516	55.6	9.80	6.42	hypothetical protein ARGLB_080_00110 [Arthrobacter globiformis NBRC 12137]
gi765006892	2.49	3	1	281	31.5	9.55	6.42	sugar ABC transporter permease [Arthrobacter sp. A3]
gi786034641	3.19	1	1	627	66.2	6.92	6.42	pectin esterase [Arthrobacter chlorophenolicus]
gi517598626	2.14	2	1	561	60.1	6.02	6.41	alkaline phosphatase [Arthrobacter sp. 162MFSha1.1]
gi654827178	2.34	5	1	512	52.0	9.92	6.41	lipid II flippase MurJ [Arthrobacter sp. H5]
gi651430497	0.88	2	1	1254	133.5	6.54	6.41	hypothetical protein [Arthrobacter sanguinis]
gi648574769	4.60	2	1	543	58.6	6.10	6.41	ATPase [Arthrobacter sp. 131MFCol6.1]
gi654823862	2.09	1	1	669	73.1	7.81	6.41	excinuclease ABC subunit C [Arthrobacter sp. I3]
gi917021963	7.49	1	1	227	22.8	5.29	6.41	keto-deoxy-phosphogluconate aldolase [Arthrobacter sp. UNC362MFTsu5.1]
gi786034067	0.77	1	1	1163	124.0	5.88	6.40	1-pyrroline-5-carboxylate dehydrogenase [Arthrobacter chlorophenolicus]
gi759766412	11.41	1	1	184	20.3	6.02	6.39	GNAT family N-acetyltransferase [Arthrobacter gangotriensis]
gi359303525	3.86	2	1	466	50.2	4.86	6.39	putative serine/threonine protein kinase [Arthrobacter globiformis NBRC 12137]
gi910742815	6.70	2	1	224	24.6	7.36	6.39	FAD dependent oxidoreductase [Arthrobacter sp. Hiyo8]
gi916781602	2.24	1	1	490	52.1	6.93	6.38	hypothetical protein [Arthrobacter sp. 35W]
gi723607282	1.97	2	1	456	47.3	5.57	6.38	UDP-glucose 6-dehydrogenase [Arthrobacter sp. PAMC25486]
gi737764858	4.17	1	1	336	35.5	5.14	6.38	MULTISPECIES: alcohol dehydrogenase [Arthrobacter]
gi759739659	3.32	1	1	452	49.8	5.24	6.38	hypothetical protein [Arthrobacter phenanthrenivorans]
gi654813538	1.24	3	1	809	87.7	4.97	6.37	phosphoenolpyruvate synthase [Arthrobacter sp. MA-N2]
gi908699531	3.18	1	1	346	36.5	5.41	6.37	ABC transporter [Arthrobacter sp. RIT-PI-e]
gi674644640	4.53	1	1	265	28.6	11.40	6.37	RDD family protein [Arthrobacter sp. 11W110_air]
gi737810694	1.94	2	1	875	93.2	5.03	6.37	nitrite reductase large subunit [Arthrobacter sp. 35/47]
gi651462314	4.76	1	1	294	32.1	5.31	6.36	LysR family transcriptional regulator [Arthrobacter sp. 35/47]
gi916834703	9.24	1	1	184	19.9	5.81	6.36	hypothetical protein, partial [Arthrobacter sp. H14]
gi542109922	4.81	2	1	187	20.9	6.54	6.36	MarR family transcriptional regulator [Arthrobacter sp. AK-YN10]

gi916692283	3.35	1	1	597	66.2	7.02	6.36	hypothetical protein [Arthrobacter castelli]
gi914717222	1.27	1	1	866	93.6	4.96	6.35	aminopeptidase [Arthrobacter sp. ZBG10]
gi767256868	1.28	1	1	1174	126.7	5.34	6.35	peptidase S41 [Arthrobacter sp. IHBB 11108]
gi654817252	6.03	1	1	232	25.7	4.97	6.35	hypothetical protein [Arthrobacter sp. UNC362MFTsu5.1]
gi651439464	3.22	1	1	311	34.4	9.74	6.35	hypothetical protein [Arthrobacter sp. H14]
gi517598979	2.89	3	1	346	38.8	5.24	6.35	glyceraldehyde 3-phosphate reductase [Arthrobacter sp. 162MFSha1.1]
gi517590187	2.89	7	1	346	38.8	5.17	6.35	glyceraldehyde 3-phosphate reductase [Arthrobacter sp. 135MFCol5.1]
gi908699133	2.34	1	1	642	70.8	6.37	6.35	DNA primase [Arthrobacter sp. RIT-PI-e]
gi674646607	5.82	3	1	189	20.3	5.26	6.35	Bacterial regulatory proteins, tetR family [Arthrobacter sp. 11W110_air]
gi640196841	3.98	1	1	452	49.2	4.74	6.34	SAM-dependent methyltransferase [Arthrobacter sp. 31Y]
gi470221272	2.34	1	1	856	91.9	5.21	6.34	class III stress response-related ATPase [Arthrobacter gangotriensis Lz1y]
gi823668473	14.63	4	1	41	4.9	6.67	6.34	transposase, partial [Arthrobacter sp. YC-RL1]
gi919219087	1.68	1	1	1074	119.0	5.17	6.34	hypothetical protein [Arthrobacter sp. YC-RL1]
gi116611798	4.12	1	1	364	38.8	5.33	6.33	transcriptional regulator, LacI family [Arthrobacter sp. FB24]
gi517603031	2.69	1	1	335	34.7	9.42	6.33	two-component system sensor histidine kinase [Arthrobacter sp. 131MFCol6.1]
gi651429248	6.16	2	1	146	16.1	6.43	6.33	hypothetical protein [Arthrobacter sanguinis]
gi742858540	3.16	1	1	380	41.7	8.43	6.33	GDP-mannose-dependent alpha-mannosyltransferase [Arthrobacter sp. W1]
gi910695253	3.44	1	1	378	40.8	7.83	6.33	putative binding protein BRA0748/BS1330_II0741 [Arthrobacter sp. Hiyo6]
gi910737849	9.29	1	1	269	27.0	5.38	6.33	uncharacterized oxidoreductase Rv1144/MT1177 [Arthrobacter sp. Hiyo4]
gi476399858	4.49	1	1	401	42.1	4.72	6.33	signal recognition particle-docking protein FtsY [Arthrobacter crystallopoietes BAB-32]
gi674645097	5.73	1	1	192	20.6	6.57	6.32	HTH-type transcriptional repressor KstR2 [Arthrobacter sp. 11W110_air]
gi759728675	6.19	3	1	291	31.9	8.97	6.32	peptide ABC transporter ATP-binding protein [Arthrobacter sp. UNC362MFTsu5.1]
gi767258999	1.46	1	1	755	84.6	8.60	6.32	GTP pyrophosphokinase [Arthrobacter sp. IHBB 11108]
gi651439483	10.41	2	1	221	24.2	5.40	6.31	NUDIX domain-containing protein [Arthrobacter sp. H14]
gi651494352	2.53	2	1	513	55.0	10.07	6.31	3-methyladenine DNA glycosylase [Arthrobacter sp. H20]
gi470216774	3.92	1	1	383	40.7	5.90	6.31	acetate kinase [Arthrobacter gangotriensis Lz1y]
gi760124212	8.00	1	1	175	19.4	8.68	6.31	hypothetical protein [Arthrobacter aurescens]
gi939036722	15.71	2	1	70	7.7	6.52	6.31	hypothetical protein [Arthrobacter nitroguajacolicus]
gi651452001	3.93	2	1	610	64.0	5.66	6.31	long-chain fatty acid--CoA ligase [Arthrobacter nicotinovorans]
gi737787168	3.41	2	1	381	41.6	5.97	6.31	hypothetical protein [Arthrobacter albus]
gi910746887	4.85	3	1	206	22.4	11.49	6.31	type II secretion system protein [Arthrobacter sp. Hiyo8]
gi443482276	4.97	1	1	382	40.5	7.80	6.30	UDP-N-acetylenolpyruvoylglucosamine reductase [Arthrobacter nitrophenolicus]
gi916863442	1.10	1	1	997	106.6	6.21	6.30	FAD-linked oxidase [Arthrobacter sp. 35/47]
gi914716104	2.31	3	1	607	66.4	5.57	6.30	glycerophosphodiester phosphodiesterase [Arthrobacter sp. ZBG10]
gi542108054	2.78	1	1	539	55.9	8.43	6.30	hypothetical protein M707_14250 [Arthrobacter sp. AK-YN10]
gi517604316	7.00	1	1	343	34.9	8.94	6.30	hypothetical protein [Arthrobacter sp. 131MFCol6.1]
gi652424520	5.34	4	1	281	30.0	4.92	6.30	aldolase [Arthrobacter castelli]
gi517590015	12.12	3	1	165	17.8	11.53	6.30	hypothetical protein [Arthrobacter sp. 135MFCol5.1]
gi517608793	2.33	1	1	558	57.6	5.59	6.29	acetolactate synthase [Arthrobacter sp. 161MFSha2.1]
gi308229520	1.90	1	1	527	61.0	9.25	6.29	AciI [Arthrobacter citreus]
gi517599458	3.02	1	1	562	57.4	8.92	6.29	hypothetical protein [Arthrobacter sp. 162MFSha1.1]
gi908698698	5.72	1	1	402	38.9	4.97	6.29	hypothetical protein [Arthrobacter sp. RIT-PI-e]
gi910692721	4.06	2	1	320	34.6	5.31	6.29	uncharacterized oxidoreductase YuiH, partial [Arthrobacter sp. Hiyo6]
gi737802276	2.75	1	1	726	79.7	5.34	6.29	NAD-dependent DNA ligase LigA [Arthrobacter castelli]
gi910737978	14.63	2	1	164	17.5	11.11	6.28	hypothetical protein AHiyo4_08000 [Arthrobacter sp. Hiyo4]
gi918449310	1.06	2	2	1795	198.5	6.83	6.28	hypothetical protein [Arthrobacter sp. SPG23]
gi651457217	4.70	1	1	447	47.5	5.90	6.28	UDP-N-acetylglucosamine 1-carboxyvinyltransferase [Arthrobacter sp. 35/47]
gi737781111	7.94	3	1	214	23.1	10.32	6.27	CDP-diacylglycerol--glycerol-3-phosphate 3-phosphatidyltransferase [Arthrobacter sp. 35W]
gi517602622	6.67	2	1	120	13.2	6.57	6.27	hypothetical protein [Arthrobacter sp. 131MFCol6.1]
gi517602509	3.72	1	1	403	42.6	5.47	6.27	hypothetical protein [Arthrobacter sp. 131MFCol6.1]
gi927294255	7.02	1	1	342	36.2	5.67	6.27	epimerase [Arthrobacter sp. ERGS1:01]
gi219859359	7.32	1	1	205	22.7	11.63	6.27	hypothetical protein Achl_1718 [Arthrobacter chlorophenolicus A6]
gi443480575	7.76	1	1	219	23.9	5.63	6.27	GntR family transcriptional regulator [Arthrobacter nitrophenolicus]
gi489894054	2.33	2	1	472	51.7	5.76	6.26	FMNH2-dependent monooxygenase [Arthrobacter globiformis]
gi759736215	1.20	1	1	1002	111.1	6.47	6.26	glutamine-synthetase [Arthrobacter sp. L77]
gi918423074	2.36	1	1	763	76.9	7.77	6.26	hypothetical protein [Arthrobacter sp. AK-YN10]
gi916813681	2.09	1	1	382	40.5	8.07	6.26	hypothetical protein [Arthrobacter nicotinovorans]
gi517591979	5.54	4	1	271	28.2	5.62	6.25	hypothetical protein [Arthrobacter sp. 135MFCol5.1]
gi742070290	8.01	1	1	312	33.8	9.94	6.25	hypothetical protein ANMWB30_29750 [Arthrobacter sp. MWB30]

gi760124246	1.91	1	1	734	76.3	5.33	6.25	hypothetical protein [Arthrobacter aurescens]
gi651431081	0.92	2	1	1199	130.5	5.07	6.25	chromosome segregation protein SMC [Arthrobacter sanguinis]
gi162953969	8.87	1	1	203	22.6	10.10	6.25	ribosomal-protein-S5-alanine acetyltransferase [Renibacterium salmoninarum ATCC 33209]
gi162955752	14.10	2	1	78	9.0	12.48	6.24	LSU ribosomal protein L34P [Renibacterium salmoninarum ATCC 33209]
gi914716844	3.28	1	1	579	61.9	9.52	6.24	hypothetical protein [Arthrobacter sp. ZBG10]
gi918265749	12.61	1	1	119	13.7	10.21	6.24	hypothetical protein AHiyo1_42460 [Arthrobacter sp. Hiyo1]
gi162953815	3.21	1	1	591	65.7	4.84	6.24	metallopeptidase, M13 family [Renibacterium salmoninarum ATCC 33209]
gi767257056	1.16	1	1	948	104.6	6.84	6.24	RNA helicase [Arthrobacter sp. IHBB 11108]
gi914715242	7.84	1	1	255	27.4	5.49	6.24	DeoR faimly transcriptional regulator [Arthrobacter sp. ZBG10]
gi359306310	3.98	1	1	251	27.1	4.86	6.23	hypothetical protein ARGLB_039_00200 [Arthrobacter globiformis NBRC 12137]
gi654813064	10.53	1	1	228	24.0	9.98	6.23	septation ring formation regulator EzrA [Arthrobacter sp. MA-N2]
gi651439212	20.31	3	1	64	7.1	4.77	6.23	hypothetical protein [Arthrobacter sp. H14]
gi742861262	7.50	1	1	280	30.7	5.14	6.23	oxidoreductase [Arthrobacter sp. W1]
gi927292727	2.81	1	1	392	42.3	5.76	6.23	toxic anion resistance protein (plasmid) [Arthrobacter sp. ERGS1:01]
gi674645371	10.05	1	1	189	20.1	7.71	6.22	Bifunctional protein pyrR [Arthrobacter sp. 11W110_air]
gi640195934	2.71	1	1	480	51.5	6.86	6.22	PucR family transcriptional regulator [Arthrobacter sp. 31Y]
gi759767544	2.55	2	1	666	74.3	6.42	6.21	cellulose synthase [Arthrobacter sp. SPG23]
gi651443555	3.18	3	1	377	41.1	5.39	6.21	acyl-CoA dehydrogenase [Arthrobacter sp. 9MFCol3.1]
gi651504417	2.87	1	1	523	55.7	6.73	6.21	sodium:proton antiporter [Arthrobacter sp. 35W]
gi916259772	6.01	1	1	383	41.4	9.19	6.21	DNA polymerase IV [Arthrobacter sp. TB 23]
gi759765729	3.29	2	1	426	44.8	7.36	6.21	hypothetical protein [Arthrobacter gangotriensis]
gi742072618	6.19	1	1	226	25.2	5.67	6.20	GntR family transcriptional regulator [Arthrobacter sp. MWB30]
gi916820557	1.05	2	1	954	103.3	5.05	6.20	hypothetical protein [Arthrobacter sp. H20]
gi635350605	2.52	3	1	873	95.7	5.86	6.20	alpha-glucan phosphorylases family protein [Arthrobacter siccitolerans]
gi757626140	1.41	1	1	853	89.7	6.27	6.20	haloacid dehalogenase [Arthrobacter sp. SPG23]
gi916692353	5.37	1	1	242	27.6	6.55	6.20	hypothetical protein [Arthrobacter castelli]
gi651431241	6.07	3	1	280	31.7	9.58	6.20	hypothetical protein [Arthrobacter sanguinis]
gi517592039	1.29	1	1	775	84.2	6.09	6.20	hypothetical protein [Arthrobacter sp. 135MFCol5.1]
gi640204531	3.80	2	1	263	28.9	5.50	6.20	hypothetical protein [Arthrobacter sp. 31Y]
gi515766463	4.86	1	1	391	42.6	6.98	6.19	hypothetical protein [Arthrobacter sp. M2012083]
gi757623644	3.31	2	1	574	61.0	8.29	6.19	AMP-dependent synthetase [Arthrobacter sp. SPG23]
gi651432906	8.06	1	1	248	26.7	7.12	6.19	hypothetical protein [Arthrobacter sp. H41]
gi636844930	2.92	2	1	479	52.4	5.52	6.19	phosphomethylpyrimidine synthase, partial [Arthrobacter sp. TB 26]
gi674646020	11.05	6	1	190	21.0	4.83	6.19	Adenylate kinase [Arthrobacter sp. 11W110_air]
gi759764399	1.49	3	1	537	57.9	4.91	6.18	SAM-dependent methyltransferase [Arthrobacter gangotriensis]
gi723608258	6.42	1	1	374	39.7	5.82	6.18	putative inosine 5-monophosphate dehydrogenase [Arthrobacter sp. PAMC25486]
gi170783487	2.99	1	1	335	37.5	6.25	6.18	sugar phosphate isomerase/epimerase (plasmid) [Arthrobacter sp. AK-1]
gi723607065	3.35	1	1	388	41.0	7.09	6.18	hypothetical protein ART_0842 [Arthrobacter sp. PAMC25486]
gi470215793	5.00	1	1	320	33.0	4.59	6.17	FKBP-type 22 kDa peptidyl-prolyl cis-trans isomerase [Arthrobacter gangotriensis Lz1y]
gi927292803	5.99	1	1	284	30.7	6.60	6.17	glutamine amidotransferase (plasmid) [Arthrobacter sp. ERGS1:01]
gi823667519	8.70	7	1	253	28.7	6.71	6.17	GntR family transcriptional regulator [Arthrobacter sp. YC-RL1]
gi695208025	3.05	3	1	393	43.3	6.48	6.17	hypothetical transcriptional regulator (plasmid) [Arthrobacter nicotinovorans]
gi517603360	2.77	2	1	289	31.0	9.51	6.17	protease [Arthrobacter sp. 131MFCol6.1]
gi749401543	5.63	1	1	302	32.2	9.38	6.16	FAD-binding dehydrogenase, partial [Arthrobacter sp. AK-YN10]
gi928488690	1.82	5	1	549	58.9	6.49	6.15	hypothetical protein AOC05_05945 [Arthrobacter alpinus]
gi470215998	1.85	1	1	596	64.7	9.77	6.15	Type IV secretory pathway, VirD4 component [Arthrobacter gangotriensis Lz1y]
gi651460113	3.85	1	1	390	42.3	5.31	6.15	pyruvate dehydrogenase [Arthrobacter sp. 35/47]
gi651444846	2.67	1	1	450	47.0	6.16	6.15	acetyl-CoA acetyltransferase [Arthrobacter nicotinovorans]
gi823668203	2.99	1	1	401	42.0	5.45	6.15	beta-ketoadipyl CoA thiolase [Arthrobacter sp. YC-RL1]
gi916863569	3.59	2	1	334	35.4	5.15	6.15	sorbitol dehydrogenase [Arthrobacter sp. 35/47]
gi908696978	3.37	1	1	563	62.3	9.74	6.14	hypothetical protein [Arthrobacter sp. RIT-PI-e]
gi517601220	4.21	3	1	309	34.5	5.39	6.14	hypothetical protein [Arthrobacter sp. 162MFSha1.1]
gi517591641	7.60	1	1	263	27.7	6.70	6.14	cytochrome C [Arthrobacter sp. 135MFCol5.1]
gi759733266	12.99	2	1	177	17.8	5.47	6.14	2-C-methyl-D-erythritol 2,4-cyclodiphosphate synthase [Arthrobacter sp. L77]
gi116611561	2.61	1	1	345	38.4	5.29	6.13	aldo/keto reductase [Arthrobacter sp. FB24]
gi648224376	1.29	1	1	1629	179.9	5.76	6.12	glutamate dehydrogenase [Arthrobacter sp. M2012083]
gi930825452	7.77	1	1	283	30.8	6.60	6.12	hypothetical protein AOZ07_03515 [Arthrobacter arilaitensis]
gi723609417	4.62	1	1	519	54.7	5.17	6.12	hypothetical protein ART_3194 [Arthrobacter sp. PAMC25486]
gi723607913	1.05	3	1	1624	180.4	5.15	6.12	NAD-specific glutamate dehydrogenase [Arthrobacter sp. PAMC25486]

gi674644309	6.52	1	1	368	42.2	6.13	6.11	Region found in RelA / SpoT proteins [Arthrobacter sp. 11W110_air]
gi654826412	5.44	2	1	331	36.5	5.12	6.11	xylose isomerase [Arthrobacter sp. H5]
gi759730871	18.32	1	1	191	21.2	6.14	6.11	deoxycytidine triphosphate deaminase [Arthrobacter sp. L77]
gi908698631	3.78	2	1	291	29.8	4.87	6.11	inositol monophosphatase [Arthrobacter sp. RIT-PI-e]
gi403231994	3.57	1	1	616	63.8	9.19	6.11	putative membrane protein (plasmid) [Arthrobacter sp. Rue61a]
gi359305020	1.37	4	1	952	97.6	4.82	6.10	hypothetical protein ARGLB_075_00010 [Arthrobacter globiformis NBRC 12137]
gi753932350	8.17	1	1	208	22.6	6.34	6.10	glutamine amidotransferase [Arthrobacter arilaitensis]
gi737809772	4.32	2	1	301	32.7	10.10	6.10	hypothetical protein [Arthrobacter sp. 35/47]
gi551256406	2.31	3	1	520	55.5	9.07	6.10	signal recognition particle protein [Arthrobacter sp. PAO19]
gi939037486	8.71	1	1	241	26.1	7.75	6.10	hypothetical protein [Arthrobacter nitroguajacolicus]
gi910742093	6.83	1	1	278	30.1	12.34	6.10	transport system permease protein [Arthrobacter sp. Hiyo4]
gi723608175	5.85	1	1	171	18.6	6.20	6.10	Transcriptional regulator, AsnC family [Arthrobacter sp. PAMC25486]
gi753940667	5.26	1	1	266	28.3	11.25	6.09	cobalt transporter [Arthrobacter phenanthrenivorans]
gi551256537	2.44	1	1	614	65.7	7.09	6.09	multidrug ABC transporter ATPase [Arthrobacter sp. PAO19]
gi742753209	4.18	1	1	287	29.4	5.21	6.09	fructose-bisphosphate aldolase [Arthrobacter phenanthrenivorans]
gi219860499	4.55	1	1	242	25.0	5.43	6.09	ANTAR domain protein with unknown sensor [Arthrobacter chlorophenolicus A6]
gi767257339	2.86	1	1	595	65.1	5.35	6.08	phosphomethylpyrimidine synthase [Arthrobacter sp. IHBB 11108]
gi742071439	4.82	1	1	353	35.3	5.35	6.08	hypothetical protein ANMWB30_15580 [Arthrobacter sp. MWB30]
gi476402465	1.27	1	1	942	105.1	8.06	6.08	superfamily II RNA helicase [Arthrobacter crystallopoietes BAB-32]
gi652424216	2.29	1	1	1006	110.2	6.15	6.08	glutamine-synthetase [Arthrobacter castelli]
gi651429178	5.34	5	1	393	42.2	8.70	6.08	hypothetical protein [Arthrobacter sanguinis]
gi651438776	6.45	3	1	310	32.6	4.91	6.07	hydroxymethylglutaryl-CoA lyase [Arthrobacter sp. H14]
gi651437903	7.21	1	1	222	23.7	9.85	6.07	50S ribosomal protein L4 [Arthrobacter sp. H14]
gi927294459	2.00	2	1	400	41.7	6.05	6.07	dephospho-CoA kinase [Arthrobacter sp. ERGS1:01]
gi917013346	8.89	1	1	225	24.2	9.69	6.06	hypothetical protein [Arthrobacter sanguinis]
gi119951121	4.32	1	1	324	36.1	9.79	6.06	conserved hypothetical protein [Arthrobacter aurescens TC1]
gi927294987	5.84	1	1	154	16.9	7.14	6.06	MarR family transcriptional regulator [Arthrobacter sp. ERGS1:01]
gi476401005	2.58	2	1	387	41.8	6.55	6.05	hypothetical protein D477_010931 [Arthrobacter crystallopoietes BAB-32]
gi651430019	5.30	1	1	283	31.5	5.05	6.05	hypothetical protein [Arthrobacter sanguinis]
gi917759835	4.31	1	1	348	35.6	11.08	6.05	hypothetical protein [Arthrobacter sp. L77]
gi639130479	6.02	1	1	349	37.9	8.94	6.05	ribosome small subunit-dependent GTPase [Arthrobacter sp. CAL618]
gi823666358	4.81	2	1	437	46.2	5.16	6.04	glutamyl-tRNA reductase [Arthrobacter sp. YC-RL1]
gi545109582	5.78	1	1	346	37.0	5.83	6.04	DNA recombination/repair protein RecA [Arthrobacter sp. AK-YN10]
gi359304186	5.99	1	1	384	40.7	5.25	6.04	NADP-dependent alcohol dehydrogenase [Arthrobacter globiformis NBRC 12137]
gi742072402	21.15	1	1	52	5.4	12.02	6.04	hypothetical protein ANMWB30_07730 [Arthrobacter sp. MWB30]
gi517603993	2.20	6	1	681	75.8	6.06	6.04	alpha-1,4-glucan--maltose-1-phosphate maltosyltransferase [Arthrobacter sp. 131MFCol6.1]
gi410689844	2.00	1	1	550	60.5	9.72	6.03	hypothetical protein (plasmid) [Arthrobacter sp. J3-53]
gi927296186	4.04	2	1	272	29.3	9.42	6.02	hypothetical protein AL755_07000 [Arthrobacter sp. ERGS1:01]
gi119951230	2.31	1	1	910	98.5	6.39	6.01	putative transcriptional regulator, LuxR family [Arthrobacter aurescens TC1]
gi639130882	1.39	1	1	793	87.9	5.22	6.01	type I restriction-modification protein subunit M [Arthrobacter sp. CAL618]
gi927296335	3.83	1	1	574	61.9	5.87	6.01	FAD-dependent oxidoreductase [Arthrobacter sp. ERGS1:01]
gi551254827	6.56	1	1	259	27.6	5.43	6.01	excisionase [Arthrobacter sp. PAO19]
gi652423331	23.47	3	1	98	10.4	4.75	6.01	molecular chaperone GroES [Arthrobacter castelli]
gi930826867	6.60	1	1	197	20.2	4.77	6.01	hypothetical protein AOZ07_11635 [Arthrobacter arilaitensis]
gi652422725	5.14	1	1	428	45.8	8.46	6.00	hypothetical protein [Arthrobacter castelli]
gi470215782	4.48	2	1	424	45.8	4.87	6.00	cysteinyl-tRNA synthetase [Arthrobacter gangotriensis Lz1y]
gi908698701	17.50	1	1	120	12.5	11.58	6.00	hypothetical protein [Arthrobacter sp. RIT-PI-e]
gi737787456	3.41	1	1	469	50.7	6.02	6.00	hypothetical protein [Arthrobacter albus]
gi470220784	2.34	1	1	427	47.4	6.01	5.99	citrate synthase II [Arthrobacter gangotriensis Lz1y]
gi640196346	6.32	1	1	269	28.6	7.55	5.99	methyltransferase type 12 [Arthrobacter sp. 31Y]
gi551255395	7.84	2	1	255	28.8	8.13	5.99	hypothetical protein [Arthrobacter sp. PAO19]
gi742755266	3.38	3	1	681	73.8	6.42	5.99	cold-shock protein [Arthrobacter phenanthrenivorans]
gi640201609	3.78	1	1	423	47.2	4.91	5.98	hypothetical protein [Arthrobacter sp. 31Y]
gi323467627	3.58	1	1	391	39.3	11.71	5.98	arabinose efflux permease family protein [Arthrobacter phenanthrenivorans Sphe3]
gi470217179	2.90	1	1	655	70.3	6.43	5.98	deoxyxylulose-5-phosphate synthase [Arthrobacter gangotriensis Lz1y]
gi928487155	4.64	2	1	345	36.2	5.24	5.98	glycerol-3-phosphate dehydrogenase [Arthrobacter alpinus]
gi219859694	2.25	1	1	445	45.6	6.58	5.98	membrane-bound transglycosylase [Arthrobacter chlorophenolicus A6]
gi116611797	3.17	1	1	473	52.4	6.39	5.97	carbohydrate ABC transporter substrate-binding protein, CUT1 family [Arthrobacter sp. FB24]
gi927295899	2.95	1	1	643	67.8	4.83	5.97	hypothetical protein AL755_20780 [Arthrobacter sp. ERGS1:01]

gi648573970	2.46	3	1	528	55.5	6.21	5.97	oxidoreductase [Arthrobacter sp. 162MFSha1.1]
gi917021998	4.17	5	1	551	57.0	11.30	5.97	hypothetical protein [Arthrobacter sp. UNC362MFTsu5.1]
gi307746316	3.01	1	1	632	70.3	5.15	5.97	putative aromatic-ring hydroxylase [Arthrobacter arilaitensis Re117]
gi690773989	6.45	1	1	310	32.4	4.61	5.97	hypothetical protein HMPREF2128_01785 [Arthrobacter albus DNF00011]
gi910739043	1.99	2	1	602	64.1	6.24	5.97	long-chain-fatty-acid--CoA ligase FadD15 [Arthrobacter sp. Hiyo4]
gi640203190	5.16	2	1	368	41.0	6.54	5.96	hypothetical protein [Arthrobacter sp. 31Y]
gi518311713	8.55	2	1	304	31.2	6.57	5.96	hypothetical protein [Arthrobacter sp. TB 23]
gi742068702	19.51	2	1	41	4.6	10.78	5.96	hypothetical protein ANMWB30_43230 [Arthrobacter sp. MWB30]
gi742858572	9.14	3	1	175	19.2	4.31	5.96	RNA-binding protein [Arthrobacter sp. W1]
gi916820122	6.02	3	1	349	37.8	6.86	5.96	hypothetical protein [Arthrobacter sp. H20]
gi443481334	5.92	1	1	287	30.2	6.02	5.95	pentapeptide repeat-containing protein [Arthrobacter nitrophenolicus]
gi652423084	5.15	2	1	272	30.3	4.97	5.95	hypothetical protein [Arthrobacter castelli]
gi910738690	14.67	1	1	150	16.1	9.79	5.95	hypothetical protein AHiyo4_15120 [Arthrobacter sp. Hiyo4]
gi908698146	9.68	1	1	186	20.5	7.03	5.95	transcriptional regulator [Arthrobacter sp. RIT-PI-e]
gi403231948	1.30	1	1	617	67.2	7.40	5.94	putative ABC transporter ATP-binding protein (plasmid) [Arthrobacter sp. Rue61a]
gi906448091	7.39	1	1	230	24.2	5.15	5.94	hypothetical protein AC792_03630 [Arthrobacter sp. RIT-PI-e]
gi476402310	5.05	1	1	535	56.4	4.84	5.94	chaperonin GroEL [Arthrobacter crystallopoietes BAB-32]
gi674644661	1.78	1	1	897	95.0	6.64	5.93	Bacterial regulatory proteins, luxR family [Arthrobacter sp. 11W110_air]
gi939037370	14.96	1	1	127	13.3	8.21	5.93	hypothetical protein [Arthrobacter nitroguajacolicus]
gi917013474	5.35	2	1	187	21.1	6.57	5.93	hypothetical protein [Arthrobacter sanguinis]
gi910248828	3.35	1	1	567	62.7	6.11	5.93	hypothetical protein [Arthrobacter siccitolerans]
gi723607742	7.76	5	1	335	34.3	6.01	5.93	hypothetical protein ART_1519 [Arthrobacter sp. PAMC25486]
gi908698087	8.31	3	1	313	31.4	4.64	5.93	ribokinase [Arthrobacter sp. RIT-PI-e]
gi914715222	2.31	1	1	692	77.7	6.38	5.92	hypothetical protein [Arthrobacter sp. ZBG10]
gi359307188	5.63	3	1	284	30.8	7.64	5.92	putative oxidoreductase [Arthrobacter globiformis NBRC 12137]
gi517593021	7.76	1	1	219	24.0	5.66	5.92	GntR family transcriptional regulator [Arthrobacter sp. 135MFCol5.1]
gi910248720	3.24	1	1	309	34.2	9.01	5.92	transporter [Arthrobacter siccitolerans]
gi910697359	13.95	1	1	86	9.2	9.52	5.91	ABC transporter, substrate binding protein [Arthrobacter sp. Hiyo6]
gi162953491	3.12	9	1	385	40.3	11.69	5.91	efflux ABC transporter, permease protein [Renibacterium salmoninarum ATCC 33209]
gi723608419	1.78	1	1	845	89.5	6.07	5.91	osmosensitive K+ channel signal transduction histidine kinase [Arthrobacter sp. PAMC25486]
gi908696887	3.76	1	1	559	58.9	8.63	5.91	hypothetical protein [Arthrobacter sp. RIT-PI-e]
gi914717747	2.10	1	1	999	107.8	5.02	5.90	hypothetical protein [Arthrobacter sp. ZBG10]
gi919219009	3.12	2	1	353	40.3	8.53	5.90	MULTISPECIES: hypothetical protein [Arthrobacter]
gi930828004	3.55	1	1	564	60.6	6.20	5.90	histidine kinase [Arthrobacter arilaitensis]
gi916872203	3.11	1	1	322	36.2	9.85	5.89	hypothetical protein [Arthrobacter sp. H5]
gi759734146	1.81	1	1	607	62.4	4.55	5.89	dihydrolipoamide acetyltransferase [Arthrobacter sp. L77]
gi937262142	1.98	1	1	1009	108.1	6.20	5.89	3-phosphoshikimate 1-carboxyvinyltransferase [Arthrobacter sp. Edens01]
gi786030935	1.41	1	1	710	72.6	11.81	5.89	competence protein ComEC [Arthrobacter chlorophenolicus]
gi759746328	1.86	1	1	538	55.4	5.86	5.89	DNA-directed RNA polymerase sigma-70 factor [Arthrobacter sp. 31Y]
gi939050379	3.46	4	1	318	36.4	4.93	5.88	hypothetical protein [Arthrobacter sp. JCM 19049]
gi517600796	2.73	1	1	477	50.2	6.86	5.88	hypothetical protein [Arthrobacter sp. 162MFSha1.1]
gi908698260	1.95	1	1	411	42.5	5.26	5.87	phosphopantothenoylcysteine decarboxylase [Arthrobacter sp. RIT-PI-e]
gi651480202	6.54	1	1	382	41.7	5.25	5.87	pyruvate dehydrogenase [Arthrobacter sp. Br18]
gi757624802	4.55	1	1	198	22.2	8.15	5.87	hypothetical protein TV39_09010 [Arthrobacter sp. SPG23]
gi916324923	2.26	2	1	576	63.9	6.62	5.87	hypothetical protein [Arthrobacter gangotriensis]
gi635351546	1.55	1	1	386	43.0	5.39	5.87	luciferase-like monooxygenase family protein [Arthrobacter siccitolerans]
gi651431275	4.15	2	1	434	47.5	6.65	5.87	exodeoxyribonuclease VII large subunit [Arthrobacter sanguinis]
gi910252413	4.20	1	1	381	41.3	5.38	5.86	hypothetical protein [Arthrobacter siccitolerans]
gi652424277	5.42	1	1	369	40.9	6.16	5.86	hypothetical protein [Arthrobacter castelli]
gi640199795	8.13	3	1	246	26.3	5.15	5.86	GntR family transcriptional regulator [Arthrobacter sp. 31Y]
gi765006466	5.13	1	1	390	42.9	6.60	5.86	hypothetical protein [Arthrobacter sp. A3]
gi654815581	0.86	1	1	927	98.8	5.48	5.85	oxidoreductase [Arthrobacter sp. PAO19]
gi937259340	3.31	3	1	363	39.4	8.63	5.85	ACP synthase [Arthrobacter sp. Edens01]
gi651494767	3.88	1	1	206	22.6	7.03	5.85	RNA polymerase sigma factor SigK [Arthrobacter sp. H20]
gi910696228	10.17	18	1	118	12.3	5.48	5.85	hypothetical protein AHiyo6_13490 [Arthrobacter sp. Hiyo6]
gi918265230	7.05	1	1	156	17.1	12.21	5.85	hypothetical protein AHiyo1_49520 [Arthrobacter sp. Hiyo1]
gi910697004	4.85	1	1	227	24.0	9.77	5.84	1-acyl-sn-glycerol-3-phosphate acyltransferase [Arthrobacter sp. Hiyo6]
gi723608345	6.79	1	1	280	29.8	9.00	5.84	50S ribosomal protein L4/L1 family protein [Arthrobacter sp. PAMC25486]
gi470220189	5.90	1	1	271	30.9	5.33	5.83	thymidylate synthase [Arthrobacter gangotriensis Lz1y]

gi651436973	1.99	2	1	704	75.9	6.19	5.83	ATP-dependent DNA helicase RecQ [Arthrobacter sp. H41]
gi914715152	4.63	1	1	389	41.0	9.36	5.83	hypothetical protein [Arthrobacter sp. ZBG10]
gi403231548	7.01	1	1	214	22.5	4.86	5.83	putative transcriptional regulator, TetR family [Arthrobacter sp. Rue61a]
gi759734547	5.02	1	1	458	46.6	9.07	5.83	3-ketoacyl-ACP reductase [Arthrobacter sp. L77]
gi652423383	5.26	2	1	361	39.0	4.83	5.83	4-hydroxy-3-methylbut-2-enyl diphosphate reductase [Arthrobacter castelli]
gi674645044	3.85	1	1	442	47.9	4.93	5.82	hypothetical protein BN1051_01212 [Arthrobacter sp. 11W110_air]
gi919219049	7.55	1	1	278	31.2	9.38	5.82	hypothetical protein [Arthrobacter sp. YC-RL1]
gi116610275	4.50	2	1	222	24.0	5.63	5.81	lipoate-protein ligase B [Arthrobacter sp. FB24]
gi765010063	1.91	1	1	1150	122.1	5.16	5.81	1-pyrroline-5-carboxylate dehydrogenase [Arthrobacter sp. A3]
gi908691000	7.85	1	1	242	25.4	9.76	5.81	hypothetical protein [Arthrobacter sp. H41]
gi651475330	4.52	1	1	398	43.3	6.43	5.81	carboxylate--amine ligase [Arthrobacter nicotinovorans]
gi737790423	12.82	2	1	156	17.2	10.30	5.80	30S ribosomal protein S7 [Arthrobacter albus]
gi823668430	7.04	1	1	213	23.0	7.44	5.80	methyltransferase [Arthrobacter sp. YC-RL1]
gi640196913	4.55	1	1	198	21.7	8.10	5.80	thymidylate kinase [Arthrobacter sp. 31Y]
gi916869533	3.47	1	1	404	43.4	10.24	5.80	transposase [Arthrobacter sp. Br18]
gi470217502	9.28	2	1	194	20.9	9.69	5.80	serine O-acetyltransferase [Arthrobacter gangotriensis Lz1y]
gi723608515	2.27	1	1	440	47.3	8.57	5.80	monooxygenase, FAD-binding protein [Arthrobacter sp. PAMC25486]
gi403230426	3.32	1	1	361	39.1	5.20	5.79	4-hydroxy-3-methylbut-2-enyl diphosphate reductase IspH [Arthrobacter sp. Rue61a]
gi918017324	1.10	1	1	729	78.4	7.44	5.79	hypothetical protein [Arthrobacter sp. FB24]
gi910249897	1.63	1	1	492	52.8	5.27	5.79	ribonuclease II [Arthrobacter siccitolerans]
gi928488306	5.33	1	1	169	18.1	5.19	5.79	hypothetical protein AOC05_17155 [Arthrobacter alpinus]
gi476399340	4.20	4	1	500	54.9	7.03	5.79	nucleoside-diphosphate sugar epimerase [Arthrobacter crystallopoietes BAB-32]
gi786033428	2.51	3	1	439	48.6	5.53	5.79	alpha/beta hydrolase [Arthrobacter chlorophenolicus]
gi658508775	2.08	1	1	577	62.4	6.70	5.78	ATP-dependent DNA helicase RecQ [Arthrobacter sp. TB 26]
gi551255190	4.26	1	1	376	41.9	6.60	5.78	23S rRNA methyltransferase [Arthrobacter sp. PAO19]
gi651430437	1.64	1	1	488	53.3	5.03	5.78	ribonuclease II [Arthrobacter sanguinis]
gi403228160	6.75	2	1	311	33.4	5.22	5.77	HTH-type transcriptional repressor PurR [Arthrobacter sp. Rue61a]
gi640199257	8.00	1	1	175	19.2	6.20	5.77	hypothetical protein [Arthrobacter sp. 31Y]
gi162953585	3.17	1	1	378	39.5	8.51	5.77	glycosyl hydrolase family 3 N terminal domain protein [Renibacterium salmoninarum ATCC 33209]
gi652424573	2.00	2	1	998	111.4	5.31	5.77	alpha-mannosidase [Arthrobacter castelli]
gi786028237	3.58	1	1	307	33.9	9.22	5.76	ABC transporter permease [Arthrobacter chlorophenolicus]
gi651430001	4.42	3	1	339	36.1	5.07	5.76	fructose-bisphosphate aldolase [Arthrobacter sanguinis]
gi307745584	8.77	1	1	114	12.2	9.94	5.76	50S ribosomal protein L24 [Arthrobacter arilaitensis Re117]
gi786032796	1.33	2	1	828	91.3	6.84	5.76	ATP-dependent DNA ligase [Arthrobacter chlorophenolicus]
gi542108085	9.80	1	1	153	16.9	4.83	5.76	hypothetical protein M707_13210 [Arthrobacter sp. AK-YN10]
gi937262367	3.87	1	1	284	30.5	9.96	5.76	type II secretion system protein F [Arthrobacter sp. Edens01]
gi927296011	12.75	3	1	149	15.6	9.61	5.76	50S ribosomal protein L9 [Arthrobacter sp. ERGS1:01]
gi737789562	3.73	1	1	402	42.4	5.31	5.75	signal recognition particle-docking protein FtsY [Arthrobacter albus]
gi551254149	2.97	1	1	640	71.1	5.02	5.75	tyramine oxidase [Arthrobacter sp. PAO19]
gi674646898	5.20	1	1	423	43.6	5.40	5.75	Allantoate amidohydrolase [Arthrobacter sp. 11W110_air]
gi939037259	6.79	3	1	280	31.5	9.80	5.75	hypothetical protein [Arthrobacter nitroguajacolicus]
gi910743180	7.25	2	1	193	20.8	6.13	5.75	nickel-binding periplasmic protein [Arthrobacter sp. Hiyo8]
gi737796655	1.92	2	1	416	42.4	4.77	5.74	phosphoglycerate kinase [Arthrobacter sp. H20]
gi359303579	4.45	1	1	292	32.4	4.77	5.74	hypothetical protein ARGLB_113_00560 [Arthrobacter globiformis NBRC 12137]
gi765008725	3.26	2	1	582	61.1	5.94	5.74	pyruvate dehydrogenase [Arthrobacter sp. A3]
gi517608893	9.09	6	1	209	21.2	4.86	5.73	hypothetical protein [Arthrobacter sp. 161MFSha2.1]
gi674644943	4.21	2	1	214	23.1	5.36	5.73	Trans-aconitate 2-methyltransferase [Arthrobacter sp. 11W110_air]
gi916820398	2.27	3	1	750	80.3	7.36	5.72	hypothetical protein [Arthrobacter sp. H20]
gi651430170	2.46	1	1	407	43.7	5.44	5.72	cytosine deaminase [Arthrobacter sanguinis]
gi937256519	1.54	1	1	974	103.9	5.19	5.72	hypothetical protein AO716_15815 [Arthrobacter sp. Edens01]
gi753931349	9.09	7	1	121	13.1	8.51	5.71	single-stranded DNA-binding protein [Arthrobacter arilaitensis]
gi323470499	1.48	2	1	948	100.3	5.99	5.71	FAD/FMN-dependent dehydrogenase [Arthrobacter phenanthrenivorans Sphe3]
gi910747574	11.63	2	1	172	18.6	5.96	5.71	hypothetical protein AHiyo8_52760 [Arthrobacter sp. Hiyo8]
gi651499819	6.42	1	1	358	38.2	6.23	5.71	alkene reductase [Arthrobacter sp. 35W]
gi403311678	2.79	2	1	215	22.8	7.47	5.71	putative MobA-like protein (plasmid) [Arthrobacter sp. Rue61a]
gi919108175	3.25	2	1	246	28.0	6.81	5.71	hypothetical protein [Arthrobacter sp. IHBB 11108]
gi916834483	2.34	2	1	342	38.8	9.45	5.71	hypothetical protein [Arthrobacter sp. H14]
gi737789565	1.90	1	1	684	73.6	9.17	5.70	hypothetical protein [Arthrobacter albus]
gi767256888	2.40	1	1	874	93.3	5.67	5.70	GCN5 family acetyltransferase [Arthrobacter sp. IHBB 11108]

gi928487038	2.82	2	1	497	51.5	5.58	5.69	aldehyde dehydrogenase [Arthrobacter alpinus]
gi640199686	9.64	8	1	249	26.2	4.79	5.69	hypothetical protein [Arthrobacter sp. 31Y]
gi927293279	4.35	1	1	414	46.0	9.23	5.69	hypothetical protein AL755_02980 (plasmid) [Arthrobacter sp. ERGS1:01]
gi359304298	3.04	1	1	560	56.1	5.41	5.68	S1 family peptidase [Arthrobacter globiformis NBRC 12137]
gi307743798	4.51	1	1	288	30.5	4.98	5.68	DSBA-like thioredoxin domain-containing protein [Arthrobacter arilaitensis Re117]
gi489897286	2.13	2	1	892	95.9	5.22	5.67	alanine--tRNA ligase [Arthrobacter globiformis]
gi116609026	5.81	3	1	344	37.2	10.29	5.66	NAD-dependent epimerase/dehydratase [Arthrobacter sp. FB24]
gi914715134	2.36	1	1	888	94.8	5.99	5.66	trehalose phosphatase [Arthrobacter sp. ZBG10]
gi651499432	1.10	1	1	1544	165.7	5.80	5.66	glutamate synthase [Arthrobacter sp. 35W]
gi910741065	33.33	2	1	57	6.0	4.64	5.66	hypothetical protein AHiyo4_38870 [Arthrobacter sp. Hiyo4]
gi786026207	2.92	2	1	411	43.2	6.30	5.66	SAM-dependent methyltransferase [Arthrobacter chlorophenolicus]
gi910250171	1.99	1	1	754	81.3	6.10	5.66	ATP-dependent DNA helicase RecG [Arthrobacter siccitolerans]
gi654827165	1.06	1	1	1133	124.3	7.14	5.66	DNA polymerase III subunit alpha [Arthrobacter sp. H5]
gi323468295	0.80	1	1	1244	135.4	5.39	5.65	alpha-1,4-glucan:alpha-1,4-glucan 6-glycosyltransferase [Arthrobacter phenanthrenivorans Sphe3]
gi517599095	18.52	1	1	108	11.4	10.33	5.65	hypothetical protein [Arthrobacter sp. 162MFSha1.1]
gi737777617	15.07	2	2	146	15.4	5.52	5.65	deoxyuridine 5'-triphosphate nucleotidohydrolase [Arthrobacter sanguinis]
gi737786463	3.75	7	1	293	32.9	6.58	5.65	DNA methyltransferase [Arthrobacter albus]
gi652423929	2.04	1	1	1521	158.0	4.28	5.65	5'-nucleotidase [Arthrobacter castelli]
gi640197663	3.97	1	1	453	48.7	6.24	5.65	hypothetical protein [Arthrobacter sp. 31Y]
gi927292901	5.24	1	1	191	20.1	9.67	5.64	hypothetical protein AL755_01235 (plasmid) [Arthrobacter sp. ERGS1:01]
gi551256519	2.34	1	1	856	92.3	6.61	5.63	DEAD/DEAH box helicase [Arthrobacter sp. PAO19]
gi908698611	1.65	1	1	546	58.7	5.44	5.63	arginine--tRNA ligase [Arthrobacter sp. RIT-PI-e]
gi359305906	4.15	7	1	410	41.7	5.92	5.63	dihydrolipoamide acyltransferase [Arthrobacter globiformis NBRC 12137]
gi470221566	3.40	1	1	441	48.4	5.11	5.62	cytoplasmic protein [Arthrobacter gangotriensis Lz1y]
gi651497916	1.68	2	2	892	92.7	5.86	5.62	hypothetical protein [Arthrobacter sp. 35W]
gi119951468	0.64	1	1	1884	203.8	5.97	5.62	putative Helicase (plasmid) [Arthrobacter aurescens TC1]
gi403231798	7.86	1	1	140	15.6	9.70	5.62	hypothetical protein ARUE_232p00110 (plasmid) [Arthrobacter sp. Rue61a]
gi359305419	4.86	3	1	288	31.0	9.86	5.62	putative LysR family transcriptional regulator [Arthrobacter globiformis NBRC 12137]
gi674646032	14.02	1	1	107	11.9	7.25	5.62	50S ribosomal protein L29 [Arthrobacter sp. 11W110_air]
gi918268088	1.89	1	1	475	52.7	9.92	5.62	conserved hypothetical protein [Arthrobacter sp. Hiyo1]
gi116609456	20.00	2	1	95	10.1	5.31	5.61	protein of unknown function DUF909 [Arthrobacter sp. FB24]
gi651430627	1.75	1	1	570	59.8	5.05	5.61	dihydroxy-acid dehydratase [Arthrobacter sanguinis]
gi651504295	6.03	1	1	199	21.4	4.73	5.61	fatty acid-binding protein [Arthrobacter sp. 35W]
gi910249965	4.22	3	1	403	45.1	9.66	5.61	hypothetical protein [Arthrobacter siccitolerans]
gi914716317	3.27	10	1	611	68.0	5.82	5.61	hypothetical protein [Arthrobacter sp. ZBG10]
gi651440035	2.35	1	1	255	28.2	4.78	5.60	hypothetical protein [Arthrobacter sp. H14]
gi476399452	4.55	1	1	154	16.8	6.80	5.60	AsnC family transcriptional regulator [Arthrobacter crystallopoietes BAB-32]
gi476400879	3.62	2	1	525	56.0	7.58	5.60	sodium-dependent transporter [Arthrobacter crystallopoietes BAB-32]
gi742852018	0.96	1	1	1151	125.8	5.03	5.60	nuclease [Arthrobacter sp. W1]
gi551254135	1.38	1	1	508	55.2	6.09	5.59	hypothetical protein [Arthrobacter sp. PAO19]
gi517589994	2.28	2	1	307	32.8	8.62	5.59	hypothetical protein [Arthrobacter sp. 135MFCol5.1]
gi690771904	3.11	1	1	482	51.5	6.96	5.58	glutamine ABC transporter permease [Arthrobacter albus DNF00011]
gi749402170	5.54	2	1	325	34.3	6.23	5.58	asparaginase [Arthrobacter sp. AK-YN10]
gi651472389	2.12	3	1	425	45.0	9.98	5.58	MFS transporter [Arthrobacter nicotinovorans]
gi219860973	3.70	8	1	459	49.9	9.55	5.57	major facilitator superfamily MFS_1 [Arthrobacter chlorophenolicus A6]
gi162953597	8.94	5	1	235	26.0	5.26	5.57	iron dependent repressor [Renibacterium salmoninarum ATCC 33209]
gi737805295	8.59	1	1	163	18.3	8.28	5.57	transcriptional regulator [Arthrobacter sp. Br18]
gi759759696	3.80	1	1	474	52.4	6.70	5.57	levanase [Arthrobacter sp. Rue61a]
gi470220739	4.83	1	1	269	27.7	7.14	5.57	type 11 methyltransferase [Arthrobacter gangotriensis Lz1y]
gi674645616	1.82	2	1	1208	135.6	7.24	5.56	CDP-glycerol:poly(glycerophosphate) glycerophosphotransferase [Arthrobacter sp. 11W110_air]
gi651438419	1.30	1	1	844	87.6	4.70	5.56	RND transporter [Arthrobacter sp. H14]
gi823668111	2.47	1	1	485	52.2	5.85	5.55	pyridoxal-dependent decarboxylase [Arthrobacter sp. YC-RL1]
gi517592051	4.12	2	1	413	42.4	10.43	5.55	MFS transporter [Arthrobacter sp. 135MFCol5.1]
gi737871543	7.32	8	1	314	33.7	5.11	5.55	prephenate dehydratase [Cryobacterium roopkundense]
gi749402436	4.88	1	1	246	25.8	6.70	5.55	ABC transporter ATP-binding protein [Arthrobacter sp. AK-YN10]
gi651429194	2.81	1	1	498	53.0	9.07	5.55	multidrug MFS transporter [Arthrobacter sanguinis]
gi916870208	4.44	1	1	315	32.7	5.52	5.54	nicotinate-nucleotide pyrophosphorylase [Arthrobacter sp. Br18]
gi737770920	7.24	2	1	304	31.4	5.06	5.54	sugar kinase [Arthrobacter sp. TB 26]
gi654817170	7.82	3	1	243	25.2	9.73	5.54	septation ring formation regulator EzrA [Arthrobacter sp. UNC362MFTsu5.1]

gi651501289	4.07	2	1	467	48.8	5.86	5.54	flavoprotein [Arthrobacter sp. 35W]
gi651436371	5.83	1	1	309	33.6	6.71	5.54	GTPase Era [Arthrobacter sp. H41]
gi551256445	4.68	1	1	299	32.3	5.20	5.54	UTP--glucose-1-phosphate uridylyltransferase [Arthrobacter sp. PAO19]
gi542106407	12.50	2	1	80	9.3	6.04	5.53	hypothetical protein M707_21935 [Arthrobacter sp. AK-YN10]
gi551253978	2.07	3	1	482	51.1	5.08	5.53	branched-chain alpha-keto acid dehydrogenase subunit E2 [Arthrobacter sp. PAO19]
gi116612426	2.33	1	1	343	36.8	7.44	5.53	transcriptional regulator, AsnC family [Arthrobacter sp. FB24]
gi690773015	9.50	1	1	221	24.5	9.69	5.52	hypothetical protein HMPREF2128_05440 [Arthrobacter albus DNF00011]
gi654812984	1.15	9	1	1047	117.0	5.88	5.52	helicase [Arthrobacter sp. MA-N2]
gi651429678	2.55	1	1	510	56.5	5.52	5.52	ribosome-associated GTPase EngA [Arthrobacter sanguinis]
gi219859666	9.16	1	1	251	27.0	4.84	5.52	protein of unknown function DUF28 [Arthrobacter chlorophenolicus A6]
gi476401847	3.86	2	1	337	36.9	7.20	5.51	ATP dependent DNA ligase [Arthrobacter crystallopoietes BAB-32]
gi651435973	4.09	1	1	513	54.2	7.36	5.51	hypothetical protein [Arthrobacter sp. H41]
gi651454809	9.89	2	1	263	27.9	5.44	5.51	multidrug transporter [Arthrobacter nicotinovorans]
gi928985910	4.12	2	1	194	20.4	5.92	5.51	hypothetical protein [Arthrobacter sp. ERGS1:01]
gi823667169	3.91	1	1	460	49.7	5.27	5.50	allantoicase [Arthrobacter sp. YC-RL1]
gi759755483	7.22	1	1	291	30.3	7.94	5.50	ArsR family transcriptional regulator [Arthrobacter sp. 131MFCol6.1]
gi916691759	3.48	1	1	374	40.9	6.16	5.50	galactose-1-phosphate uridylyltransferase [Arthrobacter castelli]
gi542110174	3.01	1	1	399	42.0	4.88	5.50	cell division protein FtsY [Arthrobacter sp. AK-YN10]
gi910738466	10.07	6	1	139	14.9	8.79	5.49	hypothetical protein AHiyo4_12880 [Arthrobacter sp. Hiyo4]
gi551254853	5.17	3	1	445	46.0	8.63	5.49	3-ketoacyl-ACP reductase [Arthrobacter sp. PAO19]
gi643036657	14.72	4	1	163	17.8	8.57	5.48	CAZy families GT2 protein, partial [uncultured Arthrobacter sp.]
gi937262282	11.64	3	1	146	15.9	5.22	5.48	preprotein translocase subunit TatA [Arthrobacter sp. Edens01]
gi654822191	2.76	5	1	688	70.4	5.19	5.47	5-oxoprolinase [Arthrobacter sp. I3]
gi542109664	2.48	8	1	886	94.5	5.12	5.46	LuxR family transcriptional regulator [Arthrobacter sp. AK-YN10]
gi937258933	6.70	3	1	358	36.6	5.60	5.46	molybdenum ABC transporter ATP-binding protein [Arthrobacter sp. Edens01]
gi323468471	21.62	7	1	111	11.8	6.80	5.46	cupin domain-containing protein [Arthrobacter phenanthrenivorans Sphe3]
gi651506398	8.12	2	1	197	20.6	5.91	5.45	hypothetical protein [Arthrobacter sp. 35W]
gi119949759	5.15	2	1	388	40.0	6.54	5.45	putative cysteine desulfurase [Arthrobacter aurescens TC1]
gi545109763	4.34	4	1	392	39.8	11.62	5.45	MFS transporter [Arthrobacter sp. AK-YN10]
gi937259036	18.39	4	2	174	18.5	6.61	5.44	hypothetical protein AO716_13925 [Arthrobacter sp. Edens01]
gi910741708	1.81	2	1	497	52.6	7.66	5.44	transcriptional regulatory protein AfsQ1 [Arthrobacter sp. Hiyo4]
gi908699164	6.50	2	1	277	31.0	5.52	5.43	oxidoreductase [Arthrobacter sp. RIT-PI-e]
gi927295496	15.34	5	1	163	17.7	5.31	5.42	PTS fructose transporter subunit IIA [Arthrobacter sp. ERGS1:01]
gi917530436	7.95	2	1	151	16.0	4.78	5.42	FHA domain-containing protein [Arthrobacter sp. PAMC25486]
gi116612854	12.75	2	1	102	11.3	8.50	5.42	hypothetical protein Arth_4405 (plasmid) [Arthrobacter sp. FB24]
gi13241964	1.14	1	1	967	102.4	5.40	5.42	sarcosine oxidase subunit A [Arthrobacter sp. 1IN]
gi723609706	6.28	3	1	398	43.1	5.60	5.41	putative acyl-CoA dehydrogenase [Arthrobacter sp. PAMC25486]
gi515764892	1.66	3	1	422	43.5	5.43	5.41	ROK family transcriptional regulator [Arthrobacter sp. M2012083]
gi651439398	2.23	1	1	449	46.6	4.93	5.41	histidinol dehydrogenase [Arthrobacter sp. H14]
gi674645956	4.49	2	1	245	25.4	9.95	5.41	Glycine betaine/carnitine/choline transport system permease protein OpuCB [Arthrobacter sp. 11W
gi651447759	5.18	3	1	309	35.5	10.05	5.40	hypothetical protein [Arthrobacter nicotinovorans]
gi307745640	10.81	2	1	148	15.9	5.82	5.40	MaoC like domain-containing protein [Arthrobacter arilaitensis Re117]
gi651480155	3.19	1	1	376	40.7	7.08	5.40	GDP-mannose-dependent alpha-mannosyltransferase [Arthrobacter sp. Br18]
gi910697873	3.71	6	1	458	50.2	7.81	5.40	uncharacterized ATP-dependent helicase SSO0112, partial [Arthrobacter sp. Hiyo6]
gi542106206	6.50	2	1	200	21.2	4.98	5.40	KfrA protein [Arthrobacter sp. AK-YN10]
gi542106461	0.95	1	1	1364	151.1	5.44	5.40	ATPase [Arthrobacter sp. AK-YN10]
gi767259096	2.87	1	1	348	37.5	5.66	5.40	L-threonine 3-dehydrogenase [Arthrobacter sp. IHBB 11108]
gi116613070	2.70	4	1	408	42.0	11.27	5.39	major facilitator superfamily MFS_1 (plasmid) [Arthrobacter sp. FB24]
gi640195872	4.87	1	1	390	41.9	4.74	5.39	PbrT family lead (Pb2+) uptake porter [Arthrobacter sp. 31Y]
gi403231993	1.92	4	1	520	56.6	8.59	5.39	hypothetical protein ARUE_232p02060 (plasmid) [Arthrobacter sp. Rue61a]
gi359307029	1.64	2	1	730	78.4	9.60	5.39	penicillin-binding protein [Arthrobacter globiformis NBRC 12137]
gi542106827	1.81	2	1	553	58.2	5.19	5.38	phosphoglucomutase [Arthrobacter sp. AK-YN10]
gi654812356	5.24	1	1	458	50.9	6.19	5.37	deoxyribodipyrimidine photolyase [Arthrobacter sp. MA-N2]
gi939036522	3.21	2	1	249	27.1	5.49	5.36	hypothetical protein [Arthrobacter nitroguajacolicus]
gi219858787	8.49	1	1	259	24.9	6.67	5.36	conserved hypothetical protein [Arthrobacter chlorophenolicus A6]
gi908698116	1.16	2	1	1119	115.9	5.11	5.36	hypothetical protein [Arthrobacter sp. RIT-PI-e]
gi654827359	1.96	2	1	408	43.9	7.30	5.36	hydroxyglutarate oxidase [Arthrobacter sp. H5]
gi651487954	3.45	1	1	609	66.0	7.33	5.36	capsule biosynthesis protein CapD [Arthrobacter sp. H20]
gi737812750	3.48	5	1	316	34.0	5.86	5.35	exopolyphosphatase [Arthrobacter sp. H14]

gi651466083	8.22	2	1	146	15.4	4.88	5.35	acyl dehydratase [Arthrobacter sp. 35/47]
gi742757495	1.98	1	1	808	87.4	5.26	5.35	phosphoenolpyruvate synthase [Arthrobacter phenanthrenivorans]
gi359305467	5.30	8	1	321	35.0	8.53	5.35	putative MmyB family DNA-binding protein [Arthrobacter globiformis NBRC 12137]
gi916691541	3.73	4	1	295	31.5	4.89	5.34	methyltransferase type 11 [Arthrobacter castelli]
gi823667609	4.02	2	1	423	45.5	5.27	5.34	isochorismate synthase [Arthrobacter sp. YC-RL1]
gi674645785	2.96	1	1	372	41.1	4.81	5.32	Peptide chain release factor 2 [Arthrobacter sp. 11W110_air]
gi742851163	2.79	1	1	358	40.5	5.43	5.32	hypothetical protein [Arthrobacter sp. W1]
gi307744157	4.81	2	1	208	22.8	5.72	5.31	putative SAM-dependent methyltransferase [Arthrobacter arilaitensis Re117]
gi916573878	1.03	1	1	1650	182.1	5.82	5.30	helicase [Arthrobacter sp. TB 26]
gi916869682	10.62	2	1	226	23.4	4.56	5.30	hypothetical protein [Arthrobacter sp. Br18]
gi651480777	6.86	2	1	277	29.6	5.05	5.30	maleylpyruvate isomerase [Arthrobacter sp. Br18]
gi518314033	8.59	4	1	128	13.7	9.14	5.30	hypothetical protein [Arthrobacter sp. TB 23]
gi162954718	1.65	1	1	424	46.3	8.97	5.30	DNA polymerase IV [Renibacterium salmoninarum ATCC 33209]
gi910738217	7.83	1	1	230	24.4	4.73	5.29	probable disulfide bond formation protein D [Arthrobacter sp. Hiyo4]
gi651454653	13.30	5	1	188	20.5	6.81	5.29	MarR family transcriptional regulator [Arthrobacter nicotinovorans]
gi910249704	2.81	1	1	320	36.0	10.18	5.29	hypothetical protein [Arthrobacter siccitolerans]
gi927033011	6.80	2	1	353	38.9	9.29	5.29	cytochrome C biogenesis protein [Arthrobacter sp. LS16]
gi470216828	4.91	1	1	407	45.3	5.66	5.29	hypothetical protein ADIAG_03027 [Arthrobacter gangotriensis Lz1y]
gi910744418	3.30	2	1	303	32.8	5.73	5.29	probable acetolactate synthase large subunit [Arthrobacter sp. Hiyo8]
gi767257660	1.80	1	1	555	60.4	7.18	5.28	DNA helicase [Arthrobacter sp. IHBB 11108]
gi654811603	23.08	1	1	78	8.0	5.26	5.28	thiamine biosynthesis protein ThiS [Arthrobacter sp. MA-N2]
gi651504066	4.20	1	1	405	43.0	5.59	5.28	SAM-dependent methyltransferase [Arthrobacter sp. 35W]
gi476400726	4.51	2	1	288	30.7	4.98	5.27	nucleoside-diphosphate sugar epimerase [Arthrobacter crystallopoietes BAB-32]
gi749402351	3.45	1	1	348	38.5	6.33	5.27	NAD-dependent dehydratase [Arthrobacter sp. AK-YN10]
gi674645086	1.72	2	1	581	61.6	5.22	5.27	V-type ATP synthase alpha chain [Arthrobacter sp. 11W110_air]
gi476401998	4.30	1	1	349	37.5	5.85	5.27	recombinase A [Arthrobacter crystallopoietes BAB-32]
gi219858748	3.35	4	1	686	73.4	5.67	5.27	conserved hypothetical protein [Arthrobacter chlorophenolicus A6]
gi307744585	2.11	1	1	475	50.8	10.51	5.26	putative D-alanyl-D-alanine carboxypeptidase [Arthrobacter arilaitensis Re117]
gi307744744	1.40	1	1	570	63.2	5.21	5.26	putative fatty-acid--Co-A ligase [Arthrobacter arilaitensis Re117]
gi918269017	20.31	14	1	64	7.0	5.02	5.26	dipeptide transport system permease protein DppB [Arthrobacter sp. Hiyo1]
gi551255618	2.95	1	1	509	56.0	5.72	5.25	(dimethylallyl)adenosine tRNA methylthiotransferase [Arthrobacter sp. PAO19]
gi910746125	14.40	6	1	125	13.1	4.84	5.25	probable NAD-dependent malic enzyme 4 [Arthrobacter sp. Hiyo8]
gi759747060	12.44	2	1	201	22.0	5.72	5.25	hypothetical protein [Arthrobacter sp. 31Y]
gi910250456	9.33	1	1	150	15.8	7.30	5.25	hypothetical protein [Arthrobacter siccitolerans]
gi307746355	13.64	4	1	88	9.8	9.74	5.24	conserved hypothetical protein [Arthrobacter arilaitensis Re117]
gi767257965	2.98	6	1	302	32.7	7.58	5.23	hypothetical protein UM93_10575 [Arthrobacter sp. IHBB 11108]
gi654822918	6.88	1	1	349	36.3	5.22	5.23	LacI family transcriptional regulator [Arthrobacter sp. I3]
gi162953526	8.67	2	1	173	17.6	5.22	5.22	carboxypeptidase G2 precursor [Renibacterium salmoninarum ATCC 33209]
gi551253735	7.05	6	1	298	32.2	9.89	5.22	glutamate ABC transporter permease [Arthrobacter sp. PAO19]
gi542108289	2.59	1	1	309	32.9	7.08	5.21	epimerase [Arthrobacter sp. AK-YN10]
gi515765729	1.27	1	1	711	76.9	5.66	5.21	amylo-alpha-1,6-glucosidase [Arthrobacter sp. M2012083]
gi636846688	5.08	1	1	236	24.0	5.43	5.21	hypothetical protein [Arthrobacter sp. TB 26]
gi636844019	5.40	4	1	278	29.8	5.44	5.21	hypothetical protein [Arthrobacter sp. TB 26]
gi640198810	4.92	1	1	183	19.6	4.28	5.20	RNA-binding protein [Arthrobacter sp. 31Y]
gi551255650	1.83	3	1	493	53.7	5.54	5.20	sugar phosphate isomerase [Arthrobacter sp. PAO19]
gi910697773	1.09	1	1	736	77.9	6.16	5.19	conserved hypothetical protein [Arthrobacter sp. Hiyo6]
gi219859544	3.17	2	1	410	43.6	5.08	5.19	cysteine/1-D-myo-inosityl 2-amino-2-deoxy-alpha-D-glucopyranoside ligase [Arthrobacter chloroph
gi116610842	2.33	15	1	558	58.5	5.41	5.19	Amidohydrolase 3 [Arthrobacter sp. FB24]
gi786027762	4.17	8	1	288	30.6	5.59	5.18	aldo/keto reductase [Arthrobacter chlorophenolicus]
gi470220665	3.23	1	1	403	43.5	9.66	5.17	transposase, IS110 family, OrfA [Arthrobacter gangotriensis Lz1y]
gi219858403	2.23	1	1	493	51.8	9.66	5.17	transcriptional regulator, GntR family with aminotransferase domain [Arthrobacter chlorophenolicu
gi930825394	9.85	4	1	132	13.8	5.80	5.17	hypothetical protein AOZ07_03145 [Arthrobacter arilaitensis]
gi551254987	7.53	2	1	186	20.5	4.44	5.16	deaminase [Arthrobacter sp. PAO19]
gi551255959	4.11	2	1	316	33.3	4.83	5.16	3-oxoacyl-ACP synthase [Arthrobacter sp. PAO19]
gi742071374	2.54	1	1	433	46.3	10.30	5.15	cell division protein FtsW [Arthrobacter sp. MWB30]
gi930828228	3.80	2	1	474	53.3	5.16	5.15	biotin carboxylase [Arthrobacter arilaitensis]
gi917572288	3.80	1	1	474	53.3	5.05	5.15	biotin carboxylase [Arthrobacter sp. PAO19]
gi910842677	6.58	4	1	243	24.9	4.73	5.14	pilus assembly protein CpaB [Arthrobacter sp. ZBG10]
gi916357053	2.36	3	1	382	40.1	11.49	5.14	arsenic transporter [Arthrobacter sp. 162MFSha1.1]

gi517606820	5.02	2	1	219	22.6	9.17	5.14	hypothetical protein [Arthrobacter sp. 161MFSha2.1]
gi654816846	15.09	5	2	265	28.8	8.95	5.14	cell division protein FtsE [Arthrobacter sp. UNC362MFTsu5.1]
gi119951470	1.80	2	1	1165	128.7	5.05	5.14	helicase, SNF2 family (plasmid) [Arthrobacter aurescens TC1]
gi786027726	0.50	1	1	1189	133.4	5.60	5.13	DNA methylase [Arthrobacter chlorophenolicus]
gi742072644	3.25	6	1	431	46.0	8.13	5.13	HNH endonuclease domain protein [Arthrobacter sp. MWB30]
gi910248793	5.36	1	1	261	28.5	4.86	5.13	hypothetical protein [Arthrobacter siccitolerans]
gi927296282	3.12	1	1	673	70.1	5.67	5.12	short-chain dehydrogenase [Arthrobacter sp. ERGS1:01]
gi749402326	2.50	2	1	559	61.4	10.10	5.12	polyprenyl glycosylphosphotransferase [Arthrobacter sp. AK-YN10]
gi116610024	4.52	5	1	221	23.9	5.66	5.12	hypothetical protein Arth_1354 [Arthrobacter sp. FB24]
gi917739509	2.16	1	1	509	54.0	6.10	5.12	sugar ABC transporter ATPase [Arthrobacter sp. W1]
gi910249149	13.83	3	1	94	9.2	9.98	5.11	hypothetical protein [Arthrobacter siccitolerans]
gi307746154	1.77	2	1	621	68.7	5.45	5.11	type I restriction-modification system modification subunit [Arthrobacter arilaitensis Re117]
gi651431487	3.30	1	1	455	49.7	5.31	5.11	histidine--tRNA ligase [Arthrobacter sanguinis]
gi219858416	4.78	2	1	293	33.0	5.80	5.11	aminoglycoside phosphotransferase [Arthrobacter chlorophenolicus A6]
gi470215932	2.55	1	1	353	37.4	9.38	5.10	binding-protein-dependent transport system inner membrane protein [Arthrobacter gangotriensis
gi476402641	4.63	2	1	281	30.9	4.82	5.10	catechol 1,2-dioxygenase [Arthrobacter crystallopoietes BAB-32]
gi928487378	6.20	1	1	258	28.0	4.86	5.10	hypothetical protein AOC05_11070 [Arthrobacter alpinus]
gi476401398	5.81	10	1	327	36.8	6.46	5.10	phenylacetate-CoA oxygenase subunit PaaA [Arthrobacter crystallopoietes BAB-32]
gi654813931	3.14	3	1	574	64.6	5.22	5.09	X-Pro dipeptidyl-peptidase [Arthrobacter sp. MA-N2]
gi517598084	3.80	5	1	527	56.8	9.50	5.09	signal recognition particle protein [Arthrobacter sp. 162MFSha1.1]
gi757625802	6.15	3	1	195	21.4	5.33	5.09	adenylate kinase [Arthrobacter sp. SPG23]
gi652424386	3.16	2	1	475	54.2	5.82	5.09	deoxyribodipyrimidine photolyase [Arthrobacter castelli]
gi648260078	3.02	2	1	497	55.4	5.00	5.09	MULTISPECIES: lysine--tRNA ligase [Arthrobacter]
gi919218993	8.82	2	1	204	21.5	9.85	5.08	hypothetical protein [Arthrobacter sp. YC-RL1]
gi918266944	13.11	3	1	122	12.9	9.58	5.08	hypothetical protein AHiyo1_34090 [Arthrobacter sp. Hiyo1]
gi767258152	6.28	3	1	191	21.4	10.42	5.08	cation:proton antiporter [Arthrobacter sp. IHBB 11108]
gi916820305	6.08	3	1	263	27.7	5.06	5.08	hypothetical protein [Arthrobacter sp. H20]
gi42627732	4.61	2	1	282	31.1	4.94	5.07	catechol 1,2-dioxygenase [Arthrobacter sp. BA-5-17]
gi219859887	5.04	10	1	258	28.3	4.73	5.07	5-carboxymethyl-2-hydroxymuconate Delta-isomerase [Arthrobacter chlorophenolicus A6]
gi928488753	1.01	4	1	1090	121.0	5.10	5.07	isoleucine--tRNA ligase [Arthrobacter alpinus]
gi918469404	2.19	2	1	548	57.0	9.91	5.06	hypothetical protein [Arthrobacter crystallopoietes]
gi917441950	6.50	2	1	123	13.0	9.98	5.06	GntR family transcriptional regulator [Arthrobacter albus]
gi654827216	2.01	1	1	497	56.2	5.41	5.06	DNA methyltransferase [Arthrobacter sp. H5]
gi515766339	5.57	7	1	305	34.1	4.78	5.06	myo-inosose-2 dehydratase [Arthrobacter sp. M2012083]
gi765007060	8.60	2	2	314	33.3	7.11	5.05	hypothetical protein [Arthrobacter sp. A3]
gi917739712	11.54	7	1	156	17.2	8.82	5.05	hypothetical protein [Arthrobacter sp. W1]
gi910250411	16.67	1	1	84	9.3	4.59	5.05	excisionase [Arthrobacter siccitolerans]
gi910741135	13.00	2	1	200	20.0	4.68	5.04	hypothetical protein AHiyo4_39570 [Arthrobacter sp. Hiyo4]
gi359306136	2.70	1	1	408	44.7	5.31	5.04	hypothetical protein ARGLB_047_00830 [Arthrobacter globiformis NBRC 12137]
gi723609895	6.05	1	1	463	49.8	8.56	5.04	general substrate transporter [Arthrobacter sp. PAMC25486]
gi930827645	4.53	2	1	265	28.2	4.77	5.04	hypothetical protein AOZ07_16065 [Arthrobacter arilaitensis]
gi910739504	5.58	1	1	197	21.0	7.34	5.04	UPF0051 protein ML0594 [Arthrobacter sp. Hiyo4]
gi517605914	4.52	2	1	221	23.7	9.98	5.04	MULTISPECIES: hypothetical protein [Arthrobacter]
gi937258424	13.82	4	1	152	16.3	8.22	5.04	hypothetical protein AO716_10230 [Arthrobacter sp. Edens01]
gi918265691	5.08	1	1	354	37.1	6.46	5.04	maleylacetate reductase [Arthrobacter sp. Hiyo1]
gi636844030	4.59	2	1	392	42.7	5.05	5.04	acyl-CoA dehydrogenase [Arthrobacter sp. TB 26]
gi737781198	3.81	1	1	473	49.5	6.70	5.04	SAM-dependent methyltransferase [Arthrobacter sp. 35W]
gi654816069	9.74	2	1	154	16.8	6.07	5.03	glutamyl-tRNA amidotransferase [Arthrobacter sp. UNC362MFTsu5.1]
gi651486945	2.27	2	1	484	51.1	5.81	5.03	inosine-5-monophosphate dehydrogenase [Arthrobacter sp. Br18]
gi910694102	6.81	1	1	235	25.3	5.24	5.03	hypothetical protein AHiyo6_28660, partial [Arthrobacter sp. Hiyo6]
gi219859202	2.27	2	1	440	44.4	9.67	5.02	Lytic transglycosylase catalytic [Arthrobacter chlorophenolicus A6]
gi443482154	1.97	1	1	610	67.1	4.97	5.02	phosphoenolpyruvate carboxykinase [Arthrobacter nitrophenolicus]
gi518313565	1.82	2	1	658	70.7	9.88	5.02	MULTISPECIES: hypothetical protein [Arthrobacter]
gi651430051	3.17	2	1	473	52.7	6.27	5.02	plasmid replication-like protein [Arthrobacter sanguinis]
gi651460251	4.72	1	1	339	36.2	6.00	5.02	hypothetical protein [Arthrobacter sp. 35/47]
gi916820226	0.92	4	1	1087	120.7	5.74	5.02	hypothetical protein [Arthrobacter sp. H20]
gi359304233	8.96	1	1	212	23.2	6.20	5.02	putative glycosyltransferase [Arthrobacter globiformis NBRC 12137]
gi927293109	1.86	2	1	969	100.4	9.06	5.01	hypothetical protein AL755_02560 (plasmid) [Arthrobacter sp. ERGS1:01]
gi639129235	10.45	2	1	201	21.1	4.97	5.01	XRE family transcriptional regulator [Arthrobacter sp. CAL618]

gi765005702	2.43	2	1	575	60.5	5.14	5.01	DNA repair protein RecN [Arthrobacter sp. A3]
gi119950304	5.35	2	1	318	34.5	6.44	5.01	putative FHA domain protein [Arthrobacter aurescens TC1]
gi116609178	4.98	1	1	482	50.8	5.36	5.00	Mannitol dehydrogenase [Arthrobacter sp. FB24]
gi651431350	2.19	2	1	869	91.7	6.46	5.00	hypothetical protein [Arthrobacter sanguinis]
gi651438938	2.00	4	1	551	59.2	5.05	5.00	urocanate hydratase [Arthrobacter sp. H14]
gi823665909	3.72	2	1	457	51.7	6.01	5.00	Pup--protein ligase [Arthrobacter sp. YC-RL1]
gi651438601	9.51	2	1	263	28.2	9.96	4.99	hypothetical protein [Arthrobacter sp. H14]
gi651436760	4.51	2	1	244	26.2	5.39	4.98	hypothetical protein [Arthrobacter sp. H41]
gi910747728	5.11	3	1	411	45.7	9.96	4.98	UPF0182 protein Arth_2749 [Arthrobacter sp. Hiyo8]
gi674644361	1.76	2	1	625	65.8	4.84	4.98	Serine/threonine-protein kinase PknB [Arthrobacter sp. 11W110_air]
gi737800388	3.73	1	1	402	42.7	4.68	4.98	signal recognition particle-docking protein FtsY [Arthrobacter castelli]
gi737784115	4.98	1	1	502	57.4	4.97	4.97	sulfatase [Arthrobacter nitrophenolicus]
gi742072727	4.03	2	1	372	41.2	6.09	4.97	phenylalanyl-tRNA synthetase subunit alpha [Arthrobacter sp. MWB30]
gi674644716	17.14	4	1	105	11.0	4.53	4.97	hypothetical protein BN1051_00873 [Arthrobacter sp. 11W110_air]
gi651491320	4.32	1	1	417	46.3	5.85	4.97	cyclopropane-fatty-acyl-phospholipid synthase [Arthrobacter sp. H20]
gi162952426	7.37	1	1	312	34.4	5.63	4.95	transcriptional regulator, LysR family [Renibacterium salmoninarum ATCC 33209]
gi914715384	5.71	2	1	333	35.0	5.92	4.95	exonuclease [Arthrobacter sp. ZBG10]
gi910741305	1.47	3	1	953	102.4	5.95	4.95	nitrite reductase [NAD(P)H] [Arthrobacter sp. Hiyo4]
gi640204645	1.21	5	1	746	84.2	8.60	4.95	glycosyltransferase [Arthrobacter sp. 31Y]
gi910745226	5.93	1	1	354	35.9	9.82	4.95	DNA polymerase III subunits gamma and tau [Arthrobacter sp. Hiyo8]
gi759746540	1.46	1	1	1303	137.5	6.84	4.94	amino acid adenylation protein [Arthrobacter sp. 31Y]
gi917013410	6.13	2	1	163	17.1	10.29	4.93	50S ribosomal protein L15 [Arthrobacter sanguinis]
gi359303411	2.49	4	1	361	37.9	4.97	4.93	zinc-containing alcohol dehydrogenase [Arthrobacter globiformis NBRC 12137]
gi917530565	3.16	5	1	380	41.8	5.86	4.93	ABC transporter ATP-binding protein [Arthrobacter sp. PAMC25486]
gi910749080	4.30	3	1	302	33.1	8.34	4.93	probable oxidoreductase OrdL (plasmid) [Arthrobacter sp. Hiyo8]
gi916782238	7.78	3	2	334	35.9	6.47	4.93	hypothetical protein [Arthrobacter sp. 35W]
gi443482252	50.00	3	1	34	3.4	4.75	4.93	HAD-superfamily hydrolase [Arthrobacter nitrophenolicus]
gi517604909	4.35	1	1	207	22.7	7.46	4.93	hypothetical protein [Arthrobacter sp. 131MFCol6.1]
gi737800293	4.46	2	1	314	33.6	5.02	4.92	5'-3' exonuclease [Arthrobacter castelli]
gi651453122	13.59	3	1	103	11.1	9.74	4.92	hypothetical protein [Arthrobacter nicotinovorans]
gi119948378	8.43	1	1	249	26.8	6.09	4.92	putative ABC transporter, ATP-binding protein [Arthrobacter aurescens TC1]
gi654811496	15.75	4	2	254	27.8	9.39	4.91	cell division protein FtsE [Arthrobacter sp. MA-N2]
gi359305339	6.02	2	1	332	37.9	5.30	4.91	hypothetical protein ARGLB_070_00150 [Arthrobacter globiformis NBRC 12137]
gi162953601	2.55	1	1	431	45.8	5.85	4.91	cysteine desulfurase [Renibacterium salmoninarum ATCC 33209]
gi918268509	5.88	6	1	340	38.5	7.80	4.91	trehalose-phosphate synthase [Arthrobacter sp. Hiyo1]
gi476400520	6.92	2	1	289	30.2	7.96	4.91	aerobic C4-dicarboxylate transporter [Arthrobacter crystallopoietes BAB-32]
gi359304214	6.12	1	1	327	33.3	6.98	4.91	putative S1 family peptidase [Arthrobacter globiformis NBRC 12137]
gi757624831	2.87	1	1	557	60.4	5.05	4.90	Triostin synthetase I [Arthrobacter sp. SPG23]
gi545111382	2.18	3	1	505	54.0	6.42	4.90	hypothetical protein [Arthrobacter sp. AK-YN10]
gi760112191	18.18	2	2	198	20.7	9.28	4.90	hypothetical protein [Arthrobacter chlorophenolicus]
gi307744885	13.45	2	1	119	13.5	10.42	4.90	hypothetical protein AARI_16430 [Arthrobacter arilaitensis Re117]
gi651430391	2.77	2	1	361	39.4	4.91	4.90	inositol-3-phosphate synthase [Arthrobacter sanguinis]
gi916871801	10.09	1	1	218	23.2	9.35	4.89	hypothetical protein [Arthrobacter sp. H5]
gi910738303	14.05	1	1	121	12.7	10.35	4.89	HTH-type transcriptional regulator RegA [Arthrobacter sp. Hiyo4]
gi759760833	3.61	6	1	332	37.5	6.28	4.89	dTDP-glucose 4,6-dehydratase [Arthrobacter sp. Rue61a]
gi651448988	11.26	3	2	373	41.5	6.61	4.89	hypothetical protein [Arthrobacter nicotinovorans]
gi476402503	10.14	5	1	148	16.2	4.50	4.89	hypothetical protein D477_003043 [Arthrobacter crystallopoietes BAB-32]
gi162955075	5.75	2	1	348	36.4	5.31	4.88	zinc-dependent alcohol dehydrogenase [Renibacterium salmoninarum ATCC 33209]
gi757624850	6.15	4	1	325	34.9	9.33	4.88	hypothetical protein TV39_09305 [Arthrobacter sp. SPG23]
gi765008815	9.62	3	1	208	22.5	6.55	4.88	TetR family transcriptional regulator [Arthrobacter sp. A3]
gi651430819	0.79	1	1	755	82.2	5.16	4.87	ATPase AAA [Arthrobacter sanguinis]
gi119948084	5.28	1	1	398	42.3	5.59	4.87	conserved hypothetical protein [Arthrobacter aurescens TC1]
gi651441093	5.43	1	1	129	14.5	5.78	4.87	hypothetical protein [Arthrobacter sp. 9MFCol3.1]
gi359307197	3.72	2	1	403	42.9	5.36	4.87	putative oxidoreductase [Arthrobacter globiformis NBRC 12137]
gi723606788	4.15	2	1	217	23.3	6.90	4.87	putative ABC-type multidrug transport system, ATPase component [Arthrobacter sp. PAMC25486]
gi651502645	3.58	3	1	307	32.8	7.21	4.86	hypothetical protein [Arthrobacter sp. 35W]
gi323471652	3.04	3	1	493	55.0	8.66	4.85	hypothetical protein Asphe3_42700 (plasmid) [Arthrobacter phenanthrenivorans Sphe3]
gi918265976	5.45	1	1	220	23.6	5.96	4.85	lipoprotein-releasing system ATP-binding protein LolD [Arthrobacter sp. Hiyo1]
gi636847354	3.29	2	1	334	36.4	5.34	4.85	aldo/keto reductase [Arthrobacter sp. TB 26]

gi723606889	3.72	1	1	457	51.5	6.32	4.84	proteasome component [Arthrobacter sp. PAMC25486]
gi443482393	1.86	4	1	485	51.0	8.69	4.84	phytoene dehydrogenase-like oxidoreductase [Arthrobacter nitrophenolicus]
gi742070521	10.38	1	1	183	18.8	6.54	4.84	CDP-diacylglycerol--inositol 3-phosphatidyltransferase PgsA [Arthrobacter sp. MWB30]
gi654811375	5.60	2	1	268	29.1	5.49	4.84	transglutaminase [Arthrobacter sp. MA-N2]
gi517590415	5.19	2	1	482	50.7	6.21	4.84	hypothetical protein [Arthrobacter sp. 135MFCol5.1]
gi307746104	3.99	3	1	501	53.4	5.34	4.83	xanthine dehydrogenase, iron-sulfur and FAD-binding subunit [Arthrobacter arilaitensis Re117]
gi476399103	5.34	2	2	468	49.9	4.78	4.83	succinate-semialdehyde dehydrogenase [Arthrobacter crystallopoietes BAB-32]
gi737812226	17.82	6	1	101	11.0	11.43	4.83	transposase, partial [Arthrobacter sp. H14]
gi823666923	9.52	4	1	252	26.6	5.33	4.83	3-hydroxybutyrate dehydrogenase [Arthrobacter sp. YC-RL1]
gi517598654	4.26	1	1	235	23.9	5.07	4.83	hypothetical protein [Arthrobacter sp. 162MFSha1.1]
gi359306187	5.88	3	1	408	42.0	6.13	4.83	putative NagC family transcriptional regulator [Arthrobacter globiformis NBRC 12137]
gi443480567	4.71	1	1	361	39.9	9.09	4.83	glycosyltransferase [Arthrobacter nitrophenolicus]
gi910694916	8.17	2	2	416	46.2	5.25	4.82	proteasome-associated ATPase [Arthrobacter sp. Hiyo6]
gi737809913	5.29	5	1	170	19.2	11.25	4.82	hypothetical protein [Arthrobacter sp. 35/47]
gi359304436	3.15	1	1	413	43.3	5.87	4.82	hypothetical protein ARGLB_085_03020 [Arthrobacter globiformis NBRC 12137]
gi307743576	43.14	8	1	51	6.1	6.57	4.82	hypothetical protein AARI_03140 [Arthrobacter arilaitensis Re117]
gi517598984	4.63	3	1	475	50.3	6.21	4.82	glycoside hydrolase [Arthrobacter sp. 162MFSha1.1]
gi928487587	6.75	1	1	252	27.4	5.81	4.82	iron ABC transporter ATP-binding protein [Arthrobacter alpinus]
gi823665387	4.95	2	1	202	22.2	9.31	4.82	CDP-diacylglycerol--glycerol-3-phosphate 3-phosphatidyltransferase [Arthrobacter sp. YC-RL1]
gi307744321	11.27	4	2	275	29.9	6.18	4.82	osmoprotectant (glycine betaine/carnitine/choline/L-proline) ABC transporter, ATP-binding subunit
gi908697195	12.21	3	1	131	14.5	4.48	4.82	glyoxalase [Arthrobacter sp. RIT-PI-e]
gi918469410	3.59	4	1	390	41.0	5.35	4.81	hypothetical protein [Arthrobacter crystallopoietes]
gi937258706	6.63	2	1	332	35.9	7.08	4.81	aspartate carbamoyltransferase [Arthrobacter sp. Edens01]
gi927293707	4.46	1	1	471	50.7	5.81	4.81	dTDP-4-dehydrorhamnose reductase [Arthrobacter sp. ERGS1:01]
gi443481210	20.45	2	1	88	9.7	5.76	4.81	flavin reductase domain-containing protein [Arthrobacter nitrophenolicus]
gi759768302	2.25	2	1	533	56.9	8.88	4.81	ABC transporter [Arthrobacter sp. SPG23]
gi654811590	5.71	1	1	333	33.8	5.26	4.81	hydroxymethylbilane synthase [Arthrobacter sp. MA-N2]
gi918266125	4.18	1	1	359	39.5	9.64	4.80	uncharacterized ABC transporter ATP-binding protein YknV [Arthrobacter sp. Hiyo1]
gi910739952	5.19	6	1	347	36.9	6.81	4.80	hypothetical protein AHiyo4_27740 [Arthrobacter sp. Hiyo4]
gi823665685	3.85	2	1	364	38.9	5.11	4.80	oxidoreductase [Arthrobacter sp. YC-RL1]
gi517603095	5.84	2	1	257	30.2	6.81	4.80	hypothetical protein [Arthrobacter sp. 131MFCol6.1]
gi116609542	4.62	4	1	260	27.4	5.55	4.80	methionine aminopeptidase, type I [Arthrobacter sp. FB24]
gi723606794	3.64	1	1	385	42.5	5.22	4.80	hypothetical protein ART_0571 [Arthrobacter sp. PAMC25486]
gi737776995	5.39	2	1	297	30.9	5.78	4.80	NADH-ubiquinone oxidoreductase [Arthrobacter sanguinis]
gi219861659	5.49	1	1	346	38.6	5.82	4.79	hypothetical protein Achl_4049 (plasmid) [Arthrobacter chlorophenolicus A6]
gi219861779	12.42	3	1	161	16.4	9.10	4.79	hypothetical protein Achl_4169 (plasmid) [Arthrobacter chlorophenolicus A6]
gi674644307	7.52	1	1	306	32.1	4.97	4.79	2-dehydro-3-deoxygluconokinase [Arthrobacter sp. 11W110_air]
gi654817827	3.45	9	1	464	50.9	9.29	4.79	MFS transporter [Arthrobacter sp. UNC362MFTsu5.1]
gi910743092	5.16	4	1	349	36.9	8.85	4.79	xanthan lyase [Arthrobacter sp. Hiyo8]
gi757625047	2.98	2	1	504	54.3	9.76	4.79	3-methyladenine DNA glycosylase [Arthrobacter sp. SPG23]
gi476402684	8.73	4	1	229	23.8	4.63	4.79	hypothetical protein D477_002441 [Arthrobacter crystallopoietes BAB-32]
gi908697387	48.65	6	1	37	4.3	10.43	4.78	50S ribosomal protein L36 [Arthrobacter sp. RIT-PI-e]
gi651441419	1.24	2	1	1048	112.1	5.77	4.78	acriflavin resistance protein [Arthrobacter sp. 9MFCol3.1]
gi651442047	7.10	5	1	169	18.7	9.55	4.78	hypothetical protein [Arthrobacter sp. 9MFCol3.1]
gi651500373	3.66	4	1	382	40.8	6.20	4.78	GntR family transcriptional regulator [Arthrobacter sp. 35W]
gi742851088	3.31	2	1	543	58.1	5.08	4.78	2-aminobenzoate-CoA ligase [Arthrobacter sp. W1]
gi476400072	9.86	1	1	71	7.6	5.96	4.78	hypothetical protein D477_015426 [Arthrobacter crystallopoietes BAB-32]
gi928486479	5.05	4	1	218	23.8	4.82	4.78	hypothetical protein AOC05_04935 [Arthrobacter alpinus]
gi930825585	4.66	3	1	386	41.3	5.39	4.78	phosphoribosylaminoimidazole carboxylase [Arthrobacter arilaitensis]
gi757624985	8.70	3	2	414	44.8	5.31	4.78	kynureninase [Arthrobacter sp. SPG23]
gi651438638	5.24	2	1	191	20.8	4.88	4.78	hypothetical protein [Arthrobacter sp. H14]
gi674644922	9.01	2	1	233	24.8	5.36	4.78	Cytidylate kinase [Arthrobacter sp. 11W110_air]
gi723607465	30.23	1	1	43	4.7	10.87	4.77	hypothetical protein ART_1242 [Arthrobacter sp. PAMC25486]
gi916691707	7.04	9	1	213	22.2	4.89	4.77	hypothetical protein [Arthrobacter castelli]
gi910249159	3.17	1	1	725	77.6	7.43	4.77	glycosyl transferase [Arthrobacter siccitolerans]
gi910742587	5.41	2	1	333	37.3	10.14	4.77	integrase family protein [Arthrobacter sp. Hiyo8]
gi927295694	3.55	1	1	423	44.0	6.10	4.77	siroheme synthase [Arthrobacter sp. ERGS1:01]
gi910746333	16.51	5	1	109	11.2	11.72	4.77	hypothetical protein AHiyo8_40350 [Arthrobacter sp. Hiyo8]
gi910742798	10.07	1	1	149	15.8	8.92	4.77	uncharacterized aromatic compound monooxygenase YhjG [Arthrobacter sp. Hiyo8]

gi757623351	5.48	3	1	420	45.3	9.85	4.77	mannosyltransferase [Arthrobacter sp. SPG23]
gi517609826	6.36	1	1	330	36.4	5.76	4.76	hypothetical protein [Arthrobacter sp. 161MFSha2.1]
gi918268921	4.90	4	1	306	33.8	6.10	4.76	GTP-binding protein TypA/BipA homolog [Arthrobacter sp. Hiyo1]
gi652422781	4.17	1	1	312	33.7	6.42	4.76	hypothetical protein [Arthrobacter castelli]
gi823666598	8.81	2	1	261	27.7	5.00	4.75	crotonase [Arthrobacter sp. YC-RL1]
gi930827545	4.13	3	1	509	54.4	5.00	4.75	oxidoreductase [Arthrobacter arilaitensis]
gi654825599	3.26	1	1	399	43.1	9.61	4.75	transposase [Arthrobacter sp. H5]
gi939051185	6.29	3	1	159	17.8	6.89	4.75	hypothetical protein [Arthrobacter sp. JCM 19049]
gi51534897	1.85	2	1	542	58.8	5.26	4.75	N-substituted formamide deformylase [Arthrobacter pascens]
gi651484035	2.31	1	1	562	62.6	5.59	4.75	hypothetical protein [Arthrobacter sp. Br18]
gi651456428	2.17	1	1	877	95.9	5.10	4.75	DNA polymerase I [Arthrobacter sp. 35/47]
gi674644252	7.24	3	2	387	42.2	9.61	4.75	hypothetical protein BN1051_00403 [Arthrobacter sp. 11W110_air]
gi742757414	5.02	1	1	219	22.7	7.36	4.74	hypothetical protein RM50_06260 [Arthrobacter phenanthrenivorans]
gi742852325	3.56	3	1	337	35.5	5.34	4.74	NADPH:quinone reductase [Arthrobacter sp. W1]
gi910744598	3.37	1	1	415	41.0	5.25	4.73	leu/Ile/Val-binding protein homolog 3 [Arthrobacter sp. Hiyo8]
gi359306658	4.17	1	1	408	43.2	5.02	4.73	hypothetical protein ARGLB_037_02150 [Arthrobacter globiformis NBRC 12137]
gi928488353	7.27	1	1	275	30.0	4.09	4.73	hypothetical protein AOC05_17425 [Arthrobacter alpinus]
gi737800948	4.47	1	1	313	31.6	5.07	4.73	hypothetical protein [Arthrobacter castelli]
gi410689885	16.67	1	1	126	14.3	6.70	4.73	hypothetical protein (plasmid) [Arthrobacter sp. J3-53]
gi910737752	3.94	2	1	330	37.1	10.08	4.72	hypothetical protein AHiyo4_05740 [Arthrobacter sp. Hiyo4]
gi651498886	7.62	1	1	210	22.1	6.68	4.72	DNA-3-methyladenine glycosidase [Arthrobacter sp. 35W]
gi116613161	5.10	1	1	353	34.2	10.52	4.72	conserved hypothetical protein (plasmid) [Arthrobacter sp. FB24]
gi517601268	15.07	3	1	146	15.1	8.56	4.72	hypothetical protein [Arthrobacter sp. 162MFSha1.1]
gi323469183	3.21	2	1	374	40.3	6.70	4.72	ABC-type spermidine/putrescine transport system, ATPase component [Arthrobacter phenanthreni
gi219857766	3.36	2	1	327	33.7	8.65	4.72	conserved hypothetical protein [Arthrobacter chlorophenolicus A6]
gi742757714	7.88	2	1	241	26.0	5.29	4.72	Asp/Glu racemase [Arthrobacter phenanthrenivorans]
gi162953843	2.87	4	1	487	53.8	6.20	4.72	sensory transduction protein kinase [Renibacterium salmoninarum ATCC 33209]
gi910252139	17.11	2	1	76	8.2	11.37	4.72	hypothetical protein [Arthrobacter siccitolerans]
gi517608657	11.61	2	1	155	16.9	6.13	4.72	GCN5 family N-acetyltransferase [Arthrobacter sp. 161MFSha2.1]
gi517607220	10.00	2	1	210	20.8	9.66	4.72	hypothetical protein [Arthrobacter sp. 161MFSha2.1]
gi651493690	2.74	1	1	511	54.4	5.57	4.72	sugar ABC transporter ATPase [Arthrobacter sp. H20]
gi927296083	4.79	1	1	605	62.7	4.75	4.71	protein kinase [Arthrobacter sp. ERGS1:01]
gi403230302	2.31	3	2	1385	145.4	7.05	4.71	protein EssC [Arthrobacter sp. Rue61a]
gi742853813	10.75	5	1	186	21.2	9.91	4.71	hypothetical protein [Arthrobacter sp. W1]
gi219859630	3.79	1	1	290	29.7	5.15	4.71	orotidine 5'-phosphate decarboxylase [Arthrobacter chlorophenolicus A6]
gi737777165	3.92	1	1	408	44.0	5.24	4.71	type I-U CRISPR-associated protein Cas7 [Arthrobacter sanguinis]
gi742861086	3.73	1	1	375	39.7	5.02	4.71	glycine cleavage system protein T [Arthrobacter sp. W1]
gi636843642	4.00	2	1	250	26.0	5.17	4.71	3-hydroxybutyrate dehydrogenase [Arthrobacter sp. TB 26]
gi651431530	10.89	1	1	101	11.1	10.11	4.70	50S ribosomal protein L23 [Arthrobacter sanguinis]
gi635350882	2.19	1	1	503	56.7	5.68	4.70	putative ATP-binding protein [Arthrobacter siccitolerans]
gi651435651	7.78	3	1	180	19.5	5.02	4.70	peptidylprolyl isomerase [Arthrobacter sp. H41]
gi651437717	2.55	2	1	432	47.0	4.97	4.70	hypothetical protein [Arthrobacter sp. H14]
gi119947680	2.05	2	1	536	56.3	5.20	4.70	UDP-N-acetylmuramyl-tripeptide synthetase [Arthrobacter aurescens TC1]
gi116610164	6.23	4	1	321	33.9	6.90	4.70	Alcohol dehydrogenase, zinc-binding domain protein [Arthrobacter sp. FB24]
gi908698542	2.98	2	1	436	48.4	8.95	4.70	glycosyl transferase [Arthrobacter sp. RIT-PI-e]
gi737812606	3.96	2	1	379	42.7	9.98	4.69	transcriptional regulator, partial [Arthrobacter sp. H14]
gi654814597	8.90	1	1	146	16.4	10.78	4.69	membrane protein insertion efficiency factor YidD [Arthrobacter sp. MA-N2]
gi219858528	11.00	2	1	100	10.9	4.26	4.69	conserved hypothetical protein [Arthrobacter chlorophenolicus A6]
gi654818585	4.63	1	1	216	23.0	5.99	4.69	16S rRNA methyltransferase [Arthrobacter sp. UNC362MFTsu5.1]
gi476401666	3.18	2	1	409	42.9	5.22	4.69	ROK family protein [Arthrobacter crystallopoietes BAB-32]
gi470221548	7.69	2	1	234	24.8	5.30	4.69	succinyl-CoA:3-ketoacid-coenzyme A transferase subunit A [Arthrobacter gangotriensis Lz1y]
gi654822422	3.01	4	1	332	35.8	7.81	4.68	glycosyltransferase [Arthrobacter sp. I3]
gi654818047	7.78	2	1	180	19.3	9.44	4.68	hypothetical protein [Arthrobacter sp. UNC362MFTsu5.1]
gi908698517	3.28	3	1	671	72.7	5.30	4.68	hypothetical protein [Arthrobacter sp. RIT-PI-e]
gi917760128	4.04	3	1	396	42.3	9.58	4.68	ATP-grasp domain-containing protein [Arthrobacter sp. L77]
gi759709810	1.36	1	1	1027	104.4	5.90	4.68	protease [Arthrobacter sp. 9MFCol3.1]
gi908690794	6.38	1	1	188	20.0	5.03	4.67	TetR family transcriptional regulator [Arthrobacter sp. H41]
gi910695487	5.81	2	1	327	35.4	5.21	4.67	uncharacterized protein Rv1841c/MT1889 [Arthrobacter sp. Hiyo6]
gi116612469	1.75	1	1	857	91.4	5.38	4.67	ATPase AAA-2 domain protein [Arthrobacter sp. FB24]

gi640201864	4.97	1	1	322	34.6	4.86	4.67	aldose epimerase [Arthrobacter sp. 31Y]
gi919134865	15.45	2	1	123	13.5	5.29	4.67	hypothetical protein [Arthrobacter chlorophenolicus]
gi504874821	5.43	1	1	221	23.6	4.64	4.67	alkylmercury lyase [Arthrobacter sp. J3-40]
gi737781633	8.24	3	1	279	30.1	5.54	4.67	sulfatase modifying factor 1 (C-alpha-formyglycine- generating enzyme 1) [Arthrobacter sp. 35W]
gi651431072	3.07	1	1	522	56.5	9.28	4.67	signal recognition particle protein [Arthrobacter sanguinis]
gi542110595	24.42	3	1	86	9.5	4.41	4.67	hypothetical protein M707_00785 [Arthrobacter sp. AK-YN10]
gi359306554	6.25	2	1	304	33.6	5.12	4.67	putative hydrolase [Arthrobacter globiformis NBRC 12137]
gi760164811	9.02	6	1	122	13.4	6.51	4.66	hypothetical protein [Arthrobacter crystallopoietes]
gi723608106	5.64	1	1	195	20.7	5.53	4.66	DNA-3-methyladenine glycosylase [Arthrobacter sp. PAMC25486]
gi916781771	0.95	2	1	838	93.2	5.31	4.66	beta-mannosidase [Arthrobacter sp. 35W]
gi767259181	10.92	6	1	229	25.1	6.80	4.66	GntR family transcriptional regulator [Arthrobacter sp. IHBB 11108]
gi919219181	3.17	2	1	378	40.9	7.09	4.65	MULTISPECIES: phosphodiesterase [Arthrobacter]
gi910694737	7.06	2	1	269	29.5	10.24	4.65	hypothetical protein AHiyo6_24300, partial [Arthrobacter sp. Hiyo6]
gi674643912	3.91	1	1	537	55.1	4.42	4.65	Flagellar M-ring protein [Arthrobacter sp. 11W110_air]
gi298379389	4.80	2	1	229	25.7	5.99	4.65	polyphosphate kinase, partial [Arthrobacter sp. AB(2010)]
gi914716106	4.88	3	1	492	50.7	5.94	4.65	bifunctional N-acetylglucosamine-1-phosphate uridyltransferase/glucosamine-1-phosphate acetyltr
gi162955574	7.43	2	1	175	18.6	9.63	4.64	A3(2) glycogen metabolism cluster I [Renibacterium salmoninarum ATCC 33209]
gi767258291	2.74	3	1	438	45.5	7.14	4.64	hypothetical protein UM93_12885 [Arthrobacter sp. IHBB 11108]
gi359304805	5.70	1	1	456	47.9	10.81	4.64	hypothetical protein ARGLB_080_01200 [Arthrobacter globiformis NBRC 12137]
gi651490917	13.85	1	1	130	13.7	5.19	4.64	flagellar basal body rod protein FlgG [Arthrobacter sp. H20]
gi749400960	3.35	3	1	328	35.3	8.90	4.64	hypothetical protein M707_27200, partial [Arthrobacter sp. AK-YN10]
gi654817306	10.77	2	1	130	13.6	9.72	4.64	epimerase [Arthrobacter sp. UNC362MFTsu5.1]
gi908690931	4.84	1	1	351	37.7	5.25	4.64	phosphate starvation-inducible protein PhoH [Arthrobacter sp. H41]
gi910695207	6.06	3	1	231	24.1	8.38	4.63	hypothetical protein AHiyo6_21030 [Arthrobacter sp. Hiyo6]
gi910697394	1.83	2	1	710	77.2	5.06	4.63	mannosylglycerate hydrolase [Arthrobacter sp. Hiyo6]
gi910737646	22.33	2	1	103	10.8	6.65	4.63	acetyltransferase [Arthrobacter sp. Hiyo4]
gi470217563	2.95	1	1	407	44.4	4.92	4.63	Recombinational DNA repair ATPase (RecF pathway) [Arthrobacter gangotriensis Lz1y]
gi651488717	2.40	2	1	459	48.0	5.60	4.63	serine--pyruvate aminotransferase [Arthrobacter sp. H20]
gi765012041	6.10	2	1	295	31.3	6.43	4.63	ABC transporter [Arthrobacter sp. A3]
gi651500262	15.38	1	1	130	14.0	4.61	4.63	glyoxalase [Arthrobacter sp. 35W]
gi916691670	1.97	4	1	660	72.8	10.08	4.63	hypothetical protein [Arthrobacter castelli]
gi908697503	5.42	2	1	295	32.1	4.98	4.62	hypothetical protein [Arthrobacter sp. RIT-PI-e]
gi651440249	9.09	1	1	132	14.3	5.52	4.62	universal stress protein UspA [Arthrobacter sp. H14]
gi737812242	3.66	4	1	437	49.3	10.64	4.62	integrase [Arthrobacter sp. H14]
gi742859165	3.96	2	1	278	29.6	9.11	4.62	phosphate ABC transporter permease [Arthrobacter sp. W1]
gi910741227	5.95	2	1	168	18.8	7.34	4.62	phosphoenolpyruvate synthase [Arthrobacter sp. Hiyo4]
gi359307159	4.61	1	1	477	50.5	4.97	4.62	putative amidase [Arthrobacter globiformis NBRC 12137]
gi759763421	8.81	3	2	352	39.2	6.61	4.62	UDP-glucose 4-epimerase [Arthrobacter gangotriensis]
gi910283668	6.19	1	1	291	30.4	6.40	4.62	hypothetical protein [Arthrobacter sp. A3]
gi651488314	5.14	3	1	292	31.8	5.17	4.62	methionine aminopeptidase [Arthrobacter sp. H20]
gi470221479	1.59	2	1	502	52.8	8.06	4.61	sulfate transporter [Arthrobacter gangotriensis Lz1y]
gi359303867	1.58	1	1	696	75.2	5.41	4.61	hypothetical protein ARGLB_094_00130 [Arthrobacter globiformis NBRC 12137]
gi219858213	3.88	1	1	464	49.7	5.40	4.61	LmbE family protein [Arthrobacter chlorophenolicus A6]
gi939036314	12.38	2	1	202	22.0	5.80	4.61	hypothetical protein [Arthrobacter nitroguajacolicus]
gi917530260	10.00	3	1	120	13.2	8.91	4.61	MerR family transcriptional regulator [Arthrobacter sp. PAMC25486]
gi119948553	6.49	2	1	262	27.0	4.86	4.61	oxidoreductase, short chain dehydrogenase/reductase family [Arthrobacter aurescens TC1]
gi219857949	12.80	2	1	125	12.8	4.46	4.61	Rhodanese domain protein [Arthrobacter chlorophenolicus A6]
gi651506468	3.01	2	1	299	32.4	10.59	4.61	secretion system protein [Arthrobacter sp. 35W]
gi651457135	2.53	1	1	396	43.8	9.16	4.61	hypothetical protein [Arthrobacter sp. 35/47]
gi651438782	4.01	2	1	548	58.4	5.19	4.60	acetolactate synthase [Arthrobacter sp. H14]
gi916815814	5.41	3	1	407	44.3	9.91	4.60	transposase [Arthrobacter sp. MA-N2]
gi517600313	12.90	5	1	155	16.7	7.69	4.60	hypothetical protein [Arthrobacter sp. 162MFSha1.1]
gi908696810	3.96	3	1	530	55.2	5.78	4.60	bifunctional hydroxymethylpyrimidine kinase/phosphomethylpyrimidine kinase [Arthrobacter sp. RI
gi654823193	7.61	2	1	184	19.5	7.68	4.60	haloacid dehalogenase [Arthrobacter sp. I3]
gi307746631	3.70	2	1	297	32.5	5.54	4.59	putative N-acylneuraminate-9-phosphate synthase [Arthrobacter arilaitensis Re117]
gi759714812	3.31	1	1	302	34.2	7.21	4.59	transposition protein TniB [Arthrobacter sp. AK-YN10]
gi914717393	1.37	2	1	875	95.6	5.77	4.59	glycogen phosphorylase [Arthrobacter sp. ZBG10]
gi219861224	4.17	1	1	503	54.3	5.39	4.59	5-carboxymethyl-2-hydroxymuconate semialdehyde dehydrogenase [Arthrobacter chlorophenolicu
gi635353039	6.45	4	1	217	24.1	7.68	4.59	putative hydrolase/acyltransferase [Arthrobacter siccitolerans]

gi323470098	5.67	1	1	247	26.7	7.14	4.59	amino acid ABC transporter ATP-binding protein, PAAT family [Arthrobacter phenanthrenivorans Sp
gi737786937	6.40	2	1	297	31.9	5.95	4.59	pseudouridine synthase [Arthrobacter albus]
gi919219195	4.60	2	2	522	57.7	6.20	4.59	transcriptional regulator, CdaR [Arthrobacter sp. YC-RL1]
gi162952380	2.84	1	1	493	54.0	5.07	4.59	peptide synthetase [Renibacterium salmoninarum ATCC 33209]
gi910739709	1.70	3	1	1057	113.1	5.52	4.59	protein HelA [Arthrobacter sp. Hiyo4]
gi521040827	5.26	1	1	228	25.7	5.31	4.59	hypothetical protein [Arthrobacter sp. M2012083]
gi917739847	0.93	3	1	752	82.7	4.79	4.58	protease [Arthrobacter sp. W1]
gi359306964	3.81	1	1	603	64.8	6.86	4.58	putative peptide ABC transporter ATP-binding protein [Arthrobacter globiformis NBRC 12137]
gi759704530	2.40	1	1	499	54.3	5.74	4.58	hypothetical protein [Arthrobacter globiformis]
gi910737672	16.54	2	1	127	12.9	5.06	4.58	putative phosphoenolpyruvate synthase [Arthrobacter sp. Hiyo4]
gi723607483	1.03	1	1	1266	142.9	6.23	4.58	hypothetical protein ART_1260 [Arthrobacter sp. PAMC25486]
gi917442037	3.42	4	1	556	61.9	9.73	4.58	hypothetical protein [Arthrobacter albus]
gi910249052	3.77	1	1	212	23.0	6.64	4.58	hypothetical protein [Arthrobacter siccitolerans]
gi640203058	3.69	3	1	379	39.3	5.24	4.58	bifunctional diaminohydroxyphosphoribosylaminopyrimidine deaminase/5-amino-6-(5-phosphoribo
gi648575002	2.81	1	1	677	67.8	4.89	4.57	penicillin-binding protein [Arthrobacter sp. 131MFCol6.1]
gi927034035	4.03	1	1	397	42.1	6.96	4.57	hypothetical protein AFL94_07855 [Arthrobacter sp. LS16]
gi636847135	3.39	1	1	766	83.5	4.92	4.57	prolyl oligopeptidase [Arthrobacter sp. TB 26]
gi476401490	5.63	1	1	231	25.6	5.50	4.57	hypothetical protein D477_008403 [Arthrobacter crystallopoietes BAB-32]
gi542110540	5.70	1	1	386	42.1	5.35	4.56	acyl-CoA dehydrogenase [Arthrobacter sp. AK-YN10]
gi939050147	4.61	1	1	477	53.6	8.21	4.56	hypothetical protein [Arthrobacter sp. JCM 19049]
gi651440016	1.33	1	1	678	75.4	5.27	4.56	DNA topoisomerase IV subunit B [Arthrobacter sp. H14]
gi917022313	3.67	2	1	627	63.0	4.72	4.56	hypothetical protein [Arthrobacter sp. UNC362MFTsu5.1]
gi652424303	4.38	1	1	571	60.1	4.70	4.56	DNA repair protein RecN [Arthrobacter castelli]
gi674646957	2.17	2	1	828	87.7	7.01	4.56	hypothetical protein BN1051_03170 [Arthrobacter sp. 11W110_air]
gi674644475	3.40	4	1	353	36.4	5.87	4.56	Lipopolysaccharide core heptosyltransferase RfaQ [Arthrobacter sp. 11W110_air]
gi654823113	7.87	5	1	254	27.8	6.60	4.56	transcriptional regulator [Arthrobacter sp. I3]
gi652425579	5.25	2	1	324	32.5	5.02	4.56	hypothetical protein [Arthrobacter castelli]
gi636845685	6.23	3	1	321	33.8	6.46	4.55	NADPH--quinone reductase [Arthrobacter sp. TB 26]
gi651498021	6.21	6	1	338	34.5	6.28	4.55	uroporphyrin-III methyltransferase [Arthrobacter sp. 35W]
gi323467788	6.69	2	1	299	31.8	5.00	4.55	dehydrogenase of unknown specificity, short-chain alcohol dehydrogenase -like protein [Arthrobac
gi742859715	2.03	2	1	492	53.0	5.08	4.55	hypothetical protein [Arthrobacter sp. W1]
gi476401614	2.53	2	1	712	74.7	4.73	4.55	penicillin-binding protein [Arthrobacter crystallopoietes BAB-32]
gi648224420	6.30	1	1	254	27.3	7.14	4.55	IclR family transcriptional regulator [Arthrobacter sp. M2012083]
gi927293823	4.97	1	1	302	32.3	5.81	4.55	LysR family transcriptional regulator [Arthrobacter sp. ERGS1:01]
gi219860176	2.46	1	1	406	45.1	5.68	4.55	aminotransferase class I and II [Arthrobacter chlorophenolicus A6]
gi518313564	2.75	2	1	437	46.5	6.39	4.55	hypothetical protein [Arthrobacter sp. TB 23]
gi651480606	8.21	4	1	195	21.1	9.76	4.55	serine acetyltransferase [Arthrobacter sp. Br18]
gi737787128	2.93	1	1	409	43.9	5.12	4.55	hypothetical protein [Arthrobacter albus]
gi403229836	5.96	3	1	151	16.4	4.41	4.55	hypothetical protein ARUE_c23570 [Arthrobacter sp. Rue61a]
gi654813369	4.43	3	1	406	43.5	10.18	4.54	pseudouridine synthase [Arthrobacter sp. MA-N2]
gi307744265	6.94	2	1	144	16.0	5.19	4.54	ribosome-binding factor A [Arthrobacter arilaitensis Re117]
gi403231189	5.17	1	1	348	36.7	5.63	4.54	D-3-phosphoglycerate dehydrogenase SerA [Arthrobacter sp. Rue61a]
gi742758541	4.78	4	1	356	36.9	6.70	4.54	tRNA(Ile)-lysidine synthetase [Arthrobacter phenanthrenivorans]
gi654827224	5.32	4	1	357	38.3	5.39	4.54	alkene reductase [Arthrobacter sp. H5]
gi916820598	5.50	1	1	400	41.5	4.78	4.54	hypothetical protein [Arthrobacter sp. H20]
gi651432327	9.32	2	1	161	17.8	9.86	4.54	hypothetical protein [Arthrobacter sp. H41]
gi651430546	4.17	1	1	480	50.9	5.48	4.54	inosine-5-monophosphate dehydrogenase [Arthrobacter sanguinis]
gi470216033	3.64	2	1	467	49.3	7.28	4.54	hypothetical protein ADIAG_03707 [Arthrobacter gangotriensis Lz1y]
gi403231331	3.05	1	1	722	76.8	9.44	4.54	hypothetical protein ARUE_c38750 [Arthrobacter sp. Rue61a]
gi651504544	3.78	2	1	423	44.2	9.91	4.54	MFS transporter [Arthrobacter sp. 35W]
gi767259345	1.87	2	1	587	63.4	5.52	4.53	hypothetical protein UM93_16710 [Arthrobacter sp. IHBB 11108]
gi916691613	3.88	4	1	515	56.1	5.27	4.53	hypothetical protein [Arthrobacter castelli]
gi910251186	8.25	3	1	206	22.5	9.82	4.53	CDP-diacylglycerol--glycerol-3-phosphate 3-phosphatidyltransferase [Arthrobacter siccitolerans]
gi910249564	3.62	1	1	276	29.9	4.97	4.53	metal-dependent hydrolase [Arthrobacter siccitolerans]
gi654812960	2.77	1	1	794	84.5	6.90	4.53	transcription accessory protein [Arthrobacter sp. MA-N2]
gi786027369	9.14	3	2	350	34.2	5.54	4.53	glycoside hydrolase, partial [Arthrobacter chlorophenolicus]
gi910251228	3.61	2	1	416	43.2	7.75	4.53	transporter [Arthrobacter siccitolerans]
gi639131099	10.75	4	1	186	21.1	10.33	4.53	integrase, partial [Arthrobacter sp. CAL618]
gi359307485	3.57	2	1	504	55.8	7.20	4.53	putative Xre family DNA-binding protein [Arthrobacter globiformis NBRC 12137]

gi640200456	5.11	1	1	470	51.0	5.72	4.53	two-component system sensor histidine kinase [Arthrobacter sp. 31Y]
gi742758708	2.96	1	1	507	52.0	5.86	4.53	aminopeptidase A [Arthrobacter phenanthrenivorans]
gi651501468	4.36	4	1	298	31.4	9.70	4.53	LysR family transcriptional regulator [Arthrobacter sp. 35W]
gi640195791	2.76	2	1	724	77.7	5.88	4.53	cold-shock protein [Arthrobacter sp. 31Y]
gi636843731	2.25	1	1	978	110.7	5.47	4.53	restriction endonuclease subunit R [Arthrobacter sp. TB 26]
gi910744953	3.28	2	1	335	35.5	6.54	4.52	conserved hypothetical protein [Arthrobacter sp. Hiyo8]
gi517592127	5.56	2	1	324	34.0	9.47	4.52	hypothetical protein [Arthrobacter sp. 135MFCol5.1]
gi916869992	7.57	2	1	185	19.5	5.55	4.52	hypothetical protein [Arthrobacter sp. Br18]
gi674644781	4.90	1	1	204	21.8	6.95	4.52	HTH-type transcriptional repressor KstR2 [Arthrobacter sp. 11W110_air]
gi910283552	5.54	2	1	343	36.8	5.74	4.52	hypothetical protein [Arthrobacter sp. A3]
gi551254435	3.09	4	1	453	46.5	5.95	4.52	gluconate transporter [Arthrobacter sp. PAO19]
gi939036636	3.09	2	1	259	29.7	5.11	4.52	hypothetical protein [Arthrobacter nitroguajacolicus]
gi323470420	5.54	1	1	307	33.7	7.25	4.52	ABC-type multidrug transport system, ATPase component [Arthrobacter phenanthrenivorans Sphe3
gi759772091	2.23	1	1	539	59.0	5.44	4.52	Pup deamidase/depupylase [Arthrobacter sp. SPG23]
gi723607820	16.09	4	1	87	10.0	9.16	4.51	hypothetical protein ART_1597 [Arthrobacter sp. PAMC25486]
gi910252173	6.90	2	1	145	16.1	4.96	4.51	dihydroneopterin aldolase [Arthrobacter siccitolerans]
gi918265053	5.96	1	1	285	30.6	4.75	4.51	PP2C-family Ser/Thr phosphatase, partial [Arthrobacter sp. Hiyo1]
gi759730475	2.40	2	1	458	48.8	4.96	4.51	cystathionine beta-synthase [Arthrobacter sp. L77]
gi759718215	1.56	1	1	641	66.1	5.24	4.51	cobalt ABC transporter ATP-binding protein [Arthrobacter sp. FB24]
gi759732599	5.65	2	1	248	27.6	5.30	4.51	phosphoglycerate mutase [Arthrobacter sp. L77]
gi518313839	5.32	1	1	357	37.5	5.91	4.51	hypothetical protein [Arthrobacter sp. TB 23]
gi542110117	1.95	1	1	461	50.2	5.06	4.51	hypothetical protein M707_02010 [Arthrobacter sp. AK-YN10]
gi767258029	10.64	1	1	235	25.6	4.92	4.51	hypothetical protein UM93_11015 [Arthrobacter sp. IHBB 11108]
gi674645204	4.12	1	1	486	50.8	7.01	4.51	hypothetical protein BN1051_01382 [Arthrobacter sp. 11W110_air]
gi767258843	3.71	1	1	431	48.0	5.67	4.51	hypothetical protein UM93_16875 [Arthrobacter sp. IHBB 11108]
gi737813029	4.92	1	1	305	34.1	7.91	4.51	NUDIX hydrolase [Arthrobacter sp. H14]
gi910739283	3.16	2	1	316	35.3	7.28	4.51	hypothetical protein AHiyo4_21050 [Arthrobacter sp. Hiyo4]
gi910743346	6.09	1	1	345	37.7	6.27	4.50	lactation elevated protein 1 [Arthrobacter sp. Hiyo8]
gi767257408	5.14	1	1	331	35.8	5.69	4.50	2-oxoisovalerate dehydrogenase [Arthrobacter sp. IHBB 11108]
gi927294444	3.17	2	1	378	40.3	6.55	4.50	spermidine/putrescine ABC transporter ATP-binding protein [Arthrobacter sp. ERGS1:01]
gi515766209	4.57	1	1	350	38.4	5.81	4.50	hypothetical protein [Arthrobacter sp. M2012083]
gi749402230	12.77	2	1	188	19.6	7.52	4.50	hypothetical protein M707_19360 [Arthrobacter sp. AK-YN10]
gi307748659	2.19	1	1	960	98.0	10.10	4.50	putative ABC transporter [Arthrobacter globiformis]
gi917530464	3.14	2	1	542	59.0	6.73	4.50	long-chain fatty acid--CoA ligase [Arthrobacter sp. PAMC25486]
gi542110744	5.37	1	1	335	36.0	5.54	4.50	ATPase AAA [Arthrobacter sp. AK-YN10]
gi918221918	11.86	2	2	194	20.9	5.24	4.50	flavin reductase [Arthrobacter sp. I3]
gi651439156	6.40	4	1	172	19.3	5.68	4.50	hypothetical protein [Arthrobacter sp. H14]
gi674645331	5.50	1	1	309	33.3	6.46	4.49	GTPase Era [Arthrobacter sp. 11W110_air]
gi654816793	5.11	1	1	235	24.6	4.79	4.49	hypothetical protein [Arthrobacter sp. UNC362MFTsu5.1]
gi760113122	4.82	1	1	249	27.7	6.18	4.49	hypothetical protein [Arthrobacter chlorophenolicus]
gi219858640	3.63	1	1	303	32.8	5.02	4.49	conserved hypothetical protein [Arthrobacter chlorophenolicus A6]
gi359306351	3.74	1	1	428	46.0	10.21	4.49	hypothetical protein ARGLB_039_00620 [Arthrobacter globiformis NBRC 12137]
gi651473745	2.54	1	1	355	37.4	11.22	4.49	fusaric acid resistance protein [Arthrobacter nicotinovorans]
gi674646087	6.41	3	1	312	34.7	9.55	4.49	Diadenosine hexaphosphate hydrolase [Arthrobacter sp. 11W110_air]
gi654826469	9.23	2	1	130	14.3	7.61	4.49	hypothetical protein [Arthrobacter sp. H5]
gi917759998	1.72	1	1	757	79.5	5.20	4.49	hypothetical protein [Arthrobacter sp. L77]
gi764161672	13.19	2	1	144	16.2	5.38	4.49	hypothetical protein ArV1_071 [Arthrobacter phage vB_ArtM-ArV1]
gi914713789	5.73	1	1	157	16.9	6.19	4.49	ArsR family transcriptional regulator [Arthrobacter sp. ZBG10]
gi737765222	2.08	1	1	626	68.3	5.66	4.49	MULTISPECIES: aspartyl-tRNA synthetase [Arthrobacter]
gi928485884	2.29	1	1	393	43.1	6.04	4.49	hypothetical protein AOC05_00720 [Arthrobacter alpinus]
gi323468096	1.93	1	1	1034	104.8	5.36	4.49	PA domain-containing protein [Arthrobacter phenanthrenivorans Sphe3]
gi651502398	2.33	1	1	514	54.2	7.01	4.48	hypothetical protein [Arthrobacter sp. 35W]
gi517598843	2.15	1	1	558	61.1	5.21	4.48	AMP-binding protein [Arthrobacter sp. 162MFSha1.1]
gi742072098	5.32	1	1	395	41.9	5.45	4.48	oxidoreductase family, NAD-binding Rossmann fold protein [Arthrobacter sp. MWB30]
gi914716984	4.93	2	1	203	21.9	8.88	4.48	TetR family transcriptional regulator [Arthrobacter sp. ZBG10]
gi908698500	6.91	2	1	188	19.6	10.30	4.48	Holliday junction resolvase [Arthrobacter sp. RIT-PI-e]
gi654811276	12.40	1	1	129	13.8	5.86	4.48	MerR family transcriptional regulator [Arthrobacter sp. MA-N2]
gi651486191	3.52	2	1	284	29.7	5.50	4.48	NAD(P)-dependent oxidoreductase [Arthrobacter sp. Br18]
gi651466199	8.23	1	1	231	24.9	5.43	4.48	GntR family transcriptional regulator [Arthrobacter sp. 35/47]

gi910248802	7.20	2	1	236	25.8	5.27	4.47	hypothetical protein [Arthrobacter siccitolerans]
gi517606802	12.66	2	1	158	16.7	9.60	4.47	hypothetical protein [Arthrobacter sp. 161MFSha2.1]
gi654826641	3.50	1	1	429	45.8	4.98	4.47	nucleotide sugar dehydrogenase [Arthrobacter sp. H5]
gi723606332	3.30	1	1	606	64.6	5.17	4.47	putative cell division protein [Arthrobacter sp. PAMC25486]
gi759725678	4.27	1	1	445	45.8	10.62	4.47	MATE family efflux transporter [Arthrobacter sp. I3]
gi927031007	1.37	2	1	873	97.0	5.26	4.47	DNA gyrase subunit A [Arthrobacter sp. LS16]
gi765009339	3.23	1	1	589	61.9	6.46	4.47	preprotein translocase subunit SecD [Arthrobacter sp. A3]
gi928488406	10.71	2	1	196	20.5	4.68	4.47	hypothetical protein AOC05_17745 [Arthrobacter alpinus]
gi767257176	4.93	1	1	446	48.2	6.57	4.47	ABC transporter substrate-binding protein [Arthrobacter sp. IHBB 11108]
gi917013323	6.10	1	1	164	18.1	9.06	4.47	hypothetical protein [Arthrobacter sanguinis]
gi737781252	6.49	1	1	339	36.1	6.90	4.47	oxidoreductase [Arthrobacter sp. 35W]
gi654814280	1.93	1	1	518	55.2	10.10	4.47	hypothetical protein [Arthrobacter sp. MA-N2]
gi737802279	5.06	1	1	395	42.1	10.33	4.47	ATP-grasp domain-containing protein [Arthrobacter castelli]
gi654827885	3.42	1	1	497	55.0	5.26	4.46	mannitol dehydrogenase [Arthrobacter sp. H5]
gi476398924	9.09	1	1	220	23.9	6.65	4.46	response regulator of citrate/malate metabolism [Arthrobacter crystallopoietes BAB-32]
gi737777245	5.77	2	1	312	32.8	4.75	4.46	hypothetical protein [Arthrobacter sanguinis]
gi908690510	3.47	2	2	749	83.6	9.14	4.46	GTP pyrophosphokinase [Arthrobacter sp. H41]
gi674645613	3.94	1	1	330	34.9	4.65	4.46	dihydroxyacetone kinase subunit DhaL [Arthrobacter sp. 11W110_air]
gi823667331	2.54	1	1	511	52.5	5.71	4.46	uracil permease [Arthrobacter sp. YC-RL1]
gi476401923	2.23	1	1	629	66.6	5.77	4.46	ABC transporter [Arthrobacter crystallopoietes BAB-32]
gi765010234	11.76	1	1	187	19.8	6.01	4.46	GNAT family N-acetyltransferase [Arthrobacter sp. A3]
gi767258125	2.02	1	1	446	47.1	9.52	4.45	MFS transporter [Arthrobacter sp. IHBB 11108]
gi551255800	3.02	1	1	364	37.2	5.39	4.45	alcohol dehydrogenase [Arthrobacter sp. PAO19]
gi823668552	13.51	1	1	74	8.5	5.22	4.45	hypothetical protein AA310_01070 [Arthrobacter sp. YC-RL1]
gi403228525	22.89	1	1	83	9.3	11.74	4.45	putative integral membrane protein [Arthrobacter sp. Rue61a]
gi162954293	27.84	1	1	97	10.0	5.17	4.45	putative carboxylesterase type B [Renibacterium salmoninarum ATCC 33209]
gi170783547	10.95	1	1	137	15.0	5.74	4.45	putative chromosome segregation ATPase (plasmid) [Arthrobacter sp. Chr15]
gi740684044	4.73	1	1	402	41.8	5.35	4.45	acetyl-CoA acetyltransferase [Arthrobacter sp. PAMC25486]
gi651453983	4.90	1	1	306	33.5	5.21	4.45	chromosome replication initiation inhibitor protein [Arthrobacter nicotinovorans]
gi116609878	1.68	1	1	831	87.5	9.99	4.44	transglutaminase domain protein [Arthrobacter sp. FB24]
gi908699293	8.15	1	1	233	25.6	6.43	4.44	cobalt ABC transporter ATP-binding protein [Arthrobacter sp. RIT-PI-e]
gi765010324	3.12	1	1	449	48.1	5.01	4.44	hypothetical protein [Arthrobacter sp. A3]
gi759733747	6.14	1	1	114	13.3	6.19	4.44	DNA-binding protein [Arthrobacter sp. L77]
gi476399469	3.99	2	1	376	40.2	6.39	4.44	cation diffusion facilitator family transporter [Arthrobacter crystallopoietes BAB-32]
gi723609762	30.95	1	1	42	4.7	11.43	4.44	hypothetical protein ART_3539 [Arthrobacter sp. PAMC25486]
gi636844728	3.13	1	1	543	57.4	6.89	4.44	two-component system sensor histidine kinase [Arthrobacter sp. TB 26]
gi323467549	7.67	1	1	313	32.3	6.47	4.44	hypothetical protein Asphe3_00140 [Arthrobacter phenanthrenivorans Sphe3]
gi476399909	5.76	2	1	382	41.6	7.42	4.44	glycosyl hydrolase family 76 protein [Arthrobacter crystallopoietes BAB-32]
gi517607302	5.33	1	1	244	25.7	10.51	4.44	membrane protein [Arthrobacter sp. 161MFSha2.1]
gi753932417	5.16	1	1	213	23.5	9.66	4.43	hypothetical protein [Arthrobacter arilaitensis]
gi443483136	2.32	1	1	691	78.7	5.92	4.43	ribonucleotide-diphosphate reductase subunit alpha [Arthrobacter nitrophenolicus]
gi551255262	2.92	3	1	274	30.3	8.37	4.43	DNA glycosylase [Arthrobacter sp. PAO19]
gi470221735	8.39	1	1	155	16.7	8.25	4.43	hypothetical protein ADIAG_00588 [Arthrobacter gangotriensis Lz1y]
gi910738041	7.55	1	1	106	11.6	11.88	4.43	conserved hypothetical protein [Arthrobacter sp. Hiyo4]
gi917012982	2.78	1	1	396	40.5	5.22	4.43	sulfonate ABC transporter substrate-binding protein [Arthrobacter sanguinis]
gi219861258	9.56	1	1	136	14.9	4.64	4.43	Rhodanese domain protein [Arthrobacter chlorophenolicus A6]
gi767256852	4.86	1	1	288	30.2	6.01	4.43	3-methyl-2-oxobutanoate hydroxymethyltransferase [Arthrobacter sp. IHBB 11108]
gi651443068	10.81	3	1	185	19.3	9.99	4.43	TspO and MBR s [Arthrobacter sp. 9MFCol3.1]
gi359306165	10.34	1	1	261	27.3	6.67	4.43	hypothetical protein ARGLB_047_01120 [Arthrobacter globiformis NBRC 12137]
gi910251130	7.69	2	1	169	18.4	4.69	4.43	ribosome-binding factor A [Arthrobacter siccitolerans]
gi674645875	4.91	1	1	346	37.1	4.94	4.43	3-ketosteroid-9-alpha-hydroxylase reductase subunit [Arthrobacter sp. 11W110_air]
gi116609923	2.54	1	1	354	38.9	6.52	4.43	esterase/lipase [Arthrobacter sp. FB24]
gi470221180	2.49	1	1	401	42.8	4.69	4.43	neutral protease [Arthrobacter gangotriensis Lz1y]
gi443483131	1.87	1	1	588	62.2	6.74	4.43	thioredoxin reductase [Arthrobacter nitrophenolicus]
gi654826513	3.50	4	1	457	48.4	5.08	4.43	serine ammonia-lyase [Arthrobacter sp. H5]
gi910746790	10.59	1	1	85	9.6	6.06	4.43	hypothetical protein AHiyo8_44920 [Arthrobacter sp. Hiyo8]
gi723609639	0.95	1	1	1160	125.0	5.02	4.43	hypothetical protein ART_3416 [Arthrobacter sp. PAMC25486]
gi674646846	3.41	1	1	411	44.8	5.57	4.43	DNA polymerase III subunit epsilon [Arthrobacter sp. 11W110_air]
gi759724350	6.44	1	1	295	31.9	5.86	4.43	hypothetical protein [Arthrobacter sp. I3]

gi742859631	11.20	1	1	125	13.8	4.84	4.42	(2Fe-2S)-binding protein [Arthrobacter sp. W1]
gi918266794	2.66	1	1	602	66.0	5.20	4.42	glutamate synthase [NADPH] large chain [Arthrobacter sp. Hiyo1]
gi928988535	4.63	1	1	216	21.8	5.01	4.42	hypothetical protein [Arthrobacter sp. ERGS1:01]
gi918469374	8.90	1	1	292	29.3	9.98	4.42	ABC transporter [Arthrobacter crystallopoietes]
gi918266866	13.95	1	1	86	9.8	4.56	4.42	hypothetical protein AHiyo1_33170 [Arthrobacter sp. Hiyo1]
gi786033256	6.44	1	1	202	22.8	7.50	4.42	hypothetical protein [Arthrobacter chlorophenolicus]
gi517603923	3.01	1	1	697	73.8	8.88	4.42	hypothetical protein [Arthrobacter sp. 131MFCol6.1]
gi542110312	9.80	2	1	245	25.8	6.80	4.41	ArsR family transcriptional regulator [Arthrobacter sp. AK-YN10]
gi648224655	6.29	1	1	159	17.7	5.81	4.41	DNA mismatch repair protein MutT [Arthrobacter sp. M2012083]
gi757624600	4.24	1	1	543	59.3	5.07	4.41	hypothetical protein TV39_07765 [Arthrobacter sp. SPG23]
gi476401048	4.03	3	1	571	62.4	7.42	4.41	Cobaltochelatase [Arthrobacter crystallopoietes BAB-32]
gi651505268	3.29	2	1	487	52.1	8.90	4.41	amino acid permease [Arthrobacter sp. 35W]
gi323470115	6.96	2	1	316	33.9	8.48	4.41	ABC-type dipeptide/oligopeptide/nickel transport system, permease component [Arthrobacter phe
gi753934229	1.37	3	1	1537	164.8	5.59	4.41	glutamate synthase [Arthrobacter arilaitensis]
gi742855613	2.81	1	1	570	63.0	5.17	4.41	AMP-dependent synthetase [Arthrobacter sp. W1]
gi639129377	8.37	1	1	263	28.2	8.56	4.41	MULTISPECIES: ABC transporter permease [Arthrobacter]
gi323468785	6.03	4	1	199	22.1	9.41	4.41	uncharacterized conserved protein [Arthrobacter phenanthrenivorans Sphe3]
gi910737939	5.51	1	1	236	25.1	6.05	4.40	27 kDa antigen Cfp30B [Arthrobacter sp. Hiyo4]
gi823667914	2.78	1	1	396	42.4	5.99	4.40	peptidase M19 [Arthrobacter sp. YC-RL1]
gi323469718	3.05	1	1	459	47.8	5.43	4.40	glutamate-5-semialdehyde dehydrogenase [Arthrobacter phenanthrenivorans Sphe3]
gi162953107	7.51	1	1	253	27.2	9.01	4.40	ABC transporter ATP-binding protein [Renibacterium salmoninarum ATCC 33209]
gi914716033	7.78	1	1	270	27.6	5.62	4.40	hypothetical protein [Arthrobacter sp. ZBG10]
gi489902867	4.78	1	1	209	23.1	6.55	4.40	TetR family transcriptional regulator [Arthrobacter globiformis]
gi359305230	8.04	2	1	286	30.4	5.00	4.40	hypothetical protein ARGLB_073_00820 [Arthrobacter globiformis NBRC 12137]
gi723606908	8.57	4	1	245	27.1	8.73	4.40	hypothetical protein ART_0685 [Arthrobacter sp. PAMC25486]
gi939050386	5.49	2	1	273	32.1	10.11	4.40	lipase [Arthrobacter sp. JCM 19049]
gi443482349	4.46	2	1	538	58.0	9.38	4.40	ABC transporter ATP-binding protein [Arthrobacter nitrophenolicus]
gi823666983	2.44	3	1	860	90.6	5.64	4.40	nitrite reductase [Arthrobacter sp. YC-RL1]
gi470217081	2.27	2	1	485	53.5	6.73	4.39	hypothetical protein ADIAG_02842 [Arthrobacter gangotriensis Lz1y]
gi323470444	7.47	1	1	241	25.2	5.48	4.39	response regulator containing a CheY-like receiver domain and an HTH DNA-binding domain prote
gi652424059	4.53	2	1	265	27.7	4.55	4.39	hydroxypyruvate isomerase [Arthrobacter castelli]
gi654812683	3.08	1	1	292	30.6	8.92	4.39	DSBA oxidoreductase [Arthrobacter sp. MA-N2]
gi765012969	9.52	1	1	210	23.0	5.06	4.39	hypothetical protein [Arthrobacter sp. A3]
gi759718418	4.97	5	1	362	38.6	5.14	4.39	agmatinase [Arthrobacter sp. FB24]
gi910252332	5.97	2	1	134	14.3	11.53	4.39	hypothetical protein [Arthrobacter siccitolerans]
gi443483301	6.78	1	1	295	30.7	5.60	4.39	short chain dehydrogenase [Arthrobacter nitrophenolicus]
gi749401979	8.02	6	1	237	26.2	5.63	4.39	GntR family transcriptional regulator [Arthrobacter sp. AK-YN10]
gi757625792	11.21	2	1	214	23.2	6.81	4.39	ArsR family transcriptional regulator [Arthrobacter sp. SPG23]
gi652424487	4.24	1	1	543	56.3	5.20	4.38	thiamine pyrophosphate-binding protein [Arthrobacter castelli]
gi654828673	2.60	1	1	462	49.9	4.88	4.38	glycosyl hydrolase family 32 [Arthrobacter sp. H5]
gi470216813	6.79	1	1	162	17.4	9.39	4.38	Holliday junction resolvase YqgF [Arthrobacter gangotriensis Lz1y]
gi443481017	1.53	1	1	786	84.0	5.54	4.38	beta-galactosidase [Arthrobacter nitrophenolicus]
gi640194351	10.57	1	1	123	14.3	5.22	4.38	hypothetical protein [Arthrobacter sp. 31Y]
gi737800903	6.93	3	1	231	25.0	5.02	4.38	histidine kinase [Arthrobacter castelli]
gi517598601	8.70	2	1	115	12.1	4.64	4.38	hypothetical protein [Arthrobacter sp. 162MFSha1.1]
gi742755139	2.36	8	1	593	64.5	9.57	4.38	glycosyltransferase [Arthrobacter phenanthrenivorans]
gi651440616	11.92	1	1	193	21.1	8.21	4.38	hypothetical protein [Arthrobacter sp. H14]
gi517606688	2.11	1	1	711	73.7	5.92	4.38	potassium-transporting ATPase subunit B [Arthrobacter sp. 161MFSha2.1]
gi910746537	9.02	1	1	122	12.9	11.46	4.38	histidine ammonia-lyase [Arthrobacter sp. Hiyo8]
gi910248926	9.39	1	1	181	19.6	6.64	4.38	acetyltransferase [Arthrobacter siccitolerans]
gi651493542	2.11	1	1	475	49.5	7.01	4.38	hypothetical protein [Arthrobacter sp. H20]
gi359305989	2.57	1	1	506	54.8	6.07	4.38	2-methylcitrate dehydratase [Arthrobacter globiformis NBRC 12137]
gi648224541	8.88	1	1	169	18.5	4.88	4.38	OHCU decarboxylase [Arthrobacter sp. M2012083]
gi916871866	3.13	1	1	256	28.1	5.08	4.38	hypothetical protein [Arthrobacter sp. H5]
gi916870087	4.61	1	1	304	31.8	5.02	4.38	hypothetical protein [Arthrobacter sp. Br18]
gi116610225	4.26	1	1	399	40.8	9.64	4.38	Peptidoglycan-binding LysM [Arthrobacter sp. FB24]
gi651444519	4.90	1	1	306	32.1	6.39	4.38	methionyl-tRNA formyltransferase [Arthrobacter nicotinovorans]
gi651441205	7.50	1	1	120	13.5	11.28	4.38	hypothetical protein [Arthrobacter sp. 9MFCol3.1]
gi651439277	4.43	2	2	451	48.1	4.84	4.38	MBL fold metallo-hydrolase [Arthrobacter sp. H14]

gi917760507	2.95	1	1	509	55.4	7.81	4.37	hypothetical protein [Arthrobacter sp. L77]
gi910749208	8.59	1	1	198	21.5	6.54	4.37	L-Carnitine dehydratase/bile acid-inducible protein F (plasmid) [Arthrobacter sp. Hiyo8]
gi648574295	6.17	1	1	162	18.6	10.86	4.37	transposase [Arthrobacter sp. 162MFSha1.1]
gi742758746	2.12	3	1	425	44.2	6.76	4.37	acetyl-CoA acetyltransferase [Arthrobacter phenanthrenivorans]
gi116610224	2.11	1	1	710	75.0	6.51	4.37	serine/threonine protein kinase [Arthrobacter sp. FB24]
gi542109402	4.70	1	1	383	42.1	9.86	4.37	glycosyl hydrolase [Arthrobacter sp. AK-YN10]
gi765004400	3.68	1	1	435	46.4	6.96	4.37	two-component system sensor histidine kinase [Arthrobacter sp. A3]
gi765001845	3.36	1	1	476	50.5	9.47	4.36	L-asparagine permease [Arthrobacter sp. M2012083]
gi476403137	1.77	1	1	620	67.2	7.50	4.36	glycosyl transferase family protein [Arthrobacter crystallopoietes BAB-32]
gi518313708	4.78	1	1	272	29.7	9.06	4.36	hypothetical protein [Arthrobacter sp. TB 23]
gi674645039	1.56	3	1	767	78.8	5.43	4.36	Copper-exporting P-type ATPase A [Arthrobacter sp. 11W110_air]
gi651434036	2.47	2	1	446	47.4	3.71	4.36	sugar ABC transporter substrate-binding protein [Arthrobacter sp. H41]
gi742758521	3.92	2	1	357	38.4	5.40	4.36	amidohydrolase [Arthrobacter phenanthrenivorans]
gi823666628	5.60	1	1	268	29.9	9.57	4.36	haloacid dehalogenase [Arthrobacter sp. YC-RL1]
gi651440214	1.61	1	1	682	75.2	5.34	4.36	pyruvate dehydrogenase, partial [Arthrobacter sp. H14]
gi737781414	4.50	2	1	311	33.5	9.73	4.36	recombinase XerC [Arthrobacter sp. 35W]
gi27657618	2.51	1	1	358	37.2	6.07	4.36	6-hydroxyhexanoate dehydrogenase [Arthrobacter sp. BP2]
gi470220850	3.88	2	1	258	28.0	6.70	4.36	hypothetical protein ADIAG_00796 [Arthrobacter gangotriensis Lz1y]
gi742859591	1.93	1	1	519	55.8	5.60	4.36	AMP-dependent synthetase [Arthrobacter sp. W1]
gi307745108	1.73	1	1	579	62.4	6.84	4.36	MOSC domain-containing protein [Arthrobacter arilaitensis Re117]
gi910746984	6.25	1	1	256	27.3	6.38	4.36	dihydrolipoyllysine-residue acyltransferase component of branched-chain alpha-ketoacid dehydrog
gi759734368	4.74	4	1	359	36.7	7.11	4.36	bifunctional diaminohydroxyphosphoribosylaminopyrimidine deaminase/5-amino-6-(5-phosphoribo
gi927032972	4.50	1	1	467	50.7	5.94	4.36	PucR family transcriptional regulator [Arthrobacter sp. LS16]
gi765007082	1.65	1	1	547	58.3	5.05	4.35	hypothetical protein [Arthrobacter sp. A3]
gi651432323	5.88	1	1	323	34.7	4.82	4.35	carboxypeptidase, partial [Arthrobacter sp. H41]
gi517608063	3.06	1	1	327	36.1	5.92	4.35	extradiol ring-cleavage dioxygenase [Arthrobacter sp. 161MFSha2.1]
gi664486476	4.22	1	1	308	33.2	5.44	4.35	prephenate dehydratase [Streptomyces sp. NRRL WC-3773]
gi767256719	14.88	1	1	121	12.4	4.67	4.35	ATPase [Arthrobacter sp. IHBB 11108]
gi651478935	4.37	3	1	343	37.1	5.45	4.35	dehydratase [Arthrobacter sp. Br18]
gi939050204	2.91	1	1	275	30.1	5.68	4.35	IclR family transcriptional regulator [Arthrobacter sp. JCM 19049]
gi767256843	5.43	1	1	313	33.5	6.42	4.35	pseudouridine synthase [Arthrobacter sp. IHBB 11108]
gi476399700	11.06	1	1	217	24.7	9.79	4.35	hypothetical protein D477_017354 [Arthrobacter crystallopoietes BAB-32]
gi759746066	3.78	1	1	423	45.4	8.79	4.35	SAM-dependent methyltransferase [Arthrobacter sp. 31Y]
gi651476397	3.74	3	1	348	36.2	9.91	4.35	ABC transporter permease [Arthrobacter nicotinovorans]
gi917013340	1.51	1	1	794	84.7	6.20	4.35	daunorubicin resistance protein DrrC [Arthrobacter sanguinis]
gi910748822	10.34	1	1	116	12.5	10.08	4.35	integration host factor [Arthrobacter sp. Hiyo8]
gi651505449	2.08	1	1	481	51.7	5.86	4.35	amidohydrolase [Arthrobacter sp. 35W]
gi737804243	2.33	1	1	557	58.4	6.39	4.35	oxidoreductase [Arthrobacter sp. Br18]
gi323469433	8.20	1	1	244	25.4	5.88	4.35	conserved hypothetical protein TIGR00046 [Arthrobacter phenanthrenivorans Sphe3]
gi930827884	3.06	1	1	294	32.8	5.71	4.35	protein iolH [Arthrobacter arilaitensis]
gi470217443	7.18	2	1	181	20.0	4.35	4.35	16S rRNA processing protein [Arthrobacter gangotriensis Lz1y]
gi737808825	4.11	2	1	414	44.6	5.47	4.34	two-component system sensor histidine kinase [Arthrobacter sp. H5]
gi937261801	4.05	1	1	395	41.8	4.61	4.34	multidrug ABC transporter permease [Arthrobacter sp. Edens01]
gi757625525	13.33	1	1	120	13.4	9.51	4.34	ArsR family transcriptional regulator [Arthrobacter sp. SPG23]
gi119947440	5.14	1	1	253	27.5	11.00	4.34	conserved hypothetical protein [Arthrobacter aurescens TC1]
gi910737842	16.36	1	1	55	6.3	10.43	4.34	conserved hypothetical protein [Arthrobacter sp. Hiyo4]
gi759733763	3.79	2	1	475	50.7	11.19	4.34	MFS transporter [Arthrobacter sp. L77]
gi908698566	4.45	1	1	427	44.6	5.16	4.34	Hrp-dependent type III effector protein [Arthrobacter sp. RIT-PI-e]
gi651500598	4.31	1	1	487	50.0	4.77	4.34	carbohydrate kinase [Arthrobacter sp. 35W]
gi162955241	13.85	1	1	65	6.9	7.25	4.34	Zn-dependent oxidoreductase [Renibacterium salmoninarum ATCC 33209]
gi910739493	3.06	1	1	589	62.4	5.03	4.34	transketolase [Arthrobacter sp. Hiyo4]
gi651444731	24.42	2	1	86	9.5	8.59	4.33	hypothetical protein [Arthrobacter nicotinovorans]
gi403228459	4.79	1	1	313	34.1	6.55	4.33	HTH-type transcriptional regulator [Arthrobacter sp. Rue61a]
gi651498981	7.88	1	1	292	30.7	10.17	4.33	ABC transporter permease [Arthrobacter sp. 35W]
gi742756582	8.99	1	1	267	28.4	8.79	4.33	PA-phosphatase [Arthrobacter phenanthrenivorans]
gi651445222	3.76	2	1	266	28.4	6.70	4.33	IclR family transcriptional regulator [Arthrobacter nicotinovorans]
gi635352279	9.47	1	1	95	10.7	5.33	4.33	chorismate mutase type II family protein [Arthrobacter siccitolerans]
gi307746272	4.43	2	1	384	41.4	5.44	4.33	Dyp-type peroxidase family protein [Arthrobacter arilaitensis Re117]
gi910692929	4.05	1	1	222	23.3	5.78	4.33	alcohol dehydrogenase, partial [Arthrobacter sp. Hiyo6]

gi737800823	4.02	1	1	224	25.2	5.00	4.33	hypothetical protein [Arthrobacter castelli]
gi910742951	5.30	1	1	415	44.9	5.06	4.33	carbon monoxide dehydrogenase large chain [Arthrobacter sp. Hiyo8]
gi307743639	2.94	1	1	578	62.4	6.39	4.33	putative drug resistance ATP-binding protein [Arthrobacter arilaitensis Re117]
gi917013199	1.51	2	1	727	78.7	9.36	4.32	hypothetical protein [Arthrobacter sanguinis]
gi927296253	4.31	1	1	394	41.9	6.55	4.32	hypothetical protein AL755_09415 [Arthrobacter sp. ERGS1:01]
gi928487609	7.69	1	1	169	18.0	9.36	4.32	50S ribosomal protein L17 [Arthrobacter alpinus]
gi307745398	6.84	1	1	351	37.7	5.52	4.32	uroporphyrinogen decarboxylase [Arthrobacter arilaitensis Re117]
gi651464935	3.32	1	1	482	49.9	5.31	4.32	branched-chain alpha-keto acid dehydrogenase subunit E2 [Arthrobacter sp. 35/47]
gi737788102	3.30	1	1	303	33.3	5.44	4.32	N-acetylneuraminate synthase [Arthrobacter albus]
gi651483630	7.24	2	1	304	33.8	6.54	4.32	NUDIX hydrolase [Arthrobacter sp. Br18]
gi737786778	9.57	1	1	230	24.7	4.61	4.32	potassium transporter TrkA [Arthrobacter albus]
gi918265278	3.08	1	1	422	47.7	5.91	4.32	conserved hypothetical protein [Arthrobacter sp. Hiyo1]
gi476399181	3.01	1	1	399	44.7	5.43	4.32	cytochrome P450 [Arthrobacter crystallopoietes BAB-32]
gi517592545	2.70	1	1	444	48.1	5.54	4.32	sugar-binding protein [Arthrobacter sp. 135MFCol5.1]
gi470216695	4.11	1	1	414	43.1	5.27	4.32	acetylornithine and succinylornithine aminotransferase [Arthrobacter gangotriensis Lz1y]
gi470216534	2.63	2	1	570	62.2	6.11	4.31	ABC-1 domain-containing protein [Arthrobacter gangotriensis Lz1y]
gi737787792	2.35	1	1	425	47.9	5.64	4.31	hypothetical protein [Arthrobacter albus]
gi359307509	6.15	1	1	325	35.4	5.87	4.31	transcription elongation protein NusA [Arthrobacter globiformis NBRC 12137]
gi910744511	4.21	1	1	214	23.2	5.96	4.31	inositol 2-dehydrogenase 1 [Arthrobacter sp. Hiyo8]
gi648224688	3.61	1	1	332	37.3	5.86	4.31	dTDP-glucose 4,6-dehydratase [Arthrobacter sp. M2012083]
gi910696567	4.66	1	1	429	45.4	6.29	4.31	trehalose/maltose-binding protein MalE [Arthrobacter sp. Hiyo6]
gi640193335	9.55	1	1	220	22.8	5.39	4.31	acetyltransferase [Arthrobacter sp. 31Y]
gi219861505	4.09	1	1	513	55.6	10.36	4.31	exopolysaccharide biosynthesis polyprenyl glycosylphosphotransferase [Arthrobacter chlorophenol
gi651456922	5.29	1	1	340	36.3	9.47	4.31	hypothetical protein [Arthrobacter sp. 35/47]
gi654828653	6.37	1	1	267	28.5	5.97	4.30	3-ketoacyl-ACP reductase [Arthrobacter sp. H5]
gi476399963	0.49	2	1	2025	208.6	4.58	4.30	hypothetical protein D477_016055 [Arthrobacter crystallopoietes BAB-32]
gi307745329	2.34	1	1	256	27.2	9.50	4.30	hypothetical membrane protein [Arthrobacter arilaitensis Re117]
gi651452638	1.03	2	1	1068	116.7	6.14	4.30	hypothetical protein [Arthrobacter nicotinovorans]
gi359304561	6.13	1	1	326	35.8	6.81	4.30	hypothetical protein ARGLB_083_00870 [Arthrobacter globiformis NBRC 12137]
gi690772838	3.65	3	1	384	41.9	5.94	4.30	hypothetical protein HMPREF2128_05975 [Arthrobacter albus DNF00011]
gi742751680	2.84	1	1	599	63.3	5.43	4.30	ATPase [Arthrobacter phenanthrenivorans]
gi654817616	8.13	1	1	160	17.1	9.04	4.30	MarR family transcriptional regulator [Arthrobacter sp. UNC362MFTsu5.1]
gi749401596	4.61	1	1	217	23.8	5.90	4.30	hypothetical protein M707_22775 [Arthrobacter sp. AK-YN10]
gi651429666	8.39	4	1	310	33.1	7.99	4.30	chitinase [Arthrobacter sanguinis]
gi769940623	9.94	2	1	181	19.2	6.55	4.30	Holliday junction DNA helicase [Arthrobacter sp. IHBB 11108]
gi937262373	7.12	1	1	267	28.8	9.47	4.30	cell division ATP-binding protein FtsE [Arthrobacter sp. Edens01]
gi908699039	3.02	1	1	331	36.2	5.21	4.29	alpha-L-glutamate ligase [Arthrobacter sp. RIT-PI-e]
gi927292783	3.34	1	1	389	40.4	6.19	4.29	FAD-dependent oxidoreductase (plasmid) [Arthrobacter sp. ERGS1:01]
gi910743902	8.61	1	1	151	16.0	9.42	4.29	acetyl-/propionyl-coenzyme A carboxylase alpha chain [Arthrobacter sp. Hiyo8]
gi742072061	7.58	2	1	211	23.6	7.12	4.29	hypothetical protein ANMWB30_10050 [Arthrobacter sp. MWB30]
gi918269267	4.43	1	1	361	41.1	7.08	4.29	cell division cycle protein 48 homolog AF_1297 [Arthrobacter sp. Hiyo1]
gi651442207	9.62	1	1	156	16.7	4.92	4.29	ribose 5-phosphate isomerase [Arthrobacter sp. 9MFCol3.1]
gi476401820	12.42	3	1	153	17.8	8.91	4.29	MarR family transcriptional regulator [Arthrobacter crystallopoietes BAB-32]
gi916926373	2.03	2	1	492	54.2	9.73	4.29	hypothetical protein [Arthrobacter sp. 9MFCol3.1]
gi517601810	2.94	1	1	544	58.2	6.11	4.29	histidine kinase [Arthrobacter sp. 162MFSha1.1]
gi914714120	3.46	1	1	318	33.8	6.81	4.29	XRE family transcriptional regulator [Arthrobacter sp. ZBG10]
gi742857478	5.38	1	1	260	28.0	5.87	4.29	ABC transporter [Arthrobacter sp. W1]
gi640194283	3.32	1	1	361	39.3	5.45	4.29	hypothetical protein [Arthrobacter sp. 31Y]
gi908698015	9.52	1	1	147	15.9	6.55	4.29	hypothetical protein [Arthrobacter sp. RIT-PI-e]
gi737809102	12.60	1	1	127	14.1	5.57	4.28	GntR family transcriptional regulator [Arthrobacter sp. H5]
gi651441065	4.57	1	1	525	56.0	6.68	4.28	ABC transporter [Arthrobacter sp. 9MFCol3.1]
gi674644324	3.11	1	1	515	55.5	9.36	4.28	D-alanyl-D-alanine carboxypeptidase precursor [Arthrobacter sp. 11W110_air]
gi939051279	9.71	2	1	103	11.6	4.59	4.28	hypothetical protein [Arthrobacter sp. JCM 19049]
gi307745578	36.76	1	1	68	7.3	10.37	4.28	50S ribosomal protein L30 [Arthrobacter arilaitensis Re117]
gi910748423	10.05	1	1	199	20.7	11.02	4.28	aminoglycoside phosphotransferase [Arthrobacter sp. Hiyo8]
gi917746045	3.01	1	1	564	58.1	5.19	4.28	hypothetical protein [Arthrobacter phenanthrenivorans]
gi517598081	5.56	1	1	360	38.8	6.20	4.28	amidohydrolase [Arthrobacter sp. 162MFSha1.1]
gi359307841	3.65	1	1	466	47.8	8.16	4.28	hypothetical protein ARGLB_008_01390 [Arthrobacter globiformis NBRC 12137]
gi759724580	8.22	1	1	219	23.4	11.46	4.28	hypothetical protein [Arthrobacter sp. I3]

gi737800297	9.21	1	1	152	16.9	4.88	4.28	GNAT family N-acetyltransferase [Arthrobacter castelli]
gi651462992	12.14	4	1	206	22.5	9.58	4.27	resolvase [Arthrobacter sp. 35/47]
gi914715291	2.45	2	1	327	32.9	11.36	4.27	hypothetical protein [Arthrobacter sp. ZBG10]
gi517609423	3.52	3	1	341	35.9	5.05	4.27	dehydrogenase [Arthrobacter sp. 161MFSha2.1]
gi162954002	2.18	1	1	733	79.1	5.54	4.27	RecG [Renibacterium salmoninarum ATCC 33209]
gi359304279	5.96	2	1	218	23.0	5.64	4.27	putative oxidoreductase [Arthrobacter globiformis NBRC 12137]
gi648575164	6.34	1	1	284	31.1	9.28	4.27	ABC transporter [Arthrobacter sp. 161MFSha2.1]
gi937262651	4.11	3	1	365	40.5	5.49	4.27	hypothetical protein AO716_00485 [Arthrobacter sp. Edens01]
gi648224518	5.82	1	1	378	40.5	5.20	4.27	Zn-dependent hydrolase, partial [Arthrobacter sp. M2012083]
gi742071201	3.98	1	1	226	25.1	6.20	4.26	hypothetical protein ANMWB30_24000 [Arthrobacter sp. MWB30]
gi742071743	3.93	1	1	433	45.9	5.53	4.26	ABC transporter substrate-binding protein YesO [Arthrobacter sp. MWB30]
gi307743476	1.72	1	1	464	51.3	5.06	4.26	signal transduction histidine kinase [Arthrobacter arilaitensis Re117]
gi219859347	2.36	1	1	721	75.5	6.51	4.26	K+-transporting ATPase, B subunit [Arthrobacter chlorophenolicus A6]
gi651447030	8.47	1	1	295	31.9	7.36	4.26	XRE family transcriptional regulator [Arthrobacter nicotinovorans]
gi515767853	3.97	3	1	252	28.0	6.44	4.25	hypothetical protein [Arthrobacter sp. M2012083]
gi742070686	1.41	3	1	1350	149.5	5.40	4.25	hypothetical protein ANMWB30_18850 [Arthrobacter sp. MWB30]
gi918221923	1.80	1	1	500	53.9	5.20	4.25	hypothetical protein [Arthrobacter sp. I3]
gi760112828	6.67	1	1	255	26.3	6.93	4.25	short-chain dehydrogenase [Arthrobacter chlorophenolicus]
gi359304500	5.53	1	1	470	49.8	5.08	4.25	aldehyde dehydrogenase [Arthrobacter globiformis NBRC 12137]
gi823667251	4.31	1	1	255	27.3	6.46	4.25	CoA-transferase [Arthrobacter sp. YC-RL1]
gi654828069	8.97	1	1	223	25.0	7.55	4.25	DNA alkylation repair protein [Arthrobacter sp. H5]
gi928487460	4.70	1	1	234	25.8	5.24	4.25	hypothetical protein AOC05_11670 [Arthrobacter alpinus]
gi515767834	9.56	1	1	136	14.9	5.29	4.25	GntR family transcriptional regulator [Arthrobacter sp. M2012083]
gi910741238	2.82	1	1	284	32.2	6.19	4.25	formyltetrahydrofolate deformylase [Arthrobacter sp. Hiyo4]
gi916820537	1.70	1	1	588	64.5	5.53	4.25	hypothetical protein [Arthrobacter sp. H20]
gi517590654	3.53	1	1	397	40.0	5.36	4.25	glycerate kinase [Arthrobacter sp. 135MFCol5.1]
gi648572640	19.23	1	1	78	9.0	4.88	4.24	hypothetical protein [Arthrobacter sp. 135MFCol5.1]
gi910743581	7.29	3	1	247	27.7	10.33	4.24	atp-Dependent DNA helicase [Arthrobacter sp. Hiyo8]
gi917760278	3.99	1	1	426	44.8	5.17	4.24	kynureninase [Arthrobacter sp. L77]
gi162954532	4.92	3	1	305	32.9	7.59	4.24	dipeptide transport ATP-binding protein [Renibacterium salmoninarum ATCC 33209]
gi674646862	2.19	1	1	366	38.1	4.92	4.24	Thermonuclease precursor [Arthrobacter sp. 11W110_air]
gi651500064	2.93	1	1	512	56.9	5.21	4.24	alpha-L-arabinofuranosidase [Arthrobacter sp. 35W]
gi116612852	19.54	1	1	87	9.8	10.13	4.24	hypothetical protein Arth_4403 (plasmid) [Arthrobacter sp. FB24]
gi917760260	5.24	1	1	191	21.2	6.00	4.24	hypothetical protein [Arthrobacter sp. L77]
gi119950830	2.48	1	1	686	72.5	5.30	4.24	ATP-dependent helicase,-like protein [Arthrobacter aurescens TC1]
gi742851258	1.50	1	1	602	64.9	4.46	4.24	ABC transporter substrate-binding protein [Arthrobacter sp. W1]
gi786026195	4.61	1	1	217	22.5	4.55	4.24	tRNA threonylcarbamoyladenosine biosynthesis protein TsaE [Arthrobacter chlorophenolicus]
gi927294264	6.27	2	1	271	29.9	6.80	4.24	transcriptional regulator [Arthrobacter sp. ERGS1:01]
gi518312854	2.85	1	1	666	74.1	6.33	4.23	hypothetical protein [Arthrobacter sp. TB 23]
gi443480801	4.80	2	1	354	38.0	6.20	4.23	tRNA methyltransferase complex GCD14 subunit [Arthrobacter nitrophenolicus]
gi917759763	12.57	1	1	175	18.4	7.03	4.23	hypothetical protein [Arthrobacter sp. L77]
gi908690925	6.58	1	1	365	38.1	6.16	4.23	hypothetical protein [Arthrobacter sp. H41]
gi654827934	7.05	1	1	156	17.0	7.14	4.23	AsnC family transcriptional regulator [Arthrobacter sp. H5]
gi654816772	8.08	1	1	260	28.1	6.46	4.23	glucosamine-6-phosphate deaminase [Arthrobacter sp. UNC362MFTsu5.1]
gi162954258	3.02	1	1	364	39.4	6.04	4.23	tRNA (adenine-N(1)-)-methyltransferase [Renibacterium salmoninarum ATCC 33209]
gi219859787	5.05	1	1	317	33.8	9.69	4.23	Formamidopyrimidine-DNA glycosylase catalytic domain protein [Arthrobacter chlorophenolicus A6
gi910697486	2.80	2	1	322	34.3	6.55	4.23	HTH-type transcriptional regulator HexR [Arthrobacter sp. Hiyo6]
gi939037113	4.23	1	1	189	21.0	4.78	4.23	dihydrofolate reductase [Arthrobacter nitroguajacolicus]
gi162955583	6.42	1	1	265	28.9	6.55	4.23	auxin-induced protein PCNT115 [Renibacterium salmoninarum ATCC 33209]
gi759705075	5.01	1	1	339	35.2	5.07	4.23	hypothetical protein [Arthrobacter globiformis]
gi753938675	9.01	1	1	233	26.6	9.31	4.22	membrane protein [Arthrobacter phenanthrenivorans]
gi518313502	4.90	3	1	469	49.2	4.73	4.22	aldehyde dehydrogenase [Arthrobacter sp. TB 23]
gi917739859	8.06	3	1	186	20.4	6.61	4.22	hypothetical protein [Arthrobacter sp. W1]
gi518313233	4.20	1	1	357	38.1	6.13	4.22	MULTISPECIES: DNA recombination/repair protein RecA [Arthrobacter]
gi737810320	2.93	2	1	443	46.2	5.02	4.22	glutamyl-tRNA reductase [Arthrobacter sp. 35/47]
gi518312060	6.77	1	1	251	26.1	5.60	4.22	SDR family oxidoreductase [Arthrobacter sp. TB 23]
gi914716142	7.73	1	1	207	21.3	10.73	4.22	amino acid transporter [Arthrobacter sp. ZBG10]
gi910747370	15.87	1	1	63	7.4	8.19	4.22	30S ribosomal protein S10 [Arthrobacter sp. Hiyo8]
gi910694771	4.55	1	1	418	44.9	8.53	4.21	probable acyl-CoA dehydrogenase FadE10 [Arthrobacter sp. Hiyo6]

gi410689653	2.93	1	1	444	47.7	5.33	4.21	hypothetical protein (plasmid) [Arthrobacter sp. J3-40]
gi307745493	3.94	4	1	406	44.8	5.81	4.21	probable alanine transaminase [Arthrobacter arilaitensis Re117]
gi652425701	8.57	1	1	140	15.9	5.08	4.21	hypothetical protein [Arthrobacter castelli]
gi742069956	4.49	1	1	178	17.9	5.01	4.21	flp pilus assembly protein [Arthrobacter sp. MWB30]
gi916834469	6.13	1	1	359	38.0	7.58	4.21	hypothetical protein [Arthrobacter sp. H14]
gi910251865	1.52	1	1	660	71.0	5.03	4.21	levanase [Arthrobacter siccitolerans]
gi470217836	3.08	1	1	292	29.9	6.87	4.21	Universal stress protein family protein [Arthrobacter gangotriensis Lz1y]
gi359305393	4.49	1	1	379	39.9	7.46	4.21	hypothetical protein ARGLB_069_00540 [Arthrobacter globiformis NBRC 12137]
gi119951743	23.40	1	1	94	10.2	8.54	4.21	conserved hypothetical protein (plasmid) [Arthrobacter aurescens TC1]
gi910250664	5.77	1	1	156	16.9	4.45	4.20	glyoxalase family protein [Arthrobacter siccitolerans]
gi476399296	2.88	1	1	278	30.7	5.19	4.20	DNA/RNA endonuclease [Arthrobacter crystallopoietes BAB-32]
gi654814819	5.37	3	1	391	42.7	5.83	4.20	hypothetical protein [Arthrobacter sp. MA-N2]
gi908699610	3.78	2	1	503	55.1	4.89	4.20	alpha-L-arabinofuranosidase [Arthrobacter sp. RIT-PI-e]
gi917739905	3.94	1	1	406	43.2	5.82	4.20	hypothetical protein [Arthrobacter sp. W1]
gi910741792	7.18	1	1	181	18.6	8.75	4.20	hypothetical protein AHiyo4_46140 [Arthrobacter sp. Hiyo4]
gi651503901	25.00	2	1	96	10.1	7.44	4.20	hypothetical protein [Arthrobacter sp. 35W]
gi918469432	2.14	1	1	560	61.9	5.36	4.20	ATP-dependent DNA helicase RecQ [Arthrobacter crystallopoietes]
gi651431450	1.55	1	1	841	87.0	10.20	4.20	hypothetical protein [Arthrobacter sanguinis]
gi914717485	4.53	1	1	419	44.5	4.92	4.20	kynureninase [Arthrobacter sp. ZBG10]
gi307743502	3.29	1	1	213	23.2	10.61	4.19	conserved hypothetical membrane protein [Arthrobacter arilaitensis Re117]
gi403228208	8.18	1	1	220	23.8	6.81	4.19	putative transcriptional regulator, TetR family [Arthrobacter sp. Rue61a]
gi757623004	4.42	1	1	407	43.2	5.20	4.19	amidohydrolase [Arthrobacter sp. SPG23]
gi518312071	2.99	1	1	401	43.9	7.47	4.19	hypothetical protein [Arthrobacter sp. TB 23]
gi914717124	4.44	1	1	518	57.5	5.06	4.19	methionine--tRNA ligase [Arthrobacter sp. ZBG10]
gi823667849	3.66	1	1	355	40.1	4.83	4.19	hypothetical protein AA310_04245 [Arthrobacter sp. YC-RL1]
gi937256471	8.38	2	1	191	20.7	9.60	4.19	hypothetical protein AO716_15505 [Arthrobacter sp. Edens01]
gi476403070	3.63	1	1	331	36.3	5.94	4.19	cyclic-nucleotide signal transduction protein, partial [Arthrobacter crystallopoietes BAB-32]
gi504875298	1.30	2	1	849	92.4	5.55	4.19	type III restriction enzyme [Arthrobacter sp. J3-37]
gi759730386	0.75	1	1	1609	180.0	5.22	4.19	glutamate dehydrogenase [Arthrobacter sp. L77]
gi654817067	10.24	4	1	205	23.7	6.55	4.19	nuclease PIN [Arthrobacter sp. UNC362MFTsu5.1]
gi162955632	4.35	1	1	322	32.6	4.81	4.18	electron transfer flavoprotein alpha-subunit [Renibacterium salmoninarum ATCC 33209]
gi910696485	12.87	1	1	171	18.5	7.02	4.18	hypothetical protein AHiyo6_11590 [Arthrobacter sp. Hiyo6]
gi517600215	1.54	1	1	907	96.2	7.56	4.18	magnesium-transporting ATPase [Arthrobacter sp. 162MFSha1.1]
gi910696908	3.59	1	1	195	20.5	9.01	4.18	hypothetical protein AHiyo6_08250, partial [Arthrobacter sp. Hiyo6]
gi823668400	4.53	1	1	265	27.1	6.92	4.18	aspartate dehydrogenase [Arthrobacter sp. YC-RL1]
gi927032513	1.78	1	1	954	103.8	5.96	4.18	ribonuclease E [Arthrobacter sp. LS16]
gi737786564	0.63	1	1	1600	171.3	4.73	4.18	hypothetical protein [Arthrobacter albus]
gi930826838	2.49	1	1	361	41.5	7.17	4.18	hypothetical protein AOZ07_11480 [Arthrobacter arilaitensis]
gi742859344	6.16	1	1	211	23.0	6.04	4.18	GntR family transcriptional regulator [Arthrobacter sp. W1]
gi116612311	1.69	1	1	888	93.0	6.10	4.17	formate dehydrogenase, alpha subunit [Arthrobacter sp. FB24]
gi674645832	4.99	2	1	361	37.4	5.77	4.17	hypothetical protein BN1051_02030 [Arthrobacter sp. 11W110_air]
gi937256432	4.29	1	1	350	36.5	5.81	4.17	oxidoreductase [Arthrobacter sp. Edens01]
gi818631445	10.49	1	1	143	15.8	5.22	4.17	hypothetical protein (plasmid) [Arthrobacter sp. 68b]
gi908699441	2.59	2	1	617	66.8	9.48	4.17	ABC transporter [Arthrobacter sp. RIT-PI-e]
gi742860771	2.53	1	1	396	42.8	8.72	4.17	alanine racemase [Arthrobacter sp. W1]
gi917759766	2.33	3	1	558	59.0	6.77	4.17	hypothetical protein [Arthrobacter sp. L77]
gi910739234	5.15	1	1	388	41.8	10.77	4.17	putative methyltransferase Rv1407/MT1451 [Arthrobacter sp. Hiyo4]
gi648574562	2.93	2	1	375	40.0	5.40	4.17	XshC-Cox1 family protein [Arthrobacter sp. 131MFCol6.1]
gi737801629	3.21	1	1	499	52.9	5.77	4.17	ABC transporter ATP-binding protein [Arthrobacter castelli]
gi939051484	4.11	3	1	438	49.3	7.09	4.16	ATPase [Arthrobacter sp. JCM 19049]
gi759725200	2.95	1	1	542	55.6	11.24	4.16	hypothetical protein [Arthrobacter sp. I3]
gi651506959	2.59	1	1	1004	106.9	6.98	4.16	hypothetical protein [Arthrobacter sp. 35W]
gi219859596	1.23	1	1	650	71.5	5.01	4.16	Endothelin-converting enzyme 1 [Arthrobacter chlorophenolicus A6]
gi928488459	11.36	1	1	88	9.4	9.95	4.16	Rho termination factor [Arthrobacter alpinus]
gi674644187	4.90	1	1	490	50.8	4.84	4.16	ATP-dependent zinc metalloprotease FtsH 3 [Arthrobacter sp. 11W110_air]
gi551256928	12.86	1	1	70	7.4	4.88	4.16	MT0933-like antitoxin protein [Arthrobacter sp. PAO19]
gi551256434	1.73	2	1	1271	142.8	6.00	4.16	hypothetical protein [Arthrobacter sp. PAO19]
gi651431940	4.18	1	1	550	57.1	6.81	4.16	hypothetical protein [Arthrobacter sanguinis]
gi823665795	3.15	1	1	317	33.8	10.10	4.16	hypothetical protein AA310_07670 [Arthrobacter sp. YC-RL1]

gi517592907	2.92	2	1	479	50.9	8.32	4.15	short-chain fatty acid transporter [Arthrobacter sp. 135MFCol5.1]
gi927294514	1.36	1	1	882	94.5	5.01	4.15	aminopeptidase [Arthrobacter sp. ERGS1:01]
gi654822586	6.61	1	1	257	26.7	5.30	4.15	glutamine amidotransferase [Arthrobacter sp. I3]
gi917760307	1.91	2	1	418	42.8	5.44	4.15	hypothetical protein [Arthrobacter sp. L77]
gi737796606	4.81	1	1	208	22.6	5.03	4.15	enoyl-CoA hydratase [Arthrobacter sp. H20]
gi742072172	8.70	2	1	92	10.5	7.43	4.15	hypothetical protein ANMWB30_08760 [Arthrobacter sp. MWB30]
gi307744759	12.78	1	1	180	20.1	4.34	4.15	16S rRNA-processing protein RimM [Arthrobacter arilaitensis Re117]
gi723606366	5.28	2	1	246	26.5	5.35	4.15	glutamine transport ATP-binding protein [Arthrobacter sp. PAMC25486]
gi759735256	9.34	1	1	182	18.6	4.83	4.14	haloacid dehalogenase [Arthrobacter sp. L77]
gi914716871	5.80	1	1	276	30.1	9.80	4.14	hypothetical protein [Arthrobacter sp. ZBG10]
gi162954217	2.93	2	1	341	36.7	5.78	4.14	holliday junction DNA helicase [Renibacterium salmoninarum ATCC 33209]
gi651452979	4.80	1	1	354	39.9	5.64	4.14	ATP-dependent DNA ligase [Arthrobacter nicotinovorans]
gi927292723	4.72	1	1	466	49.1	5.22	4.14	xylulose kinase (plasmid) [Arthrobacter sp. ERGS1:01]
gi470217162	1.94	1	1	412	43.2	5.55	4.14	beta-ketoadipyl CoA thiolase [Arthrobacter gangotriensis Lz1y]
gi476402751	3.55	1	1	338	37.3	5.26	4.14	hypothetical protein D477_001684 [Arthrobacter crystallopoietes BAB-32]
gi914714991	5.05	1	1	416	42.2	6.68	4.13	xanthine dehydrogenase [Arthrobacter sp. ZBG10]
gi476399597	3.90	1	1	461	49.5	6.09	4.13	hypothetical protein D477_017714 [Arthrobacter crystallopoietes BAB-32]
gi323467948	2.39	1	1	461	50.3	5.88	4.13	lysine/ornithine N-monooxygenase [Arthrobacter phenanthrenivorans Sphe3]
gi517589921	4.00	1	1	250	26.0	4.74	4.13	hypothetical protein [Arthrobacter sp. 135MFCol5.1]
gi639130031	2.87	3	1	663	71.1	5.12	4.13	propionyl-CoA synthetase [Arthrobacter sp. CAL618]
gi674644280	6.08	1	1	181	20.4	4.49	4.13	Inorganic pyrophosphatase [Arthrobacter sp. 11W110_air]
gi765010177	13.24	5	1	136	14.4	8.62	4.13	GntR family transcriptional regulator [Arthrobacter sp. A3]
gi219861777	4.06	1	1	419	45.5	8.78	4.13	type II secretion system protein (plasmid) [Arthrobacter chlorophenolicus A6]
gi518311185	3.47	1	1	461	48.2	5.78	4.12	MULTISPECIES: hypothetical protein [Arthrobacter]
gi654822708	6.30	1	1	270	28.3	9.42	4.12	NmrA family transcriptional regulator [Arthrobacter sp. I3]
gi917739922	8.02	1	1	162	16.8	9.57	4.12	hypothetical protein [Arthrobacter sp. W1]
gi651438659	3.91	1	1	460	49.7	6.09	4.12	hypothetical protein [Arthrobacter sp. H14]
gi470217616	1.36	3	1	884	95.7	5.22	4.12	Aminopeptidase N [Arthrobacter gangotriensis Lz1y]
gi515767124	4.48	1	1	268	28.2	4.65	4.12	hypothetical protein [Arthrobacter sp. M2012083]
gi918267220	4.63	10	1	216	23.7	9.92	4.12	DNA replication and repair protein RecF [Arthrobacter sp. Hiyo1]
gi651440493	9.47	1	1	190	20.8	7.91	4.12	hypothetical protein [Arthrobacter sp. H14]
gi740684281	2.38	1	1	421	42.7	10.39	4.12	transporter [Arthrobacter sp. PAMC25486]
gi765007449	3.85	2	1	208	22.8	4.97	4.12	DSBA oxidoreductase [Arthrobacter sp. A3]
gi33571522	1.51	1	1	793	84.1	5.49	4.12	probable dehydrogenase/oxidase [Bordetella pertussis Tohama I]
gi517591434	4.69	1	1	213	23.1	7.43	4.12	HxlR family transcriptional regulator [Arthrobacter sp. 135MFCol5.1]
gi910693722	4.55	1	1	242	26.8	5.17	4.12	transcriptional regulator NanR [Arthrobacter sp. Hiyo6]
gi786025592	6.10	3	1	328	33.6	6.81	4.11	two-component system sensor histidine kinase [Arthrobacter chlorophenolicus]
gi927296308	2.60	1	1	423	44.8	5.88	4.11	hypothetical protein AL755_11255 [Arthrobacter sp. ERGS1:01]
gi742759517	4.30	1	1	558	60.3	9.63	4.11	FAD-binding dehydrogenase [Arthrobacter phenanthrenivorans]
gi742855066	1.89	1	1	846	87.9	4.28	4.11	alkaline phosphatase [Arthrobacter sp. W1]
gi757624849	2.57	2	1	311	33.4	9.19	4.11	hypothetical protein TV39_09300 [Arthrobacter sp. SPG23]
gi654815502	4.17	2	1	456	49.9	6.37	4.11	hypothetical protein [Arthrobacter sp. PAO19]
gi927031391	1.99	2	1	854	93.9	5.25	4.11	hypothetical protein AFL94_02565 [Arthrobacter sp. LS16]
gi928486260	7.11	1	1	225	26.2	6.54	4.11	phosphoglycerate mutase [Arthrobacter alpinus]
gi937262471	1.53	1	1	1048	108.2	4.54	4.11	hydrogenase expression protein [Arthrobacter sp. Edens01]
gi916876269	4.56	2	1	417	43.4	5.59	4.11	alcohol dehydrogenase [Arthrobacter sp. 31Y]
gi916863522	0.57	1	1	1914	198.0	4.31	4.11	hypothetical protein, partial [Arthrobacter sp. 35/47]
gi786027512	6.06	1	1	264	27.7	5.47	4.10	nitrilase [Arthrobacter chlorophenolicus]
gi476402416	1.27	1	1	786	82.8	7.55	4.10	ComEC/Rec2-like protein [Arthrobacter crystallopoietes BAB-32]
gi518313183	6.63	1	1	181	18.9	6.93	4.10	hypothetical protein [Arthrobacter sp. TB 23]
gi927293066	4.71	1	1	403	43.3	5.97	4.10	hypothetical protein AL755_02290 (plasmid) [Arthrobacter sp. ERGS1:01]
gi652423455	9.45	1	1	201	21.5	6.20	4.10	hypothetical protein [Arthrobacter castelli]
gi737781372	3.43	1	1	408	43.6	5.14	4.10	carbamoyl-phosphate synthase small subunit [Arthrobacter sp. 35W]
gi918469434	3.97	1	1	353	39.8	5.25	4.10	Rieske (2Fe-2S) protein [Arthrobacter crystallopoietes]
gi823665505	10.17	2	1	236	25.0	5.01	4.10	hypothetical protein AA310_05785 [Arthrobacter sp. YC-RL1]
gi119951123	12.50	3	1	184	19.8	9.48	4.10	putative protein of unknown function (DUF721) [Arthrobacter aurescens TC1]
gi651499487	2.24	1	1	492	54.2	7.01	4.10	hypothetical protein [Arthrobacter sp. 35W]
gi651498711	7.18	1	1	209	22.5	8.15	4.10	16S rRNA methyltransferase [Arthrobacter sp. 35W]
gi742069802	4.51	1	1	244	27.3	8.31	4.09	hypothetical protein ANMWB30_32560 [Arthrobacter sp. MWB30]

gi545107978	2.05	1	1	730	80.7	5.59	4.09	amylo-alpha-1,6-glucosidase [Arthrobacter sp. AK-YN10]
gi654811873	6.54	1	1	214	23.3	7.34	4.08	hypothetical protein [Arthrobacter sp. MA-N2]
gi654811382	2.51	1	1	557	58.2	5.31	4.08	L-aspartate oxidase [Arthrobacter sp. MA-N2]
gi162953462	6.09	1	1	197	22.0	9.86	4.08	metal-dependent hydrolase [Renibacterium salmoninarum ATCC 33209]
gi651434814	5.85	1	1	188	21.3	4.53	4.08	inorganic pyrophosphatase [Arthrobacter sp. H41]
gi674646284	5.49	1	1	328	34.2	6.70	4.08	Daunorubicin/doxorubicin resistance ATP-binding protein DrrA [Arthrobacter sp. 11W110_air]
gi742758401	10.24	2	1	205	23.4	6.55	4.08	nuclease PIN [Arthrobacter phenanthrenivorans]
gi651444715	1.70	1	1	529	56.2	5.08	4.08	peptide ABC transporter [Arthrobacter nicotinovorans]
gi927293047	1.67	2	1	538	56.7	5.00	4.08	amidohydrolase (plasmid) [Arthrobacter sp. ERGS1:01]
gi916691618	5.25	2	1	362	38.6	5.26	4.08	N-succinyldiaminopimelate aminotransferase [Arthrobacter castelli]
gi515767347	2.18	1	1	413	42.7	10.23	4.08	MFS transporter [Arthrobacter sp. M2012083]
gi927295942	6.25	1	1	272	28.5	9.91	4.08	hydrolase [Arthrobacter sp. ERGS1:01]
gi674644368	6.09	1	1	312	31.2	9.95	4.08	hypothetical protein BN1051_00520 [Arthrobacter sp. 11W110_air]
gi551254443	4.16	1	1	409	43.4	5.19	4.07	monooxygenase [Arthrobacter sp. PAO19]
gi654828125	8.43	1	1	83	9.1	4.63	4.07	exodeoxyribonuclease VII small subunit [Arthrobacter sp. H5]
gi928988139	1.34	1	1	1195	124.8	5.15	4.07	hypothetical protein [Arthrobacter sp. ERGS1:01]
gi737792885	4.47	2	1	313	32.3	9.48	4.07	lipase [Arthrobacter nicotinovorans]
gi219860463	3.86	4	1	389	38.7	10.64	4.07	major facilitator superfamily MFS_1 [Arthrobacter chlorophenolicus A6]
gi746183738	2.20	1	1	364	40.9	7.68	4.07	glycosyltransferase [Arthrobacter sp. MWB30]
gi767259321	3.32	1	1	271	30.5	9.57	4.07	methylase [Arthrobacter sp. IHBB 11108]
gi654818499	1.83	2	1	436	46.0	9.48	4.07	MFS transporter [Arthrobacter sp. UNC362MFTsu5.1]
gi910737922	6.59	2	1	349	38.2	5.68	4.07	phosphoenolpyruvate carboxykinase [GTP] [Arthrobacter sp. Hiyo4]
gi742071211	4.91	1	1	224	24.3	9.82	4.06	hypothetical protein ANMWB30_24100 [Arthrobacter sp. MWB30]
gi916870226	2.08	1	1	336	36.3	5.29	4.06	hypothetical protein [Arthrobacter sp. Br18]
gi654826460	15.48	1	1	84	9.1	4.92	4.06	hypothetical protein [Arthrobacter sp. H5]
gi765004451	4.36	1	1	275	28.7	5.35	4.06	elongation factor Ts [Arthrobacter sp. A3]
gi307744778	7.33	1	1	191	21.1	5.20	4.06	RibD domain-containing protein [Arthrobacter arilaitensis Re117]
gi651449093	2.05	3	1	877	95.8	5.55	4.06	glycogen phosphorylase [Arthrobacter nicotinovorans]
gi542110374	1.80	1	1	499	53.8	10.05	4.06	oxidoreductase [Arthrobacter sp. AK-YN10]
gi910696151	4.97	2	1	302	32.4	5.53	4.05	hypothetical protein AHiyo6_14040, partial [Arthrobacter sp. Hiyo6]
gi307745032	2.09	1	1	574	62.5	5.88	4.05	pyruvate dehydrogenase (cytochrome) [Arthrobacter arilaitensis Re117]
gi916863221	5.42	1	1	332	34.5	9.79	4.05	cytochrome C biogenesis protein [Arthrobacter sp. 35/47]
gi443482462	1.97	1	1	507	54.2	5.73	4.05	ATP-dependent DNA ligase [Arthrobacter nitrophenolicus]
gi908690940	1.25	1	1	722	75.4	5.50	4.05	ATPase [Arthrobacter sp. H41]
gi517590351	2.62	1	1	534	57.3	9.89	4.05	hypothetical protein [Arthrobacter sp. 135MFCol5.1]
gi651456751	7.05	3	1	241	27.1	10.04	4.05	hypothetical protein [Arthrobacter sp. 35/47]
gi674645845	2.02	1	1	939	100.6	5.45	4.05	hypothetical protein BN1051_02043 [Arthrobacter sp. 11W110_air]
gi916324794	7.02	1	1	228	24.9	9.50	4.05	hypothetical protein [Arthrobacter gangotriensis]
gi749401795	8.93	1	1	112	11.9	9.41	4.05	malate:quinone oxidoreductase, partial [Arthrobacter sp. AK-YN10]
gi916863518	2.58	2	1	426	45.5	11.28	4.05	MFS transporter [Arthrobacter sp. 35/47]
gi654825779	7.17	1	1	223	23.1	5.38	4.05	hypothetical protein [Arthrobacter sp. H5]
gi916357055	5.59	1	1	304	32.4	8.22	4.04	hypothetical protein [Arthrobacter sp. 162MFSha1.1]
gi162954655	3.91	1	1	256	27.9	11.25	4.04	hypothetical transporter (sulfate transport family) [Renibacterium salmoninarum ATCC 33209]
gi470221366	4.78	1	1	418	45.9	7.52	4.04	NADH:flavin oxidoreductase [Arthrobacter gangotriensis Lz1y]
gi651460880	1.94	1	1	980	104.2	5.31	4.04	ferredoxin [Arthrobacter sp. 35/47]
gi917991357	4.85	1	1	268	29.0	7.05	4.04	D-beta-D-heptose 1-phosphate adenosyltransferase [Arthrobacter aurescens]
gi219858188	2.43	1	1	781	85.6	5.19	4.04	Kojibiose phosphorylase [Arthrobacter chlorophenolicus A6]
gi654813178	2.66	1	1	451	49.3	6.28	4.04	monooxygenase [Arthrobacter sp. MA-N2]
gi517599086	3.43	1	1	321	34.8	7.21	4.03	transcriptional regulator [Arthrobacter sp. 162MFSha1.1]
gi740677404	3.25	1	1	277	30.0	5.88	4.03	oxidoreductase [Arthrobacter sp. PAMC25486]
gi914716585	2.43	1	1	371	39.0	6.86	4.03	phospho-2-dehydro-3-deoxyheptonate aldolase [Arthrobacter sp. ZBG10]
gi648572505	6.57	2	1	213	22.6	7.42	4.03	hypothetical protein [Arthrobacter sp. 135MFCol5.1]
gi517590722	1.70	2	1	764	83.5	5.11	4.03	catalase/hydroperoxidase HPI(I) [Arthrobacter sp. 135MFCol5.1]
gi517608204	2.39	1	1	419	42.8	10.05	4.03	MFS transporter [Arthrobacter sp. 161MFSha2.1]
gi651505773	2.94	1	1	477	52.7	8.87	4.03	hypothetical protein [Arthrobacter sp. 35W]
gi651507052	3.17	2	1	537	56.9	5.34	4.03	MFS transporter [Arthrobacter sp. 35W]
gi517606552	1.80	1	1	444	47.5	5.02	4.03	ABC transporter substrate-binding protein [Arthrobacter sp. 161MFSha2.1]
gi917529539	2.27	2	1	264	28.3	6.54	4.02	hypothetical protein [Arthrobacter sp. PAMC25486]
gi674645813	4.65	2	1	430	43.3	5.78	4.02	antiporter inner membrane protein [Arthrobacter sp. 11W110_air]

gi652425237	6.13	3	1	212	23.9	8.13	4.02	multidrug ABC transporter ATP-binding protein [Arthrobacter castelli]
gi908740181	5.94	1	1	320	34.9	5.35	4.02	glutamyl-Q tRNA(Asp) ligase [Arthrobacter arilaitensis]
gi476402559	8.30	1	1	253	27.0	5.14	4.02	GntR family transcriptional regulator [Arthrobacter crystallopoietes BAB-32]
gi651484468	1.94	1	1	823	86.0	5.87	4.02	hypothetical protein [Arthrobacter sp. Br18]
gi737800392	4.74	1	1	274	29.2	5.97	4.02	glycosyl transferase family 8 [Arthrobacter castelli]
gi928487379	6.71	1	1	283	30.2	5.20	4.02	2,5-diketo-D-gluconic acid reductase [Arthrobacter alpinus]
gi757625571	3.01	2	1	432	46.5	6.81	4.02	histidine kinase [Arthrobacter sp. SPG23]
gi910283820	6.44	1	1	233	23.9	4.70	4.02	hypothetical protein [Arthrobacter sp. A3]
gi908698331	7.47	2	1	174	20.0	5.35	4.02	peptide-methionine (S)-S-oxide reductase [Arthrobacter sp. RIT-PI-e]
gi759746410	2.69	1	1	409	44.3	5.22	4.01	N-isopropylammelide isopropylaminohydrolase [Arthrobacter sp. 31Y]
gi651430502	2.08	2	1	289	32.3	9.20	4.01	sugar ABC transporter permease [Arthrobacter sanguinis]
gi476402790	5.17	1	1	271	29.0	5.25	4.01	triosephosphate isomerase [Arthrobacter crystallopoietes BAB-32]
gi654817320	2.28	1	1	351	38.1	5.34	4.01	excisionase [Arthrobacter sp. UNC362MFTsu5.1]
gi910743950	13.17	1	1	167	17.8	9.99	4.01	hypothetical protein AHiyo8_16520 [Arthrobacter sp. Hiyo8]
gi765009559	7.48	1	1	147	15.9	11.63	4.01	hypothetical protein [Arthrobacter sp. A3]
gi910248794	12.38	1	1	105	11.7	5.29	4.01	hypothetical protein [Arthrobacter siccitolerans]
gi767258818	2.55	1	1	549	59.0	6.43	4.01	peptide ABC transporter ATPase [Arthrobacter sp. IHBB 11108]
gi927296030	7.06	1	1	354	37.2	5.58	4.01	phosphonate ABC transporter substrate-binding protein [Arthrobacter sp. ERGS1:01]
gi916816466	4.37	1	1	366	40.2	5.59	4.01	hypothetical protein [Arthrobacter sp. MA-N2]
gi517592653	1.39	1	1	648	69.4	10.43	4.00	hypothetical protein [Arthrobacter sp. 135MFCol5.1]
gi908740343	5.20	2	1	269	32.3	11.53	4.00	hypothetical protein [Arthrobacter arilaitensis]
gi410689859	6.20	1	1	258	28.2	9.07	4.00	hypothetical protein (plasmid) [Arthrobacter sp. J3-53]
gi359303616	2.86	1	1	315	33.0	9.79	4.00	TDT family transporter [Arthrobacter globiformis NBRC 12137]
gi654822223	6.28	1	1	223	24.9	5.39	4.00	hypothetical protein [Arthrobacter sp. I3]
gi654819505	3.09	2	1	388	39.9	5.30	4.00	membrane protein [Arthrobacter sp. UNC362MFTsu5.1]
gi651481517	2.11	1	1	379	40.4	5.62	4.00	aminotransferase [Arthrobacter sp. Br18]
gi740684099	3.27	1	1	489	49.8	6.38	4.00	protoporphyrinogen oxidase [Arthrobacter sp. PAMC25486]
gi927294391	17.05	1	1	88	10.1	9.98	3.99	hypothetical protein AL755_10790 [Arthrobacter sp. ERGS1:01]
gi759704434	3.69	1	1	325	35.0	5.08	3.99	hypothetical protein [Arthrobacter globiformis]
gi651473408	2.03	1	1	836	89.2	5.83	3.99	hypothetical protein [Arthrobacter nicotinovorans]
gi915933352	2.83	1	1	353	36.4	6.84	3.99	alanine racemase [Arthrobacter globiformis]
gi723607256	4.15	1	1	579	58.4	9.60	3.99	hypothetical protein ART_1033 [Arthrobacter sp. PAMC25486]
gi470221115	2.58	1	1	737	79.7	6.13	3.99	ATP-dependent DNA helicase RecG [Arthrobacter gangotriensis Lz1y]
gi307744092	3.10	1	1	451	49.8	9.70	3.99	transposase of ISAar28, IS481 family [Arthrobacter arilaitensis Re117]
gi654817743	5.34	1	1	337	35.3	9.55	3.99	alpha-ketoglutarate decarboxylase [Arthrobacter sp. UNC362MFTsu5.1]
gi443479857	4.83	1	1	269	27.9	4.91	3.99	inositol monophosphatase/fructose-1,6-bisphosphatase family protein [Arthrobacter nitrophenolicu
gi759715921	2.27	1	1	572	62.9	5.47	3.99	ATPase AAA [Arthrobacter sp. AK-YN10]
gi737776731	6.34	1	1	268	29.2	11.14	3.98	hypothetical protein [Arthrobacter sanguinis]
gi651434799	4.80	2	1	458	46.7	5.06	3.98	hypothetical protein [Arthrobacter sp. H41]
gi219859273	1.51	1	1	463	49.8	4.94	3.98	conserved hypothetical protein [Arthrobacter chlorophenolicus A6]
gi930827632	5.91	2	1	254	27.0	5.02	3.98	transcriptional regulator [Arthrobacter arilaitensis]
gi910693235	4.93	5	1	304	32.6	6.10	3.98	HTH-type transcriptional regulator GltC [Arthrobacter sp. Hiyo6]
gi753931683	3.14	2	1	477	50.7	4.73	3.98	phosphomannomutase [Arthrobacter arilaitensis]
gi476400980	1.54	1	1	518	55.4	5.12	3.98	ABC transporter-like protein [Arthrobacter crystallopoietes BAB-32]
gi759747217	3.23	1	1	402	44.0	6.35	3.98	restriction endonuclease [Arthrobacter sp. 31Y]
gi767258678	6.08	1	1	378	41.1	7.55	3.98	hypothetical protein UM93_15655 [Arthrobacter sp. IHBB 11108]
gi759729695	2.00	2	1	850	88.9	4.75	3.97	RND transporter [Arthrobacter sp. L77]
gi651500676	6.53	1	1	199	21.9	6.60	3.97	XRE family transcriptional regulator [Arthrobacter sp. 35W]
gi759732518	13.04	1	1	69	8.1	8.05	3.97	hypothetical protein [Arthrobacter sp. L77]
gi759719018	3.21	1	1	405	42.7	5.26	3.97	aspartate aminotransferase [Arthrobacter sp. FB24]
gi759734409	2.85	1	1	562	61.2	7.03	3.97	hypothetical protein [Arthrobacter sp. L77]
gi928487718	7.43	2	1	148	15.9	9.80	3.96	ArsR family transcriptional regulator [Arthrobacter alpinus]
gi470216998	4.24	1	1	401	42.3	5.76	3.96	hypothetical protein ADIAG_02759 [Arthrobacter gangotriensis Lz1y]
gi517604442	12.12	2	1	165	17.3	5.11	3.96	proteinase inhibitor I25 cystatin [Arthrobacter sp. 131MFCol6.1]
gi674644701	3.01	1	1	266	27.5	5.71	3.96	Purine nucleoside phosphorylase [Arthrobacter sp. 11W110_air]
gi651438926	4.90	2	1	306	35.0	6.73	3.96	pseudouridine synthase [Arthrobacter sp. H14]
gi937259633	1.36	1	1	956	106.6	6.33	3.96	hypothetical protein AO716_06575 [Arthrobacter sp. Edens01]
gi723608587	4.28	1	1	397	42.9	8.98	3.96	hypothetical protein ART_2364 [Arthrobacter sp. PAMC25486]
gi654813657	2.63	2	1	532	57.1	9.85	3.96	hypothetical protein [Arthrobacter sp. MA-N2]

gi910748235	3.33	1	1	540	55.3	6.25	3.96	conserved hypothetical protein [Arthrobacter sp. Hiyo8]
gi517590922	3.60	1	1	417	45.0	7.34	3.96	hypothetical protein [Arthrobacter sp. 135MFCol5.1]
gi759731599	3.84	1	1	495	51.3	5.26	3.95	carbohydrate kinase [Arthrobacter sp. L77]
gi737788881	1.60	1	1	563	64.7	5.24	3.95	hypothetical protein [Arthrobacter albus]
gi545108942	12.24	1	1	147	15.2	10.14	3.95	membrane protein [Arthrobacter sp. AK-YN10]
gi918265919	3.32	2	1	542	59.9	5.11	3.95	putative type I restriction enzyme HindVIIP M protein [Arthrobacter sp. Hiyo1]
gi737786762	2.16	2	1	647	72.1	6.14	3.95	hypothetical protein [Arthrobacter albus]
gi908697519	7.91	1	1	278	29.9	11.24	3.95	abortive infection protein [Arthrobacter sp. RIT-PI-e]
gi652423837	3.35	2	1	179	19.2	4.49	3.95	hypothetical protein [Arthrobacter castelli]
gi116612636	7.05	1	1	298	32.2	5.55	3.95	Glutamate--tRNA ligase [Arthrobacter sp. FB24]
gi742759525	6.60	3	1	303	31.7	8.09	3.95	diacylglycerol kinase [Arthrobacter phenanthrenivorans]
gi551255966	0.82	1	1	858	93.8	6.18	3.95	hypothetical protein [Arthrobacter sp. PAO19]
gi598062302	3.51	1	1	456	48.9	5.57	3.95	PuhA [Arthrobacter sp. BS2(2014)]
gi914717278	3.83	1	1	549	58.3	5.01	3.95	SAM-dependent methyltransferase [Arthrobacter sp. ZBG10]
gi927293773	4.09	1	1	513	53.3	6.46	3.95	carboxylesterase [Arthrobacter sp. ERGS1:01]
gi636847462	6.51	1	1	292	29.6	5.35	3.95	orotidine 5'-phosphate decarboxylase [Arthrobacter sp. TB 26]
gi759724551	4.67	1	1	214	22.7	6.09	3.94	alpha/beta hydrolase, partial [Arthrobacter sp. I3]
gi760166348	6.47	1	1	309	32.6	4.96	3.94	5'-3' exonuclease [Arthrobacter crystallopoietes]
gi651442801	9.23	1	1	130	13.7	5.16	3.94	glyoxalase [Arthrobacter sp. 9MFCol3.1]
gi162955556	1.99	1	1	554	60.4	9.03	3.94	Na(+)/H(+) antiporter [Renibacterium salmoninarum ATCC 33209]
gi515765127	7.64	1	1	144	16.1	5.39	3.94	CBS domain-containing protein [Arthrobacter sp. M2012083]
gi910250673	5.73	1	1	157	16.6	4.59	3.94	hypothetical protein [Arthrobacter siccitolerans]
gi823665671	2.81	2	1	640	70.8	7.15	3.94	family 2 glycosyl transferase [Arthrobacter sp. YC-RL1]
gi908697329	3.24	1	1	278	30.6	4.72	3.94	oxidoreductase [Arthrobacter sp. RIT-PI-e]
gi116609072	5.01	3	1	339	36.5	5.12	3.94	PHP C-terminal domain protein [Arthrobacter sp. FB24]
gi767258127	6.61	1	1	363	39.1	5.55	3.94	hypothetical protein UM93_11705 [Arthrobacter sp. IHBB 11108]
gi915933965	4.64	1	1	302	33.1	8.46	3.94	NUDIX domain-containing protein [Arthrobacter globiformis]
gi916871465	3.17	1	1	726	77.6	5.02	3.93	transketolase [Arthrobacter sp. H5]
gi919108177	3.06	1	1	653	70.6	5.27	3.93	hypothetical protein [Arthrobacter sp. IHBB 11108]
gi518313965	5.56	1	1	342	36.9	5.74	3.93	MULTISPECIES: hypothetical protein [Arthrobacter]
gi928988990	2.97	2	1	269	30.1	9.55	3.93	sugar ABC transporter permease [Arthrobacter sp. ERGS1:01]
gi651436078	4.26	1	1	329	35.4	5.92	3.93	O-succinylbenzoate synthase [Arthrobacter sp. H41]
gi916876224	3.91	1	1	307	33.1	9.99	3.93	ABC transporter permease [Arthrobacter sp. 31Y]
gi914717777	6.03	1	1	365	38.5	6.14	3.93	oxidoreductase [Arthrobacter sp. ZBG10]
gi651439719	1.88	1	1	426	47.1	6.62	3.93	hypothetical protein [Arthrobacter sp. H14]
gi928985869	1.76	1	1	624	66.0	7.40	3.93	hypothetical protein [Arthrobacter sp. ERGS1:01]
gi759734512	5.25	2	1	362	39.4	5.31	3.93	lipoate--protein ligase [Arthrobacter sp. L77]
gi476399169	16.43	1	1	140	14.7	9.28	3.92	hypothetical protein D477_019838 [Arthrobacter crystallopoietes BAB-32]
gi654818769	7.78	1	1	257	26.6	5.08	3.92	short-chain dehydrogenase [Arthrobacter sp. UNC362MFTsu5.1]
gi542110513	6.83	2	1	161	17.2	5.53	3.92	prolyl-tRNA synthetase [Arthrobacter sp. AK-YN10]
gi403229668	3.48	1	1	316	34.2	4.88	3.92	5'-3' exonuclease [Arthrobacter sp. Rue61a]
gi651491155	2.00	1	1	902	93.4	6.13	3.92	hydrolase [Arthrobacter sp. H20]
gi759728158	2.02	5	1	593	62.9	5.58	3.92	ABC transporter [Arthrobacter sp. UNC362MFTsu5.1]
gi737789356	6.00	1	1	300	31.6	5.21	3.91	4-hydroxy-tetrahydrodipicolinate synthase [Arthrobacter albus]
gi759759272	9.83	1	1	234	24.4	4.81	3.91	2-deoxyglucose-6-phosphatase [Arthrobacter sp. Rue61a]
gi914716426	1.27	1	1	948	100.1	5.90	3.91	glycine dehydrogenase [Arthrobacter sp. ZBG10]
gi767257645	4.92	2	1	386	42.5	5.63	3.91	epoxide hydrolase [Arthrobacter sp. IHBB 11108]
gi639130245	5.46	1	1	293	32.0	4.92	3.91	oxidoreductase [Arthrobacter sp. CAL618]
gi470221278	2.77	1	1	470	48.7	7.71	3.91	hypothetical protein ADIAG_00131 [Arthrobacter gangotriensis Lz1y]
gi518311425	3.10	1	1	387	41.2	5.26	3.91	hypothetical protein [Arthrobacter sp. TB 23]
gi927294015	6.93	1	1	332	35.9	5.96	3.90	16S rRNA methyltransferase [Arthrobacter sp. ERGS1:01]
gi640196200	4.03	1	1	347	37.4	8.21	3.90	hypothetical protein [Arthrobacter sp. 31Y]
gi515764459	2.21	1	1	588	63.4	5.29	3.90	flavin oxidoreductase [Arthrobacter sp. M2012083]
gi1518166	5.15	1	1	272	28.8	10.33	3.90	hypothetic regulatory protein [Pimelobacter simplex]
gi916782047	3.31	1	1	302	32.7	5.48	3.90	ABC transporter ATP-binding protein [Arthrobacter sp. 35W]
gi518312446	3.04	1	1	427	44.5	5.31	3.90	MULTISPECIES: hypothetical protein [Arthrobacter]
gi651429342	1.39	1	1	717	80.0	4.68	3.90	protease 2 [Arthrobacter sanguinis]
gi517592920	2.36	2	1	339	36.0	5.12	3.90	fructose-bisphosphate aldolase [Arthrobacter sp. 135MFCol5.1]
gi359307876	4.29	1	1	420	45.9	5.62	3.90	hypothetical protein ARGLB_008_01740 [Arthrobacter globiformis NBRC 12137]

gi742853338	1.35	1	1	1187	131.4	5.55	3.90	DNA polymerase III subunit alpha [Arthrobacter sp. W1]
gi648224648	18.27	1	1	104	11.9	5.10	3.90	hypothetical protein [Arthrobacter sp. M2012083]
gi928486348	3.61	1	1	166	17.9	8.48	3.90	MarR family transcriptional regulator [Arthrobacter alpinus]
gi918267025	3.88	1	1	438	45.6	6.13	3.89	hypothetical protein AHiyo1_26150 [Arthrobacter sp. Hiyo1]
gi723608569	21.62	1	1	37	4.0	10.83	3.89	hypothetical protein ART_2346 [Arthrobacter sp. PAMC25486]
gi937259184	6.01	1	1	233	24.8	5.03	3.89	cytidylate kinase [Arthrobacter sp. Edens01]
gi511534817	4.14	1	1	314	33.8	5.21	3.89	putative soj family protein (plasmid) [Arthrobacter nicotinovorans]
gi651497898	2.49	1	1	321	32.2	5.24	3.89	4-diphosphocytidyl-2C-methyl-D-erythritol kinase [Arthrobacter sp. 35W]
gi602406	1.18	1	1	1015	110.8	5.07	3.89	beta-galactosidase [Arthrobacter sp.]
gi910693523	7.87	1	1	267	28.0	4.67	3.88	PTS-dependent dihydroxyacetone kinase, dihydroxyacetone-binding subunit DhaK [Arthrobacter sp
gi918449458	1.27	2	1	1492	157.3	5.87	3.88	hypothetical protein, partial [Arthrobacter sp. SPG23]
gi765005358	6.38	1	1	282	29.8	4.70	3.88	fumarylacetoacetate hydrolase [Arthrobacter sp. A3]
gi786031286	4.08	1	1	196	21.5	8.44	3.88	transcriptional regulator [Arthrobacter chlorophenolicus]
gi654825652	2.98	1	1	470	48.5	6.55	3.88	hypothetical protein [Arthrobacter sp. H5]
gi674645838	5.92	1	1	321	34.2	10.51	3.88	Chaperone protein DnaJ [Arthrobacter sp. 11W110_air]
gi927296019	4.44	1	1	315	35.0	9.73	3.88	hypothetical protein AL755_21595 [Arthrobacter sp. ERGS1:01]
gi759714351	4.56	5	1	526	56.1	6.10	3.87	ABC transporter, partial [Arthrobacter sp. AK-YN10]
gi914714327	7.82	1	1	307	33.3	10.08	3.87	sugar ABC transporter permease [Arthrobacter sp. ZBG10]
gi651461544	3.17	1	1	599	62.9	5.22	3.87	acetyl-CoA carboxylase [Arthrobacter sp. 35/47]
gi767258053	3.28	1	1	488	52.2	9.48	3.87	potassium transporter Trk [Arthrobacter sp. IHBB 11108]
gi651494906	15.15	1	1	66	7.3	7.14	3.87	hypothetical protein [Arthrobacter sp. H20]
gi542109307	4.56	6	1	417	43.8	5.15	3.87	pyridine nucleotide-disulfide oxidoreductase [Arthrobacter sp. AK-YN10]
gi927293150	3.40	1	1	412	43.7	5.55	3.87	hypothetical protein AL755_02905 (plasmid) [Arthrobacter sp. ERGS1:01]
gi517598803	3.81	1	1	420	43.4	6.39	3.87	uroporphyrinogen III methylase [Arthrobacter sp. 162MFSha1.1]
gi651430377	3.00	1	1	333	35.9	5.50	3.86	daunorubicin resistance protein DrrA family ABC transporter ATP-binding protein [Arthrobacter sar
gi786027951	4.38	1	1	320	34.0	9.64	3.86	translation initiation factor IF-3 [Arthrobacter chlorophenolicus]
gi651494462	5.96	2	1	369	39.5	5.16	3.86	aspartate aminotransferase [Arthrobacter sp. H20]
gi443479980	4.95	1	1	384	39.3	4.87	3.85	branched chain amino acid ABC transporter permease [Arthrobacter nitrophenolicus]
gi515764895	2.37	1	1	379	37.9	9.82	3.85	ATPase [Arthrobacter sp. M2012083]
gi654815234	13.45	1	1	119	12.8	6.80	3.85	cupin [Arthrobacter sp. PAO19]
gi323467568	4.77	2	1	461	50.2	5.35	3.85	monoamine oxidase [Arthrobacter phenanthrenivorans Sphe3]
gi652423184	5.14	1	1	214	23.5	5.87	3.85	hypothetical protein [Arthrobacter castelli]
gi918469412	2.33	1	1	472	50.7	5.15	3.84	succinate-semialdehyde dehydrogenase [Arthrobacter crystallopoietes]
gi737782940	1.49	1	1	606	63.8	8.50	3.83	multidrug ABC transporter ATPase [Arthrobacter sp. 35W]
gi937259507	12.18	1	1	156	16.7	8.95	3.83	hypothetical protein AO716_05805 [Arthrobacter sp. Edens01]
gi651430906	2.53	1	1	434	45.3	6.25	3.83	acetyl-CoA acetyltransferase [Arthrobacter sanguinis]
gi403229053	3.33	2	1	270	29.1	7.02	3.82	uncharacterized protein YqxC [Arthrobacter sp. Rue61a]
gi927033712	15.94	1	1	69	8.0	10.14	3.82	hypothetical protein AFL94_16975 [Arthrobacter sp. LS16]
gi518313449	8.10	1	1	247	27.2	5.49	3.81	hypothetical protein [Arthrobacter sp. TB 23]
gi470215997	1.32	1	1	531	57.5	7.55	3.81	conjugal transfer ATPase TrbE [Arthrobacter gangotriensis Lz1y]
gi927294265	2.51	1	1	797	86.0	4.82	3.80	phosphoenolpyruvate synthase [Arthrobacter sp. ERGS1:01]
gi651442634	1.05	1	1	1045	116.0	5.69	3.80	hypothetical protein [Arthrobacter sp. 9MFCol3.1]
gi916692399	4.56	1	1	307	31.2	10.24	3.80	hypothetical protein [Arthrobacter castelli]
gi674645804	2.21	1	1	272	30.3	9.20	3.79	Teichoic acid translocation permease protein TagG [Arthrobacter sp. 11W110_air]
gi917013028	1.40	1	1	1353	144.3	4.94	3.79	hypothetical protein [Arthrobacter sanguinis]
gi551255530	3.35	1	1	239	25.9	4.88	3.79	hypothetical protein [Arthrobacter sp. PAO19]
gi518312359	4.01	1	1	424	45.6	5.41	3.79	hypothetical protein [Arthrobacter sp. TB 23]
gi515767312	7.28	1	1	151	16.7	8.97	3.79	hypothetical protein [Arthrobacter sp. M2012083]
gi737799249	0.70	1	1	1151	123.6	5.60	3.79	1-pyrroline-5-carboxylate dehydrogenase [Arthrobacter sp. TB 23]
gi927031459	5.50	3	1	309	34.2	8.29	3.78	hypothetical protein AFL94_02995 [Arthrobacter sp. LS16]
gi651431840	3.26	1	1	276	29.7	4.86	3.78	thiosulfate sulfurtransferase [Arthrobacter sanguinis]
gi323468617	2.08	1	1	288	30.1	5.26	3.78	Cof subfamily of IIB subfamily of haloacid dehalogenase superfamily/HAD-superfamily hydrolase, s
gi928488118	5.87	2	1	392	39.7	5.24	3.78	flagellar hook protein [Arthrobacter alpinus]
gi930828232	1.40	1	1	860	92.5	5.05	3.78	glycosyl hydrolase family 32 [Arthrobacter arilaitensis]
gi759716287	5.04	1	1	238	26.5	11.55	3.77	hypothetical protein, partial [Arthrobacter sp. AK-YN10]
gi639129199	1.66	1	1	1142	124.2	5.41	3.77	DNA helicase UvrD [Arthrobacter sp. CAL618]
gi927033393	3.73	1	1	375	40.3	5.01	3.77	naringenin-chalcone synthase [Arthrobacter sp. LS16]
gi517598319	2.02	1	1	544	58.3	6.24	3.77	peptide ABC transporter substrate-binding protein [Arthrobacter sp. 162MFSha1.1]
gi759718843	2.48	2	1	686	73.1	5.15	3.76	peptidase S9 [Arthrobacter sp. FB24]

gi937256485	1.39	1	1	505	54.6	6.43	3.75	sugar ABC transporter ATP-binding protein [Arthrobacter sp. Edens01]
gi910739513	11.59	1	1	164	17.0	11.15	3.75	putative acetyl-CoA C-acetyltransferase VraB [Arthrobacter sp. Hiyo4]
gi119950024	3.26	2	1	645	69.6	6.46	3.75	conserved hypothetical protein [Arthrobacter aurescens TC1]
gi723606599	0.98	2	1	923	100.6	6.27	3.75	hypothetical protein ART_0376 [Arthrobacter sp. PAMC25486]
gi323469226	3.06	1	1	425	47.6	5.66	3.75	cytochrome P450 [Arthrobacter phenanthrenivorans Sphe3]
gi910693657	3.65	1	1	219	24.5	6.43	3.75	probable glutamine synthetase 2 [Arthrobacter sp. Hiyo6]
gi742860883	1.98	1	1	504	54.6	4.73	3.75	hypothetical protein [Arthrobacter sp. W1]
gi910748541	11.79	1	1	195	21.1	5.72	3.75	uvrABC system protein B [Arthrobacter sp. Hiyo8]
gi910745400	2.80	2	1	679	74.6	8.85	3.74	NAD-specific glutamate dehydrogenase [Arthrobacter sp. Hiyo8]
gi654824851	1.22	1	1	736	82.0	5.91	3.74	hypothetical protein [Arthrobacter sp. I3]
gi723610356	0.88	2	1	1026	109.9	4.94	3.74	hypothetical protein ART_4133 [Arthrobacter sp. PAMC25486]
gi470216265	2.67	3	1	486	54.4	5.00	3.74	FeS cluster formation protein [Arthrobacter gangotriensis Lz1y]
gi219858466	1.65	1	1	484	50.6	5.29	3.74	pyridine nucleotide-disulphide oxidoreductase dimerisation region [Arthrobacter chlorophenolicus
gi636847335	1.64	1	1	427	47.7	5.85	3.74	cytochrome P450 [Arthrobacter sp. TB 26]
gi517593247	4.30	1	1	279	28.8	5.66	3.74	citrate lyase subunit beta [Arthrobacter sp. 135MFCol5.1]
gi648573152	7.85	2	1	191	20.7	7.43	3.73	hypothetical protein [Arthrobacter sp. 135MFCol5.1]
gi518313152	2.47	1	1	567	61.7	6.24	3.73	MULTISPECIES: CTP synthetase [Arthrobacter]
gi910739579	2.82	1	1	355	38.7	6.04	3.73	aminoacylase-1 [Arthrobacter sp. Hiyo4]
gi654822944	2.21	1	1	453	47.5	6.04	3.73	MBL fold metallo-hydrolase [Arthrobacter sp. I3]
gi674644557	1.22	1	1	493	48.0	4.37	3.72	ABC transporter permease YtrF precursor [Arthrobacter sp. 11W110_air]
gi517606392	11.11	1	1	81	8.7	8.59	3.71	hypothetical protein [Arthrobacter sp. 161MFSha2.1]
gi651459600	2.64	1	1	795	86.0	6.40	3.71	xanthine dehydrogenase [Arthrobacter sp. 35/47]
gi654813592	2.00	1	1	300	32.3	5.97	3.71	LysR family transcriptional regulator [Arthrobacter sp. MA-N2]
gi403230588	4.07	2	1	491	51.8	9.45	3.71	MFS-type transporter [Arthrobacter sp. Rue61a]
gi910283644	3.90	1	1	436	44.7	10.26	3.71	hypothetical protein [Arthrobacter sp. A3]
gi910740555	2.60	1	1	346	37.4	4.81	3.70	xaa-Pro aminopeptidase 1 [Arthrobacter sp. Hiyo4]
gi323470118	3.06	2	1	588	62.4	4.72	3.70	ABC-type dipeptide transport system, periplasmic component [Arthrobacter phenanthrenivorans S
gi916782137	6.39	2	1	266	29.0	8.69	3.70	arginine ABC transporter ATP-binding protein [Arthrobacter sp. 35W]
gi654813298	5.95	1	1	252	26.0	5.55	3.70	4-hydroxy-tetrahydrodipicolinate reductase [Arthrobacter sp. MA-N2]
gi654816112	2.08	2	1	528	57.7	5.08	3.70	Xaa-Pro aminopeptidase [Arthrobacter sp. UNC362MFTsu5.1]
gi359303339	3.75	1	1	507	53.4	5.50	3.69	ATP-dependent protease FtsH [Arthrobacter globiformis NBRC 12137]
gi914716439	3.11	1	1	450	49.8	7.87	3.69	peptidoglycan bridge formation protein FemAB [Arthrobacter sp. ZBG10]
gi765004626	0.91	1	1	1544	166.2	5.90	3.69	glutamate synthase [Arthrobacter sp. A3]
gi470216059	5.07	1	1	276	30.0	5.27	3.68	carbon-nitrogen family hydrolase [Arthrobacter gangotriensis Lz1y]
gi742758474	3.75	1	1	506	52.7	6.67	3.68	acetyl-CoA carboxyl transferase [Arthrobacter phenanthrenivorans]
gi219858401	11.43	2	1	210	22.8	7.72	3.67	transcriptional regulator, ArsR family [Arthrobacter chlorophenolicus A6]
gi515767613	2.34	1	1	512	53.1	5.73	3.67	histidine kinase [Arthrobacter sp. M2012083]
gi651430837	3.03	1	1	528	56.5	6.20	3.67	methyltransferase [Arthrobacter sanguinis]
gi910747071	11.97	1	1	142	15.4	5.58	3.67	L-cystine import ATP-binding protein TcyC [Arthrobacter sp. Hiyo8]
gi640195489	1.97	1	1	457	48.7	5.31	3.67	6-phospho-beta-glucosidase [Arthrobacter sp. 31Y]
gi910738541	5.95	1	1	437	45.7	8.94	3.67	UPF0118 membrane protein Rv0205/MT0215 [Arthrobacter sp. Hiyo4]
gi737809599	2.95	1	1	339	36.2	8.47	3.66	tRNA(Ile)-lysidine synthetase [Arthrobacter sp. H5]
gi470216766	1.54	2	1	842	86.5	6.54	3.66	DNA internalization-related competence protein ComEC/Rec2 [Arthrobacter gangotriensis Lz1y]
gi742756912	1.58	1	1	950	100.7	5.49	3.65	glycine dehydrogenase [Arthrobacter phenanthrenivorans]
gi323468225	2.51	1	1	399	42.2	6.40	3.65	glycosyltransferase [Arthrobacter phenanthrenivorans Sphe3]
gi908699567	3.76	2	1	346	36.3	6.99	3.65	LacI family transcriptional regulator [Arthrobacter sp. RIT-PI-e]
gi162952915	4.52	1	1	155	16.6	10.74	3.64	hypothetical protein RSal33209_0683 [Renibacterium salmoninarum ATCC 33209]
gi517599234	2.10	1	1	666	69.6	5.91	3.64	hypothetical protein [Arthrobacter sp. 162MFSha1.1]
gi654828465	2.61	1	1	306	32.1	9.98	3.64	membrane protein [Arthrobacter sp. H5]
gi654823147	3.23	2	1	372	39.7	6.95	3.64	aminotransferase [Arthrobacter sp. I3]
gi654828156	2.05	2	1	537	57.3	6.37	3.64	hypothetical protein [Arthrobacter sp. H5]
gi742070142	2.73	1	1	366	39.0	6.10	3.64	oxidoreductase, FAD/FMN-binding family protein [Arthrobacter sp. MWB30]
gi648260106	6.45	1	1	248	25.5	5.26	3.64	3-alpha-hydroxysteroid dehydrogenase [Arthrobacter sp. TB 23]
gi651438384	4.89	1	1	348	37.3	8.70	3.64	ABC transporter permease [Arthrobacter sp. H14]
gi651430611	2.52	2	1	674	75.4	7.20	3.64	excinuclease ABC subunit C [Arthrobacter sanguinis]
gi918266936	13.11	1	1	61	6.3	9.82	3.63	sodium [Arthrobacter sp. Hiyo1]
gi908740288	4.75	1	1	316	34.9	10.08	3.62	hypothetical protein [Arthrobacter arilaitensis]
gi928488535	5.56	1	1	198	21.5	7.02	3.62	hypothetical protein AOC05_01015 [Arthrobacter alpinus]
gi359306475	2.57	1	1	662	67.4	5.17	3.62	putative penicillin-binding protein [Arthrobacter globiformis NBRC 12137]

gi147831825	1.29	1	1	465	48.8	6.21	3.62	hypothetical protein CMM_2711 [Clavibacter michiganensis subsp. michiganensis NCPPB 382]
gi918221813	2.98	2	1	537	58.1	7.50	3.61	hypothetical protein [Arthrobacter sp. I3]
gi443482178	4.62	1	1	346	38.7	8.95	3.61	hypothetical protein G205_07079 [Arthrobacter nitrophenolicus]
gi908697383	4.55	1	1	198	20.3	5.02	3.61	hypothetical protein [Arthrobacter sp. RIT-PI-e]
gi443482427	3.35	1	1	358	38.8	5.15	3.60	4-hydroxy-3-methylbut-2-enyl diphosphate reductase [Arthrobacter nitrophenolicus]
gi359304386	3.35	1	1	358	38.8	5.15	3.60	4-hydroxy-3-methylbut-2-enyl diphosphate reductase [Arthrobacter globiformis NBRC 12137]
gi517606242	5.81	1	1	344	35.9	5.20	3.60	hypothetical protein [Arthrobacter sp. 161MFSha2.1]
gi651484374	5.35	1	1	430	46.7	10.64	3.60	hypothetical protein [Arthrobacter sp. Br18]
gi654811295	4.04	1	1	421	46.0	5.66	3.60	hypothetical protein [Arthrobacter sp. MA-N2]
gi937258647	6.27	1	1	303	32.0	5.14	3.60	pyridoxal biosynthesis lyase PdxS [Arthrobacter sp. Edens01]
gi652424645	1.07	1	1	932	103.7	5.44	3.60	DNA/RNA helicase [Arthrobacter castelli]
gi635349726	2.95	1	1	475	50.3	5.17	3.59	aldehyde dehydrogenase family protein [Arthrobacter siccitolerans]
gi737802821	8.02	1	1	162	17.6	9.09	3.59	hypothetical protein [Arthrobacter castelli]
gi910697426	4.81	1	1	270	28.5	10.26	3.59	hypothetical protein AHiyo6_03890 [Arthrobacter sp. Hiyo6]
gi651438337	3.24	1	1	432	45.3	4.96	3.58	glycine oxidase [Arthrobacter sp. H14]
gi648574604	6.74	1	1	178	19.6	6.10	3.58	GCN5 family acetyltransferase [Arthrobacter sp. 131MFCol6.1]
gi119951816	1.78	1	1	507	54.8	5.50	3.57	conserved hypothetical protein (plasmid) [Arthrobacter aurescens TC1]
gi517602465	3.50	2	1	257	27.7	4.83	3.57	thiazole biosynthesis protein ThiJ [Arthrobacter sp. 131MFCol6.1]
gi654814248	3.74	1	1	321	33.5	8.97	3.57	molecular chaperone DnaJ [Arthrobacter sp. MA-N2]
gi927033688	5.51	1	1	363	38.2	5.76	3.57	dehydrogenase [Arthrobacter sp. LS16]
gi910694769	1.90	1	1	316	35.1	5.26	3.57	conserved hypothetical protein [Arthrobacter sp. Hiyo6]
gi654825449	5.06	2	1	178	18.9	7.14	3.56	pyridoxamine 5-phosphate oxidase [Arthrobacter sp. I3]
gi476399643	4.95	1	1	182	20.3	7.87	3.56	carboxymuconolactone decarboxylase [Arthrobacter crystallopoietes BAB-32]
gi651431766	2.45	1	1	654	71.9	8.48	3.56	transcription termination factor Rho [Arthrobacter sanguinis]
gi927296268	3.82	2	1	471	53.2	6.15	3.56	sugar phosphotransferase [Arthrobacter sp. ERGS1:01]
gi651487785	4.09	1	1	416	46.3	6.27	3.56	sulfate adenylyltransferase [Arthrobacter sp. H20]
gi542108881	3.40	1	1	294	31.3	4.74	3.56	aldolase [Arthrobacter sp. AK-YN10]
gi764161666	1.66	1	1	362	40.1	5.48	3.55	hypothetical protein ArV1_064 [Arthrobacter phage vB_ArtM-ArV1]
gi749401792	4.53	1	1	331	33.4	10.93	3.55	membrane protein [Arthrobacter sp. AK-YN10]
gi654818220	7.74	1	1	310	33.7	4.97	3.55	phenylacetate-CoA oxygenase [Arthrobacter sp. UNC362MFTsu5.1]
gi910283564	2.72	1	1	368	37.8	6.39	3.55	alcohol dehydrogenase [Arthrobacter sp. A3]
gi918469351	3.56	1	1	731	75.7	4.75	3.55	hypothetical protein [Arthrobacter crystallopoietes]
gi916835030	4.41	1	1	408	45.0	5.74	3.53	hypothetical protein [Arthrobacter sp. H14]
gi640198595	7.17	1	1	321	35.3	6.18	3.52	GDP-fucose synthetase [Arthrobacter sp. 31Y]
gi737790195	4.64	1	1	323	34.2	9.03	3.52	molecular chaperone DnaJ [Arthrobacter albus]
gi674645964	5.02	2	1	299	32.5	4.89	3.52	UTP--glucose-1-phosphate uridylyltransferase [Arthrobacter sp. 11W110_air]
gi162952583	5.04	1	1	357	40.0	8.51	3.51	ISRs2 transposase [Renibacterium salmoninarum ATCC 33209]
gi742858272	4.34	1	1	415	41.7	10.61	3.51	MFS transporter [Arthrobacter sp. W1]
gi651505529	3.49	1	1	544	56.7	5.44	3.51	ATPase [Arthrobacter sp. 35W]
gi307743563	2.10	1	1	525	57.2	9.33	3.51	putative Na+/H+ antiporter [Arthrobacter arilaitensis Re117]
gi723609158	1.04	1	1	769	82.0	9.45	3.51	membrane protein [Arthrobacter sp. PAMC25486]
gi517601104	5.99	1	1	267	29.7	4.70	3.50	hypothetical protein [Arthrobacter sp. 162MFSha1.1]
gi517605660	0.71	1	1	1552	161.9	5.07	3.50	hypothetical protein [Arthrobacter sp. 131MFCol6.1]
gi651499592	24.64	1	1	69	7.8	11.25	3.50	hypothetical protein [Arthrobacter sp. 35W]
gi116611006	18.80	1	1	117	13.1	11.15	3.49	hypothetical protein Arth_2350 [Arthrobacter sp. FB24]
gi307746287	4.17	1	1	264	30.0	7.30	3.49	hypothetical protein AARI_30560 [Arthrobacter arilaitensis Re117]
gi219858530	1.74	1	1	517	54.7	6.79	3.49	amino acid permease-associated region [Arthrobacter chlorophenolicus A6]
gi823667136	4.45	1	1	337	37.6	10.02	3.49	transposase [Arthrobacter sp. YC-RL1]
gi635352955	2.59	1	1	464	50.1	5.39	3.49	chaperone protein DnaK [Arthrobacter siccitolerans]
gi914714460	5.22	1	1	134	14.4	9.41	3.49	hypothetical protein [Arthrobacter sp. ZBG10]
gi910741723	3.92	1	1	255	27.2	8.44	3.48	acyl-coenzyme A oxidase 4, peroxisomal [Arthrobacter sp. Hiyo4]
gi551256471	0.77	1	1	1172	126.6	5.47	3.47	1-pyrroline-5-carboxylate dehydrogenase [Arthrobacter sp. PAO19]
gi470221477	2.61	1	1	459	48.8	5.77	3.47	succinate-semialdehyde dehydrogenase [Arthrobacter gangotriensis Lz1y]
gi786034823	2.77	1	1	253	27.0	9.73	3.47	hypothetical protein [Arthrobacter chlorophenolicus]
gi927293969	2.64	1	1	379	41.6	5.48	3.46	citrate synthase [Arthrobacter sp. ERGS1:01]
gi470221371	3.07	1	1	489	50.6	9.11	3.46	ErmB/QacA family multidrug efflux protein [Arthrobacter gangotriensis Lz1y]
gi930827648	2.24	1	1	624	66.0	5.91	3.45	hypothetical protein AOZ07_16080 [Arthrobacter arilaitensis]
gi742751507	7.50	2	1	200	22.7	10.68	3.45	membrane protein [Arthrobacter phenanthrenivorans]
gi914716304	0.94	1	1	1275	140.1	5.63	3.44	hypothetical protein [Arthrobacter sp. ZBG10]

gi148807627	7.78	1	1	90	10.3	5.27	3.44	AO19 [Arthrobacter oxydans]
gi518313458	2.64	1	1	417	43.4	5.50	3.43	MULTISPECIES: threonine dehydratase [Arthrobacter]
gi307746153	2.99	1	1	335	37.6	9.50	3.43	type I restriction-modification system specificity subunit [Arthrobacter arilaitensis Re117]
gi652424923	3.39	3	1	354	38.2	4.88	3.42	hypothetical protein [Arthrobacter castelli]
gi910748627	7.14	1	1	70	7.6	4.75	3.40	molybdenum import ATP-binding protein ModC [Arthrobacter sp. Hiyo8]
gi651497863	3.05	1	1	492	52.5	4.94	3.40	S-adenosyl-L-homocysteine hydrolase [Arthrobacter sp. 35W]
gi910741877	8.92	2	1	157	16.2	5.81	3.40	phosphoribosylformylglycinamidine cyclo-ligase [Arthrobacter sp. Hiyo4]
gi476398955	2.33	1	1	300	33.3	4.81	3.39	phosphotransferase enzyme family protein [Arthrobacter crystallopoietes BAB-32]
gi737765449	3.01	1	1	299	32.5	6.81	3.38	DNA-directed RNA polymerase sigma-70 factor [Arthrobacter sp. 161MFSha2.1]
gi651448582	2.45	1	1	368	38.9	6.37	3.38	LacI family transcriptional regulator [Arthrobacter nicotinovorans]
gi654816539	4.05	1	1	148	16.2	5.31	3.38	hypothetical protein [Arthrobacter sp. UNC362MFTsu5.1]
gi908696799	1.90	1	1	368	40.8	5.31	3.37	transcriptional regulator [Arthrobacter sp. RIT-PI-e]
gi910252726	1.75	1	1	571	61.2	4.98	3.36	ABC transporter substrate-binding protein [Arthrobacter siccitolerans]
gi654822205	12.31	1	1	65	6.9	11.55	3.34	hypothetical protein [Arthrobacter sp. I3]
gi307746148	7.45	2	1	94	10.6	9.33	3.33	transposase of ISAar43, IS3 family, IS407 group, orfA [Arthrobacter arilaitensis Re117]
gi654823499	1.64	1	1	609	66.4	5.68	3.33	glycerophosphodiester phosphodiesterase [Arthrobacter sp. I3]
gi517604456	1.64	1	1	609	66.4	5.62	3.33	ABC transporter ATPase [Arthrobacter sp. 131MFCol6.1]
gi518313676	25.37	3	1	67	7.2	4.72	3.32	MULTISPECIES: cold-shock protein [Arthrobacter]
gi651452664	2.24	2	1	313	35.4	8.51	3.32	hypothetical protein [Arthrobacter nicotinovorans]
gi648574956	2.97	1	1	303	30.9	11.36	3.32	hypothetical protein [Arthrobacter sp. 131MFCol6.1]
gi651484712	5.62	2	1	249	27.9	5.01	3.31	phosphoglycerate mutase [Arthrobacter sp. Br18]
gi517603512	5.25	3	1	305	33.9	10.02	3.27	hypothetical protein [Arthrobacter sp. 131MFCol6.1]
gi651485830	4.80	7	1	458	48.7	6.01	3.23	6-phospho-beta-glucosidase [Arthrobacter sp. Br18]
gi723608038	7.12	6	1	267	28.4	4.72	3.21	ABC-type metal ion transport system, periplasmic component/surface antigen [Arthrobacter sp. PA
gi917013202	1.93	1	1	414	46.5	6.44	3.17	hypothetical protein [Arthrobacter sanguinis]
gi927295778	3.66	1	1	328	35.5	8.91	3.17	exopolyphosphatase [Arthrobacter sp. ERGS1:01]
gi760166555	8.44	1	1	225	23.8	4.78	3.16	methyltransferase [Arthrobacter crystallopoietes]
gi759746402	0.73	1	1	1507	156.0	4.96	3.16	5'-nucleotidase [Arthrobacter sp. 31Y]
gi910696509	2.80	1	1	286	31.3	7.61	3.16	DNA translocase FtsK [Arthrobacter sp. Hiyo6]
gi674644704	2.80	4	1	607	63.8	5.29	3.09	Acetyl-/propionyl-coenzyme A carboxylase alpha chain [Arthrobacter sp. 11W110_air]
gi189038613	5.36	4	1	429	46.5	5.54	3.08	RecName: Full=Adenylosuccinate synthetase; Short=AMPSase; Short=AdSS; AltName: Full=IMP--
gi908698536	3.92	1	1	434	46.2	5.05	3.07	tryptophan synthase subunit beta [Arthrobacter sp. RIT-PI-e]
gi916781678	6.12	2	1	294	30.3	7.58	3.07	hypothetical protein [Arthrobacter sp. 35W]
gi927296411	2.33	6	1	901	93.1	6.54	3.04	hydrolase [Arthrobacter sp. ERGS1:01]
gi927031643	5.09	2	1	334	35.9	7.56	3.03	methionine ABC transporter ATP-binding protein [Arthrobacter sp. LS16]
gi742854935	7.53	2	1	186	20.4	4.64	3.03	deaminase [Arthrobacter sp. W1]
gi162953496	6.50	1	1	277	28.7	4.86	3.01	electron transfer flavoprotein beta-subunit [Renibacterium salmoninarum ATCC 33209]
gi918221831	7.96	3	1	226	24.8	6.11	3.00	hypothetical protein [Arthrobacter sp. I3]
gi767258175	6.00	1	1	300	33.4	8.76	2.99	hypothetical protein UM93_12055 [Arthrobacter sp. IHBB 11108]
gi651436113	4.10	3	1	415	44.8	6.54	2.99	sodium:proton exchanger [Arthrobacter sp. H41]
gi119951934	4.68	1	1	342	35.3	6.71	2.98	putative transcriptional regulator, lacI family (plasmid) [Arthrobacter aurescens TC1]
gi767258478	6.76	2	1	296	30.2	5.30	2.98	NADH-ubiquinone oxidoreductase [Arthrobacter sp. IHBB 11108]
gi910737449	9.55	2	1	199	20.3	10.40	2.98	conserved hypothetical protein [Arthrobacter sp. Hiyo4]
gi742857416	4.25	3	1	400	40.6	9.42	2.97	MFS transporter [Arthrobacter sp. W1]
gi737812921	6.12	1	1	278	30.3	9.14	2.97	ABC transporter [Arthrobacter sp. H14]
gi927031217	6.04	1	1	265	29.2	6.71	2.95	ABC transporter [Arthrobacter sp. LS16]
gi927295539	5.85	5	1	359	38.9	5.17	2.95	inositol-3-phosphate synthase [Arthrobacter sp. ERGS1:01]
gi723607174	7.88	2	1	292	29.0	5.78	2.95	orotidine 5'-phosphate decarboxylase [Arthrobacter sp. PAMC25486]
gi916814556	8.64	3	1	162	16.7	4.61	2.93	hypothetical protein [Arthrobacter nicotinovorans]
gi908642103	4.79	2	1	355	37.0	6.01	2.93	riboflavin biosynthesis protein RibD [Arthrobacter phenanthrenivorans]
gi219861268	10.29	1	1	136	14.7	6.95	2.93	monovalent cation/proton antiporter, MnhG/PhaG subunit [Arthrobacter chlorophenolicus A6]
gi927295959	15.31	1	1	98	10.7	4.84	2.92	hypothetical protein AL755_21190 [Arthrobacter sp. ERGS1:01]
gi219858481	6.93	2	1	202	22.2	5.68	2.92	transcriptional regulator, PadR-like family [Arthrobacter chlorophenolicus A6]
gi494058942	6.56	1	1	320	34.3	5.90	2.92	hypothetical protein [Actinobaculum massiliense]
gi518311534	1.86	3	1	539	57.5	5.22	2.92	hypothetical protein [Arthrobacter sp. TB 23]
gi910747394	4.38	1	1	365	39.4	8.47	2.92	sn-glycerol-3-phosphate-binding periplasmic protein UgpB [Arthrobacter sp. Hiyo8]
gi654816140	7.01	4	1	314	31.4	9.64	2.91	lipase [Arthrobacter sp. UNC362MFTsu5.1]
gi443479958	11.70	1	1	171	19.1	8.40	2.90	hypothetical protein G205_21464 [Arthrobacter nitrophenolicus]
gi515767693	2.71	1	1	627	67.4	5.33	2.90	glutamine amidotransferase [Arthrobacter sp. M2012083]

gi928488220	5.99	1	1	284	30.2	6.13	2.88	spermidine synthase [Arthrobacter alpinus]
gi759713924	2.61	1	1	536	58.3	5.55	2.88	ABC transporter substrate-binding protein [Arthrobacter sp. AK-YN10]
gi674645705	3.12	1	1	321	33.2	4.73	2.88	Thioredoxin-1 [Arthrobacter sp. 11W110_air]
gi765003655	4.59	2	1	436	47.1	9.03	2.88	hypothetical protein [Arthrobacter sp. A3]
gi543385185	8.14	1	1	307	31.6	6.21	2.87	prephenate dehydratase [Corynebacterium pseudodiphtheriticum 090104]
gi823665543	5.13	9	1	429	43.6	4.88	2.87	homoserine dehydrogenase [Arthrobacter sp. YC-RL1]
gi917572232	5.95	4	1	353	35.8	10.15	2.87	hypothetical protein [Arthrobacter sp. PAO19]
gi652423642	5.88	1	1	357	37.8	5.17	2.87	alkene reductase [Arthrobacter castelli]
gi823667850	5.88	2	1	187	20.8	9.63	2.87	DNA invertase [Arthrobacter sp. YC-RL1]
gi470220442	7.89	3	1	228	23.7	5.58	2.87	IclR family protein transcriptional regulator [Arthrobacter gangotriensis Lz1y]
gi639129689	4.75	3	1	421	43.3	9.91	2.87	arabinose ABC transporter permease [Arthrobacter sp. CAL618]
gi723608297	5.85	1	1	359	38.7	5.17	2.86	inositol-3-phosphate synthase [Arthrobacter sp. PAMC25486]
gi910249775	4.21	3	1	309	33.4	8.60	2.86	type II secretion system protein F [Arthrobacter siccitolerans]
gi518312742	9.49	1	1	158	17.5	7.46	2.86	hypothetical protein [Arthrobacter sp. TB 23]
gi517591630	6.04	1	1	265	27.4	11.27	2.85	hypothetical protein [Arthrobacter sp. 135MFCol5.1]
gi915933462	3.04	1	1	690	76.7	5.66	2.85	alpha-1,4-glucan:maltose-1-phosphate maltosyltransferase [Arthrobacter globiformis]
gi939050297	2.93	1	1	376	40.4	8.28	2.84	histidine kinase [Arthrobacter sp. JCM 19049]
gi737775041	9.87	1	1	223	23.1	10.29	2.84	hypothetical protein [Arthrobacter sp. MA-N2]
gi654825318	14.41	2	1	111	11.0	5.44	2.83	hypothetical protein [Arthrobacter sp. I3]
gi162954700	7.72	1	1	246	25.6	6.80	2.83	hypothetical cytosolic protein [Renibacterium salmoninarum ATCC 33209]
gi476403164	6.87	1	1	262	28.5	4.86	2.82	N-methyltryptophan oxidase [Arthrobacter crystallopoietes BAB-32]
gi651438297	3.52	1	1	426	45.7	4.67	2.82	enolase [Arthrobacter sp. H14]
gi219858674	9.91	2	1	111	11.9	6.79	2.82	Cupin 2 conserved barrel domain protein [Arthrobacter chlorophenolicus A6]
gi737810456	6.72	1	1	253	27.4	7.90	2.82	glycosyl transferase family 2 [Arthrobacter sp. 35/47]
gi918267626	14.47	1	1	76	8.1	6.62	2.82	transcriptional regulator protein [Arthrobacter sp. Hiyo1]
gi742758489	7.61	5	1	276	27.5	5.22	2.82	hypothetical protein RM50_03950 [Arthrobacter phenanthrenivorans]
gi470220102	8.85	2	1	192	20.5	5.21	2.82	N-acetyltransferase GCN5 [Arthrobacter gangotriensis Lz1y]
gi640199553	3.27	1	1	520	55.3	6.55	2.82	HNH endonuclease [Arthrobacter sp. 31Y]
gi928486421	1.39	2	1	866	93.0	5.67	2.81	hypothetical protein AOC05_04535 [Arthrobacter alpinus]
gi737807442	7.62	1	1	315	32.1	5.92	2.81	hypothetical protein, partial [Arthrobacter sp. H5]
gi651493711	2.02	4	1	642	71.7	5.05	2.81	histamine oxidase [Arthrobacter sp. H20]
gi742754729	9.16	1	1	251	25.9	5.91	2.81	short-chain dehydrogenase [Arthrobacter phenanthrenivorans]
gi654813707	2.97	1	1	572	61.1	5.29	2.80	histidine kinase [Arthrobacter sp. MA-N2]
gi742859503	4.38	3	1	388	40.7	5.90	2.80	FAD-dependent oxidoreductase [Arthrobacter sp. W1]
gi551255298	15.38	3	1	143	15.9	5.85	2.79	MarR family transcriptional regulator [Arthrobacter sp. PAO19]
gi635350182	4.98	1	1	221	22.9	4.98	2.79	short chain dehydrogenase family protein [Arthrobacter siccitolerans]
gi910746173	17.72	2	1	79	8.8	5.10	2.79	hypothetical protein AHiyo8_38750 [Arthrobacter sp. Hiyo8]
gi443481047	30.56	1	1	36	3.6	8.66	2.79	30S ribosomal protein S8 [Arthrobacter nitrophenolicus]
gi910252520	1.83	4	1	656	69.8	5.99	2.79	hypothetical protein [Arthrobacter siccitolerans]
gi639128933	17.86	3	1	84	9.2	9.63	2.78	hypothetical protein [Arthrobacter sp. CAL618]
gi652423751	6.55	1	1	290	30.6	5.16	2.78	shikimate dehydrogenase [Arthrobacter castelli]
gi927292740	4.07	6	1	295	30.8	5.15	2.78	flagellar hook-associated protein 3 (plasmid) [Arthrobacter sp. ERGS1:01]
gi757625320	3.17	1	1	347	36.8	4.89	2.77	glycosyl hydrolase [Arthrobacter sp. SPG23]
gi757624935	9.79	1	1	194	21.9	6.02	2.76	DivIVA domain repeat protein [Arthrobacter sp. SPG23]
gi759725120	14.97	2	1	147	16.0	11.44	2.75	hypothetical protein [Arthrobacter sp. I3]
gi916869680	3.75	4	1	267	29.6	8.81	2.75	hypothetical protein [Arthrobacter sp. Br18]
gi654814120	4.19	2	1	573	61.1	4.87	2.74	amidase [Arthrobacter sp. MA-N2]
gi690773354	2.37	1	1	716	76.7	7.12	2.74	hypothetical protein HMPREF2128_03755 [Arthrobacter albus DNF00011]
gi910844317	9.14	1	1	175	18.5	9.69	2.74	hypothetical protein ACU18_02075 [Arthrobacter sp. ZBG10]
gi927295127	3.64	2	1	550	59.3	5.12	2.73	arginyl-tRNA synthetase [Arthrobacter sp. ERGS1:01]
gi927031621	7.37	1	1	285	29.9	5.31	2.73	short-chain dehydrogenase [Arthrobacter sp. LS16]
gi927292751	3.77	3	1	531	53.7	5.43	2.73	flagellar M-ring protein FliF (plasmid) [Arthrobacter sp. ERGS1:01]
gi443479926	8.10	2	1	247	26.5	7.58	2.73	FHA domain-containing protein [Arthrobacter nitrophenolicus]
gi910740052	15.93	1	1	113	12.7	9.31	2.72	uncharacterized HTH-type transcriptional regulator YieP [Arthrobacter sp. Hiyo4]
gi551255982	7.39	4	1	230	24.0	5.48	2.72	hypothetical protein [Arthrobacter sp. PAO19]
gi910693554	15.09	1	1	106	11.7	4.88	2.72	triostin synthetase I [Arthrobacter sp. Hiyo6]
gi470220491	4.69	1	1	384	39.5	4.92	2.72	serine/threonine phosphatase [Arthrobacter gangotriensis Lz1y]
gi651482132	24.72	1	1	89	9.4	5.90	2.71	hypothetical protein [Arthrobacter sp. Br18]
gi695200271	4.04	1	1	421	45.8	6.13	2.71	unknown (plasmid) [Arthrobacter keyseri]

gi476400613	9.33	3	1	193	21.3	5.44	2.71	hypothetical protein D477_012715 [Arthrobacter crystallopoietes BAB-32]
gi910251827	4.03	2	1	472	49.1	4.91	2.71	oxidoreductase [Arthrobacter siccitolerans]
gi937262153	22.77	1	1	101	11.3	9.60	2.71	30S ribosomal protein S17 [Arthrobacter sp. Edens01]
gi723608659	7.69	1	1	247	24.9	5.08	2.71	hypothetical protein ART_2436 [Arthrobacter sp. PAMC25486]
gi359306516	2.49	2	1	603	62.5	5.02	2.70	serine/threonine protein kinase PknA [Arthrobacter globiformis NBRC 12137]
gi470216245	6.07	1	1	346	36.6	5.41	2.70	gluconeogenesis factor [Arthrobacter gangotriensis Lz1y]
gi674644425	2.44	1	1	409	41.4	10.32	2.70	Inner membrane transport protein YnfM [Arthrobacter sp. 11W110_air]
gi651439635	8.03	1	1	299	32.2	4.86	2.70	oxidoreductase [Arthrobacter sp. H14]
gi823668161	2.79	3	1	610	66.9	4.78	2.70	phosphoenolpyruvate carboxykinase [Arthrobacter sp. YC-RL1]
gi742858864	3.30	1	1	394	43.2	5.48	2.70	TRAP ABC transporter substrate-binding protein [Arthrobacter sp. W1]
gi639129656	3.81	1	1	499	54.8	7.44	2.70	hypothetical protein [Arthrobacter sp. CAL618]
gi910737954	4.26	1	1	423	46.8	9.57	2.70	tyrosine recombinase XerC-like [Arthrobacter sp. Hiyo4]
gi939051011	5.70	6	1	316	35.6	5.71	2.70	cytochrome, partial [Arthrobacter sp. JCM 19049]
gi651438639	17.28	1	1	81	8.7	5.99	2.70	hypothetical protein [Arthrobacter sp. H14]
gi916692528	2.42	1	1	495	55.8	6.93	2.69	cardiolipin synthase [Arthrobacter castelli]
gi916357008	5.12	1	1	371	38.6	4.82	2.69	hydroxyacid dehydrogenase [Arthrobacter sp. 162MFSha1.1]
gi219860720	5.31	2	1	339	36.6	5.38	2.69	Acetyl xylan esterase [Arthrobacter chlorophenolicus A6]
gi927296016	7.62	1	1	315	33.8	5.54	2.69	proline dehydrogenase [Arthrobacter sp. ERGS1:01]
gi765012192	8.50	1	1	306	33.1	7.75	2.68	XRE family transcriptional regulator [Arthrobacter sp. A3]
gi654823265	8.24	1	1	279	28.4	5.15	2.68	hypothetical protein [Arthrobacter sp. I3]
gi651504503	6.76	3	1	148	15.7	5.36	2.68	ArsR family transcriptional regulator [Arthrobacter sp. 35W]
gi767256739	4.52	1	1	442	45.9	5.82	2.68	acetylornithine aminotransferase [Arthrobacter sp. IHBB 11108]
gi939050969	8.89	2	1	135	15.0	8.02	2.68	hypothetical protein [Arthrobacter sp. JCM 19049]
gi916863406	2.55	1	1	589	64.4	9.74	2.68	ABC transporter [Arthrobacter sp. 35/47]
gi916782143	4.50	1	1	400	41.1	6.52	2.68	hypothetical protein [Arthrobacter sp. 35W]
gi927032669	8.63	1	1	197	21.9	5.74	2.68	hypothetical protein AFL94_10550 [Arthrobacter sp. LS16]
gi674646381	3.24	1	1	463	50.3	9.45	2.68	Sensor histidine kinase DesK [Arthrobacter sp. 11W110_air]
gi443482290	10.09	1	1	218	24.1	6.43	2.67	MOSC domain-containing protein [Arthrobacter nitrophenolicus]
gi651431134	14.68	1	1	109	11.4	8.84	2.67	hypothetical protein [Arthrobacter sanguinis]
gi119950541	15.32	1	1	124	13.7	6.68	2.67	hypothetical protein AAur_2847 [Arthrobacter aurescens TC1]
gi648575162	1.91	1	1	577	59.6	5.59	2.67	L-aspartate oxidase [Arthrobacter sp. 161MFSha2.1]
gi917022244	6.53	1	1	291	31.1	9.70	2.67	short-chain dehydrogenase [Arthrobacter sp. UNC362MFTsu5.1]
gi517598813	3.77	3	1	478	51.0	6.61	2.67	PucR family transcriptional regulator [Arthrobacter sp. 162MFSha1.1]
gi651448608	2.60	2	1	922	98.0	6.55	2.66	phosphoenolpyruvate synthase [Arthrobacter nicotinovorans]
gi928987042	4.86	2	1	391	41.6	9.51	2.66	hypothetical protein [Arthrobacter sp. ERGS1:01]
gi119951799	7.59	1	1	290	30.7	5.50	2.65	putative lipoprotein (plasmid) [Arthrobacter aurescens TC1]
gi742757431	7.19	3	1	334	34.8	8.09	2.65	endonuclease [Arthrobacter phenanthrenivorans]
gi651450573	4.58	1	1	262	28.3	6.74	2.65	esterase [Arthrobacter nicotinovorans]
gi759731241	5.11	1	1	470	50.1	7.65	2.65	hypothetical protein [Arthrobacter sp. L77]
gi1255446	2.84	4	1	598	65.8	5.00	2.65	maltooligosyl trehalose trehalohydrolase [Arthrobacter sp.]
gi742859342	3.28	1	1	549	59.5	5.25	2.65	peptidase M38 [Arthrobacter sp. W1]
gi651445011	2.99	1	1	670	73.4	5.16	2.65	hypothetical protein [Arthrobacter nicotinovorans]
gi648574192	3.41	6	1	499	52.2	9.42	2.65	sulfate permease [Arthrobacter sp. 162MFSha1.1]
gi654828337	15.27	1	1	131	13.5	5.15	2.65	twitching motility protein PilT [Arthrobacter sp. H5]
gi917442286	5.06	1	1	356	38.3	5.67	2.64	NAD kinase [Arthrobacter albus]
gi757623205	4.36	1	1	344	37.0	6.81	2.64	exopolyphosphatase [Arthrobacter sp. SPG23]
gi759725681	4.88	2	1	430	45.9	9.00	2.64	two-component system sensor histidine kinase [Arthrobacter sp. I3]
gi323468304	2.35	1	1	723	75.4	5.12	2.64	translation elongation factor 2 (EF-2/EF-G) [Arthrobacter phenanthrenivorans Sphe3]
gi914713430	13.82	2	1	152	16.5	6.57	2.64	AsnC family transcriptional regulator [Arthrobacter sp. ZBG10]
gi654815711	5.14	3	1	311	33.6	9.73	2.64	hypothetical protein [Arthrobacter sp. UNC362MFTsu5.1]
gi930828291	2.74	2	1	401	43.6	5.17	2.64	acyl-CoA dehydrogenase [Arthrobacter arilaitensis]
gi517589819	3.66	3	1	328	34.2	4.75	2.64	Dak phosphatase [Arthrobacter sp. 135MFCol5.1]
gi760166597	6.42	1	1	296	32.2	6.57	2.63	hypothetical protein [Arthrobacter crystallopoietes]
gi918266062	10.37	5	1	164	18.2	8.24	2.63	conserved hypothetical protein [Arthrobacter sp. Hiyo1]
gi914717629	4.20	2	1	262	27.3	10.18	2.63	glycosyl transferase [Arthrobacter sp. ZBG10]
gi737790002	4.74	3	1	211	22.6	5.05	2.63	DNA-binding response regulator [Arthrobacter albus]
gi359304290	5.10	1	1	314	33.9	10.02	2.63	chaperone protein DnaJ [Arthrobacter globiformis NBRC 12137]
gi403230254	4.87	1	1	267	29.2	6.39	2.63	manganese transport system ATP-binding protein MntB [Arthrobacter sp. Rue61a]
gi910697041	22.86	1	1	70	7.7	5.95	2.63	hypothetical protein AHiyo6_07230 [Arthrobacter sp. Hiyo6]

gi917530492	3.51	3	1	485	52.1	9.23	2.63	L-asparagine permease [Arthrobacter sp. PAMC25486]
gi323467860	5.51	1	1	272	28.8	8.22	2.63	A/G-specific DNA glycosylase [Arthrobacter phenanthrenivorans Sphe3]
gi654828523	11.06	6	1	208	23.7	7.58	2.63	hypothetical protein [Arthrobacter sp. H5]
gi674643883	4.56	1	1	263	29.1	10.62	2.62	hypothetical protein BN1051_00019 [Arthrobacter sp. 11W110_air]
gi651484709	4.48	1	1	268	29.7	10.14	2.62	methylase [Arthrobacter sp. Br18]
gi636844991	4.55	1	1	483	54.2	5.64	2.62	hypothetical protein [Arthrobacter sp. TB 26]
gi307744596	20.65	1	1	92	10.4	9.29	2.62	hypothetical protein AARI_13620 [Arthrobacter arilaitensis Re117]
gi910693917	3.93	2	1	483	54.0	6.16	2.62	uncharacterized ABC transporter ATP-binding protein HI_1252 [Arthrobacter sp. Hiyo6]
gi916871769	5.50	2	1	218	24.0	10.65	2.62	hypothetical protein [Arthrobacter sp. H5]
gi323468603	7.69	2	1	273	29.5	8.29	2.62	short-chain dehydrogenase of unknown substrate specificity [Arthrobacter phenanthrenivorans Sph
gi908697714	10.93	1	1	183	20.3	6.54	2.62	TetR family transcriptional regulator [Arthrobacter sp. RIT-PI-e]
gi916781961	5.83	3	1	343	35.3	5.86	2.61	carbohydrate kinase [Arthrobacter sp. 35W]
gi476399615	1.97	5	1	659	68.9	6.20	2.61	copper-translocating P-type ATPase [Arthrobacter crystallopoietes BAB-32]
gi219857741	3.80	2	1	316	34.3	4.84	2.61	flavin reductase domain protein FMN-binding [Arthrobacter chlorophenolicus A6]
gi652425698	5.35	2	1	355	36.3	4.64	2.61	hypothetical protein [Arthrobacter castelli]
gi723607792	26.83	1	1	82	9.2	8.54	2.61	hypothetical protein ART_1569 [Arthrobacter sp. PAMC25486]
gi917760412	7.76	2	1	245	25.9	6.27	2.61	oxidoreductase [Arthrobacter sp. L77]
gi786032167	3.99	1	1	276	30.0	6.44	2.61	hypothetical protein [Arthrobacter chlorophenolicus]
gi323469641	2.78	5	1	432	46.6	6.80	2.61	dinucleotide-utilizing enzyme possibly involved in molybdopterin or thiamin biosynthesis [Arthroba
gi765008802	12.56	3	1	199	21.9	5.48	2.61	recombinase RecR [Arthrobacter sp. A3]
gi937262298	4.70	1	1	234	26.5	5.07	2.60	chlorite dismutase [Arthrobacter sp. Edens01]
gi518313803	20.37	1	1	108	11.7	4.32	2.60	MULTISPECIES: rhodanese-like domain-containing protein [Arthrobacter]
gi515767148	11.18	1	1	161	17.5	9.67	2.60	membrane protein insertion efficiency factor YidD [Arthrobacter sp. M2012083]
gi742851573	9.39	3	1	213	22.4	4.63	2.60	septum formation inhibitor Maf [Arthrobacter sp. W1]
gi930824865	3.31	1	1	634	68.1	4.69	2.60	hypothetical protein AOZ07_00115 [Arthrobacter arilaitensis]
gi654814681	2.55	3	1	509	56.7	5.11	2.60	lysine--tRNA ligase [Arthrobacter sp. MA-N2]
gi651487766	5.00	1	1	280	31.2	9.58	2.60	hypothetical protein [Arthrobacter sp. H20]
gi119949676	5.01	1	1	379	40.4	6.90	2.60	putative cell envelope-related transcriptional attenuator domain protein [Arthrobacter aurescens T
gi916834653	3.00	2	1	367	38.5	5.02	2.60	N-succinyldiaminopimelate aminotransferase [Arthrobacter sp. H14]
gi937259528	4.72	1	1	508	53.9	4.83	2.60	aldehyde dehydrogenase [Arthrobacter sp. Edens01]
gi651438352	4.10	3	1	488	53.0	6.11	2.60	glyceraldehyde-3-phosphate dehydrogenase [Arthrobacter sp. H14]
gi674645562	4.16	3	1	409	43.7	8.15	2.60	carbohydrate diacid transcriptional activator CdaR [Arthrobacter sp. 11W110_air]
gi654827225	3.80	1	1	500	52.6	5.41	2.60	dehydrogenase [Arthrobacter sp. H5]
gi916782113	7.23	4	1	249	26.8	4.82	2.60	ANTAR domain-containing protein [Arthrobacter sp. 35W]
gi517604547	5.66	1	1	371	40.3	6.28	2.60	MULTISPECIES: tryptophan--tRNA ligase [Arthrobacter]
gi910250703	4.02	1	1	323	34.5	6.23	2.60	glycosyl transferase [Arthrobacter siccitolerans]
gi939051074	3.00	2	1	633	68.8	5.67	2.60	hypothetical protein [Arthrobacter sp. JCM 19049]
gi918265086	7.64	1	1	314	33.1	6.13	2.60	hypothetical protein AHiyo1_51680 [Arthrobacter sp. Hiyo1]
gi515765114	6.15	3	1	195	20.4	5.57	2.59	phosphoheptose isomerase [Arthrobacter sp. M2012083]
gi917442096	10.67	1	1	178	19.7	6.80	2.59	hypothetical protein [Arthrobacter albus]
gi119947983	4.76	2	1	336	36.6	5.94	2.59	hypothetical protein AAur_0341 [Arthrobacter aurescens TC1]
gi760113112	5.31	1	1	207	22.9	9.94	2.59	hypothetical protein [Arthrobacter chlorophenolicus]
gi767258497	3.77	1	1	371	37.8	7.09	2.59	phosphate ABC transporter substrate-binding protein [Arthrobacter sp. IHBB 11108]
gi651435860	6.58	1	1	152	17.1	6.80	2.59	transcriptional regulator [Arthrobacter sp. H41]
gi307744579	4.43	1	1	429	46.6	5.03	2.59	quinolinate synthetase A subunit [Arthrobacter arilaitensis Re117]
gi651487277	17.65	1	1	102	11.8	5.86	2.59	hypothetical protein, partial [Arthrobacter sp. Br18]
gi930825796	4.08	4	1	319	35.3	9.95	2.59	ABC transporter permease [Arthrobacter arilaitensis]
gi359306798	3.97	1	1	605	64.5	5.35	2.59	putative amidase [Arthrobacter globiformis NBRC 12137]
gi654828015	3.62	1	1	276	31.0	5.43	2.58	hypothetical protein [Arthrobacter sp. H5]
gi914713767	3.96	1	1	303	32.3	6.05	2.58	glycosidase [Arthrobacter sp. ZBG10]
gi757624395	2.55	2	1	510	53.5	6.48	2.58	acetyl-CoA carboxyl transferase [Arthrobacter sp. SPG23]
gi674645240	4.85	1	1	206	22.9	5.64	2.58	Threonylcarbamoyl-AMP synthase [Arthrobacter sp. 11W110_air]
gi760164809	14.29	1	1	126	14.3	4.65	2.58	hypothetical protein [Arthrobacter crystallopoietes]
gi307746454	3.59	1	1	306	32.4	5.21	2.58	conserved hypothetical protein [Arthrobacter arilaitensis Re117]
gi648572686	3.96	1	1	404	42.7	6.57	2.58	dephospho-CoA kinase [Arthrobacter sp. 135MFCol5.1]
gi737787196	4.98	1	1	462	50.3	7.66	2.58	NADH dehydrogenase [Arthrobacter albus]
gi757626147	6.59	1	1	258	27.7	9.55	2.58	IclR family transcriptional regulator [Arthrobacter sp. SPG23]
gi910249857	4.93	1	1	284	31.9	5.60	2.58	phosphate:nucleotide phosphotransferase [Arthrobacter siccitolerans]
gi443482569	5.62	1	1	409	42.3	5.94	2.58	class V aminotransferase [Arthrobacter nitrophenolicus]

gi654826692	7.31	2	1	342	37.2	8.85	2.58	cytochrome C biogenesis protein [Arthrobacter sp. H5]
gi759734732	3.94	2	1	254	26.4	6.90	2.58	16S rRNA methyltransferase [Arthrobacter sp. L77]
gi359303647	3.70	1	1	405	42.9	4.82	2.58	signal recognition particle receptor [Arthrobacter globiformis NBRC 12137]
gi910743671	9.09	1	1	275	28.6	8.12	2.58	4-hydroxy-tetrahydrodipicolinate reductase [Arthrobacter sp. Hiyo8]
gi307743705	2.98	2	1	403	42.1	5.77	2.58	acetyl-CoA C-acyltransferase [Arthrobacter arilaitensis Re117]
gi737792943	7.36	3	1	258	28.6	6.55	2.58	glycosyl transferase [Arthrobacter nicotinovorans]
gi918268179	9.66	4	1	176	18.9	7.36	2.58	hypothetical protein AHiyo1_15300 [Arthrobacter sp. Hiyo1]
gi517599564	6.21	1	1	354	38.9	7.33	2.57	ABC transporter [Arthrobacter sp. 162MFSha1.1]
gi723606913	9.77	3	1	174	18.6	5.24	2.57	Transcriptional regulator, TetR family [Arthrobacter sp. PAMC25486]
gi723608124	32.47	1	1	77	8.3	10.64	2.57	hypothetical protein ART_1901 [Arthrobacter sp. PAMC25486]
gi517601169	4.96	1	1	403	42.1	5.88	2.57	aldehyde dismutase [Arthrobacter sp. 162MFSha1.1]
gi765005105	3.23	1	1	526	56.9	5.94	2.57	hypothetical protein [Arthrobacter sp. A3]
gi759730302	3.04	1	1	494	53.3	5.41	2.57	ribonuclease II [Arthrobacter sp. L77]
gi651430564	8.00	3	1	200	21.4	9.25	2.57	CDP-alcohol phosphatidyltransferase [Arthrobacter sanguinis]
gi654812248	3.30	2	1	364	38.4	10.96	2.57	fusaric acid resistance protein [Arthrobacter sp. MA-N2]
gi823668020	3.51	1	1	427	44.6	4.91	2.57	allantoate amidohydrolase [Arthrobacter sp. YC-RL1]
gi765011439	6.08	1	1	263	27.6	5.69	2.57	cytochrome C [Arthrobacter sp. A3]
gi910697653	3.54	2	1	508	55.2	9.66	2.57	putative ribose/galactose/methyl galactoside import ATP-binding protein 2 [Arthrobacter sp. Hiyo6
gi740685469	2.03	2	1	543	57.3	5.49	2.57	acetolactate synthase [Arthrobacter sp. PAMC25486]
gi219859110	8.75	3	1	160	18.0	5.86	2.57	regulatory protein RecX [Arthrobacter chlorophenolicus A6]
gi515767468	5.83	1	1	240	26.3	5.81	2.56	hypothetical protein [Arthrobacter sp. M2012083]
gi917013271	1.14	1	1	963	106.0	6.99	2.56	ABC-ATPase UvrA [Arthrobacter sanguinis]
gi927294844	4.81	1	1	270	28.9	5.17	2.56	glycosyl transferase family 8 [Arthrobacter sp. ERGS1:01]
gi517589897	25.00	2	1	92	9.6	8.25	2.56	hypothetical protein [Arthrobacter sp. 135MFCol5.1]
gi910741440	4.87	6	1	308	33.9	5.97	2.56	alpha-galactosidase [Arthrobacter sp. Hiyo4]
gi654827508	3.96	1	1	328	37.1	5.60	2.56	glutathione S-transferase [Arthrobacter sp. H5]
gi651465217	2.67	1	1	487	51.9	5.60	2.56	argininosuccinate lyase [Arthrobacter sp. 35/47]
gi723606640	9.03	2	1	155	17.3	5.45	2.56	quorum-sensing autoinducer 2 (AI-2) [Arthrobacter sp. PAMC25486]
gi476402852	3.87	2	1	362	39.3	8.37	2.56	3-methyladenine DNA glycosylase, partial [Arthrobacter crystallopoietes BAB-32]
gi910692674	3.81	4	1	236	25.2	6.79	2.56	HTH-type transcriptional regulator KipR [Arthrobacter sp. Hiyo6]
gi651485358	5.38	2	1	390	41.8	5.03	2.56	acyl-CoA dehydrogenase [Arthrobacter sp. Br18]
gi910696616	11.85	1	1	135	14.8	7.15	2.56	low molecular weight protein-tyrosine-phosphatase etp, partial [Arthrobacter sp. Hiyo6]
gi939051732	11.24	1	1	178	18.5	6.09	2.56	hypothetical protein [Arthrobacter sp. JCM 19049]
gi823666433	3.66	1	1	383	40.6	6.07	2.56	inosine-5-monophosphate dehydrogenase [Arthrobacter sp. YC-RL1]
gi517598313	6.75	1	1	237	26.8	9.79	2.55	hypothetical protein [Arthrobacter sp. 162MFSha1.1]
gi917441938	13.58	1	1	162	17.2	9.85	2.55	hypothetical protein [Arthrobacter albus]
gi307745837	3.98	1	1	452	47.2	5.78	2.55	FAD linked oxidase domain-containing protein [Arthrobacter arilaitensis Re117]
gi695210494	22.22	1	1	108	11.4	9.57	2.55	hypothetical protein (plasmid) [Arthrobacter aurescens]
gi767258968	1.44	1	1	1250	135.3	5.44	2.55	glutamate synthase [Arthrobacter sp. IHBB 11108]
gi651496120	9.84	1	1	183	19.5	10.26	2.55	hypothetical protein [Arthrobacter sp. H20]
gi515766581	3.50	1	1	343	36.7	6.14	2.55	LacI family transcriptional regulator [Arthrobacter sp. M2012083]
gi908690513	6.19	1	1	323	33.8	7.65	2.55	dehydrogenase, partial [Arthrobacter sp. H41]
gi749401269	7.64	1	1	275	30.9	5.86	2.55	peptidase S9, partial [Arthrobacter sp. AK-YN10]
gi749401882	6.99	3	1	143	16.1	6.80	2.55	transporter, partial [Arthrobacter sp. AK-YN10]
gi753939882	9.09	1	1	132	14.6	9.23	2.55	transposase [Arthrobacter phenanthrenivorans]
gi916365748	8.88	2	1	214	22.4	10.07	2.54	MULTISPECIES: hypothetical protein [Arthrobacter]
gi927293187	4.55	2	1	527	55.7	9.44	2.54	MFS transporter (plasmid) [Arthrobacter sp. ERGS1:01]
gi914713619	2.46	1	1	487	52.3	9.41	2.54	hypothetical protein [Arthrobacter sp. ZBG10]
gi786026122	13.61	1	1	169	17.4	4.91	2.54	hypothetical protein [Arthrobacter chlorophenolicus]
gi759702679	3.22	1	1	342	37.6	6.20	2.54	oxidoreductase [Arthrobacter globiformis]
gi937258104	3.72	2	1	645	71.3	6.79	2.54	DNA primase [Arthrobacter sp. Edens01]
gi910745953	17.39	1	1	69	6.9	9.98	2.54	hydantoin racemase [Arthrobacter sp. Hiyo8]
gi162955701	8.39	2	1	286	30.2	5.49	2.54	universal stress protein family [Renibacterium salmoninarum ATCC 33209]
gi927032211	10.76	1	1	158	17.4	4.55	2.54	hypothetical protein AFL94_07805 [Arthrobacter sp. LS16]
gi742068729	2.85	1	1	667	68.7	4.91	2.54	beta-N-acetylglucosaminidase [Arthrobacter sp. MWB30]
gi917739306	10.50	2	1	200	21.8	11.41	2.54	hypothetical protein [Arthrobacter sp. W1]
gi930827085	3.13	1	1	416	44.3	6.77	2.54	type II/IV secretion system protein E [Arthrobacter arilaitensis]
gi517600961	6.33	3	1	158	16.5	7.52	2.54	aminoacyl-tRNA deacylase [Arthrobacter sp. 162MFSha1.1]
gi517604807	16.28	1	1	86	8.6	11.24	2.54	hypothetical protein [Arthrobacter sp. 131MFCol6.1]

gi443482583	6.50	5	1	200	20.3	11.44	2.54	amidophosphoribosyltransferase [Arthrobacter nitrophenolicus]
gi542107842	4.88	1	1	471	49.6	9.41	2.53	amino acid transporter [Arthrobacter sp. AK-YN10]
gi651433680	4.56	1	1	263	28.3	5.12	2.53	DNA-binding protein [Arthrobacter sp. H41]
gi551256741	6.82	1	1	132	15.0	9.20	2.53	AraC family transcriptional regulator [Arthrobacter sp. PAO19]
gi648575129	3.73	2	1	509	53.0	5.29	2.53	amidohydrolase [Arthrobacter sp. 131MFCol6.1]
gi551256577	7.50	1	1	280	30.7	4.75	2.53	phenazine biosynthesis protein PhzF [Arthrobacter sp. PAO19]
gi654811973	3.41	3	1	411	44.7	5.12	2.53	phenylacetic acid degradation protein [Arthrobacter sp. MA-N2]
gi654816308	4.87	1	1	267	30.4	5.59	2.53	thymidylate synthase [Arthrobacter sp. UNC362MFTsu5.1]
gi914715213	7.07	1	1	184	21.0	10.35	2.53	4-hydroxybenzoyl-CoA thioesterase [Arthrobacter sp. ZBG10]
gi470220502	1.35	1	1	1181	121.3	5.26	2.53	putative membrane protein mmpL3 [Arthrobacter gangotriensis Lz1y]
gi916872086	16.94	1	1	124	13.3	10.95	2.53	hypothetical protein [Arthrobacter sp. H5]
gi470217154	12.43	1	1	169	17.6	7.33	2.53	ybaK/ebsC family protein [Arthrobacter gangotriensis Lz1y]
gi937259177	10.20	1	1	255	28.5	5.38	2.53	MerR family transcriptional regulator [Arthrobacter sp. Edens01]
gi515764472	4.66	2	1	451	48.9	9.80	2.53	acyltransferase [Arthrobacter sp. M2012083]
gi916573801	6.88	1	1	276	29.7	5.85	2.52	enoyl-CoA hydratase [Arthrobacter sp. TB 26]
gi723606541	4.61	1	1	369	38.9	5.30	2.52	hypothetical protein ART_0318 [Arthrobacter sp. PAMC25486]
gi916574026	4.13	1	1	242	25.2	5.15	2.52	hypothetical protein [Arthrobacter sp. TB 26]
gi162955447	2.92	1	1	377	39.1	7.03	2.52	glutamate 5-kinase [Renibacterium salmoninarum ATCC 33209]
gi928487451	2.42	5	1	413	43.0	6.28	2.52	glycine oxidase [Arthrobacter alpinus]
gi517605608	3.85	1	1	338	36.0	4.74	2.52	MULTISPECIES: hypothetical protein [Arthrobacter]
gi359303929	3.62	1	1	387	39.5	8.56	2.52	putative ABC transporter substrate-binding protein [Arthrobacter globiformis NBRC 12137]
gi927294211	6.08	1	1	296	31.6	8.24	2.52	LysR family transcriptional regulator [Arthrobacter sp. ERGS1:01]
gi654818841	4.50	3	1	289	31.8	5.27	2.52	30S ribosomal protein S2 [Arthrobacter sp. UNC362MFTsu5.1]
gi515767296	26.03	1	1	73	8.0	9.41	2.52	hypothetical protein [Arthrobacter sp. M2012083]
gi937258485	6.65	1	1	346	36.4	5.34	2.52	glycerol-3-phosphate dehydrogenase [Arthrobacter sp. Edens01]
gi916691178	2.42	1	1	454	46.7	8.16	2.52	hypothetical protein [Arthrobacter castelli]
gi651439822	13.73	1	1	153	16.4	4.77	2.52	DNA mismatch repair protein MutT [Arthrobacter sp. H14]
gi927031425	3.33	1	1	451	47.4	5.11	2.52	4-aminobutyrate aminotransferase [Arthrobacter sp. LS16]
gi757624289	3.30	1	1	303	32.0	9.70	2.52	dehydrogenase [Arthrobacter sp. SPG23]
gi753932539	5.98	1	1	234	26.0	9.41	2.52	hypothetical protein [Arthrobacter arilaitensis]
gi119947489	7.28	1	1	206	23.5	10.93	2.52	ribosomal protein S4 [Arthrobacter aurescens TC1]
gi930825449	5.60	1	1	357	39.4	8.41	2.52	ACP synthase [Arthrobacter arilaitensis]
gi323468918	9.77	1	1	174	18.0	5.73	2.51	molybdopterin adenylyltransferase [Arthrobacter phenanthrenivorans Sphe3]
gi654828104	4.96	2	1	262	28.6	5.27	2.51	tRNA (guanine(37)-N(1))-methyltransferase [Arthrobacter sp. H5]
gi651482396	7.90	1	1	291	32.0	5.31	2.51	glycerophosphodiester phosphodiesterase [Arthrobacter sp. Br18]
gi765011628	3.82	2	1	314	32.5	4.73	2.51	ribokinase [Arthrobacter sp. A3]
gi910739897	14.37	1	1	160	17.8	9.20	2.51	transcriptional repressor SdpR [Arthrobacter sp. Hiyo4]
gi767257207	2.42	1	1	413	45.2	5.58	2.51	RNA polymerase subunit sigma-24 [Arthrobacter sp. IHBB 11108]
gi307744449	3.19	1	1	313	34.1	9.07	2.51	putative type II secretion system protein F [Arthrobacter arilaitensis Re117]
gi723610158	3.10	3	1	710	75.4	5.35	2.51	hypothetical protein ART_3935 [Arthrobacter sp. PAMC25486]
gi674646457	2.94	1	1	578	61.9	9.70	2.51	3-(3-hydroxy-phenyl)propionate/3-hydroxycinnamic acid hydroxylase [Arthrobacter sp. 11W110_ai
gi823665906	1.61	2	1	559	61.8	5.78	2.51	ATPase AAA [Arthrobacter sp. YC-RL1]
gi443480834	10.86	1	1	175	19.0	10.05	2.51	Ppx/GppA phosphatase [Arthrobacter nitrophenolicus]
gi443480316	4.78	1	1	356	37.7	9.38	2.51	dihydroorotate dehydrogenase 2 [Arthrobacter nitrophenolicus]
gi767257231	2.25	2	1	400	41.7	5.01	2.51	transcriptional regulator [Arthrobacter sp. IHBB 11108]
gi910694206	6.37	2	1	251	27.8	5.97	2.51	chaperone protein ClpB [Arthrobacter sp. Hiyo6]
gi219861200	4.38	1	1	297	31.5	4.70	2.51	dihydrodipicolinate synthase [Arthrobacter chlorophenolicus A6]
gi476399048	5.41	1	1	296	29.6	4.74	2.51	glycerate kinase [Arthrobacter crystallopoietes BAB-32]
gi651502924	4.55	1	1	550	59.3	5.16	2.51	arginine--tRNA ligase [Arthrobacter sp. 35W]
gi651484937	4.72	1	1	339	36.2	10.36	2.50	hypothetical protein [Arthrobacter sp. Br18]
gi476400856	8.45	1	1	213	24.0	9.60	2.50	hypothetical protein D477_011586 [Arthrobacter crystallopoietes BAB-32]
gi551254053	3.32	1	1	602	65.9	8.46	2.50	ABC transporter [Arthrobacter sp. PAO19]
gi219861881	7.47	5	1	241	25.6	4.94	2.50	hypothetical protein Achl_4271 (plasmid) [Arthrobacter chlorophenolicus A6]
gi654818680	2.46	2	1	284	32.5	6.23	2.50	tryptophan 2,3-dioxygenase [Arthrobacter sp. UNC362MFTsu5.1]
gi908699412	5.56	1	1	216	24.2	5.05	2.50	hypothetical protein [Arthrobacter sp. RIT-PI-e]
gi759728001	4.24	1	1	377	40.5	5.87	2.50	erythromycin biosynthesis sensory transduction protein eryC1 [Arthrobacter sp. UNC362MFTsu5.1]
gi759725371	4.95	2	1	202	20.7	10.14	2.50	hypothetical protein [Arthrobacter sp. I3]
gi742071204	13.37	1	1	172	19.4	5.24	2.50	hypothetical protein ANMWB30_24020 [Arthrobacter sp. MWB30]
gi927034153	10.43	1	1	163	17.4	9.85	2.50	hypothetical protein AFL94_13590 [Arthrobacter sp. LS16]

gi359306832	6.69	2	1	299	31.6	5.10	2.50	putative oxidoreductase [Arthrobacter globiformis NBRC 12137]
gi674644808	6.45	2	1	155	16.9	5.07	2.50	Ribosome-binding factor A [Arthrobacter sp. 11W110_air]
gi636845694	10.73	2	1	177	18.0	5.01	2.50	arginine repressor [Arthrobacter sp. TB 26]
gi636845470	4.70	1	1	234	25.8	9.98	2.50	hypothetical protein [Arthrobacter sp. TB 26]
gi639128949	4.76	1	1	378	40.0	7.58	2.50	secretion system protein E [Arthrobacter sp. CAL618]
gi443479811	1.80	1	1	555	59.4	5.14	2.50	monooxygenase FAD-binding protein [Arthrobacter nitrophenolicus]
gi908698374	16.49	2	1	97	10.3	8.51	2.50	hypothetical protein [Arthrobacter sp. RIT-PI-e]
gi651439092	7.51	1	1	293	31.1	5.39	2.50	universal stress protein UspA [Arthrobacter sp. H14]
gi476399242	12.07	3	1	116	12.5	6.55	2.50	4'-phosphopantetheinyl transferase [Arthrobacter crystallopoietes BAB-32]
gi759729620	15.53	1	1	103	11.4	4.51	2.49	antibiotic biosynthesis monooxygenase [Arthrobacter sp. L77]
gi927033486	7.72	1	1	298	31.5	5.00	2.49	oxidoreductase [Arthrobacter sp. LS16]
gi518311829	7.94	1	1	214	22.5	5.16	2.49	MULTISPECIES: para-aminobenzoate synthase [Arthrobacter]
gi740683083	4.98	1	1	442	45.9	6.61	2.49	adenosylmethionine--8-amino-7-oxononanoate aminotransferase BioA [Arthrobacter sp. PAMC254
gi651487091	6.76	1	1	207	21.8	6.55	2.49	16S rRNA methyltransferase [Arthrobacter sp. Br18]
gi908698806	6.23	1	1	321	32.7	5.07	2.49	ACP S-malonyltransferase [Arthrobacter sp. RIT-PI-e]
gi517593149	5.00	1	1	260	28.2	6.42	2.49	peptide ABC transporter ATP-binding protein [Arthrobacter sp. 135MFCol5.1]
gi918268649	7.00	2	1	243	27.2	7.46	2.49	hypothetical protein AHiyo1_01620 [Arthrobacter sp. Hiyo1]
gi927294599	4.07	2	1	270	30.3	8.56	2.49	UDP pyrophosphate synthase [Arthrobacter sp. ERGS1:01]
gi651458121	5.68	1	1	352	36.5	6.00	2.49	anthranilate phosphoribosyltransferase [Arthrobacter sp. 35/47]
gi927293467	4.64	1	1	431	45.3	9.50	2.49	hypothetical protein AL755_04460 [Arthrobacter sp. ERGS1:01]
gi545107624	6.31	1	1	301	33.2	6.19	2.49	acyl-CoA thioesterase II [Arthrobacter sp. AK-YN10]
gi937259337	7.62	1	1	223	24.0	9.73	2.49	hypothetical protein AO716_11365 [Arthrobacter sp. Edens01]
gi219860839	2.99	1	1	602	67.0	5.24	2.49	glycoside hydrolase 15-related [Arthrobacter chlorophenolicus A6]
gi116613015	20.59	1	1	102	10.9	10.13	2.49	hypothetical protein Arth_4287 (plasmid) [Arthrobacter sp. FB24]
gi919219103	3.47	3	1	375	39.1	8.41	2.49	S26 family signal peptidase [Arthrobacter sp. YC-RL1]
gi470221257	12.67	1	1	150	16.8	6.54	2.49	Organic hydroperoxide resistance transcriptional regulator [Arthrobacter gangotriensis Lz1y]
gi443482751	1.72	3	1	523	54.5	6.13	2.49	transcriptional regulator CdaR [Arthrobacter nitrophenolicus]
gi928987254	20.00	3	1	85	9.3	5.06	2.49	hypothetical protein [Arthrobacter sp. ERGS1:01]
gi162955487	11.27	2	1	142	15.2	5.33	2.48	NAD dependent epimerase/dehydratase [Renibacterium salmoninarum ATCC 33209]
gi116609729	5.65	2	1	283	30.7	5.41	2.48	Methyltransferase type 11 [Arthrobacter sp. FB24]
gi910696927	5.88	1	1	255	27.5	8.47	2.48	amine oxidase [Arthrobacter sp. Hiyo6]
gi651501389	2.82	1	1	355	37.7	7.56	2.48	dihydroorotate dehydrogenase 2 [Arthrobacter sp. 35W]
gi359304403	19.23	5	1	78	8.6	11.44	2.48	hypothetical protein ARGLB_085_02690 [Arthrobacter globiformis NBRC 12137]
gi908699327	1.74	2	1	691	73.7	5.02	2.48	phosphate acetyltransferase [Arthrobacter sp. RIT-PI-e]
gi654827717	1.74	3	1	689	73.5	5.31	2.48	phosphate acetyltransferase [Arthrobacter sp. H5]
gi635352829	6.08	2	1	411	45.2	5.80	2.48	type II/IV secretion system family protein [Arthrobacter siccitolerans]
gi542107937	2.50	1	1	320	34.6	5.15	2.48	2-hydroxyacid dehydrogenase [Arthrobacter sp. AK-YN10]
gi640200534	6.15	3	1	309	32.8	8.19	2.48	hypothetical protein [Arthrobacter sp. 31Y]
gi910252497	2.00	1	1	499	52.5	7.39	2.48	sulfate permease [Arthrobacter siccitolerans]
gi651430082	5.71	1	1	333	35.0	4.63	2.48	dihydroxyacetone kinase subunit DhaK [Arthrobacter sanguinis]
gi916359088	6.34	1	1	284	30.3	8.84	2.48	hypothetical protein [Arthrobacter sp. 135MFCol5.1]
gi116613203	7.69	3	1	273	28.7	6.38	2.48	aminoglycoside phosphotransferase (plasmid) [Arthrobacter sp. FB24]
gi674645167	3.22	1	1	466	50.0	5.06	2.48	Dihydrolipoyl dehydrogenase [Arthrobacter sp. 11W110_air]
gi910747287	11.90	1	1	84	8.7	5.10	2.48	hypothetical protein AHiyo8_49890 [Arthrobacter sp. Hiyo8]
gi737810593	13.94	1	1	165	18.7	7.31	2.48	MarR family transcriptional regulator [Arthrobacter sp. 35/47]
gi737802431	3.08	1	1	357	37.7	6.65	2.48	hypothetical protein [Arthrobacter castelli]
gi767257694	2.87	1	1	592	63.6	5.31	2.48	ABC transporter substrate-binding protein [Arthrobacter sp. IHBB 11108]
gi910747523	5.70	1	1	228	23.4	4.72	2.48	metalloreductase STEAP4 [Arthrobacter sp. Hiyo8]
gi517590440	3.13	1	1	734	77.9	5.36	2.48	hypothetical protein [Arthrobacter sp. 135MFCol5.1]
gi759704613	4.20	1	1	286	31.4	7.28	2.48	pseudouridine synthase [Arthrobacter globiformis]
gi119951649	1.90	1	1	578	63.1	5.87	2.48	thiamine biosynthesis (plasmid) [Arthrobacter aurescens TC1]
gi908698780	5.86	1	1	273	28.3	4.96	2.47	hypothetical protein, partial [Arthrobacter sp. RIT-PI-e]
gi651439625	2.66	1	1	414	43.0	4.91	2.47	Zn-dependent hydrolase [Arthrobacter sp. H14]
gi927033682	5.04	1	1	337	36.3	5.07	2.47	inositol 2-dehydrogenase [Arthrobacter sp. LS16]
gi636845734	6.62	3	1	272	29.5	4.83	2.47	hypothetical protein [Arthrobacter sp. TB 26]
gi651489964	3.62	1	1	470	50.8	6.83	2.47	GntR family transcriptional regulator [Arthrobacter sp. H20]
gi760166848	8.76	1	1	137	14.0	4.67	2.47	hypothetical protein [Arthrobacter crystallopoietes]
gi517599682	7.74	1	1	155	17.1	6.52	2.47	hypothetical protein [Arthrobacter sp. 162MFSha1.1]
gi162953087	9.71	1	1	103	11.3	11.34	2.47	hypothetical protein RSal33209_0858 [Renibacterium salmoninarum ATCC 33209]

gi937262072	17.39	2	1	92	10.1	4.51	2.47	antibiotic biosynthesis monooxygenase [Arthrobacter sp. Edens01]
gi162954238	3.84	1	1	469	50.8	8.88	2.47	transcriptional regulator [Renibacterium salmoninarum ATCC 33209]
gi674646780	2.39	1	1	502	53.5	5.38	2.47	Long-chain-fatty-acid--CoA ligase FadD13 [Arthrobacter sp. 11W110_air]
gi323471169	4.48	1	1	335	34.6	6.95	2.47	transcriptional regulator [Arthrobacter phenanthrenivorans Sphe3]
gi517607411	3.23	3	1	558	61.1	10.17	2.47	polyprenyl glycosylphosphotransferase [Arthrobacter sp. 161MFSha2.1]
gi753938961	4.21	1	1	523	57.1	4.78	2.47	benzene 1,2-dioxygenase [Arthrobacter phenanthrenivorans]
gi910737900	16.67	1	1	96	10.1	9.04	2.47	hypothetical protein AHiyo4_07220 [Arthrobacter sp. Hiyo4]
gi769942848	2.26	1	1	443	47.4	9.77	2.47	hypothetical protein [Arthrobacter sp. IHBB 11108]
gi648574473	6.27	6	1	335	34.3	7.64	2.47	LacI family transcriptional regulator [Arthrobacter sp. 162MFSha1.1]
gi651500322	1.95	1	1	974	107.4	5.47	2.46	hypothetical protein [Arthrobacter sp. 35W]
gi908698060	2.36	1	1	509	55.3	6.04	2.46	ABC transporter ATP-binding protein [Arthrobacter sp. RIT-PI-e]
gi219861855	6.29	2	1	159	17.2	5.03	2.46	Clp domain protein (plasmid) [Arthrobacter chlorophenolicus A6]
gi918268020	6.11	3	1	311	34.1	6.90	2.46	lipid II:glycine glycyltransferase [Arthrobacter sp. Hiyo1]
gi910744114	4.39	3	1	433	45.3	10.83	2.46	uncharacterized ABC transporter permease protein YufP [Arthrobacter sp. Hiyo8]
gi162954222	1.69	1	1	888	99.3	6.27	2.46	asparagine synthetase (glutamine-hydrolyzing) [Renibacterium salmoninarum ATCC 33209]
gi476400777	20.99	1	1	81	9.0	6.24	2.46	hypothetical protein D477_012106, partial [Arthrobacter crystallopoietes BAB-32]
gi443481855	11.54	1	1	182	19.1	4.92	2.46	hypothetical protein G205_09528 [Arthrobacter nitrophenolicus]
gi786032965	5.57	1	1	323	33.6	6.13	2.46	nucleoside-diphosphate sugar epimerase [Arthrobacter chlorophenolicus]
gi723608631	3.98	1	1	477	53.0	5.72	2.46	FAD-dependent oxidoreductase [Arthrobacter sp. PAMC25486]
gi823665669	3.78	3	1	635	65.1	4.70	2.46	hypothetical protein AA310_06825 [Arthrobacter sp. YC-RL1]
gi640193340	2.62	1	1	496	51.4	9.61	2.46	hypothetical protein [Arthrobacter sp. 31Y]
gi551255051	7.79	1	1	308	33.8	6.61	2.46	LysR family transcriptional regulator [Arthrobacter sp. PAO19]
gi651480526	3.92	1	1	332	35.3	5.34	2.46	mycothiol acetyltransferase [Arthrobacter sp. Br18]
gi307745075	7.80	1	1	205	22.1	6.35	2.46	two-component system response regulator [Arthrobacter arilaitensis Re117]
gi635350945	3.00	3	1	433	47.2	9.74	2.46	sugar (and other) transporter family protein [Arthrobacter siccitolerans]
gi651500738	6.96	1	1	316	34.7	10.08	2.46	hypothetical protein [Arthrobacter sp. 35W]
gi930825847	3.71	1	1	431	47.0	7.15	2.46	hypothetical protein AOZ07_05775 [Arthrobacter arilaitensis]
gi219858309	8.63	1	1	255	27.9	5.03	2.46	ANTAR domain protein with unknown sensor [Arthrobacter chlorophenolicus A6]
gi116612343	5.13	1	1	409	40.9	5.03	2.46	Glycerate kinase [Arthrobacter sp. FB24]
gi359307532	1.11	1	1	1354	149.4	6.58	2.46	hypothetical protein ARGLB_012_00150 [Arthrobacter globiformis NBRC 12137]
gi674647000	5.24	1	1	248	24.5	5.66	2.46	Molybdenum cofactor guanylyltransferase [Arthrobacter sp. 11W110_air]
gi307744003	20.43	2	1	93	10.3	5.14	2.46	hypothetical membrane protein [Arthrobacter arilaitensis Re117]
gi517592084	9.47	1	1	243	25.5	5.82	2.45	hypothetical protein [Arthrobacter sp. 135MFCol5.1]
gi307744253	4.38	1	1	388	41.3	9.55	2.45	putative transcriptional regulator [Arthrobacter arilaitensis Re117]
gi403231119	8.09	1	1	136	14.4	6.11	2.45	putative molybdenum-pterin binding domain protein [Arthrobacter sp. Rue61a]
gi939037398	3.64	1	1	275	30.3	5.10	2.45	ATP synthase F0F1 subunit gamma, partial [Arthrobacter nitroguajacolicus]
gi937258457	2.86	1	1	384	40.6	5.07	2.45	cystathionine gamma-synthase [Arthrobacter sp. Edens01]
gi919107986	4.63	1	1	281	28.6	9.70	2.45	hypothetical protein [Arthrobacter sp. IHBB 11108]
gi742860231	2.68	1	1	448	50.1	5.29	2.45	glutamine synthetase [Arthrobacter sp. W1]
gi927293731	2.03	1	1	592	61.0	5.19	2.45	phosphomannomutase [Arthrobacter sp. ERGS1:01]
gi307745402	2.77	1	1	470	51.4	4.84	2.45	putative exoribonuclease II [Arthrobacter arilaitensis Re117]
gi651439203	5.24	1	1	210	22.0	10.11	2.45	hypothetical protein [Arthrobacter sp. H14]
gi517593488	2.17	1	1	460	47.9	5.36	2.45	hypothetical protein [Arthrobacter sp. 135MFCol5.1]
gi757624213	5.00	2	1	440	45.7	10.51	2.45	hypothetical protein TV39_12340 [Arthrobacter sp. SPG23]
gi517599191	11.72	1	1	128	14.1	10.11	2.45	hypothetical protein [Arthrobacter sp. 162MFSha1.1]
gi759735117	16.42	1	1	134	14.1	7.20	2.45	MerR family transcriptional regulator [Arthrobacter sp. L77]
gi640199077	7.53	1	1	279	30.8	6.23	2.45	transcriptional regulator [Arthrobacter sp. 31Y]
gi653567661	7.26	2	1	317	33.9	6.18	2.45	prephenate dehydratase [Propionibacterium thoenii]
gi723608107	12.16	1	1	148	15.0	4.67	2.45	hypothetical protein ART_1884 [Arthrobacter sp. PAMC25486]
gi323471118	4.95	1	1	283	30.0	6.07	2.45	hypothetical protein Asphe3_37050 [Arthrobacter phenanthrenivorans Sphe3]
gi307744368	4.78	1	1	209	22.2	4.84	2.44	hypothetical membrane protein [Arthrobacter arilaitensis Re117]
gi517600948	1.15	1	1	872	95.2	5.19	2.44	aminopeptidase N [Arthrobacter sp. 162MFSha1.1]
gi823666144	5.99	2	1	367	39.3	4.75	2.44	transaldolase [Arthrobacter sp. YC-RL1]
gi403228422	6.77	1	1	251	25.9	9.83	2.44	putative CAAX amino terminal protease family protein [Arthrobacter sp. Rue61a]
gi542110302	6.28	3	1	239	27.0	9.39	2.44	ADP-ribose pyrophosphatase [Arthrobacter sp. AK-YN10]
gi749402615	6.50	1	1	200	21.7	5.15	2.44	RNA polymerase sigma factor SigK [Arthrobacter sp. AK-YN10]
gi219862222	2.91	1	1	344	36.8	6.55	2.44	transcriptional regulator, LacI family (plasmid) [Arthrobacter chlorophenolicus A6]
gi517605002	13.04	1	1	161	17.0	11.36	2.44	hypothetical protein [Arthrobacter sp. 131MFCol6.1]
gi910740250	5.84	2	1	308	33.3	9.66	2.44	uncharacterized protein Rv2917/MT2985 [Arthrobacter sp. Hiyo4]

gi359306951	9.03	2	1	155	17.5	9.01	2.44	hypothetical protein ARGLB_028_00270 [Arthrobacter globiformis NBRC 12137]
gi654813930	4.42	1	1	317	34.3	5.22	2.44	MBL fold metallo-hydrolase [Arthrobacter sp. MA-N2]
gi119951902	6.09	1	1	197	22.0	8.21	2.44	putative transcription regulator (plasmid) [Arthrobacter aurescens TC1]
gi443481716	2.13	1	1	988	107.1	5.22	2.44	hypothetical protein G205_10233 [Arthrobacter nitrophenolicus]
gi937258467	3.54	1	1	480	52.3	5.24	2.44	cysteine--tRNA ligase [Arthrobacter sp. Edens01]
gi654826438	5.10	4	1	255	27.0	4.87	2.44	succinate dehydrogenase [Arthrobacter sp. H5]
gi359306306	3.95	2	1	430	44.5	5.29	2.44	hypothetical protein ARGLB_039_00160 [Arthrobacter globiformis NBRC 12137]
gi162955636	4.87	2	1	226	24.9	7.52	2.44	transcriptional regulator, TetR family [Renibacterium salmoninarum ATCC 33209]
gi636846999	5.49	1	1	364	40.6	5.24	2.44	cupin, partial [Arthrobacter sp. TB 26]
gi910745066	5.47	2	1	201	22.1	8.57	2.44	VWA containing CoxE family protein [Arthrobacter sp. Hiyo8]
gi742069642	7.33	1	1	150	16.1	6.80	2.44	polyketide cyclase/dehydrase [Arthrobacter sp. MWB30]
gi927293778	1.99	1	1	502	54.6	5.17	2.44	glutamyl-tRNA amidotransferase [Arthrobacter sp. ERGS1:01]
gi765011549	2.88	1	1	832	85.6	5.24	2.44	RND transporter [Arthrobacter sp. A3]
gi908699570	3.65	1	1	658	68.2	5.33	2.43	PTS mannose transporter subunit IIA [Arthrobacter sp. RIT-PI-e]
gi517608723	5.70	1	1	263	27.6	5.14	2.43	D-beta-D-heptose 1-phosphate adenosyltransferase [Arthrobacter sp. 161MFSha2.1]
gi648574468	11.17	1	1	206	22.2	4.97	2.43	hypothetical protein [Arthrobacter sp. 162MFSha1.1]
gi651433543	5.19	1	1	270	28.6	9.29	2.43	ABC transporter [Arthrobacter sp. H41]
gi651445215	5.20	1	1	500	52.9	10.01	2.43	PA-phosphatase [Arthrobacter nicotinovorans]
gi636845738	4.91	1	1	163	18.0	5.76	2.43	hypothetical protein [Arthrobacter sp. TB 26]
gi765012202	3.33	1	1	570	62.5	6.89	2.43	ABC transporter [Arthrobacter sp. A3]
gi928486206	4.64	1	1	280	30.9	9.09	2.43	hypothetical protein AOC05_02990 [Arthrobacter alpinus]
gi359307692	4.29	1	1	396	41.6	7.11	2.43	putative transcriptional regulator [Arthrobacter globiformis NBRC 12137]
gi476401076	6.27	3	1	415	46.1	5.60	2.43	plasmid pRiA4b ORF-3 family protein [Arthrobacter crystallopoietes BAB-32]
gi917442270	3.34	1	1	509	54.5	4.73	2.43	hypothetical protein [Arthrobacter albus]
gi518312957	9.89	3	1	263	27.5	10.10	2.43	hypothetical protein [Arthrobacter sp. TB 23]
gi916692410	6.41	1	1	312	34.1	9.47	2.43	hypothetical protein [Arthrobacter castelli]
gi652423904	4.37	2	1	229	25.2	6.55	2.43	hypothetical protein [Arthrobacter castelli]
gi639130259	2.38	1	1	589	66.7	5.19	2.43	hypothetical protein [Arthrobacter sp. CAL618]
gi654813349	6.94	3	1	216	22.2	8.48	2.43	DNA-3-methyladenine glycosidase [Arthrobacter sp. MA-N2]
gi470220271	1.18	1	1	850	96.3	5.96	2.43	type II restriction endonuclease [Arthrobacter gangotriensis Lz1y]
gi323470397	3.52	1	1	426	48.0	8.31	2.43	glycosyl transferase [Arthrobacter phenanthrenivorans Sphe3]
gi635352555	4.32	1	1	301	32.4	10.29	2.43	bacterial type II secretion system F domain protein [Arthrobacter siccitolerans]
gi119949235	5.06	1	1	435	46.9	4.72	2.42	Aminotransferase classes I and II protein [Arthrobacter aurescens TC1]
gi470217276	1.43	2	1	561	60.0	5.69	2.42	oligopeptide ABC transporter, ATP-binding protein [Arthrobacter gangotriensis Lz1y]
gi515765557	1.98	1	1	555	60.8	4.98	2.42	hypothetical protein [Arthrobacter sp. M2012083]
gi648575298	3.13	1	1	512	56.4	4.94	2.42	diguanylate phosphodiesterase [Arthrobacter sp. 161MFSha2.1]
gi910748047	5.08	1	1	197	20.7	5.96	2.42	cysteine desulfurase [Arthrobacter sp. Hiyo8]
gi640193382	3.67	1	1	354	38.0	5.86	2.42	SAM-dependent methyltransferase [Arthrobacter sp. 31Y]
gi742852077	18.18	1	1	88	9.7	9.74	2.42	hypothetical protein [Arthrobacter sp. W1]
gi651487005	4.95	3	1	222	23.2	5.22	2.42	hypothetical protein [Arthrobacter sp. Br18]
gi910251600	3.69	1	1	461	46.9	5.24	2.42	amidase [Arthrobacter siccitolerans]
gi765008487	10.95	2	1	137	14.9	4.32	2.42	hypothetical protein [Arthrobacter sp. A3]
gi753940484	4.76	1	1	294	32.3	4.83	2.42	catechol 1,2-dioxygenase [Arthrobacter phenanthrenivorans]
gi910250144	3.42	2	1	322	35.5	8.76	2.42	formamidopyrimidine-DNA glycosylase [Arthrobacter siccitolerans]
gi723609599	2.08	1	1	722	81.6	5.06	2.42	hypothetical protein ART_3376 [Arthrobacter sp. PAMC25486]
gi651434522	3.43	3	1	379	40.6	5.01	2.42	homoserine acetyltransferase [Arthrobacter sp. H41]
gi470220316	4.13	1	1	315	34.5	5.27	2.42	riboflavin biosynthesis protein RibF [Arthrobacter gangotriensis Lz1y]
gi917739819	5.16	1	1	368	40.4	5.55	2.42	hypothetical protein [Arthrobacter sp. W1]
gi723607973	6.53	1	1	352	36.6	5.31	2.42	3-hydroxyisobutyryl-CoA hydrolase [Arthrobacter sp. PAMC25486]
gi359305572	1.90	1	1	735	74.8	5.86	2.42	subtilisin family peptidase [Arthrobacter globiformis NBRC 12137]
gi742857421	10.81	1	1	148	16.0	6.18	2.42	hypothetical protein [Arthrobacter sp. W1]
gi737777698	8.50	1	1	153	15.6	5.55	2.42	hypothetical protein [Arthrobacter sanguinis]
gi937259144	3.64	1	1	494	50.7	5.00	2.42	UDP-N-acetylmuramoyl-tripeptide--D-alanyl-D-alanine ligase [Arthrobacter sp. Edens01]
gi651502720	5.56	1	1	216	22.3	8.68	2.42	hypothetical protein [Arthrobacter sp. 35W]
gi551254359	2.98	2	1	504	53.5	5.85	2.42	methylmalonate-semialdehyde dehydrogenase [Arthrobacter sp. PAO19]
gi651494209	7.14	2	1	336	36.2	5.60	2.42	16S rRNA (cytosine(1402)-N(4))-methyltransferase [Arthrobacter sp. H20]
gi928487361	7.03	1	1	327	35.1	5.92	2.42	AAA family ATPase [Arthrobacter alpinus]
gi651470012	2.74	1	1	583	63.2	9.52	2.42	hypothetical protein [Arthrobacter nicotinovorans]
gi654815674	8.14	3	1	221	23.5	6.00	2.42	mechanosensitive ion channel protein MscS [Arthrobacter sp. UNC362MFTsu5.1]

gi910250531	13.64	1	1	132	13.1	5.87	2.42	hypothetical protein [Arthrobacter siccitolerans]
gi908642132	5.91	5	1	186	20.2	5.87	2.42	RNA polymerase subunit sigma-70 [Arthrobacter phenanthrenivorans]
gi652422493	3.62	1	1	497	56.4	5.72	2.42	catalase [Arthrobacter castelli]
gi518312053	5.41	1	1	314	33.5	5.12	2.42	hypothetical protein [Arthrobacter sp. TB 23]
gi219857981	5.65	1	1	301	32.1	5.14	2.42	pantoate/beta-alanine ligase [Arthrobacter chlorophenolicus A6]
gi914714718	2.93	1	1	307	32.4	5.52	2.42	LysR family transcriptional regulator [Arthrobacter sp. ZBG10]
gi742756300	3.42	3	1	497	53.5	7.30	2.42	GTP-binding protein [Arthrobacter phenanthrenivorans]
gi930827125	10.59	1	1	170	19.0	9.13	2.41	MarR family transcriptional regulator [Arthrobacter arilaitensis]
gi307745750	6.25	5	1	304	34.0	6.43	2.41	NUDIX hydrolase [Arthrobacter arilaitensis Re117]
gi517598759	3.40	3	1	265	27.9	6.46	2.41	cytochrome C [Arthrobacter sp. 162MFSha1.1]
gi651437844	5.50	2	1	309	33.1	6.24	2.41	LysR family transcriptional regulator [Arthrobacter sp. H14]
gi765006965	4.56	1	1	263	27.5	4.89	2.41	oxidoreductase [Arthrobacter sp. A3]
gi640202929	5.36	2	1	336	37.2	5.16	2.41	GCN5 family acetyltransferase [Arthrobacter sp. 31Y]
gi937262564	8.29	2	1	217	23.1	6.11	2.41	ABC transporter ATP-binding protein [Arthrobacter sp. Edens01]
gi723609043	5.07	1	1	473	50.3	4.84	2.41	ABC-type sugar transport system, periplasmic component [Arthrobacter sp. PAMC25486]
gi767256716	6.49	1	1	308	32.0	4.79	2.41	hypothetical protein UM93_01915 [Arthrobacter sp. IHBB 11108]
gi927292910	6.79	2	1	280	30.6	4.94	2.41	hypothetical protein AL755_01300 (plasmid) [Arthrobacter sp. ERGS1:01]
gi757625431	2.68	1	1	560	60.9	5.44	2.41	ATPase [Arthrobacter sp. SPG23]
gi651439656	13.00	1	1	100	11.1	5.06	2.41	MerR family transcriptional regulator, partial [Arthrobacter sp. H14]
gi916863454	4.33	2	1	577	62.6	6.49	2.41	ABC transporter ATP-binding protein [Arthrobacter sp. 35/47]
gi937257921	4.28	1	1	514	54.8	5.02	2.41	heme ABC transporter ATP-binding protein [Arthrobacter sp. Edens01]
gi759736729	11.17	2	1	206	22.0	7.53	2.41	hypothetical protein [Arthrobacter sp. L77]
gi654826824	5.36	1	1	280	30.0	9.85	2.41	hypothetical protein [Arthrobacter sp. H5]
gi651493316	8.17	1	1	306	32.4	10.30	2.41	membrane protein [Arthrobacter sp. H20]
gi489897827	6.19	1	1	291	31.1	7.58	2.41	transcriptional regulator [Arthrobacter globiformis]
gi823665380	8.94	1	1	235	25.6	9.61	2.41	hypothetical protein AA310_18455 [Arthrobacter sp. YC-RL1]
gi919134924	3.84	2	1	365	39.2	5.01	2.41	hypothetical protein [Arthrobacter chlorophenolicus]
gi189046075	10.88	1	1	193	20.0	9.98	2.41	RecName: Full=Crossover junction endodeoxyribonuclease RuvC; AltName: Full=Holliday junction
gi930825055	7.46	1	1	134	14.3	8.00	2.41	hypothetical protein AOZ07_01245 [Arthrobacter arilaitensis]
gi927292840	6.31	1	1	333	34.7	5.05	2.41	dihydroxyacetone kinase (plasmid) [Arthrobacter sp. ERGS1:01]
gi742851351	17.53	1	1	97	10.6	9.92	2.41	XRE family transcriptional regulator [Arthrobacter sp. W1]
gi916834769	6.73	2	1	223	24.6	9.63	2.41	hypothetical protein [Arthrobacter sp. H14]
gi307745753	2.76	2	1	760	82.3	6.21	2.41	penicillin-binding protein [Arthrobacter arilaitensis Re117]
gi939050914	4.85	1	1	371	40.4	5.03	2.41	succinyldiaminopimelate transaminase [Arthrobacter sp. JCM 19049]
gi654816123	6.55	1	1	275	29.3	5.82	2.40	DNA-binding protein [Arthrobacter sp. UNC362MFTsu5.1]
gi737793046	2.35	1	1	511	51.2	9.41	2.40	MFS transporter [Arthrobacter nicotinovorans]
gi914716031	4.01	1	1	299	30.8	5.99	2.40	transglycosylase, partial [Arthrobacter sp. ZBG10]
gi753938958	13.00	1	1	100	10.5	10.59	2.40	XRE family transcriptional regulator [Arthrobacter phenanthrenivorans]
gi648573062	10.89	1	1	248	25.3	5.38	2.40	hypothetical protein [Arthrobacter sp. 135MFCol5.1]
gi119947912	4.53	2	1	265	28.0	6.80	2.40	putative SIS (Sugar ISomerase) domain protein [Arthrobacter aurescens TC1]
gi674646828	2.93	1	1	409	44.8	8.70	2.40	5-methylcytosine-specific restriction enzyme subunit McrC [Arthrobacter sp. 11W110_air]
gi651504516	9.09	1	1	253	26.2	4.93	2.40	hypothetical protein [Arthrobacter sp. 35W]
gi517598318	2.78	1	1	360	38.7	9.52	2.40	peptide ABC transporter permease [Arthrobacter sp. 162MFSha1.1]
gi742069928	4.94	2	1	324	35.3	6.70	2.40	HTH-type transcriptional regulator GltC [Arthrobacter sp. MWB30]
gi910737241	12.74	1	1	157	17.6	6.95	2.40	uncharacterized protein YdfG [Arthrobacter sp. Hiyo4]
gi517590278	5.33	1	1	244	26.9	5.17	2.40	flavodoxin [Arthrobacter sp. 135MFCol5.1]
gi749402673	3.77	1	1	265	29.2	9.38	2.40	hypothetical protein M707_21605 [Arthrobacter sp. AK-YN10]
gi723609883	6.73	2	1	208	21.2	4.72	2.40	hypothetical protein ART_3660 [Arthrobacter sp. PAMC25486]
gi723607229	10.34	1	1	232	24.6	6.29	2.40	hypothetical protein ART_1006 [Arthrobacter sp. PAMC25486]
gi786034384	6.20	1	1	371	39.5	4.88	2.40	agmatine deiminase [Arthrobacter chlorophenolicus]
gi910744487	9.40	1	1	149	15.7	9.98	2.40	hypothetical protein AHiyo8_21890 [Arthrobacter sp. Hiyo8]
gi651440237	1.20	1	1	1080	116.7	5.60	2.40	DNA helicase UvrD [Arthrobacter sp. H14]
gi737813241	9.58	1	1	261	26.8	5.30	2.40	NAD(P) transhydrogenase subunit alpha, partial [Arthrobacter sp. H14]
gi651497443	6.44	1	1	264	28.8	9.89	2.40	hypothetical protein [Arthrobacter sp. 35W]
gi823666178	15.33	1	1	150	15.8	5.96	2.40	hypothetical protein AA310_09940 [Arthrobacter sp. YC-RL1]
gi551256435	2.59	1	1	424	47.8	7.24	2.40	hypothetical protein [Arthrobacter sp. PAO19]
gi652422867	3.53	2	1	283	30.6	5.07	2.40	tRNA (guanine-N1)-methyltransferase [Arthrobacter castelli]
gi651503233	4.69	1	1	213	22.0	7.40	2.40	hypothetical protein [Arthrobacter sp. 35W]
gi908690430	5.74	2	1	209	21.9	5.76	2.40	phosphoheptose isomerase [Arthrobacter sp. H41]

gi517602929	3.47	1	1	288	29.2	5.34	2.40	orotidine 5'-phosphate decarboxylase [Arthrobacter sp. 131MFCol6.1]
gi517605431	9.52	1	1	210	21.7	7.58	2.39	hypothetical protein [Arthrobacter sp. 131MFCol6.1]
gi116609389	3.44	1	1	524	55.2	5.24	2.39	tripeptidyl-peptidase B, Serine peptidase, MEROPS family S33 [Arthrobacter sp. FB24]
gi518311005	4.22	1	1	332	35.5	6.18	2.39	hypothetical protein [Arthrobacter sp. TB 23]
gi914716126	5.28	1	1	265	28.0	9.91	2.39	CDP-alcohol phosphatidyltransferase [Arthrobacter sp. ZBG10]
gi928988188	10.00	2	1	170	17.2	10.20	2.39	hypothetical protein [Arthrobacter sp. ERGS1:01]
gi517603893	11.86	1	1	177	17.4	7.06	2.39	MULTISPECIES: hypothetical protein [Arthrobacter]
gi759732465	3.13	1	1	512	52.1	10.52	2.39	lipid II flippase MurJ [Arthrobacter sp. L77]
gi517593116	6.67	1	1	300	33.4	7.12	2.39	XRE family transcriptional regulator [Arthrobacter sp. 135MFCol5.1]
gi928487448	14.85	1	1	101	10.5	10.26	2.39	hypothetical protein AOC05_11565 [Arthrobacter alpinus]
gi542107726	5.28	3	1	398	42.7	10.14	2.39	pseudouridine synthase [Arthrobacter sp. AK-YN10]
gi927293490	6.76	2	1	148	16.3	4.94	2.39	hypothetical protein AL755_04640 [Arthrobacter sp. ERGS1:01]
gi737811675	5.37	1	1	298	32.7	5.58	2.39	ABC transporter [Arthrobacter sp. 35/47]
gi219861550	7.19	1	1	153	16.6	7.40	2.39	GatB/YqeY domain protein (plasmid) [Arthrobacter chlorophenolicus A6]
gi651498748	6.30	1	1	349	36.6	9.60	2.39	translation initiation factor IF-3 [Arthrobacter sp. 35W]
gi636845922	4.75	3	1	442	45.8	9.50	2.39	MFS transporter [Arthrobacter sp. TB 26]
gi765005441	13.11	1	1	183	19.6	4.73	2.39	ATP synthase F0F1 subunit B [Arthrobacter sp. A3]
gi443481935	5.65	1	1	354	37.6	5.12	2.39	Monoamine oxidase [Arthrobacter nitrophenolicus]
gi651435774	3.05	2	1	393	43.4	5.02	2.39	pyruvate dehydrogenase [Arthrobacter sp. H41]
gi910251751	4.04	1	1	396	41.6	5.53	2.39	cystathionine gamma-synthase [Arthrobacter siccitolerans]
gi759747291	2.11	1	1	617	66.5	5.94	2.39	NADH oxidase [Arthrobacter sp. 31Y]
gi910744403	2.77	1	1	541	61.6	5.26	2.39	5-methylcytosine-specific restriction enzyme B [Arthrobacter sp. Hiyo8]
gi823666275	4.82	1	1	249	27.6	11.18	2.39	membrane protein [Arthrobacter sp. YC-RL1]
gi767257817	25.71	1	1	70	7.6	10.21	2.39	50S ribosomal protein L30 [Arthrobacter sp. IHBB 11108]
gi910695043	8.56	1	1	222	23.1	4.86	2.39	3-oxoacyl-[acyl-carrier-protein] synthase 3, partial [Arthrobacter sp. Hiyo6]
gi737788100	3.68	1	1	353	38.8	10.07	2.39	hypothetical protein [Arthrobacter albus]
gi651472532	2.46	1	1	407	41.7	10.35	2.39	transporter [Arthrobacter nicotinovorans]
gi917013363	10.13	4	1	158	17.4	6.55	2.39	ribonucleoside-diphosphate reductase [Arthrobacter sanguinis]
gi517606855	4.95	2	1	202	22.4	7.94	2.39	hypothetical protein [Arthrobacter sp. 161MFSha2.1]
gi323468562	4.69	1	1	384	40.8	4.72	2.39	monosaccharide ABC transporter substrate-binding protein, CUT2 family [Arthrobacter phenanthre
gi551255148	5.45	1	1	165	18.9	5.87	2.39	RNA polymerase sigma24 factor [Arthrobacter sp. PAO19]
gi742071081	6.87	2	1	335	36.0	6.37	2.39	heat-inducible transcription repressor HrcA [Arthrobacter sp. MWB30]
gi914713337	2.54	2	1	552	59.4	9.31	2.39	hypothetical protein [Arthrobacter sp. ZBG10]
gi742070269	7.75	1	1	129	14.2	5.69	2.39	chorismate mutase [Arthrobacter sp. MWB30]
gi517602672	3.08	1	1	584	65.6	5.16	2.39	X-Pro dipeptidyl-peptidase [Arthrobacter sp. 131MFCol6.1]
gi636845262	5.76	1	1	191	20.0	11.25	2.39	hypothetical protein [Arthrobacter sp. TB 26]
gi742860154	2.09	1	1	621	69.3	4.89	2.39	hypothetical protein [Arthrobacter sp. W1]
gi515766014	17.70	1	1	113	12.4	4.92	2.39	DivIVA domain-containing protein [Arthrobacter sp. M2012083]
gi651499732	11.56	3	1	225	23.3	5.62	2.39	BON domain-containing protein [Arthrobacter sp. 35W]
gi654819123	2.12	1	1	567	61.2	6.58	2.39	hypothetical protein [Arthrobacter sp. UNC362MFTsu5.1]
gi359303659	23.75	2	1	80	8.9	8.84	2.38	hypothetical protein ARGLB_111_00350 [Arthrobacter globiformis NBRC 12137]
gi403229865	2.33	3	1	386	42.6	7.72	2.38	PhoH-like protein [Arthrobacter sp. Rue61a]
gi930827149	3.82	1	1	340	36.6	8.28	2.38	hypothetical protein AOZ07_13260 [Arthrobacter arilaitensis]
gi403229894	5.02	1	1	418	42.9	5.68	2.38	coenzyme A biosynthesis bifunctional protein CoaBC [Arthrobacter sp. Rue61a]
gi651455161	6.52	1	1	184	19.1	5.03	2.38	hypothetical protein [Arthrobacter nicotinovorans]
gi651438145	4.55	1	1	374	40.1	8.44	2.38	cytochrome C biogenesis protein [Arthrobacter sp. H14]
gi162955460	4.14	1	1	266	27.9	5.48	2.38	short chain dehydrogenase [Renibacterium salmoninarum ATCC 33209]
gi648574560	4.85	2	1	206	22.9	6.00	2.38	translation factor Sua5 [Arthrobacter sp. 131MFCol6.1]
gi916869793	3.73	1	1	429	44.6	5.52	2.38	alpha/beta hydrolase [Arthrobacter sp. Br18]
gi476398891	9.30	1	1	258	27.2	4.88	2.38	sodium symporter [Arthrobacter crystallopoietes BAB-32]
gi906448149	4.24	2	1	566	63.2	5.01	2.38	alpha-amylase [Arthrobacter sp. RIT-PI-e]
gi765012754	5.61	1	1	303	33.8	4.98	2.38	2-keto-myo-inositol dehydratase [Arthrobacter sp. A3]
gi914713776	22.86	1	1	105	11.2	5.01	2.38	hypothetical protein [Arthrobacter sp. ZBG10]
gi443482687	5.84	1	1	377	40.4	6.67	2.38	putative sarcosine oxidase [Arthrobacter nitrophenolicus]
gi742856443	2.21	1	1	407	43.9	7.28	2.38	lipase/esterase [Arthrobacter sp. W1]
gi917739753	4.40	1	1	341	38.1	8.82	2.38	hypothetical protein [Arthrobacter sp. W1]
gi723608233	7.51	2	1	173	19.0	4.98	2.38	hypothetical protein ART_2010 [Arthrobacter sp. PAMC25486]
gi476401234	3.29	3	1	578	61.4	5.38	2.38	acetyl/propionyl-CoA carboxylase subunit alpha [Arthrobacter crystallopoietes BAB-32]
gi930828218	4.12	2	1	461	50.5	6.44	2.38	FAD-dependent oxidoreductase [Arthrobacter arilaitensis]

gi651462182	1.37	1	1	874	92.6	8.60	2.38	multicopper oxidase [Arthrobacter sp. 35/47]
gi910694840	10.69	1	1	131	14.1	5.49	2.38	acetate CoA-transferase YdiF [Arthrobacter sp. Hiyo6]
gi760111830	27.63	1	1	76	8.2	5.06	2.38	XRE family transcriptional regulator [Arthrobacter chlorophenolicus]
gi470217208	6.08	1	1	411	45.4	5.54	2.38	RNA polymerase sigma factor RpoD [Arthrobacter gangotriensis Lz1y]
gi939050116	6.41	3	1	312	34.7	9.73	2.38	hypothetical protein [Arthrobacter sp. JCM 19049]
gi652425392	2.07	2	1	434	46.5	4.81	2.38	UDP-glucose 6-dehydrogenase [Arthrobacter castelli]
gi470215916	5.98	2	1	351	38.5	5.49	2.38	cupin [Arthrobacter gangotriensis Lz1y]
gi635350591	6.57	1	1	274	29.9	4.94	2.37	aldo/keto reductase family protein [Arthrobacter siccitolerans]
gi908698356	2.50	1	1	601	67.4	5.41	2.37	hypothetical protein [Arthrobacter sp. RIT-PI-e]
gi476399874	8.43	1	1	166	18.6	6.90	2.37	HNH endonuclease, partial [Arthrobacter crystallopoietes BAB-32]
gi914715474	5.58	3	1	251	27.9	7.25	2.37	glycosyl transferase [Arthrobacter sp. ZBG10]
gi767256508	8.57	1	1	210	23.6	5.25	2.37	nucleotide pyrophosphohydrolase [Arthrobacter sp. IHBB 11108]
gi658509417	5.85	1	1	205	22.5	11.03	2.37	hypothetical protein [Arthrobacter sp. TB 26]
gi636844197	7.69	1	1	182	19.7	5.57	2.37	hypothetical protein [Arthrobacter sp. TB 26]
gi723608621	5.80	1	1	379	40.5	6.20	2.37	hypothetical protein ART_2398 [Arthrobacter sp. PAMC25486]
gi654817446	27.71	2	1	83	8.8	10.71	2.37	hypothetical protein [Arthrobacter sp. UNC362MFTsu5.1]
gi654816622	9.00	1	1	211	23.3	6.83	2.37	ArsR family transcriptional regulator [Arthrobacter sp. UNC362MFTsu5.1]
gi651439029	2.80	1	1	429	43.8	9.99	2.37	MFS transporter [Arthrobacter sp. H14]
gi765004211	6.78	1	1	339	36.2	5.47	2.37	2-hydroxyacid dehydrogenase [Arthrobacter sp. A3]
gi723607523	2.08	1	1	960	98.6	4.96	2.37	hypothetical protein ART_1300 [Arthrobacter sp. PAMC25486]
gi517600636	4.26	1	1	493	51.9	9.92	2.37	GntR family transcriptional regulator [Arthrobacter sp. 162MFSha1.1]
gi742755558	9.80	1	1	204	22.3	4.32	2.37	ribosome maturation factor RimM [Arthrobacter phenanthrenivorans]
gi651443765	4.37	1	1	389	39.5	5.00	2.37	hypothetical protein [Arthrobacter sp. 9MFCol3.1]
gi307746599	3.30	1	1	333	36.4	5.39	2.37	pirin-like protein [Arthrobacter arilaitensis Re117]
gi917760173	4.05	1	1	543	58.4	5.88	2.37	hypothetical protein [Arthrobacter sp. L77]
gi742861338	4.82	1	1	249	27.5	11.37	2.37	membrane protein [Arthrobacter sp. W1]
gi651480675	2.47	1	1	850	93.9	4.89	2.37	aminopeptidase N [Arthrobacter sp. Br18]
gi937259298	9.59	2	1	146	16.4	10.17	2.37	AraC family transcriptional regulator [Arthrobacter sp. Edens01]
gi651430137	4.25	1	1	259	27.9	4.51	2.37	xylose isomerase [Arthrobacter sanguinis]
gi651452908	2.21	1	1	497	54.1	9.35	2.37	amino acid permease [Arthrobacter nicotinovorans]
gi470221722	5.98	1	1	251	26.3	9.31	2.37	putative ABC transporter, permease protein [Arthrobacter gangotriensis Lz1y]
gi162954215	4.12	1	1	607	64.6	8.22	2.37	protein-export membrane protein [Renibacterium salmoninarum ATCC 33209]
gi517601567	2.28	1	1	438	47.6	6.18	2.37	hypothetical protein [Arthrobacter sp. 162MFSha1.1]
gi403230110	6.61	1	1	257	28.1	9.55	2.37	1-acylglycerol-3-phosphate O-acyltransferase, putative [Arthrobacter sp. Rue61a]
gi323468343	7.39	1	1	230	22.6	8.16	2.37	cell wall-associated hydrolase, invasion-associated protein [Arthrobacter phenanthrenivorans Sphe
gi359305704	6.04	1	1	265	28.2	5.36	2.37	putative IclR family transcriptional regulator [Arthrobacter globiformis NBRC 12137]
gi723609480	6.56	3	1	244	24.2	7.34	2.37	hypothetical protein ART_3257 [Arthrobacter sp. PAMC25486]
gi674644141	26.19	1	1	84	9.0	4.88	2.36	phosphoribosylformylglycinamidine synthase subunit PurS [Arthrobacter sp. 11W110_air]
gi162954092	4.06	2	1	419	45.1	5.24	2.36	tetracycline resistance protein [Renibacterium salmoninarum ATCC 33209]
gi823667420	5.34	1	1	262	28.2	5.14	2.36	enoyl-CoA hydratase [Arthrobacter sp. YC-RL1]
gi737788833	4.90	1	1	347	38.3	7.09	2.36	hypothetical protein [Arthrobacter albus]
gi937258480	6.43	1	1	140	15.7	9.54	2.36	hypothetical protein AO716_10585 [Arthrobacter sp. Edens01]
gi765004013	5.62	1	1	338	36.8	6.27	2.36	aldo/keto reductase [Arthrobacter sp. A3]
gi470221494	3.44	1	1	349	36.3	5.52	2.36	ABC transporter [Arthrobacter gangotriensis Lz1y]
gi927294807	16.47	1	1	85	9.1	5.01	2.36	hypothetical protein AL755_13635 [Arthrobacter sp. ERGS1:01]
gi517593656	6.30	3	1	365	39.7	5.71	2.36	hypothetical protein [Arthrobacter sp. 135MFCol5.1]
gi919218914	4.49	5	1	423	44.3	5.38	2.36	hypothetical protein [Arthrobacter sp. YC-RL1]
gi219860488	3.03	1	1	759	76.8	4.83	2.36	Fibronectin type III domain protein [Arthrobacter chlorophenolicus A6]
gi517602717	2.77	1	1	505	54.5	8.87	2.36	hypothetical protein [Arthrobacter sp. 131MFCol6.1]
gi927295786	3.23	1	1	495	53.4	5.66	2.36	carnitine dehydratase [Arthrobacter sp. ERGS1:01]
gi927032487	5.00	2	1	300	32.7	4.93	2.36	hypothetical protein AFL94_09405 [Arthrobacter sp. LS16]
gi910748620	7.97	1	1	138	15.2	9.36	2.36	cytochrome oxidase assembly protein shy1 [Arthrobacter sp. Hiyo8]
gi915933181	6.11	1	1	229	25.4	5.36	2.36	protein tyrosine phosphatase [Arthrobacter globiformis]
gi654824336	2.21	1	1	497	53.8	8.78	2.36	coenzyme F390 synthetase [Arthrobacter sp. I3]
gi910694444	7.89	1	1	228	25.3	5.95	2.36	galactonate operon transcriptional repressor [Arthrobacter sp. Hiyo6]
gi517593649	20.00	1	1	60	6.1	6.57	2.36	hypothetical protein [Arthrobacter sp. 135MFCol5.1]
gi640196838	4.16	1	1	361	38.1	5.34	2.36	hypothetical protein [Arthrobacter sp. 31Y]
gi916871632	2.43	3	1	535	57.2	6.02	2.36	hypothetical protein [Arthrobacter sp. H5]
gi651471600	3.24	1	1	463	49.2	5.43	2.36	AMP-dependent synthetase [Arthrobacter nicotinovorans]

gi753940479	10.20	1	1	245	25.5	5.02	2.36	alpha-dehydro-beta-deoxy-D-glucarate aldolase [Arthrobacter phenanthrenivorans]
gi116609576	3.65	2	1	301	34.0	8.69	2.36	hypothetical protein Arth_0903 [Arthrobacter sp. FB24]
gi654824157	10.33	1	1	242	24.5	5.20	2.36	hypothetical protein [Arthrobacter sp. I3]
gi910740524	8.76	2	1	194	20.4	10.10	2.36	porphobilinogen deaminase [Arthrobacter sp. Hiyo4]
gi518312059	5.23	1	1	325	33.5	5.99	2.36	hypothetical protein [Arthrobacter sp. TB 23]
gi517608096	2.59	1	1	464	46.8	9.33	2.36	MFS transporter [Arthrobacter sp. 161MFSha2.1]
gi651498418	7.92	4	1	303	31.9	9.77	2.36	membrane protein [Arthrobacter sp. 35W]
gi927032027	2.54	1	1	433	47.2	4.97	2.36	quinolinate synthetase [Arthrobacter sp. LS16]
gi823665612	2.54	1	1	433	47.1	4.97	2.36	quinolinate synthetase [Arthrobacter sp. YC-RL1]
gi759746455	9.61	3	1	229	24.0	8.21	2.36	short-chain dehydrogenase [Arthrobacter sp. 31Y]
gi162953756	2.40	1	1	458	49.1	5.44	2.36	peptidase, M20/M25/M40 family [Renibacterium salmoninarum ATCC 33209]
gi760112335	6.00	6	1	350	36.8	9.51	2.36	ribose ABC transporter permease [Arthrobacter chlorophenolicus]
gi757625770	1.14	1	1	875	96.1	5.71	2.36	glycogen phosphorylase [Arthrobacter sp. SPG23]
gi515764485	15.04	1	1	133	15.1	5.11	2.36	glyoxalase [Arthrobacter sp. M2012083]
gi757625177	7.83	1	1	230	24.9	7.31	2.35	GntR family transcriptional regulator [Arthrobacter sp. SPG23]
gi476398902	2.20	1	1	590	62.7	5.05	2.35	acetyl-CoA carboxylase biotin-containing subunit [Arthrobacter crystallopoietes BAB-32]
gi723608807	4.35	1	1	368	40.0	5.26	2.35	hypothetical protein ART_2584 [Arthrobacter sp. PAMC25486]
gi928488491	2.93	1	1	648	68.4	5.87	2.35	hypothetical protein AOC05_18400 [Arthrobacter alpinus]
gi116612025	3.70	2	1	568	62.0	7.66	2.35	DNA-directed DNA polymerase [Arthrobacter sp. FB24]
gi917013419	4.23	1	1	260	28.3	4.75	2.35	hypothetical protein [Arthrobacter sanguinis]
gi651500516	8.02	1	1	162	17.6	8.97	2.35	AsnC family transcriptional regulator [Arthrobacter sp. 35W]
gi119949569	2.80	3	1	428	44.9	9.51	2.35	putative transmembrane efflux protein (MFS) [Arthrobacter aurescens TC1]
gi651490375	10.10	1	1	208	21.5	7.43	2.35	ATP-dependent DNA helicase RuvA [Arthrobacter sp. H20]
gi651504791	7.20	1	1	236	25.1	9.00	2.35	hypothetical protein [Arthrobacter sp. 35W]
gi764161604	2.73	1	1	476	52.0	5.48	2.35	portal protein [Arthrobacter phage vB_ArtM-ArV1]
gi916781978	12.30	1	1	122	13.3	5.64	2.35	hypothetical protein [Arthrobacter sp. 35W]
gi651430205	1.29	1	1	774	87.7	5.22	2.35	hypothetical protein [Arthrobacter sanguinis]
gi759731649	5.35	6	1	430	47.1	4.79	2.35	aminotransferase [Arthrobacter sp. L77]
gi742754845	3.90	1	1	487	54.4	5.06	2.35	Fe-S cluster assembly protein SufB [Arthrobacter phenanthrenivorans]
gi323471265	6.55	1	1	168	18.5	7.59	2.35	transcriptional regulator [Arthrobacter phenanthrenivorans Sphe3]
gi930825172	1.90	2	1	525	57.4	7.78	2.35	sodium:proton antiporter [Arthrobacter arilaitensis]
gi759716461	3.09	1	1	259	26.9	9.52	2.35	hypothetical protein, partial [Arthrobacter sp. AK-YN10]
gi219861665	4.92	1	1	244	25.7	7.72	2.35	conserved hypothetical protein (plasmid) [Arthrobacter chlorophenolicus A6]
gi517604016	4.88	1	1	246	25.6	8.75	2.35	hypothetical protein [Arthrobacter sp. 131MFCol6.1]
gi927295421	7.20	1	1	250	26.1	7.96	2.35	methylenetetrahydrofolate reductase [Arthrobacter sp. ERGS1:01]
gi918469549	4.85	1	1	268	29.6	6.62	2.35	Zn-dependent hydrolase [Arthrobacter crystallopoietes]
gi765006621	4.07	1	1	221	24.6	6.40	2.35	GCN5 family acetyltransferase [Arthrobacter sp. A3]
gi916820479	7.88	1	1	241	27.0	6.10	2.35	RNA polymerase [Arthrobacter sp. H20]
gi517609907	6.64	1	1	241	25.7	4.98	2.35	hypothetical protein [Arthrobacter sp. 161MFSha2.1]
gi737809108	5.30	1	1	321	34.6	5.43	2.35	ATPase [Arthrobacter sp. H5]
gi674645181	2.33	1	1	430	42.9	10.49	2.35	Low-affinity inorganic phosphate transporter 1 [Arthrobacter sp. 11W110_air]
gi928487540	2.72	1	1	662	67.0	5.17	2.35	hypothetical protein AOC05_12195 [Arthrobacter alpinus]
gi916863546	2.23	1	1	493	51.7	4.96	2.35	hypothetical protein [Arthrobacter sp. 35/47]
gi162952384	5.56	1	1	198	21.8	4.88	2.35	NADPH-dependent fmn reductase [Renibacterium salmoninarum ATCC 33209]
gi542110713	3.27	2	1	672	74.1	5.08	2.35	hypothetical protein M707_01385 [Arthrobacter sp. AK-YN10]
gi910695847	10.99	1	1	91	10.1	9.52	2.35	30S ribosomal protein S3 [Arthrobacter sp. Hiyo6]
gi927295187	9.02	3	1	244	25.3	7.40	2.35	hypothetical protein AL755_16150 [Arthrobacter sp. ERGS1:01]
gi219860776	16.04	1	1	106	10.5	11.03	2.35	TadE family protein [Arthrobacter chlorophenolicus A6]
gi767258901	2.91	1	1	344	37.4	5.78	2.35	molybdenum cofactor biosynthesis protein MoeA [Arthrobacter sp. IHBB 11108]
gi916871839	7.81	1	1	256	26.8	9.92	2.34	molybdenum ABC transporter ATP-binding protein, partial [Arthrobacter sp. H5]
gi937259576	4.88	1	1	328	35.1	6.04	2.34	hydroxyacid dehydrogenase [Arthrobacter sp. Edens01]
gi939036367	11.51	2	1	139	14.6	9.28	2.34	hypothetical protein [Arthrobacter nitroguajacolicus]
gi651443225	3.76	1	1	452	47.7	8.15	2.34	metabolite transporter [Arthrobacter sp. 9MFCol3.1]
gi737801566	2.69	1	1	557	59.2	6.01	2.34	hypothetical protein [Arthrobacter castelli]
gi651475181	4.64	3	1	388	42.6	5.77	2.34	luciferase [Arthrobacter nicotinovorans]
gi219859174	10.13	1	1	237	25.3	6.61	2.34	response regulator receiver and unknown domain protein [Arthrobacter chlorophenolicus A6]
gi651494260	3.98	2	1	553	57.6	5.85	2.34	amidohydrolase [Arthrobacter sp. H20]
gi742755018	5.97	1	1	201	21.4	4.68	2.34	alanine racemase [Arthrobacter phenanthrenivorans]
gi930827542	3.20	1	1	749	77.9	6.39	2.34	carbonate dehydratase [Arthrobacter arilaitensis]

gi823667715	19.09	1	1	110	12.3	6.68	2.34	acetyltransferase [Arthrobacter sp. YC-RL1]
gi674644970	2.76	1	1	326	34.2	7.87	2.34	Glucokinase [Arthrobacter sp. 11W110_air]
gi759711947	10.98	1	1	173	18.8	7.14	2.34	GNAT family acetyltransferase [Arthrobacter sp. 162MFSha1.1]
gi916926491	1.57	1	1	701	76.0	5.25	2.34	4-alpha-glucanotransferase [Arthrobacter sp. 9MFCol3.1]
gi674646377	14.29	1	1	154	16.9	11.59	2.34	hypothetical protein BN1051_02585 [Arthrobacter sp. 11W110_air]
gi476400475	15.54	1	1	148	15.9	6.60	2.34	UspA domain-containing protein [Arthrobacter crystallopoietes BAB-32]
gi916834558	9.92	1	1	121	13.3	8.46	2.34	hypothetical protein [Arthrobacter sp. H14]
gi759710143	7.37	2	1	339	35.0	5.40	2.34	hypothetical protein [Arthrobacter sp. 135MFCol5.1]
gi517592603	3.24	1	1	401	44.0	7.12	2.34	DNA recombination protein RecF [Arthrobacter sp. 135MFCol5.1]
gi476399791	3.01	1	1	565	61.5	6.77	2.34	CTP synthetase [Arthrobacter crystallopoietes BAB-32]
gi651495900	2.48	1	1	323	34.8	6.54	2.34	hypothetical protein [Arthrobacter sp. H20]
gi542110207	9.78	1	1	184	20.4	6.07	2.34	hypothetical protein M707_02465 [Arthrobacter sp. AK-YN10]
gi651438047	4.79	1	1	355	39.5	7.50	2.34	hypothetical protein [Arthrobacter sp. H14]
gi654822126	1.53	1	1	718	79.3	5.92	2.34	pyruvate dehydrogenase, partial [Arthrobacter sp. I3]
gi542110138	7.32	1	1	164	17.8	5.29	2.34	hypothetical protein M707_02115 [Arthrobacter sp. AK-YN10]
gi640197322	3.97	2	1	428	44.6	5.25	2.34	sugar ABC transporter substrate-binding protein [Arthrobacter sp. 31Y]
gi476400013	5.35	1	1	449	46.3	5.27	2.34	serine protease, subtilase family protein [Arthrobacter crystallopoietes BAB-32]
gi651466494	4.13	1	1	218	24.1	4.73	2.34	cell division protein [Arthrobacter sp. 35/47]
gi908699016	4.23	1	1	355	40.4	5.74	2.34	radical SAM protein [Arthrobacter sp. RIT-PI-e]
gi759721703	26.92	3	1	78	8.4	10.36	2.34	hypothetical protein [Arthrobacter nicotinovorans]
gi517606230	3.50	1	1	429	47.6	9.45	2.34	hypothetical protein [Arthrobacter sp. 161MFSha2.1]
gi759731925	11.71	1	1	222	23.1	6.57	2.34	16S rRNA methyltransferase [Arthrobacter sp. L77]
gi930825932	2.54	5	1	629	70.1	5.62	2.34	phenol 2-monooxygenase [Arthrobacter arilaitensis]
gi749402048	5.17	1	1	232	25.5	7.34	2.34	relaxase, partial [Arthrobacter sp. AK-YN10]
gi651434146	11.90	1	1	126	13.0	4.54	2.33	50S ribosomal protein L7/L12 [Arthrobacter sp. H41]
gi916869741	7.22	1	1	194	21.1	5.80	2.33	hypothetical protein [Arthrobacter sp. Br18]
gi910696692	9.55	1	1	220	23.4	8.18	2.33	hypothetical protein AHiyo6_10020 [Arthrobacter sp. Hiyo6]
gi651461442	6.44	2	1	202	23.3	9.44	2.33	hypothetical protein [Arthrobacter sp. 35/47]
gi823666886	3.35	3	1	477	52.4	4.79	2.33	Putrescine oxidase [Arthrobacter sp. YC-RL1]
gi551255234	3.65	1	1	329	35.1	5.33	2.33	dehydrogenase [Arthrobacter sp. PAO19]
gi323470611	7.22	1	1	291	30.8	5.33	2.33	3-hydroxyacyl-CoA dehydrogenase [Arthrobacter phenanthrenivorans Sphe3]
gi918267895	7.88	1	1	241	25.7	9.67	2.33	hypothetical protein AHiyo1_11840 [Arthrobacter sp. Hiyo1]
gi443479862	8.54	1	1	246	25.3	8.13	2.33	citrate/H+ symporter, partial [Arthrobacter nitrophenolicus]
gi651488993	4.64	1	1	237	25.4	9.55	2.33	hypothetical protein, partial [Arthrobacter sp. H20]
gi517590970	20.59	1	1	102	11.2	9.86	2.33	hypothetical protein [Arthrobacter sp. 135MFCol5.1]
gi116611373	3.80	1	1	237	26.7	8.68	2.33	conserved hypothetical protein [Arthrobacter sp. FB24]
gi737783085	7.94	1	1	277	28.5	10.13	2.33	hypothetical protein [Arthrobacter sp. 35W]
gi917760149	5.88	1	1	374	39.7	9.39	2.33	pyridine nucleotide-disulfide oxidoreductase [Arthrobacter sp. L77]
gi916816094	6.82	1	1	132	14.3	5.63	2.33	hypothetical protein [Arthrobacter sp. MA-N2]
gi906448509	6.19	1	1	210	22.7	5.34	2.33	hypothetical protein AC792_01825 [Arthrobacter sp. RIT-PI-e]
gi928487926	2.52	1	1	397	40.8	9.23	2.33	potassium transporter [Arthrobacter alpinus]
gi757623793	3.36	1	1	566	60.4	5.20	2.33	lipoprotein LpqB [Arthrobacter sp. SPG23]
gi517599413	22.86	1	1	105	11.1	5.22	2.33	hypothetical protein [Arthrobacter sp. 162MFSha1.1]
gi162955044	21.59	1	1	88	9.1	9.14	2.33	hypothetical protein RSal33209_2835 [Renibacterium salmoninarum ATCC 33209]
gi652423895	15.91	1	1	88	10.0	5.03	2.33	hypothetical protein [Arthrobacter castelli]
gi742758817	6.34	1	1	363	37.9	10.30	2.33	nitrite reductase [Arthrobacter phenanthrenivorans]
gi916820560	5.36	2	1	280	31.2	9.98	2.33	hypothetical protein [Arthrobacter sp. H20]
gi928487161	3.18	1	1	535	56.5	9.57	2.33	ABC transporter [Arthrobacter alpinus]
gi937256466	2.25	1	1	400	40.2	10.92	2.33	hypothetical protein AO716_15480 [Arthrobacter sp. Edens01]
gi517600363	14.58	1	1	96	10.2	10.33	2.33	hypothetical protein [Arthrobacter sp. 162MFSha1.1]
gi551254891	2.87	1	1	349	39.4	6.14	2.33	hypothetical protein [Arthrobacter sp. PAO19]
gi470220150	7.66	1	1	222	24.3	8.79	2.33	ubiquinone/menaquinone biosynthesis methyltransferase [Arthrobacter gangotriensis Lz1y]
gi639131166	20.62	1	1	97	10.0	9.70	2.33	hypothetical protein, partial [Arthrobacter sp. CAL618]
gi517590218	3.27	1	1	397	42.5	6.02	2.33	pilus biosynthesis protein CpaE [Arthrobacter sp. 135MFCol5.1]
gi476402599	4.06	2	1	468	49.7	6.23	2.33	recombination factor protein RarA [Arthrobacter crystallopoietes BAB-32]
gi542110202	5.06	2	1	178	19.1	5.24	2.33	pyridoxamine 5-phosphate oxidase [Arthrobacter sp. AK-YN10]
gi759730411	4.03	1	1	298	33.7	6.34	2.33	glycosyl transferase family 2 [Arthrobacter sp. L77]
gi518312925	6.15	1	1	358	39.8	4.94	2.33	peptide chain release factor 1 [Arthrobacter sp. TB 23]
gi767258373	3.83	2	1	470	52.8	5.58	2.33	chromosomal replication initiation protein [Arthrobacter sp. IHBB 11108]

gi723608728	6.76	1	1	222	23.5	4.96	2.33	O-methyltransferase [Arthrobacter sp. PAMC25486]
gi651434502	1.88	1	1	639	70.4	6.46	2.33	DNA primase [Arthrobacter sp. H41]
gi823666577	4.19	1	1	454	47.4	4.74	2.33	cystathionine beta-synthase [Arthrobacter sp. YC-RL1]
gi515765558	4.78	1	1	418	43.1	9.86	2.32	hypothetical protein [Arthrobacter sp. M2012083]
gi737777950	2.17	1	1	460	52.4	6.60	2.32	hypothetical protein [Arthrobacter sanguinis]
gi518312835	6.78	1	1	177	19.4	6.54	2.32	hypothetical protein [Arthrobacter sp. TB 23]
gi651481941	3.37	1	1	416	43.4	4.96	2.32	phosphoglycerate kinase [Arthrobacter sp. Br18]
gi219859971	8.05	1	1	298	33.1	8.02	2.32	glycosyl transferase family 2 [Arthrobacter chlorophenolicus A6]
gi476400963	6.82	1	1	220	25.0	5.88	2.32	GntR family transcriptional regulator [Arthrobacter crystallopoietes BAB-32]
gi910248806	8.29	2	1	193	21.2	8.16	2.32	hypothetical protein [Arthrobacter siccitolerans]
gi742854469	8.95	1	1	190	21.3	5.55	2.32	peptide deformylase [Arthrobacter sp. W1]
gi403228077	4.94	1	1	263	28.6	6.32	2.32	glutamine transport ATP-binding protein GlnQ [Arthrobacter sp. Rue61a]
gi517603551	15.75	2	1	127	13.9	5.90	2.32	hypothetical protein [Arthrobacter sp. 131MFCol6.1]
gi674645433	6.04	1	1	265	28.3	5.31	2.32	hypothetical protein BN1051_01615 [Arthrobacter sp. 11W110_air]
gi47059634	15.93	1	1	113	12.9	7.17	2.32	AtzB, partial [Arthrobacter sp. MCMB-436]
gi116610101	2.46	2	1	284	32.6	6.18	2.32	Tryptophan 2,3-dioxygenase apoenzyme / Tryptophan 2,3-dioxygenase holoenzyme [Arthrobacter [illegible]
gi757624584	5.06	1	1	237	23.5	5.29	2.32	hypothetical protein TV39_09680 [Arthrobacter sp. SPG23]
gi219858645	4.90	1	1	286	29.7	6.09	2.32	NAD-dependent epimerase/dehydratase [Arthrobacter chlorophenolicus A6]
gi823667309	2.07	1	1	723	75.5	6.32	2.32	ATPase [Arthrobacter sp. YC-RL1]
gi916871737	5.53	1	1	416	43.3	4.88	2.32	hypothetical protein [Arthrobacter sp. H5]
gi162953249	6.08	1	1	181	19.5	6.80	2.32	luciferase-like monooxygenase [Renibacterium salmoninarum ATCC 33209]
gi759736659	31.94	4	1	72	7.1	10.48	2.32	hypothetical protein [Arthrobacter sp. L77]
gi742758445	13.79	2	1	87	9.8	4.63	2.32	hypothetical protein RM50_03690 [Arthrobacter phenanthrenivorans]
gi910740173	6.33	1	1	237	25.0	5.35	2.32	uncharacterized ABC transporter ATP-binding protein MJ1508 [Arthrobacter sp. Hiyo4]
gi753931947	12.62	2	1	103	11.3	8.92	2.32	hypothetical protein [Arthrobacter arilaitensis]
gi927294324	2.05	1	1	488	50.4	5.07	2.32	carbohydrate kinase [Arthrobacter sp. ERGS1:01]
gi910696064	1.94	1	1	413	45.7	5.00	2.32	2-isopropylmalate synthase [Arthrobacter sp. Hiyo6]
gi723609851	3.22	1	1	373	39.6	6.25	2.32	hypothetical protein ART_3628 [Arthrobacter sp. PAMC25486]
gi443482045	5.25	1	1	305	33.6	7.12	2.32	AraC family transcriptional regulator [Arthrobacter nitrophenolicus]
gi765005050	10.00	1	1	100	10.5	9.94	2.32	XRE family transcriptional regulator [Arthrobacter sp. A3]
gi914717153	2.18	1	1	550	56.9	5.49	2.32	amidohydrolase [Arthrobacter sp. ZBG10]
gi930827840	6.91	1	1	217	24.2	5.90	2.32	hypothetical protein AOZ07_17205 [Arthrobacter arilaitensis]
gi918265159	28.00	1	1	75	8.4	9.64	2.31	hypothetical protein AHiyo1_50850 [Arthrobacter sp. Hiyo1]
gi908698438	2.12	1	1	567	61.7	6.00	2.31	CTP synthetase [Arthrobacter sp. RIT-PI-e]
gi307743672	17.65	1	1	102	11.2	10.74	2.31	hypothetical protein AARI_04090 [Arthrobacter arilaitensis Re117]
gi518311560	4.62	1	1	411	42.6	4.88	2.31	acetylornithine deacetylase [Arthrobacter sp. TB 23]
gi937262020	3.01	2	1	299	31.5	10.61	2.31	hypothetical protein AO716_02015 [Arthrobacter sp. Edens01]
gi551256178	5.05	1	1	297	33.2	6.55	2.31	glmZ(sRNA)-inactivating NTPase [Arthrobacter sp. PAO19]
gi737788225	4.02	1	1	398	43.1	5.20	2.31	glutaryl-CoA dehydrogenase [Arthrobacter albus]
gi359306145	1.24	2	1	1045	112.4	6.19	2.31	putative transporter [Arthrobacter globiformis NBRC 12137]
gi930826546	6.71	1	1	313	33.7	6.18	2.31	OpcA protein [Arthrobacter arilaitensis]
gi654811865	3.41	1	1	557	61.0	5.10	2.31	AMP-binding protein [Arthrobacter sp. MA-N2]
gi917759875	3.89	1	1	566	60.7	7.56	2.31	hypothetical protein [Arthrobacter sp. L77]
gi939050801	19.66	1	1	117	12.6	5.97	2.31	hypothetical protein [Arthrobacter sp. JCM 19049]
gi740676385	8.22	2	1	219	23.9	6.81	2.31	GntR family transcriptional regulator [Arthrobacter sp. PAMC25486]
gi765010193	2.65	1	1	339	36.5	5.03	2.31	2-hydroxyacid dehydrogenase [Arthrobacter sp. A3]
gi917760267	3.54	1	1	395	41.5	9.01	2.31	hypothetical protein [Arthrobacter sp. L77]
gi651441739	3.32	1	1	512	51.1	6.30	2.31	bifunctional ADP-dependent (S)-NAD(P)H-hydrate dehydratase/NAD(P)H-hydrate epimerase [Arthr[illegible]
gi910743470	19.00	3	1	100	11.2	5.57	2.31	cell wall synthesis protein Wag31 [Arthrobacter sp. Hiyo8]
gi742070984	3.36	1	1	446	46.2	9.44	2.31	3-oxoacyl-ACP reductase [Arthrobacter sp. MWB30]
gi737793116	5.24	1	1	267	29.5	4.98	2.31	N-acyl homoserine lactonase [Arthrobacter nicotinovorans]
gi910283929	5.76	1	1	399	42.3	5.90	2.31	hypothetical protein [Arthrobacter sp. A3]
gi651462200	1.96	1	1	561	57.4	4.64	2.31	phosphoenolpyruvate-protein phosphotransferase [Arthrobacter sp. 35/47]
gi823668660	20.00	2	1	85	9.2	8.18	2.31	hypothetical protein AA310_00675 [Arthrobacter sp. YC-RL1]
gi737780214	1.75	1	1	514	56.4	6.13	2.31	MULTISPECIES: ABC transporter ATP-binding protein [Arthrobacter]
gi917739313	9.43	1	1	106	11.8	8.34	2.31	hypothetical protein [Arthrobacter sp. W1]
gi219859076	4.40	3	1	386	40.4	4.86	2.31	conserved hypothetical protein [Arthrobacter chlorophenolicus A6]
gi737813151	3.66	1	1	437	46.7	8.44	2.31	hypothetical protein [Arthrobacter sp. H14]
gi551255509	3.13	1	1	448	47.7	5.99	2.31	hypothetical protein [Arthrobacter sp. PAO19]

gi542107133	1.63	1	1	981	104.6	5.17	2.31	ferredoxin [Arthrobacter sp. AK-YN10]
gi635352435	4.13	2	1	242	25.9	5.36	2.31	ribonuclease PH [Arthrobacter siccitolerans]
gi910749295	4.68	1	1	171	19.4	7.20	2.31	integrase domain protein SAM domain protein (plasmid) [Arthrobacter sp. Hiyo8]
gi914717780	3.37	2	1	624	65.8	5.87	2.31	sodium:proton antiporter [Arthrobacter sp. ZBG10]
gi910696533	5.93	1	1	270	29.7	11.30	2.31	hypothetical protein AHiyo6_11180 [Arthrobacter sp. Hiyo6]
gi723606894	3.42	1	1	526	57.7	5.80	2.31	proteasome component [Arthrobacter sp. PAMC25486]
gi916781736	3.48	1	1	374	38.9	6.54	2.31	hypothetical protein [Arthrobacter sp. 35W]
gi651441028	8.18	1	1	110	12.3	7.11	2.31	hypothetical protein [Arthrobacter sp. 9MFCol3.1]
gi307746578	1.73	1	1	1042	114.5	5.30	2.31	beta-galactosidase [Arthrobacter arilaitensis Re117]
gi403231958	2.88	1	1	417	44.0	5.73	2.31	amidohydrolase (plasmid) [Arthrobacter sp. Rue61a]
gi767257063	5.33	2	1	300	32.5	6.96	2.31	hypothetical protein UM93_04440 [Arthrobacter sp. IHBB 11108]
gi489895533	4.16	1	1	649	69.9	5.40	2.31	3D-(3,5/4)-trihydroxycyclohexane-1,2-dione acylhydrolase (decyclizing) [Arthrobacter globiformis]
gi927293693	6.57	1	1	274	30.3	8.79	2.30	hypothetical protein AL755_06035 [Arthrobacter sp. ERGS1:01]
gi908740332	8.98	1	1	167	19.0	9.98	2.30	hypothetical protein [Arthrobacter arilaitensis]
gi470221194	3.96	1	1	278	29.1	5.58	2.30	hydrolase [Arthrobacter gangotriensis Lz1y]
gi476401301	7.26	1	1	317	35.8	5.50	2.30	Glyoxalase/bleomycin resistance protein/dioxygenase [Arthrobacter crystallopoietes BAB-32]
gi476400028	9.15	1	1	142	15.6	5.30	2.30	HIT family hydrolase, diadenosine tetraphosphate hydrolase [Arthrobacter crystallopoietes BAB-32
gi339479421	6.50	1	1	323	35.3	5.03	2.30	Prephenate dehydratase [Bifidobacterium breve UCC2003]
gi737787197	9.26	1	1	162	17.9	4.68	2.30	transcription elongation factor GreA [Arthrobacter albus]
gi910744778	12.68	2	1	142	15.2	9.29	2.30	dipeptide transport system permease protein DppB [Arthrobacter sp. Hiyo8]
gi908699196	4.06	1	1	271	30.4	7.52	2.30	UDP pyrophosphate synthase [Arthrobacter sp. RIT-PI-e]
gi917013249	3.62	1	1	470	50.2	5.40	2.30	hypothetical protein [Arthrobacter sanguinis]
gi443480255	3.89	3	1	566	63.4	6.62	2.30	NERD domain-containing protein [Arthrobacter nitrophenolicus]
gi723606232	4.44	1	1	270	28.5	6.14	2.30	cytochrome c oxidase subunit II [Arthrobacter sp. PAMC25486]
gi919218871	1.36	1	1	809	80.9	5.87	2.30	hypothetical protein [Arthrobacter sp. YC-RL1]
gi928488223	3.06	1	1	523	54.6	5.87	2.30	histidine ammonia-lyase [Arthrobacter alpinus]
gi914717844	1.57	1	1	574	62.0	9.23	2.30	choline transporter, partial [Arthrobacter sp. ZBG10]
gi515767642	4.97	2	1	322	35.3	8.51	2.30	NUDIX hydrolase [Arthrobacter sp. M2012083]
gi737765102	8.04	1	1	224	25.0	6.81	2.30	uridine kinase [Arthrobacter sp. 161MFSha2.1]
gi916820457	2.60	1	1	385	40.7	9.26	2.30	sugar ABC transporter [Arthrobacter sp. H20]
gi916835201	4.26	1	1	352	39.4	9.74	2.30	integrase [Arthrobacter sp. H14]
gi723606314	0.97	1	1	924	98.1	5.58	2.30	glycine dehydrogenase [Arthrobacter sp. PAMC25486]
gi737807755	4.86	1	1	288	30.3	5.97	2.30	hypothetical protein, partial [Arthrobacter sp. H5]
gi927294909	5.49	2	1	255	26.3	6.68	2.30	methionine aminopeptidase [Arthrobacter sp. ERGS1:01]
gi640203272	1.23	1	1	1377	144.4	4.87	2.30	hypothetical protein [Arthrobacter sp. 31Y]
gi410689683	12.40	1	1	129	14.2	5.87	2.30	Mercuric resistance operon regulatory protein (plasmid) [Arthrobacter sp. J3-40]
gi517602032	2.58	1	1	581	62.4	5.11	2.30	hypothetical protein [Arthrobacter sp. 162MFSha1.1]
gi651443602	8.62	1	1	325	32.0	8.69	2.30	polysaccharide deacetylase [Arthrobacter sp. 9MFCol3.1]
gi517599567	8.38	1	1	179	18.8	7.15	2.30	TetR family transcriptional regulator [Arthrobacter sp. 162MFSha1.1]
gi918268502	4.55	1	1	374	42.6	8.32	2.30	tyrosine recombinase XerD [Arthrobacter sp. Hiyo1]
gi769942013	3.21	1	1	374	42.1	5.29	2.30	GNAT family N-acetyltransferase [Arthrobacter sp. IHBB 11108]
gi723608790	2.44	1	1	328	34.2	5.26	2.30	pantoate--beta-alanine ligase [Arthrobacter sp. PAMC25486]
gi654823028	5.92	1	1	338	35.7	6.04	2.30	exonuclease [Arthrobacter sp. I3]
gi470220753	7.77	1	1	206	23.0	9.96	2.30	ribosomal-protein-alanine N-acetyltransferase [Arthrobacter gangotriensis Lz1y]
gi737801288	3.56	1	1	309	33.7	4.96	2.30	aldose epimerase [Arthrobacter castelli]
gi786029752	2.47	1	1	728	76.2	5.11	2.29	acetyl-CoA carboxylase [Arthrobacter chlorophenolicus]
gi359305354	3.44	1	1	378	39.5	5.16	2.29	hypothetical protein ARGLB_069_00140 [Arthrobacter globiformis NBRC 12137]
gi518313516	7.59	2	1	237	24.9	5.92	2.29	MULTISPECIES: DNA-binding response regulator [Arthrobacter]
gi916324953	3.81	1	1	525	56.5	7.39	2.29	hypothetical protein [Arthrobacter gangotriensis]
gi651429772	2.17	1	1	784	83.1	5.62	2.29	hypothetical protein [Arthrobacter sanguinis]
gi749402562	7.52	1	1	266	29.5	5.03	2.29	hypothetical protein M707_04355 [Arthrobacter sp. AK-YN10]
gi162955538	8.73	1	1	252	27.8	4.89	2.29	putative acetyltransferase [Renibacterium salmoninarum ATCC 33209]
gi823668199	7.65	2	1	183	20.0	5.80	2.29	hypothetical protein AA310_02210 [Arthrobacter sp. YC-RL1]
gi651494303	4.68	1	1	385	39.6	9.35	2.29	peptidase M50 [Arthrobacter sp. H20]
gi690774005	7.41	1	1	351	36.4	5.62	2.29	hypothetical protein HMPREF2128_01885 [Arthrobacter albus DNF00011]
gi674644720	21.05	1	1	76	8.0	8.03	2.29	hypothetical protein BN1051_00877 [Arthrobacter sp. 11W110_air]
gi323470433	6.20	1	1	242	25.7	5.99	2.29	response regulator containing a CheY-like receiver domain and an HTH DNA-binding domain prote
gi515766324	10.12	1	1	168	17.1	8.41	2.29	aminoacyl-tRNA deacylase [Arthrobacter sp. M2012083]
gi470217259	3.26	1	1	491	53.9	6.77	2.29	ethanolamine two-component sensor histidine kinase [Arthrobacter gangotriensis Lz1y]

gi742854258	2.46	1	1	284	29.5	5.67	2.29	2,3-dihydroxy-2,3-dihydrophenylpropionate dehydrogenase [Arthrobacter sp. W1]
gi654822785	5.20	1	1	346	36.7	6.01	2.29	dehydrogenase [Arthrobacter sp. I3]
gi219860906	4.59	1	1	305	34.4	9.26	2.29	protein of unknown function DUF817 [Arthrobacter chlorophenolicus A6]
gi937262335	2.46	1	1	406	41.7	4.96	2.29	hypothetical protein AO716_03795 [Arthrobacter sp. Edens01]
gi651443980	11.76	1	1	119	12.9	10.67	2.29	50S ribosomal protein L24 [Arthrobacter nicotinovorans]
gi517604408	1.42	1	1	848	87.6	6.13	2.29	RND transporter [Arthrobacter sp. 131MFCol6.1]
gi640200244	5.45	1	1	312	32.7	5.02	2.29	phosphoglycerate dehydrogenase [Arthrobacter sp. 31Y]
gi939050141	9.30	1	1	215	24.4	7.81	2.29	hypothetical protein [Arthrobacter sp. JCM 19049]
gi116612457	6.10	2	1	328	34.0	9.06	2.29	chaperone DnaJ domain protein [Arthrobacter sp. FB24]
gi759709895	2.64	2	1	454	47.9	8.28	2.29	hypothetical protein [Arthrobacter sp. 9MFCol3.1]
gi759764453	16.05	1	1	81	9.1	10.58	2.29	phage-shock protein [Arthrobacter gangotriensis]
gi674644001	5.05	1	1	198	20.6	10.14	2.29	Arginine exporter protein ArgO [Arthrobacter sp. 11W110_air]
gi760112605	16.55	1	1	139	14.6	10.26	2.29	hypothetical protein [Arthrobacter chlorophenolicus]
gi307744481	8.27	1	1	278	29.5	5.06	2.29	thiosulfate sulfurtransferase-like protein [Arthrobacter arilaitensis Re117]
gi517603326	3.53	1	1	312	33.0	6.10	2.29	MULTISPECIES: ArsR family transcriptional regulator [Arthrobacter]
gi742069581	5.66	5	1	424	45.5	4.96	2.29	isochorismate synthase [Arthrobacter sp. MWB30]
gi759746475	7.03	1	1	256	27.4	6.14	2.29	phosphonate ABC transporter [Arthrobacter sp. 31Y]
gi542106200	21.70	1	1	106	12.3	6.79	2.29	hypothetical protein M707_22960 [Arthrobacter sp. AK-YN10]
gi651498146	2.04	1	1	1027	106.1	5.29	2.29	hypothetical protein [Arthrobacter sp. 35W]
gi162952763	7.69	1	1	130	14.4	6.58	2.29	transcriptional regulator, ArsR family [Renibacterium salmoninarum ATCC 33209]
gi403227805	11.95	1	1	159	17.7	4.86	2.29	putative DNA-binding stress response protein, Dps family [Arthrobacter sp. Rue61a]
gi219858173	4.94	1	1	263	28.4	9.74	2.29	short-chain dehydrogenase/reductase SDR [Arthrobacter chlorophenolicus A6]
gi767258852	5.82	1	1	275	30.6	5.55	2.29	exodeoxyribonuclease III [Arthrobacter sp. IHBB 11108]
gi908690482	6.21	1	1	322	36.0	9.09	2.28	NUDIX hydrolase [Arthrobacter sp. H41]
gi737813310	4.68	1	1	342	36.0	8.38	2.28	DNA polymerase III subunit delta [Arthrobacter sp. H14]
gi636843634	4.79	1	1	397	40.5	10.64	2.28	MFS transporter [Arthrobacter sp. TB 26]
gi542107782	4.79	1	1	313	33.5	4.86	2.28	3-(2,3-dihydroxyphenyl)propionate dioxygenase [Arthrobacter sp. AK-YN10]
gi640193588	6.11	2	1	360	39.7	8.50	2.28	mannosyltransferase [Arthrobacter sp. 31Y]
gi651460455	2.59	1	1	347	37.5	6.20	2.28	LacI family transcriptional regulator [Arthrobacter sp. 35/47]
gi916813646	2.01	1	1	747	83.5	5.31	2.28	hypothetical protein [Arthrobacter nicotinovorans]
gi651500168	8.04	2	1	286	30.3	5.03	2.28	oxidoreductase [Arthrobacter sp. 35W]
gi927031666	8.49	2	1	259	27.5	5.90	2.28	alkaline phosphatase [Arthrobacter sp. LS16]
gi765005965	7.08	1	1	325	34.3	5.67	2.28	ABC transporter [Arthrobacter sp. A3]
gi517602605	2.81	2	1	427	45.5	10.74	2.28	hypothetical protein [Arthrobacter sp. 131MFCol6.1]
gi910252564	3.32	1	1	482	50.5	7.49	2.28	dehydrogenase [Arthrobacter siccitolerans]
gi651449462	2.37	1	1	337	37.4	5.68	2.28	dehydrogenase [Arthrobacter nicotinovorans]
gi651464454	1.45	1	1	692	76.0	5.24	2.28	glycogen debranching protein [Arthrobacter sp. 35/47]
gi470217087	6.31	1	1	222	24.2	7.71	2.28	Alpha/beta hydrolase family protein [Arthrobacter gangotriensis Lz1y]
gi359304360	10.65	1	1	216	23.1	9.41	2.28	glutamate ABC transporter permease protein [Arthrobacter globiformis NBRC 12137]
gi759763865	4.74	1	1	253	27.4	5.08	2.28	3'(2'),5'-bisphosphate nucleotidase CysQ [Arthrobacter gangotriensis]
gi654819718	4.99	1	1	341	36.9	5.08	2.28	hypothetical protein [Arthrobacter sp. UNC362MFTsu5.1]
gi908690349	3.56	1	1	281	31.3	10.46	2.28	hypothetical protein [Arthrobacter sp. H41]
gi908690897	4.02	1	1	473	50.2	5.82	2.28	oxidoreductase [Arthrobacter sp. H41]
gi307744876	2.67	1	1	375	39.6	9.29	2.28	zinc metallopeptidase [Arthrobacter arilaitensis Re117]
gi786031049	7.64	2	1	314	32.7	10.45	2.28	NERD nuclease [Arthrobacter chlorophenolicus]
gi674646850	3.72	1	1	376	39.3	7.20	2.28	Spermidine/putrescine import ATP-binding protein PotA [Arthrobacter sp. 11W110_air]
gi219858231	3.10	1	1	516	53.8	5.39	2.28	cytidyltransferase-related domain protein [Arthrobacter chlorophenolicus A6]
gi654814508	3.25	1	1	461	48.3	5.06	2.28	succinate-semialdehyde dehydrogenase [Arthrobacter sp. MA-N2]
gi910283600	2.02	2	1	544	57.6	5.29	2.28	hypothetical protein [Arthrobacter sp. A3]
gi937259156	4.33	1	1	462	50.9	5.54	2.28	phospho-2-dehydro-3-deoxyheptonate aldolase [Arthrobacter sp. Edens01]
gi359306602	3.45	1	1	348	38.8	5.26	2.28	5-methyltetrahydropteroyltriglutamate--homocysteine methyltransferase [Arthrobacter globiformis
gi765011238	9.06	1	1	254	26.3	10.21	2.28	membrane protein [Arthrobacter sp. A3]
gi927031566	2.41	1	1	540	59.0	6.46	2.28	transcriptional regulator [Arthrobacter sp. LS16]
gi323467632	5.88	1	1	119	13.3	5.35	2.28	hypothetical protein Asphe3_00980 [Arthrobacter phenanthrenivorans Sphe3]
gi651500178	6.37	1	1	251	27.0	5.41	2.28	carboxymuconolactone decarboxylase [Arthrobacter sp. 35W]
gi470220057	4.37	1	1	366	38.5	5.01	2.28	Phospho-2-dehydro-3-deoxyheptonate aldolase, Phe-sensitive [Arthrobacter gangotriensis Lz1y]
gi927296054	2.25	1	1	845	92.3	6.27	2.28	metallophosphoesterase [Arthrobacter sp. ERGS1:01]
gi723606311	5.92	1	1	169	18.5	4.70	2.28	hypothetical protein ART_0088 [Arthrobacter sp. PAMC25486]
gi640202445	4.29	1	1	396	40.7	6.80	2.28	hypothetical protein [Arthrobacter sp. 31Y]

gi914714636	9.17	1	1	229	22.5	4.65	2.28	dihydroxyacetone kinase [Arthrobacter sp. ZBG10]
gi742072867	14.02	2	1	107	11.7	9.41	2.28	hypothetical protein ANMWB30_01750 [Arthrobacter sp. MWB30]
gi927295924	1.95	2	1	410	42.2	6.14	2.28	hypothetical protein AL755_20950 [Arthrobacter sp. ERGS1:01]
gi937256478	1.62	1	1	495	52.5	5.22	2.28	hypothetical protein AO716_15560 [Arthrobacter sp. Edens01]
gi651481384	1.81	4	1	775	79.2	4.70	2.27	hypothetical protein [Arthrobacter sp. Br18]
gi403231401	18.70	1	1	123	13.9	8.56	2.27	putative transcriptional regulator, ArsR family [Arthrobacter sp. Rue61a]
gi116608990	7.63	1	1	249	25.8	4.63	2.27	LamB/YcsF family protein [Arthrobacter sp. FB24]
gi443481459	11.36	2	1	132	15.1	5.87	2.27	thioesterase superfamily protein [Arthrobacter nitrophenolicus]
gi551255676	3.49	2	1	344	36.5	6.55	2.27	LacI family transcriptional regulator [Arthrobacter sp. PAO19]
gi723609124	6.08	1	1	296	31.4	6.96	2.27	metalloendopeptidase-like membrane protein [Arthrobacter sp. PAMC25486]
gi742759349	2.24	1	1	536	57.0	9.41	2.27	FAD-dependent oxidoreductase [Arthrobacter phenanthrenivorans]
gi927295146	3.18	1	1	283	29.7	5.10	2.27	thiosulfate sulfurtransferase [Arthrobacter sp. ERGS1:01]
gi476400614	5.96	1	1	285	29.5	5.22	2.27	hypothetical protein D477_012720 [Arthrobacter crystallopoietes BAB-32]
gi517605274	2.07	1	1	532	57.6	6.67	2.27	CdaR family transcriptional regulator [Arthrobacter sp. 131MFCol6.1]
gi910696954	3.37	1	1	386	42.5	6.11	2.27	putative conjugal transfer protein Rv3659c/MT3759 [Arthrobacter sp. Hiyo6]
gi937256450	9.35	1	1	139	15.4	9.29	2.27	cation transport regulator ChaB [Arthrobacter sp. Edens01]
gi648259811	1.62	1	1	1051	117.6	6.84	2.27	helicase [Arthrobacter sp. TB 23]
gi917745857	5.07	2	1	375	39.1	7.68	2.27	ATPase [Arthrobacter phenanthrenivorans]
gi914715374	6.98	1	1	215	22.0	4.89	2.27	non-canonical purine NTP pyrophosphatase [Arthrobacter sp. ZBG10]
gi690772519	7.61	1	1	184	21.4	4.51	2.27	hypothetical protein HMPREF2128_07100 [Arthrobacter albus DNF00011]
gi651484219	3.32	1	1	602	63.4	5.14	2.27	transferase [Arthrobacter sp. Br18]
gi162952493	6.33	1	1	237	25.4	8.91	2.27	branched-chain amino acid transport ATP-binding protein [Renibacterium salmoninarum ATCC 332
gi919108105	5.17	1	1	290	30.4	8.10	2.27	hypothetical protein [Arthrobacter sp. IHBB 11108]
gi162953922	4.49	1	1	379	41.1	4.94	2.27	putative carbohydrate kinase [Renibacterium salmoninarum ATCC 33209]
gi723606322	1.59	2	1	694	72.3	5.76	2.27	hypothetical protein ART_0099 [Arthrobacter sp. PAMC25486]
gi654823264	7.69	1	1	169	18.4	9.88	2.27	MarR family transcriptional regulator [Arthrobacter sp. I3]
gi910745921	4.84	1	1	455	47.0	5.48	2.27	3-carboxy-cis,cis-muconate cycloisomerase [Arthrobacter sp. Hiyo8]
gi119949034	7.19	1	1	167	18.9	6.35	2.27	putative transcriptional regulator, MarR family [Arthrobacter aurescens TC1]
gi640201588	7.19	1	1	167	18.8	6.21	2.27	MarR family transcriptional regulator [Arthrobacter sp. 31Y]
gi927294406	2.58	1	1	466	48.9	5.88	2.27	mycothione reductase [Arthrobacter sp. ERGS1:01]
gi910744031	8.60	1	1	186	18.9	10.71	2.27	hypothetical protein AHiyo8_17330 [Arthrobacter sp. Hiyo8]
gi476399084	6.70	1	1	194	21.4	7.66	2.27	transcriptional regulator [Arthrobacter crystallopoietes BAB-32]
gi323470482	4.20	2	1	429	48.1	10.89	2.27	transposase family protein [Arthrobacter phenanthrenivorans Sphe3]
gi916357144	4.39	1	1	228	24.1	6.96	2.27	haloacid dehalogenase [Arthrobacter sp. 162MFSha1.1]
gi443481043	22.32	2	1	112	11.2	5.11	2.27	HNH nuclease, partial [Arthrobacter nitrophenolicus]
gi757624587	2.12	1	1	943	102.5	6.80	2.27	LuxR family transcriptional regulator [Arthrobacter sp. SPG23]
gi654812599	22.83	1	1	92	10.3	5.15	2.27	hypothetical protein [Arthrobacter sp. MA-N2]
gi737814712	17.86	1	1	84	9.4	4.84	2.27	hypothetical protein, partial [Arthrobacter sp. H14]
gi654813615	5.36	1	1	280	29.6	4.88	2.27	short-chain dehydrogenase/reductase [Arthrobacter sp. MA-N2]
gi757624268	9.92	2	1	242	26.5	6.54	2.27	transcriptional regulator [Arthrobacter sp. SPG23]
gi219861066	9.09	1	1	110	11.8	4.97	2.27	anti-sigma-factor antagonist [Arthrobacter chlorophenolicus A6]
gi119950007	19.78	2	1	91	10.2	7.25	2.27	conserved hypothetical protein [Arthrobacter aurescens TC1]
gi517608932	4.71	1	1	297	32.0	5.33	2.27	hypothetical protein [Arthrobacter sp. 161MFSha2.1]
gi162953869	2.33	1	1	430	47.3	10.35	2.27	hypothetical membrane protein [Renibacterium salmoninarum ATCC 33209]
gi640196731	1.84	1	1	925	97.7	5.90	2.27	aldehyde oxidase [Arthrobacter sp. 31Y]
gi928488405	6.88	1	1	189	21.0	9.80	2.27	RNA polymerase subunit sigma-70 [Arthrobacter alpinus]
gi916834848	2.59	1	1	580	61.6	5.40	2.27	histidine kinase [Arthrobacter sp. H14]
gi917442177	4.51	2	1	377	39.3	4.75	2.27	hypothetical protein [Arthrobacter albus]
gi916358045	5.79	1	1	380	39.8	5.47	2.27	galactokinase [Arthrobacter sp. 131MFCol6.1]
gi119950685	4.93	1	1	304	33.8	9.83	2.27	phage integrase family domain protein [Arthrobacter aurescens TC1]
gi323471395	6.79	1	1	265	27.6	4.82	2.27	Zn-dependent alcohol dehydrogenase, class III (plasmid) [Arthrobacter phenanthrenivorans Sphe
gi910738448	7.78	1	1	270	28.6	6.14	2.27	probable serine/threonine-protein kinase CPE1738 [Arthrobacter sp. Hiyo4]
gi359304101	1.65	1	1	848	93.3	5.08	2.27	putative ATP-dependent helicase [Arthrobacter globiformis NBRC 12137]
gi928488872	3.11	1	1	418	45.6	9.69	2.27	exodeoxyribonuclease VII large subunit [Arthrobacter alpinus]
gi765005658	3.74	1	1	321	35.3	5.05	2.27	proline iminopeptidase [Arthrobacter sp. A3]
gi403230847	6.47	1	1	340	36.3	5.57	2.27	putative maltose operon transcriptional repressor, LacI family [Arthrobacter sp. Rue61a]
gi765005148	4.57	1	1	328	35.8	10.20	2.27	DNA topoisomerase I [Arthrobacter sp. A3]
gi910737901	6.21	1	1	306	33.2	5.25	2.27	conserved hypothetical protein [Arthrobacter sp. Hiyo4]
gi542107332	18.29	1	1	82	8.9	11.09	2.27	hypothetical protein M707_17170 [Arthrobacter sp. AK-YN10]

gi737796779	7.60	1	1	263	28.0	5.01	2.27	alpha/beta hydrolase [Arthrobacter sp. H20]
gi930828133	3.15	1	1	317	33.8	7.20	2.27	transcriptional regulator [Arthrobacter arilaitensis]
gi640200948	2.66	1	1	338	37.2	5.99	2.27	hypothetical protein [Arthrobacter sp. 31Y]
gi767258638	6.82	1	1	337	37.1	4.92	2.27	hypothetical protein UM93_15345 [Arthrobacter sp. IHBB 11108]
gi162955252	6.67	1	1	255	29.1	7.61	2.27	trans,cis-farnesyl diphosphate synthase [Renibacterium salmoninarum ATCC 33209]
gi116610027	9.92	1	1	121	13.6	7.20	2.27	addiction module toxin, RelE/StbE family [Arthrobacter sp. FB24]
gi651480630	6.32	1	1	190	20.5	5.78	2.27	peptide deformylase [Arthrobacter sp. Br18]
gi674646346	9.47	3	1	95	10.1	8.19	2.26	Zinc ribbon domain protein [Arthrobacter sp. 11W110_air]
gi674644955	11.54	1	1	156	16.9	5.54	2.26	Glycogen accumulation regulator GarA [Arthrobacter sp. 11W110_air]
gi908697913	7.32	1	1	246	26.7	7.62	2.26	hypothetical protein [Arthrobacter sp. RIT-PI-e]
gi119950578	12.82	2	1	78	8.1	6.51	2.26	hypothetical protein AAur_3141 [Arthrobacter aurescens TC1]
gi767258411	4.80	3	1	479	52.0	5.48	2.26	Xaa-Pro aminopeptidase [Arthrobacter sp. IHBB 11108]
gi470220440	4.01	1	1	449	49.0	9.54	2.26	membrane protein [Arthrobacter gangotriensis Lz1y]
gi323468935	3.02	1	1	298	32.8	6.57	2.26	tRNA isopentenyltransferase MiaA [Arthrobacter phenanthrenivorans Sphe3]
gi910697098	8.33	1	1	204	22.3	8.79	2.26	hypothetical protein AHiyo6_06810 [Arthrobacter sp. Hiyo6]
gi910283735	3.51	1	1	285	29.6	7.44	2.26	hypothetical protein [Arthrobacter sp. A3]
gi910737490	7.52	1	1	226	24.1	7.50	2.26	pantothenate synthetase [Arthrobacter sp. Hiyo4]
gi757625325	4.95	2	1	384	41.4	7.15	2.26	uroporphyrinogen III synthetase [Arthrobacter sp. SPG23]
gi359305272	7.16	2	1	349	36.4	6.29	2.26	cystathionine beta-synthase [Arthrobacter globiformis NBRC 12137]
gi767256485	9.57	1	1	230	24.7	5.94	2.26	LuxR family transcriptional regulator [Arthrobacter sp. IHBB 11108]
gi823666593	5.03	3	1	318	34.4	9.91	2.26	hypothetical protein AA310_12550 [Arthrobacter sp. YC-RL1]
gi651492792	6.04	1	1	265	29.0	5.12	2.26	hypothetical protein [Arthrobacter sp. H20]
gi916835155	5.91	1	1	237	26.8	5.30	2.26	hypothetical protein [Arthrobacter sp. H14]
gi917022327	5.15	1	1	408	43.5	5.48	2.26	dehydrogenase [Arthrobacter sp. UNC362MFTsu5.1]
gi640197182	5.35	1	1	430	45.8	9.42	2.26	MFS transporter [Arthrobacter sp. 31Y]
gi910748584	7.98	2	1	238	25.5	7.28	2.26	conserved hypothetical protein [Arthrobacter sp. Hiyo8]
gi323468150	9.50	2	1	200	22.2	8.18	2.26	uncharacterized conserved protein [Arthrobacter phenanthrenivorans Sphe3]
gi476402881	14.29	1	1	98	10.5	4.53	2.26	hypothetical protein D477_001384 [Arthrobacter crystallopoietes BAB-32]
gi119950996	3.91	1	1	307	33.1	4.73	2.26	segregation and condensation protein A [Arthrobacter aurescens TC1]
gi654814196	6.29	1	1	318	33.6	7.23	2.26	NAD-dependent deacetylase [Arthrobacter sp. MA-N2]
gi403229034	5.92	1	1	321	33.8	5.30	2.26	quinone oxidoreductase Qor [Arthrobacter sp. Rue61a]
gi917013147	4.41	1	1	454	46.3	5.17	2.26	hypothetical protein [Arthrobacter sanguinis]
gi359304859	7.14	1	1	196	21.9	6.74	2.26	hypothetical protein ARGLB_080_01730 [Arthrobacter globiformis NBRC 12137]
gi517590202	11.98	1	1	167	18.2	9.76	2.26	30S ribosomal protein S9 [Arthrobacter sp. 135MFCol5.1]
gi640196379	4.53	1	1	287	30.1	5.77	2.26	3-hydroxybutyryl-CoA dehydrogenase [Arthrobacter sp. 31Y]
gi690773164	7.91	1	1	177	19.7	10.08	2.26	hypothetical protein HMPREF2128_04215 [Arthrobacter albus DNF00011]
gi654814085	5.31	1	1	320	33.3	5.55	2.26	phosphofructokinase [Arthrobacter sp. MA-N2]
gi742851714	2.60	1	1	847	85.3	5.43	2.26	hypothetical protein [Arthrobacter sp. W1]
gi915933744	4.00	1	1	375	38.8	10.36	2.26	hypothetical protein [Arthrobacter globiformis]
gi908698007	13.29	1	1	143	15.4	6.11	2.26	calpastatin [Arthrobacter sp. RIT-PI-e]
gi651480576	1.85	1	1	917	101.5	5.52	2.26	pyruvate dehydrogenase [Arthrobacter sp. Br18]
gi919134870	6.36	1	1	283	27.9	5.12	2.26	hypothetical protein [Arthrobacter chlorophenolicus]
gi470217138	8.23	1	1	243	24.5	4.87	2.26	CutC family protein [Arthrobacter gangotriensis Lz1y]
gi654825665	8.09	1	1	136	14.3	4.45	2.26	ABC transporter [Arthrobacter sp. H5]
gi908691120	19.15	1	1	94	9.6	10.07	2.26	hypothetical protein [Arthrobacter sp. H41]
gi654828135	9.42	1	1	223	24.8	5.77	2.26	DNA-binding response regulator [Arthrobacter sp. H5]
gi359305678	1.56	1	1	896	97.5	4.92	2.26	aminopeptidase N [Arthrobacter globiformis NBRC 12137]
gi476402120	4.07	1	1	246	26.9	6.02	2.26	phosphoadenylylsulfate reductase (thioredoxin) [Arthrobacter crystallopoietes BAB-32]
gi162955661	2.33	1	1	386	40.7	6.81	2.26	16S rRNA m(2)G 966 methyltransferase [Renibacterium salmoninarum ATCC 33209]
gi651450155	3.17	1	1	347	36.9	5.94	2.26	endonuclease [Arthrobacter nicotinovorans]
gi651496166	4.93	2	1	345	37.2	8.24	2.26	epimerase [Arthrobacter sp. H20]
gi823667961	3.67	1	1	572	60.8	4.87	2.26	phosphomannomutase [Arthrobacter sp. YC-RL1]
gi517608632	3.54	1	1	395	42.5	7.06	2.25	hypothetical protein [Arthrobacter sp. 161MFSha2.1]
gi635353784	2.83	1	1	389	43.2	5.19	2.25	putative cytochrome P450 [Arthrobacter siccitolerans]
gi910696569	9.57	1	1	188	21.2	5.10	2.25	oligo-1,6-glucosidase, partial [Arthrobacter sp. Hiyo6]
gi919219071	5.95	1	1	370	40.1	5.41	2.25	hypothetical protein [Arthrobacter sp. YC-RL1]
gi737786943	1.78	1	1	1013	111.7	6.00	2.25	glutamine-synthetase [Arthrobacter albus]
gi651505670	1.84	2	1	871	94.6	5.31	2.25	glycogen phosphorylase [Arthrobacter sp. 35W]
gi937259043	4.43	1	1	203	21.6	7.46	2.25	hypothetical protein AO716_13960 [Arthrobacter sp. Edens01]

gi652424604	6.09	1	1	312	32.9	4.92	2.25	hypothetical protein [Arthrobacter castelli]
gi470220218	6.06	1	1	396	43.7	9.83	2.25	hypothetical protein ADIAG_01830 [Arthrobacter gangotriensis Lz1y]
gi443481234	2.86	1	1	734	78.2	6.24	2.25	hypothetical protein G205_13657 [Arthrobacter nitrophenolicus]
gi759731339	2.65	1	1	339	36.3	6.27	2.25	hypothetical protein [Arthrobacter sp. L77]
gi219861064	5.39	1	1	371	39.6	4.83	2.25	response regulator receiver protein [Arthrobacter chlorophenolicus A6]
gi740683550	2.94	1	1	476	51.6	6.70	2.25	glyceraldehyde-3-phosphate dehydrogenase [Arthrobacter sp. PAMC25486]
gi917012972	0.97	1	1	1134	125.1	5.81	2.25	hypothetical protein [Arthrobacter sanguinis]
gi443480553	3.67	1	1	409	43.4	5.29	2.25	coproporphyrinogen III oxidase [Arthrobacter nitrophenolicus]
gi651436423	3.86	1	1	233	24.9	6.57	2.25	cytidylate kinase [Arthrobacter sp. H41]
gi162955347	2.58	1	1	387	40.2	4.69	2.25	phosphoenolpyruvate-protein phosphotransferase [Renibacterium salmoninarum ATCC 33209]
gi723609605	8.30	1	1	265	30.4	5.68	2.25	hypothetical protein ART_3382 [Arthrobacter sp. PAMC25486]
gi323468327	4.39	1	1	433	42.8	4.91	2.25	amino acid/amide ABC transporter substrate-binding protein, HAAT family [Arthrobacter phenanth
gi737813643	3.12	1	1	545	60.0	4.67	2.25	ABC transporter substrate-binding protein [Arthrobacter sp. H14]
gi515768012	7.52	1	1	266	29.1	9.33	2.25	hypothetical protein [Arthrobacter sp. M2012083]
gi476400425	8.91	1	1	101	11.7	6.54	2.25	putative PadR family transcriptional regulator, partial [Arthrobacter crystallopoietes BAB-32]
gi737790035	3.40	1	1	265	27.7	4.74	2.25	tryptophan synthase alpha chain [Arthrobacter albus]
gi651472848	10.57	1	1	123	14.5	5.02	2.25	hypothetical protein [Arthrobacter nicotinovorans]
gi323471201	4.88	1	1	205	23.1	5.87	2.25	hypothetical protein Asphe3_37970 [Arthrobacter phenanthrenivorans Sphe3]
gi119950904	17.43	1	1	109	11.9	7.20	2.25	hypothetical protein AAur_2872 [Arthrobacter aurescens TC1]
gi928487839	3.35	1	1	328	35.6	7.39	2.25	exopolyphosphatase [Arthrobacter alpinus]
gi651501713	5.81	1	1	258	27.6	6.23	2.25	GntR family transcriptional regulator [Arthrobacter sp. 35W]
gi823666163	2.99	1	1	502	55.8	5.62	2.25	hypothetical protein AA310_09855 [Arthrobacter sp. YC-RL1]
gi918267464	25.61	2	1	82	8.7	9.52	2.25	hypothetical protein AHiyo1_21310 [Arthrobacter sp. Hiyo1]
gi517600461	7.05	1	1	298	32.4	7.77	2.25	hypothetical protein [Arthrobacter sp. 162MFSha1.1]
gi823666345	1.44	1	1	1114	122.5	5.19	2.25	DNA helicase UvrD [Arthrobacter sp. YC-RL1]
gi740678121	21.31	1	1	61	7.3	12.21	2.25	hypothetical protein [Arthrobacter sp. PAMC25486]
gi742755732	5.08	1	1	236	25.9	4.98	2.25	haloacid dehalogenase [Arthrobacter phenanthrenivorans]
gi654826745	2.75	1	1	436	46.6	9.23	2.25	drug:proton antiporter [Arthrobacter sp. H5]
gi307745283	3.23	1	1	371	41.1	9.98	2.25	putative phage integrase [Arthrobacter arilaitensis Re117]
gi359305462	12.50	1	1	144	15.3	5.34	2.25	putative arsenate reductase [Arthrobacter globiformis NBRC 12137]
gi518311530	12.50	1	1	96	9.9	9.77	2.25	MULTISPECIES: transcriptional regulator [Arthrobacter]
gi939036769	6.08	1	1	148	15.4	8.94	2.25	hypothetical protein [Arthrobacter nitroguajacolicus]
gi939037270	3.94	1	1	381	42.8	8.87	2.25	hypothetical protein [Arthrobacter nitroguajacolicus]
gi769939748	4.71	1	1	340	36.4	4.75	2.25	hypothetical protein [Arthrobacter sp. IHBB 11108]
gi639130456	1.78	1	1	617	68.9	8.18	2.25	ABC transporter [Arthrobacter sp. CAL618]
gi757622480	3.96	1	1	278	31.7	9.61	2.25	lipase [Arthrobacter sp. SPG23]
gi359304875	5.48	1	1	310	33.7	9.77	2.25	putative type II secretion system protein [Arthrobacter globiformis NBRC 12137]
gi307746187	8.33	1	1	144	15.2	5.43	2.25	hypothetical protein AARI_29630 [Arthrobacter arilaitensis Re117]
gi910745578	3.97	2	1	252	27.5	8.05	2.25	4-alpha-glucanotransferase [Arthrobacter sp. Hiyo8]
gi651458061	2.60	1	1	576	63.8	5.05	2.25	2-isopropylmalate synthase [Arthrobacter sp. 35/47]
gi470220287	1.50	1	1	735	78.8	5.20	2.25	pyruvate dehydrogenase (acetyl-transferring) E1 component alpha subunit [Arthrobacter gangotri
gi652423692	5.48	2	1	420	45.7	5.43	2.25	hypothetical protein [Arthrobacter castelli]
gi476400491	2.44	1	1	451	48.1	4.12	2.25	alpha-glucosides-binding extracellular protein AglE [Arthrobacter crystallopoietes BAB-32]
gi742853133	8.27	1	1	278	29.5	5.00	2.25	thiosulfate sulfurtransferase [Arthrobacter sp. W1]
gi765012028	1.82	1	1	604	64.0	5.16	2.25	hypothetical protein [Arthrobacter sp. A3]
gi823665833	4.00	1	1	400	44.5	5.88	2.25	hypothetical protein AA310_07910 [Arthrobacter sp. YC-RL1]
gi307744107	23.61	2	1	72	7.9	11.43	2.25	acetyl-/propionyl-coenzyme A carboxylase epsilon chain [Arthrobacter arilaitensis Re117]
gi518312772	4.01	2	1	424	46.0	5.48	2.24	hypothetical protein [Arthrobacter sp. TB 23]
gi119950863	4.05	1	1	247	26.7	6.32	2.24	putative ABC transporter efflux protein, DrrB family [Arthrobacter aurescens TC1]
gi654826401	9.95	1	1	191	20.9	6.74	2.24	XRE family transcriptional regulator [Arthrobacter sp. H5]
gi648574485	5.54	1	1	289	30.3	9.42	2.24	chromosome partitioning protein ParA [Arthrobacter sp. 131MFCol6.1]
gi517606640	5.08	1	1	197	21.7	9.39	2.24	hypothetical protein [Arthrobacter sp. 161MFSha2.1]
gi651432704	2.76	1	1	471	52.2	8.69	2.24	hypothetical protein [Arthrobacter sp. H41]
gi517602894	5.13	1	1	351	37.6	7.06	2.24	DNA polymerase IV [Arthrobacter sp. 131MFCol6.1]
gi640196209	2.93	1	1	375	39.2	5.15	2.24	hypothetical protein [Arthrobacter sp. 31Y]
gi116608956	6.94	1	1	245	26.2	5.60	2.24	response regulator receiver and ANTAR domain protein [Arthrobacter sp. FB24]
gi916782076	9.02	1	1	133	14.2	6.52	2.24	phosphoribosyl-AMP cyclohydrolase [Arthrobacter sp. 35W]
gi914715018	5.71	1	1	140	15.0	11.87	2.24	peptide chain release factor 1 [Arthrobacter sp. ZBG10]
gi737815339	15.15	1	1	66	7.3	5.38	2.24	hypothetical protein [Arthrobacter sp. H14]

gi930827364	8.57	2	1	210	22.4	5.03	2.24	lactate utilization protein C [Arthrobacter arilaitensis]
gi307744800	4.03	1	1	471	48.5	8.44	2.24	gluconate permease [Arthrobacter arilaitensis Re117]
gi119948006	3.20	1	1	532	57.8	5.71	2.24	putative cytochrome d ubiquinol oxidase subunit I (CydA) [Arthrobacter aurescens TC1]
gi941912998	6.31	2	1	206	23.3	5.35	2.24	hypothetical protein [Arthrobacter sp. Edens01]
gi476401766	1.96	1	1	408	42.6	5.95	2.24	bifunctional phosphopantothenoylcysteine decarboxylase/phosphopantothenate synthase [Arthrob
gi443481796	11.18	1	1	170	17.7	11.88	2.24	major facilitator transporter [Arthrobacter nitrophenolicus]
gi116611477	2.01	1	1	547	58.5	5.45	2.24	extracellular solute-binding protein, family 5 [Arthrobacter sp. FB24]
gi651499806	4.90	1	1	286	31.5	9.57	2.24	alpha/beta hydrolase [Arthrobacter sp. 35W]
gi914714694	3.65	1	1	274	29.4	9.04	2.24	short-chain dehydrogenase [Arthrobacter sp. ZBG10]
gi914717829	16.92	1	1	130	14.2	7.40	2.24	hypothetical protein [Arthrobacter sp. ZBG10]
gi910739832	17.05	1	1	129	14.0	6.52	2.24	conserved hypothetical protein [Arthrobacter sp. Hiyo4]
gi651503806	5.31	1	1	414	44.7	8.40	2.24	exodeoxyribonuclease VII large subunit [Arthrobacter sp. 35W]
gi162954164	5.00	4	1	280	30.0	5.82	2.24	short chain dehydrogenase [Renibacterium salmoninarum ATCC 33209]
gi323470566	8.63	1	1	197	21.0	5.14	2.24	methylase involved in ubiquinone/menaquinone biosynthesis [Arthrobacter phenanthrenivorans Sp
gi910695309	15.63	1	1	96	10.8	11.27	2.24	hypothetical protein AHiyo6_20260 [Arthrobacter sp. Hiyo6]
gi640194947	9.55	1	1	178	18.8	6.54	2.24	hypothetical protein [Arthrobacter sp. 31Y]
gi914714116	1.77	1	1	679	74.0	5.25	2.24	beta-galactosidase [Arthrobacter sp. ZBG10]
gi652424419	4.39	1	1	410	42.0	5.25	2.24	hypothetical protein [Arthrobacter castelli]
gi916863665	6.67	1	1	315	34.7	8.09	2.24	sugar ABC transporter permease [Arthrobacter sp. 35/47]
gi359306275	6.81	1	1	279	30.5	4.89	2.24	hypothetical protein ARGLB_040_00250 [Arthrobacter globiformis NBRC 12137]
gi742855121	4.82	1	1	332	36.5	9.73	2.24	NAD-dependent epimerase [Arthrobacter sp. W1]
gi551254623	23.08	1	1	78	8.3	5.05	2.24	hypothetical protein [Arthrobacter sp. PAO19]
gi742069381	4.06	1	1	320	34.7	5.21	2.24	hypothetical protein ANMWB30_36590 [Arthrobacter sp. MWB30]
gi476400307	5.22	1	1	230	23.5	4.39	2.24	hypothetical protein D477_014361 [Arthrobacter crystallopoietes BAB-32]
gi651475559	9.89	2	1	263	27.5	5.05	2.24	multidrug transporter [Arthrobacter nicotinovorans]
gi916820359	6.14	1	1	228	25.6	5.01	2.24	phosphate ABC transporter permease [Arthrobacter sp. H20]
gi651435082	3.17	2	1	694	72.9	4.94	2.24	protein kinase [Arthrobacter sp. H41]
gi930827933	18.52	1	1	81	9.3	6.79	2.24	hypothetical protein AOZ07_17725 [Arthrobacter arilaitensis]
gi476399719	4.02	1	1	249	26.9	7.58	2.24	TetR family transcriptional regulator [Arthrobacter crystallopoietes BAB-32]
gi908698732	13.41	2	1	164	18.0	6.39	2.24	MarR family transcriptional regulator [Arthrobacter sp. RIT-PI-e]
gi162954212	4.41	1	1	227	24.7	10.08	2.24	conserved hypothetical protein [Renibacterium salmoninarum ATCC 33209]
gi937261723	1.53	1	1	1045	109.3	4.54	2.23	hypothetical protein AO716_04925 [Arthrobacter sp. Edens01]
gi518312607	5.99	1	1	334	36.2	6.87	2.23	MULTISPECIES: aspartate carbamoyltransferase [Arthrobacter]
gi916863230	2.76	1	1	616	67.9	8.00	2.23	hypothetical protein [Arthrobacter sp. 35/47]
gi910746347	7.44	1	1	121	12.7	5.50	2.23	rhs element Vgr protein [Arthrobacter sp. Hiyo8]
gi737813169	5.67	1	1	300	32.4	5.91	2.23	NAD-dependent deacetylase [Arthrobacter sp. H14]
gi470221140	4.47	1	1	291	31.8	5.15	2.23	glucose-1-phosphate thymidylyltransferase [Arthrobacter gangotriensis Lz1y]
gi759723412	14.56	1	1	103	11.5	9.41	2.23	hypothetical protein [Arthrobacter sp. I3]
gi517599150	4.04	1	1	570	62.1	7.28	2.23	ABC transporter [Arthrobacter sp. 162MFSha1.1]
gi769943492	1.22	2	1	820	87.8	5.49	2.23	ATPase [Arthrobacter sp. IHBB 11108]
gi359306429	15.75	1	1	146	16.0	7.05	2.23	hypothetical protein ARGLB_038_00060 [Arthrobacter globiformis NBRC 12137]
gi910748988	45.45	1	1	44	4.6	10.95	2.23	hypothetical protein AHiyo8_pI66900 (plasmid) [Arthrobacter sp. Hiyo8]
gi515765015	2.74	1	1	438	49.3	6.57	2.23	peptidoglycan bridge formation protein FemAB [Arthrobacter sp. M2012083]
gi910739994	10.49	1	1	143	15.1	4.45	2.23	isoleucine--tRNA ligase [Arthrobacter sp. Hiyo4]
gi927031699	7.05	1	1	298	31.0	5.01	2.23	hypothetical protein AFL94_04535 [Arthrobacter sp. LS16]
gi939050870	16.78	1	1	149	16.2	7.20	2.23	hypothetical protein [Arthrobacter sp. JCM 19049]
gi910251462	6.64	1	1	241	27.2	6.96	2.23	hemerythrin [Arthrobacter siccitolerans]
gi219858521	8.12	1	1	271	27.8	4.73	2.23	HAD-superfamily hydrolase, subfamily IIB [Arthrobacter chlorophenolicus A6]
gi937256574	1.85	1	1	863	94.6	6.18	2.23	ATP-dependent DNA ligase [Arthrobacter sp. Edens01]
gi545110445	2.89	1	1	761	85.2	6.21	2.23	hypothetical protein [Arthrobacter sp. AK-YN10]
gi737807565	10.27	1	1	146	15.9	8.48	2.23	hypothetical protein [Arthrobacter sp. H5]
gi767257943	5.45	1	1	385	40.7	5.43	2.23	cystathionine gamma-synthase [Arthrobacter sp. IHBB 11108]
gi119948928	5.26	1	1	323	34.0	5.12	2.23	conserved hypothetical protein [Arthrobacter aurescens TC1]
gi651439976	14.13	2	1	92	10.6	9.79	2.23	transposase [Arthrobacter sp. H14]
gi307745453	12.05	1	1	166	18.0	7.65	2.23	putative GNAT-family acetyltransferase [Arthrobacter arilaitensis Re117]
gi651464281	3.41	1	1	352	37.6	5.07	2.23	peptidase M4 [Arthrobacter sp. 35/47]
gi640204053	3.74	1	1	401	41.4	4.84	2.23	hypothetical protein [Arthrobacter sp. 31Y]
gi759730858	3.41	1	1	558	59.0	6.11	2.23	ATPase [Arthrobacter sp. L77]
gi651491232	12.43	1	1	169	18.1	5.90	2.23	hypothetical protein [Arthrobacter sp. H20]

gi757623506	7.69	1	1	260	28.2	7.18	2.23	hypothetical protein TV39_15085 [Arthrobacter sp. SPG23]
gi742070879	4.13	1	1	363	37.3	5.06	2.23	hypothetical protein ANMWB30_20780 [Arthrobacter sp. MWB30]
gi823667925	0.99	1	1	1111	118.5	6.23	2.23	hypothetical protein AA310_04725 [Arthrobacter sp. YC-RL1]
gi740685590	2.92	1	1	479	50.9	5.53	2.23	aspartate ammonia-lyase [Arthrobacter sp. PAMC25486]
gi551255993	1.84	1	1	488	50.5	5.27	2.23	ferredoxin [Arthrobacter sp. PAO19]
gi737789865	5.88	1	1	323	34.8	5.02	2.23	ornithine carbamoyltransferase [Arthrobacter albus]
gi476403131	9.59	1	1	219	23.8	5.29	2.23	GntR family transcriptional regulator [Arthrobacter crystallopoietes BAB-32]
gi219861765	26.92	2	1	52	5.5	10.83	2.23	hypothetical protein Achl_4155 (plasmid) [Arthrobacter chlorophenolicus A6]
gi323467921	4.92	1	1	244	26.0	5.69	2.23	ANTAR/GAF domain-containing protein [Arthrobacter phenanthrenivorans Sphe3]
gi928487574	1.01	1	1	1382	151.2	5.55	2.23	AAA family ATPase [Arthrobacter alpinus]
gi651443721	2.23	1	1	494	53.1	5.96	2.23	F420-dependent methylene-tetrahydromethanopterin reductase [Arthrobacter sp. 9MFCol3.1]
gi359307412	3.58	1	1	587	62.1	9.13	2.23	hypothetical protein ARGLB_015_00400 [Arthrobacter globiformis NBRC 12137]
gi323471442	7.41	1	1	162	17.8	7.15	2.23	hypothetical protein Asphe3_40540 (plasmid) [Arthrobacter phenanthrenivorans Sphe3]
gi927294695	9.64	1	1	166	18.1	6.25	2.23	acetyltransferase [Arthrobacter sp. ERGS1:01]
gi759709704	4.77	1	1	398	43.0	5.38	2.23	acyl-CoA dehydrogenase [Arthrobacter sp. 9MFCol3.1]
gi757625669	7.27	1	1	289	31.9	5.41	2.23	hypothetical protein TV39_04720 [Arthrobacter sp. SPG23]
gi307743865	7.11	1	1	239	26.3	9.70	2.22	conserved hypothetical membrane protein [Arthrobacter arilaitensis Re117]
gi937262591	10.19	1	1	157	17.2	10.23	2.22	hypothetical protein AO716_00385 [Arthrobacter sp. Edens01]
gi908697992	4.22	1	1	332	34.6	6.70	2.22	oxidoreductase [Arthrobacter sp. RIT-PI-e]
gi443482454	6.17	1	1	162	17.4	4.94	2.22	PTS transporter subunit IIA-like nitrogen-regulatory protein PtsN [Arthrobacter nitrophenolicus]
gi476402300	16.25	1	1	80	9.4	4.83	2.22	hypothetical protein D477_004389 [Arthrobacter crystallopoietes BAB-32]
gi517605037	6.59	1	1	273	29.8	9.80	2.22	cell division protein FtsE [Arthrobacter sp. 131MFCol6.1]
gi910694642	12.17	1	1	189	20.1	11.47	2.22	conserved hypothetical protein [Arthrobacter sp. Hiyo6]
gi636846760	6.27	1	1	255	27.8	6.65	2.22	hypothetical protein [Arthrobacter sp. TB 26]
gi470215880	5.59	1	1	304	33.5	9.92	2.22	integrase catalytic protein [Arthrobacter gangotriensis Lz1y]
gi640733551	3.59	1	1	334	35.3	5.03	2.22	prephenate dehydratase [Leifsonia aquatica]
gi636844587	6.08	1	1	329	33.4	10.13	2.22	hypothetical protein [Arthrobacter sp. TB 26]
gi930827425	7.25	1	1	331	33.8	5.07	2.22	asparaginase [Arthrobacter arilaitensis]
gi652424140	3.75	1	1	480	53.1	9.86	2.22	hypothetical protein [Arthrobacter castelli]
gi443483297	5.17	1	1	174	17.1	5.21	2.22	hypothetical protein G205_00590 [Arthrobacter nitrophenolicus]
gi651441698	3.75	1	1	453	47.3	8.07	2.22	histidine kinase [Arthrobacter sp. 9MFCol3.1]
gi910744137	9.03	1	1	155	16.6	6.52	2.22	UPF0477 protein BPUM_1117 [Arthrobacter sp. Hiyo8]
gi737804054	3.52	1	1	454	47.2	5.16	2.22	FAD-linked oxidase [Arthrobacter sp. Br18]
gi476401503	5.77	1	1	312	32.5	5.16	2.22	glutamate racemase [Arthrobacter crystallopoietes BAB-32]
gi753939517	4.71	1	1	276	27.4	5.43	2.22	pyridine nucleotide-disulfide oxidoreductase [Arthrobacter phenanthrenivorans]
gi737800382	3.77	1	1	398	41.7	9.64	2.22	membrane protein [Arthrobacter castelli]
gi674645701	2.19	2	1	593	64.5	6.62	2.22	Type III restriction enzyme, res subunit [Arthrobacter sp. 11W110_air]
gi81295713	7.53	1	1	186	19.1	5.38	2.22	PIB-type ATPase, partial [Arthrobacter sp. FRC_X34]
gi917013321	8.94	1	1	235	26.0	6.14	2.22	hypothetical protein [Arthrobacter sanguinis]
gi759766001	5.48	1	1	365	37.8	5.68	2.22	alanine racemase [Arthrobacter gangotriensis]
gi759710464	3.42	1	1	556	60.5	7.27	2.22	ABC transporter [Arthrobacter sp. 135MFCol5.1]
gi910696022	13.10	1	1	145	16.0	7.59	2.22	conserved hypothetical protein [Arthrobacter sp. Hiyo6]
gi759731904	4.93	1	1	365	41.0	9.66	2.22	hypothetical protein [Arthrobacter sp. L77]
gi119948791	6.35	1	1	252	27.3	5.72	2.22	putative NADP oxidoreductase coenzyme F420-dependent family protein [Arthrobacter aurescens [illegible]
gi914717467	6.13	1	1	326	35.3	5.59	2.22	transcription elongation factor NusA [Arthrobacter sp. ZBG10]
gi908696788	4.63	1	1	324	37.0	4.87	2.22	ribonucleotide-diphosphate reductase subunit beta [Arthrobacter sp. RIT-PI-e]
gi937259618	4.86	1	1	370	40.7	5.03	2.22	enterotoxin [Arthrobacter sp. Edens01]
gi749401292	8.80	1	1	216	23.3	10.46	2.22	epimerase, partial [Arthrobacter sp. AK-YN10]
gi765013337	6.96	1	1	230	24.8	5.49	2.22	GntR family transcriptional regulator [Arthrobacter sp. A3]
gi765009547	2.95	1	1	678	69.6	5.11	2.22	ABC transporter [Arthrobacter sp. A3]
gi640203379	11.22	1	1	196	21.6	4.84	2.22	adenylate kinase [Arthrobacter sp. 31Y]
gi917991648	1.48	1	1	812	85.9	5.17	2.22	glycosyl hydrolase [Arthrobacter aurescens]
gi928488908	10.64	1	1	235	24.0	6.01	2.22	cobalamin biosynthesis protein CbiX [Arthrobacter alpinus]
gi654815072	3.46	1	1	318	33.7	5.19	2.22	hypothetical protein [Arthrobacter sp. PAO19]
gi654813709	4.77	2	1	461	50.8	6.49	2.22	mRNA 3'-end processing factor [Arthrobacter sp. MA-N2]
gi517591341	5.37	1	1	335	36.3	5.54	2.22	ATPase AAA [Arthrobacter sp. 135MFCol5.1]
gi403230202	4.59	1	1	523	54.2	9.77	2.22	putative integral membrane transport protein [Arthrobacter sp. Rue61a]
gi470221191	1.88	1	1	320	34.7	10.78	2.22	sugar ABC transporter ATP-binding protein [Arthrobacter gangotriensis Lz1y]
gi914714825	4.88	1	1	287	29.0	5.02	2.22	NAD(P)-dependent oxidoreductase [Arthrobacter sp. ZBG10]

gi818631408	2.64	1	1	569	61.6	6.18	2.22	hydrolase (plasmid) [Arthrobacter sp. 68b]
gi654811452	4.14	1	1	290	31.1	4.75	2.22	membrane protein [Arthrobacter sp. MA-N2]
gi928487922	1.71	1	1	993	104.1	9.50	2.22	cation:proton antiporter [Arthrobacter alpinus]
gi914715974	1.86	1	1	645	68.8	6.01	2.22	hypothetical protein [Arthrobacter sp. ZBG10]
gi910743099	3.07	1	1	391	41.6	6.96	2.22	arabinose metabolism transcriptional repressor [Arthrobacter sp. Hiyo8]
gi518313607	7.80	1	1	282	30.8	5.19	2.22	MULTISPECIES: UDP-glucose 4-epimerase [Arthrobacter]
gi916782284	4.87	1	1	493	51.9	5.25	2.22	aspartate ammonia-lyase [Arthrobacter sp. 35W]
gi916692000	3.67	1	1	327	36.5	5.57	2.22	aminoglycoside resistance protein [Arthrobacter castelli]
gi654812944	24.68	1	1	77	8.4	11.90	2.22	membrane protein [Arthrobacter sp. MA-N2]
gi403231341	10.69	1	1	159	17.6	7.06	2.22	putative transcriptional regulator, MarR family [Arthrobacter sp. Rue61a]
gi759704242	11.96	1	1	184	20.5	10.39	2.22	transposase, partial [Arthrobacter globiformis]
gi651503069	2.80	1	1	321	34.8	9.54	2.22	hypothetical protein [Arthrobacter sp. 35W]
gi759730532	4.43	1	1	451	46.6	4.97	2.22	phosphoglucosamine mutase [Arthrobacter sp. L77]
gi919219146	3.05	1	1	492	50.5	9.14	2.22	hypothetical protein [Arthrobacter sp. YC-RL1]
gi757623393	4.92	1	1	488	52.7	5.40	2.22	ribonuclease II [Arthrobacter sp. SPG23]
gi916357122	11.27	2	1	204	19.1	4.94	2.22	hypothetical protein [Arthrobacter sp. 162MFSha1.1]
gi910737972	5.00	1	1	380	41.3	7.69	2.22	DNA polymerase III subunit gamma/tau [Arthrobacter sp. Hiyo4]
gi937258049	3.01	1	1	399	42.5	8.70	2.22	hypothetical protein AO716_07990 [Arthrobacter sp. Edens01]
gi823667040	8.47	1	1	189	20.0	4.17	2.22	hypothetical protein AA310_15230 [Arthrobacter sp. YC-RL1]
gi910248615	2.11	1	1	662	70.3	6.61	2.22	hypothetical protein [Arthrobacter siccitolerans]
gi759718930	4.17	1	1	336	34.3	4.69	2.22	co-chaperone YbbN [Arthrobacter sp. FB24]
gi219862224	3.32	1	1	301	33.3	9.79	2.22	binding-protein-dependent transport systems inner membrane component (plasmid) [Arthrobacter
gi116612742	2.92	1	1	377	40.5	5.35	2.22	conserved hypothetical protein [Arthrobacter sp. FB24]
gi674644443	1.85	1	1	648	67.8	10.24	2.22	putative ABC transporter ATP-binding protein [Arthrobacter sp. 11W110_air]
gi515768331	4.48	1	1	424	45.2	9.39	2.22	glycosyl transferase family 1 [Arthrobacter sp. M2012083]
gi119948278	3.09	1	1	648	68.6	7.20	2.22	hypothetical protein AAur_3978 [Arthrobacter aurescens TC1]
gi910743999	5.88	1	1	238	27.0	12.21	2.22	hypothetical protein AHiyo8_17010 [Arthrobacter sp. Hiyo8]
gi910747723	16.18	1	1	136	14.3	9.86	2.22	conserved hypothetical protein [Arthrobacter sp. Hiyo8]
gi359307811	6.18	1	1	340	35.5	5.11	2.22	putative UDP-glucose 4-epimerase [Arthrobacter globiformis NBRC 12137]
gi908699244	6.93	1	1	202	22.3	6.54	2.22	TetR family transcriptional regulator [Arthrobacter sp. RIT-PI-e]
gi651485730	2.36	1	1	508	53.6	6.07	2.22	inosine-5-monophosphate dehydrogenase [Arthrobacter sp. Br18]
gi636845844	3.40	1	1	412	43.5	9.28	2.21	hypothetical protein [Arthrobacter sp. TB 26]
gi654814499	5.46	1	1	403	42.7	5.58	2.21	hypothetical protein [Arthrobacter sp. MA-N2]
gi403229543	3.90	1	1	333	35.9	5.10	2.21	reductase component (flavin-iron/sulfur protein) of ring-hydroxylating oxygenase system, phthalat
gi916813711	1.50	1	1	802	87.5	6.43	2.21	hypothetical protein [Arthrobacter nicotinovorans]
gi359307296	5.74	1	1	331	34.9	5.30	2.21	ornithine carbamoyltransferase [Arthrobacter globiformis NBRC 12137]
gi737786016	13.73	2	1	102	11.5	8.53	2.21	hypothetical protein [Arthrobacter nitrophenolicus]
gi910248908	5.48	1	1	310	31.2	5.63	2.21	threonine dehydratase [Arthrobacter siccitolerans]
gi518310969	4.72	1	1	318	36.0	6.23	2.21	ATPase AAA [Arthrobacter sp. TB 23]
gi927293634	3.05	1	1	426	45.4	4.68	2.21	enolase [Arthrobacter sp. ERGS1:01]
gi654818700	6.78	1	1	295	31.3	5.12	2.21	hypothetical protein [Arthrobacter sp. UNC362MFTsu5.1]
gi765006735	7.33	1	1	273	30.0	5.29	2.21	MerR family transcriptional regulator [Arthrobacter sp. A3]
gi654815117	3.74	1	1	401	43.8	5.02	2.21	acyl-CoA dehydrogenase [Arthrobacter sp. PAO19]
gi723608326	4.71	1	1	191	20.8	4.98	2.21	adenylate kinase [Arthrobacter sp. PAMC25486]
gi648574013	8.05	1	1	149	16.3	7.36	2.21	polyketide cyclase [Arthrobacter sp. 162MFSha1.1]
gi476401526	1.95	1	1	870	93.6	5.54	2.21	non-ribosomal peptide synthetase [Arthrobacter crystallopoietes BAB-32]
gi917441957	4.01	1	1	324	34.4	4.91	2.21	hypothetical protein [Arthrobacter albus]
gi651488652	14.58	1	1	96	10.5	4.91	2.21	hypothetical protein [Arthrobacter sp. H20]
gi742758614	5.73	1	1	262	29.1	7.52	2.21	hypothetical protein RM50_02920 [Arthrobacter phenanthrenivorans]
gi323470097	5.38	1	1	260	28.3	5.35	2.21	conserved hypothetical protein, DprA/Smf-related, family 1 TIGR00725/conserved hypothetical pro
gi551255987	2.01	1	1	498	52.7	5.38	2.21	dehydrogenase [Arthrobacter sp. PAO19]
gi211591794	2.70	1	1	481	52.0	5.10	2.21	Pc22g07580 [Penicillium rubens Wisconsin 54-1255]
gi928486981	10.94	1	1	128	14.4	5.39	2.21	transcriptional regulator [Arthrobacter alpinus]
gi937261836	3.22	1	1	528	55.7	5.64	2.21	MFS transporter [Arthrobacter sp. Edens01]
gi910738629	11.05	1	1	172	17.8	5.47	2.21	uncharacterized protein YbjT [Arthrobacter sp. Hiyo4]
gi910745117	4.32	1	1	347	36.2	10.11	2.21	probable endonuclease 8 2 [Arthrobacter sp. Hiyo8]
gi723606879	3.70	2	1	243	26.6	6.70	2.21	Dolichol-phosphate mannosyltransferase [Arthrobacter sp. PAMC25486]
gi908698098	6.01	1	1	333	35.8	7.84	2.21	hypothetical protein [Arthrobacter sp. RIT-PI-e]
gi116612624	19.39	1	1	98	10.5	9.69	2.21	transcriptional regulator, XRE family [Arthrobacter sp. FB24]

gi927032031	3.73	1	1	241	25.0	5.60	2.21	hypothetical protein AFL94_06560 [Arthrobacter sp. LS16]
gi542108100	2.86	1	1	315	32.4	5.99	2.21	ribokinase [Arthrobacter sp. AK-YN10]
gi648574406	7.75	1	1	258	28.2	7.40	2.21	IclR family transcriptional regulator [Arthrobacter sp. 162MFSha1.1]
gi307745336	2.30	1	1	566	58.2	9.77	2.21	LysM domain-containing protein [Arthrobacter arilaitensis Re117]
gi765006070	2.28	1	1	571	61.6	5.90	2.21	FAD-dependent oxidoreductase [Arthrobacter sp. A3]
gi937261719	6.60	1	1	197	22.0	8.16	2.21	hypothetical protein AO716_04900 [Arthrobacter sp. Edens01]
gi542106955	5.57	1	1	395	41.0	6.84	2.21	ArsR family transcriptional regulator [Arthrobacter sp. AK-YN10]
gi651503685	3.59	1	1	251	27.3	7.55	2.21	glutamate ABC transporter ATP-binding protein [Arthrobacter sp. 35W]
gi908697415	1.86	1	1	538	58.1	5.21	2.21	phytoene dehydrogenase [Arthrobacter sp. RIT-PI-e]
gi910742353	6.95	1	1	331	35.5	5.08	2.21	protein involved in chromosome condensation [Arthrobacter sp. Hiyo8]
gi651490364	2.25	1	1	578	61.5	4.92	2.21	preprotein translocase subunit SecD [Arthrobacter sp. H20]
gi917745797	4.83	1	1	290	30.6	5.67	2.21	nitrilase [Arthrobacter phenanthrenivorans]
gi908698556	15.79	1	1	95	10.7	12.09	2.21	hypothetical protein [Arthrobacter sp. RIT-PI-e]
gi651440626	6.99	1	1	186	20.6	5.76	2.21	deaminase/reductase [Arthrobacter sp. H14]
gi359307317	4.73	1	1	296	30.8	9.79	2.21	translation initiation factor IF-3 [Arthrobacter globiformis NBRC 12137]
gi737802543	5.72	2	1	402	40.4	11.58	2.21	MFS transporter [Arthrobacter castelli]
gi651429583	15.28	1	1	144	16.1	5.55	2.21	cytidyltransferase [Arthrobacter sanguinis]
gi918469533	10.77	1	1	195	20.8	6.39	2.21	acetyltransferase [Arthrobacter crystallopoietes]
gi640199823	6.51	1	1	307	31.5	5.16	2.21	ribokinase [Arthrobacter sp. 31Y]
gi765009530	2.06	1	1	678	72.7	6.83	2.21	ABC transporter [Arthrobacter sp. A3]
gi910738342	3.28	1	1	458	50.4	8.91	2.21	hypothetical protein AHiyo4_11640 [Arthrobacter sp. Hiyo4]
gi323470151	6.17	1	1	162	17.5	5.19	2.21	phosphotransferase system mannitol/fructose-specifc IIA component (Ntr-type) [Arthrobacter phe
gi737796670	8.54	1	1	246	27.9	7.96	2.21	hypothetical protein, partial [Arthrobacter sp. H20]
gi674645961	5.00	1	1	400	41.9	10.65	2.21	Phosphotransferase enzyme family protein [Arthrobacter sp. 11W110_air]
gi359303853	9.71	1	1	206	22.2	6.81	2.21	hypothetical protein ARGLB_095_00010, partial [Arthrobacter globiformis NBRC 12137]
gi518312443	4.62	1	1	368	39.3	9.88	2.21	hypothetical protein [Arthrobacter sp. TB 23]
gi908690634	2.52	1	1	595	64.1	5.55	2.21	peptide ABC transporter ATPase [Arthrobacter sp. H41]
gi119948921	2.67	1	1	450	47.9	5.05	2.21	integral membrane transporter with CBS domains [Arthrobacter aurescens TC1]
gi786027617	7.46	1	1	134	14.4	5.81	2.21	hypothetical protein [Arthrobacter chlorophenolicus]
gi937258050	2.91	2	1	825	87.2	9.14	2.21	hypothetical protein AO716_07995 [Arthrobacter sp. Edens01]
gi765009951	2.94	1	1	476	50.8	5.27	2.20	phenylacetaldehyde dehydrogenase [Arthrobacter sp. A3]
gi403230165	3.96	1	1	227	23.9	7.58	2.20	hypothetical protein ARUE_c26980 [Arthrobacter sp. Rue61a]
gi517589823	4.01	1	1	349	36.2	6.27	2.20	glycerol-3-phosphate dehydrogenase [Arthrobacter sp. 135MFCol5.1]
gi759732232	2.18	1	1	1236	130.9	5.03	2.20	urea carboxylase [Arthrobacter sp. L77]
gi654822296	7.34	1	1	109	11.6	9.28	2.20	hypothetical protein, partial [Arthrobacter sp. I3]
gi651457451	7.34	1	1	177	19.1	4.93	2.20	glyoxalase [Arthrobacter sp. 35/47]
gi914714684	6.84	1	1	190	19.6	5.08	2.20	hypothetical protein [Arthrobacter sp. ZBG10]
gi651433180	1.69	1	1	826	92.7	5.95	2.20	kojibiose phosphorylase [Arthrobacter sp. H41]
gi927295271	4.64	1	1	323	34.1	4.96	2.20	hypothetical protein AL755_16780 [Arthrobacter sp. ERGS1:01]
gi323470728	5.50	1	1	400	43.4	5.16	2.20	putative dehydrogenase [Arthrobacter phenanthrenivorans Sphe3]
gi651442482	4.60	1	1	457	46.4	8.66	2.20	NAD(P) transhydrogenase subunit beta [Arthrobacter sp. 9MFCol3.1]
gi937259522	7.78	1	1	257	27.8	6.44	2.20	glutamine ABC transporter ATP-binding protein [Arthrobacter sp. Edens01]
gi910248936	2.07	1	1	581	64.8	6.60	2.20	hypothetical protein [Arthrobacter siccitolerans]
gi742756080	7.77	1	1	193	20.8	9.64	2.20	thymidylate kinase [Arthrobacter phenanthrenivorans]
gi753932431	1.11	1	1	1079	115.8	6.90	2.20	hypothetical protein [Arthrobacter arilaitensis]
gi323468725	3.94	1	1	609	64.5	5.91	2.20	AMP-forming long-chain acyl-CoA synthetase [Arthrobacter phenanthrenivorans Sphe3]
gi908699195	6.05	1	1	248	25.9	8.47	2.20	DNA repair protein RecO [Arthrobacter sp. RIT-PI-e]
gi476402705	10.50	1	1	181	19.9	4.42	2.20	16S rRNA-processing protein RimM [Arthrobacter crystallopoietes BAB-32]
gi910696123	4.15	1	1	241	26.6	5.55	2.20	light-repressed protein A [Arthrobacter sp. Hiyo6]
gi636847282	6.51	1	1	215	21.8	5.58	2.20	short-chain dehydrogenase, partial [Arthrobacter sp. TB 26]
gi674646926	4.24	1	1	566	58.4	5.72	2.20	Sulfoacetaldehyde acetyltransferase [Arthrobacter sp. 11W110_air]
gi928486983	33.82	1	1	68	7.4	5.26	2.20	hypothetical protein AOC05_08300 [Arthrobacter alpinus]
gi760164720	3.49	1	1	516	54.3	5.02	2.20	hypothetical protein [Arthrobacter crystallopoietes]
gi764161701	4.57	1	1	219	24.5	5.34	2.20	hypothetical protein ArV1_100 [Arthrobacter phage vB_ArtM-ArV1]
gi518311503	4.82	1	1	228	24.8	5.50	2.20	hypothetical protein [Arthrobacter sp. TB 23]
gi517607354	1.93	1	1	518	58.0	5.55	2.20	glucose 6-phosphate dehydrogenase [Arthrobacter sp. 161MFSha2.1]
gi757624843	7.02	1	1	299	32.3	6.33	2.20	LysR family transcriptional regulator [Arthrobacter sp. SPG23]
gi928487649	4.76	1	1	399	40.5	6.07	2.20	acetyl-CoA acetyltransferase [Arthrobacter alpinus]
gi517592571	7.78	1	1	257	26.5	5.16	2.20	short-chain dehydrogenase [Arthrobacter sp. 135MFCol5.1]

gi742859140	2.72	1	1	478	50.3	5.31	2.20	flavoprotein [Arthrobacter sp. W1]
gi674645299	2.78	1	1	719	76.4	5.25	2.20	Prolyl tripeptidyl peptidase precursor [Arthrobacter sp. 11W110_air]
gi307743562	4.26	1	1	376	42.1	6.15	2.20	23S rRNA (uracil-5-)-methyltransferase RumB [Arthrobacter arilaitensis Re117]
gi517604347	6.00	1	1	300	31.3	9.83	2.20	membrane protein [Arthrobacter sp. 131MFCol6.1]
gi918266042	17.24	1	1	58	6.3	9.29	2.19	hypothetical protein AHiyo1_38780 [Arthrobacter sp. Hiyo1]
gi476400395	2.27	1	1	484	51.5	5.48	2.19	inosine 5-monophosphate dehydrogenase [Arthrobacter crystallopoietes BAB-32]
gi927271905	6.90	1	1	116	12.9	5.48	2.18	hypothetical protein [Arthrobacter sp. LS16]
gi767257662	2.29	1	1	349	35.6	11.05	2.18	hypothetical protein UM93_08525 [Arthrobacter sp. IHBB 11108]
gi674646241	2.90	1	1	414	42.7	8.79	2.17	Putative conjugal transfer proteinc/MT3759 [Arthrobacter sp. 11W110_air]
gi808662301	2.85	1	1	316	32.6	5.08	2.17	prephenate dehydratase [Mycobacterium europaeum]
gi162955748	5.26	1	1	190	20.4	4.31	2.17	Jag protein [Renibacterium salmoninarum ATCC 33209]
gi651506889	6.01	1	1	183	20.2	4.96	2.16	hypoxanthine phosphoribosyltransferase [Arthrobacter sp. 35W]
gi742755204	2.28	1	1	526	57.9	5.44	2.15	hypothetical protein RM50_14945 [Arthrobacter phenanthrenivorans]
gi403229144	3.01	2	1	299	31.9	9.39	2.15	hypothetical protein ARUE_c16540 [Arthrobacter sp. Rue61a]
gi723610209	1.30	1	1	846	87.0	5.73	2.14	hypothetical protein ART_3986 [Arthrobacter sp. PAMC25486]
gi517599818	3.15	1	1	381	40.4	5.34	2.14	ROK family transcriptional regulator [Arthrobacter sp. 162MFSha1.1]
gi551254432	9.62	2	1	104	11.8	8.57	2.13	hypothetical protein [Arthrobacter sp. PAO19]
gi759734770	4.86	1	1	247	25.6	5.80	2.12	glycerol transporter [Arthrobacter sp. L77]
gi916365773	2.29	1	1	349	38.3	8.15	2.11	glycosyl transferase [Arthrobacter sp. 161MFSha2.1]
gi757623121	5.65	1	1	230	24.7	8.06	2.10	response regulator receiver protein [Arthrobacter sp. SPG23]
gi908690405	7.74	1	1	168	17.3	6.79	2.10	hypothetical protein [Arthrobacter sp. H41]
gi916869950	13.10	1	1	84	8.6	9.98	2.08	hypothetical protein [Arthrobacter sp. Br18]
gi359305029	2.34	1	1	513	55.9	6.33	2.08	putative CdaR family transcriptional regulator [Arthrobacter globiformis NBRC 12137]
gi742072225	1.16	1	1	948	100.8	5.31	2.08	glycine dehydrogenase [Arthrobacter sp. MWB30]
gi742853602	2.62	1	1	381	40.9	4.88	2.08	molybdopterin biosynthesis protein MoeZ [Arthrobacter sp. W1]
gi918265197	11.11	1	1	63	7.5	10.86	2.07	hypothetical protein AHiyo1_49160 [Arthrobacter sp. Hiyo1]
gi636843818	3.28	1	1	305	33.7	5.03	2.06	thiosulfate sulfurtransferase [Arthrobacter sp. TB 26]
gi928485859	1.66	1	1	541	58.0	8.03	2.06	hypothetical protein AOC05_00535 [Arthrobacter alpinus]
gi651461899	5.13	1	1	195	20.5	7.28	2.06	hypothetical protein [Arthrobacter sp. 35/47]
gi654814695	2.39	1	1	377	38.8	7.80	2.06	hypothetical protein [Arthrobacter sp. MA-N2]
gi937258632	4.71	1	1	255	27.5	6.58	2.05	hypothetical protein AO716_11575 [Arthrobacter sp. Edens01]
gi765013283	2.25	1	1	445	47.3	7.03	2.05	UDP-N-acetylglucosamine 1-carboxyvinyltransferase [Arthrobacter sp. A3]
gi518311692	1.53	1	1	524	56.7	4.86	2.05	hypothetical protein [Arthrobacter sp. TB 23]
gi910742993	6.50	1	1	123	12.5	5.41	2.04	uncharacterized glycosyltransferase Mb0553 [Arthrobacter sp. Hiyo8]
gi760164404	19.35	1	1	62	6.9	8.24	2.04	hypothetical protein [Arthrobacter crystallopoietes]
gi910252063	3.02	1	1	298	33.4	7.30	2.04	sugar ABC transporter permease [Arthrobacter siccitolerans]
gi162953055	1.19	2	1	504	55.1	5.72	2.04	2-methylcitrate dehydratase [Renibacterium salmoninarum ATCC 33209]
gi910695645	2.56	1	1	508	55.1	5.71	2.03	glutathione-binding protein GsiB [Arthrobacter sp. Hiyo6]
gi908696984	7.32	1	1	164	17.9	5.60	2.03	hypothetical protein [Arthrobacter sp. RIT-PI-e]
gi651443918	2.38	1	1	504	52.4	5.91	2.03	histidine kinase [Arthrobacter nicotinovorans]
gi786027649	5.23	1	1	153	16.6	11.19	2.02	hypothetical protein [Arthrobacter chlorophenolicus]
gi674646365	2.29	1	1	262	27.8	10.78	2.01	Putative aliphatic sulfonates transport permease protein SsuC [Arthrobacter sp. 11W110_air]
gi476400768	1.82	1	1	439	47.1	4.25	2.01	family 1 extracellular solute-binding protein [Arthrobacter crystallopoietes BAB-32]
gi403229413	2.43	1	1	452	47.8	5.29	2.01	putative ABC transporter substrate-binding protein [Arthrobacter sp. Rue61a]
gi737813229	2.03	1	1	492	52.3	5.39	2.00	gamma-aminobutyraldehyde dehydrogenase [Arthrobacter sp. H14]
gi517602215	0.91	1	1	659	70.7	9.09	1.99	DNA-binding protein [Arthrobacter sp. 131MFCol6.1]
gi759735385	1.99	1	1	503	52.9	10.59	1.99	hypothetical protein [Arthrobacter sp. L77]
gi651473902	3.21	1	1	343	36.6	6.33	1.99	hypothetical protein [Arthrobacter nicotinovorans]
gi759734672	13.11	1	1	61	7.1	9.41	1.99	hypothetical protein [Arthrobacter sp. L77]
gi918268048	3.11	1	1	289	31.0	9.00	1.99	probable NADH dehydrogenase [Arthrobacter sp. Hiyo1]
gi765002997	4.85	1	1	165	18.5	7.43	1.98	MarR family transcriptional regulator [Arthrobacter sp. M2012083]
gi930828208	2.94	1	1	272	30.7	7.37	1.98	hypothetical protein AOZ07_13070 [Arthrobacter arilaitensis]
gi916781575	1.64	1	1	794	81.2	8.34	1.98	hypothetical protein [Arthrobacter sp. 35W]
gi765009820	3.70	1	1	243	27.0	5.24	1.97	hypothetical protein [Arthrobacter sp. A3]
gi917760168	1.85	1	1	487	52.6	6.90	1.97	hypothetical protein [Arthrobacter sp. L77]
gi651494945	1.17	1	1	511	55.6	6.02	1.97	2-methylcitrate dehydratase [Arthrobacter sp. H20]
gi737777952	0.92	1	1	653	73.8	7.24	1.97	hypothetical protein [Arthrobacter sanguinis]
gi551256757	4.41	1	1	204	22.9	6.27	1.97	nuclease PIN [Arthrobacter sp. PAO19]
gi651439742	0.89	1	1	895	98.7	4.92	1.97	valine--tRNA ligase [Arthrobacter sp. H14]

gi542110287	3.82	1	1	157	17.2	6.02	1.97	peroxiredoxin [Arthrobacter sp. AK-YN10]
gi517590850	1.60	1	1	501	51.5	9.13	1.96	uracil permease [Arthrobacter sp. 135MFCol5.1]
gi162955446	2.01	1	1	448	47.2	5.60	1.96	gamma-glutamyl phosphate reductase [Renibacterium salmoninarum ATCC 33209]
gi910697840	8.33	1	1	84	9.4	10.17	1.96	hypothetical protein AHiyo6_00360 [Arthrobacter sp. Hiyo6]
gi928487883	7.79	1	1	77	8.9	10.55	1.96	hypothetical protein AOC05_14290 [Arthrobacter alpinus]
gi116612496	9.52	1	1	126	13.5	9.99	1.96	hypothetical protein Arth_3845 [Arthrobacter sp. FB24]
gi162953714	1.13	1	1	885	97.1	5.35	1.96	chromosome partition protein [Renibacterium salmoninarum ATCC 33209]
gi307746002	1.89	1	1	318	33.8	5.17	1.95	formimidoylglutamase [Arthrobacter arilaitensis Re117]
gi517589833	3.64	1	1	275	28.3	5.95	1.95	hypothetical protein [Arthrobacter sp. 135MFCol5.1]
gi908697474	0.92	1	1	872	95.3	5.01	1.94	aminopeptidase [Arthrobacter sp. RIT-PI-e]
gi767258068	4.07	1	1	221	23.9	4.58	1.94	NUDIX hydrolase [Arthrobacter sp. IHBB 11108]
gi916782312	3.88	1	1	258	27.5	4.77	1.94	SDR family oxidoreductase [Arthrobacter sp. 35W]
gi927295606	2.36	1	1	339	36.3	5.11	1.93	2-hydroxyacid dehydrogenase [Arthrobacter sp. ERGS1:01]
gi917442147	3.90	1	1	205	21.8	4.49	1.93	hypothetical protein [Arthrobacter albus]
gi518311318	0.84	1	1	953	103.2	5.17	1.93	hypothetical protein [Arthrobacter sp. TB 23]
gi651435220	1.46	1	1	480	54.4	5.00	1.93	hypothetical protein [Arthrobacter sp. H41]
gi910694912	7.64	1	1	144	15.1	5.91	1.93	probable enoyl-CoA hydratase echA8, partial [Arthrobacter sp. Hiyo6]
gi737808596	2.39	1	1	293	31.3	8.62	1.93	ABC transporter permease [Arthrobacter sp. H5]
gi651461364	4.91	1	1	163	18.0	5.11	1.92	GNAT family N-acetyltransferase [Arthrobacter sp. 35/47]
gi403227691	6.98	1	1	86	8.9	9.77	1.92	hypothetical protein ARUE_c01720 [Arthrobacter sp. Rue61a]
gi910692753	4.20	1	1	262	27.7	6.43	1.92	methylcrotonoyl-CoA carboxylase beta chain, mitochondrial [Arthrobacter sp. Hiyo6]
gi910747208	3.49	1	1	229	25.0	10.29	1.92	hypothetical protein AHiyo8_49100 [Arthrobacter sp. Hiyo8]
gi910740830	8.28	1	1	145	16.1	10.62	1.92	50S ribosomal protein L22 [Arthrobacter sp. Hiyo4]
gi470220154	1.92	1	1	468	50.0	5.99	1.92	transcriptional regulator of the purine degradation operon [Arthrobacter gangotriensis Lz1y]
gi927295257	2.25	1	1	355	37.2	5.50	1.92	3-hydroxyisobutyryl-CoA hydrolase [Arthrobacter sp. ERGS1:01]
gi765010588	0.96	1	1	1041	105.6	5.31	1.92	protease [Arthrobacter sp. A3]
gi927295476	1.79	1	1	447	50.6	8.43	1.91	hypothetical protein AL755_18110 [Arthrobacter sp. ERGS1:01]
gi910283554	1.87	1	1	427	46.3	4.78	1.91	sugar ABC transporter substrate-binding protein [Arthrobacter sp. A3]
gi651460825	7.41	1	1	135	14.6	4.50	1.91	glyoxalase [Arthrobacter sp. 35/47]
gi654811302	7.97	2	1	138	14.7	6.00	1.91	hypothetical protein [Arthrobacter sp. MA-N2]
gi916869651	1.00	1	1	804	82.1	7.56	1.91	hypothetical protein [Arthrobacter sp. Br18]
gi690773541	3.80	1	1	184	20.2	9.41	1.91	hypothetical protein HMPREF2128_03095 [Arthrobacter albus DNF00011]
gi640193988	4.68	1	1	171	18.9	6.32	1.91	hypothetical protein [Arthrobacter sp. 31Y]
gi162955625	2.26	1	1	576	61.4	7.71	1.91	hypothetical protein RSal33209_3431 [Renibacterium salmoninarum ATCC 33209]
gi939036997	7.97	1	1	138	15.1	9.94	1.90	50S ribosomal protein L13 [Arthrobacter nitroguajacolicus]
gi737814292	4.93	1	1	142	15.5	5.14	1.90	GNAT family acetyltransferase [Arthrobacter sp. H14]
gi723607570	2.13	1	1	329	36.9	9.58	1.90	hypothetical protein ART_1347 [Arthrobacter sp. PAMC25486]
