# Supplementary material for: Effects of two kinds of imidazolium-based ionic liquids on the characteristics of steroid-transformation Arthrobacter simplex
Source: Microb Cell Fact. 2016 Jul 1;15:118. doi: 10.1186/s12934-016-0518-3 (PMC4930596; doi:10.1186/s12934-016-0518-3)
Supplement: Supplementary file 3 — 10.1186/s12934-016-0518-3 The identification of the [PrMIm]PF6-treated cells proteins. [file 12934_2016_518_MOESM3_ESM.pdf]

Additional file 3: The identification of the [PrMIm]PF_6-treated cells proteins

Accession	Coverage	PSMs	Peptides	AAs	MW [kDa]	calc. pI	Score	Description
gi672940878	63.35	245	23	543	56.5	4.86	838.19	chaperonin GroEL [Pimelobacter simplex]
gi672940874	52.57	123	21	622	65.8	4.68	459.85	molecular chaperone DnaK [Pimelobacter simplex]
gi939050621	30.40	53	4	125	13.0	4.48	188.06	50S ribosomal protein L7 [Arthrobacter sp. JCM 19049]
gi116610479	6.06	50	1	264	27.9	4.96	135.08	short-chain dehydrogenase/reductase SDR [Arthrobacter sp. FB24]
gi749402670	2.90	103	1	552	57.1	5.72	131.14	acetolactate synthase [Arthrobacter sp. AK-YN10]
gi219861633	25.37	130	2	67	7.2	4.88	123.23	cold-shock DNA-binding domain protein (plasmid) [Arthrobacter chlorophenolicus A6]
gi443482191	25.37	79	2	67	7.2	4.94	120.09	cold-shock DNA-binding protein family protein [Arthrobacter nitrophenolicus]
gi672940880	25.00	49	12	736	79.9	4.86	101.66	catalase/peroxidase KatG [Pimelobacter simplex]
gi742859324	7.49	189	1	307	33.6	7.06	100.51	LysR family transcriptional regulator [Arthrobacter sp. W1]
gi476402309	23.47	13	1	98	10.4	5.01	94.71	co-chaperonin GroES [Arthrobacter crystallopoietes BAB-32]
gi910737718	3.79	38	1	422	42.8	9.13	81.74	putative ankyrin-containing lipoprotein Lxx09580 [Arthrobacter sp. Hiyo4]
gi737783399	3.41	33	1	410	44.0	5.21	80.18	N-isopropylammelide isopropylaminohydrolase [Arthrobacter sp. 35W]
gi939050482	4.45	26	1	292	32.3	4.64	78.74	hypothetical protein [Arthrobacter sp. JCM 19049]
gi518311313	4.21	48	1	309	34.3	9.73	78.34	hypothetical protein [Arthrobacter sp. TB 23]
gi916835164	3.47	42	1	404	44.3	5.88	76.70	ABC transporter [Arthrobacter sp. H14]
gi737800623	5.67	28	1	300	32.6	5.43	76.06	SPFH/Band 7/PHB domain protein [Arthrobacter castelli]
gi918221850	2.42	24	1	619	69.0	7.36	72.29	glycosyltransferase [Arthrobacter sp. I3]
gi651495418	4.21	52	1	309	34.2	10.10	71.61	pseudouridine synthase [Arthrobacter sp. H20]
gi910747080	15.38	72	1	104	10.8	11.18	71.56	hypothetical protein AHiyo8_47820 [Arthrobacter sp. Hiyo8]
gi1906824	20.35	26	6	462	50.0	11.56	68.89	hypothetical protein [Pimelobacter simplex]
gi928488106	3.97	6	1	277	30.0	5.72	68.60	flagellar biosynthesis protein FliA [Arthrobacter alpinus]
gi914716454	13.10	9	5	542	56.7	4.87	68.45	molecular chaperone GroEL [Arthrobacter sp. ZBG10]
gi916813687	4.53	43	1	309	34.3	6.28	68.02	hypothetical protein [Arthrobacter nicotinovorans]
gi674647015	11.93	9	5	545	57.1	4.84	66.58	60 kDa chaperonin 1 [Arthrobacter sp. 11W110_air]
gi654823983	12.84	9	5	545	56.9	4.92	66.56	molecular chaperone GroEL [Arthrobacter sp. I3]
gi910738045	11.62	9	5	542	56.7	4.92	66.55	60 kDa chaperonin 1 [Arthrobacter sp. Hiyo4]
gi517602201	16.54	60	1	127	13.7	4.59	65.81	MULTISPECIES: glyoxalase [Arthrobacter]
gi651431914	2.35	5	1	597	64.4	4.67	63.91	ABC transporter substrate-binding protein [Arthrobacter sanguinis]
gi910743568	4.01	23	1	324	34.9	4.74	63.86	DNA repair protein RecN [Arthrobacter sp. Hiyo8]
gi674645160	6.39	23	1	360	37.6	5.14	60.03	putative enoyl-CoA hydratase [Arthrobacter sp. 11W110_air]
gi652425065	18.03	5	2	122	13.4	10.40	57.29	50S ribosomal protein L14 [Arthrobacter castelli]
gi403228123	4.14	28	1	338	36.2	5.59	54.81	glycine betaine/carnitine/choline transport ATP-binding protein OpuCA [Arthrobacter sp. Ru
gi654828077	11.57	16	4	536	56.6	4.98	50.50	molecular chaperone GroEL [Arthrobacter sp. H5]
gi823667653	3.50	23	1	400	43.1	4.96	50.09	ABC transporter [Arthrobacter sp. YC-RL1]
gi737786847	9.48	13	3	485	52.6	4.91	49.77	ATP synthase subunit beta [Arthrobacter albus]
gi674646068	12.77	18	2	235	24.7	9.38	49.62	50S ribosomal protein L1 [Arthrobacter sp. 11W110_air]
gi674646289	7.70	9	3	623	66.2	4.86	49.53	Chaperone protein DnaK [Arthrobacter sp. 11W110_air]
gi651437754	14.14	12	6	488	53.9	4.60	49.41	30S ribosomal protein S1 [Arthrobacter sp. H14]
gi674645978	8.21	16	3	536	56.1	4.97	49.17	60 kDa chaperonin 1 [Arthrobacter sp. 11W110_air]
gi651431165	13.76	13	3	487	53.0	4.84	47.26	ATP synthase subunit beta [Arthrobacter sanguinis]
gi742857336	3.70	8	3	1296	142.8	6.89	44.69	DNA-directed RNA polymerase subunit beta' [Arthrobacter sp. W1]
gi910747958	15.13	13	3	304	33.7	4.88	44.45	ATP synthase subunit beta [Arthrobacter sp. Hiyo8]
gi403231179	7.73	9	3	621	66.4	4.83	43.66	chaperone protein dnaK [Arthrobacter sp. Rue61a]
gi651495309	8.33	28	1	264	28.8	7.55	43.56	glycerophosphodiester phosphodiesterase [Arthrobacter sp. H20]
gi307745781	11.11	9	1	162	18.3	9.07	42.70	putative MarR-family transcriptional regulator [Arthrobacter arilaitensis Re117]
gi517601515	3.99	2	1	526	55.1	6.57	42.04	histidine kinase [Arthrobacter sp. 162MFSha1.1]
gi119949505	4.39	24	1	319	35.2	7.21	41.55	putative transcriptional regulator, LysR family [Arthrobacter aurescens TC1]
gi928985725	7.21	7	1	305	31.7	5.77	39.09	hypothetical protein [Arthrobacter sp. ERGS1:01]
gi651504238	4.46	12	4	1300	143.5	6.74	38.84	DNA-directed RNA polymerase subunit beta' [Arthrobacter sp. 35W]
gi648573957	7.08	6	1	226	25.1	6.70	38.52	fructose 2,6-bisphosphatase [Arthrobacter sp. 162MFSha1.1]
gi443480159	4.13	2	1	387	40.9	4.86	37.32	mannitol-1-phosphate 5-dehydrogenase [Arthrobacter nitrophenolicus]
gi767258484	7.47	21	5	830	91.6	5.55	37.05	NDP-hexose 4-ketoreductase [Arthrobacter sp. IHBB 11108]
gi636845165	5.46	22	1	293	31.7	5.15	36.41	aminoglycoside resistance protein, partial [Arthrobacter sp. TB 26]
gi654813020	6.09	8	3	739	79.7	5.15	36.36	isocitrate dehydrogenase [Arthrobacter sp. MA-N2]
gi742757319	3.99	1	1	526	54.9	6.29	36.34	histidine kinase [Arthrobacter phenanthrenivorans]
gi517601584	5.82	12	3	739	79.6	4.98	36.09	isocitrate dehydrogenase [Arthrobacter sp. 162MFSha1.1]
gi910741009	3.76	1	1	426	47.5	9.85	35.09	ICEBs1 integrase [Arthrobacter sp. Hiyo4]

gi517598428	5.79	3	1	328	34.8	5.21	35.03	O-succinylbenzoate synthase [Arthrobacter sp. 162MFSha1.1]
gi654823558	5.34	19	1	262	27.1	9.85	34.42	oxidoreductase [Arthrobacter sp. I3]
gi757623099	2.86	15	1	595	63.1	6.49	33.52	HNH endonuclease [Arthrobacter sp. SPG23]
gi551253706	2.76	1	1	435	44.9	5.50	32.94	glutamate-1-semialdehyde aminotransferase [Arthrobacter sp. PAO19]
gi910739216	5.88	12	1	221	25.4	5.77	32.48	conserved hypothetical protein [Arthrobacter sp. Hiyo4]
gi927294171	6.75	11	1	237	25.4	5.19	32.35	haloacid dehalogenase [Arthrobacter sp. ERGS1:01]
gi910746946	5.07	1	1	296	31.7	5.06	31.49	UTP--glucose-1-phosphate uridylyltransferase [Arthrobacter sp. Hiyo8]
gi767257874	21.70	7	3	235	24.9	9.25	30.89	50S ribosomal protein L1 [Arthrobacter sp. IHBB 11108]
gi939050890	6.43	17	5	824	91.0	5.74	30.88	NDP-hexose 4-ketoreductase, partial [Arthrobacter sp. JCM 19049]
gi651444347	3.59	2	1	390	42.5	5.11	30.68	exonuclease SbcD [Arthrobacter nicotinovorans]
gi651457048	14.93	7	1	134	15.0	9.06	30.44	30S ribosomal protein S16 [Arthrobacter sp. 35/47]
gi937259012	1.38	9	1	1158	123.6	5.27	30.29	1-pyrroline-5-carboxylate dehydrogenase [Arthrobacter sp. Edens01]
gi927031692	6.27	5	1	271	30.1	7.06	29.95	30S ribosomal protein S2 [Arthrobacter sp. LS16]
gi654818882	4.78	13	1	335	36.5	5.72	29.94	aminoglycoside resistance protein [Arthrobacter sp. UNC362MFTsu5.1]
gi639129536	8.86	22	2	237	24.9	5.58	29.89	copper oxidase [Arthrobacter sp. CAL618]
gi742758238	14.48	20	1	145	15.6	5.00	29.40	hypothetical protein RM50_04295 [Arthrobacter phenanthrenivorans]
gi470220257	12.54	6	3	335	36.1	4.78	29.31	DNA-directed RNA polymerase subunit alpha [Arthrobacter gangotriensis Lz1y]
gi757623381	5.91	5	2	491	50.1	8.51	29.31	protoporphyrinogen oxidase [Arthrobacter sp. SPG23]
gi654814371	4.22	10	1	474	50.2	6.06	29.15	aspartate ammonia-lyase [Arthrobacter sp. MA-N2]
gi910695627	6.54	7	1	260	27.3	8.07	29.09	UPF0001 protein Cgl2153/cg2364 [Arthrobacter sp. Hiyo6]
gi651437930	8.94	8	1	235	24.9	9.26	29.03	50S ribosomal protein L1 [Arthrobacter sp. H14]
gi917760505	1.38	1	1	508	53.5	9.42	28.43	3-methyladenine DNA glycosylase [Arthrobacter sp. L77]
gi651465980	14.04	7	3	235	25.0	9.36	28.40	50S ribosomal protein L1 [Arthrobacter sp. 35/47]
gi33571522	1.51	1	1	793	84.1	5.49	28.36	probable dehydrogenase/oxidase [Bordetella pertussis Tohama I]
gi636845393	6.22	8	3	739	79.4	5.06	28.31	isocitrate dehydrogenase [Arthrobacter sp. TB 26]
gi737815326	7.98	18	5	827	91.4	5.55	28.28	NDP-hexose 4-ketoreductase [Arthrobacter sp. H14]
gi651429323	12.58	11	1	159	17.8	8.06	28.10	hypothetical protein [Arthrobacter sanguinis]
gi651459247	5.82	16	3	739	79.8	4.98	28.05	isocitrate dehydrogenase [Arthrobacter sp. 35/47]
gi910737883	2.40	1	1	500	54.2	7.28	27.99	lipoprotein LipO [Arthrobacter sp. Hiyo4]
gi919218844	1.25	2	1	1116	125.2	5.47	27.98	hypothetical protein [Arthrobacter sp. YC-RL1]
gi470219995	4.56	6	2	614	66.1	4.74	27.54	chaperone protein DnaK [Arthrobacter gangotriensis Lz1y]
gi651440580	2.47	1	1	486	54.6	5.44	27.28	hypothetical protein [Arthrobacter sp. H14]
gi908740221	4.64	7	1	323	33.6	4.44	26.90	hypothetical protein [Arthrobacter arilaitensis]
gi742754769	5.35	5	2	598	63.1	5.19	26.72	acetyl-COA carboxylase [Arthrobacter phenanthrenivorans]
gi742752690	4.01	9	1	424	45.3	6.54	26.34	dehydrogenase [Arthrobacter phenanthrenivorans]
gi654826859	27.05	6	3	122	13.3	10.23	25.98	50S ribosomal protein L14 [Arthrobacter sp. H5]
gi918449287	2.10	1	1	905	99.8	5.67	25.37	hypothetical protein [Arthrobacter sp. SPG23]
gi470221278	2.77	3	1	470	48.7	7.71	25.29	hypothetical protein ADIAG_00131 [Arthrobacter gangotriensis Lz1y]
gi759724574	2.65	4	1	415	42.2	5.77	25.15	transcriptional regulator [Arthrobacter sp. I3]
gi674645749	1.14	6	1	1317	144.9	6.98	25.06	ATP-dependent RNA helicase HrpB [Arthrobacter sp. 11W110_air]
gi760112652	6.30	11	1	254	26.3	10.45	24.47	ABC transporter [Arthrobacter chlorophenolicus]
gi542106946	4.19	5	2	739	79.6	5.03	23.79	isocitrate dehydrogenase [Arthrobacter sp. AK-YN10]
gi917760260	5.24	5	1	191	21.2	6.00	23.74	hypothetical protein [Arthrobacter sp. L77]
gi119951743	23.40	7	1	94	10.2	8.54	23.65	conserved hypothetical protein (plasmid) [Arthrobacter aurescens TC1]
gi443479714	5.15	7	1	330	35.0	5.33	23.29	oxidoreductase, aryl-alcohol dehydrogenase like protein [Arthrobacter nitrophenolicus]
gi723610089	5.82	11	3	739	78.9	5.03	23.08	isocitrate dehydrogenase [Arthrobacter sp. PAMC25486]
gi723608358	1.71	6	2	1110	121.8	4.92	23.01	DNA-directed RNA polymerase subunit beta [Arthrobacter sp. PAMC25486]
gi651431596	8.53	6	1	129	13.3	4.54	22.68	50S ribosomal protein L7/L12 [Arthrobacter sanguinis]
gi323469187	4.78	6	1	230	24.5	5.27	22.51	conserved hypothetical protein TIGR00370 [Arthrobacter phenanthrenivorans Sphe3]
gi652425290	8.97	8	1	145	16.2	6.00	22.26	MarR family transcriptional regulator [Arthrobacter castelli]
gi470220234	10.04	13	2	279	30.6	11.33	21.38	50S ribosomal protein L2 [Arthrobacter gangotriensis Lz1y]
gi759715228	0.81	2	1	1847	202.0	5.21	21.31	hypothetical protein, partial [Arthrobacter sp. AK-YN10]
gi651487766	5.00	2	1	280	31.2	9.58	21.11	hypothetical protein [Arthrobacter sp. H20]
gi916782325	2.78	4	1	396	41.4	6.34	20.94	tRNA(Ile)-lysidine synthetase [Arthrobacter sp. 35W]
gi651435705	4.04	8	3	867	93.1	4.98	20.66	ATPase AAA [Arthrobacter sp. H41]
gi219860307	10.39	11	2	279	30.7	11.36	20.59	ribosomal protein L2 [Arthrobacter chlorophenolicus A6]
gi654818595	2.73	1	1	696	76.9	5.29	20.54	DNA gyrase subunit B [Arthrobacter sp. UNC362MFTsu5.1]
gi742857001	6.10	3	1	213	23.0	6.70	20.39	methyltransferase [Arthrobacter sp. W1]

gi930827440	2.66	4	1	451	50.9	5.82	20.37	hypothetical protein AOZ07_14910 [Arthrobacter arilaitensis]
gi545107909	6.42	4	2	545	58.9	5.11	20.29	ATP synthase subunit alpha [Arthrobacter sp. AK-YN10]
gi307746328	2.40	1	1	500	54.4	8.37	20.20	D-serine/D-alanine/glycine transporter [Arthrobacter arilaitensis Re117]
gi740683298	3.16	2	1	507	54.3	7.49	19.99	LuxR family transcriptional regulator [Arthrobacter sp. PAMC25486]
gi651435994	8.02	4	1	262	28.1	10.95	19.98	phosphatidic acid phosphatase [Arthrobacter sp. H41]
gi786028847	1.18	1	1	1190	128.5	5.26	19.76	chromosome segregation protein SMC [Arthrobacter chlorophenolicus]
gi759719843	3.37	6	3	1217	130.8	5.24	19.74	chromosome segregation protein SMC [Arthrobacter sp. FB24]
gi470221631	8.00	5	2	350	38.3	5.38	19.70	hypothetical protein ADIAG_00484 [Arthrobacter gangotriensis Lz1y]
gi918269053	6.27	9	1	255	26.4	4.94	19.59	glycosyl transferase, family 2 [Arthrobacter sp. Hiyo1]
gi652425173	3.47	5	1	634	69.9	4.68	19.32	hypothetical protein [Arthrobacter castelli]
gi648573277	3.73	6	1	456	48.1	9.88	19.23	3-phenylpropionic acid transporter [Arthrobacter sp. 135MFCol5.1]
gi930828168	5.46	1	1	458	47.8	5.27	19.10	hypothetical protein AOZ07_11020 [Arthrobacter arilaitensis]
gi651440931	1.47	4	1	1088	117.4	5.57	18.87	hypothetical protein [Arthrobacter sp. 9MFCol3.1]
gi759704530	2.40	4	1	499	54.3	5.74	18.75	hypothetical protein [Arthrobacter globiformis]
gi740685683	5.37	13	1	428	46.4	6.06	18.72	ATP-binding protein [Arthrobacter sp. PAMC25486]
gi116612717	4.38	2	1	479	51.6	5.58	18.71	dihydropyrimidinase [Arthrobacter sp. FB24]
gi476402260	3.05	8	1	492	52.0	5.53	18.65	ADP-ribosylation/crystallin J1 [Arthrobacter crystallopoietes BAB-32]
gi518313974	6.44	3	1	295	33.6	5.03	18.61	hypothetical protein [Arthrobacter sp. TB 23]
gi511534741	2.55	6	1	470	51.1	6.47	18.53	transcriptional activator pmfR (plasmid) [Arthrobacter nicotinovorans]
gi928485861	3.20	8	1	656	69.5	7.94	18.46	ABC transporter [Arthrobacter alpinus]
gi765009289	4.48	6	1	201	20.8	9.70	18.42	hypothetical protein [Arthrobacter sp. A3]
gi652422725	5.14	3	1	428	45.8	8.46	18.40	hypothetical protein [Arthrobacter castelli]
gi765009954	8.81	5	2	454	47.7	5.20	18.39	4-aminobutyrate aminotransferase [Arthrobacter sp. A3]
gi916781908	2.51	2	1	479	51.1	6.81	18.37	hypothetical protein [Arthrobacter sp. 35W]
gi518311951	6.39	1	1	219	23.2	6.64	18.36	MULTISPECIES: NUDIX domain-containing protein [Arthrobacter]
gi443480201	9.86	7	1	142	15.7	4.97	18.35	DNA-binding ferritin-like protein [Arthrobacter nitrophenolicus]
gi639129639	5.44	12	1	331	33.7	5.25	18.22	thiamine-monophosphate kinase [Arthrobacter sp. CAL618]
gi652424247	2.79	1	1	537	55.4	9.50	18.10	hypothetical protein [Arthrobacter castelli]
gi737790428	6.30	6	2	397	43.8	5.02	18.07	elongation factor Tu [Arthrobacter albus]
gi930826087	3.04	6	1	559	61.1	5.43	18.06	hypothetical protein AOZ07_07160 [Arthrobacter arilaitensis]
gi765009240	21.70	2	1	106	11.6	10.52	18.05	DNA-binding protein [Arthrobacter sp. A3]
gi517603447	4.26	5	1	399	40.6	5.97	18.05	acetyl-CoA acetyltransferase [Arthrobacter sp. 131MFCol6.1]
gi307745338	1.13	2	1	1597	177.7	5.29	18.04	NAD-specific glutamate dehydrogenase [Arthrobacter arilaitensis Re117]
gi517591758	5.76	2	1	191	20.2	5.01	18.03	phosphoribosylglycinamide formyltransferase [Arthrobacter sp. 135MFCol5.1]
gi723608975	8.37	5	1	203	21.6	7.58	17.95	transposase, IS111A/IS1328/IS1533 [Arthrobacter sp. PAMC25486]
gi551254729	12.65	14	1	166	18.4	5.81	17.94	hypothetical protein [Arthrobacter sp. PAO19]
gi742853148	0.90	3	1	1326	146.2	7.05	17.92	ATP-dependent helicase [Arthrobacter sp. W1]
gi765013182	4.77	7	2	524	55.8	9.14	17.88	signal recognition particle [Arthrobacter sp. A3]
gi654826881	8.77	7	2	399	42.5	7.05	17.87	pilus assembly protein CpaE [Arthrobacter sp. H5]
gi359307391	2.35	10	2	1150	126.2	5.96	17.83	hypothetical protein ARGLB_015_00190 [Arthrobacter globiformis NBRC 12137]
gi910251429	1.40	1	1	1002	108.6	6.46	17.81	hypothetical protein [Arthrobacter siccitolerans]
gi476400051	10.81	8	1	148	15.6	4.79	17.68	OsmC-like protein [Arthrobacter crystallopoietes BAB-32]
gi910697512	5.42	4	2	406	44.4	6.38	17.57	ATP synthase subunit alpha [Arthrobacter sp. Hiyo6]
gi654813160	6.64	5	2	602	63.3	5.03	17.54	acetyl-CoA carboxylase [Arthrobacter sp. MA-N2]
gi517610087	4.27	8	2	586	64.8	5.66	17.32	hypothetical protein [Arthrobacter sp. 161MFSha2.1]
gi757626101	8.81	4	1	159	17.6	5.01	17.25	DNA-binding protein [Arthrobacter sp. SPG23]
gi759728512	5.09	1	1	373	41.5	5.64	17.14	hypothetical protein [Arthrobacter sp. UNC362MFTsu5.1]
gi551254973	3.76	1	1	452	50.4	5.94	17.10	ATPase [Arthrobacter sp. PAO19]
gi651501550	3.07	4	2	685	72.8	6.98	17.01	primosomal protein N' [Arthrobacter sp. 35W]
gi917739479	1.31	4	1	1602	178.3	5.26	16.98	glutamate dehydrogenase [Arthrobacter sp. W1]
gi307743667	1.61	2	1	1181	128.1	6.34	16.91	putative conjugal transfer protein [Arthrobacter arilaitensis Re117]
gi116608862	5.97	3	1	268	28.6	9.35	16.85	ABC transporter related protein [Arthrobacter sp. FB24]
gi930827435	5.52	3	2	471	50.1	5.81	16.82	branched-chain alpha-keto acid dehydrogenase subunit E2 [Arthrobacter arilaitensis]
gi651441036	3.53	7	2	877	97.4	5.33	16.74	protein disaggregation chaperone [Arthrobacter sp. 9MFCol3.1]
gi757624876	3.56	1	1	365	39.0	5.02	16.68	mandelate racemase [Arthrobacter sp. SPG23]
gi219859783	2.35	1	1	426	46.3	5.21	16.66	ATP-dependent Clp protease, ATP-binding subunit ClpX [Arthrobacter chlorophenolicus A6]
gi737789559	2.53	2	1	513	55.6	9.54	16.65	signal recognition particle protein [Arthrobacter albus]
gi651455857	2.47	6	2	930	100.3	4.81	16.64	aconitate hydratase [Arthrobacter sp. 35/47]

gi674645236	13.24	2	2	204	21.0	11.77	16.44	Energy-coupling factor transporter transmembrane protein BioN [Arthrobacter sp. 11W110_
gi918469314	2.81	4	1	676	74.6	6.44	16.33	glycogen debranching protein [Arthrobacter crystallopoietes]
gi651453272	4.95	1	1	222	22.9	10.77	16.33	hypothetical protein [Arthrobacter nicotinovorans]
gi651443558	1.51	2	1	1058	116.2	5.76	16.28	cytochrome P450 [Arthrobacter sp. 9MFCol3.1]
gi651502464	1.48	1	1	1216	127.9	5.53	16.26	chromosome segregation protein SMC [Arthrobacter sp. 35W]
gi518311459	4.55	12	2	440	45.7	4.87	16.25	MULTISPECIES: hypothetical protein [Arthrobacter]
gi332743185	2.70	8	1	519	58.5	9.36	16.15	hypothetical protein STTU_0837 [Streptomyces sp. Tu6071]
gi910283858	1.72	2	1	816	86.4	7.65	16.15	transcription accessory protein [Arthrobacter sp. A3]
gi517591228	4.37	2	2	892	95.9	5.31	16.09	alanine--tRNA ligase [Arthrobacter sp. 135MFCol5.1]
gi757625673	4.38	2	2	640	71.6	5.68	16.04	hypothetical protein TV39_04745 [Arthrobacter sp. SPG23]
gi651504265	10.21	5	2	235	24.9	9.26	16.02	50S ribosomal protein L1 [Arthrobacter sp. 35W]
gi928486438	4.17	1	1	503	52.5	5.69	16.01	UDP-N-acetylmuramoyl-tripeptide--D-alanyl-D-alanine ligase [Arthrobacter alpinus]
gi476402465	2.55	9	2	942	105.1	8.06	15.98	superfamily II RNA helicase [Arthrobacter crystallopoietes BAB-32]
gi652424263	7.14	1	1	224	25.4	8.60	15.98	hypothetical protein [Arthrobacter castelli]
gi403231944	3.77	2	1	478	52.2	5.73	15.79	Xaa-Pro aminopeptidase (plasmid) [Arthrobacter sp. Rue61a]
gi551256381	4.26	23	1	517	55.2	8.24	15.79	hypothetical protein [Arthrobacter sp. PAO19]
gi737803727	3.94	2	1	330	37.0	9.10	15.78	transposase, partial [Arthrobacter sp. Br18]
gi518312892	25.37	9	1	67	7.2	4.88	15.76	MULTISPECIES: cold-shock protein [Arthrobacter]
gi759730827	2.18	3	1	689	73.9	6.86	15.72	hypothetical protein [Arthrobacter sp. L77]
gi476402832	13.82	1	1	152	17.1	11.62	15.72	50S ribosomal protein L20 [Arthrobacter crystallopoietes BAB-32]
gi674644663	1.75	1	1	913	95.0	8.47	15.69	hypothetical protein BN1051_00820 [Arthrobacter sp. 11W110_air]
gi916692460	1.37	3	1	873	98.4	6.24	15.61	hypothetical protein [Arthrobacter castelli]
gi765012240	1.29	4	1	1397	152.3	5.52	15.57	ATPase AAA [Arthrobacter sp. A3]
gi737809001	14.29	1	1	147	16.6	10.90	15.57	50S ribosomal protein L20 [Arthrobacter sp. H5]
gi910694411	9.94	5	1	171	18.9	4.87	15.56	ribosome maturation factor RimP [Arthrobacter sp. Hiyo6]
gi323468320	3.43	1	1	437	48.9	6.89	15.52	uncharacterized methicillin resistance protein [Arthrobacter phenanthrenivorans Sphe3]
gi908697114	5.84	3	1	291	28.8	10.64	15.47	glycosyl transferase family 9 [Arthrobacter sp. RIT-PI-e]
gi823667929	4.89	7	2	613	66.3	7.09	15.44	long-chain fatty acid--CoA ligase [Arthrobacter sp. YC-RL1]
gi119950023	4.51	8	2	488	54.6	6.86	15.41	cardiolipin synthetase [Arthrobacter aurescens TC1]
gi742070495	1.01	2	1	892	95.8	5.38	15.34	alanyl-tRNA synthetase [Arthrobacter sp. MWB30]
gi823666791	4.59	6	2	741	78.8	7.55	15.28	hypothetical protein AA310_13715, partial [Arthrobacter sp. YC-RL1]
gi651441437	8.05	5	2	348	36.9	7.02	15.26	reductase [Arthrobacter sp. 9MFCol3.1]
gi742753833	2.91	5	1	412	45.2	5.48	15.20	DNA polymerase [Arthrobacter phenanthrenivorans]
gi515767476	5.54	5	1	325	34.3	5.05	15.19	thioredoxin reductase [Arthrobacter sp. M2012083]
gi765005702	2.43	2	1	575	60.5	5.14	15.13	DNA repair protein RecN [Arthrobacter sp. A3]
gi737787955	3.30	1	1	696	78.7	4.97	15.07	excinuclease ABC subunit B [Arthrobacter albus]
gi737813222	9.70	1	1	165	18.1	9.86	15.05	hypothetical protein [Arthrobacter sp. H14]
gi767257639	1.81	1	1	1107	119.0	5.01	15.04	DNA helicase UvrD [Arthrobacter sp. IHBB 11108]
gi674645034	1.05	2	1	1147	118.6	6.61	14.95	ATP-binding/permease protein CydD [Arthrobacter sp. 11W110_air]
gi927031409	7.33	7	1	232	25.8	5.19	14.94	dihydrofolate reductase [Arthrobacter sp. LS16]
gi927293554	7.41	1	1	189	20.5	5.55	14.91	ACP phosphodiesterase [Arthrobacter sp. ERGS1:01]
gi742756220	2.22	1	1	856	90.1	6.80	14.90	histidine kinase [Arthrobacter phenanthrenivorans]
gi916863318	3.16	1	1	475	51.5	7.93	14.75	malate:quinone oxidoreductase [Arthrobacter sp. 35/47]
gi742859711	5.52	2	2	471	50.0	5.71	14.73	branched-chain alpha-keto acid dehydrogenase subunit E2 [Arthrobacter sp. W1]
gi651500415	0.79	1	1	881	90.4	7.88	14.71	helicase [Arthrobacter sp. 35W]
gi651430724	2.11	1	1	949	98.8	6.25	14.69	translation initiation factor IF-2 [Arthrobacter sanguinis]
gi307745596	13.62	6	3	279	30.5	11.33	14.66	50S ribosomal protein L2 [Arthrobacter arilaitensis Re117]
gi910249965	8.93	6	2	403	45.1	9.66	14.66	hypothetical protein [Arthrobacter siccitolerans]
gi927033186	2.22	9	1	810	86.5	6.24	14.63	transcription accessory protein [Arthrobacter sp. LS16]
gi767258477	3.56	4	2	506	55.7	9.89	14.59	membrane protein [Arthrobacter sp. IHBB 11108]
gi359304698	2.71	2	1	516	55.6	9.80	14.52	hypothetical protein ARGLB_080_00110 [Arthrobacter globiformis NBRC 12137]
gi737774031	1.45	3	1	830	89.9	9.28	14.47	hypothetical protein [Arthrobacter sp. MA-N2]
gi654811397	2.39	2	1	1005	107.1	5.21	14.46	chromosome segregation protein SMC [Arthrobacter sp. MA-N2]
gi551254533	7.33	2	1	232	25.7	4.98	14.45	DtxR family transcriptional regulator [Arthrobacter sp. PAO19]
gi443480104	1.48	1	1	945	99.3	6.99	14.45	P-type HAD superfamily ATPase [Arthrobacter nitrophenolicus]
gi651443967	9.79	4	2	235	24.9	9.25	14.44	50S ribosomal protein L1 [Arthrobacter nicotinovorans]
gi928486124	8.22	4	2	426	45.2	4.68	14.35	enolase [Arthrobacter alpinus]
gi742856514	5.24	4	1	229	25.1	5.19	14.34	hypothetical protein [Arthrobacter sp. W1]

gi737776995	5.39	4	1	297	30.9	5.78	14.33	NADH-ubiquinone oxidoreductase [Arthrobacter sanguinis]
gi927294653	3.16	2	1	601	63.2	8.95	14.32	preprotein translocase subunit SecD [Arthrobacter sp. ERGS1:01]
gi910248756	10.77	2	1	195	21.2	6.20	14.32	DNA-binding protein [Arthrobacter siccitolerans]
gi919218798	2.99	3	1	536	55.8	11.50	14.30	hypothetical protein [Arthrobacter sp. YC-RL1]
gi765010063	1.91	2	1	1150	122.1	5.16	14.30	1-pyrroline-5-carboxylate dehydrogenase [Arthrobacter sp. A3]
gi119951113	3.38	2	1	562	59.2	5.66	14.28	Formate--tetrahydrofolate ligase [Arthrobacter aurescens TC1]
gi914713438	6.93	3	1	202	21.9	8.32	14.26	TetR family transcriptional regulator [Arthrobacter sp. ZBG10]
gi910251186	8.25	3	1	206	22.5	9.82	14.25	CDP-diacylglycerol--glycerol-3-phosphate 3-phosphatidyltransferase [Arthrobacter siccitoler
gi742071460	3.70	2	1	405	41.9	5.12	14.24	molybdopterin cofactor synthesis protein moeA [Arthrobacter sp. MWB30]
gi651449859	4.88	3	1	328	34.5	4.79	14.22	malate dehydrogenase [Arthrobacter nicotinovorans]
gi939051484	4.11	1	1	438	49.3	7.09	14.14	ATPase [Arthrobacter sp. JCM 19049]
gi654819063	6.03	3	1	365	38.1	4.77	14.09	ABC transporter substrate-binding protein [Arthrobacter sp. UNC362MFTsu5.1]
gi927294255	12.28	2	2	342	36.2	5.67	14.06	epimerase [Arthrobacter sp. ERGS1:01]
gi119949883	1.76	3	2	1480	159.5	5.96	14.05	putative FtsK/SpoIIIE family protein [Arthrobacter aurescens TC1]
gi651467094	5.63	2	1	231	25.6	5.50	14.03	hypothetical protein [Arthrobacter sp. 35/47]
gi651479522	3.59	1	1	529	57.5	5.19	14.03	GTP-binding protein [Arthrobacter sp. Br18]
gi307745970	2.38	1	1	1049	115.2	6.49	14.00	putative ATP-dependent helicase [Arthrobacter arilaitensis Re117]
gi542107526	1.58	2	1	1074	118.4	5.92	13.98	hypothetical protein M707_16435 [Arthrobacter sp. AK-YN10]
gi914714494	4.64	2	2	539	58.2	4.93	13.98	ATPase [Arthrobacter sp. ZBG10]
gi652423466	1.79	1	1	560	60.5	8.46	13.95	DNA helicase [Arthrobacter castelli]
gi930826198	17.50	3	1	80	9.0	9.67	13.91	hypothetical protein AOZ07_07820 [Arthrobacter arilaitensis]
gi723607469	1.99	2	1	752	83.3	4.91	13.88	hypothetical protein ART_1246 [Arthrobacter sp. PAMC25486]
gi323468742	3.29	3	2	912	97.5	6.13	13.84	response regulator containing a CheY-like receiver domain and an HTH DNA-binding domai
gi651458664	1.18	2	1	1104	118.1	5.87	13.83	DNA helicase UvrD [Arthrobacter sp. 35/47]
gi917013410	6.13	8	1	163	17.1	10.29	13.81	50S ribosomal protein L15 [Arthrobacter sanguinis]
gi786028216	1.13	2	2	3528	369.8	5.43	13.80	non-ribosomal peptide synthetase [Arthrobacter chlorophenolicus]
gi476399394	8.86	1	1	237	26.7	4.91	13.76	TetR family transcriptional regulator [Arthrobacter crystallopoietes BAB-32]
gi910739807	2.42	2	1	496	54.9	5.50	13.72	peptidyl-dipeptidase dcp [Arthrobacter sp. Hiyo4]
gi651503276	2.49	4	1	563	60.6	5.74	13.71	peptide ABC transporter ATPase [Arthrobacter sp. 35W]
gi769942322	4.12	1	1	388	41.4	7.06	13.67	hypothetical protein [Arthrobacter sp. IHBB 11108]
gi654812960	2.77	1	1	794	84.5	6.90	13.66	transcription accessory protein [Arthrobacter sp. MA-N2]
gi517591624	4.78	2	1	230	24.8	4.81	13.64	GCN5 family acetyltransferase [Arthrobacter sp. 135MFCol5.1]
gi910747199	4.14	2	1	362	39.5	9.70	13.57	tyrosine recombinase XerC [Arthrobacter sp. Hiyo8]
gi517605103	3.11	5	1	482	50.5	8.50	13.53	phytoene dehydrogenase [Arthrobacter sp. 131MFCol6.1]
gi917739555	3.30	4	1	333	38.1	9.52	13.52	hypothetical protein [Arthrobacter sp. W1]
gi928487494	2.36	4	1	594	63.2	5.47	13.51	hypothetical protein AOC05_11905 [Arthrobacter alpinus]
gi640199055	2.96	1	1	473	49.7	5.95	13.49	amidase [Arthrobacter sp. 31Y]
gi928542246	4.37	2	1	481	52.8	5.16	13.46	portal protein [Arthrobacter phage Brent]
gi917022122	3.04	5	2	887	92.9	6.34	13.38	haloacid dehalogenase [Arthrobacter sp. UNC362MFTsu5.1]
gi545111228	4.59	10	1	436	47.5	5.43	13.38	tyrosine--tRNA ligase [Arthrobacter sp. AK-YN10]
gi652423476	7.64	4	2	445	46.1	4.97	13.37	glutamyl-tRNA reductase [Arthrobacter castelli]
gi765013208	1.74	3	2	1209	129.8	5.30	13.36	chromosome segregation protein SMC [Arthrobacter sp. A3]
gi651452660	3.15	2	2	953	106.0	5.88	13.36	helicase [Arthrobacter nicotinovorans]
gi323467642	2.18	4	1	458	49.4	5.05	13.35	NAD-dependent aldehyde dehydrogenase [Arthrobacter phenanthrenivorans Sphe3]
gi737790493	9.42	4	2	329	35.8	4.60	13.35	DNA-directed RNA polymerase subunit alpha [Arthrobacter albus]
gi910694769	5.38	1	1	316	35.1	5.26	13.34	conserved hypothetical protein [Arthrobacter sp. Hiyo6]
gi651439807	4.30	3	2	745	81.5	5.29	13.33	ATPase AAA [Arthrobacter sp. H14]
gi918268886	11.11	2	1	180	19.9	7.05	13.28	uncharacterized HTH-type transcriptional regulator PF1543 [Arthrobacter sp. Hiyo1]
gi635351753	2.75	2	1	654	70.1	6.27	13.25	1-deoxy-D-xylulose-5-phosphate synthase [Arthrobacter siccitolerans]
gi651464991	3.01	2	1	599	65.0	5.35	13.24	proline--tRNA ligase [Arthrobacter sp. 35/47]
gi651483475	1.39	8	1	1222	131.3	5.31	13.24	chromosome segregation protein SMC [Arthrobacter sp. Br18]
gi674646812	4.52	1	1	354	36.9	11.02	13.24	hypothetical protein BN1051_03024 [Arthrobacter sp. 11W110_air]
gi910738356	3.99	2	1	426	46.8	9.60	13.21	uncharacterized oxidoreductase y4hM [Arthrobacter sp. Hiyo4]
gi476400289	8.54	3	1	199	22.1	9.86	13.20	metal-dependent hydrolase [Arthrobacter crystallopoietes BAB-32]
gi737787422	4.71	6	1	297	31.5	5.22	13.19	pyridoxal biosynthesis lyase PdxS [Arthrobacter albus]
gi908698500	6.91	5	1	188	19.6	10.30	13.18	Holliday junction resolvase [Arthrobacter sp. RIT-PI-e]
gi652423331	23.47	2	1	98	10.4	4.75	13.18	molecular chaperone GroES [Arthrobacter castelli]
gi652425597	3.10	9	1	548	58.0	5.10	13.17	acetolactate synthase [Arthrobacter castelli]

gi910283668	6.19	3	1	291	30.4	6.40	13.13	hypothetical protein [Arthrobacter sp. A3]
gi517590415	5.81	2	1	482	50.7	6.21	13.12	hypothetical protein [Arthrobacter sp. 135MFCol5.1]
gi757624514	27.50	8	1	80	8.1	11.71	13.11	hypothetical protein TV39_09470 [Arthrobacter sp. SPG23]
gi518312740	8.99	2	2	345	36.0	5.71	13.10	hypothetical protein [Arthrobacter sp. TB 23]
gi517604861	3.08	6	1	552	59.6	6.30	13.08	elongation factor 3 [Arthrobacter sp. 131MFCol6.1]
gi737815013	2.96	2	1	439	49.8	5.12	13.02	glycine--tRNA ligase, partial [Arthrobacter sp. H14]
gi652424037	2.45	1	1	572	62.3	5.47	13.02	hypothetical protein [Arthrobacter castelli]
gi916782233	5.34	8	1	337	36.4	5.95	13.00	hypothetical protein [Arthrobacter sp. 35W]
gi918268509	5.88	10	1	340	38.5	7.80	13.00	trehalose-phosphate synthase [Arthrobacter sp. Hiyo1]
gi476402199	16.44	4	1	73	8.4	9.19	12.97	translation initiation factor IF-1 [Arthrobacter crystallopoietes BAB-32]
gi927294628	9.91	2	1	111	11.9	11.39	12.97	DNA-binding protein [Arthrobacter sp. ERGS1:01]
gi359306841	3.99	1	1	326	34.3	5.12	12.97	prephenate dehydratase [Arthrobacter globiformis NBRC 12137]
gi651466358	2.55	5	1	825	88.8	6.07	12.96	DNA topoisomerase IV subunit A [Arthrobacter sp. 35/47]
gi119949759	5.15	9	1	388	40.0	6.54	12.94	putative cysteine desulfurase [Arthrobacter aurescens TC1]
gi917441885	7.50	4	1	200	22.5	5.44	12.93	nicotinate-nicotinamide nucleotide adenylyltransferase [Arthrobacter albus]
gi742070313	6.49	3	1	308	33.3	9.67	12.87	tyrosine recombinase XerC [Arthrobacter sp. MWB30]
gi908690795	4.29	2	1	513	54.4	7.58	12.77	FAD-dependent oxidoreductase [Arthrobacter sp. H41]
gi742851604	2.60	1	1	576	61.4	5.12	12.76	phosphomannomutase [Arthrobacter sp. W1]
gi908697138	2.69	2	1	818	89.7	5.94	12.75	ATP-dependent DNA helicase PcrA [Arthrobacter sp. RIT-PI-e]
gi476399783	1.58	1	1	631	67.6	5.78	12.75	AMP-dependent synthetase and ligase [Arthrobacter crystallopoietes BAB-32]
gi910741425	6.97	2	1	201	21.8	5.14	12.74	acetyl-coenzyme A synthetase [Arthrobacter sp. Hiyo4]
gi476399452	4.55	1	1	154	16.8	6.80	12.73	AsnC family transcriptional regulator [Arthrobacter crystallopoietes BAB-32]
gi910283846	1.33	1	1	2036	210.7	4.94	12.73	hypothetical protein [Arthrobacter sp. A3]
gi443482594	0.74	1	1	1358	142.4	6.98	12.69	DNA segregation ATPase FtsK [Arthrobacter nitrophenolicus]
gi323469433	13.52	5	2	244	25.4	5.88	12.68	conserved hypothetical protein TIGR00046 [Arthrobacter phenanthrenivorans Sphe3]
gi908697779	2.10	1	1	903	100.8	5.29	12.59	preprotein translocase subunit SecA [Arthrobacter sp. RIT-PI-e]
gi648574929	6.05	2	2	496	53.4	8.21	12.56	hypothetical protein [Arthrobacter sp. 131MFCol6.1]
gi542109683	2.13	2	1	610	64.7	5.96	12.51	long-chain fatty acid--CoA ligase [Arthrobacter sp. AK-YN10]
gi906448305	1.66	2	1	1147	124.7	5.68	12.51	DNA helicase UvrD [Arthrobacter sp. RIT-PI-e]
gi517603440	11.49	4	2	235	24.8	9.25	12.48	MULTISPECIES: 50S ribosomal protein L1 [Arthrobacter]
gi742756082	9.94	5	2	312	34.6	6.81	12.47	LysR family transcriptional regulator [Arthrobacter phenanthrenivorans]
gi910249972	0.93	1	1	1617	180.0	5.19	12.47	glutamate dehydrogenase [Arthrobacter siccitolerans]
gi927032399	21.15	4	1	104	11.6	5.01	12.46	hypothetical protein AFL94_08865 [Arthrobacter sp. LS16]
gi119951924	0.71	1	1	1829	200.2	5.74	12.46	putative helicase (plasmid) [Arthrobacter aurescens TC1]
gi170783527	15.24	3	1	105	11.7	5.47	12.45	unknown (plasmid) [Arthrobacter sp. AK-1]
gi489899647	2.27	2	1	837	89.9	5.85	12.45	nitrite reductase large subunit [Arthrobacter globiformis]
gi654827932	4.77	4	1	503	52.6	6.02	12.43	L-aspartate oxidase [Arthrobacter sp. H5]
gi636843728	3.62	5	1	470	51.9	8.76	12.42	hypothetical protein [Arthrobacter sp. TB 26]
gi443480208	4.76	6	1	462	50.9	5.39	12.41	putative magnesium chelatase [Arthrobacter nitrophenolicus]
gi917759812	3.23	7	1	433	46.9	8.24	12.39	exodeoxyribonuclease VII large subunit [Arthrobacter sp. L77]
gi443482468	15.87	1	1	126	13.3	4.56	12.36	hypothetical protein G205_05866 [Arthrobacter nitrophenolicus]
gi916863384	2.22	4	2	1170	126.0	5.34	12.35	DNA helicase UvrD [Arthrobacter sp. 35/47]
gi674644816	1.49	4	1	737	78.9	4.93	12.33	Polyribonucleotide nucleotidyltransferase [Arthrobacter sp. 11W110_air]
gi927033501	2.41	4	1	457	48.3	4.77	12.29	succinate-semialdehyde dehydrogenase [Arthrobacter sp. LS16]
gi651468817	4.94	2	2	526	58.4	9.39	12.27	hypothetical protein [Arthrobacter nicotinovorans]
gi640199553	3.27	2	1	520	55.3	6.55	12.24	HNH endonuclease [Arthrobacter sp. 31Y]
gi737781111	14.95	2	2	214	23.1	10.32	12.22	CDP-diacylglycerol--glycerol-3-phosphate 3-phosphatidyltransferase [Arthrobacter sp. 35W]
gi651491342	3.01	1	1	565	61.4	6.84	12.21	3'-5' exonuclease [Arthrobacter sp. H20]
gi737786465	1.58	1	1	506	55.7	8.92	12.18	hypothetical protein [Arthrobacter albus]
gi930827823	10.08	2	1	258	26.8	4.60	12.18	electron transfer flavoprotein subunit beta [Arthrobacter arilaitensis]
gi654827948	1.28	2	2	1019	111.3	5.10	12.18	hypothetical protein [Arthrobacter sp. H5]
gi937262500	2.91	11	1	654	70.8	6.73	12.14	acyl-CoA dehydrogenase [Arthrobacter sp. Edens01]
gi765009334	4.06	3	1	493	53.6	5.25	12.14	ATPase [Arthrobacter sp. A3]
gi636844938	6.04	5	1	182	19.5	4.94	12.12	ATP synthase F0F1 subunit B [Arthrobacter sp. TB 26]
gi786034262	3.44	2	2	1162	119.6	9.10	12.11	ABC transporter [Arthrobacter chlorophenolicus]
gi937258268	3.10	1	1	419	46.1	5.33	12.09	hypothetical protein AO716_09275 [Arthrobacter sp. Edens01]
gi759730994	3.34	3	1	539	59.5	5.57	12.08	choline oxidase [Arthrobacter sp. L77]
gi651439153	10.26	5	2	341	37.2	4.92	12.07	ketol-acid reductoisomerase [Arthrobacter sp. H14]

gi639128949	3.44	1	1	378	40.0	7.58	12.06	secretion system protein E [Arthrobacter sp. CAL618]
gi753931683	3.14	2	1	477	50.7	4.73	12.04	phosphomannomutase [Arthrobacter arilaitensis]
gi470221086	17.95	1	1	78	8.9	9.32	12.03	hypothetical protein ADIAG_01038 [Arthrobacter gangotriensis Lz1y]
gi476401079	7.64	2	1	144	14.9	9.32	12.01	DoxX family protein [Arthrobacter crystallopoietes BAB-32]
gi119949708	3.81	1	1	394	43.0	5.20	12.00	oxidoreductase family, NAD-binding Rossmann fold domain protein [Arthrobacter aurescens
gi757625571	3.01	1	1	432	46.5	6.81	11.99	histidine kinase [Arthrobacter sp. SPG23]
gi651491763	1.98	1	1	1062	117.9	5.57	11.93	DEAD/DEAH box helicase [Arthrobacter sp. H20]
gi517590124	5.88	1	1	255	28.0	6.02	11.89	phosphatidylinositol kinase [Arthrobacter sp. 135MFCol5.1]
gi307745648	5.45	1	1	385	40.8	5.52	11.86	cystathionine gamma-synthase [Arthrobacter arilaitensis Re117]
gi517591898	5.42	2	1	295	31.1	8.07	11.83	DSBA oxidoreductase [Arthrobacter sp. 135MFCol5.1]
gi651497916	3.03	1	1	892	92.7	5.86	11.82	hypothetical protein [Arthrobacter sp. 35W]
gi742072447	6.60	3	1	197	19.5	5.87	11.82	lipoprotein [Arthrobacter sp. MWB30]
gi470221326	2.19	3	1	868	94.8	6.34	11.79	replicative DNA helicase [Arthrobacter gangotriensis Lz1y]
gi654818841	4.50	19	2	289	31.8	5.27	11.77	30S ribosomal protein S2 [Arthrobacter sp. UNC362MFTsu5.1]
gi116609487	3.37	3	2	830	90.4	6.29	11.74	ATP-dependent DNA helicase PcrA [Arthrobacter sp. FB24]
gi823666796	3.59	1	1	474	51.1	11.93	11.73	hypothetical protein AA310_13745 [Arthrobacter sp. YC-RL1]
gi737770920	7.24	4	1	304	31.4	5.06	11.73	sugar kinase [Arthrobacter sp. TB 26]
gi517598828	4.42	3	1	498	54.8	5.59	11.69	catalase [Arthrobacter sp. 162MFSha1.1]
gi916782327	1.07	1	1	1398	147.7	5.31	11.63	hypothetical protein [Arthrobacter sp. 35W]
gi654812862	2.57	2	1	778	84.5	6.06	11.62	hypothetical protein [Arthrobacter sp. MA-N2]
gi443481017	1.53	1	1	786	84.0	5.54	11.62	beta-galactosidase [Arthrobacter nitrophenolicus]
gi518310970	0.88	1	1	1584	170.2	7.49	11.61	hypothetical protein [Arthrobacter sp. TB 23]
gi654812277	2.21	2	1	860	93.7	8.09	11.61	ATP-dependent DNA ligase [Arthrobacter sp. MA-N2]
gi517598950	3.78	1	1	397	42.7	5.71	11.59	pilus biosynthesis protein CpaE [Arthrobacter sp. 162MFSha1.1]
gi651430051	3.17	2	1	473	52.7	6.27	11.58	plasmid replication-like protein [Arthrobacter sanguinis]
gi737811255	6.02	7	1	299	32.6	6.70	11.49	peptidase M24, partial [Arthrobacter sp. 35/47]
gi651488500	3.72	2	1	619	66.1	5.59	11.48	hypothetical protein, partial [Arthrobacter sp. H20]
gi767258220	6.42	2	1	218	23.0	4.63	11.45	hypothetical protein UM93_12380 [Arthrobacter sp. IHBB 11108]
gi517604620	1.40	1	1	1071	115.0	5.90	11.43	hypothetical protein [Arthrobacter sp. 131MFCol6.1]
gi476402589	1.41	1	1	852	92.4	5.25	11.42	alanyl-tRNA ligase, partial [Arthrobacter crystallopoietes BAB-32]
gi759709557	4.32	3	1	486	52.7	5.63	11.42	FAD-dependent oxidoreductase [Arthrobacter sp. 9MFCol3.1]
gi652424369	3.60	1	1	333	35.0	8.66	11.41	hypothetical protein [Arthrobacter castelli]
gi910737436	6.63	1	1	332	32.8	4.60	11.41	glycerate 2-kinase [Arthrobacter sp. Hiyo4]
gi928485980	4.55	1	1	330	36.0	5.36	11.41	hypothetical protein AOC05_01410 [Arthrobacter alpinus]
gi651440408	11.66	2	1	163	17.7	4.75	11.40	hypothetical protein [Arthrobacter sp. H14]
gi162953844	1.48	4	1	1626	180.7	5.14	11.35	NAD-specific glutamate dehydrogenase [Renibacterium salmoninarum ATCC 33209]
gi918221819	5.72	1	1	297	32.5	5.38	11.33	hypothetical protein [Arthrobacter sp. I3]
gi651431545	0.62	1	1	2901	297.6	4.32	11.33	hypothetical protein [Arthrobacter sanguinis]
gi930825394	9.85	2	1	132	13.8	5.80	11.32	hypothetical protein AOZ07_03145 [Arthrobacter arilaitensis]
gi651500864	4.49	1	1	312	33.1	5.69	11.31	aldo/keto reductase [Arthrobacter sp. 35W]
gi910741854	6.03	4	2	564	61.1	5.25	11.30	urocanate hydratase [Arthrobacter sp. Hiyo4]
gi651439827	24.62	4	2	130	14.9	11.22	11.30	50S ribosomal protein L20 [Arthrobacter sp. H14]
gi927294707	1.00	1	1	1098	121.7	5.06	11.29	isoleucine--tRNA ligase [Arthrobacter sp. ERGS1:01]
gi651435178	1.16	2	1	1384	149.2	6.80	11.28	hypothetical protein [Arthrobacter sp. H41]
gi651438494	5.32	1	1	263	28.7	6.16	11.25	alpha/beta hydrolase [Arthrobacter sp. H14]
gi551256519	2.34	2	1	856	92.3	6.61	11.23	DEAD/DEAH box helicase [Arthrobacter sp. PAO19]
gi551255739	2.50	2	1	640	66.4	10.15	11.23	hypothetical protein [Arthrobacter sp. PAO19]
gi654827486	4.21	3	1	404	43.7	4.73	11.18	ATP-binding protein [Arthrobacter sp. H5]
gi757622516	3.01	2	1	698	75.4	7.34	11.16	hypothetical protein TV39_20210 [Arthrobacter sp. SPG23]
gi928487926	4.53	1	1	397	40.8	9.23	11.15	potassium transporter [Arthrobacter alpinus]
gi723609756	3.03	1	1	694	74.4	6.01	11.13	xanthine dehydrogenase, molybdenum binding subunit apoprotein [Arthrobacter sp. PAMC
gi910745478	6.35	4	3	551	58.3	7.24	11.10	uncharacterized protein MJ0964 [Arthrobacter sp. Hiyo8]
gi937257990	4.90	2	2	715	78.3	7.25	11.08	hypothetical protein AO716_07600 [Arthrobacter sp. Edens01]
gi908698148	2.81	1	1	498	53.6	8.21	11.07	malate:quinone oxidoreductase [Arthrobacter sp. RIT-PI-e]
gi116612156	6.07	4	2	445	47.9	5.54	11.04	glutamate dehydrogenase (NADP) [Arthrobacter sp. FB24]
gi927031007	1.83	3	1	873	97.0	5.26	11.03	DNA gyrase subunit A [Arthrobacter sp. LS16]
gi914715448	7.92	2	2	404	44.8	9.86	11.01	hypothetical protein [Arthrobacter sp. ZBG10]
gi219861908	6.10	3	1	246	27.0	9.44	11.01	hypothetical protein Achl_4298 (plasmid) [Arthrobacter chlorophenolicus A6]

gi307744653	1.26	1	1	1663	183.6	5.36	11.00	hypothetical glycosyl transferase [Arthrobacter arilaitensis Re117]
gi908699232	2.68	6	2	1009	109.1	6.06	10.99	monooxygenase [Arthrobacter sp. RIT-PI-e]
gi640203116	1.83	1	1	600	65.7	6.79	10.98	dynein regulation protein LC7 [Arthrobacter sp. 31Y]
gi930827172	1.38	1	1	508	55.3	6.33	10.96	hypothetical protein AOZ07_13385 [Arthrobacter arilaitensis]
gi917530551	1.77	1	1	678	74.7	5.73	10.94	DNA topoisomerase IV subunit B [Arthrobacter sp. PAMC25486]
gi767259001	4.58	5	2	612	67.0	5.12	10.94	aspartyl-tRNA synthetase [Arthrobacter sp. IHBB 11108]
gi930827859	3.40	2	1	588	64.9	6.10	10.93	peptide synthetase [Arthrobacter arilaitensis]
gi651430001	4.42	2	1	339	36.1	5.07	10.91	fructose-bisphosphate aldolase [Arthrobacter sanguinis]
gi742856826	8.02	4	1	212	23.1	9.26	10.90	hypothetical protein [Arthrobacter sp. W1]
gi119951337	2.07	2	1	628	68.2	5.00	10.89	putative tetratricopeptide repeat family protein [Arthrobacter aurescens TC1]
gi914716317	5.07	6	2	611	68.0	5.82	10.87	hypothetical protein [Arthrobacter sp. ZBG10]
gi765009678	1.88	2	1	691	75.6	6.51	10.87	acyl-CoA dehydrogenase [Arthrobacter sp. A3]
gi654815234	13.45	1	1	119	12.8	6.80	10.86	cupin [Arthrobacter sp. PAO19]
gi542107238	4.87	2	2	637	72.6	8.94	10.86	glycosyltransferase [Arthrobacter sp. AK-YN10]
gi517605914	4.52	2	1	221	23.7	9.98	10.85	MULTISPECIES: hypothetical protein [Arthrobacter]
gi930825084	0.74	2	1	2302	243.4	5.12	10.85	hypothetical protein AOZ07_01405 [Arthrobacter arilaitensis]
gi476402266	6.94	2	2	173	19.4	6.70	10.84	hypothetical protein D477_004501 [Arthrobacter crystallopoietes BAB-32]
gi910696462	5.03	5	1	199	21.7	6.95	10.84	hhh-Gpd family protein [Arthrobacter sp. Hiyo6]
gi723608434	0.87	1	1	1150	121.9	5.45	10.81	proline dehydrogenase/pyrroline-5-carboxylate dehydrogenase [Arthrobacter sp. PAMC2548
gi517593198	7.81	2	2	461	52.3	5.54	10.79	glycine--tRNA ligase [Arthrobacter sp. 135MFCol5.1]
gi323469031	2.00	2	1	550	57.4	5.19	10.78	UDP-N-acetylmuramoylalanyl-D-glutamate--2,6-diaminopimelate ligase [Arthrobacter phena
gi323469212	2.95	3	1	713	78.1	5.55	10.78	DNA/RNA endonuclease G, NUC1 [Arthrobacter phenanthrenivorans Sphe3]
gi695210497	1.13	1	1	971	108.6	9.79	10.77	transposase (plasmid) [Arthrobacter aurescens]
gi651446345	1.15	5	1	954	101.2	5.80	10.77	cell division protein FtsK [Arthrobacter nicotinovorans]
gi927295142	1.45	1	1	1312	145.3	6.77	10.75	ATP-dependent helicase [Arthrobacter sp. ERGS1:01]
gi651498219	3.72	2	1	403	41.1	5.31	10.74	imidazolonepropionase [Arthrobacter sp. 35W]
gi930825452	7.77	4	1	283	30.8	6.60	10.74	hypothetical protein AOZ07_03515 [Arthrobacter arilaitensis]
gi723607626	1.17	1	1	1027	106.8	5.16	10.74	nuclease SbcCD subunit C [Arthrobacter sp. PAMC25486]
gi470216774	3.92	4	1	383	40.7	5.90	10.73	acetate kinase [Arthrobacter gangotriensis Lz1y]
gi737800969	0.80	1	1	1370	146.4	7.05	10.73	hypothetical protein [Arthrobacter castelli]
gi654811873	6.54	3	1	214	23.3	7.34	10.70	hypothetical protein [Arthrobacter sp. MA-N2]
gi767258179	0.89	2	1	2461	257.0	4.96	10.69	polyketide synthase [Arthrobacter sp. IHBB 11108]
gi674645579	8.62	3	2	116	13.2	10.18	10.69	50S ribosomal protein L19 [Arthrobacter sp. 11W110_air]
gi651434174	9.17	2	1	218	23.1	10.46	10.69	50S ribosomal protein L3 [Arthrobacter sp. H41]
gi551255613	3.46	1	1	665	71.0	5.38	10.69	ATP-dependent helicase [Arthrobacter sp. PAO19]
gi359306145	1.53	1	1	1045	112.4	6.19	10.66	putative transporter [Arthrobacter globiformis NBRC 12137]
gi380875451	2.28	2	1	702	77.4	6.44	10.66	alpha-galactosidase [Arthrobacter sp. GN14]
gi651504417	2.87	2	1	523	55.7	6.73	10.66	sodium:proton antiporter [Arthrobacter sp. 35W]
gi910697653	3.54	1	1	508	55.2	9.66	10.64	putative ribose/galactose/methyl galactoside import ATP-binding protein 2 [Arthrobacter sp
gi910283724	5.44	3	1	349	36.7	5.15	10.64	hypothetical protein [Arthrobacter sp. A3]
gi162953491	3.12	5	1	385	40.3	11.69	10.63	efflux ABC transporter, permease protein [Renibacterium salmoninarum ATCC 33209]
gi927032947	2.85	3	1	738	82.8	6.01	10.63	polyphosphate kinase [Arthrobacter sp. LS16]
gi917760016	4.19	1	1	477	46.6	4.86	10.63	hypothetical protein [Arthrobacter sp. L77]
gi219859433	23.29	2	1	73	7.6	8.97	10.59	conserved hypothetical protein [Arthrobacter chlorophenolicus A6]
gi937259069	1.52	2	1	657	70.4	6.38	10.58	1-deoxy-D-xylulose-5-phosphate synthase [Arthrobacter sp. Edens01]
gi307744672	2.38	1	1	588	63.7	5.50	10.57	conserved hypothetical membrane protein [Arthrobacter arilaitensis Re117]
gi823668494	1.64	1	1	974	108.2	7.91	10.57	transposase, partial [Arthrobacter sp. YC-RL1]
gi403311678	7.91	1	1	215	22.8	7.47	10.56	putative MobA-like protein (plasmid) [Arthrobacter sp. Rue61a]
gi517609727	1.12	6	1	1157	126.2	5.66	10.54	ATP-binding protein [Arthrobacter sp. 161MFSha2.1]
gi910250094	1.74	1	1	1033	110.0	5.19	10.52	chromosome segregation protein SMC [Arthrobacter siccitolerans]
gi930825080	3.96	5	1	480	53.2	5.08	10.52	hypothetical protein AOZ07_01385 [Arthrobacter arilaitensis]
gi759736473	1.93	4	1	880	92.0	7.21	10.52	hypothetical protein [Arthrobacter sp. L77]
gi918267307	12.42	6	1	161	16.8	9.04	10.51	ATP-dependent zinc metalloprotease FtsH [Arthrobacter sp. Hiyo1]
gi927292803	5.99	2	1	284	30.7	6.60	10.51	glutamine amidotransferase (plasmid) [Arthrobacter sp. ERGS1:01]
gi640203840	3.29	1	1	487	52.2	8.56	10.51	polysaccharide biosynthesis protein [Arthrobacter sp. 31Y]
gi515767693	2.71	3	1	627	67.4	5.33	10.51	glutamine amidotransferase [Arthrobacter sp. M2012083]
gi916357053	2.36	1	1	382	40.1	11.49	10.48	arsenic transporter [Arthrobacter sp. 162MFSha1.1]
gi551256335	1.31	1	1	1602	177.8	5.16	10.47	glutamate dehydrogenase [Arthrobacter sp. PAO19]

gi908690403	4.00	5	1	425	46.3	6.68	10.47	exodeoxyribonuclease VII large subunit [Arthrobacter sp. H41]
gi652425848	7.76	4	1	245	26.2	6.55	10.44	uridylate kinase [Arthrobacter castelli]
gi930827278	1.87	2	1	965	102.8	5.26	10.43	ferredoxin [Arthrobacter arilaitensis]
gi517599069	5.30	2	1	283	30.5	8.41	10.41	hypothetical protein [Arthrobacter sp. 162MFSha1.1]
gi517605729	3.54	1	1	508	54.4	6.96	10.39	ABC transporter substrate-binding protein [Arthrobacter sp. 131MFCol6.1]
gi651494016	1.42	1	1	702	76.8	7.66	10.37	DNA topoisomerase IV subunit B [Arthrobacter sp. H20]
gi651504005	5.00	3	1	300	32.5	5.12	10.35	UTP--glucose-1-phosphate uridylyltransferase [Arthrobacter sp. 35W]
gi908697824	5.32	2	1	376	39.6	5.57	10.35	GTPase [Arthrobacter sp. RIT-PI-e]
gi651430369	17.45	3	2	149	15.5	6.58	10.35	50S ribosomal protein L9 [Arthrobacter sanguinis]
gi651505517	5.57	1	1	287	31.4	9.69	10.34	LysR family transcriptional regulator [Arthrobacter sp. 35W]
gi930825637	1.02	1	1	783	84.4	6.07	10.34	hypothetical protein AOZ07_04560 [Arthrobacter arilaitensis]
gi767257943	5.45	1	1	385	40.7	5.43	10.33	cystathionine gamma-synthase [Arthrobacter sp. IHBB 11108]
gi765008661	2.04	1	1	636	66.9	4.83	10.31	NHL repeat-containing protein [Arthrobacter sp. A3]
gi908698536	3.92	2	1	434	46.2	5.05	10.31	tryptophan synthase subunit beta [Arthrobacter sp. RIT-PI-e]
gi654812977	4.32	1	1	324	35.6	5.39	10.31	aldo/keto reductase [Arthrobacter sp. MA-N2]
gi542108271	6.01	6	2	499	54.6	5.22	10.30	glycerol kinase [Arthrobacter sp. AK-YN10]
gi759711759	2.47	2	1	811	86.8	6.16	10.30	nitrite reductase large subunit [Arthrobacter sp. 162MFSha1.1]
gi767257382	9.38	5	2	341	37.1	4.94	10.27	ketol-acid reductoisomerase [Arthrobacter sp. IHBB 11108]
gi930825488	2.74	1	1	329	37.2	5.02	10.26	adenosine deaminase [Arthrobacter arilaitensis]
gi916781754	0.88	4	1	1257	138.5	5.83	10.26	helicase [Arthrobacter sp. 35W]
gi760112697	1.60	1	1	1190	128.3	5.30	10.25	chromosome segregation protein SMC [Arthrobacter chlorophenolicus]
gi403231582	2.21	1	1	816	88.3	7.77	10.24	serine/threonine-protein kinase PknK [Arthrobacter sp. Rue61a]
gi927033260	1.68	1	1	834	87.7	10.48	10.24	hypothetical protein AFL94_14130 [Arthrobacter sp. LS16]
gi786030360	1.78	2	1	897	97.6	7.01	10.23	LuxR family transcriptional regulator [Arthrobacter chlorophenolicus]
gi651503346	2.57	1	1	545	58.0	5.10	10.22	heme ABC transporter ATP-binding protein [Arthrobacter sp. 35W]
gi651442999	1.34	1	1	1194	135.0	6.11	10.22	hypothetical protein [Arthrobacter sp. 9MFCol3.1]
gi737765091	3.34	1	1	628	68.2	5.01	10.22	hypothetical protein [Arthrobacter sp. 161MFSha2.1]
gi476400956	8.33	3	1	144	15.6	4.88	10.21	DNA-binding protein [Arthrobacter crystallopoietes BAB-32]
gi654826824	5.36	4	1	280	30.0	9.85	10.20	hypothetical protein [Arthrobacter sp. H5]
gi917739560	2.82	5	1	567	62.8	5.87	10.19	AAA family ATPase [Arthrobacter sp. W1]
gi654814374	5.24	1	1	439	48.1	5.31	10.19	ATPase AAA [Arthrobacter sp. MA-N2]
gi930824883	3.82	1	1	471	50.4	9.28	10.17	MFS transporter [Arthrobacter arilaitensis]
gi651467158	2.08	3	1	721	78.6	6.61	10.15	transcription termination factor Rho [Arthrobacter sp. 35/47]
gi652424727	4.44	1	1	293	32.8	5.60	10.15	protein iolH [Arthrobacter castelli]
gi116611753	0.62	1	1	3524	372.4	5.31	10.13	amino acid adenylation domain protein [Arthrobacter sp. FB24]
gi737794692	2.41	1	1	746	82.0	5.74	10.13	ATPase AAA [Arthrobacter sp. PAO19]
gi759734840	3.91	1	1	486	51.9	6.00	10.11	ATPase AAA [Arthrobacter sp. L77]
gi918449458	1.27	1	1	1492	157.3	5.87	10.10	hypothetical protein, partial [Arthrobacter sp. SPG23]
gi767258769	2.73	3	1	770	83.8	6.39	10.09	hypothetical protein UM93_16375 [Arthrobacter sp. IHBB 11108]
gi651447330	9.15	4	1	164	17.8	7.05	10.08	CoA-binding protein [Arthrobacter nicotinovorans]
gi723607993	1.63	2	1	675	69.8	5.36	10.07	methylcrotonoyl-CoA carboxylase subunit alpha [Arthrobacter sp. PAMC25486]
gi515767124	4.48	1	1	268	28.2	4.65	10.06	hypothetical protein [Arthrobacter sp. M2012083]
gi916816031	3.44	1	1	291	30.8	8.43	10.03	hypothetical protein [Arthrobacter sp. MA-N2]
gi742757083	3.37	3	2	830	90.6	6.44	10.03	ATP-dependent DNA helicase PcrA [Arthrobacter phenanthrenivorans]
gi219857692	6.60	2	2	379	40.3	6.62	10.01	oligopeptide/dipeptide ABC transporter, ATPase subunit [Arthrobacter chlorophenolicus A6]
gi908699039	3.02	3	1	331	36.2	5.21	10.00	alpha-L-glutamate ligase [Arthrobacter sp. RIT-PI-e]
gi651434146	11.90	1	1	126	13.0	4.54	10.00	50S ribosomal protein L7/L12 [Arthrobacter sp. H41]
gi742852061	2.19	2	1	547	58.0	5.11	10.00	acetolactate synthase [Arthrobacter sp. W1]
gi767257607	2.46	5	1	406	44.2	4.94	10.00	oxidoreductase [Arthrobacter sp. IHBB 11108]
gi910743180	7.25	4	1	193	20.8	6.13	9.97	nickel-binding periplasmic protein [Arthrobacter sp. Hiyo8]
gi308229518	2.84	3	1	703	79.7	6.67	9.96	M.AciI [Arthrobacter citreus]
gi636846565	5.12	2	1	371	41.0	4.78	9.96	peptide chain release factor 2 [Arthrobacter sp. TB 26]
gi651491746	5.90	3	1	322	34.0	9.23	9.96	NADPH:quinone reductase [Arthrobacter sp. H20]
gi674645851	2.26	1	1	707	77.0	6.77	9.95	ATP-dependent DNA helicase UvrD2 [Arthrobacter sp. 11W110_air]
gi651481624	2.68	2	1	596	65.0	9.14	9.95	ABC transporter [Arthrobacter sp. Br18]
gi759747354	1.97	1	1	608	67.1	9.57	9.95	MarR family transcriptional regulator [Arthrobacter sp. 31Y]
gi910248826	3.12	1	1	673	73.3	9.13	9.94	hypothetical protein [Arthrobacter siccitolerans]
gi651431447	3.82	1	1	340	35.9	6.90	9.94	DNA polymerase III subunit delta [Arthrobacter sanguinis]

gi742756300	3.42	3	1	497	53.5	7.30	9.93	GTP-binding protein [Arthrobacter phenanthrenivorans]
gi917013222	3.01	1	1	565	59.5	6.71	9.93	hypothetical protein [Arthrobacter sanguinis]
gi162952293	2.55	1	1	548	59.0	6.68	9.91	putative two component sensor kinase [Renibacterium salmoninarum ATCC 33209]
gi767257750	5.42	3	2	535	57.0	5.08	9.90	GMP synthase [Arthrobacter sp. IHBB 11108]
gi648259962	3.42	1	1	380	40.0	9.17	9.90	glycosyltransferase [Arthrobacter sp. TB 23]
gi723608659	7.69	2	1	247	24.9	5.08	9.90	hypothetical protein ART_2436 [Arthrobacter sp. PAMC25486]
gi737793085	22.86	3	1	70	7.3	6.00	9.86	hypothetical protein [Arthrobacter nicotinovorans]
gi476398905	22.55	5	1	102	11.4	5.05	9.86	hypothetical protein D477_021043 [Arthrobacter crystallopoietes BAB-32]
gi517593626	3.76	3	1	213	23.3	5.58	9.85	hypothetical protein [Arthrobacter sp. 135MFCol5.1]
gi219861268	10.29	2	1	136	14.7	6.95	9.83	monovalent cation/proton antiporter, MnhG/PhaG subunit [Arthrobacter chlorophenolicus A
gi470220703	7.96	1	1	201	22.3	9.54	9.81	sugar transferase [Arthrobacter gangotriensis Lz1y]
gi737813326	3.47	5	1	432	47.5	4.65	9.81	HCC family HlyC/CorC transporter [Arthrobacter sp. H14]
gi767259019	4.89	1	1	225	24.1	5.30	9.81	HxlR family transcriptional regulator [Arthrobacter sp. IHBB 11108]
gi651485181	2.82	1	1	496	52.1	4.81	9.81	peptidase M23 [Arthrobacter sp. Br18]
gi760125571	1.30	1	1	614	68.5	9.36	9.80	MarR family transcriptional regulator [Arthrobacter aurescens]
gi916820388	1.53	3	1	1108	119.8	10.13	9.80	hypothetical protein [Arthrobacter sp. H20]
gi489902116	2.77	2	2	974	107.1	6.90	9.80	ABC-ATPase UvrA [Arthrobacter globiformis]
gi651431530	10.89	4	1	101	11.1	10.11	9.80	50S ribosomal protein L23 [Arthrobacter sanguinis]
gi654824970	3.19	1	1	282	30.2	9.60	9.80	SDR family oxidoreductase [Arthrobacter sp. I3]
gi359307048	2.43	1	1	452	46.6	5.30	9.79	D-beta-D-heptose 7-phosphate kinase/D-beta-D-heptose 1-phosphate adenosyltransferase
gi116609878	1.08	1	1	831	87.5	9.99	9.79	transglutaminase domain protein [Arthrobacter sp. FB24]
gi219860176	2.46	5	1	406	45.1	5.68	9.78	aminotransferase class I and II [Arthrobacter chlorophenolicus A6]
gi542106899	2.92	3	1	308	32.6	10.21	9.77	membrane protein [Arthrobacter sp. AK-YN10]
gi767256644	4.49	1	1	535	57.5	5.40	9.77	methylcrotonoyl-CoA carboxylase [Arthrobacter sp. IHBB 11108]
gi307743859	0.90	4	1	1108	121.1	5.87	9.77	conserved hypothetical protein [Arthrobacter arilaitensis Re117]
gi765012797	2.53	1	1	791	87.9	5.55	9.76	hypothetical protein [Arthrobacter sp. A3]
gi816850540	9.71	1	1	206	23.3	5.45	9.75	superoxide dismutase [Pimelobacter simplex]
gi937261769	4.20	2	2	834	87.9	5.41	9.74	histidine kinase [Arthrobacter sp. Edens01]
gi918449310	0.61	1	1	1795	198.5	6.83	9.74	hypothetical protein [Arthrobacter sp. SPG23]
gi919218764	1.04	1	1	1540	160.7	5.27	9.73	hypothetical protein [Arthrobacter sp. YC-RL1]
gi651442237	2.93	2	1	580	62.7	6.18	9.73	multidrug ABC transporter ATPase [Arthrobacter sp. 9MFCol3.1]
gi228841151	1.58	1	1	634	70.8	8.22	9.73	Peptidase M23B [Bacillus thuringiensis serovar pulsiensis BGSC 4CC1]
gi939051050	4.39	2	1	456	49.6	7.42	9.73	hypothetical protein, partial [Arthrobacter sp. JCM 19049]
gi908697065	4.16	1	1	409	43.6	5.60	9.72	hypothetical protein [Arthrobacter sp. RIT-PI-e]
gi651493150	2.76	1	1	362	40.8	9.32	9.72	hypothetical protein [Arthrobacter sp. H20]
gi307744934	2.78	8	1	612	67.5	5.41	9.71	GTP-binding elongation factor LepA [Arthrobacter arilaitensis Re117]
gi910743557	2.46	1	1	692	75.6	6.86	9.71	citrate synthase [Arthrobacter sp. Hiyo8]
gi470217502	9.28	4	1	194	20.9	9.69	9.70	serine O-acetyltransferase [Arthrobacter gangotriensis Lz1y]
gi918221824	8.39	2	1	143	15.0	4.68	9.70	hypothetical protein [Arthrobacter sp. I3]
gi162953457	0.93	1	1	1073	116.1	5.39	9.69	ATP-dependent DNA helicase [Renibacterium salmoninarum ATCC 33209]
gi652425039	1.50	2	1	936	96.9	6.51	9.69	hypothetical protein [Arthrobacter castelli]
gi651445111	1.48	1	1	1283	131.2	6.84	9.68	hypothetical protein [Arthrobacter nicotinovorans]
gi674646607	5.82	1	1	189	20.3	5.26	9.68	Bacterial regulatory proteins, tetR family [Arthrobacter sp. 11W110_air]
gi910748105	7.39	1	1	203	21.6	4.92	9.68	3-isopropylmalate dehydratase large subunit [Arthrobacter sp. Hiyo8]
gi737810562	2.45	1	1	530	56.6	4.28	9.67	ABC transporter substrate-binding protein [Arthrobacter sp. 35/47]
gi654827558	5.73	1	1	314	35.1	9.58	9.63	hypothetical protein [Arthrobacter sp. H5]
gi323470062	5.64	2	2	443	45.9	5.22	9.63	glutamate-1-semialdehyde 2,1-aminomutase [Arthrobacter phenanthrenivorans Sphe3]
gi916259816	9.32	2	1	236	24.6	6.20	9.59	2-deoxy-D-gluconate 3-dehydrogenase [Arthrobacter sp. TB 23]
gi918266287	4.73	2	2	804	90.2	6.46	9.59	conserved hypothetical protein [Arthrobacter sp. Hiyo1]
gi765002568	2.44	1	1	616	66.9	5.82	9.58	isoniazid-inducible protein iniA [Arthrobacter sp. M2012083]
gi737790465	8.99	1	1	189	21.5	9.39	9.57	50S ribosomal protein L5 [Arthrobacter albus]
gi517609543	1.88	3	1	531	57.0	8.68	9.56	ATP/GTP-binding protein [Arthrobacter sp. 161MFSha2.1]
gi908698426	2.90	1	1	518	57.5	5.21	9.54	GTP-binding protein EngA [Arthrobacter sp. RIT-PI-e]
gi759723145	6.90	1	1	232	24.3	5.21	9.53	SDR family oxidoreductase [Arthrobacter sp. I3]
gi760112884	10.14	2	1	207	22.0	5.39	9.53	DNA-binding protein [Arthrobacter chlorophenolicus]
gi476399356	4.90	4	1	245	26.3	10.65	9.52	oligopeptide ABC transporter ATPase [Arthrobacter crystallopoietes BAB-32]
gi908698542	2.98	1	1	436	48.4	8.95	9.52	glycosyl transferase [Arthrobacter sp. RIT-PI-e]
gi674646145	3.99	2	1	451	47.5	5.21	9.52	5-aminovalerate aminotransferase DavT [Arthrobacter sp. 11W110_air]

gi674645232	2.65	3	1	491	54.7	5.00	9.51	FeS cluster assembly protein SufB [Arthrobacter sp. 11W110_air]
gi652422578	6.59	3	1	349	36.7	9.23	9.50	hypothetical protein [Arthrobacter castelli]
gi742758102	6.05	3	1	347	38.0	6.06	9.49	tryptophanyl-tRNA synthetase [Arthrobacter phenanthrenivorans]
gi651440268	2.15	1	1	512	54.6	4.59	9.48	hypothetical protein [Arthrobacter sp. H14]
gi359306522	3.46	1	1	405	45.2	10.07	9.46	putative transposase [Arthrobacter globiformis NBRC 12137]
gi403228817	3.15	1	1	444	46.1	5.59	9.45	NAD(P)H dehydrogenase (quinone) [Arthrobacter sp. Rue61a]
gi769939789	5.41	1	1	314	33.7	7.28	9.45	hypothetical protein [Arthrobacter sp. IHBB 11108]
gi757624850	6.15	10	1	325	34.9	9.33	9.45	hypothetical protein TV39_09305 [Arthrobacter sp. SPG23]
gi651430391	2.77	6	1	361	39.4	4.91	9.44	inositol-3-phosphate synthase [Arthrobacter sanguinis]
gi476401595	5.75	3	1	313	33.3	10.04	9.44	Flp pilus assembly protein TadB [Arthrobacter crystallopoietes BAB-32]
gi651442843	1.33	1	1	977	106.7	7.02	9.44	RNA helicase [Arthrobacter sp. 9MFCol3.1]
gi908698559	1.24	1	1	1612	172.9	6.55	9.42	hypothetical protein [Arthrobacter sp. RIT-PI-e]
gi219860904	8.57	1	1	140	14.5	6.16	9.42	UspA domain protein [Arthrobacter chlorophenolicus A6]
gi917021998	4.17	4	1	551	57.0	11.30	9.41	hypothetical protein [Arthrobacter sp. UNC362MFTsu5.1]
gi908697456	5.10	1	1	196	21.9	5.47	9.40	hypothetical protein [Arthrobacter sp. RIT-PI-e]
gi551253735	7.05	3	1	298	32.2	9.89	9.39	glutamate ABC transporter permease [Arthrobacter sp. PAO19]
gi470220534	6.41	3	1	312	34.5	8.79	9.38	glycosyltransferase [Arthrobacter gangotriensis Lz1y]
gi119948996	2.72	2	1	551	58.9	9.11	9.37	putative peptide ABC transporter, ATP-binding protein [Arthrobacter aurescens TC1]
gi737804784	3.05	2	1	328	35.5	9.80	9.36	methicillin resistance protein [Arthrobacter sp. Br18]
gi651430884	1.37	1	1	656	72.9	5.14	9.35	WYL domain-containing protein [Arthrobacter sanguinis]
gi651492871	3.13	2	1	607	66.2	6.64	9.34	peptide ABC transporter ATPase [Arthrobacter sp. H20]
gi919219195	1.53	1	1	522	57.7	6.20	9.34	transcriptional regulator, CdaR [Arthrobacter sp. YC-RL1]
gi910695374	5.29	1	1	378	40.0	5.26	9.34	non-hemolytic phospholipase C [Arthrobacter sp. Hiyo6]
gi518312673	2.60	1	1	385	42.0	5.26	9.32	hypothetical protein [Arthrobacter sp. TB 23]
gi910742452	2.55	1	1	548	59.9	6.43	9.31	hypothetical protein AHiyo8_01540 [Arthrobacter sp. Hiyo8]
gi639130865	1.23	1	1	1139	119.8	5.03	9.30	MULTISPECIES: hypothetical protein [Arthrobacter]
gi542109922	4.81	1	1	187	20.9	6.54	9.30	MarR family transcriptional regulator [Arthrobacter sp. AK-YN10]
gi765010282	5.02	1	1	279	30.6	10.45	9.30	30S ribosomal protein S3 [Arthrobacter sp. A3]
gi489899738	8.56	2	1	222	24.5	6.30	9.30	two-component system response regulator [Arthrobacter globiformis]
gi737808152	2.20	1	1	636	69.1	7.43	9.29	cold-shock protein [Arthrobacter sp. H5]
gi759746540	1.46	1	1	1303	137.5	6.84	9.28	amino acid adenylation protein [Arthrobacter sp. 31Y]
gi930828275	4.21	1	1	309	34.2	5.20	9.28	alpha/beta hydrolase [Arthrobacter arilaitensis]
gi476400946	3.37	1	1	267	29.5	9.96	9.27	RDD domain-containing protein [Arthrobacter crystallopoietes BAB-32]
gi823665440	4.72	1	1	509	53.4	6.89	9.27	oxidoreductase [Arthrobacter sp. YC-RL1]
gi654827647	2.67	1	1	600	65.3	9.13	9.25	ABC transporter [Arthrobacter sp. H5]
gi916834922	3.56	3	1	253	27.9	9.73	9.24	hypothetical protein, partial [Arthrobacter sp. H14]
gi640199857	6.85	5	1	321	35.0	9.42	9.24	ABC transporter permease [Arthrobacter sp. 31Y]
gi116609868	2.44	2	1	492	53.6	4.97	9.22	adenosylhomocysteinase [Arthrobacter sp. FB24]
gi916835126	8.33	2	1	168	18.1	6.39	9.22	hypothetical protein [Arthrobacter sp. H14]
gi116609659	1.35	2	1	1040	115.3	5.68	9.21	type I site-specific deoxyribonuclease, HsdR family [Arthrobacter sp. FB24]
gi648574649	2.14	1	1	608	62.3	6.04	9.21	gamma-glutamyltransferase [Arthrobacter sp. 131MFCol6.1]
gi116609894	1.01	1	1	894	95.9	5.34	9.19	transcriptional regulator, LuxR family [Arthrobacter sp. FB24]
gi742859436	4.88	6	1	430	45.7	9.60	9.19	two-component system sensor histidine kinase [Arthrobacter sp. W1]
gi910695076	8.11	1	1	185	19.1	10.40	9.19	30S ribosomal protein S5, partial [Arthrobacter sp. Hiyo6]
gi742852742	1.89	1	1	582	63.3	5.20	9.18	hypothetical protein [Arthrobacter sp. W1]
gi551253699	1.99	7	1	1104	121.5	5.00	9.17	ATP-dependent DNA helicase [Arthrobacter sp. PAO19]
gi651503395	1.81	1	1	1159	124.2	5.24	9.17	DNA helicase UvrD [Arthrobacter sp. 35W]
gi219860784	5.78	4	1	225	23.8	5.02	9.17	NUDIX hydrolase [Arthrobacter chlorophenolicus A6]
gi651439869	3.42	1	1	439	47.0	8.54	9.17	drug:proton antiporter [Arthrobacter sp. H14]
gi470215981	10.81	4	1	185	19.8	6.01	9.16	hypothetical protein ADIAG_03942 [Arthrobacter gangotriensis Lz1y]
gi542108396	8.09	2	1	173	18.8	6.90	9.16	hypothetical protein M707_12230 [Arthrobacter sp. AK-YN10]
gi760112731	4.71	1	1	361	39.1	4.83	9.15	GTP-binding protein [Arthrobacter chlorophenolicus]
gi910740596	6.49	4	2	478	52.2	5.12	9.15	GTP-binding protein TypA/BipA homolog [Arthrobacter sp. Hiyo4]
gi651496304	1.32	2	1	681	73.4	5.47	9.15	anthranilate synthase [Arthrobacter sp. H20]
gi939050526	16.87	1	1	83	9.0	4.83	9.14	hypothetical protein [Arthrobacter sp. JCM 19049]
gi757624646	2.17	1	1	600	64.3	5.44	9.14	peptidoglycan glycosyltransferase [Arthrobacter sp. SPG23]
gi654811865	1.97	1	1	557	61.0	5.10	9.14	AMP-binding protein [Arthrobacter sp. MA-N2]
gi765010324	3.12	3	1	449	48.1	5.01	9.14	hypothetical protein [Arthrobacter sp. A3]

gi759733412	2.77	2	1	433	45.4	9.61	9.14	sugar ABC transporter permease [Arthrobacter sp. L77]
gi742757154	2.75	1	1	400	43.9	6.70	9.13	transporter [Arthrobacter phenanthrenivorans]
gi910283929	5.76	2	1	399	42.3	5.90	9.12	hypothetical protein [Arthrobacter sp. A3]
gi910738261	16.38	6	1	116	12.6	5.03	9.11	conserved hypothetical protein [Arthrobacter sp. Hiyo4]
gi690772227	7.00	2	1	257	27.8	5.50	9.11	hypothetical protein HMPREF2128_08885 [Arthrobacter albus DNF00011]
gi910738525	3.01	2	1	499	53.1	7.65	9.10	probable NADH dehydrogenase [Arthrobacter sp. Hiyo4]
gi757622607	14.53	6	1	172	18.4	6.70	9.09	MarR family transcriptional regulator [Arthrobacter sp. SPG23]
gi651503930	0.71	1	1	1406	151.3	5.10	9.09	hypothetical protein [Arthrobacter sp. 35W]
gi515766044	3.46	2	1	463	49.1	8.66	9.08	HNH endonuclease [Arthrobacter sp. M2012083]
gi470221751	5.63	2	1	284	31.1	4.69	9.08	Chlorocatechol 1,2-dioxygenase [Arthrobacter gangotriensis Lz1y]
gi636843450	2.63	2	1	419	46.7	9.25	9.08	hypothetical protein [Arthrobacter sp. TB 26]
gi470217626	4.72	5	1	445	45.0	8.66	9.08	3-oxoacyl-(acyl-carrier-protein) reductase [Arthrobacter gangotriensis Lz1y]
gi723608600	1.31	2	1	1065	117.3	5.85	9.07	Type I restriction-modification system, restriction subunit R [Arthrobacter sp. PAMC25486]
gi742855448	11.86	2	2	118	14.0	9.92	9.07	hypothetical protein [Arthrobacter sp. W1]
gi908697621	5.06	3	1	435	46.3	6.01	9.06	serine hydroxymethyltransferase [Arthrobacter sp. RIT-PI-e]
gi757623782	3.07	1	1	489	54.0	5.86	9.04	ATPase [Arthrobacter sp. SPG23]
gi476399853	3.11	1	1	514	55.8	10.20	9.01	signal recognition particle protein [Arthrobacter crystallopoietes BAB-32]
gi517598458	2.44	9	1	450	47.6	10.98	9.00	hypothetical protein [Arthrobacter sp. 162MFSha1.1]
gi914715064	5.64	3	1	319	33.5	5.29	9.00	D-glycerate dehydrogenase [Arthrobacter sp. ZBG10]
gi651490537	3.55	1	1	394	43.8	7.99	8.99	fatty acid desaturase [Arthrobacter sp. H20]
gi651431081	0.92	2	1	1199	130.5	5.07	8.98	chromosome segregation protein SMC [Arthrobacter sanguinis]
gi917760243	2.25	4	1	355	36.9	9.32	8.98	hydroxymethylpyrimidine/phosphomethylpyrimidine kinase [Arthrobacter sp. L77]
gi916820398	2.27	5	1	750	80.3	7.36	8.98	hypothetical protein [Arthrobacter sp. H20]
gi517592001	7.50	2	1	160	16.9	7.05	8.97	ArsR family transcriptional regulator [Arthrobacter sp. 135MFCol5.1]
gi470217279	2.75	5	1	545	58.7	4.65	8.97	family 5 extracellular solute-binding protein [Arthrobacter gangotriensis Lz1y]
gi767257562	3.02	1	1	430	47.5	11.24	8.97	lipoprotein [Arthrobacter sp. IHBB 11108]
gi323471030	4.33	3	1	393	41.7	7.80	8.96	transcriptional regulator/sugar kinase [Arthrobacter phenanthrenivorans Sphe3]
gi742854016	2.67	1	1	486	54.1	4.98	8.96	Fe-S cluster assembly protein SufB [Arthrobacter sp. W1]
gi930825100	9.35	1	1	214	24.5	9.60	8.96	hypothetical protein AOZ07_01490 [Arthrobacter arilaitensis]
gi908690283	9.19	1	1	185	20.7	11.14	8.95	transposase [Arthrobacter sp. H41]
gi652424410	3.81	4	1	446	47.9	4.78	8.94	sugar ABC transporter substrate-binding protein [Arthrobacter castelli]
gi359303311	15.74	2	2	216	23.1	8.09	8.94	hypothetical protein ARGLB_118_00040 [Arthrobacter globiformis NBRC 12137]
gi757624841	2.03	2	1	543	60.3	5.11	8.94	cyclohexanone monooxygenase [Arthrobacter sp. SPG23]
gi307745890	4.63	1	1	410	44.6	5.88	8.93	putative acyl-CoA dehydrogenase [Arthrobacter arilaitensis Re117]
gi765009559	7.48	3	1	147	15.9	11.63	8.92	hypothetical protein [Arthrobacter sp. A3]
gi542109829	8.15	3	1	270	29.3	5.49	8.92	hypothetical protein M707_03915 [Arthrobacter sp. AK-YN10]
gi927293150	3.40	1	1	412	43.7	5.55	8.92	hypothetical protein AL755_02905 (plasmid) [Arthrobacter sp. ERGS1:01]
gi651439634	3.70	3	1	324	36.8	6.21	8.90	LuxR family transcriptional regulator [Arthrobacter sp. H14]
gi517607259	4.61	1	1	369	38.6	7.42	8.89	hypothetical protein [Arthrobacter sp. 161MFSha2.1]
gi765012437	3.14	2	1	668	69.8	4.56	8.89	hypothetical protein [Arthrobacter sp. A3]
gi786031307	4.40	1	1	273	29.5	7.33	8.87	ABC transporter ATP-binding protein [Arthrobacter chlorophenolicus]
gi757622488	5.13	6	2	507	53.4	5.63	8.86	amidohydrolase [Arthrobacter sp. SPG23]
gi937258164	14.29	2	1	161	17.6	9.38	8.86	hypothetical protein AO716_08665 [Arthrobacter sp. Edens01]
gi937258166	4.90	3	1	306	32.1	4.94	8.86	4-hydroxy-tetrahydrodipicolinate synthase [Arthrobacter sp. Edens01]
gi636845922	4.75	2	1	442	45.8	9.50	8.85	MFS transporter [Arthrobacter sp. TB 26]
gi219859496	10.93	3	2	247	27.2	5.01	8.85	transcriptional regulator, MerR family [Arthrobacter chlorophenolicus A6]
gi640196635	4.99	1	1	401	40.2	5.12	8.85	glycerate kinase [Arthrobacter sp. 31Y]
gi916863569	3.59	2	1	334	35.4	5.15	8.84	sorbitol dehydrogenase [Arthrobacter sp. 35/47]
gi651467251	1.33	4	1	1277	140.7	6.10	8.84	alpha-ketoglutarate decarboxylase [Arthrobacter sp. 35/47]
gi308229520	1.90	2	1	527	61.0	9.25	8.83	AciI [Arthrobacter citreus]
gi937258490	2.58	4	1	581	62.1	9.44	8.82	hypothetical protein AO716_10640 [Arthrobacter sp. Edens01]
gi927295255	8.14	2	1	258	27.3	6.00	8.82	enoyl-CoA hydratase [Arthrobacter sp. ERGS1:01]
gi219858748	3.35	1	1	686	73.4	5.67	8.82	conserved hypothetical protein [Arthrobacter chlorophenolicus A6]
gi651447759	5.18	1	1	309	35.5	10.05	8.82	hypothetical protein [Arthrobacter nicotinovorans]
gi517606343	6.28	4	1	207	22.4	5.27	8.81	TetR family transcriptional regulator [Arthrobacter sp. 161MFSha2.1]
gi759747291	2.11	1	1	617	66.5	5.94	8.80	NADH oxidase [Arthrobacter sp. 31Y]
gi219860049	1.41	2	1	1346	140.7	7.78	8.80	FHA domain containing protein [Arthrobacter chlorophenolicus A6]
gi918469412	2.33	2	1	472	50.7	5.15	8.80	succinate-semialdehyde dehydrogenase [Arthrobacter crystallopoietes]

gi910738331	5.00	1	1	320	33.5	6.25	8.79	uncharacterized 47.3 kDa protein in thcA 5'region [Arthrobacter sp. Hiyo4]
gi723607408	1.75	2	1	458	49.8	8.62	8.77	hypothetical protein ART_1185 [Arthrobacter sp. PAMC25486]
gi654812254	2.47	3	1	649	70.0	5.52	8.77	3D-(3,5/4)-trihydroxycyclohexane-1,2-dione acylhydrolase (decyclizing) [Arthrobacter sp. M
gi654823429	4.98	3	2	442	48.1	4.97	8.76	RNA polymerase sigma factor [Arthrobacter sp. I3]
gi542110118	1.68	3	1	595	65.6	7.24	8.76	hypothetical protein M707_02015 [Arthrobacter sp. AK-YN10]
gi116613121	0.70	1	1	1575	173.2	5.68	8.76	helicase domain protein (plasmid) [Arthrobacter sp. FB24]
gi927031566	2.41	2	1	540	59.0	6.46	8.76	transcriptional regulator [Arthrobacter sp. LS16]
gi517602216	6.11	1	1	458	48.4	7.06	8.75	SAM-dependent methyltransferase [Arthrobacter sp. 131MFCol6.1]
gi476402963	3.04	3	1	460	49.5	7.14	8.75	hypothetical protein D477_001029 [Arthrobacter crystallopoietes BAB-32]
gi823665892	11.74	5	1	213	22.7	5.44	8.74	hypothetical protein AA310_08255 [Arthrobacter sp. YC-RL1]
gi919107792	3.89	1	1	334	34.7	5.20	8.74	hypothetical protein [Arthrobacter sp. IHBB 11108]
gi927031768	2.68	1	1	411	44.6	4.84	8.74	alanine-phosphoribitol ligase [Arthrobacter sp. LS16]
gi742853716	6.50	1	1	277	29.4	10.20	8.74	hypothetical protein [Arthrobacter sp. W1]
gi765003731	3.19	2	1	376	40.3	5.08	8.74	hypothetical protein [Arthrobacter sp. A3]
gi939036645	8.29	3	1	193	21.3	8.27	8.74	thymidylate kinase [Arthrobacter nitroguajacolicus]
gi636845208	6.13	3	1	326	34.3	5.36	8.73	prephenate dehydratase [Arthrobacter sp. TB 26]
gi765004846	3.33	1	1	510	55.9	5.43	8.73	UDP-N-acetylglucosamine 1-carboxyvinyltransferase [Arthrobacter sp. A3]
gi651489352	4.19	1	1	430	44.8	4.97	8.73	pyrimidine-nucleoside phosphorylase [Arthrobacter sp. H20]
gi323469047	1.18	1	1	1185	130.5	5.25	8.72	DNA-directed DNA polymerase III PolC [Arthrobacter phenanthrenivorans Sphe3]
gi910741713	6.42	2	1	218	23.6	6.06	8.72	hypothetical protein AHiyo4_45350 [Arthrobacter sp. Hiyo4]
gi759736640	4.17	3	2	648	71.5	6.27	8.71	DNA primase [Arthrobacter sp. L77]
gi917013271	1.14	2	1	963	106.0	6.99	8.70	ABC-ATPase UvrA [Arthrobacter sanguinis]
gi323470444	7.47	3	1	241	25.2	5.48	8.69	response regulator containing a CheY-like receiver domain and an HTH DNA-binding domai
gi928486700	1.43	2	1	771	80.5	6.06	8.69	glycosyl transferase [Arthrobacter alpinus]
gi759755866	2.42	2	1	869	91.7	5.55	8.68	ABC transporter [Arthrobacter sp. 131MFCol6.1]
gi403229246	2.21	2	1	589	65.7	6.62	8.68	hypothetical protein ARUE_c17620 [Arthrobacter sp. Rue61a]
gi515766351	3.29	1	1	516	54.4	5.95	8.68	aldehyde dehydrogenase [Arthrobacter sp. M2012083]
gi917013474	5.35	2	1	187	21.1	6.57	8.67	hypothetical protein [Arthrobacter sanguinis]
gi908697404	15.05	1	1	93	10.7	10.78	8.66	30S ribosomal protein S19 [Arthrobacter sp. RIT-PI-e]
gi651444231	0.79	3	1	1390	146.9	6.86	8.64	cell division protein FtsK [Arthrobacter nicotinovorans]
gi908699004	4.56	1	1	263	29.1	8.85	8.64	peptide ABC transporter ATPase [Arthrobacter sp. RIT-PI-e]
gi307743418	5.99	3	1	217	23.1	5.74	8.64	GntR-family transcriptional regulator [Arthrobacter arilaitensis Re117]
gi928486808	3.20	2	1	469	51.1	7.25	8.64	transcriptional regulator [Arthrobacter alpinus]
gi823668424	0.91	1	1	1422	149.7	5.53	8.64	cell division protein FtsK [Arthrobacter sp. YC-RL1]
gi470215939	4.10	3	1	439	48.4	8.05	8.63	DNA-directed DNA polymerase [Arthrobacter gangotriensis Lz1y]
gi470220603	3.05	1	1	426	45.0	4.56	8.63	enolase [Arthrobacter gangotriensis Lz1y]
gi652423525	2.15	2	1	836	94.8	9.01	8.63	hypothetical protein [Arthrobacter castelli]
gi769942526	0.63	1	1	1592	170.2	5.45	8.63	hypothetical protein [Arthrobacter sp. IHBB 11108]
gi652422780	4.84	1	1	372	41.9	5.60	8.62	UDP-N-acetyl glucosamine 2-epimerase [Arthrobacter castelli]
gi219857742	6.49	1	1	231	25.8	7.15	8.61	transcriptional regulator, GntR family [Arthrobacter chlorophenolicus A6]
gi323469589	10.98	1	1	173	19.4	9.83	8.61	uncharacterized conserved protein [Arthrobacter phenanthrenivorans Sphe3]
gi219858786	9.16	4	1	273	29.6	9.19	8.60	short-chain dehydrogenase/reductase SDR [Arthrobacter chlorophenolicus A6]
gi651439727	5.67	5	1	388	40.4	4.25	8.60	ABC transporter substrate-binding protein [Arthrobacter sp. H14]
gi517600394	4.52	2	2	199	22.8	7.28	8.60	hypothetical protein [Arthrobacter sp. 162MFSha1.1]
gi737798666	9.09	3	2	308	33.0	9.98	8.60	type II secretion system protein F [Arthrobacter sp. TB 23]
gi654817545	3.61	1	1	443	46.9	11.58	8.59	MFS transporter [Arthrobacter sp. UNC362MFTsu5.1]
gi654811603	23.08	2	1	78	8.0	5.26	8.59	thiamine biosynthesis protein ThiS [Arthrobacter sp. MA-N2]
gi917739472	3.74	4	1	321	36.5	9.69	8.59	23S rRNA pseudouridylate synthase [Arthrobacter sp. W1]
gi823665683	1.49	3	1	875	94.8	6.34	8.59	hypothetical protein AA310_06895 [Arthrobacter sp. YC-RL1]
gi489901122	2.08	1	1	577	63.2	6.25	8.59	multidrug ABC transporter ATP-binding protein [Arthrobacter globiformis]
gi545107848	11.59	1	1	164	17.9	9.61	8.57	MarR family transcriptional regulator [Arthrobacter sp. AK-YN10]
gi648575162	1.91	1	1	577	59.6	5.59	8.57	L-aspartate oxidase [Arthrobacter sp. 161MFSha2.1]
gi674645805	2.08	3	1	672	75.2	7.18	8.57	Galactofuranosyl transferase GlfT2 [Arthrobacter sp. 11W110_air]
gi654824450	8.03	1	1	249	26.1	9.54	8.57	ABC transporter ATPase [Arthrobacter sp. I3]
gi765002835	2.75	2	1	363	39.8	6.68	8.57	AsnC family transcriptional regulator [Arthrobacter sp. M2012083]
gi119951086	3.28	3	1	396	41.7	8.21	8.56	putative signal transduction histidine kinase [Arthrobacter aurescens TC1]
gi767256542	2.40	1	1	416	44.0	8.53	8.56	hypothetical protein UM93_00610 [Arthrobacter sp. IHBB 11108]
gi648574295	6.17	1	1	162	18.6	10.86	8.55	transposase [Arthrobacter sp. 162MFSha1.1]

gi517604500	3.59	1	1	752	77.5	5.30	8.55	hypothetical protein [Arthrobacter sp. 131MFCol6.1]
gi916359192	7.74	1	1	297	30.7	6.11	8.53	glycosyl transferase family 1 [Arthrobacter sp. 135MFCol5.1]
gi476400586	5.09	4	2	432	47.3	5.06	8.53	RNA polymerase subunit sigma 28 [Arthrobacter crystallopoietes BAB-32]
gi916815831	8.46	6	2	402	45.2	9.94	8.52	integrase [Arthrobacter sp. MA-N2]
gi723609355	3.23	2	1	310	33.5	10.08	8.51	sensor histidine kinase [Arthrobacter sp. PAMC25486]
gi119947470	6.01	1	1	233	24.8	9.57	8.51	putative ABC transporter, ATP-binding protein [Arthrobacter aurescens TC1]
gi910746129	4.09	10	1	440	47.1	10.52	8.51	hypothetical protein AHiyo8_38310 [Arthrobacter sp. Hiyo8]
gi914714021	2.09	1	1	1003	109.6	6.60	8.50	glutamine-synthetase [Arthrobacter sp. ZBG10]
gi919108080	5.08	5	1	394	43.1	6.81	8.50	hypothetical protein [Arthrobacter sp. IHBB 11108]
gi927032493	5.46	1	1	293	31.0	8.48	8.50	3-oxoacyl-ACP reductase [Arthrobacter sp. LS16]
gi914716984	7.39	2	1	203	21.9	8.88	8.50	TetR family transcriptional regulator [Arthrobacter sp. ZBG10]
gi742852241	3.08	3	1	520	55.6	4.94	8.49	histidine kinase [Arthrobacter sp. W1]
gi757624286	2.19	4	1	593	65.3	5.17	8.48	ATPase AAA [Arthrobacter sp. SPG23]
gi443481377	2.51	1	1	438	45.6	5.19	8.48	thymidine phosphorylase [Arthrobacter nitrophenolicus]
gi674644974	3.74	3	1	481	50.0	4.96	8.48	Fumarate reductase flavoprotein subunit precursor [Arthrobacter sp. 11W110_air]
gi737792885	4.47	4	1	313	32.3	9.48	8.47	lipase [Arthrobacter nicotinovorans]
gi219858116	14.13	4	1	184	19.7	10.15	8.47	conserved hypothetical protein [Arthrobacter chlorophenolicus A6]
gi928488220	5.99	1	1	284	30.2	6.13	8.47	spermidine synthase [Arthrobacter alpinus]
gi219858624	2.08	1	1	577	63.1	6.73	8.47	ABC transporter related [Arthrobacter chlorophenolicus A6]
gi162952743	2.42	1	1	455	49.5	6.05	8.47	formate dehydrogenase alpha subunit [Renibacterium salmoninarum ATCC 33209]
gi937259184	6.87	4	1	233	24.8	5.03	8.47	cytidylate kinase [Arthrobacter sp. Edens01]
gi119947949	3.93	1	1	433	45.4	4.94	8.46	putative serpin (serine proteinase inhibitor) family protein [Arthrobacter aurescens TC1]
gi917739783	5.38	2	1	353	38.3	6.90	8.46	hypothetical protein [Arthrobacter sp. W1]
gi786030721	1.03	1	1	874	95.7	5.05	8.46	aminopeptidase N [Arthrobacter chlorophenolicus]
gi654812641	0.73	2	1	1498	155.4	5.29	8.45	5'-nucleotidase [Arthrobacter sp. MA-N2]
gi651502230	2.37	3	1	930	96.0	5.91	8.45	phosphoenolpyruvate synthase [Arthrobacter sp. 35W]
gi654815419	3.59	2	1	418	45.4	5.30	8.44	glucarate dehydratase [Arthrobacter sp. PAO19]
gi767259044	4.77	1	1	398	42.8	8.56	8.42	DNA processing protein DprA [Arthrobacter sp. IHBB 11108]
gi651430546	4.17	1	1	480	50.9	5.48	8.42	inosine-5-monophosphate dehydrogenase [Arthrobacter sanguinis]
gi518312772	4.01	1	1	424	46.0	5.48	8.42	hypothetical protein [Arthrobacter sp. TB 23]
gi639129088	4.43	1	1	361	39.2	5.26	8.42	MULTISPECIES: inositol-3-phosphate synthase [Arthrobacter]
gi927293130	2.07	3	1	871	95.1	5.43	8.41	glycogen phosphorylase (plasmid) [Arthrobacter sp. ERGS1:01]
gi723607529	4.66	3	1	536	57.1	6.84	8.41	hypothetical protein ART_1306 [Arthrobacter sp. PAMC25486]
gi116611877	3.74	1	1	561	59.9	7.18	8.41	HNH endonuclease [Arthrobacter sp. FB24]
gi767257005	4.14	6	2	532	58.0	5.88	8.40	ABC transporter [Arthrobacter sp. IHBB 11108]
gi635350625	2.56	1	1	508	53.8	4.74	8.39	TAP-like family protein [Arthrobacter siccitolerans]
gi636844341	2.07	1	1	532	56.4	5.16	8.38	glycosyl hydrolase family 18 [Arthrobacter sp. TB 26]
gi737780728	5.00	2	1	460	50.6	9.42	8.37	mRNA 3'-end processing factor [Arthrobacter sp. CAL618]
gi927033169	9.48	1	1	211	24.1	7.20	8.37	recombinase [Arthrobacter sp. LS16]
gi470217590	2.81	1	1	533	56.9	5.64	8.37	ABC transporter [Arthrobacter gangotriensis Lz1y]
gi723606479	1.51	1	1	663	71.3	6.25	8.35	1-deoxy-D-xylulose-5-phosphate synthase [Arthrobacter sp. PAMC25486]
gi823665543	5.13	19	1	429	43.6	4.88	8.35	homoserine dehydrogenase [Arthrobacter sp. YC-RL1]
gi323469616	11.90	2	1	168	19.4	5.11	8.34	hypothetical protein Asphe3_21480 [Arthrobacter phenanthrenivorans Sphe3]
gi910742099	1.88	4	1	902	97.9	6.25	8.34	protein involved in chromosome condensation [Arthrobacter sp. Hiyo4]
gi759732599	5.65	4	1	248	27.6	5.30	8.33	phosphoglycerate mutase [Arthrobacter sp. L77]
gi737802427	1.75	1	1	513	56.4	10.65	8.32	hypothetical protein [Arthrobacter castelli]
gi162953994	5.52	1	1	308	34.0	7.15	8.31	formamidopyrimidine-DNA glycosylase [Renibacterium salmoninarum ATCC 33209]
gi654825616	3.30	1	1	455	51.5	5.31	8.30	LPS kinase [Arthrobacter sp. H5]
gi916290016	2.96	2	1	371	40.3	6.21	8.30	MULTISPECIES: cyclic pyranopterin phosphate synthase MoaA [Arthrobacter]
gi470216858	1.94	1	1	1030	112.0	7.71	8.30	transposase Tn3 [Arthrobacter gangotriensis Lz1y]
gi651444020	3.38	2	1	562	59.4	5.58	8.30	formate--tetrahydrofolate ligase [Arthrobacter nicotinovorans]
gi298379389	9.17	4	2	229	25.7	5.99	8.30	polyphosphate kinase, partial [Arthrobacter sp. AB(2010)]
gi162952754	12.28	1	1	114	12.3	8.53	8.29	bicyclomycin resistance protein [Renibacterium salmoninarum ATCC 33209]
gi759731441	1.45	1	1	1448	154.3	6.09	8.29	hypothetical protein [Arthrobacter sp. L77]
gi307744014	4.36	1	1	344	36.3	5.21	8.28	fructose-bisphosphatase, class II [Arthrobacter arilaitensis Re117]
gi470220759	3.91	1	1	409	45.3	5.39	8.28	aspartate aminotransferase [Arthrobacter gangotriensis Lz1y]
gi786025635	8.08	2	1	260	28.8	7.99	8.27	hypothetical protein [Arthrobacter chlorophenolicus]
gi116611084	3.59	1	1	418	41.7	5.68	8.27	ROK family protein [Arthrobacter sp. FB24]

gi910251488	2.15	1	1	978	109.5	5.45	8.26	DEAD/DEAH box helicase [Arthrobacter siccitolerans]
gi654823970	1.98	1	1	1011	111.0	5.96	8.26	alpha-mannosidase [Arthrobacter sp. I3]
gi916814516	2.42	1	1	413	43.4	7.06	8.26	secretion system protein E [Arthrobacter nicotinovorans]
gi651443101	0.62	1	1	1441	156.3	7.85	8.26	peptide synthetase [Arthrobacter sp. 9MFCol3.1]
gi723607154	2.74	1	1	547	56.1	11.53	8.26	hypothetical protein ART_0931 [Arthrobacter sp. PAMC25486]
gi918268314	22.09	4	1	86	8.6	7.25	8.26	succinyl-CoA ligase [ADP-forming] subunit alpha [Arthrobacter sp. Hiyo1]
gi542110119	3.29	2	2	1123	124.8	5.47	8.26	hypothetical protein M707_02020 [Arthrobacter sp. AK-YN10]
gi654814900	8.37	3	2	203	22.6	10.62	8.25	DNA invertase [Arthrobacter sp. MA-N2]
gi651498748	6.30	2	1	349	36.6	9.60	8.25	translation initiation factor IF-3 [Arthrobacter sp. 35W]
gi636843386	2.02	2	1	941	99.2	6.00	8.24	oxidoreductase [Arthrobacter sp. TB 26]
gi307745115	2.68	2	1	560	60.7	6.77	8.24	FAD binding domain-containing protein [Arthrobacter arilaitensis Re117]
gi759725340	4.66	2	1	386	41.7	6.21	8.24	DNA-binding protein [Arthrobacter sp. I3]
gi823665706	1.46	1	1	820	85.5	9.76	8.24	hypothetical protein AA310_07085 [Arthrobacter sp. YC-RL1]
gi648573264	19.83	10	1	116	12.7	8.59	8.24	hypothetical protein [Arthrobacter sp. 135MFCol5.1]
gi723609676	5.43	3	1	258	28.0	6.65	8.24	Transcriptional regulator, IclR family [Arthrobacter sp. PAMC25486]
gi654813807	2.84	1	1	703	74.2	8.27	8.23	primosomal protein N' [Arthrobacter sp. MA-N2]
gi742855455	1.73	1	1	635	70.7	7.71	8.22	glycosyltransferase [Arthrobacter sp. W1]
gi470216707	1.96	4	2	764	83.8	5.34	8.22	UvrD/REP helicase [Arthrobacter gangotriensis Lz1y]
gi651429917	2.96	2	1	304	33.5	4.97	8.21	30S ribosomal protein S2 [Arthrobacter sanguinis]
gi651491464	1.87	1	1	694	73.8	5.22	8.20	enoyl-CoA hydratase [Arthrobacter sp. H20]
gi928489021	5.75	1	1	313	35.1	10.45	8.20	hypothetical protein AOC05_17080 [Arthrobacter alpinus]
gi651498472	8.23	2	1	158	17.0	6.95	8.20	AsnC family transcriptional regulator [Arthrobacter sp. 35W]
gi786027150	1.03	1	1	1063	108.1	6.35	8.19	protease [Arthrobacter chlorophenolicus]
gi906447467	1.06	2	1	755	83.5	5.53	8.19	hypothetical protein AC792_06030 [Arthrobacter sp. RIT-PI-e]
gi914715694	2.46	3	1	406	45.1	5.80	8.19	aminotransferase [Arthrobacter sp. ZBG10]
gi651435294	2.92	1	1	411	42.7	6.20	8.18	threonine dehydratase [Arthrobacter sp. H41]
gi937259416	3.33	1	1	451	51.2	5.43	8.18	lipopolysaccharide kinase [Arthrobacter sp. Edens01]
gi674644640	4.53	2	1	265	28.6	11.40	8.18	RDD family protein [Arthrobacter sp. 11W110_air]
gi648573984	0.73	1	1	1235	136.3	6.38	8.17	nitrate reductase [Arthrobacter sp. 162MFSha1.1]
gi651429487	4.00	3	1	250	27.5	11.27	8.17	membrane protein [Arthrobacter sanguinis]
gi910249123	1.30	1	1	847	92.7	5.40	8.16	hypothetical protein [Arthrobacter siccitolerans]
gi518312623	8.60	1	1	221	22.9	9.85	8.15	hypothetical protein [Arthrobacter sp. TB 23]
gi651431179	4.19	2	1	191	21.6	9.16	8.14	50S ribosomal protein L5 [Arthrobacter sanguinis]
gi654813195	2.86	1	1	315	34.0	5.54	8.14	sugar isomerase [Arthrobacter sp. MA-N2]
gi937259032	10.83	1	1	157	16.5	5.94	8.13	hypothetical protein AO716_13900 [Arthrobacter sp. Edens01]
gi654814597	8.90	6	1	146	16.4	10.78	8.13	membrane protein insertion efficiency factor YidD [Arthrobacter sp. MA-N2]
gi910842277	3.44	1	1	407	43.8	4.81	8.12	sugar ABC transporter substrate-binding protein [Arthrobacter sp. ZBG10]
gi916872091	5.81	1	1	327	32.7	5.67	8.11	N-acetylmuramic acid 6-phosphate etherase [Arthrobacter sp. H5]
gi910739428	3.47	1	1	490	50.7	9.50	8.11	sensor protein KdpD [Arthrobacter sp. Hiyo4]
gi652425041	3.56	1	1	309	33.9	9.64	8.11	pseudouridine synthase [Arthrobacter castelli]
gi759734436	1.96	2	2	970	106.8	6.52	8.10	ABC-ATPase UvrA [Arthrobacter sp. L77]
gi517591294	1.99	1	1	805	86.8	5.50	8.10	phosphoenolpyruvate synthase [Arthrobacter sp. 135MFCol5.1]
gi219861173	3.25	2	1	461	49.5	5.35	8.10	nucleotide sugar dehydrogenase [Arthrobacter chlorophenolicus A6]
gi640203726	1.74	1	1	689	72.9	5.63	8.10	phosphate acetyltransferase [Arthrobacter sp. 31Y]
gi914717100	2.97	1	1	538	56.1	10.70	8.09	ABC transporter [Arthrobacter sp. ZBG10]
gi517592744	4.90	2	1	388	39.6	7.77	8.09	transglycosylase [Arthrobacter sp. 135MFCol5.1]
gi359306171	5.06	2	1	257	28.1	6.58	8.08	hypothetical protein ARGLB_047_01190 [Arthrobacter globiformis NBRC 12137]
gi674646957	2.17	1	1	828	87.7	7.01	8.08	hypothetical protein BN1051_03170 [Arthrobacter sp. 11W110_air]
gi757625677	5.31	1	1	471	51.9	5.73	8.08	hypothetical protein TV39_04765 [Arthrobacter sp. SPG23]
gi651429872	3.20	8	1	406	43.7	4.96	8.08	2-amino-3-ketobutyrate CoA ligase [Arthrobacter sanguinis]
gi910738466	10.07	8	1	139	14.9	8.79	8.08	hypothetical protein AHiyo4_12880 [Arthrobacter sp. Hiyo4]
gi737789341	1.48	2	1	742	80.1	4.92	8.08	polynucleotide phosphorylase [Arthrobacter albus]
gi930826856	2.91	2	1	447	47.4	9.74	8.08	hypothetical protein AOZ07_11580 [Arthrobacter arilaitensis]
gi551256327	5.15	1	1	330	36.4	5.62	8.06	16S rRNA (cytosine(1402)-N(4))-methyltransferase [Arthrobacter sp. PAO19]
gi937259001	3.72	3	1	403	43.2	9.67	8.06	transposase [Arthrobacter sp. Edens01]
gi635353177	1.38	2	1	653	68.3	5.38	8.06	copper-translocating P-type ATPase [Arthrobacter siccitolerans]
gi476398940	2.48	1	1	966	100.8	5.78	8.06	molybdopterin binding aldehyde oxidase and xanthine dehydrogenase [Arthrobacter crysta
gi769943171	3.39	1	1	501	55.4	5.44	8.05	cysteine--tRNA ligase [Arthrobacter sp. IHBB 11108]

gi189044289	11.20	2	1	125	14.1	10.56	8.05	RecName: Full=30S ribosomal protein S13
gi359306181	15.89	1	1	107	11.4	10.83	8.05	putative LacI family transcriptional regulator, partial [Arthrobacter globiformis NBRC 12137
gi910743613	1.34	1	1	599	63.9	5.24	8.04	phenylalanine--tRNA ligase beta subunit [Arthrobacter sp. Hiyo8]
gi640197134	8.09	3	1	136	14.5	6.11	8.04	MerR family transcriptional regulator [Arthrobacter sp. 31Y]
gi939051656	3.26	2	1	368	38.6	8.02	8.04	hypothetical protein [Arthrobacter sp. JCM 19049]
gi563398134	4.44	2	1	495	52.3	6.80	8.04	tail fiber protein [Arthrobacter phage vB_ArS-ArV2]
gi476400721	3.99	2	1	326	33.5	6.25	8.03	peptidase S1 and S6 chymotrypsin/Hap [Arthrobacter crystallopoietes BAB-32]
gi916872148	3.05	1	1	361	37.4	6.16	8.02	hypothetical protein [Arthrobacter sp. H5]
gi654818105	2.55	1	1	785	83.2	5.41	8.01	CHAT domain-containing protein [Arthrobacter sp. UNC362MFTsu5.1]
gi937262492	3.11	3	1	837	85.2	4.65	8.00	hypothetical protein AO716_04750 [Arthrobacter sp. Edens01]
gi767257146	6.93	1	1	202	22.9	10.67	8.00	membrane protein [Arthrobacter sp. IHBB 11108]
gi636845532	2.14	1	1	1028	115.5	5.36	8.00	hypothetical protein [Arthrobacter sp. TB 26]
gi927295073	1.16	1	1	1034	106.5	4.97	8.00	hypothetical protein AL755_15435 [Arthrobacter sp. ERGS1:01]
gi767257978	5.80	1	1	414	43.6	7.91	8.00	siroheme synthase CysG [Arthrobacter sp. IHBB 11108]
gi737807811	2.17	1	1	692	74.4	5.27	8.00	acetyl-CoA carboxylase [Arthrobacter sp. H5]
gi635349606	0.95	1	1	1154	126.6	5.88	7.99	putative ATP-binding protein [Arthrobacter siccitolerans]
gi517599457	4.50	1	1	222	24.2	5.62	7.98	octanoyltransferase [Arthrobacter sp. 162MFSha1.1]
gi648574636	1.86	2	1	591	62.9	9.14	7.98	preprotein translocase subunit SecD [Arthrobacter sp. 131MFCol6.1]
gi937258424	13.82	1	1	152	16.3	8.22	7.98	hypothetical protein AO716_10230 [Arthrobacter sp. Edens01]
gi759733978	5.42	1	1	443	47.2	6.28	7.97	phosphoribosyl transferase [Arthrobacter sp. L77]
gi757624871	3.43	1	1	525	56.7	8.85	7.97	hypothetical protein TV39_08840 [Arthrobacter sp. SPG23]
gi765008351	4.92	2	1	325	34.8	5.07	7.97	NADH pyrophosphatase [Arthrobacter sp. A3]
gi674644110	3.92	1	1	383	40.3	5.43	7.96	Beta-ketoadipyl-CoA thiolase [Arthrobacter sp. 11W110_air]
gi937256301	4.67	2	1	407	41.3	5.27	7.96	N-acetylglucosamine 6-phosphate deacetylase [Arthrobacter sp. Edens01]
gi908699487	4.50	2	1	311	36.5	5.78	7.96	polyphosphate kinase [Arthrobacter sp. RIT-PI-e]
gi695270312	5.79	1	1	311	32.5	6.73	7.96	ABC transporter membrane protein [Arthrobacter globiformis]
gi116609351	1.97	1	1	659	69.8	5.66	7.95	conserved hypothetical protein [Arthrobacter sp. FB24]
gi307743467	6.13	1	1	212	23.2	9.92	7.95	possible transcriptional regulator [Arthrobacter arilaitensis Re117]
gi476402333	1.73	1	1	404	44.9	5.30	7.94	aminotransferase AlaT [Arthrobacter crystallopoietes BAB-32]
gi930827608	5.05	1	1	317	33.4	8.92	7.94	ABC transporter permease [Arthrobacter arilaitensis]
gi470220766	4.71	4	1	361	38.5	4.60	7.94	GTP-binding protein EngD [Arthrobacter gangotriensis Lz1y]
gi786027108	3.12	2	1	353	37.4	6.76	7.93	glycosyl transferase family 1 [Arthrobacter chlorophenolicus]
gi823667034	5.35	2	2	636	70.7	5.25	7.93	phenol 2-monooxygenase [Arthrobacter sp. YC-RL1]
gi910252010	2.51	1	1	358	39.0	10.11	7.93	epimerase [Arthrobacter siccitolerans]
gi476398982	3.17	2	1	663	72.3	4.89	7.92	ABC transporter, partial [Arthrobacter crystallopoietes BAB-32]
gi928487155	4.64	2	1	345	36.2	5.24	7.92	glycerol-3-phosphate dehydrogenase [Arthrobacter alpinus]
gi651498880	1.28	1	1	1560	162.4	5.14	7.92	hypothetical protein [Arthrobacter sp. 35W]
gi639131099	10.75	2	1	186	21.1	10.33	7.91	integrase, partial [Arthrobacter sp. CAL618]
gi930825234	0.71	1	1	1554	167.0	6.04	7.90	DEAD/DEAH box helicase [Arthrobacter arilaitensis]
gi939050369	6.60	1	1	212	22.4	10.21	7.90	30S ribosomal protein S5 [Arthrobacter sp. JCM 19049]
gi470220215	3.83	1	1	235	24.7	9.26	7.88	50S ribosomal protein L1 [Arthrobacter gangotriensis Lz1y]
gi823666606	2.73	2	1	439	49.0	6.23	7.88	phenylacetate--CoA ligase [Arthrobacter sp. YC-RL1]
gi651485033	2.65	2	1	491	54.9	5.17	7.88	Fe-S cluster assembly protein SufB [Arthrobacter sp. Br18]
gi908699245	3.01	4	1	698	74.9	5.62	7.88	enoyl-CoA hydratase [Arthrobacter sp. RIT-PI-e]
gi759732288	2.46	1	1	487	51.9	5.01	7.88	hypothetical protein [Arthrobacter sp. L77]
gi651437835	2.01	1	1	398	43.2	6.37	7.87	pilus assembly protein CpaE [Arthrobacter sp. H14]
gi742071661	1.15	1	1	1048	117.0	6.60	7.87	hypothetical protein ANMWB30_11730 [Arthrobacter sp. MWB30]
gi640194869	4.26	3	1	305	32.2	5.19	7.87	thiosulfate sulfurtransferase [Arthrobacter sp. 31Y]
gi654817536	2.92	2	2	1198	122.4	7.93	7.86	ABC transporter [Arthrobacter sp. UNC362MFTsu5.1]
gi910696903	0.93	2	1	1617	180.0	5.34	7.86	NAD-specific glutamate dehydrogenase [Arthrobacter sp. Hiyo6]
gi651494343	1.87	2	1	750	81.8	5.54	7.85	ATPase AAA [Arthrobacter sp. H20]
gi937262402	1.08	4	1	1298	144.1	6.52	7.84	ATP-dependent RNA helicase HrpA [Arthrobacter sp. Edens01]
gi908699306	2.13	2	1	375	40.4	8.88	7.83	fusaric acid resistance protein [Arthrobacter sp. RIT-PI-e]
gi476402255	1.91	1	1	1151	123.4	5.27	7.83	hypothetical protein D477_004446 [Arthrobacter crystallopoietes BAB-32]
gi651440541	1.70	1	1	471	49.2	7.05	7.83	DNA repair protein RadA [Arthrobacter sp. H14]
gi654825935	3.95	1	1	532	57.7	5.66	7.83	ABC transporter [Arthrobacter sp. H5]
gi737810320	2.48	1	1	443	46.2	5.02	7.82	glutamyl-tRNA reductase [Arthrobacter sp. 35/47]
gi651436466	2.42	1	1	578	60.5	4.87	7.81	DNA repair protein RecN [Arthrobacter sp. H41]

gi443480726	6.05	1	1	248	26.3	4.78	7.81	phosphoribosyl isomerase A [Arthrobacter nitrophenolicus]
gi651443992	7.26	1	1	303	32.5	9.89	7.81	NAD-dependent dehydratase [Arthrobacter nicotinovorans]
gi517601471	3.79	2	1	264	28.4	8.28	7.80	IclR family transcriptional regulator [Arthrobacter sp. 162MFSha1.1]
gi654822965	1.41	1	1	569	57.7	9.91	7.77	hypothetical protein, partial [Arthrobacter sp. I3]
gi759725168	4.62	1	1	368	39.3	9.14	7.77	hypothetical protein [Arthrobacter sp. I3]
gi648575582	7.88	2	1	203	21.9	7.65	7.77	TetR family transcriptional regulator [Arthrobacter sp. 161MFSha2.1]
gi476401632	2.35	1	1	468	51.9	5.38	7.77	glycine cleavage system protein T [Arthrobacter crystallopoietes BAB-32]
gi915933906	1.12	1	1	894	99.3	5.29	7.76	hypothetical protein [Arthrobacter globiformis]
gi928487544	2.68	1	1	448	48.1	9.07	7.76	hypothetical protein AOC05_12220 [Arthrobacter alpinus]
gi654819426	1.12	1	1	978	110.3	5.66	7.76	restriction endonuclease subunit R [Arthrobacter sp. UNC362MFTsu5.1]
gi937257854	3.65	1	1	384	40.8	5.38	7.75	spermidine/putrescine ABC transporter ATP-binding protein [Arthrobacter sp. Edens01]
gi651442567	8.70	3	1	161	16.9	11.47	7.75	hypothetical protein [Arthrobacter sp. 9MFCol3.1]
gi517607940	4.38	10	1	502	53.1	4.84	7.74	hypothetical protein [Arthrobacter sp. 161MFSha2.1]
gi517601868	3.11	1	1	289	32.1	9.28	7.74	hypothetical protein [Arthrobacter sp. 162MFSha1.1]
gi916691894	2.00	1	1	551	57.1	7.59	7.74	hypothetical protein [Arthrobacter castelli]
gi917739807	1.71	1	1	995	111.0	5.62	7.74	restriction endonuclease subunit R [Arthrobacter sp. W1]
gi219862006	1.81	1	1	1050	114.4	5.67	7.73	hypothetical protein Achl_4396 (plasmid) [Arthrobacter chlorophenolicus A6]
gi652424071	5.08	1	1	256	28.4	5.72	7.73	SGNH hydrolase [Arthrobacter castelli]
gi517603512	5.25	5	1	305	33.9	10.02	7.73	hypothetical protein [Arthrobacter sp. 131MFCol6.1]
gi116611833	1.72	1	1	638	65.0	10.15	7.72	O-antigen polymerase [Arthrobacter sp. FB24]
gi651439546	2.66	2	1	376	40.9	7.02	7.71	hypothetical protein [Arthrobacter sp. H14]
gi551255425	1.87	1	1	694	75.4	4.91	7.71	multidrug ABC transporter ATP-binding protein [Arthrobacter sp. PAO19]
gi939050298	9.22	1	1	206	22.4	5.26	7.71	LuxR family transcriptional regulator [Arthrobacter sp. JCM 19049]
gi937258933	6.70	3	1	358	36.6	5.60	7.70	molybdenum ABC transporter ATP-binding protein [Arthrobacter sp. Edens01]
gi162954744	6.67	3	1	285	30.0	9.16	7.70	short chain dehydrogenase [Renibacterium salmoninarum ATCC 33209]
gi765004447	10.27	4	1	185	20.9	5.30	7.70	ribosome-recycling factor [Arthrobacter sp. A3]
gi219857949	12.80	7	1	125	12.8	4.46	7.69	Rhodanese domain protein [Arthrobacter chlorophenolicus A6]
gi737787338	2.14	1	1	560	62.5	5.07	7.68	ABC transporter ATP-binding protein [Arthrobacter albus]
gi910739986	5.34	4	1	262	30.0	11.63	7.67	ribonuclease E/G-like protein [Arthrobacter sp. Hiyo4]
gi323468304	2.35	3	1	723	75.4	5.12	7.67	translation elongation factor 2 (EF-2/EF-G) [Arthrobacter phenanthrenivorans Sphe3]
gi651440654	7.98	1	1	263	27.6	9.66	7.66	hypothetical protein [Arthrobacter sp. H14]
gi910745071	1.71	2	1	762	82.0	5.17	7.66	carbon monoxide dehydrogenase large chain [Arthrobacter sp. Hiyo8]
gi910251228	3.61	7	1	416	43.2	7.75	7.66	transporter [Arthrobacter siccitolerans]
gi517605714	6.61	2	1	257	26.7	5.11	7.66	hypothetical protein [Arthrobacter sp. 131MFCol6.1]
gi918265189	1.40	2	1	1497	163.6	6.65	7.65	conjugal transfer protein TraA [Arthrobacter sp. Hiyo1]
gi930827483	8.37	1	1	263	28.4	5.12	7.65	hypothetical protein AOZ07_15140 [Arthrobacter arilaitensis]
gi654828296	3.89	1	1	437	46.8	5.14	7.65	Fe-S cluster assembly protein SufD [Arthrobacter sp. H5]
gi723607175	1.36	1	1	1102	118.7	4.93	7.65	carbamoyl phosphate synthase large subunit [Arthrobacter sp. PAMC25486]
gi651430445	4.87	6	1	390	42.2	5.01	7.63	molybdopterin biosynthesis protein MoeZ [Arthrobacter sanguinis]
gi219858569	9.52	4	1	231	23.1	4.73	7.63	molybdopterin-guanine dinucleotide biosynthesis protein [Arthrobacter chlorophenolicus A6
gi651432327	9.32	1	1	161	17.8	9.86	7.63	hypothetical protein [Arthrobacter sp. H41]
gi910737671	3.77	1	1	345	36.0	5.54	7.62	universal stress protein Rv2623/MT2698 [Arthrobacter sp. Hiyo4]
gi443482581	2.62	1	1	572	63.1	5.94	7.62	histidine kinase [Arthrobacter nitrophenolicus]
gi916820570	6.71	3	1	283	30.1	5.19	7.62	acetyltransferase [Arthrobacter sp. H20]
gi737789937	3.17	1	1	473	52.0	4.91	7.62	argininosuccinate synthase [Arthrobacter albus]
gi757625536	5.71	3	1	368	38.1	8.12	7.62	sugar ABC transporter substrate-binding protein [Arthrobacter sp. SPG23]
gi917759806	1.81	1	1	609	68.2	9.33	7.60	ABC transporter [Arthrobacter sp. L77]
gi759729004	5.15	1	1	369	38.9	6.30	7.60	alkane 1-monooxygenase [Arthrobacter sp. UNC362MFTsu5.1]
gi723609630	1.34	1	1	820	85.1	5.73	7.58	hypothetical protein ART_3407 [Arthrobacter sp. PAMC25486]
gi910740003	3.96	1	1	278	29.8	5.16	7.58	valine--tRNA ligase [Arthrobacter sp. Hiyo4]
gi910693985	6.18	1	1	178	19.9	5.10	7.57	glutamine synthetase 1 [Arthrobacter sp. Hiyo6]
gi917013195	12.10	8	1	157	17.0	10.30	7.56	hypothetical protein [Arthrobacter sanguinis]
gi651429675	6.52	1	1	184	19.7	5.66	7.56	hypothetical protein [Arthrobacter sanguinis]
gi323467746	1.83	2	1	981	104.4	5.33	7.54	sarcosine oxidase, alpha subunit family, heterotetrameric form [Arthrobacter phenanthreniv
gi908697788	3.57	2	2	673	75.4	6.73	7.54	glycosyl transferase [Arthrobacter sp. RIT-PI-e]
gi648259994	3.26	1	1	491	50.5	5.34	7.54	bifunctional N-acetylglucosamine-1-phosphate uridyltransferase/glucosamine-1-phosphate a
gi939050525	3.45	1	1	406	43.8	5.01	7.54	2-amino-3-ketobutyrate CoA ligase [Arthrobacter sp. JCM 19049]
gi759716863	2.76	2	1	617	70.2	7.46	7.53	hypothetical protein, partial [Arthrobacter sp. AK-YN10]

gi651429508	1.53	1	1	1175	130.6	5.24	7.53	DNA polymerase III subunit alpha [Arthrobacter sanguinis]
gi636844019	5.40	4	1	278	29.8	5.44	7.52	hypothetical protein [Arthrobacter sp. TB 26]
gi517600101	5.88	1	1	289	31.6	5.19	7.52	glycosyl transferase [Arthrobacter sp. 162MFSha1.1]
gi517590889	1.57	1	1	894	94.5	5.64	7.52	GCN5 family acetyltransferase [Arthrobacter sp. 135MFCol5.1]
gi916834607	0.79	2	1	1133	125.7	5.62	7.52	hypothetical protein [Arthrobacter sp. H14]
gi651508065	5.11	1	1	411	45.5	6.25	7.51	hypothetical protein [Arthrobacter sp. 35W]
gi551254183	2.82	2	1	425	47.1	5.15	7.49	serine--tRNA ligase [Arthrobacter sp. PAO19]
gi517605563	2.87	14	1	488	52.0	5.91	7.48	two-component system sensor histidine kinase [Arthrobacter sp. 131MFCol6.1]
gi652422516	2.25	1	1	756	76.9	4.28	7.48	hypothetical protein [Arthrobacter castelli]
gi723608334	5.67	1	1	194	21.5	9.44	7.47	50S ribosomal protein L5 [Arthrobacter sp. PAMC25486]
gi116610225	4.26	2	1	399	40.8	9.64	7.45	Peptidoglycan-binding LysM [Arthrobacter sp. FB24]
gi219858765	5.39	5	1	241	27.1	7.06	7.45	hemerythrin HHE cation binding domain protein [Arthrobacter chlorophenolicus A6]
gi517608754	4.99	4	1	401	42.3	5.48	7.45	hypothetical protein [Arthrobacter sp. 161MFSha2.1]
gi757624804	3.32	1	1	602	65.7	5.20	7.44	hypothetical protein TV39_09020 [Arthrobacter sp. SPG23]
gi742071917	1.78	8	1	732	79.7	5.20	7.43	DNA ligase LigA [Arthrobacter sp. MWB30]
gi908740352	2.89	2	1	311	34.9	5.00	7.42	adenosine deaminase [Arthrobacter arilaitensis]
gi307744090	1.03	1	1	1167	123.8	4.89	7.41	putative drug exporter of the RND superfamily [Arthrobacter arilaitensis Re117]
gi162955171	5.84	1	1	274	28.2	5.54	7.40	purine nucleoside phosphorylase [Renibacterium salmoninarum ATCC 33209]
gi119951547	1.56	2	1	1605	179.7	5.55	7.40	putative Helicase (plasmid) [Arthrobacter aurescens TC1]
gi652422968	5.67	4	1	194	21.3	7.08	7.40	bifunctional pyrimidine regulatory protein PyrR uracil phosphoribosyltransferase [Arthrobac
gi116609426	2.80	1	1	500	56.2	6.81	7.40	trehalose 6-phosphate synthase [Arthrobacter sp. FB24]
gi928486873	10.38	2	1	106	11.2	10.54	7.39	DNA-binding protein [Arthrobacter alpinus]
gi910738748	3.27	1	1	397	41.7	5.57	7.39	methylmalonate semialdehyde dehydrogenase [acylating] 2 [Arthrobacter sp. Hiyo4]
gi742068652	0.94	1	1	1378	151.5	6.28	7.39	hypothetical protein ANMWB30_42730 [Arthrobacter sp. MWB30]
gi511534814	7.88	3	1	241	26.8	10.24	7.38	putative transposase, partial (plasmid) [Arthrobacter nicotinovorans]
gi652425254	4.38	1	1	388	39.8	5.34	7.38	transcriptional regulator [Arthrobacter castelli]
gi759735038	14.41	1	1	118	12.9	6.79	7.38	hypothetical protein [Arthrobacter sp. L77]
gi518312021	8.02	1	1	162	17.9	6.34	7.38	MULTISPECIES: hypothetical protein [Arthrobacter]
gi654816796	1.65	1	1	728	78.5	9.09	7.37	transcription termination factor Rho [Arthrobacter sp. UNC362MFTsu5.1]
gi910746378	7.73	1	1	181	19.3	5.16	7.37	fumarylacetoacetate hydrolase domain-containing protein 2 [Arthrobacter sp. Hiyo8]
gi651430825	3.60	1	1	389	41.2	7.88	7.36	hypothetical protein [Arthrobacter sanguinis]
gi916691688	2.97	1	1	370	41.3	5.21	7.36	cyclic pyranopterin phosphate synthase MoaA [Arthrobacter castelli]
gi759736701	4.68	2	1	470	48.4	6.38	7.36	branched-chain alpha-keto acid dehydrogenase subunit E2 [Arthrobacter sp. L77]
gi917013249	3.62	1	1	470	50.2	5.40	7.35	hypothetical protein [Arthrobacter sanguinis]
gi737787100	2.13	1	1	423	47.9	6.68	7.35	hypothetical protein [Arthrobacter albus]
gi757626132	2.54	1	1	907	95.0	6.70	7.35	phosphoenolpyruvate synthase [Arthrobacter sp. SPG23]
gi767257690	1.74	1	1	575	62.2	6.11	7.35	ABC transporter ATP-binding protein [Arthrobacter sp. IHBB 11108]
gi916813731	1.94	1	1	929	97.7	8.90	7.35	helicase [Arthrobacter nicotinovorans]
gi927033687	4.50	2	1	333	34.9	8.06	7.34	hypothetical protein AFL94_16830 [Arthrobacter sp. LS16]
gi323468332	2.44	1	1	491	52.0	5.77	7.34	signal transduction histidine kinase [Arthrobacter phenanthrenivorans Sphe3]
gi918268814	4.04	1	1	322	36.9	9.01	7.34	pantothenate kinase [Arthrobacter sp. Hiyo1]
gi542106407	12.50	1	1	80	9.3	6.04	7.33	hypothetical protein M707_21935 [Arthrobacter sp. AK-YN10]
gi162953407	6.80	3	1	250	27.1	6.65	7.32	glutamate transport ATP-binding protein [Renibacterium salmoninarum ATCC 33209]
gi654811523	1.71	1	1	1348	141.5	6.98	7.32	hypothetical protein [Arthrobacter sp. MA-N2]
gi403231993	1.92	3	1	520	56.6	8.59	7.32	hypothetical protein ARUE_232p02060 (plasmid) [Arthrobacter sp. Rue61a]
gi910738382	1.68	2	1	895	99.0	7.30	7.32	protein involved in chromosome condensation [Arthrobacter sp. Hiyo4]
gi910697394	1.83	2	1	710	77.2	5.06	7.32	mannosylglycerate hydrolase [Arthrobacter sp. Hiyo6]
gi737792948	2.20	2	1	499	50.9	5.87	7.32	carbohydrate kinase [Arthrobacter nicotinovorans]
gi651454653	13.30	4	1	188	20.5	6.81	7.31	MarR family transcriptional regulator [Arthrobacter nicotinovorans]
gi930826069	2.78	1	1	468	49.2	10.43	7.31	hypothetical protein AOZ07_07055 [Arthrobacter arilaitensis]
gi765012154	3.36	3	1	506	55.6	5.15	7.30	arabinose isomerase [Arthrobacter sp. A3]
gi742855121	4.82	1	1	332	36.5	9.73	7.30	NAD-dependent epimerase [Arthrobacter sp. W1]
gi470220980	1.99	1	1	452	48.2	6.67	7.29	peptidase M20 [Arthrobacter gangotriensis Lz1y]
gi823666857	2.79	1	1	502	54.7	5.96	7.27	D-ribose transporter ATP-binding protein [Arthrobacter sp. YC-RL1]
gi737788308	1.09	6	1	1013	109.4	5.15	7.27	cytochrome P450 [Arthrobacter albus]
gi517591906	6.20	1	1	242	25.7	5.86	7.27	L-ribulose-5-phosphate 4-epimerase [Arthrobacter sp. 135MFCol5.1]
gi162954217	2.93	1	1	341	36.7	5.78	7.26	holliday junction DNA helicase [Renibacterium salmoninarum ATCC 33209]
gi908697195	12.21	2	1	131	14.5	4.48	7.26	glyoxalase [Arthrobacter sp. RIT-PI-e]

gi654827225	3.80	2	1	500	52.6	5.41	7.25	dehydrogenase [Arthrobacter sp. H5]
gi917021959	1.81	1	1	552	59.4	5.91	7.25	PucR family transcriptional regulator [Arthrobacter sp. UNC362MFTsu5.1]
gi517590131	3.52	1	1	284	31.9	6.57	7.25	polyphosphate--nucleotide phosphotransferase [Arthrobacter sp. 135MFCol5.1]
gi914714279	0.69	2	1	1161	126.5	7.14	7.25	DNA polymerase III subunit alpha [Arthrobacter sp. ZBG10]
gi908696986	3.63	1	1	468	53.0	7.11	7.24	hypothetical protein [Arthrobacter sp. RIT-PI-e]
gi470220210	4.01	2	1	399	42.2	5.27	7.24	class I and II aminotransferase [Arthrobacter gangotriensis Lz1y]
gi515766930	1.37	1	1	878	95.0	5.41	7.23	trehalose phosphatase [Arthrobacter sp. M2012083]
gi910741877	8.92	3	1	157	16.2	5.81	7.23	phosphoribosylformylglycinamidine cyclo-ligase [Arthrobacter sp. Hiyo4]
gi476399340	4.20	5	1	500	54.9	7.03	7.22	nucleoside-diphosphate sugar epimerase [Arthrobacter crystallopoietes BAB-32]
gi916926310	8.06	1	1	397	39.4	6.32	7.22	acetyl-CoA acetyltransferase [Arthrobacter sp. 9MFCol3.1]
gi937262344	2.30	1	1	914	101.2	5.40	7.21	preprotein translocase subunit SecA [Arthrobacter sp. Edens01]
gi786029566	5.05	2	1	198	22.1	10.43	7.21	membrane protein [Arthrobacter chlorophenolicus]
gi323469518	1.38	2	1	1162	126.5	6.87	7.21	DNA-directed DNA polymerase III PolC [Arthrobacter phenanthrenivorans Sphe3]
gi916869680	3.75	7	1	267	29.6	8.81	7.21	hypothetical protein [Arthrobacter sp. Br18]
gi651466721	6.82	3	1	308	33.1	5.08	7.20	NAD kinase [Arthrobacter sp. 35/47]
gi914715152	4.63	5	1	389	41.0	9.36	7.19	hypothetical protein [Arthrobacter sp. ZBG10]
gi654819489	8.06	1	1	211	23.2	7.05	7.19	hypothetical protein [Arthrobacter sp. UNC362MFTsu5.1]
gi651440332	1.24	1	1	970	100.7	6.86	7.19	translation initiation factor IF-2 [Arthrobacter sp. H14]
gi674644635	6.68	3	2	524	50.8	5.00	7.19	hypothetical protein BN1051_00791 [Arthrobacter sp. 11W110_air]
gi636846879	5.22	3	1	345	38.0	7.42	7.19	lysophospholipase [Arthrobacter sp. TB 26]
gi908696949	3.23	1	1	589	63.0	5.39	7.18	hypothetical protein [Arthrobacter sp. RIT-PI-e]
gi651491880	2.46	1	1	570	62.0	6.61	7.18	ABC transporter [Arthrobacter sp. H20]
gi910738448	7.78	2	1	270	28.6	6.14	7.18	probable serine/threonine-protein kinase CPE1738 [Arthrobacter sp. Hiyo4]
gi662109019	11.49	6	2	296	31.6	5.72	7.17	prephenate dehydratase [Streptomyces natalensis]
gi323468810	4.89	2	1	348	37.4	6.01	7.17	L-threonine 3-dehydrogenase [Arthrobacter phenanthrenivorans Sphe3]
gi636845086	3.96	1	1	455	47.5	5.21	7.17	FAD-linked oxidase [Arthrobacter sp. TB 26]
gi757625625	2.75	2	1	255	27.9	8.68	7.17	arginine ABC transporter ATP-binding protein [Arthrobacter sp. SPG23]
gi757623506	7.69	2	1	260	28.2	7.18	7.16	hypothetical protein TV39_15085 [Arthrobacter sp. SPG23]
gi927295070	8.46	3	2	272	28.9	5.21	7.16	hydrolase [Arthrobacter sp. ERGS1:01]
gi654822769	1.81	1	1	496	53.7	6.86	7.15	hypothetical protein [Arthrobacter sp. I3]
gi910746125	14.40	5	1	125	13.1	4.84	7.15	probable NAD-dependent malic enzyme 4 [Arthrobacter sp. Hiyo8]
gi723609043	5.07	1	1	473	50.3	4.84	7.15	ABC-type sugar transport system, periplasmic component [Arthrobacter sp. PAMC25486]
gi651473054	4.83	4	1	373	40.4	6.29	7.15	glycosyl transferase [Arthrobacter nicotinovorans]
gi651472389	2.12	3	1	425	45.0	9.98	7.14	MFS transporter [Arthrobacter nicotinovorans]
gi219858403	2.23	1	1	493	51.8	9.66	7.13	transcriptional regulator, GntR family with aminotransferase domain [Arthrobacter chloroph
gi918449299	4.38	1	1	365	38.9	4.83	7.13	hypothetical protein [Arthrobacter sp. SPG23]
gi652424301	4.89	6	1	327	34.7	9.06	7.13	tyrosine recombinase XerD [Arthrobacter castelli]
gi910249403	5.98	1	1	234	25.6	6.64	7.13	histidine kinase [Arthrobacter siccitolerans]
gi443479672	16.99	4	2	153	15.5	10.87	7.13	hypothetical protein G205_23057 [Arthrobacter nitrophenolicus]
gi737781403	3.09	3	1	712	76.0	5.15	7.13	hypothetical protein [Arthrobacter sp. 35W]
gi476402610	2.50	1	1	360	38.7	5.12	7.12	Holliday junction DNA helicase RuvB [Arthrobacter crystallopoietes BAB-32]
gi937262367	3.87	4	1	284	30.5	9.96	7.12	type II secretion system protein F [Arthrobacter sp. Edens01]
gi635350794	4.82	2	1	477	50.7	5.31	7.11	HAMP domain protein [Arthrobacter siccitolerans]
gi119949389	1.77	2	1	1131	120.7	5.39	7.11	pyruvate carboxylase [Arthrobacter aurescens TC1]
gi476399053	3.86	2	1	544	57.4	5.14	7.10	phosphoglucomutase [Arthrobacter crystallopoietes BAB-32]
gi654817067	10.24	10	1	205	23.7	6.55	7.10	nuclease PIN [Arthrobacter sp. UNC362MFTsu5.1]
gi654826837	3.81	5	1	236	26.2	5.99	7.10	GntR family transcriptional regulator [Arthrobacter sp. H5]
gi918423174	2.46	1	1	285	31.4	8.22	7.10	AsnC family transcriptional regulator, partial [Arthrobacter sp. AK-YN10]
gi737786780	1.33	1	1	675	71.6	10.65	7.10	hypothetical protein [Arthrobacter albus]
gi917739509	2.16	1	1	509	54.0	6.10	7.10	sugar ABC transporter ATPase [Arthrobacter sp. W1]
gi651436988	11.40	4	1	114	12.8	8.21	7.10	ArsR family transcriptional regulator [Arthrobacter sp. H41]
gi723609309	3.08	1	1	422	47.5	4.86	7.10	hypothetical protein ART_3086 [Arthrobacter sp. PAMC25486]
gi651445040	2.31	1	1	476	52.5	5.43	7.10	hypothetical protein [Arthrobacter nicotinovorans]
gi823666980	3.25	4	1	707	74.7	6.99	7.09	nitrite reductase [Arthrobacter sp. YC-RL1]
gi652424573	2.00	8	1	998	111.4	5.31	7.09	alpha-mannosidase [Arthrobacter castelli]
gi651440620	1.99	1	1	755	81.6	6.01	7.08	ATP-dependent DNA helicase RecG [Arthrobacter sp. H14]
gi737790431	7.84	1	1	102	11.6	9.57	7.06	30S ribosomal protein S10 [Arthrobacter albus]
gi551256886	3.55	2	1	507	53.6	5.64	7.06	ATP-binding protein [Arthrobacter sp. PAO19]

gi636844506	8.91	2	2	449	48.6	6.89	7.06	membrane protein [Arthrobacter sp. TB 26]
gi162954797	12.12	4	1	165	17.9	11.19	7.06	CPRD14 protein [Renibacterium salmoninarum ATCC 33209]
gi654822091	3.39	5	1	678	69.5	6.89	7.05	ABC transporter [Arthrobacter sp. I3]
gi651492611	3.44	2	1	581	61.5	4.94	7.05	type IV secretion protein Rhs [Arthrobacter sp. H20]
gi635351885	0.97	4	1	821	89.1	5.69	7.04	hypothetical protein ARTSIC4J27_2201 [Arthrobacter siccitolerans]
gi635352555	4.32	9	1	301	32.4	10.29	7.04	bacterial type II secretion system F domain protein [Arthrobacter siccitolerans]
gi742070667	12.44	3	2	225	24.5	6.80	7.04	putative GntR family transcriptional regulator [Arthrobacter sp. MWB30]
gi927295332	13.25	1	1	151	16.1	9.61	7.04	hypothetical protein AL755_17165 [Arthrobacter sp. ERGS1:01]
gi917442142	6.69	3	1	284	30.9	8.69	7.04	hypothetical protein [Arthrobacter albus]
gi930825585	4.66	4	1	386	41.3	5.39	7.04	phosphoribosylaminoimidazole carboxylase [Arthrobacter arilaitensis]
gi443482459	2.17	2	1	692	74.5	6.95	7.04	acyltransferase [Arthrobacter nitrophenolicus]
gi323469120	2.46	3	1	407	44.1	8.65	7.04	hypothetical protein Asphe3_16360 [Arthrobacter phenanthrenivorans Sphe3]
gi742069024	3.54	1	1	198	20.5	5.40	7.03	N-(5'phosphoribosyl)anthranilate isomerase [Arthrobacter sp. MWB30]
gi918017275	2.33	1	1	857	93.3	5.72	7.03	hypothetical protein [Arthrobacter sp. FB24]
gi517606886	3.63	3	2	743	84.5	5.31	7.02	ATPase AAA [Arthrobacter sp. 161MFSha2.1]
gi636844977	2.21	1	1	589	62.7	8.79	7.02	conjugal transfer protein [Arthrobacter sp. TB 26]
gi918221905	6.28	1	1	207	22.4	5.17	7.02	hypothetical protein [Arthrobacter sp. I3]
gi823666994	6.42	3	1	265	28.3	8.05	7.02	acetyltransferase [Arthrobacter sp. YC-RL1]
gi908699392	2.04	2	1	637	68.7	6.93	7.01	cold-shock protein [Arthrobacter sp. RIT-PI-e]
gi219859512	4.38	1	1	365	39.3	5.24	7.01	biotin/lipoate A/B protein ligase [Arthrobacter chlorophenolicus A6]
gi759730105	8.77	5	1	171	17.5	9.25	7.00	aminoacyl-tRNA deacylase [Arthrobacter sp. L77]
gi518310976	5.17	2	1	271	30.7	11.06	6.99	hypothetical protein [Arthrobacter sp. TB 23]
gi323470024	2.51	2	1	398	41.6	6.13	6.99	hypothetical protein Asphe3_25840 [Arthrobacter phenanthrenivorans Sphe3]
gi551254391	4.17	1	1	264	27.8	5.78	6.99	nitrilase [Arthrobacter sp. PAO19]
gi515767825	1.10	3	1	1003	107.7	5.11	6.99	chromosome segregation protein SMC [Arthrobacter sp. M2012083]
gi116610519	5.35	3	2	505	53.8	5.26	6.99	Aldehyde dehydrogenase (NAD(+)) [Arthrobacter sp. FB24]
gi636844385	8.21	1	1	268	28.8	6.16	6.98	alpha/beta hydrolase [Arthrobacter sp. TB 26]
gi651430494	0.93	1	1	1612	178.7	4.93	6.98	glutamate dehydrogenase [Arthrobacter sanguinis]
gi640204645	1.21	5	1	746	84.2	8.60	6.98	glycosyltransferase [Arthrobacter sp. 31Y]
gi927296550	2.82	7	1	355	40.2	10.45	6.98	delta fatty acid desaturase [Arthrobacter sp. ERGS1:01]
gi749401455	6.30	2	1	238	25.0	6.54	6.97	mercuric reductase, partial [Arthrobacter sp. AK-YN10]
gi742072944	3.55	2	1	394	40.8	7.25	6.97	tRNA(Ile)-lysidine synthetase [Arthrobacter sp. MWB30]
gi914717426	1.51	2	1	729	76.0	5.59	6.96	acetyl-CoA carboxylase [Arthrobacter sp. ZBG10]
gi737789233	1.86	1	1	537	57.9	4.91	6.96	methylmalonyl-CoA carboxyltransferase [Arthrobacter albus]
gi470216926	2.81	1	1	392	41.4	5.99	6.95	penicillin binding protein [Arthrobacter gangotriensis Lz1y]
gi517591434	4.69	2	1	213	23.1	7.43	6.95	HxlR family transcriptional regulator [Arthrobacter sp. 135MFCol5.1]
gi937259160	7.50	3	1	320	34.1	7.84	6.94	glucokinase [Arthrobacter sp. Edens01]
gi307745948	5.66	2	1	459	49.8	9.33	6.94	MFS superfamily transporter [Arthrobacter arilaitensis Re117]
gi648259963	1.50	1	1	599	63.5	6.92	6.94	ABC transporter ATP-binding protein [Arthrobacter sp. TB 23]
gi742859878	23.08	2	2	182	19.2	4.70	6.94	polyisoprenoid-binding protein [Arthrobacter sp. W1]
gi517593656	12.60	6	2	365	39.7	5.71	6.94	hypothetical protein [Arthrobacter sp. 135MFCol5.1]
gi652423784	1.60	1	1	563	59.7	5.88	6.93	formate--tetrahydrofolate ligase [Arthrobacter castelli]
gi759764399	1.49	1	1	537	57.9	4.91	6.93	SAM-dependent methyltransferase [Arthrobacter gangotriensis]
gi651501731	2.40	1	1	668	74.7	5.20	6.93	threonine--tRNA ligase [Arthrobacter sp. 35W]
gi742857921	4.49	1	1	468	48.6	5.17	6.93	3-carboxy-cis,cis-muconate cycloisomerase [Arthrobacter sp. W1]
gi651429303	1.83	2	1	763	74.1	4.96	6.93	hypothetical protein [Arthrobacter sanguinis]
gi654824189	4.37	3	2	915	94.8	7.50	6.92	phosphoenolpyruvate synthase [Arthrobacter sp. I3]
gi742071104	4.63	10	1	216	23.7	9.63	6.92	hypothetical protein ANMWB30_23030 [Arthrobacter sp. MWB30]
gi737789009	5.96	1	1	319	34.6	9.11	6.92	hypothetical protein [Arthrobacter albus]
gi928488915	3.72	6	2	780	83.5	5.07	6.91	hypothetical protein AOC05_13790 [Arthrobacter alpinus]
gi651437047	3.15	2	1	571	60.7	8.13	6.91	hypothetical protein, partial [Arthrobacter sp. H41]
gi640201625	23.53	6	2	136	15.0	9.69	6.91	molecular chaperone DnaJ [Arthrobacter sp. 31Y]
gi162955135	9.21	2	1	228	23.9	9.29	6.91	hypothetical protein RSal33209_2928 [Renibacterium salmoninarum ATCC 33209]
gi542109664	2.48	10	1	886	94.5	5.12	6.91	LuxR family transcriptional regulator [Arthrobacter sp. AK-YN10]
gi359305459	9.23	4	1	130	13.8	5.20	6.91	hypothetical protein ARGLB_064_00080 [Arthrobacter globiformis NBRC 12137]
gi517592522	6.17	1	1	389	39.5	8.16	6.90	sulfonate ABC transporter substrate-binding protein [Arthrobacter sp. 135MFCol5.1]
gi307746510	0.83	3	1	2173	225.2	5.35	6.90	non-ribosomal siderophore peptide synthetase component [Arthrobacter arilaitensis Re117]
gi651438820	2.41	7	1	581	64.1	5.02	6.90	2-isopropylmalate synthase [Arthrobacter sp. H14]

gi162954718	3.30	2	1	424	46.3	8.97	6.90	DNA polymerase IV [Renibacterium salmoninarum ATCC 33209]
gi742070270	5.05	6	1	218	22.9	5.81	6.89	putative oxidoreductase [Arthrobacter sp. MWB30]
gi517604547	2.70	1	1	371	40.3	6.28	6.89	MULTISPECIES: tryptophan--tRNA ligase [Arthrobacter]
gi470217234	1.80	2	1	1169	129.1	5.43	6.88	DNA polymerase III subunit alpha [Arthrobacter gangotriensis Lz1y]
gi116608774	5.39	1	1	167	18.1	11.34	6.88	hypothetical protein Arth_0097 [Arthrobacter sp. FB24]
gi939051220	6.69	3	1	269	29.2	6.23	6.88	ArsR family transcriptional regulator [Arthrobacter sp. JCM 19049]
gi651495900	9.29	2	2	323	34.8	6.54	6.87	hypothetical protein [Arthrobacter sp. H20]
gi737787467	1.90	1	1	897	97.7	5.11	6.87	alanine--tRNA ligase [Arthrobacter albus]
gi742855623	5.00	1	1	320	33.9	5.05	6.86	hydroxymethylglutaryl-CoA lyase [Arthrobacter sp. W1]
gi759729803	7.12	1	1	323	34.3	5.08	6.85	mycothiol acetyltransferase [Arthrobacter sp. L77]
gi917760128	4.04	1	1	396	42.3	9.58	6.85	ATP-grasp domain-containing protein [Arthrobacter sp. L77]
gi359304459	2.66	1	1	602	64.9	5.36	6.85	glucosamine--fructose-6-phosphate aminotransferase [Arthrobacter globiformis NBRC 1213
gi518313671	4.38	6	2	844	89.6	5.48	6.85	excinuclease ABC subunit A [Arthrobacter sp. TB 23]
gi542107351	7.55	1	1	278	28.6	5.71	6.85	flagellin [Arthrobacter sp. AK-YN10]
gi651430353	2.58	3	1	465	51.0	5.31	6.85	adenylosuccinate lyase [Arthrobacter sanguinis]
gi910740555	2.60	1	1	346	37.4	4.81	6.85	xaa-Pro aminopeptidase 1 [Arthrobacter sp. Hiyo4]
gi635350002	1.33	4	1	1658	175.9	5.82	6.85	DEAD/DEAH box helicase family protein [Arthrobacter siccitolerans]
gi651500258	3.74	1	1	294	32.4	7.81	6.85	XRE family transcriptional regulator [Arthrobacter sp. 35W]
gi651439295	6.15	1	1	260	28.4	6.24	6.84	IclR family transcriptional regulator [Arthrobacter sp. H14]
gi917572054	0.92	1	1	1406	152.2	6.07	6.84	DNA helicase [Arthrobacter sp. PAO19]
gi927271625	2.88	1	1	521	56.2	7.21	6.84	nucleotide pyrophosphatase [Arthrobacter sp. LS16]
gi723609187	3.33	2	1	510	55.9	5.58	6.84	UDP-N-acetylglucosamine 1-carboxyvinyltransferase [Arthrobacter sp. PAMC25486]
gi759736215	1.20	1	1	1002	111.1	6.47	6.84	glutamine-synthetase [Arthrobacter sp. L77]
gi917739744	7.58	1	1	211	22.5	11.46	6.84	hypothetical protein [Arthrobacter sp. W1]
gi551254625	12.25	3	2	204	22.1	9.74	6.83	resolvase [Arthrobacter sp. PAO19]
gi916870072	9.42	2	1	191	20.5	6.34	6.83	hypothetical protein [Arthrobacter sp. Br18]
gi652423038	3.31	2	2	937	104.8	6.86	6.83	RNA helicase [Arthrobacter castelli]
gi742072155	7.66	1	1	261	28.5	6.76	6.82	hypothetical protein ANMWB30_08590 [Arthrobacter sp. MWB30]
gi654812731	7.95	3	1	264	26.9	6.07	6.82	3-oxoacyl-ACP reductase [Arthrobacter sp. MA-N2]
gi542109239	15.45	3	1	110	12.1	4.88	6.82	hypothetical protein M707_07265 [Arthrobacter sp. AK-YN10]
gi823665314	1.67	2	1	957	99.7	8.32	6.82	translation initiation factor IF-2 [Arthrobacter sp. YC-RL1]
gi910744585	7.48	1	1	214	23.2	8.91	6.82	hypothetical protein AHiyo8_22870 [Arthrobacter sp. Hiyo8]
gi517600215	1.54	2	1	907	96.2	7.56	6.82	magnesium-transporting ATPase [Arthrobacter sp. 162MFSha1.1]
gi908699543	9.49	4	1	137	15.3	9.85	6.81	hypothetical protein [Arthrobacter sp. RIT-PI-e]
gi517590187	2.89	11	1	346	38.8	5.17	6.80	glyceraldehyde 3-phosphate reductase [Arthrobacter sp. 135MFCol5.1]
gi742758401	10.24	6	1	205	23.4	6.55	6.80	nuclease PIN [Arthrobacter phenanthrenivorans]
gi765010538	5.00	1	1	300	32.5	7.09	6.79	hypothetical protein [Arthrobacter sp. A3]
gi470220706	3.41	2	1	381	42.7	6.38	6.79	UDP-N-acetylglucosamine 2-epimerase [Arthrobacter gangotriensis Lz1y]
gi910740135	14.75	3	1	122	12.9	5.48	6.79	hypothetical protein AHiyo4_29570 [Arthrobacter sp. Hiyo4]
gi742759051	3.18	3	1	409	43.6	9.57	6.78	hypothetical protein RM50_01855 [Arthrobacter phenanthrenivorans]
gi444896989	4.86	1	1	473	46.8	6.05	6.78	Conserved protein [Mycobacterium tuberculosis H37Rv]
gi517599932	2.78	7	1	503	53.7	9.72	6.78	hypothetical protein [Arthrobacter sp. 162MFSha1.1]
gi927295127	5.45	2	2	550	59.3	5.12	6.78	arginyl-tRNA synthetase [Arthrobacter sp. ERGS1:01]
gi470221675	3.11	5	1	386	41.6	8.90	6.78	hypothetical protein ADIAG_00528 [Arthrobacter gangotriensis Lz1y]
gi742072575	8.84	1	1	215	22.5	6.95	6.78	hypothetical protein ANMWB30_04160 [Arthrobacter sp. MWB30]
gi916573746	2.03	1	1	837	89.2	5.30	6.76	hypothetical protein [Arthrobacter sp. TB 26]
gi759722521	3.97	3	1	378	42.8	9.80	6.76	hypothetical protein [Arthrobacter nicotinovorans]
gi517599720	6.54	2	1	321	34.4	4.78	6.75	hypothetical protein [Arthrobacter sp. 162MFSha1.1]
gi927032936	4.01	2	1	399	42.7	5.15	6.75	aspartate aminotransferase [Arthrobacter sp. LS16]
gi517599073	7.65	3	1	183	19.9	9.96	6.74	hypothetical protein [Arthrobacter sp. 162MFSha1.1]
gi910250341	9.58	3	1	167	18.2	7.03	6.74	metal-dependent phosphodiesterase [Arthrobacter siccitolerans]
gi759705075	5.01	2	1	339	35.2	5.07	6.74	hypothetical protein [Arthrobacter globiformis]
gi908697805	1.99	2	1	704	73.9	6.62	6.74	hypothetical protein [Arthrobacter sp. RIT-PI-e]
gi917441993	3.15	2	1	286	32.1	5.24	6.74	chromosome partitioning protein ParB [Arthrobacter albus]
gi918265856	2.56	2	1	469	50.5	8.16	6.73	gamma-glutamylputrescine oxidoreductase [Arthrobacter sp. Hiyo1]
gi307744157	4.81	1	1	208	22.8	5.72	6.73	putative SAM-dependent methyltransferase [Arthrobacter arilaitensis Re117]
gi545109768	5.44	3	1	147	15.5	11.58	6.73	hypothetical protein [Arthrobacter sp. AK-YN10]
gi517606240	2.33	1	1	430	46.6	10.93	6.73	hypothetical protein [Arthrobacter sp. 161MFSha2.1]

gi917529601	1.66	1	1	603	64.6	7.12	6.72	choline transporter [Arthrobacter sp. PAMC25486]
gi908691070	5.00	1	1	280	30.6	8.21	6.72	transcriptional regulator, IclR family protein [Arthrobacter sp. H41]
gi910737271	5.54	2	1	361	40.4	8.65	6.72	4-methylaminobutanoate oxidase (formaldehyde-forming) [Arthrobacter sp. Hiyo4]
gi767256688	5.22	1	1	249	26.5	5.78	6.72	4-hydroxy-tetrahydrodipicolinate reductase [Arthrobacter sp. IHBB 11108]
gi651431339	2.49	3	1	563	62.2	4.92	6.71	ABC transporter substrate-binding protein [Arthrobacter sanguinis]
gi937258050	2.91	2	1	825	87.2	9.14	6.71	hypothetical protein AO716_07995 [Arthrobacter sp. Edens01]
gi910746090	3.18	2	1	440	45.0	11.36	6.71	hypothetical protein AHiyo8_37920 [Arthrobacter sp. Hiyo8]
gi651465402	3.72	1	1	457	50.3	5.43	6.71	serine/threonine protein kinase [Arthrobacter sp. 35/47]
gi517600758	1.43	1	1	1255	132.4	4.96	6.70	urea amidolyase [Arthrobacter sp. 162MFSha1.1]
gi757623334	4.02	1	1	224	24.9	6.42	6.70	Fis family transcriptional regulator [Arthrobacter sp. SPG23]
gi765006911	5.61	2	1	285	31.0	5.26	6.70	ABC transporter ATP-binding protein [Arthrobacter sp. A3]
gi910252726	2.10	1	1	571	61.2	4.98	6.70	ABC transporter substrate-binding protein [Arthrobacter siccitolerans]
gi517608973	6.81	1	1	191	21.3	7.14	6.69	hypothetical protein [Arthrobacter sp. 161MFSha2.1]
gi927294082	3.52	2	2	256	27.8	10.86	6.69	hypothetical protein AL755_08635 [Arthrobacter sp. ERGS1:01]
gi307744162	6.15	2	1	260	28.5	9.52	6.69	putative CAAX amino terminal protease family protein [Arthrobacter arilaitensis Re117]
gi651460251	4.72	1	1	339	36.2	6.00	6.69	hypothetical protein [Arthrobacter sp. 35/47]
gi162952326	13.11	2	2	267	27.1	5.86	6.69	3-oxoacyl-[acyl-carrier protein] reductase [Renibacterium salmoninarum ATCC 33209]
gi914714564	3.63	1	1	248	26.9	8.00	6.69	GCN5 family acetyltransferase [Arthrobacter sp. ZBG10]
gi927295453	4.01	3	1	299	31.5	4.82	6.68	tagatose-bisphosphate aldolase [Arthrobacter sp. ERGS1:01]
gi489895533	4.16	1	1	649	69.9	5.40	6.68	3D-(3,5/4)-trihydroxycyclohexane-1,2-dione acylhydrolase (decyclizing) [Arthrobacter globi[illegible]
gi694033889	4.84	3	1	310	32.4	4.88	6.68	prephenate dehydratase [Rhodococcus fascians]
gi674644707	3.37	2	1	504	51.9	6.86	6.67	C4-dicarboxylate transport protein [Arthrobacter sp. 11W110_air]
gi927031999	4.38	2	1	388	42.8	5.48	6.67	exonuclease SbcD [Arthrobacter sp. LS16]
gi517604021	3.78	1	1	344	35.0	8.81	6.67	hypothetical protein [Arthrobacter sp. 131MFCol6.1]
gi910249631	5.42	1	1	332	37.3	10.49	6.67	DNA topoisomerase [Arthrobacter siccitolerans]
gi542106206	6.50	1	1	200	21.2	4.98	6.66	KfrA protein [Arthrobacter sp. AK-YN10]
gi518311511	3.72	3	2	913	101.5	5.33	6.66	preprotein translocase subunit SecA [Arthrobacter sp. TB 23]
gi116608947	20.00	1	1	85	9.3	5.11	6.66	protein of unknown function DUF156 [Arthrobacter sp. FB24]
gi521093715	0.57	1	1	2270	239.9	4.74	6.66	hypothetical protein [Arthrobacter sp. TB 23]
gi162953935	4.49	2	1	356	38.3	5.22	6.65	myo-inositol-1-phosphate synthase [Renibacterium salmoninarum ATCC 33209]
gi916869737	4.19	2	1	358	39.0	5.12	6.65	cyclase [Arthrobacter sp. Br18]
gi759733845	7.84	1	1	204	21.5	4.97	6.65	carbonic anhydrase [Arthrobacter sp. L77]
gi737796447	3.90	1	1	462	48.3	8.65	6.64	Mg chelatase-like protein, partial [Arthrobacter sp. H20]
gi927296079	1.70	1	1	883	95.7	5.87	6.64	hypothetical protein AL755_21985 [Arthrobacter sp. ERGS1:01]
gi908697704	4.24	1	1	495	52.1	5.34	6.64	hypothetical protein [Arthrobacter sp. RIT-PI-e]
gi737790423	12.82	3	1	156	17.2	10.30	6.64	30S ribosomal protein S7 [Arthrobacter albus]
gi910746984	6.25	4	1	256	27.3	6.38	6.64	dihydrolipoyllysine-residue acyltransferase component of branched-chain alpha-ketoacid de[illegible]
gi162954384	43.40	1	1	53	5.9	9.70	6.64	hypothetical protein RSal33209_2167 [Renibacterium salmoninarum ATCC 33209]
gi928486206	4.64	2	1	280	30.9	9.09	6.63	hypothetical protein AOC05_02990 [Arthrobacter alpinus]
gi189045002	4.42	1	1	294	32.4	5.58	6.63	RecName: Full=30S ribosomal protein S2
gi742852018	2.00	12	1	1151	125.8	5.03	6.63	nuclease [Arthrobacter sp. W1]
gi823666144	5.99	2	1	367	39.3	4.75	6.63	transaldolase [Arthrobacter sp. YC-RL1]
gi742070360	3.53	2	1	425	45.4	6.54	6.63	UDP-N-acetylglucosamine 1-carboxyvinyltransferase MurA [Arthrobacter sp. MWB30]
gi651430091	10.43	1	1	115	12.8	9.35	6.63	hypothetical protein [Arthrobacter sanguinis]
gi939036376	19.70	1	1	66	7.2	9.14	6.63	hypothetical protein [Arthrobacter nitroguajacolicus]
gi765007308	1.89	1	1	581	61.7	6.65	6.63	multidrug ABC transporter ATPase [Arthrobacter sp. A3]
gi654827088	11.54	3	2	130	14.5	6.01	6.62	MerR family transcriptional regulator [Arthrobacter sp. H5]
gi651476491	4.82	2	1	353	37.7	10.08	6.62	FAD-binding monooxygenase [Arthrobacter nicotinovorans]
gi674644662	1.34	1	1	895	96.1	6.34	6.62	DNA-binding transcriptional activator UhpA [Arthrobacter sp. 11W110_air]
gi162955217	2.46	2	1	488	53.0	4.88	6.62	adenosylhomocysteinase [Renibacterium salmoninarum ATCC 33209]
gi930824978	2.08	2	1	530	57.4	5.72	6.62	hypothetical protein AOZ07_00785 [Arthrobacter arilaitensis]
gi917013476	4.13	3	1	460	49.3	6.27	6.61	AAA family ATPase [Arthrobacter sanguinis]
gi737797899	2.75	2	1	255	28.2	5.96	6.61	GlcNAc-PI de-N-acetylase [Arthrobacter sp. H20]
gi760112804	7.81	2	1	333	35.1	4.97	6.61	flagellar motor switch protein FliG [Arthrobacter chlorophenolicus]
gi489894054	2.33	1	1	472	51.7	5.76	6.61	FMNH2-dependent monooxygenase [Arthrobacter globiformis]
gi760112933	1.65	1	1	967	105.3	5.19	6.61	glycoside hydrolase [Arthrobacter chlorophenolicus]
gi476401670	4.65	1	1	387	40.9	4.81	6.60	succinyl-CoA synthetase subunit beta [Arthrobacter crystallopoietes BAB-32]
gi910748020	5.02	1	1	219	23.1	6.38	6.60	exonuclease [Arthrobacter sp. Hiyo8]

gi910250248	1.84	4	1	490	54.5	6.19	6.60	hypothetical protein [Arthrobacter siccitolerans]
gi636846174	11.79	3	2	212	22.9	5.41	6.59	glycosyltransferase [Arthrobacter sp. TB 26]
gi908690610	2.00	1	1	800	86.5	6.04	6.59	xanthine dehydrogenase [Arthrobacter sp. H41]
gi910249348	2.21	3	1	408	42.3	6.09	6.58	beta-ketoadipyl CoA thiolase [Arthrobacter siccitolerans]
gi765010970	2.93	1	1	512	55.8	6.90	6.58	ABC transporter ATP-binding protein [Arthrobacter sp. A3]
gi695210478	6.70	2	1	209	22.6	9.88	6.58	DNA invertase-like protein (plasmid) [Arthrobacter aurescens]
gi723607501	2.76	1	1	434	48.4	5.31	6.58	hypothetical protein ART_1278 [Arthrobacter sp. PAMC25486]
gi162955570	6.91	4	1	246	25.9	5.40	6.57	phosphoribosylformylglycinamidine synthase [Renibacterium salmoninarum ATCC 33209]
gi307744573	3.74	2	2	642	67.6	5.27	6.57	dihydroxy-acid dehydratase [Arthrobacter arilaitensis Re117]
gi914714264	1.86	4	1	483	50.4	9.47	6.57	histidine kinase [Arthrobacter sp. ZBG10]
gi767257814	4.03	2	1	496	53.9	6.01	6.57	hypothetical protein UM93_09560 [Arthrobacter sp. IHBB 11108]
gi219858864	4.74	2	1	443	46.2	5.05	6.57	pyrimidine-nucleoside phosphorylase [Arthrobacter chlorophenolicus A6]
gi162953241	3.44	1	1	436	45.9	7.81	6.56	ABC-type sugar transporter, periplasmic component [Renibacterium salmoninarum ATCC 3
gi927296054	2.25	1	1	845	92.3	6.27	6.56	metallophosphoesterase [Arthrobacter sp. ERGS1:01]
gi916926231	3.01	2	1	399	40.3	11.41	6.56	MFS transporter [Arthrobacter sp. 9MFCol3.1]
gi742757181	3.76	1	1	479	50.3	5.47	6.56	succinate-semialdehyde dehydrogenase [Arthrobacter phenanthrenivorans]
gi651461830	2.59	2	1	501	54.2	5.53	6.55	sugar ABC transporter ATP-binding protein [Arthrobacter sp. 35/47]
gi517604317	2.09	1	1	526	55.4	5.57	6.55	hypothetical protein [Arthrobacter sp. 131MFCol6.1]
gi651456989	2.84	1	1	493	53.6	9.29	6.55	ATP-binding protein [Arthrobacter sp. 35/47]
gi767257965	2.98	3	1	302	32.7	7.58	6.55	hypothetical protein UM93_10575 [Arthrobacter sp. IHBB 11108]
gi759771944	6.36	4	1	236	25.4	9.51	6.55	alkaline phosphatase [Arthrobacter sp. SPG23]
gi589247822	3.81	2	1	367	38.8	6.92	6.53	opine dehydrogenase (plasmid) [Sinorhizobium meliloti RU11/001]
gi654825545	2.84	1	1	423	45.5	6.19	6.53	hypothetical protein [Arthrobacter sp. H5]
gi937259058	5.99	1	1	434	45.0	6.54	6.52	sodium:proton antiporter [Arthrobacter sp. Edens01]
gi636846649	3.55	2	1	620	67.0	9.70	6.52	hypothetical protein [Arthrobacter sp. TB 26]
gi918265741	3.08	3	1	422	45.1	9.33	6.52	uncharacterized protein MJ1024 [Arthrobacter sp. Hiyo1]
gi786028933	3.27	2	2	918	98.2	6.27	6.52	phosphoenolpyruvate synthase [Arthrobacter chlorophenolicus]
gi759726947	3.70	1	1	487	50.8	5.78	6.52	endonuclease [Arthrobacter sp. UNC362MFTsu5.1]
gi551255105	3.96	2	1	303	34.2	9.25	6.51	transposase IS401 [Arthrobacter sp. PAO19]
gi916820519	7.07	3	1	184	19.6	8.21	6.51	hypothetical protein [Arthrobacter sp. H20]
gi636844728	2.21	1	1	543	57.4	6.89	6.51	two-component system sensor histidine kinase [Arthrobacter sp. TB 26]
gi651430891	2.58	3	1	542	60.5	5.95	6.51	Pup deamidase/depupylase [Arthrobacter sanguinis]
gi170783606	11.76	1	1	102	11.4	10.35	6.50	hypothetical protein (plasmid) [Arthrobacter sp. Chr15]
gi918265712	6.95	1	1	331	35.3	8.97	6.50	transcriptional regulator LsrR [Arthrobacter sp. Hiyo1]
gi916815848	3.91	3	1	409	45.4	9.89	6.50	sugar translocase [Arthrobacter sp. MA-N2]
gi470217266	1.21	1	1	912	101.8	5.48	6.50	preprotein translocase subunit SecA [Arthrobacter gangotriensis Lz1y]
gi651429596	18.35	1	1	109	12.1	11.18	6.50	MULTISPECIES: sulfate permease [Actinobacteria]
gi917021979	9.52	2	1	189	20.1	5.12	6.50	hypothetical protein [Arthrobacter sp. UNC362MFTsu5.1]
gi654815711	5.14	2	1	311	33.6	9.73	6.50	hypothetical protein [Arthrobacter sp. UNC362MFTsu5.1]
gi737778385	2.31	3	1	607	65.8	5.25	6.50	peptide ABC transporter ATPase [Arthrobacter sanguinis]
gi652424458	2.96	3	1	507	54.1	7.33	6.49	hypothetical protein [Arthrobacter castelli]
gi470217081	2.27	2	1	485	53.5	6.73	6.49	hypothetical protein ADIAG_02842 [Arthrobacter gangotriensis Lz1y]
gi517609751	3.60	1	1	445	49.9	5.19	6.49	alpha-L-fucosidase [Arthrobacter sp. 161MFSha2.1]
gi219862125	2.00	1	1	449	48.4	5.48	6.49	FAD-dependent pyridine nucleotide-disulphide oxidoreductase (plasmid) [Arthrobacter chlo
gi651443005	2.57	4	1	272	29.7	9.83	6.48	hypothetical protein [Arthrobacter sp. 9MFCol3.1]
gi767257805	4.13	1	1	315	31.6	5.08	6.48	peptidase S58 [Arthrobacter sp. IHBB 11108]
gi930827149	3.82	1	1	340	36.6	8.28	6.48	hypothetical protein AOZ07_13260 [Arthrobacter arilaitensis]
gi917760347	1.82	3	1	604	63.6	5.29	6.47	ABC transporter [Arthrobacter sp. L77]
gi742755152	6.99	1	1	286	29.6	6.93	6.47	shikimate dehydrogenase [Arthrobacter phenanthrenivorans]
gi918268728	17.24	1	1	116	11.9	8.21	6.47	6-phosphofructokinase 1 [Arthrobacter sp. Hiyo1]
gi916870042	2.89	3	1	658	70.1	6.51	6.47	hypothetical protein [Arthrobacter sp. Br18]
gi742756618	2.67	1	1	337	35.4	9.47	6.47	hypothetical protein RM50_09290 [Arthrobacter phenanthrenivorans]
gi910738648	2.91	1	1	446	46.7	5.85	6.47	NAD(P)H dehydrogenase (quinone) [Arthrobacter sp. Hiyo4]
gi908698234	9.20	1	1	250	26.2	4.73	6.46	1-(5-phosphoribosyl)-5-[(5-phosphoribosylamino)methylideneamino] imidazole-4-carboxam
gi359305402	8.16	2	1	331	34.2	7.85	6.46	putative ABC transporter substrate-binding protein [Arthrobacter globiformis NBRC 12137]
gi551256434	1.73	1	1	1271	142.8	6.00	6.46	hypothetical protein [Arthrobacter sp. PAO19]
gi652423220	1.22	3	1	984	103.8	5.36	6.46	oxidoreductase [Arthrobacter castelli]
gi654814757	4.26	1	1	399	42.8	6.23	6.46	hypothetical protein [Arthrobacter sp. MA-N2]

gi119949256	5.22	1	1	268	29.6	9.55	6.46	glycosyl transferase, group 2 family protein [Arthrobacter aurescens TC1]
gi917572065	1.20	1	1	833	89.8	8.72	6.46	penicillin-binding protein [Arthrobacter sp. PAO19]
gi517592650	10.00	2	2	330	35.8	5.69	6.45	hypothetical protein [Arthrobacter sp. 135MFCol5.1]
gi542109612	2.94	1	1	477	49.3	9.70	6.45	sodium:proton antiporter [Arthrobacter sp. AK-YN10]
gi648224648	18.27	1	1	104	11.9	5.10	6.44	hypothetical protein [Arthrobacter sp. M2012083]
gi916781944	3.31	3	1	393	41.3	5.85	6.44	SAM-dependent methyltransferase [Arthrobacter sp. 35W]
gi476399575	2.14	4	1	701	76.4	6.55	6.44	acyl-CoA oxidase domain-containing protein [Arthrobacter crystallopoietes BAB-32]
gi476400036	4.20	2	2	547	58.9	5.17	6.44	arginyl-tRNA ligase [Arthrobacter crystallopoietes BAB-32]
gi765004628	4.13	1	1	484	52.3	5.87	6.44	glutamate synthase [Arthrobacter sp. A3]
gi767258678	8.99	3	2	378	41.1	7.55	6.44	hypothetical protein UM93_15655 [Arthrobacter sp. IHBB 11108]
gi930825299	4.13	1	1	339	36.4	5.73	6.44	LacI family transcriptional regulator [Arthrobacter arilaitensis]
gi470217563	2.95	3	1	407	44.4	4.92	6.44	Recombinational DNA repair ATPase (RecF pathway) [Arthrobacter gangotriensis Lz1y]
gi767259345	1.87	2	1	587	63.4	5.52	6.44	hypothetical protein UM93_16710 [Arthrobacter sp. IHBB 11108]
gi651492702	12.41	2	1	137	15.5	6.79	6.43	acetyltransferase [Arthrobacter sp. H20]
gi737811349	7.94	1	1	214	23.0	5.44	6.43	SAM-dependent methyltransferase [Arthrobacter sp. 35/47]
gi737786458	13.41	1	1	82	9.1	4.07	6.43	acyl carrier protein [Arthrobacter albus]
gi654816132	4.72	3	1	212	23.2	5.57	6.43	TetR family transcriptional regulator [Arthrobacter sp. UNC362MFTsu5.1]
gi470217420	5.15	4	1	408	44.4	6.16	6.43	tellurite resistance protein [Arthrobacter gangotriensis Lz1y]
gi916813860	5.32	1	1	263	28.6	5.95	6.43	methyltransferase type 11 [Arthrobacter nicotinovorans]
gi476401847	3.86	1	1	337	36.9	7.20	6.43	ATP dependent DNA ligase [Arthrobacter crystallopoietes BAB-32]
gi359305373	5.07	1	1	454	48.0	5.97	6.43	hypothetical protein ARGLB_069_00330 [Arthrobacter globiformis NBRC 12137]
gi928488366	2.18	1	1	550	56.9	5.06	6.43	hypothetical protein AOC05_17500 [Arthrobacter alpinus]
gi323471533	4.10	1	1	390	42.9	5.39	6.42	MoxR-like ATPase (plasmid) [Arthrobacter phenanthrenivorans Sphe3]
gi651439181	6.67	1	1	330	35.5	4.86	6.42	pyruvate dehydrogenase [Arthrobacter sp. H14]
gi654823259	1.36	1	1	880	94.7	6.25	6.42	phosphoenolpyruvate synthase [Arthrobacter sp. I3]
gi443483093	4.33	1	1	323	35.6	10.27	6.42	hypothetical protein G205_02093 [Arthrobacter nitrophenolicus]
gi759765729	3.29	2	1	426	44.8	7.36	6.41	hypothetical protein [Arthrobacter gangotriensis]
gi323468157	5.22	1	1	402	43.8	5.17	6.41	acyl-CoA dehydrogenase [Arthrobacter phenanthrenivorans Sphe3]
gi742859500	5.68	1	1	229	25.3	6.44	6.41	hypothetical protein [Arthrobacter sp. W1]
gi476398815	5.94	4	1	303	31.8	6.96	6.41	ABC transporter ATP-binding subunit, partial [Arthrobacter crystallopoietes BAB-32]
gi916835143	2.61	1	1	459	50.2	4.89	6.41	hypothetical protein [Arthrobacter sp. H14]
gi403311742	1.89	1	1	1166	128.8	5.21	6.41	putative ATP-binding protein (plasmid) [Arthrobacter sp. Rue61a]
gi937257983	1.12	1	1	891	95.4	6.35	6.40	hypothetical protein AO716_07565 [Arthrobacter sp. Edens01]
gi769942675	6.63	1	1	166	18.8	9.14	6.40	hypothetical protein [Arthrobacter sp. IHBB 11108]
gi443482219	3.96	3	1	328	33.9	9.69	6.40	ABC transporter [Arthrobacter nitrophenolicus]
gi908698983	9.15	1	1	153	17.3	10.29	6.40	cyclase [Arthrobacter sp. RIT-PI-e]
gi219860869	1.63	1	1	796	84.8	5.06	6.40	Beta-galactosidase [Arthrobacter chlorophenolicus A6]
gi930826490	2.03	3	1	1086	120.6	5.08	6.40	isoleucine--tRNA ligase [Arthrobacter arilaitensis]
gi742759305	2.57	5	1	545	62.1	6.40	6.39	sugar phosphotransferase [Arthrobacter phenanthrenivorans]
gi654822024	1.69	3	1	712	74.0	6.49	6.39	potassium transporter KtrB [Arthrobacter sp. I3]
gi918266794	2.66	2	1	602	66.0	5.20	6.39	glutamate synthase [NADPH] large chain [Arthrobacter sp. Hiyo1]
gi937262204	2.97	4	1	437	46.0	5.21	6.39	hypothetical protein AO716_03050 [Arthrobacter sp. Edens01]
gi937262087	5.88	2	1	391	42.0	5.76	6.39	hypothetical protein AO716_02400 [Arthrobacter sp. Edens01]
gi307743903	13.06	3	2	245	27.1	5.64	6.39	hypothetical protein AARI_06470 [Arthrobacter arilaitensis Re117]
gi551255005	7.92	1	1	202	23.0	4.86	6.38	hypothetical protein [Arthrobacter sp. PAO19]
gi636844298	6.71	1	1	343	35.8	5.38	6.38	NADP-dependent oxidoreductase [Arthrobacter sp. TB 26]
gi928488917	12.63	1	1	95	10.3	4.96	6.38	glutaredoxin [Arthrobacter alpinus]
gi937258057	1.43	1	1	1333	142.8	6.10	6.38	type VII secretion protein EccCa [Arthrobacter sp. Edens01]
gi663222902	4.22	1	1	308	32.9	5.41	6.37	prephenate dehydratase [Streptomyces violens]
gi737792924	5.14	1	1	311	33.6	9.51	6.37	recombinase XerC [Arthrobacter nicotinovorans]
gi542108530	2.68	1	1	447	47.9	4.89	6.37	zinc protease [Arthrobacter sp. AK-YN10]
gi910249655	5.02	1	1	458	48.9	9.61	6.37	ATPase [Arthrobacter siccitolerans]
gi489901550	8.48	1	1	224	24.8	5.41	6.37	DNA-binding response regulator [Arthrobacter globiformis]
gi937262349	2.75	1	1	436	45.0	5.21	6.37	chromosome partitioning protein [Arthrobacter sp. Edens01]
gi651430437	1.64	1	1	488	53.3	5.03	6.37	ribonuclease II [Arthrobacter sanguinis]
gi914714772	8.00	1	1	200	18.8	8.88	6.37	hypothetical protein [Arthrobacter sp. ZBG10]
gi651497543	7.24	2	1	221	24.7	5.91	6.37	hypothetical protein [Arthrobacter sp. 35W]
gi119950568	1.96	1	1	1023	110.7	9.47	6.37	Tn3 family transposase [Arthrobacter aurescens TC1]

gi651503072	8.04	1	1	199	21.8	8.44	6.37	PadR family transcriptional regulator [Arthrobacter sp. 35W]
gi651430622	1.40	1	1	857	89.4	4.94	6.36	hypothetical protein [Arthrobacter sanguinis]
gi690772042	1.22	1	1	1151	123.9	5.17	6.36	pyruvate carboxylase [Arthrobacter albus DNF00011]
gi651502735	1.66	1	1	1026	104.1	5.01	6.36	hypothetical protein [Arthrobacter sp. 35W]
gi116611942	2.78	2	1	576	61.8	6.57	6.36	ResB family protein [Arthrobacter sp. FB24]
gi927294230	3.89	1	1	386	39.0	6.06	6.36	hypothetical protein AL755_09645 [Arthrobacter sp. ERGS1:01]
gi654816799	4.57	1	1	438	45.5	4.83	6.36	homoserine dehydrogenase [Arthrobacter sp. UNC362MFTsu5.1]
gi651493711	2.02	3	1	642	71.7	5.05	6.36	histamine oxidase [Arthrobacter sp. H20]
gi518312933	2.27	1	1	616	66.8	8.72	6.35	MULTISPECIES: ABC transporter [Arthrobacter]
gi162952796	8.11	1	1	259	26.3	5.02	6.35	dethiobiotin synthetase [Renibacterium salmoninarum ATCC 33209]
gi910740800	6.95	2	1	259	28.0	10.65	6.35	protein involved in peptidyl-histidine phosphorylation [Arthrobacter sp. Hiyo4]
gi759708963	1.68	2	1	715	77.0	5.03	6.35	diguanylate cyclase [Arthrobacter sp. 9MFCol3.1]
gi443480776	1.85	1	1	702	76.5	7.08	6.35	acyl-CoA oxidase domain-containing protein [Arthrobacter nitrophenolicus]
gi917441950	12.20	2	1	123	13.0	9.98	6.35	GntR family transcriptional regulator [Arthrobacter albus]
gi759732475	0.66	2	1	1669	177.4	5.55	6.34	DEAD/DEAH box helicase [Arthrobacter sp. L77]
gi916871723	4.83	1	1	290	29.6	10.35	6.34	hypothetical protein [Arthrobacter sp. H5]
gi476400874	2.29	2	1	436	46.6	6.57	6.34	geranylgeranyl reductase [Arthrobacter crystallopoietes BAB-32]
gi723607483	1.03	2	1	1266	142.9	6.23	6.34	hypothetical protein ART_1260 [Arthrobacter sp. PAMC25486]
gi927032288	2.40	4	1	667	73.5	5.49	6.33	hypothetical protein AFL94_08245 [Arthrobacter sp. LS16]
gi515766740	3.57	1	1	392	42.2	4.79	6.32	cyclase [Arthrobacter sp. M2012083]
gi916691310	2.47	2	1	730	78.7	5.60	6.32	hypothetical protein [Arthrobacter castelli]
gi551256863	6.91	4	1	217	23.1	5.08	6.32	thymidylate kinase [Arthrobacter sp. PAO19]
gi917441796	4.68	1	1	278	29.3	9.35	6.31	hypothetical protein [Arthrobacter albus]
gi917759978	5.39	2	1	297	32.2	4.60	6.31	hypothetical protein [Arthrobacter sp. L77]
gi918267121	2.30	1	1	435	44.4	8.95	6.30	3-carboxy-cis,cis-muconate cycloisomerase [Arthrobacter sp. Hiyo1]
gi651438938	2.00	5	1	551	59.2	5.05	6.30	urocanate hydratase [Arthrobacter sp. H14]
gi760164809	14.29	6	1	126	14.3	4.65	6.30	hypothetical protein [Arthrobacter crystallopoietes]
gi651436973	1.99	1	1	704	75.9	6.19	6.30	ATP-dependent DNA helicase RecQ [Arthrobacter sp. H41]
gi476402702	4.26	1	1	235	25.2	5.20	6.30	signal peptidase I [Arthrobacter crystallopoietes BAB-32]
gi651476397	3.74	1	1	348	36.2	9.91	6.30	ABC transporter permease [Arthrobacter nicotinovorans]
gi742072644	3.25	2	1	431	46.0	8.13	6.30	HNH endonuclease domain protein [Arthrobacter sp. MWB30]
gi742070366	3.28	2	1	579	61.5	5.63	6.30	ATP/GTP-binding protein [Arthrobacter sp. MWB30]
gi651435241	1.59	1	1	694	74.2	6.25	6.30	daunorubicin resistance protein DrrC, partial [Arthrobacter sp. H41]
gi742757292	4.42	1	1	294	31.9	5.15	6.29	hypothetical protein RM50_07095 [Arthrobacter phenanthrenivorans]
gi219862222	2.91	4	1	344	36.8	6.55	6.29	transcriptional regulator, LacI family (plasmid) [Arthrobacter chlorophenolicus A6]
gi767256898	4.13	2	1	436	46.7	6.27	6.29	SAM-dependent methyltransferase [Arthrobacter sp. IHBB 11108]
gi116610266	6.83	1	1	249	26.9	5.68	6.28	amino acid ABC transporter ATP-binding protein, PAAT family [Arthrobacter sp. FB24]
gi767257313	3.19	1	1	313	34.2	6.62	6.28	LysR family transcriptional regulator [Arthrobacter sp. IHBB 11108]
gi759704131	5.15	1	1	388	40.6	7.91	6.28	hypothetical protein [Arthrobacter globiformis]
gi651493539	4.31	1	1	209	22.3	8.56	6.27	membrane protein [Arthrobacter sp. H20]
gi930827180	10.26	1	1	195	21.8	5.66	6.27	hypothetical protein AOZ07_13435 [Arthrobacter arilaitensis]
gi518311944	5.62	1	1	338	36.7	5.15	6.27	hypothetical protein [Arthrobacter sp. TB 23]
gi823666358	4.81	1	1	437	46.2	5.16	6.27	glutamyl-tRNA reductase [Arthrobacter sp. YC-RL1]
gi651445336	4.68	1	1	427	47.5	5.12	6.26	FAD-binding protein [Arthrobacter nicotinovorans]
gi323469646	10.85	2	1	212	23.5	9.54	6.26	integrase family protein [Arthrobacter phenanthrenivorans Sphe3]
gi786027975	6.31	3	2	523	56.4	5.35	6.26	GTP-binding protein [Arthrobacter chlorophenolicus]
gi323470166	3.39	1	1	413	43.4	5.90	6.26	hypothetical protein Asphe3_27320 [Arthrobacter phenanthrenivorans Sphe3]
gi927271451	1.21	1	1	1074	118.9	5.17	6.26	hypothetical protein [Arthrobacter sp. LS16]
gi359307802	3.99	2	1	426	46.6	5.80	6.26	ABC transporter substrate-binding protein [Arthrobacter globiformis NBRC 12137]
gi652424923	3.39	2	1	354	38.2	4.88	6.25	hypothetical protein [Arthrobacter castelli]
gi652424912	2.46	2	1	284	30.5	10.71	6.25	type II secretion system protein F [Arthrobacter castelli]
gi651452979	4.80	1	1	354	39.9	5.64	6.25	ATP-dependent DNA ligase [Arthrobacter nicotinovorans]
gi937259314	0.84	1	1	1197	127.6	5.14	6.24	chromosome segregation protein SMC [Arthrobacter sp. Edens01]
gi651494556	24.72	2	2	89	10.5	10.81	6.24	30S ribosomal protein S15 [Arthrobacter sp. H20]
gi651485830	4.80	6	1	458	48.7	6.01	6.24	6-phospho-beta-glucosidase [Arthrobacter sp. Br18]
gi914715519	2.16	2	1	742	81.0	5.59	6.23	AAA family ATPase [Arthrobacter sp. ZBG10]
gi910251760	5.83	5	1	326	34.3	5.44	6.23	hypothetical protein [Arthrobacter siccitolerans]
gi219859186	6.57	3	1	289	31.0	8.54	6.23	DNA-formamidopyrimidine glycosylase [Arthrobacter chlorophenolicus A6]

gi116609504	3.82	1	1	314	32.1	5.72	6.23	monosaccharide ABC transporter substrate-binding protein, CUT2 family [Arthrobacter sp. I
gi476399508	1.04	6	1	1634	180.0	5.85	6.23	putative helicase [Arthrobacter crystallopoietes BAB-32]
gi651437121	5.94	2	2	707	75.5	5.52	6.23	3-hydroxyacyl-CoA dehydrogenase [Arthrobacter sp. H41]
gi517591630	6.04	3	1	265	27.4	11.27	6.23	hypothetical protein [Arthrobacter sp. 135MFCol5.1]
gi916357086	4.80	3	1	271	29.3	10.35	6.23	ABC transporter [Arthrobacter sp. 162MFSha1.1]
gi323467957	4.27	2	1	281	30.6	5.24	6.23	methylase involved in ubiquinone/menaquinone biosynthesis [Arthrobacter phenanthrenivo
gi916820302	3.65	1	1	493	52.0	5.17	6.23	amidase [Arthrobacter sp. H20]
gi916813797	1.65	1	1	665	70.8	5.15	6.23	hypothetical protein [Arthrobacter nicotinovorans]
gi759736723	8.79	1	1	182	20.2	5.41	6.22	deaminase/reductase [Arthrobacter sp. L77]
gi648572902	2.51	2	1	558	60.3	5.22	6.22	histidine kinase [Arthrobacter sp. 135MFCol5.1]
gi674645527	1.39	1	1	719	73.4	5.40	6.22	Potassium-transporting ATPase B chain [Arthrobacter sp. 11W110_air]
gi651482249	2.88	1	1	660	69.6	4.97	6.21	hypothetical protein [Arthrobacter sp. Br18]
gi759732536	7.56	2	1	291	31.0	5.21	6.21	hypothetical protein [Arthrobacter sp. L77]
gi651435100	3.55	1	1	394	40.5	4.59	6.20	cell division protein FtsZ [Arthrobacter sp. H41]
gi116611217	10.26	1	1	156	16.8	8.24	6.20	transcriptional regulator, AsnC family [Arthrobacter sp. FB24]
gi910747123	4.96	2	1	282	30.0	5.20	6.20	probable aldehyde dehydrogenase [Arthrobacter sp. Hiyo8]
gi759715535	13.42	2	2	149	15.3	5.00	6.20	phosphoribosylaminoimidazole carboxylase, partial [Arthrobacter sp. AK-YN10]
gi910749243	4.13	2	1	412	46.3	9.23	6.19	transposase for transposon Tn1546 (plasmid) [Arthrobacter sp. Hiyo8]
gi910748403	4.35	1	1	299	31.9	11.11	6.19	glutamyl-tRNA(Gln) amidotransferase subunit A [Arthrobacter sp. Hiyo8]
gi654816140	7.01	1	1	314	31.4	9.64	6.19	lipase [Arthrobacter sp. UNC362MFTsu5.1]
gi518312918	0.91	2	1	1315	145.5	7.24	6.18	ATP-dependent helicase [Arthrobacter sp. TB 23]
gi910249122	2.83	1	1	637	70.7	6.20	6.18	hypothetical protein [Arthrobacter siccitolerans]
gi916820393	4.61	1	1	347	36.2	8.53	6.18	hypothetical protein [Arthrobacter sp. H20]
gi476402122	4.47	1	1	447	47.3	5.62	6.18	sulfate adenylyltransferase subunit 1 [Arthrobacter crystallopoietes BAB-32]
gi749402497	2.79	2	1	430	47.0	5.26	6.18	elongation factor G, partial [Arthrobacter sp. AK-YN10]
gi651440051	1.63	1	1	430	47.4	10.55	6.17	hypothetical protein [Arthrobacter sp. H14]
gi914716441	2.02	1	1	495	52.0	10.51	6.17	hypothetical protein [Arthrobacter sp. ZBG10]
gi918266157	7.10	1	1	352	39.4	9.32	6.17	uncharacterized protein Rv2248/MT2308 [Arthrobacter sp. Hiyo1]
gi742069369	4.60	1	1	413	41.9	10.98	6.17	major facilitator transporter [Arthrobacter sp. MWB30]
gi651434620	4.89	2	1	348	37.2	7.39	6.16	epimerase [Arthrobacter sp. H41]
gi323467809	3.47	1	1	346	37.9	9.92	6.16	hypothetical protein Asphe3_02760 [Arthrobacter phenanthrenivorans Sphe3]
gi651442448	3.19	1	1	407	41.8	5.66	6.16	molybdopterin molybdenumtransferase MoeA [Arthrobacter sp. 9MFCol3.1]
gi651439184	9.85	1	1	132	14.8	7.71	6.16	hypothetical protein [Arthrobacter sp. H14]
gi403228029	4.92	1	1	305	32.3	5.95	6.16	hypothetical protein ARUE_c05160 [Arthrobacter sp. Rue61a]
gi906448091	5.65	1	1	230	24.2	5.15	6.16	hypothetical protein AC792_03630 [Arthrobacter sp. RIT-PI-e]
gi759735032	3.01	1	1	399	41.5	7.52	6.16	DNA processing protein DprA [Arthrobacter sp. L77]
gi470221479	1.59	2	1	502	52.8	8.06	6.16	sulfate transporter [Arthrobacter gangotriensis Lz1y]
gi651462992	5.34	1	1	206	22.5	9.58	6.16	resolvase [Arthrobacter sp. 35/47]
gi767258999	1.46	1	1	755	84.6	8.60	6.16	GTP pyrophosphokinase [Arthrobacter sp. IHBB 11108]
gi551255793	6.54	1	1	321	35.0	6.58	6.16	ArsR family transcriptional regulator [Arthrobacter sp. PAO19]
gi470220272	2.27	1	1	704	81.0	5.91	6.15	hypothetical protein ADIAG_01109 [Arthrobacter gangotriensis Lz1y]
gi910741227	14.88	1	1	168	18.8	7.34	6.15	phosphoenolpyruvate synthase [Arthrobacter sp. Hiyo4]
gi917746147	3.00	2	1	400	40.6	10.30	6.15	MFS transporter [Arthrobacter phenanthrenivorans]
gi307745998	4.38	1	1	320	34.5	10.20	6.15	hypothetical secreted protein [Arthrobacter arilaitensis Re117]
gi910250144	3.42	2	1	322	35.5	8.76	6.15	formamidopyrimidine-DNA glycosylase [Arthrobacter siccitolerans]
gi651486577	4.50	2	1	333	36.2	6.73	6.14	glycosyl transferase [Arthrobacter sp. Br18]
gi551255676	3.49	2	1	344	36.5	6.55	6.13	LacI family transcriptional regulator [Arthrobacter sp. PAO19]
gi737771569	15.08	1	1	126	14.1	10.15	6.13	hypothetical protein [Arthrobacter sp. TB 26]
gi737788785	5.69	1	1	281	29.9	5.12	6.13	aldolase [Arthrobacter albus]
gi162952450	2.91	1	1	309	33.9	8.84	6.13	transcriptional regulator, LysR family [Renibacterium salmoninarum ATCC 33209]
gi359305370	5.25	1	1	457	48.8	5.34	6.13	glutamine synthetase [Arthrobacter globiformis NBRC 12137]
gi910250916	1.72	1	1	1163	119.3	9.26	6.13	ABC transporter [Arthrobacter siccitolerans]
gi759731318	3.10	1	1	355	40.4	6.73	6.13	radical SAM protein [Arthrobacter sp. L77]
gi359304272	8.51	1	1	235	25.0	5.85	6.12	hypothetical protein ARGLB_085_01360 [Arthrobacter globiformis NBRC 12137]
gi759731065	4.93	1	1	304	33.9	6.14	6.12	NUDIX hydrolase [Arthrobacter sp. L77]
gi927269835	2.79	1	1	573	60.1	7.11	6.12	hypothetical protein [Arthrobacter sp. LS16]
gi759735385	1.99	1	1	503	52.9	10.59	6.12	hypothetical protein [Arthrobacter sp. L77]
gi654813908	6.03	1	1	232	25.4	9.51	6.12	ABC transporter ATP-binding protein [Arthrobacter sp. MA-N2]

gi654815117	3.99	1	1	401	43.8	5.02	6.12	acyl-CoA dehydrogenase [Arthrobacter sp. PAO19]
gi927293158	3.42	1	1	497	54.9	4.77	6.11	hypothetical protein AL755_02975 (plasmid) [Arthrobacter sp. ERGS1:01]
gi652424254	1.75	2	1	1142	122.6	5.11	6.11	pyruvate carboxylase [Arthrobacter castelli]
gi162954137	6.13	1	1	326	35.4	7.33	6.11	GTP-binding protein [Renibacterium salmoninarum ATCC 33209]
gi648572640	19.23	1	1	78	9.0	4.88	6.11	hypothetical protein [Arthrobacter sp. 135MFCol5.1]
gi916782081	1.29	1	1	778	85.1	6.60	6.11	phosphoketolase [Arthrobacter sp. 35W]
gi651437717	2.55	2	1	432	47.0	4.97	6.11	hypothetical protein [Arthrobacter sp. H14]
gi910283708	2.99	1	1	368	40.5	7.61	6.10	hypothetical protein [Arthrobacter sp. A3]
gi654811276	12.40	1	1	129	13.8	5.86	6.10	MerR family transcriptional regulator [Arthrobacter sp. MA-N2]
gi910739111	6.20	1	1	258	27.3	5.74	6.10	hypothetical protein AHiyo4_19330 [Arthrobacter sp. Hiyo4]
gi359307188	5.63	1	1	284	30.8	7.64	6.10	putative oxidoreductase [Arthrobacter globiformis NBRC 12137]
gi759716461	8.49	2	2	259	26.9	9.52	6.10	hypothetical protein, partial [Arthrobacter sp. AK-YN10]
gi937262564	8.29	7	1	217	23.1	6.11	6.10	ABC transporter ATP-binding protein [Arthrobacter sp. Edens01]
gi759761578	5.60	2	1	464	50.6	8.98	6.10	MFS transporter permease [Arthrobacter sp. Rue61a]
gi930827109	5.80	1	1	224	24.5	5.14	6.09	transcriptional regulator [Arthrobacter arilaitensis]
gi517602165	1.69	1	1	1003	110.0	6.35	6.09	glutamine-synthetase [Arthrobacter sp. 131MFCol6.1]
gi443480174	3.75	1	1	293	31.6	6.42	6.09	glutamate--tRNA ligase [Arthrobacter nitrophenolicus]
gi759710723	1.98	1	1	1062	118.0	5.36	6.09	DEAD/DEAH box helicase [Arthrobacter sp. 135MFCol5.1]
gi742757727	4.89	1	1	225	23.8	5.53	6.08	histidine phosphatase [Arthrobacter phenanthrenivorans]
gi119950460	4.76	1	1	441	46.1	6.47	6.08	adenosylmethionine-8-amino-7-oxononanoate aminotransferase [Arthrobacter aurescens T(
gi723607569	1.31	1	1	839	84.8	5.72	6.08	hypothetical protein ART_1346 [Arthrobacter sp. PAMC25486]
gi937259419	3.08	2	1	292	30.9	4.53	6.08	hypothetical protein AO716_05280 [Arthrobacter sp. Edens01]
gi651444177	1.43	1	1	1116	120.1	5.50	6.08	DNA helicase UvrD [Arthrobacter nicotinovorans]
gi515767999	5.45	1	1	330	35.9	4.77	6.08	epimerase [Arthrobacter sp. M2012083]
gi930825243	13.51	3	1	148	16.5	6.15	6.08	thioesterase [Arthrobacter arilaitensis]
gi910748742	5.94	3	1	286	31.2	10.78	6.07	hypothetical protein AHiyo8_64440 [Arthrobacter sp. Hiyo8]
gi910251166	10.50	1	1	238	25.7	6.81	6.07	DNA-binding response regulator [Arthrobacter siccitolerans]
gi823665799	6.17	1	1	243	27.2	7.36	6.07	transcriptional regulator [Arthrobacter sp. YC-RL1]
gi323470805	1.25	2	1	721	77.8	7.46	6.07	membrane carboxypeptidase (penicillin-binding protein) [Arthrobacter phenanthrenivorans
gi654828527	4.82	1	1	228	24.3	6.40	6.07	SDR family oxidoreductase [Arthrobacter sp. H5]
gi910696780	5.73	1	1	314	34.1	6.13	6.07	protein involved in mismatch repair, partial [Arthrobacter sp. Hiyo6]
gi476401571	3.81	1	1	394	44.0	7.03	6.06	hypothetical protein D477_008233 [Arthrobacter crystallopoietes BAB-32]
gi162955275	3.76	1	1	346	36.4	7.01	6.06	transcriptional repressor [Renibacterium salmoninarum ATCC 33209]
gi723607702	1.17	1	1	1111	120.2	5.31	6.06	hypothetical protein ART_1479 [Arthrobacter sp. PAMC25486]
gi759767544	2.55	1	1	666	74.3	6.42	6.06	cellulose synthase [Arthrobacter sp. SPG23]
gi742852660	2.05	1	1	537	56.2	10.26	6.06	hypothetical protein [Arthrobacter sp. W1]
gi917746221	3.34	1	1	869	91.8	9.57	6.05	transglutaminase [Arthrobacter phenanthrenivorans]
gi910748664	6.57	1	1	198	20.5	10.10	6.05	3-ketoacyl-CoA thiolase [Arthrobacter sp. Hiyo8]
gi652423094	3.99	1	1	376	40.4	4.82	6.05	transaldolase [Arthrobacter castelli]
gi551255566	9.68	1	1	186	20.8	6.30	6.05	MarR family transcriptional regulator [Arthrobacter sp. PAO19]
gi910741003	8.88	1	1	169	18.0	11.55	6.05	hypothetical protein AHiyo4_38250 [Arthrobacter sp. Hiyo4]
gi930828277	2.49	1	1	482	50.4	4.92	6.05	hypothetical protein AOZ07_16390 [Arthrobacter arilaitensis]
gi489902862	2.10	1	1	667	68.6	7.30	6.05	peptide ABC transporter ATP-binding protein [Arthrobacter globiformis]
gi651456105	2.89	3	1	588	64.5	5.48	6.05	ATPase AAA [Arthrobacter sp. 35/47]
gi674644458	6.00	1	1	400	41.6	5.34	6.05	N-acetylglucosamine repressor [Arthrobacter sp. 11W110_air]
gi189045102	3.49	1	1	573	60.0	5.35	6.05	RecName: Full=Dihydroxy-acid dehydratase; Short=DAD
gi307745493	3.94	1	1	406	44.8	5.81	6.04	probable alanine transaminase [Arthrobacter arilaitensis Re117]
gi443482665	4.12	1	1	364	38.6	5.35	6.04	aminotransferase [Arthrobacter nitrophenolicus]
gi742070797	2.10	1	1	572	61.0	5.05	6.04	redox sensor histidine kinase response regulator DevS [Arthrobacter sp. MWB30]
gi470217837	2.53	1	1	474	51.8	4.92	6.04	amine oxidase [Arthrobacter gangotriensis Lz1y]
gi476400085	5.66	1	1	318	34.4	9.23	6.04	transcriptional regulator, AraC family protein [Arthrobacter crystallopoietes BAB-32]
gi786032727	2.20	1	1	728	75.8	8.97	6.04	MFS transporter [Arthrobacter chlorophenolicus]
gi786030005	11.40	8	1	114	12.6	6.40	6.04	hypothetical protein [Arthrobacter chlorophenolicus]
gi443479561	7.14	1	1	210	23.1	8.21	6.04	glycine dehydrogenase, partial [Arthrobacter nitrophenolicus]
gi470220991	7.63	1	1	249	26.8	5.74	6.03	DeoR family transcriptional regulator [Arthrobacter gangotriensis Lz1y]
gi636844739	1.77	1	1	789	85.8	4.84	6.02	phosphoenolpyruvate synthase, partial [Arthrobacter sp. TB 26]
gi914715286	5.39	1	1	371	39.4	5.21	6.02	homoserine acetyltransferase [Arthrobacter sp. ZBG10]
gi139167	15.12	1	1	86	9.5	4.59	6.02	RecName: Full=Protein P17

gi910252452	2.58	1	1	621	65.1	5.21	6.02	dihydroxy-acid dehydratase [Arthrobacter siccitolerans]
gi908690231	5.00	1	1	360	39.3	5.08	6.02	phenylalanine--tRNA ligase subunit alpha [Arthrobacter sp. H41]
gi939037255	5.35	1	1	187	21.8	6.54	6.02	hypothetical protein [Arthrobacter nitroguajacolicus]
gi927296481	7.34	2	1	327	33.4	4.88	6.02	porphobilinogen deaminase [Arthrobacter sp. ERGS1:01]
gi737796581	0.87	1	1	1151	123.5	5.95	6.02	1-pyrroline-5-carboxylate dehydrogenase [Arthrobacter sp. H20]
gi636845504	3.63	4	1	496	53.0	5.35	6.02	chromosome partitioning protein ParA [Arthrobacter sp. TB 26]
gi737789714	2.85	1	1	701	75.4	5.19	6.01	phosphate acetyltransferase [Arthrobacter albus]
gi517609166	4.74	1	1	549	58.6	5.47	6.01	N-acyl-D-amino acid deacylase [Arthrobacter sp. 161MFSha2.1]
gi916692408	8.89	2	1	135	15.1	11.60	6.01	hypothetical protein [Arthrobacter castelli]
gi928485836	2.59	2	1	617	67.5	6.55	6.01	hypothetical protein AOC05_00355 [Arthrobacter alpinus]
gi651442553	4.12	3	1	413	42.5	5.97	6.01	cysteine desulfurase [Arthrobacter sp. 9MFCol3.1]
gi917441797	1.50	1	1	600	65.7	6.40	6.00	hypothetical protein [Arthrobacter albus]
gi545111548	1.89	3	1	689	76.1	4.68	6.00	hypothetical protein [Arthrobacter sp. AK-YN10]
gi517600131	5.00	1	1	320	31.4	4.81	6.00	ribokinase [Arthrobacter sp. 162MFSha1.1]
gi470220667	1.57	1	1	510	55.9	5.76	6.00	deoxyguanosinetriphosphate triphosphohydrolase-like protein [Arthrobacter gangotriensis L
gi914713669	2.78	1	1	431	46.5	5.12	5.99	sugar ABC transporter substrate-binding protein [Arthrobacter sp. ZBG10]
gi908690477	1.51	1	1	1261	136.7	5.87	5.99	AAA family ATPase [Arthrobacter sp. H41]
gi928487427	4.94	1	1	405	43.8	8.38	5.99	transposase [Arthrobacter alpinus]
gi823668203	6.23	3	2	401	42.0	5.45	5.99	beta-ketoadipyl CoA thiolase [Arthrobacter sp. YC-RL1]
gi760124848	3.29	1	1	365	38.8	5.07	5.99	nitrilotriacetate monooxygenase [Arthrobacter aurescens]
gi767258478	6.76	4	1	296	30.2	5.30	5.99	NADH-ubiquinone oxidoreductase [Arthrobacter sp. IHBB 11108]
gi914715222	1.45	2	1	692	77.7	6.38	5.99	hypothetical protein [Arthrobacter sp. ZBG10]
gi786027924	1.99	2	1	452	49.2	5.47	5.99	peptidase M1 [Arthrobacter chlorophenolicus]
gi916813788	2.36	1	1	890	94.3	7.09	5.98	FAD-dependent oxidoreductase [Arthrobacter nicotinovorans]
gi910748568	5.26	2	1	228	25.3	6.55	5.98	uvrABC system protein C [Arthrobacter sp. Hiyo8]
gi917013199	1.51	3	1	727	78.7	9.36	5.98	hypothetical protein [Arthrobacter sanguinis]
gi476401397	2.30	1	1	740	81.5	4.87	5.98	protease 2 [Arthrobacter crystallopoietes BAB-32]
gi654823577	2.59	2	1	502	54.1	7.43	5.98	hypothetical protein [Arthrobacter sp. I3]
gi916782376	2.21	2	1	498	48.8	5.44	5.97	hypothetical protein [Arthrobacter sp. 35W]
gi908699402	5.88	1	1	289	29.8	4.41	5.96	flagellar biosynthesis protein flip [Arthrobacter sp. RIT-PI-e]
gi119950765	6.53	1	1	199	22.0	5.63	5.96	putative DNA-3-methyladenine glycosylase I [Arthrobacter aurescens TC1]
gi219860436	7.23	1	1	166	18.5	8.98	5.96	conserved hypothetical protein [Arthrobacter chlorophenolicus A6]
gi910742951	5.30	1	1	415	44.9	5.06	5.96	carbon monoxide dehydrogenase large chain [Arthrobacter sp. Hiyo8]
gi742069381	4.06	1	1	320	34.7	5.21	5.96	hypothetical protein ANMWB30_36590 [Arthrobacter sp. MWB30]
gi674645913	5.73	1	1	192	20.4	6.93	5.96	DNA-3-methyladenine glycosylase 1 [Arthrobacter sp. 11W110_air]
gi723608235	2.20	2	1	364	40.5	6.18	5.95	hypothetical protein ART_2012 [Arthrobacter sp. PAMC25486]
gi307743639	2.94	1	1	578	62.4	6.39	5.95	putative drug resistance ATP-binding protein [Arthrobacter arilaitensis Re117]
gi443480663	24.32	1	1	37	4.4	10.61	5.93	50S ribosomal protein L36 [Arthrobacter nitrophenolicus]
gi651441396	3.96	1	1	303	30.7	5.59	5.93	shikimate dehydrogenase [Arthrobacter sp. 9MFCol3.1]
gi674645789	10.17	1	1	177	18.1	9.51	5.92	hypothetical protein BN1051_01987 [Arthrobacter sp. 11W110_air]
gi652423843	5.23	1	1	344	36.3	5.21	5.92	fructose 1,6-bisphosphatase [Arthrobacter castelli]
gi359307792	2.21	1	1	678	71.4	5.49	5.92	putative oxidoreductase [Arthrobacter globiformis NBRC 12137]
gi517600335	3.74	2	1	428	45.0	5.63	5.92	hypothetical protein [Arthrobacter sp. 162MFSha1.1]
gi640197663	3.97	2	1	453	48.7	6.24	5.92	hypothetical protein [Arthrobacter sp. 31Y]
gi359305622	3.80	1	1	553	58.0	5.14	5.92	hypothetical protein ARGLB_058_00300 [Arthrobacter globiformis NBRC 12137]
gi918469404	2.19	2	1	548	57.0	9.91	5.92	hypothetical protein [Arthrobacter crystallopoietes]
gi654812052	2.20	1	1	908	99.2	6.27	5.91	DNA topoisomerase I [Arthrobacter sp. MA-N2]
gi323471565	7.63	2	1	262	29.0	4.92	5.91	hypothetical protein Asphe3_41840 (plasmid) [Arthrobacter phenanthrenivorans Sphe3]
gi823667533	2.79	3	1	358	39.8	5.20	5.91	magnesium transporter [Arthrobacter sp. YC-RL1]
gi517598084	3.80	2	1	527	56.8	9.50	5.91	signal recognition particle protein [Arthrobacter sp. 162MFSha1.1]
gi916863668	2.43	1	1	575	60.3	5.58	5.90	oxidoreductase, partial [Arthrobacter sp. 35/47]
gi723609944	3.31	4	1	363	36.4	5.19	5.90	hypothetical protein ART_3721 [Arthrobacter sp. PAMC25486]
gi927295811	1.82	4	1	658	71.6	5.29	5.89	acetyl-CoA synthetase [Arthrobacter sp. ERGS1:01]
gi939037203	4.88	1	1	389	40.4	9.07	5.89	sugar ABC transporter [Arthrobacter nitroguajacolicus]
gi930827289	4.18	2	1	263	28.5	9.35	5.89	IclR family transcriptional regulator [Arthrobacter arilaitensis]
gi753939617	3.79	1	1	369	40.0	5.44	5.89	threonine aldolase [Arthrobacter phenanthrenivorans]
gi742757779	5.86	1	1	273	29.2	9.70	5.89	alpha/beta hydrolase [Arthrobacter phenanthrenivorans]
gi914715046	1.47	2	1	882	90.9	6.87	5.88	hydrolase [Arthrobacter sp. ZBG10]

gi517605239	1.76	2	1	910	96.4	7.34	5.88	magnesium-transporting ATPase [Arthrobacter sp. 131MFCol6.1]
gi542108488	5.28	1	1	284	31.4	5.10	5.88	UDP-glucose 4-epimerase [Arthrobacter sp. AK-YN10]
gi476402224	0.62	2	1	1131	126.5	6.64	5.88	hypothetical protein D477_004676 [Arthrobacter crystallopoietes BAB-32]
gi651503237	1.19	1	1	924	102.0	5.16	5.88	preprotein translocase subunit SecA [Arthrobacter sp. 35W]
gi918469367	7.01	1	1	157	17.2	8.98	5.87	ribonucleoside-diphosphate reductase [Arthrobacter crystallopoietes]
gi654827028	5.73	1	1	192	20.9	5.60	5.87	XRE family transcriptional regulator [Arthrobacter sp. H5]
gi476403108	5.36	4	1	261	28.5	5.76	5.87	IclR family transcripitonal regulator [Arthrobacter crystallopoietes BAB-32]
gi927032104	2.62	2	1	420	46.1	7.61	5.87	hypothetical protein AFL94_07015 [Arthrobacter sp. LS16]
gi910746423	4.33	1	1	277	28.2	11.66	5.86	DEAD-box ATP-dependent RNA helicase 7 [Arthrobacter sp. Hiyo8]
gi910740820	6.30	1	1	127	13.7	11.40	5.85	50S ribosomal protein L18 [Arthrobacter sp. Hiyo4]
gi116612511	2.74	1	1	402	42.9	5.14	5.85	O-succinylhomoserine sulfhydrylase [Arthrobacter sp. FB24]
gi470220772	4.38	2	1	434	48.2	5.21	5.85	glycine cleavage system T protein [Arthrobacter gangotriensis Lz1y]
gi723608038	7.12	6	1	267	28.4	4.72	5.85	ABC-type metal ion transport system, periplasmic component/surface antigen [Arthrobacter
gi651429854	1.92	1	1	312	31.9	4.59	5.85	zinc-binding dehydrogenase [Arthrobacter sanguinis]
gi359304092	5.30	1	1	264	27.8	5.25	5.85	putative phosphopantetheinyl transferase [Arthrobacter globiformis NBRC 12137]
gi918267149	3.10	1	1	581	59.9	6.49	5.85	probable copper-exporting P-type ATPase V [Arthrobacter sp. Hiyo1]
gi908697381	2.60	1	1	538	56.9	5.30	5.84	hypothetical protein [Arthrobacter sp. RIT-PI-e]
gi910695867	4.65	1	1	258	27.7	11.08	5.84	uncharacterized membrane protein slr0232 [Arthrobacter sp. Hiyo6]
gi927296406	3.34	1	1	419	42.5	10.42	5.84	arabinose ABC transporter permease [Arthrobacter sp. ERGS1:01]
gi640203129	3.24	1	1	586	62.9	5.34	5.83	hydantoinase [Arthrobacter sp. 31Y]
gi674645557	1.64	2	1	912	101.6	5.44	5.83	Pyruvate dehydrogenase E1 component [Arthrobacter sp. 11W110_air]
gi765004150	10.00	1	1	140	15.4	11.60	5.83	peptide chain release factor 1 [Arthrobacter sp. A3]
gi742757217	3.49	1	1	487	51.8	5.43	5.83	mannitol dehydrogenase [Arthrobacter phenanthrenivorans]
gi651457044	23.75	1	1	80	8.9	7.44	5.82	KH domain-containing protein [Arthrobacter sp. 35/47]
gi723607757	2.65	1	1	339	35.8	8.56	5.82	putative transcriptional regulator, LacI family [Arthrobacter sp. PAMC25486]
gi767259331	3.30	3	1	455	49.6	5.14	5.82	beta-glucosidase [Arthrobacter sp. IHBB 11108]
gi162953027	4.02	1	1	423	45.5	10.07	5.81	DNA methylation and regulatory protein ADA [Renibacterium salmoninarum ATCC 33209]
gi654817170	7.82	3	1	243	25.2	9.73	5.81	septation ring formation regulator EzrA [Arthrobacter sp. UNC362MFTsu5.1]
gi542106680	1.46	1	1	1161	129.5	5.30	5.81	ATP-binding protein [Arthrobacter sp. AK-YN10]
gi910741186	5.95	2	1	437	46.5	9.44	5.81	hypothetical protein AHiyo4_40080 [Arthrobacter sp. Hiyo4]
gi651434916	4.95	1	1	404	43.5	9.47	5.81	pyridine nucleotide-disulfide oxidoreductase [Arthrobacter sp. H41]
gi648572686	3.96	1	1	404	42.7	6.57	5.80	dephospho-CoA kinase [Arthrobacter sp. 135MFCol5.1]
gi517604666	3.55	1	1	451	48.5	5.33	5.80	acetyl-CoA carboxylase biotin carboxylase subunit [Arthrobacter sp. 131MFCol6.1]
gi910248652	2.60	2	1	384	42.0	5.43	5.80	alanine dehydrogenase [Arthrobacter siccitolerans]
gi551255787	2.28	1	1	395	44.5	7.11	5.80	glycosyl transferase family 1 [Arthrobacter sp. PAO19]
gi760125766	2.85	1	1	492	53.0	9.99	5.80	hypothetical protein [Arthrobacter aurescens]
gi918221963	3.39	1	1	383	39.6	7.09	5.80	acetylornithine deacetylase, partial [Arthrobacter sp. I3]
gi916835012	2.32	1	1	691	77.7	5.06	5.80	threonine--tRNA ligase [Arthrobacter sp. H14]
gi651500725	0.92	1	1	1088	117.3	5.77	5.80	hypothetical protein [Arthrobacter sp. 35W]
gi674645676	2.21	1	1	542	54.2	6.73	5.80	L-aspartate oxidase [Arthrobacter sp. 11W110_air]
gi927296248	3.62	1	1	470	50.0	5.14	5.79	aldehyde dehydrogenase [Arthrobacter sp. ERGS1:01]
gi651484712	5.62	4	1	249	27.9	5.01	5.79	phosphoglycerate mutase [Arthrobacter sp. Br18]
gi636847109	2.37	1	1	465	51.6	7.43	5.79	hypothetical protein [Arthrobacter sp. TB 26]
gi908740287	4.02	5	1	323	35.2	5.63	5.79	hypothetical protein [Arthrobacter arilaitensis]
gi927296047	2.27	2	1	705	75.2	6.30	5.79	peptide ABC transporter ATP-binding protein [Arthrobacter sp. ERGS1:01]
gi916691212	1.98	1	1	606	63.9	5.26	5.78	ABC transporter [Arthrobacter castelli]
gi518312359	3.30	1	1	424	45.6	5.41	5.78	hypothetical protein [Arthrobacter sp. TB 23]
gi551255966	0.82	1	1	858	93.8	6.18	5.78	hypothetical protein [Arthrobacter sp. PAO19]
gi654812750	5.04	1	1	397	43.6	5.30	5.76	acyl-CoA dehydrogenase [Arthrobacter sp. MA-N2]
gi119948710	2.76	3	1	507	55.0	5.44	5.76	aldehyde dehydrogenase (NAD) family protein [Arthrobacter aurescens TC1]
gi914715294	4.04	1	1	272	29.5	10.68	5.75	glycerol acyltransferase [Arthrobacter sp. ZBG10]
gi640196032	6.21	1	1	338	36.0	4.93	5.75	O-succinylbenzoate synthase [Arthrobacter sp. 31Y]
gi656049967	3.02	1	1	331	34.7	5.06	5.75	prephenate dehydratase [Brevibacterium album]
gi910747464	7.66	1	1	248	27.4	9.50	5.75	uncharacterized protein Rv2248/MT2308 [Arthrobacter sp. Hiyo8]
gi476402751	3.55	5	1	338	37.3	5.26	5.75	hypothetical protein D477_001684 [Arthrobacter crystallopoietes BAB-32]
gi767258934	1.33	1	1	602	65.0	4.81	5.75	beta-glucosidase [Arthrobacter sp. IHBB 11108]
gi323468295	0.80	3	1	1244	135.4	5.39	5.74	alpha-1,4-glucan:alpha-1,4-glucan 6-glycosyltransferase [Arthrobacter phenanthrenivorans
gi928488438	1.91	2	1	681	77.2	9.60	5.74	hypothetical protein AOC05_17975 [Arthrobacter alpinus]

gi219860471	2.48	2	1	404	44.1	10.35	5.74	monooxygenase FAD-binding [Arthrobacter chlorophenolicus A6]
gi359307509	6.15	1	1	325	35.4	5.87	5.74	transcription elongation protein NusA [Arthrobacter globiformis NBRC 12137]
gi910738120	12.41	2	1	145	15.0	6.93	5.74	uncharacterized oxidoreductase YisS [Arthrobacter sp. Hiyo4]
gi786030935	1.41	1	1	710	72.6	11.81	5.74	competence protein ComEC [Arthrobacter chlorophenolicus]
gi916834541	8.73	4	1	126	13.9	4.63	5.73	hypothetical protein [Arthrobacter sp. H14]
gi939050189	4.49	3	1	401	43.1	6.19	5.73	aminotransferase [Arthrobacter sp. JCM 19049]
gi916871994	1.83	1	1	546	59.3	6.02	5.73	hypothetical protein [Arthrobacter sp. H5]
gi640196311	9.60	1	1	198	21.8	7.40	5.73	protein-tyrosine phosphatase [Arthrobacter sp. 31Y]
gi651499552	1.49	2	1	1338	148.5	5.48	5.73	type II restriction enzyme, methylase subunits [Arthrobacter sp. 35W]
gi654818129	4.89	1	1	307	32.7	4.97	5.73	diacylglycerol kinase [Arthrobacter sp. UNC362MFTsu5.1]
gi930825931	7.50	3	1	280	30.3	4.97	5.72	hypothetical protein AOZ07_06255 [Arthrobacter arilaitensis]
gi910249548	2.05	1	1	877	94.8	6.65	5.72	AMP-dependent synthetase [Arthrobacter siccitolerans]
gi116611372	2.91	1	1	412	45.2	10.74	5.71	protein of unknown function DUF58 [Arthrobacter sp. FB24]
gi119951606	5.05	1	1	317	34.0	10.26	5.71	putative Membrane protein (plasmid) [Arthrobacter aurescens TC1]
gi786027565	4.58	2	1	240	25.8	8.87	5.71	hypothetical protein [Arthrobacter chlorophenolicus]
gi654817001	1.78	1	1	337	36.5	5.14	5.71	2-hydroxyacid dehydrogenase [Arthrobacter sp. UNC362MFTsu5.1]
gi916834609	2.31	1	1	562	58.8	5.00	5.71	acetolactate synthase [Arthrobacter sp. H14]
gi742072610	3.09	2	1	421	43.1	6.40	5.71	molybdopterin biosynthesis protein MoeA [Arthrobacter sp. MWB30]
gi551256406	2.31	1	1	520	55.5	9.07	5.70	signal recognition particle protein [Arthrobacter sp. PAO19]
gi917021956	1.80	1	1	388	41.0	5.21	5.70	hypothetical protein [Arthrobacter sp. UNC362MFTsu5.1]
gi476399074	6.61	4	1	242	26.0	11.12	5.70	hypothetical protein D477_020318, partial [Arthrobacter crystallopoietes BAB-32]
gi119951660	2.90	1	1	207	22.8	8.41	5.69	hypothetical protein AAur_pTC10007 (plasmid) [Arthrobacter aurescens TC1]
gi927295947	0.75	1	1	1335	133.2	5.45	5.69	hypothetical protein AL755_21095 [Arthrobacter sp. ERGS1:01]
gi640195932	5.04	3	1	476	50.7	5.34	5.69	phenylacetaldehyde dehydrogenase [Arthrobacter sp. 31Y]
gi162954497	1.86	1	1	646	70.4	6.49	5.69	ABC transporter, transmembrane region [Renibacterium salmoninarum ATCC 33209]
gi910697105	5.62	1	1	249	26.9	5.76	5.69	putative protein methyltransferase MJ0928, partial [Arthrobacter sp. Hiyo6]
gi908698630	12.26	1	1	155	17.1	5.44	5.69	hypothetical protein [Arthrobacter sp. RIT-PI-e]
gi219860414	5.42	1	1	332	37.0	10.43	5.69	conserved hypothetical protein [Arthrobacter chlorophenolicus A6]
gi517604588	3.82	1	1	262	26.7	6.80	5.68	3-oxoacyl-ACP reductase [Arthrobacter sp. 131MFCol6.1]
gi908699167	1.72	1	1	582	63.7	5.36	5.67	FAD-dependent oxidoreductase [Arthrobacter sp. RIT-PI-e]
gi908697387	48.65	2	1	37	4.3	10.43	5.67	50S ribosomal protein L36 [Arthrobacter sp. RIT-PI-e]
gi654828166	2.18	1	1	689	73.0	6.51	5.67	hypothetical protein [Arthrobacter sp. H5]
gi674643955	1.64	1	1	489	50.6	5.14	5.66	Dihydrolipoyl dehydrogenase [Arthrobacter sp. 11W110_air]
gi517605371	7.61	1	1	197	21.3	5.57	5.66	hypothetical protein [Arthrobacter sp. 131MFCol6.1]
gi910739166	10.00	2	1	70	7.2	5.26	5.66	hypothetical protein AHiyo4_19880 [Arthrobacter sp. Hiyo4]
gi651506641	3.40	1	1	235	25.6	6.20	5.66	GntR family transcriptional regulator [Arthrobacter sp. 35W]
gi651484318	4.88	1	1	389	39.7	4.96	5.65	N-acetylglutamate synthase [Arthrobacter sp. Br18]
gi651460772	2.04	1	1	637	69.3	5.47	5.65	hypothetical protein [Arthrobacter sp. 35/47]
gi517592442	2.31	1	1	519	56.0	10.32	5.64	membrane protein [Arthrobacter sp. 135MFCol5.1]
gi654817601	5.60	1	1	250	26.4	6.96	5.64	TetR family transcriptional regulator [Arthrobacter sp. UNC362MFTsu5.1]
gi917739667	4.73	1	1	275	29.8	5.55	5.63	spermidine synthase [Arthrobacter sp. W1]
gi910745659	3.89	1	1	334	36.0	8.34	5.63	conserved hypothetical protein [Arthrobacter sp. Hiyo8]
gi769943077	2.18	1	1	597	64.3	4.86	5.62	hypothetical protein [Arthrobacter sp. IHBB 11108]
gi917013004	1.98	2	1	961	102.9	5.22	5.62	glycine dehydrogenase [Arthrobacter sanguinis]
gi116609349	2.27	1	1	529	55.6	5.54	5.62	phytoene dehydrogenase [Arthrobacter sp. FB24]
gi918269017	20.31	13	1	64	7.0	5.02	5.62	dipeptide transport system permease protein DppB [Arthrobacter sp. Hiyo1]
gi119947513	4.74	1	1	211	23.3	10.89	5.62	putative S4 domain protein [Arthrobacter aurescens TC1]
gi119949986	4.42	5	1	362	38.7	4.67	5.61	conserved hypothetical protein [Arthrobacter aurescens TC1]
gi917760043	11.00	1	1	200	20.4	5.63	5.61	hypothetical protein [Arthrobacter sp. L77]
gi443481304	7.92	2	1	101	11.7	5.73	5.61	hypothetical protein G205_13072 [Arthrobacter nitrophenolicus]
gi307744254	2.18	1	1	596	66.0	5.74	5.59	putative FAD-dependent pyridine nucleotide-disulphide oxidoreductase [Arthrobacter arilait
gi910744526	14.29	1	1	70	7.5	9.77	5.59	ATP-dependent DNA helicase PcrA [Arthrobacter sp. Hiyo8]
gi542106916	1.34	1	1	447	49.6	6.83	5.59	F420-dependent methylene-tetrahydromethanopterin reductase [Arthrobacter sp. AK-YN10
gi910250456	9.33	2	1	150	15.8	7.30	5.58	hypothetical protein [Arthrobacter siccitolerans]
gi219862011	1.56	1	1	833	90.2	6.34	5.58	hypothetical protein Achl_4401 (plasmid) [Arthrobacter chlorophenolicus A6]
gi551253804	6.27	1	1	287	30.6	10.11	5.58	hypothetical protein [Arthrobacter sp. PAO19]
gi307744843	2.23	1	1	582	62.7	5.62	5.58	VanW-like protein [Arthrobacter arilaitensis Re117]
gi916869682	10.62	1	1	226	23.4	4.56	5.58	hypothetical protein [Arthrobacter sp. Br18]

gi651431350	1.27	1	1	869	91.7	6.46	5.58	hypothetical protein [Arthrobacter sanguinis]
gi443481971	2.69	1	1	372	38.8	5.55	5.57	bifunctional phosphopantothenoylcysteine decarboxylase/phosphopantothenate synthase [A
gi674645240	4.85	1	1	206	22.9	5.64	5.57	Threonylcarbamoyl-AMP synthase [Arthrobacter sp. 11W110_air]
gi323469116	7.79	1	1	244	25.0	6.55	5.57	short-chain alcohol dehydrogenase [Arthrobacter phenanthrenivorans Sphe3]
gi219860995	8.56	2	1	222	23.1	6.35	5.57	two component transcriptional regulator, LuxR family [Arthrobacter chlorophenolicus A6]
gi742859171	9.09	15	1	209	23.4	9.42	5.56	transcriptional regulator [Arthrobacter sp. W1]
gi651429178	5.34	1	1	393	42.2	8.70	5.56	hypothetical protein [Arthrobacter sanguinis]
gi651492912	1.14	1	1	615	69.3	6.01	5.56	hypothetical protein [Arthrobacter sp. H20]
gi654817904	2.03	1	1	887	93.6	7.17	5.56	magnesium-translocating P-type ATPase [Arthrobacter sp. UNC362MFTsu5.1]
gi219859110	8.75	8	1	160	18.0	5.86	5.55	regulatory protein RecX [Arthrobacter chlorophenolicus A6]
gi908698025	4.02	1	1	572	61.9	9.25	5.55	hypothetical protein [Arthrobacter sp. RIT-PI-e]
gi654813178	2.66	8	1	451	49.3	6.28	5.54	monooxygenase [Arthrobacter sp. MA-N2]
gi517609022	6.43	6	1	389	44.8	6.23	5.53	hypothetical protein [Arthrobacter sp. 161MFSha2.1]
gi767258788	2.33	1	1	687	75.2	4.86	5.53	multidrug ABC transporter ATP-binding protein [Arthrobacter sp. IHBB 11108]
gi651503294	2.21	1	1	407	41.9	6.20	5.53	cysteine desulfurase [Arthrobacter sp. 35W]
gi757624761	23.46	1	1	81	9.0	9.79	5.53	hypothetical protein TV39_08735 [Arthrobacter sp. SPG23]
gi918267429	3.33	1	1	511	54.3	7.50	5.53	hypothetical protein AHiyo1_20830 [Arthrobacter sp. Hiyo1]
gi476399179	4.56	2	1	329	35.7	5.12	5.52	hypothetical protein D477_019793 [Arthrobacter crystallopoietes BAB-32]
gi651430481	2.19	1	1	456	49.7	10.18	5.52	hypothetical protein [Arthrobacter sanguinis]
gi723606498	3.33	1	1	360	39.1	6.67	5.51	ATPase, AFG1 family protein [Arthrobacter sp. PAMC25486]
gi651440128	2.17	1	1	877	95.4	5.97	5.51	hypothetical protein [Arthrobacter sp. H14]
gi517592051	4.12	2	1	413	42.4	10.43	5.50	MFS transporter [Arthrobacter sp. 135MFCol5.1]
gi914716977	3.14	1	1	287	31.5	9.11	5.50	alpha/beta hydrolase [Arthrobacter sp. ZBG10]
gi914717757	1.89	1	1	1112	114.6	9.83	5.50	glycosyl transferase [Arthrobacter sp. ZBG10]
gi170783480	4.28	1	1	397	43.1	5.77	5.49	putative oxidoreductase domain protein (plasmid) [Arthrobacter sp. AK-1]
gi916834745	1.17	1	1	1027	109.2	6.01	5.49	FAD-linked oxidase [Arthrobacter sp. H14]
gi916782148	3.83	4	1	549	58.4	9.39	5.48	hypothetical protein [Arthrobacter sp. 35W]
gi651459304	11.18	2	1	152	16.4	6.55	5.47	hypothetical protein [Arthrobacter sp. 35/47]
gi359305903	1.19	1	1	505	51.9	6.14	5.47	putative aminopeptidase [Arthrobacter globiformis NBRC 12137]
gi651433030	3.76	1	1	399	43.2	9.70	5.47	transposase [Arthrobacter sp. H41]
gi916820204	3.75	3	1	373	40.5	9.33	5.47	hypothetical protein [Arthrobacter sp. H20]
gi517601220	4.21	2	1	309	34.5	5.39	5.46	hypothetical protein [Arthrobacter sp. 162MFSha1.1]
gi674645956	4.49	2	1	245	25.4	9.95	5.45	Glycine betaine/carnitine/choline transport system permease protein OpuCB [Arthrobacter s
gi927293773	4.09	2	1	513	53.3	6.46	5.45	carboxylesterase [Arthrobacter sp. ERGS1:01]
gi910738178	2.10	2	1	668	71.4	7.80	5.44	uncharacterized protein HI_0568 [Arthrobacter sp. Hiyo4]
gi470215927	4.95	2	1	202	21.9	5.17	5.44	hypothetical protein ADIAG_03888 [Arthrobacter gangotriensis Lz1y]
gi517590501	0.94	1	1	1172	124.6	6.27	5.43	1-pyrroline-5-carboxylate dehydrogenase [Arthrobacter sp. 135MFCol5.1]
gi470217147	6.99	4	1	329	35.0	5.24	5.43	luciferase-like monooxygenase [Arthrobacter gangotriensis Lz1y]
gi651508041	3.29	1	1	395	44.5	8.00	5.42	hypothetical protein [Arthrobacter sp. 35W]
gi674645239	2.44	1	1	532	57.5	5.68	5.41	putative ABC transporter ATP-binding protein YheS [Arthrobacter sp. 11W110_air]
gi742853756	2.66	1	1	413	44.6	6.21	5.40	SAM-dependent methyltransferase [Arthrobacter sp. W1]
gi910747370	15.87	2	1	63	7.4	8.19	5.40	30S ribosomal protein S10 [Arthrobacter sp. Hiyo8]
gi674646899	3.30	4	1	575	61.4	5.85	5.40	Urocanate hydratase [Arthrobacter sp. 11W110_air]
gi910741299	18.18	1	1	110	11.7	5.45	5.40	dienelactone hydrolase [Arthrobacter sp. Hiyo4]
gi651441879	15.71	3	1	140	15.4	11.44	5.39	hypothetical protein [Arthrobacter sp. 9MFCol3.1]
gi517592505	5.90	1	1	271	30.1	9.38	5.38	hypothetical protein [Arthrobacter sp. 135MFCol5.1]
gi651463296	2.52	1	1	317	34.4	6.74	5.38	sugar ABC transporter ATP-binding protein [Arthrobacter sp. 35/47]
gi640193416	0.91	1	1	990	109.8	6.90	5.38	RNA helicase [Arthrobacter sp. 31Y]
gi742071249	8.73	4	1	252	26.7	4.69	5.38	hypothetical protein ANMWB30_17750 [Arthrobacter sp. MWB30]
gi674646966	4.40	1	1	386	40.8	11.22	5.38	Pentachlorophenol 4-monooxygenase [Arthrobacter sp. 11W110_air]
gi769943287	3.36	3	1	506	53.5	5.03	5.37	hypothetical protein [Arthrobacter sp. IHBB 11108]
gi742858932	2.68	1	1	298	32.2	6.46	5.37	hypothetical protein [Arthrobacter sp. W1]
gi651434286	4.96	1	1	282	30.0	5.11	5.37	pyridoxal biosynthesis lyase PdxS [Arthrobacter sp. H41]
gi910249149	13.83	4	1	94	9.2	9.98	5.37	hypothetical protein [Arthrobacter siccitolerans]
gi517598979	2.89	3	1	346	38.8	5.24	5.36	glyceraldehyde 3-phosphate reductase [Arthrobacter sp. 162MFSha1.1]
gi518312377	3.82	1	1	498	53.8	5.19	5.35	hypothetical protein [Arthrobacter sp. TB 23]
gi916691436	2.76	1	1	399	41.9	9.39	5.34	hypothetical protein [Arthrobacter castelli]
gi757622369	4.66	3	1	343	36.4	9.96	5.34	FAD-binding monooxygenase [Arthrobacter sp. SPG23]

gi917529696	11.54	3	1	104	10.9	9.98	5.34	hypothetical protein [Arthrobacter sp. PAMC25486]
gi443482553	4.97	9	1	382	39.8	6.13	5.34	ROK family transcriptional regulator [Arthrobacter nitrophenolicus]
gi359305419	4.86	2	1	288	31.0	9.86	5.33	putative LysR family transcriptional regulator [Arthrobacter globiformis NBRC 12137]
gi359303403	1.43	3	1	700	72.9	5.90	5.33	putative oxidoreductase [Arthrobacter globiformis NBRC 12137]
gi916781622	2.81	3	1	462	47.3	5.25	5.32	hypothetical protein [Arthrobacter sp. 35W]
gi917759797	2.61	3	1	575	61.5	6.40	5.32	hypothetical protein [Arthrobacter sp. L77]
gi652424255	1.99	2	1	954	102.3	5.12	5.32	glycine dehydrogenase [Arthrobacter castelli]
gi908696786	7.19	1	1	292	32.9	9.80	5.32	transposase [Arthrobacter sp. RIT-PI-e]
gi674644704	2.80	4	1	607	63.8	5.29	5.32	Acetyl-/propionyl-coenzyme A carboxylase alpha chain [Arthrobacter sp. 11W110_air]
gi786027072	7.26	1	1	303	33.0	10.56	5.31	NAD-dependent epimerase [Arthrobacter chlorophenolicus]
gi928485928	4.11	3	1	389	41.3	5.59	5.31	XshC-Cox1 family protein [Arthrobacter alpinus]
gi518313275	4.52	1	1	199	20.9	7.36	5.31	hypothetical protein [Arthrobacter sp. TB 23]
gi674644087	6.21	6	1	306	33.1	4.22	5.30	hypothetical protein BN1051_00229 [Arthrobacter sp. 11W110_air]
gi757624278	1.15	1	1	872	95.1	4.97	5.30	aminopeptidase N [Arthrobacter sp. SPG23]
gi542106855	8.90	6	1	191	19.9	5.29	5.30	hypothetical protein M707_19805 [Arthrobacter sp. AK-YN10]
gi939050335	3.17	1	1	221	24.0	4.94	5.29	hypothetical protein [Arthrobacter sp. JCM 19049]
gi307744495	3.04	3	1	428	43.3	4.77	5.29	homoserine dehydrogenase [Arthrobacter arilaitensis Re117]
gi443479743	4.05	1	1	469	48.9	4.96	5.29	diaminopimelate decarboxylase [Arthrobacter nitrophenolicus]
gi517601118	4.51	4	1	377	40.1	5.31	5.28	oxidoreductase [Arthrobacter sp. 162MFSha1.1]
gi470217443	7.18	4	1	181	20.0	4.35	5.28	16S rRNA processing protein [Arthrobacter gangotriensis Lz1y]
gi489901731	6.25	3	2	432	45.4	9.47	5.28	MFS transporter [Arthrobacter globiformis]
gi651430563	2.93	2	1	307	32.4	4.89	5.27	pyridoxal biosynthesis lyase PdxS [Arthrobacter sanguinis]
gi910741474	10.17	4	1	177	19.3	5.36	5.27	uncharacterized protein C4H3.03c [Arthrobacter sp. Hiyo4]
gi162955075	5.75	1	1	348	36.4	5.31	5.26	zinc-dependent alcohol dehydrogenase [Renibacterium salmoninarum ATCC 33209]
gi919219009	3.12	2	1	353	40.3	8.53	5.26	MULTISPECIES: hypothetical protein [Arthrobacter]
gi917012965	6.14	2	1	228	24.5	6.00	5.25	potassium transporter [Arthrobacter sanguinis]
gi916782042	4.85	1	1	412	43.3	5.47	5.24	molybdopterin biosynthesis-like protein MoeZ [Arthrobacter sp. 35W]
gi515767642	4.97	6	1	322	35.3	8.51	5.24	NUDIX hydrolase [Arthrobacter sp. M2012083]
gi403228513	2.65	2	1	567	60.0	4.96	5.23	putative acetolactate synthase large subunit [Arthrobacter sp. Rue61a]
gi910739674	2.71	2	1	443	47.8	4.77	5.22	UPF0053 protein Mb2387c [Arthrobacter sp. Hiyo4]
gi651444846	2.67	2	1	450	47.0	6.16	5.22	acetyl-CoA acetyltransferase [Arthrobacter nicotinovorans]
gi742856249	5.95	2	1	185	20.1	5.27	5.22	hypothetical protein [Arthrobacter sp. W1]
gi737812459	3.25	8	1	400	43.1	5.54	5.21	dephospho-CoA kinase [Arthrobacter sp. H14]
gi927294446	3.98	2	1	352	36.0	6.33	5.21	hypothetical protein AL755_11175 [Arthrobacter sp. ERGS1:01]
gi916324716	4.20	1	1	333	35.1	4.92	5.20	thioredoxin reductase [Arthrobacter gangotriensis]
gi916813652	11.73	4	2	341	37.3	9.19	5.20	hypothetical protein [Arthrobacter nicotinovorans]
gi470217452	4.07	2	1	246	26.1	6.96	5.20	ribonuclease H [Arthrobacter gangotriensis Lz1y]
gi654811375	5.60	2	1	268	29.1	5.49	5.20	transglutaminase [Arthrobacter sp. MA-N2]
gi916813632	2.94	1	1	612	64.1	9.19	5.20	hypothetical protein [Arthrobacter nicotinovorans]
gi515766339	5.57	9	1	305	34.1	4.78	5.19	myo-inosose-2 dehydratase [Arthrobacter sp. M2012083]
gi823666748	4.26	1	1	470	49.3	4.87	5.19	aldehyde dehydrogenase [Arthrobacter sp. YC-RL1]
gi651463030	2.32	7	1	561	61.3	7.14	5.19	ATP-dependent DNA helicase PcrA [Arthrobacter sp. 35/47]
gi651441426	3.71	1	1	512	51.2	11.66	5.18	competence protein ComEC [Arthrobacter sp. 9MFCol3.1]
gi551256905	4.76	3	1	357	40.2	7.40	5.18	mannosyltransferase [Arthrobacter sp. PAO19]
gi917013430	4.56	2	1	373	40.0	5.31	5.17	glycine/betaine ABC transporter ATPase [Arthrobacter sanguinis]
gi786028680	2.97	1	1	572	59.9	5.60	5.17	dihydroxy-acid dehydratase [Arthrobacter chlorophenolicus]
gi651441390	6.07	3	1	214	22.6	9.95	5.17	hypothetical protein [Arthrobacter sp. 9MFCol3.1]
gi916692132	1.98	2	1	810	85.9	5.59	5.16	xanthine dehydrogenase [Arthrobacter castelli]
gi742069395	5.59	9	1	322	33.7	6.43	5.16	HTH-type transcriptional regulator [Arthrobacter sp. MWB30]
gi927033011	6.80	2	1	353	38.9	9.29	5.16	cytochrome C biogenesis protein [Arthrobacter sp. LS16]
gi917013200	5.96	1	1	285	31.4	9.48	5.16	hypothetical protein [Arthrobacter sanguinis]
gi652424911	4.15	1	1	313	33.3	9.66	5.15	type II secretion system protein F [Arthrobacter castelli]
gi162954278	3.42	1	1	380	39.3	7.87	5.15	putative Iron(III) dicitrate-binding protein [Renibacterium salmoninarum ATCC 33209]
gi939050770	3.55	2	1	620	67.7	6.43	5.15	recombinase RecQ, partial [Arthrobacter sp. JCM 19049]
gi651435082	2.16	3	1	694	72.9	4.94	5.14	protein kinase [Arthrobacter sp. H41]
gi403228653	2.05	2	1	537	57.9	5.68	5.14	putative regulatory protein [Arthrobacter sp. Rue61a]
gi757624374	4.19	4	1	334	36.3	5.26	5.14	flavodoxin [Arthrobacter sp. SPG23]
gi917012971	4.39	4	1	456	49.4	4.98	5.14	hypothetical protein [Arthrobacter sanguinis]

gi403231189	5.17	3	1	348	36.7	5.63	5.14	D-3-phosphoglycerate dehydrogenase SerA [Arthrobacter sp. Rue61a]
gi654823280	8.24	2	1	255	27.5	6.65	5.13	daunorubicin C-13 ketoreductase [Arthrobacter sp. I3]
gi307744354	2.99	1	1	368	39.8	5.11	5.13	histidinol-phosphate transaminase [Arthrobacter arilaitensis Re117]
gi908690255	4.37	2	1	481	51.7	6.62	5.12	hypothetical protein [Arthrobacter sp. H41]
gi359305020	1.37	2	1	952	97.6	4.82	5.12	hypothetical protein ARGLB_075_00010 [Arthrobacter globiformis NBRC 12137]
gi760112191	18.18	2	2	198	20.7	9.28	5.12	hypothetical protein [Arthrobacter chlorophenolicus]
gi757625112	4.53	1	1	243	25.7	6.35	5.12	hypothetical protein TV39_06210 [Arthrobacter sp. SPG23]
gi767257412	2.28	2	1	483	51.8	5.02	5.11	hypothetical protein UM93_06770 [Arthrobacter sp. IHBB 11108]
gi518312811	7.94	1	1	252	27.8	9.66	5.11	MULTISPECIES: hypothetical protein [Arthrobacter]
gi910252132	3.06	2	1	294	32.5	6.00	5.11	LysR family transcriptional regulator [Arthrobacter siccitolerans]
gi916835173	2.99	1	1	335	35.3	4.77	5.11	thioredoxin reductase [Arthrobacter sp. H14]
gi759725502	4.23	2	1	449	47.9	4.75	5.11	kynureninase [Arthrobacter sp. I3]
gi517604278	4.30	2	1	442	47.0	4.77	5.11	kynureninase [Arthrobacter sp. 131MFCol6.1]
gi742861247	2.41	2	1	706	77.1	5.80	5.11	ATP-dependent DNA helicase RecQ [Arthrobacter sp. W1]
gi737786463	3.75	4	1	293	32.9	6.58	5.11	DNA methyltransferase [Arthrobacter albus]
gi910742815	6.70	2	1	224	24.6	7.36	5.10	FAD dependent oxidoreductase [Arthrobacter sp. Hiyo8]
gi651440616	11.92	2	1	193	21.1	8.21	5.10	hypothetical protein [Arthrobacter sp. H14]
gi307744410	3.56	2	1	309	33.4	4.75	5.10	methylisocitrate lyase [Arthrobacter arilaitensis Re117]
gi640193361	3.41	4	1	469	51.0	5.71	5.10	dTDP-4-dehydrorhamnose reductase [Arthrobacter sp. 31Y]
gi307746563	4.34	5	1	461	48.3	5.20	5.10	malate dehydrogenase (oxaloacetate-decarboxylating) [Arthrobacter arilaitensis Re117]
gi742855548	1.65	6	1	1271	142.9	5.64	5.10	hypothetical protein [Arthrobacter sp. W1]
gi910737946	22.50	3	1	80	8.3	8.27	5.09	hypothetical protein AHiyo4_07680 [Arthrobacter sp. Hiyo4]
gi767259182	2.33	2	1	900	98.5	5.59	5.09	DNA topoisomerase I [Arthrobacter sp. IHBB 11108]
gi760166555	8.44	2	1	225	23.8	4.78	5.08	methyltransferase [Arthrobacter crystallopoietes]
gi551255650	1.83	2	1	493	53.7	5.54	5.08	sugar phosphate isomerase [Arthrobacter sp. PAO19]
gi476401763	10.00	1	1	100	10.8	10.58	5.07	hypothetical protein D477_007159 [Arthrobacter crystallopoietes BAB-32]
gi757625997	23.81	2	1	63	6.7	11.55	5.07	hypothetical protein TV39_02370 [Arthrobacter sp. SPG23]
gi918469448	3.68	2	1	543	56.8	6.71	5.07	hypothetical protein [Arthrobacter crystallopoietes]
gi517608657	11.61	2	1	155	16.9	6.13	5.07	GCN5 family N-acetyltransferase [Arthrobacter sp. 161MFSha2.1]
gi476400207	4.12	1	1	340	36.7	10.17	5.06	peptide ABC transporter permease [Arthrobacter crystallopoietes BAB-32]
gi759718744	3.36	12	1	327	34.7	6.42	5.06	LacI family transcriptional regulator [Arthrobacter sp. FB24]
gi476400695	6.91	2	1	275	29.9	9.29	5.05	short-chain dehydrogenase [Arthrobacter crystallopoietes BAB-32]
gi765005866	4.03	2	1	248	27.0	9.29	5.05	hypothetical protein [Arthrobacter sp. A3]
gi651439927	10.91	3	1	110	12.4	10.26	5.05	transposase, partial [Arthrobacter sp. H14]
gi765005831	2.33	2	1	557	59.0	6.58	5.05	FAD-binding dehydrogenase [Arthrobacter sp. A3]
gi517602922	4.68	3	1	363	40.1	8.87	5.05	mannosyltransferase [Arthrobacter sp. 131MFCol6.1]
gi654826597	2.30	1	1	521	56.7	5.73	5.05	GTP-binding protein [Arthrobacter sp. H5]
gi359305273	3.11	1	1	547	56.9	5.05	5.03	histidine ammonia-lyase [Arthrobacter globiformis NBRC 12137]
gi757625047	2.98	2	1	504	54.3	9.76	5.03	3-methyladenine DNA glycosylase [Arthrobacter sp. SPG23]
gi654824711	5.45	6	1	257	27.4	5.99	5.03	DeoR family transcriptional regulator [Arthrobacter sp. I3]
gi917760327	1.97	1	1	507	55.1	4.36	5.03	trigger factor [Arthrobacter sp. L77]
gi737805280	3.05	1	1	525	57.2	5.03	5.02	Xaa-Pro aminopeptidase [Arthrobacter sp. Br18]
gi162955632	4.35	3	1	322	32.6	4.81	5.02	electron transfer flavoprotein alpha-subunit [Renibacterium salmoninarum ATCC 33209]
gi742755834	3.86	3	1	415	44.9	9.67	5.01	DNA polymerase IV [Arthrobacter phenanthrenivorans]
gi671528765	5.83	2	1	309	32.9	5.01	5.01	prephenate dehydratase [Streptomyces sp. NRRL F-5123]
gi651485724	2.79	3	1	574	62.6	6.83	5.00	glycerol-3-phosphate dehydrogenase [Arthrobacter sp. Br18]
gi470221403	3.81	1	1	446	50.7	5.94	5.00	putative acrAB operon repressor [Arthrobacter gangotriensis Lz1y]
gi652424980	5.28	1	1	417	44.3	5.62	5.00	two-component system sensor histidine kinase [Arthrobacter castelli]
gi927032014	11.39	6	1	158	17.0	6.52	4.99	phosphohydrolase [Arthrobacter sp. LS16]
gi914716033	7.78	3	1	270	27.6	5.62	4.99	hypothetical protein [Arthrobacter sp. ZBG10]
gi916574055	8.00	2	1	275	27.8	5.30	4.99	hypothetical protein [Arthrobacter sp. TB 26]
gi918469316	4.64	1	1	345	37.9	5.14	4.98	hypothetical protein [Arthrobacter crystallopoietes]
gi930827677	1.81	2	1	553	58.6	5.08	4.98	amidohydrolase [Arthrobacter arilaitensis]
gi654828318	10.00	2	1	220	24.1	5.33	4.97	hypothetical protein [Arthrobacter sp. H5]
gi116609542	4.62	5	1	260	27.4	5.55	4.96	methionine aminopeptidase, type I [Arthrobacter sp. FB24]
gi654819352	10.19	1	1	206	22.1	4.93	4.96	Zn-dependent hydrolase [Arthrobacter sp. UNC362MFTsu5.1]
gi918268074	7.58	1	1	277	30.9	5.97	4.96	exodeoxyribonuclease [Arthrobacter sp. Hiyo1]
gi116608716	17.17	2	2	233	24.9	5.27	4.96	transcriptional regulator [Arthrobacter sp. FB24]

gi767257458	5.47	2	1	128	14.1	8.34	4.96	MerR family transcriptional regulator [Arthrobacter sp. IHBB 11108]
gi162953499	3.49	2	1	315	33.7	4.93	4.96	flavoprotein involved in K+ transport [Renibacterium salmoninarum ATCC 33209]
gi542110283	2.97	1	1	573	60.0	5.54	4.96	dihydroxy-acid dehydratase [Arthrobacter sp. AK-YN10]
gi937261958	4.30	2	1	349	36.8	6.37	4.95	exopolyphosphatase [Arthrobacter sp. Edens01]
gi757624363	3.50	6	1	400	43.7	5.85	4.95	acyl-CoA dehydrogenase [Arthrobacter sp. SPG23]
gi765012202	3.33	2	1	570	62.5	6.89	4.94	ABC transporter [Arthrobacter sp. A3]
gi927032810	3.76	1	1	426	47.1	5.81	4.94	hypothetical protein AFL94_11430 [Arthrobacter sp. LS16]
gi910252429	5.58	5	1	197	21.5	5.41	4.94	TetR family transcriptional regulator [Arthrobacter siccitolerans]
gi162955208	2.38	1	1	589	66.4	9.77	4.94	decaprenyl-phosphate-mannose--protein mannosyltransferase [Renibacterium salmoninaru
gi823665719	4.19	1	1	501	56.0	5.10	4.93	glutamyl-tRNA synthetase [Arthrobacter sp. YC-RL1]
gi640197665	3.03	4	1	495	52.2	5.41	4.93	methylmalonate-semialdehyde dehydrogenase [Arthrobacter sp. 31Y]
gi551254507	5.33	1	1	319	33.7	4.97	4.93	D-glycerate dehydrogenase [Arthrobacter sp. PAO19]
gi651501025	4.14	1	1	532	57.8	5.59	4.92	ABC transporter [Arthrobacter sp. 35W]
gi914715602	1.94	2	1	773	83.0	5.00	4.92	maltooligosyl trehalose synthase [Arthrobacter sp. ZBG10]
gi916691314	5.83	3	1	206	21.6	8.62	4.92	hypothetical protein [Arthrobacter castelli]
gi742757134	2.96	1	1	575	61.4	6.39	4.92	membrane protein [Arthrobacter phenanthrenivorans]
gi648575298	3.13	2	1	512	56.4	4.94	4.92	diguanylate phosphodiesterase [Arthrobacter sp. 161MFSha2.1]
gi162955574	7.43	5	1	175	18.6	9.63	4.92	A3(2) glycogen metabolism cluster I [Renibacterium salmoninarum ATCC 33209]
gi767258123	8.95	1	1	257	27.1	5.85	4.91	gluconate 5-dehydrogenase [Arthrobacter sp. IHBB 11108]
gi918267670	4.53	4	1	375	39.3	10.23	4.91	O-acetyl transferase [Arthrobacter sp. Hiyo1]
gi517602679	6.12	3	1	392	40.8	10.70	4.91	hypothetical protein [Arthrobacter sp. 131MFCol6.1]
gi760166425	6.10	2	1	295	32.6	5.39	4.90	acyl-CoA thioesterase II [Arthrobacter crystallopoietes]
gi737779809	4.49	5	1	245	26.0	6.80	4.90	aldolase [Arthrobacter sp. CAL618]
gi917760241	1.83	1	1	1310	135.8	11.63	4.90	hypothetical protein [Arthrobacter sp. L77]
gi742755452	5.19	1	1	231	24.4	4.70	4.89	molecular chaperone GrpE [Arthrobacter phenanthrenivorans]
gi767257845	10.90	1	1	156	17.2	10.33	4.89	30S ribosomal protein S7 [Arthrobacter sp. IHBB 11108]
gi937258762	1.98	4	1	353	37.7	10.77	4.89	hypothetical protein AO716_12365 [Arthrobacter sp. Edens01]
gi908696808	3.61	2	1	527	56.0	5.49	4.89	GTPase CgtA [Arthrobacter sp. RIT-PI-e]
gi910743479	11.38	5	1	123	12.4	4.88	4.89	cell division protein FtsZ [Arthrobacter sp. Hiyo8]
gi116612148	7.55	4	1	265	28.9	5.94	4.89	transcriptional regulator, IclR family [Arthrobacter sp. FB24]
gi760113122	4.82	5	1	249	27.7	6.18	4.89	hypothetical protein [Arthrobacter chlorophenolicus]
gi651431360	7.46	2	1	295	30.6	5.27	4.89	bifunctional 5,10-methylene-tetrahydrofolate dehydrogenase/5,10-methylene-tetrahydrofol
gi767258586	4.18	2	1	287	30.1	11.41	4.89	hypothetical protein UM93_14955 [Arthrobacter sp. IHBB 11108]
gi654817616	8.13	2	1	160	17.1	9.04	4.88	MarR family transcriptional regulator [Arthrobacter sp. UNC362MFTsu5.1]
gi927033791	2.74	1	1	402	43.2	7.05	4.88	hydroxyglutarate oxidase [Arthrobacter sp. LS16]
gi651462200	1.96	2	1	561	57.4	4.64	4.88	phosphoenolpyruvate-protein phosphotransferase [Arthrobacter sp. 35/47]
gi116611012	7.41	8	1	162	17.7	8.02	4.88	NrdI protein [Arthrobacter sp. FB24]
gi517606855	4.95	1	1	202	22.4	7.94	4.88	hypothetical protein [Arthrobacter sp. 161MFSha2.1]
gi443479943	1.44	1	1	417	43.0	5.52	4.88	acetylornithine aminotransferase [Arthrobacter nitrophenolicus]
gi517602517	1.71	1	1	642	67.4	6.18	4.86	hypothetical protein [Arthrobacter sp. 131MFCol6.1]
gi674645086	1.72	2	1	581	61.6	5.22	4.86	V-type ATP synthase alpha chain [Arthrobacter sp. 11W110_air]
gi910251229	2.58	2	1	854	90.0	9.23	4.86	transglutaminase [Arthrobacter siccitolerans]
gi162955635	4.65	1	1	409	43.0	5.34	4.86	3-ketoacyl-CoA thiolase [Renibacterium salmoninarum ATCC 33209]
gi914715504	6.18	2	1	275	28.4	5.81	4.86	hypothetical protein [Arthrobacter sp. ZBG10]
gi823667626	7.74	1	1	155	16.8	4.78	4.86	AsnC family transcriptional regulator [Arthrobacter sp. YC-RL1]
gi916820278	6.25	1	1	192	21.1	5.74	4.85	5-formyltetrahydrofolate cyclo-ligase [Arthrobacter sp. H20]
gi651436947	8.41	1	1	226	25.1	5.53	4.85	GntR family transcriptional regulator [Arthrobacter sp. H41]
gi518312044	3.42	1	1	497	52.7	4.97	4.85	MULTISPECIES: NAD-dependent succinate-semialdehyde dehydrogenase [Arthrobacter]
gi759735310	3.38	2	1	592	64.6	6.57	4.85	hypothetical protein [Arthrobacter sp. L77]
gi910696792	21.62	2	1	74	7.7	5.17	4.85	uncharacterized protein YbxB [Arthrobacter sp. Hiyo6]
gi746184670	3.22	2	1	497	54.0	6.27	4.84	PucR family transcriptional regulator [Arthrobacter sp. MWB30]
gi551255594	3.31	3	2	846	89.4	4.89	4.84	phenylalanine--tRNA ligase subunit beta [Arthrobacter sp. PAO19]
gi742071743	3.93	3	1	433	45.9	5.53	4.84	ABC transporter substrate-binding protein YesO [Arthrobacter sp. MWB30]
gi765011169	0.46	1	1	3477	371.9	5.38	4.84	non-ribosomal peptide synthetase [Arthrobacter sp. A3]
gi651435596	3.89	2	1	257	28.0	5.63	4.83	ABC transporter ATP-binding protein [Arthrobacter sp. H41]
gi654816357	4.35	1	1	322	34.3	7.33	4.83	ABC transporter [Arthrobacter sp. UNC362MFTsu5.1]
gi651438720	6.50	1	1	277	30.6	5.10	4.83	oxidoreductase [Arthrobacter sp. H14]
gi307745787	1.94	3	1	775	81.4	6.28	4.83	ABC transporter, inner membrane and ATP-binding subunits [Arthrobacter arilaitensis Re11

gi654816793	5.11	7	1	235	24.6	4.79	4.83	hypothetical protein [Arthrobacter sp. UNC362MFTsu5.1]
gi517599721	8.64	2	1	220	24.9	4.65	4.83	hypothetical protein [Arthrobacter sp. 162MFSha1.1]
gi162953692	6.21	2	2	306	32.3	5.47	4.82	glutamate racemase [Renibacterium salmoninarum ATCC 33209]
gi443480216	4.36	2	1	275	29.7	7.40	4.82	carbohydrate ABC transporter membrane protein 1, CUT1 family [Arthrobacter nitrophenoli
gi443483043	3.33	4	1	660	69.8	7.77	4.82	putative amino acid transporter [Arthrobacter nitrophenolicus]
gi737786861	5.73	3	2	593	64.9	6.29	4.82	hypothetical protein [Arthrobacter albus]
gi930827660	1.98	1	1	506	53.5	4.67	4.82	ABC transporter substrate-binding protein [Arthrobacter arilaitensis]
gi651439134	4.62	1	1	346	37.3	7.11	4.82	LacI family transcriptional regulator [Arthrobacter sp. H14]
gi116610101	4.23	4	1	284	32.6	6.18	4.82	Tryptophan 2,3-dioxygenase apoenzyme / Tryptophan 2,3-dioxygenase holoenzyme [Arthr
gi651506456	5.25	3	1	305	33.2	7.05	4.81	hypothetical protein [Arthrobacter sp. 35W]
gi542106678	3.83	1	1	209	23.5	8.84	4.81	hypothetical protein M707_20580 [Arthrobacter sp. AK-YN10]
gi542110621	4.58	4	1	262	27.0	6.39	4.81	cystathionine beta-lyase [Arthrobacter sp. AK-YN10]
gi740676385	11.87	3	2	219	23.9	6.81	4.80	GntR family transcriptional regulator [Arthrobacter sp. PAMC25486]
gi654819266	2.40	3	1	375	40.4	7.62	4.80	hypothetical protein [Arthrobacter sp. UNC362MFTsu5.1]
gi916813813	5.10	1	1	510	53.0	7.56	4.80	oxidoreductase [Arthrobacter nicotinovorans]
gi654815847	7.01	5	1	157	17.3	4.41	4.80	16S rRNA maturation RNase YbeY [Arthrobacter sp. UNC362MFTsu5.1]
gi759725730	3.70	1	1	459	50.5	8.16	4.80	hypothetical protein [Arthrobacter sp. I3]
gi823667609	4.02	2	1	423	45.5	5.27	4.80	isochorismate synthase [Arthrobacter sp. YC-RL1]
gi916691670	1.97	5	1	660	72.8	10.08	4.80	hypothetical protein [Arthrobacter castelli]
gi910744418	3.30	4	1	303	32.8	5.73	4.79	probable acetolactate synthase large subunit [Arthrobacter sp. Hiyo8]
gi742759537	3.56	3	1	449	47.6	5.19	4.79	acetyl-CoA carboxylase [Arthrobacter phenanthrenivorans]
gi939036636	3.09	2	1	259	29.7	5.11	4.79	hypothetical protein [Arthrobacter nitroguajacolicus]
gi742857416	4.25	2	1	400	40.6	9.42	4.79	MFS transporter [Arthrobacter sp. W1]
gi651484943	5.05	2	1	436	45.4	6.34	4.79	acetyl-CoA acetyltransferase [Arthrobacter sp. Br18]
gi443482393	1.86	4	1	485	51.0	8.69	4.79	phytoene dehydrogenase-like oxidoreductase [Arthrobacter nitrophenolicus]
gi916692542	4.58	2	1	262	28.3	5.63	4.79	hypothetical protein [Arthrobacter castelli]
gi937258264	1.93	4	1	727	72.4	5.19	4.79	PTS lactose transporter subunit IIC [Arthrobacter sp. Edens01]
gi818631453	11.17	3	1	188	21.1	10.13	4.78	putative RNA polymerase sigma factor (plasmid) [Arthrobacter sp. 68b]
gi928487115	2.11	3	1	521	54.2	8.47	4.78	Mg chelatase-like protein [Arthrobacter alpinus]
gi674645039	1.56	3	1	767	78.8	5.43	4.78	Copper-exporting P-type ATPase A [Arthrobacter sp. 11W110_air]
gi652423074	3.19	5	2	879	96.4	4.93	4.78	aminopeptidase N [Arthrobacter castelli]
gi640199686	9.64	3	1	249	26.2	4.79	4.77	hypothetical protein [Arthrobacter sp. 31Y]
gi116610375	4.73	2	1	444	47.5	5.54	4.77	carbohydrate ABC transporter substrate-binding protein, CUT1 family [Arthrobacter sp. FB2
gi640200244	5.45	2	1	312	32.7	5.02	4.77	phosphoglycerate dehydrogenase [Arthrobacter sp. 31Y]
gi742853133	8.27	1	1	278	29.5	5.00	4.77	thiosulfate sulfurtransferase [Arthrobacter sp. W1]
gi640199853	16.54	1	1	133	14.8	9.25	4.77	DNA-binding protein [Arthrobacter sp. 31Y]
gi651438782	4.01	5	1	548	58.4	5.19	4.76	acetolactate synthase [Arthrobacter sp. H14]
gi654814076	5.05	1	1	198	22.3	9.20	4.76	hypothetical protein [Arthrobacter sp. MA-N2]
gi917739712	11.54	5	1	156	17.2	8.82	4.76	hypothetical protein [Arthrobacter sp. W1]
gi928488442	13.94	5	2	208	22.7	5.27	4.76	hypothetical protein AOC05_18000 [Arthrobacter alpinus]
gi652424810	3.42	3	1	527	55.2	5.24	4.76	histidine ammonia-lyase [Arthrobacter castelli]
gi930825449	5.60	2	1	357	39.4	8.41	4.76	ACP synthase [Arthrobacter arilaitensis]
gi760164616	8.03	1	1	249	27.0	5.94	4.76	peptide ABC transporter ATP-binding protein [Arthrobacter crystallopoietes]
gi636845420	2.97	3	1	538	58.6	9.32	4.76	relaxase [Arthrobacter sp. TB 26]
gi823666259	2.93	2	1	341	37.1	9.14	4.75	cytochrome C oxidase assembly protein [Arthrobacter sp. YC-RL1]
gi767258852	5.82	1	1	275	30.6	5.55	4.75	exodeoxyribonuclease III [Arthrobacter sp. IHBB 11108]
gi757624253	7.46	3	1	228	25.8	8.91	4.75	DNA alkylation repair protein [Arthrobacter sp. SPG23]
gi359304718	2.91	3	1	516	51.5	9.96	4.75	peptidase M23 family protein [Arthrobacter globiformis NBRC 12137]
gi767258842	1.04	2	1	1349	148.7	5.58	4.75	Type II restriction enzyme methylase subunit [Arthrobacter sp. IHBB 11108]
gi219858528	11.00	2	1	100	10.9	4.26	4.75	conserved hypothetical protein [Arthrobacter chlorophenolicus A6]
gi162952426	7.37	1	1	312	34.4	5.63	4.75	transcriptional regulator, LysR family [Renibacterium salmoninarum ATCC 33209]
gi119951470	3.26	2	2	1165	128.7	5.05	4.75	helicase, SNF2 family (plasmid) [Arthrobacter aurescens TC1]
gi476399242	12.07	5	1	116	12.5	6.55	4.75	4'-phosphopantetheinyl transferase [Arthrobacter crystallopoietes BAB-32]
gi759723191	2.64	1	1	454	48.3	8.92	4.75	ATPase [Arthrobacter sp. I3]
gi908699005	2.66	1	1	865	93.9	4.61	4.75	beta-mannosidase [Arthrobacter sp. RIT-PI-e]
gi651506468	3.01	2	1	299	32.4	10.59	4.74	secretion system protein [Arthrobacter sp. 35W]
gi651465976	6.03	2	1	199	20.3	4.82	4.74	50S ribosomal protein L10 [Arthrobacter sp. 35/47]
gi916691541	3.73	4	1	295	31.5	4.89	4.74	methyltransferase type 11 [Arthrobacter castelli]

gi916357144	4.39	2	1	228	24.1	6.96	4.74	haloacid dehalogenase [Arthrobacter sp. 162MFSha1.1]
gi742861338	4.82	2	1	249	27.5	11.37	4.74	membrane protein [Arthrobacter sp. W1]
gi517605646	2.36	5	1	679	73.3	5.40	4.74	alpha-glycosidase [Arthrobacter sp. 131MFCol6.1]
gi818631445	10.49	2	1	143	15.8	5.22	4.74	hypothetical protein (plasmid) [Arthrobacter sp. 68b]
gi116610285	4.30	3	1	442	46.5	11.55	4.74	major facilitator superfamily MFS_1 [Arthrobacter sp. FB24]
gi652425393	5.67	10	1	282	31.9	9.52	4.74	phosphate ABC transporter permease [Arthrobacter castelli]
gi742853590	12.87	2	1	101	10.8	11.71	4.74	hypothetical protein [Arthrobacter sp. W1]
gi916834581	2.83	5	1	565	63.2	6.87	4.74	hypothetical protein [Arthrobacter sp. H14]
gi908698050	2.39	2	1	293	32.0	6.10	4.73	ABC transporter ATP-binding protein [Arthrobacter sp. RIT-PI-e]
gi518311499	2.68	2	1	336	36.2	5.74	4.73	MULTISPECIES: hypothetical protein [Arthrobacter]
gi652424475	3.98	3	1	427	44.6	4.32	4.73	hypothetical protein [Arthrobacter castelli]
gi470216532	5.56	3	1	414	44.5	5.26	4.73	assimilatory nitrate reductase electron transfer subunit [Arthrobacter gangotriensis Lz1y]
gi518313803	20.37	2	1	108	11.7	4.32	4.73	MULTISPECIES: rhodanese-like domain-containing protein [Arthrobacter]
gi654822833	3.17	1	1	473	50.8	5.78	4.73	dihydropyrimidinase, partial [Arthrobacter sp. I3]
gi323469345	3.65	2	1	384	40.0	5.54	4.73	ADP-heptose:LPS heptosyltransferase [Arthrobacter phenanthrenivorans Sphe3]
gi930827505	7.98	6	1	326	35.8	5.12	4.73	hydrolase [Arthrobacter arilaitensis]
gi737790026	6.23	3	1	273	29.4	5.24	4.72	indole-3-glycerol phosphate synthase [Arthrobacter albus]
gi651436602	3.05	2	1	459	50.0	7.81	4.72	MFS transporter [Arthrobacter sp. H41]
gi307746355	13.64	3	1	88	9.8	9.74	4.72	conserved hypothetical protein [Arthrobacter arilaitensis Re117]
gi651474564	2.19	2	1	593	65.9	5.17	4.72	2-isopropylmalate synthase [Arthrobacter nicotinovorans]
gi648572501	1.47	4	1	817	85.4	6.64	4.72	glycoside hydrolase [Arthrobacter sp. 135MFCol5.1]
gi470216472	1.56	1	1	512	57.1	6.86	4.72	putative rhizopine catabolism regulatory protein mocR [Arthrobacter gangotriensis Lz1y]
gi937262032	1.70	2	1	471	50.2	5.83	4.72	PucR family transcriptional regulator [Arthrobacter sp. Edens01]
gi517606784	3.96	2	1	429	47.1	5.39	4.72	ABC transporter substrate-binding protein [Arthrobacter sp. 161MFSha2.1]
gi927033576	4.12	1	1	388	38.8	4.63	4.71	glycerate kinase [Arthrobacter sp. LS16]
gi652425078	14.74	1	1	156	17.0	10.35	4.71	30S ribosomal protein S7 [Arthrobacter castelli]
gi908699164	6.50	1	1	277	31.0	5.52	4.71	oxidoreductase [Arthrobacter sp. RIT-PI-e]
gi674645075	8.86	2	2	361	39.0	5.63	4.71	Low specificity L-threonine aldolase [Arthrobacter sp. 11W110_air]
gi517603095	5.84	2	1	257	30.2	6.81	4.71	hypothetical protein [Arthrobacter sp. 131MFCol6.1]
gi910695207	6.06	3	1	231	24.1	8.38	4.71	hypothetical protein AHiyo6_21030 [Arthrobacter sp. Hiyo6]
gi654813783	2.93	2	1	443	47.8	4.59	4.71	hypothetical protein [Arthrobacter sp. MA-N2]
gi914714953	1.58	1	1	507	52.1	9.45	4.71	transporter [Arthrobacter sp. ZBG10]
gi737814365	26.89	3	2	119	13.4	9.70	4.71	hypothetical protein [Arthrobacter sp. H14]
gi651448821	2.92	2	1	479	51.7	5.91	4.71	membrane protein [Arthrobacter nicotinovorans]
gi517602509	3.72	1	1	403	42.6	5.47	4.70	hypothetical protein [Arthrobacter sp. 131MFCol6.1]
gi651501289	4.07	1	1	467	48.8	5.86	4.70	flavoprotein [Arthrobacter sp. 35W]
gi403229534	4.86	3	1	391	42.7	6.76	4.70	transcriptional regulator, TetR family [Arthrobacter sp. Rue61a]
gi937259361	2.09	1	1	719	76.1	8.05	4.70	primosomal protein N' [Arthrobacter sp. Edens01]
gi307745698	5.41	1	1	407	42.5	5.80	4.70	putative acetyl-CoA C-acyltransferase [Arthrobacter arilaitensis Re117]
gi639131125	12.77	2	1	141	15.2	5.26	4.70	hypothetical protein [Arthrobacter sp. CAL618]
gi674646311	4.56	2	1	439	46.8	4.25	4.70	Trehalose-binding lipoprotein LpqY precursor [Arthrobacter sp. 11W110_air]
gi928486488	7.50	1	1	200	21.4	5.12	4.70	hypothetical protein AOC05_04980 [Arthrobacter alpinus]
gi910283811	3.26	1	1	522	57.2	10.71	4.69	hypothetical protein [Arthrobacter sp. A3]
gi767256471	8.41	3	1	214	23.4	5.44	4.69	TetR family transcriptional regulator [Arthrobacter sp. IHBB 11108]
gi928486985	22.08	4	2	77	8.5	4.48	4.69	hypothetical protein AOC05_08310 [Arthrobacter alpinus]
gi651495361	4.42	2	1	226	23.9	4.89	4.69	hypothetical protein [Arthrobacter sp. H20]
gi910697381	13.27	1	1	98	10.8	4.74	4.69	rutC family protein bbp_334 [Arthrobacter sp. Hiyo6]
gi823666275	4.82	2	1	249	27.6	11.18	4.69	membrane protein [Arthrobacter sp. YC-RL1]
gi760112227	3.30	3	1	546	58.9	7.12	4.69	peptide ABC transporter substrate-binding protein [Arthrobacter chlorophenolicus]
gi551256874	4.28	2	1	304	33.5	4.58	4.68	hypothetical protein [Arthrobacter sp. PAO19]
gi518314033	8.59	3	1	128	13.7	9.14	4.68	hypothetical protein [Arthrobacter sp. TB 23]
gi359307728	4.85	1	1	268	28.6	9.17	4.68	hypothetical protein ARGLB_008_00240 [Arthrobacter globiformis NBRC 12137]
gi443479959	3.64	1	1	494	52.4	5.54	4.68	methylmalonate-semialdehyde dehydrogenase [Arthrobacter nitrophenolicus]
gi937261888	2.56	1	1	703	76.4	6.10	4.68	choline transporter [Arthrobacter sp. Edens01]
gi651499769	7.23	1	1	235	25.3	6.57	4.67	hypothetical protein [Arthrobacter sp. 35W]
gi651438047	4.79	1	1	355	39.5	7.50	4.67	hypothetical protein [Arthrobacter sp. H14]
gi476399679	2.78	2	1	395	40.4	6.14	4.67	galactokinase [Arthrobacter crystallopoietes BAB-32]
gi723606816	9.31	2	1	204	21.1	5.01	4.67	dimethylmenaquinone methyltransferase [Arthrobacter sp. PAMC25486]

gi639129117	2.55	3	1	666	74.2	6.42	4.67	glycosyl transferase [Arthrobacter sp. CAL618]
gi674645478	5.49	2	1	273	28.3	4.64	4.67	Virginiamycin B lyase [Arthrobacter sp. 11W110_air]
gi910250284	8.59	4	1	198	22.4	9.14	4.67	hypothetical protein [Arthrobacter siccitolerans]
gi753932847	17.65	3	1	85	9.9	11.41	4.66	hypothetical protein [Arthrobacter arilaitensis]
gi917442071	1.72	2	1	1107	115.8	6.70	4.66	hypothetical protein [Arthrobacter albus]
gi910743300	2.74	3	1	328	35.8	6.02	4.66	probable aldo-keto reductase 2 [Arthrobacter sp. Hiyo8]
gi635353690	2.91	1	1	309	33.3	5.30	4.66	aldo/keto reductase family protein [Arthrobacter siccitolerans]
gi551254947	0.92	1	1	1092	117.3	4.88	4.66	carbamoyl phosphate synthase large subunit [Arthrobacter sp. PAO19]
gi927292895	5.42	1	1	332	35.0	4.93	4.66	universal stress protein UspA (plasmid) [Arthrobacter sp. ERGS1:01]
gi749402326	2.50	1	1	559	61.4	10.10	4.66	polyprenyl glycosylphosphotransferase [Arthrobacter sp. AK-YN10]
gi927293644	4.37	1	1	412	42.9	6.20	4.66	threonine dehydratase [Arthrobacter sp. ERGS1:01]
gi359303909	4.20	2	1	333	32.9	5.03	4.65	glycerate kinase [Arthrobacter globiformis NBRC 12137]
gi916816056	4.04	3	1	446	50.4	9.73	4.65	hypothetical protein [Arthrobacter sp. MA-N2]
gi757624382	1.59	2	1	628	70.7	5.10	4.65	hypothetical protein TV39_10210 [Arthrobacter sp. SPG23]
gi928488541	2.42	6	1	661	71.7	7.78	4.65	ABC transporter [Arthrobacter alpinus]
gi723608731	17.48	1	1	103	10.4	11.05	4.65	hypothetical protein ART_2508 [Arthrobacter sp. PAMC25486]
gi654824336	2.21	2	1	497	53.8	8.78	4.65	coenzyme F390 synthetase [Arthrobacter sp. I3]
gi517592204	3.26	1	1	215	23.8	5.30	4.64	hypothetical protein [Arthrobacter sp. 135MFCol5.1]
gi908699196	4.06	3	1	271	30.4	7.52	4.64	UDP pyrophosphate synthase [Arthrobacter sp. RIT-PI-e]
gi307744464	10.69	1	1	159	17.0	6.27	4.64	universal stress family domain-containing protein [Arthrobacter arilaitensis Re117]
gi765006852	4.23	1	1	355	36.7	5.19	4.64	dipeptide epimerase [Arthrobacter sp. A3]
gi636843570	2.41	1	1	290	30.9	6.04	4.64	universal stress protein UspA [Arthrobacter sp. TB 26]
gi651445156	5.60	1	1	232	24.9	6.80	4.63	GntR family transcriptional regulator [Arthrobacter nicotinovorans]
gi323467813	2.13	2	1	798	88.6	6.09	4.63	phosphoketolase [Arthrobacter phenanthrenivorans Sphe3]
gi927033431	12.30	1	1	187	20.4	4.79	4.63	deaminase [Arthrobacter sp. LS16]
gi651504344	4.66	2	1	365	38.5	7.28	4.63	hypothetical protein [Arthrobacter sp. 35W]
gi765006833	2.72	1	1	514	55.6	5.73	4.63	hypothetical protein [Arthrobacter sp. A3]
gi916692066	3.79	1	1	422	44.6	10.42	4.63	transporter [Arthrobacter castelli]
gi915933513	6.19	1	1	194	20.2	11.80	4.63	hypothetical protein [Arthrobacter globiformis]
gi916820479	7.88	1	1	241	27.0	6.10	4.63	RNA polymerase [Arthrobacter sp. H20]
gi737776180	6.94	2	1	317	33.8	5.94	4.63	hypothetical protein [Arthrobacter sanguinis]
gi765003943	9.09	1	1	132	14.4	9.98	4.63	hypothetical protein [Arthrobacter sp. A3]
gi219862185	2.82	1	1	354	36.8	6.06	4.62	iron-containing alcohol dehydrogenase (plasmid) [Arthrobacter chlorophenolicus A6]
gi916781775	2.16	2	1	462	49.8	10.02	4.62	oxidoreductase [Arthrobacter sp. 35W]
gi910696616	11.85	2	1	135	14.8	7.15	4.62	low molecular weight protein-tyrosine-phosphatase etp, partial [Arthrobacter sp. Hiyo6]
gi307746516	2.29	3	1	393	42.0	5.64	4.62	isochorismate synthase [Arthrobacter arilaitensis Re117]
gi910749262	19.35	1	1	93	9.7	9.31	4.62	hypothetical protein AHiyo8_pI69640 (plasmid) [Arthrobacter sp. Hiyo8]
gi359307216	6.61	2	1	333	34.6	8.02	4.62	hypothetical protein ARGLB_023_00240 [Arthrobacter globiformis NBRC 12137]
gi323467643	4.38	2	1	388	43.2	5.88	4.62	putative dehydrogenase [Arthrobacter phenanthrenivorans Sphe3]
gi470217479	8.00	2	1	250	27.5	4.64	4.62	MerR family transcriptional regulator [Arthrobacter gangotriensis Lz1y]
gi651451812	5.23	2	1	344	35.7	5.31	4.62	uroporphyrin-III methyltransferase [Arthrobacter nicotinovorans]
gi759731294	27.40	1	1	73	8.1	6.92	4.62	hypothetical protein [Arthrobacter sp. L77]
gi759732379	9.73	2	2	257	26.6	3.94	4.62	hypothetical protein [Arthrobacter sp. L77]
gi515767468	5.83	1	1	240	26.3	5.81	4.62	hypothetical protein [Arthrobacter sp. M2012083]
gi651436578	5.39	1	1	241	26.3	5.88	4.61	GntR family transcriptional regulator [Arthrobacter sp. H41]
gi654813700	1.24	2	1	808	89.2	6.11	4.61	phosphoketolase [Arthrobacter sp. MA-N2]
gi651493041	9.45	1	1	201	22.1	5.33	4.61	SAM-dependent methyltransferase [Arthrobacter sp. H20]
gi742857135	4.26	1	1	329	35.3	9.92	4.61	hypothetical protein [Arthrobacter sp. W1]
gi470220405	6.52	3	1	414	46.3	8.68	4.61	transposase IS204//IS1096/IS1165 family protein [Arthrobacter gangotriensis Lz1y]
gi740683971	1.19	2	1	670	74.6	6.37	4.61	glycosyl transferase [Arthrobacter sp. PAMC25486]
gi914716259	1.81	2	1	498	52.5	4.86	4.61	betaine-aldehyde dehydrogenase [Arthrobacter sp. ZBG10]
gi917759998	1.72	1	1	757	79.5	5.20	4.61	hypothetical protein [Arthrobacter sp. L77]
gi930825367	5.56	1	1	252	28.4	5.11	4.61	hypothetical protein AOZ07_03005 [Arthrobacter arilaitensis]
gi639128918	3.18	5	1	534	57.8	8.31	4.60	ATP/GTP-binding protein [Arthrobacter sp. CAL618]
gi323469438	3.56	1	1	393	41.9	5.57	4.60	coproporphyrinogen III oxidase, anaerobic [Arthrobacter phenanthrenivorans Sphe3]
gi636843716	4.12	1	1	437	47.9	5.58	4.60	hypothetical protein [Arthrobacter sp. TB 26]
gi162953030	7.26	1	1	234	24.8	6.05	4.60	DNA-3-methyladenine glycosylase II [Renibacterium salmoninarum ATCC 33209]
gi651480448	2.41	1	1	704	77.7	5.20	4.60	elongation factor G [Arthrobacter sp. Br18]

gi757625829	5.09	1	1	393	43.2	5.33	4.60	acyl-CoA dehydrogenase [Arthrobacter sp. SPG23]
gi359305367	2.43	1	1	411	43.9	5.11	4.60	CaiB/BaiF family protein [Arthrobacter globiformis NBRC 12137]
gi403231692	16.30	1	1	92	10.2	6.10	4.60	hypothetical protein ARUE_c42420 [Arthrobacter sp. Rue61a]
gi636845306	4.87	9	1	472	48.9	5.99	4.59	acetyl-CoA acetyltransferase [Arthrobacter sp. TB 26]
gi170783579	3.80	1	1	316	32.9	8.00	4.59	unknown (plasmid) [Arthrobacter sp. Chr15]
gi786031312	5.11	1	1	313	32.5	5.34	4.59	hydroxyacid dehydrogenase [Arthrobacter chlorophenolicus]
gi651506104	4.29	1	1	443	47.2	5.82	4.59	sugar ABC transporter substrate-binding protein [Arthrobacter sp. 35W]
gi930826093	9.09	2	1	165	18.2	9.28	4.59	hypothetical protein AOZ07_07210 [Arthrobacter arilaitensis]
gi916870109	8.15	1	1	270	30.1	10.77	4.59	hypothetical protein [Arthrobacter sp. Br18]
gi937256401	4.36	2	1	505	54.2	4.81	4.59	oxidoreductase [Arthrobacter sp. Edens01]
gi219860420	7.60	1	1	171	17.7	8.98	4.59	hypothetical protein Achl_2797 [Arthrobacter chlorophenolicus A6]
gi359304283	2.76	1	1	544	57.7	5.64	4.58	putative peptide ABC transporter peptide-binding protein [Arthrobacter globiformis NBRC 1
gi930825471	5.95	2	1	269	28.2	4.84	4.58	alpha-dehydro-beta-deoxy-D-glucarate aldolase [Arthrobacter arilaitensis]
gi737814712	17.86	1	1	84	9.4	4.84	4.58	hypothetical protein, partial [Arthrobacter sp. H14]
gi654822520	4.04	1	1	446	46.1	9.44	4.58	3-ketoacyl-ACP reductase [Arthrobacter sp. I3]
gi651502102	5.43	1	1	405	44.5	4.96	4.58	beta-glucosidase [Arthrobacter sp. 35W]
gi119948363	5.67	2	1	335	36.6	5.38	4.58	putative acetyl xylan esterase [Arthrobacter aurescens TC1]
gi742756507	5.75	1	1	348	35.6	8.75	4.58	hypothetical protein RM50_09745 [Arthrobacter phenanthrenivorans]
gi737789473	8.63	2	1	139	14.9	9.63	4.58	hypothetical protein [Arthrobacter albus]
gi652423585	4.37	2	1	252	27.0	4.92	4.58	hypothetical protein [Arthrobacter castelli]
gi648574604	6.74	2	1	178	19.6	6.10	4.58	GCN5 family acetyltransferase [Arthrobacter sp. 131MFCol6.1]
gi640203458	2.44	1	1	573	62.1	6.40	4.57	hypothetical protein [Arthrobacter sp. 31Y]
gi517608824	4.26	1	1	258	26.8	8.57	4.57	multidrug ABC transporter ATP-binding protein [Arthrobacter sp. 161MFSha2.1]
gi917407336	9.87	1	1	152	17.2	4.73	4.57	ribosomal-protein-alanine N-acetyltransferase RimI [Arthrobacter nitrophenolicus]
gi918266124	7.78	4	1	167	17.5	5.34	4.57	lipid A export ATP-binding/permease protein MsbA [Arthrobacter sp. Hiyo1]
gi307743240	10.84	2	2	286	31.8	5.68	4.57	hypothetical protein AARI_pI00220 (plasmid) [Arthrobacter arilaitensis Re117]
gi910695457	4.20	1	1	381	41.5	7.58	4.57	ribosomal RNA small subunit methyltransferase H, partial [Arthrobacter sp. Hiyo6]
gi119949382	2.60	1	1	384	41.7	9.58	4.57	putative DNA polymerase IV [Arthrobacter aurescens TC1]
gi307746118	4.66	1	1	279	30.6	7.50	4.57	putative GNAT-family acetyltransferase [Arthrobacter arilaitensis Re117]
gi930827562	2.96	2	1	405	43.9	6.83	4.57	lactate dehydrogenase [Arthrobacter arilaitensis]
gi927296107	2.97	3	1	370	40.1	6.47	4.57	GntR family transcriptional regulator [Arthrobacter sp. ERGS1:01]
gi737781476	1.21	2	1	663	74.0	6.32	4.57	glycosyl transferase [Arthrobacter sp. 35W]
gi753933108	1.40	1	1	927	99.0	5.29	4.56	oxidoreductase [Arthrobacter arilaitensis]
gi517592288	1.24	1	1	804	89.0	5.05	4.56	hypothetical protein [Arthrobacter sp. 135MFCol5.1]
gi823666923	9.52	2	1	252	26.6	5.33	4.56	3-hydroxybutyrate dehydrogenase [Arthrobacter sp. YC-RL1]
gi636845549	6.47	1	1	201	22.3	5.85	4.56	3-methyladenine DNA glycosylase [Arthrobacter sp. TB 26]
gi916869891	2.18	2	1	824	90.0	6.20	4.56	hypothetical protein [Arthrobacter sp. Br18]
gi937259324	2.46	2	1	407	43.4	9.35	4.56	PucR family transcriptional regulator [Arthrobacter sp. Edens01]
gi517601234	8.73	1	1	126	13.9	9.32	4.56	hypothetical protein [Arthrobacter sp. 162MFSha1.1]
gi359306186	1.24	2	1	1051	110.0	4.97	4.56	putative glycosidase [Arthrobacter globiformis NBRC 12137]
gi916871400	7.46	1	1	201	21.7	9.58	4.56	Holliday junction resolvase [Arthrobacter sp. H5]
gi119949645	4.09	1	1	538	56.6	5.63	4.56	putative cell envelope-related transcriptional attenuator domain protein [Arthrobacter aures
gi765010967	4.05	1	1	395	41.5	9.50	4.56	sugar ABC transporter permease [Arthrobacter sp. A3]
gi517606082	3.96	1	1	227	23.9	7.52	4.55	phosphoribosyl transferase [Arthrobacter sp. 161MFSha2.1]
gi551254980	9.76	1	1	205	21.1	5.33	4.55	Holliday junction DNA helicase RuvA [Arthrobacter sp. PAO19]
gi470217162	1.94	1	1	412	43.2	5.55	4.55	beta-ketoadipyl CoA thiolase [Arthrobacter gangotriensis Lz1y]
gi916871372	2.71	1	1	442	46.7	9.00	4.55	hypothetical protein [Arthrobacter sp. H5]
gi116654	3.04	5	1	395	43.4	5.36	4.55	RecName: Full=Major capsid protein P3; AltName: Full=Protein P3
gi928488237	9.22	1	1	206	21.7	10.32	4.55	hypothetical protein AOC05_16690 [Arthrobacter alpinus]
gi930825357	9.18	1	1	98	11.2	9.98	4.55	hypothetical protein AOZ07_02955 [Arthrobacter arilaitensis]
gi740680285	25.00	3	2	168	18.6	11.31	4.55	hypothetical protein [Arthrobacter sp. PAMC25486]
gi742758455	2.77	1	1	686	74.7	5.43	4.55	cell division protein FtsH [Arthrobacter phenanthrenivorans]
gi742854018	6.75	2	1	252	27.7	5.16	4.55	ABC transporter ATP-binding protein [Arthrobacter sp. W1]
gi162953597	8.94	4	1	235	26.0	5.26	4.55	iron dependent repressor [Renibacterium salmoninarum ATCC 33209]
gi674644955	11.54	3	1	156	16.9	5.54	4.54	Glycogen accumulation regulator GarA [Arthrobacter sp. 11W110_air]
gi765006390	4.89	1	1	573	61.4	6.49	4.54	ABC transporter ATP-binding protein [Arthrobacter sp. A3]
gi765009845	5.97	1	1	201	21.6	7.59	4.54	hypothetical protein [Arthrobacter sp. A3]
gi917441906	2.27	1	1	528	57.0	5.71	4.54	hypothetical protein [Arthrobacter albus]

gi910697692	3.15	1	1	317	33.0	5.07	4.54	D-3-phosphoglycerate dehydrogenase [Arthrobacter sp. Hiyo6]
gi742854789	1.94	1	1	566	62.0	6.29	4.54	FAD-dependent oxidoreductase [Arthrobacter sp. W1]
gi517602196	7.26	1	1	358	38.7	6.15	4.54	GNAT family N-acetyltransferase [Arthrobacter sp. 131MFCol6.1]
gi517607752	3.83	4	1	678	69.9	5.85	4.54	ABC transporter [Arthrobacter sp. 161MFSha2.1]
gi517605294	8.09	1	1	136	14.6	7.36	4.54	MULTISPECIES: MerR family transcriptional regulator [Arthrobacter]
gi737788351	3.25	1	1	308	32.7	5.14	4.54	dihydropteroate synthase [Arthrobacter albus]
gi116613092	10.55	2	2	199	22.6	6.23	4.54	phthalate 3,4-dioxygenase, beta subunit (plasmid) [Arthrobacter sp. FB24]
gi307745135	2.98	1	1	704	75.6	5.06	4.54	transketolase [Arthrobacter arilaitensis Re117]
gi307745177	2.74	1	1	548	60.1	6.80	4.54	conserved hypothetical protein [Arthrobacter arilaitensis Re117]
gi937257853	4.70	3	1	404	44.1	4.51	4.53	polyamine ABC transporter substrate-binding protein [Arthrobacter sp. Edens01]
gi910696845	2.98	1	1	436	45.4	6.48	4.53	hypothetical protein AHiyo6_08830 [Arthrobacter sp. Hiyo6]
gi918269308	4.73	1	1	296	31.6	5.25	4.53	hypothetical protein AHiyo1_09510 [Arthrobacter sp. Hiyo1]
gi651470914	4.40	1	1	273	29.0	9.10	4.53	D-ala-D-ala transporter subunit [Arthrobacter nicotinovorans]
gi648575039	3.48	1	1	431	45.5	7.23	4.53	sugar-binding protein [Arthrobacter sp. 131MFCol6.1]
gi542110202	5.06	5	1	178	19.1	5.24	4.53	pyridoxamine 5-phosphate oxidase [Arthrobacter sp. AK-YN10]
gi515767225	4.60	1	1	326	34.2	6.67	4.53	nucleoside-diphosphate sugar epimerase [Arthrobacter sp. M2012083]
gi116609706	3.44	1	1	524	56.9	5.43	4.53	extracellular solute-binding protein [Arthrobacter sp. FB24]
gi737812442	3.56	2	1	365	40.6	10.71	4.53	integrase [Arthrobacter sp. H14]
gi908699055	13.04	2	1	92	9.7	9.19	4.53	integration host factor [Arthrobacter sp. RIT-PI-e]
gi517604419	3.74	1	1	214	22.7	4.98	4.53	hypothetical protein [Arthrobacter sp. 131MFCol6.1]
gi636843809	5.06	1	1	237	25.6	5.15	4.53	ABC transporter ATP-binding protein [Arthrobacter sp. TB 26]
gi737812226	17.82	3	1	101	11.0	11.43	4.52	transposase, partial [Arthrobacter sp. H14]
gi823667228	2.50	1	1	839	92.0	5.72	4.52	sarcosine dehydrogenase [Arthrobacter sp. YC-RL1]
gi307746034	8.09	2	1	235	26.2	6.98	4.52	GntR-family transcriptional regulator [Arthrobacter arilaitensis Re117]
gi651429348	4.42	1	1	294	30.6	9.22	4.52	1,4-dihydroxy-2-naphthoate octaprenyltransferase [Arthrobacter sanguinis]
gi648259950	5.26	1	1	399	44.3	9.22	4.52	glycosyl transferase group 1 [Arthrobacter sp. TB 23]
gi651437844	5.50	1	1	309	33.1	6.24	4.52	LysR family transcriptional regulator [Arthrobacter sp. H14]
gi742851595	3.45	1	1	551	59.7	6.00	4.52	ABC transporter [Arthrobacter sp. W1]
gi654825449	5.06	5	1	178	18.9	7.14	4.52	pyridoxamine 5-phosphate oxidase [Arthrobacter sp. I3]
gi651438951	10.58	1	1	208	23.1	9.58	4.52	hypothetical protein [Arthrobacter sp. H14]
gi723607714	18.56	1	1	97	10.5	10.59	4.52	putative transcriptional regulator [Arthrobacter sp. PAMC25486]
gi916871566	2.38	1	1	336	35.5	9.36	4.52	glucokinase [Arthrobacter sp. H5]
gi927032972	4.50	1	1	467	50.7	5.94	4.52	PucR family transcriptional regulator [Arthrobacter sp. LS16]
gi908698697	4.75	1	1	295	31.9	4.75	4.51	methyltransferase type 12 [Arthrobacter sp. RIT-PI-e]
gi742854907	4.48	4	1	580	62.1	5.78	4.51	preprotein translocase subunit SecD [Arthrobacter sp. W1]
gi219858685	7.81	1	1	192	20.6	9.69	4.51	SOUL heme-binding protein [Arthrobacter chlorophenolicus A6]
gi636847186	5.05	1	1	396	41.2	6.81	4.51	SAM-dependent methyltransferase [Arthrobacter sp. TB 26]
gi219860883	11.64	1	1	189	20.7	8.50	4.51	RNA polymerase, sigma-24 subunit, ECF subfamily [Arthrobacter chlorophenolicus A6]
gi910834043	21.99	2	2	141	15.6	9.17	4.51	hypothetical protein ACU18_18830, partial [Arthrobacter sp. ZBG10]
gi759710391	3.35	3	1	328	34.9	5.67	4.51	LacI family transcriptional regulator [Arthrobacter sp. 135MFCol5.1]
gi142204	3.24	4	1	340	37.4	11.28	4.51	erythromycin resistance (ermA) protein [Arthrobacter sp.]
gi307743672	17.65	1	1	102	11.2	10.74	4.51	hypothetical protein AARI_04090 [Arthrobacter arilaitensis Re117]
gi927294399	4.76	2	1	294	32.0	5.01	4.51	ATPase [Arthrobacter sp. ERGS1:01]
gi654815573	1.85	2	1	649	72.6	4.83	4.51	peptidase M13 [Arthrobacter sp. PAO19]
gi219857705	4.50	2	1	200	21.2	4.37	4.51	conserved hypothetical protein [Arthrobacter chlorophenolicus A6]
gi742755322	13.74	1	1	131	14.6	4.89	4.50	flagellar biosynthesis protein [Arthrobacter phenanthrenivorans]
gi652423769	10.64	1	1	188	20.4	5.64	4.50	phosphoribosylglycinamide formyltransferase [Arthrobacter castelli]
gi323468151	1.35	2	1	666	71.1	5.33	4.50	beta-fructosidase, levanase/invertase [Arthrobacter phenanthrenivorans Sphe3]
gi749401198	5.41	3	1	259	28.7	7.03	4.50	glycerophosphodiester phosphodiesterase [Arthrobacter sp. AK-YN10]
gi517608622	2.71	1	1	479	51.2	8.70	4.50	MFS transporter [Arthrobacter sp. 161MFSha2.1]
gi916814486	3.86	1	1	414	45.5	5.03	4.50	hypothetical protein [Arthrobacter nicotinovorans]
gi307743849	3.60	2	1	361	39.9	7.18	4.50	conserved hypothetical protein [Arthrobacter arilaitensis Re117]
gi654822191	2.76	1	1	688	70.4	5.19	4.50	5-oxoprolinase [Arthrobacter sp. I3]
gi919218980	3.43	2	1	554	57.4	9.09	4.50	hypothetical protein [Arthrobacter sp. YC-RL1]
gi651503327	7.17	1	1	251	27.3	6.06	4.50	MerR family transcriptional regulator [Arthrobacter sp. 35W]
gi635350182	4.98	1	1	221	22.9	4.98	4.50	short chain dehydrogenase family protein [Arthrobacter siccitolerans]
gi765005558	2.70	2	1	407	41.3	9.89	4.50	MFS transporter [Arthrobacter sp. A3]
gi917739367	3.67	1	1	327	34.4	9.45	4.50	hypothetical protein [Arthrobacter sp. W1]

gi517606820	5.02	1	1	219	22.6	9.17	4.49	hypothetical protein [Arthrobacter sp. 161MFSha2.1]
gi517604830	3.93	1	1	433	46.6	6.39	4.49	hypothetical protein [Arthrobacter sp. 131MFCol6.1]
gi786033744	4.31	2	1	209	24.1	8.73	4.49	hypothetical protein [Arthrobacter chlorophenolicus]
gi916869572	12.42	2	1	153	15.7	9.28	4.49	hypothetical protein [Arthrobacter sp. Br18]
gi116612812	3.77	1	1	318	33.7	4.93	4.49	thioredoxin reductase [Arthrobacter sp. FB24]
gi542106955	5.57	2	1	395	41.0	6.84	4.49	ArsR family transcriptional regulator [Arthrobacter sp. AK-YN10]
gi636844369	2.62	5	1	611	65.1	7.39	4.49	ABC transporter ATP-binding protein [Arthrobacter sp. TB 26]
gi742755348	5.83	1	1	326	35.8	5.99	4.49	hypothetical protein RM50_14280 [Arthrobacter phenanthrenivorans]
gi916820561	5.90	1	1	373	40.9	10.42	4.49	transposase [Arthrobacter sp. H20]
gi403228103	8.45	3	1	213	23.7	6.40	4.49	transcription regulator, GntR-family [Arthrobacter sp. Rue61a]
gi919219103	3.47	3	1	375	39.1	8.41	4.49	S26 family signal peptidase [Arthrobacter sp. YC-RL1]
gi651454897	6.88	4	1	320	34.5	4.91	4.48	peptidase M19 [Arthrobacter nicotinovorans]
gi916359263	3.96	1	1	480	53.1	5.66	4.48	mannitol dehydrogenase [Arthrobacter sp. 135MFCol5.1]
gi742072727	4.03	1	1	372	41.2	6.09	4.48	phenylalanyl-tRNA synthetase subunit alpha [Arthrobacter sp. MWB30]
gi652424883	4.46	1	1	448	48.1	4.65	4.48	membrane protein [Arthrobacter castelli]
gi737787168	3.41	1	1	381	41.6	5.97	4.48	hypothetical protein [Arthrobacter albus]
gi742852944	8.89	1	1	180	19.7	5.96	4.48	hypothetical protein [Arthrobacter sp. W1]
gi654827483	6.10	1	1	295	33.0	9.52	4.48	XRE family transcriptional regulator [Arthrobacter sp. H5]
gi517602462	3.40	2	1	353	37.4	5.24	4.48	sorbitol dehydrogenase [Arthrobacter sp. 131MFCol6.1]
gi910746173	17.72	1	1	79	8.8	5.10	4.47	hypothetical protein AHiyo8_38750 [Arthrobacter sp. Hiyo8]
gi517590615	8.27	2	1	278	26.7	7.83	4.47	hypothetical protein [Arthrobacter sp. 135MFCol5.1]
gi765012467	16.30	3	1	92	10.9	4.82	4.47	hypothetical protein [Arthrobacter sp. A3]
gi759731269	1.78	1	1	787	81.5	6.42	4.47	carbonate dehydratase [Arthrobacter sp. L77]
gi928486621	4.02	2	1	249	26.0	4.74	4.47	succinate dehydrogenase [Arthrobacter alpinus]
gi162953869	3.02	1	1	430	47.3	10.35	4.47	hypothetical membrane protein [Renibacterium salmoninarum ATCC 33209]
gi551254470	2.83	1	1	743	78.6	5.48	4.47	4-hydroxythreonine-4-phosphate dehydrogenase [Arthrobacter sp. PAO19]
gi651441844	6.11	1	1	262	27.7	4.65	4.46	serine/threonine protein phosphatase [Arthrobacter sp. 9MFCol3.1]
gi489895814	1.90	1	1	789	86.7	5.26	4.46	trehalose phosphorylase [Arthrobacter globiformis]
gi910741826	6.37	2	1	251	27.5	5.77	4.46	transcriptional activator AdeR [Arthrobacter sp. Hiyo4]
gi918268088	1.89	1	1	475	52.7	9.92	4.46	conserved hypothetical protein [Arthrobacter sp. Hiyo1]
gi737771586	8.90	2	1	146	16.1	10.32	4.46	membrane protein insertion efficiency factor YidD [Arthrobacter sp. TB 26]
gi403229413	2.43	1	1	452	47.8	5.29	4.46	putative ABC transporter substrate-binding protein [Arthrobacter sp. Rue61a]
gi939050293	2.21	1	1	634	71.7	6.95	4.46	glycosyltransferase [Arthrobacter sp. JCM 19049]
gi742851216	5.69	1	1	246	25.0	4.31	4.46	hypothetical protein [Arthrobacter sp. W1]
gi219862248	8.88	1	1	214	22.7	5.15	4.46	hypothetical protein Achl_4637 (plasmid) [Arthrobacter chlorophenolicus A6]
gi910693425	2.07	1	1	627	69.4	6.01	4.46	protein involved in chromosome condensation [Arthrobacter sp. Hiyo6]
gi323471395	6.79	1	1	265	27.6	4.82	4.45	Zn-dependent alcohol dehydrogenase, class III (plasmid) [Arthrobacter phenanthrenivoran
gi518313839	5.32	1	1	357	37.5	5.91	4.45	hypothetical protein [Arthrobacter sp. TB 23]
gi914717629	4.20	1	1	262	27.3	10.18	4.45	glycosyl transferase [Arthrobacter sp. ZBG10]
gi443480993	4.79	1	1	313	33.6	7.28	4.45	chromosome replication initiation inhibitor protein [Arthrobacter nitrophenolicus]
gi918269037	7.11	1	1	197	21.5	7.61	4.45	hypothetical protein AHiyo1_06300 [Arthrobacter sp. Hiyo1]
gi470216257	2.56	6	1	703	74.8	5.02	4.45	transketolase [Arthrobacter gangotriensis Lz1y]
gi517607511	5.80	1	1	293	32.1	6.54	4.45	oxidoreductase [Arthrobacter sp. 161MFSha2.1]
gi910737672	16.54	2	1	127	12.9	5.06	4.45	putative phosphoenolpyruvate synthase [Arthrobacter sp. Hiyo4]
gi927293146	2.17	1	1	414	43.1	10.46	4.45	hypothetical protein AL755_02880 (plasmid) [Arthrobacter sp. ERGS1:01]
gi18031684	9.55	1	1	220	22.6	5.39	4.45	NADPH-dependent F420 reductase [Pimelobacter simplex]
gi470220508	3.39	1	1	443	46.3	5.50	4.45	dihydrolipoamide dehydrogenase [Arthrobacter gangotriensis Lz1y]
gi910250664	5.77	2	1	156	16.9	4.45	4.45	glyoxalase family protein [Arthrobacter siccitolerans]
gi742751524	5.21	2	1	384	40.3	8.70	4.44	FAD-binding monooxygenase [Arthrobacter phenanthrenivorans]
gi654819129	11.31	1	1	168	18.5	9.79	4.44	hypothetical protein [Arthrobacter sp. UNC362MFTsu5.1]
gi765013095	4.42	1	1	385	38.2	4.86	4.44	glycerate kinase [Arthrobacter sp. A3]
gi910743950	13.17	2	1	167	17.8	9.99	4.44	hypothetical protein AHiyo8_16520 [Arthrobacter sp. Hiyo8]
gi640201018	10.49	2	1	162	18.7	7.05	4.44	hypothetical protein [Arthrobacter sp. 31Y]
gi551255380	3.49	1	1	315	33.6	9.79	4.44	peptide ABC transporter [Arthrobacter sp. PAO19]
gi654814541	2.61	3	1	460	48.6	8.78	4.44	metabolite transporter [Arthrobacter sp. MA-N2]
gi910738325	7.89	2	1	279	29.1	10.77	4.44	uncharacterized 47.3 kDa protein in thcA 5'region [Arthrobacter sp. Hiyo4]
gi640201173	2.19	1	1	821	87.5	6.55	4.44	hypothetical protein [Arthrobacter sp. 31Y]
gi910737646	22.33	1	1	103	10.8	6.65	4.44	acetyltransferase [Arthrobacter sp. Hiyo4]

gi910742220	5.56	1	1	288	31.5	7.12	4.44	pca regulon regulatory protein [Arthrobacter sp. Hiyo4]
gi403228966	14.77	2	1	149	15.6	10.23	4.44	hypothetical protein ARUE_c14720 [Arthrobacter sp. Rue61a]
gi749402148	4.43	2	1	384	41.7	9.33	4.44	DNA polymerase IV [Arthrobacter sp. AK-YN10]
gi219859596	1.23	1	1	650	71.5	5.01	4.44	Endothelin-converting enzyme 1 [Arthrobacter chlorophenolicus A6]
gi937261750	2.53	1	1	474	49.7	5.00	4.44	pyridine nucleotide-disulfide oxidoreductase [Arthrobacter sp. Edens01]
gi359306548	10.34	1	1	145	15.8	5.30	4.43	hypothetical protein ARGLB_037_01050 [Arthrobacter globiformis NBRC 12137]
gi740683261	3.18	1	1	377	41.9	5.41	4.43	oxidoreductase [Arthrobacter sp. PAMC25486]
gi517603219	3.32	1	1	301	32.1	6.73	4.43	NAD-dependent deacetylase [Arthrobacter sp. 131MFCol6.1]
gi759729940	9.54	2	1	262	28.3	5.99	4.43	DNA-binding response regulator [Arthrobacter sp. L77]
gi551253868	3.59	1	1	557	59.3	5.44	4.43	amidohydrolase [Arthrobacter sp. PAO19]
gi116608911	3.13	1	1	448	48.4	5.24	4.43	L-fuconate dehydratase [Arthrobacter sp. FB24]
gi757624772	6.81	3	1	191	21.3	6.68	4.43	hypothetical protein TV39_08810 [Arthrobacter sp. SPG23]
gi651439562	16.19	1	1	105	11.7	9.47	4.43	MarR family transcriptional regulator [Arthrobacter sp. H14]
gi939051248	6.86	4	1	175	19.2	7.46	4.43	dehydratase, partial [Arthrobacter sp. JCM 19049]
gi910693140	7.14	1	1	140	15.4	9.45	4.43	HTH-type transcriptional regulator AlsR, partial [Arthrobacter sp. Hiyo6]
gi910743470	15.00	1	1	100	11.2	5.57	4.42	cell wall synthesis protein Wag31 [Arthrobacter sp. Hiyo8]
gi640197953	5.45	1	1	202	22.5	8.91	4.42	TetR family transcriptional regulator [Arthrobacter sp. 31Y]
gi651464150	3.93	1	1	484	51.3	7.17	4.42	FAD-dependent oxidoreductase [Arthrobacter sp. 35/47]
gi470220363	4.90	1	1	204	21.9	9.42	4.42	TetR family transcriptional regulator [Arthrobacter gangotriensis Lz1y]
gi910740016	8.40	1	1	262	29.8	5.11	4.42	aminopeptidase N [Arthrobacter sp. Hiyo4]
gi517600780	1.37	1	1	873	90.4	8.91	4.42	hypothetical protein [Arthrobacter sp. 162MFSha1.1]
gi916814597	4.62	1	1	411	41.9	6.38	4.42	calcium-binding protein [Arthrobacter nicotinovorans]
gi937258722	4.03	1	1	347	37.7	5.57	4.42	hypothetical protein AO716_12140 [Arthrobacter sp. Edens01]
gi219858810	8.23	3	1	158	17.0	4.68	4.42	conserved hypothetical protein [Arthrobacter chlorophenolicus A6]
gi323471301	3.70	1	1	433	47.0	6.60	4.42	flavin-dependent oxidoreductase, F420-dependent methylene-tetrahydromethanopterin rec
gi403230252	12.26	1	1	155	16.6	6.27	4.42	ferric uptake regulation protein Fur [Arthrobacter sp. Rue61a]
gi651441441	3.16	1	1	443	48.0	4.67	4.42	hypothetical protein [Arthrobacter sp. 9MFCol3.1]
gi786025805	1.74	1	1	689	73.4	5.92	4.42	phosphatase [Arthrobacter chlorophenolicus]
gi916573912	14.02	1	1	164	17.3	8.03	4.42	hypothetical protein [Arthrobacter sp. TB 26]
gi470221054	2.13	1	1	845	92.0	7.05	4.42	ATP-dependent DNA ligase [Arthrobacter gangotriensis Lz1y]
gi651485489	4.60	1	1	326	33.6	6.98	4.41	hypothetical protein [Arthrobacter sp. Br18]
gi742072181	4.98	1	1	261	28.6	5.21	4.41	hypothetical protein ANMWB30_08850 [Arthrobacter sp. MWB30]
gi651464454	1.45	1	1	692	76.0	5.24	4.41	glycogen debranching protein [Arthrobacter sp. 35/47]
gi654813449	14.84	5	1	128	13.7	7.59	4.41	transcriptional regulator [Arthrobacter sp. MA-N2]
gi910748607	11.45	2	1	166	18.1	9.28	4.41	amp-Dependent synthetase and ligase [Arthrobacter sp. Hiyo8]
gi767259181	10.92	3	1	229	25.1	6.80	4.41	GntR family transcriptional regulator [Arthrobacter sp. IHBB 11108]
gi654817769	9.14	1	1	175	19.7	7.65	4.41	histidine phosphatase [Arthrobacter sp. UNC362MFTsu5.1]
gi640194996	0.87	1	1	1374	146.1	7.93	4.41	cell division protein FtsK [Arthrobacter sp. 31Y]
gi759730981	6.87	2	1	233	26.0	9.92	4.41	hypothetical protein [Arthrobacter sp. L77]
gi116609729	5.65	2	1	283	30.7	5.41	4.41	Methyltransferase type 11 [Arthrobacter sp. FB24]
gi323469889	4.53	1	1	265	28.3	5.01	4.41	metal-dependent hydrolase, beta-lactamase superfamily III [Arthrobacter phenanthrenivora
gi823668551	2.64	1	1	492	52.3	5.02	4.41	hypothetical protein AA310_01065 [Arthrobacter sp. YC-RL1]
gi551254435	3.09	2	1	453	46.5	5.95	4.40	gluconate transporter [Arthrobacter sp. PAO19]
gi654826811	2.88	1	1	555	58.7	11.06	4.40	hypothetical protein [Arthrobacter sp. H5]
gi635353039	6.45	3	1	217	24.1	7.68	4.40	putative hydrolase/acyltransferase [Arthrobacter siccitolerans]
gi937259211	1.76	1	1	568	59.2	5.08	4.40	peptidase M38 family protein [Arthrobacter sp. Edens01]
gi654818262	3.60	1	1	445	46.7	5.94	4.40	histidine kinase [Arthrobacter sp. UNC362MFTsu5.1]
gi636844028	1.33	1	1	903	95.1	6.09	4.40	histidine kinase [Arthrobacter sp. TB 26]
gi640195967	3.66	1	1	383	39.7	5.82	4.40	sulfonate ABC transporter substrate-binding protein [Arthrobacter sp. 31Y]
gi674646415	1.65	1	1	547	57.3	5.33	4.40	N-substituted formamide deformylase precursor [Arthrobacter sp. 11W110_air]
gi908697080	4.07	2	1	344	36.5	5.86	4.40	LacI family transcriptional regulator [Arthrobacter sp. RIT-PI-e]
gi307745463	4.51	1	1	399	43.3	6.09	4.40	putative acyl-CoA dehydrogenase [Arthrobacter arilaitensis Re117]
gi917530200	2.27	1	1	353	38.9	9.80	4.40	hypothetical protein [Arthrobacter sp. PAMC25486]
gi823667592	5.93	1	1	270	31.3	6.67	4.39	phosphate:nucleotide phosphotransferase [Arthrobacter sp. YC-RL1]
gi651436926	6.12	1	1	245	26.3	6.81	4.39	peptide ABC transporter ATP-binding protein [Arthrobacter sp. H41]
gi648224145	3.82	1	1	497	53.4	5.47	4.39	pyruvate kinase [Arthrobacter sp. M2012083]
gi937258706	6.63	1	1	332	35.9	7.08	4.39	aspartate carbamoyltransferase [Arthrobacter sp. Edens01]
gi651459290	3.62	1	1	387	42.2	8.02	4.39	DNA polymerase III subunit delta' [Arthrobacter sp. 35/47]

gi636846548	8.57	1	1	175	19.1	8.66	4.39	MarR family transcriptional regulator [Arthrobacter sp. TB 26]
gi757623004	4.42	2	1	407	43.2	5.20	4.39	amidohydrolase [Arthrobacter sp. SPG23]
gi665847878	2.57	1	1	506	54.1	5.36	4.39	bifunctional hydroxymethylpyrimidine kinase/phosphomethylpyrimidine kinase [Arthrobacte
gi910741765	7.97	1	1	138	15.5	9.57	4.39	transposase [Arthrobacter sp. Hiyo4]
gi928488080	7.77	1	1	206	21.4	10.35	4.39	amino acid transporter [Arthrobacter alpinus]
gi651454514	4.92	2	1	264	27.9	10.95	4.39	cobalt transporter [Arthrobacter nicotinovorans]
gi654825575	3.86	1	1	440	47.1	6.21	4.39	histidinol dehydrogenase [Arthrobacter sp. H5]
gi765002314	2.41	1	1	497	54.0	4.86	4.39	glycosyl hydrolase family 32 [Arthrobacter sp. M2012083]
gi918221831	7.96	3	1	226	24.8	6.11	4.39	hypothetical protein [Arthrobacter sp. I3]
gi767257493	2.44	1	1	492	51.8	5.34	4.39	inosine-5-monophosphate dehydrogenase [Arthrobacter sp. IHBB 11108]
gi910740830	6.90	1	1	145	16.1	10.62	4.39	50S ribosomal protein L22 [Arthrobacter sp. Hiyo4]
gi930825270	4.40	1	1	318	34.8	9.07	4.39	transcriptional regulator [Arthrobacter arilaitensis]
gi219861659	5.49	1	1	346	38.6	5.82	4.39	hypothetical protein Achl_4049 (plasmid) [Arthrobacter chlorophenolicus A6]
gi359305996	5.45	1	1	220	24.0	4.88	4.39	ADP-ribose pyrophosphatase [Arthrobacter globiformis NBRC 12137]
gi119951738	5.30	1	1	264	29.2	5.07	4.39	conserved hypothetical protein (plasmid) [Arthrobacter aurescens TC1]
gi359303521	3.19	1	1	251	26.7	9.14	4.38	hypothetical protein ARGLB_114_00170 [Arthrobacter globiformis NBRC 12137]
gi517591992	6.73	1	1	297	31.4	4.73	4.38	5-deoxy-glucuronate isomerase [Arthrobacter sp. 135MFCol5.1]
gi930828103	2.04	1	1	589	63.1	5.49	4.38	ABC transporter [Arthrobacter arilaitensis]
gi551254395	3.07	2	1	618	66.5	7.37	4.38	ABC transporter [Arthrobacter sp. PAO19]
gi915330475	4.01	1	1	274	29.4	9.60	4.38	IclR family transcriptional regulator [Arthrobacter chlorophenolicus]
gi910697544	14.78	1	1	115	12.8	8.43	4.38	elongation factor G [Arthrobacter sp. Hiyo6]
gi914717713	1.66	1	1	841	87.7	5.07	4.38	RND transporter [Arthrobacter sp. ZBG10]
gi910746347	7.44	1	1	121	12.7	5.50	4.38	rhs element Vgr protein [Arthrobacter sp. Hiyo8]
gi323470573	7.66	1	1	222	24.2	8.18	4.38	response regulator with putative antiterminator output domain [Arthrobacter phenanthreni
gi515765710	2.23	1	1	718	79.3	5.41	4.38	amylo-alpha-1,6-glucosidase [Arthrobacter sp. M2012083]
gi823668022	5.26	1	1	399	41.7	4.86	4.38	imidazolonepropionase [Arthrobacter sp. YC-RL1]
gi737804243	2.87	1	1	557	58.4	6.39	4.37	oxidoreductase [Arthrobacter sp. Br18]
gi654816087	3.60	1	1	417	44.8	5.45	4.37	ferredoxin [Arthrobacter sp. UNC362MFTsu5.1]
gi742758583	8.09	1	1	235	24.8	5.76	4.37	GntR family transcriptional regulator [Arthrobacter phenanthrenivorans]
gi937258501	1.55	1	1	1159	124.6	6.11	4.37	hypothetical protein AO716_10700 [Arthrobacter sp. Edens01]
gi765008551	2.18	1	1	504	53.1	5.05	4.37	hypothetical protein [Arthrobacter sp. A3]
gi674645053	3.17	1	1	442	47.7	6.95	4.37	putative RNA methyltransferase/cg2084 [Arthrobacter sp. 11W110_air]
gi823668474	1.73	1	1	578	63.1	7.96	4.37	conjugal transfer protein TraG [Arthrobacter sp. YC-RL1]
gi930827397	3.98	1	1	226	24.6	6.44	4.37	hypothetical protein AOZ07_14670 [Arthrobacter arilaitensis]
gi749402513	7.59	1	1	303	32.7	8.02	4.37	NADH dehydrogenase, partial [Arthrobacter sp. AK-YN10]
gi723608078	3.65	1	1	438	46.4	6.09	4.37	serine hydroxymethyltransferase [Arthrobacter sp. PAMC25486]
gi403227581	2.72	1	1	368	42.3	6.23	4.37	putative RelA / SpoT family protein [Arthrobacter sp. Rue61a]
gi518312434	6.09	3	1	279	29.9	5.02	4.37	hypothetical protein [Arthrobacter sp. TB 23]
gi742854935	7.53	2	1	186	20.4	4.64	4.37	deaminase [Arthrobacter sp. W1]
gi939050333	9.57	1	1	94	10.2	8.59	4.37	50S ribosomal protein L25 [Arthrobacter sp. JCM 19049]
gi927295363	8.33	1	1	108	11.5	4.94	4.37	hypothetical protein AL755_17360 [Arthrobacter sp. ERGS1:01]
gi910697367	4.98	1	1	201	22.9	6.65	4.37	biphenyl dioxygenase subunit beta [Arthrobacter sp. Hiyo6]
gi515765292	7.97	2	1	276	30.1	7.58	4.37	hypothetical protein [Arthrobacter sp. M2012083]
gi757623578	2.74	1	1	475	48.8	8.05	4.36	histidine kinase [Arthrobacter sp. SPG23]
gi651455161	6.52	1	1	184	19.1	5.03	4.36	hypothetical protein [Arthrobacter nicotinovorans]
gi939051583	4.78	1	1	230	24.2	8.97	4.36	hypothetical protein [Arthrobacter sp. JCM 19049]
gi403230546	8.99	1	1	178	19.1	9.82	4.36	50S ribosomal protein L6 [Arthrobacter sp. Rue61a]
gi654824156	5.29	1	1	359	38.9	5.80	4.36	aminoglycoside resistance protein [Arthrobacter sp. I3]
gi767258996	7.72	1	1	246	26.9	5.94	4.36	dolichol-phosphate mannosyltransferase [Arthrobacter sp. IHBB 11108]
gi910742037	28.21	1	1	39	4.3	9.99	4.36	hypothetical protein AHiyo4_48590 [Arthrobacter sp. Hiyo4]
gi636843278	5.23	3	1	325	33.3	6.24	4.36	thiamine biosynthesis protein [Arthrobacter sp. TB 26]
gi517605533	23.33	1	1	90	9.1	11.52	4.36	hypothetical protein [Arthrobacter sp. 131MFCol6.1]
gi116609403	12.96	3	1	108	11.5	7.08	4.36	hypothetical protein Arth_0729 [Arthrobacter sp. FB24]
gi517589916	12.50	1	1	160	16.6	9.99	4.36	ATP synthase [Arthrobacter sp. 135MFCol5.1]
gi551254987	7.53	2	1	186	20.5	4.44	4.36	deaminase [Arthrobacter sp. PAO19]
gi489900701	3.24	2	1	463	51.2	5.14	4.36	two-component system sensor histidine kinase [Arthrobacter globiformis]
gi937256378	7.18	1	1	195	22.6	6.55	4.36	hypothetical protein AO716_14975 [Arthrobacter sp. Edens01]
gi119951865	7.66	6	1	248	26.6	7.40	4.36	transcriptional regulator, IclR family (plasmid) [Arthrobacter aurescens TC1]

gi635353454	3.07	1	1	651	70.6	5.88	4.36	isoniazid-inductible protein iniA [Arthrobacter siccitolerans]
gi914715060	7.06	2	1	269	27.0	5.26	4.36	molybdate-binding protein [Arthrobacter sp. ZBG10]
gi739798514	4.19	1	1	310	33.4	5.33	4.36	prephenate dehydratase [Streptomyces griseofuscus]
gi910692929	4.05	1	1	222	23.3	5.78	4.35	alcohol dehydrogenase, partial [Arthrobacter sp. Hiyo6]
gi551286208	4.21	1	1	309	32.5	5.49	4.35	prephenate dehydratase, partial [Curtobacterium sp. B18]
gi651503054	3.28	2	1	335	35.5	5.48	4.35	dehydrogenase [Arthrobacter sp. 35W]
gi542110312	9.80	2	1	245	25.8	6.80	4.35	ArsR family transcriptional regulator [Arthrobacter sp. AK-YN10]
gi636843493	3.81	2	1	289	30.9	8.76	4.35	DNA lyase [Arthrobacter sp. TB 26]
gi930827885	6.53	2	1	337	36.4	5.02	4.35	inositol 2-dehydrogenase [Arthrobacter arilaitensis]
gi654816353	2.82	1	1	461	48.9	5.10	4.35	cystathionine beta-synthase [Arthrobacter sp. UNC362MFTsu5.1]
gi517592145	4.30	1	1	349	38.6	9.50	4.34	hypothetical protein [Arthrobacter sp. 135MFCol5.1]
gi723607507	5.28	1	1	417	44.8	4.97	4.34	UDP-N-acetyl-D-mannosamine dehydrogenase [Arthrobacter sp. PAMC25486]
gi917013443	2.38	1	1	715	75.0	5.27	4.34	potassium transporter KtrB [Arthrobacter sanguinis]
gi919108052	4.23	1	1	331	36.5	8.57	4.34	hypothetical protein [Arthrobacter sp. IHBB 11108]
gi307745966	4.28	1	1	257	28.1	5.16	4.34	IclR-family transcriptional regulator [Arthrobacter arilaitensis Re117]
gi910740448	4.15	2	1	241	26.0	11.12	4.34	hypothetical protein AHiyo4_32700 [Arthrobacter sp. Hiyo4]
gi765013077	3.68	2	1	462	49.3	5.69	4.34	ATPase AAA [Arthrobacter sp. A3]
gi635352855	3.76	1	1	558	58.4	6.71	4.34	formate--tetrahydrofolate ligase [Arthrobacter siccitolerans]
gi910744936	13.39	1	1	112	11.6	5.03	4.34	hypothetical protein AHiyo8_26380 [Arthrobacter sp. Hiyo8]
gi910252520	1.52	1	1	656	69.8	5.99	4.34	hypothetical protein [Arthrobacter siccitolerans]
gi914717147	3.05	2	1	393	42.3	7.65	4.34	histidine kinase [Arthrobacter sp. ZBG10]
gi908697446	3.82	1	1	340	36.5	5.47	4.34	geranylgeranyl pyrophosphate synthase [Arthrobacter sp. RIT-PI-e]
gi651458546	1.60	1	1	564	60.0	4.54	4.34	hypothetical protein [Arthrobacter sp. 35/47]
gi723608532	3.72	1	1	538	55.8	7.77	4.34	glycolate oxidase subunit [Arthrobacter sp. PAMC25486]
gi323469336	9.18	1	1	196	20.7	6.54	4.34	transcriptional regulator [Arthrobacter phenanthrenivorans Sphe3]
gi470221523	8.79	1	1	239	25.8	7.15	4.34	IclR family transcriptional regulator [Arthrobacter gangotriensis Lz1y]
gi749401240	3.48	5	1	517	58.9	5.91	4.34	hypothetical protein M707_26470, partial [Arthrobacter sp. AK-YN10]
gi654815437	3.41	1	1	411	44.5	5.01	4.34	quinolinate synthetase [Arthrobacter sp. PAO19]
gi403230704	17.19	1	1	128	14.6	4.77	4.34	hypothetical protein ARUE_c32450 [Arthrobacter sp. Rue61a]
gi916816466	4.64	1	1	366	40.2	5.59	4.34	hypothetical protein [Arthrobacter sp. MA-N2]
gi651498742	2.73	2	1	439	45.1	7.50	4.34	MFS transporter [Arthrobacter sp. 35W]
gi651481529	2.52	7	1	476	51.8	5.77	4.34	adenylosuccinate lyase [Arthrobacter sp. Br18]
gi742758956	2.59	1	1	540	55.1	9.76	4.34	hypothetical protein RM50_01435 [Arthrobacter phenanthrenivorans]
gi910747980	5.54	1	1	289	30.1	9.32	4.33	hypothetical protein AHiyo8_56820 [Arthrobacter sp. Hiyo8]
gi919218786	3.41	1	1	411	42.8	10.13	4.33	MFS transporter [Arthrobacter sp. YC-RL1]
gi910249258	15.87	1	1	63	7.0	7.25	4.33	hypothetical protein [Arthrobacter siccitolerans]
gi476402684	8.73	1	1	229	23.8	4.63	4.33	hypothetical protein D477_002441 [Arthrobacter crystallopoietes BAB-32]
gi476402543	2.80	1	1	429	45.8	5.20	4.33	nucleotide sugar dehydrogenase [Arthrobacter crystallopoietes BAB-32]
gi470220090	2.71	1	1	554	59.9	5.49	4.33	ABC transporter [Arthrobacter gangotriensis Lz1y]
gi757624994	3.67	1	1	409	42.0	5.25	4.33	molybdenum cofactor biosynthesis protein MoaA [Arthrobacter sp. SPG23]
gi636843467	3.21	1	1	405	42.4	5.34	4.33	cystathionine gamma-synthase [Arthrobacter sp. TB 26]
gi928487753	3.44	1	1	465	49.8	5.40	4.33	sulfate adenylyltransferase [Arthrobacter alpinus]
gi916820283	5.65	1	1	301	31.5	4.77	4.33	flagellar biosynthesis protein flip [Arthrobacter sp. H20]
gi916871637	8.97	1	1	156	16.4	4.92	4.33	hypothetical protein [Arthrobacter sp. H5]
gi652424850	1.81	1	1	608	63.1	6.24	4.33	hypothetical protein [Arthrobacter castelli]
gi651429472	3.58	1	1	447	49.6	4.79	4.33	hypothetical protein [Arthrobacter sanguinis]
gi767258212	0.72	1	1	1531	160.3	5.44	4.33	5'-nucleotidase [Arthrobacter sp. IHBB 11108]
gi359306550	8.00	1	1	250	26.3	4.93	4.33	oxidoreductase [Arthrobacter globiformis NBRC 12137]
gi914714694	3.65	1	1	274	29.4	9.04	4.33	short-chain dehydrogenase [Arthrobacter sp. ZBG10]
gi651444719	5.45	10	1	440	48.2	9.13	4.33	pyridine nucleotide-disulfide oxidoreductase [Arthrobacter nicotinovorans]
gi723609851	3.22	1	1	373	39.6	6.25	4.32	hypothetical protein ART_3628 [Arthrobacter sp. PAMC25486]
gi908691055	4.49	1	1	245	25.6	6.06	4.32	beta-ketoacyl-ACP reductase [Arthrobacter sp. H41]
gi511534834	12.78	1	1	133	15.0	11.88	4.32	hypothetical protein (plasmid) [Arthrobacter nicotinovorans]
gi916863548	8.30	1	1	241	25.6	8.88	4.32	hypothetical protein [Arthrobacter sp. 35/47]
gi515767277	11.21	1	1	107	11.6	4.60	4.32	hypothetical protein [Arthrobacter sp. M2012083]
gi470217534	3.63	1	1	358	38.7	5.83	4.32	hypothetical protein ADIAG_02368 [Arthrobacter gangotriensis Lz1y]
gi916869743	10.14	1	1	148	15.5	10.32	4.32	hypothetical protein [Arthrobacter sp. Br18]
gi910696370	19.13	2	1	115	12.4	5.25	4.32	uncharacterized HTH-type transcriptional regulator YagI [Arthrobacter sp. Hiyo6]

gi767258125	2.02	1	1	446	47.1	9.52	4.32	MFS transporter [Arthrobacter sp. IHBB 11108]
gi443481494	7.61	1	1	289	28.5	4.39	4.32	ribokinase [Arthrobacter nitrophenolicus]
gi651497998	2.98	1	1	571	60.0	6.54	4.32	hypothetical protein [Arthrobacter sp. 35W]
gi651460447	4.00	1	1	300	33.6	4.63	4.32	allophanate hydrolase [Arthrobacter sp. 35/47]
gi914716493	8.98	3	1	245	25.7	5.96	4.32	hypothetical protein [Arthrobacter sp. ZBG10]
gi723609612	3.92	2	1	357	39.5	5.97	4.32	hypothetical protein ART_3389 [Arthrobacter sp. PAMC25486]
gi937259173	7.87	1	1	254	25.9	5.22	4.32	hypothetical protein AO716_14675 [Arthrobacter sp. Edens01]
gi759773096	1.55	4	1	1358	141.6	7.39	4.32	hypothetical protein [Arthrobacter sp. SPG23]
gi651490184	7.87	1	1	254	26.4	5.30	4.31	short-chain dehydrogenase [Arthrobacter sp. H20]
gi403229297	5.39	1	1	241	24.3	6.20	4.31	short-chain dehydrogenase/reductase family protein [Arthrobacter sp. Rue61a]
gi908699046	2.76	1	1	688	73.2	6.11	4.31	hypothetical protein [Arthrobacter sp. RIT-PI-e]
gi767259174	3.05	1	1	328	35.0	5.07	4.31	2-oxoisovalerate dehydrogenase [Arthrobacter sp. IHBB 11108]
gi674644943	4.21	1	1	214	23.1	5.36	4.31	Trans-aconitate 2-methyltransferase [Arthrobacter sp. 11W110_air]
gi640196820	3.15	1	1	349	37.1	5.19	4.31	hypothetical protein [Arthrobacter sp. 31Y]
gi914714253	5.01	1	1	399	40.6	5.66	4.31	acetyl-CoA acetyltransferase [Arthrobacter sp. ZBG10]
gi651491103	12.07	1	1	116	13.1	8.59	4.31	ArsR family transcriptional regulator [Arthrobacter sp. H20]
gi916871733	7.06	1	1	170	16.9	4.77	4.31	damage-inducible protein CinA [Arthrobacter sp. H5]
gi517602659	4.37	2	1	252	27.3	6.11	4.31	maleylpyruvate isomerase [Arthrobacter sp. 131MFCol6.1]
gi652424793	1.26	1	1	636	71.9	5.30	4.31	hypothetical protein [Arthrobacter castelli]
gi654819629	2.32	1	1	561	59.5	5.35	4.31	protein kinase [Arthrobacter sp. UNC362MFTsu5.1]
gi639129957	15.89	1	1	107	12.5	9.00	4.31	hypothetical protein [Arthrobacter sp. CAL618]
gi910250475	1.85	3	1	809	91.3	6.10	4.31	phosphoketolase [Arthrobacter siccitolerans]
gi651499202	0.85	1	1	1176	123.7	5.80	4.30	peptidase S41 [Arthrobacter sp. 35W]
gi521040836	6.31	1	1	333	34.8	5.06	4.30	thioredoxin reductase [Arthrobacter sp. M2012083]
gi359303819	2.85	1	1	316	34.5	9.73	4.30	hypothetical protein ARGLB_096_00120 [Arthrobacter globiformis NBRC 12137]
gi823668552	13.51	1	1	74	8.5	5.22	4.30	hypothetical protein AA310_01070 [Arthrobacter sp. YC-RL1]
gi939051634	5.47	1	1	274	30.7	6.29	4.30	WYL domain-containing protein [Arthrobacter sp. JCM 19049]
gi651465631	15.63	1	1	64	6.9	4.42	4.30	hypothetical protein [Arthrobacter sp. 35/47]
gi518312608	10.05	1	1	199	21.5	6.55	4.30	MULTISPECIES: uracil phosphoribosyltransferase [Arthrobacter]
gi651489592	8.97	1	1	223	24.3	5.24	4.30	hypothetical protein [Arthrobacter sp. H20]
gi910251705	3.40	1	1	382	40.2	5.29	4.30	glycine cleavage system protein T [Arthrobacter siccitolerans]
gi737777950	2.17	1	1	460	52.4	6.60	4.30	hypothetical protein [Arthrobacter sanguinis]
gi937258729	6.34	1	1	205	21.7	10.80	4.30	hypothetical protein AO716_12180 [Arthrobacter sp. Edens01]
gi403231992	2.93	1	1	546	59.7	7.01	4.30	protein containing ATP/GTP-binding site motif A (plasmid) [Arthrobacter sp. Rue61a]
gi759746402	1.46	1	1	1507	156.0	4.96	4.30	5'-nucleotidase [Arthrobacter sp. 31Y]
gi937259584	6.15	1	1	260	28.2	7.52	4.30	hypothetical protein AO716_06295 [Arthrobacter sp. Edens01]
gi765012204	10.00	1	1	180	19.0	5.59	4.29	PadR family transcriptional regulator [Arthrobacter sp. A3]
gi759725488	6.40	2	1	344	35.4	9.88	4.29	ATPase [Arthrobacter sp. I3]
gi652422867	3.53	2	1	283	30.6	5.07	4.29	tRNA (guanine-N1)-methyltransferase [Arthrobacter castelli]
gi823666720	11.39	1	1	202	21.8	10.18	4.29	hypothetical protein AA310_13290 [Arthrobacter sp. YC-RL1]
gi919108158	1.83	1	1	547	59.0	7.43	4.29	hypothetical protein [Arthrobacter sp. IHBB 11108]
gi759765902	4.33	1	1	277	30.5	8.62	4.29	DNA glycosylase [Arthrobacter gangotriensis]
gi651442696	4.00	1	1	375	38.4	5.27	4.29	sodium:proton antiporter [Arthrobacter sp. 9MFCol3.1]
gi914714152	4.64	1	1	345	36.6	5.91	4.28	hypothetical protein [Arthrobacter sp. ZBG10]
gi651438942	4.60	4	1	413	44.1	5.00	4.28	2-amino-3-ketobutyrate CoA ligase [Arthrobacter sp. H14]
gi651438948	5.26	1	1	171	19.0	5.00	4.28	GCN5 family acetyltransferase [Arthrobacter sp. H14]
gi651504373	8.05	1	1	149	15.9	6.19	4.28	hypothetical protein [Arthrobacter sp. 35W]
gi651430501	1.64	1	1	669	75.4	6.68	4.28	glycosyl transferase [Arthrobacter sanguinis]
gi517601734	4.70	1	1	234	24.6	6.95	4.28	hypothetical protein [Arthrobacter sp. 162MFSha1.1]
gi119947569	4.64	1	1	560	59.0	8.88	4.27	conserved hypothetical protein [Arthrobacter aurescens TC1]
gi323468208	4.49	1	1	468	51.9	5.94	4.27	glucuronate isomerase [Arthrobacter phenanthrenivorans Sphe3]
gi757624600	4.24	1	1	543	59.3	5.07	4.27	hypothetical protein TV39_07765 [Arthrobacter sp. SPG23]
gi757626376	14.48	1	1	145	15.9	9.52	4.27	ATPase [Arthrobacter sp. SPG23]
gi674643986	11.01	1	1	109	11.8	5.50	4.27	hypothetical protein BN1051_00128 [Arthrobacter sp. 11W110_air]
gi651430180	4.73	1	1	296	32.2	5.00	4.27	6-phosphogluconate dehydrogenase [Arthrobacter sanguinis]
gi910697428	2.95	2	1	407	45.5	9.72	4.27	integrase [Arthrobacter sp. Hiyo6]
gi759702733	16.95	1	1	59	6.1	9.99	4.26	hypothetical protein [Arthrobacter globiformis]
gi786033428	2.51	2	1	439	48.6	5.53	4.26	alpha/beta hydrolase [Arthrobacter chlorophenolicus]

gi737814723	4.85	1	1	206	22.1	8.03	4.26	cysteine methyltransferase [Arthrobacter sp. H14]
gi648575757	4.16	1	1	409	44.1	6.16	4.26	hypothetical protein [Arthrobacter sp. 161MFSha2.1]
gi917745888	3.42	1	1	292	31.6	5.17	4.26	amidohydrolase [Arthrobacter phenanthrenivorans]
gi489895868	4.85	1	1	309	34.5	6.64	4.26	PaaX family transcriptional regulator [Arthrobacter globiformis]
gi651438780	4.09	1	1	464	49.3	8.82	4.26	MFS transporter [Arthrobacter sp. H14]
gi759723216	4.05	1	1	346	36.7	5.82	4.26	hypothetical protein [Arthrobacter sp. I3]
gi930827072	3.93	2	1	280	28.5	4.86	4.26	pyrroline-5-carboxylate reductase [Arthrobacter arilaitensis]
gi651500583	2.52	1	1	437	44.8	6.21	4.26	hypothetical protein [Arthrobacter sp. 35W]
gi359307186	3.25	1	1	461	48.5	7.75	4.26	UDP-glucose 6-dehydrogenase [Arthrobacter globiformis NBRC 12137]
gi515767419	3.79	1	1	634	67.0	5.78	4.26	glutamine amidotransferase [Arthrobacter sp. M2012083]
gi737800842	10.30	1	1	165	18.4	7.56	4.26	transcriptional regulator [Arthrobacter castelli]
gi542110540	5.70	1	1	386	42.1	5.35	4.26	acyl-CoA dehydrogenase [Arthrobacter sp. AK-YN10]
gi651507355	8.57	1	1	210	22.9	5.01	4.26	TetR family transcriptional regulator [Arthrobacter sp. 35W]
gi403231086	4.41	1	1	340	36.8	6.28	4.26	hypothetical protein ARUE_c36290 [Arthrobacter sp. Rue61a]
gi765005490	5.94	2	1	286	28.8	11.53	4.25	hypothetical protein [Arthrobacter sp. A3]
gi636843828	5.26	1	1	304	31.8	6.64	4.25	hypothetical protein [Arthrobacter sp. TB 26]
gi910748822	10.34	1	1	116	12.5	10.08	4.25	integration host factor [Arthrobacter sp. Hiyo8]
gi910740290	4.81	1	1	291	31.5	10.10	4.25	putative threonylcarbamoyl-AMP synthase [Arthrobacter sp. Hiyo4]
gi116608848	15.09	1	1	106	12.2	9.52	4.25	hypothetical protein Arth_0171 [Arthrobacter sp. FB24]
gi219858833	2.52	1	1	555	58.4	6.65	4.25	transcriptional regulator, CdaR [Arthrobacter chlorophenolicus A6]
gi654826525	6.48	1	1	247	26.2	6.68	4.25	3-oxoacyl-ACP reductase [Arthrobacter sp. H5]
gi737778879	9.55	1	1	178	19.3	4.70	4.25	MULTISPECIES: hypothetical protein [Arthrobacter]
gi654818175	4.43	1	1	406	44.2	6.76	4.25	PucR family transcriptional regulator [Arthrobacter sp. UNC362MFTsu5.1]
gi470217648	3.64	1	1	439	46.8	5.99	4.25	serine hydroxymethyltransferase [Arthrobacter gangotriensis Lz1y]
gi116612852	19.54	1	1	87	9.8	10.13	4.25	hypothetical protein Arth_4403 (plasmid) [Arthrobacter sp. FB24]
gi476403118	11.70	1	1	171	18.4	5.08	4.25	mycothiol-dependent formaldehyde dehydrogenase, partial [Arthrobacter crystallopoietes B
gi742071439	4.82	3	1	353	35.3	5.35	4.24	hypothetical protein ANMWB30_15580 [Arthrobacter sp. MWB30]
gi910741949	7.20	1	1	236	24.7	6.35	4.24	N-acetylglucosamine-6-phosphate deacetylase [Arthrobacter sp. Hiyo4]
gi786033053	4.51	1	1	421	45.9	6.37	4.24	XRE family transcriptional regulator [Arthrobacter chlorophenolicus]
gi910696012	7.82	1	1	179	18.5	5.54	4.24	uncharacterized zinc-type alcohol dehydrogenase-like protein YycR [Arthrobacter sp. Hiyo6
gi651483164	4.03	3	1	397	42.8	5.11	4.24	molybdopterin biosynthesis protein MoeZ [Arthrobacter sp. Br18]
gi818631394	14.08	1	1	142	15.7	4.67	4.24	hypothetical protein (plasmid) [Arthrobacter sp. 68b]
gi939051313	9.79	1	1	143	16.3	4.70	4.24	hypothetical protein [Arthrobacter sp. JCM 19049]
gi651507893	6.02	1	1	166	18.5	8.21	4.24	hypothetical protein [Arthrobacter sp. 35W]
gi910251092	4.70	1	1	298	32.5	6.33	4.24	tRNA dimethylallyltransferase [Arthrobacter siccitolerans]
gi742859249	8.04	2	1	224	22.7	7.34	4.24	hypothetical protein [Arthrobacter sp. W1]
gi908697961	1.62	1	1	986	108.6	5.21	4.24	beta-galactosidase [Arthrobacter sp. RIT-PI-e]
gi323467568	4.77	1	1	461	50.2	5.35	4.24	monoamine oxidase [Arthrobacter phenanthrenivorans Sphe3]
gi651429730	6.59	1	1	273	30.5	5.34	4.24	exodeoxyribonuclease III [Arthrobacter sanguinis]
gi219858464	2.36	1	1	678	69.7	5.50	4.24	YhgE/Pip C-terminal domain protein [Arthrobacter chlorophenolicus A6]
gi219859202	2.27	1	1	440	44.4	9.67	4.24	Lytic transglycosylase catalytic [Arthrobacter chlorophenolicus A6]
gi749401232	1.70	1	1	648	69.5	6.38	4.23	AMP-dependent synthetase, partial [Arthrobacter sp. AK-YN10]
gi476402345	9.91	1	1	222	22.0	6.34	4.23	short-chain dehydrogenase/reductase SDR [Arthrobacter crystallopoietes BAB-32]
gi674644960	1.85	1	1	1134	121.7	5.10	4.23	Biotin carboxylase [Arthrobacter sp. 11W110_air]
gi765012622	11.11	1	1	126	13.4	5.25	4.23	transcriptional regulator [Arthrobacter sp. A3]
gi930827536	2.82	1	1	390	42.8	6.05	4.23	DNA-binding protein [Arthrobacter arilaitensis]
gi930826074	9.21	1	1	152	17.2	9.42	4.23	transcriptional regulator [Arthrobacter arilaitensis]
gi640202478	3.13	1	1	447	47.9	4.96	4.23	zinc protease [Arthrobacter sp. 31Y]
gi767257835	6.80	3	1	309	32.8	8.85	4.23	50S ribosomal protein L4 [Arthrobacter sp. IHBB 11108]
gi760166893	3.55	1	1	282	30.3	4.51	4.23	iron transporter [Arthrobacter crystallopoietes]
gi307744356	6.16	1	1	211	22.7	5.74	4.23	imidazole glycerol phosphate synthase, glutamine amidotransferase subunit [Arthrobacter a
gi914713744	6.00	1	1	417	43.9	11.30	4.23	MFS transporter [Arthrobacter sp. ZBG10]
gi359306817	2.76	2	1	471	48.9	6.43	4.22	adenosylmethionine-8-amino-7-oxononanoate aminotransferase [Arthrobacter globiformis N
gi823668528	3.37	1	1	475	52.2	11.00	4.22	hypothetical protein AA310_00920 [Arthrobacter sp. YC-RL1]
gi470219967	7.27	1	1	344	35.7	5.66	4.22	Malate dehydrogenase [Arthrobacter gangotriensis Lz1y]
gi757623371	1.01	1	1	1183	127.3	5.31	4.22	DNA helicase UvrD [Arthrobacter sp. SPG23]
gi910283552	5.54	1	1	343	36.8	5.74	4.22	hypothetical protein [Arthrobacter sp. A3]
gi116612383	5.45	1	1	165	18.0	4.89	4.22	conserved hypothetical protein [Arthrobacter sp. FB24]

gi551255262	2.92	2	1	274	30.3	8.37	4.22	DNA glycosylase [Arthrobacter sp. PAO19]
gi753938929	5.75	2	1	313	34.4	7.01	4.22	hypothetical protein [Arthrobacter phenanthrenivorans]
gi723607053	4.72	1	1	254	28.8	5.68	4.22	hypothetical protein ART_0830 [Arthrobacter sp. PAMC25486]
gi742855066	1.89	1	1	846	87.9	4.28	4.22	alkaline phosphatase [Arthrobacter sp. W1]
gi654811402	5.64	3	1	337	35.7	5.80	4.21	exonuclease [Arthrobacter sp. MA-N2]
gi651507327	4.17	1	1	360	37.8	5.95	4.21	alkene reductase [Arthrobacter sp. 35W]
gi542109078	5.69	1	1	211	22.1	8.40	4.21	hypothetical protein M707_08210 [Arthrobacter sp. AK-YN10]
gi307746285	3.71	1	1	350	39.6	5.44	4.21	hypothetical protein AARI_30540 [Arthrobacter arilaitensis Re117]
gi651499384	3.59	1	1	529	55.3	7.94	4.21	methyltransferase [Arthrobacter sp. 35W]
gi651437558	9.52	1	1	84	8.8	5.01	4.21	MULTISPECIES: hypothetical protein [Arthrobacter]
gi742071844	1.91	3	1	889	96.6	6.15	4.21	LuxR family transcriptional regulator [Arthrobacter sp. MWB30]
gi639131136	5.23	1	1	344	37.3	10.39	4.21	transposase [Arthrobacter sp. CAL618]
gi654819614	3.88	2	1	490	51.7	6.48	4.21	oxidoreductase [Arthrobacter sp. UNC362MFTsu5.1]
gi651492560	4.38	2	1	434	48.0	5.48	4.21	glycine cleavage system protein T [Arthrobacter sp. H20]
gi917442237	2.00	1	1	701	74.9	4.36	4.20	hypothetical protein [Arthrobacter albus]
gi517593335	4.15	1	1	410	44.3	9.35	4.20	DNA polymerase IV [Arthrobacter sp. 135MFCol5.1]
gi640196200	4.03	3	1	347	37.4	8.21	4.20	hypothetical protein [Arthrobacter sp. 31Y]
gi757623958	2.72	1	1	257	27.4	6.15	4.20	cobalt ABC transporter ATP-binding protein [Arthrobacter sp. SPG23]
gi323469266	3.40	1	1	588	62.0	6.09	4.20	ABC-type multidrug transport system, ATPase and permease component [Arthrobacter phe
gi162952480	3.29	3	1	486	51.4	5.06	4.20	predicted O-Glycosyl hydrolase, family 3 [Renibacterium salmoninarum ATCC 33209]
gi765005366	2.46	1	1	447	48.1	4.83	4.20	quinolinate synthetase [Arthrobacter sp. A3]
gi651489303	4.56	1	1	417	43.4	5.87	4.20	threonine dehydratase [Arthrobacter sp. H20]
gi654822422	3.01	1	1	332	35.8	7.81	4.20	glycosyltransferase [Arthrobacter sp. I3]
gi651436212	10.07	1	1	149	16.1	6.01	4.20	hypothetical protein [Arthrobacter sp. H41]
gi517591247	7.20	1	1	236	25.0	6.06	4.20	glutamine amidotransferase [Arthrobacter sp. 135MFCol5.1]
gi914716013	4.76	2	1	273	27.9	8.24	4.20	hypothetical protein [Arthrobacter sp. ZBG10]
gi635352487	3.68	2	1	571	60.5	5.15	4.20	putative ABC transporter [Arthrobacter siccitolerans]
gi654817676	1.61	1	1	1183	126.0	5.57	4.20	1-pyrroline-5-carboxylate dehydrogenase [Arthrobacter sp. UNC362MFTsu5.1]
gi910747027	6.38	1	1	235	26.0	5.67	4.19	uncharacterized protein YbjT [Arthrobacter sp. Hiyo8]
gi742854494	4.56	1	1	307	33.5	6.55	4.19	hypothetical protein [Arthrobacter sp. W1]
gi119949434	5.42	2	1	369	39.7	6.60	4.19	oxidoreductase family, NAD-binding Rossmann fold domain protein [Arthrobacter aurescens
gi651475549	6.09	1	1	279	30.0	4.84	4.19	alpha/beta hydrolase [Arthrobacter nicotinovorans]
gi910252521	4.57	1	1	416	43.1	4.98	4.19	alcohol dehydrogenase [Arthrobacter siccitolerans]
gi674646942	2.00	1	1	401	41.3	6.13	4.19	Carboxypeptidase G2 precursor [Arthrobacter sp. 11W110_air]
gi551256183	1.73	1	1	520	56.6	5.60	4.19	hypothetical protein [Arthrobacter sp. PAO19]
gi470220715	2.39	1	1	545	57.9	5.36	4.19	capsular polysaccharide biosynthesis protein YveL [Arthrobacter gangotriensis Lz1y]
gi651442365	1.69	1	1	1184	125.7	5.67	4.19	1-pyrroline-5-carboxylate dehydrogenase [Arthrobacter sp. 9MFCol3.1]
gi323470333	2.77	1	1	289	31.1	9.85	4.19	Heat shock protein [Arthrobacter phenanthrenivorans Sphe3]
gi723608904	4.11	2	1	389	42.2	5.44	4.19	hypothetical protein ART_2681 [Arthrobacter sp. PAMC25486]
gi910694642	12.17	2	1	189	20.1	11.47	4.18	conserved hypothetical protein [Arthrobacter sp. Hiyo6]
gi765006568	5.94	1	1	202	21.5	9.69	4.18	ArsR family transcriptional regulator [Arthrobacter sp. A3]
gi162952845	3.45	1	1	319	34.0	5.12	4.18	fumarylacetoacetate (FAA) hydrolase family [Renibacterium salmoninarum ATCC 33209]
gi518312776	2.59	1	1	580	62.6	8.51	4.18	hypothetical protein [Arthrobacter sp. TB 23]
gi648572962	4.01	1	1	324	35.8	5.19	4.18	aldo/keto reductase [Arthrobacter sp. 135MFCol5.1]
gi937259043	4.43	1	1	203	21.6	7.46	4.18	hypothetical protein AO716_13960 [Arthrobacter sp. Edens01]
gi476402590	22.08	1	1	77	8.3	8.68	4.18	hypothetical protein D477_002698 [Arthrobacter crystallopoietes BAB-32]
gi786031847	3.45	1	1	406	44.3	7.34	4.18	PucR family transcriptional regulator [Arthrobacter chlorophenolicus]
gi515766219	2.79	2	1	394	42.6	5.03	4.18	aminotransferase [Arthrobacter sp. M2012083]
gi916815971	10.43	2	1	115	13.2	8.60	4.18	hypothetical protein [Arthrobacter sp. MA-N2]
gi359304859	4.59	1	1	196	21.9	6.74	4.18	hypothetical protein ARGLB_080_01730 [Arthrobacter globiformis NBRC 12137]
gi517598073	5.80	1	1	276	30.1	6.62	4.18	S26 family signal peptidase [Arthrobacter sp. 162MFSha1.1]
gi651443677	2.33	1	1	688	74.6	5.41	4.18	cell division protein FtsH [Arthrobacter sp. 9MFCol3.1]
gi759736362	3.75	5	1	373	38.4	9.82	4.17	UDP-N-acetylglucosamine--N-acetylmuramyl-(pentapeptide) pyrophosphoryl-undecaprenol
gi651506212	11.21	1	1	107	12.3	10.07	4.17	hypothetical protein [Arthrobacter sp. 35W]
gi786026143	1.86	1	1	484	52.8	5.81	4.17	pilus assembly protein CpaF [Arthrobacter chlorophenolicus]
gi517598468	2.89	2	1	415	45.3	5.57	4.17	hypothetical protein [Arthrobacter sp. 162MFSha1.1]
gi927033480	11.26	1	1	151	16.5	8.56	4.17	hypothetical protein AFL94_15505 [Arthrobacter sp. LS16]
gi651498330	8.48	1	1	165	17.8	5.11	4.17	OHCU decarboxylase [Arthrobacter sp. 35W]

gi916816345	11.34	1	1	97	10.3	8.02	4.17	hypothetical protein [Arthrobacter sp. MA-N2]
gi757623082	3.03	1	1	627	65.6	5.36	4.17	hypothetical protein TV39_16195 [Arthrobacter sp. SPG23]
gi786027163	9.78	1	1	184	19.4	10.71	4.17	hypothetical protein [Arthrobacter chlorophenolicus]
gi908699408	3.26	2	1	337	36.3	6.25	4.17	exopolyphosphatase [Arthrobacter sp. RIT-PI-e]
gi742858903	2.54	1	1	512	55.4	4.74	4.17	benzene 1,2-dioxygenase [Arthrobacter sp. W1]
gi916870087	4.61	1	1	304	31.8	5.02	4.17	hypothetical protein [Arthrobacter sp. Br18]
gi910745729	31.91	2	1	47	5.3	4.82	4.17	hypothetical protein AHiyo8_34310 [Arthrobacter sp. Hiyo8]
gi116612649	2.91	1	1	550	57.7	9.28	4.17	drug resistance transporter, EmrB/QacA subfamily [Arthrobacter sp. FB24]
gi753937926	3.98	1	1	352	36.8	5.88	4.17	tRNA(Ile)-lysidine synthetase [Arthrobacter phenanthrenivorans]
gi767256825	2.40	1	1	458	47.4	10.52	4.16	lytic transglycosylase [Arthrobacter sp. IHBB 11108]
gi786026797	4.94	1	1	425	45.7	6.90	4.16	tRNA-specific 2-thiouridylase [Arthrobacter chlorophenolicus]
gi930825794	3.01	1	1	365	39.7	6.27	4.16	hypothetical protein AOZ07_05460 [Arthrobacter arilaitensis]
gi908690734	8.61	2	1	209	21.8	4.77	4.16	hypothetical protein [Arthrobacter sp. H41]
gi307746526	2.81	1	1	392	39.5	4.69	4.16	glycerate kinase [Arthrobacter arilaitensis Re117]
gi910696485	12.87	2	1	171	18.5	7.02	4.16	hypothetical protein AHiyo6_11590 [Arthrobacter sp. Hiyo6]
gi742068657	5.13	1	1	390	43.9	6.49	4.16	hypothetical protein ANMWB30_42780 [Arthrobacter sp. MWB30]
gi489894346	3.26	1	1	337	35.7	5.54	4.15	LacI family transcriptional regulator [Arthrobacter globiformis]
gi723609809	5.11	3	1	235	25.8	9.76	4.15	ABC-1 domain protein [Arthrobacter sp. PAMC25486]
gi765009530	2.06	1	1	678	72.7	6.83	4.15	ABC transporter [Arthrobacter sp. A3]
gi916835018	1.66	1	1	782	87.2	7.61	4.15	GTP pyrophosphokinase [Arthrobacter sp. H14]
gi307746071	3.20	1	1	562	61.3	6.28	4.15	putative fatty-acid--CoA ligase [Arthrobacter arilaitensis Re117]
gi639130245	5.46	2	1	293	32.0	4.92	4.15	oxidoreductase [Arthrobacter sp. CAL618]
gi910696586	4.57	1	1	569	62.5	5.94	4.15	amylo-Alpha-1,6-Glucosidase [Arthrobacter sp. Hiyo6]
gi910697797	3.87	1	1	413	44.2	5.94	4.15	hypothetical protein AHiyo6_00680 [Arthrobacter sp. Hiyo6]
gi917760048	3.19	1	1	313	34.3	10.98	4.15	hypothetical protein [Arthrobacter sp. L77]
gi757624445	7.56	1	1	238	24.7	4.89	4.14	haloacid dehalogenase [Arthrobacter sp. SPG23]
gi916816361	2.34	1	1	513	55.6	6.20	4.14	hypothetical protein [Arthrobacter sp. MA-N2]
gi939037328	6.92	1	1	130	13.7	8.73	4.14	hypothetical protein [Arthrobacter nitroguajacolicus]
gi470219993	6.37	3	1	157	16.8	9.70	4.14	putative small multidrug efflux protein [Arthrobacter gangotriensis Lz1y]
gi651445064	1.90	1	1	422	45.0	5.63	4.14	glycosyl transferase [Arthrobacter nicotinovorans]
gi639130794	2.02	1	1	642	68.6	6.01	4.14	hypothetical protein [Arthrobacter sp. CAL618]
gi914717124	4.44	1	1	518	57.5	5.06	4.14	methionine--tRNA ligase [Arthrobacter sp. ZBG10]
gi910283780	11.41	1	1	184	20.4	6.73	4.14	GNAT family N-acetyltransferase [Arthrobacter sp. A3]
gi918267365	4.00	1	1	350	36.2	5.01	4.14	homoserine dehydrogenase [Arthrobacter sp. Hiyo1]
gi786034424	1.91	1	1	419	46.1	9.69	4.14	membrane protein [Arthrobacter chlorophenolicus]
gi917759761	4.59	1	1	218	23.9	5.21	4.14	hypothetical protein [Arthrobacter sp. L77]
gi651504926	7.10	1	1	169	18.1	6.52	4.14	cysteine methyltransferase [Arthrobacter sp. 35W]
gi116609045	14.13	1	1	92	10.3	9.69	4.13	hypothetical protein Arth_0368 [Arthrobacter sp. FB24]
gi916813851	3.22	2	1	311	33.5	5.57	4.13	LacI family transcriptional regulator [Arthrobacter nicotinovorans]
gi323469778	22.83	2	1	92	10.2	11.60	4.13	hypothetical protein Asphe3_23270 [Arthrobacter phenanthrenivorans Sphe3]
gi916869698	8.23	1	1	243	25.2	11.30	4.13	hypothetical protein [Arthrobacter sp. Br18]
gi690773152	3.44	1	1	349	38.9	5.43	4.13	hypothetical protein HMPREF2128_04125 [Arthrobacter albus DNF00011]
gi910695299	7.43	2	1	148	15.4	7.69	4.13	glutamyl-tRNA(Gln) amidotransferase subunit A, partial [Arthrobacter sp. Hiyo6]
gi674645366	1.37	1	1	1096	116.8	4.86	4.13	Carbamoyl-phosphate synthase large chain [Arthrobacter sp. 11W110_air]
gi654818704	21.24	4	1	113	12.0	6.29	4.13	LuxR family transcriptional regulator [Arthrobacter sp. UNC362MFTsu5.1]
gi759729687	3.16	1	1	507	55.2	5.14	4.12	aldehyde dehydrogenase [Arthrobacter sp. L77]
gi162954573	10.53	1	1	114	13.0	5.82	4.12	hypothetical protein RSal33209_2358 [Renibacterium salmoninarum ATCC 33209]
gi928542296	5.64	1	1	319	35.8	5.43	4.12	hypothetical protein SEA_BRENT_53 [Arthrobacter phage Brent]
gi518311327	5.16	3	1	252	27.9	6.74	4.12	hypothetical protein [Arthrobacter sp. TB 23]
gi651429435	1.96	1	1	408	45.6	5.85	4.12	bifunctional D-altronate/D-mannonate dehydratase [Arthrobacter sanguinis]
gi910744953	3.28	1	1	335	35.5	6.54	4.12	conserved hypothetical protein [Arthrobacter sp. Hiyo8]
gi937259028	4.58	3	1	415	45.1	6.48	4.12	lipase [Arthrobacter sp. Edens01]
gi651435651	7.78	1	1	180	19.5	5.02	4.12	peptidylprolyl isomerase [Arthrobacter sp. H41]
gi928487063	9.76	1	1	123	13.9	5.27	4.12	hypothetical protein AOC05_08870 [Arthrobacter alpinus]
gi651494521	2.84	1	1	563	61.0	5.62	4.11	RNase J family beta-CASP ribonuclease [Arthrobacter sp. H20]
gi517591888	1.46	1	1	549	56.7	5.30	4.11	hypothetical protein [Arthrobacter sp. 135MFCol5.1]
gi515765558	4.78	1	1	418	43.1	9.86	4.11	hypothetical protein [Arthrobacter sp. M2012083]
gi651442037	2.41	1	1	580	58.8	4.93	4.11	dihydroxyacetone kinase [Arthrobacter sp. 9MFCol3.1]

gi476401128	4.58	1	1	415	45.5	6.06	4.11	cytochrome P450 [Arthrobacter crystallopoietes BAB-32]
gi517591850	3.97	1	1	478	49.2	5.67	4.11	hypothetical protein [Arthrobacter sp. 135MFCol5.1]
gi765010710	3.39	1	1	295	30.9	5.01	4.11	hypothetical protein [Arthrobacter sp. A3]
gi162953022	1.97	1	1	557	61.6	5.92	4.11	transcriptional regulator, LuxR family [Renibacterium salmoninarum ATCC 33209]
gi919134909	1.54	1	1	844	90.3	8.82	4.11	hypothetical protein [Arthrobacter chlorophenolicus]
gi651485165	4.10	1	1	366	40.0	5.05	4.11	hypothetical protein [Arthrobacter sp. Br18]
gi654815622	8.13	1	1	160	18.6	9.73	4.11	transposase [Arthrobacter sp. PAO19]
gi476399947	11.06	1	1	199	21.2	6.34	4.10	hypothetical protein D477_016180 [Arthrobacter crystallopoietes BAB-32]
gi757624845	3.99	1	1	326	33.3	11.06	4.10	hypothetical protein TV39_09280 [Arthrobacter sp. SPG23]
gi723609638	2.21	1	1	543	56.5	8.54	4.10	hypothetical protein ART_3415 [Arthrobacter sp. PAMC25486]
gi654827534	6.63	1	1	347	37.0	4.67	4.10	peptidase M4 [Arthrobacter sp. H5]
gi674646834	1.58	5	1	1141	126.5	5.34	4.10	hypothetical protein BN1051_03046 [Arthrobacter sp. 11W110_air]
gi640198549	0.88	2	1	909	92.7	5.39	4.10	hypothetical protein [Arthrobacter sp. 31Y]
gi654828751	21.79	1	1	78	8.6	10.42	4.10	hypothetical protein [Arthrobacter sp. H5]
gi723608555	1.86	1	1	377	40.0	5.54	4.10	acetate kinase [Arthrobacter sp. PAMC25486]
gi651440493	4.74	1	1	190	20.8	7.91	4.10	hypothetical protein [Arthrobacter sp. H14]
gi742069482	12.57	2	1	175	17.8	5.25	4.10	lipoprotein [Arthrobacter sp. MWB30]
gi723609682	3.27	1	1	275	29.0	6.15	4.10	short-chain dehydrogenase [Arthrobacter sp. PAMC25486]
gi654814255	10.00	1	1	220	23.9	5.91	4.09	hypothetical protein [Arthrobacter sp. MA-N2]
gi919134862	1.70	1	1	941	95.6	6.02	4.09	hypothetical protein [Arthrobacter chlorophenolicus]
gi116611609	3.20	1	1	344	35.7	5.41	4.09	3-oxoacyl-(acyl-carrier-protein) synthase [Arthrobacter sp. FB24]
gi910737468	35.42	1	1	48	5.3	9.99	4.09	conserved hypothetical protein [Arthrobacter sp. Hiyo4]
gi927296367	5.79	1	1	242	25.8	5.14	4.09	hypothetical protein AL755_13360 [Arthrobacter sp. ERGS1:01]
gi651439206	3.97	1	1	428	48.1	4.86	4.09	biotin carboxylase [Arthrobacter sp. H14]
gi403230766	4.29	1	1	280	31.6	8.59	4.09	putative NAD(P)H:flavin oxidoreductase / NAD(P)H nitroreductase [Arthrobacter sp. Rue61a
gi162953115	3.16	2	1	348	38.4	6.74	4.09	RtcB [Renibacterium salmoninarum ATCC 33209]
gi765012192	8.50	2	1	306	33.1	7.75	4.09	XRE family transcriptional regulator [Arthrobacter sp. A3]
gi652423216	6.16	1	1	341	36.6	4.73	4.09	agmatine deiminase [Arthrobacter castelli]
gi674646667	1.02	1	1	1079	120.6	5.38	4.09	hypothetical protein BN1051_02877 [Arthrobacter sp. 11W110_air]
gi307746156	6.93	1	1	202	22.4	5.29	4.09	tetratrico peptide repeat-containing protein [Arthrobacter arilaitensis Re117]
gi916871478	8.28	1	1	169	19.0	9.52	4.08	adenylate kinase [Arthrobacter sp. H5]
gi757624353	2.61	1	1	805	87.1	4.87	4.08	phosphoenolpyruvate synthase [Arthrobacter sp. SPG23]
gi674645767	9.38	1	1	192	19.9	11.87	4.08	DoxX [Arthrobacter sp. 11W110_air]
gi651459195	11.04	1	1	163	17.4	9.25	4.08	hypothetical protein [Arthrobacter sp. 35/47]
gi651440288	4.83	1	1	207	22.8	10.81	4.08	CDP-diacylglycerol--glycerol-3-phosphate 3-phosphatidyltransferase [Arthrobacter sp. H14]
gi916782314	4.10	1	1	415	44.1	6.81	4.08	hypothetical protein [Arthrobacter sp. 35W]
gi928487765	6.87	1	1	233	25.3	7.34	4.08	ubiquinone biosynthesis methyltransferase UbiE [Arthrobacter alpinus]
gi162955484	5.35	1	1	243	26.5	9.20	4.08	probable dehydrogenase, putative [Renibacterium salmoninarum ATCC 33209]
gi517604034	0.92	2	1	1520	155.7	5.29	4.08	multifunctional nuclease/2',3'-cyclic-nucleotide 2'-phosphodiesterase/5'-nucleotidase/3'-nuc
gi759775904	15.38	1	1	104	11.4	10.95	4.08	XRE family transcriptional regulator [Arthrobacter sp. SPG23]
gi639130248	4.42	1	1	339	37.3	5.72	4.08	aldo/keto reductase [Arthrobacter sp. CAL618]
gi116612679	2.89	1	1	415	45.3	4.83	4.08	Pyruvate dehydrogenase (acetyl-transferring) [Arthrobacter sp. FB24]
gi652423934	10.34	1	1	116	12.5	8.44	4.08	nitrogen regulatory protein P-II [Arthrobacter castelli]
gi914717849	5.26	1	1	361	38.1	6.67	4.08	methionine ABC transporter ATP-binding protein [Arthrobacter sp. ZBG10]
gi640203282	5.38	5	1	409	41.0	5.43	4.08	3-oxoacyl-ACP synthase [Arthrobacter sp. 31Y]
gi767258515	13.41	1	1	82	9.1	7.43	4.07	hypothetical protein UM93_14475 [Arthrobacter sp. IHBB 11108]
gi651471963	5.82	1	1	275	28.3	5.11	4.07	competence protein ComEA [Arthrobacter nicotinovorans]
gi470221486	4.11	1	1	292	30.8	4.86	4.07	nucleoside diphosphate glycosyl epimerase [Arthrobacter gangotriensis Lz1y]
gi759732423	4.25	1	1	306	35.1	7.24	4.07	3-methyladenine DNA glycosylase [Arthrobacter sp. L77]
gi651429190	2.68	1	1	410	44.3	6.37	4.07	two-component system sensor histidine kinase [Arthrobacter sanguinis]
gi170783524	8.08	1	1	198	21.6	9.96	4.07	putative site-specific recombinase (plasmid) [Arthrobacter sp. AK-1]
gi648574524	4.47	2	1	246	26.5	5.26	4.07	hypothetical protein [Arthrobacter sp. 131MFCol6.1]
gi119951273	5.58	1	1	251	28.0	5.67	4.07	conserved hypothetical protein [Arthrobacter aurescens TC1]
gi162953819	5.08	1	1	315	33.4	5.48	4.07	homoserine O-acetyltransferase [Renibacterium salmoninarum ATCC 33209]
gi219860477	5.80	2	1	276	30.1	5.03	4.07	alpha/beta hydrolase fold protein [Arthrobacter chlorophenolicus A6]
gi737789865	5.88	1	1	323	34.8	5.02	4.06	ornithine carbamoyltransferase [Arthrobacter albus]
gi162954231	8.94	1	1	246	26.9	6.35	4.06	transcriptional regulator, GntR family [Renibacterium salmoninarum ATCC 33209]
gi742759517	4.30	1	1	558	60.3	9.63	4.06	FAD-binding dehydrogenase [Arthrobacter phenanthrenivorans]

gi635351215	2.04	1	1	587	61.2	4.79	4.06	phosphoglucomutase/phosphomannomutase, alpha/beta/alpha domain I family protein [Ar
gi753938942	0.99	1	1	810	90.5	5.24	4.06	hypothetical protein [Arthrobacter phenanthrenivorans]
gi517600088	7.82	2	1	294	31.5	5.41	4.06	hypothetical protein [Arthrobacter sp. 162MFSha1.1]
gi359307159	4.61	1	1	477	50.5	4.97	4.06	putative amidase [Arthrobacter globiformis NBRC 12137]
gi927295942	6.25	1	1	272	28.5	9.91	4.06	hydrolase [Arthrobacter sp. ERGS1:01]
gi910738013	4.53	1	1	287	30.5	5.12	4.06	glycolate oxidase subunit GlcD [Arthrobacter sp. Hiyo4]
gi765008024	7.30	2	1	274	30.6	8.98	4.06	hypothetical protein [Arthrobacter sp. A3]
gi551254578	4.62	1	1	433	46.0	5.78	4.06	CAIB/BAIF family acyl-CoA thioesterase [Arthrobacter sp. PAO19]
gi910738465	4.26	1	1	305	32.8	7.59	4.05	uncharacterized protein in pkwA 5'regio [Arthrobacter sp. Hiyo4]
gi916876263	0.43	2	1	3503	372.3	5.07	4.05	non-ribosomal peptide synthetase [Arthrobacter sp. 31Y]
gi742853051	4.59	1	1	283	29.1	5.00	4.05	hypothetical protein [Arthrobacter sp. W1]
gi910693523	7.87	1	1	267	28.0	4.67	4.05	PTS-dependent dihydroxyacetone kinase, dihydroxyacetone-binding subunit DhaK [Arthrob
gi786026289	7.91	2	1	316	32.8	5.64	4.05	ATPase [Arthrobacter chlorophenolicus]
gi651430296	19.00	1	1	100	11.1	9.14	4.05	hypothetical protein [Arthrobacter sanguinis]
gi518311309	13.60	1	1	125	14.1	10.67	4.05	MULTISPECIES: 30S ribosomal protein S13 [Arthrobacter]
gi651430135	1.72	2	1	466	47.5	8.10	4.05	permease [Arthrobacter sanguinis]
gi937258477	4.70	1	1	362	40.8	6.67	4.05	hypothetical protein AO716_10565 [Arthrobacter sp. Edens01]
gi651498425	3.28	3	1	396	43.3	5.44	4.05	acyl-CoA dehydrogenase [Arthrobacter sp. 35W]
gi916869858	2.83	1	1	495	52.4	6.40	4.05	hypothetical protein [Arthrobacter sp. Br18]
gi470215930	5.54	1	1	271	29.1	8.24	4.05	dipeptide ABC transporter ATP-binding protein [Arthrobacter gangotriensis Lz1y]
gi910748477	4.26	1	1	282	30.3	10.71	4.05	uncharacterized transporter Mb2022c [Arthrobacter sp. Hiyo8]
gi443480564	2.66	1	1	526	56.5	9.29	4.05	transcriptional regulator [Arthrobacter nitrophenolicus]
gi651430616	3.47	1	1	576	62.8	5.78	4.05	multidrug ABC transporter ATPase [Arthrobacter sanguinis]
gi757626382	10.21	2	1	235	25.6	10.14	4.05	response regulator receiver protein [Arthrobacter sp. SPG23]
gi928985603	1.80	1	1	555	60.3	7.72	4.04	hypothetical protein [Arthrobacter sp. ERGS1:01]
gi654827857	3.87	2	1	388	40.2	6.80	4.04	ROK family transcriptional regulator [Arthrobacter sp. H5]
gi910251031	6.78	2	1	236	24.2	5.39	4.04	hypothetical protein [Arthrobacter siccitolerans]
gi823668149	19.05	1	1	63	7.4	8.54	4.04	hypothetical protein AA310_01875 [Arthrobacter sp. YC-RL1]
gi639130875	18.27	1	1	104	12.0	8.79	4.04	hypothetical protein [Arthrobacter sp. CAL618]
gi651433180	1.69	1	1	826	92.7	5.95	4.04	kojibiose phosphorylase [Arthrobacter sp. H41]
gi737800297	9.21	1	1	152	16.9	4.88	4.04	GNAT family N-acetyltransferase [Arthrobacter castelli]
gi636843763	9.43	1	1	212	22.6	7.12	4.03	GntR family transcriptional regulator, partial [Arthrobacter sp. TB 26]
gi917013340	2.52	2	1	794	84.7	6.20	4.03	daunorubicin resistance protein DrrC [Arthrobacter sanguinis]
gi917739780	5.11	1	1	411	44.4	5.10	4.03	aspartate aminotransferase [Arthrobacter sp. W1]
gi636844380	2.20	2	1	773	82.9	9.01	4.03	hypothetical protein, partial [Arthrobacter sp. TB 26]
gi470216697	11.11	2	1	171	17.9	5.30	4.03	arginine repressor ArgR [Arthrobacter gangotriensis Lz1y]
gi654819605	4.89	1	1	327	36.5	5.86	4.03	glycosyl transferase [Arthrobacter sp. UNC362MFTsu5.1]
gi916782288	2.46	2	1	406	42.9	5.45	4.03	hypothetical protein [Arthrobacter sp. 35W]
gi723610320	1.32	1	1	986	105.8	6.35	4.03	hypothetical protein ART_4097 [Arthrobacter sp. PAMC25486]
gi443481461	8.77	1	1	171	18.7	6.55	4.03	MarR family transcriptional regulator [Arthrobacter nitrophenolicus]
gi470217079	0.99	1	1	1107	119.6	5.86	4.03	SMC domain-containing protein [Arthrobacter gangotriensis Lz1y]
gi654823286	3.92	1	1	408	45.7	8.40	4.03	transposase [Arthrobacter sp. I3]
gi757626262	3.91	2	1	562	58.2	5.20	4.02	urea amidolyase [Arthrobacter sp. SPG23]
gi674646237	11.49	1	1	148	14.7	5.50	4.02	hypothetical protein BN1051_02441 [Arthrobacter sp. 11W110_air]
gi908699551	2.24	1	1	401	42.2	6.07	4.02	diguanylate cyclase [Arthrobacter sp. RIT-PI-e]
gi823668187	4.34	1	1	346	37.9	8.44	4.02	cyclic pyranopterin monophosphate synthase [Arthrobacter sp. YC-RL1]
gi665847987	5.68	1	1	229	23.6	5.02	4.02	hypothetical protein [Arthrobacter sp. AK-YN10]
gi470221303	19.79	4	1	96	10.9	11.12	4.02	hypothetical protein ADIAG_00156 [Arthrobacter gangotriensis Lz1y]
gi470216766	1.54	1	1	842	86.5	6.54	4.01	DNA internalization-related competence protein ComEC/Rec2 [Arthrobacter gangotriensis L
gi742855134	3.71	1	1	458	47.4	9.83	4.01	amino acid transporter [Arthrobacter sp. W1]
gi470217592	9.32	1	1	118	12.5	5.41	4.01	Acetyltransferase (GNAT) family protein [Arthrobacter gangotriensis Lz1y]
gi928488010	5.16	1	1	310	32.9	5.83	4.01	hypothetical protein AOC05_15145 [Arthrobacter alpinus]
gi823668058	1.31	1	1	994	110.1	5.29	4.01	alpha-mannosidase, partial [Arthrobacter sp. YC-RL1]
gi162952257	2.89	1	1	381	39.9	5.39	4.01	hypothetical protein RSal33209_0014 [Renibacterium salmoninarum ATCC 33209]
gi219857730	2.92	1	1	342	36.1	7.96	4.01	transcriptional regulator, LacI family [Arthrobacter chlorophenolicus A6]
gi910249849	3.59	2	1	334	35.9	9.29	4.01	LysR family transcriptional regulator [Arthrobacter siccitolerans]
gi651444668	2.78	2	1	683	70.5	7.33	4.01	peptide ABC transporter ATPase [Arthrobacter nicotinovorans]
gi918469464	2.62	1	1	496	50.3	10.02	4.01	lytic transglycosylase [Arthrobacter crystallopoietes]

gi674645562	4.16	3	1	409	43.7	8.15	4.01	carbohydrate diacid transcriptional activator CdaR [Arthrobacter sp. 11W110_air]
gi636843755	3.59	1	1	418	46.9	4.94	4.01	hypothetical protein [Arthrobacter sp. TB 26]
gi651443009	3.29	1	1	578	62.2	9.35	4.00	hypothetical protein [Arthrobacter sp. 9MFCol3.1]
gi908699568	3.03	1	1	561	56.8	5.02	4.00	phosphoenolpyruvate-protein phosphotransferase [Arthrobacter sp. RIT-PI-e]
gi162952618	4.15	3	1	217	23.4	8.84	4.00	ABC transporter ATP-binding protein [Renibacterium salmoninarum ATCC 33209]
gi654813806	3.60	1	1	361	40.1	9.04	4.00	mannosyltransferase [Arthrobacter sp. MA-N2]
gi654827706	2.95	1	1	339	35.5	5.40	4.00	universal stress protein UspA [Arthrobacter sp. H5]
gi918268413	4.21	1	1	285	32.0	5.38	3.99	uncharacterized oxidoreductase MSMEG_2408/MSMEI_2347 [Arthrobacter sp. Hiyo1]
gi908697324	2.40	1	1	458	47.9	5.74	3.99	hypothetical protein [Arthrobacter sp. RIT-PI-e]
gi517601939	6.25	1	1	288	30.5	10.21	3.99	SDR family oxidoreductase [Arthrobacter sp. 162MFSha1.1]
gi654813054	6.29	1	1	143	14.8	5.55	3.99	organic hydroperoxide resistance protein [Arthrobacter sp. MA-N2]
gi654819332	9.56	1	1	136	14.5	5.27	3.99	MerR family transcriptional regulator [Arthrobacter sp. UNC362MFTsu5.1]
gi928486534	0.97	1	1	1132	117.8	7.90	3.99	hypothetical protein AOC05_05225 [Arthrobacter alpinus]
gi910251007	2.25	1	1	579	61.0	4.97	3.99	DNA repair protein RecN [Arthrobacter siccitolerans]
gi767258727	3.79	1	1	343	36.1	5.67	3.99	cystathionine beta-synthase [Arthrobacter sp. IHBB 11108]
gi651437927	2.33	1	1	387	42.3	4.79	3.99	GNAT family N-acetyltransferase [Arthrobacter sp. H14]
gi651441345	1.94	1	1	515	53.8	7.20	3.98	acetyl-CoA carboxyltransferase [Arthrobacter sp. 9MFCol3.1]
gi443483075	8.20	1	1	183	18.5	5.97	3.98	hypothetical protein G205_01993 [Arthrobacter nitrophenolicus]
gi742756320	13.60	1	1	125	13.6	6.54	3.98	hypothetical protein RM50_10475 [Arthrobacter phenanthrenivorans]
gi786029378	4.43	1	1	451	47.7	5.47	3.98	LuxR family transcriptional regulator [Arthrobacter chlorophenolicus]
gi219859544	3.17	1	1	410	43.6	5.08	3.98	cysteine/1-D-myo-inosityl 2-amino-2-deoxy-alpha-D-glucopyranoside ligase [Arthrobacter c
gi914715248	5.02	1	1	299	32.0	9.26	3.98	hypothetical protein [Arthrobacter sp. ZBG10]
gi476399091	1.31	1	1	609	67.1	6.34	3.98	hypothetical protein D477_020183 [Arthrobacter crystallopoietes BAB-32]
gi651464770	3.67	1	1	409	42.5	5.05	3.98	Zn-dependent hydrolase [Arthrobacter sp. 35/47]
gi636847426	3.39	1	1	236	24.5	5.20	3.98	short-chain dehydrogenase [Arthrobacter sp. TB 26]
gi654816053	1.54	2	1	520	54.7	7.05	3.98	acetyl-CoA carboxyltransferase [Arthrobacter sp. UNC362MFTsu5.1]
gi476399643	4.95	1	1	182	20.3	7.87	3.98	carboxymuconolactone decarboxylase [Arthrobacter crystallopoietes BAB-32]
gi759704592	6.48	3	1	355	37.0	9.25	3.98	hypothetical protein [Arthrobacter globiformis]
gi651432359	16.87	1	1	83	8.6	5.01	3.98	molybdenum cofactor biosynthesis protein MoaD [Arthrobacter sp. H41]
gi927294108	3.38	1	1	444	47.6	4.88	3.97	hypothetical protein AL755_08790 [Arthrobacter sp. ERGS1:01]
gi654812134	2.96	1	1	304	32.8	6.04	3.97	transketolase [Arthrobacter sp. MA-N2]
gi651429337	3.16	4	1	632	66.1	5.48	3.97	2-succinyl-5-enolpyruvyl-6-hydroxy-3-cyclohexene-1-carboxylate synthase [Arthrobacter sa
gi651480018	3.23	1	1	279	30.0	5.47	3.97	hypothetical protein [Arthrobacter sp. Br18]
gi470216628	5.37	1	1	242	26.5	7.06	3.97	BshB2 potential contributor to bacillithiol synthesis [Arthrobacter gangotriensis Lz1y]
gi651484518	0.75	1	1	1610	178.4	5.43	3.97	glutamate dehydrogenase [Arthrobacter sp. Br18]
gi759760762	4.14	1	1	556	58.9	5.63	3.97	histidine kinase [Arthrobacter sp. Rue61a]
gi359304565	1.84	1	1	599	63.6	5.36	3.97	gamma-glutamyltranspeptidase [Arthrobacter globiformis NBRC 12137]
gi928987108	1.78	1	1	562	56.4	10.48	3.97	hypothetical protein [Arthrobacter sp. ERGS1:01]
gi937258467	3.54	1	1	480	52.3	5.24	3.97	cysteine--tRNA ligase [Arthrobacter sp. Edens01]
gi742068602	1.25	1	1	1282	135.2	5.82	3.96	glycosyl hydrolase family 31 domain-containing protein [Arthrobacter sp. MWB30]
gi651479962	4.63	1	1	216	23.0	8.88	3.96	molybdenum cofactor sulfurase [Arthrobacter sp. Br18]
gi916816432	5.45	1	1	202	21.8	6.02	3.96	hypothetical protein [Arthrobacter sp. MA-N2]
gi910742063	10.83	1	1	157	16.4	8.44	3.96	uncharacterized protein YjgR [Arthrobacter sp. Hiyo4]
gi517591762	3.77	1	1	371	39.8	8.44	3.96	glycosyl transferase [Arthrobacter sp. 135MFCol5.1]
gi674645452	4.32	1	1	301	31.9	5.40	3.96	hypothetical protein BN1051_01634 [Arthrobacter sp. 11W110_air]
gi470220662	9.31	1	1	247	25.9	5.05	3.96	dehydrogenase [Arthrobacter gangotriensis Lz1y]
gi823666071	8.48	1	1	165	18.1	4.65	3.96	ATPase [Arthrobacter sp. YC-RL1]
gi939051765	3.86	1	1	207	22.4	5.21	3.96	hypothetical protein [Arthrobacter sp. JCM 19049]
gi823666578	4.64	1	1	323	35.0	9.23	3.96	hypothetical protein AA310_12450 [Arthrobacter sp. YC-RL1]
gi939050412	4.32	3	1	370	41.3	6.46	3.96	hypothetical protein [Arthrobacter sp. JCM 19049]
gi759702539	3.23	2	1	310	32.8	4.86	3.95	phosphoesterase [Arthrobacter globiformis]
gi307746604	4.43	1	1	451	50.0	5.12	3.95	replicative DNA helicase [Arthrobacter arilaitensis Re117]
gi916692057	4.36	1	1	367	40.8	7.64	3.95	hypothetical protein [Arthrobacter castelli]
gi928487805	6.98	1	1	129	14.4	9.70	3.95	hypothetical protein AOC05_13795 [Arthrobacter alpinus]
gi651452664	6.71	1	1	313	35.4	8.51	3.95	hypothetical protein [Arthrobacter nicotinovorans]
gi937258080	6.04	1	1	298	32.6	5.24	3.95	hypothetical protein AO716_08170 [Arthrobacter sp. Edens01]
gi307743696	7.98	1	1	263	27.1	6.05	3.94	putative aspartate dehydrogenase [Arthrobacter arilaitensis Re117]
gi674646977	7.33	1	1	191	20.6	5.40	3.94	HTH-type transcriptional regulator PuuR [Arthrobacter sp. 11W110_air]

gi674645612	1.35	1	1	740	80.2	5.82	3.94	ATP-dependent DNA helicase RecG [Arthrobacter sp. 11W110_air]
gi674644113	5.96	1	1	285	31.0	6.62	3.94	1,4-Dihydroxy-2-naphthoyl-CoA synthase [Arthrobacter sp. 11W110_air]
gi910737972	5.00	1	1	380	41.3	7.69	3.94	DNA polymerase III subunit gamma/tau [Arthrobacter sp. Hiyo4]
gi307744715	2.29	1	1	393	41.5	4.64	3.94	signal recognition particle-docking protein FtsY [Arthrobacter arilaitensis Re117]
gi651503481	3.33	1	1	330	35.2	5.36	3.94	alkane 1-monooxygenase [Arthrobacter sp. 35W]
gi651447172	3.31	4	1	605	67.5	5.14	3.94	siderophore biosynthesis protein IucA [Arthrobacter nicotinovorans]
gi116612168	4.62	1	1	238	25.8	6.30	3.94	transcriptional regulator, GntR family [Arthrobacter sp. FB24]
gi910744499	5.45	1	1	275	28.8	9.44	3.93	ribose transport system permease protein RbsC [Arthrobacter sp. Hiyo8]
gi910746731	12.92	1	1	178	19.9	10.55	3.93	sn-glycerol-3-phosphate transport system permease protein UgpE [Arthrobacter sp. Hiyo8]
gi635349975	5.18	1	1	193	20.6	6.79	3.93	enoyl-CoA hydratase subunit II [Arthrobacter siccitolerans]
gi470217836	3.08	1	1	292	29.9	6.87	3.93	Universal stress protein family protein [Arthrobacter gangotriensis Lz1y]
gi489899572	5.47	1	1	457	48.8	5.16	3.93	two-component system sensor histidine kinase [Arthrobacter globiformis]
gi737790501	9.56	2	1	136	15.5	8.43	3.93	hypothetical protein [Arthrobacter albus]
gi403228560	1.33	1	1	1205	130.7	5.44	3.93	hypothetical protein ARUE_c10610 [Arthrobacter sp. Rue61a]
gi742759041	8.09	1	1	309	31.7	6.60	3.93	diacylglycerol kinase [Arthrobacter phenanthrenivorans]
gi640199153	1.29	1	1	855	95.6	6.57	3.93	hypothetical protein [Arthrobacter sp. 31Y]
gi916692353	5.37	1	1	242	27.6	6.55	3.92	hypothetical protein [Arthrobacter castelli]
gi918266021	9.70	1	1	165	18.7	4.44	3.92	activator of Hsp90 ATPase 1 family protein [Arthrobacter sp. Hiyo1]
gi515764995	2.23	1	1	359	38.3	9.04	3.92	dihydroorotate dehydrogenase 2 [Arthrobacter sp. M2012083]
gi652424238	4.09	1	1	367	38.8	8.46	3.92	phospho-N-acetylmuramoyl-pentapeptide-transferase [Arthrobacter castelli]
gi648574590	4.60	3	1	261	28.0	5.10	3.92	MULTISPECIES: iron ABC transporter ATP-binding protein [Arthrobacter]
gi162953898	3.45	1	1	406	43.1	5.19	3.91	methyltransferase [Renibacterium salmoninarum ATCC 33209]
gi908698631	3.78	1	1	291	29.8	4.87	3.91	inositol monophosphatase [Arthrobacter sp. RIT-PI-e]
gi654826907	3.23	1	1	371	38.6	5.06	3.91	oxidoreductase [Arthrobacter sp. H5]
gi651437319	4.90	2	1	204	23.7	7.84	3.90	hypothetical protein [Arthrobacter sp. H14]
gi162952790	4.61	1	1	282	29.6	5.00	3.90	citrate lyase beta chain [Renibacterium salmoninarum ATCC 33209]
gi517600507	6.70	1	1	194	21.0	9.80	3.90	hypothetical protein [Arthrobacter sp. 162MFSha1.1]
gi476399518	4.10	1	1	317	34.9	9.98	3.90	IS110 family transposase [Arthrobacter crystallopoietes BAB-32]
gi515766563	1.60	1	1	687	72.9	5.40	3.90	oxidoreductase [Arthrobacter sp. M2012083]
gi930827679	3.80	1	1	342	36.9	5.44	3.90	hypothetical protein AOZ07_16260 [Arthrobacter arilaitensis]
gi759704609	6.51	1	1	261	28.6	6.60	3.90	DNA-binding response regulator [Arthrobacter globiformis]
gi928487518	12.87	2	1	171	18.6	6.20	3.89	AsnC family transcriptional regulator [Arthrobacter alpinus]
gi1906823	10.85	1	1	212	22.7	10.96	3.89	hypothetical protein [Pimelobacter simplex]
gi116608779	2.85	1	1	351	35.7	9.41	3.89	Bile acid:sodium symporter [Arthrobacter sp. FB24]
gi470220207	4.01	1	1	424	44.9	6.67	3.89	nitrate/nitrite sensor protein NarQ [Arthrobacter gangotriensis Lz1y]
gi916816088	6.04	1	1	298	32.5	5.67	3.89	prephenate dehydratase [Arthrobacter sp. MA-N2]
gi116612878	1.69	1	1	651	67.2	5.34	3.88	heavy metal translocating P-type ATPase (plasmid) [Arthrobacter sp. FB24]
gi219860499	4.55	1	1	242	25.0	5.43	3.88	ANTAR domain protein with unknown sensor [Arthrobacter chlorophenolicus A6]
gi640193885	5.32	1	1	188	21.7	9.95	3.88	hypothetical protein [Arthrobacter sp. 31Y]
gi737786219	5.30	1	1	264	29.1	8.44	3.88	endonuclease III [Arthrobacter nitrophenolicus]
gi517598214	3.10	2	1	290	30.4	4.70	3.88	hypothetical protein [Arthrobacter sp. 162MFSha1.1]
gi651433503	7.11	1	1	225	23.5	4.96	3.88	hypothetical protein [Arthrobacter sp. H41]
gi757622837	6.82	1	1	337	35.8	7.61	3.88	allophanate hydrolase [Arthrobacter sp. SPG23]
gi919134904	1.56	1	1	1154	120.4	5.02	3.88	hypothetical protein [Arthrobacter chlorophenolicus]
gi654817061	5.33	1	1	375	41.0	8.22	3.88	fusaric acid resistance protein [Arthrobacter sp. UNC362MFTsu5.1]
gi742069453	5.48	2	1	310	34.5	10.04	3.87	ABC-type sugar transport system, permease component [Arthrobacter sp. MWB30]
gi916691942	3.33	1	1	330	35.3	5.17	3.87	exopolyphosphatase [Arthrobacter castelli]
gi916871742	9.79	1	1	143	16.0	11.12	3.87	hypothetical protein, partial [Arthrobacter sp. H5]
gi937258422	14.29	1	1	63	7.3	8.34	3.87	hypothetical protein AO716_10215 [Arthrobacter sp. Edens01]
gi723606905	18.99	2	1	79	9.5	9.63	3.87	hypothetical protein ART_0682 [Arthrobacter sp. PAMC25486]
gi765006892	2.49	1	1	281	31.5	9.55	3.87	sugar ABC transporter permease [Arthrobacter sp. A3]
gi928985252	2.68	1	1	299	32.8	5.25	3.87	hypothetical protein [Arthrobacter sp. ERGS1:01]
gi759733266	12.99	1	1	177	17.8	5.47	3.87	2-C-methyl-D-erythritol 2,4-cyclodiphosphate synthase [Arthrobacter sp. L77]
gi654817220	4.95	1	1	404	42.4	5.17	3.86	serine/threonine protein kinase [Arthrobacter sp. UNC362MFTsu5.1]
gi767257765	2.83	1	1	353	38.5	5.36	3.86	hypothetical protein UM93_09255 [Arthrobacter sp. IHBB 11108]
gi914715671	0.99	1	1	908	102.0	5.39	3.86	hypothetical protein [Arthrobacter sp. ZBG10]
gi937257974	3.66	1	1	383	40.8	7.23	3.86	hypothetical protein AO716_07510 [Arthrobacter sp. Edens01]
gi916782383	1.52	6	1	1584	163.8	5.26	3.85	hypothetical protein [Arthrobacter sp. 35W]

gi928988946	2.97	1	1	337	37.8	5.52	3.85	hypothetical protein [Arthrobacter sp. ERGS1:01]
gi403227891	7.09	1	1	141	15.4	10.70	3.85	hypothetical protein ARUE_c03730 [Arthrobacter sp. Rue61a]
gi307743453	6.38	1	1	298	30.6	4.94	3.85	3-hydroxyisobutyrate dehydrogenase [Arthrobacter arilaitensis Re117]
gi522097319	5.44	1	1	423	46.1	5.40	3.85	acyl-CoA dehydrogenase [Arthrobacter sp. 161MFSha2.1]
gi737800851	1.72	2	1	523	58.2	6.18	3.85	copper oxidase [Arthrobacter castelli]
gi918267806	3.04	2	1	395	42.9	6.92	3.84	GTPase HflX [Arthrobacter sp. Hiyo1]
gi939037370	14.96	1	1	127	13.3	8.21	3.84	hypothetical protein [Arthrobacter nitroguajacolicus]
gi930827097	4.10	1	1	317	34.3	8.69	3.84	metallophosphoesterase [Arthrobacter arilaitensis]
gi759730629	2.79	1	1	573	60.6	7.37	3.84	multidrug ABC transporter permease [Arthrobacter sp. L77]
gi518311056	7.63	1	1	236	26.5	7.55	3.83	hypothetical protein [Arthrobacter sp. TB 23]
gi476398902	2.20	1	1	590	62.7	5.05	3.83	acetyl-CoA carboxylase biotin-containing subunit [Arthrobacter crystallopoietes BAB-32]
gi917022157	3.04	1	1	690	76.7	6.07	3.83	alpha-1,4-glucan:maltose-1-phosphate maltosyltransferase [Arthrobacter sp. UNC362MFTsu
gi742859399	11.84	2	1	152	16.6	5.47	3.83	glutamyl-tRNA amidotransferase [Arthrobacter sp. W1]
gi476398944	2.36	1	1	423	46.0	6.71	3.82	hydroxydechloroatrazine ethylaminohydrolase [Arthrobacter crystallopoietes BAB-32]
gi542107378	4.26	1	1	305	31.2	4.83	3.82	carbohydrate kinase [Arthrobacter sp. AK-YN10]
gi476398858	3.37	1	1	386	42.1	5.74	3.82	putative glutaryl-CoA dehydrogenase [Arthrobacter crystallopoietes BAB-32]
gi652425293	10.06	1	1	179	19.2	6.37	3.82	RecX family transcriptional regulator [Arthrobacter castelli]
gi654819062	6.05	1	1	248	26.8	6.96	3.82	hypothetical protein [Arthrobacter sp. UNC362MFTsu5.1]
gi639128931	2.99	1	1	334	37.1	10.07	3.82	hypothetical protein [Arthrobacter sp. CAL618]
gi116608934	5.75	4	1	313	33.1	7.52	3.81	modification methylase, HemK family [Arthrobacter sp. FB24]
gi359306489	3.76	1	1	213	22.5	5.68	3.81	ribosomal RNA small subunit methyltransferase G [Arthrobacter globiformis NBRC 12137]
gi910249764	2.16	3	1	555	59.5	7.97	3.81	two-component system sensor histidine kinase [Arthrobacter siccitolerans]
gi910742004	2.78	1	1	539	55.7	6.23	3.81	putative HMP/thiamine import ATP-binding protein YkoD [Arthrobacter sp. Hiyo4]
gi937259228	4.46	1	1	314	34.5	5.60	3.81	ABC transporter ATP-binding protein [Arthrobacter sp. Edens01]
gi162953380	1.85	1	1	378	40.7	5.96	3.81	RmuC family protein [Renibacterium salmoninarum ATCC 33209]
gi307743705	2.98	1	1	403	42.1	5.77	3.81	acetyl-CoA C-acyltransferase [Arthrobacter arilaitensis Re117]
gi917759887	4.00	1	1	450	49.5	7.94	3.80	hypothetical protein [Arthrobacter sp. L77]
gi651453543	7.36	2	1	231	25.3	5.16	3.80	GntR family transcriptional regulator [Arthrobacter nicotinovorans]
gi654819570	6.22	1	1	193	21.0	4.97	3.80	hypothetical protein [Arthrobacter sp. UNC362MFTsu5.1]
gi517603993	2.20	1	1	681	75.8	6.06	3.80	alpha-1,4-glucan--maltose-1-phosphate maltosyltransferase [Arthrobacter sp. 131MFCol6.1
gi742852031	2.17	1	1	831	86.3	4.93	3.80	hypothetical protein [Arthrobacter sp. W1]
gi640193741	6.45	1	1	248	27.4	5.22	3.80	hypothetical protein [Arthrobacter sp. 31Y]
gi517598576	1.37	1	1	658	71.1	6.95	3.80	amino acid transporter [Arthrobacter sp. 162MFSha1.1]
gi517601627	4.48	1	1	402	44.0	5.34	3.79	methionine synthase [Arthrobacter sp. 162MFSha1.1]
gi764161638	2.71	1	1	517	56.2	5.94	3.78	recombinational DNA repair ATPase [Arthrobacter phage vB_ArtM-ArV1]
gi823665446	3.90	1	1	154	17.6	6.30	3.78	hypothetical protein AA310_18525 [Arthrobacter sp. YC-RL1]
gi917441900	6.55	1	1	275	27.9	6.70	3.78	hypothetical protein [Arthrobacter albus]
gi651489694	5.61	1	1	196	22.0	8.16	3.78	hypothetical protein [Arthrobacter sp. H20]
gi517601247	4.45	1	1	292	33.0	6.57	3.78	hypothetical protein [Arthrobacter sp. 162MFSha1.1]
gi651498726	4.66	2	1	365	38.7	5.08	3.78	aspartate aminotransferase [Arthrobacter sp. 35W]
gi823668473	14.63	1	1	41	4.9	6.67	3.78	transposase, partial [Arthrobacter sp. YC-RL1]
gi937258040	2.69	1	1	594	62.6	5.14	3.77	acetyl-/propionyl-CoA carboxylase subunit alpha [Arthrobacter sp. Edens01]
gi517609870	3.49	1	1	544	58.4	5.21	3.77	hypothetical protein [Arthrobacter sp. 161MFSha2.1]
gi759715510	2.75	6	1	472	51.5	5.03	3.77	diguanylate phosphodiesterase, partial [Arthrobacter sp. AK-YN10]
gi551256474	3.07	1	1	293	31.8	5.66	3.76	ABC transporter ATP-binding protein [Arthrobacter sp. PAO19]
gi359304805	5.70	1	1	456	47.9	10.81	3.76	hypothetical protein ARGLB_080_01200 [Arthrobacter globiformis NBRC 12137]
gi443479610	3.00	1	1	200	20.6	5.10	3.75	aspartyl/glutamyl-tRNA amidotransferase subunit A, partial [Arthrobacter nitrophenolicus]
gi635350179	4.18	1	1	287	31.3	6.46	3.75	putative spermidine synthase [Arthrobacter siccitolerans]
gi651484600	6.20	1	1	258	28.1	5.83	3.75	glutamine ABC transporter ATP-binding protein [Arthrobacter sp. Br18]
gi937258240	2.70	3	1	296	31.0	5.01	3.74	pantoate--beta-alanine ligase [Arthrobacter sp. Edens01]
gi928487038	2.82	1	1	497	51.5	5.58	3.74	aldehyde dehydrogenase [Arthrobacter alpinus]
gi918265940	5.63	1	1	320	34.7	4.93	3.74	uncharacterized oxidoreductase YccK [Arthrobacter sp. Hiyo1]
gi674647037	4.00	2	1	350	38.0	6.47	3.74	AFG1-like ATPase [Arthrobacter sp. 11W110_air]
gi470221009	1.90	1	1	686	73.2	10.05	3.73	hypothetical protein ADIAG_00958 [Arthrobacter gangotriensis Lz1y]
gi651430502	2.08	1	1	289	32.3	9.20	3.73	sugar ABC transporter permease [Arthrobacter sanguinis]
gi914715599	2.64	1	1	531	52.0	8.13	3.73	bifunctional ADP-dependent (S)-NAD(P)H-hydrate dehydratase/NAD(P)H-hydrate epimerase
gi908697950	9.68	5	1	217	23.2	11.08	3.73	hypothetical protein [Arthrobacter sp. RIT-PI-e]
gi916871975	4.34	1	1	369	39.6	5.91	3.73	glycine/betaine ABC transporter ATP-binding protein [Arthrobacter sp. H5]

gi189038078	1.22	1	1	655	73.0	6.87	3.72	RecName: Full=UvrABC system protein C; Short=Protein UvrC; AltName: Full=Excinuclease
gi323467988	3.41	1	1	352	37.8	8.97	3.71	nucleoside-diphosphate-sugar epimerase [Arthrobacter phenanthrenivorans Sphe3]
gi930828102	3.70	1	1	324	37.3	8.92	3.71	glycosyl transferase [Arthrobacter arilaitensis]
gi654823147	3.23	1	1	372	39.7	6.95	3.71	aminotransferase [Arthrobacter sp. I3]
gi515766266	3.23	1	1	619	68.4	5.71	3.71	glucoamylase [Arthrobacter sp. M2012083]
gi651459068	3.55	1	1	197	21.0	11.52	3.71	rhomboid family intramembrane serine protease [Arthrobacter sp. 35/47]
gi211590064	2.85	1	1	492	54.1	5.35	3.71	Pc21g13350 [Penicillium rubens Wisconsin 54-1255]
gi470221608	2.96	1	1	473	52.3	5.94	3.70	hypothetical protein ADIAG_00461 [Arthrobacter gangotriensis Lz1y]
gi476401650	0.97	1	1	725	74.7	5.94	3.70	hypothetical protein D477_007519, partial [Arthrobacter crystallopoietes BAB-32]
gi917013123	3.34	1	1	419	45.5	4.96	3.70	hypothetical protein [Arthrobacter sanguinis]
gi919218766	4.04	1	1	297	33.4	9.09	3.70	hypothetical protein [Arthrobacter sp. YC-RL1]
gi219860735	4.89	1	1	225	23.7	8.69	3.70	protein of unknown function DUF624 [Arthrobacter chlorophenolicus A6]
gi476401226	12.70	1	1	126	14.3	6.58	3.70	hypothetical protein D477_009775 [Arthrobacter crystallopoietes BAB-32]
gi476401993	2.33	1	1	516	56.1	5.87	3.69	GTP-binding protein [Arthrobacter crystallopoietes BAB-32]
gi915933657	8.00	1	1	225	21.6	9.96	3.69	hypothetical protein [Arthrobacter globiformis]
gi767258892	6.72	1	1	119	13.1	8.56	3.69	membrane protein [Arthrobacter sp. IHBB 11108]
gi908698854	3.02	1	1	430	46.6	6.57	3.69	exodeoxyribonuclease VII large subunit [Arthrobacter sp. RIT-PI-e]
gi517593247	4.30	1	1	279	28.8	5.66	3.68	citrate lyase subunit beta [Arthrobacter sp. 135MFCol5.1]
gi651439920	1.71	1	1	467	52.0	6.89	3.68	hypothetical protein [Arthrobacter sp. H14]
gi767258567	2.50	1	1	721	77.0	5.15	3.68	3-hydroxyacyl-CoA dehydrogenase [Arthrobacter sp. IHBB 11108]
gi654811293	1.59	1	1	566	62.3	6.74	3.67	hypothetical protein [Arthrobacter sp. MA-N2]
gi908697037	5.42	1	1	203	21.7	4.49	3.67	thiol-disulfide isomerase [Arthrobacter sp. RIT-PI-e]
gi403230592	3.77	1	1	239	26.5	5.82	3.65	putative manganese transport protein MntH [Arthrobacter sp. Rue61a]
gi636846955	5.69	2	1	246	26.7	8.75	3.65	hypothetical protein [Arthrobacter sp. TB 26]
gi162955679	10.83	1	1	157	16.7	10.70	3.65	DedA family protein# [Renibacterium salmoninarum ATCC 33209]
gi910738812	2.02	1	1	446	47.3	4.53	3.64	acetyl-/propionyl-coenzyme A carboxylase alpha chain [Arthrobacter sp. Hiyo4]
gi651458056	1.23	1	1	649	71.8	5.08	3.64	peptidase M13 [Arthrobacter sp. 35/47]
gi674645442	7.50	1	1	280	28.0	5.11	3.64	Glucose 1-dehydrogenase 2 [Arthrobacter sp. 11W110_air]
gi476402489	3.22	1	1	342	34.7	5.27	3.64	glutamate-1-semialdehyde aminotransferase [Arthrobacter crystallopoietes BAB-32]
gi517589808	1.79	1	1	336	35.1	6.14	3.64	hypothetical protein [Arthrobacter sp. 135MFCol5.1]
gi767259106	3.28	1	1	305	31.1	5.29	3.63	porphobilinogen deaminase [Arthrobacter sp. IHBB 11108]
gi910250480	7.25	2	1	207	22.7	5.24	3.63	DNA-binding response regulator [Arthrobacter siccitolerans]
gi918269275	7.66	1	1	235	25.5	6.28	3.62	conserved hypothetical protein [Arthrobacter sp. Hiyo1]
gi737807866	2.72	1	1	404	42.2	4.72	3.62	pyridine nucleotide-disulfide oxidoreductase [Arthrobacter sp. H5]
gi323468725	3.94	1	1	609	64.5	5.91	3.62	AMP-forming long-chain acyl-CoA synthetase [Arthrobacter phenanthrenivorans Sphe3]
gi648259933	1.83	1	1	872	96.6	5.07	3.61	valine--tRNA ligase [Arthrobacter sp. TB 23]
gi640196783	4.82	1	1	394	42.8	6.81	3.61	GntR family transcriptional regulator [Arthrobacter sp. 31Y]
gi918267558	12.20	1	1	164	17.8	7.05	3.61	organic hydroperoxide resistance transcriptional regulator [Arthrobacter sp. Hiyo1]
gi116610641	2.78	1	1	360	38.8	5.62	3.60	transcriptional regulator, LacI family [Arthrobacter sp. FB24]
gi219862014	4.08	1	1	147	16.6	11.72	3.60	hypothetical protein Achl_4404 (plasmid) [Arthrobacter chlorophenolicus A6]
gi910696719	6.88	4	1	218	24.1	11.28	3.60	uncharacterized HTH-type transcriptional regulator YagI, partial [Arthrobacter sp. Hiyo6]
gi542110290	0.65	1	1	1073	110.9	7.28	3.60	hydrogenase expression protein [Arthrobacter sp. AK-YN10]
gi917013484	3.60	1	1	361	39.4	5.60	3.60	ATP-dependent DNA helicase RuvB [Arthrobacter sanguinis]
gi443482932	6.23	1	1	305	31.9	5.64	3.59	nicotinate-nucleotide pyrophosphorylase [Arthrobacter nitrophenolicus]
gi403229371	4.16	1	1	409	45.7	5.12	3.59	putative cytochrome P450 [Arthrobacter sp. Rue61a]
gi470221262	7.52	2	1	226	23.6	4.88	3.58	thiamine-phosphate pyrophosphorylase [Arthrobacter gangotriensis Lz1y]
gi403230606	4.79	1	1	334	37.8	10.15	3.58	hypothetical protein ARUE_c31440 [Arthrobacter sp. Rue61a]
gi651501204	5.19	2	1	347	37.9	5.26	3.58	luciferase [Arthrobacter sp. 35W]
gi551256017	1.63	1	1	492	52.0	6.39	3.58	MFS transporter [Arthrobacter sp. PAO19]
gi759731474	6.88	1	1	247	25.4	8.98	3.58	ArsR family transcriptional regulator [Arthrobacter sp. L77]
gi674645804	2.21	1	1	272	30.3	9.20	3.58	Teichoic acid translocation permease protein TagG [Arthrobacter sp. 11W110_air]
gi927296379	2.72	1	1	441	47.7	4.87	3.58	hypothetical protein AL755_13925 [Arthrobacter sp. ERGS1:01]
gi162953314	3.91	1	1	307	32.0	7.02	3.58	Mg chelatase-related protein [Renibacterium salmoninarum ATCC 33209]
gi651434955	1.07	1	1	654	71.6	5.34	3.58	acetyl-coenzyme A synthetase [Arthrobacter sp. H41]
gi916259848	1.58	1	1	442	48.5	7.09	3.57	MULTISPECIES: hypothetical protein [Arthrobacter]
gi910696263	2.27	1	1	308	34.5	9.29	3.57	multifunctional 2-oxoglutarate metabolism enzyme [Arthrobacter sp. Hiyo6]
gi652423642	5.88	1	1	357	37.8	5.17	3.56	alkene reductase [Arthrobacter castelli]
gi162954901	3.03	1	1	231	24.8	8.79	3.56	putative CAAX amino terminal protease family [Renibacterium salmoninarum ATCC 33209]

gi651479080	4.56	1	1	307	33.4	8.63	3.55	hypothetical protein, partial [Arthrobacter sp. Br18]
gi742859424	2.02	1	1	494	54.7	4.96	3.55	mannitol dehydrogenase [Arthrobacter sp. W1]
gi443480962	6.63	1	1	181	19.3	9.76	3.55	hypothetical protein G205_15180 [Arthrobacter nitrophenolicus]
gi908740357	8.93	1	1	168	18.7	7.62	3.54	GNAT family N-acetyltransferase [Arthrobacter arilaitensis]
gi517590825	5.40	1	1	389	43.3	8.81	3.54	fatty acid desaturase [Arthrobacter sp. 135MFCol5.1]
gi917442012	5.67	2	1	335	35.7	5.24	3.53	prephenate dehydratase [Arthrobacter albus]
gi654811541	4.12	1	1	243	26.9	8.98	3.53	hypothetical protein [Arthrobacter sp. MA-N2]
gi443480906	2.64	2	1	832	88.0	5.05	3.53	beta-glucosidase-like glycosyl hydrolase [Arthrobacter nitrophenolicus]
gi908690378	5.93	1	1	270	29.4	6.02	3.53	spermidine synthase [Arthrobacter sp. H41]
gi219861654	0.81	1	1	867	94.7	6.05	3.52	type III restriction protein res subunit (plasmid) [Arthrobacter chlorophenolicus A6]
gi359303616	2.86	1	1	315	33.0	9.79	3.52	TDT family transporter [Arthrobacter globiformis NBRC 12137]
gi654825710	2.15	1	1	699	72.6	4.59	3.50	peptidase [Arthrobacter sp. H5]
gi476400350	8.54	1	1	199	22.9	5.67	3.50	Low molecular weight phosphotyrosine protein phosphatase [Arthrobacter crystallopoietes
gi651444043	11.11	1	1	162	17.5	5.33	3.49	PTS fructose transporter subunit IIA [Arthrobacter nicotinovorans]
gi910744347	3.54	1	1	339	35.6	5.49	3.49	uncharacterized protein HI_0568 [Arthrobacter sp. Hiyo8]
gi786028535	6.60	2	1	288	29.8	5.20	3.49	protein-(glutamine-N5) methyltransferase, release factor-specific [Arthrobacter chloropheno
gi674644991	3.55	1	1	366	37.5	9.61	3.48	UDP-N-acetylglucosamine--N-acetylmuramyl-(pentapeptide) pyrophosphoryl-undecaprenol
gi651502162	3.43	1	1	350	37.2	5.33	3.47	D-3-phosphoglycerate dehydrogenase [Arthrobacter sp. 35W]
gi674644086	2.20	1	1	499	55.2	6.79	3.47	hypothetical protein BN1051_00228 [Arthrobacter sp. 11W110_air]
gi910737956	5.91	1	1	203	22.5	6.05	3.47	transcriptional regulator protein [Arthrobacter sp. Hiyo4]
gi654826692	7.31	1	1	342	37.2	8.85	3.47	cytochrome C biogenesis protein [Arthrobacter sp. H5]
gi651443984	20.59	1	1	68	7.3	9.82	3.47	50S ribosomal protein L30 [Arthrobacter nicotinovorans]
gi359307433	3.73	1	1	295	29.9	4.59	3.47	hypothetical protein ARGLB_014_00070 [Arthrobacter globiformis NBRC 12137]
gi651499610	2.57	1	1	545	58.3	5.81	3.46	PucR family transcriptional regulator [Arthrobacter sp. 35W]
gi219860492	4.31	2	1	232	24.9	5.33	3.46	two component transcriptional regulator, winged helix family [Arthrobacter chlorophenolicu
gi476399791	3.01	2	1	565	61.5	6.77	3.46	CTP synthetase [Arthrobacter crystallopoietes BAB-32]
gi672940882	4.57	4	1	350	37.2	5.12	3.46	recombination protein RecA [Pimelobacter simplex]
gi403227591	1.86	2	1	323	34.7	4.98	3.45	D-3-phosphoglycerate dehydrogenase SerA [Arthrobacter sp. Rue61a]
gi917013478	1.92	1	1	626	65.9	7.39	3.45	preprotein translocase subunit SecD [Arthrobacter sanguinis]
gi654816667	2.31	1	1	346	36.9	5.58	3.44	LacI family transcriptional regulator [Arthrobacter sp. UNC362MFTsu5.1]
gi742756026	2.23	1	1	539	61.1	7.65	3.43	deoxyribodipyrimidine photolyase [Arthrobacter phenanthrenivorans]
gi654818629	2.25	1	1	267	28.3	5.52	3.38	hypothetical protein [Arthrobacter sp. UNC362MFTsu5.1]
gi651460825	7.41	1	1	135	14.6	4.50	3.38	glyoxalase [Arthrobacter sp. 35/47]
gi910694579	27.87	2	1	61	6.7	9.92	3.38	hypothetical protein AHiyo6_25420 [Arthrobacter sp. Hiyo6]
gi518311575	1.18	1	1	507	55.4	5.25	3.35	aldehyde dehydrogenase [Arthrobacter sp. TB 23]
gi654823265	8.24	1	1	279	28.4	5.15	3.35	hypothetical protein [Arthrobacter sp. I3]
gi323471007	7.81	1	1	256	26.3	7.47	3.34	ABC-type dipeptide/oligopeptide/nickel transport system, ATPase component [Arthrobacter
gi651507514	5.82	3	1	275	29.6	4.72	3.32	hypothetical protein [Arthrobacter sp. 35W]
gi636847335	1.64	1	1	427	47.7	5.85	3.31	cytochrome P450 [Arthrobacter sp. TB 26]
gi737813418	2.87	2	1	488	52.4	6.33	3.27	betaine-aldehyde dehydrogenase [Arthrobacter sp. H14]
gi651466083	8.22	1	1	146	15.4	4.88	3.26	acyl dehydratase [Arthrobacter sp. 35/47]
gi767259236	1.66	1	1	422	46.8	5.68	3.25	queuine tRNA-ribosyltransferase [Arthrobacter sp. IHBB 11108]
gi767257980	1.72	1	1	407	42.3	7.06	3.24	sulfonate ABC transporter substrate-binding protein [Arthrobacter sp. IHBB 11108]
gi654812369	9.39	2	1	213	22.9	5.00	3.19	NADP oxidoreductase [Arthrobacter sp. MA-N2]
gi916814556	8.64	3	1	162	16.7	4.61	3.17	hypothetical protein [Arthrobacter nicotinovorans]
gi939051674	12.37	3	1	97	10.5	6.77	3.17	hypothetical protein [Arthrobacter sp. JCM 19049]
gi765006934	6.40	1	1	297	32.1	6.90	3.16	hypothetical protein [Arthrobacter sp. A3]
gi651442322	7.75	4	1	271	29.5	5.45	3.14	ubiquinone biosynthesis methyltransferase UbiE [Arthrobacter sp. 9MFCol3.1]
gi403229116	5.16	1	1	252	26.4	5.85	3.07	hypothetical protein ARUE_c16260 [Arthrobacter sp. Rue61a]
gi651494084	3.36	1	1	476	51.5	9.14	3.06	transposase [Arthrobacter sp. H20]
gi928488353	7.27	4	1	275	30.0	4.09	3.06	hypothetical protein AOC05_17425 [Arthrobacter alpinus]
gi927296325	3.86	1	1	466	50.8	5.53	3.04	FAD-dependent oxidoreductase [Arthrobacter sp. ERGS1:01]
gi636846706	10.00	1	1	170	17.9	7.01	3.04	universal stress protein UspA [Arthrobacter sp. TB 26]
gi640197103	14.16	1	1	113	12.1	4.96	3.03	5-hydroxyisourate hydrolase [Arthrobacter sp. 31Y]
gi651487277	17.65	2	1	102	11.8	5.86	3.03	hypothetical protein, partial [Arthrobacter sp. Br18]
gi359304909	13.86	4	1	101	11.1	10.05	3.02	50S ribosomal protein L23 [Arthrobacter globiformis NBRC 12137]
gi476401923	2.23	2	1	629	66.6	5.77	3.01	ABC transporter [Arthrobacter crystallopoietes BAB-32]
gi518311145	4.56	2	1	461	46.9	9.57	3.01	MULTISPECIES: hypothetical protein [Arthrobacter]

gi640200226	2.75	3	1	472	51.8	5.73	3.00	diguanylate phosphodiesterase [Arthrobacter sp. 31Y]
gi910693892	3.31	3	1	483	52.6	4.97	2.99	NADPH-ferredoxin reductase FprA [Arthrobacter sp. Hiyo6]
gi695200271	4.04	1	1	421	45.8	6.13	2.99	unknown (plasmid) [Arthrobacter keyseri]
gi654827190	7.35	1	1	136	14.9	4.77	2.94	lyase [Arthrobacter sp. H5]
gi652425744	4.18	1	1	335	35.9	5.11	2.93	hydroxylase [Arthrobacter castelli]
gi742851125	2.78	4	1	863	90.6	6.98	2.93	hydrolase [Arthrobacter sp. W1]
gi489901154	5.98	2	1	301	32.2	5.05	2.92	5-deoxy-glucuronate isomerase [Arthrobacter globiformis]
gi723607174	7.88	1	1	292	29.0	5.78	2.90	orotidine 5'-phosphate decarboxylase [Arthrobacter sp. PAMC25486]
gi918221940	3.68	1	1	516	51.7	11.80	2.90	competence protein ComEC [Arthrobacter sp. I3]
gi910742161	20.21	4	1	94	10.3	11.50	2.89	hypothetical protein AHiyo4_49830 [Arthrobacter sp. Hiyo4]
gi928487606	3.79	1	1	448	46.9	5.14	2.89	phosphoglucosamine mutase [Arthrobacter alpinus]
gi759771883	15.20	8	1	125	14.0	7.93	2.88	electron transporter [Arthrobacter sp. SPG23]
gi674645813	4.65	2	1	430	43.3	5.78	2.87	antiporter inner membrane protein [Arthrobacter sp. 11W110_air]
gi651441604	6.98	5	1	258	28.3	8.62	2.87	arginine ABC transporter ATP-binding protein [Arthrobacter sp. 9MFCol3.1]
gi769943104	5.70	3	1	316	31.3	4.74	2.87	ribokinase [Arthrobacter sp. IHBB 11108]
gi359304287	8.30	1	1	229	23.9	6.01	2.86	putative SufR family transcriptional regulator [Arthrobacter globiformis NBRC 12137]
gi910748131	10.22	4	1	137	14.7	5.29	2.86	ribonuclease 3 [Arthrobacter sp. Hiyo8]
gi517599455	1.92	2	1	781	86.4	5.24	2.86	kojibiose phosphorylase [Arthrobacter sp. 162MFSha1.1]
gi737809633	5.28	2	1	303	32.6	4.60	2.85	serine/threonine protein phosphatase [Arthrobacter sp. H5]
gi918269021	6.97	1	1	244	27.0	8.16	2.85	uncharacterized protein YndH [Arthrobacter sp. Hiyo1]
gi654813361	10.06	1	1	169	19.3	6.93	2.85	DNA-directed RNA polymerase sigma-70 factor [Arthrobacter sp. MA-N2]
gi323470419	6.39	1	1	266	27.8	5.19	2.85	hypothetical protein Asphe3_29930 [Arthrobacter phenanthrenivorans Sphe3]
gi916259925	5.07	2	1	276	29.4	10.59	2.84	MULTISPECIES: nitrate ABC transporter permease [Arthrobacter]
gi476401141	7.10	1	1	169	18.8	9.96	2.84	hypothetical protein D477_010271 [Arthrobacter crystallopoietes BAB-32]
gi910251467	8.54	2	1	164	18.2	5.73	2.84	GCN5 family acetyltransferase [Arthrobacter siccitolerans]
gi937256430	13.71	1	1	124	13.2	9.55	2.84	hypothetical protein AO716_15275 [Arthrobacter sp. Edens01]
gi476398924	9.09	2	1	220	23.9	6.65	2.83	response regulator of citrate/malate metabolism [Arthrobacter crystallopoietes BAB-32]
gi916835131	3.69	3	1	352	37.5	9.04	2.83	nickel transporter [Arthrobacter sp. H14]
gi116611764	11.64	2	1	146	15.6	5.96	2.82	conserved hypothetical protein [Arthrobacter sp. FB24]
gi910743292	19.66	2	1	117	11.7	5.36	2.82	PTS system glucose-specific EIICBA component [Arthrobacter sp. Hiyo8]
gi928488025	4.43	1	1	429	45.9	6.74	2.82	histidine kinase [Arthrobacter alpinus]
gi760124521	4.53	1	1	375	40.7	5.50	2.82	hypothetical protein [Arthrobacter aurescens]
gi517599768	10.45	1	1	134	14.4	7.59	2.81	sodium:proton antiporter [Arthrobacter sp. 162MFSha1.1]
gi737807523	7.98	2	1	263	28.1	7.56	2.81	ArsR family transcriptional regulator [Arthrobacter sp. H5]
gi470215926	13.16	2	1	76	8.2	7.08	2.81	hypothetical protein ADIAG_03887 [Arthrobacter gangotriensis Lz1y]
gi917530436	7.95	3	1	151	16.0	4.78	2.81	FHA domain-containing protein [Arthrobacter sp. PAMC25486]
gi742858572	9.14	1	1	175	19.2	4.31	2.80	RNA-binding protein [Arthrobacter sp. W1]
gi910743092	5.16	1	1	349	36.9	8.85	2.80	xanthan lyase [Arthrobacter sp. Hiyo8]
gi737789911	3.64	1	1	577	61.2	4.69	2.80	DNA repair protein RecN [Arthrobacter albus]
gi823667881	5.32	3	1	376	39.7	4.94	2.80	mannose-1-phosphate guanylyltransferase [Arthrobacter sp. YC-RL1]
gi219859201	2.12	4	1	707	73.8	6.15	2.80	serine/threonine protein kinase with PASTA sensor(s) [Arthrobacter chlorophenolicus A6]
gi651503901	25.00	1	1	96	10.1	7.44	2.80	hypothetical protein [Arthrobacter sp. 35W]
gi651492630	5.29	1	1	208	22.9	6.55	2.79	hypothetical protein [Arthrobacter sp. H20]
gi765010274	13.86	3	1	101	11.2	10.11	2.79	50S ribosomal protein L23 [Arthrobacter sp. A3]
gi443481833	4.89	1	1	348	38.8	5.26	2.79	5-methyltetrahydropteroyltriglutamate--homocysteine methyltransferase [Arthrobacter nitro
gi757624149	1.11	1	1	1166	126.9	6.87	2.79	DNA polymerase III subunit alpha [Arthrobacter sp. SPG23]
gi916872086	16.94	1	1	124	13.3	10.95	2.78	hypothetical protein [Arthrobacter sp. H5]
gi765008297	7.75	1	1	258	27.9	4.93	2.78	hypothetical protein [Arthrobacter sp. A3]
gi651436289	8.55	1	1	152	16.9	10.56	2.77	hypothetical protein [Arthrobacter sp. H41]
gi767257438	5.24	1	1	229	25.5	9.70	2.77	membrane protein [Arthrobacter sp. IHBB 11108]
gi652423979	14.12	1	1	177	19.3	4.78	2.77	hypothetical protein [Arthrobacter castelli]
gi742851573	9.39	5	1	213	22.4	4.63	2.77	septum formation inhibitor Maf [Arthrobacter sp. W1]
gi651429242	1.98	1	1	808	86.0	5.44	2.77	hypothetical protein [Arthrobacter sanguinis]
gi910745062	18.37	1	1	98	10.6	11.88	2.76	hypothetical protein AHiyo8_27640 [Arthrobacter sp. Hiyo8]
gi219859319	13.79	2	1	87	9.6	4.67	2.76	phosphoribosyl-ATP diphosphatase [Arthrobacter chlorophenolicus A6]
gi652422560	5.11	1	1	235	24.8	9.25	2.76	hypothetical protein [Arthrobacter castelli]
gi651437903	7.21	1	1	222	23.7	9.85	2.76	50S ribosomal protein L4 [Arthrobacter sp. H14]
gi767257237	13.83	1	1	94	10.3	9.72	2.76	hypothetical protein UM93_05565 [Arthrobacter sp. IHBB 11108]

gi916871563	3.90	1	1	410	44.3	8.69	2.75	DNA polymerase IV [Arthrobacter sp. H5]
gi116612272	4.89	2	1	307	32.9	6.40	2.75	transcriptional regulator, LysR family [Arthrobacter sp. FB24]
gi551253849	2.70	1	1	444	48.5	4.72	2.75	sugar-binding protein [Arthrobacter sp. PAO19]
gi162953052	3.62	1	1	359	37.5	9.32	2.75	hypothetical protein RSal33209_0823 [Renibacterium salmoninarum ATCC 33209]
gi476401746	5.06	7	1	395	43.4	6.99	2.74	chromosome segregation protein SMC, partial [Arthrobacter crystallopoietes BAB-32]
gi651447798	8.75	1	1	263	26.7	5.63	2.74	3-hydroxy-2-methylbutyryl-CoA dehydrogenase [Arthrobacter nicotinovorans]
gi517598891	2.80	1	1	322	35.2	8.82	2.74	NUDIX hydrolase [Arthrobacter sp. 162MFSha1.1]
gi443482366	21.84	2	1	87	9.5	6.05	2.74	hypothetical protein G205_06173 [Arthrobacter nitrophenolicus]
gi910738469	6.15	1	1	260	27.2	5.60	2.73	alanine racemase [Arthrobacter sp. Hiyo4]
gi916876262	4.09	1	1	318	33.6	5.94	2.73	antibiotic ABC transporter ATP-binding protein [Arthrobacter sp. 31Y]
gi518313786	3.25	2	1	338	36.0	8.07	2.73	MULTISPECIES: tRNA(Ile)-lysidine synthetase [Arthrobacter]
gi737812921	6.12	1	1	278	30.3	9.14	2.73	ABC transporter [Arthrobacter sp. H14]
gi765005658	3.74	1	1	321	35.3	5.05	2.73	proline iminopeptidase [Arthrobacter sp. A3]
gi162954511	9.49	1	1	137	14.5	7.23	2.72	hypothetical protein RSal33209_2295 [Renibacterium salmoninarum ATCC 33209]
gi918268043	4.51	1	1	399	42.6	9.33	2.72	high-affinity branched-chain amino acid transport system permease protein BraE [Arthroba
gi443481047	30.56	1	1	36	3.6	8.66	2.72	30S ribosomal protein S8 [Arthrobacter nitrophenolicus]
gi518312167	1.61	1	1	1241	136.6	5.21	2.72	hypothetical protein [Arthrobacter sp. TB 23]
gi908699601	4.41	1	1	340	36.5	5.41	2.72	O-succinylbenzoate synthase [Arthrobacter sp. RIT-PI-e]
gi551256448	9.62	1	1	260	28.5	5.10	2.71	SAM-dependent methyltransferase [Arthrobacter sp. PAO19]
gi652425626	5.25	1	1	438	46.7	4.97	2.71	hypothetical protein [Arthrobacter castelli]
gi914717314	4.91	4	1	346	37.5	4.96	2.71	sialic acid transporter [Arthrobacter sp. ZBG10]
gi723609321	7.08	3	1	339	35.9	6.25	2.71	Transcriptional regulator, LacI family [Arthrobacter sp. PAMC25486]
gi737809913	5.29	3	1	170	19.2	11.25	2.71	hypothetical protein [Arthrobacter sp. 35/47]
gi654828015	3.62	1	1	276	31.0	5.43	2.70	hypothetical protein [Arthrobacter sp. H5]
gi823667348	6.52	1	1	184	19.9	5.33	2.70	Cro/Cl family transcriptional regulator [Arthrobacter sp. YC-RL1]
gi742851499	2.67	1	1	637	65.6	4.64	2.70	penicillin-binding protein [Arthrobacter sp. W1]
gi654816495	8.99	3	1	178	19.2	10.14	2.70	hypothetical protein [Arthrobacter sp. UNC362MFTsu5.1]
gi651496166	4.93	2	1	345	37.2	8.24	2.70	epimerase [Arthrobacter sp. H20]
gi635350882	2.19	1	1	503	56.7	5.68	2.69	putative ATP-binding protein [Arthrobacter siccitolerans]
gi916834538	3.93	1	1	433	47.6	5.36	2.69	hypothetical protein [Arthrobacter sp. H14]
gi470220102	8.85	1	1	192	20.5	5.21	2.68	N-acetyltransferase GCN5 [Arthrobacter gangotriensis Lz1y]
gi914715474	5.58	3	1	251	27.9	7.25	2.68	glycosyl transferase [Arthrobacter sp. ZBG10]
gi403230605	7.69	2	1	364	38.8	4.91	2.68	aspartate-semialdehyde dehydrogenase Asd [Arthrobacter sp. Rue61a]
gi651448426	8.05	1	1	236	24.8	9.26	2.68	ABC transporter ATP-binding protein [Arthrobacter nicotinovorans]
gi737796655	1.92	2	1	416	42.4	4.77	2.68	phosphoglycerate kinase [Arthrobacter sp. H20]
gi323470866	1.78	2	1	956	98.1	9.96	2.68	ATPase component of various ABC-type transport systems with duplicated ATPase domain
gi635352573	5.01	3	1	359	37.9	7.55	2.68	conserved hypothetical protein [Arthrobacter siccitolerans]
gi654818220	7.74	1	1	310	33.7	4.97	2.67	phenylacetate-CoA oxygenase [Arthrobacter sp. UNC362MFTsu5.1]
gi116612639	4.50	1	1	222	23.6	5.17	2.67	hypothetical protein Arth_3988 [Arthrobacter sp. FB24]
gi443482228	4.38	5	1	525	55.2	5.69	2.67	signal transduction histidine kinase regulating citrate/malate metabolism [Arthrobacter nitro
gi307744885	13.45	1	1	119	13.5	10.42	2.67	hypothetical protein AARI_16430 [Arthrobacter arilaitensis Re117]
gi939051134	12.18	1	1	156	16.8	7.25	2.66	hypothetical protein [Arthrobacter sp. JCM 19049]
gi651443636	2.63	1	1	495	52.2	4.94	2.66	hypothetical protein [Arthrobacter sp. 9MFCol3.1]
gi908740319	3.74	1	1	561	62.9	6.09	2.66	hypothetical protein [Arthrobacter arilaitensis]
gi918265001	23.61	1	1	72	7.5	7.40	2.66	conserved hypothetical protein, partial [Arthrobacter sp. Hiyo1]
gi307743832	5.99	4	1	367	39.1	6.74	2.66	conserved hypothetical membrane protein [Arthrobacter arilaitensis Re117]
gi723608396	7.43	2	1	377	40.2	4.91	2.66	aspartate-semialdehyde dehydrogenase [Arthrobacter sp. PAMC25486]
gi299482989	4.67	1	1	450	47.6	6.20	2.66	DFA-III-forming inulin fructotransferase [Arthrobacter aurescens]
gi651474334	3.13	2	1	416	46.5	5.06	2.66	hypothetical protein [Arthrobacter nicotinovorans]
gi927033732	3.44	3	1	465	48.8	5.58	2.66	flavoprotein [Arthrobacter sp. LS16]
gi162952515	7.21	1	1	222	24.4	5.14	2.66	transcriptional regulator, TetR family [Renibacterium salmoninarum ATCC 33209]
gi403230457	8.33	1	1	168	18.2	5.64	2.66	putative transcriptional regulator, PadR family domain protein [Arthrobacter sp. Rue61a]
gi939036467	6.16	2	1	211	22.5	8.57	2.65	TetR family transcriptional regulator [Arthrobacter nitroguajacolicus]
gi937258665	3.13	1	1	447	49.0	5.20	2.65	histidine--tRNA ligase [Arthrobacter sp. Edens01]
gi517609655	1.99	1	1	654	70.4	5.25	2.65	alpha-glycosidase [Arthrobacter sp. 161MFSha2.1]
gi517603893	11.86	1	1	177	17.4	7.06	2.65	MULTISPECIES: hypothetical protein [Arthrobacter]
gi119947489	7.28	1	1	206	23.5	10.93	2.65	ribosomal protein S4 [Arthrobacter aurescens TC1]
gi723609475	5.56	1	1	378	41.1	5.17	2.64	hypothetical protein ART_3252 [Arthrobacter sp. PAMC25486]

gi918449462	2.07	2	1	581	63.5	5.22	2.64	arginine--tRNA ligase [Arthrobacter sp. SPG23]
gi654827583	9.84	1	1	193	21.4	8.48	2.64	hypothetical protein [Arthrobacter sp. H5]
gi651447727	3.82	1	1	471	50.6	5.91	2.64	two-component system sensor histidine kinase [Arthrobacter nicotinovorans]
gi910250736	5.53	2	1	253	26.2	5.08	2.64	short-chain dehydrogenase [Arthrobacter siccitolerans]
gi640193588	6.11	2	1	360	39.7	8.50	2.64	mannosyltransferase [Arthrobacter sp. 31Y]
gi654823597	2.58	3	1	776	85.2	5.01	2.64	kojibiose phosphorylase [Arthrobacter sp. I3]
gi757625798	6.84	1	1	263	26.8	6.80	2.64	nuclear export factor GLE1 [Arthrobacter sp. SPG23]
gi723607520	9.76	2	1	123	13.6	6.32	2.63	hypothetical protein ART_1297 [Arthrobacter sp. PAMC25486]
gi542110518	5.60	2	1	250	26.1	5.06	2.63	short-chain dehydrogenase [Arthrobacter sp. AK-YN10]
gi648575002	2.81	1	1	677	67.8	4.89	2.63	penicillin-binding protein [Arthrobacter sp. 131MFCol6.1]
gi542109019	5.91	1	1	203	21.9	9.77	2.63	transposon DNA-invertase [Arthrobacter sp. AK-YN10]
gi760112853	5.56	1	1	378	40.1	4.84	2.63	esterase [Arthrobacter chlorophenolicus]
gi823667252	4.41	1	1	363	38.2	6.81	2.63	2-nitropropane dioxygenase [Arthrobacter sp. YC-RL1]
gi651458246	3.04	4	1	527	55.8	5.58	2.63	P-aminobenzoyl-glutamate transporter [Arthrobacter sp. 35/47]
gi651429594	7.28	1	1	302	33.5	6.05	2.63	DNA methyltransferase [Arthrobacter sanguinis]
gi323467956	2.99	1	1	402	42.6	5.64	2.63	DNA end-binding protein Ku [Arthrobacter phenanthrenivorans Sphe3]
gi930824964	11.96	2	1	184	18.7	4.97	2.63	TetR family transcriptional regulator [Arthrobacter arilaitensis]
gi939051042	11.92	2	1	151	15.4	11.58	2.62	ABC transporter permease, partial [Arthrobacter sp. JCM 19049]
gi916692510	12.50	1	1	152	16.2	11.90	2.62	hypothetical protein [Arthrobacter castelli]
gi476402805	10.92	2	1	119	12.7	4.28	2.62	Rieske (2Fe-2S) domain-containing protein [Arthrobacter crystallopoietes BAB-32]
gi723607788	21.13	1	1	71	8.1	9.72	2.62	L-serine ammonia-lyase [Arthrobacter sp. PAMC25486]
gi307744800	4.03	1	1	471	48.5	8.44	2.62	gluconate permease [Arthrobacter arilaitensis Re117]
gi823667624	4.98	1	1	301	32.1	9.98	2.62	hypothetical protein AA310_13305 [Arthrobacter sp. YC-RL1]
gi757625669	3.81	2	1	289	31.9	5.41	2.62	hypothetical protein TV39_04720 [Arthrobacter sp. SPG23]
gi635350630	6.28	1	1	223	24.5	6.07	2.61	putative uncharacterized protein [Arthrobacter siccitolerans]
gi916820585	5.20	1	1	269	28.8	9.60	2.61	CAAX protease [Arthrobacter sp. H20]
gi651430062	2.86	1	1	489	52.3	4.61	2.61	hypothetical protein [Arthrobacter sanguinis]
gi939050634	4.82	1	1	311	32.9	4.69	2.61	hypothetical protein [Arthrobacter sp. JCM 19049]
gi654816296	5.16	2	1	252	26.6	5.14	2.61	GCN5 family acetyltransferase [Arthrobacter sp. UNC362MFTsu5.1]
gi654812725	9.58	8	1	240	25.4	9.00	2.61	ABC transporter ATP-binding protein [Arthrobacter sp. MA-N2]
gi489902829	5.82	1	1	292	29.7	5.24	2.61	2-hydroxy-3-oxopropionate reductase [Arthrobacter globiformis]
gi910252630	4.80	1	1	479	48.8	5.36	2.61	3-carboxy-cis,cis-muconate cycloisomerase [Arthrobacter siccitolerans]
gi162955204	7.95	2	1	176	19.0	8.50	2.61	MoxR-like ATPase [Renibacterium salmoninarum ATCC 33209]
gi910740971	3.85	3	1	286	30.4	9.23	2.61	daunorubicin/doxorubicin resistance ATP-binding protein DrrA [Arthrobacter sp. Hiyo4]
gi652424748	7.32	4	1	164	18.1	9.28	2.61	HNH endonuclease [Arthrobacter castelli]
gi927032369	10.57	1	1	123	12.5	9.63	2.60	hypothetical protein AFL94_08700 [Arthrobacter sp. LS16]
gi219860459	5.71	1	1	333	34.9	5.27	2.60	Mandelate racemase/muconate lactonizing protein [Arthrobacter chlorophenolicus A6]
gi651500629	7.14	1	1	224	24.6	6.52	2.60	ArsR family transcriptional regulator [Arthrobacter sp. 35W]
gi359307703	2.09	1	1	860	92.9	5.53	2.60	putative ATP-dependent Clp protease ATP-binding subunit ClpC [Arthrobacter globiformis N
gi765009586	15.31	6	1	98	11.2	9.52	2.60	hypothetical protein [Arthrobacter sp. A3]
gi323471431	5.62	1	1	409	41.4	6.46	2.60	3-oxoacyl-(acyl-carrier-protein) synthase II (plasmid) [Arthrobacter phenanthrenivorans Sp
gi737781732	3.59	2	1	390	41.9	9.60	2.60	DNA polymerase IV [Arthrobacter sp. 35W]
gi323468508	3.21	3	1	436	45.6	6.25	2.59	hypothetical protein Asphe3_10060 [Arthrobacter phenanthrenivorans Sphe3]
gi742851455	3.32	1	1	452	48.0	4.41	2.59	sugar ABC transporter substrate-binding protein [Arthrobacter sp. W1]
gi742069802	7.38	3	1	244	27.3	8.31	2.59	hypothetical protein ANMWB30_32560 [Arthrobacter sp. MWB30]
gi928486066	3.14	1	1	350	39.6	8.27	2.59	hypothetical protein AOC05_01975 [Arthrobacter alpinus]
gi917739625	2.96	3	1	540	57.5	5.57	2.59	hypothetical protein [Arthrobacter sp. W1]
gi542107494	6.94	1	1	216	24.0	5.87	2.59	hypothetical protein M707_16275 [Arthrobacter sp. AK-YN10]
gi651482132	24.72	1	1	89	9.4	5.90	2.59	hypothetical protein [Arthrobacter sp. Br18]
gi651485358	5.38	4	1	390	41.8	5.03	2.59	acyl-CoA dehydrogenase [Arthrobacter sp. Br18]
gi910739804	11.11	2	1	126	14.0	9.63	2.59	udp-N-Acetylmuramyl tripeptide [Arthrobacter sp. Hiyo4]
gi769941746	4.49	1	1	334	35.0	5.83	2.59	hypothetical protein [Arthrobacter sp. IHBB 11108]
gi545109604	7.32	1	1	205	22.4	5.39	2.59	hypothetical protein [Arthrobacter sp. AK-YN10]
gi651443803	8.55	1	1	234	25.6	5.86	2.59	hypothetical protein [Arthrobacter sp. 9MFCol3.1]
gi476402298	3.16	1	1	507	53.6	7.39	2.58	glycine/D-amino acid oxidase, deaminating [Arthrobacter crystallopoietes BAB-32]
gi765012005	6.88	3	1	189	20.6	6.54	2.58	3-methyladenine DNA glycosylase [Arthrobacter sp. A3]
gi654814467	4.48	1	1	290	31.2	6.71	2.58	phosphonate ABC transporter [Arthrobacter sp. MA-N2]
gi910696260	7.39	1	1	230	25.2	9.89	2.58	hypothetical protein AHiyo6_13230 [Arthrobacter sp. Hiyo6]

gi116608960	23.85	5	1	109	11.3	5.34	2.58	anti-sigma-factor antagonist [Arthrobacter sp. FB24]
gi517598421	4.73	1	1	317	32.8	5.27	2.58	hypothetical protein [Arthrobacter sp. 162MFSha1.1]
gi910740677	23.17	2	1	82	9.3	10.35	2.58	probable transposase for insertion sequence element ISRM3-like [Arthrobacter sp. Hiyo4]
gi927293353	7.08	3	1	325	34.0	8.31	2.58	hypothetical protein AL755_03740 (plasmid) [Arthrobacter sp. ERGS1:01]
gi654813319	5.77	1	1	208	22.2	6.40	2.58	imidazoleglycerol-phosphate dehydratase [Arthrobacter sp. MA-N2]
gi927293187	4.55	1	1	527	55.7	9.44	2.58	MFS transporter (plasmid) [Arthrobacter sp. ERGS1:01]
gi759717140	2.56	1	1	430	46.6	6.00	2.58	acetyltransferase, partial [Arthrobacter sp. AK-YN10]
gi928486211	4.58	2	1	284	32.4	9.38	2.58	integrase [Arthrobacter alpinus]
gi910252719	4.47	1	1	246	26.8	6.87	2.58	IclR family transcriptional regulator [Arthrobacter siccitolerans]
gi737772149	4.47	1	1	246	26.7	6.16	2.58	IclR family transcriptional regulator [Arthrobacter sp. TB 26]
gi651437040	2.60	2	1	691	73.8	8.25	2.58	hypothetical protein [Arthrobacter sp. H41]
gi654814001	5.62	1	1	249	25.9	4.94	2.58	short-chain dehydrogenase [Arthrobacter sp. MA-N2]
gi914714805	6.02	2	1	349	35.9	4.98	2.58	formimidoylglutamase [Arthrobacter sp. ZBG10]
gi917760389	1.58	5	1	888	93.7	7.84	2.57	hypothetical protein [Arthrobacter sp. L77]
gi737765071	7.78	2	1	270	28.2	4.56	2.57	MULTISPECIES: peptidase M10 [Arthrobacter]
gi651481030	4.67	1	1	257	28.8	8.43	2.57	GntR family transcriptional regulator [Arthrobacter sp. Br18]
gi742859344	6.16	2	1	211	23.0	6.04	2.57	GntR family transcriptional regulator [Arthrobacter sp. W1]
gi517590090	3.67	1	1	381	39.0	5.40	2.57	sodium:proton antiporter [Arthrobacter sp. 135MFCol5.1]
gi651438300	2.55	1	1	509	55.6	5.74	2.57	ABC transporter ATP-binding protein [Arthrobacter sp. H14]
gi742856879	4.31	1	1	325	34.1	5.11	2.57	universal stress protein UspA [Arthrobacter sp. W1]
gi746182788	8.22	1	1	219	23.5	5.16	2.57	SAM-dependent methyltransferase [Arthrobacter sp. MWB30]
gi470220753	7.77	1	1	206	23.0	9.96	2.57	ribosomal-protein-alanine N-acetyltransferase [Arthrobacter gangotriensis Lz1y]
gi542107943	13.89	1	1	108	11.4	4.93	2.57	hypothetical protein M707_14735 [Arthrobacter sp. AK-YN10]
gi742071944	1.81	3	1	1435	152.0	4.98	2.57	hypothetical protein ANMWB30_14570 [Arthrobacter sp. MWB30]
gi515767834	9.56	1	1	136	14.9	5.29	2.57	GntR family transcriptional regulator [Arthrobacter sp. M2012083]
gi517590781	2.83	1	1	459	50.2	5.85	2.56	monooxygenase [Arthrobacter sp. 135MFCol5.1]
gi753939542	3.67	1	1	409	43.3	10.26	2.56	Bcr/CflA family drug resistance efflux transporter [Arthrobacter phenanthrenivorans]
gi740682588	10.83	3	1	157	17.4	7.64	2.56	MarR family transcriptional regulator [Arthrobacter sp. PAMC25486]
gi916782113	7.23	4	1	249	26.8	4.82	2.56	ANTAR domain-containing protein [Arthrobacter sp. 35W]
gi759719808	8.68	1	1	219	23.7	9.89	2.56	amino acid ABC transporter permease [Arthrobacter sp. FB24]
gi542110244	5.25	1	1	324	35.7	6.40	2.56	hypothetical protein M707_02675 [Arthrobacter sp. AK-YN10]
gi757625055	5.69	1	1	281	30.3	6.23	2.56	SGNH hydrolase [Arthrobacter sp. SPG23]
gi937259036	7.47	1	1	174	18.5	6.61	2.56	hypothetical protein AO716_13925 [Arthrobacter sp. Edens01]
gi517600256	3.12	1	1	417	42.0	5.85	2.56	hypothetical protein [Arthrobacter sp. 162MFSha1.1]
gi323471302	4.04	1	1	322	35.4	6.70	2.56	putative taurine catabolism dioxygenase [Arthrobacter phenanthrenivorans Sphe3]
gi928985695	2.75	1	1	473	48.7	9.54	2.56	hypothetical protein [Arthrobacter sp. ERGS1:01]
gi753931349	9.09	3	1	121	13.1	8.51	2.56	single-stranded DNA-binding protein [Arthrobacter arilaitensis]
gi674644425	2.44	1	1	409	41.4	10.32	2.56	Inner membrane transport protein YnfM [Arthrobacter sp. 11W110_air]
gi917530260	10.00	1	1	120	13.2	8.91	2.56	MerR family transcriptional regulator [Arthrobacter sp. PAMC25486]
gi648572961	6.32	1	1	285	29.8	5.39	2.55	NAD(P)-dependent oxidoreductase [Arthrobacter sp. 135MFCol5.1]
gi737813648	12.50	1	1	168	18.6	4.82	2.55	gluconate kinase [Arthrobacter sp. H14]
gi119947517	1.89	1	1	476	48.3	4.92	2.55	xylulokinase [Arthrobacter aurescens TC1]
gi908699479	5.53	2	1	217	23.4	4.87	2.55	hypothetical protein [Arthrobacter sp. RIT-PI-e]
gi219860488	3.03	1	1	759	76.8	4.83	2.55	Fibronectin type III domain protein [Arthrobacter chlorophenolicus A6]
gi690773989	6.45	1	1	310	32.4	4.61	2.55	hypothetical protein HMPREF2128_01785 [Arthrobacter albus DNF00011]
gi517603203	8.62	1	1	174	19.8	5.52	2.55	MULTISPECIES: peptide-methionine (S)-S-oxide reductase [Arthrobacter]
gi823667783	6.57	1	1	198	22.5	6.37	2.55	3-methyladenine DNA glycosylase [Arthrobacter sp. YC-RL1]
gi443479791	5.02	1	1	299	33.0	5.94	2.55	hypothetical protein G205_22471 [Arthrobacter nitrophenolicus]
gi518313079	19.32	1	1	88	9.5	6.74	2.55	MULTISPECIES: hypothetical protein [Arthrobacter]
gi219859919	2.00	1	1	550	56.7	6.33	2.55	L-aspartate oxidase [Arthrobacter chlorophenolicus A6]
gi823667146	5.25	1	1	305	32.3	6.18	2.55	monooxygenase [Arthrobacter sp. YC-RL1]
gi765006782	4.27	1	1	328	34.9	4.94	2.54	aldo/keto reductase [Arthrobacter sp. A3]
gi470221416	4.97	1	1	382	37.3	5.01	2.54	glycerate kinase [Arthrobacter gangotriensis Lz1y]
gi635350870	27.78	1	1	72	8.1	4.67	2.54	DNA binding, excisionase family domain protein [Arthrobacter siccitolerans]
gi737787565	10.87	1	1	138	15.7	6.95	2.54	UTRA domain-containing protein [Arthrobacter albus]
gi910249061	2.66	1	1	451	48.3	9.57	2.54	3-phenylpropionic acid transporter [Arthrobacter siccitolerans]
gi162954487	4.48	2	1	335	35.6	5.34	2.54	glyceraldehyde 3-phosphate dehydrogenase [Renibacterium salmoninarum ATCC 33209]
gi654823193	7.61	1	1	184	19.5	7.68	2.54	haloacid dehalogenase [Arthrobacter sp. I3]

gi765003729	9.64	1	1	197	20.7	5.39	2.54	hypothetical protein [Arthrobacter sp. A3]
gi916876162	7.98	1	1	163	18.0	6.42	2.54	hypothetical protein [Arthrobacter sp. 31Y]
gi359307160	4.73	1	1	317	34.3	7.75	2.54	putative AsnC family transcriptional regulator [Arthrobacter globiformis NBRC 12137]
gi916820239	7.04	2	1	142	15.8	5.02	2.54	hypothetical protein [Arthrobacter sp. H20]
gi652423571	23.33	1	1	60	6.6	8.76	2.54	hypothetical protein [Arthrobacter castelli]
gi654813844	1.38	1	1	797	88.8	7.96	2.54	GTP pyrophosphokinase [Arthrobacter sp. MA-N2]
gi723607561	3.46	3	1	433	47.1	4.91	2.54	putative ABC-type sugar transport system, periplasmic component [Arthrobacter sp. PAMC[illegible]
gi908696833	8.33	3	1	240	26.0	8.24	2.54	hypothetical protein, partial [Arthrobacter sp. RIT-PI-e]
gi651438212	25.97	3	1	77	8.5	5.20	2.54	hypothetical protein [Arthrobacter sp. H14]
gi443479926	8.10	1	1	247	26.5	7.58	2.54	FHA domain-containing protein [Arthrobacter nitrophenolicus]
gi742861268	5.21	1	1	384	42.2	7.44	2.54	DNA polymerase III subunit delta' [Arthrobacter sp. W1]
gi50978534	1.55	2	1	965	103.9	4.74	2.54	6-alpha-glucosyltransferase [Arthrobacter globiformis]
gi542106842	4.43	4	1	316	33.7	5.67	2.54	glycosyl transferase [Arthrobacter sp. AK-YN10]
gi917021963	7.49	7	1	227	22.8	5.29	2.54	keto-deoxy-phosphogluconate aldolase [Arthrobacter sp. UNC362MFTsu5.1]
gi517592486	5.17	1	1	406	41.6	6.46	2.53	ROK family transcriptional regulator [Arthrobacter sp. 135MFCol5.1]
gi323471103	2.54	1	1	787	81.8	5.01	2.53	beta-glucosidase-like glycosyl hydrolase [Arthrobacter phenanthrenivorans Sphe3]
gi652423858	3.53	1	1	340	36.3	8.02	2.53	short-chain dehydrogenase [Arthrobacter castelli]
gi937259305	6.16	1	1	211	22.3	5.20	2.53	thymidylate kinase [Arthrobacter sp. Edens01]
gi918267626	14.47	1	1	76	8.1	6.62	2.53	transcriptional regulator protein [Arthrobacter sp. Hiyo1]
gi927295786	3.23	2	1	495	53.4	5.66	2.53	carnitine dehydratase [Arthrobacter sp. ERGS1:01]
gi162953037	2.45	1	1	531	59.2	9.31	2.53	conserved hypothetical protein [Renibacterium salmoninarum ATCC 33209]
gi476402267	12.28	1	1	171	18.2	8.97	2.53	hypothetical protein D477_004506 [Arthrobacter crystallopoietes BAB-32]
gi517591853	5.17	3	1	290	30.8	5.33	2.53	hypothetical protein [Arthrobacter sp. 135MFCol5.1]
gi517600443	3.63	1	1	303	31.3	6.10	2.53	hypothetical protein [Arthrobacter sp. 162MFSha1.1]
gi476400113	11.63	1	1	86	10.4	9.20	2.53	Txe/YoeB family addiction module toxin [Arthrobacter crystallopoietes BAB-32]
gi737793078	2.30	1	1	565	59.6	7.87	2.52	FAD-binding dehydrogenase [Arthrobacter nicotinovorans]
gi930827936	9.18	1	1	196	21.4	4.89	2.52	deaminase [Arthrobacter arilaitensis]
gi403230235	2.99	1	1	435	45.2	4.96	2.52	homoserine dehydrogenase Hom [Arthrobacter sp. Rue61a]
gi219860720	5.31	2	1	339	36.6	5.38	2.52	Acetyl xylan esterase [Arthrobacter chlorophenolicus A6]
gi545109387	3.76	1	1	425	44.2	5.22	2.52	alanine racemase [Arthrobacter sp. AK-YN10]
gi443481311	1.90	1	1	527	57.3	9.92	2.52	hypothetical protein G205_13117 [Arthrobacter nitrophenolicus]
gi518311534	1.86	1	1	539	57.5	5.22	2.52	hypothetical protein [Arthrobacter sp. TB 23]
gi119947817	2.01	2	1	349	37.2	5.24	2.52	oxidoreductase family, NAD-binding Rossmann fold domain protein [Arthrobacter aurescen[illegible]
gi162953890	30.00	1	1	50	5.5	6.62	2.52	dipeptide-binding protein [Renibacterium salmoninarum ATCC 33209]
gi651464139	4.65	1	1	452	50.0	5.52	2.52	hypothetical protein [Arthrobacter sp. 35/47]
gi654825918	3.63	2	1	441	46.1	6.32	2.52	acetyl-CoA acetyltransferase [Arthrobacter sp. H5]
gi742755498	2.18	1	1	733	76.8	5.78	2.52	membrane protein [Arthrobacter phenanthrenivorans]
gi517605195	4.56	3	1	351	37.3	5.43	2.52	oxidoreductase [Arthrobacter sp. 131MFCol6.1]
gi760112289	30.51	2	1	59	6.4	11.58	2.52	hypothetical protein [Arthrobacter chlorophenolicus]
gi757626090	5.16	1	1	446	48.3	6.98	2.52	membrane protein [Arthrobacter sp. SPG23]
gi359305409	5.09	1	1	334	34.8	5.87	2.52	putative LacI family transcriptional regulator [Arthrobacter globiformis NBRC 12137]
gi927033465	7.96	2	1	201	22.5	5.27	2.52	hypothetical protein AFL94_15415 [Arthrobacter sp. LS16]
gi651444596	7.56	1	1	238	25.6	5.03	2.51	hydrolase [Arthrobacter nicotinovorans]
gi652423086	4.26	2	1	235	25.4	5.68	2.51	cobalt ABC transporter ATP-binding protein [Arthrobacter castelli]
gi654811780	8.99	1	1	178	19.0	9.91	2.51	50S ribosomal protein L6 [Arthrobacter sp. MA-N2]
gi767257571	2.75	1	1	545	59.0	5.52	2.51	peptide ABC transporter substrate-binding protein [Arthrobacter sp. IHBB 11108]
gi927033712	15.94	1	1	69	8.0	10.14	2.51	hypothetical protein AFL94_16975 [Arthrobacter sp. LS16]
gi307745899	8.60	6	1	221	24.0	6.06	2.51	two-component system response regulator [Arthrobacter arilaitensis Re117]
gi517599564	6.21	6	1	354	38.9	7.33	2.51	ABC transporter [Arthrobacter sp. 162MFSha1.1]
gi759726347	4.72	2	1	254	27.1	9.54	2.51	lysophospholipase [Arthrobacter sp. I3]
gi551256454	4.59	1	1	370	40.0	4.82	2.51	D-alanine--D-alanine ligase [Arthrobacter sp. PAO19]
gi219861313	5.69	1	1	299	33.0	5.76	2.51	Xylose isomerase domain protein TIM barrel [Arthrobacter chlorophenolicus A6]
gi651491370	2.95	1	1	271	29.6	6.06	2.51	sugar phosphate isomerase [Arthrobacter sp. H20]
gi908690897	3.59	2	1	473	50.2	5.82	2.50	oxidoreductase [Arthrobacter sp. H41]
gi928986577	3.64	1	1	330	33.5	10.52	2.50	hypothetical protein [Arthrobacter sp. ERGS1:01]
gi651436320	4.37	1	1	252	24.3	6.27	2.50	transglycosylase [Arthrobacter sp. H41]
gi737802986	7.84	1	1	204	22.4	8.54	2.50	TetR family transcriptional regulator [Arthrobacter castelli]
gi654827519	9.26	1	1	162	18.1	8.54	2.50	MarR family transcriptional regulator [Arthrobacter sp. H5]

gi476400726	4.51	1	1	288	30.7	4.98	2.50	nucleoside-diphosphate sugar epimerase [Arthrobacter crystallopoietes BAB-32]
gi651441193	2.80	1	1	429	46.7	5.22	2.50	hypothetical protein [Arthrobacter sp. 9MFCol3.1]
gi542108384	1.34	1	1	1192	129.9	5.47	2.50	nuclease [Arthrobacter sp. AK-YN10]
gi443482560	5.37	2	1	354	37.2	5.49	2.50	oxidoreductase [Arthrobacter nitrophenolicus]
gi765012779	8.13	1	1	283	30.5	5.60	2.50	epimerase [Arthrobacter sp. A3]
gi517607854	2.87	1	1	349	37.1	5.35	2.50	LacI family transcriptional regulator [Arthrobacter sp. 161MFSha2.1]
gi652425008	4.08	1	1	466	50.4	6.05	2.50	hypothetical protein [Arthrobacter castelli]
gi651501166	6.93	2	1	231	24.4	6.21	2.50	hypothetical protein [Arthrobacter sp. 35W]
gi759718930	4.17	1	1	336	34.3	4.69	2.50	co-chaperone YbbN [Arthrobacter sp. FB24]
gi765007060	4.14	1	1	314	33.3	7.11	2.50	hypothetical protein [Arthrobacter sp. A3]
gi654825524	18.18	2	1	66	6.6	12.91	2.50	hypothetical protein [Arthrobacter sp. H5]
gi759755372	2.54	2	1	629	66.3	5.87	2.50	AMP-binding protein [Arthrobacter sp. 131MFCol6.1]
gi910747559	4.61	1	1	412	42.1	11.31	2.50	bacillibactin exporter [Arthrobacter sp. Hiyo8]
gi517590813	6.06	1	1	264	27.1	4.96	2.50	nitrilase [Arthrobacter sp. 135MFCol5.1]
gi219859734	5.83	1	1	240	26.1	5.45	2.50	phage shock protein A, PspA [Arthrobacter chlorophenolicus A6]
gi916871509	6.06	1	1	297	32.4	6.96	2.50	peptidase M24, partial [Arthrobacter sp. H5]
gi917739313	9.43	1	1	106	11.8	8.34	2.49	hypothetical protein [Arthrobacter sp. W1]
gi749402241	19.59	1	1	97	11.1	4.55	2.49	membrane protein [Arthrobacter sp. AK-YN10]
gi648260004	30.59	2	1	85	8.9	9.52	2.49	hypothetical protein [Arthrobacter sp. TB 23]
gi749402470	4.55	2	1	286	30.7	5.91	2.49	16S rRNA methyltransferase [Arthrobacter sp. AK-YN10]
gi517607385	2.95	1	1	509	54.9	6.23	2.49	hypothetical protein [Arthrobacter sp. 161MFSha2.1]
gi930827784	4.43	4	1	271	28.7	5.35	2.49	citrate lyase [Arthrobacter arilaitensis]
gi654813931	3.14	2	1	574	64.6	5.22	2.49	X-Pro dipeptidyl-peptidase [Arthrobacter sp. MA-N2]
gi742068749	16.07	1	1	112	11.3	5.26	2.49	PEP-utilizing enzyme [Arthrobacter sp. MWB30]
gi723609565	5.13	1	1	409	44.0	5.55	2.49	N-isopropylammelide isopropylaminohydrolase [Arthrobacter sp. PAMC25486]
gi651485516	5.49	1	1	182	19.4	6.93	2.49	transcriptional regulator [Arthrobacter sp. Br18]
gi917739438	11.27	1	1	142	15.7	7.27	2.49	hypothetical protein [Arthrobacter sp. W1]
gi323468262	10.07	1	1	149	16.4	6.10	2.49	hypothetical protein Asphe3_07430 [Arthrobacter phenanthrenivorans Sphe3]
gi359304767	3.31	3	1	423	47.6	5.19	2.49	putative aminotransferase [Arthrobacter globiformis NBRC 12137]
gi476400044	6.39	1	1	219	24.1	5.86	2.49	GntR family transcriptional regulator [Arthrobacter crystallopoietes BAB-32]
gi654812984	1.15	3	1	1047	117.0	5.88	2.49	helicase [Arthrobacter sp. MA-N2]
gi651494966	4.32	3	1	324	34.8	9.66	2.49	tyrosine recombinase XerD [Arthrobacter sp. H20]
gi443481258	18.28	1	1	93	10.8	8.75	2.49	hypothetical protein G205_13327 [Arthrobacter nitrophenolicus]
gi737777698	8.50	2	1	153	15.6	5.55	2.49	hypothetical protein [Arthrobacter sanguinis]
gi757624875	7.75	3	1	129	14.4	5.47	2.49	hypothetical protein TV39_09050 [Arthrobacter sp. SPG23]
gi757622749	5.50	1	1	327	35.2	6.70	2.49	capsular biosynthesis protein [Arthrobacter sp. SPG23]
gi307743765	2.41	1	1	706	77.0	6.11	2.49	putative ATP-dependent DNA helicase [Arthrobacter arilaitensis Re117]
gi219859705	9.21	1	1	152	16.4	4.83	2.48	conserved hypothetical protein [Arthrobacter chlorophenolicus A6]
gi759731241	5.11	1	1	470	50.1	7.65	2.48	hypothetical protein [Arthrobacter sp. L77]
gi919218993	8.82	1	1	204	21.5	9.85	2.48	hypothetical protein [Arthrobacter sp. YC-RL1]
gi917442286	5.06	1	1	356	38.3	5.67	2.48	NAD kinase [Arthrobacter albus]
gi765001845	3.36	2	1	476	50.5	9.47	2.48	L-asparagine permease [Arthrobacter sp. M2012083]
gi757625845	3.94	1	1	355	37.1	4.69	2.48	hypothetical protein TV39_02750 [Arthrobacter sp. SPG23]
gi654818945	13.33	2	1	75	8.6	10.13	2.48	hypothetical protein [Arthrobacter sp. UNC362MFTsu5.1]
gi518313914	9.52	1	1	126	13.4	8.34	2.48	MULTISPECIES: hypothetical protein [Arthrobacter]
gi742855359	3.79	1	1	449	48.2	6.84	2.48	two-component system sensor histidine kinase [Arthrobacter sp. W1]
gi927293399	23.81	3	1	63	6.7	7.61	2.48	hypothetical protein AL755_04030 (plasmid) [Arthrobacter sp. ERGS1:01]
gi403230228	6.90	1	1	261	28.5	9.38	2.48	putative glycosyl transferase, WecB/TagA/CpsF family protein [Arthrobacter sp. Rue61a]
gi551254988	4.22	1	1	308	34.2	6.48	2.48	taurine dioxygenase [Arthrobacter sp. PAO19]
gi759730822	6.31	1	1	206	22.2	7.03	2.47	hypothetical protein [Arthrobacter sp. L77]
gi910283928	2.37	1	1	801	80.3	6.43	2.47	hypothetical protein [Arthrobacter sp. A3]
gi927293613	6.36	1	1	220	23.7	5.91	2.47	LuxR family transcriptional regulator [Arthrobacter sp. ERGS1:01]
gi908696812	4.04	3	1	446	47.7	7.36	2.47	SAM-dependent methyltransferase [Arthrobacter sp. RIT-PI-e]
gi937259144	3.64	1	1	494	50.7	5.00	2.47	UDP-N-acetylmuramoyl-tripeptide--D-alanyl-D-alanine ligase [Arthrobacter sp. Edens01]
gi307744036	6.29	3	1	318	34.8	5.64	2.47	putative TatD-related deoxyribonuclease [Arthrobacter arilaitensis Re117]
gi723606342	7.05	1	1	241	24.6	6.20	2.47	hypothetical protein ART_0119 [Arthrobacter sp. PAMC25486]
gi723607530	2.19	1	1	594	62.7	4.87	2.47	putative ABC transporter [Arthrobacter sp. PAMC25486]
gi470220548	2.89	6	1	380	39.9	7.42	2.47	G5 domain-containing protein [Arthrobacter gangotriensis Lz1y]

gi917022271	2.51	2	1	438	46.3	5.87	2.47	hypothetical protein [Arthrobacter sp. UNC362MFTsu5.1]
gi930826654	1.28	1	1	1015	109.2	6.58	2.47	hypothetical protein AOZ07_10455 [Arthrobacter arilaitensis]
gi910248959	7.17	2	1	307	34.3	6.73	2.47	AraC family transcriptional regulator [Arthrobacter siccitolerans]
gi323470098	5.67	1	1	247	26.7	7.14	2.47	amino acid ABC transporter ATP-binding protein, PAAT family [Arthrobacter phenanthreniv
gi323470970	6.25	1	1	272	27.4	4.26	2.47	hypothetical protein Asphe3_35540 [Arthrobacter phenanthrenivorans Sphe3]
gi517602605	3.04	1	1	427	45.5	10.74	2.47	hypothetical protein [Arthrobacter sp. 131MFCol6.1]
gi674644439	7.31	5	1	219	23.7	6.52	2.47	putative DNA-binding transcriptional regulator [Arthrobacter sp. 11W110_air]
gi116609923	2.54	1	1	354	38.9	6.52	2.46	esterase/lipase [Arthrobacter sp. FB24]
gi937261768	6.25	1	1	224	24.7	8.44	2.46	Fis family transcriptional regulator [Arthrobacter sp. Edens01]
gi470220621	2.35	1	1	595	65.8	5.57	2.46	succinate dehydrogenase or fumarate reductase, flavoprotein subunit [Arthrobacter gangot
gi674644150	5.39	1	1	297	32.5	4.67	2.46	Methionine-binding lipoprotein MetQ precursor [Arthrobacter sp. 11W110_air]
gi917739558	7.64	7	1	301	32.0	7.27	2.46	ABC transporter permease [Arthrobacter sp. W1]
gi651429624	4.66	1	1	236	24.2	5.80	2.46	excisionase [Arthrobacter sanguinis]
gi219858749	6.98	1	1	215	23.3	9.26	2.46	hypothetical protein Achl_1098 [Arthrobacter chlorophenolicus A6]
gi910744245	12.98	2	1	131	14.7	4.65	2.46	hypothetical protein AHiyo8_19470 [Arthrobacter sp. Hiyo8]
gi307746219	5.78	1	1	329	37.4	8.12	2.46	putative abortive infection bacteriophage resistance protein [Arthrobacter arilaitensis Re11
gi767257269	2.59	1	1	849	93.7	4.75	2.46	aminopeptidase N [Arthrobacter sp. IHBB 11108]
gi652423565	4.67	1	1	300	31.6	5.01	2.46	hypothetical protein [Arthrobacter castelli]
gi654827508	4.27	2	1	328	37.1	5.60	2.46	glutathione S-transferase [Arthrobacter sp. H5]
gi517603848	2.74	1	1	402	42.9	5.26	2.46	O-succinylhomoserine sulfhydrylase [Arthrobacter sp. 131MFCol6.1]
gi908698087	8.31	1	1	313	31.4	4.64	2.45	ribokinase [Arthrobacter sp. RIT-PI-e]
gi759718539	3.32	1	1	211	23.1	4.89	2.45	nucleotide pyrophosphohydrolase [Arthrobacter sp. FB24]
gi742757431	7.19	1	1	334	34.8	8.09	2.45	endonuclease [Arthrobacter phenanthrenivorans]
gi737789675	1.76	1	1	511	56.8	4.98	2.45	lysine--tRNA ligase [Arthrobacter albus]
gi654827881	2.15	1	1	838	90.7	5.90	2.45	glycine cleavage system protein T [Arthrobacter sp. H5]
gi654814276	6.30	2	1	254	27.3	5.33	2.45	hypothetical protein [Arthrobacter sp. MA-N2]
gi757624252	5.65	1	1	301	32.4	5.03	2.45	galactose mutarotase [Arthrobacter sp. SPG23]
gi918266019	14.29	3	1	119	13.0	7.84	2.45	sulfatase-modifying factor 2 [Arthrobacter sp. Hiyo1]
gi651429892	7.26	1	1	234	26.2	5.07	2.45	phosphoglycerate mutase [Arthrobacter sanguinis]
gi323471220	19.12	1	1	68	7.1	4.65	2.45	hypothetical protein Asphe3_38170 [Arthrobacter phenanthrenivorans Sphe3]
gi910249469	3.03	1	1	692	73.7	5.33	2.45	phosphate acetyltransferase [Arthrobacter siccitolerans]
gi470220552	1.34	2	1	821	86.1	5.43	2.45	membrane-bound protease, transglutaminase superfamily [Arthrobacter gangotriensis Lz1y
gi786025726	3.80	1	1	553	58.8	6.19	2.45	two-component system sensor histidine kinase [Arthrobacter chlorophenolicus]
gi757625986	11.86	1	1	194	20.1	5.26	2.45	orotate phosphoribosyltransferase [Arthrobacter sp. SPG23]
gi654816416	2.78	1	1	467	53.0	5.34	2.45	hypothetical protein [Arthrobacter sp. UNC362MFTsu5.1]
gi651436354	5.48	2	1	219	23.9	10.07	2.45	amino acid ABC transporter permease [Arthrobacter sp. H41]
gi307743341	4.35	1	1	276	29.6	4.69	2.45	haloacid dehalogenase-like hydrolase [Arthrobacter arilaitensis Re117]
gi910738034	8.33	1	1	120	13.1	12.70	2.45	hypothetical protein AHiyo4_08560 [Arthrobacter sp. Hiyo4]
gi119951746	3.86	1	1	285	31.3	5.55	2.45	conserved hypothetical protein (plasmid) [Arthrobacter aurescens TC1]
gi403231902	4.12	1	1	364	39.0	5.97	2.45	hypothetical protein ARUE_232p01150 (plasmid) [Arthrobacter sp. Rue61a]
gi765009876	4.49	1	1	445	46.8	5.11	2.45	isochorismate synthase [Arthrobacter sp. A3]
gi916813799	4.36	1	1	321	34.8	6.55	2.45	LacI family transcriptional regulator [Arthrobacter nicotinovorans]
gi918266944	13.11	2	1	122	12.9	9.58	2.45	hypothetical protein AHiyo1_34090 [Arthrobacter sp. Hiyo1]
gi917442132	2.31	3	1	649	68.1	5.07	2.44	cobalt ABC transporter ATP-binding protein [Arthrobacter albus]
gi640203497	6.60	9	1	303	32.7	5.86	2.44	LysR family transcriptional regulator [Arthrobacter sp. 31Y]
gi757625802	6.15	2	1	195	21.4	5.33	2.44	adenylate kinase [Arthrobacter sp. SPG23]
gi918267888	21.13	4	1	71	7.8	11.53	2.44	hypothetical protein AHiyo1_11770 [Arthrobacter sp. Hiyo1]
gi162953526	8.67	1	1	173	17.6	5.22	2.44	carboxypeptidase G2 precursor [Renibacterium salmoninarum ATCC 33209]
gi742071297	2.66	1	1	526	56.8	5.06	2.44	anthranilate synthase component 1 [Arthrobacter sp. MWB30]
gi219861219	1.71	1	1	643	71.7	5.22	2.44	alpha amylase catalytic region [Arthrobacter chlorophenolicus A6]
gi742758467	3.99	1	1	326	33.9	5.73	2.44	prephenate dehydratase [Arthrobacter phenanthrenivorans]
gi910740786	2.91	1	1	481	52.1	5.92	2.44	putative conjugal transfer protein Rv3659c/MT3759 [Arthrobacter sp. Hiyo4]
gi723607728	5.59	2	1	286	29.5	4.92	2.44	rhodanese-related sulfurtransferase [Arthrobacter sp. PAMC25486]
gi323467612	19.40	2	1	67	7.3	6.95	2.44	hypothetical protein Asphe3_00780 [Arthrobacter phenanthrenivorans Sphe3]
gi917442050	5.99	1	1	267	29.3	9.10	2.44	SDR family oxidoreductase [Arthrobacter albus]
gi916834713	4.52	2	1	310	32.1	9.57	2.44	hypothetical protein [Arthrobacter sp. H14]
gi910739144	3.69	1	1	407	43.3	5.63	2.44	hypothetical protein AHiyo4_19660 [Arthrobacter sp. Hiyo4]
gi930826891	2.43	1	1	741	78.7	5.45	2.44	hypothetical protein AOZ07_11770 [Arthrobacter arilaitensis]

gi910283622	5.84	2	1	291	31.4	8.70	2.44	3-methyladenine DNA glycosylase 2 [Arthrobacter sp. A3]
gi910741947	25.58	2	1	43	4.8	5.21	2.44	glutamate dehydrogenase [Arthrobacter sp. Hiyo4]
gi674646418	3.69	1	1	271	29.4	4.84	2.44	Sigma factor SigB regulation protein RsbQ [Arthrobacter sp. 11W110_air]
gi654816496	2.24	1	1	402	42.4	5.10	2.44	aspartate aminotransferase [Arthrobacter sp. UNC362MFTsu5.1]
gi916869830	3.49	1	1	459	48.2	5.19	2.44	aspartate ammonia-lyase [Arthrobacter sp. Br18]
gi740684044	4.73	1	1	402	41.8	5.35	2.44	acetyl-CoA acetyltransferase [Arthrobacter sp. PAMC25486]
gi403228525	26.51	7	1	83	9.3	11.74	2.44	putative integral membrane protein [Arthrobacter sp. Rue61a]
gi742759009	10.65	1	1	169	17.4	9.52	2.44	hypothetical protein RM50_01820 [Arthrobacter phenanthrenivorans]
gi651460880	1.94	3	1	980	104.2	5.31	2.44	ferredoxin [Arthrobacter sp. 35/47]
gi654826097	1.66	1	1	542	59.7	9.61	2.44	transposase [Arthrobacter sp. H5]
gi219858238	5.56	2	1	288	29.4	4.86	2.44	NmrA family protein [Arthrobacter chlorophenolicus A6]
gi786030236	2.61	1	1	345	34.6	5.38	2.44	patatin [Arthrobacter chlorophenolicus]
gi635352914	7.97	1	1	138	14.5	11.58	2.44	hypothetical protein ARTSIC4J27_1215 [Arthrobacter siccitolerans]
gi651488738	16.81	1	1	113	12.6	9.96	2.44	hypothetical protein [Arthrobacter sp. H20]
gi914715479	7.96	1	1	289	31.8	5.31	2.43	glucose-1-phosphate thymidylyltransferase [Arthrobacter sp. ZBG10]
gi918268352	5.96	1	1	302	32.9	5.72	2.43	lysine export transcriptional regulatory protein LysG [Arthrobacter sp. Hiyo1]
gi767257336	12.00	6	1	175	19.6	4.42	2.43	ribosome maturation factor RimM [Arthrobacter sp. IHBB 11108]
gi651502639	5.87	1	1	375	41.3	5.62	2.43	5,10-methylene tetrahydromethanopterin reductase [Arthrobacter sp. 35W]
gi116611989	3.11	1	1	322	34.1	4.97	2.43	NAD-dependent epimerase/dehydratase [Arthrobacter sp. FB24]
gi517598728	3.12	2	1	321	33.7	5.25	2.43	oxidoreductase [Arthrobacter sp. 162MFSha1.1]
gi916781678	6.12	1	1	294	30.3	7.58	2.43	hypothetical protein [Arthrobacter sp. 35W]
gi116611509	8.24	2	1	170	18.8	7.14	2.43	transcriptional regulator, AsnC family [Arthrobacter sp. FB24]
gi742858558	2.93	2	1	375	40.9	4.91	2.43	DNA polymerase III subunit beta [Arthrobacter sp. W1]
gi359305562	8.61	4	1	244	25.8	7.34	2.43	hypothetical protein ARGLB_064_01120 [Arthrobacter globiformis NBRC 12137]
gi651459890	6.02	1	1	216	24.5	4.89	2.43	hypothetical protein [Arthrobacter sp. 35/47]
gi765005560	10.66	2	1	122	13.4	5.59	2.43	GntR family transcriptional regulator [Arthrobacter sp. A3]
gi917022133	3.79	1	1	343	37.4	6.01	2.43	hypothetical protein [Arthrobacter sp. UNC362MFTsu5.1]
gi917739772	3.64	1	1	412	43.9	5.38	2.43	carnitine dehydratase [Arthrobacter sp. W1]
gi636843630	2.17	1	1	554	62.5	5.67	2.43	malate synthase A [Arthrobacter sp. TB 26]
gi652423026	19.74	1	1	76	7.8	7.12	2.43	hypothetical protein [Arthrobacter castelli]
gi545108338	5.81	1	1	344	35.5	5.63	2.43	uroporphyrinogen III methyltransferase [Arthrobacter sp. AK-YN10]
gi654815713	4.15	1	1	337	35.3	5.07	2.43	VWA domain-containing protein [Arthrobacter sp. UNC362MFTsu5.1]
gi910283564	2.72	1	1	368	37.8	6.39	2.43	alcohol dehydrogenase [Arthrobacter sp. A3]
gi910746647	13.25	1	1	83	8.7	9.45	2.43	conserved hypothetical protein [Arthrobacter sp. Hiyo8]
gi651474064	3.77	1	1	345	36.1	5.08	2.43	universal stress protein UspA [Arthrobacter nicotinovorans]
gi823665671	2.81	1	1	640	70.8	7.15	2.43	family 2 glycosyl transferase [Arthrobacter sp. YC-RL1]
gi742857285	8.99	1	1	178	19.1	9.92	2.43	50S ribosomal protein L6 [Arthrobacter sp. W1]
gi443480925	1.68	1	1	653	72.3	6.06	2.43	cellulose synthase [Arthrobacter nitrophenolicus]
gi219862116	2.92	1	1	308	34.2	5.11	2.43	hypothetical protein Achl_4505 (plasmid) [Arthrobacter chlorophenolicus A6]
gi765010971	5.63	1	1	373	39.3	5.11	2.43	sugar ABC transporter substrate-binding protein [Arthrobacter sp. A3]
gi910693554	15.09	1	1	106	11.7	4.88	2.43	triostin synthetase I [Arthrobacter sp. Hiyo6]
gi908699633	11.80	1	1	161	16.8	6.52	2.43	hypothetical protein [Arthrobacter sp. RIT-PI-e]
gi910738907	3.86	2	1	363	39.3	10.14	2.43	DNA translocase FtsK [Arthrobacter sp. Hiyo4]
gi749400960	3.35	2	1	328	35.3	8.90	2.43	hypothetical protein M707_27200, partial [Arthrobacter sp. AK-YN10]
gi636843843	9.20	1	1	250	25.4	7.42	2.43	short-chain dehydrogenase [Arthrobacter sp. TB 26]
gi517605003	4.76	1	1	189	20.0	5.87	2.43	hypothetical protein [Arthrobacter sp. 131MFCol6.1]
gi916816026	1.64	1	1	549	59.0	5.20	2.43	hypothetical protein [Arthrobacter sp. MA-N2]
gi757623776	4.00	1	1	300	33.6	5.99	2.43	glycosyl transferase [Arthrobacter sp. SPG23]
gi651448360	19.44	1	1	72	7.3	4.64	2.43	hypothetical protein [Arthrobacter nicotinovorans]
gi640193625	4.64	1	1	431	47.3	10.39	2.43	membrane protein [Arthrobacter sp. 31Y]
gi162954620	5.43	1	1	221	24.4	4.91	2.42	thymidine kinase [Renibacterium salmoninarum ATCC 33209]
gi219861588	2.84	1	1	422	45.4	5.60	2.42	filamentation induced by cAMP protein Fic (plasmid) [Arthrobacter chlorophenolicus A6]
gi307746561	4.56	1	1	417	44.4	5.47	2.42	conserved hypothetical protein [Arthrobacter arilaitensis Re117]
gi651433680	4.56	1	1	263	28.3	5.12	2.42	DNA-binding protein [Arthrobacter sp. H41]
gi910693983	5.52	1	1	145	15.2	9.06	2.42	hypothetical protein AHiyo6_29390 [Arthrobacter sp. Hiyo6]
gi359304065	2.88	1	1	312	34.5	7.14	2.42	putative LysR family transcriptional regulator [Arthrobacter globiformis NBRC 12137]
gi116609140	21.11	1	1	90	10.6	4.83	2.42	DNA gyrase, A subunit [Arthrobacter sp. FB24]
gi767256771	5.84	1	1	291	31.2	8.72	2.42	hypothetical protein UM93_02335 [Arthrobacter sp. IHBB 11108]

gi518314076	3.75	5	1	320	33.3	10.43	2.42	hypothetical protein [Arthrobacter sp. TB 23]
gi9931315	4.36	1	1	344	37.1	6.93	2.42	lacI repressor homolog [Arthrobacter aurescens]
gi359303701	2.48	1	1	605	66.4	5.30	2.42	putative phenol 2-monooxygenase [Arthrobacter globiformis NBRC 12137]
gi916872219	2.59	1	1	773	83.2	5.06	2.42	transglutaminase [Arthrobacter sp. H5]
gi930824989	3.45	2	1	550	57.0	4.97	2.42	thiamine pyrophosphate-binding protein [Arthrobacter arilaitensis]
gi908698519	5.73	1	1	384	40.6	4.31	2.42	hypothetical protein [Arthrobacter sp. RIT-PI-e]
gi403228602	1.57	2	1	1018	114.3	6.44	2.42	type III restriction enzyme, res subunit [Arthrobacter sp. Rue61a]
gi443481167	1.41	1	1	778	80.4	4.44	2.42	hypothetical protein G205_14256 [Arthrobacter nitrophenolicus]
gi515764565	2.46	1	1	569	58.2	5.74	2.42	2-succinyl-5-enolpyruvyl-6-hydroxy-3-cyclohexene-1-carboxylate synthase [Arthrobacter sp
gi737800903	6.93	1	1	231	25.0	5.02	2.42	histidine kinase [Arthrobacter castelli]
gi651456304	2.88	1	1	695	74.9	4.93	2.42	transketolase, partial [Arthrobacter sp. 35/47]
gi927031197	5.79	2	1	363	37.2	10.87	2.42	iron ABC transporter permease [Arthrobacter sp. LS16]
gi654827094	12.16	1	1	74	8.1	8.22	2.42	hypothetical protein [Arthrobacter sp. H5]
gi908698748	2.33	1	1	473	52.6	8.09	2.42	glycosyl transferase [Arthrobacter sp. RIT-PI-e]
gi910696892	8.24	1	1	279	28.6	5.29	2.41	uncharacterized oxidoreductase YxbG [Arthrobacter sp. Hiyo6]
gi476399615	1.97	2	1	659	68.9	6.20	2.41	copper-translocating P-type ATPase [Arthrobacter crystallopoietes BAB-32]
gi639129335	6.11	1	1	262	29.1	8.38	2.41	DNA replication protein [Arthrobacter sp. CAL618]
gi651499189	7.48	2	1	254	27.6	11.27	2.41	membrane protein [Arthrobacter sp. 35W]
gi674644137	6.33	1	1	221	23.5	5.33	2.41	HTH-type transcriptional regulator BetI [Arthrobacter sp. 11W110_air]
gi116609986	5.71	1	1	210	22.6	5.26	2.41	conserved hypothetical protein [Arthrobacter sp. FB24]
gi307745208	13.79	1	1	87	9.7	4.74	2.41	phosphoribosyl-ATP diphosphatase [Arthrobacter arilaitensis Re117]
gi742071810	9.57	1	1	188	20.9	9.31	2.41	GtrA family protein [Arthrobacter sp. MWB30]
gi695210649	1.51	1	1	661	73.0	9.35	2.41	TraA-like protein, partial (plasmid) [Arthrobacter aurescens]
gi927292765	2.82	1	1	674	70.1	5.22	2.41	flagellar biosynthesis protein FlhA (plasmid) [Arthrobacter sp. ERGS1:01]
gi737777295	4.36	1	1	321	34.5	4.89	2.41	2-oxoisovalerate dehydrogenase [Arthrobacter sanguinis]
gi470221734	11.18	1	1	161	18.1	5.00	2.41	peptide methionine sulfoxide reductase [Arthrobacter gangotriensis Lz1y]
gi654827359	1.96	1	1	408	43.9	7.30	2.41	hydroxyglutarate oxidase [Arthrobacter sp. H5]
gi928487067	8.39	1	1	155	17.3	6.18	2.41	S-ribosylhomocysteine lyase [Arthrobacter alpinus]
gi917442074	2.52	1	1	595	63.5	4.77	2.41	amidase [Arthrobacter albus]
gi219857727	4.49	1	1	379	38.6	5.41	2.41	oxidoreductase domain protein [Arthrobacter chlorophenolicus A6]
gi917760041	8.78	1	1	205	20.6	4.49	2.41	hypothetical protein [Arthrobacter sp. L77]
gi219858559	2.06	2	1	826	88.0	6.33	2.41	conserved hypothetical protein [Arthrobacter chlorophenolicus A6]
gi916357047	5.08	3	1	394	40.6	6.95	2.41	secretion system protein E [Arthrobacter sp. 162MFSha1.1]
gi116612413	4.32	1	1	417	43.4	10.58	2.41	transcriptional regulator, LacI family [Arthrobacter sp. FB24]
gi517604258	4.33	1	1	254	26.7	5.74	2.41	hypothetical protein [Arthrobacter sp. 131MFCol6.1]
gi517600373	3.33	1	1	600	58.5	6.07	2.41	serine protease [Arthrobacter sp. 162MFSha1.1]
gi476399432	6.86	1	1	277	29.7	6.37	2.41	amidase, partial [Arthrobacter crystallopoietes BAB-32]
gi674646377	14.29	1	1	154	16.9	11.59	2.41	hypothetical protein BN1051_02585 [Arthrobacter sp. 11W110_air]
gi651506803	2.59	1	1	501	53.5	4.89	2.41	hypothetical protein [Arthrobacter sp. 35W]
gi910744994	5.36	1	1	429	45.6	10.32	2.41	uncharacterized protein YhgE [Arthrobacter sp. Hiyo8]
gi640203960	3.89	1	1	411	45.6	5.20	2.40	hypothetical protein [Arthrobacter sp. 31Y]
gi927295978	1.91	1	1	786	85.7	5.20	2.40	kojibiose phosphorylase [Arthrobacter sp. ERGS1:01]
gi753932921	18.10	1	1	105	11.5	9.38	2.40	hypothetical protein [Arthrobacter arilaitensis]
gi674646423	1.90	1	1	474	50.3	5.25	2.40	Putrescine oxidase [Arthrobacter sp. 11W110_air]
gi517598764	3.79	2	1	448	47.8	6.46	2.40	ABC transporter substrate-binding protein [Arthrobacter sp. 162MFSha1.1]
gi674644001	5.05	1	1	198	20.6	10.14	2.40	Arginine exporter protein ArgO [Arthrobacter sp. 11W110_air]
gi939050379	3.46	1	1	318	36.4	4.93	2.40	hypothetical protein [Arthrobacter sp. JCM 19049]
gi917407320	3.03	1	1	396	41.2	9.92	2.40	hypothetical protein [Arthrobacter nitrophenolicus]
gi470216241	8.94	1	1	235	23.8	4.51	2.40	haloacid dehalogenase [Arthrobacter gangotriensis Lz1y]
gi759704613	4.20	1	1	286	31.4	7.28	2.40	pseudouridine synthase [Arthrobacter globiformis]
gi916834491	5.69	1	1	334	34.9	6.23	2.40	hypothetical protein [Arthrobacter sp. H14]
gi162953087	9.71	1	1	103	11.3	11.34	2.40	hypothetical protein RSal33209_0858 [Renibacterium salmoninarum ATCC 33209]
gi162952734	2.68	2	1	336	34.3	9.63	2.40	peptidase M23B [Renibacterium salmoninarum ATCC 33209]
gi759718565	3.13	1	1	384	39.5	7.15	2.40	transglycosylase [Arthrobacter sp. FB24]
gi749402062	2.96	1	1	574	65.5	6.32	2.40	DNA primase [Arthrobacter sp. AK-YN10]
gi759709876	7.95	2	1	264	28.8	4.83	2.40	diguanylate phosphodiesterase [Arthrobacter sp. 9MFCol3.1]
gi515767322	3.16	1	1	538	58.1	4.75	2.40	beta-N-acetylhexosaminidase [Arthrobacter sp. M2012083]
gi910741537	5.46	1	1	238	25.4	8.70	2.40	uncharacterized HTH-type transcriptional regulator PH0140 [Arthrobacter sp. Hiyo4]

gi307743599	5.02	3	1	299	32.5	4.81	2.40	hypothetical secreted protein [Arthrobacter arilaitensis Re117]
gi742859230	4.73	2	1	465	50.6	9.60	2.40	hypothetical protein [Arthrobacter sp. W1]
gi651461364	4.91	1	1	163	18.0	5.11	2.40	GNAT family N-acetyltransferase [Arthrobacter sp. 35/47]
gi116612856	3.30	1	1	546	59.3	9.47	2.40	Relaxase/mobilization nuclease family protein (plasmid) [Arthrobacter sp. FB24]
gi651475475	6.43	1	1	311	33.5	9.70	2.40	sugar ABC transporter permease [Arthrobacter nicotinovorans]
gi674643880	4.55	1	1	396	42.1	5.29	2.40	Signal-transduction histidine kinase senX3 [Arthrobacter sp. 11W110_air]
gi928485864	2.80	3	1	286	31.8	9.33	2.40	hypothetical protein AOC05_00580 [Arthrobacter alpinus]
gi737789286	1.44	1	1	696	77.9	4.97	2.40	peptidase S15 [Arthrobacter albus]
gi927294967	0.88	2	1	1358	134.2	5.06	2.40	hypothetical protein AL755_14685 [Arthrobacter sp. ERGS1:01]
gi403230503	3.94	1	1	457	48.5	5.11	2.40	6-phospho-alpha-glucosidase 1 [Arthrobacter sp. Rue61a]
gi640201901	14.75	2	1	183	19.1	9.72	2.40	peptidase A24 [Arthrobacter sp. 31Y]
gi742070463	6.73	1	1	312	32.2	6.57	2.40	DegV domain-containing protein [Arthrobacter sp. MWB30]
gi542110045	5.86	2	1	222	23.1	9.47	2.40	membrane protein [Arthrobacter sp. AK-YN10]
gi737773327	15.79	1	1	133	14.6	6.52	2.39	hypothetical protein [Arthrobacter sp. MA-N2]
gi476399714	4.91	1	1	285	29.9	4.64	2.39	DSBA oxidoreductase [Arthrobacter crystallopoietes BAB-32]
gi116609628	3.66	1	1	355	37.6	4.49	2.39	UBA/THIF-type NAD/FAD binding protein [Arthrobacter sp. FB24]
gi542107666	10.67	1	1	178	18.9	5.35	2.39	GCN5 family acetyltransferase [Arthrobacter sp. AK-YN10]
gi674643980	8.81	1	1	159	17.5	8.24	2.39	hypothetical protein BN1051_00122 [Arthrobacter sp. 11W110_air]
gi119951778	20.00	1	1	105	11.3	5.44	2.39	hypothetical protein AAur_pTC20117 (plasmid) [Arthrobacter aurescens TC1]
gi651433867	2.44	1	1	450	45.4	5.35	2.39	succinate-semialdehyde dehydrogenase [Arthrobacter sp. H41]
gi651475858	12.90	1	1	124	13.5	5.15	2.39	hypothetical protein [Arthrobacter nicotinovorans]
gi219862035	8.72	1	1	218	22.1	5.97	2.39	hypothetical protein Achl_4425 (plasmid) [Arthrobacter chlorophenolicus A6]
gi651435568	6.90	1	1	145	16.5	4.97	2.39	hypothetical protein [Arthrobacter sp. H41]
gi916926385	7.79	1	1	321	33.2	9.32	2.39	hypothetical protein [Arthrobacter sp. 9MFCol3.1]
gi910741645	22.03	1	1	59	6.4	9.76	2.39	oxidoreductase, acting on single donors with incorporation of molecular oxygen, incorporat
gi651438495	2.67	1	1	487	53.6	5.24	2.39	asparagine synthetase B [Arthrobacter sp. H14]
gi757625320	3.17	1	1	347	36.8	4.89	2.39	glycosyl hydrolase [Arthrobacter sp. SPG23]
gi723607877	4.52	3	1	332	36.6	8.43	2.39	glycosyl transferase family protein [Arthrobacter sp. PAMC25486]
gi916692093	2.95	3	1	305	33.3	5.29	2.39	multidrug ABC transporter ATPase [Arthrobacter castelli]
gi910694217	7.69	1	1	221	24.0	9.50	2.39	uncharacterized protein ORF2 in unstable DNA locus, partial [Arthrobacter sp. Hiyo6]
gi654826177	2.19	1	1	1003	110.2	6.74	2.39	glutamine-synthetase [Arthrobacter sp. H5]
gi914714546	4.43	3	1	316	33.5	5.10	2.39	hypothetical protein [Arthrobacter sp. ZBG10]
gi737808296	6.80	1	1	206	23.4	5.25	2.39	hypothetical protein [Arthrobacter sp. H5]
gi918221985	6.20	3	1	274	29.6	7.59	2.39	hypothetical protein [Arthrobacter sp. I3]
gi910740370	7.05	1	1	156	18.4	9.77	2.39	ssrA-binding protein [Arthrobacter sp. Hiyo4]
gi517605538	7.14	1	1	238	25.0	6.80	2.39	MULTISPECIES: ArsR family transcriptional regulator [Arthrobacter]
gi162955602	2.13	1	1	890	89.9	5.64	2.39	putative glycosyl hydrolase [Renibacterium salmoninarum ATCC 33209]
gi930828050	3.36	2	1	506	54.5	5.35	2.39	pyridoxal-dependent decarboxylase [Arthrobacter arilaitensis]
gi654812344	4.42	1	1	339	36.7	9.86	2.39	ABC transporter permease [Arthrobacter sp. MA-N2]
gi170783523	6.29	1	1	302	31.5	6.54	2.39	putative smf family protein (plasmid) [Arthrobacter sp. AK-1]
gi928486029	3.35	1	1	269	28.9	5.68	2.39	ArsR family transcriptional regulator [Arthrobacter alpinus]
gi742859469	3.30	1	1	394	43.5	9.23	2.39	hypothetical protein [Arthrobacter sp. W1]
gi470217673	2.54	2	1	552	55.9	4.77	2.39	phosphoenolpyruvate-protein phosphotransferase [Arthrobacter gangotriensis Lz1y]
gi742859652	2.83	1	1	777	83.4	6.18	2.39	xanthine dehydrogenase [Arthrobacter sp. W1]
gi651465665	3.38	2	1	562	59.8	6.11	2.39	hypothetical protein [Arthrobacter sp. 35/47]
gi909698091	5.62	2	1	338	36.8	9.32	2.39	sugar ABC transporter permease [Arthrobacter sp. M2012083]
gi927295165	2.41	3	1	623	66.2	6.96	2.39	ABC transporter [Arthrobacter sp. ERGS1:01]
gi742859780	7.55	1	1	265	27.6	10.37	2.38	peptide ABC transporter permease [Arthrobacter sp. W1]
gi651464935	3.32	1	1	482	49.9	5.31	2.38	branched-chain alpha-keto acid dehydrogenase subunit E2 [Arthrobacter sp. 35/47]
gi359306099	1.08	1	1	929	95.3	9.66	2.38	hypothetical protein ARGLB_047_00460 [Arthrobacter globiformis NBRC 12137]
gi916692509	4.19	1	1	430	46.8	4.61	2.38	sugar ABC transporter substrate-binding protein [Arthrobacter castelli]
gi919219058	4.80	1	1	229	24.4	7.40	2.38	hypothetical protein [Arthrobacter sp. YC-RL1]
gi651444335	4.15	1	1	265	28.4	5.25	2.38	metal-dependent hydrolase [Arthrobacter nicotinovorans]
gi930826827	3.16	2	1	570	64.2	6.62	2.38	hypothetical protein AOZ07_11425 [Arthrobacter arilaitensis]
gi551254660	1.79	1	1	504	53.1	4.89	2.38	betaine-aldehyde dehydrogenase [Arthrobacter sp. PAO19]
gi742857714	5.52	1	1	163	18.6	4.98	2.38	hypothetical protein [Arthrobacter sp. W1]
gi737812750	3.48	2	1	316	34.0	5.86	2.38	exopolyphosphatase [Arthrobacter sp. H14]
gi767258462	3.14	1	1	350	37.5	7.59	2.38	diacylglycerol kinase [Arthrobacter sp. IHBB 11108]

gi765005392	5.99	2	1	317	34.6	6.58	2.38	multidrug ABC transporter ATPase [Arthrobacter sp. A3]
gi759733607	2.17	1	1	508	52.2	5.03	2.38	hypothetical protein [Arthrobacter sp. L77]
gi518314093	12.50	1	1	112	12.3	6.79	2.38	hypothetical protein [Arthrobacter sp. TB 23]
gi723607997	7.50	3	1	200	21.8	6.64	2.38	Transcriptional regulator, TetR family [Arthrobacter sp. PAMC25486]
gi737786778	9.57	1	1	230	24.7	4.61	2.38	potassium transporter TrkA [Arthrobacter albus]
gi742070188	4.35	1	1	299	31.8	4.89	2.38	thiosulfate sulfurtransferase SseB [Arthrobacter sp. MWB30]
gi443480128	20.99	1	1	81	8.8	9.86	2.38	amidohydrolase [Arthrobacter nitrophenolicus]
gi910743405	12.50	1	1	152	16.3	8.50	2.38	transcriptional regulator BlaI [Arthrobacter sp. Hiyo8]
gi517600149	1.75	1	1	799	85.5	6.02	2.38	ABC transporter [Arthrobacter sp. 162MFSha1.1]
gi737813342	7.46	1	1	268	29.4	5.20	2.38	carbon-nitrogen hydrolase [Arthrobacter sp. H14]
gi910841093	3.19	2	1	407	43.1	5.73	2.38	hypothetical protein ACU18_07140 [Arthrobacter sp. ZBG10]
gi937258107	4.90	1	1	245	26.3	10.18	2.37	hypothetical protein AO716_08330 [Arthrobacter sp. Edens01]
gi443480771	4.92	1	1	366	37.2	9.82	2.37	undecaprenyldiphospho-muramoylpentapeptide beta-N- acetylglucosaminyltransferase [Art
gi823666515	9.63	2	1	218	22.2	9.96	2.37	hypothetical protein AA310_12055 [Arthrobacter sp. YC-RL1]
gi545111146	4.23	1	1	426	46.8	5.15	2.37	serine--tRNA ligase [Arthrobacter sp. AK-YN10]
gi219860045	4.78	1	1	293	32.2	9.35	2.37	ABC-2 type transporter [Arthrobacter chlorophenolicus A6]
gi651464566	9.42	1	1	138	14.9	5.29	2.37	nucleoside diphosphate kinase [Arthrobacter sp. 35/47]
gi930826546	6.71	1	1	313	33.7	6.18	2.37	OpcA protein [Arthrobacter arilaitensis]
gi517609560	4.22	2	1	308	32.7	5.81	2.37	hypothetical protein [Arthrobacter sp. 161MFSha2.1]
gi636846441	2.44	1	1	532	56.1	8.70	2.37	amino acid permease [Arthrobacter sp. TB 26]
gi917572261	4.82	2	1	394	43.2	6.73	2.37	phosphodiesterase [Arthrobacter sp. PAO19]
gi937262456	3.40	1	1	441	43.9	9.70	2.37	MFS transporter [Arthrobacter sp. Edens01]
gi219862251	24.00	2	1	75	8.2	5.41	2.37	conserved hypothetical protein (plasmid) [Arthrobacter chlorophenolicus A6]
gi917760239	6.03	1	1	282	31.0	5.31	2.37	phytanoyl-CoA dioxygenase [Arthrobacter sp. L77]
gi636843332	12.82	1	1	195	20.4	9.54	2.37	crossover junction endodeoxyribonuclease RuvC [Arthrobacter sp. TB 26]
gi916813719	1.82	1	1	879	93.4	6.24	2.37	histidine kinase [Arthrobacter nicotinovorans]
gi910694291	5.26	1	1	380	39.0	6.64	2.37	N-acetylglucosamine-6-phosphate deacetylase [Arthrobacter sp. Hiyo6]
gi307743697	14.07	1	1	135	15.2	5.02	2.37	4-carboxymuconolactone decarboxylase [Arthrobacter arilaitensis Re117]
gi652425327	4.06	1	1	443	48.2	5.62	2.37	tyrosine--tRNA ligase [Arthrobacter castelli]
gi654827248	5.26	2	1	152	16.6	9.41	2.37	ribonucleoside-diphosphate reductase [Arthrobacter sp. H5]
gi116610500	6.20	2	1	387	42.5	10.02	2.37	Integrase, catalytic region [Arthrobacter sp. FB24]
gi927293482	2.78	1	1	791	83.1	6.49	2.37	hypothetical protein AL755_04595 [Arthrobacter sp. ERGS1:01]
gi910746406	3.23	2	1	341	38.0	7.08	2.37	lipoyl synthase [Arthrobacter sp. Hiyo8]
gi651429404	4.78	1	1	314	34.3	7.94	2.37	metallophosphoesterase [Arthrobacter sanguinis]
gi651507339	4.90	1	1	204	21.3	5.03	2.37	hypothetical protein [Arthrobacter sp. 35W]
gi927293625	3.74	1	1	401	42.6	5.35	2.37	N-acyl-L-amino acid amidohydrolase [Arthrobacter sp. ERGS1:01]
gi542108793	12.27	1	1	163	18.3	5.81	2.37	hypothetical protein M707_10015 [Arthrobacter sp. AK-YN10]
gi517590795	5.34	1	1	206	21.5	11.47	2.37	hypothetical protein [Arthrobacter sp. 135MFCol5.1]
gi403229702	4.12	2	1	437	47.3	5.35	2.37	hypothetical protein ARUE_c22210 [Arthrobacter sp. Rue61a]
gi742070373	6.10	1	1	164	18.9	5.07	2.37	hypothetical protein ANMWB30_24140 [Arthrobacter sp. MWB30]
gi737800971	3.00	1	1	367	39.5	5.24	2.37	two-component system sensor histidine kinase [Arthrobacter castelli]
gi651438847	5.28	1	1	284	30.7	6.98	2.37	hypothetical protein [Arthrobacter sp. H14]
gi162954343	8.21	1	1	195	20.1	9.28	2.37	glycerol-3-phosphate-binding protein [Renibacterium salmoninarum ATCC 33209]
gi749401042	7.57	1	1	304	33.5	7.42	2.37	amino acid transporter, partial [Arthrobacter sp. AK-YN10]
gi403230801	11.11	2	1	153	17.6	7.31	2.36	hypothetical protein ARUE_c33420 [Arthrobacter sp. Rue61a]
gi470215894	3.71	2	1	404	44.1	5.82	2.36	FAD-binding monooxygenase [Arthrobacter gangotriensis Lz1y]
gi551254175	3.08	2	1	454	46.9	5.48	2.36	D-alanyl-D-alanine carboxypeptidase [Arthrobacter sp. PAO19]
gi908699494	9.05	2	1	232	25.6	6.67	2.36	GntR family transcriptional regulator [Arthrobacter sp. RIT-PI-e]
gi749402549	4.89	1	1	225	22.7	8.94	2.36	PTS lactose transporter subunit IIC, partial [Arthrobacter sp. AK-YN10]
gi654819634	4.07	3	1	344	36.0	10.04	2.36	peptide ABC transporter permease [Arthrobacter sp. UNC362MFTsu5.1]
gi307746568	4.17	1	1	264	28.3	5.58	2.36	putative carbon-nitrogen hydrolase [Arthrobacter arilaitensis Re117]
gi542107360	6.04	1	1	331	34.4	6.05	2.36	thioredoxin reductase [Arthrobacter sp. AK-YN10]
gi674645091	3.76	1	1	319	33.1	10.84	2.36	hypothetical protein BN1051_01259 [Arthrobacter sp. 11W110_air]
gi651481634	3.90	1	1	333	34.5	6.11	2.36	asparaginase [Arthrobacter sp. Br18]
gi651452473	9.87	1	1	223	23.4	4.88	2.36	haloacid dehalogenase [Arthrobacter nicotinovorans]
gi654813242	7.59	2	1	290	31.5	6.19	2.36	SGNH hydrolase [Arthrobacter sp. MA-N2]
gi651434482	3.28	3	1	396	43.6	5.24	2.36	acyl-CoA dehydrogenase [Arthrobacter sp. H41]
gi757623647	16.67	1	1	144	15.2	10.51	2.36	50S ribosomal protein L15 [Arthrobacter sp. SPG23]

gi765005965	3.69	1	1	325	34.3	5.67	2.36	ABC transporter [Arthrobacter sp. A3]
gi910251939	4.12	1	1	243	25.6	5.47	2.36	succinyl-CoA:3-ketoacid-CoA transferase [Arthrobacter siccitolerans]
gi767257726	6.38	1	1	235	25.2	9.58	2.36	hypothetical protein UM93_08960 [Arthrobacter sp. IHBB 11108]
gi359307938	6.03	1	1	232	24.5	5.57	2.36	gluconate 5-dehydrogenase [Arthrobacter globiformis NBRC 12137]
gi757624730	3.14	2	1	414	44.4	6.30	2.36	lipase/esterase [Arthrobacter sp. SPG23]
gi910251499	5.02	1	1	259	27.9	6.24	2.36	amidohydrolase [Arthrobacter siccitolerans]
gi767256806	8.78	1	1	148	16.3	5.26	2.36	hypothetical protein UM93_02610 [Arthrobacter sp. IHBB 11108]
gi307745284	6.08	1	1	148	16.5	5.92	2.36	helix-turn-helix domain-containing protein [Arthrobacter arilaitensis Re117]
gi651494634	2.60	1	1	578	60.9	4.79	2.36	phosphomannomutase [Arthrobacter sp. H20]
gi823668506	6.02	6	1	299	32.8	5.27	2.36	hydroxyquinol 1,2-dioxygenase, partial [Arthrobacter sp. YC-RL1]
gi767258036	7.17	1	1	293	30.9	9.85	2.36	1,4-dihydroxy-2-naphthoate prenyltransferase [Arthrobacter sp. IHBB 11108]
gi910738667	5.20	1	1	423	46.3	6.14	2.36	conserved hypothetical protein [Arthrobacter sp. Hiyo4]
gi918268170	14.19	1	1	155	17.4	7.72	2.36	hypothetical protein AHiyo1_15210 [Arthrobacter sp. Hiyo1]
gi542110634	4.44	3	1	338	37.4	6.47	2.36	lysophospholipase [Arthrobacter sp. AK-YN10]
gi823668639	5.01	1	1	519	57.7	5.20	2.36	lysyl-tRNA synthetase [Arthrobacter sp. YC-RL1]
gi742853813	10.75	2	1	186	21.2	9.91	2.36	hypothetical protein [Arthrobacter sp. W1]
gi652424909	9.60	1	1	125	13.1	5.82	2.36	pilus assembly protein TadE [Arthrobacter castelli]
gi654828494	2.34	1	1	428	45.5	5.00	2.36	Zn-dependent hydrolase [Arthrobacter sp. H5]
gi551256382	15.00	2	1	120	13.4	9.25	2.36	hypothetical protein [Arthrobacter sp. PAO19]
gi443482464	9.55	1	1	199	20.8	5.36	2.36	deaminase-reductase domain-containing protein [Arthrobacter nitrophenolicus]
gi542110519	3.92	1	1	510	55.6	5.90	2.36	ribose ABC transporter ATP-binding protein [Arthrobacter sp. AK-YN10]
gi759730379	2.42	1	1	578	60.8	4.69	2.35	hypothetical protein [Arthrobacter sp. L77]
gi918265625	23.81	3	1	63	7.0	7.24	2.35	bacterial periplasmic substrate-binding proteins [Arthrobacter sp. Hiyo1]
gi674645701	2.19	1	1	593	64.5	6.62	2.35	Type III restriction enzyme, res subunit [Arthrobacter sp. 11W110_air]
gi914715441	2.70	4	1	741	78.7	6.42	2.35	penicillin amidase [Arthrobacter sp. ZBG10]
gi767258959	4.80	1	1	396	42.6	8.35	2.35	phosphodiesterase [Arthrobacter sp. IHBB 11108]
gi917739673	4.97	1	1	382	40.0	10.48	2.35	hypothetical protein [Arthrobacter sp. W1]
gi916863458	8.79	1	1	239	25.0	10.67	2.35	hypothetical protein [Arthrobacter sp. 35/47]
gi359304306	10.10	3	1	198	21.7	9.88	2.35	hypothetical protein ARGLB_085_01700 [Arthrobacter globiformis NBRC 12137]
gi517607512	3.18	3	1	314	34.3	7.05	2.35	hydrolase [Arthrobacter sp. 161MFSha2.1]
gi116611824	5.69	2	1	246	26.6	5.15	2.35	putative GAF sensor protein [Arthrobacter sp. FB24]
gi518312306	16.28	1	1	129	14.7	7.42	2.35	hypothetical protein [Arthrobacter sp. TB 23]
gi359306758	4.17	2	1	288	31.5	10.52	2.35	hypothetical protein ARGLB_035_00210 [Arthrobacter globiformis NBRC 12137]
gi651429176	2.63	1	1	570	62.6	6.13	2.35	hypothetical protein [Arthrobacter sanguinis]
gi359307289	6.60	1	1	212	22.3	9.13	2.35	putative 3-methyladenine DNA glycosylase [Arthrobacter globiformis NBRC 12137]
gi910737849	9.29	1	1	269	27.0	5.38	2.35	uncharacterized oxidoreductase Rv1144/MT1177 [Arthrobacter sp. Hiyo4]
gi652425681	4.76	1	1	273	29.6	5.86	2.35	hypothetical protein [Arthrobacter castelli]
gi648573110	4.33	1	1	323	34.9	5.35	2.35	oxidoreductase [Arthrobacter sp. 135MFCol5.1]
gi654825832	10.81	1	1	111	12.0	11.25	2.35	DNA-binding protein [Arthrobacter sp. H5]
gi517603018	12.90	1	1	155	16.9	8.35	2.35	transcriptional regulator [Arthrobacter sp. 131MFCol6.1]
gi823666903	4.06	1	1	394	41.6	9.88	2.35	MFS transporter [Arthrobacter sp. YC-RL1]
gi928487698	1.40	1	1	857	91.0	5.73	2.35	histidine kinase [Arthrobacter alpinus]
gi916815820	5.35	1	1	299	32.9	8.46	2.35	hypothetical protein [Arthrobacter sp. MA-N2]
gi517608760	1.67	1	1	539	57.0	9.41	2.35	hypothetical protein [Arthrobacter sp. 161MFSha2.1]
gi515767684	4.01	1	1	399	42.8	6.70	2.35	pilus biosynthesis protein CpaE [Arthrobacter sp. M2012083]
gi470220150	7.66	5	1	222	24.3	8.79	2.35	ubiquinone/menaquinone biosynthesis methyltransferase [Arthrobacter gangotriensis Lz1y]
gi323469382	8.47	1	1	248	27.4	9.13	2.35	glycosyl transferase [Arthrobacter phenanthrenivorans Sphe3]
gi651443602	8.62	1	1	325	32.0	8.69	2.35	polysaccharide deacetylase [Arthrobacter sp. 9MFCol3.1]
gi759733106	4.95	1	1	323	33.9	7.56	2.35	Fe-S cluster assembly protein HesB [Arthrobacter sp. L77]
gi511534767	3.29	2	1	425	46.3	5.07	2.35	6-hydroxy-L-nicotine oxidase (plasmid) [Arthrobacter nicotinovorans]
gi916820153	1.40	1	1	641	68.8	5.58	2.35	aconitate hydratase [Arthrobacter sp. H20]
gi786029697	3.28	1	1	396	43.2	5.66	2.35	acyl-CoA dehydrogenase [Arthrobacter chlorophenolicus]
gi723608102	6.51	1	1	215	24.0	8.78	2.34	putative RNA polymerase ECF-type sigma factor [Arthrobacter sp. PAMC25486]
gi651465234	13.14	1	1	175	17.8	5.27	2.34	adenine phosphoribosyltransferase [Arthrobacter sp. 35/47]
gi323467676	2.58	1	1	621	65.3	5.26	2.34	dihydroxyacid dehydratase [Arthrobacter phenanthrenivorans Sphe3]
gi651444339	5.16	1	1	310	34.3	5.71	2.34	multidrug ABC transporter ATPase [Arthrobacter nicotinovorans]
gi476401426	4.69	1	1	405	44.0	5.94	2.34	sarcosine oxidase subunit beta [Arthrobacter crystallopoietes BAB-32]
gi749401927	6.72	1	1	268	30.3	5.08	2.34	exodeoxyribonuclease III [Arthrobacter sp. AK-YN10]

gi652425674	4.78	1	1	251	28.0	5.24	2.34	hypothetical protein [Arthrobacter castelli]
gi517603287	2.02	1	1	645	69.8	5.88	2.34	hypothetical protein [Arthrobacter sp. 131MFCol6.1]
gi737786711	3.26	4	1	429	44.0	5.25	2.34	hypothetical protein [Arthrobacter albus]
gi908740174	11.83	1	1	169	18.2	4.98	2.34	hypothetical protein [Arthrobacter arilaitensis]
gi219858577	2.35	1	1	637	67.4	6.77	2.34	ABC transporter related [Arthrobacter chlorophenolicus A6]
gi927293494	4.48	1	1	268	27.9	4.89	2.34	3-methyl-2-oxobutanoate hydroxymethyltransferase [Arthrobacter sp. ERGS1:01]
gi916692276	2.78	1	1	575	59.8	9.55	2.34	hypothetical protein [Arthrobacter castelli]
gi759732518	21.74	1	1	69	8.1	8.05	2.34	hypothetical protein [Arthrobacter sp. L77]
gi917760132	7.88	2	1	203	22.1	9.72	2.34	polysaccharide synthesis protein GtrA [Arthrobacter sp. L77]
gi307744921	2.46	1	1	570	62.9	4.91	2.34	2-isopropylmalate synthase [Arthrobacter arilaitensis Re117]
gi910696446	14.29	1	1	119	13.3	5.87	2.34	hypothetical protein AHiyo6_11970, partial [Arthrobacter sp. Hiyo6]
gi823668345	21.43	1	1	98	10.4	7.53	2.34	hypothetical protein AA310_03100 [Arthrobacter sp. YC-RL1]
gi765012041	6.10	1	1	295	31.3	6.43	2.34	ABC transporter [Arthrobacter sp. A3]
gi759759598	23.94	1	1	71	7.3	5.24	2.34	hypothetical protein [Arthrobacter sp. Rue61a]
gi914716082	1.81	1	1	609	63.6	6.99	2.34	long-chain fatty acid--CoA ligase [Arthrobacter sp. ZBG10]
gi518313695	8.00	1	1	275	30.2	4.89	2.34	hypothetical protein [Arthrobacter sp. TB 23]
gi927294799	5.71	1	1	210	22.7	10.52	2.34	hypothetical protein AL755_13590 [Arthrobacter sp. ERGS1:01]
gi759746455	9.61	3	1	229	24.0	8.21	2.34	short-chain dehydrogenase [Arthrobacter sp. 31Y]
gi910744778	12.68	1	1	142	15.2	9.29	2.34	dipeptide transport system permease protein DppB [Arthrobacter sp. Hiyo8]
gi823665386	1.78	2	1	1009	108.4	5.31	2.34	cell division protein FtsK [Arthrobacter sp. YC-RL1]
gi307743776	6.49	1	1	385	40.8	5.62	2.34	cystathionine gamma-synthase [Arthrobacter arilaitensis Re117]
gi219861269	26.09	2	1	92	9.6	8.28	2.34	conserved hypothetical protein [Arthrobacter chlorophenolicus A6]
gi517604591	5.22	1	1	402	42.9	6.87	2.34	hypothetical protein [Arthrobacter sp. 131MFCol6.1]
gi767258975	5.91	1	1	237	25.2	4.88	2.34	haloacid dehalogenase [Arthrobacter sp. IHBB 11108]
gi765005717	6.72	2	1	253	28.0	6.76	2.34	IclR family transcriptional regulator [Arthrobacter sp. A3]
gi919218959	2.90	1	1	449	50.6	6.68	2.34	hypothetical protein [Arthrobacter sp. YC-RL1]
gi914713731	10.34	1	1	145	16.0	5.78	2.34	polyketide cyclase [Arthrobacter sp. ZBG10]
gi162954614	9.76	2	1	123	13.8	8.84	2.34	conserved hypothetical protein [Renibacterium salmoninarum ATCC 33209]
gi927294051	8.97	1	1	156	17.2	8.41	2.34	hypothetical protein AL755_08455 [Arthrobacter sp. ERGS1:01]
gi910696073	4.75	1	1	295	31.7	6.43	2.34	zinc transporter 9 [Arthrobacter sp. Hiyo6]
gi636843631	4.48	2	1	268	28.5	8.44	2.34	hypothetical protein [Arthrobacter sp. TB 26]
gi910739719	2.50	1	1	561	61.0	7.77	2.34	glycerol-3-phosphate dehydrogenase [Arthrobacter sp. Hiyo4]
gi742755410	11.44	1	1	201	21.7	8.94	2.34	hypothetical protein RM50_14010 [Arthrobacter phenanthrenivorans]
gi916871477	3.56	1	1	281	31.0	4.78	2.33	methyltransferase type 12 [Arthrobacter sp. H5]
gi823668189	3.46	1	1	318	35.2	6.95	2.33	transcriptional regulator [Arthrobacter sp. YC-RL1]
gi70983378	3.69	3	1	542	60.1	6.84	2.33	choline oxidase (CodA) [Aspergillus fumigatus Af293]
gi937256649	2.24	1	1	402	40.6	7.58	2.33	hypothetical protein AO716_16560 [Arthrobacter sp. Edens01]
gi654822216	4.67	1	1	300	31.8	5.24	2.33	dienelactone hydrolase [Arthrobacter sp. I3]
gi651450222	9.18	1	1	196	21.1	4.82	2.33	TetR family transcriptional regulator [Arthrobacter nicotinovorans]
gi654827751	21.31	1	1	61	6.5	7.25	2.33	hypothetical protein [Arthrobacter sp. H5]
gi648224258	6.65	1	1	391	41.6	6.11	2.33	glycosyl transferase [Arthrobacter sp. M2012083]
gi916834513	6.21	1	1	306	30.3	4.72	2.33	hypothetical protein [Arthrobacter sp. H14]
gi917745902	12.82	1	1	156	16.7	9.98	2.33	hypothetical protein [Arthrobacter phenanthrenivorans]
gi695270302	6.17	2	1	243	26.8	7.53	2.33	LacI family transcriptional regulator, partial [Arthrobacter globiformis]
gi723609806	6.31	1	1	206	22.2	6.43	2.33	two-component system response regulator [Arthrobacter sp. PAMC25486]
gi742758445	13.79	1	1	87	9.8	4.63	2.33	hypothetical protein RM50_03690 [Arthrobacter phenanthrenivorans]
gi219859924	6.05	1	1	215	23.0	6.70	2.33	phosphoribosyltransferase [Arthrobacter chlorophenolicus A6]
gi651444955	2.07	1	1	580	59.4	9.77	2.33	competence protein ComEC [Arthrobacter nicotinovorans]
gi737789394	7.08	1	1	212	23.8	9.95	2.33	translation initiation factor IF-3 [Arthrobacter albus]
gi652424835	3.27	1	1	459	47.3	5.73	2.33	hypothetical protein [Arthrobacter castelli]
gi654816093	4.94	1	1	263	28.7	9.09	2.33	IclR family transcriptional regulator [Arthrobacter sp. UNC362MFTsu5.1]
gi116611774	3.87	2	1	388	43.2	9.35	2.33	protein of unknown function UPF0027 [Arthrobacter sp. FB24]
gi723609712	8.38	1	1	167	19.0	5.73	2.33	hypothetical protein ART_3489 [Arthrobacter sp. PAMC25486]
gi443480196	12.73	6	1	110	12.2	8.97	2.33	hypothetical protein G205_20183 [Arthrobacter nitrophenolicus]
gi359307635	6.04	2	1	331	35.5	5.30	2.33	putative aldose 1-epimerase [Arthrobacter globiformis NBRC 12137]
gi674646258	3.12	1	1	609	65.6	5.53	2.33	Putative multidrug export ATP-binding/permease protein [Arthrobacter sp. 11W110_air]
gi654818814	8.54	1	1	199	21.2	7.27	2.33	heme-binding protein [Arthrobacter sp. UNC362MFTsu5.1]
gi910250633	8.16	1	1	282	30.4	6.71	2.33	hypothetical protein [Arthrobacter siccitolerans]

gi219861109	2.61	1	1	499	52.4	4.92	2.33	Aldehyde Dehydrogenase [Arthrobacter chlorophenolicus A6]
gi917012979	3.65	1	1	384	41.0	5.17	2.33	amidohydrolase [Arthrobacter sanguinis]
gi737781982	4.52	2	1	310	33.4	6.55	2.33	hypothetical protein [Arthrobacter sp. 35W]
gi651470736	9.96	6	1	241	26.2	5.85	2.33	hypothetical protein [Arthrobacter nicotinovorans]
gi517603311	1.95	1	1	461	44.7	9.52	2.33	hypothetical protein [Arthrobacter sp. 131MFCol6.1]
gi652424118	3.70	1	1	513	55.1	5.24	2.33	amidophosphoribosyltransferase [Arthrobacter castelli]
gi674646632	3.73	1	1	429	46.4	6.00	2.33	hypothetical protein BN1051_02842 [Arthrobacter sp. 11W110_air]
gi517602622	6.67	1	1	120	13.2	6.57	2.33	hypothetical protein [Arthrobacter sp. 131MFCol6.1]
gi917022422	4.79	2	1	292	33.0	5.96	2.33	formyltetrahydrofolate deformylase [Arthrobacter sp. UNC362MFTsu5.1]
gi162955447	2.92	1	1	377	39.1	7.03	2.33	glutamate 5-kinase [Renibacterium salmoninarum ATCC 33209]
gi654825732	4.74	2	1	253	28.1	4.70	2.33	hypothetical protein [Arthrobacter sp. H5]
gi916259788	3.39	2	1	384	43.2	9.89	2.32	hypothetical protein [Arthrobacter sp. TB 23]
gi917739520	3.51	1	1	399	44.5	5.02	2.32	hydrolase [Arthrobacter sp. W1]
gi219860496	26.87	2	1	67	6.8	7.43	2.32	transcriptional regulator, XRE family [Arthrobacter chlorophenolicus A6]
gi914717719	4.29	3	1	350	36.1	10.05	2.32	hypothetical protein [Arthrobacter sp. ZBG10]
gi786029038	7.41	2	1	189	18.9	12.07	2.32	hypothetical protein [Arthrobacter chlorophenolicus]
gi927294199	4.82	1	1	249	26.6	5.41	2.32	hypothetical protein AL755_09425 [Arthrobacter sp. ERGS1:01]
gi759711947	10.98	1	1	173	18.8	7.14	2.32	GNAT family acetyltransferase [Arthrobacter sp. 162MFSha1.1]
gi928488545	4.63	1	1	475	50.3	10.35	2.32	hypothetical protein AOC05_01380 [Arthrobacter alpinus]
gi737812177	6.64	1	1	226	24.2	11.19	2.32	hypothetical protein [Arthrobacter sp. H14]
gi651439143	3.47	1	1	317	33.6	5.05	2.32	hydroxyacid dehydrogenase [Arthrobacter sp. H14]
gi737812951	5.28	1	1	303	33.1	5.10	2.32	GlcNAc-PI de-N-acetylase [Arthrobacter sp. H14]
gi640200444	9.31	1	1	204	22.5	6.14	2.32	TetR family transcriptional regulator [Arthrobacter sp. 31Y]
gi927294160	2.87	2	1	522	56.2	5.07	2.32	anthranilate synthase [Arthrobacter sp. ERGS1:01]
gi910738119	8.68	1	1	242	25.7	4.89	2.32	hypothetical protein AHiyo4_09410 [Arthrobacter sp. Hiyo4]
gi651504614	6.46	2	1	325	33.9	5.11	2.32	2-hydroxyacid dehydrogenase [Arthrobacter sp. 35W]
gi723606894	3.42	1	1	526	57.7	5.80	2.32	proteasome component [Arthrobacter sp. PAMC25486]
gi910696131	4.15	1	1	434	47.6	6.00	2.32	long-chain-fatty-acid--CoA ligase [Arthrobacter sp. Hiyo6]
gi759725836	3.46	3	1	433	46.5	9.52	2.32	glycosyl transferase [Arthrobacter sp. I3]
gi651493316	7.52	2	1	306	32.4	10.30	2.32	membrane protein [Arthrobacter sp. H20]
gi476400753	12.96	2	1	162	17.5	4.59	2.32	hypothetical protein D477_012123 [Arthrobacter crystallopoietes BAB-32]
gi651500577	2.60	1	1	385	38.8	5.33	2.32	hypothetical protein [Arthrobacter sp. 35W]
gi652423122	2.62	1	1	458	49.9	5.66	2.32	hypothetical protein [Arthrobacter castelli]
gi742069081	9.13	1	1	263	29.3	9.89	2.32	hypothetical protein ANMWB30_38740 [Arthrobacter sp. MWB30]
gi939051240	3.25	1	1	431	45.0	5.10	2.32	hypothetical protein, partial [Arthrobacter sp. JCM 19049]
gi737803075	3.90	1	1	436	45.6	5.47	2.32	amidase [Arthrobacter castelli]
gi695210623	8.25	2	1	194	21.0	8.07	2.32	hypothetical protein (plasmid) [Arthrobacter aurescens]
gi918469371	5.59	1	1	358	39.0	6.80	2.32	cyclic pyranopterin phosphate synthase MoaA [Arthrobacter crystallopoietes]
gi937258944	4.50	1	1	555	56.6	9.38	2.32	multidrug MFS transporter [Arthrobacter sp. Edens01]
gi651430726	7.77	1	1	193	20.8	5.59	2.32	hypothetical protein [Arthrobacter sanguinis]
gi910747673	8.06	1	1	248	26.8	5.50	2.32	probable exosome complex exonuclease RRP44 [Arthrobacter sp. Hiyo8]
gi651499073	3.91	1	1	358	37.4	5.34	2.32	polyprenyl synthetase [Arthrobacter sp. 35W]
gi648224249	9.95	1	1	191	20.3	4.97	2.32	TetR family transcriptional regulator [Arthrobacter sp. M2012083]
gi917760180	6.29	1	1	302	31.2	4.58	2.32	hypothetical protein [Arthrobacter sp. L77]
gi823668148	13.76	1	1	109	11.8	8.50	2.32	hypothetical protein AA310_01870 [Arthrobacter sp. YC-RL1]
gi915330581	7.26	2	1	303	31.5	5.22	2.32	LacI family transcriptional regulator [Arthrobacter chlorophenolicus]
gi930827666	3.72	1	1	349	39.0	4.79	2.32	hypothetical protein AOZ07_16195 [Arthrobacter arilaitensis]
gi928486154	2.44	1	1	533	55.6	7.39	2.32	transcriptional regulator [Arthrobacter alpinus]
gi654826000	6.96	1	1	273	29.3	5.34	2.32	indole-3-glycerol phosphate synthase [Arthrobacter sp. H5]
gi651431667	6.93	1	1	274	30.2	5.26	2.32	hypothetical protein [Arthrobacter sanguinis]
gi737789030	9.94	1	1	181	20.2	7.94	2.32	hypothetical protein, partial [Arthrobacter albus]
gi910697862	3.91	1	1	230	25.8	5.69	2.32	uncharacterized protein HI_1410 [Arthrobacter sp. Hiyo6]
gi937258476	5.24	1	1	248	26.8	4.87	2.32	haloacid dehalogenase [Arthrobacter sp. Edens01]
gi742070802	2.32	1	1	691	72.3	5.49	2.32	phosphate acetyltransferase [Arthrobacter sp. MWB30]
gi917013310	2.81	1	1	462	49.3	6.40	2.31	hypothetical protein [Arthrobacter sanguinis]
gi323469683	3.72	1	1	323	36.3	9.63	2.31	hypothetical protein Asphe3_22240 [Arthrobacter phenanthrenivorans Sphe3]
gi737800634	3.55	3	1	423	45.3	4.78	2.31	Fe-S cluster assembly protein SufD [Arthrobacter castelli]
gi767258943	6.96	1	1	230	25.1	6.89	2.31	MerR family transcriptional regulator [Arthrobacter sp. IHBB 11108]

gi651439066	4.74	1	1	359	39.7	5.10	2.31	peptide chain release factor 1 [Arthrobacter sp. H14]
gi767257314	4.30	3	1	582	63.3	5.64	2.31	succinate dehydrogenase [Arthrobacter sp. IHBB 11108]
gi910697188	4.45	1	1	337	35.9	6.74	2.31	lactose operon repressor [Arthrobacter sp. Hiyo6]
gi910738320	2.03	1	1	543	59.9	6.01	2.31	protein involved in DNA alkylation [Arthrobacter sp. Hiyo4]
gi737786726	3.58	1	1	335	36.0	5.30	2.31	hypothetical protein [Arthrobacter albus]
gi910742441	4.59	1	1	327	35.6	5.49	2.31	single-stranded-DNA-specific exonuclease RecJ [Arthrobacter sp. Hiyo8]
gi651501468	4.36	3	1	298	31.4	9.70	2.31	LysR family transcriptional regulator [Arthrobacter sp. 35W]
gi119950541	15.32	1	1	124	13.7	6.68	2.31	hypothetical protein AAur_2847 [Arthrobacter aurescens TC1]
gi651438261	10.05	2	1	189	18.6	4.61	2.31	hypothetical protein [Arthrobacter sp. H14]
gi910738303	14.05	1	1	121	12.7	10.35	2.31	HTH-type transcriptional regulator RegA [Arthrobacter sp. Hiyo4]
gi654822018	2.57	1	1	428	45.1	7.34	2.31	membrane protein [Arthrobacter sp. I3]
gi542110005	17.11	1	1	76	8.5	4.83	2.31	hypothetical protein M707_04815 [Arthrobacter sp. AK-YN10]
gi737880933	4.84	1	1	310	32.9	5.16	2.31	prephenate dehydratase [Cryptosporangium arvum]
gi916835087	7.09	1	1	254	27.3	7.12	2.31	ABC transporter [Arthrobacter sp. H14]
gi162955598	6.02	4	1	349	37.7	5.39	2.31	beta-N-acetylhexosaminidase [Renibacterium salmoninarum ATCC 33209]
gi765013764	3.96	1	1	429	45.4	5.17	2.31	ABC transporter substrate-binding protein [Arthrobacter sp. A3]
gi651464404	4.78	2	1	209	22.5	5.60	2.31	hypothetical protein [Arthrobacter sp. 35/47]
gi219860467	5.53	2	1	217	22.7	10.08	2.31	peptidase S26B, signal peptidase [Arthrobacter chlorophenolicus A6]
gi737802543	5.72	1	1	402	40.4	11.58	2.31	MFS transporter [Arthrobacter castelli]
gi518311001	10.48	1	1	124	13.6	5.10	2.31	GntR family transcriptional regulator [Arthrobacter sp. TB 23]
gi518313565	1.82	1	1	658	70.7	9.88	2.31	MULTISPECIES: hypothetical protein [Arthrobacter]
gi742851248	2.12	1	1	566	61.0	5.83	2.31	peptide ABC transporter ATPase [Arthrobacter sp. W1]
gi742852980	16.67	1	1	90	10.3	10.35	2.31	hypothetical protein [Arthrobacter sp. W1]
gi651480511	7.04	1	1	341	36.1	6.15	2.31	6-phosphofructokinase [Arthrobacter sp. Br18]
gi767257499	9.55	1	1	178	18.6	10.32	2.31	DoxX family protein [Arthrobacter sp. IHBB 11108]
gi737764867	3.69	1	1	325	34.2	5.64	2.31	2-hydroxyacid dehydrogenase [Arthrobacter sp. 161MFSha2.1]
gi910283652	3.35	1	1	538	60.4	5.66	2.31	hypothetical protein [Arthrobacter sp. A3]
gi916872090	9.77	2	1	215	23.4	10.27	2.31	hypothetical protein [Arthrobacter sp. H5]
gi654828762	4.70	1	1	319	34.8	9.82	2.31	sugar ABC transporter permease [Arthrobacter sp. H5]
gi517608933	21.24	1	1	113	12.0	6.02	2.31	hypothetical protein [Arthrobacter sp. 161MFSha2.1]
gi323471382	12.04	1	1	191	19.3	4.74	2.31	putative dinucleotide-binding enzyme (plasmid) [Arthrobacter phenanthrenivorans Sphe3]
gi517603388	7.91	1	1	253	26.4	5.08	2.31	short-chain dehydrogenase [Arthrobacter sp. 131MFCol6.1]
gi403228459	4.79	1	1	313	34.1	6.55	2.31	HTH-type transcriptional regulator [Arthrobacter sp. Rue61a]
gi651453983	4.90	1	1	306	33.5	5.21	2.31	chromosome replication initiation inhibitor protein [Arthrobacter nicotinovorans]
gi939036944	3.27	1	1	306	33.1	5.14	2.31	hypothetical protein [Arthrobacter nitroguajacolicus]
gi916782137	6.39	2	1	266	29.0	8.69	2.31	arginine ABC transporter ATP-binding protein [Arthrobacter sp. 35W]
gi910747071	11.97	1	1	142	15.4	5.58	2.31	L-cystine import ATP-binding protein TcyC [Arthrobacter sp. Hiyo8]
gi219861711	12.30	1	1	122	13.3	6.67	2.31	hypothetical protein Achl_4101 (plasmid) [Arthrobacter chlorophenolicus A6]
gi823668586	7.38	1	1	271	29.6	5.25	2.31	fimbrial protein [Arthrobacter sp. YC-RL1]
gi551253840	2.64	1	1	493	53.8	4.93	2.31	glycosyl hydrolase family 32 [Arthrobacter sp. PAO19]
gi651494477	8.91	1	1	202	21.8	6.05	2.31	16S rRNA methyltransferase [Arthrobacter sp. H20]
gi927296528	3.75	1	1	293	31.1	5.54	2.31	ABC transporter [Arthrobacter sp. ERGS1:01]
gi654822843	8.33	1	1	252	27.3	11.19	2.31	hypothetical protein [Arthrobacter sp. I3]
gi918267809	4.47	1	1	313	33.9	6.13	2.31	tRNA dimethylallyltransferase [Arthrobacter sp. Hiyo1]
gi652423131	5.03	2	1	318	32.7	5.38	2.31	hypothetical protein [Arthrobacter castelli]
gi639128880	2.24	1	1	535	58.7	5.01	2.31	restriction endonuclease subunit M [Arthrobacter sp. CAL618]
gi648224420	6.30	1	1	254	27.3	7.14	2.30	IclR family transcriptional regulator [Arthrobacter sp. M2012083]
gi636845595	14.13	1	1	92	9.9	5.78	2.30	hypothetical protein [Arthrobacter sp. TB 26]
gi928485937	19.79	1	1	96	10.4	10.42	2.30	hypothetical protein AOC05_01065 [Arthrobacter alpinus]
gi323470413	1.93	1	1	622	67.5	9.14	2.30	hypothetical protein Asphe3_29870 [Arthrobacter phenanthrenivorans Sphe3]
gi910248845	3.09	1	1	486	53.8	8.56	2.30	hypothetical protein [Arthrobacter siccitolerans]
gi917739364	14.79	1	1	142	15.5	4.70	2.30	GNAT family N-acetyltransferase [Arthrobacter sp. W1]
gi476399923	3.46	1	1	260	28.0	6.55	2.30	transcriptional regulator [Arthrobacter crystallopoietes BAB-32]
gi910693079	9.76	1	1	123	13.3	5.45	2.30	uncharacterized 42.6 kDa protein in isoamylase 3'region, partial [Arthrobacter sp. Hiyo6]
gi916871801	7.80	2	1	218	23.2	9.35	2.30	hypothetical protein [Arthrobacter sp. H5]
gi916863518	2.58	3	1	426	45.5	11.28	2.30	MFS transporter [Arthrobacter sp. 35/47]
gi910738505	2.21	1	1	407	41.9	10.59	2.30	uncharacterized ABC transporter permease protein YufP [Arthrobacter sp. Hiyo4]
gi927295455	5.00	1	1	280	29.5	5.67	2.30	transcriptional regulator [Arthrobacter sp. ERGS1:01]

gi765004081	5.24	1	1	267	28.2	5.94	2.30	transcriptional regulator [Arthrobacter sp. A3]
gi674645040	2.18	1	1	826	89.0	6.09	2.30	DNA gyrase subunit A [Arthrobacter sp. 11W110_air]
gi651437776	4.06	3	1	271	29.0	5.35	2.30	triosephosphate isomerase [Arthrobacter sp. H14]
gi116609344	10.07	2	1	149	16.1	9.19	2.30	transcriptional regulator, BadM/Rrf2 family [Arthrobacter sp. FB24]
gi517600522	6.79	1	1	162	17.2	8.84	2.30	membrane protein [Arthrobacter sp. 162MFSha1.1]
gi910739504	5.58	1	1	197	21.0	7.34	2.30	UPF0051 protein ML0594 [Arthrobacter sp. Hiyo4]
gi910695468	1.85	1	1	758	82.2	6.06	2.30	carbamoyl-phosphate synthase large chain, partial [Arthrobacter sp. Hiyo6]
gi443482569	5.62	1	1	409	42.3	5.94	2.30	class V aminotransferase [Arthrobacter nitrophenolicus]
gi654826198	2.64	1	1	530	55.5	5.53	2.30	UDP-N-acetylmuramoylalanyl-D-glutamate--2, 6-diaminopimelate ligase [Arthrobacter sp. H
gi517590218	3.27	1	1	397	42.5	6.02	2.30	pilus biosynthesis protein CpaE [Arthrobacter sp. 135MFCol5.1]
gi476399983	4.59	1	1	370	39.1	10.30	2.30	phosphate ABC transporter permease [Arthrobacter crystallopoietes BAB-32]
gi119951623	9.17	1	1	218	23.8	4.67	2.30	putative Thioredoxin domain protein (DSBA) (plasmid) [Arthrobacter aurescens TC1]
gi910738708	6.81	1	1	191	20.7	9.74	2.30	DNA ligase [Arthrobacter sp. Hiyo4]
gi916816200	7.98	4	1	263	27.2	6.40	2.30	hypothetical protein [Arthrobacter sp. MA-N2]
gi470221257	12.67	1	1	150	16.8	6.54	2.30	Organic hydroperoxide resistance transcriptional regulator [Arthrobacter gangotriensis Lz1y
gi476399555	2.46	1	1	406	42.7	6.10	2.30	transcriptional regulator/sugar kinase [Arthrobacter crystallopoietes BAB-32]
gi323470499	1.79	1	1	948	100.3	5.99	2.30	FAD/FMN-dependent dehydrogenase [Arthrobacter phenanthrenivorans Sphe3]
gi765003866	2.06	1	1	874	95.6	5.35	2.30	glycogen phosphorylase [Arthrobacter sp. A3]
gi914714906	4.07	1	1	344	35.1	6.52	2.30	uroporphyrin-III methyltransferase [Arthrobacter sp. ZBG10]
gi443480547	4.71	2	1	361	39.8	6.55	2.30	PhoH family protein [Arthrobacter nitrophenolicus]
gi648224519	4.20	1	1	405	41.6	5.83	2.30	ROK family transcriptional regulator [Arthrobacter sp. M2012083]
gi476399808	4.21	1	1	261	28.9	4.96	2.30	2-oxo-hepta-3-ene-1,7-dioic acid hydratase [Arthrobacter crystallopoietes BAB-32]
gi651483478	6.03	1	1	398	41.9	4.87	2.30	signal recognition particle-docking protein FtsY [Arthrobacter sp. Br18]
gi765012050	10.28	1	1	214	23.3	6.14	2.30	DNA-binding protein [Arthrobacter sp. A3]
gi737800837	1.58	4	1	569	60.7	8.09	2.30	ABC transporter ATP-binding protein [Arthrobacter castelli]
gi517591764	3.48	1	1	287	29.8	5.11	2.30	haloacid dehalogenase [Arthrobacter sp. 135MFCol5.1]
gi910743094	4.46	2	1	269	29.5	10.61	2.30	D-3-phosphoglycerate dehydrogenase [Arthrobacter sp. Hiyo8]
gi759730876	2.61	1	1	536	56.0	5.99	2.29	monovalent cation/H+ antiporter subunit D [Arthrobacter sp. L77]
gi742859730	3.75	1	1	347	36.6	6.32	2.29	l-threonine 3-dehydrogenase [Arthrobacter sp. W1]
gi476398817	5.95	1	1	269	28.1	5.53	2.29	acetyl-CoA acetyltransferase, partial [Arthrobacter crystallopoietes BAB-32]
gi403229336	1.38	1	1	939	101.5	5.07	2.29	alpha-L-rhamnosidase [Arthrobacter sp. Rue61a]
gi674646284	6.10	1	1	328	34.2	6.70	2.29	Daunorubicin/doxorubicin resistance ATP-binding protein DrrA [Arthrobacter sp. 11W110_a
gi119948610	2.88	1	1	417	46.1	5.36	2.29	hypothetical protein AAur_3784 [Arthrobacter aurescens TC1]
gi515767478	6.70	2	1	224	24.2	7.06	2.29	ArsR family transcriptional regulator [Arthrobacter sp. M2012083]
gi476401998	5.73	1	1	349	37.5	5.85	2.29	recombinase A [Arthrobacter crystallopoietes BAB-32]
gi914717019	8.04	1	1	224	24.6	7.05	2.29	DNA alkylation repair protein [Arthrobacter sp. ZBG10]
gi517592340	7.43	1	1	350	37.3	6.04	2.29	hypothetical protein [Arthrobacter sp. 135MFCol5.1]
gi914716585	2.43	1	1	371	39.0	6.86	2.29	phospho-2-dehydro-3-deoxyheptonate aldolase [Arthrobacter sp. ZBG10]
gi937256568	4.63	1	1	367	42.0	5.74	2.29	(p)ppGpp synthetase [Arthrobacter sp. Edens01]
gi759704434	3.69	1	1	325	35.0	5.08	2.29	hypothetical protein [Arthrobacter globiformis]
gi742754123	6.10	1	1	213	21.5	5.40	2.29	hypothetical protein RM50_17540 [Arthrobacter phenanthrenivorans]
gi910283753	8.97	1	1	156	16.2	6.29	2.29	hypothetical protein [Arthrobacter sp. A3]
gi916820226	0.92	1	1	1087	120.7	5.74	2.29	hypothetical protein [Arthrobacter sp. H20]
gi910697300	2.75	1	1	509	53.8	5.88	2.29	tape measure protein [Arthrobacter sp. Hiyo6]
gi476402269	4.23	1	1	567	62.5	5.54	2.29	hypothetical protein D477_004516 [Arthrobacter crystallopoietes BAB-32]
gi937262241	5.85	1	1	188	21.4	5.07	2.29	hypothetical protein AO716_03265 [Arthrobacter sp. Edens01]
gi759729972	1.94	1	1	720	75.0	6.24	2.29	nitrite reductase [Arthrobacter sp. L77]
gi476400145	2.71	2	1	517	52.3	4.53	2.29	poly(3-hydroxybutyrate) depolymerase [Arthrobacter crystallopoietes BAB-32]
gi759736659	31.94	2	1	72	7.1	10.48	2.29	hypothetical protein [Arthrobacter sp. L77]
gi910745527	5.97	1	1	134	14.4	10.86	2.29	3-oxoacyl-[acyl-carrier-protein] reductase 1 [Arthrobacter sp. Hiyo8]
gi651441058	2.26	1	1	575	60.9	5.11	2.29	ribulokinase [Arthrobacter sp. 9MFCol3.1]
gi359307770	2.52	1	1	317	35.3	5.90	2.29	naphthoate synthase [Arthrobacter globiformis NBRC 12137]
gi517598482	15.00	1	1	120	13.3	10.42	2.29	hypothetical protein [Arthrobacter sp. 162MFSha1.1]
gi651460491	4.50	1	1	378	38.8	5.20	2.29	alcohol dehydrogenase [Arthrobacter sp. 35/47]
gi551254009	5.37	1	1	354	38.8	8.72	2.29	cytochrome C biogenesis protein [Arthrobacter sp. PAO19]
gi930827535	7.06	1	1	255	28.5	8.57	2.29	hypothetical protein AOZ07_15435 [Arthrobacter arilaitensis]
gi737808114	9.74	2	1	154	16.8	10.39	2.29	transcriptional regulator [Arthrobacter sp. H5]
gi652424930	2.33	1	1	430	45.8	5.96	2.29	membrane protein [Arthrobacter castelli]

gi767256563	6.77	2	1	192	20.9	10.05	2.29	hypothetical protein UM93_00755 [Arthrobacter sp. IHBB 11108]
gi757624199	5.88	2	1	323	34.7	6.01	2.29	5,10-methylenetetrahydrofolate reductase [Arthrobacter sp. SPG23]
gi927031666	8.49	1	1	259	27.5	5.90	2.29	alkaline phosphatase [Arthrobacter sp. LS16]
gi307743422	14.61	1	1	89	9.6	4.78	2.29	hypothetical protein AARI_01570 [Arthrobacter arilaitensis Re117]
gi116610271	15.17	1	1	145	15.4	9.06	2.29	RDD domain containing protein [Arthrobacter sp. FB24]
gi307744718	1.57	1	1	700	75.9	5.68	2.28	catalase [Arthrobacter arilaitensis Re117]
gi651466431	16.08	1	1	143	15.0	4.68	2.28	peroxiredoxin [Arthrobacter sp. 35/47]
gi654817608	4.85	2	1	309	34.5	6.07	2.28	PaaX family transcriptional regulator [Arthrobacter sp. UNC362MFTsu5.1]
gi307743281	7.57	1	1	185	20.5	9.70	2.28	conserved hypothetical protein [Arthrobacter arilaitensis Re117]
gi517593149	5.00	1	1	260	28.2	6.42	2.28	peptide ABC transporter ATP-binding protein [Arthrobacter sp. 135MFCol5.1]
gi910250104	6.51	1	1	384	39.4	6.07	2.28	cysteine desulfurase [Arthrobacter siccitolerans]
gi651434036	2.47	1	1	446	47.4	3.71	2.28	sugar ABC transporter substrate-binding protein [Arthrobacter sp. H41]
gi937259495	2.83	1	1	566	61.0	8.98	2.28	hypothetical protein AO716_05730 [Arthrobacter sp. Edens01]
gi937258227	4.02	2	1	423	46.8	5.50	2.28	serine--tRNA ligase [Arthrobacter sp. Edens01]
gi767258650	8.22	1	1	304	32.5	5.03	2.28	arginase [Arthrobacter sp. IHBB 11108]
gi742070301	14.95	1	1	107	11.3	12.00	2.28	hypothetical protein ANMWB30_29860 [Arthrobacter sp. MWB30]
gi918266305	25.93	1	1	54	5.7	11.88	2.28	coenzyme A biosynthesis bifunctional protein CoaBC [Arthrobacter sp. Hiyo1]
gi654828534	3.34	1	1	299	32.9	9.20	2.28	hypothetical protein [Arthrobacter sp. H5]
gi652424994	7.51	3	1	213	22.8	5.47	2.28	DNA-binding response regulator [Arthrobacter castelli]
gi939051279	9.71	1	1	103	11.6	4.59	2.28	hypothetical protein [Arthrobacter sp. JCM 19049]
gi910739549	4.43	2	1	429	46.6	5.68	2.28	uncharacterized protein ML0605 [Arthrobacter sp. Hiyo4]
gi916869949	10.17	1	1	177	18.6	10.40	2.28	hypothetical protein [Arthrobacter sp. Br18]
gi823667477	2.08	2	1	529	53.7	7.65	2.28	multidrug transporter [Arthrobacter sp. YC-RL1]
gi786031132	7.81	1	1	269	27.6	5.31	2.28	SDR family oxidoreductase [Arthrobacter chlorophenolicus]
gi654815608	4.55	1	1	242	28.3	9.73	2.28	metal-dependent hydrolase [Arthrobacter sp. PAO19]
gi651440251	6.63	1	1	332	34.7	4.74	2.28	hypothetical protein [Arthrobacter sp. H14]
gi635354038	4.10	1	1	268	29.2	5.00	2.28	aldo/keto reductase family protein [Arthrobacter siccitolerans]
gi742757441	3.13	1	1	383	39.6	7.09	2.28	gamma-glutamyl kinase [Arthrobacter phenanthrenivorans]
gi910748047	5.08	1	1	197	20.7	5.96	2.28	cysteine desulfurase [Arthrobacter sp. Hiyo8]
gi916876223	3.31	1	1	393	42.5	5.74	2.28	alpha-hydroxy-acid oxidizing enzyme [Arthrobacter sp. 31Y]
gi759708263	5.40	3	1	278	29.0	6.54	2.28	shikimate dehydrogenase [Arthrobacter sp. 9MFCol3.1]
gi651499409	4.30	1	1	256	26.7	5.38	2.28	imidazole glycerol phosphate synthase [Arthrobacter sp. 35W]
gi648572740	4.26	4	1	399	42.1	6.55	2.28	chorismate synthase [Arthrobacter sp. 135MFCol5.1]
gi760112202	6.43	2	1	140	16.4	12.37	2.28	hypothetical protein [Arthrobacter chlorophenolicus]
gi927296314	6.93	1	1	231	24.8	6.61	2.28	cobalt ABC transporter ATP-binding protein [Arthrobacter sp. ERGS1:01]
gi695210546	3.65	1	1	274	27.6	7.24	2.28	peptidase (plasmid) [Arthrobacter aurescens]
gi737790625	9.65	1	1	228	24.2	5.31	2.28	ABC transporter [Arthrobacter albus]
gi937258186	5.22	1	1	249	26.3	4.63	2.28	1-(5-phosphoribosyl)-5-[(5-phosphoribosylamino)methylideneamino] imidazole-4-carboxam
gi910250887	2.64	2	1	455	50.5	4.84	2.28	hypothetical protein [Arthrobacter siccitolerans]
gi765006854	4.00	1	1	450	48.5	9.52	2.28	MFS transporter [Arthrobacter sp. A3]
gi917442086	9.40	1	1	117	13.2	5.80	2.28	hypothetical protein [Arthrobacter albus]
gi636846586	6.88	2	1	320	33.5	5.02	2.28	hypothetical protein [Arthrobacter sp. TB 26]
gi654824910	5.58	1	1	215	22.7	9.44	2.28	GCN5 family acetyltransferase [Arthrobacter sp. I3]
gi927294645	19.23	1	1	78	8.3	9.98	2.28	hypothetical protein AL755_12475 [Arthrobacter sp. ERGS1:01]
gi635350826	2.17	3	1	323	35.8	6.57	2.28	bacterial regulatory helix-turn-helix, lysR family protein [Arthrobacter siccitolerans]
gi917759787	8.27	5	1	266	27.1	5.01	2.28	hypothetical protein [Arthrobacter sp. L77]
gi757625290	6.83	1	1	161	17.6	9.06	2.28	Rrf2 family transcriptional regulator [Arthrobacter sp. SPG23]
gi767258004	4.65	1	1	473	49.5	5.12	2.28	branched-chain alpha-keto acid dehydrogenase subunit E2 [Arthrobacter sp. IHBB 11108]
gi190015975	5.38	1	1	279	30.2	6.02	2.28	hypothetical protein PNSL1.080 (plasmid) [Rhodococcus sp. NS1]
gi910696546	3.36	1	1	417	42.9	6.09	2.28	xylose repressor [Arthrobacter sp. Hiyo6]
gi928486489	2.15	1	1	466	51.0	5.11	2.28	hypothetical protein AOC05_04990 [Arthrobacter alpinus]
gi654827731	16.48	1	1	91	9.3	4.51	2.28	phosphotransferase [Arthrobacter sp. H5]
gi910739573	4.04	1	1	322	35.7	6.24	2.28	glutathionyl-hydroquinone reductase YqjG [Arthrobacter sp. Hiyo4]
gi323469761	6.75	1	1	237	26.0	9.96	2.28	putative DNA alkylation repair enzyme [Arthrobacter phenanthrenivorans Sphe3]
gi116609669	3.34	2	1	299	30.9	4.79	2.28	hypothetical protein Arth_0996 [Arthrobacter sp. FB24]
gi917733032	2.84	1	1	423	44.1	5.52	2.28	peptidase M20 [Arthrobacter sp. MWB30]
gi759723168	7.78	1	1	257	27.0	6.21	2.28	dehydrogenase [Arthrobacter sp. I3]
gi515766734	3.57	1	1	560	61.3	4.88	2.27	hypothetical protein [Arthrobacter sp. M2012083]

gi910748428	30.36	1	1	56	5.8	8.56	2.27	hypothetical protein AHiyo8_61300 [Arthrobacter sp. Hiyo8]
gi116608772	3.42	1	1	555	57.1	5.24	2.27	urea amidolyase related protein [Arthrobacter sp. FB24]
gi674646714	5.73	1	1	349	34.5	4.89	2.27	Excalibur calcium-binding domain protein [Arthrobacter sp. 11W110_air]
gi476401216	1.51	1	1	794	85.6	5.81	2.27	hypothetical protein D477_009880 [Arthrobacter crystallopoietes BAB-32]
gi927294849	1.85	1	1	701	76.2	6.51	2.27	phospholipase [Arthrobacter sp. ERGS1:01]
gi910745262	9.70	1	1	134	14.8	5.82	2.27	uncharacterized protein SCO4760 [Arthrobacter sp. Hiyo8]
gi737792917	3.38	1	1	266	27.7	5.69	2.27	Zn-dependent hydrolase [Arthrobacter nicotinovorans]
gi654826381	5.56	1	1	252	27.4	5.66	2.27	glutamine amidotransferase [Arthrobacter sp. H5]
gi307746389	8.29	2	1	217	23.6	5.07	2.27	helix-turn-helix domain-containing protein [Arthrobacter arilaitensis Re117]
gi742854547	2.72	1	1	882	96.9	4.86	2.27	aminopeptidase N [Arthrobacter sp. W1]
gi476402292	8.78	1	1	262	28.0	5.11	2.27	inositol monophosphatase [Arthrobacter crystallopoietes BAB-32]
gi823666256	2.26	1	1	886	94.9	6.11	2.27	GCN5 family acetyltransferase [Arthrobacter sp. YC-RL1]
gi910746484	15.52	1	1	116	11.8	4.74	2.27	tropinone reductase-like 2 [Arthrobacter sp. Hiyo8]
gi651438929	2.36	1	1	509	54.3	9.20	2.27	proline:sodium symporter PutP [Arthrobacter sp. H14]
gi651441765	0.98	1	1	1430	154.9	5.90	2.27	ATPase AAA [Arthrobacter sp. 9MFCol3.1]
gi517599818	3.94	2	1	381	40.4	5.34	2.27	ROK family transcriptional regulator [Arthrobacter sp. 162MFSha1.1]
gi737802987	5.65	1	1	230	25.1	5.15	2.27	hypothetical protein [Arthrobacter castelli]
gi908699412	8.33	1	1	216	24.2	5.05	2.27	hypothetical protein [Arthrobacter sp. RIT-PI-e]
gi723606710	8.92	1	1	213	22.7	8.73	2.27	oxidoreductase domain-containing protein [Arthrobacter sp. PAMC25486]
gi654814845	5.90	1	1	407	41.9	10.70	2.27	ABC transporter permease [Arthrobacter sp. MA-N2]
gi410689701	2.89	2	1	415	46.5	6.02	2.27	DNA-cytosine methyltransferase (plasmid) [Arthrobacter sp. J3-40]
gi654814638	5.28	1	1	322	33.6	4.84	2.27	ribokinase [Arthrobacter sp. MA-N2]
gi651488993	4.64	1	1	237	25.4	9.55	2.27	hypothetical protein, partial [Arthrobacter sp. H20]
gi918266859	2.65	1	1	642	68.8	5.67	2.27	tricorn protease homolog 1 [Arthrobacter sp. Hiyo1]
gi908740405	2.24	1	1	491	53.6	5.92	2.27	acetyl-CoA hydrolase [Arthrobacter arilaitensis]
gi737808668	3.20	1	1	344	36.8	6.33	2.27	LacI family transcriptional regulator [Arthrobacter sp. H5]
gi723606599	1.52	1	1	923	100.6	6.27	2.27	hypothetical protein ART_0376 [Arthrobacter sp. PAMC25486]
gi654813712	9.29	1	1	140	16.0	5.07	2.27	heat-shock protein Hsp20 [Arthrobacter sp. MA-N2]
gi654827604	2.59	2	1	540	57.9	6.14	2.27	hypothetical protein [Arthrobacter sp. H5]
gi742858978	5.26	1	1	266	28.4	5.14	2.27	citrate lyase [Arthrobacter sp. W1]
gi765005785	5.40	1	1	352	36.6	7.20	2.27	hypothetical protein [Arthrobacter sp. A3]
gi765004712	4.41	1	1	431	45.1	5.66	2.27	hypothetical protein [Arthrobacter sp. A3]
gi723607876	1.67	1	1	599	64.4	6.24	2.27	ABC-type multidrug transport system, ATPase and permease component [Arthrobacter sp.
gi757625488	7.33	1	1	273	30.5	7.66	2.27	endonuclease [Arthrobacter sp. SPG23]
gi823667251	4.31	2	1	255	27.3	6.46	2.27	CoA-transferase [Arthrobacter sp. YC-RL1]
gi908698853	21.79	1	1	78	8.4	4.41	2.27	exodeoxyribonuclease VII small subunit [Arthrobacter sp. RIT-PI-e]
gi219857913	4.69	2	1	320	34.4	9.88	2.27	binding-protein-dependent transport systems inner membrane component [Arthrobacter ch
gi651438571	2.30	1	1	434	46.0	4.92	2.27	UDP-glucose 6-dehydrogenase [Arthrobacter sp. H14]
gi654826325	2.84	1	1	599	64.8	5.45	2.27	proline--tRNA ligase [Arthrobacter sp. H5]
gi910252045	4.83	1	1	269	28.5	9.52	2.27	short-chain dehydrogenase [Arthrobacter siccitolerans]
gi910746547	19.05	1	1	84	9.3	8.73	2.27	N-carbamoyl-L-amino acid amidohydrolase [Arthrobacter sp. Hiyo8]
gi654812766	7.44	2	1	403	42.3	5.91	2.27	acetyl-CoA acetyltransferase [Arthrobacter sp. MA-N2]
gi910250932	2.86	2	1	560	57.8	9.52	2.26	serine/threonine protein kinase [Arthrobacter siccitolerans]
gi737781118	8.61	1	1	209	22.7	8.53	2.26	TetR family transcriptional regulator [Arthrobacter sp. 35W]
gi765012770	1.91	1	1	787	82.6	5.16	2.26	beta-glucosidase [Arthrobacter sp. A3]
gi765006320	6.11	1	1	311	31.9	4.92	2.26	NADPH:quinone reductase [Arthrobacter sp. A3]
gi916692464	1.95	1	1	873	92.5	6.51	2.26	ABC transporter [Arthrobacter castelli]
gi928487192	1.45	1	1	1104	113.1	6.06	2.26	hydrogenase expression protein [Arthrobacter alpinus]
gi476402423	4.64	2	1	280	30.3	6.98	2.26	hypothetical protein D477_003433 [Arthrobacter crystallopoietes BAB-32]
gi759756652	16.85	1	1	89	9.2	9.98	2.26	hypothetical protein [Arthrobacter sp. 131MFCol6.1]
gi927270544	1.64	1	1	1281	136.5	4.49	2.26	hypothetical protein [Arthrobacter sp. LS16]
gi119950617	4.72	3	1	339	36.8	6.40	2.26	putative UDP-glucose 4-epimerase [Arthrobacter aurescens TC1]
gi654825245	14.38	1	1	153	15.5	5.06	2.26	hypothetical protein [Arthrobacter sp. I3]
gi742755018	5.97	1	1	201	21.4	4.68	2.26	alanine racemase [Arthrobacter phenanthrenivorans]
gi674646842	1.17	2	1	1109	120.6	5.66	2.26	ATP-dependent helicase/nuclease subunit A [Arthrobacter sp. 11W110_air]
gi910844317	9.14	1	1	175	18.5	9.69	2.26	hypothetical protein ACU18_02075 [Arthrobacter sp. ZBG10]
gi522045869	7.94	1	1	315	33.4	5.52	2.26	hypothetical protein [Streptomyces scabrisporus]
gi654813450	3.90	1	1	538	58.2	7.55	2.26	cytochrome BD ubiquinol oxidase subunit I [Arthrobacter sp. MA-N2]

gi759730133	8.58	1	1	233	24.1	4.68	2.26	haloacid dehalogenase [Arthrobacter sp. L77]
gi759729767	3.37	1	1	356	39.2	5.77	2.26	ATP-dependent DNA ligase [Arthrobacter sp. L77]
gi443480899	3.55	1	1	338	36.2	5.38	2.26	oxidoreductase [Arthrobacter nitrophenolicus]
gi908697498	1.18	1	1	931	97.2	5.57	2.26	hypothetical protein [Arthrobacter sp. RIT-PI-e]
gi916871433	6.13	1	1	310	33.1	9.77	2.26	hypothetical protein [Arthrobacter sp. H5]
gi927031071	6.25	2	1	288	31.2	5.16	2.26	pantoate--beta-alanine ligase [Arthrobacter sp. LS16]
gi476399781	3.07	2	1	521	56.9	11.33	2.26	signal transduction histidine kinase regulating citrate/malate metabolism [Arthrobacter crys
gi651499850	4.04	1	1	446	44.5	10.04	2.26	MFS transporter [Arthrobacter sp. 35W]
gi910748960	8.82	1	1	170	18.1	9.58	2.26	conserved hypothetical protein (plasmid) [Arthrobacter sp. Hiyo8]
gi737785595	4.61	1	1	304	32.0	4.97	2.26	hypothetical protein [Arthrobacter nitrophenolicus]
gi323470944	3.47	1	1	403	42.4	4.82	2.26	uncharacterized conserved protein [Arthrobacter phenanthrenivorans Sphe3]
gi323470232	8.99	1	1	178	19.0	9.91	2.26	LSU ribosomal protein L6P [Arthrobacter phenanthrenivorans Sphe3]
gi654811711	7.58	1	1	264	28.1	8.87	2.26	NmrA family transcriptional regulator [Arthrobacter sp. MA-N2]
gi410689484	5.26	1	1	380	41.5	11.37	2.26	hypothetical protein (plasmid) [Arthrobacter sp. 31-32]
gi927033466	2.09	1	1	717	76.0	4.87	2.26	hypothetical protein AFL94_15420 [Arthrobacter sp. LS16]
gi765004630	3.84	1	1	495	52.5	5.26	2.26	pyruvate kinase [Arthrobacter sp. A3]
gi916863651	6.07	1	1	214	22.4	5.30	2.26	methyltransferase [Arthrobacter sp. 35/47]
gi51534897	1.85	1	1	542	58.8	5.26	2.26	N-substituted formamide deformylase [Arthrobacter pascens]
gi518313599	6.01	1	1	316	33.5	5.54	2.26	MULTISPECIES: hypothetical protein [Arthrobacter]
gi639130725	4.81	1	1	208	22.6	5.03	2.26	enoyl-CoA hydratase [Arthrobacter sp. CAL618]
gi651442095	5.11	1	1	372	40.1	5.12	2.26	aminotransferase [Arthrobacter sp. 9MFCol3.1]
gi443481395	3.46	1	1	434	45.1	7.06	2.26	hypothetical protein G205_12440 [Arthrobacter nitrophenolicus]
gi651449772	4.17	2	1	360	39.1	8.32	2.26	hypothetical protein [Arthrobacter nicotinovorans]
gi476401697	5.86	1	1	290	30.2	5.74	2.26	hydroxymethylglutaryl-CoA lyase [Arthrobacter crystallopoietes BAB-32]
gi908691041	8.65	1	1	266	29.3	5.49	2.26	photosystem reaction center subunit H [Arthrobacter sp. H41]
gi740685394	6.12	1	1	196	20.2	11.56	2.26	hypothetical protein [Arthrobacter sp. PAMC25486]
gi307745575	7.37	1	1	190	20.6	4.77	2.26	adenylate kinase [Arthrobacter arilaitensis Re117]
gi517590728	2.05	1	1	537	59.5	6.11	2.26	hypothetical protein [Arthrobacter sp. 135MFCol5.1]
gi651431189	7.97	1	1	276	28.8	5.15	2.26	methionine aminopeptidase [Arthrobacter sanguinis]
gi917760240	5.57	3	1	287	32.4	7.52	2.26	alpha/beta hydrolase [Arthrobacter sp. L77]
gi767259291	2.77	1	1	397	43.8	8.78	2.26	hypothetical protein UM93_14870 [Arthrobacter sp. IHBB 11108]
gi737802934	4.18	1	1	263	28.5	9.14	2.26	hydrolase [Arthrobacter castelli]
gi476401757	4.23	1	1	331	35.7	6.90	2.25	aspartate carbamoyltransferase catalytic subunit [Arthrobacter crystallopoietes BAB-32]
gi908690647	7.69	1	1	195	20.1	5.29	2.25	hypothetical protein [Arthrobacter sp. H41]
gi786029633	3.82	1	1	262	27.6	5.78	2.25	alkaline phosphatase [Arthrobacter chlorophenolicus]
gi742851908	5.76	1	1	191	21.8	5.74	2.25	hypothetical protein [Arthrobacter sp. W1]
gi443480681	7.00	1	1	200	21.6	9.70	2.25	hypothetical protein G205_17049 [Arthrobacter nitrophenolicus]
gi654825914	12.86	1	1	140	15.6	11.43	2.25	peptide chain release factor 1 [Arthrobacter sp. H5]
gi651441333	1.43	1	1	1046	112.3	6.09	2.25	acriflavin resistance protein [Arthrobacter sp. 9MFCol3.1]
gi651502352	3.66	1	1	355	38.0	5.08	2.25	2-hydroxyacid dehydrogenase [Arthrobacter sp. 35W]
gi162953346	9.16	2	1	131	14.7	6.29	2.25	hypothetical protein RSal33209_1123 [Renibacterium salmoninarum ATCC 33209]
gi908698573	4.55	1	1	352	37.2	10.45	2.25	hypothetical protein [Arthrobacter sp. RIT-PI-e]
gi927293987	3.00	1	1	367	38.1	6.00	2.25	prephenate dehydrogenase [Arthrobacter sp. ERGS1:01]
gi307745071	2.67	1	1	450	48.2	4.51	2.25	tetrahydrofolate synthase [Arthrobacter arilaitensis Re117]
gi651434278	6.75	1	1	252	26.5	5.66	2.25	glutamine amidotransferase [Arthrobacter sp. H41]
gi674646926	4.24	3	1	566	58.4	5.72	2.25	Sulfoacetaldehyde acetyltransferase [Arthrobacter sp. 11W110_air]
gi910696749	4.53	1	1	375	40.3	6.16	2.25	transcriptional regulator, CdaR protein [Arthrobacter sp. Hiyo6]
gi674644278	5.87	1	1	375	40.6	7.36	2.25	hypothetical protein BN1051_00429 [Arthrobacter sp. 11W110_air]
gi359304235	2.85	1	1	421	46.2	5.12	2.25	hypothetical protein ARGLB_085_00990 [Arthrobacter globiformis NBRC 12137]
gi918266712	12.06	1	1	141	15.7	4.78	2.25	18 kDa antigen [Arthrobacter sp. Hiyo1]
gi918268424	5.79	1	1	363	37.2	5.14	2.25	flagellar M-ring protein [Arthrobacter sp. Hiyo1]
gi918268667	11.26	1	1	151	15.5	5.94	2.25	hypothetical protein AHiyo1_01820 [Arthrobacter sp. Hiyo1]
gi737797400	9.44	1	1	233	24.7	7.80	2.25	hypothetical protein [Arthrobacter sp. H20]
gi674644634	1.31	1	1	1149	117.3	6.64	2.25	Glycosyl transferase family 2 [Arthrobacter sp. 11W110_air]
gi654822870	2.33	1	1	386	42.0	9.13	2.25	DNA polymerase IV [Arthrobacter sp. I3]
gi542106414	2.02	1	1	643	70.4	6.24	2.25	hypothetical protein M707_21970 [Arthrobacter sp. AK-YN10]
gi654818151	3.52	1	1	341	36.0	9.83	2.25	FAD-binding monooxygenase [Arthrobacter sp. UNC362MFTsu5.1]
gi916871701	3.70	1	1	324	34.6	4.30	2.25	enterobactin ABC transporter substrate-binding protein [Arthrobacter sp. H5]

gi910842202	3.26	1	1	522	55.7	6.58	2.25	peptide ABC transporter substrate-binding protein [Arthrobacter sp. ZBG10]
gi651450155	3.17	1	1	347	36.9	5.94	2.25	endonuclease [Arthrobacter nicotinovorans]
gi654812352	6.38	1	1	235	24.6	4.63	2.25	succinyl-CoA--3-ketoacid-CoA transferase [Arthrobacter sp. MA-N2]
gi551255735	6.12	1	1	196	21.4	9.80	2.25	hypothetical protein [Arthrobacter sp. PAO19]
gi654826755	4.10	1	1	244	25.9	7.66	2.25	sulfate ABC transporter ATP-binding protein [Arthrobacter sp. H5]
gi786032752	14.63	1	1	164	18.1	9.52	2.25	hypothetical protein [Arthrobacter chlorophenolicus]
gi928985932	2.09	1	1	526	52.4	6.76	2.25	peptidase S8 [Arthrobacter sp. ERGS1:01]
gi654828688	3.40	1	1	471	48.1	4.68	2.25	hypothetical protein [Arthrobacter sp. H5]
gi928488090	7.77	1	1	193	20.8	4.67	2.25	chemical-damaging agent resistance protein C [Arthrobacter alpinus]
gi654816347	4.44	1	1	248	26.2	5.00	2.25	short-chain dehydrogenase [Arthrobacter sp. UNC362MFTsu5.1]
gi910742967	8.64	1	1	162	17.2	10.04	2.25	diacylglycerol kinase catalytic region [Arthrobacter sp. Hiyo8]
gi939036176	10.83	1	1	157	16.4	5.49	2.25	aminoacyl-tRNA deacylase [Arthrobacter nitroguajacolicus]
gi551254149	2.97	1	1	640	71.1	5.02	2.25	tyramine oxidase [Arthrobacter sp. PAO19]
gi927295761	9.05	1	1	199	20.9	7.24	2.25	thiol-disulfide isomerase [Arthrobacter sp. ERGS1:01]
gi651463818	2.85	1	1	386	40.6	5.40	2.25	transcriptional regulator [Arthrobacter sp. 35/47]
gi742759334	2.33	1	1	559	59.7	5.88	2.25	levanase [Arthrobacter phenanthrenivorans]
gi654819558	5.97	1	1	201	21.2	4.79	2.25	hypothetical protein [Arthrobacter sp. UNC362MFTsu5.1]
gi737786828	5.05	1	1	436	45.6	4.75	2.25	homoserine dehydrogenase [Arthrobacter albus]
gi517590965	7.73	1	1	194	21.8	6.57	2.25	hypothetical protein [Arthrobacter sp. 135MFCol5.1]
gi759736292	4.28	1	1	374	39.0	5.67	2.25	prephenate dehydrogenase [Arthrobacter sp. L77]
gi737814106	3.94	1	1	330	36.4	6.55	2.25	hypothetical protein [Arthrobacter sp. H14]
gi651434397	3.70	1	1	459	47.5	5.08	2.25	glutamyl-tRNA reductase [Arthrobacter sp. H41]
gi930828250	5.24	1	1	191	20.3	6.33	2.25	peptide synthetase [Arthrobacter arilaitensis]
gi116613045	3.52	1	1	341	38.7	5.44	2.25	hypothetical protein Arth_4317 (plasmid) [Arthrobacter sp. FB24]
gi470216488	10.16	1	1	128	13.7	11.56	2.25	amino acid permease [Arthrobacter gangotriensis Lz1y]
gi359303338	12.31	2	1	130	13.8	4.55	2.25	hypothetical protein ARGLB_118_00310 [Arthrobacter globiformis NBRC 12137]
gi765013381	24.24	1	1	66	6.9	11.33	2.25	hypothetical protein [Arthrobacter sp. A3]
gi908699423	7.14	1	1	224	23.3	11.81	2.25	hypothetical protein [Arthrobacter sp. RIT-PI-e]
gi916820115	3.96	1	1	429	46.2	5.68	2.24	osmotically inducible protein C [Arthrobacter sp. H20]
gi916573794	9.56	1	1	136	15.1	5.41	2.24	heat-shock protein Hsp20 [Arthrobacter sp. TB 26]
gi470216658	7.69	1	1	247	26.3	4.92	2.24	phosphoribosylformimino-5-aminoimidazole carboxamide ribotide isomerase [Arthrobacter [illegible]
gi928487138	4.26	1	1	282	31.1	9.29	2.24	hypothetical protein AOC05_09380 [Arthrobacter alpinus]
gi6694852	8.91	1	1	247	26.2	6.42	2.24	RecA protein, partial [Arthrobacter protophormiae]
gi648575020	6.83	1	1	249	27.2	6.27	2.24	transcriptional regulator [Arthrobacter sp. 131MFCol6.1]
gi759731896	7.75	1	1	142	15.8	5.19	2.24	hypothetical protein [Arthrobacter sp. L77]
gi504874968	4.14	1	1	338	36.9	8.92	2.24	MULTISPECIES: ATP-dependent DNA ligase [Arthrobacter]
gi323470027	3.80	1	1	237	26.8	9.48	2.24	hypothetical protein Asphe3_25870 [Arthrobacter phenanthrenivorans Sphe3]
gi654816889	3.80	1	1	237	26.8	8.98	2.24	hypothetical protein [Arthrobacter sp. UNC362MFTsu5.1]
gi928486403	16.15	1	1	161	17.0	9.99	2.24	hypothetical protein AOC05_04370 [Arthrobacter alpinus]
gi916816422	2.81	1	1	320	35.4	10.04	2.24	ABC transporter permease [Arthrobacter sp. MA-N2]
gi323471661	11.63	1	1	86	10.2	9.82	2.24	hypothetical protein Asphe3_42790 (plasmid) [Arthrobacter phenanthrenivorans Sphe3]
gi651500178	7.17	1	1	251	27.0	5.41	2.24	carboxymuconolactone decarboxylase [Arthrobacter sp. 35W]
gi928486296	5.95	1	1	336	36.2	5.54	2.24	2-oxoisovalerate dehydrogenase [Arthrobacter alpinus]
gi119950775	3.29	1	1	456	46.6	5.25	2.24	UDP-N-acetylmuramate--alanine ligase [Arthrobacter aurescens TC1]
gi917021958	4.93	1	1	203	22.7	6.35	2.24	hypothetical protein [Arthrobacter sp. UNC362MFTsu5.1]
gi654812334	20.90	1	1	67	7.8	9.69	2.24	hypothetical protein [Arthrobacter sp. MA-N2]
gi917572144	4.85	1	1	433	46.6	5.57	2.24	gamma-glutamyl phosphate reductase [Arthrobacter sp. PAO19]
gi504875391	17.28	1	1	81	9.2	4.97	2.24	DNA-binding domain, excisionase family [Arthrobacter sp. J3-49]
gi648575639	4.56	1	1	307	33.2	5.73	2.24	LysR family transcriptional regulator [Arthrobacter sp. 161MFSha2.1]
gi476400363	1.90	1	1	526	56.5	5.62	2.24	acyltransferase domain-containing membrane protein [Arthrobacter crystallopoietes BAB-3[illegible]
gi640202773	3.85	1	1	364	38.7	5.00	2.24	polyprenyl synthetase [Arthrobacter sp. 31Y]
gi915330547	3.83	2	1	235	26.7	9.60	2.24	hypothetical protein [Arthrobacter chlorophenolicus]
gi517602039	10.40	1	1	173	19.0	9.13	2.24	GNAT family N-acetyltransferase [Arthrobacter sp. 162MFSha1.1]
gi640203143	8.24	1	1	255	26.4	6.01	2.24	short-chain dehydrogenase [Arthrobacter sp. 31Y]
gi737795939	5.28	1	1	246	27.6	9.66	2.24	integrase, partial [Arthrobacter sp. H20]
gi654825674	1.57	1	1	572	60.1	4.89	2.24	DNA repair protein RecN [Arthrobacter sp. H5]
gi651488797	1.52	1	1	592	67.1	7.08	2.24	hypothetical protein [Arthrobacter sp. H20]
gi651483383	6.94	1	1	173	17.5	6.06	2.24	anhydrase [Arthrobacter sp. Br18]

gi359306035	4.61	1	1	347	37.3	5.55	2.24	putative S12 family peptidase [Arthrobacter globiformis NBRC 12137]
gi930825845	8.30	2	1	265	28.2	7.25	2.24	hypothetical protein AOZ07_05765 [Arthrobacter arilaitensis]
gi910283559	9.80	1	1	204	20.9	11.66	2.24	hypothetical protein [Arthrobacter sp. A3]
gi910250413	7.41	1	1	135	15.1	9.36	2.24	hypothetical protein [Arthrobacter siccitolerans]
gi116613074	2.77	1	1	505	57.2	5.43	2.24	Rieske (2Fe-2S) domain protein (plasmid) [Arthrobacter sp. FB24]
gi515767314	7.49	1	1	307	33.2	6.70	2.24	ABC transporter [Arthrobacter sp. M2012083]
gi910252296	5.17	1	1	329	32.6	4.72	2.24	carbohydrate kinase [Arthrobacter siccitolerans]
gi119949960	1.29	1	1	541	58.9	5.40	2.24	putative signal transduction histidine kinase domains protein [Arthrobacter aurescens TC1]
gi674645781	4.36	1	1	459	48.4	5.01	2.24	Glycyl-glycine endopeptidase ALE-1 precursor [Arthrobacter sp. 11W110_air]
gi654827974	3.44	2	1	262	28.3	5.07	2.24	alpha/beta hydrolase [Arthrobacter sp. H5]
gi654823549	3.96	2	1	379	41.2	5.57	2.24	GCN5 family acetyltransferase [Arthrobacter sp. I3]
gi757622588	2.57	1	1	662	71.3	5.08	2.24	levanase [Arthrobacter sp. SPG23]
gi749402401	4.44	1	1	405	42.7	5.05	2.24	hypothetical protein M707_16060 [Arthrobacter sp. AK-YN10]
gi476399993	3.95	1	1	354	37.3	4.81	2.24	hydrolase or acyltransferase [Arthrobacter crystallopoietes BAB-32]
gi916834929	10.67	1	1	150	16.5	4.55	2.24	hypothetical protein [Arthrobacter sp. H14]
gi654827570	12.14	1	1	140	15.8	11.24	2.24	heat-shock protein [Arthrobacter sp. H5]
gi742851088	3.31	1	1	543	58.1	5.08	2.24	2-aminobenzoate-CoA ligase [Arthrobacter sp. W1]
gi652422588	6.32	1	1	269	28.5	6.70	2.24	hypothetical protein [Arthrobacter castelli]
gi654826534	1.99	1	1	402	43.0	5.95	2.24	saframycin Mx1 synthetase B [Arthrobacter sp. H5]
gi651438775	2.17	1	1	415	44.7	4.92	2.24	CoA-transferase [Arthrobacter sp. H14]
gi927296373	6.33	1	1	237	25.9	5.16	2.24	hypothetical protein AL755_13510 [Arthrobacter sp. ERGS1:01]
gi914716619	3.96	1	1	429	46.9	5.38	2.24	ABC transporter substrate-binding protein [Arthrobacter sp. ZBG10]
gi927032389	12.20	1	1	123	13.8	4.51	2.24	hypothetical protein AFL94_08805 [Arthrobacter sp. LS16]
gi927032976	9.81	1	1	265	27.9	4.83	2.24	ABC transporter substrate-binding protein [Arthrobacter sp. LS16]
gi517609482	3.09	2	1	388	40.6	5.17	2.24	acetate kinase [Arthrobacter sp. 161MFSha2.1]
gi916816165	6.61	3	1	257	29.5	11.37	2.24	hypothetical protein [Arthrobacter sp. MA-N2]
gi654827056	9.62	1	1	239	25.2	10.58	2.24	IclR family transcriptional regulator [Arthrobacter sp. H5]
gi916781983	10.20	1	1	196	19.6	4.70	2.24	hypothetical protein [Arthrobacter sp. 35W]
gi651445782	10.81	1	1	185	18.4	9.09	2.24	hypothetical protein [Arthrobacter nicotinovorans]
gi765005876	6.13	1	1	310	31.5	5.11	2.24	threonine dehydratase [Arthrobacter sp. A3]
gi916816001	4.08	1	1	294	30.0	5.63	2.24	hypothetical protein [Arthrobacter sp. MA-N2]
gi170783539	5.71	1	1	210	23.2	7.52	2.23	hypothetical methyltransferase (plasmid) [Arthrobacter sp. AK-1]
gi757625261	1.23	1	1	893	95.3	5.16	2.23	hypothetical protein TV39_05725 [Arthrobacter sp. SPG23]
gi476399869	4.98	1	1	201	21.3	6.55	2.23	primosomal protein [Arthrobacter crystallopoietes BAB-32]
gi470221062	6.84	1	1	351	36.5	5.48	2.23	hypothetical protein ADIAG_01014 [Arthrobacter gangotriensis Lz1y]
gi219860230	3.86	1	1	363	38.6	5.10	2.23	PfkB domain protein [Arthrobacter chlorophenolicus A6]
gi359304535	7.73	1	1	207	21.0	4.59	2.23	2-keto-3-deoxy-phosphogluconate/4-hydroxy-2-oxoglutarate aldolase [Arthrobacter globifo
gi914713708	5.58	1	1	251	26.9	7.14	2.23	hypothetical protein [Arthrobacter sp. ZBG10]
gi737777033	2.67	2	1	825	86.2	4.81	2.23	DNA polymerase III subunit gamma/tau [Arthrobacter sanguinis]
gi545110729	5.31	1	1	339	35.4	6.38	2.23	LacI family transcriptional regulator [Arthrobacter sp. AK-YN10]
gi742755281	23.91	1	1	92	10.2	4.37	2.23	antibiotic biosynthesis monooxygenase [Arthrobacter phenanthrenivorans]
gi674646749	6.32	1	1	269	26.7	4.61	2.23	Hydroxyethylthiazole kinase [Arthrobacter sp. 11W110_air]
gi517590305	2.29	1	1	523	55.8	8.60	2.23	sodium:proton antiporter [Arthrobacter sp. 135MFCol5.1]
gi759714447	1.84	1	1	543	59.8	5.25	2.23	amine oxidase [Arthrobacter sp. AK-YN10]
gi518311340	2.75	1	1	726	81.3	4.94	2.23	hypothetical protein [Arthrobacter sp. TB 23]
gi476402613	7.14	1	1	252	27.1	4.74	2.23	hypothetical protein D477_002813 [Arthrobacter crystallopoietes BAB-32]
gi219858225	3.22	1	1	342	37.3	6.76	2.23	transferase [Arthrobacter chlorophenolicus A6]
gi443483222	6.99	1	1	286	30.9	5.60	2.23	galactose mutarotase [Arthrobacter nitrophenolicus]
gi910252643	4.90	1	1	367	39.5	11.58	2.23	fusaric acid resistance protein [Arthrobacter siccitolerans]
gi937256302	21.05	1	1	95	9.9	9.25	2.23	hypothetical protein AO716_00290 [Arthrobacter sp. Edens01]
gi654814859	2.40	1	1	501	54.5	6.62	2.23	hypothetical protein [Arthrobacter sp. MA-N2]
gi307743429	3.79	1	1	396	43.0	9.32	2.23	conserved hypothetical protein [Arthrobacter arilaitensis Re117]
gi823666185	8.16	1	1	196	21.0	5.02	2.23	dephospho-CoA kinase [Arthrobacter sp. YC-RL1]
gi651444828	5.60	1	1	250	26.0	5.00	2.23	short-chain dehydrogenase [Arthrobacter nicotinovorans]
gi443483269	6.75	1	1	326	35.1	6.55	2.23	ribose-phosphate pyrophosphokinase [Arthrobacter nitrophenolicus]
gi551256929	7.01	1	1	214	24.1	6.71	2.23	GntR family transcriptional regulator [Arthrobacter sp. PAO19]
gi765003701	7.43	1	1	269	29.0	5.12	2.23	haloacid dehalogenase [Arthrobacter sp. A3]
gi737804075	6.58	1	1	152	16.4	11.12	2.23	hypothetical protein [Arthrobacter sp. Br18]

gi470216455	2.64	1	1	417	45.7	4.97	2.23	bifunctional ATP-dependent DNA helicase/DNA polymerase III subunit epsilon [Arthrobacte
gi359306002	3.94	1	1	330	34.4	4.84	2.23	putative phosphatase [Arthrobacter globiformis NBRC 12137]
gi470220769	5.88	1	1	255	28.0	5.10	2.23	hypothetical protein ADIAG_00715 [Arthrobacter gangotriensis Lz1y]
gi654812162	1.56	1	1	577	62.7	5.60	2.23	multidrug ABC transporter ATPase [Arthrobacter sp. MA-N2]
gi910740313	3.95	1	1	481	51.4	6.65	2.23	hypothetical protein AHiyo4_31350 [Arthrobacter sp. Hiyo4]
gi639130803	4.92	1	1	488	52.1	9.32	2.23	MULTISPECIES: MFS transporter [Arthrobacter]
gi759765092	11.11	1	1	180	19.7	4.93	2.23	peptide-methionine (S)-S-oxide reductase [Arthrobacter gangotriensis]
gi162954849	2.25	1	1	445	48.1	5.10	2.23	isochorismate synthase [Renibacterium salmoninarum ATCC 33209]
gi910740630	8.96	1	1	212	22.6	9.14	2.23	HTH-type transcriptional regulator GltC [Arthrobacter sp. Hiyo4]
gi759776160	10.34	1	1	87	9.3	4.59	2.23	hypothetical protein [Arthrobacter sp. SPG23]
gi651429241	4.21	2	1	404	43.0	4.74	2.23	amidohydrolase [Arthrobacter sanguinis]
gi651436434	3.14	1	1	446	47.2	7.23	2.23	aldehyde dehydrogenase, partial [Arthrobacter sp. H41]
gi910742360	5.80	1	1	276	29.8	9.55	2.23	uncharacterized protein y4oD [Arthrobacter sp. Hiyo8]
gi690772417	8.59	2	1	198	22.3	5.71	2.23	hypothetical protein HMPREF2128_08265 [Arthrobacter albus DNF00011]
gi551254977	1.73	1	1	577	61.6	5.80	2.23	preprotein translocase subunit SecD [Arthrobacter sp. PAO19]
gi518311910	3.30	3	1	515	53.3	9.64	2.23	hypothetical protein [Arthrobacter sp. TB 23]
gi737773656	3.56	1	1	590	61.2	4.86	2.23	phosphomannomutase [Arthrobacter sp. MA-N2]
gi916710582	3.26	1	1	430	47.3	8.81	2.23	relaxase, partial [Arthrobacter sp. CAL618]
gi767256718	5.93	1	1	236	26.0	5.87	2.23	hypothetical protein UM93_01930 [Arthrobacter sp. IHBB 11108]
gi928487480	5.91	1	1	203	22.3	5.34	2.23	hypothetical protein AOC05_11815 [Arthrobacter alpinus]
gi910252056	2.82	1	1	496	52.2	5.00	2.23	hypothetical protein [Arthrobacter siccitolerans]
gi928485884	2.29	1	1	393	43.1	6.04	2.23	hypothetical protein AOC05_00720 [Arthrobacter alpinus]
gi489902456	3.48	1	1	603	64.7	5.10	2.23	proline--tRNA ligase [Arthrobacter globiformis]
gi917739648	0.89	1	1	1127	118.1	5.06	2.23	hypothetical protein [Arthrobacter sp. W1]
gi635352118	8.93	2	1	224	24.5	5.45	2.23	bacterial regulatory s, gntR family protein [Arthrobacter siccitolerans]
gi914714357	13.16	1	1	114	12.5	5.24	2.23	hypothetical protein [Arthrobacter sp. ZBG10]
gi928542303	42.86	1	1	42	4.8	4.59	2.23	HTH DNA binding protein [Arthrobacter phage Brent]
gi910739126	2.66	1	1	413	45.0	9.70	2.23	tricorn protease homolog 1 [Arthrobacter sp. Hiyo4]
gi723609953	6.39	3	1	219	23.8	9.58	2.23	ABC-type amino acid transport system, permease component [Arthrobacter sp. PAMC2548(
gi786035308	11.33	1	1	203	22.2	4.17	2.23	ribosome maturation factor RimM [Arthrobacter chlorophenolicus]
gi651437967	1.81	1	1	609	66.6	5.44	2.22	hypothetical protein [Arthrobacter sp. H14]
gi443480258	2.71	1	1	332	38.1	5.00	2.22	hypothetical protein G205_19763 [Arthrobacter nitrophenolicus]
gi403229331	5.32	1	1	376	40.3	7.42	2.22	glucose-resistance amylase regulator [Arthrobacter sp. Rue61a]
gi518314060	19.23	1	1	78	8.4	4.78	2.22	hypothetical protein [Arthrobacter sp. TB 23]
gi723609274	3.83	1	1	313	33.4	4.91	2.22	hypothetical protein ART_3051 [Arthrobacter sp. PAMC25486]
gi323468536	9.95	1	1	191	20.5	11.33	2.22	HNH endonuclease [Arthrobacter phenanthrenivorans Sphe3]
gi937261914	6.46	1	1	263	28.9	8.40	2.22	endonuclease III [Arthrobacter sp. Edens01]
gi119951419	1.76	1	1	511	56.4	6.58	2.22	putative ATP-binding protein (plasmid) [Arthrobacter aurescens TC1]
gi918264984	6.79	1	1	221	24.0	8.21	2.22	UDP-N-acetylmuramoylalanine--D-glutamate ligase, partial [Arthrobacter sp. Hiyo1]
gi517600816	3.27	1	1	336	35.6	5.31	2.22	4Fe-4S ferredoxin [Arthrobacter sp. 162MFSha1.1]
gi654826412	5.44	2	1	331	36.5	5.12	2.22	xylose isomerase [Arthrobacter sp. H5]
gi648574738	5.68	2	1	387	39.8	6.42	2.22	cystathionine gamma-synthase [Arthrobacter sp. 131MFCol6.1]
gi916815954	5.90	2	1	288	28.5	8.43	2.22	hypothetical protein [Arthrobacter sp. MA-N2]
gi937258172	8.13	1	1	160	17.5	9.17	2.22	MarR family transcriptional regulator [Arthrobacter sp. Edens01]
gi517591993	4.47	1	1	246	27.1	6.71	2.22	GntR family transcriptional regulator [Arthrobacter sp. 135MFCol5.1]
gi551255091	7.08	1	1	240	26.4	5.01	2.22	phosphoadenosine phosphosulfate reductase [Arthrobacter sp. PAO19]
gi910249117	29.17	1	1	72	7.8	8.60	2.22	hypothetical protein [Arthrobacter siccitolerans]
gi908699636	3.45	1	1	290	30.5	4.96	2.22	hypothetical protein [Arthrobacter sp. RIT-PI-e]
gi930826078	3.61	1	1	305	32.4	6.98	2.22	hypothetical protein AOZ07_07105 [Arthrobacter arilaitensis]
gi918265178	2.80	1	1	607	65.2	7.66	2.22	type IV secretion system-coupling protein virD4 [Arthrobacter sp. Hiyo1]
gi403229677	2.21	1	1	543	58.7	5.25	2.22	putative metal-dependent amidohydrolase [Arthrobacter sp. Rue61a]
gi674645059	1.98	1	1	858	90.9	6.00	2.22	UvrABC system protein A [Arthrobacter sp. 11W110_air]
gi307745673	2.13	1	1	469	49.2	5.49	2.22	2-oxoacid dehydrogenase E2 component [Arthrobacter arilaitensis Re117]
gi476403176	3.74	1	1	428	47.5	6.28	2.22	hypothetical protein D477_000090 [Arthrobacter crystallopoietes BAB-32]
gi910738892	10.44	1	1	182	19.8	9.19	2.22	molybdopterin molybdenumtransferase 2 [Arthrobacter sp. Hiyo4]
gi786026240	6.34	1	1	205	21.5	10.65	2.22	hypothetical protein [Arthrobacter chlorophenolicus]
gi910693388	4.27	1	1	398	41.5	9.13	2.22	C4-dicarboxylate transport protein [Arthrobacter sp. Hiyo6]
gi927031430	3.70	1	1	514	55.2	6.09	2.22	hypothetical protein AFL94_02820 [Arthrobacter sp. LS16]

gi737802462	5.56	1	1	342	36.5	5.76	2.22	6-phosphofructokinase [Arthrobacter castelli]
gi910739894	3.48	1	1	287	30.4	10.59	2.22	mercuric reductase [Arthrobacter sp. Hiyo4]
gi916871898	2.61	2	1	574	60.5	5.02	2.22	hypothetical protein, partial [Arthrobacter sp. H5]
gi917013451	4.29	1	1	303	33.5	10.17	2.22	hypothetical protein [Arthrobacter sanguinis]
gi470219959	2.53	1	1	395	40.2	8.68	2.22	sodium/hydrogen exchanger family protein [Arthrobacter gangotriensis Lz1y]
gi908740205	3.55	1	1	338	38.3	9.41	2.22	integrase [Arthrobacter arilaitensis]
gi476399234	5.44	1	1	147	15.8	8.24	2.22	polyamine ABC transporter ATP-binding subunit, partial [Arthrobacter crystallopoietes BAB-
gi674646953	4.39	1	1	319	32.0	5.36	2.22	putative L-asparaginase [Arthrobacter sp. 11W110_air]
gi908642087	2.76	1	1	399	41.5	7.24	2.22	ATPase [Arthrobacter phenanthrenivorans]
gi765010193	2.65	1	1	339	36.5	5.03	2.22	2-hydroxyacid dehydrogenase [Arthrobacter sp. A3]
gi517601219	7.61	1	1	197	21.3	9.66	2.22	resolvase [Arthrobacter sp. 162MFSha1.1]
gi517591768	4.37	1	1	183	20.2	5.00	2.22	riboflavin biosynthesis protein RibD [Arthrobacter sp. 135MFCol5.1]
gi759702506	6.05	1	1	215	21.7	6.54	2.22	hypothetical protein [Arthrobacter globiformis]
gi147829052	3.75	1	1	240	26.0	5.19	2.22	hypothetical protein pCM2_0012 (plasmid) [Clavibacter michiganensis subsp. michiganensis
gi723608456	7.41	1	1	162	18.2	5.53	2.22	hypothetical protein ART_2233 [Arthrobacter sp. PAMC25486]
gi916691842	4.78	1	1	230	24.9	6.42	2.22	hypothetical protein [Arthrobacter castelli]
gi651431656	7.17	1	1	237	24.4	5.16	2.22	2-C-methyl-D-erythritol 4-phosphate cytidylyltransferase [Arthrobacter sanguinis]
gi916782262	2.54	1	1	393	43.5	5.11	2.22	glycoside hydrolase family 15 [Arthrobacter sp. 35W]
gi916782200	4.40	2	1	364	38.0	5.95	2.22	hypothetical protein [Arthrobacter sp. 35W]
gi359306266	13.10	1	1	145	15.9	6.54	2.22	putative transposase [Arthrobacter globiformis NBRC 12137]
gi654818772	4.03	1	1	397	41.3	7.31	2.22	membrane protein [Arthrobacter sp. UNC362MFTsu5.1]
gi765005821	1.95	1	1	668	73.1	4.84	2.22	peptidase M13 [Arthrobacter sp. A3]
gi654812545	13.74	1	1	131	14.3	4.82	2.22	6-pyruvoyl tetrahydrobiopterin synthase [Arthrobacter sp. MA-N2]
gi359307673	6.42	1	1	187	19.8	9.52	2.22	hypothetical protein ARGLB_010_00840 [Arthrobacter globiformis NBRC 12137]
gi162953864	6.47	2	1	278	29.4	9.20	2.22	hypothetical membrane protein [Renibacterium salmoninarum ATCC 33209]
gi823665842	4.33	1	1	300	31.5	5.85	2.21	dihydropteroate synthase [Arthrobacter sp. YC-RL1]
gi786030629	4.27	1	1	211	21.4	11.39	2.21	lysine transporter LysE [Arthrobacter chlorophenolicus]
gi654828686	9.86	1	1	142	14.8	11.17	2.21	hypothetical protein [Arthrobacter sp. H5]
gi651466560	4.88	1	1	369	39.1	5.00	2.21	polyprenyl synthetase [Arthrobacter sp. 35/47]
gi219861365	3.11	1	1	322	36.0	5.11	2.21	conserved hypothetical protein [Arthrobacter chlorophenolicus A6]
gi517606740	5.79	1	1	242	26.5	5.48	2.21	protein-tyrosine phosphatase [Arthrobacter sp. 161MFSha2.1]
gi916289997	7.25	1	1	276	29.3	6.43	2.21	iron-dicitrate transporter ATP-binding subunit [Arthrobacter sp. Rue61a]
gi651431152	3.85	1	1	260	28.4	5.58	2.21	glucosamine-6-phosphate deaminase [Arthrobacter sanguinis]
gi914715384	5.71	1	1	333	35.0	5.92	2.21	exonuclease [Arthrobacter sp. ZBG10]
gi927293076	5.83	1	1	309	30.4	5.49	2.21	ROK family transcriptional regulator (plasmid) [Arthrobacter sp. ERGS1:01]
gi470217775	3.43	1	1	408	42.3	11.44	2.21	phosphoglycerate transporter family protein [Arthrobacter gangotriensis Lz1y]
gi651438645	9.49	1	1	158	17.7	5.27	2.21	hypothetical protein [Arthrobacter sp. H14]
gi403209501	3.16	1	1	316	33.5	6.00	2.21	prephenate dehydratase [Kineosphaera limosa NBRC 100340]
gi742071329	8.92	1	1	213	23.0	6.16	2.21	GntR family transcriptional regulator [Arthrobacter sp. MWB30]
gi476399092	2.70	1	1	555	58.9	7.62	2.21	GTP-binding protein [Arthrobacter crystallopoietes BAB-32]
gi640202768	6.03	1	1	448	45.7	9.92	2.21	lytic transglycosylase [Arthrobacter sp. 31Y]
gi654823118	8.52	1	1	223	22.6	5.10	2.21	aldolase [Arthrobacter sp. I3]
gi937262489	3.97	1	1	453	46.5	10.84	2.21	hypothetical protein AO716_04735 [Arthrobacter sp. Edens01]
gi517603553	6.14	1	1	228	26.0	10.33	2.21	hypothetical protein [Arthrobacter sp. 131MFCol6.1]
gi635351097	10.53	1	1	171	18.6	6.10	2.21	tetR [Arthrobacter siccitolerans]
gi651431158	3.44	1	1	262	28.0	4.92	2.21	alpha/beta hydrolase [Arthrobacter sanguinis]
gi742759525	3.96	1	1	303	31.7	8.09	2.21	diacylglycerol kinase [Arthrobacter phenanthrenivorans]
gi910737557	6.64	1	1	286	30.6	6.92	2.21	release factor glutamine methyltransferase [Arthrobacter sp. Hiyo4]
gi162954411	13.45	1	1	171	17.8	9.45	2.21	putative esterase [Renibacterium salmoninarum ATCC 33209]
gi517609489	1.73	1	1	635	65.1	6.57	2.21	hypothetical protein [Arthrobacter sp. 161MFSha2.1]
gi928488730	7.82	1	1	179	19.6	9.86	2.21	hypothetical protein AOC05_07365 [Arthrobacter alpinus]
gi654817778	13.60	1	1	125	13.8	4.64	2.21	glyoxalase [Arthrobacter sp. UNC362MFTsu5.1]
gi723606392	3.10	1	1	355	38.5	5.33	2.21	hypothetical protein ART_0169 [Arthrobacter sp. PAMC25486]
gi116609554	3.13	1	1	351	36.6	5.20	2.21	Alcohol dehydrogenase GroES domain protein [Arthrobacter sp. FB24]
gi918269193	12.50	1	1	80	8.3	6.61	2.21	dihydroxy-acid dehydratase [Arthrobacter sp. Hiyo1]
gi759730259	1.98	1	1	504	54.9	6.09	2.21	hypothetical protein [Arthrobacter sp. L77]
gi654814082	5.77	1	1	156	17.0	5.06	2.21	flavin-nucleotide-binding protein [Arthrobacter sp. MA-N2]
gi219858325	2.68	1	1	298	29.2	6.42	2.21	conserved hypothetical protein [Arthrobacter chlorophenolicus A6]

gi928489057	2.81	1	1	356	37.8	7.61	2.21	sugar ABC transporter [Arthrobacter alpinus]
gi937258437	2.05	1	1	536	55.1	4.54	2.21	flagellar M-ring protein FliF [Arthrobacter sp. Edens01]
gi917013242	5.75	1	1	226	24.7	5.58	2.21	hypothetical protein [Arthrobacter sanguinis]
gi916869622	5.07	1	1	276	30.2	8.13	2.21	prenyltransferase [Arthrobacter sp. Br18]
gi737802283	4.71	1	1	255	28.2	9.29	2.21	hypothetical protein [Arthrobacter castelli]
gi654816964	3.80	1	1	368	42.5	6.55	2.21	(p)ppGpp synthetase [Arthrobacter sp. UNC362MFTsu5.1]
gi219859419	13.68	1	1	117	11.3	12.31	2.21	conserved hypothetical protein [Arthrobacter chlorophenolicus A6]
gi359304526	6.88	1	1	247	25.5	5.59	2.21	putative oxidoreductase [Arthrobacter globiformis NBRC 12137]
gi651473745	3.66	1	1	355	37.4	11.22	2.21	fusaric acid resistance protein [Arthrobacter nicotinovorans]
gi219859174	10.13	1	1	237	25.3	6.61	2.21	response regulator receiver and unknown domain protein [Arthrobacter chlorophenolicus A
gi654817739	6.35	1	1	252	25.9	5.85	2.21	short-chain dehydrogenase [Arthrobacter sp. UNC362MFTsu5.1]
gi742860883	1.98	1	1	504	54.6	4.73	2.21	hypothetical protein [Arthrobacter sp. W1]
gi219860178	4.44	1	1	248	26.0	9.60	2.21	binding-protein-dependent transport systems inner membrane component [Arthrobacter ch
gi504874918	7.78	1	1	167	17.8	6.80	2.21	MULTISPECIES: MarR family transcriptional regulator [Arthrobacter]
gi910748082	22.50	1	1	80	8.7	7.53	2.21	uncharacterized ABC transporter ATP-binding protein MJ1508 [Arthrobacter sp. Hiyo8]
gi551255309	3.27	1	1	397	41.9	4.78	2.21	amidohydrolase [Arthrobacter sp. PAO19]
gi162953147	1.49	1	1	737	77.1	7.47	2.21	MFS transporter [Renibacterium salmoninarum ATCC 33209]
gi930825593	1.84	1	1	490	51.4	9.69	2.21	hypothetical protein AOZ07_04315 [Arthrobacter arilaitensis]
gi918267295	21.62	1	1	74	8.4	8.60	2.21	hypothetical protein AHiyo1_29370 [Arthrobacter sp. Hiyo1]
gi470220807	23.26	1	1	43	5.1	9.96	2.21	hypothetical protein ADIAG_00753 [Arthrobacter gangotriensis Lz1y]
gi517600911	6.59	1	1	273	29.0	6.35	2.21	proteasome subunit beta [Arthrobacter sp. 162MFSha1.1]
gi674645818	8.57	1	1	210	22.6	9.44	2.21	hypothetical protein BN1051_02016 [Arthrobacter sp. 11W110_air]
gi545108942	12.24	1	1	147	15.2	10.14	2.21	membrane protein [Arthrobacter sp. AK-YN10]
gi927293803	2.70	1	1	371	39.4	7.77	2.21	histidine kinase [Arthrobacter sp. ERGS1:01]
gi651452222	5.32	1	1	451	46.0	4.97	2.21	amino acid ABC transporter substrate-binding protein [Arthrobacter nicotinovorans]
gi759724642	2.76	1	1	362	38.7	5.44	2.21	CoA-transferase, partial [Arthrobacter sp. I3]
gi916820471	3.61	1	1	499	53.2	6.84	2.21	hypothetical protein [Arthrobacter sp. H20]
gi823665542	2.11	1	1	473	50.0	5.11	2.21	diaminopimelate decarboxylase [Arthrobacter sp. YC-RL1]
gi914715890	6.28	1	1	191	19.7	6.54	2.21	3-hexulose-6-phosphate isomerase [Arthrobacter sp. ZBG10]
gi119948535	4.74	1	1	211	22.6	9.99	2.21	putative oxidoreductase, short chain dehydrogenase/reductase family protein [Arthrobacter
gi219859856	3.08	1	1	422	42.6	10.98	2.21	major facilitator superfamily MFS_1 [Arthrobacter chlorophenolicus A6]
gi219858646	6.54	1	1	306	34.3	7.09	2.20	conserved hypothetical protein [Arthrobacter chlorophenolicus A6]
gi742857487	3.93	1	1	331	35.8	5.06	2.20	O-succinylbenzoate synthase [Arthrobacter sp. W1]
gi551254970	6.75	1	1	252	27.7	5.30	2.20	hypothetical protein [Arthrobacter sp. PAO19]
gi651465302	10.66	1	1	122	13.6	9.89	2.20	hypothetical protein [Arthrobacter sp. 35/47]
gi917739500	4.70	2	1	447	46.2	9.67	2.20	hypothetical protein [Arthrobacter sp. W1]
gi910696737	7.09	1	1	268	30.0	9.73	2.20	putative DNA ligase-like protein Rv0938/MT0965 [Arthrobacter sp. Hiyo6]
gi636846712	26.44	1	1	87	9.4	8.47	2.20	hypothetical protein [Arthrobacter sp. TB 26]
gi654819239	16.04	2	1	106	11.3	9.52	2.20	hypothetical protein [Arthrobacter sp. UNC362MFTsu5.1]
gi916357124	2.02	1	1	644	68.8	6.06	2.20	amino acid transporter [Arthrobacter sp. 162MFSha1.1]
gi518311413	3.12	1	1	513	55.3	8.90	2.20	hypothetical protein [Arthrobacter sp. TB 23]
gi910250105	4.63	1	1	281	29.6	4.87	2.20	fumarylacetoacetate hydrolase [Arthrobacter siccitolerans]
gi927296425	3.30	1	1	333	35.3	5.27	2.20	hypothetical protein AL755_15205 [Arthrobacter sp. ERGS1:01]
gi910740357	7.63	1	1	262	27.3	6.29	2.20	putative ribosome biogenesis GTPase RsgA [Arthrobacter sp. Hiyo4]
gi939037359	11.63	1	1	86	8.7	4.65	2.20	hypothetical protein [Arthrobacter nitroguajacolicus]
gi636845602	10.67	1	1	75	8.3	9.52	2.20	prevent-host-death family protein [Arthrobacter sp. TB 26]
gi517602383	4.19	1	1	406	45.2	5.52	2.20	isocitrate dehydrogenase [Arthrobacter sp. 131MFCol6.1]
gi651483580	6.37	1	1	267	27.7	10.65	2.20	hypothetical protein [Arthrobacter sp. Br18]
gi219858372	19.79	1	1	96	10.0	10.87	2.20	conserved hypothetical protein [Arthrobacter chlorophenolicus A6]
gi518312602	5.80	1	1	293	29.5	5.66	2.20	MULTISPECIES: orotidine 5'-phosphate decarboxylase [Arthrobacter]
gi914244856	9.30	1	1	172	18.9	5.95	2.20	hydroxyatrazine hydrolase, partial [Arthrobacter sp. T3AB1]
gi651458631	5.88	1	1	255	27.9	4.89	2.20	hypothetical protein [Arthrobacter sp. 35/47]
gi443480970	4.00	1	1	400	41.4	7.06	2.20	tRNA(Ile)-lysidine synthetase [Arthrobacter nitrophenolicus]
gi651430586	3.59	1	1	418	45.2	4.96	2.20	Fe-S cluster assembly protein SufD [Arthrobacter sanguinis]
gi654818057	6.63	1	1	332	35.5	4.93	2.20	hydroxylase [Arthrobacter sp. UNC362MFTsu5.1]
gi652424180	5.44	1	1	294	30.5	9.11	2.20	1,4-dihydroxy-2-naphthoate octaprenyltransferase [Arthrobacter castelli]
gi636845212	5.86	1	1	290	31.0	5.27	2.20	haloacid dehalogenase [Arthrobacter sp. TB 26]
gi917572043	3.70	1	1	270	30.2	7.53	2.20	AraC family transcriptional regulator [Arthrobacter sp. PAO19]

gi723608670	2.98	1	1	637	65.3	9.39	2.20	hypothetical protein ART_2447 [Arthrobacter sp. PAMC25486]
gi470221268	8.89	1	1	90	10.4	4.70	2.20	DNA gyrase subunit A [Arthrobacter gangotriensis Lz1y]
gi219858769	8.03	1	1	249	27.7	8.05	2.20	ABC transporter related [Arthrobacter chlorophenolicus A6]
gi908698169	2.87	1	1	766	76.2	4.54	2.20	hypothetical protein [Arthrobacter sp. RIT-PI-e]
gi517602888	3.87	1	1	362	39.9	6.47	2.20	MULTISPECIES: phosphate starvation protein PhoH [Arthrobacter]
gi551254084	7.23	1	1	83	8.7	10.37	2.16	hypothetical protein [Arthrobacter sp. PAO19]
gi515767613	2.34	1	1	512	53.1	5.73	2.15	histidine kinase [Arthrobacter sp. M2012083]
gi737773933	18.97	1	1	58	6.3	10.93	2.15	hypothetical protein [Arthrobacter sp. MA-N2]
gi908698717	2.78	1	1	252	27.2	6.24	2.14	glycerophosphodiester phosphodiesterase [Arthrobacter sp. RIT-PI-e]
gi652425838	8.40	1	1	119	13.8	5.03	2.12	membrane protein [Arthrobacter castelli]
gi651440976	2.18	1	1	505	54.0	5.12	2.12	sorbosone dehydrogenase [Arthrobacter sp. 9MFCol3.1]
gi939037015	3.30	7	1	182	19.3	4.79	2.11	hypothetical protein [Arthrobacter nitroguajacolicus]
gi517598539	1.76	2	1	512	53.3	5.71	2.10	LPS biosynthesis protein [Arthrobacter sp. 162MFSha1.1]
gi476401914	1.42	1	1	493	51.9	8.70	2.09	hypothetical protein D477_006546 [Arthrobacter crystallopoietes BAB-32]
gi219859360	12.00	1	1	50	5.4	10.67	2.09	hypothetical protein Achl_1720 [Arthrobacter chlorophenolicus A6]
gi759733619	3.99	2	1	301	33.8	5.67	2.09	hypothetical protein [Arthrobacter sp. L77]
gi910283916	4.33	2	1	254	26.0	9.61	2.08	hypothetical protein [Arthrobacter sp. A3]
gi765009820	3.70	1	1	243	27.0	5.24	2.07	hypothetical protein [Arthrobacter sp. A3]
gi162953201	10.98	2	1	82	9.2	10.92	2.05	putative stress-responsive transcriptional regulator [Renibacterium salmoninarum ATCC 332
gi640193986	6.98	1	1	129	14.1	6.25	2.05	hypothetical protein [Arthrobacter sp. 31Y]
gi767258068	4.07	1	1	221	23.9	4.58	2.04	NUDIX hydrolase [Arthrobacter sp. IHBB 11108]
gi916781575	1.64	1	1	794	81.2	8.34	2.04	hypothetical protein [Arthrobacter sp. 35W]
gi742860425	2.99	1	1	234	25.1	6.35	2.04	GntR family transcriptional regulator [Arthrobacter sp. W1]
gi359306129	1.41	1	1	708	71.4	10.11	2.03	hypothetical protein ARGLB_047_00760 [Arthrobacter globiformis NBRC 12137]
gi476402625	6.94	1	1	144	15.7	8.28	2.02	hypothetical protein D477_002643 [Arthrobacter crystallopoietes BAB-32]
gi742756498	14.75	1	1	61	7.0	4.48	2.01	hypothetical protein RM50_09740 [Arthrobacter phenanthrenivorans]
gi403230371	1.48	1	1	473	48.2	6.67	2.01	protoporphyrinogen oxidase HemG [Arthrobacter sp. Rue61a]
gi470217828	5.70	1	1	158	17.5	5.43	2.01	transcriptional regulator [Arthrobacter gangotriensis Lz1y]
gi727802698	3.37	1	1	326	35.7	5.72	2.01	chorismate mutase [Bifidobacterium saguini]
gi636846585	1.06	1	1	662	70.2	9.50	2.01	hypothetical protein [Arthrobacter sp. TB 26]
gi939051255	4.57	1	1	219	24.8	5.48	2.00	GntR family transcriptional regulator [Arthrobacter sp. JCM 19049]
gi403231978	1.42	1	1	918	100.9	6.00	2.00	hypothetical protein ARUE_232p01910 (plasmid) [Arthrobacter sp. Rue61a]
gi928488635	1.20	2	1	665	69.6	6.07	2.00	ATP-dependent helicase [Arthrobacter alpinus]
gi219861726	2.07	1	1	434	46.5	5.01	2.00	conserved hypothetical protein (plasmid) [Arthrobacter chlorophenolicus A6]
gi755973045	3.81	1	1	315	33.1	5.71	2.00	Prephenate dehydratase [Corynebacterium marinum DSM 44953]
gi654813586	2.70	1	1	259	28.0	9.20	1.99	hypothetical protein [Arthrobacter sp. MA-N2]
gi651444744	2.03	1	1	296	32.4	8.43	1.99	XRE family transcriptional regulator [Arthrobacter nicotinovorans]
gi307744768	2.36	1	1	297	32.4	4.31	1.98	osmoprotectant (glycine betaine/carnitine/choline/L-proline) ABC transporter, substrate-bin
gi162952931	2.43	2	1	288	30.3	10.51	1.98	conserved hypothetical protein [Renibacterium salmoninarum ATCC 33209]
gi759709578	2.30	1	1	305	33.2	6.06	1.96	RNA polymerase sigma24 factor [Arthrobacter sp. 9MFCol3.1]
gi403227783	1.79	1	1	447	49.8	5.06	1.96	putative lysine/ornithine N-monooxygenase [Arthrobacter sp. Rue61a]
gi928486675	1.78	1	1	505	56.9	5.19	1.96	sulfatase [Arthrobacter alpinus]
gi937256683	2.89	1	1	380	41.1	6.90	1.96	hypothetical protein AO716_16775 [Arthrobacter sp. Edens01]
gi737809920	2.70	1	1	259	27.5	5.07	1.95	SAM-dependent methyltransferase [Arthrobacter sp. 35/47]
gi551254432	9.62	2	1	104	11.8	8.57	1.95	hypothetical protein [Arthrobacter sp. PAO19]
gi651435865	2.78	1	1	288	30.3	5.39	1.95	nicotinate-nucleotide pyrophosphorylase [Arthrobacter sp. H41]
gi640196048	3.74	2	1	321	35.1	10.14	1.94	sugar ABC transporter permease [Arthrobacter sp. 31Y]
gi927294307	3.00	1	1	233	25.9	6.02	1.93	phosphatidylinositol kinase [Arthrobacter sp. ERGS1:01]
gi916835244	3.54	1	1	198	22.1	7.14	1.92	cation:proton antiporter [Arthrobacter sp. H14]
gi219858119	3.00	1	1	400	44.6	9.57	1.92	Integrase catalytic region [Arthrobacter chlorophenolicus A6]
gi323470590	1.62	1	1	743	82.0	4.88	1.92	oligopeptidase B [Arthrobacter phenanthrenivorans Sphe3]
gi652424698	4.48	1	1	223	24.9	5.77	1.92	GntR family transcriptional regulator [Arthrobacter castelli]
gi767257627	3.61	1	1	249	26.8	10.36	1.92	hypothetical protein UM93_08265 [Arthrobacter sp. IHBB 11108]
gi910746159	2.68	1	1	299	31.8	7.09	1.92	D-threo-aldose 1-dehydrogenase [Arthrobacter sp. Hiyo8]
gi403229425	7.34	1	1	109	12.0	9.86	1.91	hypothetical protein ARUE_c19410 [Arthrobacter sp. Rue61a]
gi545110192	1.65	1	1	547	59.7	6.44	1.91	hypothetical protein [Arthrobacter sp. AK-YN10]
gi517601099	2.55	1	1	275	29.6	4.83	1.91	hypothetical protein [Arthrobacter sp. 162MFSha1.1]
gi636843818	3.28	1	1	305	33.7	5.03	1.91	thiosulfate sulfurtransferase [Arthrobacter sp. TB 26]
