# Supplementary material for: Effects of two kinds of imidazolium-based ionic liquids on the characteristics of steroid-transformation Arthrobacter simplex
Source: Microb Cell Fact. 2016 Jul 1;15:118. doi: 10.1186/s12934-016-0518-3 (PMC4930596; doi:10.1186/s12934-016-0518-3)
Supplement: Supplementary file 4 — 10.1186/s12934-016-0518-3 The bioinformatics analysis of ASP reduced proteins affected by ILs. NCBI searches were performed in Uniprot protein database for analysis of the location and function classify. [file 12934_2016_518_MOESM4_ESM.pdf]

Additional file 4: The bioinformatics analysis of *ASP* reduced proteins affected by ILs. NCBI searches were performed in Uniprot protein database for analysis of the location and function classify.

	Accession	MW [kDa]	calc. pI	Location	FC[a]	Description
A	gi690773186	69.0	5.05	Cytoplasm	T	GTP-binding protein TypA
	gi690771960	12.9	4.51	Cytoplasm	J	50S ribosomal protein L7
	gi674646060	142.9	6.16	Cytoplasm	K	DNA-directed RNA polymerase subunit beta'
	gi674645978	56.1	4.97	Cytoplasm	O	60 kDa chaperonin 1
	gi672940878	56.5	4.86	Cytoplasm	O	chaperonin GroEL
	gi672940874	65.8	4.68	Cytoplasm	O	molecular chaperone DnaK
	gi651439668	21.6	10.08	Cytoplasm	S	hypothetical protein
	gi651431413	77.7	5.59	Cytoplasm	L	ATP-dependent DNA helicase RecQ
	gi545108141	25.3	4.67	Cytoplasm	R	haloacid dehalogenase
	gi476399111	48.9	4.96	Cytoplasm	C	succinate-semialdehyde dehydrogenase
	gi219861633	7.2	4.88	Cytoplasm	K	cold-shock DNA-binding domain protein
	gi163841921	91.4	5.60	Cytoplasm	J	hemolysin
	gi163840368	180.7	5.14	Cytoplasm	E	NAD-specific glutamate dehydrogenase
	gi163841228	47.9	5.22	Cytoplasm	M	UDP-N-acetylmuramate--L-alanine ligase
	gi116609147	7.2	4.94	Cytoplasm	K	cold-shock DNA-binding protein family
	gi651445684	213.6	5.11	Membrane	W	fibronectin
	gi651431168	59.1	5.05	Membrane	C	ATP synthase F0F1 subunit alpha
	gi651431165	53.0	4.84	Membrane	C	ATP F0F1 synthase subunit beta
	gi489897952	95.8	5.35	Membrane	O	ATP-dependent Clp protease ATP-binding protein
	gi116609504	32.1	5.72	Membrane	G	monosaccharide ABC transporter substrate-binding protein, CUT2 family
	gi443481594	26.9	10.52	Unknown	I	hypothetical protein G205_11704
	gi443479672	15.5	10.87	Unknown	S	hypothetical protein G205_23057
B	gi690772404	80.1	4.92	Cytoplasm	J	polynucleotide phosphorylase
	gi674645749	144.9	6.98	Cytoplasm	L	ATP-dependent RNA helicase HrpB
	gi654815574	92.5	5.6	Cytoplasm	C	NDP-hexose 4-ketoreductase
	gi652423038	104.8	6.86	Cytoplasm	L	RNA helicase
	gi651505257	93.9	5.07	Cytoplasm	O	ATPase AAA
	gi651453813	91.3	6.14	Cytoplasm	L	ATP-dependent DNA helicase PcrA
	gi651447330	17.8	7.05	Cytoplasm	R	CoA-binding protein
	gi651440158	45.4	5.39	Cytoplasm	K	RNA polymerase sigma factor
	gi635352443	110	5.19	Cytoplasm	L	recF/RecN/SMC N terminal domain protein
	gi551254973	50.4	5.94	Cytoplasm	P	ATPase
	gi551253914	24.8	9.29	Cytoplasm	J	50S ribosomal protein L1
	gi518312569	36.4	6.19	Cytoplasm	K	HrcA family transcriptional regulator
	gi476401088	101.8	4.84	Cytoplasm	C	aconitate hydratase
	gi470220608	45.8	5.27	Cytoplasm	F	pyrimidine-nucleoside phosphorylase

	gi163840927	129.2	5.03	Cytoplasm	K	DNA-directed RNA polymerase subunit beta
	gi163840894	13.3	10.15	Cytoplasm	J	50S ribosomal protein L14
	gi119950562	46.6	8.68	Cytoplasm	C	L-lactate dehydrogenase
	gi665848643	126.6	5.74	Membrane	M	peptidase S41
	gi636845560	73.8	5.24	Unknown	S	hypothetical protein
	gi1906824	50	11.56	Unknown	S	hypothetical protein
C	gi674647015	57.1	4.84	Cytoplasm	O	60 kDa chaperonin 1
	gi674646030	13.4	10.15	Cytoplasm	J	50S ribosomal protein L14
	gi674644816	78.9	4.93	Cytoplasm	J	Polyribonucleotide nucleotidyltransferase
	gi654819237	62.9	5.12	Cytoplasm	I	acetyl-COA carboxylase
	gi654816002	93.5	5.16	Cytoplasm	L	DEAD/DEAH box helicase
	gi654813326	23.7	10.05	Cytoplasm	J	50S ribosomal protein L20
	gi652425290	16.2	6	Cytoplasm	K	MarR family transcriptional regulator
	gi652423332	56	4.78	Cytoplasm	O	molecular chaperone GroEL
	gi651502944	144.7	6.87	Cytoplasm	L	ATP-dependent helicase
	gi651485631	39.5	4.89	Cytoplasm	R	GTP-binding protein
	gi651457875	96.5	5.43	Cytoplasm	J	alanyl-tRNA synthetase
	gi651457048	15	9.06	Cytoplasm	J	30S ribosomal protein S16
	gi651443967	24.9	9.25	Cytoplasm	J	50S ribosomal protein L1
	gi651442444	41	5.82	Cytoplasm	H	molybdenum cofactor biosynthesis protein MoeA
	gi651440341	81.3	4.91	Cytoplasm	J	polynucleotide phosphorylase
	gi651431245	56.1	4.74	Cytoplasm	O	molecular chaperone GroEL
	gi640195629	43.6	5.19	Cytoplasm	J	elongation factor Tu
	gi635351932	95.9	5.38	Cytoplasm	J	alanyl-tRNA synthetase
	gi545111051	79.6	5.03	Cytoplasm	C	isocitrate dehydrogenase
	gi517598212	59	5.43	Cytoplasm	J	arginyl-tRNA synthetase
	gi470219995	66.1	4.74	Cytoplasm	O	chaperone protein DnaK
	gi307745569	36.1	4.75	Cytoplasm	K	DNA-directed RNA polymerase alpha chain
	gi219858819	79.4	4.92	Cytoplasm	C	isocitrate dehydrogenase, NADP-dependent
	gi163840517	130	5.22	Cytoplasm	K	chromosome segregation protein
	gi116610730	93.5	5.14	Cytoplasm	L	DEAD/DEAH box helicase domain protein
	gi640203686	114.8	6.46	Membrane	R	beta-phosphoglucomutase
	gi517602042	196	5.01	Membrane	W	fibronectin, partial
	gi515767041	56.2	9.47	Membrane	U	signal recognition particle
	gi489902339	39.5	9.91	Membrane	E	amino acid ABC transporter permease
	gi325962997	118.4	9.17	Membrane	C	cysteine ABC transporter ATP-binding protein/permease
	gi163840640	43.8	5.64	Membrane	E	glycine betaine transport ATP-binding protein
	gi325963125	78.1	5.55	Unknown	F	DNA/RNA endonuclease G, NUC1
	gi654824335	50.4	6.18	Unknown	S	hypothetical protein
	gi654811523	141.5	6.98	Unknown	S	hypothetical protein
	gi116610004	25.8	9.82	Unknown	S	conserved hypothetical protein

A：[PrMIm]PF_6-treated cells，B：[PrMIm]BF_4-treated cells，C: Both two ILs -treated cells

[a] Function Classify :1, Amino acid transport and metabolism [E]; 2, Carbohydrate transport and metabolism [G]; 3, Cell wall/membrane/envelope biogenesis[M]; 4, Coenzyme transport and metabolism[H]; 5, Energy production and conversion[C]; 6, Extracellular structures[W]; 7, Function unknown or No information for functional categories [S];8, General function prediction only[R]; 9, Inorganic ion transport and metabolism[P]; 10, Lipid transport and metabolism[I]; 11, Nucleotide transport and metabolism[F]; 12, Posttranslational modification, protein turnover, chaperones[O]; 13, Replication, recombination and repair [L]; 14, Signal transduction mechanisms[T]; 15, Transcription[K]; 16, Translation, ribosomal structure and biogenesis [J]
